# Human *ex vivo* prostate tissue model system identifies ING3 as an oncoprotein

**DOI:** 10.1038/bjc.2017.447

**Published:** 2018-01-30

**Authors:** Urszula L McClurg, Arash Nabbi, Charles Ricordel, Svitlana Korolchuk, Stuart McCracken, Rakesh Heer, Laura Wilson, Lisa M Butler, Bronwyn Kate Irving-Hooper, Rémy Pedeux, Craig N Robson, Karl T Riabowol, Olivier Binda

**Affiliations:** 1 Newcastle Cancer Centre at the Northern Institute for Cancer Research, Newcastle University, Newcastle-upon-Tyne NE2 4HH, England; 2 Department of Biochemistry and Molecular Biology, University of Calgary, Calgary, AB T2N 1N4, Canada; 3 Department of Oncology, University of Calgary, Calgary, AB T2N 1N4, Canada; 4 Université Rennes 1, CLCC Eugène Marquis, INSERM ERL440-OSS, Rue Bataille Flandres Dunkerque, Batiment D, 1er étage, Rennes 35042, France; 5 School of Medicine and Freemasons Foundation Centre for Men’s Health, University of Adelaide, South Australian Health and Medical Research Institute, Adelaide, SA 5005, Australia

**Keywords:** ING3, H3K4^me3^, chromatin, methylated histones, prostate cancer

## Abstract

**Background:**

Although the founding members of the INhibitor of Growth (ING) family of histone mark readers, ING1 and ING2, were defined as tumour suppressors in animal models, the role of other ING proteins in cellular proliferation and cancer progression is unclear.

**Methods:**

We transduced *ex vivo* benign prostate hyperplasia tissues with inducible lentiviral particles to express ING proteins. Proliferation was assessed by H3S10^phos^ immunohistochemistry (IHC). The expression of ING3 was assessed by IHC on a human prostate cancer tissue microarray (TMA). Gene expression was measured by DNA microarray and validated by real-time qPCR.

**Results:**

We found that ING3 stimulates cellular proliferation in *ex vivo* tissues, suggesting that ING3 could be oncogenic. Indeed, ING3 overexpression transformed normal human dermal fibroblasts. We observed elevated levels of ING3 in prostate cancer samples, which correlated with poorer patient survival. Consistent with an oncogenic role, gene-silencing experiments revealed that ING3 is required for the proliferation of breast, ovarian, and prostate cancer cells. Finally, ING3 controls the expression of an intricate network of cell cycle genes by associating with chromatin modifiers and the H3K4^me3^ mark at transcriptional start sites.

**Conclusions:**

Our investigations create a shift in the prevailing view that ING proteins are tumour suppressors and redefine ING3 as an oncoprotein.

In 2012, it was estimated that >1 million men were diagnosed with prostate cancer (PC) and 307 000 died from it worldwide, placing PC as the fifth leading cause of death from cancer in men (excluding non-melanoma skin cancers) (Ferlay *et al*, 2015). PC initiation and progression depend on androgens and the androgen receptor (AR) (Wong *et al*, 2014; Ferraldeschi *et al*, 2015). Although androgen-deprivation-based therapies (termed chemical castration) initially benefit most patients, PC inevitably recurs but in a castrate-resistant and invariably fatal form. Current PC diagnosis incorporates assessment of prostate-specific antigen (PSA) levels in patient sera with trans-rectal ultrasound-guided prostate biopsies. However, PSA thresholds are unreliable and biopsies are misdiagnosed in about 30–50% of cases. Although the recently developed urinary PCA3 (PC gene 3) and PSA-based prostate health index blood tests offer alternative assessment methods and multiparametric magnetic resonance imaging improves tumour detection, novel, more reliable prognostic biomarkers are still required. Thus a better understanding of the molecular mechanisms driving PC is vital.

The INhibitor of Growth (ING) family of chromatin readers was established over 20 years ago with the identification of the tumour-suppressor ING1 (Garkavtsev *et al*, 1996). ING proteins regulate access to genetic information in part through associating with the histone H3 trimethylated on lysine 4 (H3K4^me3^) active gene expression mark and tethering enzymatic activities to facilitate (e.g. ING4 via the acetyltransferase HBO1; Hung *et al*, 2009) or to restrict (e.g. ING2 via the deacetylase HDAC1; Shi *et al*, 2006) access to genetic information. The interaction with H3K4^me3^ is mediated via a plant homeodomain (PHD) located at the carboxy terminus of each ING protein. Although ING1 and ING2 associate with the mSIN3A/HDAC1 histone deacetylase complex, ING3, ING4, and ING5 associate with either TIP60, HBO1, or MOZ histone acetyltransferase complexes (Doyon *et al*, 2006). Of note, ING3 is required along with EPC1 for full TIP60 histone acetyltransferase activity on nucleosomes (Doyon *et al*, 2004). In addition, ING3 is part of the ANP32E histone exchange complex (Obri *et al*, 2014), which is similar to the core ING3/TIP60 complex (Doyon *et al*, 2004, 2006). Given that ING1 and ING2 associate with histone deacetylases (HDACs), they are believed to principally function as transcriptional repressors, while ING3-5 would function as transcriptional activators (Bua and Binda, 2009).

Initially identified as a transcriptional co-activator that associates with the Tat transactivating factor from human immunodeficiency virus 1 (HIV-1) (Kamine *et al*, 1996), TIP60 (Tat interactive protein, 60 kDa) was rapidly found to have histone acetyltransferase (HAT) activity (Yamamoto and Horikoshi, 1997) and to potentiate the transcriptional activity of AR (Brady *et al*, 1999). As an integral part of the TIP60 complex, we hypothesised that ING proteins could have an unsuspected role in AR signalling, potentially regulating a transcriptional network and cellular proliferation. We thus established an inducible expression system to screen ING proteins for proliferative potential in an *ex vivo* human prostate tissue model and identified ING3 as an inducer of growth. Then, we measured ING3 levels in benign tissues compared with matched cancer samples and found that elevated ING3 levels correlate with treatment resistance and poor survival, corroborating *in vitro* assays suggesting that ING3 has oncogenic properties. Indeed, ING3 expression was sufficient to transform normal human cells as assessed by anchorage-independent growth. Gene expression profiling identified several cell cycle regulatory genes as well as AR- and p53-responsive genes, whose expression was altered in the absence of ING3. The silencing of ING3 in breast cancer, ovarian cancer, and PC cell line models led to inhibition of proliferation characterised by a G_1_/S arrest accompanied by an induction of apoptosis. Mechanistically, ING3 associates with chromatin modifiers and H3K4^me3^ at the transcriptional start site (TSS) of cell cycle genes to regulate gene expression. Collectively, we show that ING3 associates with gene promoters to regulate a transcriptional network that is required for cellular proliferation. Importantly, ING3 elevated copy number and protein levels in cancer patients, particularly in treatment-resistant patients, designate ING3 as a novel marker of poor survival for cancer patients and an unsuspected oncoprotein. We thus propose to rename the INhibitor of Growth 3 the INducer of Growth 3 to retain the gene name while highlighting the potential of ING3 to promote cellular proliferation.

## Materials and methods

### Antibodies and plasmids

The cDNA of human ING3 was cloned by PCR from total RNA extracted from the MCF7 cell line using forward 5′-GGCCAGATCTTTGTACCTAGAAGACTATCTGGA-3′ and reverse 5′-AGGACTCGAGTTATTTGTGTCTGCTGCCTCT-3′ primers, inserted in pCMV-3Tag-1A (Stratagene, Agilent, CA, USA) in frame with the 3xFLAG tag. The 3xFLAG-tagged ING3 cDNA was also inserted in the pLVX Lenti-X Tet-One inducible expression system (Clontech, Takara, France) using the In-Fusion HD enzyme (Clontech). The mouse monoclonal *α*-ING3 antibody was previously described and extensively characterised (Nabbi *et al*, 2015). The anti-*α*-tubulin and *α*-FLAG antibodies, as well as *α*-FLAG M2-agarose were purchased from Sigma (Gillingham, UK). The HRP-conjugated *α*-GST (ab3416), *α*-H3 (ab1791), *α*-H3K4^me3^ (ab8580), and *α*-TIP49A (ab133513) antibodies were purchased from Abcam (Cambridge, UK).

### Cell culture, transfections, and lentiviral transductions

LNCaP, CWR22Rv1, MCF7, and PC3 cells were obtained from American Type Culture Collection (Manassas, VA, USA), while MX-1, PEO1, and PEO4 cells were obtained from the Northern Institute for Cancer Research authenticated cell bank. Cells were maintained in RPMI 1640 media supplemented with 2 mM L-glutamine (Invitrogen, Paisley, UK) and 10% (v/v) foetal calf serum (FCS) at 37 °C in 5% CO_2_. LNCaP-AI variant cell line was derived in-house by culturing LNCaP cells in steroid-depleted media to allow for the development of androgen independence (Lu *et al*, 1999). Cell lines were maintained for up to 30 passages or a maximum of 2 months of continuous culturing. As per institutional policy, cell lines were tested for mycoplasma on a tri-monthly basis. For p53 ChIP experiments, U2OS were maintained in modified McCoy’s 5A medium supplemented with GlutaMAX and 10% FCS. Proliferation was measured by live cell imaging four times a day with the Incucyte system for 114 h postsilencing. Lentiviral particles were generated by co-transfecting HEK293T with pLVX-3xFLAG-ING3 (ING3_WT_, ING3_Y362A_, or ING3_W385A_), pMD2.G, and psPAX2 (latter two were gifts from Didier Trono; Addgene (Cambridge, UK) 12260 and 12259, respectively) and used as described previously (O'Neill *et al*, 2014). The expression of FLAG-ING3 was induced using doxycycline at 10 ng ml^−1^.

### *Ex vivo* culture

Benign prostatic hyperplasia (BPH) samples were obtained from cancer-free patients as established by the Pathology Department at the Freeman Hospital (Newcastle upon Tyne, UK). The tissue samples were obtained with full ethical approval from Newcastle & North Tyneside 1 NHS Strategic Health Authority Local Research Ethics Committee (reference 15/NE/0400). All methods were performed in accordance with the relevant guidelines and regulations. BPH samples were removed surgically following patient’s written informed consent and placed in ice-cold culture media. Within 48 h from the surgery, tissues were dissected to 1 mm^3^ pieces and cultured in duplicates on gelatin sponges (Spongostan, Johnson & Johnson, New Brunswick, NJ, USA) presoaked in culture media supplemented with 1 × antimycotic solution (Sigma), 10 μg ml^−1^ hydrocortisone, and 10 μg ml^−1^ insulin solution from bovine pancreas (Sigma) as previously described (Centenera *et al*, 2012). *Ex vivo* explants were transduced with empty vector (pLVX) or FLAG-ING3 lentiviral particles in media supplemented with 10 ng ml^−1^ of doxycycline and tissues were cultured for 72 h. At the termination of the experiments, samples were immediately placed in 4% formalin for 1 h followed by processing in ethanol then xylene and finally paraffin embedding. Formalin-fixed paraffin-embedded tissues were stained as indicated and scored automatically using the Aperio imaging system (Milton Keynes, UK). Error bars represent s.e.m. of three independent patient samples.

### Anchorage-independent growth

Normal human dermal fibroblasts (NHDF) were isolated from juvenile foreskin (PromoCell C-12300, Heidelberg, Germany) and maintained in fibroblast growth medium (PromoCell C-23010). NHDF were transduced with lentiviral particles, ING3 expression was induced with doxycycline (10 ng ml^−1^), and 48 h later cultured in fibroblast media containing 0.56% methylcellulose. Fibroblasts were seeded at a density of 2500 cells per well in a 24-well plate and incubated at 37 °C in 5% CO_2_ for 14 days followed by imaging with IncuCyte and automated colony counting. Transformation assays with 3T3-L1 murine fibroblasts were performed essentially as with NHDF but were seeded at 250 cells per well in a 24-well plate.

### siRNA gene silencing and gene expression analysis

The *ING3* targeting siRNA sequences were (no.1) CAAUCACCAUGCUCAUUCA[dTdT] and (no.2) CUAUAGAAUGGUUCCAUUA[dTdT]. Cells were transfected with siRNA using RNAiMax (Invitrogen) according to the manufacturer’s instructions and incubated in culture media for 96 h prior to cell lysis and analysis by immunoblotting or real-time qPCR using specific primers (sequences available in Supplementary Table S1). For real-time qPCR, total RNA was extracted using TRIzol (Invitrogen, 15596-026), RNA quality and yields were assessed using a NanoDrop 2000 (NanoDrop, UK), 1 μg of total RNA was reverse transcribed using SuperScript VILO (Invitrogen, 11755-050), and qPCR was performed using QuantiTect SYBR Green (QIAGEN, 204143, Manchester, UK) on an ABI PRISM 7900HT Sequence Detection System (Applied Biosystems, Foster City, CA, USA). Data were tested for parametric distribution. Parametric data were analysed using appropriate *t*-tests or ANOVA with Bonferroni’s comparison test for multiple group comparisons. Non-parametric data were analysed using Wilcoxon signed-rank test. By convention, *P*-values are marked as follows; ***P<0.001, **P<0.01, and *P<0.05.

### Microarray

LNCaP cells were transfected with siRNA and cultured in steroid-depleted media for 72 h followed by 24 h stimulation with 10 nM dihydrotestosterone (DHT). Total RNA was extracted (RNeasy Plus, QIAGEN) and its purity confirmed both using the NanoDrop and Bioanalyser. Samples were processed on the Illumina Human HT-12 platform by High Throughput Genomics (The Wellcome Trust Centre for Human Genetics, University of Oxford, Oxford, UK) and analysed using the GenomeStudio software (Illumina, Cambridge, UK). Experiments consisted of four independent biological repeats of scrambled control-, siRNA no.1-, or siRNA no.2-treated samples. Analysis was performed by comparing *ING3* siRNA to scrambled siRNA and results compiled.

### Immunohistochemistry

Tissue microarrays (TMA) containing 0.6 mm cores of benign prostatic hyperplasia (BPH) (*n*=41), PC (*n*=81), and control tissues, including breast, kidney, placenta, ovary, and liver, were used (Coffey *et al*, 2013). These samples were obtained with full ethical approval from the Northumberland, Tyne, and Wear NHS Strategic Health Authority Local Research Ethics Committee (reference 2003/11). Antigens were retrieved by pressure cooking the TMA in 10 mM citrate pH 6.0 followed by staining the tissues with an extensively validated (Nabbi *et al*, 2015) mouse monoclonal *α*-ING3 antibody. The TMA were independently scored by 2 individuals using the 0–300 *H*-score method (Kirkegaard *et al*, 2006). Briefly, percentage and intensity of staining for positive cells was estimated (0, 1, 2, 3) using the following equation *H*-score=(% of cells with low-level positivity)+2 × (% of cells with medium-level positivity)+3 × (% of cells with high-level positivity).

### Flow cytometry

Cell cycle profiles were generated by propidium iodide (PI) staining; cells were permeabilised with 1% Triton X-100 and incubated with 1 μg ml^−1^ RNaseA and PI followed by analysis using a BD FACScan, as described previously (Burska *et al*, 2013). Levels of apoptosis were analysed after 96 h of gene silencing by Annexin V assay (BD, Oxford, UK) according to the manufacturer’s instructions and analysed using a BD FACScan. Cells were stained for both Annexin V and PI positivity and, during analysis, divided into quarters representing normal cells, necrotic cells, and apoptotic cells.

### Chromatin immunoprecipitation

LNCaP cells were maintained in steroid-depleted media for 72 h followed by stimulation with 10 nM DHT for 120 min. Then ChIP were performed as described previously (Gaughan *et al*, 2002). To quantify the binding of AR or ING3 at ARE or TSS, we performed real-time qPCR on ChIPed DNA using specific primers (sequences available in Supplementary Table S2). Data are represented as percentage of input fold change (% input=100 × 2^(CTinput−CTChIP)^); CT refers to cycle threshold.

## Results

### ING3 functions as an oncoprotein

**Figure 1:**
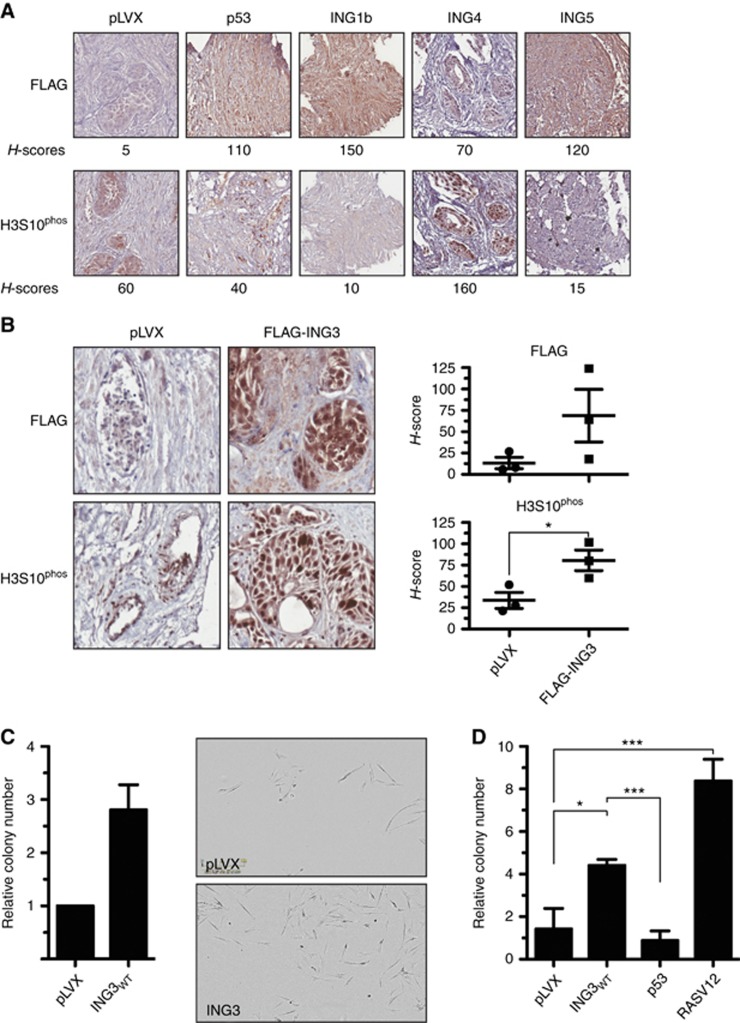
***Ex vivo* screen and transformation assays.** (**A**) *Ex vivo* patient BPH tissues were transduced with negative control vector pLVX, positive control FLAG-p53, or FLAG-INGs. The expression of these genes was induced with doxycycline. Tissues were fixed and processed for staining by IHC for H3S10^phos^ or FLAG. (**B**) *Ex vivo* patient benign tissues were transduced with control (pLVX) or FLAG-tagged ING3-expressing lentiviral particles. The expression of proliferation marker H3S10^phos^ and ING3 was assessed by IHC. (**C**) Normal human fibroblast were transduced with pLVX or FLAG-ING3 and anchorage-independent growth assessed by colony-formation assays in methylcellulose. The transformation assays were performed in three independent experiments and combined in one graphic with representative pictures. (**D**) As in panel (**C**) but performed in 3T3-L1 cells. By convention, *P<0.05, ***P<0.001, and NS indicates not significant, as calculated by *t*-test. When not indicated, *P*-values are not significant. ING=INhibitor of Growth.

By maintaining native tissue architecture, including epithelia and stroma, *ex vivo* 3D culture of intact tissues is a more representative and robust disease model of cancer than cell line or animal xenograft models (Centenera *et al*, 2012; Centenera *et al*, 2013). To evaluate the proliferative role of ING proteins, we used a doxycycline-inducible lentiviral system composed of a minimal CMV promoter regulated by tetracycline-response elements (TRE). *Ex vivo* tissue cultures of benign prostate hyperplasia (BPH) tissues isolated from three different cancer-free patients were transduced with lentiviral particles and exposed to doxycycline to induce the expression of full-length wild-type FLAG-tagged ING proteins or FLAG-p53, as a known tumour-suppressor control. The expression of INGs and proliferation (mitosis) marker H3S10^phos^ were assessed by IHC. In agreement with ING1b being a tumour suppressor, H3S10^phos^ levels were decreased similarly to p53 compared with the empty vector control (Figure 1A). Interestingly, samples expressing ING4 had increased levels of H3S10^phos^, while H3S10^phos^ was lower in ING5 samples (Figure 1A). These results confirm the tumour-suppressive functions of ING1b and suggest that ING4 may have pleiotropic roles (i.e., reported to be a tumour suppressor but appears to stimulate proliferation in prostate tissues).

Strikingly, ING3-transduced tissues had high levels of H3S10^phos^ (not shown) thus, as ING3 associates with the AR co-activator TIP60, we extended our investigations and transduced ING3 in *ex vivo* tissues from three additional patients and confirmed that ING3 expression enhances H3S10^phos^ levels (Figure 1B), suggesting that ING3 has oncogenic properties. To test the hypothesis that ING3 may be an oncoprotein, we expressed ING3 in NHDF in a classical anchorage-independent growth assay. In agreement with these observations, ING3 expression in NHDF was sufficient to stimulate anchorage-independent growth (Figure 1C), suggesting cellular transformation and further supporting the notion that ING3 is an oncoprotein. To further validate ING3 oncogenic properties, transformation assays were also performed in 3T3-L1 murine fibroblasts using p53 as an established tumour-suppressor control and RASV12 as an established oncoprotein control. As expected, p53-expressing cells did not form colonies, while RASV12-expressing cells formed colonies (Figure 1D). Importantly, ING3 expression also led to colony formation (Figure 1D), confirming that ING3 expression does transform cells.

### Elevated ING3 expression is a marker of poor prognosis in cancer patients

Analysis of various cancer databases (Cerami *et al*, 2012; Gao *et al*, 2013) showed a significant increase in the copy number of *ING3* in breast cancer, melanoma cancer, ovarian cancer, and PC patients. A more thorough analysis of The Cancer Genome Atlas (TCGA) database showed that an increased copy number of *ING3* correlates with development of prostate adenocarcinoma (Supplementary Figure S1A). These results suggest that ING3 may have a role in PC initiation or progression. We thus compared *ING3* copy number with the disease outcome and observed that patients with increased *ING3* copy number were less likely to undergo remission (Supplementary Figure S1B) and relapsed earlier (Supplementary Figure S1C). These results imply a correlation between the amplification of *ING3* and poor outcome for PC patients and a potential application of ING3 as a biomarker.

**Figure 2:**
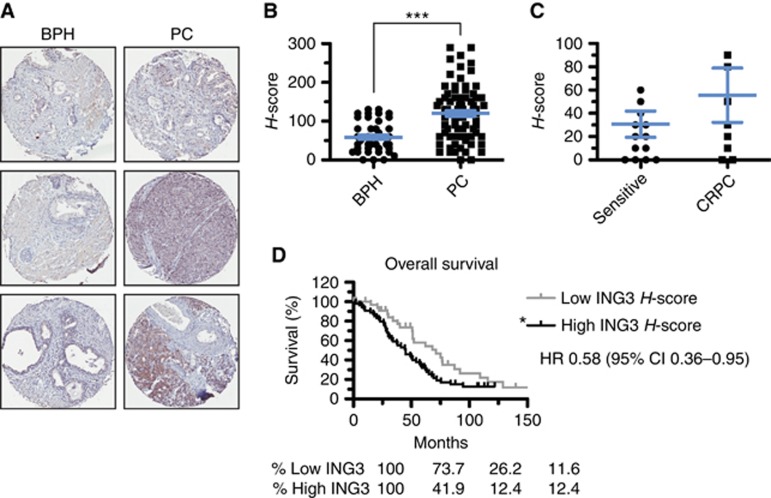
**The expression of ING3 is a poor prognostic marker for PC patients.** (**A**) Prostate tissue microarray (TMA) were stained for ING3 by immunohistochemistry (**B**) TMA were scored for positive staining (histoscore or *H*-score). Data represent *H*-score for ING3 staining of PC (*n*=81) samples and BPH (*n*=41) samples. ***P<0.001. (**C**) ING3 *H*-scores comparison between castration sensitive (*n*=14) and castrate-resistant prostate cancer (CRPC) patients (*n*=9). (**D**) Kaplan–Meier overall survival curve based on patients with low (*n*=35) or high (*n*=55) ING3 protein levels (*P-value of 0.03). All error bars represent the s.e.m. BPH=benign prostatic hyperplasia; ING=INhibitor of Growth; PC=prostate cancer.

To further investigate the expression of ING3 in PC tissues, we used an extensively characterised and validated ING3 antibody, which was also optimised for IHC (Nabbi *et al*, 2015). Specifically, we examined ING3 protein levels by IHC analysis of a TMA (described previously; Coffey *et al*, 2013) from BPH and PC specimens (Figure 2A) and observed elevated protein levels of ING3 in PC when compared with BPH samples (Figure 2B). In addition, ING3 levels were found to be elevated in treatment-resistant compared with castration-sensitive PC patients (Figure 2C). Elevated ING3 protein levels also correlated with decreased overall survival (Figure 2D). Finally, we observed that ING3 levels in the TMA samples correlate with an increase in proliferation markers MCM2, Ki-67, and Geminin (Supplementary Figures S1D–F). Together, our data indicate that *ING3* copy number and ING3 protein levels correlate with cellular proliferation and that ING3 could be used as a biomarker of poor prognosis in PC. Our data strikingly indicate that ING3 does not behave as a tumour suppressor but rather as an oncoprotein.

### Identification of an ING3 transcriptional network

**Figure 3:**
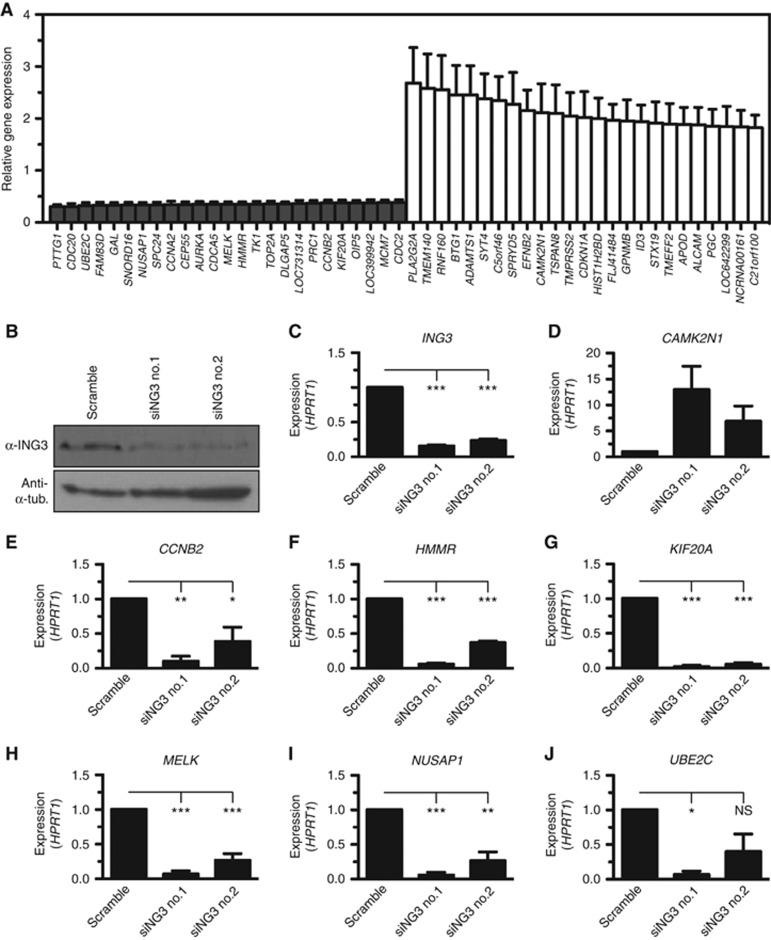
**Identification of an ING3 transcriptional network.** (**A**) The expression of *ING3* was silenced in the LNCaP cell line using two different siRNA in four independent biological replicates, total RNA isolated, and hybridised on Illumina Human HT-12 arrays. The top 25 downregulated and top 25 upregulated genes were plotted. (**B**) Silencing of ING3 was assessed by immunoblotting using the indicated antibodies. (**C**) Silencing of *ING3* was confirmed by real-time qPCR. (**D**–**J**) Hits from the microarray analysis were validated by standard real-time qPCR on reverse transcribed total RNA isolated from control (Scramble siRNA) or ING3-silenced (siING3 no.1 and siING3 no.2) LNCaP cells. Expressed values are the average of three biological replicates, each performed in technical triplicate, normalised to the expression of *HPRT1*, with s.e.m. as error bars (*P<0.05, **P<0.01, ***P<0.001, and NS indicates not significant, as calculated by *t*-test). ING=INhibitor of Growth.

As ING3 associates with methylated histones and chromatin-modifying enzymatic complexes and to understand how ING3 may function as an oncoprotein, we performed a microarray gene expression analysis. Given that ING3 is a potential AR co-regulator, we addressed the question whether ING3 was required for AR-dependent transcription upon androgen stimulation. Following the rationale that DHT would have a minimal impact on AR-independent transcription, the microarray survey was performed on androgen-stimulated LNCaP cells. To validate the specificity of the siRNAs used, the microarray data for each gene was plotted and the overlay of both siING3 no.1 and siING3 no.2 showed nearly identical gene expression profiles (Supplementary Figure S2). Upon silencing the expression of ING3 in the LNCaP PC cell line, we identified a number of genes that were either upregulated or downregulated (see Supplementary Table S3 for complete list). Among the top 25 downregulated genes, cell cycle regulators *CCNA2*, *CCNB2*, *CDC2*, *CDC20*, and *AURKA* were identified (Figure 3A). The silencing of ING3 was confirmed by immunoblotting using an extensively validated antibody (Nabbi *et al*, 2015) (Figure 3B) and real-time qPCR (Figure 3C). Importantly, only a single band appeared on immunoblots and decreased upon ING3 silencing (Figure 3B). Interestingly, the expression of *MELK* highly correlates with cell cycle genes *AURKB*, *CCNB2*, *TOP2A*, and *UBE2C*, which are upregulated in high-grade PC (Kuner *et al*, 2013) and appear to form a transcriptional network whose expression requires ING3 (Figure 3A), suggesting that ING3 regulates this pathway. Similarly, the expression of several genes from the EGFR inside-out pathway (*CCNB2*-*HMMR*-*KIF11*-*NUSAP1*-*PRC1*-*SLC2A1*-*UBE2C*) (Zhou *et al*, 2015) require the expression of ING3 (Figure 3A). The requirement of ING3 for the expression of these genes was validated by real-time qPCR (Figures 3D–J). Although ING3 associates with histone acetyltransferase activity and thus should mainly function as a transcriptional activator, we observed that a number of genes were upregulated in the absence of ING3, including *CAMK2N1* (Figure 3A and D), a gene that inhibits proliferation of PC cells (Wang *et al*, 2014a, b), the cyclin-dependent kinase inhibitor *CDKN1A* (p21^CIP1/WAF1^), and the antiproliferative gene *BTG1* (Figure 3A). Together, these results demonstrate that ING3 directly or indirectly regulates genes involved in cell cycle progression and cell survival.

### Silencing of ING3 expression regulates genes associated with cell cycle progression and slows cellular proliferation

**Figure 4:**
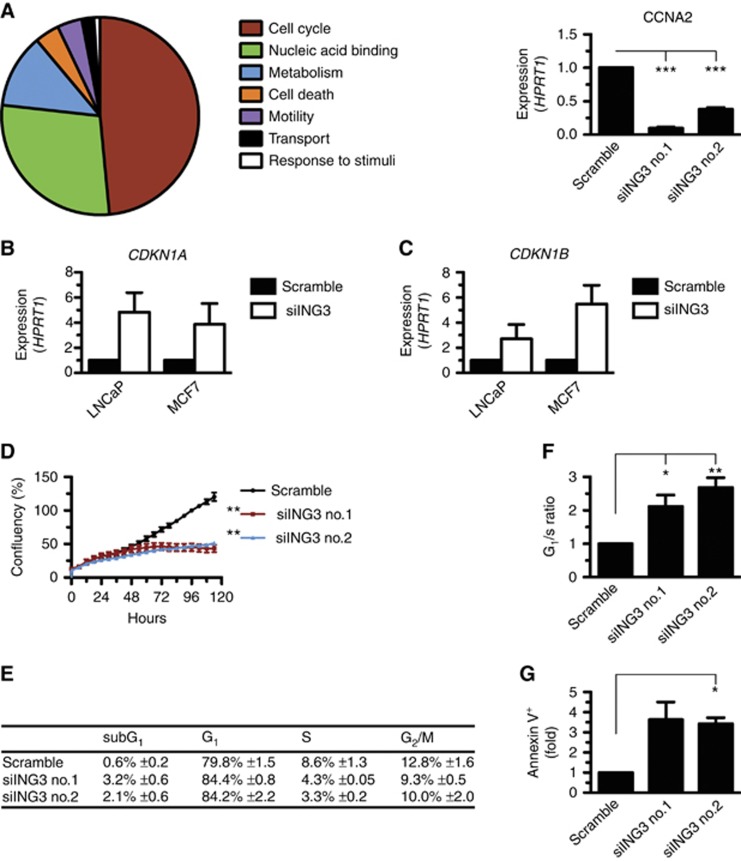
**ING3 regulates genes associated with cell cycle progression.** (**A**) Gene ontology analysis of downregulated genes upon silencing of ING3. A major fraction of genes requiring ING3 for expression is involved in cell cycle regulation. For instance, the expression of cyclin A (*CCNA2*) is impaired in the absence of ING3. (**B**) The expression of *CDKN1A* assessed by real-time qPCR on reverse transcribed total RNA isolated from control (Scramble) or ING3-silenced (siING3) prostate (LNCaP) and breast (MCF7) cells. (**C**) As in (**B**) but for *CDKN1B*. (**D**) Proliferation of control (Scramble siRNA) or ING3-silenced (siING3 no.1 and siING3 no.2) LNCaP cells was monitored for 5 consecutive days by IncuCyte. Data were analysed using Wilcoxon signed-rank test. (**E**) Flow cytometric analysis of control (Scramble siRNA) or ING3-silenced (siING3 no.1 and siING3 no.2) LNCaP cells was conducted and (**F**) G_1_/S ratio was calculated. (**G**) Control (Scramble siRNA) or ING3-silenced (siING3 no.1 and siING3 no.2) LNCaP cells were stained for Annexin V and analysed by flow cytometry. By convention, *P<0.05, **P<0.01, ***P<0.001, and NS indicates not significant, as calculated by *t*-test. When not indicated, *P*-values are not significant.

In agreement with ING3-stimulating cellular proliferation (Figure 2E), close to half of the genes found to be affected by the depletion of ING3 are involved in cell cycle progression, including cyclin A (*CCNA2*) (Figure 3A and 4A). Also, about a quarter are DNA-binding factors, and a small, but significant, portion of genes regulate cell death. We validated by real-time qPCR the increased expression of *CDKN1A* (Figure 4B) and *CDKN1B* (Figure 4C) in PC and breast cancer cells.

Although the exogenous expression of ING3 in various cell lines presumably induces proliferation defects (Nagashima *et al*, 2003; Chen *et al*, 2010), little is known about the roles of ING3 in the control of cellular proliferation and the molecular mechanisms underlying its functions. To determine the potential role of ING3 in regulating cellular proliferation, we silenced the expression of ING3. Consistent with *ex vivo* experiments (Figure 1B), silencing the expression of ING3 reproducibly led to severe proliferation defects in PC cells. Specifically, LNCaP (DHT sensitive (DHT^s^)) cells treated with either siING3 no.1 or no.2 barely proliferated over a 5-day period (Figure 4D). Interestingly, silencing of ING3 expression had comparable consequences on the proliferation of the androgen-independent LNCaP-AI (DHT^i^) isogenic cell line (Supplementary Figure S3). Similarly, the proliferation of PC (CWR22Rv1 and PC3), breast cancer (MCF7 and MX-1), and ovarian cancer (PEO1 and PEO4) cell lines (see Supplementary Table S4 for characteristics) were also affected by silencing of ING3 (Supplementary Figure S3). To further investigate the potential causes of cellular proliferation defects induced by the silencing of ING3, we performed cell cycle analysis of the LNCaP cell line. In agreement with slower proliferation rates (Figure 4D), ING3-silenced cells had a marked increase in the G_0_/G_1_ phase population accompanied by lower S and G_2_/M populations (Figure 4E). The increased G_0_/G_1_ population translates into an increase in G_1_/S ratio (Figure 4F), a hallmark of cell cycle arrest. Furthermore, we observed a small but consistent increase in the subG_1_ population (Figure 4E), suggesting that the silencing of ING3 may induce apoptosis. Indeed, Annexin V staining of ING3-silenced cells showed a threefold increase in apoptotic cells (Figure 4G), consistent with severely reduced proliferation as well as accumulation of G_1_/S and subG_1_ populations (Figures 4D–F). These results demonstrate that ING3 is essential for the proliferation of a broad range of cancer cell types, further demonstrating the oncogenic properties of ING3.

### ING3 regulates the expression of androgen-responsive genes

**Figure 5:**
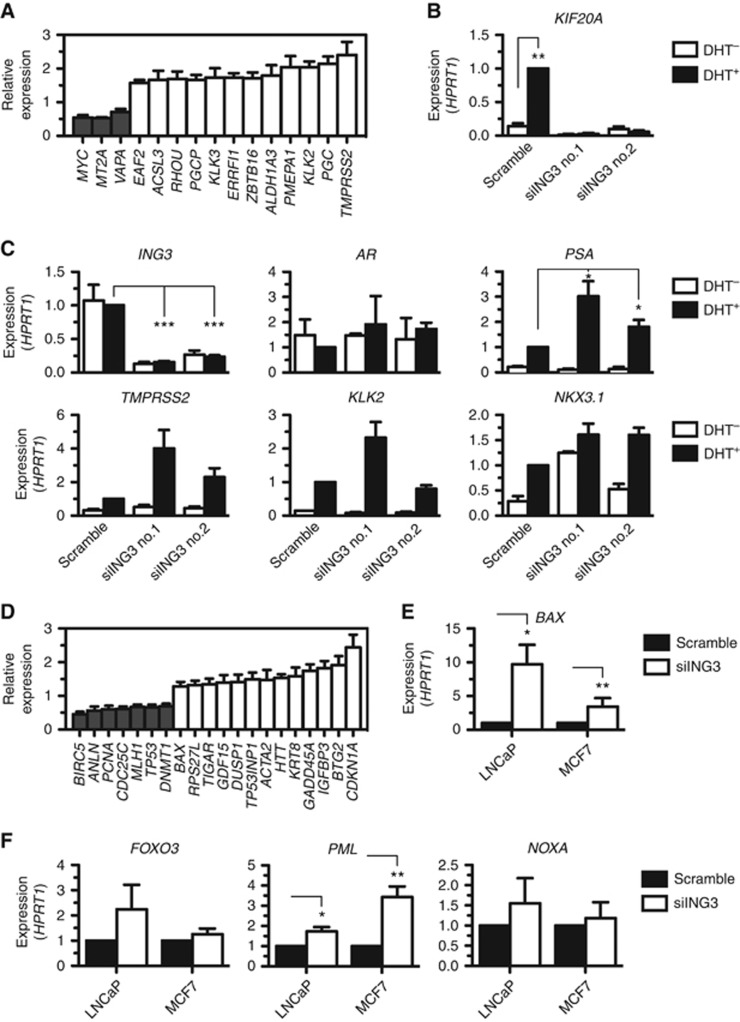
**ING3 regulates a subset of AR-responsive genes and p53-responsive genes.** (**A**) The microarray gene expression analysis identified a number of AR-target genes that are regulated by ING3. (**B**) A novel androgen-responsive gene *KIF20A* was found to be regulated by ING3. (**C**) The silencing of *ING3* in the absence (DHT^−^) or presence (DHT^+^) of androgen stimulation was assessed by real-time qPCR. The expression of *AR* was measured in *ING3*-depleted cells. Validation by real-time qPCR of the effect of ING3 depletion on the expression of known AR-responsive genes. (**D**) The microarray gene expression analysis identified a number of ING3-regulated genes that are known p53-responsive genes. (**E**) The expression of p53-responsive gene *BAX* was assessed from LNCaP and MCF7 cells by real-time qPCR. (**F**) Validation by real-time qPCR of the effect of ING3 depletion on the expression of known p53-responsive genes in LNCaP and MCF7 cells. All experiments were conducted three times (*n*=3 biological replicates) in triplicate (technical replicates). The values represent the average of *n*=3 with s.e.m. as error bars. By convention, *P<0.05, **P<0.01, ***P<0.001, and NS indicates not significant, as calculated by *t*-test. When not indicated, *P*-values are not significant.

Hypothetically, ING3 could regulate AR-dependent transcription via the acetyltransferase TIP60 (Brady *et al*, 1999; Doyon *et al*, 2004, 2006), which regulates cellular proliferation and the expression of cell cycle genes. We thus performed an extensive survey of known AR-regulated genes from the ING3 transcriptional network. We identified three genes requiring the expression of ING3 for normal expression and a dozen that were stimulated in the absence of ING3 (Figure 5A). To assess whether androgens affect ING3-regulated genes, all validation experiments were performed with and without DHT. Interestingly, through validation of the microarray analysis, we identified a novel androgen-responsive gene, *KIF20A*, which is stimulated by DHT, but requires ING3 for expression (Figure 5B). Reduced levels of ING3 (Figure 5C) did not affect the expression of the *AR* itself (Figure 5C) but nonetheless appeared to further stimulate the DHT-induced expression of androgen-responsive genes such as *PSA* (*KLK3*), *TMPRSS2*, and *KLK2* (only with siING3 no.1) (Figure 5C), while having inconclusive effects on the expression of other androgen-responsive genes, such as *NKX3.1* (Figure 5C).

### ING3 regulates the expression of a subset of p53-responsive genes

Given the potential role of ING3 in the regulation of p53-dependent transcription (Nagashima *et al*, 2003), which also regulates the expression of cell cycle genes, proliferation, and apoptosis, we compared known p53-responsive genes (Riley *et al*, 2008) to the list of genes identified in the ING3 transcriptional network (highlighted in Supplementary Figure S4). Out of the 129 known p53-responsive genes (Riley *et al*, 2008), 7 were downregulated (5%) and 13 upregulated (10%) following the silencing of ING3 expression (Figure 5D). The increased expression of *BAX* (Figure 5D) was validated by real-time qPCR in both LNCaP and breast cancer MCF7 cell lines (Figure 5E). Notably, the induced expression of *CDKN1A* (Figure 5D) had already been validated in LNCaP and MCF7 (Figure 4B). The expression of other p53-regulated genes not identified in the microarray analysis was also investigated. In particular, we found that the transcription of *FOXO3*, *PML*, and NOXA was activated by the silencing of ING3 in LNCaP (Figure 5F), whereas only *PML* was induced in MCF7 (Figure 5F). These results, alongside published work (Nagashima *et al*, 2003), suggest that ING3 functions as a p53 co-factor for a subset of genes.

### Characterisation of ING3 interaction with H3K4^me3^

**Figure 6:**
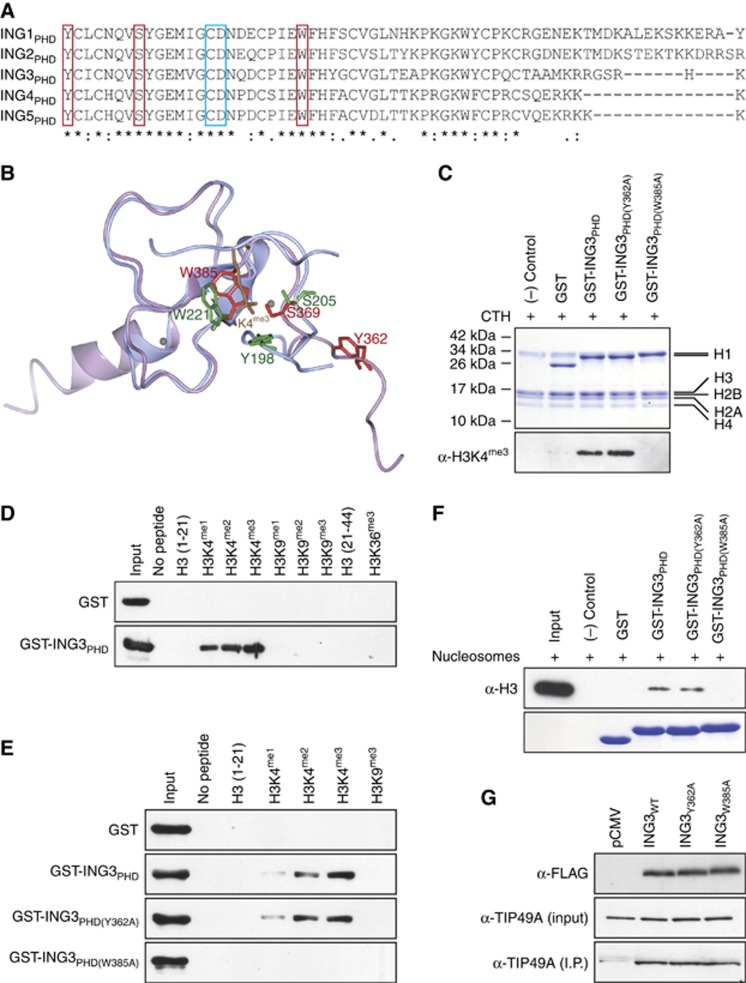
**The aromatic cage of ING3 mediates interactions with H3K4^me3^.** (**A**) Alignment of the PHD from all the members of the ING family. Amino acids boxed in red form the aromatic cage that encloses the side chain of H3K4^me3^, while amino acids boxed in blue restrain the unmodified side chain of H3R2. *P<0.05. (**B**) The structure of ING3_PHD_ (lilac) was predicted using Phyre^2^ and overlaid with the published structure of H3K4^me3^-bound ING4_PHD_ (blue). (**C**) The aromatic cage amino acids Y362 and W385 of ING3 were converted to alanine. The ING3_PHD_ and aromatic cage mutants ING3_PHD[Y362A]_ and ING3_PHD[W385A]_ were expressed in *Escherichia coli* BL21, affinity purified, and used in pulldown assays to assess the interaction between ING3_PHD_ and calf thymus histones (CTH). The top panel represents a Coomassie stained gel showing the level of ING3_PHD_ forms and CTH used (input). The lower panel represents an *α*-H3K4^me3^ immunoblot of a representative pulldown experiment. (**D**) GST or GST-ING3_PHD_ were incubated with the indicated biotinylated histone peptides and pulled down using streptavidin-sepharose. Histone peptide-bound GST proteins were detected by immunoblotting using an *α*-GST antibody. (**E**) As in (**D**) but ING3_PHD[Y362A]_ and ING3_PHD[W385A]_ were also used. (**F**) GST pulldown assay as in (**C**), but crude nucleosomes isolated from HEK293T cells were used. Binding of ING3_PHD_ to nucleosomes was assessed by immunoblotting using *α*-H3 (top) and the level of ING3_PHD_ forms was assessed by Coomassie staining. (**G**) HEK293T were transfected with empty vector (pCMV) or FLAG-tagged ING3-expressing constructs, as indicated, and *α*-FLAG immunoprecipitates were analysed with *α*-FLAG or *α*-TIP49A antibodies.

As the PHD of ING proteins has a central role in controlling proliferation, survival, and other cellular functions, we investigated this key region within ING3. The PHD of ING3 (ING3_PHD_) shares extensive primary amino-acid sequence similarity with other family members (Figure 6A). Specifically, tyrosine 362 (Y362), serine 369 (S369), and tryptophan 385 (W385) (Figure 6A, red boxes) are perfectly conserved with amino acids that form an H3K4^me3^-binding aromatic cage in other ING proteins (Shi *et al*, 2006; Hung *et al*, 2009). Thus we predicted the structure of ING3_PHD_ using Phyre^2^ (Kelley and Sternberg, 2009) and superimposed it with the structure of ING4_PHD_-bound to H3K4^me3^ using CCP4mg (McNicholas *et al*, 2011) (Figure 6B). The overlay of ING3_PHD_ and ING4_PHD_ suggests that Y362, S369, and W385 of ING3 would indeed form an aromatic cage similar to ING4 and thus mediate interactions with H3K4^me3^. To confirm their role in H3 binding, the Y362 and W385 sites were converted to alanine (A) and used to investigate the interaction between ING3_PHD_ and histones purified from calf thymus. This experiment confirmed that the PHD of ING3 does bind to histone H3 (Figure 6C). In addition, the failure of ING3_PHD[W385A]_ to bind to H3 (Figure 6C) suggests that the aromatic amino-acid residue is responsible for interactions with H3K4^me3^, as predicted (Figures 6A and B). However, ING3_PHD[Y362A]_ retained wild-type H3-binding capacity (Figure 6C). The interaction between ING3_PHD_ and H3 was further investigated using synthetic H3 peptides harboring mono-, di-, or tri-methyl groups at K4 or K9, as well as unmodified or tri-methylated H3K36. As expected, ING3_PHD_ specifically bound the methylated H3K4 forms, with an affinity increasing (H3^unmod^<<H3K4^me1^<H3K4^me2^<H3K4^me3^) concomitantly with the methylation state of K4 (Figure 6D). Interestingly, both ING3_PHD_ and ING3_PHD[Y362A]_ associated more stably with H3K4^me2/3^, while the ING3_PHD[W385A]_ mutant completely failed to interact with any histone modifications assessed (Figure 6E). Similar interaction results were obtained between ING3 and nucleosomes, where ING3_Y362A_ retained wild-type binding to H3, whereas ING3_W385A_ failed to associate with H3 (Figure 6F). For subsequent gene expression and chromatin immunoprecipitation (ChIP) experiments, it is worth mentioning that both ING3_Y362A_ and ING3_W385A_ forms retain wild-type capacity to associate with subunits of the TIP60 complex, such as TIP49A (Figure 6G). Together, these results establish that the PHD of ING3 associates with methylated H3K4 and requires conserved amino-acid residues that, based on sequence similarities to other ING protein and structure prediction, form an aromatic cage.

### ING3 associates with the TSS of cell cycle genes

**Figure 7:**
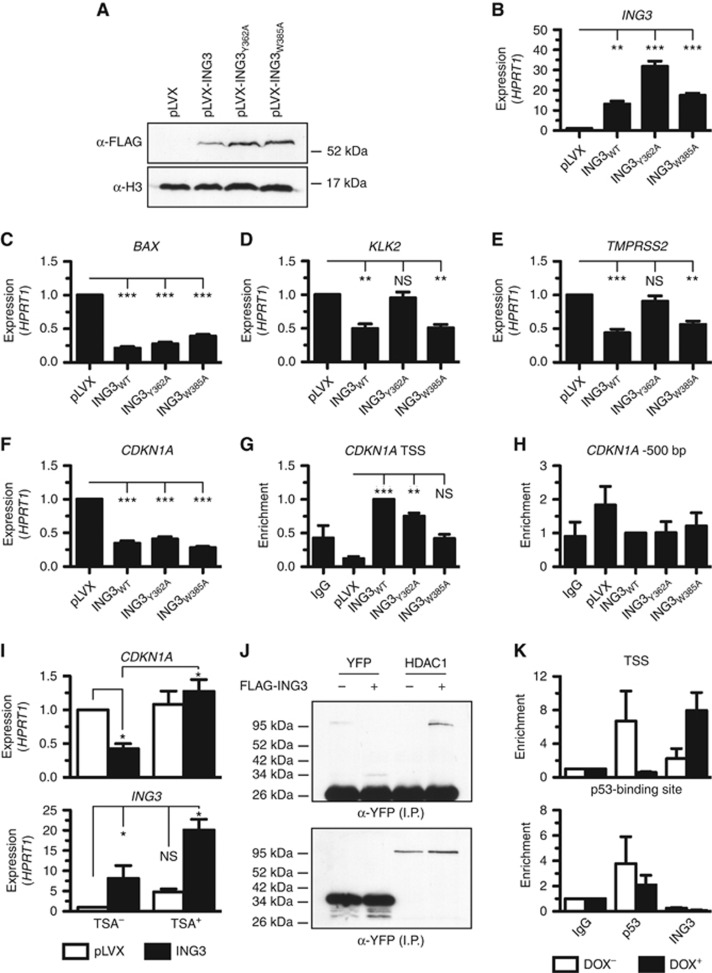
**ING3 associates with H3K4^me3^ at TSSs.** (**A**) A tetracycline-inducible system was used to express FLAG-tagged ING3 (predicted molecular weight of ∼50 kDa) forms in LNCaP cells. (**B**) The expression of *ING3*, (**C**) *BAX*, (**D**) *KLK2*, (**E**) *TMPRSS2*, and (**F**) *CDKN1A* was assessed by real-time qPCR from total RNA isolated from ING3-expressing LNCaP cells. (**G**) ChIP experiments of FLAG-ING3 at the TSS of *CDKN1A* using a FLAG antibody. (**H**) As in (**G**), but real-time qPCR assay was performed at a control region about 500 base pairs (−500 bp) from the TSS of *CDKN1A* (**P<0.01, ***P<0.001, and NS indicates not significant, as calculated by *t*-test). (**I**) FLAG-ING3 was expressed as in (**B**–**F**), but in the absence (TSA^−^) or presence (TSA^+^) of the histone deacetylase Trichostatin A (TSA), then the expression of *CDKN1A* was assessed by real-time qPCR. (**J**) FLAG-ING3 and YFP-HDAC1 were co-expressed in HEK293T cells, immunoprecipitated with *α*-FLAG M2 agarose, and analysed by immunoblotting using *α*-GFP antibody (recognises all GFP variants). (**K**) ChIP experiments were conducted in U2OS cells in the absence (DOX^−^) or presence (DOX^+^) of doxorubicin. By convention, *P<0.05, **P<0.01, ***P<0.001, and NS indicates not significant, as calculated by *t*-test. When not indicated, *P*-values are not significant. ING=INhibitor of Growth; TSS=transcriptional start site.

We have so far demonstrated that ING3 is required for the expression of an intricate transcriptional network involved in regulating the proliferation of cancer cells. However, ING3 may directly or indirectly regulate the expression of these genes. We thus performed ChIP experiments to investigate the association of ING3 with genes that were identified in our gene expression survey (Figure 3). Also, as the PHD of ING3 binds H3K4^me3^ (Figure 6), a modification marking the TSS of most genes (Barski *et al*, 2007), and a similar molecular mechanism for the regulation of gene expression was described for ING2 (Shi *et al*, 2006) and ING4 (Hung *et al*, 2009), we investigated that region in particular. Upon induction of ING3 expression (Figures 7A and B), the expression of *BAX*, *KLK2*, *TMPRSS2*, and *CDKN1A* (genes induced in ING3-silenced cells) was repressed (Figures 7C–F). Interestingly, ING3_WT_ and ING3_Y362A_ were found at the TSS of *CDKN1A*, while the H3K4^me3^-defective mutant ING3_W385A_ was absent from this region (Figure 7G). Importantly, all ING3 forms were absent from a control region situated approximately 500 base pairs upstream (−500 bp) of the TSS (Figure 7H). In agreement with ING3 repressing the expression of *CDKN1A*, inhibition of HDAC activity with trichostatin A (TSA) completely relieved ING3-mediated transcriptional repression of *CDKN1A* (Figure 7I). An HDAC-dependent and TSA-sensitive transcriptional repression mechanism was further explored, and we found that ING3 associates with HDAC1 (Figure 7J). Given that ING proteins often cooperate with p53, we investigated whether p53 and ING3 could occupy the same sites on the *CDKN1A* promoter in U2OS human osteosarcoma cells (wild-type p53). Interestingly, ChIP experiments revealed that both p53 and ING3 occupied the TSS of *CDKN1A* (Figure 7K). Moreover, in response to DNA damage induced by doxorubicin, p53 vacated the TSS while ING3 bound more avidly (Figure 7K). Despite this dynamic occupancy at the TSS, ING3 did not occupy the p53-binding site upstream of *CDKN1A* (Figure 7K).

In addition, further supporting the role of ING3 in regulating the expression of cell cycle genes, ING3 was also found at the TSS of *CCND1* and *PCNA* but remained undetectable at both the androgen response element (ARE) ARE_III_ of *PSA* and the ARE of *KLK2* (Supplementary Figure S5). Interestingly, the presence of AR at the ARE of *KLK2* was reduced when ING3 was expressed (Supplementary Figure S5), potentially explaining the increased *KLK2* expression observed in ING3-silenced cells (Figure 5C). These results suggest that ING3 indirectly regulates the expression of AR-regulated genes (e.g., *KLK2* and *PSA*). However, ING3 also appears to use a direct mechanism to regulate gene expression involving the binding to H3K4^me3^ at the TSS of cell cycle genes (e.g. *CDKN1A*) to regulate their expression. Taken together with the histone interaction studies (Figure 6), these results demonstrate that ING3 associates with H3K4^me3^ at the TSS of cell cycle genes via its PHD to regulate gene expression.

In conclusion, ING3 associates with TSSs to regulate the expression of an intricate transcriptional network involving cell cycle genes as well as AR- and p53-dependent genes to control the proliferation of cancer cells. Elevated levels of ING3 correlate with increased cellular proliferation and lower survival of PC patients and, as such, represents a novel marker of poor prognosis for PC patients and herein redefined as an oncoprotein.

## Discussion

Genetic deletion of the *Ing1* locus in a mouse model leads to early onset and increased incidence of lymphomas (Kichina *et al*, 2006). The *Ing1*^−/−^ animals also harbour increased sensitivity to *γ*-radiation (Kichina *et al*, 2006). Deletion of the p37Ing1 isoform in a mouse model causes spontaneous development of follicular B-cell lymphomas (Coles *et al*, 2007). Similarly, the *Ing2*^−/−^ animals have a threefold higher incidence of soft-tissue sarcomas (Saito *et al*, 2010), providing concrete evidence that at least Ing1 and Ing2 are bona fide tumour suppressors. However, the evidence that ING3-5 are tumour suppressors is minimal and largely based on adenovirus delivery and supraphysiological overexpression or correlative expression studies. Indeed, silencing of *ING4* prevents the transition from the G_2_/M phase to G_1_ phase of the cell cycle, while silencing of *ING5* results in S-phase blockade (Doyon *et al*, 2006). Moreover, the *Ing4*^−/−^ mouse model does not develop cancer under reported experimental conditions (Coles *et al*, 2010), suggesting that these proteins are not tumour suppressors, or have complex yet unidentified cellular functions. Indeed, as recently reported by one of our groups, ING3 is expressed preferentially in proliferating cells (Nabbi *et al*, 2015), suggesting that ING3 may not inhibit growth but rather drive cellular proliferation. In agreement, we have found elevated levels of ING3 in various cancers and using an *ex vivo* human tissue explant model demonstrated that ING3 does have oncogenic properties by stimulating cellular proliferation. Precisely, our data demonstrate that (i) ING3 levels are elevated in PC patients and correlate with poor outcome; (ii) ING3 is required for the proliferation of breast cancer, ovarian cancer, and PC cell lines; and (iii) ING3 expression is sufficient to elicit anchorage-independent growth. Together, these results strongly suggest that ING3 functions as an oncoprotein.

Herein we have identified ING3 as a cellular proliferation-regulating factor. Interestingly, loss of ING3 expression led to decreased expression of *MELK*, *UBE2C*, *TOP2A*, *CCNB2*, and *AURKB*. These genes have been reported to be highly expressed in high-grade PC patients (Kuner *et al*, 2013), while *CAMK2N1* forms a transcriptional network with *NUSAP*, *UBE2C*, and *HMMR* whose expression regulates proliferation of treatment-resistant cancer cells (Wang *et al*, 2014a). In addition, the silencing of ING3 expression led to proliferation defects in a wide array of cancer cell models, including treatment-sensitive and -resistant PC cell lines, suggesting that inactivation of ING3 functions could be a viable therapeutic avenue to eliminate cancer cells in early as well as in advanced cases.

Notably, silencing of ING3 resulted in decreased *AURKA* and *AURKB* expression (Supplementary Table S3), two H3S10 kinases, in agreement with increased H3S10^phos^ in cells expressing ING3 (Figure 1B).

Consistent with previous studies (Kim *et al*, 2016) showing that the ING3_PHD[Y362A]_-H3K4^me3^ interaction has a *K*_d_ of 46 μM, compared with 0.9 μM for ING3_PHD_-H3K4^me3^, 3.0 μM for ING3_PHD_-H3K4^me2^, and 23 μM for ING3_PHD_-H3K4^me1^, we found that ING3_PHD[Y362A]_ retains H3K4^me3^-binding capacity (Figures 6C, E, and F). Interestingly, the ING3_PHD[S369A]_–H3K4^me3^ interaction, with a *K*_d_ of 1.5 μM, also retains binding capacity (Kim *et al*, 2016). Although highly conserved, Y362 is dispensable for the ING3_PHD_–H3K4^me3^ interaction, unlike the corresponding Y198 in ING4_PHD_ (Hung *et al*, 2009) or Y214 in ING2_PHD_ (Shi *et al*, 2006). The Phyre^2^-predicted structures of ING3_PHD_ suggest that Y362 may be positioned away from the aromatic cage (Figure 6B and Supplementary Figure S6), leaving the binding burden on W385 and a different mode of interaction to methyl-lysine. Interestingly, the structure predictions including or excluding PNEPR peptide preceding Y362 suggest that the sequence located on the amino side of the PHD may be flexible and thus may fold upon binding to H3K4^me3^. Further structural biology investigations will be required to elucidate the precise mode of interaction between ING3_PHD_ and H3K4^me3^ and how it differs from other INGs.

The importance of the role played by ING3 in tightly controlling cellular proliferation of cancer cells is also highlighted by the loss of expression of several cyclin genes (*CCNA2*, *CCNB1*, *CCNB2*, and *CCND1*) and gain of cell cycle inhibitors (*CDKN1A* and *CDKN1B*) as well as apoptotic genes (*BAX*) concomitant with the silencing of ING3. Importantly, ChIP analysis located ING3 at the TSS of genes upregulated (i.e., *CDKN1A*) and downregulated (e.g., *CCND1*) in the absence of ING3, demonstrating that ING3 has a direct role in controlling the expression of genes associated with cellular proliferation. However, as ING3-regulated genes can be either negatively or positively regulated in the absence of ING3, there would be more than one transcriptional mechanism involved. Potentially, ING3 may positively regulate gene expression via TIP60 histone acetyltransferase activity (Doyon *et al*, 2004) and negatively regulate transcription via an ANP32E histone exchange mechanism (Obri *et al*, 2014) or via HDAC1, which we found in association with ING3 (Figure 7J). Indeed, the ING3-mediated transcriptional repression of *CDKN1A* was completely relieved by the deacetylase inhibitor Trichostatin A (Figures 7I), demonstrating that ING3 can silence gene expression via histone deacetylation. Future investigations should address the acetylation state of histones at ING3-bound chromatin sites as well as the presence of histone variants deposited by ANP32E. Interestingly, according to the TCGA database and in agreement with our microarray analysis, the expression of several cell cycle genes correlated in a similar way with the expression of ING3 in human PC (Supplementary Figure S7). Although ING3 was previously reported to activate the p53-responsive gene *CDKN1A* using a transient luciferase reporter assays in colon carcinoma cells (Nagashima *et al*, 2003; Doyon *et al*, 2006), we observed that silencing of ING3 induced the expression of *CDKN1A* in breast cancer and PC cells (Figure 4B), whereas exogenous expression of ING3 driven by a doxycycline-inducible system repressed the expression of *CDKN1A* (Figures 7F and I). We can only speculate on the causes of these discrepancies, but they are most likely due to the different cell lines or systems used. Although several ING proteins have been reported to associate with p53, we failed to observe any interactions between p53 and ING3 in LNCaP cells under mild immunoprecipitation conditions. In ChIP experiments at *CDKN1A*, we found p53 at the p53-binding site, but ING3 was not detected. In addition, p53 could be detected at the TSS, while ING3 levels were low, but under stressed conditions, p53 was released and ING3 occupied the TSS more avidly (Figure 7K). Together, these results suggest that p53 and ING3 do not occupy chromatin regions at the same time in agreement with the observation that the two factors do not seem to interact.

Although ING3 associates with TIP60 (Doyon *et al*, 2006) and AR (Nabbi *et al*, 2017), this interaction occurs in the cytoplasm and regulates acetylation-dependent AR shuttling (Nabbi *et al*, 2017). Moreover, although AR was present at the ARE of *KLK2* and *KLK3* (PSA), ChIP experiments did not detect enrichment of ING3 at these sites (Supplementary Figure S5), suggesting that ING3 is not recruited via AR. Indeed, ING3 associates with the TSS (H3K4^me3^ marked region) of genes while AR binds the ARE regions, thus suggesting that ING3 can also regulate AR-responsive transcription independently of AR. Indeed, our silencing experiments (Figures 4D–G and Supplementary Figure S3) clearly show that ING3 is required for the proliferation of both AR^+^ (e.g., LNCaP) and AR^−^ (e.g., PC3) cells, demonstrating that ING3, although able to regulate some AR-responsive genes, regulates cell proliferation through AR-independent pathways. Indeed, ING3 regulates the expression of p53-responsive genes (Figures 5D–F) as well as several cell cycle genes (Figure 4A).

Although the ING3 siRNAs had consistent effects on most AR-responsive genes (e.g., *KIF20A*, *PSA*, *TMPRSS2*), they had different effects on *KLK2*. Interestingly, *KLK2* and *KLK3* (PSA) are located ∼12 kb apart on chromosome 19. However, the chromatin landscape seems highly divergent. Specifically, a clear TSS (H3K4^me3^ double peak) is found at *KLK2*, whereas no such regulatory element is found in the ENCODE project data at *KLK3* and very few transcription factors bind the *KLK3* locus compared with *KLK2*. We hypothesise that the difference in *KLK2* and *KLK3* response to ING3 siRNAs may be a reflection of different mechanisms regulating these genes.

In agreement with ING4 and ING5 being required for normal cell cycle progression (Doyon *et al*, 2006), we observed that ING3 is also required for cellular proliferation of cancer cells. Importantly, our ING3-dependent proliferation results are in line with a recent publication showing that ING3 protein levels correlate with the proliferation status of cells (Nabbi *et al*, 2015). Our data from cell line models showing the requirement of ING3 for the proliferation of cancer cells are also in agreement with the elevated levels of ING3 in a subset of tumours in cancer patients. The requirement of ING3 for the proliferation of breast cancer, ovarian cancer, and PC cells and its elevated levels in prostate tumours, which also correlate with poor survival, define ING3 as a novel marker of poor prognosis and a potential therapeutic target. Overexpression-based studies and sequence similarities to the tumour-suppressor ING1 led to the hypothesis that ING3 may be a tumour suppressor. However, our investigations using a more physiologically relevant *ex vivo* explant model define ING3 as an oncoprotein, which is supported by transformation and proliferation assays.

Although mutations of ING proteins are rarely reported (Cerami *et al*, 2012; Gao *et al*, 2013), there are many cases of either amplification or deletion (Supplementary Figure S8). In agreement with our observations that ING3 levels are elevated in PC compared with BPH patient samples, most PC databases report ING3 amplification (Supplementary Figure S8). Interestingly, one case of mutation could be found in a PC patient, a nonsense mutation at E125, which likely results in a truncated form lacking the PHD and 70% of the protein.

Together, our data demonstrate that ING3 is required for proliferation of cancer cells via an intricate transcriptional network involving cell cycle regulators, androgen, and p53 signalling pathways. Importantly, elevated ING3 expression in cancer patients and correlation with poor survival support ING3 as a biomarker and potentially a therapeutic target in both early and advanced treatment-resistant cancer patients. Our results redefine ING3 as an INducer of Growth (ING) and an oncoprotein.

## References

[bib1] Barski A, Cuddapah S, Cui K, Roh T-Y, Schones DE, Wang Z, Wei G, Chepelev I, Zhao K (2007) High-resolution profiling of histone methylations in the human genome. Cell 129(4): 823–837. 17512414 10.1016/j.cell.2007.05.009

[bib2] Brady ME, Ozanne DM, Gaughan L, Waite I, Cook S, Neal DE, Robson CN (1999) Tip60 is a nuclear hormone receptor coactivator. J Biol Chem 274(25): 17599–17604. 10364196 10.1074/jbc.274.25.17599

[bib3] Bua DJ, Binda O (2009) The return of the INGs, histone mark sensors and phospholipid signaling effectors. Curr Drug Targets 10(5): 418–431. 19442114 10.2174/138945009788185112

[bib4] Burska UL, Harle VJ, Coffey K, Darby S, Ramsey H, O'Neill D, Logan IR, Gaughan L, Robson CN (2013) Deubiquitinating enzyme Usp12 is a novel co-activator of the androgen receptor. J Biol Chem 288(45): 32641–32650. 24056413 10.1074/jbc.M113.485912 PMC3820899

[bib5] Centenera MM, Gillis JL, Hanson AR, Jindal S, Taylor RA, Risbridger GP, Sutherland PD, Scher HI, Raj GV, Knudsen KE, Yeadon T, Tilley WD, Butler LM (2012) Evidence for efficacy of new Hsp90 inhibitors revealed by *ex vivo* culture of human prostate tumors. Clin Cancer Res 18(13): 3562–3570. 22573351 10.1158/1078-0432.CCR-12-0782

[bib6] Centenera MM, Raj GV, Knudsen KE, Tilley WD, Butler LM (2013) *Ex vivo* culture of human prostate tissue and drug development. Nat Rev Urol 10(8): 483–487. 23752995 10.1038/nrurol.2013.126

[bib7] Cerami E, Gao J, Dogrusoz U, Gross BE, Sumer SO, Aksoy BA, Jacobsen A, Byrne CJ, Heuer ML, Larsson E, Antipin Y, Reva B, Goldberg AP, Sander C, Schultz N (2012) The cBio cancer genomics portal: an open platform for exploring multidimensional cancer genomics data. Cancer Discov 2(5): 401–404. 22588877 10.1158/2159-8290.CD-12-0095 PMC3956037

[bib8] Chen G, Wang Y, Garate M, Zhou J, Li G (2010) The tumor suppressor ING3 is degraded by SCF(Skp2)-mediated ubiquitin-proteasome system. Oncogene 29(10): 1498–1508. 19935701 10.1038/onc.2009.424

[bib9] Coffey K, Rogerson L, Ryan-Munden C, Alkharaif D, Stockley J, Heer R, Sahadevan K, O'Neill D, Jones D, Darby S, Staller P, Mantilla A, Gaughan L, Robson CN (2013) The lysine demethylase, KDM4B, is a key molecule in androgen receptor signalling and turnover. Nucleic Acids Res 41(8): 4433–4446. 23435229 10.1093/nar/gkt106 PMC3632104

[bib10] Coles AH, Gannon H, Cerny A, Kurt-Jones E, Jones SN (2010) Inhibitor of growth-4 promotes IkappaB promoter activation to suppress NF-kappaB signaling and innate immunity. Proc Natl Acad Sci USA 107(25): 11423–11428. 20534538 10.1073/pnas.0912116107 PMC2895088

[bib11] Coles AH, Liang H, Zhu Z, Marfella CGA, Kang J, Imbalzano AN, Jones SN (2007) Deletion of p37Ing1 in mice reveals a p53-independent role for Ing1 in the suppression of cell proliferation, apoptosis, and tumorigenesis. Cancer Res 67(5): 2054–2061. 17332334 10.1158/0008-5472.CAN-06-3558 PMC2872148

[bib12] Doyon Y, Cayrou C, Ullah M, Landry A-J, Côté V, Selleck W, Lane WS, Tan S, Yang X-J, Côté J (2006) ING tumor suppressor proteins are critical regulators of chromatin acetylation required for genome expression and perpetuation. Mol Cell 21(1): 51–64. 16387653 10.1016/j.molcel.2005.12.007

[bib13] Doyon Y, Selleck W, Lane WS, Tan S, Côté J (2004) Structural and functional conservation of the NuA4 histone acetyltransferase complex from yeast to humans. Mol Cell Biol 24(5): 1884–1896. 14966270 10.1128/MCB.24.5.1884-1896.2004 PMC350560

[bib14] Ferlay J, Soerjomataram I, Dikshit R, Eser S, Mathers C, Rebelo M, Parkin DM, Forman D, Bray F (2015) Cancer incidence and mortality worldwide: sources, methods and major patterns in GLOBOCAN 2012. Int J Cancer 136(5): E359–E386. 25220842 10.1002/ijc.29210

[bib15] Ferraldeschi R, Welti J, Luo J, Attard G, de Bono JS (2015) Targeting the androgen receptor pathway in castration-resistant prostate cancer: progresses and prospects. Oncogene 34(14): 1745–1757. 24837363 10.1038/onc.2014.115 PMC4333106

[bib16] Gao J, Aksoy BA, Dogrusoz U, Dresdner G, Gross B, Sumer SO, Sun Y, Jacobsen A, Sinha R, Larsson E, Cerami E, Sander C, Schultz N (2013) Integrative analysis of complex cancer genomics and clinical profiles using the cBioPortal. Sci Signal 6(269): pl1. 23550210 10.1126/scisignal.2004088 PMC4160307

[bib17] Garkavtsev I, Kazarov A, Gudkov A, Riabowol K (1996) Suppression of the novel growth inhibitor p33ING1 promotes neoplastic transformation. Nat Genet 14(4): 415–420. 8944021 10.1038/ng1296-415

[bib18] Gaughan L, Logan IR, Cook S, Neal DE, Robson CN (2002) Tip60 and histone deacetylase 1 regulate androgen receptor activity through changes to the acetylation status of the receptor. J Biol Chem 277(29): 25904–25913. 11994312 10.1074/jbc.M203423200

[bib19] Hung T, Binda O, Champagne KS, Kuo AJ, Johnson K, Chang HY, Simon MD, Kutateladze TG, Gozani O (2009) ING4 mediates crosstalk between histone H3 K4 trimethylation and H3 acetylation to attenuate cellular transformation. Mol Cell 33(2): 248–256. 19187765 10.1016/j.molcel.2008.12.016 PMC2650391

[bib20] Kamine J, Elangovan B, Subramanian T, Coleman D, Chinnadurai G (1996) Identification of a cellular protein that specifically interacts with the essential cysteine region of the HIV-1 Tat transactivator. Virology 216(2): 357–366. 8607265 10.1006/viro.1996.0071

[bib21] Kelley LA, Sternberg MJE (2009) Protein structure prediction on the Web: a case study using the Phyre server. Nat Protoc 4(3): 363–371. 19247286 10.1038/nprot.2009.2

[bib22] Kichina JV, Zeremski M, Aris L, Gurova KV, Walker E, Franks R, Nikitin AY, Kiyokawa H, Gudkov AV (2006) Targeted disruption of the mouse ing1 locus results in reduced body size, hypersensitivity to radiation and elevated incidence of lymphomas. Oncogene 25(6): 857–866. 16170338 10.1038/sj.onc.1209118

[bib23] Kim S, Natesan S, Cornilescu G, Carlson S, Tonelli M, McClurg UL, Binda O, Robson CN, Markley JL, Balaz S, Glass KC (2016) Mechanism of histone H3K4me3 recognition by the plant homeodomain of inhibitor of growth 3. J Biol Chem 291(35): 18326–18341. 27281824 10.1074/jbc.M115.690651 PMC5000080

[bib24] Kirkegaard T, Edwards J, Tovey S, McGlynn LM, Krishna SN, Mukherjee R, Tam L, Munro AF, Dunne B, Bartlett JM (2006) Observer variation in immunohistochemical analysis of protein expression, time for a change? Histopathology 48(7): 787–794. 16722926 10.1111/j.1365-2559.2006.02412.x

[bib25] Kuner R, Falth M, Pressinotti NC, Brase JC, Puig SB, Metzger J, Gade S, Schafer G, Bartsch G, Steiner E, Klocker H, Sultmann H (2013) The maternal embryonic leucine zipper kinase (MELK) is upregulated in high-grade prostate cancer. J Mol Med 91(2): 237–248. 22945237 10.1007/s00109-012-0949-1

[bib26] Lu S, Tsai SY, Tsai MJ (1999) Molecular mechanisms of androgen-independent growth of human prostate cancer LNCaP-AI cells. Endocrinology 140(11): 5054–5059. 10537131 10.1210/endo.140.11.7086

[bib27] McNicholas S, Potterton E, Wilson KS, Noble ME (2011) Presenting your structures: the CCP4mg molecular-graphics software. Acta Crystallogr D Biol Crystallogr 67(Pt 4): 386–394. 21460457 10.1107/S0907444911007281 PMC3069754

[bib28] Nabbi A, Almami A, Thakur S, Suzuki K, Boland D, Bismar TA, Riabowol K (2015) ING3 protein expression profiling in normal human tissues suggest its role in cellular growth and self-renewal. Eur J Cell Biol 94(5): 214–222. 25819753 10.1016/j.ejcb.2015.03.002

[bib29] Nabbi A, McClurg UL, Thalappilly S, Almami A, Mobahat M, Bismar TA, Binda O, Riabowol KT (2017) ING3 promotes prostate cancer growth by activating the androgen receptor. BMC Med 15(1): 103. 28511652 10.1186/s12916-017-0854-0 PMC5434536

[bib30] Nagashima M, Shiseki M, Pedeux RM, Okamura S, Kitahama-Shiseki M, Miura K, Yokota J, Harris CC (2003) A novel PHD-finger motif protein, p47ING3, modulates p53-mediated transcription, cell cycle control, and apoptosis. Oncogene 22(3): 343–350. 12545155 10.1038/sj.onc.1206115

[bib31] O'Neill DJ, Williamson SC, Alkharaif D, Monteiro ICM, Goudreault M, Gaughan L, Robson CN, Gingras A-C, Binda O (2014) SETD6 controls the expression of estrogen-responsive genes and proliferation of breast carcinoma cells. Epigenetics 9(7): 942–950. 24751716 10.4161/epi.28864 PMC4143409

[bib32] Obri A, Ouararhni K, Papin C, Diebold M-L, Padmanabhan K, Marek M, Stoll I, Roy L, Reilly PT, Mak TW, Dimitrov S, Romier C, Hamiche A (2014) ANP32E is a histone chaperone that removes H2A.Z from chromatin. Nature 505(7485): 648–653. 24463511 10.1038/nature12922

[bib33] Riley T, Sontag E, Chen P, Levine A (2008) Transcriptional control of human p53-regulated genes. Nat Rev Mol Cell Biol 9(5): 402–412. 18431400 10.1038/nrm2395

[bib34] Saito M, Kumamoto K, Robles AI, Horikawa I, Furusato B, Okamura S, Goto A, Yamashita T, Nagashima M, Lee T-L, Baxendale VJ, Rennert OM, Takenoshita S, Yokota J, Sesterhenn IA, Trivers GE, Hussain SP, Harris CC (2010) Targeted disruption of Ing2 results in defective spermatogenesis and development of soft-tissue sarcomas. PLoS One 5(11): e15541. 21124965 10.1371/journal.pone.0015541 PMC2988811

[bib35] Shi X, Hong T, Walter KL, Ewalt M, Michishita E, Hung T, Carney D, Peña P, Lan F, Kaadige MR, Lacoste N, Cayrou C, Davrazou F, Saha A, Cairns BR, Ayer DE, Kutateladze TG, Shi Y, Côté J, Chua KF, Gozani O (2006) ING2 PHD domain links histone H3 lysine 4 methylation to active gene repression. Nature 442(7098): 96–99. 16728974 10.1038/nature04835 PMC3089773

[bib36] Wang T, Guo S, Liu Z, Wu L, Li M, Yang J, Chen R, Liu X, Xu H, Cai S, Chen H, Li W, Xu S, Wang L, Hu Z, Zhuang Q, Wang L, Wu K, Liu J, Ye Z, Ji J-Y, Wang C, Chen K (2014a) CAMK2N1 inhibits prostate cancer progression through androgen receptor-dependent signaling. Oncotarget 5(21): 10293–10306. 25296973 10.18632/oncotarget.2511 PMC4279373

[bib37] Wang T, Liu Z, Guo S, Wu L, Li M, Yang J, Chen R, Xu H, Cai S, Chen H, Li W, Wang L, Hu Z, Zhuang Q, Xu S, Wang L, Liu J, Ye Z, Ji J-Y, Wang C, Chen K (2014b) The tumor suppressive role of CAMK2N1 in castration-resistant prostate cancer. Oncotarget 5(11): 3611–3621. 25003983 10.18632/oncotarget.1968 PMC4116507

[bib38] Wong YNS, Ferraldeschi R, Attard G, de Bono J (2014) Evolution of androgen receptor targeted therapy for advanced prostate cancer. Nat Rev Clin Oncol 11(6): 365–376. 24840076 10.1038/nrclinonc.2014.72

[bib39] Yamamoto T, Horikoshi M (1997) Novel substrate specificity of the histone acetyltransferase activity of HIV-1-Tat interactive protein Tip60. J Biol Chem 272(49): 30595–30598. 9388189 10.1074/jbc.272.49.30595

[bib40] Zhou H, Wang L, Huang J, Jiang M, Zhang X, Zhang L, Wang Y, Jiang Z, Zhang Z (2015) High EGFR_1 inside-out activated inflammation-induced motility through SLC2A1-CCNB2-HMMR-KIF11-NUSAP1-PRC1-UBE2C. J Cancer 6(6): 519–524. 26000042 10.7150/jca.11404 PMC4439936

