# Supplementary material for: Human ex vivo prostate tissue model system identifies ING3 as an oncoprotein
Source: Br J Cancer. 2018 Jan 30;118(5):713–26. doi: 10.1038/bjc.2017.447 (PMC5846061; doi:10.1038/bjc.2017.447)
Supplement: Supplementary Figures and Tables [file bjc2017447x1.pdf]

Supplementary Figure and Table legends

Figure S1: (**A**) The Cancer Genome Atlas (TCGA) database was accessed through oncomine.org and the copy number of *ING3* from paired normal tissue (i.e. blood or prostate gland; n = 207) and prostate adenocarcinoma (n = 169) plotted (*** indicates a p-value < 0.001). (**B**) *ING3* copy number counts from the TCGA prostate cancer dataset between patients who achieved complete or partial remission during androgen ablation (n = 185) and patients who experienced no remission (n = 34). (**C**) Kaplan-Meier survival curve of patients in the TCGA prostate cancer dataset based on patients with low (n = 46) or high (n = 24) ING3 counts (* indicates a p-value of 0.02). (**D-F**) TMA from main Figure 1A were stained for indicated proliferation markers and plotted against ING3 H-score.

Figure S2: The microarray data for each gene was plotted for both siING3 no. 1 and siING3 no. 2.

Figure S3: Proliferation of breast, ovarian, and prostate cancer cell lines. Proliferation of control (Scramble siRNA) or ING3-silenced (siING3 no.1) CWR22Rv1, LNCaP-AI, MCF7, MX-1, PC3, PEO1, and PEO4 cells was monitored for 5 consecutive days by IncuCyte. Data was analysed using Wilcoxon signed-rank test.

Figure S4: Kegg pathway analysis.

Figure S5: ING3 associates with $H3K4^{me3}$ at transcriptional start sites. ChIP experiments of ING3 at the transcriptional start site (TSS) of *CCND1* and *PCNA* were conducted using an α-FLAG antibody. Similarly, ChIP experiments of ING3 and AR at the ARE_{III} of *PSA* and the ARE of *KLK2* were conducted using α-FLAG or α-AR antibodies, respectively.

Figure S6: Structure prediction of $ING3_{PHD}$ with (**A**) or without (**B**) the PNEPR sequence preceding the PHD domain.

Figure S7: The TCGA database was analysed for the expression of ING3 and cell cycle genes in prostate cancer tissues and the expression of genes that correlated with the expression of ING3 was plotted.

Figure S8: The cBioPortal website was queried for alterations in ING proteins in various cancers.

Table S1: List of primers used for real-time qPCR gene expression analysis.

Table S2: List of primers used for real-time qPCR analysis of ChIP.

Table S3: Complete list of genes from the siING3 microarray survey.

Table S4: Summary of characteristics of cell lines used in our studies.

Figure S1

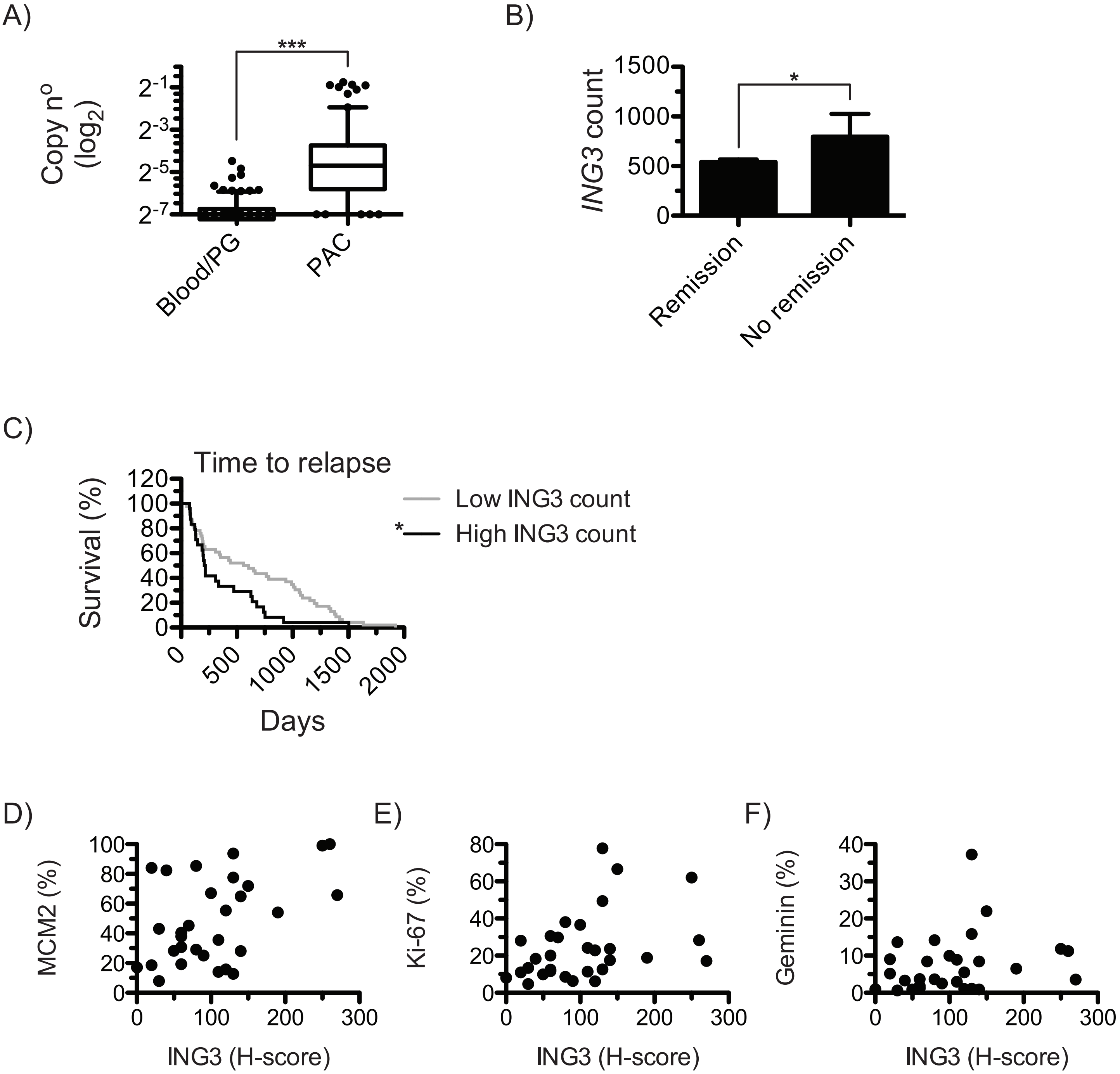
A)
***
Copy nº (log2)
2-1
2-3
2-5
2-7
Blood/PG
PAC
B)
ING3 count
1500
1000
500
0
*
Remission
No remission
C)
Time to relapse
Survival (%)
120
100
80
60
40
20
0
Low ING3 count
*
High ING3 count
0
500
1000
1500
2000
Days
D)
MCM2 (%)
ING3 (H-score)
E)
Ki-67 (%)
ING3 (H-score)
F)
Geminin (%)
ING3 (H-score)

Figure S2

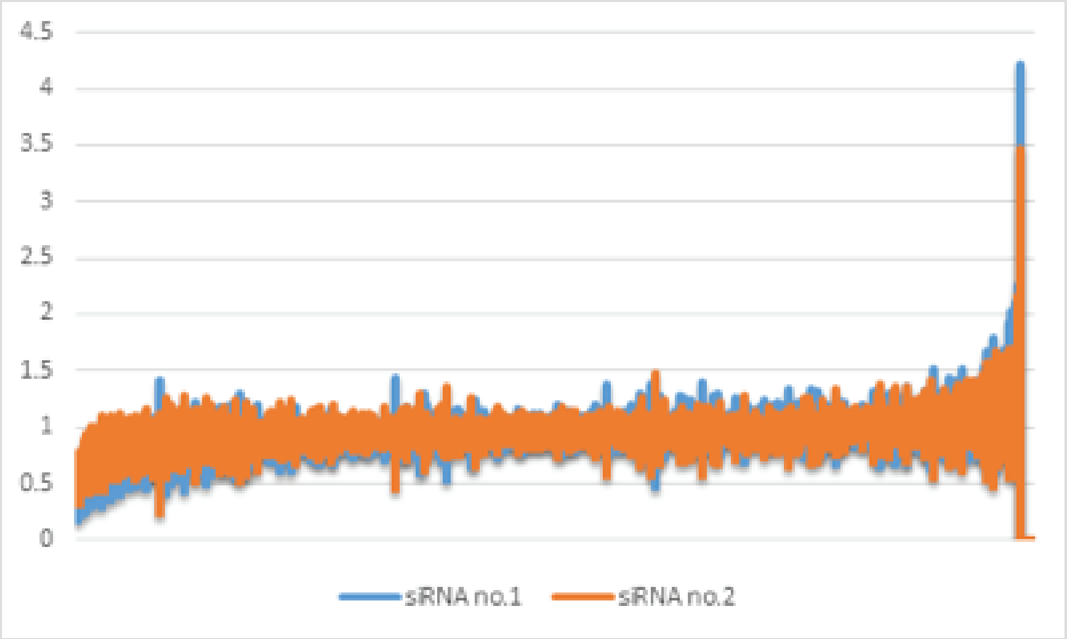

Figure S3

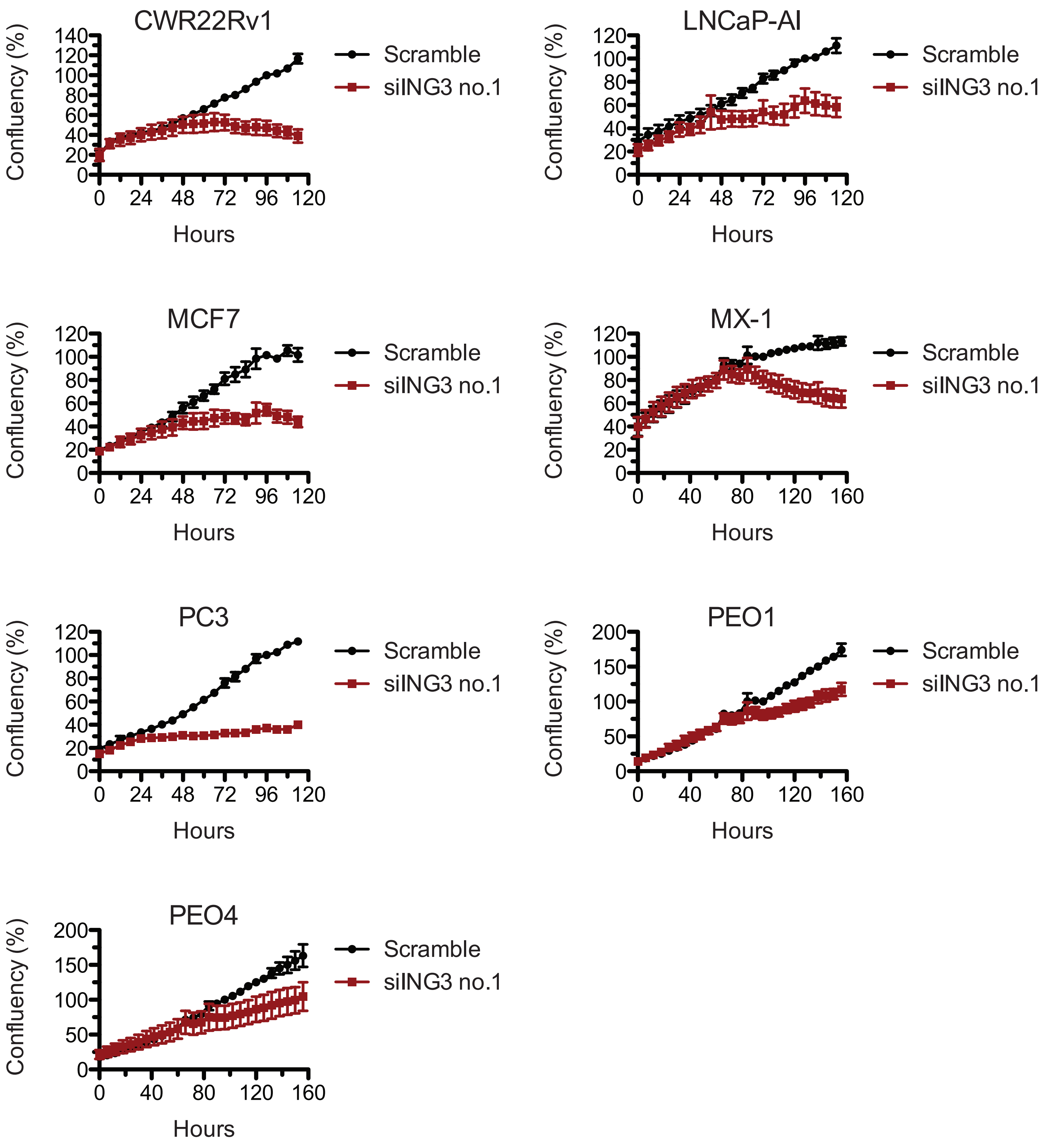
CWR22Rv1
Confluency (%)
Hours
Scramble
siING3 no.1
LNCaP-AI
Confluency (%)
Hours
Scramble
siING3 no.1
MCF7
Confluency (%)
Hours
Scramble
siING3 no.1
MX-1
Confluency (%)
Hours
Scramble
siING3 no.1
PC3
Confluency (%)
Hours
Scramble
siING3 no.1
PEO1
Confluency (%)
Hours
Scramble
siING3 no.1
PEO4
Confluency (%)
Hours
Scramble
siING3 no.1

Figure S4

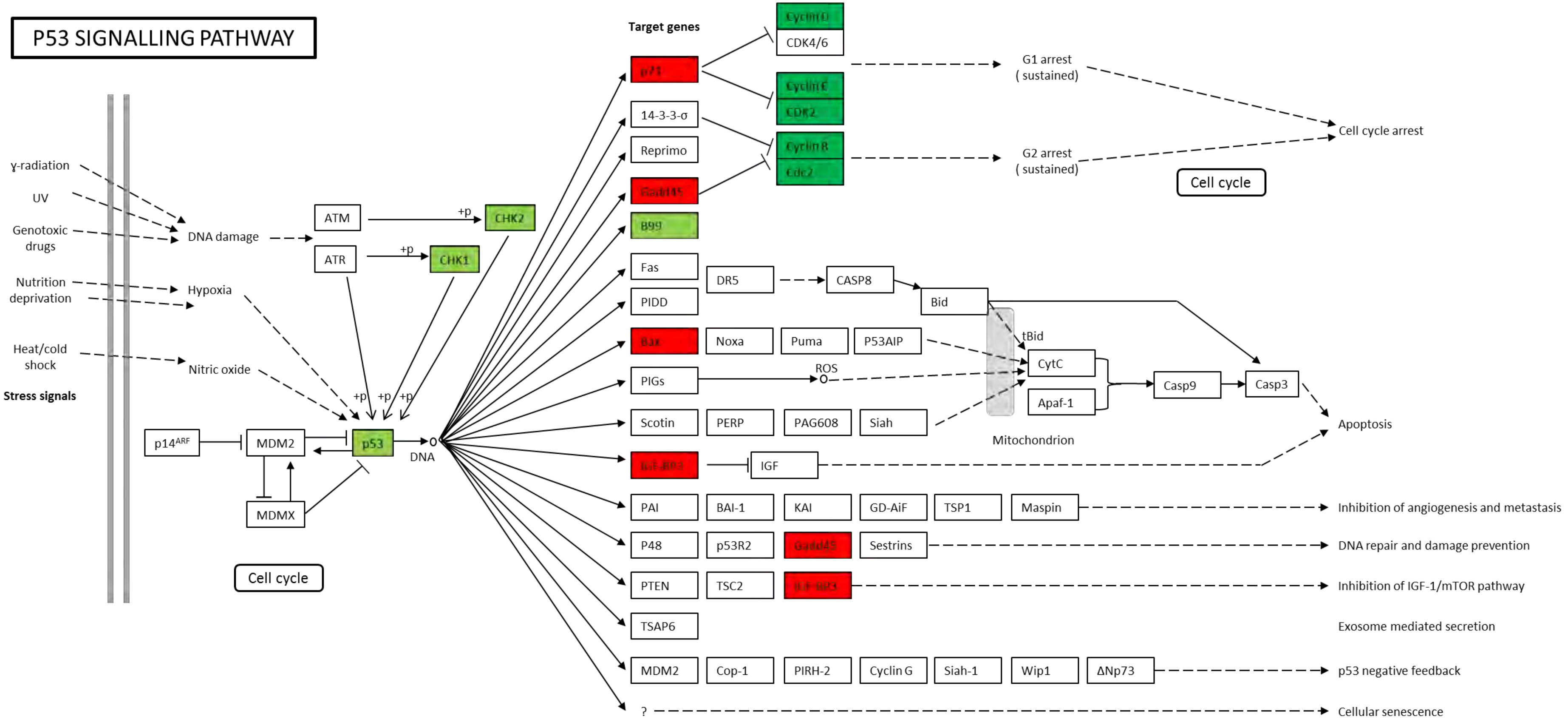
P53 SIGNALLING PATHWAY
γ-radiation
UV
Genotoxic drugs
Nutrition deprivation
Heat/cold shock
Stress signals
DNA damage
Hypoxia
Nitric oxide
ATM
ATR
+p
CHK2
CHK1
+p
+p
+p
p14ARF
MDM2
MDMX
p53
DNA
Cell cycle
Target genes
Cyclin D
CDK4/6
Cyclin E
CDK2
14-3-3-σ
Reprimo
Cyclin B
Cdc2
B99
G1 arrest (sustained)
G2 arrest (sustained)
Cell cycle arrest
Cell cycle
Fas
DR5
CASP8
Bid
PIDD
Noxa
Puma
P53AIP
tBid
CytC
PIGs
ROS
Apaf-1
Casp9
Casp3
Scotin
PERP
PAG608
Siah
Mitochondrion
Apoptosis
IGF
PAI
BAI-1
KAI
GD-AiF
TSP1
Maspin
Inhibition of angiogenesis and metastasis
P48
p53R2
Sestrins
DNA repair and damage prevention
PTEN
TSC2
Inhibition of IGF-1/mTOR pathway
TSAP6
Exosome mediated secretion
MDM2
Cop-1
PIRH-2
Cyclin G
Siah-1
Wip1
ΔNp73
p53 negative feedback
?
Cellular senescence

Figure S5

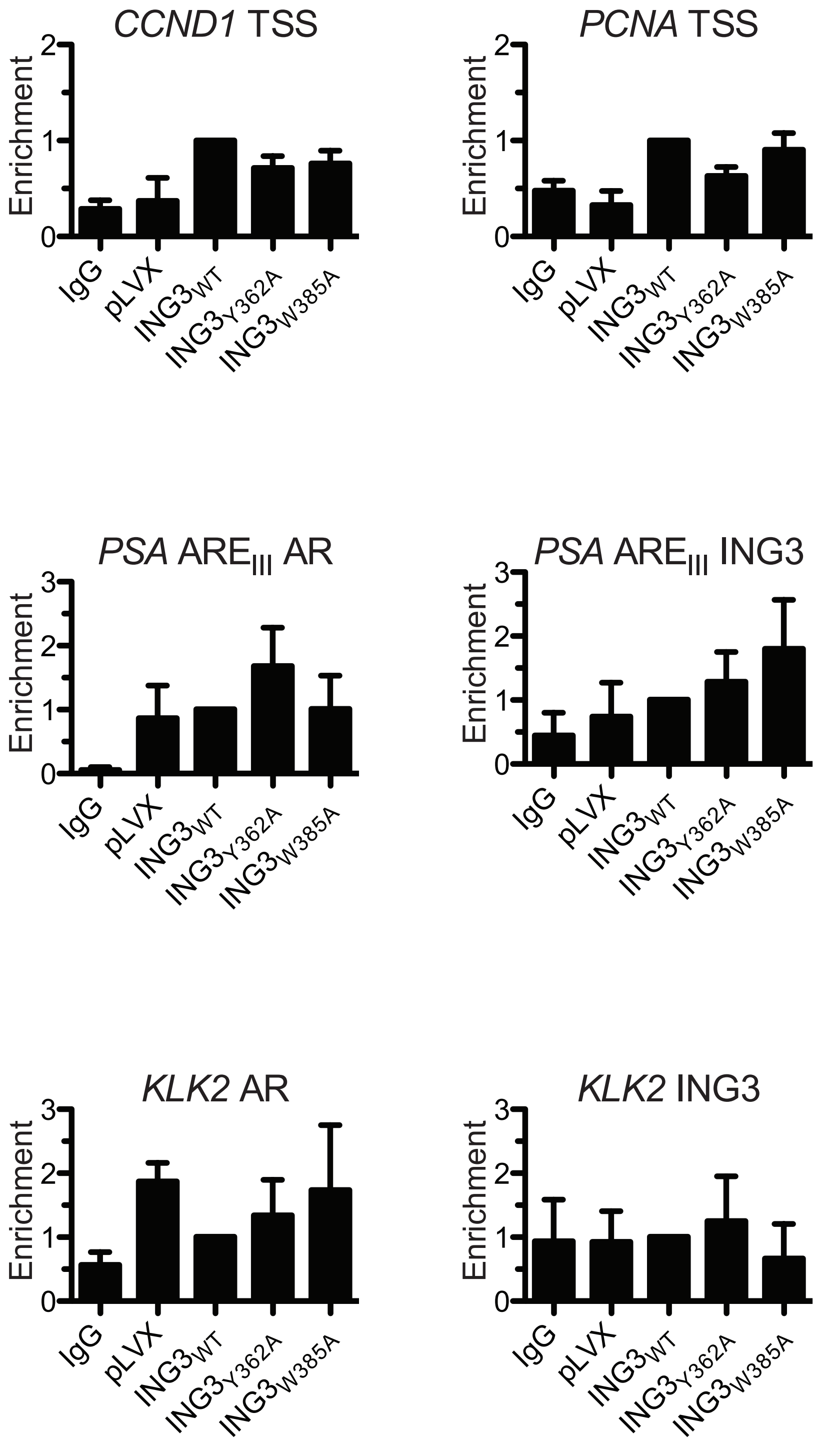

CCND1 TSS
PCNA TSS
PSA ARE$_{III}$ AR
PSA ARE$_{III}$ ING3
KLK2 AR
KLK2 ING3
Enrichment
0
1
2
3
IgG
pLVX
ING3$_{WT}$
ING3$_{Y362A}$
ING3$_{W385A}$

Figure S6

A)

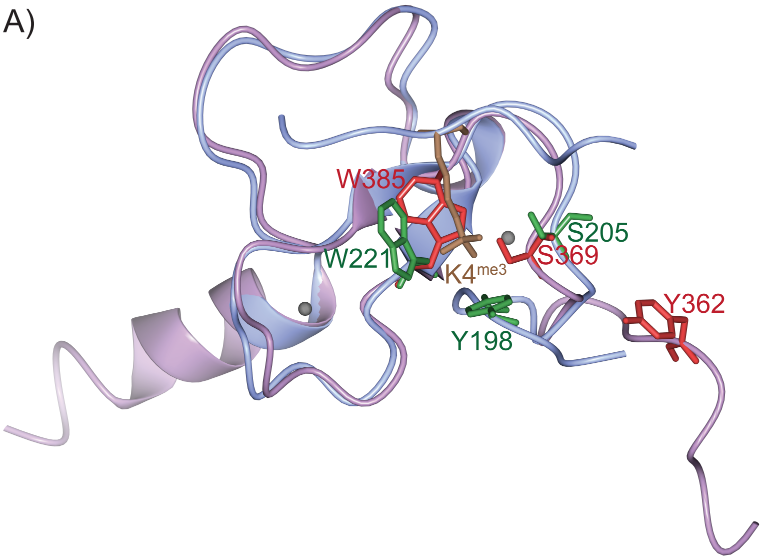

W385
S205
W221
S369
K4me3
Y362
Y198

B)

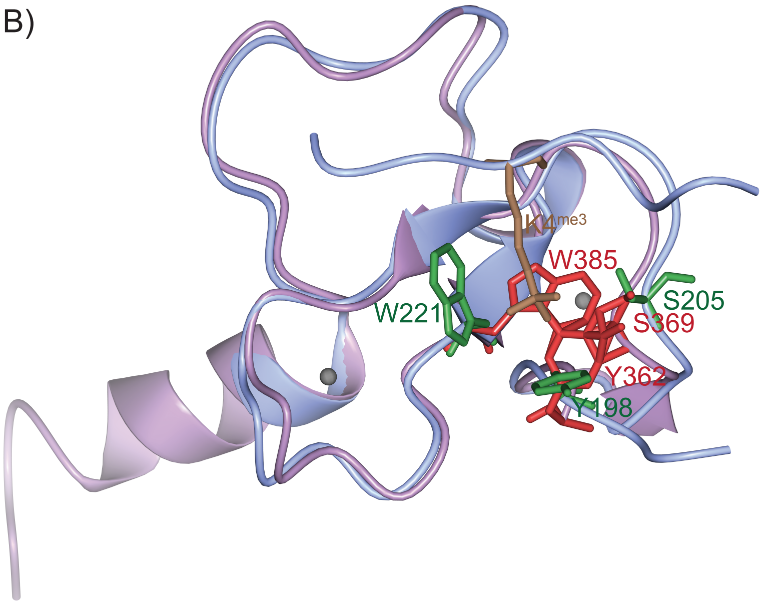

K4me3
W385
S205
W221
S369
Y362
Y198

Figure S7

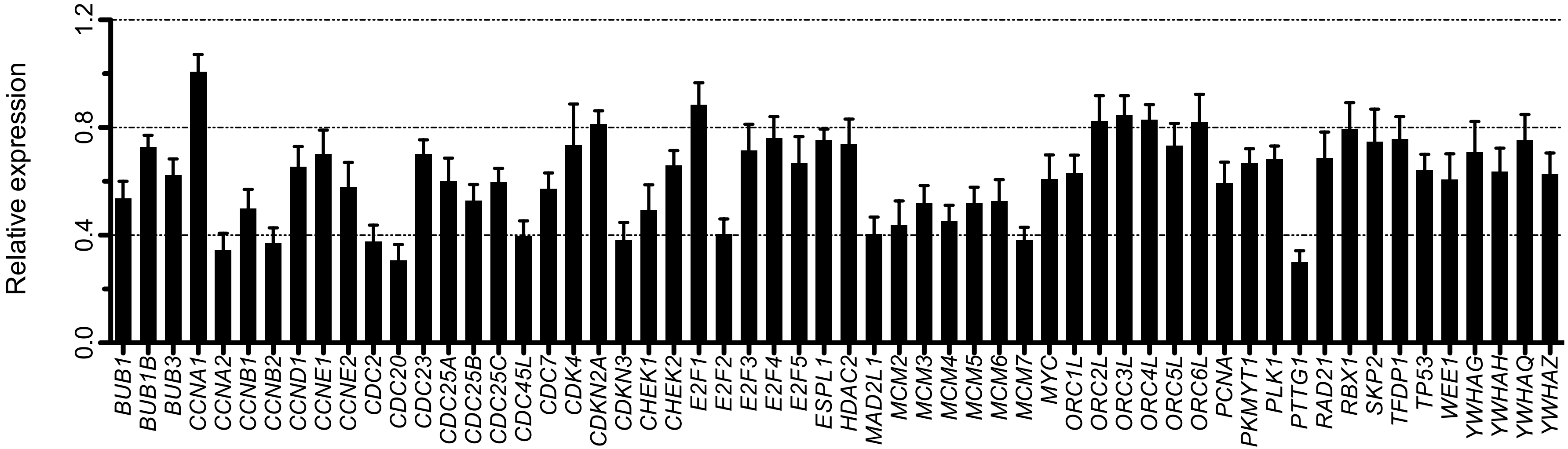

Relative expression
0.0
0.4
0.8
1.2
BUB1
BUB1B
BUB3
CCNA1
CCNA2
CCNB1
CCNB2
CCND1
CCNE1
CCNE2
CDC2
CDC20
CDC23
CDC25A
CDC25B
CDC25C
CDC45L
CDC7
CDK4
CDKN2A
CDKN3
CHEK1
CHEK2
E2F1
E2F2
E2F3
E2F4
E2F5
ESPL1
HDAC2
MAD2L1
MCM2
MCM3
MCM4
MCM5
MCM6
MCM7
MYC
ORC1L
ORC2L
ORC3L
ORC4L
ORC5L
ORC6L
PCNA
PKMYT1
PLK1
PTTG1
RAD21
RBX1
SKP2
TFDP1
TP53
WEE1
YWHAG
YWHAH
YWHAQ
YWHAZ

Figure S8

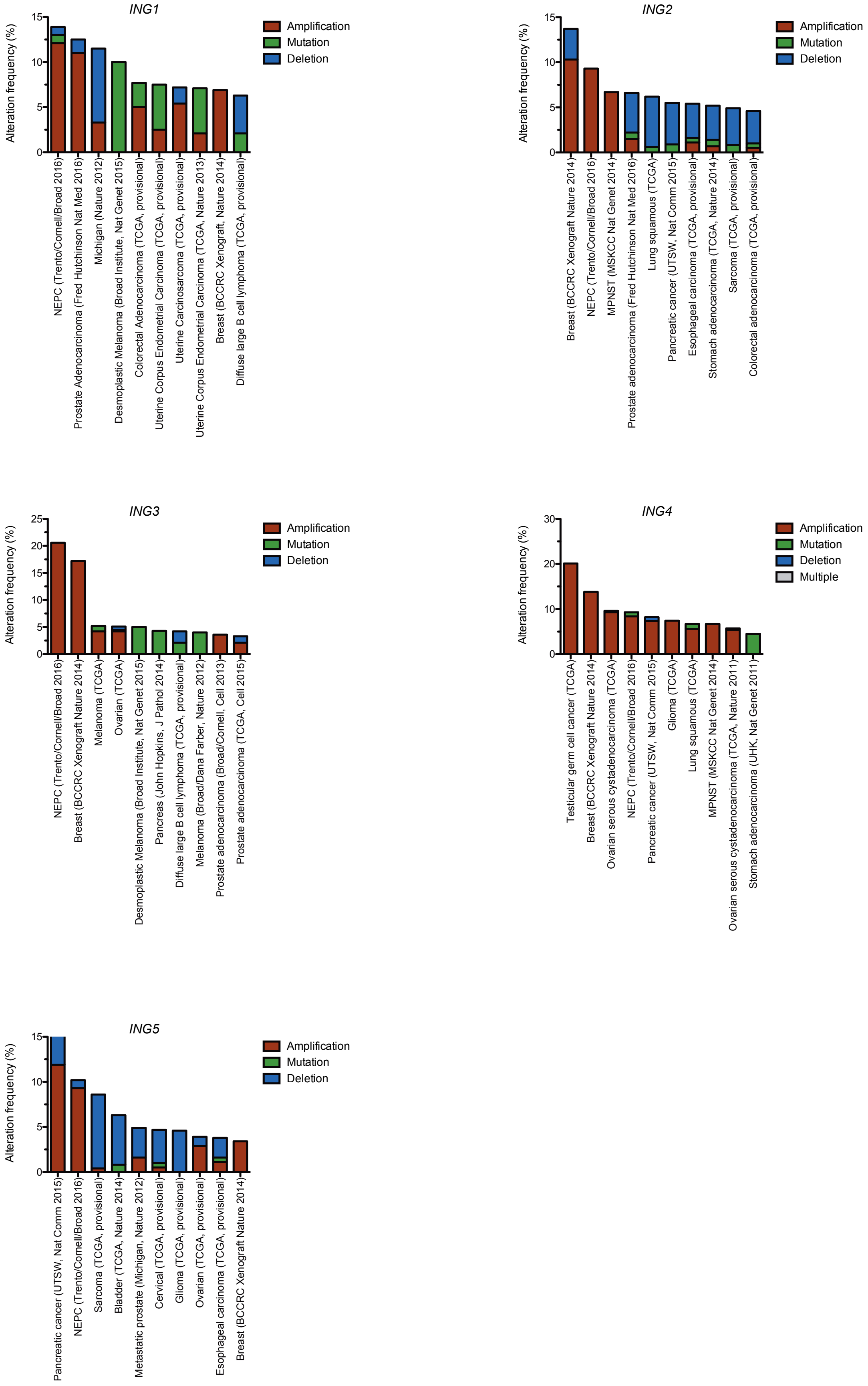
ING1
Alteration frequency (%)
0
5
10
15
Amplification
Mutation
Deletion
NEPC (Trento/Cornell/Broad 2016)
Prostate Adenocarcinoma (Fred Hutchinson Nat Med 2016)
Michigan (Nature 2012)
Desmoplastic Melanoma (Broad Institute, Nat Genet 2015)
Colorectal Adenocarcinoma (TCGA, provisional)
Uterine Corpus Endometrial Carcinoma (TCGA, provisional)
Uterine Carcinosarcoma (TCGA, provisional)
Uterine Corpus Endometrial Carcinoma (TCGA, Nature 2013)
Breast (BCCRC Xenograft, Nature 2014)
Diffuse large B cell lymphoma (TCGA, provisional)
ING2
Alteration frequency (%)
0
5
10
15
Amplification
Mutation
Deletion
Breast (BCCRC Xenograft Nature 2014)
NEPC (Trento/Cornell/Broad 2016)
MPNST (MSKCC Nat Genet 2014)
Prostate adenocarcinoma (Fred Hutchinson Nat Med 2016)
Lung squamous (TCGA)
Pancreatic cancer (UTSW, Nat Comm 2015)
Esophageal carcinoma (TCGA, provisional)
Stomach adenocarcinoma (TCGA, Nature 2014)
Sarcoma (TCGA, provisional)
Colorectal adenocarcinoma (TCGA, provisional)
ING3
Alteration frequency (%)
0
5
10
15
20
25
Amplification
Mutation
Deletion
NEPC (Trento/Cornell/Broad 2016)
Breast (BCCRC Xenograft Nature 2014)
Melanoma (TCGA)
Ovarian (TCGA)
Desmoplastic Melanoma (Broad Institute, Nat Genet 2015)
Pancreas (John Hopkins, J Pathol 2014)
Diffuse large B cell lymphoma (TCGA, provisional)
Melanoma (Broad/Dana Farber, Nature 2012)
Prostate adenocarcinoma (Broad/Cornell, Cell 2013)
Prostate adenocarcinoma (TCGA, Cell 2015)
ING4
Alteration frequency (%)
0
10
20
30
Amplification
Mutation
Deletion
Multiple
Testicular germ cell cancer (TCGA)
Breast (BCCRC Xenograft Nature 2014)
Ovarian serous cystadenocarcinoma (TCGA)
NEPC (Trento/Cornell/Broad 2016)
Pancreatic cancer (UTSW, Nat Comm 2015)
Glioma (TCGA)
Lung squamous (TCGA)
MPNST (MSKCC Nat Genet 2014)
Ovarian serous cystadenocarcinoma (TCGA, Nature 2011)
Stomach adenocarcinoma (UHK, Nat Genet 2011)
ING5
Alteration frequency (%)
0
5
10
15
Amplification
Mutation
Deletion
Pancreatic cancer (UTSW, Nat Comm 2015)
NEPC (Trento/Cornell/Broad 2016)
Sarcoma (TCGA, provisional)
Bladder (TCGA, Nature 2014)
Metastatic prostate (Michigan, Nature 2012)
Cervical (TCGA, provisional)
Glioma (TCGA, provisional)
Ovarian (TCGA, provisional)
Esophageal carcinoma (TCGA, provisional)
Breast (BCCRC Xenograft Nature 2014)

Table S1: Real-time qPCR primers for gene expression. The primers were selected from the NCBI Nucleotide database using the utility Primer-BLAST. The primers used for gene expression were designed to span exon-exon junctions and be separated by at least one intron when possible.

Gene	Forward sequence 5'-3'	Reverse sequence 5'-3'
AR	GCAAAGCCTAAAGCCAGAT	GAGTTCATGGGTGGCAAAG
BAX	GCCCTTTTGCTTCAGGGTTTCA	TCAGCTTCTTGGTGGACGCA
CAMK2N1	GCACGTCATCAATCCTATCATC	ACACCAACAACTTCTTCGGC
CCNA2	CACTCACTGGCTTTTCATCTTC	CAGAAAACCATTGGTCCCTC
CCNB2	GATTTTGCAGAGCAAGGCAT	TGGAAAAGTTGGCTCCAAAG
CDKN1A	CAGCTGCCGAAGTCAGTTCC	GTTCTGACATGGCGCCTCCT
FOXO3	AACGTGGGGAACTTCACTGGT	TTTGAGGGTCTGCTTTGCCCA
GREB1	GGATGAGGAGCTGGGGACAG	GCTGAACCGGAAGCCTTGGA
HMMR	AAGCTGACAGCGGAGTTTTG	TGGGTCATCAGAATTTGAAACA
HPRT1	GAACGTCTTGCTCGAGAGATGTG	CCAGCAGGTCAGCAAAGAATTT
ING3	TGGAGGGAAGAGCAAATGGCA	TCTCAAGTGTCGATCTACCAAGTCA
KIF20A	TAACAAGGGCCTAACCCTCA	TGCTCTGTCGTCTCTACCTCC
KLK2	AGCATCGAACCAGAGGAGTTCT	TGGAGGCTCACACACTGAAGA
MELK	GAGAGCCACTTGGGAACATC	TGTGTGGATTTCTACCATTTGA
NKX3.1	AGCCAGAAAGGCACTTGGG	GGCGCCTGAAGTGTTTTCA
NOXA	CTCTGTAGCTGAGTGGGCGG	ACACTCGACTTCCAGCTCTGC
NUSAP1	TCATTTCCTTTTCTTGCCTCA	GCCAAGAGTCTGGGTCTCC
PIRC1	TACTATGGACTCCTCCGCCA	CCGTGTGGAGTAGGTCTGGA
PML	GTCTTCCTGCCCAACAGCAAC	AGCTCTCGGGAGGACGAGTT
PSA	ACTGCATCAGGAACAAAAGCGT	TGTGGGAAGCTGTGGCTGAC
TMPRSS2	CTGCTGGATTTCCGGGTG	TTCTGAGGTCTTCCCTTTCTCCT
UBE2C	TTGTAAGGGTAGCCACTGGG	TCAAATGGGTAGGGACCATC

Table S2: Real-time qPCR primers for ChIP analysis

Gene	Forward sequence 5’-3’	Reverse sequence 5’-3’
$AURKA_{TSS}$	AGAGATTCTGATTTACCGGGCTCT	TCCGTACTTCTCAGACCGTAGACC
$CCNB1_{TSS}$	TCTTTCTGGGAACTTCTCCTTGTG	TCGAGTTCTGAAAGAAGCAAGACC
$CCND1_{TSS}$	GTCTCCAAATGGTCACCAAGAAAT	CTGTCTTCTTTAAACCCACCACGA
$CCNE2_{TSS}$	GGTCACCTGATCGTAAGCAGAACT	TTCCCACAAACGTTACTCGACATA
$CDC2_{TSS}$	GGAATAATAAGCCGGGTACAGTGG	TAGAGGACCCCGTTCCTCAATACT
$CDK2_{TSS}$	GAAGAAGGCATCTTTTTAGCACCA	TGCAAATGAGCACCCTTTTCTTAT
$CDKN1A_{-500bp}$	GGAGACTGCAGTGAGCTGAGAT	CCCTGGCTTTTTGTTTTCATTT
$CDKN1A_{TSS}$	CTGTGGCTCTGATTGGCTTTCT	GACAAAATAGCCACCAGCCTCT
$CDKN2B_{TSS}$	TGGCCGTAAACTTAACGACACTCT	ACGGGAGGGTAATGAAGCTGAG
$KLK2_{ARE}$	ACCCCTGTTGCTGTTCATCCTG	CCGCCCTTGCCCTGTTGG
$PCNA_{TSS}$	AGGAGGAAAGTCTAGCTGGTTTCG	TGGTCGTTGTCTTTCTAGGTCTCA
PSA_{AREIII}	TGGGACAACTTGCAAACCTG	CCAGAGTAGGTCTGTTTTCAATCCA
$TMPRSS2_{TSS}$	TGGTCCTGGATGATAAAAAAAGTTT	GACATACGCCCCACAACAGA

Table S3: Microarray data from siING3 LNCaP.

SYMBOL	repeat 1	repeat 2	repeat 3	repeat 4	n=4 average	S.D.M.
PTTG1	0.1788	0.3643	0.3575	0.3044	0.3012	0.0430
CDC20	0.1359	0.3863	0.3803	0.3187	0.3053	0.0585
UBE2C	0.1621	0.4084	0.3986	0.3329	0.3255	0.0570
FAM83D	0.1542	0.3745	0.3844	0.4071	0.3300	0.0590
GAL	0.1844	0.3757	0.4043	0.3653	0.3324	0.0500
SNORD16	0.1892	0.3848	0.4103	0.3647	0.3372	0.0502
NUSAP1	0.2043	0.3992	0.4035	0.3433	0.3376	0.0465
SPC24	0.1891	0.4030	0.3784	0.3893	0.3400	0.0505
CCNA2	0.1661	0.3966	0.4601	0.3533	0.3440	0.0632
CEP55	0.1973	0.3994	0.3888	0.3932	0.3447	0.0492
AURKA	0.1958	0.4288	0.4010	0.3692	0.3487	0.0524
CDCA5	0.2289	0.4406	0.4057	0.3206	0.3489	0.0473
MELK	0.2323	0.4513	0.3531	0.3663	0.3508	0.0451
HMMR	0.2280	0.4003	0.4225	0.3587	0.3524	0.0435
TK1	0.2131	0.4460	0.4145	0.3372	0.3527	0.0519
TOP2A	0.1870	0.4605	0.3730	0.3928	0.3533	0.0585
DLGAP5	0.2390	0.3972	0.3913	0.3996	0.3568	0.0393
LOC731314	0.1842	0.4761	0.4060	0.3816	0.3620	0.0626
PRC1	0.2231	0.4509	0.4075	0.4021	0.3709	0.0505
CCNB2	0.2149	0.4545	0.4251	0.4032	0.3744	0.0542
KIF20A	0.2305	0.4718	0.3680	0.4304	0.3752	0.0527
OIP5	0.2344	0.4493	0.4255	0.3950	0.3761	0.0485
LOC399942	0.2116	0.4766	0.4457	0.3728	0.3767	0.0592
MCM7	0.2597	0.4699	0.4355	0.3560	0.3803	0.0467
CDC2	0.2239	0.4737	0.4711	0.3538	0.3806	0.0593
PTTG3P	0.1978	0.4419	0.4556	0.4359	0.3828	0.0618
CDKN3	0.2112	0.5224	0.4293	0.3709	0.3834	0.0653
NCAPG	0.2065	0.4762	0.4562	0.3962	0.3838	0.0615
C16orf75	0.2102	0.4667	0.4131	0.4826	0.3932	0.0628
CDC45L	0.2312	0.4717	0.4576	0.4321	0.3981	0.0562
TMSB15A	0.2714	0.4406	0.4354	0.4473	0.3987	0.0425
FAM64A	0.2733	0.4806	0.4312	0.4135	0.3996	0.0445
CKS1B	0.2459	0.5311	0.4068	0.4200	0.4009	0.0587
TYMS	0.2172	0.5749	0.4812	0.3338	0.4018	0.0790
NMU	0.2512	0.4487	0.4810	0.4281	0.4023	0.0515
MAD2L1	0.2169	0.4870	0.4715	0.4405	0.4039	0.0631
AURKB	0.2213	0.5332	0.4099	0.4518	0.4040	0.0661
PBK	0.2503	0.4480	0.4499	0.4688	0.4043	0.0515
E2F2	0.2478	0.4739	0.4008	0.4999	0.4056	0.0566
CENPM	0.2545	0.5062	0.4324	0.4447	0.4094	0.0541
CDCA8	0.2835	0.4743	0.4793	0.4248	0.4155	0.0457
KIFC1	0.2673	0.4892	0.4565	0.4778	0.4227	0.0522
HES6	0.2690	0.5558	0.4785	0.3896	0.4232	0.0616

HMGB2	0.2579	0.5502	0.4490	0.4374	0.4236	0.0608
MMD	0.2728	0.5823	0.4069	0.4333	0.4238	0.0635
APOBEC3B	0.2840	0.5371	0.4155	0.4644	0.4253	0.0533
TPX2	0.3024	0.5247	0.4265	0.4516	0.4263	0.0463
C11orf82	0.2495	0.5887	0.4895	0.3838	0.4279	0.0727
CDCA3	0.3360	0.4502	0.4873	0.4401	0.4284	0.0324
PRIM1	0.2761	0.5158	0.4590	0.4655	0.4291	0.0525
CENPF	0.2987	0.5941	0.4112	0.4162	0.4300	0.0610
CKS2	0.2437	0.4920	0.5922	0.3930	0.4302	0.0743
RACGAP1	0.2866	0.5166	0.4649	0.4560	0.4310	0.0500
FAM57A	0.3213	0.6587	0.3769	0.3712	0.4320	0.0766
LMNB1	0.2643	0.6002	0.4373	0.4348	0.4342	0.0686
WDR51A	0.3117	0.5682	0.4509	0.4136	0.4361	0.0529
MCM2	0.2560	0.6886	0.3934	0.4104	0.4371	0.0907
UGT2B15	0.2941	0.5351	0.4677	0.4518	0.4372	0.0510
H2AFX	0.2461	0.5753	0.4654	0.4655	0.4381	0.0690
CLDND2	0.3817	0.5382	0.4192	0.4178	0.4392	0.0341
C18orf56	0.2959	0.5222	0.4790	0.4611	0.4396	0.0496
ADIPOR1	0.3382	0.5502	0.4460	0.4293	0.4409	0.0434
RAD51AP1	0.2889	0.5197	0.4739	0.4833	0.4414	0.0518
CENPA	0.2943	0.5321	0.4633	0.4919	0.4454	0.0523
LOC651816	0.2974	0.5243	0.5043	0.4637	0.4474	0.0516
NETO2	0.3124	0.5105	0.4992	0.4701	0.4480	0.0460
BIRC5	0.3022	0.5476	0.5055	0.4383	0.4484	0.0537
PDSS1	0.3366	0.4991	0.5198	0.4404	0.4490	0.0411
MCM4	0.2862	0.5664	0.4779	0.4661	0.4492	0.0587
RRM2	0.2416	0.5798	0.4884	0.4892	0.4497	0.0726
FEN1	0.2995	0.5458	0.4794	0.4754	0.4500	0.0527
TRIP13	0.2497	0.6060	0.4399	0.5183	0.4535	0.0759
SFXN1	0.2791	0.5629	0.4955	0.4803	0.4545	0.0612
OAF	0.3242	0.5594	0.4588	0.4876	0.4575	0.0492
LOC729779	0.3171	0.5937	0.4655	0.4537	0.4575	0.0565
TTK	0.3005	0.4847	0.5557	0.5084	0.4623	0.0559
CDT1	0.2999	0.6573	0.4774	0.4219	0.4641	0.0743
SLC30A7	0.4327	0.5239	0.4479	0.4653	0.4674	0.0200
FBXO5	0.3148	0.5467	0.5040	0.5081	0.4684	0.0521
GINS2	0.2610	0.5977	0.5054	0.5415	0.4764	0.0743
CKAP2L	0.3071	0.6246	0.4864	0.4878	0.4765	0.0651
HJURP	0.3464	0.6252	0.4479	0.4901	0.4774	0.0578
GGH	0.3854	0.4977	0.5961	0.4313	0.4776	0.0457
BCL2L12	0.3507	0.5957	0.4617	0.5103	0.4796	0.0511
ATAD2	0.4097	0.5505	0.4484	0.5100	0.4797	0.0314
LOC731049	0.3521	0.4802	0.5714	0.5180	0.4804	0.0467
UHRF1	0.3457	0.6192	0.4567	0.5001	0.4804	0.0565
MTAP	0.3588	0.5800	0.4997	0.4954	0.4835	0.0459
RBPMS2	0.3335	0.5896	0.5424	0.4753	0.4852	0.0557

MTFR1	0.4183	0.4733	0.5710	0.4829	0.4864	0.0316
PLK4	0.3073	0.6535	0.5199	0.4736	0.4886	0.0715
PRR11	0.3331	0.6171	0.5148	0.4905	0.4889	0.0587
RAB31	0.3900	0.6081	0.4430	0.5184	0.4899	0.0474
CHEK1	0.2631	0.7175	0.5316	0.4625	0.4936	0.0938
KIF4A	0.3863	0.5739	0.4686	0.5467	0.4939	0.0422
ZWILCH	0.3456	0.6199	0.5512	0.4640	0.4952	0.0592
KIAA0114	0.3341	0.6494	0.5493	0.4516	0.4961	0.0674
MTP18	0.4112	0.5148	0.5474	0.5150	0.4971	0.0296
CCNB1	0.3509	0.5738	0.6640	0.4171	0.5014	0.0715
LOC440063	0.3781	0.5792	0.6164	0.4332	0.5017	0.0571
ASPM	0.3565	0.6487	0.5075	0.5047	0.5043	0.0597
SNHG1	0.3530	0.6720	0.4605	0.5353	0.5052	0.0670
PSAT1	0.3625	0.5633	0.5453	0.5545	0.5064	0.0481
KIF11	0.4253	0.6061	0.5332	0.4749	0.5099	0.0389
MDC1	0.3760	0.5781	0.5322	0.5532	0.5099	0.0456
KIF14	0.4288	0.5862	0.4749	0.5545	0.5111	0.0361
RDH10	0.4304	0.5515	0.4774	0.5866	0.5115	0.0354
SGOL1	0.3521	0.5883	0.5632	0.5484	0.5130	0.0543
DDX39	0.3627	0.5944	0.5816	0.5170	0.5139	0.0532
RFC4	0.2961	0.6916	0.5428	0.5294	0.5150	0.0817
FAM96A	0.3948	0.6583	0.5671	0.4427	0.5157	0.0598
LOC728160	0.3991	0.6154	0.5639	0.4852	0.5159	0.0472
NCAPD2	0.3441	0.6432	0.5519	0.5268	0.5165	0.0627
MCM3	0.3765	0.6869	0.5271	0.4763	0.5167	0.0648
SLC38A1	0.3053	0.6635	0.5737	0.5244	0.5168	0.0761
MPDU1	0.3933	0.6158	0.5630	0.4965	0.5172	0.0479
C12orf32	0.4187	0.6792	0.4622	0.5104	0.5176	0.0570
LOC729964	0.3491	0.5792	0.6490	0.4954	0.5182	0.0645
XPOT	0.2793	0.6693	0.5675	0.5568	0.5182	0.0836
LOC134997	0.4134	0.5835	0.6145	0.4636	0.5187	0.0479
MCM5	0.4005	0.6763	0.4814	0.5172	0.5188	0.0579
LOC728643	0.3187	0.6848	0.5527	0.5225	0.5197	0.0757
POLE2	0.3892	0.6211	0.5802	0.4914	0.5205	0.0514
KNTC1	0.4570	0.6800	0.5056	0.4408	0.5209	0.0548
GPSM2	0.3707	0.6885	0.4785	0.5506	0.5221	0.0667
GINS3	0.3817	0.6362	0.5493	0.5215	0.5222	0.0528
ATF4	0.4973	0.5565	0.5545	0.4844	0.5232	0.0189
CCDC86	0.4001	0.6482	0.5994	0.4490	0.5242	0.0592
NT5DC2	0.3440	0.6523	0.5289	0.5723	0.5244	0.0653
UBE2T	0.3760	0.7733	0.5417	0.4084	0.5249	0.0902
MTHFD2	0.4124	0.6193	0.5377	0.5317	0.5253	0.0426
SERP1	0.3922	0.5994	0.6263	0.4855	0.5258	0.0540
SMC4	0.3392	0.6124	0.5232	0.6327	0.5269	0.0669
NAP1L1	0.3539	0.5496	0.6642	0.5404	0.5270	0.0642
DEPDC1B	0.3838	0.6733	0.5295	0.5224	0.5272	0.0591

MCM10	0.3773	0.5880	0.6526	0.4921	0.5275	0.0599
MCM6	0.3508	0.7271	0.5413	0.4913	0.5276	0.0778
LOC650157	0.4105	0.6068	0.6682	0.4284	0.5285	0.0643
PFKP	0.4405	0.7169	0.4811	0.4767	0.5288	0.0633
TMEM48	0.3852	0.7184	0.5391	0.4748	0.5294	0.0705
CCDC58	0.3758	0.5415	0.5367	0.6658	0.5300	0.0594
CDC25B	0.4150	0.6919	0.4860	0.5295	0.5306	0.0587
POLQ	0.3833	0.6694	0.5187	0.5522	0.5309	0.0588
MTHFD1L	0.4605	0.5930	0.4982	0.5753	0.5318	0.0314
NUDT1	0.4234	0.6338	0.5197	0.5509	0.5320	0.0435
SLC25A15	0.3398	0.6291	0.6011	0.5606	0.5327	0.0658
RNASEH2A	0.3390	0.7165	0.5696	0.5057	0.5327	0.0782
FIGNL1	0.3614	0.6543	0.5734	0.5424	0.5329	0.0618
MLF1IP	0.3766	0.5634	0.5864	0.6054	0.5330	0.0528
CENTA1	0.4568	0.6013	0.5352	0.5408	0.5335	0.0296
ATF5	0.3892	0.6879	0.5497	0.5092	0.5340	0.0616
U2AF2	0.4021	0.7090	0.5261	0.5011	0.5346	0.0640
GLT25D1	0.3951	0.6361	0.5348	0.5876	0.5384	0.0521
BUB1	0.3928	0.4831	0.6664	0.6143	0.5391	0.0622
IMPDH1	0.4255	0.6419	0.5754	0.5184	0.5403	0.0458
MGC40489	0.3553	0.7141	0.6076	0.4858	0.5407	0.0774
KPNA2	0.3154	0.6750	0.6260	0.5485	0.5412	0.0796
SNORD96A	0.4103	0.6339	0.5320	0.5892	0.5413	0.0484
WARS	0.4096	0.7114	0.5353	0.5103	0.5417	0.0628
HNRPA2B1	0.4070	0.6408	0.6275	0.4946	0.5425	0.0559
CHAF1B	0.3784	0.7668	0.5095	0.5160	0.5427	0.0812
ADM2	0.5247	0.5758	0.5310	0.5448	0.5441	0.0114
SLC1A5	0.5785	0.5435	0.6264	0.4295	0.5445	0.0419
POLE3	0.3478	0.7399	0.6001	0.4918	0.5449	0.0830
ZAK	0.3993	0.6271	0.5928	0.5625	0.5454	0.0505
CDCA2	0.3709	0.6571	0.5416	0.6129	0.5456	0.0629
TRAPPC3	0.4450	0.6752	0.5214	0.5412	0.5457	0.0479
LOC644774	0.3370	0.8430	0.5137	0.4901	0.5460	0.1065
RRP1B	0.4347	0.5859	0.5201	0.6439	0.5462	0.0449
F12	0.3660	0.7693	0.4952	0.5544	0.5462	0.0841
WDR43	0.3554	0.7379	0.6182	0.4754	0.5467	0.0834
TXNDC5	0.4152	0.7118	0.5277	0.5391	0.5484	0.0612
LOC652595	0.3635	0.7768	0.5020	0.5527	0.5487	0.0859
CTSL2	0.4133	0.7613	0.5458	0.4767	0.5493	0.0757
SNHG3-RCC1	0.3526	0.7287	0.5501	0.5667	0.5495	0.0770
PLOD3	0.4789	0.5745	0.6426	0.5039	0.5500	0.0369
SUV39H1	0.3814	0.6490	0.6406	0.5292	0.5501	0.0625
CENPE	0.4136	0.6486	0.5613	0.5783	0.5505	0.0494
CHDH	0.4667	0.6307	0.5563	0.5506	0.5511	0.0335
RFC5	0.4460	0.6172	0.6140	0.5307	0.5520	0.0406
SLC44A1	0.4149	0.7385	0.5481	0.5079	0.5524	0.0680

CDCA7	0.3753	0.7220	0.5295	0.5826	0.5524	0.0716
MNS1	0.4224	0.6583	0.6216	0.5071	0.5524	0.0540
RRM1	0.3799	0.6479	0.6397	0.5431	0.5527	0.0623
ASF1B	0.3376	0.7221	0.5662	0.5855	0.5529	0.0797
NP	0.3750	0.7855	0.6273	0.4271	0.5537	0.0945
EARS2	0.5142	0.6358	0.5930	0.4773	0.5551	0.0361
KIF2C	0.4189	0.6873	0.5302	0.5851	0.5554	0.0559
SERBP1	0.5138	0.6730	0.6067	0.4335	0.5568	0.0525
POLA2	0.4443	0.7049	0.5128	0.5671	0.5573	0.0552
TIMELESS	0.3465	0.8643	0.5412	0.4832	0.5588	0.1097
CDCA4	0.4293	0.6519	0.5581	0.6000	0.5598	0.0475
HNRNPH1	0.4311	0.7132	0.5975	0.4998	0.5604	0.0613
USP3	0.5477	0.6945	0.5137	0.4867	0.5606	0.0463
RAD54L	0.4731	0.6455	0.5437	0.5832	0.5614	0.0361
ASNS	0.4867	0.7339	0.5402	0.4850	0.5614	0.0589
HEATR2	0.4947	0.6731	0.5608	0.5198	0.5621	0.0394
C19orf51	0.4547	0.6463	0.5204	0.6271	0.5621	0.0453
LGI2	0.4040	0.6841	0.5435	0.6178	0.5624	0.0601
CENPK	0.3523	0.7505	0.6199	0.5274	0.5625	0.0837
KIAA0391	0.4647	0.6511	0.5970	0.5475	0.5651	0.0396
FOXM1	0.4340	0.6622	0.5467	0.6210	0.5660	0.0501
LOC402644	0.3326	0.6760	0.7100	0.5461	0.5662	0.0855
TMEM194A	0.4009	0.7021	0.6010	0.5634	0.5669	0.0626
OVGP1	0.4206	0.7172	0.6267	0.5045	0.5672	0.0655
ZNF621	0.4735	0.6693	0.5902	0.5386	0.5679	0.0414
C7orf44	0.3848	0.7265	0.6014	0.5596	0.5681	0.0706
LOC653874	0.3709	0.6401	0.7355	0.5272	0.5684	0.0784
TRIM47	0.4799	0.6224	0.5566	0.6169	0.5689	0.0332
DKFZp686O2	0.3835	0.7522	0.5286	0.6119	0.5691	0.0772
VGF	0.4562	0.7061	0.4919	0.6286	0.5707	0.0585
ECT2	0.4546	0.6644	0.6695	0.4953	0.5710	0.0561
DIS3L	0.4007	0.7155	0.6062	0.5631	0.5714	0.0653
EXOSC9	0.3999	0.6956	0.6640	0.5283	0.5719	0.0679
MGC39900	0.4573	0.6865	0.5776	0.5678	0.5723	0.0468
HIST1H2BK	0.4623	0.7648	0.5685	0.4949	0.5727	0.0678
CDC7	0.4713	0.6289	0.7110	0.4799	0.5728	0.0586
MOCOS	0.4204	0.6880	0.6057	0.5822	0.5741	0.0560
SLC16A10	0.3624	0.6950	0.6487	0.5914	0.5744	0.0737
ANLN	0.3240	0.7131	0.6456	0.6154	0.5745	0.0860
C15orf23	0.4399	0.7349	0.6003	0.5236	0.5747	0.0627
GALK1	0.4409	0.7242	0.6326	0.5023	0.5750	0.0638
CCDC34	0.4174	0.7037	0.6317	0.5478	0.5752	0.0615
ATP5C1	0.4365	0.5574	0.7964	0.5123	0.5756	0.0777
LOC92659	0.3766	0.6496	0.6839	0.5952	0.5763	0.0690
SLC29A2	0.4057	0.7177	0.6631	0.5198	0.5766	0.0706
SLC25A19	0.3780	0.7307	0.6888	0.5108	0.5771	0.0817

TROAP	0.4462	0.6526	0.6153	0.5950	0.5773	0.0453
LOC1001343	0.4063	0.8331	0.5615	0.5091	0.5775	0.0911
FANCG	0.3631	0.7845	0.6220	0.5411	0.5777	0.0876
BZW2	0.3915	0.6759	0.6790	0.5691	0.5789	0.0675
LOC645436	0.3125	0.8380	0.6131	0.5523	0.5790	0.1080
MT1F	0.5375	0.6134	0.5814	0.5843	0.5791	0.0156
PRMT3	0.3839	0.6893	0.7212	0.5243	0.5797	0.0782
LYAR	0.3948	0.7439	0.6912	0.4905	0.5801	0.0824
NPTX2	0.4116	0.6490	0.6024	0.6576	0.5802	0.0575
TTF2	0.4015	0.7409	0.6154	0.5633	0.5803	0.0703
RPRML	0.3798	0.7375	0.6180	0.5868	0.5805	0.0744
CCNE2	0.3537	0.7942	0.5757	0.5986	0.5805	0.0901
RAB11FIP2	0.4580	0.7544	0.5542	0.5597	0.5816	0.0622
LOC652903	0.3879	0.7585	0.6044	0.5827	0.5834	0.0760
RFC3	0.4295	0.6841	0.6263	0.5982	0.5845	0.0547
NR2C2AP	0.5581	0.7472	0.6056	0.4276	0.5847	0.0660
MCOLN2	0.4009	0.7467	0.6563	0.5352	0.5848	0.0751
LOC1001305	0.4256	0.7876	0.5763	0.5503	0.5849	0.0751
C9orf140	0.5019	0.7057	0.5713	0.5629	0.5854	0.0430
TRNP1	0.3809	0.6361	0.6031	0.7236	0.5859	0.0729
LOC392285	0.4763	0.6635	0.7047	0.4994	0.5860	0.0575
LOC650215	0.4816	0.7680	0.5591	0.5353	0.5860	0.0628
LOC727761	0.3795	0.8183	0.5887	0.5577	0.5860	0.0901
ADSS	0.4778	0.7221	0.5894	0.5551	0.5861	0.0510
C6orf173	0.4930	0.5922	0.6656	0.5937	0.5861	0.0355
EXO1	0.4512	0.6344	0.6619	0.5990	0.5866	0.0469
KIF20B	0.4385	0.7196	0.6127	0.5763	0.5868	0.0580
SRPK1	0.5134	0.8415	0.5223	0.4706	0.5870	0.0856
SAC3D1	0.4521	0.6698	0.6502	0.5779	0.5875	0.0493
STIL	0.4783	0.6317	0.6978	0.5423	0.5875	0.0484
TAGLN2	0.4302	0.7743	0.5694	0.5771	0.5877	0.0708
TMEM20	0.4004	0.6847	0.6596	0.6088	0.5884	0.0646
SEPHS1	0.4343	0.6724	0.6939	0.5587	0.5898	0.0597
C17orf58	0.5047	0.7221	0.5962	0.5372	0.5901	0.0479
AIF1L	0.4509	0.7850	0.5978	0.5280	0.5904	0.0715
LOC1001291	0.4372	0.7748	0.5789	0.5709	0.5904	0.0695
CDC42SE1	0.4699	0.6840	0.6203	0.5876	0.5905	0.0449
NME1-NME2	0.4329	0.7705	0.6949	0.4649	0.5908	0.0836
GINS4	0.4476	0.6218	0.6350	0.6606	0.5912	0.0486
CDK2	0.4219	0.8328	0.5879	0.5260	0.5922	0.0872
CKLF	0.4278	0.7633	0.6773	0.5008	0.5923	0.0774
NDC80	0.4292	0.6029	0.7309	0.6073	0.5926	0.0620
MT1A	0.4581	0.7729	0.6717	0.4696	0.5931	0.0774
FANCI	0.4313	0.6742	0.6858	0.5812	0.5931	0.0588
C9orf58	0.4899	0.7082	0.5902	0.5847	0.5933	0.0447
AK3L1	0.4573	0.7531	0.5996	0.5650	0.5938	0.0611

IL17RB	0.4646	0.6923	0.6556	0.5653	0.5945	0.0509
NCAPG2	0.4938	0.6655	0.6138	0.6055	0.5946	0.0361
LOC653505	0.4565	0.7426	0.6294	0.5500	0.5946	0.0607
PCNA	0.4121	0.6230	0.7757	0.5685	0.5948	0.0751
ISOC2	0.4771	0.7179	0.6517	0.5336	0.5951	0.0548
PPIL5	0.3750	0.7610	0.6497	0.5959	0.5954	0.0811
PPPDE1	0.6091	0.7221	0.5601	0.4924	0.5959	0.0484
LOC645385	0.4084	0.7503	0.6613	0.5660	0.5965	0.0731
C12orf48	0.4902	0.6721	0.6632	0.5618	0.5968	0.0435
RBMX	0.4578	0.6944	0.7136	0.5231	0.5972	0.0632
CCDC99	0.4219	0.8674	0.6249	0.4753	0.5973	0.0997
MND1	0.4739	0.6307	0.7003	0.5864	0.5978	0.0475
LOC1001316	0.4139	0.8358	0.5758	0.5661	0.5979	0.0875
BRI3BP	0.3977	0.7399	0.6838	0.5705	0.5980	0.0755
POLR2F	0.4903	0.7484	0.5936	0.5597	0.5980	0.0545
CDC25C	0.4866	0.7335	0.6121	0.5618	0.5985	0.0519
CXCR7	0.4690	0.8090	0.5829	0.5393	0.6001	0.0735
LIN9	0.5255	0.5910	0.7228	0.5617	0.6003	0.0430
NT5C3	0.4116	0.7298	0.6857	0.5762	0.6008	0.0709
SPATA5L1	0.5260	0.6497	0.7150	0.5146	0.6013	0.0487
SLBP	0.3899	0.7951	0.6238	0.5977	0.6016	0.0830
MT1X	0.4285	0.7115	0.6796	0.5872	0.6017	0.0635
LOC81691	0.4480	0.7901	0.5610	0.6082	0.6018	0.0712
LOC727803	0.4514	0.7584	0.7203	0.4787	0.6022	0.0798
RCC1	0.4317	0.7291	0.6136	0.6350	0.6023	0.0622
UCHL5	0.3936	0.7695	0.6787	0.5681	0.6025	0.0809
CTXN1	0.4897	0.6456	0.6281	0.6476	0.6027	0.0379
NUP85	0.4758	0.7894	0.5945	0.5515	0.6028	0.0669
CDC25A	0.4082	0.8234	0.6004	0.5795	0.6029	0.0852
LOC402112	0.3081	0.9262	0.6725	0.5063	0.6033	0.1309
DHX33	0.4041	0.8286	0.6866	0.4955	0.6037	0.0953
GTSE1	0.5293	0.6870	0.6509	0.5492	0.6041	0.0384
EIF3CL	0.4173	0.8288	0.5635	0.6073	0.6042	0.0852
UTP11L	0.4378	0.8244	0.6297	0.5258	0.6044	0.0832
C1orf112	0.4644	0.7008	0.6190	0.6349	0.6048	0.0500
CBFB	0.4555	0.7994	0.6223	0.5426	0.6049	0.0732
GMFB	0.4920	0.6641	0.7285	0.5357	0.6051	0.0550
DHX15	0.4568	0.7781	0.7083	0.4774	0.6051	0.0811
C4orf46	0.5235	0.6228	0.6884	0.5861	0.6052	0.0345
LOC729816	0.4586	0.7347	0.7183	0.5097	0.6053	0.0708
XRCC3	0.4892	0.7767	0.5570	0.6005	0.6058	0.0614
FBL	0.3913	0.8243	0.6354	0.5745	0.6064	0.0893
WDR34	0.4779	0.7525	0.6627	0.5326	0.6064	0.0622
KCTD20	0.4654	0.7067	0.6054	0.6483	0.6065	0.0514
SNHG4	0.4033	0.7662	0.5499	0.7078	0.6068	0.0818
ST6GALNAC4	0.4600	0.7307	0.6284	0.6103	0.6073	0.0558

LOC643300	0.4467	0.7523	0.7909	0.4402	0.6076	0.0951
ZWINT	0.4496	0.7647	0.6601	0.5573	0.6079	0.0677
SLC20A1	0.4144	0.8301	0.6531	0.5345	0.6080	0.0886
RPL22	0.4974	0.8027	0.5796	0.5531	0.6082	0.0671
STC2	0.6133	0.7876	0.5031	0.5294	0.6084	0.0642
WEE1	0.3979	0.8628	0.6046	0.5708	0.6090	0.0959
EFHD2	0.4682	0.7102	0.6218	0.6379	0.6095	0.0509
PGK1	0.4237	0.9386	0.6019	0.4762	0.6101	0.1157
SIGMAR1	0.4531	0.7767	0.6603	0.5518	0.6105	0.0697
LOC1001333	0.3519	0.8802	0.7147	0.4954	0.6105	0.1168
MYC	0.4216	0.8202	0.6778	0.5228	0.6106	0.0875
LOC1001296	0.5382	0.7742	0.5987	0.5316	0.6107	0.0566
SKA1	0.5371	0.6664	0.5432	0.6990	0.6114	0.0417
DPAGT1	0.4982	0.7984	0.6172	0.5320	0.6115	0.0672
LOC1001325	0.3930	0.8867	0.6562	0.5127	0.6121	0.1061
PSMG1	0.3860	0.7691	0.7474	0.5464	0.6122	0.0906
EIF4EBP1	0.6543	0.6802	0.5235	0.5941	0.6130	0.0349
PMM2	0.4151	0.7660	0.7124	0.5588	0.6131	0.0793
HDGF	0.5023	0.7505	0.6702	0.5304	0.6134	0.0586
CNKSR3	0.5273	0.9059	0.5568	0.4643	0.6136	0.0993
MMACHC	0.4330	0.7791	0.6896	0.5538	0.6139	0.0760
LOC643997	0.4659	0.7745	0.6871	0.5283	0.6140	0.0709
PHGDH	0.5011	0.7126	0.6571	0.5864	0.6143	0.0457
RANBP1	0.4068	0.8080	0.7287	0.5143	0.6145	0.0929
GMNN	0.5547	0.6227	0.7950	0.4872	0.6149	0.0661
LOC128192	0.3753	0.6963	0.8521	0.5368	0.6151	0.1026
C1orf174	0.4475	0.7821	0.6593	0.5727	0.6154	0.0705
C20orf27	0.4543	0.7693	0.6645	0.5750	0.6158	0.0669
MRPS2	0.5296	0.7406	0.6603	0.5328	0.6158	0.0515
LEPROTL1	0.4793	0.7026	0.7435	0.5382	0.6159	0.0636
TUBG1	0.5580	0.7616	0.5918	0.5530	0.6161	0.0493
L2HGDH	0.4929	0.7998	0.5550	0.6188	0.6166	0.0662
PTGFR	0.4447	0.7229	0.6196	0.6817	0.6172	0.0613
C11orf75	0.5742	0.8181	0.4949	0.5824	0.6174	0.0697
IMPAD1	0.4657	0.7064	0.7771	0.5205	0.6174	0.0741
DSC2	0.4614	0.8309	0.6405	0.5390	0.6180	0.0799
EZH2	0.4649	0.7917	0.6206	0.5963	0.6183	0.0671
CNOT6	0.5095	0.7111	0.6677	0.5864	0.6187	0.0446
CHMP5	0.4216	0.7480	0.7573	0.5481	0.6188	0.0815
HOOK1	0.4360	0.7235	0.6821	0.6337	0.6188	0.0636
STMN1	0.4856	0.7895	0.6279	0.5724	0.6188	0.0640
SPRYD4	0.4647	0.7480	0.6617	0.6015	0.6190	0.0596
HNRNPA1	0.3875	0.9259	0.5480	0.6162	0.6194	0.1128
ANGEL1	0.4972	0.8060	0.5771	0.5993	0.6199	0.0658
DSCC1	0.3812	0.7196	0.6900	0.6908	0.6204	0.0800
MRPL23	0.5148	0.8067	0.6420	0.5183	0.6205	0.0688

DCK	0.4623	0.8545	0.5716	0.5946	0.6207	0.0831
KLHDC5	0.4956	0.7484	0.6280	0.6140	0.6215	0.0517
C12orf24	0.3952	0.9127	0.6111	0.5673	0.6216	0.1076
LSMD1	0.4629	0.7906	0.7202	0.5148	0.6221	0.0790
LSM2	0.4739	0.7575	0.6401	0.6175	0.6222	0.0582
RDH13	0.3860	0.8216	0.7059	0.5773	0.6227	0.0933
RSRC1	0.4406	0.7359	0.7072	0.6075	0.6228	0.0667
SNRPA1	0.4269	0.7963	0.7083	0.5598	0.6228	0.0815
CHTF18	0.5395	0.8350	0.5702	0.5477	0.6231	0.0709
AADAT	0.4940	0.7967	0.6487	0.5534	0.6232	0.0660
LYSMD2	0.4289	0.7890	0.7443	0.5314	0.6234	0.0858
ANG	0.5974	0.8084	0.4991	0.5888	0.6234	0.0655
BUB3	0.4817	0.7580	0.6564	0.6011	0.6243	0.0576
ADA	0.4567	0.8704	0.6030	0.5676	0.6244	0.0877
ME2	0.4335	0.8668	0.6696	0.5280	0.6245	0.0942
VRK1	0.3910	0.7376	0.7995	0.5721	0.6251	0.0916
CLIC4	0.5770	0.7312	0.6349	0.5583	0.6253	0.0389
ACTR2	0.5765	0.9046	0.5333	0.4874	0.6255	0.0948
LSM14A	0.5661	0.7533	0.5979	0.5851	0.6256	0.0431
ZPLD1	0.5376	0.6656	0.7454	0.5543	0.6257	0.0490
LOC653071	0.4749	0.8548	0.7192	0.4548	0.6259	0.0971
TP53RK	0.4914	0.9005	0.5243	0.5890	0.6263	0.0936
C20orf72	0.4557	0.8980	0.6192	0.5333	0.6266	0.0964
SSR1	0.4919	0.7117	0.7620	0.5415	0.6268	0.0652
DPH2	0.4803	0.7615	0.6986	0.5668	0.6268	0.0635
MT2A	0.5505	0.9342	0.5519	0.4738	0.6276	0.1038
LY6E	0.4266	0.9271	0.6625	0.4945	0.6277	0.1114
EDG4	0.4612	0.7864	0.6418	0.6216	0.6277	0.0665
YARS2	0.5105	0.7253	0.7044	0.5731	0.6283	0.0517
NOP58	0.4614	0.7214	0.7763	0.5542	0.6283	0.0730
NEK2	0.5227	0.7441	0.6171	0.6299	0.6285	0.0453
YWHAZ	0.4408	0.8187	0.6532	0.6029	0.6289	0.0778
BOLA2	0.4612	0.8903	0.6078	0.5567	0.6290	0.0922
VEGFB	0.5362	0.7437	0.6949	0.5428	0.6294	0.0529
HNRNPM	0.5290	0.7996	0.5984	0.5910	0.6295	0.0588
LOC728732	0.4363	0.8098	0.7411	0.5342	0.6303	0.0873
LOC1001327	0.4624	0.7605	0.7480	0.5508	0.6304	0.0738
BLMH	0.3875	0.7777	0.6817	0.6768	0.6309	0.0844
ECE2	0.4536	0.6738	0.6995	0.6970	0.6310	0.0594
LBR	0.4362	0.8703	0.6354	0.5844	0.6316	0.0901
GEMIN4	0.4536	0.8210	0.6938	0.5604	0.6322	0.0798
FRAG1	0.5123	0.7630	0.6834	0.5703	0.6322	0.0562
ALG3	0.4961	0.7953	0.6452	0.5937	0.6326	0.0624
CAPN5	0.5805	0.8130	0.6066	0.5313	0.6328	0.0620
NDUFB4	0.4578	0.8984	0.6397	0.5361	0.6330	0.0960
PIGW	0.3962	0.8326	0.6847	0.6188	0.6331	0.0907

MYO5C	0.5614	0.8035	0.5861	0.5817	0.6332	0.0570
LOC1001317	0.4498	0.9648	0.5898	0.5286	0.6333	0.1142
LYRM7	0.4766	0.8078	0.6204	0.6286	0.6333	0.0678
ORC1L	0.4492	0.7322	0.6340	0.7185	0.6334	0.0651
LOC391532	0.5720	0.6678	0.7248	0.5693	0.6335	0.0381
PNN	0.5256	0.7227	0.7618	0.5242	0.6336	0.0632
HADH	0.4454	0.7753	0.7028	0.6111	0.6336	0.0712
MRPL24	0.5050	0.7613	0.6924	0.5764	0.6338	0.0574
WNT10B	0.4826	0.8055	0.6686	0.5797	0.6341	0.0686
LOC649555	0.4243	0.8898	0.7251	0.4982	0.6343	0.1065
PIR	0.4365	0.7720	0.7085	0.6207	0.6344	0.0729
MLH1	0.5281	0.7800	0.7017	0.5312	0.6352	0.0630
C19orf23	0.5238	0.7232	0.6865	0.6096	0.6357	0.0442
TMEM52	0.4359	0.8518	0.6701	0.5881	0.6365	0.0866
YWHAH	0.4527	0.8393	0.7067	0.5478	0.6366	0.0855
ARPP19	0.5069	0.7137	0.6916	0.6344	0.6367	0.0464
CHAF1A	0.4798	0.7474	0.6531	0.6666	0.6367	0.0563
MTMR9	0.4776	0.8768	0.6203	0.5744	0.6373	0.0852
YARS	0.5351	0.7699	0.6729	0.5720	0.6375	0.0529
MGC70857	0.5944	0.8523	0.5896	0.5150	0.6378	0.0738
SFXN4	0.4258	0.8813	0.6588	0.5868	0.6382	0.0945
PGRMC2	0.6405	0.7085	0.6978	0.5060	0.6382	0.0465
DLAT	0.4944	0.7394	0.7254	0.5950	0.6386	0.0580
LOC643319	0.4562	0.8391	0.6100	0.6496	0.6387	0.0787
HNRNPC	0.5613	0.8023	0.6380	0.5540	0.6389	0.0577
ANP32B	0.4939	0.7642	0.6476	0.6502	0.6390	0.0555
PIGC	0.4941	0.7495	0.7169	0.5973	0.6394	0.0585
RPL36A	0.4285	0.7973	0.7457	0.5870	0.6396	0.0834
UBE2Q2	0.5002	0.7151	0.8374	0.5061	0.6397	0.0827
TP53	0.5061	0.7675	0.7001	0.5862	0.6400	0.0582
TMED5	0.4692	0.8336	0.6928	0.5644	0.6400	0.0791
IMPDH2	0.3819	0.9326	0.7817	0.4645	0.6402	0.1301
NUDT19	0.3939	0.9322	0.6593	0.5767	0.6405	0.1119
HNRPA1L-2	0.5444	0.7430	0.7541	0.5230	0.6411	0.0622
ANKRD16	0.5767	0.7869	0.6376	0.5642	0.6413	0.0511
WNT10A	0.5257	0.7704	0.5761	0.6942	0.6416	0.0556
TUBA1B	0.4597	0.8056	0.8051	0.4962	0.6417	0.0948
VBP1	0.4883	0.8262	0.7044	0.5509	0.6425	0.0762
EBNA1BP2	0.4401	0.8092	0.7842	0.5365	0.6425	0.0913
ASCC1	0.5464	0.8177	0.6423	0.5644	0.6427	0.0619
BMP6	0.4651	0.7385	0.7666	0.6011	0.6428	0.0694
LOC1001328	0.4826	0.7989	0.7212	0.5702	0.6432	0.0715
NTHL1	0.5210	0.8991	0.5271	0.6272	0.6436	0.0886
MLST8	0.4900	0.8330	0.7114	0.5412	0.6439	0.0788
LOC1001340	0.4096	0.9160	0.6893	0.5611	0.6440	0.1072
C17orf53	0.5014	0.7801	0.6231	0.6714	0.6440	0.0578

MRPL15	0.5319	0.7324	0.8117	0.5000	0.6440	0.0760
CACYBP	0.4500	0.8949	0.6394	0.5923	0.6442	0.0928
LRP8	0.4858	0.7934	0.6481	0.6503	0.6444	0.0629
HIRIP3	0.4787	0.8365	0.6996	0.5629	0.6444	0.0785
SLC4A7	0.4903	0.8105	0.7104	0.5669	0.6445	0.0717
IARS	0.5438	0.8622	0.6261	0.5474	0.6449	0.0749
LEF1	0.5921	0.7182	0.6264	0.6430	0.6449	0.0266
PRR7	0.4954	0.8623	0.6191	0.6040	0.6452	0.0774
LOC1001341	0.5925	0.8059	0.5769	0.6063	0.6454	0.0538
RCN1	0.6034	0.7084	0.7314	0.5392	0.6456	0.0451
TMPO	0.4912	0.8105	0.6465	0.6352	0.6459	0.0653
RPL29	0.4099	0.9096	0.7305	0.5351	0.6463	0.1098
LOC645691	0.4463	0.7551	0.8065	0.5777	0.6464	0.0828
TMEM97	0.4580	0.8551	0.6930	0.5817	0.6470	0.0844
LOC1001301	0.4633	0.7917	0.7782	0.5554	0.6472	0.0818
EBP	0.5044	0.8470	0.6642	0.5731	0.6472	0.0742
LOC388275	0.4363	1.0004	0.6205	0.5319	0.6472	0.1236
SNHG9	0.5969	0.7991	0.7134	0.4810	0.6476	0.0693
C13orf37	0.4527	0.7914	0.7368	0.6099	0.6477	0.0753
LOC493869	0.5134	0.6992	0.7658	0.6125	0.6477	0.0547
LOC1001305	0.4665	0.8581	0.6184	0.6479	0.6477	0.0806
SFRS3	0.5431	0.8096	0.7121	0.5281	0.6482	0.0681
FAM136A	0.4913	0.8735	0.6811	0.5479	0.6484	0.0849
DNMT1	0.5378	0.8196	0.7068	0.5299	0.6485	0.0701
FAM116A	0.5207	0.7713	0.7390	0.5634	0.6486	0.0625
ZNF239	0.4467	0.7879	0.7382	0.6226	0.6489	0.0758
TIMM23	0.5326	0.8216	0.6761	0.5659	0.6490	0.0652
MRPS6	0.5079	0.7667	0.7406	0.5831	0.6496	0.0623
CMTM7	0.4570	0.8151	0.7403	0.5872	0.6499	0.0799
HNRPA1P4	0.4295	1.0410	0.6469	0.4828	0.6501	0.1383
SNRPB	0.5166	0.8046	0.6993	0.5808	0.6503	0.0638
TACC3	0.4993	0.8049	0.6912	0.6059	0.6503	0.0648
CYC1	0.5100	0.8960	0.6609	0.5354	0.6506	0.0882
HSPC111	0.4850	0.8638	0.6624	0.5927	0.6510	0.0798
ALG6	0.4753	0.7758	0.7337	0.6207	0.6514	0.0672
MGC61598	0.5211	0.9444	0.6036	0.5393	0.6521	0.0990
GHR	0.4580	0.8162	0.7647	0.5698	0.6522	0.0837
FKBP11	0.5613	0.8462	0.6618	0.5395	0.6522	0.0699
DONSON	0.5403	0.7463	0.6827	0.6405	0.6524	0.0432
ALG8	0.5111	0.8186	0.7090	0.5718	0.6526	0.0691
ADCY3	0.5454	0.8150	0.6799	0.5708	0.6528	0.0614
LOC643357	0.5541	0.8155	0.6701	0.5720	0.6529	0.0599
TIMM9	0.5126	0.7420	0.7675	0.5912	0.6533	0.0609
SNRPA	0.5471	0.8554	0.6411	0.5700	0.6534	0.0702
PAQR4	0.5628	0.8535	0.5543	0.6476	0.6546	0.0696
NIPA1	0.6093	0.7797	0.7177	0.5119	0.6546	0.0592

NOP56	0.4377	0.7665	0.8641	0.5507	0.6547	0.0976
TGM3	0.4740	0.9725	0.6157	0.5573	0.6549	0.1098
PTRH1	0.4475	0.8667	0.6938	0.6116	0.6549	0.0872
HSD3B7	0.5336	0.8342	0.6443	0.6086	0.6552	0.0640
EI24	0.4843	0.7981	0.7655	0.5731	0.6552	0.0756
CCND1	0.5447	0.8687	0.6407	0.5675	0.6554	0.0740
TH1L	0.5062	0.9402	0.6052	0.5706	0.6556	0.0971
FAM91A1	0.5220	0.8535	0.6754	0.5724	0.6558	0.0732
PSRC1	0.4795	0.7659	0.7200	0.6586	0.6560	0.0628
RNMT	0.5018	0.8293	0.6871	0.6064	0.6561	0.0691
NIF3L1	0.5344	0.7674	0.7490	0.5740	0.6562	0.0596
ATL2	0.5199	0.7693	0.6825	0.6539	0.6564	0.0517
PUS7	0.4809	0.7754	0.6978	0.6723	0.6566	0.0626
TYSND1	0.5046	0.8404	0.6978	0.5847	0.6569	0.0729
ZCCHC17	0.5143	0.8631	0.6296	0.6235	0.6576	0.0734
SFRS10	0.4494	0.9264	0.7526	0.5028	0.6578	0.1113
C16orf33	0.5068	0.8283	0.7108	0.5864	0.6581	0.0706
MTERFD1	0.4450	0.8759	0.6851	0.6268	0.6582	0.0888
TIMM10	0.5144	0.7691	0.7494	0.5998	0.6582	0.0610
CCT6A	0.4885	0.8309	0.8177	0.4986	0.6589	0.0955
RAI14	0.5185	0.8627	0.5769	0.6780	0.6590	0.0755
CHEK2	0.5058	0.7593	0.7208	0.6503	0.6590	0.0558
PIK3C2A	0.5447	0.7922	0.7310	0.5683	0.6591	0.0607
TMEM194	0.4671	0.7992	0.7421	0.6278	0.6591	0.0732
LDHB	0.4290	0.8108	0.8383	0.5585	0.6591	0.0992
MRPL9	0.6094	0.7379	0.7265	0.5632	0.6592	0.0432
MBP	0.5047	0.8108	0.7026	0.6198	0.6595	0.0647
PPTC7	0.4592	0.9071	0.6500	0.6229	0.6598	0.0926
IMPA2	0.5675	0.8885	0.6132	0.5701	0.6598	0.0770
LOC653506	0.5866	0.8535	0.6658	0.5345	0.6601	0.0699
NARG1	0.5008	0.7731	0.7260	0.6405	0.6601	0.0598
SPIN4	0.4888	0.7768	0.7995	0.5753	0.6601	0.0762
TBRG4	0.5658	0.7870	0.6582	0.6296	0.6602	0.0465
LOC642031	0.5413	0.9046	0.6682	0.5277	0.6604	0.0873
FCRLB	0.6280	0.7656	0.6351	0.6139	0.6607	0.0353
STT3B	0.5046	0.7721	0.7389	0.6276	0.6608	0.0605
FANCD2	0.5409	0.7123	0.7486	0.6422	0.6610	0.0457
LOC1001334	0.4912	0.9363	0.5561	0.6608	0.6611	0.0982
BRIX1	0.4374	0.8050	0.7634	0.6400	0.6615	0.0825
PSMG2	0.4973	0.8650	0.7480	0.5359	0.6616	0.0874
GATC	0.4566	0.9379	0.6710	0.5812	0.6617	0.1020
NOP16	0.4641	0.9426	0.6413	0.5993	0.6618	0.1009
EEF1E1	0.6169	0.7752	0.7090	0.5482	0.6624	0.0500
ELOVL6	0.5218	0.7798	0.7298	0.6194	0.6627	0.0577
HDDC3	0.5175	0.8538	0.6196	0.6611	0.6630	0.0704
SGOL2	0.5034	0.8563	0.6272	0.6652	0.6630	0.0731

IMP4	0.4515	0.8740	0.7179	0.6089	0.6631	0.0891
KIF23	0.5238	0.7364	0.7237	0.6692	0.6633	0.0487
RAD51C	0.4617	0.8637	0.7235	0.6044	0.6633	0.0856
C19orf48	0.5326	0.8524	0.7081	0.5612	0.6635	0.0738
MYO19	0.5059	0.8173	0.6969	0.6341	0.6635	0.0649
SLC46A1	0.4975	0.8710	0.7268	0.5598	0.6638	0.0843
CCDC85B	0.4613	0.8919	0.7592	0.5429	0.6638	0.0986
SLC12A2	0.5238	0.7987	0.7177	0.6155	0.6639	0.0599
LOC730820	0.6297	0.7782	0.6053	0.6429	0.6640	0.0388
SLC19A1	0.5536	0.8313	0.6715	0.5997	0.6640	0.0608
CYP2R1	0.5833	0.7652	0.6645	0.6432	0.6640	0.0378
BOLA3	0.5404	0.8570	0.7045	0.5565	0.6646	0.0740
MRPS27	0.5203	0.8599	0.6508	0.6275	0.6647	0.0710
THOC4	0.3985	0.8890	0.7628	0.6090	0.6648	0.1056
LOC390466	0.5198	0.9324	0.6052	0.6020	0.6649	0.0914
C11orf71	0.4992	0.9240	0.7107	0.5261	0.6650	0.0983
SRM	0.4975	0.8631	0.7464	0.5533	0.6651	0.0849
TYW3	0.5148	0.8253	0.6689	0.6551	0.6660	0.0635
SLC35F2	0.5549	0.8240	0.7282	0.5575	0.6661	0.0664
ACYP1	0.5275	0.8451	0.6447	0.6472	0.6661	0.0659
LOC652481	0.5167	0.9481	0.6398	0.5612	0.6664	0.0973
SLC31A1	0.4596	0.7627	0.7434	0.7008	0.6666	0.0702
IGF2BP3	0.4953	0.8753	0.6960	0.6003	0.6667	0.0807
SNAPC4	0.5093	0.9399	0.6330	0.5852	0.6668	0.0945
LOC255783	0.5189	0.9206	0.6270	0.6008	0.6668	0.0877
RALBP1	0.5950	0.7328	0.7069	0.6327	0.6669	0.0320
CFDP1	0.6024	0.8155	0.6035	0.6464	0.6669	0.0506
DTL	0.4997	0.7683	0.7603	0.6411	0.6673	0.0630
HAUS8	0.5001	0.7892	0.7007	0.6796	0.6674	0.0606
TOMM40	0.3778	0.8605	0.7401	0.6915	0.6675	0.1029
SNX26	0.6530	0.8063	0.5757	0.6353	0.6676	0.0491
TRUB1	0.3417	1.0425	0.7496	0.5366	0.6676	0.1502
MRPL1	0.4836	0.6821	0.9848	0.5205	0.6678	0.1141
UBL3	0.5882	0.8865	0.6506	0.5462	0.6679	0.0760
PA2G4	0.4970	0.8774	0.7053	0.5921	0.6680	0.0818
SAAL1	0.4803	0.7676	0.7365	0.6875	0.6680	0.0647
DR1	0.5030	0.7194	0.8885	0.5611	0.6680	0.0866
CARS	0.6735	0.7987	0.6225	0.5775	0.6680	0.0478
PKMYT1	0.5202	0.7843	0.6675	0.7007	0.6682	0.0551
AUH	0.6119	0.8707	0.6022	0.5883	0.6683	0.0677
GLS	0.5712	0.7959	0.7696	0.5378	0.6686	0.0665
NAT10	0.5255	0.8203	0.6649	0.6639	0.6687	0.0602
E2F5	0.4917	0.9507	0.6250	0.6077	0.6688	0.0985
HMGA1	0.5655	0.7592	0.7008	0.6497	0.6688	0.0411
SNORA67	0.4338	0.8433	0.7878	0.6102	0.6688	0.0928
ARL2BP	0.4926	1.0441	0.6529	0.4862	0.6690	0.1309

SHC1	0.5918	0.7607	0.6286	0.6949	0.6690	0.0373
SMC2	0.5002	0.8036	0.7473	0.6258	0.6692	0.0675
CCDC5	0.5075	0.8413	0.6855	0.6440	0.6696	0.0687
SEH1L	0.4691	0.9198	0.6875	0.6040	0.6701	0.0946
LOC730183	0.5987	0.8433	0.6275	0.6111	0.6701	0.0580
SLC29A3	0.6240	0.7316	0.6455	0.6795	0.6702	0.0235
GFPT1	0.6415	0.7773	0.6725	0.5896	0.6702	0.0396
E2F7	0.4929	0.8043	0.6742	0.7103	0.6704	0.0652
ETFDH	0.6146	0.7622	0.7177	0.5881	0.6707	0.0414
LIN52	0.4741	0.8500	0.7860	0.5730	0.6708	0.0883
CDCA1	0.5225	0.7515	0.6654	0.7438	0.6708	0.0531
UGT2B10	0.5185	0.7892	0.7217	0.6539	0.6708	0.0578
ALB	0.4974	0.7796	0.6999	0.7091	0.6715	0.0607
LOC1001295	0.4939	0.9044	0.6865	0.6015	0.6716	0.0870
ATP5G3	0.6132	0.7927	0.6287	0.6522	0.6717	0.0411
SSRP1	0.5054	0.7624	0.7971	0.6225	0.6719	0.0671
ENDOG	0.4844	0.9970	0.6577	0.5486	0.6719	0.1141
MARS	0.7264	0.8412	0.6159	0.5045	0.6720	0.0723
KTELC1	0.4984	0.8451	0.7151	0.6295	0.6720	0.0729
C16orf48	0.5617	0.8222	0.6964	0.6077	0.6720	0.0573
C7orf23	0.5288	0.9516	0.6206	0.5873	0.6721	0.0951
ARMET	0.5411	0.8425	0.7316	0.5738	0.6722	0.0704
PCK2	0.6663	0.7961	0.6229	0.6037	0.6722	0.0433
CSE1L	0.5329	0.9181	0.7011	0.5374	0.6724	0.0908
CXCL16	0.5432	0.8417	0.6636	0.6414	0.6725	0.0622
MRPS17	0.4823	0.8036	0.8572	0.5472	0.6726	0.0927
UIMC1	0.4634	0.9692	0.6145	0.6443	0.6728	0.1064
DNAJA3	0.5048	0.9061	0.7330	0.5475	0.6729	0.0922
C3orf26	0.4766	0.9994	0.7059	0.5109	0.6732	0.1199
ACN9	0.5566	0.8408	0.7394	0.5564	0.6733	0.0705
LOC646817	0.3385	1.1014	0.7242	0.5305	0.6737	0.1629
WDR67	0.5668	0.7950	0.6319	0.7012	0.6737	0.0489
ERI2	0.5326	0.8653	0.6970	0.6011	0.6740	0.0721
PLEKHG3	0.5155	0.8561	0.6353	0.6892	0.6740	0.0707
DSN1	0.5866	0.8145	0.6961	0.6000	0.6743	0.0527
RBPJ	0.4866	0.9085	0.6681	0.6342	0.6743	0.0874
TMEM62	0.5864	0.8494	0.6026	0.6598	0.6746	0.0604
PFKFB3	0.5731	0.8387	0.6566	0.6311	0.6749	0.0573
HSPA14	0.5021	0.8594	0.7593	0.5789	0.6749	0.0818
IMMT	0.5644	0.7188	0.8477	0.5689	0.6750	0.0678
LAGE3	0.5667	0.9039	0.6779	0.5524	0.6752	0.0812
CENPL	0.4573	0.9248	0.7687	0.5509	0.6755	0.1057
HIST1H4C	0.5008	0.9305	0.7228	0.5485	0.6756	0.0974
RNASE4	0.6090	0.8435	0.5944	0.6558	0.6757	0.0575
GLRX5	0.6127	0.8257	0.7022	0.5629	0.6759	0.0576
TOMM70A	0.5136	0.8405	0.7972	0.5522	0.6759	0.0834

HDDC2	0.5348	0.7428	0.8141	0.6124	0.6760	0.0629
SCML2	0.5127	0.7701	0.7619	0.6598	0.6761	0.0600
MRPL11	0.3984	0.8506	0.7786	0.6773	0.6762	0.0992
PPIA	0.5406	0.7808	0.8117	0.5730	0.6765	0.0697
CTBP1	0.5320	0.8911	0.7028	0.5807	0.6767	0.0800
BLM	0.5631	0.7436	0.7251	0.6762	0.6770	0.0405
SNORA10	0.4984	0.8532	0.6760	0.6805	0.6770	0.0724
XRCC6BP1	0.4836	0.7929	0.8118	0.6212	0.6774	0.0775
PDHX	0.4594	0.9115	0.8338	0.5051	0.6775	0.1142
RPL23A	0.4705	0.7706	0.9078	0.5613	0.6776	0.0992
FBXO46	0.6087	0.8880	0.6082	0.6054	0.6776	0.0701
TCP1	0.5408	0.7638	0.8421	0.5648	0.6779	0.0741
BPHL	0.5463	0.8660	0.6383	0.6613	0.6780	0.0674
C3orf21	0.5824	0.8351	0.6968	0.5982	0.6781	0.0581
C14orf126	0.4839	0.9717	0.7072	0.5501	0.6782	0.1085
FAM125A	0.5976	0.7818	0.7063	0.6273	0.6782	0.0414
RPA3	0.6357	0.7829	0.7051	0.5895	0.6783	0.0422
MGC3731	0.5146	0.9605	0.6702	0.5683	0.6784	0.0994
LSM4	0.4912	0.9215	0.6830	0.6179	0.6784	0.0903
LOC648210	0.4107	0.9650	0.7643	0.5738	0.6784	0.1198
MRPS35	0.4385	0.9183	0.8042	0.5532	0.6786	0.1105
HIST1H2BJ	0.2703	1.1521	0.7512	0.5407	0.6786	0.1860
REEP4	0.6046	0.7163	0.6918	0.7023	0.6787	0.0252
NARS2	0.5983	0.7570	0.7990	0.5609	0.6788	0.0584
HNRPH1	0.6255	0.7677	0.7457	0.5762	0.6788	0.0463
TTR	0.4355	1.0754	0.6994	0.5049	0.6788	0.1435
LOC391811	0.4814	0.8669	0.6695	0.6977	0.6789	0.0790
AFMID	0.5362	0.9060	0.6857	0.5892	0.6793	0.0817
C20orf20	0.5791	0.8616	0.6555	0.6212	0.6793	0.0627
CLINT1	0.5305	0.9653	0.6971	0.5247	0.6794	0.1033
CISD1	0.4548	0.8990	0.7876	0.5764	0.6794	0.1004
C1orf93	0.7888	0.5806	0.6613	0.6874	0.6795	0.0429
LDHA	0.5513	0.9005	0.6753	0.5914	0.6796	0.0780
FAM189B	0.5128	0.9361	0.6573	0.6125	0.6797	0.0907
CBX2	0.5136	0.8285	0.6797	0.6975	0.6798	0.0646
ITGB3BP	0.6124	0.7222	0.7980	0.5867	0.6798	0.0491
ARL6IP1	0.6187	0.8760	0.6947	0.5309	0.6801	0.0734
GTF2H2B	0.4373	0.9505	0.7307	0.6038	0.6806	0.1082
PPIH	0.4779	0.9180	0.7620	0.5646	0.6806	0.0990
EXOSC6	0.4619	0.9104	0.6926	0.6581	0.6808	0.0919
DOLK	0.6016	0.8112	0.6455	0.6663	0.6812	0.0454
NXT1	0.4290	0.8820	0.7795	0.6343	0.6812	0.0982
TMEM99	0.6139	0.8806	0.6928	0.5379	0.6813	0.0736
MAPK3	0.7128	0.8508	0.5763	0.5853	0.6813	0.0645
RPL22L1	0.5881	0.8127	0.7418	0.5834	0.6815	0.0571
RRAGD	0.5678	0.7646	0.8029	0.5918	0.6818	0.0596

TMEM160	0.5579	0.9184	0.6361	0.6150	0.6819	0.0806
COQ3	0.5097	0.9250	0.6800	0.6128	0.6819	0.0883
PUS1	0.5242	0.9215	0.6931	0.5892	0.6820	0.0871
WDR54	0.4787	0.9685	0.6853	0.5955	0.6820	0.1044
USP1	0.5122	0.9060	0.6979	0.6120	0.6820	0.0838
CREBZF	0.6288	0.8875	0.6439	0.5695	0.6824	0.0702
LOC644879	0.5283	0.8767	0.7139	0.6112	0.6825	0.0750
ZBTB33	0.5545	0.8213	0.7780	0.5764	0.6825	0.0683
UCK2	0.4904	0.9030	0.7434	0.5940	0.6827	0.0899
TDG	0.5243	0.8720	0.7263	0.6093	0.6830	0.0754
GSG2	0.4939	0.7622	0.7289	0.7471	0.6831	0.0634
C21orf45	0.5863	0.7577	0.7321	0.6567	0.6832	0.0388
PDLIM1	0.5483	0.8219	0.7045	0.6582	0.6832	0.0567
LOC400506	0.4682	0.9618	0.6839	0.6194	0.6833	0.1033
HSPH1	0.5002	0.9114	0.7286	0.5934	0.6834	0.0893
TOMM5	0.5122	0.8440	0.8095	0.5679	0.6834	0.0838
TIA1	0.6078	0.8491	0.6312	0.6457	0.6835	0.0558
C20orf134	0.4662	0.9446	0.6786	0.6445	0.6835	0.0987
RCN3	0.5782	0.8204	0.7024	0.6341	0.6838	0.0521
NUP210	0.5387	0.8845	0.5759	0.7360	0.6838	0.0794
SHMT2	0.4940	0.9437	0.7200	0.5775	0.6838	0.0984
CCDC138	0.5442	0.8224	0.7795	0.5893	0.6839	0.0688
LOC391075	0.4001	1.0057	0.8260	0.5039	0.6839	0.1404
UBIAD1	0.4410	1.0763	0.6944	0.5252	0.6842	0.1409
SF3B1	0.5939	0.8499	0.6398	0.6535	0.6843	0.0567
LOC730083	0.5253	0.8530	0.7837	0.5756	0.6844	0.0793
LOC283267	0.6293	0.7474	0.6358	0.7252	0.6844	0.0303
MED16	0.6834	0.8013	0.6101	0.6430	0.6845	0.0417
PLK1	0.5749	0.7561	0.7737	0.6334	0.6845	0.0480
MRPL19	0.5244	0.7611	0.9033	0.5497	0.6846	0.0902
SLC25A10	0.5345	0.8550	0.7612	0.5881	0.6847	0.0746
GRPEL2	0.6264	0.8684	0.7449	0.5002	0.6849	0.0790
NIPA2	0.6697	0.8102	0.6416	0.6189	0.6851	0.0430
CIAPIN1	0.5588	0.8353	0.8377	0.5103	0.6855	0.0877
LOC147727	0.6319	0.8542	0.7183	0.5380	0.6856	0.0672
CSTF2	0.4614	0.8179	0.8872	0.5760	0.6856	0.1002
LOC1001329	0.5180	0.8386	0.7603	0.6259	0.6857	0.0711
MRPL2	0.5295	0.9439	0.6879	0.5823	0.6859	0.0921
C14orf106	0.5768	0.7490	0.7619	0.6561	0.6860	0.0434
NDE1	0.5931	0.8225	0.7011	0.6278	0.6861	0.0507
RPS21	0.5088	0.8258	0.7729	0.6368	0.6861	0.0713
TUBGCP4	0.5278	0.8187	0.7432	0.6547	0.6861	0.0625
LOC644877	0.4877	0.8574	0.7615	0.6408	0.6869	0.0798
BFAR	0.5480	0.8329	0.7119	0.6549	0.6869	0.0593
WRNIP1	0.5758	0.8310	0.7263	0.6151	0.6871	0.0576
RRP7A	0.5023	0.8931	0.7473	0.6057	0.6871	0.0851

NCL	0.4891	0.9259	0.7060	0.6281	0.6873	0.0913
RTN4IP1	0.5365	0.8641	0.7366	0.6127	0.6875	0.0719
UPF3B	0.6003	0.8866	0.6340	0.6292	0.6875	0.0668
RAD21	0.4875	0.9126	0.7798	0.5712	0.6878	0.0969
LOC1001343	0.4225	0.8554	0.9515	0.5218	0.6878	0.1277
MAGEA12	0.5509	0.8322	0.7415	0.6269	0.6879	0.0620
LOC643287	0.5283	0.8064	0.7998	0.6170	0.6879	0.0690
ARHGAP19	0.6245	0.8865	0.6743	0.5683	0.6884	0.0695
ADCK4	0.5755	0.9168	0.6399	0.6217	0.6885	0.0773
LOC645249	0.5093	0.8553	0.7501	0.6398	0.6886	0.0742
OXA1L	0.4953	1.0573	0.6762	0.5259	0.6887	0.1291
YEATS4	0.5551	0.8370	0.8000	0.5634	0.6889	0.0752
MRPL12	0.5346	0.8283	0.7633	0.6293	0.6889	0.0660
STAU1	0.5233	0.8211	0.8101	0.6012	0.6889	0.0749
PHLDA2	0.5814	0.8417	0.7329	0.5999	0.6890	0.0611
SLC25A22	0.4986	0.9437	0.6519	0.6624	0.6892	0.0927
EXOSC8	0.3185	1.3352	0.6191	0.4839	0.6892	0.2239
RMND1	0.5540	0.8903	0.6648	0.6477	0.6892	0.0713
CCT2	0.5180	0.8843	0.8000	0.5546	0.6892	0.0903
TACO1	0.5488	0.9410	0.6876	0.5796	0.6893	0.0890
EXOSC2	0.4959	0.9314	0.7234	0.6076	0.6896	0.0930
NME2	0.5791	0.8550	0.7078	0.6165	0.6896	0.0614
FUS	0.5382	0.7568	0.7027	0.7616	0.6898	0.0523
C13orf3	0.5997	0.6906	0.7934	0.6759	0.6899	0.0398
RPS7	0.4134	0.9170	0.7960	0.6343	0.6902	0.1089
GGA2	0.5484	0.9444	0.6795	0.5886	0.6902	0.0891
FAF2	0.6399	0.8442	0.6687	0.6091	0.6905	0.0527
RTN4R	0.5847	0.8256	0.6956	0.6563	0.6905	0.0505
HNRNPD	0.4768	0.8515	0.7987	0.6366	0.6909	0.0847
DUSP12	0.5582	0.9352	0.6379	0.6326	0.6910	0.0834
C7orf47	0.5036	0.9802	0.7341	0.5469	0.6912	0.1086
C19orf10	0.6761	0.9617	0.6212	0.5060	0.6912	0.0969
IPO4	0.6874	0.7344	0.7055	0.6382	0.6914	0.0202
PSMB10	0.5244	0.8861	0.7447	0.6113	0.6916	0.0791
GNG5	0.6679	0.8076	0.7284	0.5645	0.6921	0.0513
HEBP1	0.5627	0.8274	0.7358	0.6428	0.6922	0.0573
MRI1	0.5509	0.7877	0.7274	0.7036	0.6924	0.0504
SIP1	0.5607	0.8863	0.7009	0.6224	0.6926	0.0707
SCARB1	0.5114	0.9971	0.6683	0.5938	0.6926	0.1064
CASP2	0.5548	0.8434	0.6935	0.6797	0.6929	0.0591
YIF1A	0.4702	1.0879	0.6687	0.5448	0.6929	0.1379
DDEF2	0.6378	0.8838	0.6636	0.5866	0.6930	0.0656
UBQLN4	0.4908	1.0297	0.7393	0.5122	0.6930	0.1255
PTBP1	0.5778	0.8779	0.7169	0.6008	0.6933	0.0686
GTF3C2	0.6103	0.7832	0.7423	0.6394	0.6938	0.0411
ING3	0.5639	0.7645	0.7318	0.7157	0.6940	0.0445

PTGES3	0.5354	0.7374	1.0278	0.4758	0.6941	0.1245
TKT	0.5131	1.0304	0.7023	0.5310	0.6942	0.1199
GOLGA7	0.6908	0.7940	0.7362	0.5571	0.6945	0.0504
DERA	0.5601	0.8324	0.7905	0.5955	0.6946	0.0684
NUP62	0.5284	0.9281	0.7511	0.5713	0.6947	0.0915
C16orf59	0.5581	1.0155	0.6263	0.5792	0.6948	0.1079
LOC728873	0.5504	0.9925	0.6588	0.5781	0.6950	0.1018
LOC284023	0.6250	0.8485	0.6801	0.6265	0.6950	0.0527
LOC90624	0.5884	0.8652	0.8161	0.5109	0.6952	0.0861
C5orf33	0.6236	0.8086	0.7708	0.5778	0.6952	0.0559
PARP2	0.5597	0.8538	0.7856	0.5819	0.6952	0.0733
SDF2L1	0.5451	0.8428	0.6795	0.7139	0.6953	0.0612
KAT2A	0.4994	1.0564	0.6485	0.5786	0.6957	0.1240
PSMC3IP	0.5281	0.8639	0.6904	0.7019	0.6961	0.0686
PGAM5	0.5434	0.9382	0.6782	0.6249	0.6962	0.0853
LMBR1	0.5319	0.9123	0.7732	0.5675	0.6962	0.0895
CCDC115	0.6498	0.8710	0.6960	0.5684	0.6963	0.0639
GNL3L	0.4592	0.9088	0.8048	0.6125	0.6963	0.1001
TRAP1	0.4488	1.0806	0.7167	0.5401	0.6966	0.1396
FEZ2	0.5183	0.8898	0.7332	0.6450	0.6966	0.0781
DDX46	0.5154	0.9665	0.7003	0.6046	0.6967	0.0975
SDAD1	0.6158	0.8089	0.6522	0.7105	0.6969	0.0421
NUP35	0.4774	0.9112	0.7513	0.6481	0.6970	0.0910
NIPSNAP1	0.6173	0.8802	0.7206	0.5702	0.6970	0.0686
ZDHHC9	0.5218	0.9204	0.7139	0.6324	0.6971	0.0842
METTL3	0.5999	0.8996	0.6736	0.6161	0.6973	0.0693
MSH6	0.5313	0.9191	0.7701	0.5694	0.6975	0.0906
CLEC11A	0.6105	0.8524	0.6971	0.6300	0.6975	0.0548
APEX2	0.5152	0.9137	0.6576	0.7037	0.6975	0.0825
SLC11A2	0.5890	0.8640	0.6720	0.6664	0.6978	0.0585
DDX21	0.5481	0.8301	0.7746	0.6387	0.6979	0.0641
TLCD1	0.5216	0.8432	0.7609	0.6658	0.6979	0.0690
GLOD4	0.5582	0.7925	0.8422	0.5990	0.6980	0.0702
AGT	0.6598	0.7324	0.7579	0.6419	0.6980	0.0280
HSPD1	0.5286	0.8515	0.8356	0.5765	0.6981	0.0846
DUT	0.5359	0.8676	0.7331	0.6569	0.6984	0.0695
SCAMP3	0.5565	0.8578	0.7652	0.6147	0.6985	0.0689
LOC729708	0.5150	0.7529	0.9238	0.6025	0.6986	0.0897
LOC646407	0.5840	0.9886	0.6703	0.5513	0.6986	0.0999
CCNE1	0.4974	0.9251	0.7050	0.6670	0.6986	0.0880
HSPC159	0.5721	0.7051	0.7898	0.7285	0.6989	0.0459
C17orf96	0.5618	0.9231	0.5888	0.7219	0.6989	0.0825
SMG5	0.5750	0.8174	0.8291	0.5748	0.6991	0.0717
PPP1R14B	0.5531	0.9050	0.6907	0.6480	0.6992	0.0744
VDAC3	0.5647	0.8434	0.7721	0.6167	0.6992	0.0652
PRUNE2	0.5514	0.9865	0.5093	0.7498	0.6993	0.1092

C1orf135	0.5040	0.8944	0.7130	0.6856	0.6993	0.0799
C15orf42	0.5459	0.7212	0.7140	0.8159	0.6993	0.0561
UBR7	0.6738	0.7988	0.7172	0.6075	0.6993	0.0401
ERP29	0.6519	0.8946	0.6459	0.6054	0.6994	0.0659
POP7	0.6409	0.8652	0.6628	0.6291	0.6995	0.0557
THEM4	0.5520	0.8964	0.5964	0.7535	0.6996	0.0786
SKIV2L2	0.4686	0.8954	0.8497	0.5862	0.7000	0.1029
ORMDL1	0.6203	0.9122	0.7085	0.5591	0.7000	0.0771
PRDX4	0.5265	0.9035	0.8386	0.5319	0.7001	0.0996
DNAJC9	0.5313	0.9651	0.6769	0.6278	0.7003	0.0933
RANGAP1	0.4696	0.9757	0.7361	0.6202	0.7004	0.1068
C19orf40	0.5700	0.8919	0.6360	0.7047	0.7007	0.0694
CTPS	0.4924	0.9842	0.6860	0.6403	0.7007	0.1031
ATP5G1	0.4914	1.0103	0.7360	0.5655	0.7008	0.1152
SSU72	0.5324	0.8633	0.7812	0.6267	0.7009	0.0746
LPAR2	0.5512	0.8686	0.7493	0.6351	0.7011	0.0690
GARS	0.5630	0.9329	0.6981	0.6111	0.7013	0.0821
CSTF3	0.5671	0.8791	0.7253	0.6340	0.7014	0.0675
GLRX3	0.5298	0.9108	0.7424	0.6227	0.7014	0.0822
C8orf38	0.5672	0.8139	0.8421	0.5826	0.7014	0.0734
SDCCAG3	0.5095	0.9530	0.6914	0.6524	0.7016	0.0925
ACOT7	0.5617	0.8613	0.7523	0.6309	0.7016	0.0662
LOC731007	0.6182	0.8871	0.7394	0.5617	0.7016	0.0721
C9orf40	0.5777	0.7803	0.8562	0.5924	0.7017	0.0692
TUBA1C	0.5344	0.9313	0.7725	0.5697	0.7020	0.0927
SUMO2	0.6752	0.8436	0.6740	0.6160	0.7022	0.0491
CDC23	0.6061	0.8214	0.7646	0.6172	0.7023	0.0537
FRMD8	0.7145	0.7801	0.6280	0.6872	0.7024	0.0316
PHACTR4	0.6786	0.8411	0.6289	0.6612	0.7025	0.0473
ABCE1	0.5588	0.8406	0.8471	0.5643	0.7027	0.0815
CBS	0.6474	0.9304	0.6382	0.5952	0.7028	0.0767
HMGB1L1	0.5433	0.8258	0.7742	0.6686	0.7030	0.0625
LOC652489	0.6541	0.9249	0.5993	0.6346	0.7032	0.0748
LOC652864	0.5577	0.8512	0.7648	0.6417	0.7039	0.0649
MRPL18	0.5567	0.9298	0.6875	0.6415	0.7039	0.0800
SFRS2	0.5423	0.8995	0.7733	0.6006	0.7039	0.0816
HAT1	0.5292	0.7767	0.9210	0.5891	0.7040	0.0895
UCKL1	0.6258	0.9311	0.6403	0.6210	0.7045	0.0756
C1orf131	0.5416	0.8278	0.7838	0.6655	0.7046	0.0643
VPS4B	0.6152	1.0217	0.6398	0.5419	0.7047	0.1077
PDHA1	0.6103	0.8650	0.8782	0.4652	0.7047	0.1009
PSMD2	0.4842	1.0549	0.7459	0.5338	0.7047	0.1298
C18orf45	0.4530	1.0435	0.7183	0.6050	0.7050	0.1253
FAM20B	0.6197	0.9718	0.6621	0.5663	0.7050	0.0911
DDX41	0.6145	0.8702	0.7049	0.6304	0.7050	0.0585
DPM2	0.6149	0.8952	0.6893	0.6208	0.7050	0.0656

HIATL1	0.5614	0.9607	0.6318	0.6662	0.7050	0.0880
RBM3	0.5693	0.9706	0.7008	0.5800	0.7052	0.0934
CCNF	0.6161	0.9155	0.7091	0.5800	0.7052	0.0752
ALDH1B1	0.4941	0.8769	0.8360	0.6146	0.7054	0.0910
ACER3	0.5885	0.9210	0.7370	0.5754	0.7055	0.0806
NUP188	0.6058	0.9049	0.6960	0.6162	0.7057	0.0694
DFFB	0.5374	0.8858	0.7731	0.6272	0.7059	0.0772
TSEN54	0.5753	0.8259	0.7255	0.6974	0.7060	0.0516
STRA13	0.5022	0.9743	0.7691	0.5788	0.7061	0.1055
DUSP23	0.5451	0.9506	0.8258	0.5030	0.7061	0.1085
XPNPEP1	0.6212	0.8360	0.7334	0.6341	0.7062	0.0500
MRPS12	0.5472	0.8383	0.8097	0.6306	0.7064	0.0702
LOC653972	0.5544	0.9519	0.7403	0.5795	0.7065	0.0916
WSB2	0.5464	0.8466	0.8788	0.5550	0.7067	0.0903
FEM1A	0.5369	0.9682	0.7281	0.5961	0.7073	0.0957
CISD2	0.5542	0.8982	0.7807	0.5964	0.7074	0.0804
RFWD3	0.4645	0.9771	0.6875	0.7005	0.7074	0.1049
IPO5	0.5486	0.9709	0.6575	0.6529	0.7075	0.0913
NFE2L2	0.5128	0.8752	0.8342	0.6109	0.7083	0.0873
ATP6V0C	0.5770	0.9649	0.7130	0.5786	0.7084	0.0912
LOC1001279	0.6040	0.8365	0.7681	0.6252	0.7084	0.0561
INO80E	0.5901	0.9059	0.6745	0.6637	0.7085	0.0684
STIP1	0.5421	1.0073	0.7142	0.5712	0.7087	0.1064
LOC1001282	0.5057	0.9819	0.7485	0.5995	0.7089	0.1038
SLCO4A1	0.5149	0.9220	0.6719	0.7271	0.7090	0.0840
CABLES1	0.5892	0.9000	0.6850	0.6621	0.7091	0.0668
RTKN	0.5891	0.8995	0.7063	0.6420	0.7092	0.0678
RBM14	0.5956	0.9498	0.7346	0.5568	0.7092	0.0888
SUPV3L1	0.6414	0.8735	0.6680	0.6543	0.7093	0.0550
C12orf52	0.5148	0.8945	0.7345	0.6941	0.7095	0.0780
RQCD1	0.4860	0.8939	0.7616	0.6968	0.7096	0.0851
HMBS	0.5744	1.0509	0.6349	0.5785	0.7097	0.1146
CYB5B	0.5189	0.8513	0.8140	0.6549	0.7098	0.0766
LOC728556	0.4919	1.0739	0.7387	0.5356	0.7100	0.1327
FLJ39827	0.6132	0.8704	0.7311	0.6255	0.7100	0.0596
NUP62CL	0.6523	0.7801	0.7510	0.6573	0.7102	0.0325
CECR5	0.4967	1.0904	0.7488	0.5052	0.7103	0.1395
NUP155	0.5457	0.8272	0.8297	0.6386	0.7103	0.0708
SHPK	0.6631	0.9068	0.6732	0.5984	0.7104	0.0675
POLD2	0.5380	0.9486	0.7132	0.6426	0.7106	0.0871
GPR172A	0.6467	0.8906	0.6685	0.6366	0.7106	0.0604
RHEB	0.4996	1.0896	0.6828	0.5709	0.7107	0.1318
STAMBPL1	0.5935	0.8470	0.7819	0.6209	0.7108	0.0615
C7orf27	0.5324	0.9180	0.7698	0.6230	0.7108	0.0846
PBX3	0.6413	0.8168	0.7184	0.6670	0.7109	0.0388
C14orf173	0.6623	0.8460	0.6400	0.6953	0.7109	0.0464

SMNDC1	0.5228	0.8621	0.8591	0.6003	0.7111	0.0878
CBR3	0.6509	0.8536	0.6821	0.6580	0.7111	0.0480
PPIL1	0.5159	0.9605	0.7069	0.6618	0.7113	0.0925
RPF1	0.6081	0.8638	0.8769	0.4969	0.7114	0.0946
COPS7A	0.5947	0.9956	0.7128	0.5433	0.7116	0.1011
USE1	0.6418	0.9434	0.7078	0.5543	0.7118	0.0833
SEC22C	0.6329	0.9659	0.6687	0.5806	0.7120	0.0865
YWHAG	0.6048	0.7763	0.9928	0.4745	0.7121	0.1122
KIF21A	0.5708	0.8672	0.7524	0.6586	0.7122	0.0636
LCMT2	0.5055	1.0618	0.6818	0.6003	0.7124	0.1219
KIAA0672	0.5606	0.9101	0.7149	0.6646	0.7125	0.0733
NDUFB10	0.6142	0.8061	0.8523	0.5777	0.7126	0.0684
C15orf39	0.6221	0.9809	0.7180	0.5294	0.7126	0.0974
VKORC1	0.6016	0.8620	0.7060	0.6822	0.7130	0.0545
DLST	0.5839	0.9087	0.7603	0.5995	0.7131	0.0764
C19orf24	0.5174	1.0087	0.7856	0.5407	0.7131	0.1157
PFAS	0.5900	0.7978	0.8300	0.6348	0.7131	0.0592
FNTB	0.6724	0.8368	0.7160	0.6276	0.7132	0.0450
SFRS7	0.5900	0.8462	0.8344	0.5829	0.7134	0.0733
TMEM185B	0.6513	0.9211	0.6543	0.6275	0.7135	0.0694
LOC647150	0.5122	0.9116	0.8552	0.5751	0.7135	0.0996
GPC4	0.6445	0.8805	0.6814	0.6477	0.7135	0.0563
LOC644422	0.4825	0.8990	0.7688	0.7043	0.7136	0.0870
ZNF296	0.6018	0.8282	0.7802	0.6444	0.7137	0.0539
C2orf43	0.7151	0.8477	0.7221	0.5700	0.7137	0.0568
TIMM44	0.5127	0.9836	0.7016	0.6576	0.7139	0.0985
E2F3	0.5037	0.8559	0.8979	0.5981	0.7139	0.0964
DUSP2	0.6187	0.7602	0.7747	0.7022	0.7139	0.0354
LMAN1	0.4316	0.9928	0.8025	0.6288	0.7140	0.1199
LOC1001305	0.5331	0.9305	0.8289	0.5636	0.7140	0.0981
ROD1	0.5955	0.8798	0.7514	0.6300	0.7142	0.0645
CPOX	0.6720	0.8169	0.6983	0.6697	0.7142	0.0348
LOC729887	0.5931	0.9375	0.7489	0.5775	0.7142	0.0839
FH	0.6128	0.7870	0.8658	0.5914	0.7142	0.0669
METTL1	0.5707	0.8821	0.7610	0.6434	0.7143	0.0683
C13orf27	0.5871	0.8011	0.8758	0.5935	0.7144	0.0733
TMEM199	0.6937	0.8036	0.8095	0.5509	0.7144	0.0607
LOC729406	0.5617	0.9743	0.7594	0.5626	0.7145	0.0983
ARL1	0.5976	0.9001	0.7405	0.6205	0.7147	0.0693
POLR3G	0.5151	0.8929	0.7577	0.6935	0.7148	0.0785
LIAS	0.5983	0.8741	0.8125	0.5746	0.7149	0.0754
SSBP1	0.5613	0.7887	0.8695	0.6403	0.7149	0.0698
CPSF6	0.5830	0.8338	0.8012	0.6430	0.7152	0.0607
NPM3	0.5466	0.9653	0.7425	0.6067	0.7153	0.0929
CEP78	0.5462	0.8327	0.7876	0.6955	0.7155	0.0633
CCRN4L	0.5313	0.7850	0.8373	0.7086	0.7156	0.0668

FZD2	0.6526	0.9086	0.6201	0.6814	0.7157	0.0655
MAPK6	0.6892	0.7194	0.8038	0.6506	0.7157	0.0326
SPCS3	0.5717	0.8657	0.7994	0.6265	0.7158	0.0696
GRWD1	0.5281	0.9326	0.6931	0.7099	0.7159	0.0831
LOC729102	0.4875	1.0280	0.7247	0.6235	0.7159	0.1148
ERI3	0.6408	0.7579	0.8306	0.6351	0.7161	0.0475
TUBA1A	0.5867	0.8682	0.8177	0.5920	0.7162	0.0739
C7orf28B	0.5328	0.8705	0.8533	0.6086	0.7163	0.0856
TXNL2	0.5071	0.9167	0.7820	0.6596	0.7163	0.0873
MRPL35	0.5510	0.8745	0.8152	0.6250	0.7164	0.0766
LOC730107	0.5431	0.8603	0.8775	0.5854	0.7166	0.0884
HDHD1A	0.5224	0.9784	0.7662	0.5993	0.7166	0.1010
POLR1E	0.5026	0.9114	0.8621	0.5903	0.7166	0.1004
ZBTB9	0.5694	0.9786	0.6953	0.6237	0.7167	0.0910
CABC1	0.6306	0.9243	0.7147	0.5981	0.7169	0.0734
NDUFAB1	0.6050	0.7479	0.9332	0.5818	0.7170	0.0809
INCENP	0.5508	0.8681	0.7292	0.7203	0.7171	0.0649
UGT2B11	0.5173	1.0116	0.8267	0.5132	0.7172	0.1226
C1orf109	0.5547	0.9556	0.7057	0.6529	0.7172	0.0854
MRPS26	0.5883	0.9174	0.7641	0.5995	0.7173	0.0779
EMG1	0.5153	1.0166	0.7196	0.6183	0.7174	0.1081
MRPL37	0.5826	0.9966	0.7125	0.5798	0.7179	0.0979
FAM65A	0.6253	0.9378	0.6490	0.6597	0.7179	0.0736
WDR4	0.5351	0.8972	0.6748	0.7652	0.7181	0.0762
GLDC	0.6091	0.9610	0.6760	0.6270	0.7183	0.0821
NHP2	0.5345	0.9793	0.7712	0.5890	0.7185	0.1006
CLN3	0.5927	0.9150	0.6360	0.7307	0.7186	0.0715
C8orf55	0.5779	0.9032	0.6654	0.7280	0.7186	0.0688
IARS2	0.6753	0.9069	0.7163	0.5765	0.7187	0.0692
PPAT	0.4952	1.0317	0.6994	0.6489	0.7188	0.1130
EIF5A	0.6158	0.8822	0.7595	0.6178	0.7188	0.0640
GPATCH4	0.5866	0.9932	0.6947	0.6014	0.7190	0.0945
VDAC1	0.5618	0.9078	0.8014	0.6052	0.7190	0.0817
PRMT5	0.5668	0.9459	0.7716	0.5925	0.7192	0.0882
PRELID1	0.5427	1.0038	0.7663	0.5640	0.7192	0.1074
PSPH	0.7165	0.8798	0.6562	0.6258	0.7196	0.0566
SLC25A13	0.5408	0.8770	0.8247	0.6364	0.7198	0.0789
NHP2L1	0.4974	0.9877	0.7860	0.6081	0.7198	0.1073
LOC1001292	0.4686	0.9153	0.8602	0.6353	0.7198	0.1033
SRP72	0.5966	0.8904	0.8495	0.5429	0.7199	0.0878
PHF13	0.6550	0.9702	0.6500	0.6043	0.7199	0.0842
NUP160	0.5559	1.0232	0.7162	0.5843	0.7199	0.1070
EXT2	0.6845	0.8491	0.7109	0.6351	0.7199	0.0458
PSMD14	0.5873	0.8117	0.9599	0.5207	0.7199	0.1014
EME1	0.6076	0.8988	0.7324	0.6409	0.7199	0.0652
DNAJC11	0.6527	0.8214	0.6930	0.7129	0.7200	0.0360

DIAPH3	0.6204	0.8363	0.7408	0.6833	0.7202	0.0459
C14orf109	0.5732	0.9078	0.7335	0.6662	0.7202	0.0706
CD320	0.5640	0.9490	0.7790	0.5899	0.7205	0.0900
NUP153	0.6519	0.8469	0.7691	0.6149	0.7207	0.0534
SLC29A4	0.6325	0.8448	0.7545	0.6509	0.7207	0.0493
ZNF593	0.5447	1.0059	0.7062	0.6261	0.7208	0.1006
CKAP5	0.5659	1.0007	0.6976	0.6189	0.7208	0.0972
RC3H2	0.5675	0.9359	0.7678	0.6123	0.7209	0.0835
PTMA	0.5830	0.7336	0.9233	0.6440	0.7210	0.0742
LAS1L	0.5933	0.9388	0.6968	0.6558	0.7212	0.0756
SH2D4A	0.6168	0.8653	0.7273	0.6757	0.7213	0.0531
LOC1001281	0.5613	0.8861	0.7599	0.6779	0.7213	0.0684
RABEPK	0.5450	1.0208	0.7749	0.5454	0.7215	0.1135
FAM108C1	0.5610	0.8345	0.8186	0.6729	0.7218	0.0648
TMX1	0.6194	0.8854	0.7761	0.6064	0.7218	0.0668
VHL	0.5877	1.0123	0.6475	0.6399	0.7219	0.0977
SSB	0.5414	0.9044	0.8186	0.6234	0.7220	0.0841
ERCC6L	0.6007	0.7950	0.8059	0.6867	0.7221	0.0486
MRTO4	0.5964	0.7750	0.8598	0.6571	0.7221	0.0590
PGD	0.5286	1.0277	0.7001	0.6332	0.7224	0.1077
SLC29A1	0.5649	0.8374	0.7360	0.7514	0.7224	0.0571
LOC389599	0.6685	0.8241	0.8673	0.5301	0.7225	0.0770
SEC61A2	0.5583	0.8731	0.7315	0.7274	0.7226	0.0644
ARMC10	0.5754	0.9276	0.7584	0.6293	0.7227	0.0784
MTMR4	0.5817	0.9539	0.7750	0.5802	0.7227	0.0896
WDR18	0.4876	0.9991	0.7088	0.6953	0.7227	0.1051
DEK	0.6334	0.9842	0.7403	0.5332	0.7227	0.0969
LOC730246	0.5534	0.9297	0.7910	0.6172	0.7228	0.0853
MAT2A	0.5976	1.0191	0.6414	0.6333	0.7228	0.0992
LOC647340	0.5395	1.0180	0.7309	0.6030	0.7229	0.1061
NELF	0.5230	0.9745	0.8049	0.5897	0.7230	0.1032
Magmas	0.5798	0.7735	0.8476	0.6915	0.7231	0.0574
TUBA4A	0.6815	0.8652	0.7665	0.5793	0.7231	0.0609
ILF2	0.5574	0.9535	0.7344	0.6474	0.7232	0.0849
TMEM70	0.5407	0.8554	0.8073	0.6894	0.7232	0.0701
LRP11	0.5835	0.8277	0.8893	0.5928	0.7233	0.0791
CDR2L	0.6085	1.0224	0.7007	0.5623	0.7235	0.1037
C1orf52	0.6474	0.8900	0.7981	0.5585	0.7235	0.0743
ARD1A	0.5305	0.8925	0.9015	0.5699	0.7236	0.1004
RNASEH1	0.5225	1.0070	0.7286	0.6363	0.7236	0.1034
DLEU1	0.5244	0.9931	0.7976	0.5809	0.7240	0.1073
CIRH1A	0.4785	0.9300	0.7845	0.7035	0.7241	0.0943
DLL3	0.6343	0.8360	0.7335	0.6929	0.7242	0.0425
ATP6V0A2	0.7154	0.9243	0.6428	0.6143	0.7242	0.0700
SNORA61	0.5337	0.9718	0.6882	0.7032	0.7242	0.0910
DARS2	0.5911	0.9253	0.7898	0.5911	0.7243	0.0817

GPR180	0.6066	0.9383	0.7161	0.6366	0.7244	0.0749
FNBP4	0.5995	0.9132	0.6868	0.6983	0.7244	0.0667
DNASE2B	0.4954	0.9580	0.8167	0.6284	0.7246	0.1020
SLC25A28	0.5281	1.0106	0.7256	0.6345	0.7247	0.1035
CHCHD3	0.5783	0.9047	0.8258	0.5901	0.7247	0.0828
OSGEPL1	0.6293	0.7469	0.8367	0.6861	0.7248	0.0444
HNRPM	0.5588	1.0712	0.7097	0.5594	0.7248	0.1208
JTB	0.5985	1.0008	0.7367	0.5634	0.7248	0.0993
RBBP8	0.5355	0.8913	0.8662	0.6065	0.7249	0.0902
RUVBL2	0.5924	1.0081	0.7588	0.5406	0.7250	0.1052
ECSIT	0.6370	0.9062	0.7178	0.6394	0.7251	0.0632
C6orf192	0.6357	0.9612	0.7423	0.5612	0.7251	0.0870
UFSP1	0.5778	0.8713	0.7848	0.6668	0.7252	0.0646
PITX1	0.5994	0.8855	0.8004	0.6159	0.7253	0.0702
LOC644214	0.5397	0.8193	0.9853	0.5570	0.7253	0.1077
PATE3	0.5206	0.8596	0.8784	0.6434	0.7255	0.0866
CYP4V2	0.7465	0.8092	0.6881	0.6585	0.7256	0.0334
TROVE2	0.5703	0.9906	0.7507	0.5908	0.7256	0.0971
ICK	0.5713	0.8929	0.7693	0.6697	0.7258	0.0688
DDT	0.6028	0.9226	0.7732	0.6048	0.7258	0.0768
DNASE2	0.5395	0.9262	0.8072	0.6309	0.7260	0.0869
C1orf71	0.4928	1.0357	0.7779	0.5986	0.7263	0.1188
STARD3NL	0.5161	1.0124	0.7893	0.5875	0.7263	0.1115
TFB2M	0.5236	0.9136	0.8561	0.6120	0.7264	0.0940
EIF3J	0.5412	0.9335	0.7958	0.6352	0.7264	0.0868
C7orf30	0.5720	0.8897	0.7456	0.6986	0.7265	0.0656
FN3KRP	0.6710	0.9206	0.7217	0.5929	0.7266	0.0699
SNX5	0.5212	1.0792	0.6219	0.6841	0.7266	0.1222
HES4	0.6377	0.9853	0.6754	0.6082	0.7266	0.0873
LETM1	0.7330	0.8710	0.7038	0.5990	0.7267	0.0560
ZYX	0.5873	0.9692	0.7001	0.6506	0.7268	0.0840
SLC27A5	0.5121	1.0143	0.7633	0.6176	0.7268	0.1088
ZCCHC8	0.6633	0.9559	0.6455	0.6433	0.7270	0.0764
FAH	0.5502	0.9858	0.7746	0.5974	0.7270	0.0989
C2orf29	0.5896	0.9093	0.7784	0.6308	0.7270	0.0730
LOC1001345	0.4977	1.0142	0.8096	0.5868	0.7271	0.1160
CCDC51	0.5064	1.0808	0.7176	0.6041	0.7272	0.1255
TMEM138	0.5377	0.9570	0.7778	0.6371	0.7274	0.0910
FAHD1	0.5400	0.8770	0.8975	0.5953	0.7274	0.0930
CLASP2	0.6080	0.9685	0.6764	0.6569	0.7275	0.0816
WDHD1	0.6107	0.8106	0.7876	0.7010	0.7275	0.0455
LOC646791	0.5426	1.0453	0.7252	0.5972	0.7276	0.1126
C10orf2	0.5510	0.8806	0.7997	0.6797	0.7277	0.0719
RBBP9	0.5267	0.9509	0.8200	0.6133	0.7277	0.0965
TMEM177	0.5790	0.9917	0.7553	0.5853	0.7278	0.0970
GSTT2B	0.6426	0.9591	0.6442	0.6661	0.7280	0.0772

BUB1B	0.5999	0.7896	0.7414	0.7817	0.7282	0.0440
BARD1	0.5557	0.9386	0.6885	0.7300	0.7282	0.0794
TMEM144	0.4916	0.9084	0.9665	0.5466	0.7283	0.1219
GLMN	0.5604	0.9517	0.8539	0.5470	0.7283	0.1028
C4orf18	0.5513	0.9783	0.7937	0.5901	0.7284	0.0988
MRPS28	0.6530	0.8755	0.9255	0.4597	0.7285	0.1074
LOC728564	0.4879	0.8493	0.9239	0.6527	0.7285	0.0985
PWP1	0.5918	0.9726	0.7667	0.5828	0.7285	0.0917
ZNF598	0.6245	0.9859	0.7085	0.5953	0.7285	0.0891
RBBP7	0.5448	0.8114	1.0287	0.5292	0.7285	0.1192
DUS3L	0.5230	1.0158	0.6786	0.6970	0.7286	0.1034
VTA1	0.5193	1.0919	0.7098	0.5936	0.7287	0.1273
GUF1	0.5942	0.9958	0.6939	0.6313	0.7288	0.0914
OSGEP	0.5995	0.9291	0.7750	0.6117	0.7288	0.0778
GMPS	0.4186	1.1257	0.7462	0.6249	0.7288	0.1486
EFNA4	0.6034	0.9308	0.6815	0.7005	0.7290	0.0704
YDJC	0.6157	0.8365	0.8917	0.5726	0.7291	0.0792
CUEDC2	0.7110	0.8754	0.6739	0.6564	0.7292	0.0501
CLDN7	0.5717	0.9575	0.8181	0.5702	0.7294	0.0958
C19orf50	0.5915	0.8292	0.8878	0.6092	0.7294	0.0756
FARSA	0.6238	0.8225	0.7465	0.7256	0.7296	0.0410
MSL3L1	0.5630	0.9148	0.8223	0.6196	0.7299	0.0830
MYCBP2	0.6521	0.9039	0.7349	0.6303	0.7303	0.0621
MTDH	0.5864	0.8624	0.9929	0.4798	0.7304	0.1190
CBR4	0.6727	0.8791	0.6958	0.6742	0.7304	0.0498
SNRPD3	0.5652	0.8166	0.8031	0.7369	0.7305	0.0578
COQ9	0.5750	0.8709	0.7598	0.7164	0.7305	0.0612
SCO2	0.5337	0.9893	0.7932	0.6066	0.7307	0.1021
ELL3	0.5640	0.9832	0.7322	0.6437	0.7308	0.0909
STARD7	0.5706	0.9668	0.7865	0.5995	0.7309	0.0921
PTDSS1	0.4514	1.2532	0.6592	0.5608	0.7311	0.1791
SLC25A39	0.5692	1.0621	0.7157	0.5789	0.7315	0.1152
GART	0.6048	0.9326	0.7416	0.6477	0.7317	0.0728
RPL12P6	0.3941	0.8908	0.9901	0.6518	0.7317	0.1331
LARP1	0.6398	0.9224	0.6576	0.7070	0.7317	0.0651
SNAP23	0.5388	0.9265	0.7698	0.6920	0.7318	0.0807
SNRNP25	0.5953	1.0207	0.7570	0.5552	0.7320	0.1056
NPTN	0.6225	0.8254	0.7875	0.6928	0.7321	0.0459
RNU6-15	0.5284	1.0153	0.8587	0.5259	0.7321	0.1225
CDK4	0.4660	1.1609	0.7352	0.5664	0.7321	0.1533
TXNDC12	0.5843	0.9135	0.7364	0.6947	0.7322	0.0684
ORC5L	0.5869	0.9349	0.8062	0.6017	0.7324	0.0840
C9orf30	0.6743	0.7853	0.8628	0.6083	0.7327	0.0567
C14orf32	0.6031	0.8948	0.8444	0.5893	0.7329	0.0796
STOML2	0.5699	0.9265	0.7748	0.6608	0.7330	0.0769
CEBPG	0.7808	0.8133	0.7388	0.5993	0.7330	0.0471

HIST2H3C	0.4880	0.9200	0.7761	0.7487	0.7332	0.0900
COPZ1	0.5711	0.9756	0.7454	0.6406	0.7332	0.0884
H2AFV	0.5891	0.8438	0.7696	0.7307	0.7333	0.0535
LOC732360	0.5511	0.9925	0.7630	0.6265	0.7333	0.0969
SLC39A10	0.5182	0.9264	0.8872	0.6018	0.7334	0.1019
SCLY	0.6132	0.8469	0.6661	0.8077	0.7334	0.0558
PHCA	0.5210	0.8729	0.7674	0.7728	0.7335	0.0749
NKD2	0.5878	0.9634	0.7333	0.6504	0.7337	0.0822
CTDSPL2	0.5915	0.9608	0.7406	0.6425	0.7339	0.0817
ACBD6	0.5182	0.9999	0.8502	0.5676	0.7340	0.1149
RAD54B	0.6540	0.8576	0.7249	0.6999	0.7341	0.0437
COPS6	0.5271	1.1623	0.6789	0.5691	0.7343	0.1462
CRTC3	0.6540	0.9776	0.7039	0.6024	0.7345	0.0836
C10orf125	0.5530	0.9277	0.7912	0.6660	0.7345	0.0807
ANKLE2	0.5881	1.0185	0.6690	0.6623	0.7345	0.0964
LOC1001284	0.5001	0.7996	1.1232	0.5153	0.7345	0.1467
SURF6	0.5195	1.0133	0.7701	0.6354	0.7346	0.1061
C12orf49	0.5982	0.8929	0.7649	0.6825	0.7346	0.0628
AMMECR1	0.5936	0.8703	0.8242	0.6512	0.7348	0.0666
C14orf167	0.5567	0.9963	0.7789	0.6074	0.7348	0.0993
SLC38A2	0.6262	0.9186	0.9290	0.4658	0.7349	0.1139
C7orf36	0.6046	0.8685	0.8874	0.5798	0.7351	0.0827
APITD1	0.5571	0.8993	0.8194	0.6657	0.7354	0.0767
RSL1D1	0.6069	0.8823	0.8145	0.6381	0.7355	0.0670
ARL2	0.5610	0.9998	0.8066	0.5746	0.7355	0.1046
LOC651316	0.5732	0.9371	0.7372	0.6946	0.7355	0.0756
ADCK2	0.6068	0.8994	0.7985	0.6375	0.7355	0.0689
LOC442727	0.5412	0.8433	0.8249	0.7328	0.7355	0.0691
MRPL3	0.5155	1.0202	0.7649	0.6417	0.7356	0.1077
SNORD110	0.4555	0.9334	0.7921	0.7613	0.7356	0.1006
SDHD	0.5622	0.7749	1.0632	0.5423	0.7356	0.1212
TRMU	0.6781	0.9540	0.6689	0.6419	0.7357	0.0732
INSIG1	0.5859	0.9867	0.8152	0.5559	0.7359	0.1017
BTN3A3	0.6385	0.9179	0.6947	0.6926	0.7360	0.0620
GAMT	0.4929	1.0438	0.7493	0.6582	0.7360	0.1155
EXOSC5	0.5864	0.9034	0.8492	0.6056	0.7361	0.0818
SARS	0.7544	0.7680	0.8701	0.5523	0.7362	0.0665
ST3GAL1	0.5497	1.1164	0.7078	0.5714	0.7363	0.1314
ABCF1	0.5315	1.0825	0.7350	0.5965	0.7364	0.1229
ACPP	0.8772	0.8299	0.6817	0.5571	0.7365	0.0729
MTA2	0.4386	1.0263	0.8245	0.6568	0.7365	0.1248
UNG	0.4972	1.0560	0.7233	0.6703	0.7367	0.1169
SEC22A	0.7080	0.8631	0.7083	0.6686	0.7370	0.0430
DERL1	0.7006	0.8387	0.6998	0.7089	0.7370	0.0340
LEO1	0.6716	0.9759	0.7095	0.5913	0.7371	0.0833
ABCB10	0.6063	0.9534	0.7588	0.6301	0.7371	0.0795

C17orf89	0.5154	1.0858	0.7035	0.6447	0.7373	0.1226
HDAC2	0.5713	0.8052	0.9686	0.6044	0.7374	0.0928
GTPBP3	0.6505	0.9551	0.6970	0.6473	0.7375	0.0734
C14orf104	0.6022	0.8764	0.9236	0.5477	0.7375	0.0950
NFYC	0.6769	0.7965	0.7928	0.6837	0.7375	0.0330
TBC1D4	0.6227	0.8914	0.7962	0.6401	0.7376	0.0644
FXC1	0.6121	1.0180	0.7412	0.5791	0.7376	0.0998
OGG1	0.5999	0.9826	0.7278	0.6414	0.7379	0.0858
PSMB5	0.5657	1.1540	0.6486	0.5843	0.7382	0.1397
PAICS	0.5485	1.0001	0.7676	0.6366	0.7382	0.0982
CRTAP	0.5992	0.9825	0.7237	0.6475	0.7382	0.0854
LOC201725	0.6361	0.7843	0.8482	0.6853	0.7385	0.0478
WDR90	0.6600	0.9393	0.6918	0.6629	0.7385	0.0673
ACADSB	0.6647	0.9376	0.7067	0.6453	0.7386	0.0676
LIG1	0.6895	0.9037	0.6397	0.7214	0.7386	0.0576
TMEM126A	0.6360	0.8259	0.8130	0.6801	0.7387	0.0475
PPIAL4A	0.5729	0.8543	0.9329	0.5954	0.7389	0.0909
UBXN8	0.6148	0.8645	0.7990	0.6772	0.7389	0.0567
ZFPM1	0.6304	0.9132	0.7316	0.6812	0.7391	0.0616
PIK3CB	0.5236	0.8911	0.8109	0.7308	0.7391	0.0789
FAM122B	0.7001	0.8115	0.7826	0.6633	0.7393	0.0346
TXNDC15	0.5216	0.9212	0.9303	0.5843	0.7394	0.1084
RAN	0.6036	0.9241	0.8174	0.6126	0.7394	0.0789
TUSC2	0.5562	1.0233	0.7663	0.6122	0.7395	0.1045
TCFL5	0.5505	1.0297	0.6722	0.7060	0.7396	0.1023
MRPS15	0.6164	0.9251	0.8271	0.5900	0.7396	0.0815
ARHGEF2	0.6637	0.8804	0.7237	0.6910	0.7397	0.0485
YEATS2	0.6675	0.9017	0.7379	0.6519	0.7397	0.0571
SEL1L3	0.6640	0.9316	0.6825	0.6810	0.7398	0.0641
SLC30A5	0.6284	0.9323	0.7761	0.6229	0.7399	0.0733
PIGO	0.6408	0.9648	0.7130	0.6414	0.7400	0.0768
THOC1	0.6217	0.8259	0.8375	0.6759	0.7402	0.0540
SMPDL3B	0.6140	0.9436	0.7309	0.6727	0.7403	0.0718
LIN54	0.6114	0.7077	0.8372	0.8048	0.7403	0.0510
LOC647302	0.5863	0.9662	0.7974	0.6118	0.7404	0.0887
LOC653994	0.4306	1.2515	0.6534	0.6266	0.7405	0.1774
APRT	0.4735	1.0540	0.8024	0.6339	0.7409	0.1241
SPAG5	0.5748	0.8808	0.7400	0.7692	0.7412	0.0632
SF3A2	0.5524	0.8939	0.8593	0.6595	0.7413	0.0814
MFF	0.5941	0.8654	0.8187	0.6870	0.7413	0.0619
GALK2	0.6136	1.0470	0.7102	0.5945	0.7413	0.1050
LOC145837	0.6030	1.0659	0.6637	0.6333	0.7415	0.1088
MYNN	0.6573	0.8351	0.8060	0.6677	0.7415	0.0461
NAE1	0.5957	1.0700	0.7149	0.5858	0.7416	0.1133
HIST1H3H	0.4738	1.0515	0.7533	0.6885	0.7418	0.1193
LOC647859	0.6357	0.8563	0.7545	0.7220	0.7421	0.0456

H3F3B	0.5979	0.8771	0.7967	0.6971	0.7422	0.0606
C16orf13	0.5052	1.0101	0.8085	0.6455	0.7423	0.1086
YRDC	0.5678	0.9677	0.7773	0.6566	0.7424	0.0865
DENR	0.6227	0.9208	0.8354	0.5914	0.7426	0.0804
TUBB	0.6197	1.0452	0.7226	0.5833	0.7427	0.1051
STRBP	0.7180	0.8620	0.7245	0.6676	0.7430	0.0417
NXF1	0.6001	1.0265	0.6546	0.6910	0.7430	0.0963
MRPL42	0.5364	0.9618	0.8884	0.5857	0.7431	0.1066
EIF4EBP2	0.6518	0.9765	0.6464	0.6980	0.7432	0.0786
RAB22A	0.6543	0.9565	0.7637	0.5984	0.7432	0.0790
MKRN1	0.6787	0.9439	0.7292	0.6214	0.7433	0.0704
MAK16	0.5947	0.8201	1.0166	0.5417	0.7433	0.1093
SFRS1	0.6374	1.0079	0.7933	0.5347	0.7433	0.1030
NUP88	0.5576	0.8638	0.9088	0.6437	0.7435	0.0848
FLJ22184	0.7095	0.8418	0.7101	0.7127	0.7435	0.0328
VASH2	0.6573	0.8827	0.7265	0.7079	0.7436	0.0486
CCNO	0.6729	0.9439	0.6782	0.6802	0.7438	0.0667
URM1	0.6282	0.9278	0.8198	0.5994	0.7438	0.0784
PPIL3	0.6223	0.9648	0.7869	0.6015	0.7439	0.0845
CA12	0.5481	0.9343	0.7581	0.7351	0.7439	0.0790
VARS2	0.6188	0.9394	0.7457	0.6718	0.7439	0.0702
LOC728698	0.4825	1.0397	0.8370	0.6174	0.7441	0.1226
BAG3	0.6193	0.8900	0.8181	0.6496	0.7442	0.0654
TRIT1	0.6163	0.8569	0.9626	0.5413	0.7443	0.0991
LOC728825	0.6077	0.9423	0.7105	0.7169	0.7444	0.0706
ATRIP	0.5975	0.9605	0.7326	0.6872	0.7445	0.0773
RNU6-1	0.5165	0.8822	0.8733	0.7060	0.7445	0.0861
MOBKL3	0.6538	0.9213	0.8049	0.5983	0.7446	0.0733
NGDN	0.5973	0.9321	0.7896	0.6600	0.7447	0.0742
NDUFA9	0.5638	1.0284	0.7724	0.6143	0.7447	0.1045
PHKA1	0.7209	0.8865	0.7335	0.6381	0.7447	0.0518
HMGB3	0.7053	0.9377	0.6654	0.6707	0.7448	0.0649
KLHDC4	0.6247	0.8702	0.8450	0.6396	0.7449	0.0654
LOC653820	0.6334	0.8231	0.7859	0.7372	0.7449	0.0411
C9orf100	0.6485	0.8428	0.7598	0.7292	0.7451	0.0402
TNPO2	0.5373	1.0219	0.8113	0.6105	0.7452	0.1089
METTL5	0.5774	0.8959	0.8620	0.6459	0.7453	0.0787
PAK1IP1	0.5824	1.0340	0.7610	0.6039	0.7453	0.1041
EML1	0.6285	0.9575	0.6744	0.7211	0.7454	0.0732
SNRPG	0.6060	0.8600	0.8956	0.6203	0.7455	0.0768
SET	0.5995	0.9658	0.8283	0.5887	0.7456	0.0919
C1orf41	0.6364	0.9257	0.6945	0.7258	0.7456	0.0628
FAM45A	0.6321	0.8201	0.9402	0.5900	0.7456	0.0819
RPL28	0.6873	0.9801	0.5862	0.7288	0.7456	0.0837
C7orf28A	0.6177	0.9536	0.7483	0.6646	0.7460	0.0743
HCFC1R1	0.5714	1.0533	0.6798	0.6797	0.7461	0.1056

PPP1R16A	0.6013	1.0726	0.7032	0.6076	0.7462	0.1113
MYH10	0.7225	0.9668	0.6722	0.6234	0.7462	0.0762
STX10	0.5748	1.0622	0.6332	0.7147	0.7463	0.1092
CALM2	0.5518	0.9156	0.8586	0.6593	0.7463	0.0850
FASTKD2	0.5924	1.0320	0.7313	0.6296	0.7463	0.0996
TIGD5	0.6509	0.9945	0.7408	0.5993	0.7464	0.0877
FAM117B	0.6573	0.8705	0.7478	0.7108	0.7466	0.0453
RBM25	0.5466	1.0733	0.8191	0.5484	0.7468	0.1262
LOC643856	0.5540	1.0419	0.7499	0.6415	0.7469	0.1062
PRMT1	0.5398	1.0761	0.7626	0.6089	0.7469	0.1192
ACTL6A	0.5632	1.0332	0.8100	0.5811	0.7469	0.1107
MIR1228	0.6041	1.0214	0.7824	0.5799	0.7469	0.1020
PROCR	0.6970	0.9162	0.6438	0.7308	0.7469	0.0592
CDC42SE2	0.6055	0.8764	0.8768	0.6291	0.7470	0.0750
BYSL	0.5138	1.0745	0.7680	0.6315	0.7470	0.1209
EXOC5	0.6062	0.9437	0.7848	0.6533	0.7470	0.0757
HSD17B6	0.6435	0.8426	0.7621	0.7398	0.7470	0.0410
AVPI1	0.5208	1.0474	0.8173	0.6025	0.7470	0.1180
C20orf29	0.6203	0.9274	0.8157	0.6252	0.7472	0.0754
SLC10A3	0.6289	0.9534	0.7326	0.6742	0.7473	0.0719
TMEM118	0.6024	0.9439	0.7452	0.6977	0.7473	0.0719
FAM54A	0.6405	0.8257	0.7863	0.7369	0.7473	0.0400
TOPBP1	0.6608	0.9824	0.6844	0.6618	0.7474	0.0785
NEK7	0.6669	0.8576	0.7693	0.6965	0.7476	0.0425
EHF	0.4350	1.1360	0.7775	0.6421	0.7477	0.1474
SKP2	0.5210	1.0607	0.8135	0.5955	0.7477	0.1214
KLHL23	0.6699	0.8942	0.6913	0.7356	0.7477	0.0507
AGGF1	0.6761	0.9346	0.7357	0.6450	0.7479	0.0650
RNF121	0.6231	0.9534	0.7672	0.6483	0.7480	0.0753
SLC39A4	0.6879	0.9162	0.7365	0.6517	0.7481	0.0587
VAPA	0.5480	0.8536	0.8142	0.7770	0.7482	0.0685
AGTRAP	0.6480	0.9395	0.7912	0.6140	0.7482	0.0744
FYCO1	0.5477	1.1567	0.6541	0.6349	0.7484	0.1381
SMCR7L	0.7023	0.9847	0.7783	0.5281	0.7484	0.0946
SRD5A3	0.6429	0.8273	0.8290	0.6944	0.7484	0.0472
LOC653884	0.5661	1.0143	0.7508	0.6627	0.7484	0.0963
ADORA2B	0.6740	0.9148	0.6968	0.7083	0.7485	0.0559
PSMC6	0.6179	1.0008	0.7987	0.5765	0.7485	0.0970
KCTD15	0.6349	0.9289	0.6990	0.7313	0.7485	0.0634
ASAP2	0.6791	0.8999	0.6990	0.7165	0.7486	0.0510
LOC642817	0.4054	1.2096	0.8203	0.5591	0.7486	0.1759
CORO1C	0.5632	0.9427	0.8082	0.6806	0.7487	0.0818
PTGES2	0.5804	1.0830	0.7130	0.6187	0.7488	0.1148
SFRS18	0.6349	0.8837	0.7661	0.7104	0.7488	0.0524
CCT7	0.5856	0.9721	0.8014	0.6361	0.7488	0.0875
BCLAF1	0.5730	1.0429	0.7423	0.6373	0.7489	0.1040

WDR13	0.6155	1.0239	0.7548	0.6013	0.7489	0.0980
ANKRD57	0.6109	0.9526	0.7266	0.7055	0.7489	0.0724
SESN1	0.6766	0.9836	0.7122	0.6236	0.7490	0.0803
C3orf39	0.6693	0.9126	0.7695	0.6453	0.7492	0.0608
C6orf136	0.6211	0.9657	0.8065	0.6037	0.7492	0.0855
LOC729423	0.6187	1.0174	0.6762	0.6851	0.7493	0.0906
DDX49	0.7552	0.9167	0.6646	0.6617	0.7496	0.0598
TMEM14A	0.5283	1.0638	0.7726	0.6337	0.7496	0.1161
FLJ20674	0.4847	0.9122	0.8292	0.7727	0.7497	0.0929
CHCHD1	0.6392	0.8892	0.9166	0.5544	0.7498	0.0902
SDHAF2	0.6821	0.9482	0.7238	0.6459	0.7500	0.0680
SYPL1	0.6371	0.9937	0.7837	0.5856	0.7501	0.0914
SRFBP1	0.4951	0.9518	0.8676	0.6858	0.7501	0.1015
JAG2	0.6234	0.9484	0.7251	0.7035	0.7501	0.0696
PABPN1	0.6078	1.0081	0.7599	0.6250	0.7502	0.0925
KIAA0514	0.6988	0.8879	0.6907	0.7234	0.7502	0.0464
CLEC2D	0.5311	0.9093	0.9547	0.6058	0.7502	0.1065
LOC646849	0.5256	0.9589	0.9088	0.6080	0.7503	0.1078
YWHAQ	0.6071	0.9970	0.8054	0.5919	0.7504	0.0955
C7orf68	0.5175	0.9274	0.7973	0.7593	0.7504	0.0856
MAPKAPK5	0.6986	0.8988	0.7708	0.6336	0.7505	0.0568
NME1	0.5809	0.9349	0.8270	0.6599	0.7507	0.0800
C18orf8	0.5793	0.9273	0.8100	0.6864	0.7508	0.0754
BAZ1A	0.5781	0.9777	0.7765	0.6710	0.7508	0.0858
FLJ38482	0.4862	0.8774	0.9483	0.6915	0.7509	0.1035
ARV1	0.6630	0.8836	0.8286	0.6283	0.7509	0.0622
ADRM1	0.6583	0.9197	0.7986	0.6275	0.7510	0.0674
CNOT7	0.5985	0.9173	0.8335	0.6553	0.7512	0.0747
MRPS5	0.6929	0.9977	0.7459	0.5684	0.7512	0.0902
SH2B3	0.8375	0.8533	0.7412	0.5730	0.7513	0.0644
NQO2	0.5474	1.0918	0.7981	0.5688	0.7515	0.1268
TOX3	0.6321	1.0243	0.7236	0.6271	0.7518	0.0935
C16orf91	0.7010	0.9046	0.7861	0.6162	0.7520	0.0616
TMEM192	0.6523	0.8736	0.8179	0.6642	0.7520	0.0554
SLC39A11	0.7149	1.0156	0.6710	0.6068	0.7521	0.0906
UBQLN1	0.6244	1.0043	0.7660	0.6137	0.7521	0.0909
MARS2	0.6418	0.9720	0.7809	0.6142	0.7522	0.0819
CHCHD7	0.5548	0.9865	0.7776	0.6901	0.7523	0.0905
NOB1	0.5060	1.0916	0.7889	0.6226	0.7523	0.1271
PCBP1	0.5630	1.0180	0.7710	0.6578	0.7524	0.0982
CPSF2	0.5563	0.9834	0.7585	0.7120	0.7526	0.0883
MAD2L2	0.6205	0.8847	0.7543	0.7516	0.7528	0.0539
LOC643438	0.6641	0.9108	0.8384	0.5979	0.7528	0.0731
KIF2A	0.6224	0.9319	0.7987	0.6583	0.7529	0.0708
NUP205	0.6128	0.9276	0.7549	0.7164	0.7529	0.0655
LOC1001315	0.7180	0.9335	0.7933	0.5670	0.7529	0.0764

PSMC4	0.6138	1.0772	0.7190	0.6018	0.7530	0.1112
MRPS30	0.4871	1.0320	0.8401	0.6533	0.7531	0.1177
GLA	0.5201	0.9972	0.8329	0.6629	0.7533	0.1034
LOC652615	0.6556	0.9406	0.6783	0.7389	0.7533	0.0648
NTAN1	0.6950	0.7473	0.8821	0.6890	0.7534	0.0449
KIF15	0.6594	0.9157	0.6888	0.7496	0.7534	0.0573
STXBP5L	0.6996	0.9231	0.6912	0.6998	0.7534	0.0566
ACD	0.6954	0.9787	0.6866	0.6537	0.7536	0.0756
P15RS	0.7271	0.9963	0.7412	0.5498	0.7536	0.0919
TACSTD1	0.5379	0.9250	0.8863	0.6653	0.7536	0.0919
RNF38	0.5897	0.9709	0.7415	0.7125	0.7537	0.0795
SARS2	0.5537	0.9619	0.8193	0.6801	0.7538	0.0881
DPH5	0.6797	0.9407	0.7551	0.6399	0.7538	0.0667
GAGE7	0.5806	0.7257	1.1088	0.6004	0.7539	0.1226
LTBP3	0.5139	1.0016	0.8117	0.6883	0.7539	0.1027
UBE2J1	0.6535	0.9202	0.7546	0.6873	0.7539	0.0593
GEMIN5	0.6731	1.0311	0.7034	0.6081	0.7539	0.0945
TRABD	0.6544	1.0735	0.6989	0.5889	0.7539	0.1089
TNFAIP8L1	0.6328	0.8382	0.8512	0.6937	0.7540	0.0539
ALS2CR4	0.5044	0.9878	0.7992	0.7251	0.7541	0.0999
GTF2E1	0.5370	1.0626	0.7064	0.7106	0.7541	0.1105
WDR53	0.5404	0.9375	0.8256	0.7130	0.7541	0.0847
GAR1	0.5542	1.0137	0.8132	0.6360	0.7543	0.1020
NSMAF	0.5390	0.9821	0.7704	0.7257	0.7543	0.0910
NCBP1	0.7093	0.9401	0.6894	0.6786	0.7543	0.0622
C19orf54	0.5948	1.0325	0.7079	0.6825	0.7544	0.0958
C5orf24	0.6152	0.8515	0.8106	0.7407	0.7545	0.0518
CDV3	0.6292	0.9736	0.7977	0.6185	0.7547	0.0837
FKBP4	0.5305	1.0181	0.8326	0.6379	0.7548	0.1078
DUS4L	0.6076	0.8499	0.8422	0.7202	0.7550	0.0574
LARS	0.6724	1.0303	0.7134	0.6038	0.7550	0.0945
ZNF614	0.5983	0.9872	0.7776	0.6570	0.7550	0.0859
HSD17B10	0.5232	0.9723	0.8263	0.6986	0.7551	0.0954
BRMS1	0.6412	0.9888	0.7684	0.6220	0.7551	0.0844
RNMTL1	0.5275	0.9556	0.8561	0.6815	0.7552	0.0947
ESPL1	0.7537	0.8562	0.7429	0.6680	0.7552	0.0387
TMEM109	0.6507	0.9840	0.7407	0.6455	0.7552	0.0793
UGDH	0.7601	0.9240	0.8188	0.5183	0.7553	0.0860
RUVBL1	0.6224	0.9412	0.8279	0.6303	0.7555	0.0781
NDUFAF2	0.5670	1.0056	0.8513	0.5982	0.7555	0.1049
ADCY9	0.6652	0.9500	0.7186	0.6886	0.7556	0.0657
GCLM	0.5910	1.0608	0.7528	0.6177	0.7556	0.1077
AATF	0.6090	1.0280	0.7592	0.6264	0.7557	0.0968
CDR2	0.5705	1.0308	0.8007	0.6211	0.7558	0.1041
C1orf69	0.7174	0.9051	0.7217	0.6789	0.7558	0.0507
LOC1001280	0.5830	0.9322	0.7897	0.7182	0.7558	0.0728

C10orf78	0.5749	0.9696	0.7285	0.7501	0.7558	0.0812
GPN3	0.5559	1.1129	0.7645	0.5899	0.7558	0.1275
LOC646044	0.5982	0.9551	0.7318	0.7384	0.7559	0.0738
SLC35A2	0.5908	0.9462	0.7906	0.6960	0.7559	0.0754
ESRRAP2	0.6082	0.9867	0.7474	0.6813	0.7559	0.0820
RIMKLB	0.6344	0.8856	0.8184	0.6853	0.7559	0.0581
SNORA70	0.6140	0.9992	0.7237	0.6870	0.7559	0.0842
PPM1K	0.7043	0.9029	0.7923	0.6244	0.7560	0.0598
FLJ12949	0.6587	0.8785	0.8087	0.6783	0.7561	0.0527
EBPL	0.5954	1.0520	0.8545	0.5233	0.7563	0.1216
NOLA1	0.5553	0.9303	0.8183	0.7223	0.7566	0.0794
AASDHPPT	0.6287	0.9136	0.8579	0.6263	0.7566	0.0754
TBCA	0.5306	1.0159	0.8656	0.6149	0.7568	0.1119
ZNHIT1	0.6213	1.0820	0.7496	0.5747	0.7569	0.1145
PCGF6	0.6213	0.8819	0.7902	0.7346	0.7570	0.0545
LOC1001324	0.5894	0.8159	1.0095	0.6133	0.7570	0.0983
LIMS1	0.6202	0.9805	0.8306	0.5968	0.7570	0.0912
NOL11	0.6232	1.0056	0.8183	0.5814	0.7572	0.0976
SRI	0.6601	0.9268	0.7516	0.6907	0.7573	0.0596
ZFP36L2	0.6096	0.9057	0.7886	0.7253	0.7573	0.0618
VPS37D	0.6987	1.0242	0.6606	0.6460	0.7574	0.0896
C4orf27	0.7317	0.8907	0.8197	0.5876	0.7574	0.0653
UTP14A	0.5930	1.0437	0.7681	0.6251	0.7575	0.1027
UBAP2L	0.5850	1.0318	0.7243	0.6888	0.7575	0.0961
BAG5	0.6670	0.9585	0.7725	0.6328	0.7577	0.0732
C9orf21	0.5521	0.9154	0.9058	0.6578	0.7578	0.0908
APEX1	0.5802	1.0125	0.8178	0.6206	0.7578	0.0995
ANKRD46	0.5806	1.0456	0.8450	0.5604	0.7579	0.1158
LTV1	0.5647	0.9389	0.8377	0.6903	0.7579	0.0822
ISG20	0.7117	1.1463	0.5808	0.5930	0.7580	0.1328
BEX4	0.6395	0.9277	0.8660	0.5994	0.7581	0.0815
RAC3	0.6347	1.0576	0.7261	0.6142	0.7581	0.1027
SNORD36A	0.6493	0.8851	0.8417	0.6565	0.7582	0.0614
TTYH2	0.6934	0.9356	0.6813	0.7226	0.7582	0.0598
ZNF511	0.6245	1.0385	0.7651	0.6049	0.7582	0.1000
CCDC77	0.6218	0.8989	0.8745	0.6383	0.7584	0.0743
MRPL55	0.6573	0.9589	0.7953	0.6224	0.7585	0.0765
PDE3B	0.5402	0.9245	0.8194	0.7500	0.7585	0.0811
C17orf80	0.5764	0.9710	0.7258	0.7610	0.7586	0.0813
PARVB	0.5090	0.9757	0.9076	0.6424	0.7587	0.1100
PFDN6	0.6323	1.0224	0.7428	0.6378	0.7588	0.0915
CCT6P1	0.6247	0.8830	0.9561	0.5719	0.7589	0.0946
DBNDD2	0.5835	0.9883	0.7262	0.7378	0.7589	0.0841
LOC92755	0.5081	1.2170	0.7214	0.5892	0.7589	0.1589
C11orf48	0.6134	0.9300	0.7870	0.7055	0.7590	0.0671
NUP50	0.6064	0.8731	0.8049	0.7520	0.7591	0.0566

RASSF7	0.6139	0.9945	0.7584	0.6697	0.7591	0.0839
TMEM45A	0.7440	0.9056	0.6356	0.7513	0.7591	0.0555
MIPEP	0.6970	1.0552	0.6216	0.6630	0.7592	0.0999
LOC729123	0.6738	0.8215	0.8994	0.6422	0.7592	0.0609
RPP21	0.6504	0.9106	0.8986	0.5776	0.7593	0.0852
GNL2	0.5492	0.8886	0.9736	0.6257	0.7593	0.1019
GSTT2	0.6984	0.9891	0.6983	0.6514	0.7593	0.0774
NUP37	0.5214	1.1409	0.7772	0.5977	0.7593	0.1380
FKBP2	0.7276	0.9806	0.7232	0.6061	0.7594	0.0789
KHSRP	0.5295	0.9937	0.8423	0.6724	0.7595	0.1009
BRMS1L	0.7164	0.9822	0.7127	0.6267	0.7595	0.0771
MGC57346	0.6992	0.9328	0.7779	0.6282	0.7595	0.0653
ENOPH1	0.6343	1.0401	0.8399	0.5240	0.7596	0.1141
SMARCAD1	0.6620	0.9268	0.7512	0.6984	0.7596	0.0587
ADAM7	0.5731	0.8883	0.8018	0.7752	0.7596	0.0667
HRAS	0.6267	0.9127	0.8652	0.6339	0.7596	0.0753
DNAJC15	0.7139	0.9056	0.7211	0.6982	0.7597	0.0489
UGT8	0.6004	0.9361	0.7743	0.7281	0.7597	0.0693
PAXIP1	0.5651	1.1171	0.7162	0.6417	0.7600	0.1230
LOC1001279	0.7581	0.9327	0.7289	0.6208	0.7601	0.0647
LOC402509	0.8140	0.8297	0.7562	0.6410	0.7602	0.0428
C6orf64	0.6484	1.2118	0.6674	0.5136	0.7603	0.1544
KPNA3	0.6052	0.8895	0.8481	0.6986	0.7604	0.0660
TXNL1	0.6144	0.9109	0.8149	0.7016	0.7604	0.0648
CYB5D1	0.6180	1.0099	0.8128	0.6013	0.7605	0.0960
BOP1	0.5425	1.0240	0.8644	0.6111	0.7605	0.1118
LOC729148	0.6265	0.9910	0.7954	0.6293	0.7606	0.0864
SPC25	0.6290	0.8013	0.9163	0.6967	0.7608	0.0628
LSM3	0.5913	1.0375	0.7961	0.6184	0.7608	0.1028
HLTF	0.5735	1.1149	0.7417	0.6140	0.7610	0.1233
TFDP1	0.6026	0.9848	0.7823	0.6747	0.7611	0.0832
MORF4L1	0.6318	0.9443	0.8913	0.5776	0.7612	0.0917
TMEM55B	0.7878	0.9107	0.6120	0.7348	0.7613	0.0619
PRNPIP	0.7346	0.8363	0.8352	0.6399	0.7615	0.0470
MTR	0.6531	0.8388	0.9234	0.6309	0.7615	0.0713
HSDL2	0.6910	0.8946	0.8302	0.6304	0.7616	0.0610
E2F4	0.6190	0.9530	0.8325	0.6420	0.7616	0.0797
FOXRED1	0.6063	1.0115	0.7388	0.6901	0.7617	0.0877
ITPR3	0.7112	1.0680	0.6428	0.6246	0.7617	0.1038
PDCD5	0.5952	1.0658	0.8330	0.5532	0.7618	0.1186
PDIA4	0.6741	0.8883	0.7691	0.7157	0.7618	0.0464
ARHGEF19	0.7078	1.0668	0.6172	0.6556	0.7619	0.1033
AGPAT5	0.4822	1.0362	0.7926	0.7367	0.7619	0.1137
LOC401588	0.6512	0.8751	0.7240	0.7974	0.7619	0.0481
CCM2	0.6400	1.0173	0.7280	0.6627	0.7620	0.0871
FAIM	0.6091	0.9799	0.8355	0.6237	0.7620	0.0892

BLOC1S1	0.8309	0.8680	0.9525	0.3970	0.7621	0.1243
NRSN2	0.6283	0.9244	0.7691	0.7267	0.7621	0.0616
C12orf66	0.5373	0.9807	0.8438	0.6880	0.7624	0.0959
TMED10	0.6293	1.0387	0.7436	0.6383	0.7625	0.0957
MPZL2	0.7065	0.9477	0.7510	0.6452	0.7626	0.0654
PPP1R10	0.7456	0.9982	0.7153	0.5913	0.7626	0.0853
APOA1	0.7469	0.8643	0.7542	0.6851	0.7626	0.0373
PQLC3	0.6462	0.9343	0.8169	0.6532	0.7626	0.0695
ARMC1	0.6896	0.9082	0.8029	0.6501	0.7627	0.0583
WDR36	0.5857	1.0468	0.7823	0.6363	0.7628	0.1034
LOC642897	0.5419	0.9782	0.8561	0.6753	0.7629	0.0964
POLR1C	0.5804	1.0724	0.7599	0.6390	0.7629	0.1097
FOXN2	0.6136	0.8887	0.8788	0.6708	0.7630	0.0707
LOC1001295	0.6081	0.8537	0.8743	0.7160	0.7630	0.0625
CMC1	0.6700	0.9841	0.7348	0.6634	0.7631	0.0754
C12orf4	0.5624	1.0309	0.8072	0.6521	0.7632	0.1026
FZD9	0.6220	0.9366	0.7993	0.6948	0.7632	0.0683
CREB3L2	0.7299	0.9943	0.6813	0.6474	0.7632	0.0789
ATP5D	0.6239	0.9940	0.8512	0.5838	0.7632	0.0969
LOC647081	0.5364	0.9877	0.9571	0.5724	0.7634	0.1210
PEMT	0.6702	1.0111	0.7575	0.6149	0.7634	0.0876
SRPK2	0.5705	1.0836	0.7684	0.6313	0.7634	0.1145
AHCTF1	0.5941	1.0025	0.8233	0.6339	0.7635	0.0941
TSEN2	0.6610	0.9599	0.7606	0.6724	0.7635	0.0692
DCTPP1	0.5631	0.9768	0.8669	0.6473	0.7635	0.0957
PDPR	0.6686	0.9746	0.7128	0.6981	0.7635	0.0710
PSMG4	0.5867	1.1856	0.6805	0.6016	0.7636	0.1422
RAGE	0.6360	0.9829	0.6688	0.7669	0.7636	0.0782
BNIP3L	0.8629	0.8718	0.7327	0.5871	0.7636	0.0669
MRPL20	0.6154	1.0005	0.7640	0.6747	0.7637	0.0846
LOC647000	0.5755	1.0394	0.8019	0.6383	0.7637	0.1035
MRPL36	0.6391	0.9468	0.8315	0.6379	0.7639	0.0761
PRIM2A	0.5885	0.9790	0.7581	0.7304	0.7640	0.0807
DNAJC27	0.6507	0.9939	0.7115	0.7000	0.7640	0.0777
SLC36A4	0.7031	0.7824	0.8674	0.7033	0.7641	0.0392
LOC653310	0.6554	0.9409	0.7956	0.6645	0.7641	0.0671
RNF220	0.6240	1.0466	0.7682	0.6178	0.7642	0.1004
TSKU	0.7063	0.9602	0.6958	0.6947	0.7643	0.0654
POLG2	0.5912	0.9392	0.8916	0.6351	0.7643	0.0882
C18orf54	0.6178	1.0019	0.6948	0.7430	0.7644	0.0833
RAB21	0.7492	0.9661	0.7847	0.5577	0.7644	0.0837
C16orf53	0.5212	1.0766	0.8369	0.6231	0.7645	0.1231
HSD17B1	0.6609	0.8879	0.8174	0.6917	0.7645	0.0533
MRPL46	0.5690	1.0253	0.8147	0.6490	0.7645	0.1009
TMEM123	0.5688	1.0713	0.7763	0.6419	0.7646	0.1109
TEAD4	0.6206	0.9147	0.8231	0.7004	0.7647	0.0651

TMEM126B	0.5298	1.0335	0.8760	0.6201	0.7648	0.1157
MTIF2	0.5977	0.9861	0.8520	0.6240	0.7649	0.0932
CCDC45	0.6355	0.8492	0.8094	0.7658	0.7650	0.0464
OXTR	0.6664	0.8608	0.8397	0.6944	0.7653	0.0496
KIAA1333	0.7183	0.8993	0.7746	0.6695	0.7654	0.0495
NUCKS1	0.5050	0.8433	0.9887	0.7251	0.7656	0.1022
POLR2H	0.5531	1.1062	0.7895	0.6139	0.7657	0.1241
MORC3	0.6380	0.9319	0.8419	0.6514	0.7658	0.0724
KIAA1468	0.5758	0.9552	0.8515	0.6809	0.7659	0.0849
PAN2	0.7891	0.9307	0.6990	0.6447	0.7659	0.0625
EXOSC4	0.6157	0.9992	0.9068	0.5421	0.7659	0.1107
XBP1	0.6187	1.0192	0.7685	0.6573	0.7659	0.0902
CD3EAP	0.5773	0.8570	0.8558	0.7738	0.7660	0.0658
KEAP1	0.6814	1.0282	0.7004	0.6539	0.7660	0.0879
LOC1001315	0.5646	0.9635	0.8744	0.6617	0.7661	0.0923
RGS10	0.6726	0.9953	0.7759	0.6208	0.7661	0.0829
FLJ12684	0.6086	0.9707	0.7614	0.7239	0.7662	0.0755
LOC648024	0.5313	1.0265	0.8862	0.6215	0.7664	0.1148
ABCA1	0.7374	0.9165	0.6942	0.7174	0.7664	0.0508
LOC729852	0.5497	1.0472	0.7964	0.6722	0.7664	0.1063
STX4	0.7064	1.0389	0.6960	0.6244	0.7664	0.0926
FAM86B1	0.5603	1.0158	0.8263	0.6639	0.7666	0.0995
C18orf55	0.5352	0.8745	0.9492	0.7082	0.7668	0.0922
TMEM147	0.6320	1.0478	0.7621	0.6254	0.7668	0.0988
YIF1B	0.6244	1.0135	0.7847	0.6455	0.7670	0.0895
PARP1	0.6379	0.9849	0.8141	0.6317	0.7671	0.0840
SNORD35B	0.7734	0.7827	0.8431	0.6693	0.7671	0.0361
LOC345041	0.5202	1.1538	0.7781	0.6168	0.7672	0.1394
RPUSD1	0.5803	0.9054	0.8414	0.7419	0.7672	0.0708
TRAIP	0.6993	0.7973	0.7583	0.8141	0.7673	0.0255
NUPR1	0.7716	0.9138	0.6272	0.7565	0.7673	0.0586
STRADB	0.6157	1.1121	0.6665	0.6750	0.7673	0.1157
SPATS2L	0.6144	1.0148	0.8583	0.5818	0.7673	0.1030
APOA1BP	0.5581	0.9601	0.9558	0.5953	0.7673	0.1103
ANAPC5	0.6292	1.1286	0.7625	0.5493	0.7674	0.1282
CCDC12	0.6986	0.9895	0.6739	0.7079	0.7675	0.0743
ACP1	0.5710	1.0788	0.7638	0.6563	0.7675	0.1110
LOC644033	0.6443	0.9544	0.8744	0.5971	0.7675	0.0869
TAF6L	0.6090	1.0090	0.8126	0.6405	0.7678	0.0920
LOC1001338	0.6184	1.0243	0.7868	0.6420	0.7679	0.0932
C9orf5	0.5961	0.8593	1.0036	0.6133	0.7681	0.0989
SLC25A36	0.6211	1.0643	0.7370	0.6503	0.7682	0.1017
HTRA2	0.6422	0.9631	0.7460	0.7220	0.7683	0.0686
NOP14	0.5282	0.8883	0.9826	0.6742	0.7683	0.1028
LOC652826	0.5995	1.1157	0.7656	0.5926	0.7683	0.1225
NRTN	0.6350	0.9057	0.8028	0.7306	0.7685	0.0572

DENND1A	0.5959	1.0010	0.8108	0.6675	0.7688	0.0894
ISCA2	0.6736	0.8895	0.8402	0.6720	0.7688	0.0563
KPNB1	0.6306	0.9098	0.9199	0.6154	0.7689	0.0843
TCTA	0.5918	1.0399	0.7726	0.6718	0.7690	0.0976
PABPC4	0.5977	1.0428	0.7546	0.6811	0.7690	0.0967
PYCR1	0.7784	0.9737	0.6436	0.6809	0.7692	0.0739
WDR81	0.7184	0.9304	0.7245	0.7041	0.7694	0.0539
POLD1	0.7012	0.9168	0.7064	0.7533	0.7694	0.0505
BCL7C	0.6965	0.8600	0.8497	0.6720	0.7695	0.0495
USP14	0.5753	0.9300	0.8359	0.7371	0.7696	0.0758
NCOA6	0.6723	1.2147	0.6103	0.5815	0.7697	0.1495
GOLM1	0.7332	0.9061	0.8293	0.6103	0.7697	0.0638
SP1	0.6447	0.9611	0.8229	0.6504	0.7698	0.0760
LOC390557	0.5852	1.2160	0.7099	0.5682	0.7698	0.1520
ADAP2	0.6416	1.0302	0.7684	0.6392	0.7698	0.0919
CLPTM1L	0.5646	1.0451	0.8025	0.6671	0.7698	0.1039
C18orf19	0.5591	0.9827	0.8244	0.7132	0.7699	0.0894
HNRNPR	0.6649	1.0092	0.7689	0.6364	0.7699	0.0847
ZNF583	0.5949	0.9024	0.8710	0.7114	0.7699	0.0718
LOC1001329	0.6442	1.0040	0.8470	0.5844	0.7699	0.0962
DCPS	0.6802	0.9676	0.7186	0.7134	0.7699	0.0664
FANCE	0.6221	1.0115	0.8037	0.6426	0.7700	0.0902
HSPB1	0.6081	0.9929	0.8481	0.6311	0.7701	0.0919
ZDHHC6	0.7059	0.9962	0.7858	0.5928	0.7702	0.0851
LPAR3	0.5289	1.0407	0.9214	0.5903	0.7703	0.1247
GHDC	0.7993	0.7964	0.7759	0.7098	0.7704	0.0208
LOC728368	0.6431	0.8864	0.9148	0.6373	0.7704	0.0754
GDI2	0.6203	0.9669	0.9165	0.5780	0.7704	0.0998
LOC644101	0.6127	0.8968	0.8378	0.7345	0.7704	0.0624
TMEM209	0.6036	0.9634	0.9021	0.6129	0.7705	0.0945
TRIM61	0.5680	1.0135	0.8481	0.6529	0.7706	0.1000
ADAM9	0.3985	1.2766	0.7467	0.6607	0.7706	0.1842
CEBPB	0.6501	1.0624	0.7120	0.6581	0.7707	0.0982
FSIP1	0.7225	0.8358	0.7942	0.7306	0.7708	0.0270
TOMM6	0.5978	0.9228	0.9181	0.6446	0.7708	0.0869
WDR12	0.5796	1.0237	0.8017	0.6783	0.7708	0.0958
PLEKHB2	0.5656	1.0207	0.7367	0.7603	0.7708	0.0939
RPAP3	0.5843	1.0338	0.7829	0.6824	0.7709	0.0966
MIR586	0.5727	1.0885	0.7974	0.6253	0.7710	0.1162
EHBP1L1	0.7621	0.8413	0.7184	0.7623	0.7710	0.0256
MRRF	0.6192	1.0662	0.7387	0.6603	0.7711	0.1014
LOC643167	0.7321	1.0229	0.7458	0.5838	0.7712	0.0916
HSD11B2	0.5604	0.8968	0.8929	0.7346	0.7712	0.0798
TPI1	0.7425	0.9055	0.8315	0.6053	0.7712	0.0646
DBR1	0.6334	0.8988	0.7975	0.7553	0.7712	0.0549
DHFR	0.6523	0.8675	0.9002	0.6649	0.7712	0.0654

TCERG1	0.6793	0.9145	0.7572	0.7340	0.7713	0.0504
FAM98A	0.5445	0.8318	1.0769	0.6326	0.7714	0.1182
FLJ45248	0.8161	0.8426	0.7735	0.6538	0.7715	0.0417
ARF3	0.6321	1.0959	0.7297	0.6287	0.7716	0.1106
SLC39A9	0.6818	0.9409	0.8585	0.6052	0.7716	0.0774
CCDC14	0.6968	1.0617	0.7232	0.6052	0.7717	0.0999
RPE	0.6301	1.0274	0.7581	0.6719	0.7719	0.0892
EPDR1	0.7062	0.9967	0.7747	0.6099	0.7719	0.0822
CDCA7L	0.5748	1.0014	0.9055	0.6065	0.7721	0.1067
ZNF275	0.5828	1.0176	0.8185	0.6695	0.7721	0.0952
SNF8	0.8074	0.7735	0.7944	0.7132	0.7721	0.0208
C5orf34	0.6132	0.8959	0.7614	0.8182	0.7722	0.0597
MGC72080	0.6265	0.9396	0.8732	0.6495	0.7722	0.0788
SLC35D2	0.6936	0.9815	0.7758	0.6382	0.7723	0.0753
ANKRD29	0.6633	0.8224	0.9489	0.6545	0.7723	0.0704
BCCIP	0.5505	1.0531	0.8302	0.6554	0.7723	0.1100
ZNF787	0.6537	0.8821	0.8557	0.6978	0.7723	0.0567
C6orf125	0.7221	0.8847	0.8293	0.6531	0.7723	0.0521
ASXL2	0.5471	1.1161	0.7449	0.6814	0.7724	0.1218
RNF167	0.6705	0.9930	0.8540	0.5721	0.7724	0.0939
CCDC124	0.5898	1.0119	0.7740	0.7141	0.7725	0.0886
BNIP3	0.8201	1.0468	0.6619	0.5611	0.7725	0.1059
PSMD11	0.5221	1.0070	0.8292	0.7320	0.7726	0.1010
C16orf80	0.7167	1.0152	0.7551	0.6040	0.7728	0.0869
TMEM167A	0.6008	0.9236	0.9801	0.5869	0.7728	0.1040
CTCF	0.6388	1.0675	0.7037	0.6820	0.7730	0.0991
EIF2AK2	0.6164	0.8456	0.9773	0.6528	0.7730	0.0847
PKM2	0.6636	1.0553	0.7307	0.6428	0.7731	0.0959
SLTM	0.6371	0.9909	0.8046	0.6598	0.7731	0.0815
FAM89A	0.6251	0.9599	0.8210	0.6866	0.7731	0.0745
ZP3	0.7613	0.9579	0.7385	0.6350	0.7732	0.0674
ACTR6	0.6478	1.0428	0.8065	0.5958	0.7732	0.1004
MRPL22	0.6179	1.0026	0.8078	0.6647	0.7732	0.0865
MRPL38	0.6638	0.9091	0.8705	0.6502	0.7734	0.0677
HAGHL	0.6164	0.9693	0.7575	0.7514	0.7736	0.0729
NUDT18	0.7169	1.0297	0.6813	0.6668	0.7737	0.0860
RAPGEF5	0.6824	0.9506	0.8182	0.6437	0.7737	0.0698
NSMCE4A	0.7248	0.9301	0.7549	0.6853	0.7738	0.0540
NRBF2	0.6615	0.7407	0.9388	0.7544	0.7739	0.0587
LOC441050	0.5118	1.2444	0.7674	0.5721	0.7739	0.1660
RNF26	0.6692	0.9503	0.8029	0.6743	0.7742	0.0663
PI4K2B	0.6072	1.0055	0.7626	0.7214	0.7742	0.0838
TFRC	0.6598	1.0776	0.7801	0.5798	0.7743	0.1092
CXorf15	0.6308	0.9349	0.8096	0.7223	0.7744	0.0648
LOC1001336	0.5903	0.9976	0.7885	0.7214	0.7745	0.0850
UHMK1	0.6469	0.9704	0.7889	0.6922	0.7746	0.0717

PARP16	0.8040	0.7915	0.7040	0.7990	0.7746	0.0237
HNRNPAB	0.5948	1.0909	0.7736	0.6391	0.7746	0.1121
MRPL4	0.6674	0.9540	0.7750	0.7021	0.7746	0.0638
SMUG1	0.7090	0.9188	0.8946	0.5765	0.7747	0.0810
MRPS31	0.6151	0.9341	0.8866	0.6633	0.7748	0.0795
ABHD11	0.6570	0.9183	0.8391	0.6847	0.7748	0.0624
EIF2A	0.5872	1.0107	0.8491	0.6523	0.7748	0.0963
LOC1001300	0.6460	0.9487	0.8854	0.6192	0.7748	0.0833
RPF2	0.5738	0.8921	0.9884	0.6451	0.7748	0.0986
TRIM37	0.6661	0.9506	0.7614	0.7214	0.7749	0.0618
SLC35A4	0.5852	1.1698	0.7001	0.6446	0.7749	0.1337
KIAA0367	0.7173	0.9809	0.7440	0.6577	0.7750	0.0710
WDR8	0.6357	0.9760	0.8177	0.6705	0.7750	0.0778
TGIF1	0.6302	0.9918	0.7707	0.7076	0.7750	0.0777
DPP3	0.7061	0.9609	0.7655	0.6678	0.7751	0.0651
KBTBD11	0.6754	1.0535	0.7214	0.6503	0.7751	0.0940
FAM83H	0.7150	1.0064	0.6906	0.6887	0.7752	0.0773
TMEM218	0.6360	0.9504	0.8904	0.6239	0.7752	0.0848
TSSC1	0.6750	1.0854	0.7955	0.5449	0.7752	0.1154
IFIH1	0.6539	0.9106	0.8438	0.6926	0.7752	0.0609
SLC39A14	0.6845	1.0198	0.7654	0.6314	0.7753	0.0860
LOC728666	0.5261	1.1391	0.8254	0.6110	0.7754	0.1366
LOC1001323	0.6118	0.9742	0.7397	0.7761	0.7754	0.0750
TEAD2	0.7609	0.9877	0.6410	0.7123	0.7755	0.0749
CKAP2	0.6128	1.0575	0.7418	0.6898	0.7755	0.0977
SLC37A3	0.6666	1.0819	0.6747	0.6789	0.7755	0.1022
C1orf163	0.5885	0.9986	0.8729	0.6422	0.7756	0.0966
WDR74	0.6419	0.9893	0.8310	0.6409	0.7758	0.0840
DEPDC1	0.6440	0.9170	0.8276	0.7146	0.7758	0.0604
POLD3	0.6721	0.8970	0.7573	0.7769	0.7758	0.0464
ALG10B	0.6621	0.9438	0.7785	0.7189	0.7758	0.0608
ZNF207	0.5838	1.0337	0.8308	0.6551	0.7759	0.1004
LARS2	0.6731	1.0410	0.7890	0.6006	0.7759	0.0965
KRTCAP3	0.5516	0.9730	0.9559	0.6237	0.7761	0.1098
FJX1	0.5792	0.9207	0.8761	0.7289	0.7762	0.0774
MRPL45	0.6042	0.9745	0.9236	0.6029	0.7763	0.1003
ZNF395	0.7654	0.9566	0.6762	0.7073	0.7764	0.0629
TUSC4	0.6185	0.9972	0.8333	0.6565	0.7764	0.0872
HERC3	0.5927	1.0981	0.7404	0.6745	0.7764	0.1114
FLAD1	0.6887	0.9366	0.8020	0.6784	0.7764	0.0603
ZFYVE19	0.6753	1.0372	0.7661	0.6273	0.7765	0.0916
ABHD8	0.5738	1.1459	0.6785	0.7081	0.7766	0.1264
C1orf19	0.6919	0.9490	0.8551	0.6106	0.7766	0.0767
TM2D1	0.6753	1.0135	0.8411	0.5769	0.7767	0.0959
LOC1001288	0.6640	0.8697	0.7153	0.8580	0.7768	0.0514
MPV17L	0.7352	0.8392	0.8550	0.6778	0.7768	0.0424

AGK	0.5908	1.0036	0.8473	0.6657	0.7769	0.0928
ZNF282	0.7561	0.9075	0.8086	0.6353	0.7769	0.0567
NAV1	0.6506	0.9954	0.6992	0.7624	0.7769	0.0763
NEIL3	0.7406	0.8302	0.7895	0.7473	0.7769	0.0208
CHRNA5	0.6114	0.9528	0.8558	0.6876	0.7769	0.0778
BRCC3	0.6991	0.8838	0.8315	0.6933	0.7769	0.0478
BUD31	0.6375	1.0079	0.8238	0.6385	0.7769	0.0886
TEX10	0.5944	1.0423	0.8274	0.6438	0.7770	0.1017
LOC645015	0.6199	0.9429	0.8881	0.6571	0.7770	0.0811
RRP15	0.5369	1.0229	0.9382	0.6101	0.7770	0.1197
PELP1	0.6975	0.9039	0.8675	0.6396	0.7771	0.0642
C19orf6	0.7335	0.9808	0.7325	0.6621	0.7772	0.0699
PSME3	0.6233	0.9839	0.8701	0.6317	0.7772	0.0895
RPA1	0.6294	1.0315	0.7900	0.6584	0.7773	0.0917
NRAS	0.6377	0.9990	0.8128	0.6598	0.7773	0.0835
RAVER2	0.8246	0.8681	0.6742	0.7431	0.7775	0.0431
TRMT112	0.6457	1.0099	0.8002	0.6543	0.7775	0.0852
CHCHD8	0.4852	1.1120	0.8403	0.6728	0.7776	0.1330
CAPRIN1	0.6193	1.0434	0.7758	0.6720	0.7776	0.0944
LOC729680	0.6123	0.9305	0.8949	0.6729	0.7776	0.0793
C19orf42	0.6173	1.0767	0.7509	0.6658	0.7776	0.1034
MAPKAP1	0.6689	1.0185	0.7848	0.6385	0.7777	0.0862
ZNF581	0.6603	1.0325	0.7316	0.6866	0.7778	0.0862
NUDC	0.6227	1.0570	0.8274	0.6044	0.7779	0.1059
ACY1	0.6247	1.0172	0.7903	0.6796	0.7779	0.0869
YTHDF2	0.6937	0.9110	0.8500	0.6573	0.7780	0.0609
SPR	0.6916	0.9709	0.8014	0.6481	0.7780	0.0719
SBF2	0.6859	0.9697	0.7982	0.6583	0.7780	0.0707
LOC645233	0.6539	0.9622	0.8996	0.5965	0.7781	0.0900
UCHL5IP	0.6679	0.9371	0.8446	0.6627	0.7781	0.0678
FAM81A	0.6598	0.9309	0.8784	0.6432	0.7781	0.0739
RAB8B	0.8140	0.8785	0.8136	0.6062	0.7781	0.0593
NIP7	0.5772	1.0030	0.9372	0.5951	0.7781	0.1117
ADH5	0.8056	1.0387	0.6047	0.6634	0.7781	0.0966
PSMB3	0.6304	1.0959	0.7111	0.6750	0.7781	0.1072
EPRS	0.7195	0.9855	0.8206	0.5881	0.7784	0.0838
HCCS	0.6593	0.8437	0.8913	0.7194	0.7784	0.0537
BCS1L	0.5915	1.0271	0.8229	0.6722	0.7784	0.0958
QTRTD1	0.7034	0.9824	0.7933	0.6348	0.7785	0.0753
PRPS2	0.6251	1.0148	0.7748	0.6995	0.7785	0.0845
RRP9	0.6999	0.9258	0.7585	0.7304	0.7786	0.0505
RBM22	0.6340	1.0366	0.7659	0.6785	0.7787	0.0902
KIAA1524	0.5951	0.8469	0.8237	0.8496	0.7788	0.0615
C17orf79	0.5211	1.2007	0.7527	0.6409	0.7789	0.1483
RHOQ	0.5695	1.0302	0.8039	0.7122	0.7790	0.0966
FBXO21	0.6330	1.0344	0.7590	0.6895	0.7790	0.0890

LOC648695	0.5616	1.0716	0.8517	0.6311	0.7790	0.1155
KCTD6	0.6408	0.8914	0.8811	0.7029	0.7790	0.0632
UTP18	0.8265	0.9005	0.8032	0.5865	0.7792	0.0675
POLA1	0.6643	0.9414	0.7411	0.7701	0.7793	0.0585
PES1	0.6035	0.9662	0.9081	0.6394	0.7793	0.0922
MGST3	0.6316	1.1951	0.7548	0.5358	0.7793	0.1456
SLCO5A1	0.7490	0.8755	0.7369	0.7561	0.7794	0.0323
LMNB2	0.5780	1.2195	0.7172	0.6031	0.7794	0.1498
TMEM208	0.6520	1.0679	0.8199	0.5780	0.7794	0.1087
MID1IP1	0.7429	0.9027	0.8446	0.6277	0.7795	0.0604
FAM50A	0.6938	1.0837	0.7804	0.5603	0.7795	0.1110
DNLZ	0.6401	1.1839	0.7734	0.5208	0.7795	0.1443
PPAN	0.6402	1.0086	0.7400	0.7297	0.7796	0.0796
GNPNAT1	0.7148	0.9280	0.8283	0.6477	0.7797	0.0619
PEX11B	0.6903	1.1050	0.6904	0.6341	0.7800	0.1091
GNB1L	0.5658	1.1838	0.7595	0.6109	0.7800	0.1408
CHPT1	0.7065	1.0381	0.7834	0.5922	0.7801	0.0946
NDUFA6	0.6265	1.0057	0.8731	0.6151	0.7801	0.0959
PYCRL	0.6410	0.8907	0.8423	0.7466	0.7801	0.0552
CLCC1	0.7052	0.8841	0.7740	0.7575	0.7802	0.0376
LOC389322	0.6761	0.9518	0.8987	0.5948	0.7804	0.0860
SHQ1	0.6128	1.0552	0.7979	0.6569	0.7807	0.0996
DCAF16	0.5398	1.0214	0.8344	0.7277	0.7808	0.1007
TUBB2C	0.5773	0.9902	0.9119	0.6439	0.7808	0.1005
PSMA2	0.5967	0.8916	0.8873	0.7481	0.7809	0.0699
WDR79	0.6438	0.9339	0.8925	0.6536	0.7810	0.0768
C19orf70	0.5546	1.0693	0.8710	0.6290	0.7810	0.1175
LMAN2L	0.6656	1.0892	0.7699	0.5993	0.7810	0.1086
HELLS	0.6100	0.9927	0.8215	0.6999	0.7810	0.0828
UQCRFS1	0.6481	1.0333	0.9138	0.5290	0.7810	0.1163
HIST1H3F	0.5542	1.2150	0.7396	0.6155	0.7811	0.1497
TAF5	0.6405	0.9224	0.7594	0.8020	0.7811	0.0582
C6orf111	0.6617	0.9913	0.7417	0.7297	0.7811	0.0723
JPH1	0.5493	1.0095	0.8532	0.7125	0.7811	0.0982
PLEKHN1	0.7629	0.8350	0.7395	0.7871	0.7811	0.0204
KLF11	0.6166	1.0239	0.7980	0.6863	0.7812	0.0891
DRG1	0.5983	1.0024	0.8900	0.6345	0.7813	0.0982
CMTM4	0.6373	1.0637	0.7234	0.7016	0.7815	0.0958
CHCHD4	0.5552	1.1044	0.8349	0.6316	0.7815	0.1228
LOC653119	0.6451	0.9841	0.8046	0.6926	0.7816	0.0753
NUPL2	0.6233	1.0929	0.7578	0.6529	0.7817	0.1077
MPHOSPH9	0.6707	0.9878	0.7706	0.6979	0.7817	0.0718
SUSD2	0.7618	1.0321	0.6819	0.6512	0.7817	0.0866
NUDT6	0.6621	1.0456	0.7874	0.6321	0.7818	0.0941
CARM1	0.7203	1.0406	0.7009	0.6654	0.7818	0.0870
GEMIN6	0.5300	1.1571	0.8094	0.6307	0.7818	0.1378

UBE2Q1	0.7180	0.9683	0.7959	0.6451	0.7819	0.0694
NUDT11	0.7478	0.8889	0.7912	0.6996	0.7819	0.0403
MTX1	0.6441	1.0496	0.7895	0.6445	0.7819	0.0956
DCTD	0.5744	1.1302	0.7906	0.6325	0.7819	0.1247
RBM39	0.7046	0.9803	0.8120	0.6311	0.7820	0.0758
SNORD31	0.6538	1.0009	0.7105	0.7628	0.7820	0.0763
ATAD3A	0.5831	1.1487	0.7449	0.6516	0.7821	0.1266
SNORD80	0.7452	0.8734	0.7972	0.7126	0.7821	0.0351
TMED4	0.5659	1.1684	0.8052	0.5891	0.7821	0.1396
ABHD14A	0.6393	1.0067	0.8022	0.6806	0.7822	0.0824
LOC440498	0.6515	1.0780	0.7877	0.6117	0.7822	0.1055
SFRS8	0.7476	0.9324	0.7839	0.6657	0.7824	0.0558
LOC730052	0.6078	0.9799	0.9178	0.6244	0.7825	0.0970
DOHH	0.7185	0.9680	0.8430	0.6007	0.7825	0.0792
HSPE1	0.7098	0.9520	0.9313	0.5374	0.7826	0.0984
C5orf54	0.6539	1.0370	0.7671	0.6727	0.7827	0.0883
PLS3	0.6742	0.9628	0.8387	0.6553	0.7828	0.0728
CEP135	0.7436	0.9444	0.7570	0.6867	0.7829	0.0559
PTPN3	0.6639	0.9677	0.7766	0.7236	0.7830	0.0657
LOC441019	0.6865	0.8802	0.8060	0.7595	0.7830	0.0407
SLC25A11	0.6704	0.9927	0.8617	0.6077	0.7831	0.0883
SC65	0.6205	1.0036	0.8866	0.6219	0.7831	0.0965
SRBD1	0.6607	0.9937	0.8147	0.6636	0.7832	0.0788
ISG15	0.6347	1.0910	0.7314	0.6758	0.7832	0.1045
TFAM	0.6309	0.9704	0.8178	0.7142	0.7833	0.0731
SNORA6	0.6243	1.1740	0.7235	0.6114	0.7833	0.1326
ST6GALNAC6	0.6256	0.9822	0.7791	0.7466	0.7834	0.0741
ZNHIT2	0.6898	1.0199	0.8150	0.6088	0.7834	0.0895
VPS52	0.7257	0.9973	0.7544	0.6562	0.7834	0.0742
LYPLA2	0.7164	1.0137	0.7136	0.6901	0.7834	0.0770
C5orf25	0.7194	0.8913	0.7706	0.7527	0.7835	0.0375
PTCD2	0.6176	1.0267	0.8454	0.6451	0.7837	0.0956
PRPF3	0.5576	1.1454	0.6932	0.7387	0.7837	0.1265
RBM28	0.7552	0.8795	0.7134	0.7877	0.7839	0.0353
LOC221710	0.6617	0.8053	0.8996	0.7693	0.7840	0.0492
LCLAT1	0.6767	0.9843	0.8379	0.6369	0.7840	0.0797
MRPS14	0.6326	1.0629	0.7785	0.6622	0.7840	0.0981
TMEM143	0.7339	0.8214	0.8015	0.7795	0.7841	0.0188
PHB2	0.5106	1.1098	0.8892	0.6269	0.7841	0.1344
STK24	0.6941	1.0495	0.7605	0.6326	0.7842	0.0922
SF3B5	0.6276	1.0968	0.8385	0.5741	0.7842	0.1188
ULBP1	0.7886	0.9200	0.6751	0.7532	0.7842	0.0511
SLC25A40	0.6418	0.9675	0.8107	0.7172	0.7843	0.0702
NME6	0.7220	0.8835	0.8275	0.7043	0.7843	0.0428
SLC7A11	0.8384	0.8497	0.6776	0.7717	0.7843	0.0395
CDH15	0.7061	0.8982	0.7613	0.7721	0.7845	0.0406

PRKAR1B	0.7000	1.0124	0.7821	0.6435	0.7845	0.0811
MRPS7	0.6010	1.0509	0.8207	0.6654	0.7845	0.1001
CEP192	0.6677	0.9346	0.7798	0.7561	0.7846	0.0555
CCDC76	0.6669	1.0364	0.6826	0.7525	0.7846	0.0860
BAT1	0.5611	1.1026	0.8148	0.6599	0.7846	0.1182
LOC729535	0.4840	1.0509	0.8509	0.7530	0.7847	0.1179
EML4	0.7975	0.9693	0.7258	0.6464	0.7847	0.0688
GAS8	0.7343	0.9960	0.6978	0.7110	0.7848	0.0708
PSMA1	0.6223	1.0612	0.8211	0.6345	0.7848	0.1028
BCKDK	0.6934	1.0617	0.7011	0.6835	0.7849	0.0923
DDX10	0.5586	1.0364	0.8242	0.7207	0.7850	0.1001
C20orf7	0.6446	0.8963	0.9575	0.6420	0.7851	0.0828
GAGE2A	0.6013	1.0297	0.9323	0.5772	0.7851	0.1149
LOC644762	0.5106	1.2693	0.8295	0.5313	0.7852	0.1771
GNAI3	0.6934	0.8529	0.9189	0.6759	0.7853	0.0597
LOC1001319	0.6207	1.0412	0.8456	0.6340	0.7854	0.0996
SFRS17A	0.6604	0.9643	0.8086	0.7083	0.7854	0.0672
LOC729768	0.5558	1.0930	0.8548	0.6380	0.7854	0.1204
SIX4	0.5410	1.1415	0.7626	0.6966	0.7854	0.1274
PPP2R4	0.6335	0.9983	0.8604	0.6496	0.7855	0.0878
PINX1	0.6208	1.0289	0.7894	0.7029	0.7855	0.0881
AMY1C	0.5689	0.8642	1.0191	0.6897	0.7855	0.0987
PAK1	0.6551	1.0351	0.7964	0.6556	0.7855	0.0896
IFITM2	0.7355	1.0315	0.7969	0.5784	0.7856	0.0940
POP1	0.6003	1.0011	0.7898	0.7512	0.7856	0.0827
FAM58A	0.6009	1.0230	0.8469	0.6719	0.7856	0.0945
SRXN1	0.7238	1.0736	0.7741	0.5713	0.7857	0.1052
TXN2	0.7347	1.0149	0.7822	0.6111	0.7857	0.0845
TIMP2	0.8045	0.9114	0.7559	0.6712	0.7858	0.0501
PPARBP	0.6121	0.9118	0.8756	0.7439	0.7858	0.0682
LOC1001344	0.6684	0.9736	0.8638	0.6379	0.7859	0.0801
FREQ	0.6792	1.0248	0.7070	0.7329	0.7860	0.0804
EIF2B5	0.7298	1.0363	0.7174	0.6606	0.7860	0.0848
NRGN	0.6847	0.8368	0.8286	0.7940	0.7860	0.0350
PSMD1	0.7046	0.9959	0.7870	0.6568	0.7861	0.0749
ITPKA	0.7874	0.9194	0.7948	0.6428	0.7861	0.0565
C8orf76	0.6004	1.0834	0.8392	0.6217	0.7862	0.1128
ACTR5	0.6875	0.9626	0.7438	0.7510	0.7862	0.0605
RIF1	0.6395	0.9435	0.8352	0.7267	0.7862	0.0660
MAST3	0.7111	0.9547	0.7127	0.7669	0.7863	0.0576
DCLRE1A	0.7350	0.9242	0.8977	0.5886	0.7864	0.0781
HOXB13	0.7249	1.0571	0.7502	0.6136	0.7864	0.0950
SNUPN	0.7756	0.9400	0.7902	0.6401	0.7865	0.0613
C12orf31	0.5754	1.0318	0.8732	0.6658	0.7866	0.1028
LOC729217	0.5694	1.2063	0.7645	0.6062	0.7866	0.1462
PDIA6	0.5538	1.1725	0.7065	0.7135	0.7866	0.1338

SLC2A6	0.6327	1.0282	0.7984	0.6872	0.7866	0.0876
SS18L2	0.6046	0.9881	0.8669	0.6871	0.7867	0.0866
LOC1001330	0.7449	0.8063	0.8883	0.7080	0.7869	0.0394
SLC27A2	0.6626	1.0285	0.8220	0.6345	0.7869	0.0905
GNB5	0.7115	0.9568	0.7676	0.7119	0.7869	0.0581
HIP2	0.5438	1.0053	0.9190	0.6799	0.7870	0.1063
CWF19L1	0.6273	1.1147	0.7669	0.6392	0.7870	0.1137
LOC1001322	0.7397	0.7847	0.9544	0.6694	0.7871	0.0606
LOC400013	0.6068	1.1240	0.8138	0.6038	0.7871	0.1226
C21orf51	0.6781	1.0377	0.7171	0.7155	0.7871	0.0840
PPA1	0.5579	1.1298	0.8434	0.6182	0.7873	0.1296
UBA6	0.6880	0.8903	0.9677	0.6039	0.7875	0.0850
VPS35	0.6493	0.9398	0.8969	0.6639	0.7875	0.0761
PTPN2	0.6664	0.9303	0.8416	0.7119	0.7876	0.0603
GAS2L3	0.6223	0.9036	0.8194	0.8050	0.7876	0.0592
EIF2C2	0.6359	1.1292	0.6896	0.6958	0.7876	0.1147
C5orf22	0.6086	0.8751	0.9878	0.6798	0.7878	0.0873
ORAI1	0.6214	0.8838	0.7532	0.8938	0.7880	0.0641
LITAF	0.6263	0.9260	0.9250	0.6749	0.7881	0.0800
NAPG	0.6907	1.0350	0.7724	0.6543	0.7881	0.0859
TATDN2	0.6357	1.0239	0.8795	0.6137	0.7882	0.0990
WSB1	0.6897	0.9730	0.7898	0.7003	0.7882	0.0656
MAPRE1	0.6298	1.0933	0.8800	0.5496	0.7882	0.1237
LOC730358	0.5746	1.1886	0.7871	0.6027	0.7882	0.1415
DHODH	0.6370	1.0193	0.8109	0.6858	0.7882	0.0853
AP4M1	0.7647	0.8017	0.7850	0.8019	0.7883	0.0088
TERF2	0.6717	1.1953	0.6932	0.5935	0.7884	0.1373
NOL7	0.6425	1.0480	0.7703	0.6933	0.7885	0.0904
LOC653479	0.6012	1.1533	0.8064	0.5935	0.7886	0.1312
SNX12	0.7224	1.0364	0.7623	0.6339	0.7887	0.0868
DCUN1D5	0.6189	0.9868	0.8594	0.6900	0.7888	0.0831
PITRM1	0.6439	1.0599	0.8042	0.6470	0.7888	0.0978
ZFAND1	0.6405	1.0357	0.8305	0.6487	0.7889	0.0932
C13orf34	0.6553	0.9070	0.7467	0.8465	0.7889	0.0555
C11orf73	0.6341	1.0838	0.7367	0.7010	0.7889	0.1006
CBX3	0.5565	1.1041	0.7463	0.7489	0.7889	0.1143
KISS1R	0.7647	0.9474	0.7493	0.6946	0.7890	0.0549
GNA13	0.7685	0.9449	0.7602	0.6825	0.7890	0.0554
ANAPC1	0.7389	1.0224	0.6914	0.7036	0.7891	0.0784
PDCD4	0.6921	1.0281	0.8126	0.6234	0.7891	0.0888
MUTYH	0.5989	1.1483	0.7808	0.6291	0.7893	0.1261
RBM15	0.6806	1.0459	0.7564	0.6744	0.7893	0.0875
LOC400455	0.5668	1.0948	0.9044	0.5914	0.7893	0.1276
GOLPH4	0.4951	1.0387	0.9325	0.6915	0.7894	0.1221
KIAA1430	0.6852	0.9999	0.7684	0.7048	0.7896	0.0723
GAGE12B	0.6607	0.9973	1.0093	0.4910	0.7896	0.1282

CENPQ	0.7290	0.8950	0.8849	0.6495	0.7896	0.0602
HNRNPA2B1	0.6242	1.0796	0.8253	0.6293	0.7896	0.1074
MORC2	0.6944	1.0358	0.7355	0.6930	0.7897	0.0826
TRIM27	0.6557	1.0151	0.7502	0.7379	0.7897	0.0780
ZIK1	0.7224	0.9994	0.7533	0.6839	0.7897	0.0713
ZNF264	0.6296	1.0130	0.8261	0.6909	0.7899	0.0849
VIL2	0.7358	0.9600	0.7643	0.6995	0.7899	0.0582
CARHSP1	0.6733	1.1035	0.7443	0.6385	0.7899	0.1068
SLC35B4	0.7545	0.8569	0.8085	0.7398	0.7899	0.0268
C20orf24	0.6081	1.0767	0.8346	0.6404	0.7899	0.1079
CEBPA	0.6947	1.0624	0.6965	0.7063	0.7900	0.0908
RG9MTD1	0.6185	0.8883	1.0445	0.6090	0.7901	0.1067
POMP	0.6146	1.0298	0.9152	0.6008	0.7901	0.1079
C9orf123	0.6853	0.9306	0.8822	0.6623	0.7901	0.0680
TAF4	0.6520	0.9289	0.9121	0.6676	0.7901	0.0754
LOC643668	0.5716	1.1432	0.8013	0.6445	0.7901	0.1271
EML3	0.7009	0.9435	0.8199	0.6963	0.7901	0.0586
LOC651697	0.6159	1.0348	0.9200	0.5902	0.7902	0.1107
UGT2B17	0.4790	1.1760	0.8649	0.6410	0.7902	0.1510
XPO1	0.5824	1.0863	0.9014	0.5910	0.7903	0.1234
FAM69A	0.6761	0.9288	0.8179	0.7386	0.7904	0.0545
MXD3	0.6504	0.8580	0.8477	0.8053	0.7904	0.0480
DUSP5	0.7415	0.9035	0.8024	0.7147	0.7905	0.0419
WDR77	0.6725	1.0744	0.7955	0.6200	0.7906	0.1015
LOC645762	0.7391	0.9517	0.8661	0.6057	0.7907	0.0755
TMEM60	0.6550	0.9376	0.8849	0.6852	0.7907	0.0707
LOC1001280	0.5396	1.2064	0.7806	0.6362	0.7907	0.1471
NSUN5	0.6795	0.9633	0.8560	0.6643	0.7908	0.0721
MT1G	0.7767	0.8003	0.7674	0.8191	0.7909	0.0117
LOC647597	0.6293	1.1289	0.7080	0.6973	0.7909	0.1140
ZC3H10	0.7795	0.8981	0.7860	0.7000	0.7909	0.0407
TMEM106C	0.5317	1.0859	0.8842	0.6621	0.7910	0.1223
ENO1	0.6688	0.9782	0.9040	0.6134	0.7911	0.0887
LOC441484	0.6373	0.9489	0.9445	0.6338	0.7911	0.0898
CASP3	0.6730	1.0358	0.7811	0.6748	0.7912	0.0854
SPIRE1	0.6502	1.1139	0.7418	0.6588	0.7912	0.1095
TIMM8A	0.6669	0.9721	0.9454	0.5806	0.7912	0.0984
FTSJ1	0.6844	0.9043	0.9302	0.6461	0.7913	0.0734
ZNF668	0.6644	1.0243	0.8251	0.6513	0.7913	0.0871
LNPEP	0.6911	0.9003	0.8603	0.7139	0.7914	0.0522
SUMO3	0.6228	1.0219	0.8319	0.6893	0.7915	0.0883
ALG13	0.5749	1.0384	0.9043	0.6484	0.7915	0.1084
BTN3A2	0.7003	0.9335	0.8036	0.7285	0.7915	0.0521
LOC441455	0.7331	1.0741	0.6983	0.6608	0.7916	0.0953
RPUSD4	0.6463	1.0571	0.8261	0.6368	0.7916	0.0986
ZNF446	0.7101	1.0501	0.7693	0.6371	0.7916	0.0903

BRCA1	0.7030	0.9588	0.7770	0.7280	0.7917	0.0578
NOMO3	0.5855	1.0386	0.8931	0.6497	0.7917	0.1057
C6orf66	0.5557	1.0341	0.8261	0.7513	0.7918	0.0989
C3orf64	0.6877	0.8961	0.8391	0.7444	0.7918	0.0467
BOLA1	0.6298	0.9101	0.8256	0.8018	0.7918	0.0588
INTS3	0.6304	0.9922	0.8038	0.7414	0.7919	0.0758
CEBPZ	0.6157	0.9003	0.9588	0.6929	0.7919	0.0819
MEPCE	0.7529	0.9754	0.8269	0.6127	0.7920	0.0756
RNASET2	0.6538	1.0226	0.8144	0.6771	0.7920	0.0846
LOC723972	0.5666	0.9589	0.9121	0.7305	0.7920	0.0899
NOP2	0.6549	1.0804	0.7989	0.6339	0.7920	0.1029
ZMYND19	0.5803	1.1313	0.8273	0.6294	0.7920	0.1250
ELK1	0.6163	1.0708	0.8291	0.6524	0.7922	0.1039
LOC399748	0.7031	0.9559	0.8380	0.6721	0.7923	0.0654
PDE12	0.7055	0.9276	0.7827	0.7534	0.7923	0.0478
TRAPPC2L	0.7359	0.9972	0.8365	0.5999	0.7923	0.0837
MRPS18B	0.5785	0.9869	0.9457	0.6583	0.7924	0.1021
PON2	0.6636	1.0903	0.8459	0.5697	0.7924	0.1147
SLC7A5	0.8522	0.8468	0.7103	0.7605	0.7924	0.0345
C2orf47	0.6600	1.0231	0.9565	0.5305	0.7925	0.1177
NCBP2	0.7688	0.9767	0.7338	0.6908	0.7925	0.0634
LOC650515	0.6174	0.9768	0.8350	0.7410	0.7925	0.0759
GYG2	0.6922	1.0518	0.7583	0.6679	0.7926	0.0885
ZFP62	0.7629	0.9625	0.7369	0.7080	0.7926	0.0577
POLE	0.7978	0.8279	0.6927	0.8519	0.7926	0.0351
CD59	0.7137	0.9525	0.8215	0.6827	0.7926	0.0610
MBTPS2	0.6824	1.0546	0.7462	0.6873	0.7926	0.0885
NOL12	0.6658	0.9951	0.8148	0.6951	0.7927	0.0748
C14orf93	0.7843	0.9529	0.7632	0.6706	0.7927	0.0588
SPAG9	0.6689	0.9906	0.8257	0.6857	0.7927	0.0747
SFPQ	0.7724	0.8442	0.9470	0.6080	0.7929	0.0713
KANK2	0.7179	1.0880	0.7455	0.6203	0.7929	0.1020
TRIM45	0.8464	0.8423	0.7462	0.7376	0.7931	0.0296
POLDIP3	0.6484	1.0433	0.7907	0.6902	0.7931	0.0886
DOLPP1	0.6002	1.1341	0.8171	0.6211	0.7931	0.1237
LOC400657	0.6740	0.9262	0.8569	0.7155	0.7932	0.0591
MEA1	0.7111	0.9104	0.8979	0.6535	0.7932	0.0652
DTWD1	0.6755	0.9479	0.8809	0.6685	0.7932	0.0713
HOMER2	0.7517	1.1502	0.7289	0.5424	0.7933	0.1279
CES2	0.7201	0.9629	0.7754	0.7149	0.7933	0.0582
ZNF326	0.6406	0.9376	0.8178	0.7790	0.7937	0.0612
LYPLA1	0.4830	1.1296	0.9071	0.6553	0.7938	0.1418
SNRPC	0.6561	1.1467	0.8014	0.5709	0.7938	0.1269
LOC389137	0.7345	1.0239	0.7387	0.6784	0.7939	0.0779
KCTD3	0.7045	1.0417	0.8155	0.6139	0.7939	0.0923
DNAJC8	0.6526	0.9342	0.8594	0.7294	0.7939	0.0633

ITGAE	0.6194	1.0046	0.8526	0.6993	0.7940	0.0853
SERPINB6	0.7527	0.9641	0.8189	0.6402	0.7940	0.0676
DDX3X	0.7015	1.0032	0.8316	0.6398	0.7940	0.0804
RECQL4	0.7397	0.9071	0.8138	0.7156	0.7940	0.0431
CTSL1	0.5372	1.2072	0.8302	0.6016	0.7941	0.1514
CMBL	0.5253	1.0109	1.0138	0.6263	0.7941	0.1277
IFT74	0.7140	1.0077	0.7673	0.6875	0.7941	0.0731
RRS1	0.5816	1.0884	0.8688	0.6380	0.7942	0.1161
RFC2	0.7257	0.8704	0.8802	0.7006	0.7942	0.0471
C12orf10	0.5875	1.0087	0.8719	0.7091	0.7943	0.0922
WDR75	0.6560	0.9614	0.9387	0.6214	0.7944	0.0903
WDR76	0.6537	0.9238	0.7891	0.8110	0.7944	0.0554
C7orf25	0.7379	0.9818	0.7757	0.6824	0.7945	0.0653
TSGA14	0.8054	0.8965	0.7904	0.6857	0.7945	0.0432
LOC1001329	0.6106	0.9791	0.9819	0.6070	0.7947	0.1073
CCDC110	0.6684	0.9592	0.8520	0.6991	0.7947	0.0680
TMED2	0.6329	1.0846	0.8952	0.5662	0.7948	0.1199
LOC645332	0.5991	1.0224	0.8016	0.7558	0.7948	0.0874
VRK3	0.6999	0.9553	0.7802	0.7439	0.7948	0.0559
SPG21	0.6278	1.1065	0.8181	0.6270	0.7949	0.1132
HNRNPA0	0.6677	1.1306	0.7457	0.6356	0.7949	0.1143
UBE2G2	0.6649	1.0070	0.7754	0.7324	0.7949	0.0743
MTCP1	0.7440	0.9368	0.7677	0.7315	0.7950	0.0479
GGCT	0.5987	1.0498	0.9544	0.5769	0.7950	0.1212
PDCD6IP	0.6132	1.0527	0.8040	0.7101	0.7950	0.0943
KIAA1147	0.5952	1.1077	0.8189	0.6583	0.7950	0.1144
TBCD	0.7752	1.0440	0.7346	0.6264	0.7950	0.0887
LOC729082	0.6102	1.0342	0.8125	0.7234	0.7951	0.0898
RPA2	0.5997	1.1623	0.7785	0.6398	0.7951	0.1282
RNASEH2B	0.7503	0.9181	0.8563	0.6556	0.7951	0.0580
TUBB4Q	0.7370	0.7583	1.0199	0.6652	0.7951	0.0775
VPS41	0.7090	0.9999	0.8160	0.6555	0.7951	0.0760
G3BP1	0.6060	1.0611	0.7999	0.7138	0.7952	0.0971
PTCD1	0.6701	1.0817	0.8173	0.6119	0.7953	0.1048
C1orf43	0.6665	0.9616	0.8833	0.6698	0.7953	0.0751
STAT1	0.6259	1.0819	0.8247	0.6487	0.7953	0.1054
SNX13	0.7288	0.8956	0.8751	0.6817	0.7953	0.0530
XPO6	0.6795	1.0325	0.7876	0.6820	0.7954	0.0829
DDX47	0.5409	1.0813	0.8918	0.6678	0.7955	0.1198
LOC399804	0.5712	1.1627	0.9110	0.5374	0.7956	0.1486
PIGU	0.8346	0.8618	0.8070	0.6792	0.7956	0.0404
UQCRHL	0.5878	1.0111	1.0443	0.5394	0.7956	0.1345
ARID3A	0.6763	1.0312	0.6865	0.7885	0.7956	0.0825
NCAPD3	0.8583	0.9262	0.7487	0.6498	0.7957	0.0608
UBL7	0.6255	1.0089	0.8349	0.7136	0.7957	0.0830
ST3GAL4	0.6752	0.9797	0.8027	0.7255	0.7958	0.0667

COPS5	0.5946	1.1399	0.8170	0.6323	0.7959	0.1245
LOC651143	0.7149	1.0290	0.7748	0.6652	0.7960	0.0808
WDSUB1	0.6902	0.9410	0.8493	0.7035	0.7960	0.0603
S100PBP	0.6976	0.9763	0.7992	0.7109	0.7960	0.0642
B4GALT2	0.7109	1.0378	0.7630	0.6725	0.7960	0.0827
LOC1001282	0.8447	1.0178	0.7174	0.6046	0.7961	0.0887
C16orf42	0.7223	0.8115	0.9438	0.7069	0.7961	0.0544
TRPT1	0.6838	1.0056	0.8211	0.6742	0.7962	0.0775
TTL	0.5932	1.1592	0.7706	0.6619	0.7962	0.1264
LOC644334	0.6217	1.0498	0.8395	0.6745	0.7964	0.0964
SYNCRIP	0.6702	0.9467	0.8697	0.6991	0.7964	0.0667
TSTA3	0.8331	1.0067	0.7973	0.5487	0.7965	0.0944
LOC1001303	0.7121	0.8707	0.9572	0.6468	0.7967	0.0712
TAP2	0.5976	1.0265	0.7923	0.7706	0.7967	0.0881
RPS6KA5	0.6701	0.9560	0.8536	0.7072	0.7967	0.0662
CPT1A	0.6173	0.9816	0.8225	0.7661	0.7969	0.0753
ALKBH2	0.5211	1.3291	0.6982	0.6394	0.7969	0.1812
SMARCD2	0.6814	1.2232	0.7173	0.5659	0.7969	0.1457
CSRP2BP	0.7449	0.9542	0.8265	0.6625	0.7970	0.0622
C14orf80	0.6140	1.2146	0.7201	0.6395	0.7971	0.1410
RSU1	0.7304	0.8504	0.8833	0.7242	0.7971	0.0409
FAM90A3	0.6830	0.9191	0.8144	0.7720	0.7971	0.0490
SCARNA10	0.6656	0.8955	0.8787	0.7487	0.7971	0.0548
BID	0.7233	0.9370	0.8390	0.6895	0.7972	0.0565
RPS6KA1	0.7773	0.9437	0.7357	0.7328	0.7974	0.0498
BLVRA	0.6514	0.9828	0.9760	0.5799	0.7975	0.1060
SLC22A23	0.6701	0.9958	0.7911	0.7333	0.7976	0.0705
TYRO3	0.6951	1.0110	0.7857	0.6987	0.7976	0.0741
SLC37A4	0.6546	1.1979	0.7043	0.6338	0.7976	0.1342
CBX5	0.5325	1.0349	0.8275	0.7957	0.7977	0.1031
TOR3A	0.6078	1.4435	0.5596	0.5801	0.7977	0.2155
WDR21A	0.6814	0.9425	0.8675	0.6997	0.7978	0.0639
ALG9	0.5150	1.4040	0.6559	0.6164	0.7978	0.2042
DPY30	0.7078	0.8948	0.9091	0.6801	0.7980	0.0604
MAPK12	0.6456	1.0521	0.7938	0.7006	0.7980	0.0900
LOC648434	0.7666	0.8793	0.8485	0.6977	0.7980	0.0411
GPI	0.5898	1.2528	0.7151	0.6345	0.7980	0.1538
CTNNAL1	0.5989	1.0982	0.8032	0.6921	0.7981	0.1084
LOC1001301	0.7196	0.9801	0.8221	0.6713	0.7983	0.0683
MTHFD1	0.7179	0.9670	0.8209	0.6876	0.7983	0.0630
PARP12	0.6661	1.0497	0.7308	0.7470	0.7984	0.0856
SLC1A3	0.6241	0.9371	0.8024	0.8299	0.7984	0.0650
MTPN	0.5718	0.9821	0.9967	0.6430	0.7984	0.1113
FAM119B	0.7608	0.9645	0.7560	0.7124	0.7984	0.0564
ALG1	0.5645	1.1036	0.7814	0.7441	0.7984	0.1122
ATP2A2	0.6475	1.0860	0.7159	0.7445	0.7985	0.0980

C7orf26	0.7211	1.1481	0.7579	0.5669	0.7985	0.1237
MRPL48	0.6651	0.8869	1.0256	0.6169	0.7986	0.0958
CCDC137	0.6579	0.9843	0.8563	0.6961	0.7986	0.0753
RBX1	0.8340	0.9837	0.8549	0.5223	0.7987	0.0979
LOC1001337	0.8058	0.9330	0.7189	0.7371	0.7987	0.0485
DBNDD1	0.7902	0.9631	0.8551	0.5866	0.7988	0.0792
RIC8B	0.8203	0.8599	0.8063	0.7089	0.7989	0.0321
RAPGEF2	0.6922	0.9065	0.9095	0.6873	0.7989	0.0630
PFN1	0.6202	1.0691	0.8659	0.6407	0.7989	0.1059
ZDHHC24	0.6140	1.0235	0.9097	0.6487	0.7990	0.0998
AXIN1	0.6527	1.0370	0.8238	0.6824	0.7990	0.0877
LRRC40	0.6530	1.0254	0.8727	0.6452	0.7991	0.0921
LOC124512	0.7107	0.9451	0.9602	0.5803	0.7991	0.0926
SORBS3	0.7411	0.9348	0.7953	0.7251	0.7991	0.0477
VKORC1L1	0.6474	0.9381	0.9244	0.6865	0.7991	0.0768
SIX5	0.6643	1.0961	0.7261	0.7100	0.7991	0.0999
ESCO1	0.8089	0.8569	0.7565	0.7746	0.7992	0.0221
LRRCC1	0.6622	0.8994	0.8338	0.8017	0.7993	0.0500
ACTN1	0.7554	1.0044	0.7944	0.6435	0.7994	0.0754
TMED10P	0.6264	1.0432	0.9467	0.5813	0.7994	0.1150
MPHOSPH6	0.6327	1.0271	0.9505	0.5876	0.7995	0.1108
C6orf154	0.7064	0.9789	0.7696	0.7432	0.7995	0.0612
PRKACB	0.7088	1.0769	0.7750	0.6375	0.7995	0.0966
DNAJB1	0.8414	0.9878	0.7459	0.6236	0.7997	0.0769
CAPZA2	0.5142	0.9352	0.9546	0.7948	0.7997	0.1016
NUTF2	0.6455	1.0234	0.8915	0.6385	0.7998	0.0950
RHBDD1	0.7742	0.9488	0.7856	0.6906	0.7998	0.0540
C9orf114	0.5507	1.1414	0.8077	0.6996	0.7998	0.1254
VARS	0.6803	0.9794	0.8713	0.6685	0.7999	0.0758
BCL6	0.6947	0.9947	0.8666	0.6435	0.7999	0.0806
IPO7	0.5145	1.1667	0.8286	0.6898	0.7999	0.1381
SNRPF	0.5388	1.1335	0.8552	0.6723	0.7999	0.1287
MRPS23	0.6860	1.0648	0.8682	0.5813	0.8001	0.1063
LOC728229	0.7053	1.1298	0.7239	0.6419	0.8003	0.1113
NUCB2	0.7633	0.8168	1.0585	0.5627	0.8003	0.1020
CTDSP1	0.7042	1.0045	0.7298	0.7633	0.8004	0.0691
MRPL40	0.7496	0.9335	0.8832	0.6356	0.8005	0.0673
ARRB1	0.7123	0.9155	0.8266	0.7477	0.8005	0.0452
LOC643507	0.6315	1.0000	0.8887	0.6827	0.8007	0.0866
NFE2L3	0.7469	0.8605	0.9197	0.6760	0.8008	0.0549
LYPLA2P1	0.7324	0.9880	0.7708	0.7120	0.8008	0.0636
GTF2I	0.5866	1.1398	0.8074	0.6698	0.8009	0.1218
SAP30L	0.7497	0.9073	0.9230	0.6238	0.8010	0.0708
ALDH16A1	0.7051	1.0600	0.7219	0.7169	0.8010	0.0864
HADH2	0.7433	0.9086	0.8806	0.6716	0.8010	0.0563
COTL1	0.7040	0.9779	0.7731	0.7491	0.8010	0.0607

SPA17	0.6791	0.9553	0.9472	0.6226	0.8010	0.0875
NKRF	0.6265	0.9156	0.8960	0.7663	0.8011	0.0670
PRCC	0.6616	1.0594	0.7865	0.6975	0.8012	0.0900
RHOT2	0.7013	1.0258	0.7809	0.6970	0.8013	0.0773
MAGEA6	0.6870	1.0617	0.8052	0.6511	0.8013	0.0928
ATP5G2	0.7075	1.0812	0.8081	0.6084	0.8013	0.1018
ZNF696	0.6470	1.0325	0.8305	0.6956	0.8014	0.0863
UBXN2B	0.7123	0.9711	0.7519	0.7705	0.8015	0.0578
ODF2	0.7494	1.0192	0.7770	0.6602	0.8015	0.0767
COPS3	0.5997	0.9965	0.9382	0.6717	0.8015	0.0976
MFSD3	0.6821	1.0973	0.7990	0.6278	0.8016	0.1048
MTIF3	0.6028	1.0095	0.8864	0.7079	0.8017	0.0907
SOHLH2	0.7611	0.9807	0.8375	0.6274	0.8017	0.0738
DHRS11	0.7681	0.9321	0.7722	0.7344	0.8017	0.0443
SNORD68	0.6630	0.8992	0.8968	0.7480	0.8018	0.0582
HK2	0.6909	1.1379	0.7428	0.6361	0.8019	0.1141
PAPD1	0.6038	1.0208	0.9060	0.6772	0.8019	0.0973
STAG1	0.7531	0.8566	0.8938	0.7044	0.8020	0.0441
TSC22D3	0.7643	0.9999	0.7223	0.7215	0.8020	0.0667
METAP2	0.6183	1.0598	0.8634	0.6666	0.8020	0.1010
CDC42EP2	0.6433	1.1240	0.7302	0.7107	0.8020	0.1089
SNORA18	0.6363	1.1337	0.7278	0.7107	0.8021	0.1123
LOC1001295	0.6508	1.0626	0.7399	0.7554	0.8022	0.0898
DNAJC10	0.7463	0.9052	0.9198	0.6382	0.8024	0.0674
RINT1	0.6203	1.1057	0.8215	0.6622	0.8024	0.1100
THOP1	0.5890	1.0912	0.7758	0.7540	0.8025	0.1049
TARBP2	0.7215	0.9633	0.8350	0.6902	0.8025	0.0620
CSNK2B	0.7412	1.1038	0.8221	0.5430	0.8025	0.1163
WDR41	0.6526	1.0754	0.8219	0.6607	0.8026	0.0989
C16orf74	0.7386	0.9307	0.8041	0.7375	0.8027	0.0454
PACSIN3	0.8231	0.9533	0.7033	0.7313	0.8027	0.0563
ABHD5	0.7032	0.9037	0.9110	0.6931	0.8028	0.0604
DDX28	0.7841	0.9976	0.7683	0.6610	0.8028	0.0705
C3orf15	0.7180	0.9284	0.8193	0.7454	0.8028	0.0470
NIT2	0.6162	0.9785	0.8767	0.7399	0.8028	0.0791
LYSMD1	0.5595	1.0041	0.8912	0.7568	0.8029	0.0956
MRPL14	0.6870	1.0276	0.7862	0.7109	0.8029	0.0778
FAM69B	0.7359	0.9569	0.7997	0.7195	0.8030	0.0541
CD83	0.6980	1.0063	0.8222	0.6857	0.8030	0.0744
GCH1	0.6373	1.0173	0.8709	0.6869	0.8031	0.0873
EPCAM	0.7012	0.8718	1.0004	0.6396	0.8032	0.0820
NUMB	0.7514	1.0140	0.8372	0.6107	0.8033	0.0843
CDC123	0.6247	1.1867	0.7466	0.6553	0.8033	0.1304
RDBP	0.6506	0.9736	0.8546	0.7346	0.8034	0.0705
BRWD1	0.6803	0.9671	0.8345	0.7316	0.8034	0.0633
PIGM	0.7044	1.0510	0.7290	0.7293	0.8034	0.0827

C17orf106	0.6399	1.0707	0.8145	0.6887	0.8034	0.0964
DKC1	0.6013	1.1952	0.8281	0.5897	0.8036	0.1416
GRAMD1A	0.7160	1.1661	0.7194	0.6129	0.8036	0.1233
C6orf115	0.6729	0.9743	0.9393	0.6280	0.8036	0.0892
PSMA5	0.6864	0.9815	0.9286	0.6184	0.8037	0.0891
LOC643387	0.7271	0.8953	0.8938	0.6988	0.8038	0.0528
CLPX	0.6380	1.1902	0.7926	0.5942	0.8038	0.1357
GNPDA2	0.7021	1.0006	0.8002	0.7125	0.8038	0.0692
C9orf116	0.7426	1.0096	0.8246	0.6386	0.8039	0.0784
MTX2	0.6976	0.9172	0.9476	0.6535	0.8040	0.0749
GMPPA	0.6973	0.9969	0.8024	0.7195	0.8040	0.0682
ERI1	0.6507	1.0931	0.7716	0.7015	0.8042	0.0994
NFXL1	0.6176	0.9925	0.9347	0.6725	0.8043	0.0934
C11orf83	0.7332	1.0032	0.7890	0.6918	0.8043	0.0692
ZNF367	0.6554	1.0037	0.7481	0.8104	0.8044	0.0737
HNRPR	0.6739	1.0920	0.7291	0.7227	0.8044	0.0966
ZNF142	0.6166	1.1456	0.7604	0.6953	0.8045	0.1174
NUDCD2	0.6437	0.9150	1.1092	0.5503	0.8045	0.1276
KIAA0895	0.7092	1.0314	0.8048	0.6728	0.8046	0.0806
TMEM5	0.5599	1.0346	0.8920	0.7317	0.8046	0.1024
DCUN1D4	0.6418	1.0401	0.9118	0.6248	0.8046	0.1024
KIAA0020	0.7690	0.9998	0.8107	0.6397	0.8048	0.0745
TMEM186	0.8891	0.9039	0.7693	0.6571	0.8048	0.0577
GPR89C	0.6383	0.9870	0.9764	0.6177	0.8049	0.1022
ERAL1	0.6781	0.9945	0.8883	0.6587	0.8049	0.0818
LRRC3	0.7107	0.9519	0.8054	0.7522	0.8051	0.0527
LOC731878	0.6513	1.0434	0.8601	0.6655	0.8051	0.0926
AKT1S1	0.7084	1.0149	0.7237	0.7734	0.8051	0.0713
GBA	0.6297	1.0799	0.8146	0.6962	0.8051	0.0993
OBFC2A	0.4810	1.3014	0.8768	0.5612	0.8051	0.1862
BIVM	0.6969	0.9937	0.8054	0.7252	0.8053	0.0669
C11orf24	0.6849	0.9094	0.9278	0.6992	0.8053	0.0656
UBE2D3	0.6432	1.0692	0.8609	0.6479	0.8053	0.1016
CTSA	0.6363	1.0772	0.7791	0.7288	0.8054	0.0953
SIVA1	0.5865	1.1675	0.8004	0.6672	0.8054	0.1285
ZNF695	0.6746	0.9274	0.8344	0.7853	0.8054	0.0526
NAGPA	0.7326	0.9785	0.8239	0.6868	0.8054	0.0644
ASPH	0.7427	0.9266	0.8042	0.7483	0.8054	0.0427
TRAM2	0.7570	1.0063	0.7596	0.6988	0.8054	0.0684
TIGD7	0.6697	1.0873	0.7701	0.6948	0.8055	0.0963
C14orf129	0.6431	1.0572	0.8016	0.7207	0.8056	0.0899
EXOSC10	0.6762	1.1270	0.7700	0.6497	0.8057	0.1102
UHRF1BP1	0.6690	0.9549	0.8576	0.7415	0.8057	0.0631
ADAL	0.6968	0.9909	0.8100	0.7254	0.8058	0.0662
CHRAC1	0.7378	0.9177	0.7836	0.7840	0.8058	0.0388
LOC728026	0.7020	0.8694	0.9751	0.6766	0.8058	0.0708

LOC653156	0.2427	1.1476	1.2614	0.5714	0.8058	0.2409
C14orf142	0.7284	0.7755	0.9508	0.7692	0.8060	0.0494
POLRMT	0.6435	1.0178	0.7741	0.7885	0.8060	0.0778
LOC1001343	0.6741	1.0416	0.8296	0.6787	0.8060	0.0864
PTDSS2	0.6156	1.1141	0.8716	0.6232	0.8061	0.1186
MCRS1	0.7026	0.9739	0.8128	0.7353	0.8061	0.0605
MOSC2	0.5553	1.0926	0.8714	0.7058	0.8063	0.1152
LOC727900	0.6472	1.0870	0.7683	0.7230	0.8064	0.0968
SPIN1	0.6444	0.8966	0.8742	0.8104	0.8064	0.0570
TMEM191B	0.7241	1.0248	0.7869	0.6900	0.8065	0.0755
PTMS	0.7147	1.0946	0.7108	0.7065	0.8066	0.0960
DHRS3	0.7260	0.9329	0.8305	0.7371	0.8066	0.0482
ESD	0.6912	0.9983	0.8573	0.6800	0.8067	0.0757
SMARCA2	0.7539	0.9823	0.8978	0.5929	0.8067	0.0855
INPP5E	0.7290	1.0296	0.7402	0.7282	0.8067	0.0743
LOC284167	0.6562	1.1204	0.7894	0.6610	0.8067	0.1090
PRSS8	0.6917	1.0348	0.8322	0.6686	0.8068	0.0841
LOC649821	0.6033	1.1070	0.9102	0.6067	0.8068	0.1232
POLS	0.7209	0.9409	0.8361	0.7295	0.8069	0.0518
XRCC5	0.6548	1.0725	0.8855	0.6147	0.8069	0.1068
HNRNPH3	0.6049	1.0093	0.9696	0.6441	0.8070	0.1060
LOC340260	0.6843	1.0574	0.7527	0.7336	0.8070	0.0847
GTF3A	0.5634	1.1048	0.9448	0.6156	0.8071	0.1303
FAM86A	0.5799	1.0959	0.9247	0.6281	0.8071	0.1228
ABHD2	0.6759	1.1215	0.7952	0.6363	0.8072	0.1101
SMARCD1	0.7602	0.9858	0.7759	0.7069	0.8072	0.0613
TMEM69	0.5770	1.2122	0.7774	0.6626	0.8073	0.1411
USP13	0.6280	0.9837	0.9107	0.7067	0.8073	0.0837
CLPP	0.7236	1.1608	0.7250	0.6198	0.8073	0.1204
UNC84A	0.6928	1.0368	0.7942	0.7061	0.8075	0.0797
LOC729057	0.6725	0.9636	0.8575	0.7363	0.8075	0.0647
RMI1	0.7424	0.9919	0.8690	0.6270	0.8076	0.0788
SF3B4	0.6445	1.0338	0.8929	0.6592	0.8076	0.0945
C6orf162	0.8101	0.8857	0.7927	0.7418	0.8076	0.0298
LGALS3BP	0.7030	1.0060	0.9247	0.5966	0.8076	0.0951
PET112L	0.7335	1.0450	0.8135	0.6385	0.8076	0.0868
THOC3	0.7105	1.0666	0.8014	0.6522	0.8077	0.0916
HSBP1	0.7616	1.1353	0.7199	0.6143	0.8078	0.1135
MLKL	0.6854	0.9924	0.8185	0.7349	0.8078	0.0674
DHX9	0.6967	1.1152	0.7810	0.6385	0.8078	0.1065
MRPL21	0.6528	1.0325	0.8591	0.6874	0.8080	0.0874
NEK6	0.6589	0.9933	0.8309	0.7488	0.8080	0.0711
ZNF746	0.7270	1.0846	0.7475	0.6733	0.8081	0.0935
SEMA4F	0.6532	1.0526	0.6571	0.8697	0.8081	0.0959
KHDRBS1	0.6385	1.1155	0.8858	0.5928	0.8082	0.1210
LANCL1	0.7632	0.9368	0.8471	0.6856	0.8082	0.0541

ZNF259	0.6157	1.0855	0.8737	0.6579	0.8082	0.1083
XYLB	0.7368	0.9231	0.7990	0.7741	0.8082	0.0404
MTE	0.7192	1.0035	0.8033	0.7076	0.8084	0.0684
ZNF643	0.7320	0.8780	0.8506	0.7730	0.8084	0.0338
FANCB	0.6370	0.9367	0.8103	0.8498	0.8084	0.0630
CDH26	0.7787	0.9342	0.7842	0.7368	0.8085	0.0432
CCDC117	0.6052	1.2403	0.7509	0.6376	0.8085	0.1473
LOC1001334	0.6498	1.0329	0.8278	0.7235	0.8085	0.0832
VPS54	0.7280	0.9433	0.7882	0.7745	0.8085	0.0467
ZNF7	0.7517	1.0332	0.7407	0.7085	0.8085	0.0755
ARFGEF2	0.7188	0.9685	0.7980	0.7488	0.8085	0.0558
LOC1001331	0.6004	1.0812	0.8825	0.6706	0.8087	0.1088
S100A6	0.5732	1.2507	0.5200	0.8911	0.8088	0.1685
ANAPC11	0.7255	1.0656	0.7828	0.6613	0.8088	0.0891
MKI67IP	0.5913	1.0164	0.9587	0.6695	0.8090	0.1050
LOC440093	0.6260	1.1116	0.8651	0.6332	0.8090	0.1151
C6orf120	0.7650	0.8071	0.9536	0.7103	0.8090	0.0521
LOC653752	0.6375	0.9587	0.8471	0.7930	0.8091	0.0668
CDK5RAP1	0.6943	1.0525	0.8131	0.6765	0.8091	0.0866
TRIM28	0.8317	0.9624	0.7590	0.6835	0.8091	0.0594
RAB36	0.7313	1.0697	0.8131	0.6225	0.8092	0.0952
RFC1	0.7122	1.0841	0.7847	0.6557	0.8092	0.0954
MDK	0.7653	0.9879	0.7661	0.7176	0.8092	0.0606
YME1L1	0.6882	0.9956	0.8102	0.7430	0.8092	0.0669
UGCGL1	0.6502	0.9731	0.8172	0.7965	0.8092	0.0661
CCDC28B	0.7008	1.0070	0.8078	0.7213	0.8093	0.0699
PHF16	0.6507	0.9421	0.9057	0.7386	0.8093	0.0690
AFF3	0.6872	0.9289	0.8853	0.7358	0.8093	0.0580
KRI1	0.7014	0.9073	0.8554	0.7731	0.8093	0.0453
METAP1	0.7225	1.1708	0.7907	0.5533	0.8093	0.1304
OSCP1	0.6864	0.9911	0.8897	0.6702	0.8094	0.0785
GTF2E2	0.7135	1.0362	0.8513	0.6366	0.8094	0.0877
PRPF4	0.6263	0.9695	0.9159	0.7260	0.8094	0.0803
TBCE	0.6437	1.0178	0.8708	0.7056	0.8095	0.0844
CNIH	0.5874	1.0914	0.9064	0.6530	0.8095	0.1164
RICH2	0.7190	1.0012	0.7868	0.7313	0.8096	0.0656
OSTC	0.6755	1.0481	0.8815	0.6341	0.8098	0.0961
GMPPB	0.6404	1.0818	0.8799	0.6372	0.8098	0.1070
PPP1R11	0.7365	1.0387	0.8338	0.6304	0.8099	0.0869
POMZP3	0.7215	0.9997	0.8632	0.6552	0.8099	0.0767
SPNS1	0.7474	1.0986	0.7344	0.6595	0.8100	0.0981
MAEA	0.5811	1.1637	0.8214	0.6742	0.8101	0.1278
LOC286016	0.5689	1.2484	0.7738	0.6496	0.8102	0.1520
ADAT1	0.6426	1.0891	0.8469	0.6622	0.8102	0.1037
FCHSD2	0.6479	0.8647	0.9703	0.7582	0.8103	0.0693
PPP2R5C	0.6646	1.0726	0.8354	0.6686	0.8103	0.0961

MRPL50	0.6050	0.8988	1.0000	0.7377	0.8104	0.0872
AOF2	0.6344	1.1238	0.8190	0.6657	0.8107	0.1119
PIK3C2B	0.7037	1.0403	0.7749	0.7239	0.8107	0.0780
LOC439953	0.7413	0.9069	0.8761	0.7187	0.8108	0.0473
TMEM39A	0.7627	0.9591	0.7939	0.7274	0.8108	0.0513
S100A4	0.7024	0.8720	0.8551	0.8140	0.8109	0.0381
XPO4	0.7204	1.0137	0.7804	0.7293	0.8109	0.0689
CXXC1	0.7023	1.0498	0.7616	0.7303	0.8110	0.0805
FAM83F	0.6763	0.9276	0.8578	0.7827	0.8111	0.0538
LRRC42	0.6769	1.0078	0.8754	0.6844	0.8111	0.0801
BAMBI	0.7020	0.8899	0.9875	0.6652	0.8111	0.0767
PTS	0.6870	1.0208	0.9141	0.6228	0.8112	0.0937
TRIM11	0.6490	1.0897	0.7649	0.7413	0.8112	0.0961
C19orf25	0.6877	0.9780	0.8209	0.7584	0.8113	0.0619
ABCB9	0.7005	1.0058	0.8534	0.6854	0.8113	0.0751
NCRNA0009‹	0.7305	1.0185	0.8379	0.6582	0.8113	0.0783
PSMC3	0.6053	1.1325	0.8602	0.6472	0.8113	0.1207
RAD51	0.7178	0.9839	0.8065	0.7377	0.8115	0.0605
SSSCA1	0.6007	1.0492	0.8565	0.7398	0.8115	0.0949
NDUFA10	0.6415	1.1313	0.8322	0.6412	0.8116	0.1157
CWC15	0.6592	1.0025	0.9074	0.6772	0.8116	0.0851
TARS	0.7770	0.9059	1.0356	0.5280	0.8116	0.1083
IRAK1	0.7738	1.0470	0.7759	0.6498	0.8116	0.0838
ZFAND2B	0.7400	0.9036	0.8622	0.7407	0.8116	0.0420
LOC653604	0.6192	0.9617	0.8769	0.7892	0.8117	0.0732
TIPIN	0.6154	0.9619	0.9409	0.7288	0.8117	0.0840
RDM1	0.6942	1.0360	0.8255	0.6912	0.8117	0.0811
LOC729200	0.6631	0.9373	1.0482	0.5985	0.8118	0.1077
C2orf7	0.6996	0.9879	0.9842	0.5754	0.8118	0.1038
CXorf56	0.7205	0.9752	0.8495	0.7022	0.8119	0.0636
GMPR2	0.7729	0.8944	0.8546	0.7258	0.8119	0.0383
REXO4	0.5743	1.1709	0.9548	0.5476	0.8119	0.1515
KIFC2	0.6219	1.2915	0.7184	0.6163	0.8120	0.1615
GPT2	0.7424	1.2903	0.7007	0.5150	0.8121	0.1669
SIKE	0.6578	1.0219	0.8262	0.7425	0.8121	0.0779
PPM1G	0.6363	1.1333	0.8634	0.6156	0.8121	0.1209
CDC26	0.6041	1.1986	0.8308	0.6152	0.8122	0.1390
YBX1	0.6331	0.9949	1.0102	0.6106	0.8122	0.1101
ARFIP1	0.7320	0.9751	0.8730	0.6689	0.8122	0.0690
C22orf40	0.7375	1.0103	0.7809	0.7203	0.8122	0.0672
LSM7	0.6376	1.1247	0.8424	0.6443	0.8122	0.1145
LOC1001326	0.6898	0.9438	0.9942	0.6211	0.8122	0.0922
LHX2	0.7090	1.0489	0.8114	0.6800	0.8123	0.0837
MRPS34	0.7252	1.0797	0.7387	0.7063	0.8125	0.0893
ARPC5L	0.5671	1.2920	0.7397	0.6514	0.8125	0.1636
ROGDI	0.7033	0.9857	0.7942	0.7670	0.8126	0.0608

HMGN4	0.7255	1.0747	0.8218	0.6283	0.8126	0.0959
LOC729608	0.5940	1.0153	0.9744	0.6666	0.8126	0.1066
NAGLU	0.8696	1.0602	0.7278	0.5933	0.8127	0.0999
LOC644330	0.5826	1.2466	0.7819	0.6402	0.8128	0.1505
SLC2A4RG	0.6810	1.0403	0.8082	0.7219	0.8129	0.0803
CXorf26	0.6952	0.9158	0.9392	0.7019	0.8130	0.0663
NANS	0.7056	1.0593	0.8798	0.6080	0.8132	0.0995
FADS1	0.6497	1.0878	0.8654	0.6501	0.8132	0.1047
PRDX1	0.6839	1.1245	0.8668	0.5778	0.8133	0.1197
DYNLT1	0.8522	1.0487	0.7585	0.5937	0.8133	0.0949
NOC2L	0.6808	1.0020	0.8398	0.7306	0.8133	0.0711
OBFC2B	0.7380	1.1036	0.7250	0.6867	0.8133	0.0974
LOC1001291	0.6638	0.8842	1.0314	0.6740	0.8134	0.0887
AFG3L2	0.6407	1.2655	0.7412	0.6061	0.8134	0.1534
MASTL	0.7442	0.9451	0.7346	0.8297	0.8134	0.0488
C13orf23	0.7040	1.0617	0.7760	0.7119	0.8134	0.0843
DMTF1	0.7308	0.9756	0.8188	0.7289	0.8135	0.0580
C2orf25	0.6391	1.0146	0.9135	0.6870	0.8135	0.0898
DBP	0.7623	0.9480	0.8896	0.6548	0.8137	0.0656
PGS1	0.7227	0.9456	0.9132	0.6737	0.8138	0.0678
COASY	0.8133	1.0239	0.7783	0.6400	0.8139	0.0794
SPIRE2	0.8025	0.9680	0.7131	0.7720	0.8139	0.0546
IFITM1	0.7877	0.8625	0.9025	0.7030	0.8139	0.0440
SEC11C	0.6835	1.0002	0.9876	0.5845	0.8140	0.1059
GPR175	0.7232	0.9509	0.8547	0.7272	0.8140	0.0549
UBE2N	0.8204	0.9424	0.9509	0.5424	0.8140	0.0953
TOP3A	0.7014	0.9818	0.8341	0.7390	0.8141	0.0625
ZNF74	0.7948	0.9632	0.8172	0.6813	0.8141	0.0579
ATP1B3	0.6609	0.9665	1.0289	0.6001	0.8141	0.1075
PSMG3	0.6686	1.2170	0.7251	0.6458	0.8141	0.1353
C9orf142	0.6167	1.1855	0.7980	0.6565	0.8142	0.1297
FLJ22639	0.6564	0.9758	0.7698	0.8547	0.8142	0.0675
PDHB	0.7917	0.9900	0.9496	0.5256	0.8142	0.1053
FBLN2	0.7492	0.9711	0.7843	0.7523	0.8142	0.0529
EIF2S2	0.7749	0.9267	0.8028	0.7525	0.8142	0.0389
PDK3	0.6899	1.0874	0.8427	0.6372	0.8143	0.1009
LOC387882	0.6145	1.1262	0.8573	0.6592	0.8143	0.1166
GAGE8	0.8083	0.8251	1.0840	0.5399	0.8143	0.1111
LOC441408	0.8956	0.8985	0.8370	0.6264	0.8144	0.0642
ZIC2	0.5170	1.2140	0.8319	0.6951	0.8145	0.1479
TOMM40L	0.6598	0.9808	0.8842	0.7332	0.8145	0.0725
TMEM188	0.6933	1.0282	0.8573	0.6794	0.8145	0.0819
KIAA0892	0.7658	0.9597	0.7505	0.7825	0.8146	0.0488
SP8	0.6923	0.9777	0.8707	0.7180	0.8147	0.0671
RNF187	0.7024	1.1137	0.6999	0.7428	0.8147	0.1002
HPS5	0.6910	0.9988	0.8843	0.6848	0.8147	0.0769

KIAA0564	0.7504	0.9325	0.8190	0.7569	0.8147	0.0422
POGK	0.6844	1.0587	0.8295	0.6863	0.8147	0.0881
FKSG30	0.6611	1.1075	0.9049	0.5855	0.8147	0.1190
CDKN2A	0.7571	0.9535	0.8249	0.7236	0.8148	0.0508
TRA2A	0.6807	1.1442	0.8230	0.6112	0.8148	0.1183
RSBN1L	0.7198	1.0625	0.9236	0.5536	0.8149	0.1120
DIMT1L	0.6074	1.0136	0.9236	0.7150	0.8149	0.0932
ZNF252	0.7742	1.1552	0.6954	0.6350	0.8149	0.1169
VPS37A	0.6361	0.9480	1.0453	0.6311	0.8151	0.1067
NUP107	0.6744	1.1585	0.7966	0.6310	0.8151	0.1197
FIBP	0.6496	1.0597	0.8292	0.7220	0.8151	0.0895
RFXAP	0.7009	1.0220	0.8063	0.7315	0.8152	0.0724
STARD8	0.8431	0.7345	0.9095	0.7738	0.8152	0.0386
API5	0.6747	1.0501	0.8458	0.6906	0.8153	0.0873
HNRNPF	0.7604	0.9631	0.7603	0.7773	0.8153	0.0494
CHAC1	0.9088	0.8128	0.7642	0.7757	0.8154	0.0328
LOC641844	0.4825	1.3058	0.9228	0.5504	0.8154	0.1900
HNRPUL2	0.7746	0.9584	0.7818	0.7468	0.8154	0.0483
TXN	0.6988	1.0781	0.8846	0.6002	0.8154	0.1056
RCC2	0.5803	1.3476	0.7233	0.6106	0.8154	0.1800
SUPT16H	0.6590	1.1021	0.8153	0.6860	0.8156	0.1014
COX5A	0.6745	0.8858	0.9582	0.7439	0.8156	0.0648
PLSCR3	0.8289	1.1128	0.6753	0.6455	0.8156	0.1069
LSM8	0.6314	1.1213	0.7786	0.7313	0.8156	0.1064
PLEKHJ1	0.6440	0.8842	1.0589	0.6755	0.8157	0.0970
SMYD4	0.7598	0.9377	0.8771	0.6885	0.8158	0.0562
ANAPC7	0.6109	1.1879	0.7772	0.6870	0.8158	0.1286
KAZALD1	0.6457	0.8994	0.8301	0.8880	0.8158	0.0587
GRSF1	0.5950	1.1129	0.7742	0.7814	0.8159	0.1080
TCEAL8	0.6906	1.0817	0.8581	0.6331	0.8159	0.1006
DHX36	0.6895	0.9941	0.9170	0.6631	0.8159	0.0823
RING1	0.7581	0.9788	0.7953	0.7317	0.8160	0.0558
FAM175B	0.6523	1.0058	0.9022	0.7037	0.8160	0.0831
PAFAH2	0.6342	1.0499	0.7898	0.7902	0.8160	0.0862
LOC647481	0.6963	1.0078	0.7996	0.7606	0.8161	0.0674
GNL1	0.7396	1.0921	0.7593	0.6734	0.8161	0.0938
MYD88	0.6764	1.2041	0.7590	0.6250	0.8161	0.1322
C20orf3	0.7049	1.2278	0.7245	0.6074	0.8162	0.1396
C1orf124	0.6693	1.0731	0.8342	0.6880	0.8162	0.0932
ZNF480	0.6272	1.0744	0.8596	0.7040	0.8163	0.0987
SNORD83B	0.7373	0.9489	0.8519	0.7272	0.8163	0.0525
LOC653383	0.7797	1.1000	0.7799	0.6061	0.8164	0.1030
HAUS4	0.7289	1.0967	0.7505	0.6897	0.8165	0.0943
B9D1	0.7170	0.9254	0.8627	0.7608	0.8165	0.0474
UBXN2A	0.6539	0.9838	0.8576	0.7707	0.8165	0.0696
PSMA3	0.6905	1.0157	0.9125	0.6475	0.8165	0.0882

PXMP2	0.7162	1.0422	0.8084	0.7001	0.8167	0.0789
RBM27	0.7594	0.9685	0.8332	0.7058	0.8167	0.0569
EFNB3	0.8132	1.0303	0.7288	0.6948	0.8168	0.0754
GTF2H3	0.6921	1.0243	0.9037	0.6471	0.8168	0.0890
SAMM50	0.6037	1.1688	0.8234	0.6714	0.8168	0.1260
RMND5A	0.7219	1.0982	0.6723	0.7751	0.8168	0.0961
C2orf15	0.6117	1.0424	0.9141	0.6992	0.8169	0.0984
FAM136B	0.7630	0.8375	0.8865	0.7805	0.8169	0.0281
TMEM168	0.5388	1.2658	0.8033	0.6595	0.8169	0.1591
SOX13	0.7837	0.9709	0.8180	0.6949	0.8169	0.0575
LOC345645	0.6826	0.9266	0.9528	0.7056	0.8169	0.0713
ADCK1	0.8128	0.8989	0.7995	0.7564	0.8169	0.0299
WDR82	0.7219	0.9038	1.0300	0.6120	0.8169	0.0931
C10orf61	0.7048	1.0419	0.8521	0.6690	0.8169	0.0848
LOC644063	0.5910	1.2007	0.8097	0.6669	0.8171	0.1357
SNAPC2	0.7511	1.0268	0.8232	0.6672	0.8171	0.0768
RNF113A	0.6304	0.9840	1.0070	0.6470	0.8171	0.1032
LOC654244	0.6734	1.0065	0.9809	0.6076	0.8171	0.1030
COX7B	0.7394	0.9161	1.0583	0.5549	0.8172	0.1091
LOC644096	0.7175	1.0173	0.7860	0.7482	0.8172	0.0681
OCIAD2	0.8422	1.0137	0.8077	0.6054	0.8173	0.0838
FAM86C	0.6524	1.0763	0.8335	0.7071	0.8173	0.0943
RAB3IL1	0.6261	1.1917	0.8242	0.6272	0.8173	0.1332
TBX1	0.6989	0.9829	0.8772	0.7105	0.8174	0.0686
ATG4B	0.6475	1.1172	0.8343	0.6707	0.8174	0.1082
LOC441714	0.7146	1.0059	0.8598	0.6894	0.8174	0.0732
HSPC171	0.7190	1.0482	0.8481	0.6546	0.8175	0.0868
NIN	0.7367	1.0242	0.7448	0.7646	0.8176	0.0691
EPHB4	0.7456	1.0595	0.7182	0.7472	0.8176	0.0809
RP11-529I10	0.8400	1.0168	0.7381	0.6757	0.8176	0.0745
ORC6L	0.6769	1.0923	0.8653	0.6360	0.8176	0.1043
METTL13	0.6785	1.0895	0.8100	0.6925	0.8176	0.0953
ISCA1L	0.7028	0.9673	1.0167	0.5840	0.8177	0.1040
LOC1001338	0.7518	1.0468	0.7567	0.7157	0.8177	0.0769
NSUN2	0.6094	1.0363	0.8502	0.7751	0.8177	0.0885
BET1	0.7459	1.0481	0.7673	0.7097	0.8177	0.0777
DBN1	0.6836	1.0392	0.8444	0.7039	0.8178	0.0820
DHRS4	0.6568	1.2177	0.7681	0.6286	0.8178	0.1367
RHOG	0.6804	1.0050	0.8632	0.7227	0.8178	0.0736
KIAA1712	0.7070	0.9823	0.8581	0.7241	0.8179	0.0644
C5orf37	0.7947	1.0260	0.7970	0.6539	0.8179	0.0770
PDCD2	0.6951	1.0230	0.8733	0.6801	0.8179	0.0813
CHD1	0.6238	0.9680	1.0347	0.6453	0.8179	0.1069
CCDC94	0.7700	0.8608	0.9075	0.7336	0.8180	0.0401
MCM8	0.6347	1.0538	0.8332	0.7501	0.8180	0.0885
HSPBL2	0.6196	1.1379	0.9016	0.6130	0.8180	0.1261

C16orf58	0.8126	0.9970	0.8171	0.6459	0.8181	0.0717
CTH	0.8174	0.9617	0.7641	0.7299	0.8183	0.0511
SAE1	0.6283	1.0712	0.8872	0.6868	0.8184	0.1009
CERK	0.7530	0.9712	0.8362	0.7132	0.8184	0.0570
RTCD1	0.6995	0.9648	0.9767	0.6329	0.8185	0.0890
GRPEL1	0.7290	1.0534	0.7991	0.6926	0.8185	0.0814
CD58	0.6865	0.8719	0.9948	0.7215	0.8187	0.0712
METRNL	0.7778	0.9345	0.8089	0.7536	0.8187	0.0402
MED8	0.7312	1.0519	0.7857	0.7060	0.8187	0.0795
ACOT2	0.7043	1.1134	0.8354	0.6221	0.8188	0.1076
ACTR3	0.6854	0.8848	1.0959	0.6093	0.8189	0.1091
FAM104B	0.7898	0.8895	0.8368	0.7594	0.8189	0.0284
NLN	0.6230	1.1016	0.8739	0.6772	0.8189	0.1086
CEP152	0.6721	0.9579	0.8984	0.7476	0.8190	0.0660
RAB23	0.6769	0.9505	0.9048	0.7438	0.8190	0.0649
DYM	0.6582	1.1847	0.7816	0.6516	0.8190	0.1255
CCDC125	0.8207	0.8991	0.9224	0.6342	0.8191	0.0654
ARS2	0.6371	1.2647	0.7813	0.5933	0.8191	0.1539
MUTED	0.7540	0.9575	0.8674	0.6975	0.8191	0.0581
FBXL10	0.6553	1.0198	0.8789	0.7226	0.8191	0.0816
DACT3	0.6986	1.0587	0.7782	0.7412	0.8192	0.0815
PIGF	0.7667	0.9808	0.8480	0.6812	0.8192	0.0637
ZNF232	0.6762	1.0170	0.9190	0.6646	0.8192	0.0882
CBY1	0.6715	1.0663	0.8098	0.7293	0.8192	0.0871
CCDC15	0.6989	1.0271	0.8234	0.7280	0.8193	0.0742
IRX5	0.6522	1.0407	0.9410	0.6436	0.8194	0.1011
COX4NB	0.7215	1.0317	0.8400	0.6845	0.8194	0.0781
FLJ20125	0.6193	1.0692	0.8626	0.7265	0.8194	0.0970
TRMT11	0.6875	0.9704	0.8697	0.7503	0.8195	0.0629
CARD10	0.5974	1.0313	0.8720	0.7774	0.8195	0.0907
LOC644254	0.7689	0.8872	0.8370	0.7851	0.8195	0.0268
DCP1A	0.7410	1.0943	0.7035	0.7396	0.8196	0.0920
LOC388564	0.6650	1.1801	0.7925	0.6410	0.8196	0.1247
AVEN	0.7014	0.8944	0.9576	0.7252	0.8197	0.0629
MEMO1	0.7629	1.0068	0.8203	0.6887	0.8197	0.0679
NLE1	0.7453	0.9387	0.8830	0.7117	0.8197	0.0543
HMGN2	0.6666	0.9689	0.9218	0.7214	0.8197	0.0740
STAM	0.6674	1.0885	0.8741	0.6487	0.8197	0.1031
XPO7	0.6646	1.0211	0.7945	0.7987	0.8197	0.0740
DNAJB11	0.7300	1.1457	0.7296	0.6736	0.8197	0.1094
PRRC1	0.8225	1.0712	0.8822	0.5030	0.8197	0.1181
LOC643988	0.7642	0.8728	0.9272	0.7148	0.8197	0.0487
VPS33B	0.8567	1.0471	0.7667	0.6086	0.8198	0.0915
LOC728263	0.7287	0.9296	0.8971	0.7241	0.8199	0.0544
SFT2D3	0.6939	1.0460	0.8270	0.7127	0.8199	0.0809
MRPL52	0.6791	1.1000	0.8788	0.6217	0.8199	0.1084

C2orf56	0.6085	1.0829	0.9122	0.6761	0.8199	0.1092
NDUFS3	0.6433	1.0228	0.9066	0.7071	0.8199	0.0878
TADA1L	0.7146	1.0009	0.9237	0.6407	0.8200	0.0850
NT5M	0.7199	0.9067	0.8400	0.8133	0.8200	0.0387
GLCE	0.7254	1.0347	0.8232	0.6967	0.8200	0.0765
TM9SF4	0.7183	1.1198	0.7345	0.7076	0.8200	0.1001
MARK1	0.7723	0.9766	0.8354	0.6960	0.8201	0.0595
ABCF2	0.6292	1.1530	0.8066	0.6917	0.8201	0.1169
LOC389049	0.7027	1.0625	0.8390	0.6764	0.8202	0.0883
ZDHHC13	0.6770	1.0491	0.8583	0.6964	0.8202	0.0865
PPP2R2C	0.7991	0.9844	0.7572	0.7402	0.8202	0.0561
RHOA	0.7032	0.9878	1.0208	0.5697	0.8204	0.1098
LOC441150	0.8518	0.9764	0.7829	0.6707	0.8205	0.0640
LOC729375	0.6527	1.0437	0.9048	0.6808	0.8205	0.0934
SMARCB1	0.6566	1.1244	0.7977	0.7032	0.8205	0.1055
SNAP29	0.7525	1.1086	0.7705	0.6505	0.8205	0.0996
TCEA3	0.6484	1.0830	0.8742	0.6765	0.8205	0.1009
TMTC4	0.6523	1.0322	0.8614	0.7364	0.8206	0.0826
MAP4K5	0.7696	0.9604	0.8362	0.7163	0.8206	0.0527
ILF3	0.6358	1.1592	0.8134	0.6743	0.8207	0.1191
GATAD2A	0.7155	1.1691	0.7844	0.6138	0.8207	0.1213
LZTR1	0.6949	1.0796	0.8307	0.6778	0.8208	0.0928
H2AFZ	0.7209	1.0603	0.8768	0.6254	0.8208	0.0952
MRPL51	0.7233	1.0176	0.9280	0.6147	0.8209	0.0923
MTMR15	0.7518	1.0719	0.7300	0.7299	0.8209	0.0838
ZNF559	0.8375	0.9471	0.8441	0.6552	0.8210	0.0607
LOC1001298	0.7522	0.8082	1.0687	0.6551	0.8210	0.0884
C1QL4	0.8055	0.9172	0.7715	0.7901	0.8211	0.0328
AVL9	0.7404	0.9996	0.8727	0.6717	0.8211	0.0727
DRAM1	0.6961	0.9856	0.8065	0.7962	0.8211	0.0602
CHKA	0.6994	1.0347	0.8415	0.7088	0.8211	0.0783
RHOF	0.8593	0.9257	0.9384	0.5612	0.8211	0.0884
LOC642033	0.6105	1.0351	0.9451	0.6939	0.8211	0.1007
EAF1	0.7483	0.8903	1.0124	0.6336	0.8212	0.0826
GARNL4	0.7411	1.0392	0.7782	0.7263	0.8212	0.0735
LOC1001309	0.7073	1.2098	0.7226	0.6451	0.8212	0.1306
RIPK5	0.7316	0.9582	0.8229	0.7721	0.8212	0.0493
THG1L	0.6415	1.1296	0.8486	0.6655	0.8213	0.1127
GPHN	0.6046	1.0878	0.8370	0.7560	0.8214	0.1010
ZDHHC17	0.9138	0.8466	0.7547	0.7704	0.8214	0.0368
WDR68	0.6615	1.0140	0.9506	0.6597	0.8214	0.0938
LOC441191	0.6609	1.0774	0.8000	0.7477	0.8215	0.0900
ASTE1	0.7666	0.9283	0.8892	0.7020	0.8215	0.0527
SFRS13A	0.6739	1.0515	0.8914	0.6696	0.8216	0.0925
JUB	0.7256	0.9552	0.8135	0.7922	0.8216	0.0483
CTNNBL1	0.6846	1.1636	0.7505	0.6878	0.8216	0.1150

FAM44B	0.7962	1.0224	0.8364	0.6315	0.8216	0.0803
LOC1001322	0.6798	1.1404	0.8178	0.6490	0.8218	0.1124
LOC730167	0.5588	1.0888	1.0007	0.6387	0.8218	0.1310
C9orf9	0.7532	1.0863	0.7702	0.6775	0.8218	0.0904
FANCC	0.7761	0.8680	0.9560	0.6873	0.8219	0.0580
ATG4C	0.6409	1.0803	0.7982	0.7681	0.8219	0.0926
C19orf60	0.6737	1.1901	0.7852	0.6385	0.8219	0.1267
DHX29	0.7144	1.0010	0.8772	0.6951	0.8219	0.0723
SNORA76	0.6097	0.8690	0.8437	0.9655	0.8220	0.0755
ENTPD7	0.6661	1.0145	0.8766	0.7308	0.8220	0.0778
CNOT8	0.7381	0.9694	0.9176	0.6630	0.8220	0.0726
PSMC5	0.7751	0.9181	1.0145	0.5806	0.8221	0.0943
DGCR8	0.6762	1.1744	0.8436	0.5940	0.8221	0.1284
KIF1A	0.7095	1.1491	0.7698	0.6601	0.8221	0.1113
ZMYM1	0.7476	1.0116	0.8616	0.6678	0.8221	0.0746
RBMX2	0.7792	0.9173	0.9545	0.6377	0.8222	0.0721
PRR3	0.6810	0.9632	0.8858	0.7588	0.8222	0.0632
NASP	0.6750	1.0817	0.8115	0.7206	0.8222	0.0910
FHOD1	0.8080	1.0169	0.7688	0.6952	0.8222	0.0690
SNRPB2	0.6813	1.0943	0.8794	0.6339	0.8222	0.1051
C17orf81	0.7566	1.0875	0.7917	0.6531	0.8222	0.0932
ATIC	0.6621	1.1712	0.8848	0.5713	0.8224	0.1336
PDGFRL	0.7769	1.0559	0.7199	0.7369	0.8224	0.0787
CCDC71	0.7486	1.0401	0.7471	0.7539	0.8224	0.0726
NSUN5B	0.6870	0.9925	0.9084	0.7020	0.8225	0.0759
BCAS4	0.6957	1.0069	0.8340	0.7533	0.8225	0.0677
HNRPDL	0.7118	1.1041	0.8061	0.6680	0.8225	0.0982
SGK3	0.7048	0.9606	0.8345	0.7902	0.8225	0.0533
PSMC1	0.6173	1.0518	0.8819	0.7394	0.8226	0.0936
DNAJC22	0.6884	1.0867	0.7852	0.7303	0.8226	0.0902
CENPO	0.6525	0.9188	0.8613	0.8579	0.8226	0.0584
SDCCAG10	0.7172	0.9581	0.9320	0.6833	0.8227	0.0712
MAP2K6	0.7162	0.9970	0.8233	0.7542	0.8227	0.0622
TMEM149	0.6739	1.0138	0.7481	0.8550	0.8227	0.0738
SMARCC1	0.8075	0.9840	0.8366	0.6629	0.8228	0.0658
LOC729647	0.7423	0.9673	0.8782	0.7034	0.8228	0.0610
PPRC1	0.5500	1.2000	0.8789	0.6628	0.8229	0.1430
UGT2B7	0.5773	1.1547	0.9563	0.6038	0.8230	0.1403
C9orf41	0.7501	0.9343	0.8697	0.7379	0.8230	0.0475
MBD3	0.7837	1.0015	0.8060	0.7011	0.8231	0.0636
MMP11	0.8005	0.9542	0.7580	0.7797	0.8231	0.0446
TPM3	0.6163	1.1311	0.8320	0.7132	0.8231	0.1117
NEIL2	0.9461	0.8776	0.9112	0.5577	0.8231	0.0896
SAFB2	0.6634	1.1168	0.8651	0.6476	0.8232	0.1097
THAP10	0.6235	1.1060	0.8248	0.7386	0.8232	0.1029
ASAP1	0.6538	1.1533	0.7425	0.7437	0.8233	0.1120

CCNT1	0.5298	1.0967	0.9535	0.7135	0.8234	0.1258
MRPS9	0.5857	1.2170	0.8002	0.6909	0.8234	0.1383
AGPAT2	0.7802	0.9908	0.8419	0.6808	0.8234	0.0649
ZNF295	0.6305	1.0684	0.8891	0.7059	0.8235	0.0980
SNORD57	0.6827	0.8627	0.9403	0.8083	0.8235	0.0542
ABHD12	0.7657	0.9706	0.8433	0.7146	0.8236	0.0557
IL27RA	0.7124	0.9716	0.8423	0.7681	0.8236	0.0561
USP46	0.7061	1.1165	0.7856	0.6862	0.8236	0.1000
NUBP1	0.6851	1.1858	0.7888	0.6348	0.8236	0.1249
IDH3B	0.6461	1.0970	0.8535	0.6982	0.8237	0.1012
C10orf57	0.9315	1.0246	0.7421	0.5968	0.8237	0.0958
MGC3196	0.6527	1.0576	0.8816	0.7031	0.8237	0.0921
LOC732007	0.6555	1.1993	0.8190	0.6213	0.8238	0.1324
PLEKHA3	0.7426	1.0907	0.7759	0.6858	0.8238	0.0909
TOM1L1	0.5396	1.0621	0.8930	0.8005	0.8238	0.1091
FAM114A1	0.6711	1.1208	0.8206	0.6829	0.8239	0.1046
LOC93622	0.7106	1.0037	0.8275	0.7537	0.8239	0.0646
C7orf41	0.6915	1.0398	0.8669	0.6974	0.8239	0.0827
TUB	0.7379	1.0009	0.8437	0.7132	0.8239	0.0654
LOC647719	0.7191	0.9025	0.9546	0.7194	0.8239	0.0613
LOC389873	0.5819	1.2040	0.7945	0.7155	0.8240	0.1341
COG8	0.7712	1.0456	0.8126	0.6667	0.8240	0.0800
PMS2	0.6157	1.0117	0.9428	0.7259	0.8240	0.0924
MLF2	0.7233	1.0984	0.8121	0.6624	0.8240	0.0965
ELAC2	0.6645	1.1429	0.8364	0.6524	0.8241	0.1143
LOC652388	0.7383	1.2099	0.6670	0.6811	0.8241	0.1295
ACAA2	0.7975	0.8981	0.8105	0.7903	0.8241	0.0250
ZNRD1	0.6647	1.1172	0.8517	0.6629	0.8241	0.1073
ARID2	0.7030	1.0209	0.8593	0.7132	0.8241	0.0747
THAP11	0.6391	1.1099	0.8821	0.6654	0.8241	0.1097
WASF3	0.6714	1.1378	0.8104	0.6774	0.8242	0.1093
SLC7A1	0.7203	1.1680	0.7929	0.6158	0.8243	0.1202
PHTF2	0.7204	1.1056	0.8182	0.6532	0.8243	0.0997
LOC1001313	0.7692	0.9336	0.8647	0.7302	0.8244	0.0461
PER3	0.5443	1.1153	0.8902	0.7479	0.8244	0.1202
R3HDM1	0.6841	1.0264	0.8202	0.7671	0.8244	0.0729
DHX38	0.8060	0.8833	0.9002	0.7083	0.8245	0.0438
TARBP1	0.7204	1.0746	0.7061	0.7968	0.8245	0.0857
LOC440145	0.7572	0.8879	0.9910	0.6618	0.8245	0.0723
TBC1D23	0.6453	0.9905	0.9291	0.7332	0.8245	0.0811
GPRC5C	0.8221	0.8884	0.8410	0.7467	0.8245	0.0295
SCYL1	0.7907	0.9767	0.8708	0.6602	0.8246	0.0667
DNAJC13	0.7861	1.0626	0.7194	0.7303	0.8246	0.0807
RRP1	0.8109	0.9013	0.8133	0.7730	0.8246	0.0272
ATP6V1C1	0.8054	0.9601	0.8197	0.7134	0.8247	0.0509
ANKFY1	0.7550	1.1059	0.7318	0.7067	0.8249	0.0942

ATG10	0.6993	1.0967	0.7905	0.7130	0.8249	0.0928
CSRP1	0.5128	1.1936	0.8375	0.7557	0.8249	0.1409
MRS2	0.5943	1.2140	0.9086	0.5831	0.8250	0.1500
DCUN1D1	0.7392	0.9811	0.8251	0.7547	0.8250	0.0553
LOC645726	0.8166	0.8971	0.8137	0.7727	0.8250	0.0260
SNX6	0.7673	1.0091	0.7155	0.8085	0.8251	0.0642
LOC654135	0.7113	1.0143	0.8615	0.7135	0.8251	0.0722
GBAS	0.7491	1.1427	0.8160	0.5929	0.8252	0.1157
PHAX	0.6663	1.0391	0.9603	0.6354	0.8253	0.1022
TBL2	0.6516	1.0449	0.8872	0.7174	0.8253	0.0884
FSCN1	0.7786	1.0129	0.8006	0.7092	0.8254	0.0655
UBE2M	0.7024	1.0880	0.8969	0.6142	0.8254	0.1056
CDC2L6	0.5673	1.3082	0.8053	0.6208	0.8254	0.1688
C1orf177	0.8129	0.8038	0.9496	0.7352	0.8254	0.0449
MTERF	0.6775	1.0621	0.8216	0.7406	0.8254	0.0842
CASC5	0.7657	0.9007	0.8580	0.7774	0.8254	0.0324
C3orf31	0.5647	1.1649	0.8638	0.7084	0.8254	0.1286
LOC1001296	0.7674	0.8797	0.8603	0.7944	0.8255	0.0266
TINP1	0.6781	0.9953	0.9626	0.6661	0.8255	0.0889
SH3BP1	0.7985	0.9843	0.7076	0.8117	0.8255	0.0578
COMMD5	0.7008	1.0416	0.8537	0.7060	0.8255	0.0803
EIF3M	0.6496	1.1355	0.8757	0.6412	0.8255	0.1167
LOC644131	0.7972	0.8051	1.0840	0.6159	0.8255	0.0966
TRIM25	0.8647	1.0030	0.7774	0.6573	0.8256	0.0728
MDM4	0.7758	0.9561	0.8289	0.7417	0.8256	0.0470
PGAM1	0.5765	1.1525	0.8927	0.6808	0.8256	0.1273
MLLT6	0.6028	1.1653	0.8204	0.7144	0.8257	0.1216
FLJ10374	0.6252	1.0944	0.8056	0.7777	0.8257	0.0979
FUCA2	0.7292	1.0046	0.8664	0.7028	0.8257	0.0696
DKFZP564O0	0.7065	1.0401	0.8351	0.7214	0.8258	0.0770
C1orf85	0.7393	1.1252	0.7989	0.6397	0.8258	0.1051
SMG7	0.7739	1.0684	0.8031	0.6582	0.8259	0.0867
KHDRBS3	0.7306	1.0159	0.8553	0.7022	0.8260	0.0715
SMARCA4	0.6757	1.1000	0.8551	0.6731	0.8260	0.1008
PELO	0.6431	1.2485	0.8476	0.5648	0.8260	0.1529
LOC652183	0.6686	1.0900	0.8140	0.7317	0.8260	0.0929
LSM10	0.6318	1.2072	0.8332	0.6322	0.8261	0.1356
ATP8B1	0.7763	0.9790	0.7990	0.7502	0.8261	0.0519
TTC38	0.8192	0.9861	0.8219	0.6774	0.8262	0.0631
C19orf43	0.7167	1.0342	0.8253	0.7286	0.8262	0.0735
ORC2L	0.5798	1.0144	0.9210	0.7896	0.8262	0.0942
ADSL	0.6581	1.2933	0.7968	0.5570	0.8263	0.1633
RBM34	0.6317	0.9956	1.0053	0.6725	0.8263	0.1009
CA11	0.7059	0.9901	0.8106	0.7988	0.8263	0.0594
LOC654053	0.8397	0.9356	0.8598	0.6704	0.8264	0.0559
PSMD3	0.6621	1.1553	0.7588	0.7293	0.8264	0.1115

GNG4	0.7579	1.1055	0.7138	0.7286	0.8265	0.0935
RFXANK	0.7525	1.0182	0.7946	0.7407	0.8265	0.0649
CUL1	0.6181	1.0576	0.8845	0.7459	0.8265	0.0943
RPUSD3	0.7883	1.0833	0.8071	0.6276	0.8266	0.0946
RBM45	0.6590	1.0002	0.9594	0.6881	0.8267	0.0890
SETD6	0.7947	0.9281	0.8616	0.7225	0.8267	0.0441
MCAT	0.7269	1.0933	0.8044	0.6822	0.8267	0.0924
ADM	0.7690	0.8911	0.8210	0.8257	0.8267	0.0250
LRRC28	0.6601	1.0689	0.8404	0.7375	0.8267	0.0888
LOC1001335	0.7500	1.1978	0.6815	0.6776	0.8267	0.1248
UBE2I	0.6518	1.1658	0.8304	0.6590	0.8268	0.1203
SH3KBP1	0.6611	1.0779	0.8486	0.7196	0.8268	0.0924
LOC645979	0.5829	1.1287	0.9521	0.6439	0.8269	0.1290
ELOF1	0.7172	1.0705	0.9161	0.6038	0.8269	0.1037
EIF2B4	0.6419	1.1324	0.8807	0.6528	0.8270	0.1158
HEG1	0.7549	0.8786	0.8038	0.8708	0.8270	0.0293
C17orf93	0.6228	1.0868	0.9335	0.6649	0.8270	0.1106
TIMM22	0.6350	1.1187	0.7931	0.7614	0.8270	0.1030
LOC1001317	0.7436	1.0881	0.8008	0.6758	0.8271	0.0907
LOC1001333	0.5806	1.1482	1.0043	0.5752	0.8271	0.1468
AAMP	0.7688	1.0660	0.8555	0.6180	0.8271	0.0935
NUDT9	0.7145	1.1054	0.8458	0.6429	0.8271	0.1018
SCAMP1	0.7187	1.0445	0.8621	0.6833	0.8272	0.0821
PLLP	0.7814	1.0214	0.7963	0.7097	0.8272	0.0674
TAF12	0.7775	1.1944	0.7209	0.6161	0.8272	0.1269
PPP1R3D	0.8090	1.0293	0.8344	0.6363	0.8272	0.0804
ZNF830	0.7822	0.9001	0.8719	0.7550	0.8273	0.0348
ASPSCR1	0.7147	1.0750	0.8597	0.6598	0.8273	0.0927
FLJ20628	0.6867	1.0464	0.8947	0.6816	0.8273	0.0883
MRPL17	0.8097	0.9916	0.8276	0.6805	0.8274	0.0638
LOC1001285	0.8008	0.9650	0.8285	0.7152	0.8274	0.0518
POLR3B	0.6914	1.1524	0.7644	0.7014	0.8274	0.1095
LOC150223	0.7588	1.0464	0.8105	0.6941	0.8274	0.0768
RAB26	0.6624	1.1360	0.9039	0.6081	0.8276	0.1212
C15orf44	0.7585	1.0414	0.8227	0.6882	0.8277	0.0763
CDKAL1	0.6605	1.0519	0.8748	0.7235	0.8277	0.0872
MGAT2	0.6762	1.0442	0.8992	0.6914	0.8278	0.0883
SLC39A6	0.6716	1.1336	0.8298	0.6760	0.8278	0.1084
B9D2	0.7114	1.0120	0.8698	0.7178	0.8278	0.0715
DFFA	0.6058	1.0722	0.8934	0.7397	0.8278	0.1004
STEAP3	0.7023	1.0143	0.8346	0.7600	0.8278	0.0678
ALG14	0.6376	1.2342	0.8400	0.5994	0.8278	0.1454
LOC1001338	0.6881	0.9462	0.8430	0.8344	0.8279	0.0531
SDCCAG1	0.6907	1.0476	0.8262	0.7473	0.8279	0.0783
ETFB	0.6095	1.3176	0.7468	0.6379	0.8280	0.1659
DRAP1	0.7810	1.0956	0.7933	0.6421	0.8280	0.0955

DNAJC7	0.6965	1.1521	0.7662	0.6974	0.8280	0.1092
SGTA	0.7139	1.0176	0.8042	0.7767	0.8281	0.0659
FDX1	0.7393	0.8678	0.9690	0.7363	0.8281	0.0561
SFRS4	0.6006	1.2193	0.8239	0.6688	0.8282	0.1385
SLC6A9	0.7512	0.9420	0.8338	0.7857	0.8282	0.0416
SPATA2L	0.7304	1.0469	0.8178	0.7179	0.8282	0.0762
RPRD1A	0.5875	1.3814	0.7145	0.6296	0.8283	0.1863
PSMC2	0.6759	1.0819	0.8572	0.6982	0.8283	0.0937
SRPX	0.7451	0.9532	0.8811	0.7341	0.8284	0.0534
KENAE	0.7316	1.0054	0.8692	0.7076	0.8284	0.0689
LOC152195	0.7649	1.0598	0.7851	0.7040	0.8285	0.0790
LOC647474	0.6211	1.0648	0.8207	0.8075	0.8285	0.0910
ABHD6	0.7976	1.1316	0.7286	0.6564	0.8286	0.1050
LOC642333	0.7235	0.9331	0.9772	0.6805	0.8286	0.0742
LOC1001341	0.6008	0.9980	0.9130	0.8027	0.8286	0.0858
CPSF4	0.7289	1.0554	0.8389	0.6915	0.8287	0.0818
LOC644237	0.6337	1.0889	0.9064	0.6858	0.8287	0.1050
AIMP2	0.6604	1.1365	0.9185	0.5994	0.8287	0.1237
ARG2	0.8157	1.0453	0.8008	0.6532	0.8287	0.0810
DHX40	0.7171	1.0786	0.8306	0.6887	0.8287	0.0887
THEM2	0.6847	1.1049	0.7696	0.7558	0.8288	0.0939
ITGB2	0.7645	1.0693	0.8085	0.6728	0.8288	0.0850
MSTO1	0.7121	0.8682	1.0029	0.7321	0.8288	0.0676
LOC653110	0.8786	0.8888	0.8193	0.7293	0.8290	0.0366
HGS	0.6087	1.2277	0.7616	0.7180	0.8290	0.1368
NT5C3L	0.6688	1.1504	0.8021	0.6949	0.8290	0.1109
COX8A	0.7306	1.0883	0.7573	0.7402	0.8291	0.0866
ANKRD9	0.7349	1.1727	0.7989	0.6101	0.8291	0.1210
CPD	0.7974	1.0891	0.7646	0.6655	0.8291	0.0911
CENPP	0.7523	0.8681	0.8841	0.8121	0.8292	0.0299
ICAM3	0.7018	1.2013	0.7841	0.6294	0.8292	0.1280
ORC4L	0.7212	0.8789	0.9561	0.7604	0.8292	0.0540
IGBP1	0.7962	0.9887	0.8801	0.6518	0.8292	0.0711
LOC25845	0.6744	1.1947	0.7977	0.6503	0.8293	0.1260
CCDC88C	0.6491	1.0691	0.7027	0.8964	0.8293	0.0960
NUDT16L1	0.6805	1.1347	0.7845	0.7176	0.8293	0.1040
TPD52L1	0.6449	1.2272	0.7798	0.6654	0.8293	0.1359
UBAC1	0.6358	1.0670	0.8158	0.7988	0.8293	0.0890
C3orf37	0.7231	1.2082	0.7702	0.6164	0.8295	0.1303
KBTBD8	0.6013	1.0203	0.9162	0.7810	0.8297	0.0905
ACOT9	0.7125	1.1132	0.8615	0.6320	0.8298	0.1057
STT3A	0.8081	1.0374	0.8120	0.6619	0.8298	0.0775
SEC23IP	0.6921	1.1216	0.8487	0.6570	0.8298	0.1058
CGN	0.7395	1.1130	0.7548	0.7123	0.8299	0.0948
LOC284988	0.7128	0.9764	0.9060	0.7245	0.8299	0.0659
OXSM	0.8213	0.9605	0.8945	0.6436	0.8300	0.0683

UQCRC1	0.7465	1.0741	0.8168	0.6824	0.8300	0.0859
PNO1	0.6583	0.9418	1.0443	0.6756	0.8300	0.0965
RFX5	0.6730	1.1833	0.7995	0.6641	0.8300	0.1218
CDC6	0.7579	0.9379	0.8372	0.7870	0.8300	0.0395
LOC158160	0.9605	0.8887	0.8368	0.6341	0.8300	0.0700
RAE1	0.6648	1.1251	0.8423	0.6879	0.8300	0.1060
TPRKB	0.7912	1.0126	0.8102	0.7063	0.8301	0.0649
CXorf64	0.5151	1.0627	0.9704	0.7722	0.8301	0.1212
RECQL	0.6607	1.0316	0.9668	0.6614	0.8301	0.0985
LEMD3	0.7481	1.0972	0.7908	0.6846	0.8301	0.0917
ARCN1	0.7611	1.1209	0.7661	0.6727	0.8302	0.0992
SUCLG1	0.5991	1.0533	0.9883	0.6805	0.8303	0.1120
FARS2	0.6988	1.0886	0.8439	0.6900	0.8303	0.0930
UBE1DC1	0.6769	1.0308	0.9382	0.6753	0.8303	0.0910
SNORA25	0.6663	1.0320	0.8496	0.7734	0.8303	0.0770
SKA2	0.6729	0.9715	1.0472	0.6297	0.8303	0.1049
LOC341457	0.7502	0.9608	0.9989	0.6115	0.8304	0.0912
AK2	0.6503	1.1457	0.8819	0.6436	0.8304	0.1188
TUBGCP2	0.8432	1.1162	0.7188	0.6438	0.8305	0.1037
C2orf18	0.7517	0.9736	0.8667	0.7306	0.8307	0.0563
MRPL30	0.6445	0.9237	1.0634	0.6912	0.8307	0.0987
KCNS3	0.7484	0.9971	0.8203	0.7574	0.8308	0.0577
DAZAP1	0.7075	1.0533	0.8465	0.7158	0.8308	0.0807
KLHL18	0.7350	1.0717	0.8230	0.6938	0.8308	0.0847
LOC143543	0.7286	0.8159	1.0308	0.7487	0.8310	0.0692
ZMYND11	0.6515	1.1434	0.8304	0.6997	0.8312	0.1107
TUFM	0.6928	1.0646	0.8696	0.6986	0.8314	0.0879
LOC649553	0.5765	1.2084	0.8294	0.7114	0.8314	0.1359
NDUFV3	0.6631	1.0782	0.9020	0.6823	0.8314	0.0985
RNF14	0.7035	0.9748	0.9903	0.6574	0.8315	0.0878
EIF3A	0.6625	1.0036	1.0672	0.5927	0.8315	0.1193
C1orf31	0.7470	0.9310	1.0288	0.6193	0.8315	0.0917
CCDC90A	0.6699	0.9752	1.0141	0.6670	0.8316	0.0945
UCRC	0.6804	1.1747	0.8431	0.6280	0.8316	0.1232
GRTP1	0.8116	0.9849	0.8778	0.6520	0.8316	0.0697
NIPSNAP3A	0.8251	1.0280	0.8255	0.6478	0.8316	0.0777
TWF1	0.7248	0.9980	0.9549	0.6489	0.8316	0.0855
COPB1	0.7902	1.0206	0.8956	0.6203	0.8317	0.0847
NDUFS1	0.7029	0.9658	0.8807	0.7774	0.8317	0.0577
RPUSD2	0.6324	1.0323	0.9503	0.7120	0.8318	0.0950
IPO8	0.8138	1.1079	0.7994	0.6060	0.8318	0.1035
HNRNPL	0.6829	1.2134	0.7566	0.6742	0.8318	0.1285
SLC22A3	0.7630	1.0214	0.8027	0.7403	0.8318	0.0645
AKNA	0.8261	0.8939	0.8040	0.8037	0.8319	0.0213
NSUN5C	0.6924	1.0065	0.8606	0.7683	0.8320	0.0676
MTHFS	0.6658	1.2303	0.8024	0.6294	0.8320	0.1379

ZFYVE20	0.7093	1.0854	0.8546	0.6788	0.8320	0.0928
INO80B	0.7506	0.9940	0.9431	0.6404	0.8320	0.0826
ALG5	0.7090	0.9611	0.9938	0.6645	0.8321	0.0847
NR1H3	0.7448	0.8888	0.8638	0.8311	0.8321	0.0314
TCAM1	0.7417	1.0247	0.8151	0.7471	0.8321	0.0663
TBCB	0.7942	1.1882	0.7274	0.6193	0.8323	0.1240
NUDT2	0.6611	1.1707	0.7810	0.7165	0.8323	0.1154
LOC727980	0.6577	1.1736	0.7939	0.7043	0.8324	0.1172
MRPS22	0.6484	1.0950	0.8482	0.7383	0.8325	0.0966
MAP3K4	0.7274	0.9884	0.8478	0.7664	0.8325	0.0577
RPP40	0.6740	1.0952	0.8952	0.6657	0.8325	0.1024
SENP6	0.8226	1.0061	0.8287	0.6731	0.8326	0.0681
OVOL2	0.6997	1.2366	0.8000	0.5942	0.8326	0.1411
DDX55	0.5621	1.4103	0.7498	0.6086	0.8327	0.1966
KLHL8	0.6467	1.0729	0.8747	0.7366	0.8327	0.0928
LOC730534	0.5934	1.1015	0.9760	0.6604	0.8328	0.1224
UPF2	0.6570	1.1639	0.8050	0.7054	0.8328	0.1146
TAF1B	0.7269	0.9935	0.8729	0.7381	0.8328	0.0630
FAM3A	0.7979	1.0343	0.8707	0.6285	0.8329	0.0842
TULP3	0.8032	1.1433	0.7445	0.6405	0.8329	0.1088
CD302	0.7857	0.9211	0.8775	0.7473	0.8329	0.0401
LRRC45	0.7798	0.9572	0.8249	0.7698	0.8329	0.0431
KIAA1530	0.7570	0.9918	0.8105	0.7724	0.8329	0.0541
SLAIN1	0.6295	1.0597	0.9931	0.6495	0.8329	0.1126
LOC202227	0.7501	0.9883	0.7742	0.8193	0.8330	0.0537
HOXC9	0.7427	1.0244	0.7698	0.7953	0.8330	0.0647
LOC1001285	0.8123	0.9407	0.7960	0.7833	0.8331	0.0364
C9orf69	0.7801	0.9946	0.8181	0.7394	0.8331	0.0562
PGRMC1	0.7618	1.0722	0.8636	0.6350	0.8331	0.0924
CNNM3	0.6585	1.0839	0.8877	0.7025	0.8331	0.0972
CRSP9	0.7460	1.1661	0.7699	0.6509	0.8332	0.1139
HMGCS2	0.6852	1.1682	0.8380	0.6415	0.8332	0.1193
IREB2	0.5773	1.2890	0.7448	0.7221	0.8333	0.1564
HEBP2	0.6175	0.9284	1.1583	0.6291	0.8334	0.1301
BTF3L4	0.7493	1.1992	0.7582	0.6267	0.8334	0.1256
TIAL1	0.7438	0.9904	0.9134	0.6858	0.8334	0.0712
C21orf24	0.7563	0.9804	0.9768	0.6201	0.8334	0.0883
LOC641768	0.6969	0.7218	1.3388	0.5762	0.8334	0.1714
LOC202781	0.7703	1.0800	0.8444	0.6389	0.8334	0.0925
PHRF1	0.6336	1.1355	0.8547	0.7099	0.8334	0.1106
NFATC2IP	0.7042	1.0166	0.8780	0.7351	0.8334	0.0718
LOC646197	0.6704	1.0885	0.9122	0.6628	0.8335	0.1029
FAM165B	0.7125	1.0953	0.8308	0.6954	0.8335	0.0923
CLN6	0.6945	0.9707	0.8496	0.8191	0.8335	0.0567
C1orf156	0.7037	1.0191	0.8386	0.7726	0.8335	0.0677
ISCA1	0.6728	1.0778	0.9421	0.6414	0.8335	0.1057

MRPL44	0.6457	1.0809	0.9435	0.6641	0.8336	0.1069
DDX23	0.7763	1.1868	0.7623	0.6088	0.8336	0.1237
FAM111B	0.6878	0.9615	0.8603	0.8249	0.8336	0.0566
DAD1	0.7214	1.0974	0.8505	0.6651	0.8336	0.0961
ZNF775	0.7691	1.0241	0.7897	0.7515	0.8336	0.0640
HMOX1	0.7556	1.0068	0.7689	0.8034	0.8337	0.0586
AHCY	0.6805	1.0295	0.8988	0.7260	0.8337	0.0804
ARL5A	0.5425	1.1142	0.9781	0.7002	0.8338	0.1298
SF3B3	0.6222	1.0436	0.9470	0.7223	0.8338	0.0975
RASL11B	0.7155	1.0049	0.9312	0.6839	0.8339	0.0792
ARHGEF10	0.8526	0.9865	0.7595	0.7369	0.8339	0.0567
CENPI	0.7009	0.9147	0.8864	0.8338	0.8340	0.0474
CANX	0.6935	1.1354	0.8548	0.6523	0.8340	0.1096
EIF2B3	0.7094	0.8995	1.1409	0.5863	0.8340	0.1209
LOC649864	0.6696	0.9947	0.9007	0.7712	0.8341	0.0714
RCHY1	0.7725	0.9460	0.8843	0.7339	0.8342	0.0491
EPPB9	0.7134	0.9218	0.8805	0.8214	0.8342	0.0453
LOC644404	0.7211	1.0019	0.7557	0.8588	0.8344	0.0630
PRCP	0.7295	1.0461	0.8576	0.7046	0.8344	0.0781
LOC440731	0.7940	0.8577	1.0455	0.6410	0.8345	0.0837
PPP2CA	0.6633	1.1049	0.9330	0.6370	0.8346	0.1122
MALL	0.8237	0.9483	0.8717	0.6945	0.8346	0.0533
LOC728059	0.5892	1.0464	0.9310	0.7719	0.8346	0.0993
CUGBP1	0.6186	1.1508	0.8924	0.6767	0.8346	0.1207
NANP	0.6428	1.1469	0.8395	0.7095	0.8347	0.1118
LOC442075	0.7061	1.0205	0.8064	0.8058	0.8347	0.0663
TMEM14B	0.6963	0.9026	1.1665	0.5739	0.8348	0.1297
POLR3K	0.6041	1.0553	0.9550	0.7249	0.8349	0.1034
MED25	0.7870	1.0958	0.7940	0.6627	0.8349	0.0920
LONP1	0.7924	1.0585	0.7469	0.7421	0.8350	0.0754
GLB1L2	0.6959	1.1084	0.7523	0.7832	0.8350	0.0929
HPRT1	0.6366	1.1046	0.9250	0.6739	0.8350	0.1103
TCTN3	0.8265	0.9353	0.9419	0.6364	0.8350	0.0713
ATP5O	0.7318	1.0169	0.8746	0.7173	0.8352	0.0702
ENOSF1	0.7156	0.9927	1.0586	0.5739	0.8352	0.1145
POLR2G	0.6796	1.1076	0.8658	0.6877	0.8352	0.1005
HIST1H2BO	0.7555	0.9289	0.8812	0.7752	0.8352	0.0417
HSPA4	0.7694	0.9525	0.9626	0.6563	0.8352	0.0743
MED10	0.6876	1.1003	0.8900	0.6630	0.8352	0.1019
RNF19B	0.9105	0.8694	0.8182	0.7427	0.8352	0.0362
ATE1	0.7017	1.0085	0.8574	0.7732	0.8352	0.0659
VPS39	0.6686	0.9863	0.8996	0.7865	0.8353	0.0690
GRINA	0.7231	1.0798	0.8353	0.7030	0.8353	0.0865
PUF60	0.6226	1.0714	0.8849	0.7623	0.8353	0.0952
LOC392635	0.7988	0.8737	0.8755	0.7933	0.8353	0.0227
BCAT2	0.7159	1.1415	0.8409	0.6430	0.8353	0.1099

DPM3	0.6657	1.2045	0.8382	0.6332	0.8354	0.1310
SNORD56	0.7198	0.9978	0.8255	0.7987	0.8354	0.0586
HPS1	0.7860	1.0322	0.8085	0.7155	0.8356	0.0685
LOC151579	0.6229	1.2145	1.0103	0.4946	0.8356	0.1672
TTC39C	0.6227	1.0453	0.9328	0.7421	0.8357	0.0946
FASTKD1	0.6039	1.1799	0.8751	0.6841	0.8357	0.1281
C2orf76	0.7788	0.7732	1.0607	0.7305	0.8358	0.0758
C21orf66	0.7403	0.9963	0.8371	0.7694	0.8358	0.0572
MTO1	0.6977	1.0747	0.8524	0.7185	0.8358	0.0867
LOC1001335	0.6606	1.0500	0.8315	0.8015	0.8359	0.0805
YOD1	0.8272	1.0942	0.7471	0.6752	0.8359	0.0915
LOC399900	0.6816	1.1343	0.8518	0.6760	0.8359	0.1075
C2orf69	0.6203	1.1510	0.8518	0.7208	0.8360	0.1152
KIAA0664	0.7012	1.1223	0.8468	0.6739	0.8361	0.1027
NPEPPS	0.8680	0.9497	0.8223	0.7044	0.8361	0.0512
LOC554206	0.7378	1.1002	0.8440	0.6624	0.8361	0.0956
TMEM135	0.7990	0.9313	0.9501	0.6643	0.8362	0.0664
SCLT1	0.8041	0.9771	0.8435	0.7203	0.8363	0.0535
C7orf50	0.5836	1.0798	0.9338	0.7479	0.8363	0.1082
HSD17B11	0.6948	1.0350	0.9345	0.6810	0.8363	0.0881
RBBP4	0.7925	1.0299	0.7853	0.7377	0.8363	0.0656
MAP4K2	0.6109	1.2267	0.7936	0.7143	0.8363	0.1354
PLDN	0.6601	1.2429	0.7623	0.6803	0.8364	0.1373
SSX2IP	0.7940	1.0234	0.7751	0.7532	0.8364	0.0629
THOC6	0.6665	1.0509	0.8569	0.7716	0.8365	0.0814
STK38	0.7574	1.0793	0.7799	0.7293	0.8365	0.0816
SNORD4A	0.7363	1.0458	0.8226	0.7413	0.8365	0.0725
LRP5	0.6966	1.0756	0.8216	0.7523	0.8365	0.0837
LOC729646	0.6744	1.1656	0.9392	0.5672	0.8366	0.1347
LOC151162	0.6358	1.0491	0.9803	0.6815	0.8367	0.1042
HNRPH3	0.7302	0.9586	1.0283	0.6296	0.8367	0.0939
SLC43A1	0.6285	1.1638	0.7159	0.8386	0.8367	0.1172
CDKN1C	0.7967	0.8645	0.9033	0.7824	0.8367	0.0285
TTC31	0.7960	0.9726	0.8232	0.7552	0.8367	0.0474
SRRD	0.6913	0.9917	0.9835	0.6805	0.8368	0.0871
PPP1CC	0.7191	1.0442	0.9384	0.6454	0.8368	0.0930
FAM3C	0.7170	1.0865	0.8242	0.7196	0.8368	0.0869
PIAS3	0.8314	0.9611	0.8716	0.6834	0.8369	0.0579
SNRNP70	0.7562	1.0572	0.8354	0.6993	0.8370	0.0785
GAGE12F	0.7210	1.0555	0.8613	0.7104	0.8371	0.0805
TK2	0.8044	1.1136	0.7818	0.6485	0.8371	0.0984
ACPL2	0.8544	1.0485	0.7229	0.7225	0.8371	0.0770
CIDEB	0.7343	0.9943	0.8255	0.7942	0.8371	0.0557
TM2D3	0.7121	1.1774	0.8360	0.6229	0.8371	0.1216
BAT4	0.7620	1.0228	0.8082	0.7553	0.8371	0.0630
CCDC56	0.6999	1.0550	0.9212	0.6724	0.8371	0.0915

POP4	0.6951	0.9628	1.0925	0.5984	0.8372	0.1148
DEDD	0.8231	1.0288	0.7696	0.7273	0.8372	0.0668
ZNF573	0.6265	1.1023	0.8647	0.7554	0.8372	0.1009
FTO	0.7638	1.1192	0.8163	0.6498	0.8373	0.1002
WAC	0.7079	1.0250	0.9736	0.6426	0.8373	0.0951
SNORD25	0.7176	0.9162	0.8944	0.8210	0.8373	0.0448
GIT1	0.8288	0.9189	0.8183	0.7833	0.8373	0.0289
HES1	0.6944	1.0406	0.9464	0.6682	0.8374	0.0923
HECTD2	0.7428	1.0217	0.8607	0.7245	0.8374	0.0684
KIAA2013	0.7312	1.2065	0.8110	0.6011	0.8374	0.1304
C17orf90	0.7079	1.1131	0.8371	0.6918	0.8375	0.0975
KIAA0895L	0.7122	1.0030	0.8831	0.7516	0.8375	0.0662
TAPT1	0.7880	1.0728	0.8827	0.6065	0.8375	0.0971
USP5	0.7101	1.1579	0.7887	0.6932	0.8375	0.1088
FBXO33	0.7452	1.0243	0.9020	0.6785	0.8375	0.0779
FOXK1	0.6872	1.0854	0.8041	0.7733	0.8375	0.0863
C6orf85	0.7691	1.0774	0.8117	0.6918	0.8375	0.0837
RNY3	0.6594	0.9510	0.9750	0.7652	0.8377	0.0757
LOC642255	0.7719	0.8988	0.8374	0.8426	0.8377	0.0259
ZNF69	0.6518	1.0559	0.9018	0.7413	0.8377	0.0892
LOC139046	0.7453	1.0505	0.8514	0.7036	0.8377	0.0775
LOC729342	0.7458	0.9201	0.9733	0.7117	0.8377	0.0642
APOO	0.6066	1.3866	0.7931	0.5648	0.8378	0.1895
PBRM1	0.6710	1.1217	0.7978	0.7609	0.8378	0.0983
LOC1001296	0.3824	1.0026	1.2322	0.7341	0.8378	0.1828
MRPL16	0.7269	1.0980	0.8833	0.6433	0.8379	0.1000
CAMSAP1L1	0.6755	1.0963	0.9710	0.6094	0.8380	0.1166
ADAM23	0.7446	1.0216	0.7954	0.7909	0.8381	0.0622
SORL1	0.7394	0.9762	0.7921	0.8449	0.8382	0.0508
KTI12	0.7488	1.0793	0.8209	0.7038	0.8382	0.0839
ATP5SL	0.7608	1.0397	0.8786	0.6739	0.8383	0.0792
FOXD1	0.7903	1.1049	0.7512	0.7067	0.8383	0.0905
ECHDC3	0.7693	1.1240	0.8065	0.6537	0.8384	0.1006
TMTC3	0.7165	1.0253	0.8895	0.7224	0.8384	0.0741
FAM116B	0.7480	1.0549	0.8241	0.7266	0.8384	0.0751
LOC1001330	0.7586	1.0069	0.8358	0.7524	0.8384	0.0593
EFTUD2	0.6775	1.1605	0.9067	0.6091	0.8385	0.1248
FAM173A	0.7718	1.2661	0.7499	0.5662	0.8385	0.1498
FAM131A	0.7162	1.3037	0.7536	0.5806	0.8385	0.1595
NOC3L	0.6938	0.8785	1.0389	0.7430	0.8385	0.0774
ESRRA	0.6778	1.2240	0.7786	0.6738	0.8386	0.1308
COIL	0.7020	1.1679	0.7687	0.7162	0.8387	0.1107
SMARCD3	0.7444	0.9932	0.8761	0.7411	0.8387	0.0603
MKI67	0.6941	1.0029	0.8662	0.7918	0.8387	0.0651
C11orf17	0.6788	1.1216	0.8888	0.6657	0.8388	0.1073
ZNF124	0.6746	0.9740	0.8973	0.8100	0.8390	0.0642

CNPY3	0.7258	1.0279	0.8864	0.7158	0.8390	0.0741
HMGXB4	0.7833	1.0386	0.8276	0.7066	0.8390	0.0711
DCBLD1	0.7109	0.9934	0.9279	0.7241	0.8391	0.0715
LOC401076	0.6917	0.9720	0.9783	0.7143	0.8391	0.0787
ZMPSTE24	0.7423	1.2289	0.7427	0.6425	0.8391	0.1321
MGMT	0.7024	1.1375	0.9003	0.6162	0.8391	0.1159
CTNS	0.6674	1.1662	0.8416	0.6815	0.8392	0.1159
CHCHD5	0.8452	1.0740	0.8251	0.6128	0.8393	0.0943
UBE2V1	0.6506	1.0042	0.8676	0.8348	0.8393	0.0728
LZTFL1	0.8057	0.9899	0.8647	0.6971	0.8394	0.0610
SNORA79	0.4582	1.0056	1.0852	0.8085	0.8394	0.1397
CORO1A	0.8221	0.8982	0.8635	0.7740	0.8394	0.0268
ATAD3B	0.7399	0.9431	0.8997	0.7751	0.8394	0.0487
CNN2	0.7190	1.1143	0.7953	0.7294	0.8395	0.0931
LOC644295	0.7024	0.9715	0.8887	0.7956	0.8396	0.0581
LOC1001301	0.7381	0.9466	0.9318	0.7417	0.8396	0.0576
FLJ25363	0.7803	0.9005	0.8676	0.8098	0.8396	0.0272
ENHO	0.8373	0.9431	0.8722	0.7057	0.8396	0.0498
TOP1MT	0.6632	1.1853	0.7651	0.7448	0.8396	0.1173
LOC728505	0.5658	1.2093	0.8538	0.7296	0.8396	0.1366
FAM72A	0.7821	0.9128	0.9170	0.7467	0.8396	0.0440
POLR3A	0.7510	1.1919	0.7824	0.6334	0.8397	0.1217
CPSF3	0.7018	1.1569	0.8979	0.6022	0.8397	0.1223
SFRS2IP	0.7512	1.0204	0.9363	0.6511	0.8397	0.0844
COX7A2L	0.7866	1.1076	0.8795	0.5854	0.8398	0.1083
SRPRB	0.5673	1.2786	0.9224	0.5909	0.8398	0.1672
NUF2	0.7716	0.9058	0.9179	0.7645	0.8399	0.0416
ITPA	0.7010	1.1398	0.8799	0.6391	0.8400	0.1122
LOC148915	0.6663	1.1625	1.0113	0.5199	0.8400	0.1489
NSA2	0.6918	1.1180	0.8758	0.6742	0.8400	0.1033
XRCC6	0.7421	1.1396	0.8647	0.6136	0.8400	0.1123
GOLSYN	0.7062	0.9930	0.8777	0.7831	0.8400	0.0619
ASPHD1	0.6670	1.0800	0.9093	0.7038	0.8400	0.0961
RBM47	0.7367	1.0813	0.8788	0.6640	0.8402	0.0919
FBXO7	0.7878	1.1358	0.7830	0.6542	0.8402	0.1033
EIF1AX	0.7606	0.8984	0.9751	0.7272	0.8403	0.0582
PRDX2	0.6665	1.1897	0.8567	0.6485	0.8404	0.1256
COMMD3	0.6914	1.1177	0.8635	0.6888	0.8404	0.1011
RNF5	0.7530	1.1350	0.7856	0.6878	0.8404	0.1003
LOC644310	0.6383	0.9910	1.0276	0.7049	0.8404	0.0987
IRX3	0.6291	1.1872	0.8532	0.6925	0.8405	0.1248
C2orf28	0.6723	1.1182	0.9122	0.6595	0.8406	0.1093
LOC645781	0.7549	1.0192	0.8747	0.7134	0.8406	0.0687
LOC341965	0.7393	0.9412	0.8931	0.7888	0.8406	0.0464
PHF10	0.7490	0.8873	0.9242	0.8020	0.8406	0.0399
THAP4	0.7120	0.9865	0.8355	0.8285	0.8406	0.0563

WDYHV1	0.7987	1.1443	0.8441	0.5756	0.8406	0.1170
VPS29	0.7833	1.0024	0.9432	0.6338	0.8407	0.0830
ADRBK1	0.7426	1.1471	0.8026	0.6706	0.8407	0.1056
TECR	0.6178	1.1295	0.9728	0.6427	0.8407	0.1257
LOC283932	0.8332	1.0144	0.7791	0.7364	0.8407	0.0612
GNPAT	0.7562	1.1493	0.8195	0.6388	0.8409	0.1094
LOC730432	0.7087	1.0178	0.9258	0.7116	0.8410	0.0778
SLC45A3	0.8539	1.0211	0.8185	0.6707	0.8410	0.0719
LOC1001301	0.6736	1.1232	0.9068	0.6606	0.8411	0.1098
ZNF195	0.5788	1.0689	1.0460	0.6705	0.8411	0.1264
DDOST	0.7173	1.0124	0.9517	0.6829	0.8411	0.0826
WDR42A	0.7118	1.3015	0.7720	0.5791	0.8411	0.1587
SDCBP	0.7782	0.9825	0.8780	0.7257	0.8411	0.0568
POLR3E	0.6695	1.1810	0.8293	0.6847	0.8411	0.1189
C2orf77	0.7722	0.9115	0.8929	0.7880	0.8412	0.0356
MIOS	0.7120	1.1536	0.8210	0.6783	0.8412	0.1085
SURF1	0.6235	1.1514	0.9765	0.6134	0.8412	0.1335
HPS6	0.6979	1.0645	0.9252	0.6774	0.8413	0.0932
TRMT5	0.6801	1.1548	0.9300	0.6003	0.8413	0.1259
SNORA24	0.6472	1.1399	0.8194	0.7587	0.8413	0.1057
ZNF622	0.6869	1.0622	0.9315	0.6848	0.8413	0.0936
PCGF5	0.7710	0.9466	0.8735	0.7743	0.8413	0.0424
SMC3	0.6975	1.1678	0.7085	0.7916	0.8413	0.1108
CDK5RAP2	0.7893	1.0622	0.7990	0.7156	0.8415	0.0759
XYLT2	0.7828	1.0404	0.9076	0.6362	0.8417	0.0864
GPC2	0.9083	0.9616	0.6158	0.8814	0.8418	0.0772
C9orf91	0.7864	1.1216	0.7369	0.7222	0.8418	0.0943
KLHL36	0.6911	1.1784	0.8032	0.6944	0.8418	0.1152
KIAA1958	0.7110	1.0387	0.8798	0.7376	0.8418	0.0754
LOC85389	0.6763	1.0054	0.9264	0.7594	0.8419	0.0753
PAPOLA	0.7466	1.0100	0.9830	0.6280	0.8419	0.0926
OTUD6B	0.6565	1.2158	0.7615	0.7339	0.8419	0.1266
GPRIN2	0.7874	0.9771	0.8692	0.7341	0.8420	0.0529
RAP1GDS1	0.6712	1.2040	0.8478	0.6450	0.8420	0.1288
HPS3	0.7131	1.1694	0.8730	0.6127	0.8420	0.1216
LOC646993	0.7688	1.0242	0.8327	0.7426	0.8421	0.0636
HOXC8	0.8808	0.9019	0.7585	0.8272	0.8421	0.0320
TAZ	0.7686	0.9319	0.8604	0.8076	0.8421	0.0353
TNFRSF19	0.8308	1.0981	0.7452	0.6946	0.8422	0.0898
C12orf45	0.7332	0.9331	0.9854	0.7171	0.8422	0.0685
DCTN5	0.6947	1.1680	0.7280	0.7783	0.8422	0.1099
PLEKHA1	0.7170	1.0916	0.8951	0.6652	0.8422	0.0966
FAAH	0.7403	1.2256	0.7563	0.6468	0.8423	0.1301
C6orf108	0.7284	1.1011	0.8217	0.7179	0.8423	0.0894
PPP2R5D	0.8223	0.9898	0.8294	0.7276	0.8423	0.0544
U1SNRNPBP	0.6468	1.2570	0.7396	0.7259	0.8423	0.1397

SFRS2B	0.8094	1.0616	0.7888	0.7095	0.8423	0.0762
S100A13	0.7161	1.0505	0.8713	0.7316	0.8424	0.0777
PSPC1	0.6947	1.0433	0.9205	0.7110	0.8424	0.0844
PGP	0.7701	1.0247	0.8222	0.7525	0.8424	0.0626
MKKS	0.7644	1.0502	0.8959	0.6592	0.8424	0.0845
FLJ39653	0.8062	0.8970	0.8810	0.7855	0.8424	0.0274
LOC1001297	0.7522	0.9993	1.0249	0.5935	0.8425	0.1033
HNRNPA3	0.5417	1.3538	0.7783	0.6962	0.8425	0.1774
HP1BP3	0.6595	1.0654	0.8758	0.7693	0.8425	0.0864
ITGB5	0.7584	1.0412	0.8695	0.7009	0.8425	0.0749
C12orf11	0.6896	1.0740	0.9075	0.6988	0.8425	0.0921
MAPKAPK3	0.8079	1.1816	0.7769	0.6036	0.8425	0.1216
PQBP1	0.5998	1.1862	0.8371	0.7469	0.8425	0.1246
FZD3	0.7198	1.0758	0.6984	0.8761	0.8425	0.0873
SNORA73A	0.6548	0.9936	0.8956	0.8261	0.8425	0.0714
EIF1B	0.7989	0.9288	1.0434	0.5993	0.8426	0.0952
SLK	0.7368	1.0354	0.9054	0.6928	0.8426	0.0789
ZNF529	0.7104	1.1165	0.9565	0.5870	0.8426	0.1193
BAT2D1	0.7415	1.2624	0.7645	0.6023	0.8427	0.1444
ZNF525	0.6732	0.9101	1.0066	0.7809	0.8427	0.0730
LXN	0.7817	0.9365	0.8688	0.7840	0.8428	0.0372
BTG3	0.6492	1.0420	0.9353	0.7447	0.8428	0.0891
SRP54	0.7769	1.1621	0.7808	0.6521	0.8430	0.1105
EIF2AK1	0.7774	1.0627	0.8380	0.6944	0.8431	0.0789
SH3BGRL3	0.8356	0.9524	0.9009	0.6837	0.8432	0.0583
FXR1	0.7108	1.0704	0.8839	0.7075	0.8432	0.0862
PMF1	0.5925	1.2214	0.9097	0.6492	0.8432	0.1438
SNRNP40	0.5959	1.2159	0.8292	0.7319	0.8433	0.1331
MTRR	0.7350	1.0023	0.8828	0.7535	0.8434	0.0623
RNF126	0.7815	1.0524	0.8708	0.6690	0.8434	0.0810
PYGO2	0.8406	1.0574	0.8251	0.6506	0.8434	0.0833
LOC654189	0.6441	1.1867	0.8941	0.6489	0.8435	0.1285
WTAP	0.7161	0.9853	0.9550	0.7178	0.8435	0.0733
RPAIN	0.7442	1.1244	0.7720	0.7336	0.8436	0.0940
SCML1	0.6447	1.0314	0.9620	0.7362	0.8436	0.0915
LOC652545	0.7119	1.0632	0.8584	0.7413	0.8437	0.0797
GSTO1	0.7930	0.9637	0.9400	0.6784	0.8438	0.0668
MED31	0.6732	1.0844	0.8907	0.7276	0.8440	0.0925
LOC642489	0.6325	1.3078	0.7868	0.6488	0.8440	0.1584
MRPL32	0.6527	1.0485	1.0161	0.6586	0.8440	0.1090
LOC1001297	0.8467	0.9572	0.7755	0.7969	0.8441	0.0405
GCAT	0.6427	1.0936	0.9733	0.6669	0.8441	0.1122
C9orf89	0.6920	1.1640	0.8592	0.6614	0.8441	0.1151
DOM3Z	0.7259	1.1431	0.7870	0.7206	0.8441	0.1008
JMJD8	0.6205	1.2274	0.8765	0.6523	0.8442	0.1399
ME1	0.7900	0.7600	1.2460	0.5809	0.8442	0.1416

NSDHL	0.6941	1.1231	0.8772	0.6825	0.8442	0.1031
CARS2	0.8177	1.0506	0.8530	0.6557	0.8442	0.0811
THOC7	0.6890	1.0179	1.0012	0.6689	0.8442	0.0956
LOC653453	0.7115	0.9697	0.8999	0.7960	0.8443	0.0568
ZNF383	0.8625	0.8668	0.9168	0.7309	0.8443	0.0397
NEDD1	0.7123	1.0555	0.8815	0.7278	0.8443	0.0801
METT11D1	0.6919	1.1672	0.8114	0.7066	0.8443	0.1109
PRICKLE4	0.6010	1.3291	0.8832	0.5639	0.8443	0.1766
C4orf32	0.7943	1.0198	0.8613	0.7019	0.8443	0.0670
CNOT1	0.7032	1.1071	0.9090	0.6583	0.8444	0.1032
MRPL39	0.6318	1.1229	0.9112	0.7118	0.8444	0.1098
TRIM41	0.7548	1.1475	0.8740	0.6016	0.8445	0.1154
D2HGDH	0.7335	1.1841	0.8130	0.6473	0.8445	0.1182
LMLN	0.7569	0.8913	0.7869	0.9429	0.8445	0.0436
RP5-1022P6.	0.8365	0.8076	0.9061	0.8278	0.8445	0.0214
LOC650254	0.8103	0.8769	0.8395	0.8516	0.8446	0.0138
RNF138	0.5882	1.2343	0.9421	0.6139	0.8446	0.1528
CORO2A	0.7225	1.1098	0.7664	0.7799	0.8446	0.0892
HSPA4L	0.5707	1.0010	1.1136	0.6934	0.8447	0.1274
SNORA57	0.7678	1.0454	0.8698	0.6959	0.8447	0.0758
TSFM	0.7406	1.1240	0.8182	0.6964	0.8448	0.0964
SNHG7	0.8251	1.0263	0.7943	0.7335	0.8448	0.0634
PCBD1	0.6621	1.1867	0.8800	0.6505	0.8448	0.1256
PCYT2	0.7662	1.0049	0.9066	0.7016	0.8448	0.0684
SAR1A	0.7090	0.9458	1.0298	0.6949	0.8449	0.0843
WDR5	0.7303	1.0221	0.8401	0.7870	0.8449	0.0632
RAB28	0.7960	1.0693	0.7623	0.7521	0.8449	0.0754
EIF3D	0.7089	1.1403	0.9017	0.6289	0.8449	0.1139
QPCTL	0.7024	0.9871	0.7705	0.9199	0.8450	0.0656
DKFZp667M2	0.7745	0.8906	0.9635	0.7515	0.8450	0.0499
ZNF800	0.6445	1.1030	0.9332	0.6995	0.8450	0.1063
SNTA1	0.7096	0.9925	0.8789	0.7992	0.8451	0.0601
FGL1	0.7737	0.9780	0.8887	0.7401	0.8451	0.0545
CCDC74B	0.8673	1.1487	0.6801	0.6843	0.8451	0.1102
ORC3L	0.7986	0.9751	0.9391	0.6678	0.8452	0.0703
C7orf20	0.7027	1.0558	0.9045	0.7181	0.8453	0.0838
TRNT1	0.6738	0.9563	1.0588	0.6923	0.8453	0.0961
RCAN1	0.7334	1.0926	0.7803	0.7750	0.8453	0.0831
ZRANB2	0.7184	1.0271	1.0099	0.6260	0.8454	0.1018
NUDT15	0.7159	0.9955	0.9306	0.7397	0.8454	0.0694
PGAM4	0.7741	1.0859	0.8758	0.6463	0.8455	0.0929
MXI1	0.7768	1.0238	0.8048	0.7768	0.8455	0.0598
ANKRD39	0.8493	0.9852	0.7890	0.7588	0.8456	0.0502
HPCAL4	0.6817	0.9371	0.9224	0.8413	0.8456	0.0586
ZDHHC23	0.7708	1.0288	0.8839	0.6994	0.8457	0.0719
PIH1D1	0.9183	1.0618	0.7090	0.6937	0.8457	0.0884

TSPO	0.7088	1.1744	0.8420	0.6578	0.8458	0.1162
LOC650251	0.8246	0.8752	0.9843	0.6988	0.8458	0.0592
ACSM3	0.7456	1.0900	0.8658	0.6817	0.8458	0.0899
TMEM19	0.8359	0.9891	0.9516	0.6064	0.8458	0.0862
CDK5RAP3	0.6705	1.1766	0.8483	0.6878	0.8458	0.1173
WASF2	0.6846	1.0706	0.8514	0.7768	0.8459	0.0823
BNIP1	0.6585	1.1277	0.8572	0.7405	0.8459	0.1024
LRRFIP1	0.7270	1.1177	0.8161	0.7230	0.8460	0.0931
C15orf63	0.7357	0.9896	0.8555	0.8031	0.8460	0.0538
TOB1	0.7465	1.0470	0.8531	0.7376	0.8460	0.0719
TLK1	0.6983	0.9347	1.0563	0.6951	0.8461	0.0898
PRPSAP2	0.7141	1.0497	0.9670	0.6539	0.8462	0.0959
TTC14	0.7775	1.0084	0.9377	0.6613	0.8462	0.0783
ARSK	0.7193	1.0865	0.8846	0.6945	0.8462	0.0905
LTBR	0.8151	1.1086	0.8597	0.6015	0.8462	0.1040
EIF3K	0.6724	1.1241	0.9315	0.6570	0.8462	0.1120
TRRAP	0.6909	1.1599	0.7426	0.7918	0.8463	0.1065
PAFAH1B3	0.6924	1.1823	0.8327	0.6780	0.8464	0.1173
CCDC55	0.7462	1.0129	0.8910	0.7353	0.8464	0.0659
LOC389168	0.7003	1.1500	0.8608	0.6748	0.8464	0.1092
GALNTL4	0.7360	1.1103	0.7469	0.7926	0.8465	0.0888
QRSL1	0.8384	0.9705	0.8640	0.7130	0.8465	0.0529
C7orf43	0.8254	0.9929	0.8230	0.7448	0.8465	0.0522
SUMO1P3	0.6442	0.9346	1.0116	0.7958	0.8465	0.0809
MIS12	0.6360	1.0762	0.9607	0.7137	0.8466	0.1032
MCM3APAS	0.7276	1.0330	0.8951	0.7309	0.8467	0.0734
GTF3C3	0.8210	1.0550	0.8876	0.6231	0.8467	0.0893
ANKRD49	0.7631	1.1146	0.8271	0.6821	0.8467	0.0941
BTBD3	0.7031	1.0972	0.8331	0.7535	0.8467	0.0877
ZC3H3	0.7819	1.0827	0.8171	0.7053	0.8468	0.0821
GAGE6	0.7282	1.1405	0.8781	0.6405	0.8468	0.1095
ARHGAP11B	0.7637	0.9364	0.9285	0.7587	0.8468	0.0495
LOC392437	0.6284	1.0315	1.0220	0.7056	0.8469	0.1051
ZNF485	0.7649	1.0371	0.8844	0.7013	0.8469	0.0739
HNRNPH2	0.6291	1.2198	0.7889	0.7500	0.8469	0.1288
KLHDC3	0.7098	1.0820	0.8360	0.7603	0.8470	0.0825
SETD8	0.7630	1.0781	0.8580	0.6890	0.8470	0.0844
LOC731658	0.7200	0.9202	0.8916	0.8567	0.8471	0.0443
FAM80A	0.7179	1.0236	0.8672	0.7802	0.8472	0.0663
EXOSC3	0.6508	1.1657	0.9355	0.6370	0.8472	0.1265
ATPBD4	0.7789	0.9464	0.9097	0.7542	0.8473	0.0475
THUMPD2	0.7156	1.1445	0.8963	0.6331	0.8474	0.1133
SNRPD2	0.6377	1.0877	0.9088	0.7557	0.8475	0.0974
C20orf100	0.7154	1.1584	0.8416	0.6745	0.8475	0.1096
LOC643873	0.6837	1.0536	1.0785	0.5740	0.8475	0.1283
LOC728888	0.8112	1.2074	0.6839	0.6874	0.8475	0.1236

PRPF38B	0.7161	1.1272	0.8298	0.7170	0.8475	0.0970
GMDS	0.7212	1.0871	0.9108	0.6711	0.8475	0.0951
OKL38	0.7610	1.0064	0.8771	0.7456	0.8475	0.0606
COX5B	0.6433	1.2444	0.8743	0.6283	0.8476	0.1437
LOC1001281	0.7437	0.9728	0.9033	0.7706	0.8476	0.0544
LOC647250	0.7621	0.9355	0.9530	0.7399	0.8476	0.0561
TPST2	0.7598	1.1529	0.7494	0.7284	0.8476	0.1020
SPATA2	0.7061	1.0830	0.8586	0.7429	0.8476	0.0849
NET1	0.6090	1.2464	0.8366	0.6986	0.8477	0.1409
LOC1001281	0.6877	0.9903	0.9996	0.7133	0.8477	0.0852
ZNF667	0.7745	0.9987	0.8041	0.8137	0.8477	0.0510
AMOT	0.8469	1.1626	0.6724	0.7092	0.8478	0.1115
APH1A	0.6946	1.0872	0.8500	0.7596	0.8479	0.0859
ANKRD32	0.7487	0.9956	0.9191	0.7282	0.8479	0.0652
FAF1	0.7897	1.1085	0.8643	0.6293	0.8480	0.0997
WDR46	0.7202	1.1505	0.9308	0.5905	0.8480	0.1228
PPHLN1	0.7415	1.1133	0.8627	0.6747	0.8480	0.0966
CENPB	0.6812	1.1575	0.8912	0.6623	0.8481	0.1154
XRCC2	0.6438	1.0161	0.9417	0.7908	0.8481	0.0827
HIST1H2AG	0.6506	1.0839	0.9677	0.6902	0.8481	0.1056
AACS	0.7183	1.0894	0.8819	0.7032	0.8482	0.0900
NOL8	0.7179	1.0010	0.8548	0.8193	0.8482	0.0586
PEX1	0.7382	0.9712	0.9148	0.7688	0.8483	0.0562
STXBP3	0.7270	1.1551	0.8468	0.6643	0.8483	0.1090
ATXN7L3	0.8843	1.0084	0.7779	0.7227	0.8483	0.0630
LOC647030	0.7035	0.8849	1.2721	0.5328	0.8483	0.1585
CENPH	0.7220	0.9596	0.8618	0.8503	0.8484	0.0488
HNRPK	0.6891	1.1870	0.8938	0.6238	0.8484	0.1267
CEP70	0.7813	1.0834	0.8116	0.7175	0.8484	0.0807
LOC203547	0.6494	1.1118	0.9593	0.6737	0.8485	0.1125
LOC644544	0.7851	1.0467	0.8545	0.7078	0.8485	0.0725
PKP4	0.7041	1.0624	0.8208	0.8072	0.8486	0.0759
WDR60	0.8346	0.9976	0.7446	0.8176	0.8486	0.0534
ARSB	0.7944	1.0123	0.8342	0.7536	0.8486	0.0570
CITED1	0.6848	1.0812	0.8578	0.7709	0.8487	0.0852
PKNOX2	0.8187	0.9371	0.7517	0.8873	0.8487	0.0404
HEXB	0.7137	1.2090	0.8788	0.5931	0.8487	0.1336
PPOX	0.8981	1.0977	0.7231	0.6760	0.8487	0.0958
MIR599	0.7306	0.9552	0.8759	0.8335	0.8488	0.0468
IPPK	0.7193	1.0313	0.8379	0.8073	0.8489	0.0658
PRPSAP1	0.7121	1.1352	0.9429	0.6059	0.8490	0.1185
UBA5	0.7674	1.0551	0.9347	0.6389	0.8490	0.0916
SSBP4	0.8291	1.0186	0.8152	0.7335	0.8491	0.0603
AAAS	0.7345	0.9556	0.8623	0.8440	0.8491	0.0453
PTPRG	0.7082	0.8698	1.0213	0.7975	0.8492	0.0662
MDM1	0.7414	1.0176	0.8260	0.8119	0.8492	0.0591

MBD4	0.7798	0.9988	0.8846	0.7338	0.8493	0.0590
MSN	0.6276	1.0612	0.9898	0.7187	0.8493	0.1044
C10orf47	0.7159	1.0626	0.8613	0.7576	0.8494	0.0774
UBQLN2	0.7682	1.0574	0.9847	0.5871	0.8494	0.1068
ST13	0.7496	1.0493	0.9912	0.6074	0.8494	0.1035
INTS1	0.7168	1.2745	0.7624	0.6442	0.8495	0.1438
LOC1001305	0.7820	1.0260	0.8723	0.7177	0.8495	0.0668
LOC1001286	0.6424	1.1091	1.0016	0.6450	0.8495	0.1208
BMI1	0.7400	1.3148	0.7363	0.6071	0.8496	0.1581
GALNT1	0.8457	0.9315	0.9371	0.6840	0.8496	0.0590
C16orf93	0.7305	1.1381	0.8148	0.7150	0.8496	0.0986
GNAT2	0.7523	0.9272	0.8827	0.8363	0.8496	0.0374
LOC1001292	0.6999	0.9432	1.0013	0.7542	0.8496	0.0726
EMD	0.7060	1.1463	0.9550	0.5916	0.8497	0.1246
RAVER1	0.8110	0.8861	0.8697	0.8320	0.8497	0.0172
C21orf37	0.8376	0.9744	0.8463	0.7406	0.8497	0.0480
CDK2AP1	0.7710	1.1280	0.9074	0.5925	0.8497	0.1130
KIAA0182	0.9178	1.2065	0.6421	0.6326	0.8498	0.1361
HEATR5B	0.7825	1.1787	0.7555	0.6824	0.8498	0.1117
KIAA0406	0.7047	1.0273	0.8577	0.8095	0.8498	0.0672
ADNP	0.7618	0.9254	1.0280	0.6840	0.8498	0.0778
FAM59A	0.7357	0.9832	0.9256	0.7553	0.8500	0.0616
TIMM23B	0.7819	0.9982	0.8099	0.8098	0.8500	0.0499
SLC25A26	0.6778	1.1689	0.8706	0.6826	0.8500	0.1154
RRAGC	0.6117	1.2606	0.8526	0.6752	0.8500	0.1460
ZNF343	0.7747	1.1015	0.8412	0.6827	0.8500	0.0899
CCT6B	0.7296	1.0265	1.0247	0.6196	0.8501	0.1038
ZNF143	0.7432	1.1306	0.8801	0.6469	0.8502	0.1050
CINP	0.7775	1.1229	0.8086	0.6919	0.8502	0.0942
SMYD2	0.7621	1.0052	0.8970	0.7373	0.8504	0.0624
NAT6	0.7316	0.9719	0.9130	0.7853	0.8504	0.0555
MCART1	0.7640	1.2097	0.8203	0.6082	0.8505	0.1279
TARDBP	0.7364	1.0653	0.8116	0.7888	0.8505	0.0733
RPL23AP7	0.6716	1.0969	0.8763	0.7574	0.8506	0.0922
RIMS4	0.7269	1.0615	0.7720	0.8424	0.8507	0.0742
TCTEX1D2	0.7828	1.0508	0.8821	0.6871	0.8507	0.0777
PAAF1	0.6936	1.1200	0.8860	0.7034	0.8508	0.1000
CHP	0.7377	0.8615	1.1292	0.6749	0.8508	0.1006
DPP9	0.7431	1.1790	0.7888	0.6924	0.8508	0.1112
HSDL1	0.8030	0.9651	0.8836	0.7518	0.8509	0.0468
NIP30	0.7804	1.2107	0.7937	0.6188	0.8509	0.1263
REEP1	0.7430	0.8767	0.9160	0.8679	0.8509	0.0375
RWDD3	0.8334	1.1554	0.7380	0.6771	0.8510	0.1065
FERMT1	0.7389	0.9438	0.9050	0.8163	0.8510	0.0459
CXCR3	0.8286	0.9332	0.9488	0.6933	0.8510	0.0589
RNF4	0.7648	1.1604	0.7933	0.6855	0.8510	0.1056

LOC731835	0.5905	0.8882	0.9925	0.9331	0.8510	0.0894
LOC654121	0.5730	1.1864	1.0524	0.5925	0.8511	0.1574
MRPS16	0.6707	1.2794	0.7998	0.6545	0.8511	0.1464
F8A2	0.7391	1.0179	0.8538	0.7938	0.8511	0.0603
MBLAC2	0.7070	1.0524	0.9548	0.6907	0.8512	0.0903
BUD13	0.8390	0.9305	0.9077	0.7277	0.8512	0.0455
FAM75B	0.7429	0.9842	0.9513	0.7272	0.8514	0.0676
LOC646038	0.8075	1.0960	0.7627	0.7393	0.8514	0.0828
DAZAP2	0.7951	1.0450	0.8188	0.7468	0.8514	0.0662
USP7	0.6817	1.1484	0.7896	0.7861	0.8514	0.1021
KIAA1731	0.7866	0.9728	0.9672	0.6792	0.8515	0.0719
LOC1001336	0.6898	0.9492	0.8274	0.9396	0.8515	0.0606
SNX7	0.7745	0.9603	0.9477	0.7237	0.8516	0.0601
GTF2H2	0.6843	0.9343	0.9756	0.8121	0.8516	0.0657
PMAIP1	0.7854	0.8563	0.9774	0.7873	0.8516	0.0451
E2F8	0.7410	1.0291	0.8256	0.8109	0.8516	0.0620
RRP12	0.6857	1.1549	0.8446	0.7215	0.8517	0.1066
LRIG1	0.8720	0.9914	0.9230	0.6205	0.8517	0.0809
LOC651064	0.7529	0.9699	0.9849	0.6991	0.8517	0.0735
CHERP	0.6582	1.1254	0.9331	0.6902	0.8517	0.1099
FBXO8	0.6730	1.0381	1.0505	0.6454	0.8517	0.1113
GEMIN8	0.7675	1.0623	0.8966	0.6805	0.8517	0.0830
POLR2I	0.6424	1.2018	0.8566	0.7062	0.8517	0.1250
DEAF1	0.8636	1.0475	0.8014	0.6945	0.8517	0.0740
LOC1001320	0.7210	0.9750	0.7967	0.9143	0.8517	0.0572
ARSD	0.8630	1.0459	0.7321	0.7659	0.8518	0.0704
ARF4	0.7009	1.2817	0.8249	0.5997	0.8518	0.1505
LOC728739	0.7335	1.1428	0.8138	0.7172	0.8518	0.0993
PRKCD	0.7531	1.2153	0.7393	0.6997	0.8518	0.1217
COPG	0.8059	0.9825	0.9428	0.6763	0.8519	0.0697
MAGEA3	0.7208	1.0397	0.9542	0.6928	0.8519	0.0857
TMEM56	0.7622	0.9239	0.9325	0.7891	0.8519	0.0444
KCTD5	0.7855	1.1036	0.8163	0.7025	0.8520	0.0872
QSOX2	0.7059	1.1366	0.8605	0.7049	0.8520	0.1017
GOLPH3	0.7142	1.0029	1.0707	0.6203	0.8520	0.1093
RAD23B	0.7923	1.1342	0.9019	0.5802	0.8522	0.1153
LRRC20	0.7795	1.1534	0.8363	0.6395	0.8522	0.1086
ASF1A	0.6598	1.1469	0.9426	0.6598	0.8522	0.1187
HSPBP1	0.7624	1.0544	0.8807	0.7118	0.8523	0.0761
C1orf56	0.9295	1.0371	0.7518	0.6909	0.8523	0.0797
INO80C	0.6927	1.3029	0.7936	0.6202	0.8524	0.1543
SLC25A35	0.8465	0.9404	0.8508	0.7720	0.8524	0.0345
GPR98	0.8459	1.0752	0.7368	0.7518	0.8524	0.0781
MCCC2	0.5892	1.1275	0.9961	0.6970	0.8524	0.1257
MAGOH	0.6774	1.1305	0.8884	0.7137	0.8525	0.1035
LRFN1	0.7215	0.9349	0.8841	0.8693	0.8525	0.0459

URB2	0.6778	1.1946	0.8312	0.7064	0.8525	0.1188
LOC730744	0.6892	1.1086	0.8848	0.7274	0.8525	0.0953
ZNF679	0.7199	1.0894	0.9199	0.6813	0.8526	0.0947
CUTC	0.6597	1.1114	0.9223	0.7172	0.8526	0.1030
RNY1	0.6708	1.0081	0.9691	0.7631	0.8528	0.0810
RNF34	0.7074	1.0479	0.9461	0.7097	0.8528	0.0858
MRPL27	0.7457	1.0659	0.9092	0.6903	0.8528	0.0849
SENP2	0.7640	1.1233	0.8041	0.7201	0.8529	0.0918
GTPBP6	0.7010	1.1686	0.9203	0.6219	0.8530	0.1227
PIAS2	0.6570	1.2392	0.7875	0.7282	0.8530	0.1315
VAV2	0.8132	1.0522	0.8096	0.7370	0.8530	0.0687
LOC729774	0.5902	1.0154	0.9789	0.8275	0.8530	0.0966
KATNB1	0.7481	1.1683	0.8603	0.6354	0.8530	0.1147
GDF11	0.7660	1.0243	0.8686	0.7532	0.8530	0.0627
FAM100A	0.7468	0.9441	0.8916	0.8297	0.8531	0.0424
LOC653566	0.7746	1.0041	0.9682	0.6654	0.8531	0.0803
NR2C1	0.7360	0.9923	0.8922	0.7921	0.8532	0.0565
PRUNE	0.8205	1.1071	0.7297	0.7556	0.8532	0.0867
ZNF165	0.6979	1.0809	0.9003	0.7338	0.8532	0.0878
GRK6	0.7882	1.0812	0.8126	0.7310	0.8533	0.0779
RIOK2	0.7124	1.1335	0.9196	0.6476	0.8533	0.1099
UXT	0.6365	1.2106	0.8615	0.7046	0.8533	0.1281
NR2F2	0.8182	0.8630	0.9747	0.7573	0.8533	0.0459
C8orf48	0.8861	1.0179	0.8284	0.6809	0.8533	0.0698
SLC25A14	0.7711	1.1158	0.8747	0.6519	0.8534	0.0986
CYCSL1	0.5606	1.1266	1.0642	0.6623	0.8534	0.1418
DNA2	0.7606	1.0059	0.8463	0.8010	0.8534	0.0537
STX7	0.8237	0.9258	0.9219	0.7429	0.8536	0.0438
PDCD6	0.7865	1.0646	0.9055	0.6578	0.8536	0.0866
CCDC50	0.7159	1.0810	0.9651	0.6523	0.8536	0.1015
PHLDB1	0.8862	1.0750	0.7703	0.6830	0.8536	0.0847
BBS10	0.7099	1.1642	0.8389	0.7018	0.8537	0.1081
CSNK2A1	0.6893	1.1802	0.8536	0.6918	0.8537	0.1154
LOC1001329	0.7596	0.9528	0.8998	0.8026	0.8537	0.0442
DHRS2	0.6513	0.9014	0.9725	0.8897	0.8538	0.0699
EIF5B	0.6237	1.1115	0.9293	0.7506	0.8538	0.1064
LOC401127	0.7874	0.9503	0.8994	0.7783	0.8538	0.0423
LOC387703	0.8572	1.0857	0.8815	0.5913	0.8539	0.1014
UBE2L3	0.8602	0.9717	0.8510	0.7330	0.8540	0.0488
RPL7L1	0.6237	1.3372	0.8032	0.6519	0.8540	0.1658
LPIN1	0.6839	1.1397	0.9048	0.6878	0.8540	0.1083
TMEM189-U	0.6524	1.1530	0.9154	0.6956	0.8541	0.1151
ATF6	0.6775	1.0570	0.9729	0.7090	0.8541	0.0946
NPL	0.7528	1.0558	0.9006	0.7074	0.8542	0.0789
DBNL	0.7314	1.1390	0.8687	0.6778	0.8542	0.1031
C5orf51	0.7266	1.1469	0.8172	0.7265	0.8543	0.0998

DSG2	0.7334	1.1108	0.9006	0.6725	0.8543	0.0982
KREMEN2	0.8116	1.0142	0.8014	0.7901	0.8543	0.0535
SCFD2	0.6764	0.9550	1.0718	0.7143	0.8544	0.0952
NCRNA0008!	0.8584	0.9400	0.9220	0.6972	0.8544	0.0552
KLHL17	0.7212	1.0785	0.8036	0.8142	0.8544	0.0775
VRK2	0.7332	0.9277	1.0659	0.6910	0.8544	0.0873
WDR92	0.6967	0.9889	0.9003	0.8323	0.8545	0.0616
LOC644584	0.8385	1.1377	0.7470	0.6951	0.8546	0.0989
LOC143666	0.8729	1.1289	0.7276	0.6892	0.8546	0.0996
LOC729769	0.7480	0.9464	1.0780	0.6462	0.8547	0.0971
LOC645452	0.6644	1.1663	0.9342	0.6538	0.8547	0.1225
FBXO30	0.7700	0.9872	0.9072	0.7546	0.8548	0.0559
LOC1001332	0.7536	0.9804	0.8852	0.7999	0.8548	0.0500
KDELC1	0.8703	0.9711	0.8810	0.6970	0.8548	0.0573
PHB	0.7446	1.1234	0.9098	0.6416	0.8549	0.1052
KLHL9	0.7938	1.0025	0.9814	0.6419	0.8549	0.0851
LOC642236	0.8162	1.0039	0.9559	0.6440	0.8550	0.0808
PAN3	0.7072	1.1852	0.7553	0.7726	0.8551	0.1109
DAGLA	0.8193	1.0665	0.7892	0.7453	0.8551	0.0721
METRN	0.6494	1.0729	0.9972	0.7011	0.8552	0.1055
C12orf30	0.6668	1.1523	0.8853	0.7163	0.8552	0.1095
STK11IP	0.8665	0.9158	0.7793	0.8593	0.8552	0.0283
PPP2R3C	0.7094	1.0412	0.9749	0.6954	0.8552	0.0893
PPP4R1	0.7256	1.1594	0.8972	0.6387	0.8552	0.1147
RGPD6	0.8711	0.8849	0.8290	0.8359	0.8552	0.0135
LOC1001709	0.8614	1.1651	0.6758	0.7187	0.8553	0.1106
C21orf59	0.7377	1.1098	0.8693	0.7043	0.8553	0.0920
LOC645733	0.9046	0.9385	0.7810	0.7971	0.8553	0.0390
LOC1001338	0.7240	0.8554	1.1910	0.6510	0.8553	0.1196
DDX54	0.7288	1.0395	0.8799	0.7732	0.8554	0.0691
EIF3G	0.6546	1.1808	0.9300	0.6561	0.8554	0.1263
FGD3	0.7821	1.0516	0.8221	0.7657	0.8554	0.0665
EAPP	0.7120	0.9421	1.1198	0.6477	0.8554	0.1084
MLLT11	0.7657	1.0766	0.8772	0.7026	0.8555	0.0821
BAIAP2L1	0.6987	1.2218	0.8278	0.6739	0.8555	0.1267
DYNLT3	0.7759	0.9412	0.9374	0.7679	0.8556	0.0484
HSD11B1L	0.8587	1.0403	0.7965	0.7271	0.8556	0.0672
CHST3	0.7847	1.0249	0.7823	0.8308	0.8557	0.0575
PREI3	0.7237	1.1876	0.8894	0.6221	0.8557	0.1236
TCP11L1	0.7596	0.9774	0.8544	0.8317	0.8558	0.0453
MRPS24	0.6695	1.2203	0.7902	0.7431	0.8558	0.1240
STK4	0.7223	1.0480	0.8464	0.8064	0.8558	0.0691
ATP6V1D	0.7058	1.0067	0.9744	0.7362	0.8558	0.0783
GTF2A1	0.7968	0.9820	0.8846	0.7597	0.8558	0.0496
SACM1L	0.7040	1.0773	1.0444	0.5974	0.8558	0.1206
KIAA1161	0.8063	1.0077	0.9219	0.6874	0.8558	0.0697

LOC1001339	0.6858	1.0072	0.9072	0.8232	0.8559	0.0680
BPNT1	0.6780	0.9804	1.0512	0.7141	0.8559	0.0937
CEP63	0.6931	1.0658	0.8849	0.7801	0.8560	0.0802
CYTH3	0.7780	1.1147	0.7307	0.8005	0.8560	0.0875
SRD5A1	0.7584	1.0467	0.9438	0.6752	0.8560	0.0848
LOC728620	0.7026	1.0187	1.0033	0.6996	0.8561	0.0895
HOOK2	0.8173	1.2385	0.7078	0.6606	0.8561	0.1316
ZNF215	0.8030	0.9164	0.9429	0.7621	0.8561	0.0436
RALA	0.9217	1.0501	0.8716	0.5810	0.8561	0.0991
LRPPRC	0.6217	1.3261	0.7897	0.6870	0.8561	0.1604
LOC1001339	0.7898	1.0088	0.8016	0.8244	0.8562	0.0514
CCT4	0.6773	0.9587	1.0391	0.7497	0.8562	0.0853
GTPBP4	0.6568	1.1308	0.9071	0.7305	0.8563	0.1055
ZFP161	0.8782	1.0040	0.8227	0.7203	0.8563	0.0591
LOC642458	0.7722	0.9437	0.8865	0.8228	0.8563	0.0373
HSPA13	0.9036	0.9822	0.7184	0.8212	0.8563	0.0565
MFSD5	0.8406	1.1694	0.7763	0.6391	0.8564	0.1125
HNRPUL1	0.7338	1.0380	0.9055	0.7482	0.8564	0.0720
MITD1	0.6290	1.1100	0.8872	0.7995	0.8564	0.1001
UTP3	0.7359	0.9801	1.0093	0.7007	0.8565	0.0803
SEC63	0.7076	1.0465	0.8251	0.8471	0.8565	0.0703
SNORA9	0.8061	0.9741	0.8663	0.7797	0.8565	0.0432
TMEM184A	0.8233	1.0000	0.8770	0.7259	0.8566	0.0571
STRADA	0.8434	1.1276	0.8055	0.6499	0.8566	0.0996
SLC35F5	0.6908	1.1772	0.8973	0.6611	0.8566	0.1191
RAD51L3	0.7362	1.0271	0.8862	0.7769	0.8566	0.0651
FTSJ2	0.6782	1.1129	0.9316	0.7040	0.8567	0.1026
ERICH1	0.6778	1.1082	0.9435	0.6974	0.8567	0.1034
WBSCR27	0.8149	1.0498	0.9183	0.6439	0.8567	0.0857
PALB2	0.8361	1.0073	0.8021	0.7814	0.8567	0.0514
MAP3K8	0.7346	1.0214	0.8426	0.8286	0.8568	0.0599
SASS6	0.7618	0.9917	0.8718	0.8019	0.8568	0.0504
MAP2K4	0.7887	0.9585	1.0070	0.6730	0.8568	0.0771
ANO6	0.9123	0.9682	0.7831	0.7637	0.8568	0.0496
IRF2BP2	0.6747	1.1636	0.8541	0.7349	0.8568	0.1089
COMTD1	0.9939	0.9278	0.7525	0.7531	0.8568	0.0616
WBSCR22	0.7159	1.1922	0.8150	0.7044	0.8569	0.1145
SLC25A46	0.7154	0.9909	1.0364	0.6849	0.8569	0.0912
NLGN2	0.8403	0.9282	0.8197	0.8395	0.8569	0.0242
IQCK	0.8186	0.9473	0.9314	0.7304	0.8569	0.0510
LOC1001332	0.7447	0.9007	1.0189	0.7633	0.8569	0.0642
C18orf32	0.7191	0.9860	1.0259	0.6967	0.8569	0.0865
MNAT1	0.7265	0.9129	1.0452	0.7436	0.8570	0.0755
MCTP2	0.6859	1.2122	0.8230	0.7072	0.8571	0.1221
TMEM64	0.7545	0.9644	0.9447	0.7648	0.8571	0.0564
LOC650826	0.8294	1.0971	0.8241	0.6778	0.8571	0.0874

SIRPA	0.8509	0.9573	0.7992	0.8215	0.8572	0.0350
RP9	0.6569	1.1701	0.7560	0.8463	0.8573	0.1112
PSME1	0.6481	1.1511	0.9575	0.6726	0.8573	0.1205
PFKFB4	0.8809	1.0126	0.8016	0.7345	0.8574	0.0598
GAGE12E	0.5174	1.1597	0.9947	0.7580	0.8574	0.1402
UPF1	0.7016	1.2122	0.7544	0.7617	0.8575	0.1190
MAP7	0.6977	1.1471	0.8319	0.7532	0.8575	0.1004
GLTPD1	0.6812	1.1474	0.8529	0.7485	0.8575	0.1029
LARP7	0.6967	1.1564	0.9126	0.6644	0.8575	0.1138
LOC727758	0.7281	1.0956	0.9007	0.7058	0.8576	0.0905
PDE6D	0.8236	1.0185	0.8555	0.7326	0.8576	0.0596
TBL3	0.7867	1.0134	0.8540	0.7764	0.8576	0.0547
DHRSX	0.8708	0.9981	0.7918	0.7699	0.8577	0.0516
EIF4ENIF1	0.6164	1.2226	0.9121	0.6797	0.8577	0.1372
SAMD1	0.6700	1.0526	0.9197	0.7886	0.8577	0.0826
CECR4	0.7699	1.0665	0.8234	0.7711	0.8577	0.0707
PTPLAD1	0.6748	1.1435	0.9717	0.6410	0.8577	0.1208
SLC3A2	0.6844	1.1117	0.8739	0.7611	0.8578	0.0932
LOC729317	0.5818	1.1850	1.0413	0.6233	0.8579	0.1505
GAPDH	0.7016	1.1174	0.9431	0.6692	0.8579	0.1059
LOC649991	0.8490	0.9409	0.9562	0.6854	0.8579	0.0622
LOC653308	0.8138	1.0447	0.9103	0.6627	0.8579	0.0805
AKAP10	0.8338	1.0663	0.7508	0.7807	0.8579	0.0715
FANCF	0.7188	0.8925	0.9262	0.8942	0.8579	0.0470
TXLNA	0.8457	1.1777	0.7655	0.6434	0.8581	0.1144
PECR	0.6791	1.1810	0.9355	0.6369	0.8581	0.1262
MRPL34	0.7654	1.1287	0.8364	0.7020	0.8581	0.0943
DDRGK1	0.5933	1.3186	0.8583	0.6624	0.8582	0.1634
CRTC2	0.7751	1.0446	0.7986	0.8145	0.8582	0.0627
TFB1M	0.7040	1.2084	0.9081	0.6124	0.8582	0.1321
DET1	0.7624	1.0523	0.8228	0.7955	0.8583	0.0658
PDP2	0.6446	1.0813	0.8720	0.8353	0.8583	0.0895
UCHL3	0.7178	1.1809	0.9042	0.6304	0.8583	0.1217
FHL3	0.7202	1.0116	0.8612	0.8403	0.8583	0.0598
PANK2	0.6855	1.0915	0.9325	0.7238	0.8583	0.0948
IGF2R	0.8988	1.3455	0.6054	0.5837	0.8584	0.1776
ZBTB24	0.6969	1.3048	0.7402	0.6917	0.8584	0.1492
DTWD2	0.6164	1.0244	1.0842	0.7091	0.8585	0.1153
MPHOSPH8	0.7085	1.2608	0.7996	0.6652	0.8585	0.1370
GK	0.6976	1.1116	0.8821	0.7430	0.8586	0.0930
TRIB3	0.8229	1.1152	0.8819	0.6147	0.8586	0.1029
C16orf57	0.8676	1.0866	0.7511	0.7294	0.8587	0.0818
ATP5E	0.6624	1.2619	0.8276	0.6830	0.8587	0.1393
MFSD10	0.7276	1.2388	0.7984	0.6702	0.8587	0.1294
SLC1A1	0.7969	0.9271	0.9048	0.8063	0.8588	0.0334
MRPL47	0.8169	1.0523	0.9885	0.5776	0.8588	0.1061

DDX52	0.8032	0.9023	0.9725	0.7580	0.8590	0.0484
EIF4H	0.7438	1.2324	0.7928	0.6672	0.8590	0.1271
LOC648570	0.7800	0.8424	0.9657	0.8482	0.8591	0.0388
ICT1	0.7222	1.0514	0.9347	0.7281	0.8591	0.0809
AEBP2	0.7581	1.1130	0.9050	0.6602	0.8591	0.0985
MIR300	0.6975	1.0475	0.8879	0.8036	0.8591	0.0739
HPCAL1	0.7126	1.1192	0.8865	0.7184	0.8592	0.0956
APOLD1	0.8438	1.0446	0.8744	0.6738	0.8592	0.0759
ARL6IP6	0.7735	1.1435	0.8338	0.6859	0.8592	0.0995
LOC643233	0.7162	1.0184	0.8469	0.8553	0.8592	0.0619
MGC35361	0.7843	1.1666	0.7282	0.7578	0.8592	0.1031
RSL24D1	0.6746	1.1255	1.0571	0.5799	0.8593	0.1361
RNF181	0.8028	1.0654	0.9397	0.6294	0.8593	0.0935
CCT8	0.6395	1.0613	1.0481	0.6886	0.8594	0.1133
GOLT1B	0.8566	1.0073	0.8840	0.6897	0.8594	0.0654
SLC7A7	0.7575	1.0412	0.8977	0.7414	0.8594	0.0700
ZBTB41	0.7946	1.1150	0.7874	0.7406	0.8594	0.0860
TTC18	0.7796	1.0147	0.8067	0.8369	0.8595	0.0531
BRD1	0.8075	0.9756	0.9219	0.7333	0.8596	0.0548
UBE2D2	0.7356	1.0065	0.9372	0.7590	0.8596	0.0665
FLJ45337	0.7035	1.1231	0.9596	0.6522	0.8596	0.1106
PIM1	0.8612	0.9472	0.8421	0.7878	0.8596	0.0331
SNRNP48	0.7685	1.0891	0.9004	0.6803	0.8596	0.0889
ZC3H18	0.7384	0.9275	0.9152	0.8574	0.8596	0.0432
MGAT4B	0.8254	1.0757	0.7623	0.7752	0.8597	0.0733
ABTB2	0.8043	1.0671	0.8558	0.7117	0.8597	0.0753
C11orf58	0.7818	1.1136	0.8070	0.7366	0.8597	0.0859
PIGY	0.6815	1.0792	0.9815	0.6969	0.8598	0.1005
IQCC	0.7426	1.1634	0.8959	0.6374	0.8598	0.1143
DDIT4	0.8994	1.1017	0.7858	0.6524	0.8598	0.0951
UBE2G1	0.8535	1.0821	0.8621	0.6415	0.8598	0.0900
SHANK3	0.7606	1.1276	0.8515	0.6998	0.8599	0.0945
PRPF38A	0.8172	1.1544	0.8456	0.6226	0.8599	0.1100
PICALM	0.6997	1.1580	0.9256	0.6565	0.8599	0.1155
SNORA64	0.6790	1.0282	0.9013	0.8313	0.8600	0.0728
LOC644517	0.7265	1.0455	0.8801	0.7878	0.8600	0.0694
PRKAG2	0.7594	1.0073	0.9358	0.7380	0.8601	0.0661
NDUFB9	0.7482	1.0597	1.0171	0.6155	0.8601	0.1068
METTL11A	0.6698	1.0968	0.9216	0.7523	0.8601	0.0947
SDHAF1	0.7542	1.2252	0.8038	0.6573	0.8601	0.1254
GNAI2	0.9112	1.0411	0.8171	0.6712	0.8601	0.0779
PHF19	0.7977	1.0655	0.8166	0.7609	0.8602	0.0694
C9orf23	0.7166	1.1182	0.9060	0.6999	0.8602	0.0979
TET1	0.7809	0.8832	0.8700	0.9067	0.8602	0.0275
CYCS	0.7766	1.1296	0.8416	0.6931	0.8602	0.0948
TSPYL6	0.7470	0.8981	1.0386	0.7575	0.8603	0.0687

TIPRL	0.6595	1.1411	0.9240	0.7168	0.8604	0.1095
MED22	0.6889	1.3200	0.7757	0.6570	0.8604	0.1552
NAF1	0.6889	1.1233	0.8477	0.7816	0.8604	0.0935
C7orf40	0.5962	1.1470	1.0019	0.6966	0.8604	0.1287
C10orf137	0.7556	1.0424	0.9380	0.7056	0.8604	0.0786
CAP1	0.7532	1.1121	0.8467	0.7299	0.8605	0.0876
COPS7B	0.7557	1.1119	0.7961	0.7782	0.8605	0.0842
SLC35B2	0.8386	1.1624	0.8326	0.6086	0.8606	0.1140
C11orf51	0.7426	0.9991	1.0088	0.6917	0.8606	0.0835
TUBB4	0.7152	1.1766	0.8073	0.7432	0.8606	0.1071
TCHP	0.7603	1.0530	0.9386	0.6905	0.8606	0.0827
ZBTB17	0.8468	1.2240	0.5721	0.7998	0.8607	0.1351
MAL2	0.6999	1.0780	0.9107	0.7541	0.8607	0.0851
NMB	0.7874	0.9516	0.8981	0.8056	0.8607	0.0388
TCF19	0.7814	0.9841	0.8534	0.8240	0.8607	0.0437
TRIM66	0.6781	1.0336	1.0043	0.7270	0.8607	0.0921
C20orf177	0.5952	1.1971	0.8729	0.7779	0.8608	0.1261
CYB561D2	0.6895	1.1108	0.9269	0.7159	0.8608	0.0988
TRIM65	0.7992	1.0236	0.8232	0.7972	0.8608	0.0546
NFS1	0.7521	1.0842	0.8622	0.7447	0.8608	0.0792
B3GAT3	0.7603	0.9834	0.7792	0.9204	0.8609	0.0543
ZC3H14	0.7624	1.0321	0.9058	0.7432	0.8609	0.0676
GAGE12D	0.7506	0.9054	1.0983	0.6895	0.8609	0.0912
IFT20	0.6398	0.9986	1.0163	0.7891	0.8609	0.0900
SOCS4	0.7112	1.0457	0.9319	0.7551	0.8610	0.0779
FASTKD3	0.6642	1.2334	0.8139	0.7323	0.8610	0.1279
MED7	0.7913	1.0259	0.8885	0.7383	0.8610	0.0631
NEURL1B	0.7752	1.1117	0.8280	0.7293	0.8611	0.0859
RAB15	0.8254	0.9794	0.8792	0.7604	0.8611	0.0463
HLA-E	0.6764	1.1225	0.8942	0.7515	0.8611	0.0981
LOC728554	0.9038	1.1148	0.8770	0.5493	0.8612	0.1168
EDC4	0.8065	1.1485	0.7949	0.6951	0.8612	0.0990
HSP90AB1	0.7882	1.1481	0.8953	0.6134	0.8613	0.1119
EDG7	0.5821	1.2351	0.9420	0.6858	0.8613	0.1458
TUT1	0.9231	1.0215	0.8226	0.6783	0.8614	0.0733
C14orf133	0.8083	0.9936	0.8938	0.7498	0.8614	0.0531
SNORA63	0.6881	1.0543	0.9432	0.7600	0.8614	0.0838
B4GALNT1	0.8309	0.9397	0.7984	0.8769	0.8614	0.0306
MRPL53	0.7820	1.1577	0.8498	0.6563	0.8615	0.1066
PHYH	0.8143	1.0324	0.8989	0.7007	0.8616	0.0699
KLHL5	0.7359	1.0541	0.9355	0.7209	0.8616	0.0807
LOC152024	0.7759	0.9706	0.9658	0.7341	0.8616	0.0621
RPN2	0.7967	0.9730	1.0042	0.6727	0.8616	0.0778
USP21	0.7511	1.0717	0.9001	0.7237	0.8616	0.0800
PNMA3	0.7600	0.9589	0.9099	0.8178	0.8617	0.0448
FFAR2	0.7889	0.9825	0.8322	0.8430	0.8617	0.0419

LOC647121	0.8481	0.8680	0.9084	0.8222	0.8617	0.0182
POFUT1	0.6419	1.2088	0.9138	0.6825	0.8617	0.1303
RNF125	0.7560	1.0546	0.8659	0.7704	0.8617	0.0688
CLK1	0.7888	1.0823	0.8098	0.7661	0.8618	0.0741
SH3BGRL	0.8704	0.9603	0.9271	0.6892	0.8618	0.0605
NDRG2	0.6649	1.2619	0.8428	0.6777	0.8618	0.1394
C8orf51	0.7329	1.0435	0.9159	0.7550	0.8618	0.0730
GPR63	0.7497	1.0180	0.8675	0.8121	0.8619	0.0573
HEATR1	0.6529	1.2855	0.7768	0.7323	0.8619	0.1435
LOC729692	0.6856	1.0291	0.9525	0.7808	0.8620	0.0784
PRSS1	0.8301	0.9471	0.8190	0.8519	0.8620	0.0292
ST7	0.7014	1.2278	0.9147	0.6043	0.8621	0.1381
MPPE1	0.8811	1.0514	0.8509	0.6650	0.8621	0.0791
RPS6KA4	0.9844	0.9607	0.7382	0.7652	0.8621	0.0642
ITGB4BP	0.6534	1.1474	0.9713	0.6763	0.8621	0.1195
PTRH2	0.8003	1.1044	0.8455	0.6984	0.8621	0.0864
PCYOX1L	0.8239	1.0316	0.9194	0.6739	0.8622	0.0758
ETNK1	0.7133	1.0520	1.0012	0.6824	0.8622	0.0957
NCAPH2	0.7603	0.9989	0.9008	0.7889	0.8622	0.0547
KDELR2	0.7093	1.2244	0.9300	0.5852	0.8622	0.1402
ZNF318	0.7597	1.0507	0.8403	0.7985	0.8623	0.0649
LOC390876	0.8316	0.8628	0.9189	0.8360	0.8623	0.0201
PYGL	0.8645	0.9663	0.8606	0.7579	0.8623	0.0426
SOD1	0.6648	1.1115	1.0224	0.6507	0.8624	0.1195
SYCE2	0.7834	0.9216	0.9433	0.8014	0.8624	0.0408
DDX56	0.7985	1.0847	0.8315	0.7350	0.8624	0.0767
LUC7L2	0.7922	0.8802	0.9473	0.8301	0.8625	0.0335
MIF	0.7262	1.0549	0.9580	0.7109	0.8625	0.0855
VPS33A	0.6911	1.1751	0.8069	0.7770	0.8625	0.1070
ATXN7L2	0.7744	0.9964	0.7873	0.8921	0.8625	0.0518
SEPX1	0.6860	1.2380	0.8017	0.7245	0.8625	0.1275
UTP6	0.7473	1.0360	0.9774	0.6895	0.8626	0.0849
ST7OT1	0.7879	0.8532	0.9280	0.8812	0.8626	0.0293
PSMB6	0.6747	1.2946	0.7741	0.7068	0.8626	0.1455
TMEM17	0.6973	1.1361	0.9089	0.7079	0.8626	0.1034
GAGE12H	0.7608	1.1782	0.8783	0.6331	0.8626	0.1165
RAD9A	0.7691	1.0040	0.8773	0.8002	0.8626	0.0523
LSM5	0.6835	1.2264	0.9167	0.6239	0.8627	0.1367
SFT2D2	0.7642	1.0138	0.8971	0.7756	0.8627	0.0587
NDUFA12	0.7605	1.1273	0.9179	0.6450	0.8627	0.1044
LOC648682	0.7847	1.0921	0.8460	0.7280	0.8627	0.0802
GOLGA5	0.7251	1.0936	0.9210	0.7112	0.8627	0.0906
GLRX2	0.7316	1.1090	0.9782	0.6323	0.8628	0.1097
KBTBD2	0.7068	1.3124	0.7330	0.6991	0.8628	0.1500
SBNO1	0.7578	0.8803	0.9531	0.8601	0.8628	0.0403
POLR2L	0.7222	1.0902	0.9073	0.7317	0.8629	0.0869

HSPA9	0.6852	1.2161	0.8439	0.7065	0.8629	0.1229
SNTB2	0.6017	1.3559	0.9001	0.5939	0.8629	0.1791
GPR89B	0.6083	1.1305	0.9818	0.7313	0.8630	0.1183
PIGA	0.7725	1.0721	0.8889	0.7185	0.8630	0.0782
DUSP9	0.8393	0.9035	0.9096	0.7998	0.8631	0.0264
HGSNAT	0.7372	1.0650	0.9170	0.7333	0.8631	0.0798
MED9	0.8396	0.9455	1.0290	0.6384	0.8631	0.0843
UBXN1	0.6926	1.2659	0.8439	0.6505	0.8632	0.1405
SAP30	0.7772	0.9165	0.9996	0.7597	0.8632	0.0574
GPR19	0.8189	0.9642	0.8388	0.8311	0.8632	0.0339
MGC12982	0.8071	0.8998	0.9391	0.8070	0.8633	0.0334
MAN2A1	0.7559	0.9384	0.9481	0.8108	0.8633	0.0476
AURKAIP1	0.7830	1.1349	0.9241	0.6111	0.8633	0.1109
TCEA1	0.6822	1.0888	0.9883	0.6939	0.8633	0.1033
TRAM1	0.6976	1.1220	0.9299	0.7037	0.8633	0.1018
MRPS36	0.8046	1.0375	0.8677	0.7436	0.8634	0.0633
NDUFB7	0.7166	1.2202	0.8866	0.6301	0.8634	0.1303
TTC26	0.8044	0.9889	0.9592	0.7009	0.8634	0.0676
LOC1001309	0.7823	0.9371	1.0322	0.7020	0.8634	0.0745
SAMD11	0.8340	0.9485	0.9326	0.7386	0.8634	0.0487
SUB1	0.7478	1.0478	1.0265	0.6317	0.8634	0.1032
SEC24B	0.8151	1.0140	0.9170	0.7077	0.8635	0.0659
LOC1001288	0.6868	1.0844	0.8978	0.7849	0.8635	0.0853
CHCHD10	0.8203	1.1767	0.7736	0.6837	0.8636	0.1082
NME7	0.7068	1.1377	0.9351	0.6747	0.8636	0.1082
ABT1	0.8769	1.0023	0.7398	0.8354	0.8636	0.0544
FAM184A	0.8405	1.0000	0.7998	0.8141	0.8636	0.0462
LOC1001446	0.8973	1.0025	0.9770	0.5777	0.8636	0.0979
SNORA22	0.6725	1.0291	1.0013	0.7517	0.8637	0.0892
RRP8	0.7007	1.2121	0.9114	0.6307	0.8637	0.1306
DPY19L4	0.7040	1.0276	0.9377	0.7857	0.8637	0.0730
CAND1	0.6639	1.0474	1.0608	0.6830	0.8638	0.1100
NUAK1	0.7528	1.0533	0.8950	0.7543	0.8639	0.0714
EIF4G2	0.7151	1.0854	0.9863	0.6686	0.8639	0.1018
TNPO1	0.6740	1.0941	0.9425	0.7449	0.8639	0.0955
DNAJC3	0.7878	0.9969	0.9276	0.7435	0.8640	0.0592
MAT2B	0.7500	1.0479	0.9122	0.7458	0.8640	0.0725
MKRN2	0.7722	1.1565	0.7756	0.7517	0.8640	0.0976
RSRC2	0.7801	1.1453	0.8575	0.6733	0.8641	0.1011
FAHD2B	0.7503	1.1571	0.9087	0.6403	0.8641	0.1121
LOC729992	0.6502	1.2620	0.8783	0.6661	0.8641	0.1424
PROK1	0.8097	0.9320	0.9927	0.7222	0.8641	0.0607
TMEM39B	0.7825	1.0756	0.9484	0.6503	0.8642	0.0932
LOC651511	0.7277	0.9627	0.9877	0.7790	0.8643	0.0651
ADPGK	0.8096	1.1108	0.8310	0.7058	0.8643	0.0866
TRMT1	0.6778	1.2913	0.7962	0.6920	0.8643	0.1447

LOC1001292	0.9501	0.8739	0.8122	0.8213	0.8644	0.0317
C21orf55	0.7082	1.1550	1.0149	0.5794	0.8644	0.1331
ZNF146	0.8522	0.9782	0.9377	0.6896	0.8644	0.0639
LOC1001338	0.6240	1.3303	0.6998	0.8037	0.8644	0.1596
CCDC49	0.6709	1.1092	0.9065	0.7714	0.8645	0.0948
TST	0.7111	1.1501	0.9162	0.6804	0.8645	0.1086
LOC727825	0.7534	0.9744	1.0321	0.6981	0.8645	0.0818
UBE4B	0.7393	1.1507	0.8667	0.7014	0.8645	0.1017
AP1M1	0.8326	1.0603	0.9613	0.6041	0.8646	0.0985
SH3BP4	0.8762	1.0837	0.7288	0.7696	0.8646	0.0794
SLC25A12	0.8136	0.9788	0.8840	0.7821	0.8646	0.0436
AURKAPS1	0.8691	0.9655	0.7977	0.8262	0.8646	0.0367
KIAA0753	0.9403	0.8605	0.8399	0.8178	0.8646	0.0267
DUXAP3	0.6911	1.1025	0.9181	0.7469	0.8647	0.0928
LOC652190	0.8756	0.9669	0.9438	0.6725	0.8647	0.0669
NUBP2	0.8180	1.0455	0.8760	0.7193	0.8647	0.0684
CRK	0.7636	1.2273	0.7775	0.6905	0.8647	0.1224
XKR8	0.8522	0.9192	0.9282	0.7595	0.8648	0.0390
LMOD3	0.8004	1.1486	0.8528	0.6574	0.8648	0.1032
C14orf166	0.6904	1.0450	1.1267	0.5973	0.8649	0.1301
LOC646531	0.6101	1.1620	0.9895	0.6980	0.8649	0.1280
LOC728188	0.5465	1.3646	0.8582	0.6903	0.8649	0.1783
CFL2	0.7623	0.9631	0.8968	0.8375	0.8649	0.0427
EXOSC7	0.7677	1.1096	0.8630	0.7201	0.8651	0.0867
TGFBRAP1	0.7995	0.9945	0.7608	0.9055	0.8651	0.0529
LOC728715	0.7881	0.9996	0.8356	0.8370	0.8651	0.0463
PDF	0.7763	0.9734	0.9393	0.7714	0.8651	0.0532
FAM49B	0.8689	0.9170	1.0102	0.6644	0.8651	0.0730
VPS24	0.6768	1.0851	1.0089	0.6897	0.8651	0.1062
LOC1001278	0.8834	1.0782	0.8167	0.6823	0.8651	0.0824
PPP2R2A	0.6949	1.0955	0.8585	0.8118	0.8652	0.0841
PATL1	0.7632	1.2116	0.8018	0.6842	0.8652	0.1180
NUDT16P	0.7295	1.0548	0.9167	0.7598	0.8652	0.0754
STAG3L1	0.7372	1.1304	0.8546	0.7386	0.8652	0.0926
ZC3H12C	0.8190	0.9962	0.9414	0.7044	0.8652	0.0652
LOC392301	0.8382	0.7842	0.9494	0.8891	0.8653	0.0353
TBC1D2B	0.9111	0.9637	0.7878	0.7991	0.8654	0.0430
PDIK1L	0.7646	1.1128	0.8899	0.6946	0.8655	0.0918
C1orf25	0.8387	1.0845	0.8388	0.6999	0.8655	0.0800
LRP5L	0.8132	1.0716	0.7939	0.7832	0.8655	0.0690
CYBASC3	0.7136	1.1501	0.9037	0.6946	0.8655	0.1060
CUL4A	0.6781	1.2343	0.9174	0.6324	0.8656	0.1379
LOC1001280	0.7091	1.2021	0.9200	0.6310	0.8656	0.1277
LOC441241	0.6734	1.1844	0.8940	0.7105	0.8656	0.1167
HIAT1	0.7871	1.1172	0.8471	0.7110	0.8656	0.0884
RNF216L	0.8297	0.9787	0.8503	0.8038	0.8656	0.0389

NRG4	0.7050	1.2040	0.8208	0.7328	0.8656	0.1155
ABCB7	0.6714	1.1064	1.0296	0.6555	0.8657	0.1179
ZNF285A	0.7314	1.0302	0.8619	0.8396	0.8658	0.0618
ECHDC2	0.6751	1.2468	0.8476	0.6939	0.8659	0.1327
HRK	0.8230	0.9269	0.8956	0.8183	0.8659	0.0269
BRD7	0.7997	1.1040	0.8707	0.6893	0.8659	0.0877
OCIAD1	0.6524	1.2700	0.9054	0.6359	0.8659	0.1482
MMADHC	0.7556	1.0314	1.0727	0.6041	0.8659	0.1121
LOC654085	0.8199	0.9959	0.9312	0.7169	0.8660	0.0616
ENTPD3	0.7135	1.2020	0.9124	0.6363	0.8661	0.1262
LOC653589	0.7121	0.9504	0.9822	0.8201	0.8662	0.0622
SUZ12	0.6938	1.1109	0.9486	0.7116	0.8662	0.1001
DEF8	0.7535	1.1671	0.8549	0.6900	0.8664	0.1058
POT1	0.7572	1.1114	0.8948	0.7021	0.8664	0.0912
DIAPH1	0.8090	1.1114	0.8758	0.6695	0.8664	0.0923
TTC25	0.8510	0.8898	0.9054	0.8197	0.8665	0.0193
BAK1	0.8211	1.0772	0.7993	0.7684	0.8665	0.0711
FIZ1	0.8565	1.1300	0.7764	0.7032	0.8665	0.0932
DUS2L	0.8314	0.9727	0.7873	0.8749	0.8666	0.0396
DDX17	0.7502	1.0696	0.8947	0.7520	0.8666	0.0757
ZFP82	0.7562	1.1386	0.8498	0.7223	0.8667	0.0945
GNPDA1	0.7330	1.1912	0.9370	0.6058	0.8668	0.1278
ABLIM1	0.6850	1.2278	0.8296	0.7247	0.8668	0.1241
LHFPL2	0.7620	1.0965	0.8747	0.7340	0.8668	0.0824
CTPS2	0.7961	1.0045	0.8478	0.8189	0.8668	0.0471
LOC147804	0.6945	1.1992	0.8829	0.6907	0.8668	0.1195
RUSC1	0.8676	1.1139	0.7785	0.7075	0.8669	0.0886
LRRC47	0.6600	1.1769	0.9622	0.6686	0.8669	0.1249
MRE11A	0.7580	1.0451	0.8688	0.7961	0.8670	0.0637
PDCD10	0.6820	1.0505	1.0626	0.6730	0.8670	0.1095
PPFIA1	0.7779	1.0757	0.9147	0.6998	0.8670	0.0825
SKP1	0.8025	1.1254	0.8548	0.6855	0.8670	0.0931
DCAF8L1	0.7686	0.9017	0.9935	0.8045	0.8671	0.0507
RAPH1	0.7926	0.9944	0.8528	0.8286	0.8671	0.0442
STUB1	0.8709	1.0821	0.8304	0.6852	0.8671	0.0820
RBM10	0.8041	1.0630	0.8394	0.7621	0.8671	0.0672
HNRNPK	0.6457	1.2815	0.8520	0.6894	0.8672	0.1451
KIAA0368	0.8548	1.0886	0.9002	0.6252	0.8672	0.0952
LRDD	0.8034	1.0049	0.8354	0.8255	0.8673	0.0464
RNF44	0.8491	1.0861	0.7578	0.7764	0.8674	0.0755
ATG3	0.7894	1.3072	0.8385	0.5345	0.8674	0.1610
FAM62B	0.8548	1.1227	0.7547	0.7376	0.8674	0.0889
C6orf129	0.7111	1.1915	0.8510	0.7164	0.8675	0.1127
LOC653375	0.7818	1.1426	0.8225	0.7232	0.8675	0.0939
TOR1B	0.7243	1.2090	0.8827	0.6543	0.8676	0.1234
WDR51B	0.7683	1.0689	0.8776	0.7555	0.8676	0.0725

TATDN1	0.7667	1.0733	0.9829	0.6476	0.8676	0.0975
SNORD104	0.6445	1.1985	0.9525	0.6749	0.8676	0.1303
LOC728193	0.9353	0.8871	0.8765	0.7716	0.8676	0.0345
SP2	0.8241	1.1379	0.7578	0.7512	0.8677	0.0916
UBE3C	0.7587	1.2719	0.7260	0.7145	0.8678	0.1350
C14orf43	0.8611	1.0534	0.8246	0.7320	0.8678	0.0676
LOC653497	0.8155	0.9980	0.8985	0.7594	0.8679	0.0520
dJ222E13.2	0.8155	0.9428	0.8736	0.8396	0.8679	0.0277
FAR1	0.8167	1.1149	0.7683	0.7717	0.8679	0.0831
ITPRIP	0.7777	0.9947	0.9055	0.7939	0.8680	0.0509
LOC1001339	0.8707	1.0413	0.8862	0.6737	0.8680	0.0753
VTI1B	0.7279	0.9889	1.0167	0.7386	0.8680	0.0781
UBE2W	0.6986	1.1278	0.9213	0.7243	0.8680	0.0999
GP1BA	0.7326	1.0904	0.9297	0.7195	0.8680	0.0884
INPP5J	0.8616	0.9004	0.9225	0.7876	0.8680	0.0296
POTEF	0.8299	1.0695	0.8757	0.6973	0.8681	0.0771
LOC1001317	0.7564	1.0812	0.8477	0.7870	0.8681	0.0735
PIM2	0.7539	1.1816	0.7782	0.7588	0.8681	0.1046
FLYWCH2	0.6764	1.2590	0.8700	0.6672	0.8681	0.1384
RNU105A	0.7017	1.1023	0.8763	0.7924	0.8682	0.0858
KIAA0562	0.9538	0.9857	0.8016	0.7320	0.8683	0.0607
ABHD7	0.6773	1.1883	0.8955	0.7121	0.8683	0.1169
TCEAL3	0.6993	1.2191	0.8650	0.6900	0.8683	0.1236
MRPS11	0.7590	1.2167	0.8546	0.6430	0.8683	0.1239
GPSM1	0.7644	1.2079	0.7716	0.7294	0.8683	0.1136
LOC645175	0.7560	0.9545	0.9677	0.7950	0.8683	0.0542
TWSG1	0.7222	1.1456	0.9572	0.6483	0.8683	0.1135
FBXO4	0.7049	1.0476	0.9586	0.7623	0.8684	0.0807
MRPL28	0.5903	1.2919	0.8983	0.6928	0.8684	0.1550
CDKN2D	0.8312	0.9900	0.9125	0.7399	0.8684	0.0537
DNPEP	0.8124	1.0583	0.7572	0.8457	0.8684	0.0659
IQGAP3	0.8170	0.9997	0.8922	0.7648	0.8684	0.0510
SMPD4	0.6964	1.1309	0.7851	0.8613	0.8684	0.0938
B3GNT4	0.8304	0.9717	0.9215	0.7502	0.8684	0.0491
IFT81	0.8459	0.9549	0.8909	0.7823	0.8685	0.0364
S100P	0.8482	0.9182	0.8872	0.8204	0.8685	0.0215
INF2	0.8014	1.0666	0.8439	0.7620	0.8685	0.0681
XAGE1B	0.7725	1.0629	0.9232	0.7154	0.8685	0.0782
FRG1	0.7076	1.0033	1.0965	0.6667	0.8685	0.1067
FKBP9L	0.8499	1.0749	0.8265	0.7229	0.8685	0.0741
MCPH1	0.7848	1.0236	0.8817	0.7842	0.8686	0.0565
SCP2	0.6352	1.2326	0.9022	0.7043	0.8686	0.1339
ZNF700	0.8062	1.0727	0.8479	0.7478	0.8686	0.0711
LOC441086	0.7487	0.9753	0.9307	0.8198	0.8687	0.0516
CXorf57	0.7187	1.1362	0.8564	0.7633	0.8687	0.0937
TLE2	0.8620	0.9931	0.7932	0.8266	0.8687	0.0438

LYRM4	0.7220	1.1195	0.9239	0.7096	0.8688	0.0970
SNRNP27	0.6680	1.1488	0.9716	0.6867	0.8688	0.1164
ZSWIM1	0.7519	1.0679	0.8878	0.7680	0.8689	0.0729
UTS2R	0.8862	0.7827	0.9521	0.8549	0.8690	0.0352
C17orf97	0.7761	1.2180	0.7829	0.6987	0.8690	0.1179
PPCS	0.7072	1.0212	1.0460	0.7017	0.8690	0.0952
CNPY2	0.7238	1.2769	0.8243	0.6513	0.8691	0.1405
TIMM17B	0.7074	1.0857	0.9329	0.7504	0.8691	0.0872
USP39	0.8115	1.1067	0.8231	0.7351	0.8691	0.0816
HSP90AA1	0.6702	1.1464	1.0103	0.6497	0.8692	0.1240
SNORA45	0.7566	1.0026	0.9163	0.8014	0.8692	0.0557
GSR	0.8053	1.2559	0.7342	0.6817	0.8693	0.1313
C5orf21	0.7462	1.1090	0.8307	0.7915	0.8693	0.0817
ARHGAP17	0.7691	1.0466	0.8395	0.8224	0.8694	0.0609
C9orf6	0.7608	1.1261	0.9354	0.6555	0.8694	0.1032
ZNF561	0.8920	1.0053	0.8839	0.6967	0.8695	0.0639
SNORA73B	0.6954	0.9747	0.9162	0.8922	0.8696	0.0606
NCK1	0.8025	1.0983	0.9625	0.6155	0.8697	0.1041
ADORA2A	0.9509	0.9335	0.8247	0.7700	0.8698	0.0434
CHD4	0.7007	1.1641	0.8617	0.7527	0.8698	0.1037
LOC1001325	0.7579	1.0567	0.9116	0.7532	0.8699	0.0723
P8	0.9021	1.0641	0.7703	0.7431	0.8699	0.0735
SDHB	0.6469	1.1587	0.9503	0.7238	0.8699	0.1158
USP33	0.7004	1.1624	0.9280	0.6889	0.8699	0.1120
LOC285412	0.7591	1.0703	0.8793	0.7709	0.8699	0.0720
LOC1001317	0.7491	1.1211	0.8129	0.7966	0.8699	0.0848
LOC650832	0.7738	1.2663	0.7963	0.6434	0.8700	0.1363
FLJ20254	0.7306	1.1404	0.8892	0.7204	0.8701	0.0980
DOPEY2	0.8699	1.0901	0.7860	0.7346	0.8701	0.0784
SS18L1	0.7490	1.1095	0.8083	0.8138	0.8702	0.0811
LZIC	0.7599	1.1427	0.8863	0.6918	0.8702	0.0994
ARPC3	0.8109	1.0195	0.9425	0.7078	0.8702	0.0692
IMP3	0.6961	1.1226	0.9611	0.7009	0.8702	0.1045
ALG2	0.8481	1.0213	0.8947	0.7168	0.8702	0.0629
CXXC5	0.8726	1.0601	0.8457	0.7025	0.8702	0.0735
LOC728352	0.7810	0.9576	0.8752	0.8672	0.8702	0.0361
NDUFS5	0.7670	1.0790	0.9424	0.6926	0.8703	0.0871
LOC727950	0.8263	1.0472	0.9040	0.7036	0.8703	0.0720
CWF19L2	0.7169	1.0671	0.9505	0.7466	0.8703	0.0837
FAM134A	0.7857	0.9923	0.8937	0.8095	0.8703	0.0468
OSBPL3	0.7794	0.9258	0.9131	0.8629	0.8703	0.0332
C15orf24	0.8163	1.1497	0.9138	0.6016	0.8703	0.1137
KIAA1012	0.7042	1.1446	0.9122	0.7203	0.8703	0.1029
RBM26	0.7117	1.0498	1.0022	0.7177	0.8704	0.0904
LOC1001341	0.6878	1.1160	0.9825	0.6953	0.8704	0.1068
TMUB2	0.7642	1.0868	0.8721	0.7585	0.8704	0.0767

CDC34	0.7313	1.1707	0.8657	0.7141	0.8704	0.1057
LOC1001342	0.7619	0.9183	0.9024	0.8992	0.8704	0.0364
C8ORFK29	0.8899	0.9356	0.9047	0.7516	0.8704	0.0407
GMEB1	0.7581	1.0556	0.8731	0.7952	0.8705	0.0662
LOC729340	0.6697	0.9909	1.2044	0.6171	0.8705	0.1386
ZNF714	0.7639	1.0846	0.8144	0.8195	0.8706	0.0724
KRT18	0.7266	1.1184	0.9269	0.7106	0.8706	0.0961
KATNA1	0.6900	1.1601	0.8495	0.7835	0.8708	0.1018
TGIF2	0.7800	1.1721	0.7958	0.7352	0.8708	0.1013
ACLY	0.7858	1.1517	0.9186	0.6274	0.8709	0.1109
CCRK	0.7496	1.2083	0.7985	0.7271	0.8709	0.1134
THRAP5	0.7707	0.9652	0.9682	0.7797	0.8709	0.0553
ING2	0.7964	1.0587	0.8394	0.7893	0.8709	0.0636
LOC644617	0.8317	1.0038	0.8817	0.7668	0.8710	0.0501
PIGP	0.7250	1.0978	0.9634	0.6978	0.8710	0.0963
FLJ10081	0.7829	1.0962	0.8946	0.7106	0.8711	0.0840
LAPTM4B	0.7005	1.2063	0.8305	0.7472	0.8711	0.1149
LOC401238	0.7432	1.2127	0.8472	0.6817	0.8712	0.1188
TIAF1	0.8302	1.0815	0.8355	0.7376	0.8712	0.0736
FAM24B	0.7681	1.0589	0.8494	0.8088	0.8713	0.0647
LOC644936	0.8519	0.9593	0.9370	0.7371	0.8713	0.0504
LOC653524	0.6805	1.1239	0.8241	0.8569	0.8713	0.0925
WBP11	0.6917	1.1027	0.9495	0.7418	0.8714	0.0952
DCST1	0.7901	0.9005	0.9938	0.8014	0.8714	0.0477
C8orf40	0.7282	1.3929	0.8472	0.5175	0.8715	0.1867
CCDC21	0.7213	1.0698	0.9424	0.7524	0.8715	0.0822
PPP1CA	0.9125	1.1053	0.7638	0.7044	0.8715	0.0894
ANKRD10	0.7963	0.9709	0.9374	0.7817	0.8716	0.0482
NT5C	0.7849	1.0900	0.9724	0.6392	0.8716	0.0998
LOC1001319	0.8487	0.7869	0.9671	0.8839	0.8717	0.0376
RRAS2	0.6252	1.2169	0.9881	0.6566	0.8717	0.1414
CCDC111	0.8224	1.0075	0.8707	0.7863	0.8717	0.0485
SIAH1	0.6488	1.1334	0.8833	0.8214	0.8717	0.1004
ATP8B2	0.7559	1.0984	0.8509	0.7817	0.8717	0.0782
UBFD1	0.6404	1.1041	0.9947	0.7477	0.8718	0.1072
ECGF1	0.7090	1.3189	0.8556	0.6035	0.8718	0.1577
MNT	0.7238	1.2147	0.7840	0.7647	0.8718	0.1150
LOC1001337	0.8553	1.2733	0.7809	0.5776	0.8718	0.1461
WNT5A	0.7891	0.9414	0.9099	0.8468	0.8718	0.0339
LOC1001308	0.7379	1.1811	0.8886	0.6798	0.8719	0.1121
AMDHD2	0.7723	0.9550	0.9191	0.8410	0.8719	0.0408
C15orf34	0.9643	0.8071	0.8097	0.9068	0.8720	0.0386
ASB13	0.7396	1.2712	0.7901	0.6872	0.8720	0.1347
FUT11	0.7179	1.0137	0.8956	0.8612	0.8721	0.0609
HEATR3	0.6599	1.1315	0.9748	0.7222	0.8721	0.1100
NAT8L	0.7512	1.1491	0.8321	0.7560	0.8721	0.0942

F8	0.7734	1.0395	0.9006	0.7752	0.8722	0.0632
LOC645236	0.8097	0.9524	0.9651	0.7616	0.8722	0.0510
TOMM20	0.7220	1.2438	0.8843	0.6391	0.8723	0.1339
PIM3	0.8037	1.0228	0.8937	0.7689	0.8723	0.0566
ZIC1	0.8189	0.7931	0.9565	0.9209	0.8723	0.0393
PCNT	0.7983	1.2068	0.7206	0.7636	0.8723	0.1126
TMEM203	0.7287	1.1864	0.8346	0.7399	0.8724	0.1073
ZSCAN16	0.8510	0.9950	0.9254	0.7183	0.8724	0.0592
USP8	0.8607	0.9413	0.9346	0.7531	0.8724	0.0438
COBRA1	0.8432	1.0855	0.8245	0.7369	0.8725	0.0747
CENTG3	0.7799	1.1731	0.8641	0.6730	0.8725	0.1075
TBCEL	0.7601	1.0660	0.8926	0.7715	0.8725	0.0711
DHX30	0.7458	1.1350	0.8305	0.7790	0.8726	0.0892
ITGB7	0.8223	1.0417	0.8655	0.7611	0.8726	0.0603
JMJD6	0.7827	1.0016	0.8602	0.8462	0.8727	0.0462
NDUFV2	0.6679	1.2246	0.9462	0.6521	0.8727	0.1353
EFR3A	0.6012	1.2216	0.9568	0.7111	0.8727	0.1380
PLSCR4	0.9125	0.8824	0.9574	0.7385	0.8727	0.0473
IL13RA1	0.8157	1.0694	0.9271	0.6786	0.8727	0.0830
DIABLO	0.7524	1.0512	0.9271	0.7602	0.8727	0.0719
LOC732165	0.7438	1.1008	1.0074	0.6391	0.8728	0.1085
ARGLU1	0.7696	1.2141	0.8894	0.6183	0.8728	0.1265
PPP6C	0.7223	1.1458	0.9768	0.6464	0.8728	0.1152
MANEA	0.6595	1.2005	0.9942	0.6373	0.8728	0.1363
TESC	0.7387	1.0932	0.9518	0.7079	0.8729	0.0913
PALLD	0.7420	1.1655	0.8149	0.7693	0.8729	0.0987
ZBTB38	0.8078	1.1566	0.8182	0.7091	0.8729	0.0977
CUL2	0.6977	1.0844	0.8241	0.8856	0.8729	0.0806
ZBED3	0.8429	0.9975	0.8410	0.8105	0.8730	0.0422
MYO5A	0.7851	1.0247	0.9195	0.7627	0.8730	0.0613
C9orf169	0.9320	1.1599	0.7522	0.6480	0.8730	0.1122
HINT2	0.9100	1.0955	0.8331	0.6535	0.8730	0.0916
TMEM4	0.8171	1.0555	0.8972	0.7225	0.8731	0.0705
LOC1001293	0.7256	1.0075	0.8925	0.8668	0.8731	0.0579
EGR1	0.8723	0.9930	0.8669	0.7604	0.8731	0.0475
NDUFB8	0.7087	1.3167	0.8571	0.6101	0.8731	0.1563
SNORD109B	0.7138	0.9056	0.9954	0.8779	0.8731	0.0587
FAM76B	0.8014	1.0204	0.8649	0.8060	0.8732	0.0512
LOC440354	0.7978	1.0086	0.8767	0.8096	0.8732	0.0484
THNSL1	0.7283	1.0862	0.9065	0.7719	0.8732	0.0805
PRR14	0.7315	1.2304	0.7946	0.7365	0.8733	0.1199
FLJ41649	0.8542	0.8952	0.8429	0.9008	0.8733	0.0145
LARP1B	0.7950	1.0131	0.9463	0.7388	0.8733	0.0640
AAGAB	0.7610	1.0995	0.9898	0.6433	0.8734	0.1042
LOC654174	0.6385	1.3260	0.8700	0.6591	0.8734	0.1597
C10orf119	0.7684	1.0063	0.8742	0.8446	0.8734	0.0496

INTS4	0.7629	1.2335	0.8035	0.6939	0.8734	0.1221
INTS8	0.9298	0.8858	0.9947	0.6834	0.8734	0.0672
C14orf79	0.7334	1.2017	0.8451	0.7135	0.8734	0.1132
CHMP4A	0.8038	1.1995	0.8659	0.6247	0.8735	0.1201
TMEM51	0.7806	1.0333	0.9122	0.7680	0.8735	0.0624
CHUK	0.9265	0.9618	0.8683	0.7375	0.8735	0.0493
GKAP1	0.9020	0.8293	0.9670	0.7958	0.8735	0.0382
LOC1001335	0.6620	0.7450	1.2255	0.8619	0.8736	0.1243
CENTG2	0.7553	1.0430	0.9272	0.7688	0.8736	0.0686
LOC1001303	0.8680	0.9551	0.8565	0.8148	0.8736	0.0295
LOC727726	0.7917	1.1510	0.8065	0.7452	0.8736	0.0934
WDFY1	0.7342	1.1658	0.8660	0.7285	0.8736	0.1024
FBXO16	0.8029	1.0845	0.8001	0.8073	0.8737	0.0703
GAGE5	0.7327	1.1275	0.9154	0.7192	0.8737	0.0957
IRF3	0.7942	1.1025	0.8825	0.7160	0.8738	0.0835
RAB8A	0.6979	1.2259	0.8407	0.7310	0.8739	0.1212
SNORD36C	0.7956	1.0030	0.9399	0.7570	0.8739	0.0583
HN1	0.8383	1.0305	1.0168	0.6099	0.8739	0.0983
SLC16A3	0.7880	1.0296	0.8731	0.8049	0.8739	0.0551
OSBPL1A	0.7191	1.1538	0.9116	0.7112	0.8739	0.1042
LOC1001324	0.8120	1.0827	0.7937	0.8073	0.8739	0.0697
B4GALT7	0.7122	1.2479	0.8302	0.7054	0.8740	0.1279
LOC652877	0.8822	0.9283	0.8492	0.8362	0.8740	0.0205
ZFP30	0.8233	0.8488	0.9530	0.8710	0.8740	0.0281
TBCCD1	0.7938	1.0203	0.9285	0.7536	0.8740	0.0614
GCN1L1	0.5427	1.3437	0.9340	0.6758	0.8741	0.1764
LOC339352	0.7203	1.0607	0.8982	0.8173	0.8741	0.0720
KIAA1949	0.8166	1.0227	0.8925	0.7650	0.8742	0.0560
METTL6	0.7216	0.9787	1.0068	0.7898	0.8742	0.0701
CCDC42	0.7878	1.0596	0.7827	0.8668	0.8743	0.0647
RABGGTB	0.6493	1.1338	1.0369	0.6770	0.8743	0.1236
UEVLD	0.8280	0.9806	0.9809	0.7076	0.8743	0.0662
LOC440043	0.7227	1.2012	0.8937	0.6795	0.8743	0.1184
TMEM55A	0.8950	0.9406	0.9253	0.7363	0.8743	0.0470
C1orf107	0.8018	0.9299	0.9377	0.8280	0.8743	0.0348
B4GALT4	0.8010	1.0693	0.8885	0.7386	0.8743	0.0719
ACOT1	0.8142	1.0106	1.0260	0.6467	0.8744	0.0899
RAB7L1	0.7850	1.0519	0.9092	0.7516	0.8744	0.0682
ALOXE3	0.8201	0.9726	0.8975	0.8076	0.8744	0.0383
PAG1	0.8011	1.0663	0.8954	0.7350	0.8744	0.0719
PSMB7	0.7624	1.2091	0.8188	0.7076	0.8745	0.1138
SNORD62B	0.9636	0.8561	0.8242	0.8541	0.8745	0.0306
GADD45GIP1	0.8858	0.9610	0.9257	0.7256	0.8745	0.0520
LOC1001323	0.7806	1.0936	0.8882	0.7357	0.8745	0.0797
FAM156B	0.7041	1.0659	0.8785	0.8501	0.8747	0.0743
PREB	0.7464	1.1395	0.8985	0.7144	0.8747	0.0970

UMPS	0.7317	1.0725	0.9567	0.7379	0.8747	0.0842
TTLL7	0.7854	1.1377	0.8249	0.7508	0.8747	0.0890
NUP98	0.7712	1.0942	0.8472	0.7862	0.8747	0.0750
NDUFS8	0.6691	1.3028	0.8308	0.6961	0.8747	0.1470
FAM53A	0.8748	0.8955	0.9564	0.7722	0.8747	0.0383
BEXL1	0.8835	1.0651	0.8689	0.6814	0.8747	0.0784
LOC388654	0.5866	1.1624	1.0946	0.6554	0.8748	0.1478
MED27	0.6747	1.1492	0.9490	0.7263	0.8748	0.1091
LOC1001321	0.8121	0.9708	0.9598	0.7568	0.8749	0.0535
C8orf59	0.7492	1.1098	1.0604	0.5803	0.8749	0.1266
DCLRE1B	0.7887	0.9246	0.9190	0.8675	0.8750	0.0315
LOC645251	0.7654	0.9805	0.9923	0.7617	0.8750	0.0644
LOC645478	0.7584	1.0534	0.8303	0.8578	0.8750	0.0631
EIF2S3	0.6297	1.2321	0.9035	0.7345	0.8750	0.1317
LOC646533	0.9282	0.8435	0.9162	0.8121	0.8750	0.0281
LOC1001313	0.8351	0.9927	0.8844	0.7879	0.8750	0.0439
PXDN	0.6871	1.0405	1.0009	0.7716	0.8750	0.0862
FLJ35024	0.8623	0.9922	0.8781	0.7680	0.8751	0.0460
BAZ1B	0.7078	1.1870	0.8261	0.7797	0.8751	0.1068
DHX16	0.7831	1.1667	0.8506	0.7002	0.8752	0.1019
HS1BP3	0.7866	1.1601	0.8385	0.7155	0.8752	0.0983
MAGT1	0.5789	1.1953	0.9006	0.8259	0.8752	0.1269
LOC1001280	0.8030	1.0233	0.9331	0.7414	0.8752	0.0635
LYRM2	0.6557	1.0865	0.9159	0.8430	0.8753	0.0892
ENY2	0.7139	1.1267	0.9600	0.7005	0.8753	0.1029
LOC402377	0.7912	0.9303	0.9628	0.8169	0.8753	0.0420
MAN1A2	0.7940	1.0943	0.8741	0.7387	0.8753	0.0781
PRKAA1	0.7515	1.0988	1.0287	0.6222	0.8753	0.1129
SERPINE1	0.8059	1.0081	0.9775	0.7097	0.8753	0.0709
BSCL2	0.7708	1.2515	0.8411	0.6383	0.8754	0.1322
ELL	0.8828	1.0303	0.8487	0.7400	0.8755	0.0599
SLC35B3	0.8389	1.0412	0.9450	0.6768	0.8755	0.0781
LOC1001293	0.7703	1.0255	0.9392	0.7669	0.8755	0.0642
ARFGAP1	0.8083	1.0323	0.8931	0.7683	0.8755	0.0584
DHX35	0.7117	1.2424	0.7339	0.8144	0.8756	0.1242
GTF2IP1	0.6797	1.1934	0.9731	0.6565	0.8757	0.1281
KIAA0101	0.7670	1.1797	0.8598	0.6962	0.8757	0.1067
LOC730274	0.8693	0.9592	0.9368	0.7377	0.8758	0.0498
ACBD7	0.7743	1.0363	0.9168	0.7756	0.8758	0.0631
GNB2	0.8412	1.1091	0.8320	0.7209	0.8758	0.0824
FAM8A1	0.8399	0.8728	1.0460	0.7444	0.8758	0.0629
PRMT7	0.6889	1.3303	0.7273	0.7568	0.8758	0.1521
LOC1001290	0.6603	0.9137	1.3837	0.5458	0.8759	0.1859
CTSZ	0.7917	1.0694	0.8559	0.7866	0.8759	0.0664
TRAF2	0.7826	0.9949	0.9695	0.7566	0.8759	0.0618
C1QBP	0.7147	1.3126	0.8123	0.6641	0.8759	0.1488

TUG1	0.8588	1.1867	0.8197	0.6386	0.8760	0.1141
TSPAN33	0.6562	1.2228	0.9512	0.6738	0.8760	0.1339
RCE1	0.7603	1.1175	0.8384	0.7878	0.8760	0.0821
FGD1	0.7513	1.0922	0.8498	0.8112	0.8761	0.0748
C14orf162	0.8770	0.9226	0.8091	0.8961	0.8762	0.0242
EZR	0.8554	1.0787	0.8855	0.6855	0.8763	0.0806
GYLTL1B	0.8512	0.9733	0.8560	0.8246	0.8763	0.0331
SNORA62	0.7910	0.9532	0.9021	0.8590	0.8763	0.0343
CISD3	0.6001	1.1756	0.9207	0.8090	0.8764	0.1199
CYorf15A	0.6827	1.2385	0.9575	0.6268	0.8764	0.1407
NEURL2	0.7796	0.9623	0.9434	0.8205	0.8765	0.0451
SNORA17	0.6409	1.0301	0.9182	0.9171	0.8766	0.0829
AHSA2	0.7785	1.0543	0.9649	0.7088	0.8767	0.0802
RALB	0.6709	1.2140	0.9411	0.6807	0.8767	0.1287
CNTNAP2	0.8138	0.9941	0.8778	0.8213	0.8767	0.0416
PIN1	0.7188	1.2294	0.9210	0.6378	0.8768	0.1318
ZXDB	0.7801	1.1177	0.8437	0.7656	0.8768	0.0821
MTHFD2L	0.8650	1.0664	0.8529	0.7229	0.8768	0.0709
REEP3	0.5990	1.1656	1.0690	0.6736	0.8768	0.1411
FAM21A	0.7535	1.0839	0.8771	0.7928	0.8768	0.0737
POLR1B	0.6995	1.0791	0.9761	0.7525	0.8768	0.0902
HCG2P7	0.7472	1.0910	0.9171	0.7522	0.8769	0.0816
MSL3	0.7781	1.1217	0.8644	0.7434	0.8769	0.0855
RCBTB1	0.8149	1.0355	0.8198	0.8376	0.8770	0.0531
BEX5	0.8115	0.9486	0.9113	0.8365	0.8770	0.0319
SNORD49A	0.8354	1.0202	0.8763	0.7762	0.8770	0.0520
HYOU1	0.7912	1.1081	0.8763	0.7324	0.8770	0.0825
LOC1001322	0.6462	1.3729	0.7679	0.7213	0.8771	0.1672
LOC653888	0.8000	1.1188	0.7820	0.8079	0.8772	0.0807
RPL21	0.6086	1.2357	1.0663	0.5982	0.8772	0.1618
LMNA	0.8002	1.1524	0.8425	0.7138	0.8772	0.0956
ZNF93	0.7535	1.0212	0.9353	0.7990	0.8772	0.0616
LOC652517	0.8563	0.9172	0.9209	0.8147	0.8772	0.0256
BEND3	0.7641	1.0936	0.8572	0.7943	0.8773	0.0747
CPNE1	0.7102	1.1922	0.8721	0.7348	0.8773	0.1108
LOC1001301	0.8168	1.0047	0.9000	0.7879	0.8773	0.0486
SNORD17	0.8169	1.0455	0.7898	0.8573	0.8774	0.0577
ZNF217	0.8418	1.1811	0.9125	0.5742	0.8774	0.1247
FKBPL	0.7276	1.0509	0.8916	0.8395	0.8774	0.0672
LOC645168	0.8109	0.9684	0.8734	0.8570	0.8774	0.0331
LOC388588	0.6957	1.1744	0.9436	0.6960	0.8774	0.1149
LOC1001318	0.5417	1.3632	0.9319	0.6732	0.8775	0.1810
NCLN	0.7797	1.2902	0.7915	0.6490	0.8776	0.1413
LGALS1	0.8174	1.0579	0.8480	0.7871	0.8776	0.0614
KIAA1143	0.7222	1.0845	0.9601	0.7435	0.8776	0.0874
ZNF677	0.9437	0.9625	0.9047	0.6996	0.8776	0.0606

CCNL1	0.7259	1.2547	0.8500	0.6801	0.8777	0.1307
ZNF827	0.7652	1.0891	0.8756	0.7808	0.8777	0.0746
NARG1L	0.8433	0.9569	0.9221	0.7885	0.8777	0.0381
SIVA	0.6077	1.3101	0.9513	0.6418	0.8777	0.1635
PEX16	0.7600	1.2822	0.8593	0.6094	0.8777	0.1443
SLC25A1	0.7564	1.1896	0.8904	0.6745	0.8777	0.1131
PFDN1	0.6988	1.1695	0.9296	0.7132	0.8778	0.1106
LARP6	0.7240	1.0846	0.9708	0.7320	0.8779	0.0896
APOOL	0.6658	1.0447	0.9854	0.8156	0.8779	0.0857
HS3ST3B1	0.7614	0.9233	0.8844	0.9426	0.8779	0.0407
LOC731724	0.9487	0.9555	0.9079	0.6995	0.8779	0.0604
CNOT6L	0.7313	1.0335	0.9434	0.8036	0.8779	0.0680
CRYZ	0.6152	1.1826	1.0669	0.6472	0.8780	0.1446
CCDC74A	0.8723	1.1398	0.7326	0.7673	0.8780	0.0922
AUP1	0.8833	0.8781	1.0047	0.7461	0.8781	0.0528
ATP1B1	0.7596	1.2561	0.9375	0.5592	0.8781	0.1478
FLJ20273	0.8815	1.0179	0.8853	0.7278	0.8781	0.0593
EP400	0.7689	1.1743	0.7577	0.8115	0.8781	0.0994
PPM1E	0.8301	0.9973	0.9519	0.7333	0.8782	0.0598
AP3D1	0.7691	1.2316	0.8338	0.6783	0.8782	0.1220
RIOK1	0.7532	1.1047	0.8911	0.7639	0.8782	0.0817
KRIT1	0.8050	1.0123	0.9581	0.7375	0.8782	0.0642
NARG2	0.8385	1.0182	0.8924	0.7638	0.8782	0.0536
INTS12	0.7165	1.0842	0.9397	0.7728	0.8783	0.0834
SGK196	0.7636	1.0239	0.9545	0.7712	0.8783	0.0656
KHK	0.8279	0.9735	0.9201	0.7917	0.8783	0.0417
C9orf119	0.8111	1.1160	0.8925	0.6937	0.8783	0.0891
LOC1001334	0.8813	0.9997	0.8929	0.7396	0.8784	0.0534
SPRED1	0.7401	1.1500	0.9377	0.6856	0.8784	0.1055
PSIP1	0.7435	1.0469	0.9736	0.7495	0.8784	0.0776
LOC645430	0.7172	1.0956	1.0151	0.6857	0.8784	0.1037
FAM10A7	0.6359	1.3356	0.8576	0.6848	0.8785	0.1596
TMUB1	0.8463	1.0166	0.8825	0.7687	0.8785	0.0518
FAM101B	0.8177	0.9900	0.8486	0.8578	0.8785	0.0381
LOC1001324	0.7711	1.0132	0.9852	0.7449	0.8786	0.0701
TRIM59	0.8305	1.0867	0.9157	0.6816	0.8786	0.0846
ENPP1	0.7765	1.0198	0.9081	0.8102	0.8787	0.0547
RNF25	0.8666	0.9657	0.8741	0.8083	0.8787	0.0325
PIF1	0.8562	0.9639	0.8860	0.8087	0.8787	0.0326
C17orf42	0.7682	1.0846	0.9595	0.7027	0.8787	0.0876
PDIA5	0.7929	1.1895	0.9086	0.6239	0.8787	0.1189
NACC2	0.6790	1.0309	0.9312	0.8739	0.8787	0.0741
LOC1001909	0.6229	1.0575	0.9065	0.9282	0.8788	0.0916
PANK1	0.7536	1.0948	0.9478	0.7190	0.8788	0.0878
DDA1	0.7873	1.1270	0.8813	0.7196	0.8788	0.0891
LOC399815	0.7745	0.9967	0.8403	0.9037	0.8788	0.0473

HIC2	0.6712	1.1646	0.8415	0.8381	0.8789	0.1032
VPS37C	0.8668	1.1235	0.8089	0.7164	0.8789	0.0872
CBL	0.6974	1.1067	0.9232	0.7883	0.8789	0.0890
CELSR2	0.8592	1.0229	0.9484	0.6852	0.8789	0.0727
C20orf86	0.9409	0.7096	1.0708	0.7945	0.8789	0.0798
RNFT1	0.7901	1.0960	0.8814	0.7482	0.8790	0.0775
PUSL1	0.7269	1.1405	0.9112	0.7373	0.8790	0.0969
GBE1	0.9217	1.1001	0.8008	0.6933	0.8790	0.0872
ANKDD1A	0.8153	1.0194	0.8496	0.8318	0.8790	0.0473
DUSP18	0.9027	0.9275	0.8518	0.8341	0.8790	0.0218
RPP25	0.8053	0.9787	0.8102	0.9220	0.8790	0.0428
UHRF2	0.8426	0.9321	0.9123	0.8292	0.8790	0.0254
ZNF225	0.8369	0.9718	0.9116	0.7962	0.8791	0.0391
DPM1	0.6860	1.1374	0.9749	0.7185	0.8792	0.1076
PGLS	0.8696	1.0831	0.8842	0.6799	0.8792	0.0824
STK17B	0.7631	1.0195	0.9172	0.8172	0.8792	0.0566
OR7G1	0.8383	1.0610	0.8632	0.7545	0.8793	0.0649
GAGE2E	0.8332	1.2374	0.8215	0.6250	0.8793	0.1286
PPARGC1B	0.7718	0.9025	0.9328	0.9100	0.8793	0.0364
LOC387791	0.7154	1.0262	1.1961	0.5795	0.8793	0.1410
C1orf57	0.6495	1.1214	1.0327	0.7136	0.8793	0.1163
RELA	0.8267	1.0253	0.8570	0.8083	0.8793	0.0497
RBM18	0.7160	1.1411	0.9599	0.7005	0.8794	0.1055
ZNF26	0.8094	1.0201	0.9528	0.7353	0.8794	0.0651
PRDX6	0.6748	1.1228	0.9987	0.7213	0.8794	0.1082
C4orf39	0.8893	0.9827	0.8660	0.7797	0.8794	0.0417
TMEM108	0.8040	1.0000	0.9209	0.7933	0.8795	0.0495
IPO9	0.8078	1.1386	0.8640	0.7081	0.8796	0.0922
PRRG4	0.7169	1.2457	0.8312	0.7249	0.8797	0.1248
CSNK2A1P	0.7322	1.2283	0.9143	0.6439	0.8797	0.1291
ACTR3B	0.8052	1.0845	0.8935	0.7358	0.8798	0.0755
SCAND1	0.7234	1.2501	0.8865	0.6590	0.8798	0.1324
C1orf97	0.7064	1.1897	0.9933	0.6298	0.8798	0.1296
TSEN15	0.7699	1.0207	0.9594	0.7693	0.8798	0.0649
WWOX	0.7825	1.0049	0.9275	0.8044	0.8798	0.0525
SLC7A6	0.7879	1.0508	0.8564	0.8242	0.8798	0.0587
LOC652657	0.7872	1.0054	0.9439	0.7829	0.8799	0.0562
TIMM17A	0.8475	0.9543	0.9256	0.7923	0.8799	0.0369
SMARCAL1	0.7984	1.2648	0.8249	0.6316	0.8799	0.1352
IGFBP2	0.8721	1.0279	0.8741	0.7457	0.8800	0.0578
EPM2AIP1	0.8467	1.1037	0.8806	0.6889	0.8800	0.0855
SRF	0.8435	1.0701	0.8723	0.7342	0.8800	0.0700
LOC652607	0.9324	0.8688	0.8662	0.8526	0.8800	0.0178
NSMCE1	0.6925	1.2419	0.8900	0.6957	0.8800	0.1292
WRN	0.8108	1.0752	0.8199	0.8143	0.8801	0.0651
ISG20L2	0.7682	1.2454	0.8706	0.6361	0.8801	0.1309

PIGN	0.8312	1.1725	0.8070	0.7096	0.8801	0.1010
MAK10	0.7512	1.1542	0.8835	0.7316	0.8801	0.0974
LOC1001330	0.7774	0.9426	0.9489	0.8517	0.8802	0.0408
CBX1	0.8079	0.9326	1.0642	0.7159	0.8802	0.0757
LOC1001336	0.8339	1.0172	1.0754	0.5942	0.8802	0.1083
SLC30A3	0.7485	1.1212	0.8737	0.7773	0.8802	0.0847
ACTG1	0.7092	1.1169	0.9705	0.7241	0.8802	0.0991
LOC1001307	0.6687	1.0840	1.1431	0.6250	0.8802	0.1356
C21orf70	0.6988	1.1304	0.9309	0.7610	0.8803	0.0967
SDF4	0.8977	1.0191	0.8860	0.7183	0.8803	0.0618
TOR1A	0.9039	1.1957	0.7563	0.6654	0.8803	0.1160
C4orf42	0.8054	1.0873	0.9284	0.7002	0.8803	0.0833
WRB	0.7042	1.1356	1.0866	0.5951	0.8804	0.1354
LIPA	0.6716	1.0857	1.0584	0.7058	0.8804	0.1110
LOC648984	0.7259	1.0850	0.9460	0.7645	0.8804	0.0834
LOC643310	0.7220	1.0641	1.1809	0.5544	0.8804	0.1459
INO80	0.8249	1.1220	0.8464	0.7281	0.8804	0.0846
LOC729841	0.8066	1.2130	0.8130	0.6888	0.8804	0.1145
LOC285216	0.7891	0.9256	0.9182	0.8887	0.8804	0.0315
TRMT61A	0.8062	1.0374	0.8885	0.7895	0.8804	0.0566
ANKRD40	0.7682	1.0565	0.8820	0.8150	0.8804	0.0632
HAS3	0.8329	0.9037	0.9170	0.8682	0.8804	0.0189
ZNF670	0.7353	0.9996	0.9465	0.8405	0.8805	0.0586
ATP13A1	0.8518	1.0034	0.9202	0.7466	0.8805	0.0543
RNF8	0.8497	1.0589	0.8607	0.7527	0.8805	0.0642
LOC644615	0.6631	1.0026	1.1025	0.7538	0.8805	0.1031
MPI	0.7329	1.3074	0.7639	0.7178	0.8805	0.1426
FKTN	0.7542	1.1398	0.9689	0.6594	0.8805	0.1080
CHAC2	0.7508	0.8993	1.0004	0.8717	0.8806	0.0513
LOC729142	0.8296	0.9055	1.0394	0.7480	0.8806	0.0619
CENPV	0.7374	1.1063	0.9291	0.7496	0.8806	0.0871
COL20A1	0.7056	0.9609	0.9780	0.8780	0.8806	0.0623
RBM12	0.7619	1.0894	0.9236	0.7477	0.8807	0.0802
C1orf96	0.8988	0.9237	0.9567	0.7436	0.8807	0.0472
ORAOV1	0.6655	1.2060	0.8345	0.8169	0.8807	0.1149
CASD1	0.7607	1.1305	0.9153	0.7168	0.8808	0.0935
BEND7	0.7383	1.0468	0.9159	0.8227	0.8809	0.0661
B3GNT1	0.8198	1.0025	1.0268	0.6747	0.8809	0.0828
B4GALT3	0.8183	1.1057	0.9324	0.6674	0.8809	0.0925
PRRG1	0.7287	1.1189	0.9629	0.7133	0.8810	0.0977
ZNF57	0.7423	1.0802	0.9665	0.7350	0.8810	0.0854
NRM	0.8684	1.0215	0.8482	0.7859	0.8810	0.0500
DDHD2	0.6808	1.1920	0.9183	0.7332	0.8811	0.1155
LOC651166	0.7845	0.9596	0.8741	0.9061	0.8811	0.0367
C2orf44	0.8090	1.1608	0.8886	0.6661	0.8811	0.1040
FAM162A	0.7592	0.8998	1.1538	0.7116	0.8811	0.0993

LOC1001310	0.8475	0.9654	0.8884	0.8232	0.8811	0.0312
ARMC6	0.7751	1.1443	0.8613	0.7438	0.8811	0.0912
UBAP2	0.8263	1.1024	0.8922	0.7037	0.8812	0.0835
DUSP28	0.7929	1.2684	0.8721	0.5913	0.8812	0.1420
CLDN3	0.7158	1.2664	0.8314	0.7110	0.8812	0.1314
PPPDE2	0.6672	1.2419	0.9063	0.7092	0.8812	0.1311
UFD1L	0.8258	0.9822	0.9368	0.7800	0.8812	0.0471
FAM156A	0.7433	1.0751	0.8754	0.8311	0.8812	0.0702
ZCCHC11	0.8178	1.1844	0.8229	0.6999	0.8813	0.1050
VEZT	0.7664	1.1338	0.8466	0.7783	0.8813	0.0860
ATP5I	0.7223	1.1419	0.9515	0.7096	0.8813	0.1031
LRFN4	0.7414	0.9283	1.0287	0.8269	0.8813	0.0622
CEPT1	0.6547	1.1742	0.9585	0.7379	0.8813	0.1168
LOC729686	0.6995	1.0397	1.1481	0.6381	0.8814	0.1253
ATXN10	0.8607	1.0284	0.8972	0.7392	0.8814	0.0595
GAGE12J	0.7811	1.0859	0.8465	0.8119	0.8814	0.0695
LOC649009	0.8437	1.0062	0.8803	0.7953	0.8814	0.0451
ATP6V0A1	0.8309	1.1992	0.7635	0.7321	0.8814	0.1079
SH3D19	0.8014	1.0999	1.0319	0.5928	0.8815	0.1155
LOC728723	0.7389	1.1580	0.8899	0.7393	0.8815	0.0988
LFNG	0.7497	1.0339	0.9277	0.8150	0.8816	0.0627
ISYNA1	0.9127	0.8779	0.9397	0.7961	0.8816	0.0312
ITGB1BP1	0.7234	1.0714	0.9782	0.7534	0.8816	0.0851
MUDENG	0.7094	1.1512	0.8995	0.7667	0.8817	0.0983
PRPF8	0.8340	1.0698	0.8346	0.7887	0.8818	0.0636
CD22	0.8049	0.9941	0.8377	0.8904	0.8818	0.0414
RBM24	0.8188	0.9671	0.9875	0.7538	0.8818	0.0569
MKNK1	0.7493	1.2335	0.7933	0.7511	0.8818	0.1177
LOC728965	0.7606	0.9797	0.9738	0.8132	0.8818	0.0558
LOC1001341	0.7262	1.0994	0.8390	0.8628	0.8818	0.0784
AKAP7	0.7777	1.0275	0.8740	0.8483	0.8819	0.0526
FNIP2	0.8502	0.8365	0.9899	0.8509	0.8819	0.0362
EGLN1	0.7151	1.1171	0.9283	0.7670	0.8819	0.0906
PASK	0.8874	1.2003	0.7684	0.6717	0.8819	0.1149
UBE2V2	0.8486	1.1000	0.9812	0.5979	0.8820	0.1077
UBXN7	0.7463	1.0541	0.8650	0.8627	0.8820	0.0637
PKN1	0.8542	1.0860	0.8513	0.7367	0.8821	0.0733
MIR26B	0.8753	0.9391	0.8948	0.8190	0.8821	0.0249
SNX3	0.7994	1.1325	0.9965	0.6002	0.8821	0.1162
FLJ20489	0.8047	1.0519	0.9220	0.7503	0.8822	0.0670
FKSG2	0.8441	0.9942	0.8002	0.8903	0.8822	0.0416
C4orf41	0.8937	1.0937	0.8491	0.6924	0.8822	0.0827
LOC653450	0.7184	1.2376	0.8786	0.6944	0.8823	0.1253
LASS1	0.7413	0.9742	0.9494	0.8642	0.8823	0.0526
RNGTT	0.7909	1.0130	0.9587	0.7671	0.8824	0.0609
LOC648626	0.7633	0.9182	0.9058	0.9426	0.8825	0.0405

GAGE12C	0.6746	1.1510	1.0050	0.6994	0.8825	0.1168
LOC645723	0.8265	1.0124	0.9577	0.7334	0.8825	0.0632
TMEM50A	0.7578	1.0994	0.8785	0.7944	0.8825	0.0766
LOC644000	0.8405	0.9651	0.8525	0.8719	0.8825	0.0283
LOC1001299	0.7106	1.1551	0.9292	0.7352	0.8825	0.1032
ADCY1	0.7677	1.1531	0.7989	0.8106	0.8825	0.0906
TRIO	0.8686	0.9621	0.8402	0.8594	0.8825	0.0272
CDC37	0.7774	1.2152	0.9144	0.6232	0.8826	0.1258
NACAP1	0.7719	1.0014	1.1371	0.6201	0.8826	0.1155
TMEM164	0.8167	1.1191	0.8634	0.7311	0.8826	0.0835
B3GNTL1	0.8667	1.0365	0.9029	0.7245	0.8826	0.0641
FDX1L	0.6907	1.2921	0.8816	0.6663	0.8827	0.1447
STXBP2	0.8241	1.0740	0.8836	0.7491	0.8827	0.0695
LASP1	0.7980	1.2795	0.7858	0.6676	0.8827	0.1355
FAM89B	0.7984	1.2805	0.7353	0.7169	0.8828	0.1337
LRRC58	0.9232	1.0360	0.9149	0.6571	0.8828	0.0802
AMY1B	0.8565	0.9553	0.9262	0.7932	0.8828	0.0364
WDR20	0.7677	1.1382	0.8311	0.7942	0.8828	0.0861
LOC643446	0.6946	1.2989	0.8438	0.6939	0.8828	0.1431
PIPSL	0.7214	1.1511	0.9150	0.7438	0.8828	0.0993
C10orf28	0.7119	1.1555	0.8862	0.7777	0.8828	0.0977
TMEM120A	0.8729	1.0936	0.9170	0.6479	0.8828	0.0917
LOC1001255	0.8568	0.9908	0.9303	0.7535	0.8828	0.0511
DHRS13	0.7082	1.2713	0.8969	0.6551	0.8829	0.1395
PLIN5	0.7855	0.8779	1.0688	0.7994	0.8829	0.0652
EGFR	0.7847	1.0287	0.9002	0.8181	0.8829	0.0543
ZC3H4	0.7583	1.0899	0.9186	0.7650	0.8829	0.0783
OXSR1	0.8634	1.0979	0.8808	0.6899	0.8830	0.0836
HSPC157	0.7144	1.1733	0.9525	0.6920	0.8831	0.1133
LOC1001320	0.8652	0.9773	0.9165	0.7734	0.8831	0.0431
HNRPLL	0.7209	1.0331	1.0685	0.7098	0.8831	0.0971
TBP	0.7848	1.1018	0.9112	0.7346	0.8831	0.0818
LOC649970	0.7648	1.1513	0.9448	0.6715	0.8831	0.1059
GPR157	0.7869	1.0424	0.8938	0.8102	0.8833	0.0578
DDX18	0.6544	1.1721	0.9447	0.7621	0.8833	0.1134
STXBP6	0.7347	1.0465	0.9576	0.7946	0.8834	0.0720
BCL9	0.8874	1.0248	0.8686	0.7532	0.8835	0.0557
STAM2	0.7692	1.1728	0.9232	0.6689	0.8835	0.1097
LOC729524	0.8044	1.0488	0.8526	0.8284	0.8836	0.0559
PAQR3	0.7768	0.9693	1.0948	0.6933	0.8836	0.0911
NDUFV1	0.7811	1.2250	0.8711	0.6571	0.8836	0.1220
ATP5H	0.7006	1.1757	0.9406	0.7175	0.8836	0.1117
C12orf43	0.7424	1.0347	1.0474	0.7099	0.8836	0.0912
MIR1909	0.7916	1.0225	0.8714	0.8490	0.8836	0.0492
MZF1	0.7562	1.2224	0.7970	0.7591	0.8836	0.1133
PRKRIP1	0.6417	1.1879	0.8388	0.8664	0.8837	0.1131

GJC1	0.6602	1.1539	0.9401	0.7805	0.8837	0.1068
ZNF280D	0.8317	1.0819	0.9350	0.6863	0.8837	0.0834
HMOX2	0.8754	1.0764	0.8567	0.7264	0.8837	0.0723
ILKAP	0.7782	1.0947	0.8638	0.7983	0.8838	0.0726
RHOD	0.6377	1.3128	0.8753	0.7092	0.8838	0.1514
LOC407835	0.8968	1.0567	0.8309	0.7507	0.8838	0.0649
LOC729298	0.7324	1.1744	0.8957	0.7329	0.8839	0.1042
FAM105B	0.7923	1.1602	0.8740	0.7090	0.8839	0.0981
HRASLS3	0.8453	1.1881	0.8390	0.6632	0.8839	0.1098
CGI-96	0.7823	0.9738	0.9953	0.7844	0.8839	0.0583
NOL3	0.7660	1.2058	0.8754	0.6887	0.8840	0.1139
VWA1	0.8118	1.1144	0.8321	0.7775	0.8840	0.0776
SQSTM1	0.8530	1.2942	0.7712	0.6176	0.8840	0.1452
PEPD	0.7590	1.1058	0.8612	0.8099	0.8840	0.0768
CCDC66	0.8318	0.9924	0.8799	0.8320	0.8840	0.0379
SNORA11	0.8597	1.0234	0.8639	0.7891	0.8840	0.0495
LOC134541	0.8720	0.8861	1.0114	0.7667	0.8840	0.0501
OR6C75	0.7513	0.9360	1.0022	0.8467	0.8841	0.0545
NPAT	0.7382	1.0253	1.0090	0.7638	0.8841	0.0771
LOC401010	0.8598	0.9850	0.9320	0.7595	0.8841	0.0488
LOC648966	0.7705	0.9725	0.9505	0.8432	0.8842	0.0473
TCIRG1	0.7735	1.0375	0.9254	0.8003	0.8842	0.0609
GAGE12I	0.7557	1.0933	0.9797	0.7080	0.8842	0.0915
FAM84A	0.8109	1.0272	0.9128	0.7858	0.8842	0.0550
RBM35A	0.8848	1.0465	0.8068	0.7989	0.8842	0.0574
LEMD2	0.8369	1.0912	0.8206	0.7883	0.8843	0.0697
MRPS18C	0.7611	1.3051	0.8049	0.6660	0.8843	0.1432
MPP6	0.7323	1.1641	0.9039	0.7368	0.8843	0.1015
FKBP10	0.8577	1.0453	0.8547	0.7795	0.8843	0.0566
LOC644827	0.9643	0.8674	0.9051	0.8007	0.8844	0.0343
LOC728635	0.7750	1.2460	0.8428	0.6738	0.8844	0.1254
NFX1	0.7299	1.1077	1.0003	0.6996	0.8844	0.1006
COMMD4	0.7083	1.2403	0.8456	0.7434	0.8844	0.1222
ZC3HAV1	0.7448	1.2048	0.8867	0.7015	0.8844	0.1139
TIFA	0.7905	1.0626	0.8191	0.8656	0.8845	0.0614
PSTK	0.8063	1.0162	0.9110	0.8044	0.8845	0.0505
KLHDC2	0.8433	1.0159	1.0022	0.6764	0.8845	0.0797
LOC730455	0.7514	1.2078	0.8742	0.7046	0.8845	0.1135
TM9SF1	0.8304	1.1878	0.7889	0.7309	0.8845	0.1031
ZNF467	0.7875	1.1883	0.8373	0.7250	0.8845	0.1038
MAP3K11	0.8552	1.0045	0.9528	0.7256	0.8845	0.0613
ZFPL1	0.8377	0.9939	0.8815	0.8252	0.8846	0.0384
TMEM181	0.8412	1.2011	0.8155	0.6806	0.8846	0.1112
SNX27	0.8282	1.2164	0.8127	0.6810	0.8846	0.1154
LOC1001298	0.9666	1.0123	0.8864	0.6738	0.8848	0.0750
TCEB1	0.7325	1.0724	1.0581	0.6763	0.8848	0.1048

HIST1H3B	0.7982	0.9876	0.9610	0.7926	0.8849	0.0519
MED14	0.7827	1.1293	0.8679	0.7595	0.8849	0.0848
C17orf95	0.7431	1.2371	0.8622	0.6971	0.8849	0.1225
LOC402175	0.7577	1.1519	0.9318	0.6982	0.8849	0.1019
TMEM11	0.8292	1.0643	0.9736	0.6725	0.8849	0.0858
LOC728945	0.7986	0.9996	0.9637	0.7779	0.8849	0.0565
E2F1	0.6640	0.9673	1.0282	0.8804	0.8850	0.0797
M6PRBP1	0.8062	1.0487	1.0417	0.6433	0.8850	0.0983
RPAP2	0.6391	1.2518	0.8722	0.7768	0.8850	0.1313
LOC652675	0.7743	0.9750	0.9085	0.8822	0.8850	0.0418
CCNY	0.7563	1.1224	0.9478	0.7136	0.8850	0.0941
DSTYK	0.7990	1.1839	0.8132	0.7443	0.8851	0.1007
SLC30A1	0.7467	1.0860	0.9473	0.7605	0.8851	0.0811
TYK2	0.8074	1.1967	0.7901	0.7464	0.8851	0.1046
NR2C2	0.6452	1.2367	0.9366	0.7220	0.8851	0.1324
LOC1001304	0.8164	0.8844	0.8586	0.9811	0.8851	0.0349
C20orf45	0.7058	1.2830	0.8847	0.6671	0.8852	0.1408
WDR73	0.7150	1.2440	0.8048	0.7770	0.8852	0.1211
LOC1001280	0.8272	1.0355	0.8415	0.8367	0.8852	0.0502
ARPC1A	0.7577	1.2290	0.8929	0.6614	0.8852	0.1240
DPH1	0.8699	1.1039	0.8110	0.7562	0.8852	0.0765
RAI1	0.8061	1.1578	0.8582	0.7190	0.8853	0.0953
MED24	0.7927	1.2301	0.7857	0.7326	0.8853	0.1157
GNL3	0.7136	1.2381	0.8555	0.7341	0.8853	0.1217
STAG3L2	0.6861	1.2647	0.7666	0.8240	0.8853	0.1296
POLR2E	0.8125	1.1000	0.8282	0.8006	0.8853	0.0718
TMEM132A	1.0572	1.0680	0.7466	0.6696	0.8854	0.1036
PDK2	0.8635	0.9995	0.8769	0.8016	0.8854	0.0414
PMVK	1.0179	1.1915	0.7515	0.5807	0.8854	0.1360
C20orf186	0.9554	0.9240	0.8737	0.7886	0.8854	0.0364
KLHL21	0.8519	1.1599	0.8296	0.7005	0.8855	0.0974
BSG	0.7170	1.3055	0.8876	0.6318	0.8855	0.1498
LOC646210	0.8725	0.8872	0.8792	0.9030	0.8855	0.0066
P2RY11	0.7537	1.1226	0.8559	0.8098	0.8855	0.0818
KIAA0258	0.8642	0.9560	0.8546	0.8673	0.8855	0.0236
LOC652874	0.9527	0.9255	0.8284	0.8356	0.8855	0.0314
ZNF706	0.6887	1.1233	0.9597	0.7707	0.8856	0.0975
LDLRAP1	0.9225	1.0598	0.7702	0.7901	0.8856	0.0672
LOC652736	0.7971	0.9881	0.9317	0.8258	0.8857	0.0448
KTN1	0.6944	1.0580	1.0668	0.7237	0.8857	0.1022
LOC1001321	0.8644	0.9584	0.9795	0.7406	0.8857	0.0545
PPP2R5E	0.7379	1.1651	1.0096	0.6303	0.8858	0.1226
LOC651829	0.9012	0.8841	0.9583	0.7994	0.8858	0.0329
NAGA	0.8794	1.0615	0.8495	0.7528	0.8858	0.0645
KIAA0196	0.8204	1.1322	0.9230	0.6677	0.8858	0.0974
C2CD2	0.8825	1.1442	0.8541	0.6626	0.8858	0.0990

PRR13	0.7330	1.1834	0.9502	0.6767	0.8858	0.1154
MAPK13	0.8049	1.2090	0.8466	0.6830	0.8859	0.1132
CBLN3	0.8246	1.0861	0.9407	0.6922	0.8859	0.0838
PTAR1	0.6978	1.2176	0.9717	0.6564	0.8859	0.1309
DDX19A	0.7862	1.1210	0.8585	0.7779	0.8859	0.0804
TRA1P2	0.8161	1.0400	0.9038	0.7838	0.8859	0.0573
UQCRC2	0.7916	0.9473	1.3431	0.4617	0.8859	0.1829
LOC645317	0.6411	1.0651	1.0795	0.7582	0.8860	0.1102
ZDHHC4	0.8029	1.1966	0.8457	0.6990	0.8861	0.1080
SLC35C2	0.7928	1.1152	0.8546	0.7818	0.8861	0.0780
LOC572558	0.7292	0.9836	1.0834	0.7482	0.8861	0.0876
IFITM3	0.7368	1.2238	0.8972	0.6867	0.8861	0.1212
LOC1001288	0.8490	1.0001	0.8252	0.8702	0.8861	0.0391
ZNF324B	0.7414	1.2237	0.8494	0.7300	0.8862	0.1157
HIST2H3D	0.7844	1.0820	0.9040	0.7743	0.8862	0.0716
FASTK	0.7344	1.2135	0.9013	0.6955	0.8862	0.1179
CCT3	0.6141	1.4134	0.9034	0.6141	0.8862	0.1885
LOC643918	0.8261	0.9799	0.8787	0.8604	0.8863	0.0331
ZNF213	0.8145	1.0542	0.9292	0.7473	0.8863	0.0674
LOC650938	0.9764	0.9179	0.8515	0.7994	0.8863	0.0386
RFP	0.7755	1.1650	0.8207	0.7840	0.8863	0.0934
TMEM107	0.7951	0.9521	0.9936	0.8046	0.8863	0.0507
KDELR3	0.7740	1.0887	0.9518	0.7308	0.8863	0.0827
BTBD7	0.8138	1.1049	0.8614	0.7654	0.8864	0.0754
DPP7	0.7145	1.2066	0.9131	0.7115	0.8864	0.1167
IFRD1	0.8702	1.0426	0.8513	0.7815	0.8864	0.0554
OPA1	0.7350	1.1562	0.8986	0.7560	0.8864	0.0970
CCDC106	0.7232	1.2239	0.9259	0.6728	0.8864	0.1251
NCR3	0.8793	0.9518	0.8895	0.8252	0.8864	0.0259
TMEM80	0.8072	1.1684	0.7797	0.7904	0.8864	0.0942
PPP2R3B	0.8120	0.9476	0.9444	0.8418	0.8865	0.0349
C22orf26	0.8182	0.9573	0.9250	0.8454	0.8865	0.0327
LOC1001291	0.8437	1.0256	0.8871	0.7896	0.8865	0.0505
AP1S1	0.7675	1.2055	0.8636	0.7097	0.8866	0.1109
MUC5AC	0.8510	0.9792	0.8640	0.8522	0.8866	0.0310
LOC401152	0.8954	1.0223	0.7622	0.8668	0.8867	0.0535
ENAH	0.8531	0.9943	0.8964	0.8030	0.8867	0.0406
EIF4A3	0.7120	1.1942	0.9779	0.6629	0.8867	0.1236
DLG3	0.8742	1.0580	0.8441	0.7708	0.8868	0.0611
LOC652634	0.8655	0.9252	0.9180	0.8383	0.8868	0.0209
IRF1	0.6813	1.1073	0.9700	0.7886	0.8868	0.0946
LOC728004	0.8205	0.9625	0.9404	0.8237	0.8868	0.0376
LOC1001287	0.8019	0.9681	0.9675	0.8097	0.8868	0.0468
LOC648370	0.8463	1.0095	0.9398	0.7516	0.8868	0.0561
INTS9	0.7854	1.1566	0.8699	0.7355	0.8868	0.0941
ZNF786	0.7132	1.2287	0.8128	0.7927	0.8869	0.1160

TMEM201	0.8316	0.9959	0.9206	0.7994	0.8869	0.0445
NRIP2	0.9126	0.9084	0.8624	0.8642	0.8869	0.0137
FAM149A	0.8438	0.9342	0.9215	0.8481	0.8869	0.0238
IGF2AS	0.8191	0.8972	0.8594	0.9719	0.8869	0.0325
MGC48628	0.8372	0.8362	1.0027	0.8718	0.8870	0.0395
LAP3	0.8829	1.0905	0.9808	0.5939	0.8870	0.1065
GADD45B	0.7835	1.1429	0.7809	0.8409	0.8870	0.0864
ZBTB39	0.8976	1.0024	0.8173	0.8312	0.8871	0.0422
DMBX1	0.8586	1.0170	0.8849	0.7880	0.8871	0.0479
LOC389199	0.7803	0.9741	0.9300	0.8641	0.8871	0.0422
ZNF2	0.8069	1.0505	0.8627	0.8285	0.8872	0.0557
LOC646697	0.7176	0.9876	0.9484	0.8952	0.8872	0.0596
MEF2B	0.9161	0.8472	0.9419	0.8436	0.8872	0.0247
LOC1001289	0.6689	1.2145	0.9438	0.7216	0.8872	0.1243
MRPS18A	0.7796	1.1905	0.8636	0.7152	0.8872	0.1056
TP53BP2	0.8097	1.1759	0.8677	0.6957	0.8872	0.1026
CXXC6	0.8186	1.0324	0.9006	0.7978	0.8874	0.0532
FTSJD2	0.8168	1.1348	0.7654	0.8326	0.8874	0.0837
BANF1	0.6752	1.2451	0.8842	0.7451	0.8874	0.1269
LOC727826	0.8415	0.9380	1.0897	0.6804	0.8874	0.0859
IQGAP1	0.8199	1.0673	0.9355	0.7270	0.8874	0.0736
FPR1	0.8394	0.9655	0.8231	0.9217	0.8874	0.0338
CDC5L	0.8148	1.0369	1.0369	0.6614	0.8875	0.0918
SHFM1	0.7906	1.1638	0.9516	0.6443	0.8876	0.1114
DENND4B	0.8367	1.1332	0.7826	0.7978	0.8876	0.0827
SLMO1	0.8002	1.0427	0.8615	0.8459	0.8876	0.0533
MST4	0.7931	1.0041	1.0653	0.6878	0.8876	0.0885
SEC31A	0.8218	1.1214	0.9573	0.6499	0.8876	0.1001
MARVELD3	0.7637	1.1039	0.8742	0.8085	0.8876	0.0756
MUS81	0.7697	1.3120	0.8566	0.6122	0.8876	0.1502
LOC1001285	0.7486	1.0893	0.8715	0.8411	0.8876	0.0721
GRHPR	0.7794	1.1850	0.9141	0.6721	0.8877	0.1108
THAP7	0.7435	1.0932	0.9308	0.7831	0.8877	0.0795
SIGIRR	0.7705	1.1860	0.8536	0.7406	0.8877	0.1023
LOC441528	0.8181	0.8859	0.9724	0.8744	0.8877	0.0319
CBX6	0.7314	1.2002	1.0405	0.5788	0.8877	0.1417
FLJ35767	0.8038	0.9659	0.9129	0.8684	0.8877	0.0343
CATSPER2P1	0.7688	1.0220	0.8851	0.8752	0.8878	0.0519
LOC642513	0.9675	0.9482	0.8826	0.7527	0.8878	0.0485
KIAA0141	0.9714	0.9713	0.9151	0.6935	0.8878	0.0661
LOC728138	0.9049	1.1156	0.8725	0.6587	0.8879	0.0935
USP10	0.7971	1.1733	0.8828	0.6988	0.8880	0.1022
ARIH2	0.6898	1.2509	0.8747	0.7366	0.8880	0.1272
LGALS2	0.7798	0.9705	0.9270	0.8752	0.8881	0.0410
PHTF1	0.8432	0.9358	0.9783	0.7953	0.8881	0.0419
AMY1A	0.7840	0.9510	0.9761	0.8415	0.8882	0.0454

PQLC1	0.7178	1.1431	0.9352	0.7566	0.8882	0.0973
UTP23	0.6845	1.0560	1.0092	0.8029	0.8882	0.0874
RBL1	0.8146	1.0270	0.8992	0.8121	0.8882	0.0505
RASSF2	0.8461	1.0782	0.8982	0.7305	0.8882	0.0724
LOC1001282	0.8394	0.9877	0.8866	0.8393	0.8883	0.0350
ZNF444	0.9575	1.0321	0.8826	0.6809	0.8883	0.0756
SNORD34	0.7648	0.9908	1.0421	0.7556	0.8883	0.0747
ZDHHC12	0.8872	1.1250	0.9061	0.6353	0.8884	0.1001
CDCP1	0.6906	1.1709	0.8714	0.8208	0.8884	0.1016
STRAP	0.7043	1.0841	1.1224	0.6427	0.8884	0.1249
ZNF652	0.7152	1.1967	0.9369	0.7048	0.8884	0.1159
ATP10A	0.7723	1.0726	0.9447	0.7641	0.8884	0.0742
C7orf54	0.8488	1.0911	0.7226	0.8911	0.8884	0.0765
PRKX	0.8852	1.1060	0.8380	0.7244	0.8884	0.0800
LOC729505	0.8098	0.9053	0.9695	0.8691	0.8884	0.0334
COPS8	0.8792	1.0373	0.9780	0.6593	0.8885	0.0831
RASA1	0.7947	1.1426	0.8894	0.7272	0.8885	0.0910
RNF135	0.8473	1.0382	0.9246	0.7439	0.8885	0.0621
GSPT1	0.7871	1.1649	0.9619	0.6401	0.8885	0.1132
LOC647606	0.7755	0.8321	1.0807	0.8660	0.8886	0.0667
TICAM1	0.7012	1.1408	1.1098	0.6025	0.8886	0.1383
C11orf10	0.8327	1.0929	0.9294	0.6993	0.8886	0.0829
RELL1	0.8391	1.0098	0.9998	0.7057	0.8886	0.0724
CHMP2A	0.6807	1.3050	0.9428	0.6259	0.8886	0.1551
GTF3C5	0.7646	1.2287	0.8449	0.7163	0.8886	0.1164
HSP90B1	0.7454	1.2163	0.9644	0.6285	0.8886	0.1295
LOC645086	0.8058	1.0262	0.9133	0.8092	0.8886	0.0522
ZHX3	0.7719	1.1534	0.8970	0.7324	0.8887	0.0950
LOC651589	0.8682	0.8560	0.8930	0.9375	0.8887	0.0180
B3GALTL	0.8088	1.0377	0.8394	0.8689	0.8887	0.0512
BHLHA15	0.6996	0.9247	0.9680	0.9627	0.8888	0.0638
LOC645609	0.6538	1.3295	0.8688	0.7030	0.8888	0.1539
GLCCI1	0.8047	1.0009	0.9253	0.8242	0.8888	0.0458
TNFRSF18	0.9005	0.9387	0.8724	0.8438	0.8888	0.0203
RBM4B	0.7327	1.1991	0.8886	0.7350	0.8889	0.1097
USF1	0.6415	1.2580	1.0196	0.6363	0.8889	0.1523
PPAPDC2	0.9318	1.1619	0.8457	0.6161	0.8889	0.1128
ABHD15	0.8159	0.9689	0.9367	0.8340	0.8889	0.0377
KIAA0907	0.8510	1.1192	0.8610	0.7244	0.8889	0.0828
TMEM200B	0.9289	1.0620	0.8960	0.6688	0.8889	0.0817
POLDIP2	0.7015	1.1338	0.9399	0.7805	0.8890	0.0955
KIAA0586	0.8406	1.1482	0.9020	0.6649	0.8890	0.1000
MRPL43	0.7968	1.2714	0.8283	0.6593	0.8890	0.1327
CEP72	0.7204	1.0516	0.9084	0.8756	0.8890	0.0680
LOC654254	0.7533	1.2068	0.9416	0.6543	0.8890	0.1215
INTS5	0.7326	1.1559	0.9368	0.7308	0.8890	0.1012

TOR1AIP2	0.6295	1.1301	1.0282	0.7683	0.8891	0.1153
ASPHD2	0.7749	0.9854	0.8973	0.8987	0.8891	0.0433
SLC16A14	0.8775	0.9730	0.9702	0.7356	0.8891	0.0558
ARHGEF16	0.8370	1.0904	0.8965	0.7325	0.8891	0.0752
KIAA1310	0.7747	1.2660	0.8247	0.6909	0.8891	0.1286
ZNF816A	0.7992	1.1906	0.9264	0.6402	0.8891	0.1163
EBAG9	0.7357	1.0659	1.0511	0.7038	0.8891	0.0981
ZNF16	0.8642	1.0399	0.8739	0.7786	0.8891	0.0546
ARHGAP11A	0.8707	0.9924	0.8770	0.8166	0.8892	0.0370
EIF2AK4	0.7546	1.2226	0.8090	0.7704	0.8892	0.1117
DHFRL1	0.7830	1.1085	0.8502	0.8149	0.8892	0.0744
VAMP8	0.7290	1.2590	0.9086	0.6602	0.8892	0.1339
OR9A4	0.7495	1.0205	0.9903	0.7965	0.8892	0.0680
LOC1001312	0.5496	1.0436	1.2781	0.6856	0.8892	0.1663
LOC648609	0.8477	0.9354	0.9628	0.8111	0.8892	0.0358
RB1CC1	0.8775	1.0814	0.9138	0.6842	0.8892	0.0815
SPCS1	0.7083	1.1434	0.9969	0.7085	0.8893	0.1086
LOC1001340	0.8739	1.0320	0.8688	0.7823	0.8893	0.0520
SFRS12	0.7959	1.0095	0.9329	0.8189	0.8893	0.0500
ZNF37B	0.8444	0.9564	0.9375	0.8191	0.8894	0.0339
GALR2	0.8275	1.1070	0.8341	0.7888	0.8894	0.0732
ZNF320	0.7252	1.1840	0.8933	0.7550	0.8894	0.1048
LOC730202	0.7007	1.2416	0.7931	0.8222	0.8894	0.1202
C19orf28	0.7660	1.1396	0.8861	0.7660	0.8894	0.0881
C1orf77	0.6648	1.2576	0.8473	0.7881	0.8895	0.1285
NXT2	0.8325	0.9202	1.0871	0.7182	0.8895	0.0778
MMGT1	0.8134	1.0871	0.9588	0.6988	0.8895	0.0846
SIRT7	0.8085	1.0078	0.9369	0.8048	0.8895	0.0500
FLJ40473	0.8752	1.0783	0.7541	0.8506	0.8895	0.0681
TDRD3	0.7348	0.9962	0.9753	0.8521	0.8896	0.0606
SMAD1	0.8384	1.0493	0.8936	0.7771	0.8896	0.0583
EIF2C3	0.8277	0.9567	0.9058	0.8682	0.8896	0.0275
NOMO2	0.8702	1.0878	0.9688	0.6319	0.8897	0.0968
LOC732419	0.7522	1.0792	0.9608	0.7666	0.8897	0.0791
ANKRA2	0.7967	1.2190	0.8083	0.7349	0.8897	0.1109
PLOD1	0.8733	1.3396	0.7184	0.6277	0.8897	0.1583
MIR564	0.8161	1.0973	0.9352	0.7103	0.8897	0.0831
QRFPR	0.7365	1.1084	1.0052	0.7090	0.8898	0.0989
PABPC1L	0.7657	1.1420	0.7832	0.8682	0.8898	0.0870
C1orf144	0.6408	1.1837	0.9197	0.8149	0.8898	0.1136
ZNF12	0.8480	0.9971	0.9408	0.7734	0.8898	0.0495
FGFR1OP	0.8103	1.1362	0.8499	0.7630	0.8899	0.0840
ZMYND15	0.8354	1.0816	0.9409	0.7016	0.8899	0.0805
KBTBD6	0.6736	1.0565	0.9888	0.8406	0.8899	0.0850
KDM4D	0.8789	0.9572	0.9350	0.7885	0.8899	0.0376
PLA2G15	0.8904	1.0812	0.8399	0.7483	0.8899	0.0702

LOC730324	0.6623	1.2968	0.9573	0.6435	0.8900	0.1535
MBD6	0.8896	1.0786	0.7569	0.8348	0.8900	0.0685
STAU2	0.9129	1.1418	0.7693	0.7360	0.8900	0.0923
REM2	0.8463	1.0269	0.9385	0.7483	0.8900	0.0599
NDUFA8	0.6883	1.2002	1.0490	0.6226	0.8900	0.1395
LOC729366	0.7773	1.0351	0.9808	0.7670	0.8900	0.0690
LOC647346	0.8179	1.0541	0.9074	0.7809	0.8901	0.0608
LOC643717	0.8040	0.8712	0.9920	0.8932	0.8901	0.0389
HNF1B	0.8188	1.0722	0.8471	0.8224	0.8901	0.0610
EIF4B	0.8663	1.0191	0.9244	0.7507	0.8901	0.0561
SLC39A3	0.7708	1.1667	0.8524	0.7707	0.8901	0.0942
LOC728031	0.7077	1.1116	1.0711	0.6702	0.8901	0.1167
LOC389641	0.7304	1.1658	1.0013	0.6632	0.8902	0.1174
LOC400558	0.9125	0.9138	0.9445	0.7901	0.8902	0.0342
RFX1	0.8749	1.0172	0.8813	0.7876	0.8902	0.0474
UPF3A	0.8379	1.1528	0.8514	0.7188	0.8902	0.0925
PPT1	0.6988	1.0083	1.1361	0.7177	0.8902	0.1083
TRPM1	0.9083	0.9361	0.8613	0.8553	0.8902	0.0193
AKAP1	0.8340	0.9968	0.9933	0.7369	0.8903	0.0637
SCARNA13	0.7467	0.9409	0.9650	0.9084	0.8903	0.0492
EIF1	0.7628	1.1393	1.0079	0.6511	0.8903	0.1115
LOC647805	0.9078	0.8576	0.9694	0.8264	0.8903	0.0312
NT5DC1	0.9337	1.0715	0.8770	0.6790	0.8903	0.0814
PPM1A	0.5941	1.2397	0.9508	0.7766	0.8903	0.1374
C12orf47	0.7644	1.1940	1.0045	0.5984	0.8903	0.1311
LSM11	0.7764	0.9826	0.9093	0.8931	0.8904	0.0427
PRDX5	0.7037	1.2223	0.9176	0.7178	0.8904	0.1209
RENBP	0.7670	1.0142	0.8616	0.9187	0.8904	0.0518
LOC152586	0.7487	1.0361	0.9326	0.8442	0.8904	0.0614
SERTAD2	0.7640	1.1413	0.9579	0.6984	0.8904	0.1002
LOC729930	0.7696	0.9158	0.9825	0.8940	0.8905	0.0445
CTBP2	0.7651	1.0228	0.9842	0.7899	0.8905	0.0659
CTRL	0.8309	0.9221	0.9250	0.8839	0.8905	0.0220
RSAD1	0.8139	1.0466	0.9200	0.7815	0.8905	0.0599
LOC400721	0.7282	1.2534	0.8820	0.6985	0.8905	0.1275
TMEM117	0.8190	1.0598	0.9794	0.7040	0.8906	0.0798
PLAT	0.8816	0.9334	0.8605	0.8867	0.8906	0.0154
DEF6	0.7761	1.0115	0.8539	0.9211	0.8906	0.0500
LOC1001339	0.7778	1.0962	0.8256	0.8631	0.8907	0.0707
TMEM1	0.8408	1.0122	0.9250	0.7847	0.8907	0.0497
PDLIM7	0.8132	1.0295	0.9185	0.8016	0.8907	0.0532
MAP3K7IP1	0.7973	1.1081	0.8564	0.8010	0.8907	0.0737
SLC46A3	0.7650	1.0804	0.8701	0.8475	0.8907	0.0671
HS6ST2	0.8485	1.0107	0.9272	0.7767	0.8908	0.0504
GLE1	0.8058	1.1717	0.8533	0.7323	0.8908	0.0969
SPIN3	0.8082	1.1568	0.9217	0.6765	0.8908	0.1018

SLC35F1	0.7483	1.0752	0.9158	0.8240	0.8908	0.0703
GAGE2B	0.7947	1.1683	0.9464	0.6539	0.8908	0.1101
RAB3D	0.8223	0.9681	0.8797	0.8933	0.8909	0.0300
LOC253820	0.9324	0.8867	0.8532	0.8912	0.8909	0.0162
USP6NL	0.8533	1.0877	0.8601	0.7624	0.8909	0.0693
LOC441054	0.8610	0.9611	0.8934	0.8480	0.8909	0.0253
LSM12	0.7078	1.1761	0.9561	0.7235	0.8909	0.1107
C8orf33	0.7933	1.1986	0.7938	0.7779	0.8909	0.1026
DHPS	0.8102	1.1842	0.8703	0.6990	0.8909	0.1040
LOC1001347	0.8524	0.9217	0.9192	0.8707	0.8910	0.0174
LASS2	0.7695	1.1991	0.9817	0.6137	0.8910	0.1274
LOC729085	0.8351	0.9896	0.9742	0.7650	0.8910	0.0545
TMEM187	0.8424	1.2913	0.8193	0.6110	0.8910	0.1432
RNF41	0.8036	1.2265	0.8754	0.6586	0.8910	0.1206
ZC3H8	0.7993	1.0408	0.9690	0.7551	0.8910	0.0679
C17orf41	0.7995	1.0154	0.8864	0.8629	0.8911	0.0453
MGC10997	0.8169	1.0677	0.9758	0.7038	0.8911	0.0811
HNRNPA3P1	0.6808	1.0568	1.0249	0.8018	0.8911	0.0902
LOC646981	0.6850	1.0717	1.0262	0.7815	0.8911	0.0937
RPGRIP1L	0.8287	0.9742	0.8860	0.8757	0.8911	0.0304
LPGAT1	0.8218	1.0569	0.8730	0.8130	0.8912	0.0568
ATP5A1	0.7055	1.2004	0.9992	0.6596	0.8912	0.1276
RNF114	0.7068	1.2234	0.9441	0.6906	0.8912	0.1250
TSR1	0.6664	1.2951	0.9091	0.6944	0.8912	0.1451
GATAD1	0.7565	1.1241	0.8951	0.7892	0.8912	0.0831
NEK4	0.6830	1.1539	0.9389	0.7893	0.8913	0.1021
HMBOX1	0.6687	1.2408	0.8227	0.8330	0.8913	0.1224
MECR	0.8176	1.0482	0.9491	0.7502	0.8913	0.0667
DIAPH2	0.8010	1.0458	0.9209	0.7976	0.8913	0.0589
FKBP1A	0.6991	1.4018	0.7974	0.6671	0.8913	0.1724
SNORD99	0.8045	1.0538	0.9482	0.7589	0.8914	0.0675
LOC649977	0.8838	0.9430	0.8723	0.8665	0.8914	0.0176
CCDC109B	0.7462	0.9499	0.8745	0.9952	0.8914	0.0545
LOC653079	0.6132	1.1320	1.1615	0.6591	0.8915	0.1478
LOC644046	0.8336	0.9020	1.0145	0.8158	0.8915	0.0450
B3GNT8	0.8542	0.9685	0.8881	0.8550	0.8915	0.0269
FOXO1	0.7778	1.0865	0.9422	0.7594	0.8915	0.0769
FAM72B	0.8423	1.0292	0.8757	0.8188	0.8915	0.0474
SMAP1	0.9188	0.9540	0.9384	0.7550	0.8916	0.0461
EDEM2	0.7351	1.2255	0.9059	0.6998	0.8916	0.1200
FOXRED2	0.7398	1.1570	0.8783	0.7912	0.8916	0.0930
ROBO3	0.7863	1.1303	0.8745	0.7754	0.8916	0.0826
COX6B1	0.7129	1.2962	0.8878	0.6696	0.8916	0.1429
GSTP1	0.7632	1.0470	0.8204	0.9360	0.8916	0.0630
TEX264	0.7498	1.2768	0.8783	0.6618	0.8917	0.1358
C22orf39	0.7079	1.1681	0.9599	0.7308	0.8917	0.1083

HRIHFB2122	1.0009	0.9353	0.9513	0.6793	0.8917	0.0722
GRM2	0.7877	1.0833	0.8500	0.8458	0.8917	0.0654
ZNF524	0.8147	1.0893	0.9112	0.7517	0.8917	0.0736
NEO1	0.6824	1.1430	0.9517	0.7899	0.8917	0.1004
COQ2	0.6646	1.1037	0.9912	0.8075	0.8917	0.0973
WNT3	0.7925	0.9619	0.9309	0.8818	0.8918	0.0370
CCNK	0.6456	1.3808	0.8307	0.7101	0.8918	0.1674
MID2	0.7903	1.1427	0.8774	0.7569	0.8918	0.0874
E4F1	0.7989	1.0789	0.8811	0.8084	0.8918	0.0650
GPR160	0.7272	1.1172	1.0247	0.6985	0.8919	0.1053
LOC440396	0.8021	0.9417	0.9369	0.8868	0.8919	0.0324
COMMD2	0.7010	1.0935	1.0777	0.6958	0.8920	0.1118
LOC730809	0.7918	1.1090	0.8614	0.8059	0.8920	0.0739
GPS1	0.6862	1.1876	0.9098	0.7845	0.8920	0.1086
TMEM121	0.8117	1.0057	0.8358	0.9150	0.8921	0.0438
C10orf46	0.8305	1.0207	0.9278	0.7894	0.8921	0.0518
LOC1001343	0.8042	0.9835	0.9197	0.8609	0.8921	0.0385
LRRC49	0.7403	1.1493	0.9549	0.7239	0.8921	0.1006
LOC441662	0.8160	0.9885	0.9346	0.8295	0.8922	0.0416
THAP1	0.7471	1.1264	0.9713	0.7238	0.8922	0.0960
LOC730077	0.8683	1.0425	0.8350	0.8231	0.8922	0.0510
PLAA	0.7723	1.0759	0.9302	0.7905	0.8922	0.0707
LRWD1	0.6785	1.3823	0.8979	0.6103	0.8923	0.1745
PTBP2	0.7138	1.1326	0.9629	0.7598	0.8923	0.0967
AARSD1	0.7806	1.2453	0.8395	0.7039	0.8923	0.1209
LOC730041	0.7452	0.9966	0.9770	0.8507	0.8924	0.0588
UBR5	0.8224	1.2344	0.8478	0.6650	0.8924	0.1209
NAT14	0.8027	1.2846	0.8525	0.6302	0.8925	0.1391
TRIM24	0.7762	0.9880	1.0807	0.7252	0.8925	0.0847
C1orf159	0.8369	0.9714	0.8719	0.8901	0.8926	0.0285
MRPS21	0.7450	1.1552	1.0207	0.6495	0.8926	0.1177
LOC1001294	0.7388	0.9313	0.9467	0.9536	0.8926	0.0515
LOC642290	0.8110	1.0607	0.9239	0.7748	0.8926	0.0644
MIR1914	0.9294	0.9222	0.9123	0.8066	0.8926	0.0289
MGC2752	0.7155	1.2371	0.9443	0.6739	0.8927	0.1293
PPP2R1B	0.7598	1.0638	0.9771	0.7703	0.8928	0.0758
C11orf61	0.8159	1.0758	0.8703	0.8091	0.8928	0.0625
DMXL2	0.8516	1.1126	0.7973	0.8096	0.8928	0.0742
USP37	0.8499	1.1401	0.8327	0.7485	0.8928	0.0854
JARID1D	0.8829	1.1055	0.8511	0.7318	0.8928	0.0780
FBXL15	0.7906	1.0558	0.9376	0.7874	0.8928	0.0646
PITPNB	0.8227	1.0802	0.9355	0.7330	0.8928	0.0749
MRPL13	0.7183	1.0990	1.0371	0.7170	0.8929	0.1019
LOC643109	0.8919	0.8624	0.9060	0.9110	0.8929	0.0109
TTC32	0.7589	1.1831	0.8867	0.7429	0.8929	0.1019
MEN1	0.8622	1.0206	0.9158	0.7731	0.8929	0.0517

ERGIC1	0.7240	1.2729	0.9129	0.6619	0.8929	0.1374
NDUFS2	0.8302	1.0036	0.9860	0.7519	0.8929	0.0611
ARRDC2	0.7610	1.0599	0.9982	0.7527	0.8930	0.0796
LOC644701	0.9004	0.8018	0.9057	0.9641	0.8930	0.0337
NFU1	0.7843	1.0711	0.9926	0.7241	0.8930	0.0827
KIF24	0.7540	1.0304	0.9769	0.8107	0.8930	0.0658
TMEM87B	0.7448	1.1384	0.9773	0.7116	0.8930	0.1009
ANO10	0.8378	1.1854	0.8755	0.6738	0.8931	0.1068
NHEDC2	0.7961	0.9380	0.9483	0.8902	0.8931	0.0347
LOC400061	0.7295	1.0192	1.0457	0.7782	0.8932	0.0812
ARL5B	0.7880	1.0511	0.9596	0.7741	0.8932	0.0675
CAMKV	0.8774	0.9997	0.8270	0.8689	0.8932	0.0371
NHLRC3	0.8865	0.9316	0.9989	0.7561	0.8933	0.0512
METTL2A	0.7615	1.0270	0.9636	0.8210	0.8933	0.0615
ZNF185	0.9544	1.0170	0.9027	0.6989	0.8933	0.0689
C6orf105	0.8157	1.0087	0.9056	0.8431	0.8933	0.0428
SETMAR	0.7226	1.2133	0.9944	0.6429	0.8933	0.1305
PSMD6	0.7600	1.0865	1.0390	0.6878	0.8933	0.0994
POLR3D	0.8114	1.0401	1.0046	0.7173	0.8933	0.0773
OTUD1	0.8764	0.9554	0.9591	0.7825	0.8933	0.0416
FBXO3	0.7973	1.1866	0.8932	0.6963	0.8934	0.1057
ARPC4	0.7118	1.2341	0.9202	0.7073	0.8934	0.1240
UBE2J2	0.8926	1.0243	0.8791	0.7774	0.8934	0.0507
LOC168474	0.8615	0.9360	1.0113	0.7647	0.8934	0.0527
LOC1001320	0.8440	1.0609	0.8336	0.8350	0.8934	0.0559
ARID4B	0.7861	1.1346	0.9841	0.6689	0.8934	0.1034
LOC653214	0.8147	0.9235	0.9559	0.8796	0.8934	0.0305
C3orf75	0.7644	1.1777	0.8945	0.7372	0.8935	0.1008
SENP7	0.7950	1.0965	0.8662	0.8162	0.8935	0.0693
PLEKHA9	0.7384	1.1564	0.8678	0.8116	0.8935	0.0915
DPY19L3	0.7418	1.0818	0.9241	0.8266	0.8936	0.0730
C4orf14	0.7841	1.1284	0.9760	0.6860	0.8936	0.0987
HDGFRP3	0.9338	0.9091	1.0007	0.7309	0.8936	0.0576
STAG3L3	0.7185	1.1694	0.8554	0.8314	0.8937	0.0966
LOC389293	0.8681	0.9810	1.0518	0.6740	0.8937	0.0824
CBR1	0.7869	0.9730	0.9481	0.8670	0.8938	0.0422
DENND2C	0.8000	1.0460	0.9566	0.7724	0.8938	0.0650
VN1R1	0.8451	0.9353	0.9154	0.8795	0.8938	0.0199
MIR646	0.8209	0.9898	0.9440	0.8206	0.8938	0.0432
MSRA	0.7709	1.0192	1.0374	0.7479	0.8939	0.0779
LOC648408	0.8689	0.9317	0.9451	0.8298	0.8939	0.0270
DHRS4L2	0.6594	1.4475	0.7550	0.7137	0.8939	0.1856
ZBTB48	0.7781	1.2547	0.7851	0.7577	0.8939	0.1204
SNORD88B	0.7789	1.0125	0.8942	0.8901	0.8939	0.0477
MKL2	0.9140	0.9334	0.9459	0.7824	0.8939	0.0377
MAPBPIP	0.8314	1.1181	0.8921	0.7343	0.8940	0.0815

IGSF9	0.9066	1.1381	0.7861	0.7451	0.8940	0.0883
ZUFSP	0.7564	1.1137	0.9355	0.7704	0.8940	0.0838
PHIP	0.7234	1.2038	0.9721	0.6767	0.8940	0.1219
C1orf35	0.7359	1.1837	0.9885	0.6681	0.8940	0.1186
LOC1001325	0.8653	0.9732	0.9905	0.7473	0.8940	0.0562
BDP1	0.7689	1.1177	0.8395	0.8501	0.8940	0.0767
LOC648581	0.8137	1.0533	0.9531	0.7561	0.8941	0.0673
FAM19A4	0.8298	1.0180	0.8846	0.8438	0.8941	0.0429
LOC642888	0.8775	0.9508	0.9276	0.8204	0.8941	0.0289
SPATA13	0.8347	0.9532	0.9521	0.8364	0.8941	0.0338
PRPF40A	0.8297	0.9660	1.0000	0.7809	0.8941	0.0527
ITIH1	0.8322	0.9029	1.0077	0.8338	0.8942	0.0413
RAB2A	0.6888	1.2141	0.9504	0.7233	0.8942	0.1214
LOC339483	0.7800	0.9757	0.9324	0.8889	0.8942	0.0420
DSC3	0.7987	1.0595	0.8983	0.8205	0.8942	0.0591
VGLL4	0.8816	1.3537	0.7800	0.5617	0.8942	0.1671
KIAA1751	0.7148	1.0000	1.0585	0.8038	0.8942	0.0809
PHF6	0.8156	1.0296	0.9200	0.8119	0.8943	0.0516
SFRS6	0.8062	1.1748	0.8126	0.7836	0.8943	0.0937
KCNK1	0.8451	0.9475	1.0260	0.7587	0.8943	0.0585
WDR48	0.7967	1.1551	0.9334	0.6923	0.8944	0.0999
MAP3K14	0.8516	1.0414	0.8494	0.8350	0.8944	0.0492
KIAA0247	0.8159	1.1348	0.8771	0.7497	0.8944	0.0843
ZNF789	0.7917	1.0315	0.9241	0.8301	0.8944	0.0535
LSM6	0.7912	0.9932	0.9916	0.8016	0.8944	0.0566
IFT52	0.7925	1.1667	0.9563	0.6622	0.8944	0.1089
BANP	0.8175	1.1055	0.9195	0.7354	0.8945	0.0798
FLJ16686	0.7628	1.0498	0.9440	0.8213	0.8945	0.0641
IFI16	0.9772	0.8151	0.9031	0.8827	0.8945	0.0334
NR2F6	0.7160	1.3646	0.7963	0.7013	0.8945	0.1581
ARF1	0.7876	1.0869	0.9498	0.7540	0.8946	0.0771
LOC728262	0.7573	1.0217	0.9569	0.8425	0.8946	0.0589
CKMT1B	0.8944	1.0512	1.0159	0.6169	0.8946	0.0985
ZSWIM7	0.9012	1.0542	0.9022	0.7209	0.8946	0.0682
DNAJA2	0.6529	1.2118	1.0351	0.6786	0.8946	0.1371
RNF115	0.8026	1.2191	0.9032	0.6538	0.8946	0.1197
LOC645434	0.8687	1.0442	0.8463	0.8195	0.8947	0.0508
RAB40B	0.8374	1.1814	0.8374	0.7227	0.8947	0.0993
LOC728673	0.8639	0.8720	1.0534	0.7896	0.8947	0.0560
LOC387870	0.7918	0.9498	1.0058	0.8317	0.8948	0.0500
C2orf61	0.8919	0.9614	0.8537	0.8720	0.8948	0.0235
LOC731139	0.8508	1.0166	0.9752	0.7364	0.8948	0.0635
LOC151534	0.8836	0.8738	0.9924	0.8293	0.8948	0.0346
SLC38A7	0.9461	1.0606	0.7817	0.7908	0.8948	0.0669
TSPAN14	0.7319	1.1683	0.8835	0.7955	0.8948	0.0963
NMI	0.8930	1.1012	0.8481	0.7369	0.8948	0.0762

DDX50	0.8042	1.1444	0.9387	0.6920	0.8948	0.0973
MGC26356	0.7670	1.2943	0.9022	0.6158	0.8948	0.1454
CNIH2	0.8786	0.9988	0.8606	0.8413	0.8948	0.0355
PER2	0.7539	1.0852	1.0288	0.7118	0.8949	0.0947
LOC654342	0.7882	1.0891	0.9018	0.8005	0.8949	0.0695
UROS	0.7838	1.1736	0.8906	0.7318	0.8949	0.0986
MDN1	0.7908	1.0054	0.9428	0.8407	0.8949	0.0485
LOC645605	0.9052	0.9581	0.9260	0.7905	0.8949	0.0365
TTLL8	0.8893	0.9281	0.8838	0.8787	0.8950	0.0112
C1orf58	0.6850	1.0440	1.0739	0.7772	0.8950	0.0967
ATPAF2	0.7719	1.1057	0.9601	0.7429	0.8951	0.0851
SATB2	0.8668	1.0904	0.8264	0.7970	0.8952	0.0666
C8orf30B	0.7156	1.0446	0.9993	0.8211	0.8952	0.0769
GPR89A	0.7529	1.2970	0.8104	0.7204	0.8952	0.1352
BEND4	0.8336	1.0578	0.8910	0.7984	0.8952	0.0575
VPS37B	1.0666	1.1647	0.8078	0.5416	0.8952	0.1399
GSTM1L	0.8182	0.9785	0.9472	0.8369	0.8952	0.0398
SHISA4	0.8234	1.0444	0.8707	0.8424	0.8952	0.0507
CRKRS	0.7028	1.1884	0.8999	0.7898	0.8952	0.1057
IER5L	0.8180	1.0114	0.9756	0.7762	0.8953	0.0578
PLEKHG2	0.9231	0.9733	0.9550	0.7298	0.8953	0.0561
C18orf21	0.7208	1.1438	1.0068	0.7099	0.8953	0.1076
ZFP36L1	0.8859	0.8965	1.0527	0.7463	0.8953	0.0626
RAB18	0.7118	1.0807	1.0765	0.7124	0.8953	0.1058
TNRC6A	0.7545	1.1605	0.7959	0.8705	0.8953	0.0916
PSAPL1	0.8799	0.9788	0.9862	0.7366	0.8954	0.0582
SCO1	0.7046	1.1520	1.0463	0.6788	0.8954	0.1197
LOC646426	0.8152	0.9603	0.9782	0.8279	0.8954	0.0429
OVCH2	0.8823	0.9507	0.9698	0.7789	0.8954	0.0431
IK	0.7912	1.2206	0.9026	0.6673	0.8954	0.1186
PMPCA	0.8062	1.2109	0.9111	0.6536	0.8955	0.1177
B4GALT1	0.9449	0.9246	0.9646	0.7479	0.8955	0.0499
RDX	0.7402	1.2091	0.9743	0.6584	0.8955	0.1241
CCNYL1	0.7402	1.1821	0.8933	0.7664	0.8955	0.1012
LOC146177	0.8719	1.0357	0.8787	0.7958	0.8955	0.0504
SMARCC2	0.7413	1.1330	0.9150	0.7929	0.8955	0.0871
TMEM18	0.8459	1.0048	0.9377	0.7938	0.8955	0.0470
NCF1	0.8185	1.0036	0.9611	0.7990	0.8956	0.0510
ZNF319	0.7243	1.2096	0.8606	0.7878	0.8956	0.1083
LOC728686	0.8595	0.9663	0.9116	0.8449	0.8956	0.0276
MGAT4A	0.8669	1.0565	0.8566	0.8025	0.8956	0.0555
DHX37	0.8067	1.2168	0.8542	0.7047	0.8956	0.1115
MOGAT2	0.7832	1.1266	0.8875	0.7852	0.8956	0.0808
FOXP1	0.8236	1.0826	0.9131	0.7633	0.8956	0.0695
KLF10	0.8099	1.0952	0.9043	0.7733	0.8957	0.0720
LOC653219	0.8023	1.1664	0.9448	0.6691	0.8957	0.1064

ERCC8	0.8122	1.0849	0.8834	0.8022	0.8957	0.0656
RBM38	0.8751	1.0069	0.8983	0.8024	0.8957	0.0423
TMEM98	0.7231	1.1763	0.9097	0.7735	0.8957	0.1015
LOC641694	0.7685	1.0885	1.0039	0.7222	0.8958	0.0890
CLDN15	0.8269	1.1172	0.8767	0.7623	0.8958	0.0774
SCARNA18	0.6625	1.0614	1.0532	0.8060	0.8958	0.0977
ADO	0.7825	1.1614	0.9659	0.6732	0.8958	0.1072
HINFP	0.8299	1.1583	0.7707	0.8242	0.8958	0.0885
LOC654126	0.8771	1.0030	0.9009	0.8023	0.8958	0.0415
NUFIP1	0.7854	1.0921	0.8888	0.8171	0.8958	0.0689
CS	0.7851	1.1800	0.9054	0.7129	0.8959	0.1027
C1GALT1C1	0.7640	1.1057	0.9615	0.7523	0.8959	0.0848
EVI5	0.7784	1.1107	0.9817	0.7126	0.8959	0.0917
LOC645123	0.8675	0.8895	1.0153	0.8112	0.8959	0.0431
DARS	0.8222	1.2258	0.9230	0.6125	0.8959	0.1276
LOC730020	0.8047	1.0658	0.9482	0.7650	0.8959	0.0689
LOC728098	0.7921	1.2007	0.8761	0.7149	0.8959	0.1068
GFM2	0.7860	1.2295	0.9227	0.6456	0.8959	0.1247
LOC648740	0.8132	1.0544	0.9376	0.7787	0.8960	0.0629
LOC1001341	0.8422	1.0785	0.7720	0.8911	0.8960	0.0656
SPTBN5	0.8563	1.0719	0.8710	0.7847	0.8960	0.0616
PHF17	0.8006	1.0852	0.9201	0.7782	0.8960	0.0703
DNAJB13	0.8739	0.9711	0.8206	0.9187	0.8961	0.0321
NME5	0.7979	1.2800	0.8373	0.6691	0.8961	0.1329
MIR1253	0.8189	1.0015	0.9424	0.8215	0.8961	0.0454
LOC1001278	0.7747	0.9736	0.9671	0.8695	0.8962	0.0470
LOC1001346	0.6592	1.2411	0.8784	0.8061	0.8962	0.1237
CHRNB2	0.8929	0.9850	1.0057	0.7013	0.8962	0.0694
EIF3C	0.8787	0.9887	0.8668	0.8507	0.8962	0.0313
WDR62	0.7448	1.0241	0.8892	0.9269	0.8963	0.0579
VPS28	0.8572	1.1809	0.8584	0.6887	0.8963	0.1029
SH3PXD2B	0.8375	1.0388	0.9050	0.8039	0.8963	0.0520
B3GNT6	0.7704	1.2084	0.8949	0.7116	0.8963	0.1108
LOC341784	0.7890	1.1419	0.9292	0.7252	0.8963	0.0923
SFT2D1	0.7602	1.2075	0.9313	0.6865	0.8964	0.1157
C9orf85	0.7963	1.0247	1.0039	0.7607	0.8964	0.0686
LOC1001340	0.7986	0.9268	1.0762	0.7839	0.8964	0.0680
SNX8	0.8076	1.1790	0.8985	0.7005	0.8964	0.1025
C5orf28	0.6876	1.2434	0.9364	0.7183	0.8964	0.1282
SUCLA2	0.8386	1.0281	1.0104	0.7086	0.8964	0.0758
PRDM12	0.7523	1.0881	0.9527	0.7927	0.8965	0.0772
LOC730883	0.6821	1.1179	0.9190	0.8669	0.8965	0.0896
GCDH	0.9449	1.0200	0.9309	0.6902	0.8965	0.0715
C9orf95	0.8470	0.9921	1.0991	0.6478	0.8965	0.0977
LOC1001305	0.6829	1.2011	0.9966	0.7058	0.8966	0.1241
PGGT1B	0.7706	1.0909	0.9828	0.7421	0.8966	0.0841

C14orf131	0.8630	1.0535	0.8414	0.8287	0.8966	0.0528
DAXX	0.7089	1.1041	0.9909	0.7828	0.8967	0.0913
PPP1R12B	0.9867	0.9845	0.9159	0.6998	0.8967	0.0677
SNW1	0.8217	1.1313	1.0042	0.6298	0.8967	0.1093
ABCG1	0.8106	1.0437	0.9311	0.8016	0.8968	0.0572
LOC439949	0.7581	0.9924	1.0196	0.8169	0.8968	0.0644
PPARG	0.8543	1.0399	0.8552	0.8377	0.8968	0.0479
TOE1	0.7130	0.9442	1.0093	0.9207	0.8968	0.0641
TMEM120B	0.7553	1.0300	0.9556	0.8463	0.8968	0.0604
LOC643761	0.8333	0.9141	0.9828	0.8571	0.8968	0.0333
ZNF773	0.6646	1.1000	1.0561	0.7667	0.8968	0.1070
HARBI1	0.8831	1.0336	0.9242	0.7466	0.8969	0.0593
CRYZL1	0.7581	1.0806	1.0043	0.7445	0.8969	0.0855
CDC42BPG	0.8794	1.0218	0.8970	0.7893	0.8969	0.0479
LOC728927	0.8698	0.9306	0.8990	0.8881	0.8969	0.0127
LOC1001320	0.6200	0.9798	1.1291	0.8586	0.8969	0.1076
ZNF286C	0.8008	1.0699	0.9172	0.7997	0.8969	0.0639
ATPGD1	0.9422	0.9655	0.8842	0.7957	0.8969	0.0378
C1R	0.7913	1.0526	0.9096	0.8343	0.8970	0.0574
SYNGR1	0.8549	1.0110	0.8781	0.8438	0.8970	0.0387
FAM195A	0.8541	1.1779	0.8141	0.7419	0.8970	0.0965
NPAL3	0.7159	1.0595	1.0247	0.7880	0.8970	0.0853
CCDC16	0.7683	1.1749	0.9135	0.7315	0.8970	0.1006
ZNF813	0.7395	1.1224	0.9251	0.8014	0.8971	0.0844
ODZ4	0.8652	1.0236	0.7811	0.9185	0.8971	0.0508
NRIP3	0.7994	1.0562	0.9250	0.8080	0.8971	0.0603
LOC1001302	0.9428	1.0004	0.8494	0.7961	0.8972	0.0459
GUCY1A3	0.7648	1.1867	0.8797	0.7574	0.8972	0.1005
LOC1001345	0.8314	0.9794	0.8612	0.9166	0.8972	0.0326
STX8	0.6839	1.1412	1.0003	0.7633	0.8972	0.1055
LOC1001314	0.8685	1.0333	0.8366	0.8502	0.8972	0.0459
LOC642732	0.8165	1.0552	0.8632	0.8538	0.8972	0.0536
ZNF101	0.7714	1.0085	0.9364	0.8724	0.8972	0.0503
SESTD1	0.7087	1.1193	0.9783	0.7825	0.8972	0.0934
PPM1M	0.8797	1.0369	0.9038	0.7685	0.8972	0.0551
MARVELD2	0.8336	1.0102	1.0173	0.7279	0.8972	0.0706
SURF2	0.8908	1.0822	0.8059	0.8102	0.8973	0.0647
EPS15L1	0.7980	1.0317	0.9656	0.7938	0.8973	0.0601
RG9MTD3	0.8488	1.0902	0.8132	0.8371	0.8973	0.0647
C11orf68	0.8329	1.1598	0.8941	0.7025	0.8973	0.0962
WDR65	0.9098	0.9750	0.8663	0.8382	0.8973	0.0298
IFT88	0.8382	0.9870	0.9721	0.7923	0.8974	0.0484
CLCN5	0.8656	1.0364	0.9406	0.7471	0.8974	0.0611
LOC642302	0.9289	0.8935	0.8962	0.8711	0.8974	0.0119
KRBA2	0.8216	1.0971	0.8690	0.8022	0.8975	0.0680
CABLES2	0.9398	1.0199	0.8062	0.8239	0.8975	0.0504

ELF4	0.8215	1.0367	0.9040	0.8277	0.8975	0.0501
GGTLC1	0.8208	1.0388	0.9552	0.7754	0.8976	0.0606
NAB1	0.8638	0.8569	0.9611	0.9088	0.8976	0.0241
LDOC1L	1.0199	1.0562	0.8655	0.6490	0.8976	0.0926
KIAA0133	0.8071	1.1109	0.8680	0.8046	0.8977	0.0726
TMOD3	0.7152	1.2122	0.8567	0.8065	0.8977	0.1089
LOC645875	0.6990	1.0894	0.9134	0.8889	0.8977	0.0799
LOC390298	0.8489	1.1422	0.8833	0.7162	0.8977	0.0891
NXPH4	0.8526	1.0297	0.9240	0.7846	0.8977	0.0524
TNPO3	0.7568	1.3296	0.7528	0.7515	0.8977	0.1440
MIR1912	0.8760	0.9086	0.9797	0.8265	0.8977	0.0321
LOC1001305	0.8761	0.9612	0.9028	0.8508	0.8977	0.0237
BRD8	0.9130	1.1929	0.7722	0.7128	0.8977	0.1070
OSBPL9	0.8230	1.1773	0.8109	0.7797	0.8977	0.0936
FAM102A	0.7865	1.1414	0.9109	0.7521	0.8977	0.0881
ONECUT2	0.8434	1.0638	0.8361	0.8476	0.8977	0.0554
GRIN1	0.8475	0.9585	0.9022	0.8827	0.8977	0.0232
PIGV	0.6164	0.8602	1.3582	0.7562	0.8977	0.1614
LOC1001325	0.9038	1.0621	0.8622	0.7629	0.8977	0.0623
C19orf52	0.7117	1.2009	0.9736	0.7048	0.8978	0.1189
OVCA2	0.7636	1.2592	0.8792	0.6890	0.8978	0.1267
GLT8D1	0.7466	1.0732	0.9438	0.8275	0.8978	0.0711
LMF1	0.8014	1.0577	0.9149	0.8171	0.8978	0.0589
KLHL1	0.8334	0.9028	0.9546	0.9005	0.8978	0.0248
ULBP2	0.9811	1.0566	0.7848	0.7690	0.8979	0.0716
CXorf39	0.7985	1.0061	1.0566	0.7304	0.8979	0.0789
MGC88374	0.9109	0.9257	0.8877	0.8674	0.8979	0.0128
ELOVL7	0.8221	1.0073	0.9442	0.8181	0.8979	0.0467
LOC1001288	0.8487	0.9897	0.8759	0.8775	0.8979	0.0313
MGC35440	0.7866	0.9824	0.9591	0.8637	0.8980	0.0451
IRX2	0.8818	1.1796	0.7690	0.7615	0.8980	0.0978
VAMP7	0.7936	1.2643	0.9127	0.6212	0.8980	0.1360
SFMBT1	0.7794	1.0895	0.8895	0.8336	0.8980	0.0677
ARR3	0.9908	1.0074	0.7254	0.8684	0.8980	0.0654
LOC1001339	0.8690	0.9516	0.9685	0.8029	0.8980	0.0385
C9orf80	0.8281	1.1922	0.8067	0.7653	0.8981	0.0989
FLJ27255	0.8361	1.0437	0.8669	0.8457	0.8981	0.0490
THAP3	0.8323	1.0143	0.9379	0.8080	0.8981	0.0479
NOL9	0.7914	1.0520	0.9282	0.8211	0.8982	0.0591
ZFP37	0.7904	1.1593	0.7305	0.9124	0.8982	0.0949
LOC113230	0.7708	0.9862	0.9740	0.8617	0.8982	0.0509
MIR658	0.8061	1.0966	0.9095	0.7807	0.8982	0.0718
DHX34	0.8046	1.1174	0.8787	0.7922	0.8982	0.0755
LOC389958	0.9026	1.0400	0.8319	0.8185	0.8982	0.0507
LOC200030	0.7402	1.3661	0.7447	0.7422	0.8983	0.1559
DCAF7	0.8698	1.1466	0.8677	0.7092	0.8983	0.0909

LOC441087	0.6037	1.1676	1.1933	0.6286	0.8983	0.1631
PRNP	0.6806	1.2195	1.0187	0.6745	0.8983	0.1339
STARD3	0.8688	1.0723	0.8424	0.8100	0.8984	0.0592
LOC653803	0.7480	1.2029	0.8790	0.7635	0.8984	0.1056
LOC1001317(	0.8259	1.0014	0.9504	0.8158	0.8984	0.0460
DIRC2	0.7469	1.2472	0.8508	0.7487	0.8984	0.1188
SNORD30	0.8429	1.0258	0.8875	0.8375	0.8984	0.0439
PSMF1	0.6870	1.1566	0.9394	0.8108	0.8984	0.1003
JAGN1	0.7879	1.0830	0.8898	0.8332	0.8985	0.0649
KRBA1	0.8412	1.1782	0.8017	0.7729	0.8985	0.0943
DDX27	0.7668	1.2039	0.9378	0.6855	0.8985	0.1146
LOC652815	0.9274	1.0596	0.8645	0.7425	0.8985	0.0660
LOC728606	0.8244	1.0342	0.8965	0.8390	0.8985	0.0478
HK1	0.8405	1.2913	0.7933	0.6689	0.8985	0.1358
FES	0.7552	0.9723	0.9803	0.8865	0.8986	0.0523
HOOK3	0.8224	1.0355	0.9037	0.8328	0.8986	0.0491
LOC85390	0.7793	1.1937	0.7977	0.8237	0.8986	0.0988
DKFZp434N0	0.8382	1.0248	0.9443	0.7872	0.8986	0.0533
LOC644162	0.7151	1.1635	0.9734	0.7424	0.8986	0.1056
RNF123	0.8251	1.1228	0.9528	0.6939	0.8986	0.0915
C18orf34	0.8857	0.9788	0.8627	0.8675	0.8987	0.0271
GLUL	0.8191	1.0470	0.9758	0.7529	0.8987	0.0680
CTU2	0.8222	1.0860	0.8528	0.8338	0.8987	0.0628
LOC649445	0.8050	0.9628	0.9773	0.8499	0.8987	0.0423
GALE	0.8951	1.1087	0.8159	0.7753	0.8987	0.0743
TXNL4B	0.8379	1.0067	0.9209	0.8295	0.8987	0.0415
FYTTD1	0.8338	1.0436	0.9997	0.7179	0.8988	0.0753
GPM6B	0.8016	0.9917	0.9658	0.8360	0.8988	0.0470
WFDC13	0.6608	1.0235	1.0803	0.8306	0.8988	0.0956
LOC642587	0.8153	0.9318	0.9957	0.8525	0.8988	0.0404
LOC648638	0.7531	1.1952	0.9456	0.7015	0.8989	0.1119
SNORA55	0.8235	0.9144	0.9063	0.9513	0.8989	0.0270
LOC643802	0.8207	0.9557	0.8930	0.9262	0.8989	0.0290
LOC646730	0.9228	0.9820	0.8684	0.8224	0.8989	0.0345
FAM21C	0.8390	1.0242	0.9047	0.8279	0.8989	0.0451
VISA	0.7790	1.2004	0.8273	0.7892	0.8990	0.1010
SHROOM1	0.9133	1.0189	0.9053	0.7582	0.8990	0.0536
LOC1001313	0.7362	1.0744	0.9814	0.8042	0.8990	0.0780
ISM1	0.8625	1.0655	0.9322	0.7359	0.8990	0.0688
LOC647970	0.7771	1.0884	0.9633	0.7673	0.8990	0.0776
COX6B2	0.8844	0.9578	0.9294	0.8247	0.8991	0.0290
RCCD1	0.8598	1.0136	0.8838	0.8391	0.8991	0.0393
LOC643461	0.6818	1.0183	1.0322	0.8642	0.8991	0.0818
NFKB1	0.7955	1.2726	0.8623	0.6661	0.8991	0.1310
LOC1001305	0.8979	1.1709	0.7907	0.7369	0.8991	0.0966
TRDMT1	0.8363	1.0302	0.9041	0.8259	0.8991	0.0470

LOC152217	0.9258	0.9288	0.9049	0.8371	0.8991	0.0214
RNF168	0.7201	0.9645	1.0008	0.9111	0.8991	0.0624
ASAH2B	0.8015	1.0529	0.8963	0.8458	0.8991	0.0548
FUBP3	0.7230	1.0823	0.9643	0.8271	0.8992	0.0785
CTSK	0.8348	1.0702	0.8737	0.8180	0.8992	0.0582
NONO	0.6806	1.3393	0.8910	0.6859	0.8992	0.1547
SEMA3G	0.9067	0.9077	0.9120	0.8704	0.8992	0.0097
LOC644934	0.9342	0.9134	1.0202	0.7290	0.8992	0.0613
TNFRSF1A	0.7788	1.3021	0.7622	0.7536	0.8992	0.1344
TJAP1	0.8210	1.1467	0.8439	0.7852	0.8992	0.0834
ZNF577	0.7456	1.0119	1.0463	0.7930	0.8992	0.0759
SLC31A2	0.8065	1.1763	0.9598	0.6545	0.8993	0.1114
CTR9	0.8740	0.9779	0.9433	0.8019	0.8993	0.0390
TAF9	0.7915	1.0335	1.0084	0.7637	0.8993	0.0707
MYO6	0.6530	1.2836	0.9170	0.7438	0.8993	0.1393
CDH9	0.8994	0.9227	0.9762	0.7994	0.8994	0.0370
PHF23	0.8036	1.0110	1.1525	0.6306	0.8994	0.1147
LOC730877	0.7438	0.9390	0.9845	0.9304	0.8994	0.0532
LOC647331	0.8832	0.8805	0.9297	0.9043	0.8994	0.0114
LOC728811	0.8053	1.0164	0.9344	0.8420	0.8995	0.0475
LOC1001302	0.8040	0.9965	1.0002	0.7974	0.8995	0.0571
ZDHHC19	0.8169	1.0104	0.9064	0.8644	0.8995	0.0412
PMS2L2	0.8394	1.0292	0.8850	0.8446	0.8996	0.0444
LMO4	0.7589	1.1680	1.0149	0.6563	0.8996	0.1170
SLC35A5	0.8290	1.0518	1.0811	0.6363	0.8996	0.1043
MAP2K3	0.7704	1.1577	0.8905	0.7799	0.8996	0.0902
CLCN2	0.7791	1.0384	0.9575	0.8233	0.8996	0.0598
LOC388780	0.8975	0.9141	0.8818	0.9050	0.8996	0.0068
FBXO6	0.8753	1.0576	0.9209	0.7448	0.8996	0.0645
DNMT3B	0.8235	1.0366	0.9335	0.8049	0.8996	0.0538
LOC728014	0.7792	1.2305	0.8150	0.7741	0.8997	0.1106
C14orf156	0.8128	1.1535	0.9065	0.7261	0.8997	0.0923
LOC649783	0.8093	1.0167	0.8947	0.8782	0.8997	0.0432
LOC642666	0.8708	0.9697	0.9280	0.8304	0.8997	0.0307
GULP1	0.6811	1.2960	0.9804	0.6417	0.8998	0.1522
SPAG17	0.8125	0.9896	0.8769	0.9203	0.8998	0.0372
DNAJC17	0.8444	1.1849	0.8305	0.7398	0.8999	0.0978
DYRK2	0.7600	1.0145	1.0089	0.8162	0.8999	0.0656
DECR2	0.6604	1.3522	0.9234	0.6636	0.8999	0.1629
LOC390349	0.8394	0.9924	0.9541	0.8138	0.8999	0.0434
LOC643894	0.8419	1.0129	0.9016	0.8433	0.8999	0.0401
RNU105C	0.8453	0.8854	0.9071	0.9619	0.8999	0.0243
LOC645435	0.8959	1.0124	0.8095	0.8821	0.9000	0.0420
PNPT1	0.7078	1.2163	0.9348	0.7413	0.9000	0.1167
LOC1001282	0.7867	1.0247	0.9862	0.8026	0.9000	0.0614
LOC644745	0.6325	1.3078	0.9096	0.7505	0.9001	0.1473

PGBD4	0.8113	1.0231	0.9586	0.8075	0.9001	0.0540
LOC1001309	0.7756	1.0513	0.9309	0.8427	0.9001	0.0596
FLJ20699	0.8385	1.2806	0.7984	0.6831	0.9001	0.1310
LOC286208	0.9290	1.0689	0.9036	0.6993	0.9002	0.0762
RASAL3	0.6955	1.1577	0.8933	0.8543	0.9002	0.0959
LOC1001339	0.7443	1.1463	1.0005	0.7098	0.9002	0.1046
LOC642672	0.8432	0.9855	0.9262	0.8461	0.9002	0.0343
GIT2	0.9142	0.9912	0.9882	0.7073	0.9002	0.0667
ARL17B	0.8755	1.0897	0.8600	0.7757	0.9002	0.0669
ULK3	0.8362	1.1004	0.8002	0.8644	0.9003	0.0680
CHCHD2	0.8308	0.9852	0.9777	0.8076	0.9003	0.0471
RALGAPA1	0.9089	1.0772	0.8783	0.7369	0.9003	0.0699
LOC644638	0.9297	0.9624	1.0017	0.7076	0.9004	0.0659
LOC441261	0.8113	0.9559	1.0308	0.8035	0.9004	0.0558
LOC650111	0.7815	1.0780	0.8073	0.9351	0.9005	0.0680
GPR17	0.8796	1.0072	0.8896	0.8257	0.9005	0.0382
FAM60A	0.7759	1.1681	0.9592	0.6989	0.9005	0.1046
MRPS33	0.7761	1.0651	1.0254	0.7354	0.9005	0.0844
MGAT1	0.8185	1.1731	0.8944	0.7162	0.9006	0.0979
LOC644195	0.8820	1.0133	0.8203	0.8868	0.9006	0.0405
NUP133	0.7610	1.3397	0.8159	0.6857	0.9006	0.1488
CHD2	0.8287	0.9881	0.9728	0.8129	0.9006	0.0463
MEST	0.7782	1.0469	0.9800	0.7976	0.9007	0.0667
FLJ35390	0.8473	1.0675	1.0011	0.6868	0.9007	0.0849
ANKRD53	0.8334	0.9985	0.9718	0.7991	0.9007	0.0496
LOC651774	0.9015	0.9038	0.9642	0.8334	0.9007	0.0267
MIR618	0.9098	1.0895	0.7586	0.8449	0.9007	0.0701
ERF	0.9706	1.0277	0.8456	0.7591	0.9007	0.0606
FCN1	0.7809	1.0191	0.9040	0.8990	0.9007	0.0486
TMEM34	0.7165	1.1691	0.9705	0.7469	0.9008	0.1059
LOC654202	0.8283	0.8663	1.0032	0.9052	0.9008	0.0376
ZNF584	0.7903	1.1863	0.8687	0.7580	0.9008	0.0980
SYNGR4	0.8528	0.9767	0.8948	0.8789	0.9008	0.0267
H2AFY	0.7164	1.1992	0.9966	0.6911	0.9008	0.1212
LOC646858	0.8688	0.9880	0.9439	0.8029	0.9009	0.0409
LOC729264	0.8405	1.1265	0.9544	0.6823	0.9009	0.0936
LOC390874	0.8306	0.9609	0.9257	0.8866	0.9009	0.0279
C2orf30	1.1098	1.0624	0.7552	0.6763	0.9009	0.1086
HEXIM1	0.8204	1.0931	0.8961	0.7943	0.9010	0.0676
GAGE4	0.8075	1.1309	0.9379	0.7277	0.9010	0.0880
C1orf212	0.8579	0.9967	0.9198	0.8298	0.9010	0.0370
HEPACAM	0.8435	1.0978	0.8998	0.7631	0.9011	0.0713
PLEKHF1	0.7502	1.1698	0.7993	0.8851	0.9011	0.0938
RRN3	0.7734	1.0439	1.0552	0.7318	0.9011	0.0862
LOC643423	0.9021	0.9095	0.9649	0.8279	0.9011	0.0281
LOC646527	0.7690	0.9535	1.2350	0.6468	0.9011	0.1279

ARIH1	0.8210	1.2013	0.9646	0.6176	0.9011	0.1228
LOC642607	0.7241	0.9596	1.0532	0.8678	0.9012	0.0701
RNPS1	0.8262	1.1700	0.8892	0.7193	0.9012	0.0962
C10orf76	0.8988	1.1120	0.8753	0.7187	0.9012	0.0808
LOC643905	0.8075	1.1585	0.8809	0.7581	0.9012	0.0894
MTL5	0.8332	1.0149	0.9513	0.8054	0.9012	0.0494
RNPC2	0.8200	1.0391	0.8862	0.8596	0.9012	0.0479
PPAPDC1A	0.8236	0.9797	0.9822	0.8195	0.9012	0.0460
NSUN3	0.6811	1.2886	0.9337	0.7018	0.9013	0.1412
FAM90A19	0.8838	0.9296	0.8917	0.9002	0.9013	0.0100
C12orf41	0.8138	1.1393	0.9383	0.7139	0.9013	0.0917
LOC441493	0.8881	0.9079	0.9202	0.8893	0.9014	0.0077
RNF214	0.8669	1.0278	0.8774	0.8335	0.9014	0.0432
HIST3H3	0.9411	0.9604	0.9344	0.7698	0.9014	0.0442
LOC1001287	0.7045	1.2585	1.0521	0.5904	0.9014	0.1543
LOC642661	0.8166	0.9997	0.9842	0.8053	0.9014	0.0524
LOC441131	0.7734	1.1713	0.9593	0.7019	0.9015	0.1050
C7orf51	0.7810	1.0776	0.8972	0.8502	0.9015	0.0634
TNFSF9	0.8308	1.0761	0.8560	0.8433	0.9015	0.0584
CDKN2AIP	0.6590	1.1597	1.0156	0.7718	0.9015	0.1138
ATG12	0.7491	1.2511	0.9413	0.6647	0.9016	0.1301
TMCO7	0.8182	1.0285	0.9607	0.7988	0.9016	0.0556
PPFIA3	0.9097	0.9914	0.8625	0.8426	0.9016	0.0331
RABL3	0.6311	1.2884	0.9580	0.7288	0.9016	0.1460
CLRN1	0.7687	1.1317	0.9306	0.7753	0.9016	0.0853
LOC1001345	0.8799	0.9680	0.9395	0.8190	0.9016	0.0331
EXOG	0.7436	1.0799	0.9505	0.8325	0.9016	0.0730
PUM2	0.8373	1.2156	0.7966	0.7570	0.9016	0.1059
RAD51L1	0.8361	0.9646	0.9711	0.8348	0.9017	0.0382
SNORA68	0.7171	1.0822	1.0330	0.7743	0.9017	0.0913
LOC402096	0.8463	0.9786	0.9360	0.8458	0.9017	0.0333
C6orf168	0.8172	1.0645	0.9197	0.8054	0.9017	0.0600
IHH	0.8373	1.0048	0.9756	0.7892	0.9017	0.0523
LOC643262	0.9383	0.9638	0.8973	0.8075	0.9017	0.0343
FOXJ2	0.7830	1.2399	0.8753	0.7088	0.9018	0.1177
C14orf138	0.8045	1.0804	0.9174	0.8048	0.9018	0.0652
WDR89	0.8136	1.0208	0.9360	0.8367	0.9018	0.0478
COX10	0.6562	1.2057	0.9696	0.7757	0.9018	0.1201
ATG4D	0.8210	1.0598	0.9443	0.7821	0.9018	0.0630
PMS2L5	0.7465	1.2327	0.9395	0.6886	0.9018	0.1226
C1orf216	0.8142	1.1834	0.8735	0.7363	0.9018	0.0980
LOC731227	0.9122	0.9207	0.9721	0.8023	0.9018	0.0357
MTBP	0.8761	1.0305	0.9016	0.7993	0.9019	0.0481
LOC128102	0.7817	1.0231	0.9685	0.8342	0.9019	0.0564
TMEM214	0.8453	1.1989	0.8322	0.7313	0.9019	0.1022
POLR2K	0.9378	1.0430	0.8137	0.8132	0.9019	0.0554

LOC652067	0.6641	1.0800	0.9267	0.9369	0.9019	0.0866
ZNF256	0.7737	1.0968	0.9874	0.7499	0.9020	0.0841
LOC641700	0.8537	0.9533	1.0154	0.7854	0.9020	0.0512
LOC169834	0.8150	1.0351	1.0342	0.7236	0.9020	0.0789
ERLIN2	0.8785	1.0148	0.9601	0.7545	0.9020	0.0566
LAMA1	0.7801	1.0248	0.9589	0.8441	0.9020	0.0552
LOC1001284	0.8602	1.0276	0.9523	0.7679	0.9020	0.0563
P2RX6P	0.7848	1.0742	0.9162	0.8328	0.9020	0.0635
LOC729234	0.9040	1.0393	0.8497	0.8151	0.9020	0.0493
EGFL7	0.8532	1.0036	0.8885	0.8630	0.9021	0.0346
LOC730340	0.8454	1.0987	0.8934	0.7708	0.9021	0.0702
CLDN23	0.6984	1.3593	0.8176	0.7331	0.9021	0.1544
RTKN2	0.7095	1.0397	1.0466	0.8125	0.9021	0.0841
ZNF280C	0.7968	1.1857	0.8299	0.7960	0.9021	0.0949
LZTS2	0.9506	0.9413	0.9420	0.7746	0.9021	0.0425
BOK	0.8109	1.0725	0.9971	0.7284	0.9022	0.0799
ALG1L	0.8149	1.0241	0.8991	0.8707	0.9022	0.0442
MGST2	0.7695	1.1545	0.9112	0.7738	0.9022	0.0903
FLNA	0.6350	1.0714	1.0361	0.8664	0.9022	0.0997
LOC402145	0.8030	0.9408	0.9926	0.8727	0.9022	0.0412
ZC3HC1	0.8091	1.1390	1.0025	0.6583	0.9023	0.1058
NFKB2	0.8107	1.0318	0.9365	0.8300	0.9023	0.0513
RILP	0.8273	1.1489	0.8108	0.8221	0.9023	0.0823
ZNF454	0.7933	1.1003	0.9840	0.7315	0.9023	0.0851
UCK1	0.9635	1.1311	0.7818	0.7331	0.9024	0.0909
DPH3	0.7668	1.1498	0.9425	0.7504	0.9024	0.0932
LOC144776	0.9133	0.8905	0.9181	0.8876	0.9024	0.0078
LOC642165	0.7597	1.0541	0.9867	0.8091	0.9024	0.0702
LOC1001310	0.8707	1.0620	0.9781	0.6990	0.9024	0.0783
LOC1001279	0.8669	1.0724	0.9216	0.7488	0.9024	0.0672
LOC647037	0.8107	1.0496	1.0709	0.6787	0.9025	0.0951
MXRA5	0.9097	1.0057	0.7603	0.9343	0.9025	0.0516
TJP1	0.7965	1.2440	0.8791	0.6904	0.9025	0.1202
LOC1001288	0.8581	0.9781	0.8970	0.8768	0.9025	0.0264
LOC1001296	0.8593	0.8873	1.0188	0.8447	0.9025	0.0398
NDUFB1	0.6796	1.0218	0.9396	0.9691	0.9025	0.0762
CAPZA1	0.7757	1.0014	0.9773	0.8557	0.9025	0.0530
LOC392489	0.8333	1.1069	0.8532	0.8167	0.9025	0.0685
TPRX1	0.9346	0.9157	0.9051	0.8547	0.9025	0.0171
DEFB112	0.8362	0.8977	1.0103	0.8660	0.9026	0.0380
RNU86	0.7866	1.0932	0.8458	0.8847	0.9026	0.0667
BEX1	0.7201	1.1799	1.0401	0.6704	0.9026	0.1235
LOC1001308	0.8380	0.9667	0.9787	0.8271	0.9026	0.0406
LOC648174	0.8404	0.9122	0.9809	0.8770	0.9026	0.0299
DNAJC5	0.7904	1.1847	0.9004	0.7351	0.9026	0.1001
KRTAP12-2	0.8653	0.9213	0.9835	0.8405	0.9027	0.0318

ZNF799	0.8262	0.8629	1.1095	0.8121	0.9027	0.0698
MFSD8	0.7090	1.0724	1.0588	0.7705	0.9027	0.0950
ZNF519	0.8260	0.9491	0.9332	0.9025	0.9027	0.0273
XRCC1	0.8077	1.1586	0.9924	0.6522	0.9027	0.1100
GABPB1	0.8259	1.0207	0.9978	0.7666	0.9027	0.0628
PTPRZ1	0.9023	0.9402	0.9358	0.8327	0.9028	0.0248
SEC16A	0.7616	1.3773	0.7704	0.7017	0.9028	0.1589
PDE6B	0.7903	1.0271	0.9126	0.8811	0.9028	0.0489
DNAJC16	0.6564	1.0890	0.9678	0.8980	0.9028	0.0911
FOXI2	0.8840	0.9219	0.8932	0.9121	0.9028	0.0086
MAP2K2	0.8427	1.2811	0.7925	0.6949	0.9028	0.1298
ZNF829	0.8859	0.9495	0.9546	0.8213	0.9028	0.0314
FLJ12688	0.8128	1.0625	0.9279	0.8080	0.9028	0.0600
FLVCR1	0.7311	1.0462	1.0973	0.7369	0.9028	0.0981
ZNF860	0.8268	0.8176	0.9786	0.9884	0.9029	0.0466
LOC648603	0.8349	1.0595	0.8493	0.8678	0.9029	0.0526
PHLPP2	0.5920	1.1612	1.1160	0.7424	0.9029	0.1398
GABRB3	0.7867	1.0937	0.9204	0.8109	0.9029	0.0699
HDAC3	0.8084	1.3131	0.8153	0.6749	0.9029	0.1405
NPIP	0.9387	0.9598	0.8419	0.8714	0.9029	0.0277
ABHD3	0.8262	1.1106	0.9598	0.7152	0.9030	0.0854
KRR1	0.6745	1.1189	0.9907	0.8277	0.9030	0.0967
LOC1001330	0.9586	0.9295	0.8458	0.8781	0.9030	0.0253
UGT2B28	0.7693	1.1649	1.0568	0.6211	0.9030	0.1257
PTPRE	0.8807	1.0363	0.9124	0.7826	0.9030	0.0523
LOC653265	0.8728	0.9855	0.9313	0.8224	0.9030	0.0354
GLT1D1	0.8423	1.0377	0.9093	0.8228	0.9030	0.0486
LOC732432	0.5974	1.3881	0.9160	0.7105	0.9030	0.1746
LOC729362	0.5958	1.0985	1.2111	0.7068	0.9030	0.1489
ZNF550	0.8397	1.0128	0.9616	0.7981	0.9030	0.0504
EIF3I	0.7433	1.2328	0.9117	0.7244	0.9031	0.1177
SNORA84	0.6890	1.1796	0.8909	0.8528	0.9031	0.1020
FAM183A	0.8499	0.8944	1.0283	0.8399	0.9031	0.0434
KLHL12	0.6862	1.3044	0.9339	0.6881	0.9032	0.1459
C12orf76	0.8416	1.2797	0.8117	0.6798	0.9032	0.1303
MAST2	0.8152	0.9368	0.9764	0.8844	0.9032	0.0349
NMRAL1	0.7610	1.3336	0.9102	0.6082	0.9032	0.1561
C7orf60	0.9499	0.9546	0.9261	0.7823	0.9032	0.0408
GAGE12G	0.8320	1.0951	0.9250	0.7609	0.9032	0.0722
CSTF1	0.7311	1.1747	0.9324	0.7749	0.9033	0.1003
POLR3F	0.9608	0.9934	1.0099	0.6490	0.9033	0.0854
WASH3P	0.8350	0.9542	0.9375	0.8865	0.9033	0.0269
DPF2	0.7883	1.3093	0.8392	0.6765	0.9033	0.1395
SGPP2	0.8053	1.0171	1.0067	0.7842	0.9033	0.0629
MIR34B	0.9263	0.8447	0.9792	0.8631	0.9033	0.0307
LOC642765	0.7799	1.0660	0.8837	0.8836	0.9033	0.0595

CHM	0.7917	1.0809	1.0146	0.7262	0.9034	0.0855
LOC648196	0.9576	0.9754	0.9039	0.7765	0.9034	0.0449
RBM6	0.9207	1.0935	0.8150	0.7842	0.9034	0.0698
PIK3R2	0.8303	1.1621	0.9433	0.6779	0.9034	0.1019
FLJ38576	0.7666	1.1052	0.9483	0.7935	0.9034	0.0783
DCTN3	0.9033	1.1174	0.9765	0.6164	0.9034	0.1055
KIAA0251	0.7682	1.1902	0.8626	0.7926	0.9034	0.0977
LOC653429	0.7584	0.9609	1.0315	0.8631	0.9035	0.0594
HIST1H1B	0.8066	0.9371	0.9831	0.8871	0.9035	0.0378
SLC7A6OS	0.7147	1.1528	0.7988	0.9477	0.9035	0.0960
BSPRY	0.7770	1.1802	0.8982	0.7586	0.9035	0.0973
LOC643452	0.8500	0.9630	1.0370	0.7641	0.9035	0.0603
RDH14	0.7567	1.2875	0.9111	0.6588	0.9035	0.1381
C17orf75	0.8641	1.0442	0.8952	0.8107	0.9035	0.0500
LOC442501	0.8342	0.9775	0.9619	0.8406	0.9035	0.0383
KRTAP11-1	0.8401	0.9598	0.9260	0.8883	0.9036	0.0257
SEPN1	0.7792	1.1437	0.9417	0.7498	0.9036	0.0905
LOC652726	0.7494	1.0838	1.0258	0.7554	0.9036	0.0881
LOC643980	0.8816	0.9827	0.9956	0.7545	0.9036	0.0558
LOC651621	0.8032	1.2420	0.8753	0.6939	0.9036	0.1188
DNAJB14	0.8431	1.0630	1.0147	0.6936	0.9036	0.0844
IL10	0.6682	1.2411	0.9378	0.7674	0.9036	0.1255
LOC728969	0.8411	1.0929	0.8736	0.8070	0.9037	0.0645
SSNA1	1.0177	1.0925	0.7799	0.7245	0.9037	0.0895
LOC650028	0.8872	1.1386	0.7837	0.8052	0.9037	0.0814
C2orf63	0.8933	1.0968	0.8552	0.7694	0.9037	0.0694
LOC652417	0.8581	1.0619	0.8065	0.8882	0.9037	0.0554
MESTIT1	0.8623	1.0214	0.7828	0.9483	0.9037	0.0518
SRRM2	0.7757	1.1703	0.9215	0.7473	0.9037	0.0967
NCOA6IP	0.8235	1.0371	0.9188	0.8356	0.9037	0.0492
METTL4	0.8510	1.0893	0.8366	0.8381	0.9037	0.0619
TRMT12	0.7997	1.1326	0.9836	0.6992	0.9038	0.0964
LOC1001302	0.8659	1.0411	0.8993	0.8087	0.9038	0.0495
HSPA12A	0.8323	1.0142	0.9295	0.8391	0.9038	0.0430
LOC729764	0.6964	1.2689	0.9114	0.7388	0.9039	0.1303
SCARF2	0.8903	0.9978	0.9592	0.7683	0.9039	0.0504
C10orf58	0.7604	1.0604	1.0438	0.7509	0.9039	0.0857
IPO11	0.7200	1.3026	0.8534	0.7396	0.9039	0.1361
CRIPAK	0.8406	1.2120	0.7701	0.7929	0.9039	0.1037
GOT1	0.7204	1.0912	1.0240	0.7801	0.9039	0.0906
MIR188	0.8799	0.8043	1.0671	0.8643	0.9039	0.0568
AMFR	0.7536	1.1669	1.0034	0.6920	0.9040	0.1105
AGPAT3	0.8712	1.0507	0.8906	0.8035	0.9040	0.0523
LOC1001313	0.8940	1.0229	0.8326	0.8667	0.9040	0.0416
SF3A1	0.7709	1.0767	0.9923	0.7763	0.9040	0.0773
RNFT2	0.8004	1.1485	0.8594	0.8079	0.9040	0.0825

GAFA2	0.8400	0.9264	0.9602	0.8898	0.9041	0.0258
LOC729655	0.8937	1.0757	0.8905	0.7566	0.9041	0.0655
CD2	0.9187	0.8856	0.9510	0.8613	0.9041	0.0196
RAG1	0.7921	1.1372	0.8942	0.7932	0.9042	0.0813
UPRT	0.8174	1.1464	0.9316	0.7212	0.9042	0.0915
LOC642662	0.9939	0.8832	0.9356	0.8040	0.9042	0.0403
GIN1	0.8442	1.0738	0.9675	0.7313	0.9042	0.0743
LOC651576	0.8048	1.0587	0.9706	0.7825	0.9042	0.0664
MAPK1	0.7769	1.1571	0.9226	0.7601	0.9042	0.0919
PPFIBP1	0.7490	1.0639	0.9906	0.8133	0.9042	0.0738
SNORD42A	0.8053	0.9845	1.0003	0.8267	0.9042	0.0512
PADI2	0.8578	1.0783	0.9478	0.7330	0.9042	0.0728
LOC1001292	0.8328	1.1330	0.8954	0.7557	0.9042	0.0814
TUBGCP6	0.8668	1.0040	0.8903	0.8559	0.9042	0.0340
MAN2A2	0.9269	1.0021	0.8744	0.8135	0.9042	0.0400
C17orf88	0.8759	1.0051	0.9950	0.7411	0.9043	0.0618
LOC91431	0.8725	0.9708	0.8873	0.8864	0.9043	0.0224
PIGX	0.8286	1.1146	0.8523	0.8218	0.9043	0.0704
BCL10	0.7249	1.0839	1.0229	0.7855	0.9043	0.0879
LOC1001327	0.8956	1.0011	0.9203	0.8002	0.9043	0.0414
PTP4A1	0.9108	0.9644	1.0827	0.6593	0.9043	0.0892
ABCB6	0.7258	1.2637	0.9988	0.6290	0.9043	0.1431
LOC391742	0.7439	1.0638	0.9837	0.8258	0.9043	0.0728
NCKAP1	0.7336	1.2465	0.8726	0.7645	0.9043	0.1179
MREG	0.7094	1.1028	0.8819	0.9232	0.9043	0.0807
LOC650681	0.8909	1.0737	0.9844	0.6685	0.9044	0.0870
CREBL1	0.8991	1.0592	0.8981	0.7611	0.9044	0.0609
LOC652139	0.7305	0.9793	1.0191	0.8886	0.9044	0.0641
LOC652389	0.8531	1.0160	0.9555	0.7931	0.9044	0.0501
LOC1001324	0.6777	0.9836	0.9125	1.0440	0.9044	0.0802
ZNF791	0.8301	0.9888	1.0462	0.7526	0.9044	0.0682
SNORD12B	0.9422	0.8360	0.8918	0.9478	0.9044	0.0261
KIAA0831	0.7325	1.1506	1.0041	0.7306	0.9044	0.1042
LOC648775	0.8940	1.0285	0.9323	0.7630	0.9045	0.0550
LOC283174	0.8555	1.0324	0.9423	0.7877	0.9045	0.0531
LOC1001343	0.8293	1.0348	0.9069	0.8471	0.9045	0.0465
RPAP1	0.9297	1.0754	0.8605	0.7526	0.9045	0.0676
PLEKHA7	0.8871	1.0463	0.9026	0.7822	0.9046	0.0543
C16orf88	0.9786	0.8892	0.9604	0.7901	0.9046	0.0427
SLC48A1	0.6984	1.1579	0.9119	0.8501	0.9046	0.0956
SHPRH	0.8885	1.0915	0.8967	0.7416	0.9046	0.0718
LOC728476	0.7418	1.2556	0.8525	0.7685	0.9046	0.1193
UBL4B	0.8438	1.0239	0.9217	0.8293	0.9046	0.0446
CCDC101	0.8284	1.1121	0.9028	0.7753	0.9047	0.0739
MTCH1	0.8134	1.2578	0.8790	0.6686	0.9047	0.1256
CHST13	0.9153	1.0498	0.8547	0.7990	0.9047	0.0539

APTX	0.7681	1.2045	1.0027	0.6437	0.9047	0.1246
LOC441205	0.9886	0.9306	0.8525	0.8472	0.9047	0.0338
SDHA	0.7335	1.4051	0.8535	0.6269	0.9047	0.1731
GCSH	0.6699	1.2898	0.9302	0.7291	0.9047	0.1399
LOC643854	0.8616	1.0221	0.8686	0.8668	0.9047	0.0391
LOC651407	0.8244	1.0008	0.9614	0.8324	0.9048	0.0448
NPHP4	0.8039	1.1778	0.8189	0.8184	0.9048	0.0911
SPIN2A	0.9650	1.0000	0.9507	0.7032	0.9048	0.0680
ATP6V1B1	0.7850	1.1379	0.8796	0.8166	0.9048	0.0802
NDNL2	0.9124	0.9424	0.9494	0.8150	0.9048	0.0310
FLJ40292	0.9366	0.9777	0.8886	0.8163	0.9048	0.0347
LOC728953	0.7692	1.0788	0.9361	0.8352	0.9048	0.0674
MAGEA2	0.8628	1.0525	0.9176	0.7865	0.9048	0.0561
LOC643888	0.8407	1.2707	0.9018	0.6063	0.9049	0.1376
EFHA1	0.7610	0.9410	1.2026	0.7148	0.9049	0.1106
LOC1001327	0.8458	1.1111	0.8450	0.8176	0.9049	0.0690
SYT3	0.8650	1.0747	0.8257	0.8542	0.9049	0.0572
RFESD	0.8658	1.0399	0.9359	0.7781	0.9049	0.0554
HMGB1	0.7961	0.9526	1.0543	0.8168	0.9049	0.0607
METTL9	0.8715	0.9369	0.9658	0.8457	0.9050	0.0279
LOC85391	0.8243	0.8862	1.0968	0.8125	0.9050	0.0660
NBPF14	0.7909	1.0761	0.8486	0.9043	0.9050	0.0616
LOC1001341	0.8446	0.9654	1.0013	0.8087	0.9050	0.0464
IAH1	0.6566	1.3307	0.9407	0.6919	0.9050	0.1554
RGS14	0.8269	1.0069	0.8927	0.8935	0.9050	0.0374
C14orf101	0.6861	1.1222	1.1561	0.6557	0.9050	0.1355
PTAFR	0.7084	1.1580	0.9258	0.8280	0.9050	0.0953
SNORD1A	0.7633	0.9533	1.0289	0.8748	0.9051	0.0568
TMEM150C	0.9048	0.9289	0.9427	0.8439	0.9051	0.0219
SNORD38A	0.7637	0.9816	1.0212	0.8538	0.9051	0.0591
DVL2	0.9377	1.1176	0.8253	0.7397	0.9051	0.0816
SNORA3	0.7271	1.1373	0.9695	0.7864	0.9051	0.0930
SNORD46	0.7760	0.9990	1.0142	0.8311	0.9051	0.0598
WHSC1	0.8332	0.9991	0.9496	0.8387	0.9051	0.0412
EPOR	0.8813	1.0549	0.9345	0.7499	0.9051	0.0632
SERPINA5	0.9211	1.0994	1.0077	0.5925	0.9052	0.1104
OR4N4	0.8240	0.8808	1.0110	0.9049	0.9052	0.0391
ZNF460	0.8558	1.0376	0.8530	0.8744	0.9052	0.0444
FSD1CL	0.8426	0.9574	0.9795	0.8413	0.9052	0.0368
TRIM21	0.8258	1.0414	1.0096	0.7440	0.9052	0.0717
LOC652610	0.7938	0.8910	0.9852	0.9508	0.9052	0.0419
C13orf15	0.7019	1.1667	1.0780	0.6744	0.9053	0.1268
SLC15A4	0.8326	1.1345	0.9168	0.7371	0.9053	0.0848
LOC644294	0.8767	0.9359	0.8872	0.9213	0.9053	0.0140
LOC642947	0.7544	1.1676	0.9711	0.7280	0.9053	0.1030
AGR2	0.8329	0.9336	1.0861	0.7686	0.9053	0.0692

TTF1	0.9296	0.9655	0.9532	0.7728	0.9053	0.0448
SCARB2	0.8934	1.2215	0.7882	0.7182	0.9053	0.1114
FAM14B	1.0316	0.9219	0.9515	0.7163	0.9053	0.0671
C22orf24	0.7686	0.9435	0.9637	0.9456	0.9053	0.0458
MIR16-1	0.9551	0.9252	0.8465	0.8946	0.9053	0.0232
IL4R	0.7684	0.9921	0.9691	0.8918	0.9054	0.0504
SART3	0.7909	1.1105	0.9829	0.7374	0.9054	0.0863
ZNF835	0.8898	0.9396	0.9718	0.8206	0.9054	0.0329
LOC553158	0.7646	1.1167	0.9587	0.7818	0.9054	0.0830
C14orf181	0.8033	1.1074	0.7980	0.9130	0.9054	0.0724
SNORD1B	0.8396	0.9734	0.9304	0.8784	0.9054	0.0293
ENTPD4	0.5918	1.4198	0.8130	0.7973	0.9055	0.1787
LOC652235	0.8662	0.9952	0.9889	0.7718	0.9055	0.0535
GJB2	0.8225	1.0088	0.9268	0.8640	0.9055	0.0406
LOC728806	0.8337	1.0453	0.8619	0.8813	0.9056	0.0476
ANKRD35	0.7149	1.0717	0.9213	0.9145	0.9056	0.0732
FALZ	0.8042	1.0327	0.9506	0.8348	0.9056	0.0528
AGPS	0.6849	1.3224	0.8753	0.7399	0.9056	0.1446
LOC731751	0.8105	1.0723	1.0402	0.6995	0.9056	0.0901
FBXL22	0.8853	1.1856	0.8625	0.6891	0.9056	0.1031
LOC645173	0.7122	1.1734	1.0257	0.7112	0.9056	0.1159
LOC649469	0.8335	0.9633	1.0867	0.7391	0.9056	0.0759
LOC652224	0.8656	0.8979	0.9371	0.9219	0.9056	0.0156
LOC1001345	0.7990	0.8758	1.0884	0.8597	0.9057	0.0631
PKP3	0.8528	1.1107	0.8865	0.7729	0.9057	0.0723
HIP1	0.7937	1.0676	0.9246	0.8369	0.9057	0.0604
SRP9	0.9066	1.0598	0.9709	0.6858	0.9057	0.0798
FOXD2	0.7757	1.0761	0.8656	0.9059	0.9058	0.0629
TTC27	0.8571	1.0725	0.9861	0.7077	0.9058	0.0795
MPV17L2	0.7439	1.1666	0.9548	0.7581	0.9059	0.0994
LOC1001346	0.8609	0.9941	0.9470	0.8215	0.9059	0.0394
LOC1001293	0.8115	1.0429	1.0178	0.7514	0.9059	0.0731
REST	0.8247	0.9211	0.9373	0.9405	0.9059	0.0274
SOD2	0.8027	1.0634	0.9349	0.8227	0.9059	0.0600
MIR1180	0.8825	0.9233	0.9719	0.8460	0.9059	0.0271
LOC441249	1.0037	0.9810	0.9141	0.7248	0.9059	0.0633
LOC1001344	0.8366	1.0057	0.9908	0.7906	0.9059	0.0542
PEX7	0.6866	1.3292	0.9971	0.6108	0.9059	0.1640
TRIP10	0.7676	1.0864	0.9308	0.8389	0.9059	0.0688
ZNF384	0.8697	1.0538	0.8992	0.8011	0.9059	0.0534
TMEM163	0.8659	1.1762	0.8164	0.7654	0.9060	0.0924
CUL3	0.8808	0.9328	0.9450	0.8653	0.9060	0.0195
SETD5	0.7889	1.1511	0.8435	0.8404	0.9060	0.0826
FBXW9	0.7663	1.3323	0.8606	0.6647	0.9060	0.1476
ZNF223	0.9229	0.9335	0.9763	0.7914	0.9060	0.0399
LOC1001295	0.7632	1.0688	1.0253	0.7668	0.9060	0.0819

HSF2	0.8180	1.1386	0.8679	0.7996	0.9060	0.0789
C9orf156	0.7430	1.2989	0.8434	0.7390	0.9061	0.1331
ZBTB8A	0.8596	1.0032	0.9681	0.7935	0.9061	0.0484
LOC728249	0.8174	1.1465	0.8467	0.8139	0.9061	0.0805
PRDX3	0.7053	1.3069	0.9594	0.6529	0.9061	0.1494
ZKSCAN3	0.8457	1.1343	0.8706	0.7739	0.9061	0.0788
LST-3TM12	0.9069	0.9422	0.9400	0.8356	0.9062	0.0249
FLJ32214	0.9236	0.7798	0.9953	0.9261	0.9062	0.0453
LOC648481	0.8383	0.9882	0.9442	0.8541	0.9062	0.0359
SEC24D	0.7917	1.1134	0.8816	0.8382	0.9062	0.0715
GRK4	0.8705	1.0835	0.8614	0.8096	0.9062	0.0606
LOC648509	0.8897	1.0282	0.9221	0.7850	0.9062	0.0501
SEMA6C	0.9054	0.9420	0.9377	0.8399	0.9062	0.0236
DIP2B	0.8386	1.1937	0.8557	0.7370	0.9063	0.0993
TAAR2	0.9234	1.0519	0.8014	0.8483	0.9063	0.0547
LOC1001344	0.8249	1.0376	0.9745	0.7884	0.9063	0.0595
LOC642784	0.7798	1.1139	0.9808	0.7509	0.9063	0.0860
EBF2	0.8465	0.9849	0.9659	0.8284	0.9064	0.0402
WDFY2	0.8791	1.0600	0.9248	0.7619	0.9064	0.0616
NRARP	0.8754	1.0099	0.9049	0.8356	0.9064	0.0373
LOC642503	0.8209	1.0616	0.8304	0.9129	0.9064	0.0557
C2orf73	0.9225	0.8845	0.9553	0.8637	0.9065	0.0203
LOC1001305	0.7996	1.0258	0.9320	0.8686	0.9065	0.0481
SLC25A27	0.8228	1.0013	0.9290	0.8729	0.9065	0.0383
OPN1LW	0.8988	0.9970	0.9254	0.8049	0.9065	0.0397
SRRM1	0.9606	1.1499	0.8543	0.6614	0.9065	0.1020
FAM119A	0.7371	1.0900	1.0906	0.7085	0.9066	0.1063
LOC643778	0.9405	1.0273	0.8568	0.8017	0.9066	0.0493
NAT13	0.7661	1.0385	1.0947	0.7269	0.9066	0.0934
PHF3	0.7644	1.2637	0.8302	0.7680	0.9066	0.1200
RPP30	0.8508	0.9672	0.9623	0.8462	0.9066	0.0336
USP41	0.8852	0.9633	0.9800	0.7978	0.9066	0.0417
PODXL	0.8552	1.0762	0.9602	0.7348	0.9066	0.0729
LOC728557	0.8296	1.0122	0.8497	0.9352	0.9067	0.0420
UQCRH	0.7893	1.2251	0.8753	0.7369	0.9067	0.1099
MTSS1	0.7594	1.0757	0.9811	0.8107	0.9067	0.0736
UBE2R2	0.8248	1.0143	0.9087	0.8790	0.9067	0.0398
SENP5	0.8079	1.1564	0.9524	0.7102	0.9067	0.0970
NUDT14	0.7762	1.2046	0.9134	0.7327	0.9067	0.1065
LOC648556	0.8246	1.0693	0.8823	0.8508	0.9067	0.0554
LOC652741	0.9082	1.0322	0.9025	0.7842	0.9067	0.0506
RCAN3	0.9526	0.8687	0.9833	0.8224	0.9068	0.0371
LOC400214	0.8640	1.0743	0.9002	0.7886	0.9068	0.0605
CLDND1	0.6374	1.2107	1.1525	0.6265	0.9068	0.1591
IKBKB	0.8316	1.2118	0.8149	0.7688	0.9068	0.1025
HIST3H2BB	0.7441	1.0679	0.9653	0.8499	0.9068	0.0702

ARHGAP5	0.8124	1.0921	1.0041	0.7186	0.9068	0.0857
LOC654074	0.7874	0.9987	0.9483	0.8929	0.9068	0.0453
ZNF672	0.7748	1.2157	0.9277	0.7092	0.9069	0.1127
BGN	0.9098	0.9278	0.9512	0.8386	0.9069	0.0243
LOC391509	0.7801	1.1963	0.9119	0.7392	0.9069	0.1033
MOSPD3	0.8885	1.1196	0.8233	0.7963	0.9069	0.0735
USP17	0.8330	0.9573	0.8619	0.9756	0.9069	0.0351
BRSK2	0.8437	1.0096	0.9464	0.8281	0.9069	0.0431
CDRT15	0.9426	0.9469	1.0029	0.7355	0.9070	0.0588
TSEN34	0.8545	1.2528	0.8593	0.6613	0.9070	0.1242
LOC654042	0.8374	0.9664	1.0084	0.8157	0.9070	0.0474
SNORA43	0.8496	1.0765	0.8678	0.8340	0.9070	0.0569
PI15	0.8363	1.1187	0.9196	0.7534	0.9070	0.0783
LOC441533	0.8391	1.0086	0.8725	0.9078	0.9070	0.0367
NOMO1	0.8036	1.1962	0.8503	0.7781	0.9070	0.0975
LOC651638	0.7880	1.0612	0.8476	0.9314	0.9070	0.0592
CECR2	0.8786	0.9354	0.9382	0.8760	0.9070	0.0172
ZNF281	0.8621	1.0661	1.0017	0.6984	0.9071	0.0815
HSN2	0.8156	1.0319	0.9204	0.8605	0.9071	0.0468
AQP12B	0.7551	1.2575	0.9819	0.6340	0.9072	0.1373
ZNF24	0.7768	1.2061	0.8809	0.7649	0.9072	0.1030
PPP1R8	0.8382	1.0824	1.0017	0.7064	0.9072	0.0840
OR51B4	0.6510	0.9981	1.0098	0.9700	0.9072	0.0858
FLJ20297	0.9708	0.9301	0.9320	0.7960	0.9072	0.0382
XRN2	0.7695	1.2001	0.9905	0.6688	0.9072	0.1185
ZNF398	0.8235	1.0276	0.9081	0.8700	0.9073	0.0437
C14orf145	0.8053	1.0462	0.9839	0.7939	0.9073	0.0635
FLJ38973	0.8454	1.0139	0.9477	0.8223	0.9073	0.0448
TREM2	0.8507	0.9375	0.9212	0.9199	0.9073	0.0193
LOC1001304	0.8107	0.9959	0.9690	0.8539	0.9074	0.0446
CEP76	0.8314	1.0206	0.9508	0.8267	0.9074	0.0474
KIAA1688	0.7740	1.1541	0.9997	0.7020	0.9074	0.1038
DNAJC18	0.9659	1.0278	0.9027	0.7333	0.9074	0.0634
DAK	0.9023	1.0788	0.8929	0.7558	0.9075	0.0662
FAM73A	0.7804	1.0354	0.9604	0.8537	0.9075	0.0564
PCOLCE	0.9037	1.0885	0.8551	0.7827	0.9075	0.0653
LOC645010	0.9660	0.9641	0.8670	0.8331	0.9075	0.0339
HIST2H3A	0.7078	1.1704	0.8634	0.8887	0.9075	0.0963
IDE	0.6908	1.2746	0.9660	0.6988	0.9076	0.1381
LOC651524	0.9002	0.8182	0.9488	0.9630	0.9076	0.0327
C8orf73	0.8684	1.0580	0.9035	0.8002	0.9076	0.0546
DOCK7	0.8205	1.2428	0.8607	0.7063	0.9076	0.1164
CETN2	0.7644	1.1239	1.0284	0.7137	0.9076	0.0998
HIF1AN	0.8447	1.1520	0.8938	0.7399	0.9076	0.0875
FLJ32065	0.8052	0.9819	0.9873	0.8559	0.9076	0.0457
IMPG1	0.8508	0.9984	0.9567	0.8244	0.9076	0.0416

DUSP21	0.8846	1.0906	0.7891	0.8662	0.9076	0.0644
LOC648189	0.7791	1.0361	0.9624	0.8529	0.9076	0.0570
PAFAH1B2	0.8027	1.1211	0.8946	0.8121	0.9076	0.0741
CSNK1G1	0.8009	1.1297	0.9647	0.7352	0.9077	0.0884
LOC645600	0.8983	0.9555	0.9845	0.7926	0.9077	0.0423
CNOT10	0.6618	1.2962	0.8637	0.8091	0.9077	0.1363
C18orf10	0.7861	1.2929	0.9185	0.6335	0.9077	0.1410
ATP6V1G2	0.7977	1.0533	0.9552	0.8248	0.9078	0.0595
ZXDC	0.8842	1.0536	0.9364	0.7569	0.9078	0.0615
LOC651272	0.8372	0.9015	1.0114	0.8811	0.9078	0.0370
ZNF669	0.7607	1.0911	0.9750	0.8044	0.9078	0.0766
LOC1001323	0.9798	0.9349	0.9877	0.7290	0.9078	0.0607
C16orf61	0.7272	1.2322	1.0256	0.6464	0.9078	0.1354
C7orf46	0.7984	1.0086	0.9757	0.8487	0.9079	0.0502
LOC51152	0.8072	1.1355	0.9063	0.7824	0.9079	0.0805
ATG2B	0.9043	1.0806	0.8640	0.7825	0.9079	0.0629
LOC649125	0.9072	0.9899	0.8889	0.8456	0.9079	0.0302
HNRNPU	0.6816	1.3081	0.8237	0.8185	0.9080	0.1374
PRR5	0.8097	1.0998	0.9002	0.8220	0.9080	0.0670
SPANXA2	0.8340	1.0214	0.9539	0.8227	0.9080	0.0480
RNPEP	0.7875	1.2593	0.9290	0.6563	0.9080	0.1296
LOC442517	0.8327	0.9246	1.0008	0.8740	0.9080	0.0362
RPL10L	0.9149	1.0416	0.9906	0.6850	0.9080	0.0788
SAMD8	0.8318	0.9944	0.9708	0.8352	0.9081	0.0433
LOC1001305	0.8840	0.9274	0.9646	0.8563	0.9081	0.0239
LOC645143	0.9671	0.8612	0.9346	0.8694	0.9081	0.0256
PRKD1	0.7332	1.4236	0.8057	0.6698	0.9081	0.1741
CNIH4	0.7277	1.1579	1.1515	0.5952	0.9081	0.1449
LRRC37A2	0.8648	0.8411	1.0151	0.9113	0.9081	0.0385
LOC654134	0.9414	1.0515	0.9136	0.7259	0.9081	0.0676
FDPS	0.9730	0.9086	0.9409	0.8100	0.9081	0.0352
FLJ12355	0.9040	1.0822	0.8532	0.7932	0.9082	0.0623
UBE2F	0.7231	1.3001	0.9347	0.6748	0.9082	0.1423
ABCG4	0.8772	1.0355	0.9634	0.7565	0.9082	0.0600
C2orf16	0.8474	0.9327	0.8855	0.9672	0.9082	0.0263
BTBD2	0.8283	1.2318	0.8739	0.6988	0.9082	0.1141
SNORD65	0.9000	1.0723	0.8445	0.8162	0.9082	0.0574
LHX5	0.8826	1.0215	0.8996	0.8293	0.9083	0.0406
KCNK16	0.8911	0.9616	0.9762	0.8042	0.9083	0.0393
PPWD1	0.8536	1.0522	0.9763	0.7510	0.9083	0.0665
EPN1	0.8488	1.1367	0.8668	0.7810	0.9083	0.0783
LOC1001341	0.8329	1.1553	0.9890	0.6561	0.9083	0.1068
RANBP2	0.7550	0.9703	1.1418	0.7662	0.9083	0.0922
LOC731528	0.8857	1.0004	0.8784	0.8688	0.9083	0.0309
MTG1	0.7288	1.1165	1.0000	0.7879	0.9083	0.0906
TTLL4	0.8126	1.1680	0.8502	0.8025	0.9083	0.0872

MGC26733	0.8118	1.0924	0.8369	0.8922	0.9083	0.0636
SIRT1	0.8992	1.0067	1.0227	0.7048	0.9084	0.0732
LOC648176	0.8691	1.0085	0.9781	0.7779	0.9084	0.0528
TEC	0.7855	1.0215	1.0071	0.8195	0.9084	0.0616
TXNDC17	0.8327	1.0502	1.0877	0.6631	0.9084	0.0992
NAIF1	0.8479	1.1863	0.8816	0.7180	0.9084	0.0991
BTF3	0.8190	1.0724	1.0350	0.7075	0.9085	0.0872
EXOSC1	0.8153	1.1595	0.9713	0.6878	0.9085	0.1018
LDB2	0.8791	0.9657	0.8823	0.9067	0.9085	0.0201
FAM72D	0.7864	1.0930	0.9463	0.8083	0.9085	0.0709
LOC1001300	0.8316	0.9649	0.8748	0.9627	0.9085	0.0331
LOC283487	0.6843	1.0428	0.9549	0.9520	0.9085	0.0776
THAP8	0.9166	1.0056	0.9416	0.7704	0.9085	0.0497
LOC649679	0.8737	0.9912	0.9849	0.7844	0.9085	0.0494
NKX2-2	0.8596	0.9705	0.9351	0.8691	0.9086	0.0266
UNKL	0.8052	1.0101	0.9684	0.8505	0.9086	0.0483
LRRC50	0.8039	0.9453	0.9682	0.9169	0.9086	0.0364
MKLN1	0.8435	1.2479	0.8263	0.7166	0.9086	0.1165
ZBTB25	0.8570	1.1060	0.9188	0.7526	0.9086	0.0742
GSTCD	0.7862	0.9985	0.9303	0.9195	0.9086	0.0444
KIAA1797	0.9046	1.1060	0.9888	0.6351	0.9086	0.1001
LOC284757	0.8172	0.9791	0.9585	0.8796	0.9086	0.0373
CIP29	0.7071	1.2729	0.9581	0.6965	0.9086	0.1356
RAP1GAP	0.9954	1.1210	0.8384	0.6799	0.9087	0.0957
SLC30A4	0.8143	1.1395	0.9241	0.7567	0.9087	0.0844
MOCS1	0.8090	1.1787	0.9298	0.7172	0.9087	0.1000
GOT2	0.7012	1.4254	0.8314	0.6766	0.9087	0.1756
LOC388436	0.9279	0.8684	0.9603	0.8780	0.9087	0.0216
MYO1F	0.8846	0.9478	0.9522	0.8502	0.9087	0.0249
NCRNA0009	0.8320	1.0609	0.9890	0.7528	0.9087	0.0706
LOC652437	0.8648	0.9648	0.9890	0.8163	0.9087	0.0409
SYNGR3	0.9154	1.0989	0.8184	0.8023	0.9087	0.0681
LOC642156	0.7595	1.0594	0.8145	1.0015	0.9087	0.0721
RBMXL2	0.8951	1.0175	0.8669	0.8555	0.9088	0.0372
ZMYM5	0.8631	1.0793	0.9181	0.7745	0.9088	0.0641
C12orf73	0.8296	1.1287	0.9052	0.7715	0.9088	0.0783
OR9G9	0.8576	1.0221	0.8819	0.8734	0.9088	0.0381
LOC1001283	0.8474	0.9662	0.9414	0.8802	0.9088	0.0273
LOC652022	0.7400	1.1177	0.8320	0.9454	0.9088	0.0813
LOC124216	0.7540	0.9811	1.0488	0.8512	0.9088	0.0659
MAST4	0.7863	1.2283	0.9201	0.7005	0.9088	0.1157
LOC390834	0.8299	1.0090	0.9837	0.8126	0.9088	0.0509
FAM127C	0.7946	1.1237	0.9263	0.7906	0.9088	0.0783
LOC645700	0.8166	1.1022	1.0253	0.6911	0.9088	0.0944
ZNF707	0.7981	1.0135	0.9999	0.8240	0.9089	0.0568
REPIN1	0.8029	1.3182	0.7858	0.7286	0.9089	0.1374

SNORD96B	0.8901	0.9193	1.0092	0.8169	0.9089	0.0398
TPP2	0.8237	1.0770	0.9896	0.7452	0.9089	0.0757
UTP15	0.7941	1.0596	1.0607	0.7211	0.9089	0.0886
SLC38A10	0.8692	1.0794	0.9051	0.7819	0.9089	0.0625
C15orf28	0.8145	1.0231	0.8780	0.9199	0.9089	0.0438
LOC728178	0.8316	1.0987	0.8460	0.8593	0.9089	0.0635
ZNF35	0.7348	1.0742	1.0548	0.7718	0.9089	0.0903
LOC642681	0.8638	1.0408	0.9651	0.7661	0.9089	0.0598
SMAP2	0.8174	1.2094	0.9422	0.6668	0.9089	0.1149
LOC653630	0.8269	0.8928	0.9648	0.9513	0.9089	0.0315
MED6	0.7032	1.2681	0.9370	0.7275	0.9090	0.1307
ITGA5	0.8074	1.1679	0.8794	0.7812	0.9090	0.0888
FBXL20	0.8036	1.1893	0.9199	0.7233	0.9090	0.1018
LOC652369	0.8579	0.9710	0.8413	0.9659	0.9090	0.0345
PPIF	0.7483	1.0147	1.0095	0.8638	0.9091	0.0640
GLS2	0.8289	1.0475	0.9569	0.8030	0.9091	0.0571
PRKG1	0.9084	0.9761	0.9682	0.7838	0.9091	0.0444
MRPL54	0.7221	1.2371	0.9603	0.7170	0.9091	0.1232
TBC1D17	1.0217	1.0847	0.7695	0.7606	0.9091	0.0842
SOS2	0.8815	1.0664	0.8539	0.8347	0.9091	0.0533
STK32C	0.7759	1.1185	0.9911	0.7511	0.9091	0.0882
HCST	0.7700	1.1413	0.9091	0.8162	0.9092	0.0826
TOMM34	0.7748	1.2943	0.8551	0.7125	0.9092	0.1317
LOC285296	0.8689	1.0141	0.9661	0.7877	0.9092	0.0505
SFRS12IP1	0.6811	1.1560	0.9527	0.8470	0.9092	0.0995
LOC646801	0.9197	0.9680	0.9434	0.8059	0.9092	0.0358
LOC283914	0.9036	0.9463	0.9581	0.8290	0.9093	0.0292
FVT1	0.7877	1.2814	0.8530	0.7150	0.9093	0.1272
LOC729222	0.8032	1.0647	0.9446	0.8247	0.9093	0.0604
ERCC-00019	0.7327	0.9946	0.9758	0.9343	0.9093	0.0602
LOC441957	0.8280	1.0130	0.9421	0.8543	0.9093	0.0423
EID1	0.7893	1.2254	0.8634	0.7594	0.9094	0.1076
RNPC3	0.8649	1.0257	0.9055	0.8414	0.9094	0.0410
FSD1L	0.7152	1.1755	0.9543	0.7924	0.9094	0.1018
C9orf173	0.9160	0.8216	1.0184	0.8816	0.9094	0.0412
SOX15	0.8169	0.9392	0.9634	0.9182	0.9094	0.0322
FNDC3B	0.6820	1.1727	1.0694	0.7136	0.9094	0.1242
DEFB4	1.0694	0.9307	0.8332	0.8045	0.9094	0.0598
LOC645466	0.7047	1.1474	0.9580	0.8278	0.9095	0.0947
ZNF425	0.8656	1.0872	0.9889	0.6963	0.9095	0.0843
GFRA2	0.8366	1.1031	0.8516	0.8468	0.9095	0.0646
LOC652082	0.7971	1.0621	0.9699	0.8091	0.9095	0.0643
LOC646550	0.9155	0.9588	0.9611	0.8028	0.9095	0.0371
RAP2C	0.8234	1.0205	1.0550	0.7393	0.9095	0.0763
LOC728324	0.8336	1.2437	0.8629	0.6980	0.9096	0.1170
SLCO4C1	0.8893	0.9932	0.9179	0.8378	0.9096	0.0324

LOC389031	0.8731	0.8886	0.9865	0.8900	0.9096	0.0259
M6PR	0.6077	1.3802	0.9383	0.7121	0.9096	0.1714
SERINC3	0.8624	1.1279	0.9129	0.7353	0.9096	0.0818
S100A14	0.8171	0.8299	0.9787	1.0127	0.9096	0.0503
LOC728416	0.8435	1.0063	1.0197	0.7691	0.9096	0.0617
SHC4	0.9313	0.9630	0.8583	0.8861	0.9097	0.0233
WDR88	0.9458	0.9681	0.9093	0.8156	0.9097	0.0336
LOC731432	0.7928	1.0215	0.9384	0.8861	0.9097	0.0479
BTN1A1	0.9335	0.8861	0.9539	0.8653	0.9097	0.0205
LOC644184	0.8888	0.9449	0.8405	0.9647	0.9097	0.0281
N6AMT1	0.7732	1.1377	0.9612	0.7668	0.9097	0.0884
INS	0.8145	0.9192	0.9888	0.9164	0.9097	0.0359
NARS	0.8248	1.1216	1.1387	0.5541	0.9098	0.1387
SPTA1	0.9201	1.0258	0.8464	0.8469	0.9098	0.0424
ACOT11	0.8138	1.1706	0.8782	0.7766	0.9098	0.0894
F2RL1	0.7237	1.1959	1.0312	0.6883	0.9098	0.1226
LOC1001308	0.8962	0.9655	0.8981	0.8796	0.9098	0.0190
FMNL1	0.8305	1.0025	1.1036	0.7028	0.9098	0.0891
CCR7	0.9037	0.8929	0.9329	0.9099	0.9099	0.0084
LOC645314	0.8449	1.0918	0.9428	0.7601	0.9099	0.0712
RNASEH2C	0.9235	1.0057	0.9145	0.7958	0.9099	0.0432
ABI2	0.8672	1.0391	0.9442	0.7892	0.9099	0.0534
LRRC8B	0.8850	1.0681	0.8812	0.8054	0.9099	0.0558
AZI1	0.8888	1.1139	0.8002	0.8369	0.9100	0.0704
LOC729911	0.8649	0.9202	0.8998	0.9551	0.9100	0.0189
LOC1001313	0.7059	1.1229	1.0343	0.7768	0.9100	0.1001
RAMP1	0.7403	1.1619	0.9760	0.7618	0.9100	0.0994
OR52M1	0.6285	0.9693	1.0948	0.9475	0.9100	0.0993
LOC646764	0.8297	1.0643	0.9632	0.7829	0.9100	0.0640
MIRH1	0.8215	0.9945	0.9026	0.9216	0.9100	0.0356
MTRF1	0.8384	1.0905	0.8965	0.8149	0.9101	0.0625
LOC650976	0.8245	1.0806	0.9838	0.7514	0.9101	0.0747
GPR161	0.8926	1.0200	0.8492	0.8785	0.9101	0.0377
LOC730255	0.5604	1.0320	1.3090	0.7390	0.9101	0.1647
NDUFA4L2	0.8644	1.0929	0.7194	0.9640	0.9102	0.0789
LOC643479	0.8405	0.9824	1.0046	0.8131	0.9102	0.0487
LOC645413	0.9096	0.9213	1.0052	0.8045	0.9102	0.0412
NUDT3	0.7195	1.2418	0.9383	0.7413	0.9102	0.1210
LOC646714	1.0064	0.9964	0.8437	0.7946	0.9103	0.0536
KCTD1	0.9114	0.9380	0.9739	0.8177	0.9103	0.0334
OR4S1	0.7777	0.9024	0.9529	1.0080	0.9103	0.0492
MYO1C	0.8135	1.0932	0.9589	0.7755	0.9103	0.0726
OR5B12	0.8100	1.0154	0.9560	0.8599	0.9103	0.0463
SNORD52	0.8321	1.0314	0.9234	0.8543	0.9103	0.0448
LOC220930	0.8468	1.0340	0.9209	0.8396	0.9103	0.0451
LAD1	0.7145	1.3205	0.9473	0.6592	0.9104	0.1503

LOC158318	0.7633	0.9858	0.9511	0.9414	0.9104	0.0499
CSNK1G2	0.9319	1.0507	0.9658	0.6932	0.9104	0.0766
B4GALT5	0.7721	1.1149	0.9446	0.8100	0.9104	0.0776
TFF1	0.7680	1.0426	0.9033	0.9279	0.9104	0.0564
LOC650098	0.8796	0.9709	0.9153	0.8759	0.9105	0.0220
LOC653481	0.8639	1.0168	0.9137	0.8475	0.9105	0.0381
QRICH1	0.8333	1.2172	0.8445	0.7471	0.9105	0.1045
LOC1001297	0.8004	0.9885	1.0074	0.8458	0.9105	0.0515
FBXO28	0.6570	1.3521	1.0046	0.6283	0.9105	0.1702
SNHG8	0.8113	1.0718	0.9766	0.7824	0.9105	0.0687
STK36	0.9766	1.1929	0.6790	0.7936	0.9105	0.1123
CRLF3	0.8399	1.1112	0.9762	0.7149	0.9105	0.0856
ERCC-00002	0.8099	1.0110	0.9045	0.9169	0.9105	0.0411
FRS2	0.8441	1.0192	0.9445	0.8344	0.9106	0.0439
AAK1	0.7280	1.1240	0.9481	0.8422	0.9106	0.0841
CHMP6	0.8928	1.1401	0.9044	0.7050	0.9106	0.0891
OTUD4	0.8000	1.0685	0.9158	0.8580	0.9106	0.0577
LOC650717	0.8460	1.0577	0.9225	0.8163	0.9106	0.0539
CCDC123	0.8696	1.2459	0.7990	0.7280	0.9106	0.1154
ALG11	0.8401	1.0441	0.9687	0.7894	0.9106	0.0584
MIR551B	0.8884	0.9677	0.8895	0.8968	0.9106	0.0191
ZNF473	0.8020	1.1492	0.9406	0.7507	0.9106	0.0891
LOC1001297	0.8095	1.0329	0.9879	0.8122	0.9106	0.0583
XAGE1	0.8328	1.1586	0.9828	0.6683	0.9106	0.1047
LOC642035	0.9074	0.9342	0.9722	0.8287	0.9106	0.0304
PVR	0.8672	1.0468	0.9128	0.8158	0.9106	0.0495
LOC644451	0.8912	1.1387	0.7680	0.8447	0.9106	0.0801
PABPC1L2A	0.9115	0.9626	0.9261	0.8425	0.9107	0.0251
LOC1001331	0.8209	1.1233	0.9234	0.7752	0.9107	0.0773
LOC652065	0.8514	1.0327	0.9612	0.7976	0.9107	0.0530
CTDSP2	0.5999	1.4473	0.9616	0.6342	0.9107	0.1965
GBX1	0.8759	1.0238	0.8883	0.8549	0.9107	0.0383
C14orf2	0.8846	1.1814	0.8424	0.7346	0.9107	0.0956
LOC643868	0.7787	0.9991	1.0179	0.8473	0.9108	0.0583
LOC1001330	0.6308	1.2060	1.0695	0.7368	0.9108	0.1357
LOC645232	0.8127	0.9091	0.9916	0.9298	0.9108	0.0371
ATP7B	0.8656	0.9798	0.9180	0.8799	0.9108	0.0255
OPLAH	0.7881	1.2570	0.8987	0.6996	0.9108	0.1224
DTNBP1	0.8710	1.0594	0.9535	0.7595	0.9108	0.0635
NSUN6	0.8599	1.0326	1.0130	0.7379	0.9108	0.0694
C1QL1	0.8535	1.0386	0.8700	0.8813	0.9109	0.0430
LOC652472	0.7899	0.9835	1.0631	0.8070	0.9109	0.0670
UBP1	0.8478	1.2925	0.7998	0.7033	0.9109	0.1307
FLJ33590	0.7701	0.9855	0.9793	0.9088	0.9109	0.0501
ASNSD1	0.6635	1.2987	0.9400	0.7414	0.9109	0.1418
ZBBX	0.8231	1.1766	0.8602	0.7838	0.9109	0.0899

CAD	0.7960	1.2690	0.8522	0.7266	0.9110	0.1221
KIAA0090	0.7287	1.1798	1.0061	0.7293	0.9110	0.1109
LOC728940	0.9448	0.9826	0.8790	0.8377	0.9110	0.0325
SLC46A2	0.8613	0.9532	0.9446	0.8850	0.9110	0.0225
ZNF570	0.8873	0.9604	0.9565	0.8399	0.9110	0.0291
LOC644065	0.8973	1.0252	0.9182	0.8034	0.9111	0.0455
LOC402508	0.9100	0.9954	0.9475	0.7913	0.9111	0.0436
ADI1	0.8787	1.0224	0.9771	0.7660	0.9111	0.0569
ZNF345	0.8454	0.9907	0.9692	0.8392	0.9111	0.0400
AKAP8	0.7809	1.0939	0.9358	0.8339	0.9111	0.0689
LOC648625	0.8482	0.8323	0.9830	0.9810	0.9112	0.0411
TNFRSF1B	0.8482	0.9855	0.9441	0.8669	0.9112	0.0323
PIP5K2B	0.8290	1.1900	1.0123	0.6135	0.9112	0.1236
DIP2A	0.8652	1.0836	0.8913	0.8046	0.9112	0.0603
LOC400389	0.8779	0.9568	1.0058	0.8044	0.9112	0.0443
VWA2	0.8539	0.9661	0.9831	0.8417	0.9112	0.0369
GAL3ST2	0.9207	0.9998	0.9423	0.7821	0.9112	0.0462
TIRAP	0.8652	1.0478	0.8918	0.8403	0.9112	0.0467
BIN3	0.8402	0.9407	0.9216	0.9426	0.9112	0.0242
RARS	0.7503	1.1795	0.9830	0.7322	0.9112	0.1061
LOC1001304	0.9239	0.9959	0.9023	0.8229	0.9113	0.0356
LOC644577	0.9333	0.9857	0.8563	0.8700	0.9113	0.0299
SPCS2	0.6907	1.2903	1.0514	0.6127	0.9113	0.1584
APPL1	0.8276	1.1988	0.7914	0.8274	0.9113	0.0962
IL1R2	0.8053	1.0306	1.0036	0.8059	0.9113	0.0613
C11orf54	0.7985	1.1807	1.0152	0.6510	0.9113	0.1168
LOC652623	0.8714	0.8520	0.9790	0.9430	0.9113	0.0298
FLJ42280	0.8027	0.9568	0.9620	0.9240	0.9114	0.0372
HES5	0.7947	0.9974	0.9733	0.8800	0.9114	0.0464
CLCN7	0.8628	1.2346	0.9407	0.6074	0.9114	0.1291
LOC340357	0.7724	0.9827	0.9740	0.9167	0.9114	0.0486
MDH2	0.8651	1.1124	0.9775	0.6907	0.9114	0.0893
PLEKHF2	0.7568	1.0910	0.9916	0.8063	0.9114	0.0783
FAM87B	0.8596	0.8950	1.0399	0.8513	0.9115	0.0439
ERCC-00126	0.9166	0.9172	0.9722	0.8399	0.9115	0.0272
TWF2	0.8725	1.2046	0.9160	0.6530	0.9115	0.1134
NBPF12	0.8257	1.0217	0.9644	0.8342	0.9115	0.0485
BRUNOL6	0.8580	0.9852	0.9027	0.9001	0.9115	0.0266
LOC646501	0.8916	1.0607	0.8313	0.8625	0.9115	0.0512
NUDCD1	0.8367	0.9376	0.9997	0.8721	0.9115	0.0361
DLEU2	0.8106	1.0352	0.9911	0.8093	0.9116	0.0593
FLJ31306	0.8196	0.9731	1.0759	0.7776	0.9116	0.0690
LOC283788	0.9050	1.0572	0.8642	0.8200	0.9116	0.0516
LOC642449	0.8618	1.0722	0.8825	0.8299	0.9116	0.0546
LOC653175	0.8774	1.0584	0.8642	0.8464	0.9116	0.0493
LOC649260	0.8670	0.9908	0.9563	0.8324	0.9116	0.0371

KLHL15	0.8869	0.9513	0.9241	0.8841	0.9116	0.0161
GAPVD1	0.7345	1.1872	1.0404	0.6843	0.9116	0.1210
SSR2	0.7910	1.2325	0.9659	0.6570	0.9116	0.1242
SEC62	0.9351	1.0061	0.8777	0.8276	0.9116	0.0384
LOC1001322	0.8715	1.0194	0.9458	0.8097	0.9116	0.0454
LOC1001332	0.7888	0.9796	1.0410	0.8372	0.9117	0.0591
DDX60L	0.8724	1.0345	0.9493	0.7904	0.9117	0.0523
DDX19B	0.7399	1.2692	0.9212	0.7164	0.9117	0.1277
LOC728588	0.7840	1.0466	0.9841	0.8320	0.9117	0.0620
CMTM5	0.7555	1.0914	0.9292	0.8707	0.9117	0.0699
TINAGL1	0.8602	0.9426	1.0382	0.8058	0.9117	0.0507
PNMA6A	0.8783	1.0982	0.9147	0.7557	0.9117	0.0708
LRRC1	0.7918	1.1784	0.9356	0.7411	0.9117	0.0980
YTHDC2	0.8448	1.0118	0.8450	0.9454	0.9117	0.0409
CHST1	0.9158	0.9861	1.0542	0.6908	0.9118	0.0789
LOC1001327	0.7926	1.1356	0.8143	0.9047	0.9118	0.0784
ATP13A3	0.7493	1.0449	0.9245	0.9285	0.9118	0.0609
LOC441743	0.6066	1.0301	1.2317	0.7788	0.9118	0.1376
REV3L	0.8584	1.1403	0.8340	0.8146	0.9118	0.0767
P2RX6	0.7643	0.9951	1.0174	0.8706	0.9118	0.0588
GTF2F2	0.6483	1.2970	0.9585	0.7435	0.9118	0.1439
PTPN1	0.8031	1.1730	0.9479	0.7235	0.9119	0.0987
LOC642486	0.7353	1.0869	0.8511	0.9742	0.9119	0.0760
LOC1001308	0.7943	1.0624	0.9376	0.8532	0.9119	0.0582
LOC1001302	0.8673	0.9729	0.8344	0.9731	0.9119	0.0359
FAM131C	0.8367	0.9638	0.9708	0.8764	0.9120	0.0330
LOC644754	0.8554	0.9845	0.8942	0.9139	0.9120	0.0270
SMN2	0.9180	0.9105	1.0197	0.7997	0.9120	0.0450
C14orf100	0.7930	1.0955	1.0987	0.6608	0.9120	0.1102
TMEM125	0.7513	1.4123	0.7797	0.7047	0.9120	0.1675
SFRS15	0.9396	1.1749	0.8019	0.7317	0.9120	0.0977
SCARNA17	0.9429	0.9297	0.8975	0.8780	0.9120	0.0148
TTLL5	0.8447	1.0928	0.9262	0.7844	0.9121	0.0669
C14orf178	0.8572	0.9961	0.9579	0.8372	0.9121	0.0385
MYH13	0.9418	0.9247	0.9580	0.8238	0.9121	0.0302
TMEM161A	0.8002	1.0936	0.9124	0.8423	0.9121	0.0648
LOC649873	0.8005	1.0650	0.9172	0.8657	0.9121	0.0563
MIR1250	0.8877	0.9722	0.9825	0.8061	0.9121	0.0412
SMO	0.9290	1.0768	0.9105	0.7322	0.9121	0.0706
ARID1A	0.6772	1.3008	0.8976	0.7730	0.9121	0.1372
GSTM2	0.9239	1.0081	0.9332	0.7835	0.9122	0.0469
LOC651701	0.8608	1.0767	0.8237	0.8874	0.9122	0.0564
GPS2	0.8295	0.9959	0.9412	0.8821	0.9122	0.0361
TFG	0.7835	1.2182	0.9583	0.6888	0.9122	0.1163
HCN1	0.8627	1.0423	0.9361	0.8078	0.9122	0.0507
PITPNC1	0.8335	1.0097	0.9637	0.8419	0.9122	0.0441

UQCR	0.6779	1.3430	0.8618	0.7663	0.9122	0.1484
PAGE3	0.8227	1.0854	0.8670	0.8740	0.9122	0.0588
IL1F9	0.9497	0.9171	0.9718	0.8103	0.9123	0.0358
ELK4	0.8269	1.0811	0.9507	0.7904	0.9123	0.0659
HAS1	0.9597	0.9757	0.8707	0.8431	0.9123	0.0326
GOLGA4	0.8988	1.0793	0.9104	0.7606	0.9123	0.0652
LOC145853	0.8612	1.1171	0.9753	0.6956	0.9123	0.0892
SOX12	0.8675	1.0639	0.8795	0.8383	0.9123	0.0513
LOC649639	0.8816	1.0560	0.8991	0.8126	0.9123	0.0514
LOC442519	0.8810	1.0855	0.9009	0.7820	0.9124	0.0633
PIP4K2A	0.8617	1.1224	0.9884	0.6770	0.9124	0.0948
TMEM155	0.8909	0.9573	1.0169	0.7845	0.9124	0.0498
CCDC25	0.9626	1.1620	0.8755	0.6496	0.9124	0.1062
VSX2	0.8717	0.9738	0.9663	0.8379	0.9124	0.0340
HOMEZ	0.7987	1.0891	0.9045	0.8574	0.9124	0.0627
SCFD1	0.7172	1.2586	1.0146	0.6593	0.9124	0.1392
CSAD	0.8285	1.0004	1.0127	0.8083	0.9125	0.0545
SPINK1	0.8713	0.9299	0.9653	0.8833	0.9125	0.0217
LOC649280	0.9554	1.0355	0.8015	0.8576	0.9125	0.0519
LOC728467	0.8561	1.0448	0.9706	0.7786	0.9125	0.0592
LOC728024	0.7353	1.0240	1.0491	0.8418	0.9125	0.0750
LOC1001339	0.8290	1.0403	0.9568	0.8240	0.9125	0.0525
COL1A1	0.8726	1.1346	0.8246	0.8185	0.9126	0.0750
LOC729983	0.7647	1.0142	0.9770	0.8943	0.9126	0.0553
LOC647983	0.8193	0.9418	1.0455	0.8437	0.9126	0.0516
MOGS	0.7444	1.0536	1.0233	0.8290	0.9126	0.0750
STRN4	0.9281	1.0564	0.8945	0.7714	0.9126	0.0586
C9orf43	0.7781	1.1575	0.8424	0.8727	0.9127	0.0839
TBX10	0.8579	0.9980	0.9796	0.8153	0.9127	0.0449
LOC651554	0.9258	0.9496	0.9533	0.8221	0.9127	0.0308
LOC1001292	0.9405	0.9312	0.8266	0.9524	0.9127	0.0290
LOC1001314	0.9153	0.9997	0.8639	0.8719	0.9127	0.0311
AMOTL2	0.9100	1.0602	0.8254	0.8553	0.9127	0.0522
C14orf85	0.6079	1.0267	1.2683	0.7480	0.9127	0.1471
MIR15B	0.8943	1.0470	0.9627	0.7469	0.9127	0.0635
POLE4	0.8609	1.2082	0.9213	0.6606	0.9128	0.1132
C21orf69	0.8188	1.1260	0.9277	0.7785	0.9128	0.0778
TMEM161B	0.7984	1.1096	0.9045	0.8386	0.9128	0.0692
POLN	0.7413	1.1146	0.9672	0.8281	0.9128	0.0818
SPAG7	0.8095	1.2387	0.8426	0.7604	0.9128	0.1099
LOC647349	0.6323	1.3456	1.0566	0.6170	0.9129	0.1766
TNFRSF12A	0.9068	1.1218	0.9085	0.7144	0.9129	0.0832
PPAP2A	0.7747	1.3077	0.9279	0.6412	0.9129	0.1441
LOC1001343	0.8565	1.0968	0.8805	0.8177	0.9129	0.0626
PEX5L	0.8019	0.9769	1.0481	0.8247	0.9129	0.0595
C2orf37	0.9149	0.9329	1.0297	0.7742	0.9129	0.0527

RNF133	0.8685	0.9677	0.9835	0.8320	0.9129	0.0371
LOC1001293	0.7834	1.0219	0.9113	0.9351	0.9130	0.0493
LOC642691	0.9935	0.8930	0.9935	0.7719	0.9130	0.0527
CYFIP1	0.8712	1.2270	0.8647	0.6890	0.9130	0.1128
PISD	0.8193	1.0970	0.9491	0.7865	0.9130	0.0707
ZNF566	0.7862	1.0849	0.9387	0.8421	0.9130	0.0654
LOC648829	0.8057	1.0170	0.9743	0.8549	0.9130	0.0496
PRPF6	0.7070	1.2687	0.9854	0.6908	0.9130	0.1365
SLFN12L	0.8671	0.9256	0.9495	0.9098	0.9130	0.0174
RGS12	0.8583	1.0440	0.9048	0.8449	0.9130	0.0455
COPS4	0.7581	1.0909	1.1444	0.6587	0.9130	0.1204
ZNF408	0.7321	1.3397	0.8772	0.7032	0.9131	0.1472
TTC30B	0.8684	1.0398	0.9558	0.7884	0.9131	0.0543
ZFX	0.9512	1.1703	0.8480	0.6829	0.9131	0.1020
METT10D	0.7588	1.1992	0.7835	0.9109	0.9131	0.1010
A2LD1	0.9162	0.9326	0.9414	0.8624	0.9131	0.0177
NAGK	0.9323	0.9635	0.9698	0.7871	0.9132	0.0428
ALOX12	0.8553	0.9839	1.0166	0.7969	0.9132	0.0521
C9orf110	0.9069	0.9474	0.9684	0.8302	0.9132	0.0305
PDXK	0.7813	1.2990	0.8465	0.7261	0.9132	0.1309
LOC390570	0.9749	0.9297	0.8628	0.8856	0.9132	0.0248
LOC643373	0.8260	1.1479	0.8127	0.8665	0.9133	0.0790
LMBR1L	0.7750	1.1434	0.9698	0.7649	0.9133	0.0901
LOC648533	0.7584	1.0082	1.0800	0.8065	0.9133	0.0776
SLC4A2	0.8888	1.1754	0.9060	0.6830	0.9133	0.1010
TMED9	0.8103	1.3771	0.8798	0.5859	0.9133	0.1668
LOC649071	0.8431	0.9570	0.9638	0.8893	0.9133	0.0288
LOC729164	0.7667	1.0243	0.9732	0.8890	0.9133	0.0563
SQLE	0.8366	1.1862	0.9744	0.6561	0.9133	0.1119
LOC646358	0.8296	1.1466	0.8953	0.7818	0.9133	0.0812
KLRA1	0.8639	1.0522	0.8896	0.8478	0.9134	0.0471
LOC730740	0.8253	1.1798	1.0919	0.5565	0.9134	0.1408
ZNF138	0.8382	1.0263	0.9325	0.8565	0.9134	0.0428
C1orf61	0.7684	0.9868	1.0562	0.8423	0.9134	0.0657
LOC653489	0.8001	1.0057	1.0219	0.8259	0.9134	0.0583
BDKRB2	0.8217	1.1155	0.8708	0.8457	0.9134	0.0681
LOC90120	0.6957	1.2714	0.7906	0.8962	0.9135	0.1262
TLE3	0.8172	1.1401	0.9348	0.7617	0.9135	0.0837
BCL2	0.8810	1.0117	0.9270	0.8341	0.9135	0.0378
CCT5	0.9049	1.0460	0.9115	0.7917	0.9135	0.0520
TBCC	0.7954	1.0724	0.9297	0.8566	0.9135	0.0597
LOC1001281	0.8878	0.9759	0.9044	0.8860	0.9135	0.0212
LOC1001313	0.7716	1.0468	0.8943	0.9414	0.9135	0.0570
KIAA1632	0.8569	1.0046	0.9215	0.8711	0.9136	0.0334
E2F6	0.8140	1.1047	0.9646	0.7709	0.9136	0.0761
CRYGB	0.8925	0.9626	0.9401	0.8591	0.9136	0.0233

DMRTB1	0.9253	0.9748	0.9499	0.8043	0.9136	0.0378
FUT8	0.8495	1.0367	1.0008	0.7673	0.9136	0.0634
PLOD2	0.9046	1.0698	0.8091	0.8709	0.9136	0.0557
LOC1001323	0.7177	1.0395	0.9821	0.9151	0.9136	0.0701
SEC23A	0.7196	1.2572	0.8640	0.8136	0.9136	0.1184
LOC730393	0.9353	0.8491	1.0451	0.8250	0.9136	0.0498
USPL1	0.8890	0.9597	0.9978	0.8080	0.9136	0.0418
LOC282997	0.8032	1.0920	0.9838	0.7756	0.9136	0.0753
ADORA1	0.8604	0.9950	0.9088	0.8905	0.9137	0.0289
SNORD84	0.8065	1.1373	0.8647	0.8462	0.9137	0.0755
ZNF302	0.9178	1.1584	0.9645	0.6140	0.9137	0.1127
GFPT2	0.8633	1.0140	0.8828	0.8946	0.9137	0.0341
NFYB	0.9270	0.9981	0.8696	0.8600	0.9137	0.0318
LOC731338	0.8760	0.9480	0.9685	0.8623	0.9137	0.0262
TAF2	0.7946	1.2658	0.8991	0.6953	0.9137	0.1245
CLPB	0.8521	1.0043	1.0590	0.7395	0.9137	0.0727
CHURC1	0.9041	1.1430	0.9216	0.6862	0.9137	0.0933
GRB14	0.8550	1.0136	1.0152	0.7711	0.9137	0.0606
LOC646762	0.8710	1.0159	0.9865	0.7816	0.9137	0.0540
LOC730396	0.9055	0.9714	0.8567	0.9214	0.9138	0.0236
C6orf211	0.8461	1.2184	0.9331	0.6575	0.9138	0.1167
LOC727848	0.8885	1.0524	0.8641	0.8501	0.9138	0.0469
IRX4	0.8780	1.2325	0.8225	0.7221	0.9138	0.1110
CITED4	0.7821	1.1136	1.0241	0.7356	0.9138	0.0918
MIR519A2	0.8578	1.0002	0.9268	0.8707	0.9138	0.0324
GPR101	0.8417	1.0494	0.9632	0.8011	0.9139	0.0568
IL6R	0.7643	1.0972	0.9518	0.8425	0.9139	0.0722
MORF4L2	0.6908	1.3641	0.9207	0.6802	0.9139	0.1600
HEATR5A	0.7349	1.2672	0.9030	0.7507	0.9139	0.1237
FARSLB	0.8120	1.3612	0.8264	0.6563	0.9140	0.1540
ALS2CR14	0.8281	0.9697	0.9801	0.8779	0.9140	0.0367
C2orf60	0.8636	1.0050	0.8799	0.9074	0.9140	0.0317
UBE2O	0.7775	1.1552	0.8893	0.8340	0.9140	0.0836
CCDC91	0.8449	1.0248	1.0108	0.7755	0.9140	0.0616
KIAA1671	0.7220	1.3401	0.9144	0.6795	0.9140	0.1509
RUFY1	0.8050	1.2514	0.8544	0.7454	0.9140	0.1146
KRTCAP2	0.7364	1.3398	0.8747	0.7053	0.9140	0.1466
LOC646049	0.8240	1.0639	0.9343	0.8340	0.9141	0.0558
WDR45	0.8322	1.0971	0.9514	0.7756	0.9141	0.0712
OR7A5	0.8224	1.0266	0.9683	0.8391	0.9141	0.0497
DKFZp686I15	0.8671	1.0152	0.8913	0.8828	0.9141	0.0341
LOC730012	0.8092	1.0228	0.9195	0.9050	0.9141	0.0437
LOC646301	0.8987	1.0520	0.8231	0.8827	0.9141	0.0487
ART4	0.8931	0.9524	0.9429	0.8682	0.9141	0.0201
CCDC75	0.9074	1.0562	0.9024	0.7907	0.9142	0.0545
PPIB	0.7386	0.9663	1.0354	0.9165	0.9142	0.0634

LOC644937	0.8433	0.9788	1.0692	0.7655	0.9142	0.0679
LOC650561	0.7682	1.0394	1.0188	0.8306	0.9142	0.0676
CDC73	0.8227	1.0576	1.0035	0.7731	0.9142	0.0688
SNAPC5	0.7877	1.0812	0.9968	0.7911	0.9142	0.0741
LOC649350	0.8890	1.0250	0.9147	0.8281	0.9142	0.0412
CHRNE	0.8008	1.0325	0.9828	0.8409	0.9142	0.0555
LRCH3	0.8336	1.1526	0.8786	0.7922	0.9143	0.0814
C20orf85	0.8437	0.9604	0.9234	0.9294	0.9143	0.0249
COG3	0.8478	1.1216	0.9095	0.7782	0.9143	0.0741
LOC728115	0.9389	0.9555	0.9536	0.8093	0.9143	0.0352
MLH3	0.8803	1.0721	0.8992	0.8056	0.9143	0.0563
RPS26	0.9352	1.0164	1.0381	0.6675	0.9143	0.0852
GIMAP4	0.9042	0.9581	1.0155	0.7795	0.9143	0.0503
DEFB128	0.8669	1.0286	0.9385	0.8233	0.9143	0.0449
ITGA2	0.8279	1.2397	0.8883	0.7014	0.9143	0.1152
ANKMY1	0.8774	1.0809	0.8477	0.8513	0.9143	0.0559
MANEAL	0.7972	1.0376	0.9470	0.8755	0.9143	0.0512
LOC648771	0.7260	1.2316	1.0005	0.6993	0.9143	0.1258
PARG	0.8475	1.0872	0.9065	0.8163	0.9144	0.0606
NUDT5	0.6983	1.2114	1.0711	0.6768	0.9144	0.1341
SS18	0.8901	1.0734	0.9687	0.7255	0.9144	0.0733
ANO8	0.8883	1.0846	0.8583	0.8266	0.9144	0.0581
LOC649404	0.8746	0.9269	0.9672	0.8892	0.9145	0.0207
CCDC41	0.8440	1.0827	0.9257	0.8055	0.9145	0.0614
SGMS2	0.8601	1.0004	0.9983	0.7993	0.9145	0.0505
SPRR2G	0.8142	1.1417	0.8133	0.8889	0.9145	0.0778
MGC42105	0.8756	0.9741	0.9861	0.8224	0.9146	0.0394
PARP10	0.9054	1.0618	0.9131	0.7780	0.9146	0.0580
C17orf56	0.7010	1.0994	0.9450	0.9129	0.9146	0.0820
LOC644993	0.8836	0.9192	0.9930	0.8625	0.9146	0.0287
TMEM105	0.8390	1.0723	0.9311	0.8160	0.9146	0.0581
MGC42157	0.9278	0.9948	0.9076	0.8283	0.9146	0.0343
LOC645835	0.7197	0.9012	1.0358	1.0017	0.9146	0.0710
DNAJA1	0.8944	1.2225	0.9065	0.6352	0.9146	0.1202
DGKH	0.7923	1.1518	0.9760	0.7385	0.9147	0.0940
C16orf52	0.8387	1.1494	0.9698	0.7007	0.9147	0.0956
KBTBD4	0.8834	1.1012	0.8940	0.7801	0.9147	0.0673
ARL13B	0.8637	1.0767	0.9293	0.7891	0.9147	0.0611
EIF3E	0.6719	1.3421	0.9650	0.6798	0.9147	0.1579
FBXL16	0.8722	1.0434	0.9609	0.7823	0.9147	0.0563
LOC645118	0.8376	1.0521	0.9549	0.8143	0.9147	0.0552
FZD8	1.0082	0.9993	0.8781	0.7734	0.9148	0.0557
ZNF699	0.8455	0.9731	0.9851	0.8554	0.9148	0.0373
KRTAP4-1	0.7134	1.0235	0.9647	0.9577	0.9148	0.0687
ZNF222	0.8674	1.0348	0.9174	0.8397	0.9148	0.0431
LOC643017	0.9081	0.8621	0.9604	0.9288	0.9148	0.0206

LOC643986	0.9222	0.9612	0.9584	0.8175	0.9148	0.0336
LOC646246	0.8678	0.9971	0.9887	0.8057	0.9148	0.0468
FGD6	0.8424	1.0591	0.9238	0.8341	0.9148	0.0522
LOC728979	0.8818	0.9818	0.9574	0.8384	0.9149	0.0332
APLN	0.9596	1.0085	0.8812	0.8102	0.9149	0.0436
LOC441487	0.8144	0.9603	1.0687	0.8161	0.9149	0.0616
LOC643224	0.7819	1.0337	1.0527	0.7912	0.9149	0.0742
ZNF684	0.7573	1.0501	1.0144	0.8378	0.9149	0.0701
LOC730081	0.8955	1.0057	1.0192	0.7393	0.9149	0.0648
LOC645342	0.9111	0.9748	0.8801	0.8937	0.9149	0.0210
ZNF200	0.8037	1.1652	0.9409	0.7500	0.9149	0.0926
ITSN2	0.8882	0.9931	0.9589	0.8197	0.9149	0.0385
LOC646444	0.8658	1.0104	0.9352	0.8485	0.9150	0.0369
C6orf122	0.8364	1.0270	0.9740	0.8225	0.9150	0.0506
CCDC18	0.7484	1.2079	0.9001	0.8034	0.9150	0.1026
PRR23A	0.8456	0.9503	0.9727	0.8913	0.9150	0.0288
LOC727967	0.7981	1.0721	0.9889	0.8010	0.9150	0.0688
PRKRIR	0.7159	1.2257	0.9800	0.7384	0.9150	0.1196
LOC643713	0.8883	0.9306	0.9645	0.8767	0.9150	0.0201
OAT	0.9108	1.1525	0.8691	0.7278	0.9151	0.0883
SH2D2A	0.7546	1.1131	0.9379	0.8546	0.9151	0.0759
LOC653420	0.8254	0.9921	0.9791	0.8636	0.9151	0.0416
UGT1A3	0.8705	1.1428	0.8645	0.7825	0.9151	0.0785
OR9I1	0.7850	0.9656	0.9603	0.9495	0.9151	0.0435
AZIN1	0.8163	1.1924	0.9413	0.7104	0.9151	0.1038
LOC387876	0.8949	0.9833	0.8639	0.9183	0.9151	0.0253
SNORA65	0.7690	1.1864	0.9424	0.7626	0.9151	0.0996
LOC644433	0.8502	0.9747	0.9059	0.9297	0.9151	0.0259
PKN2	0.7697	1.0902	0.9302	0.8703	0.9151	0.0671
RASSF5	0.8210	1.1587	0.9069	0.7740	0.9152	0.0857
MSC	0.8690	0.9576	0.9825	0.8517	0.9152	0.0323
ASCC3	0.8652	1.1128	1.0199	0.6628	0.9152	0.0984
SMR3A	0.8828	0.9851	0.9307	0.8621	0.9152	0.0274
SQRDL	0.6937	1.3004	0.9256	0.7411	0.9152	0.1378
ZNF532	0.8379	1.1466	0.9093	0.7669	0.9152	0.0824
C1orf162	0.9086	0.9968	0.8438	0.9118	0.9152	0.0314
ZNF250	0.8344	1.0021	1.0480	0.7765	0.9152	0.0651
ZNF483	0.7204	1.1494	0.9725	0.8187	0.9153	0.0937
LOC652294	0.9271	1.0722	0.8791	0.7827	0.9153	0.0603
TCHH	0.9870	0.8705	0.9322	0.8714	0.9153	0.0279
LOC1001343	0.7878	1.1174	0.9452	0.8107	0.9153	0.0758
DDIT3	0.9115	1.2525	0.7851	0.7121	0.9153	0.1197
DUSP11	0.8218	1.2293	0.8306	0.7796	0.9153	0.1052
LOC645937	1.0040	1.0265	0.9514	0.6795	0.9153	0.0802
LOC731170	0.7970	1.0505	0.9528	0.8612	0.9154	0.0552
TMEM207	0.8045	1.1449	0.9397	0.7725	0.9154	0.0847

CLNS1A	0.8045	1.3486	0.8681	0.6404	0.9154	0.1522
ZNF580	0.8943	1.0789	0.8603	0.8281	0.9154	0.0561
IRGM	0.8861	0.9491	0.9028	0.9237	0.9154	0.0136
DALRD3	0.8189	1.1399	0.9631	0.7398	0.9154	0.0880
IFFO1	0.8233	0.9760	0.9752	0.8872	0.9154	0.0371
FAIM3	0.8490	1.0510	0.9175	0.8444	0.9155	0.0482
LOC651617	0.8879	0.9713	0.9107	0.8920	0.9155	0.0192
LOH12CR2	0.8610	1.1197	0.9584	0.7228	0.9155	0.0835
HPSE	0.8130	1.0110	1.0178	0.8201	0.9155	0.0571
OBP2B	0.9447	0.8244	0.9524	0.9405	0.9155	0.0305
MARVELD1	0.9248	0.9116	0.9860	0.8397	0.9155	0.0300
ARFIP2	0.9073	1.0646	0.8428	0.8475	0.9155	0.0518
HOXA13	0.9395	0.9555	0.8801	0.8871	0.9156	0.0188
LOC205251	0.7578	1.3181	0.8713	0.7152	0.9156	0.1381
PSCD2	0.9397	1.1090	0.9031	0.7106	0.9156	0.0817
TBC1D24	0.8472	1.0262	0.9492	0.8398	0.9156	0.0445
MIF4GD	0.7828	1.2418	0.8889	0.7490	0.9156	0.1127
LOC651126	0.9002	1.0911	0.7905	0.8807	0.9156	0.0632
C17orf101	0.8616	1.1320	0.9047	0.7643	0.9157	0.0779
NAT12	0.8596	1.1661	0.9735	0.6635	0.9157	0.1052
ETV7	0.7397	1.0117	0.9455	0.9658	0.9157	0.0603
IL1RAP	0.8423	0.9951	0.9634	0.8619	0.9157	0.0375
SCGB1A1	0.8292	0.9888	0.9726	0.8722	0.9157	0.0387
C20orf141	0.8974	0.9776	0.9702	0.8175	0.9157	0.0374
UBE2MP1	0.9076	0.9192	0.9559	0.8801	0.9157	0.0157
ZNF692	1.0020	1.0969	0.8140	0.7499	0.9157	0.0807
CRTAM	0.9096	0.9235	0.9113	0.9186	0.9157	0.0032
NTF3	0.7841	1.0184	0.9416	0.9189	0.9157	0.0488
ETS1	0.7588	1.0810	1.1473	0.6760	0.9158	0.1166
AOX2P	0.8265	1.0136	0.9550	0.8681	0.9158	0.0422
C14orf65	0.8552	1.0080	0.9428	0.8571	0.9158	0.0369
ASGR1	0.9194	0.9632	0.8689	0.9116	0.9158	0.0193
LOC644783	0.8427	0.9573	1.0789	0.7844	0.9158	0.0652
SENP8	0.9154	1.0300	0.9402	0.7778	0.9158	0.0522
RILPL1	0.8425	1.1038	0.9654	0.7516	0.9158	0.0764
HIGD2A	0.7307	1.2529	1.0696	0.6101	0.9158	0.1486
UBA3	0.6596	1.3589	0.9217	0.7231	0.9158	0.1579
LOC1001314	0.9716	1.0195	0.8494	0.8230	0.9159	0.0473
PRSSL1	0.8503	0.9900	0.8950	0.9282	0.9159	0.0294
FAM86D	0.8574	0.9387	1.0840	0.7835	0.9159	0.0644
ZNF644	0.7499	1.2394	0.9338	0.7405	0.9159	0.1167
LOC347119	0.8833	0.9518	0.9847	0.8440	0.9159	0.0320
C7orf49	0.8140	1.0770	1.0475	0.7252	0.9159	0.0866
AGBL5	0.8447	1.1692	0.9323	0.7176	0.9159	0.0952
LOC388789	0.9612	0.9721	1.0672	0.6633	0.9160	0.0875
LOC653135	0.8438	1.0654	0.9210	0.8336	0.9160	0.0535

FCF1	0.7393	1.0894	1.0020	0.8331	0.9160	0.0793
CXorf40B	0.9120	1.0632	0.9810	0.7077	0.9160	0.0760
LOC1001315	0.9441	1.0516	0.8878	0.7805	0.9160	0.0565
ZFP3	0.8430	0.9734	0.9841	0.8635	0.9160	0.0365
IDH2	0.9090	1.1859	0.8938	0.6755	0.9160	0.1046
EHMT2	0.7926	1.0326	0.9760	0.8629	0.9160	0.0542
SDK2	0.8668	1.0551	0.8742	0.8680	0.9160	0.0464
IL23A	0.9130	1.1825	0.8442	0.7245	0.9160	0.0970
LOC1001325	0.8283	1.0430	0.8985	0.8944	0.9160	0.0453
DPP8	0.7997	1.2116	0.9006	0.7525	0.9161	0.1032
FAT2	0.9664	0.9475	1.0043	0.7461	0.9161	0.0579
LOC285501	0.9089	0.8464	0.9832	0.9259	0.9161	0.0281
LOC650680	0.9427	0.9847	0.9836	0.7533	0.9161	0.0551
MAPK1IP1L	0.8471	1.0955	0.9115	0.8103	0.9161	0.0634
SPAST	0.8716	1.0106	1.0954	0.6869	0.9161	0.0893
VPS26B	0.8415	1.0804	0.9740	0.7688	0.9162	0.0693
EN2	0.8176	1.0135	0.9372	0.8963	0.9162	0.0408
HMGN1	0.7551	1.2351	0.9465	0.7280	0.9162	0.1169
KIF5B	0.5280	1.4347	0.9992	0.7028	0.9162	0.1983
LOC731488	0.9191	0.9290	0.9807	0.8360	0.9162	0.0299
LOC641922	0.7283	1.1385	0.9064	0.8916	0.9162	0.0844
DHH	0.9046	1.0869	0.9394	0.7340	0.9162	0.0725
MAD2L1BP	0.7196	1.2780	0.9910	0.6764	0.9162	0.1392
TMC8	0.8375	1.0150	0.8717	0.9408	0.9162	0.0393
ZNF551	0.7743	0.9479	1.0712	0.8717	0.9163	0.0627
MRC1L1	0.8632	0.8885	1.0567	0.8567	0.9163	0.0473
MGA	0.8728	1.0157	0.9307	0.8461	0.9163	0.0375
GPHA2	0.8680	1.1147	0.9285	0.7540	0.9163	0.0754
LOC390760	0.7904	0.9535	1.0816	0.8398	0.9163	0.0648
C22orf25	0.7980	1.1621	0.9608	0.7445	0.9163	0.0940
LOC644684	0.7239	1.3101	0.8460	0.7854	0.9163	0.1336
LOC649887	0.9070	0.9817	0.8930	0.8836	0.9163	0.0223
LOC650475	0.8866	0.8897	0.9575	0.9316	0.9163	0.0171
LOC653736	0.9649	0.9413	0.8758	0.8836	0.9164	0.0218
ZNF441	0.8988	0.9522	0.9735	0.8409	0.9164	0.0297
PHC3	0.8130	1.0779	0.9553	0.8195	0.9164	0.0630
GPD1	0.9308	0.9081	0.9061	0.9207	0.9164	0.0058
TNFSF10	0.9100	1.0312	0.9408	0.7837	0.9164	0.0512
ZBTB3	0.7971	1.2282	0.8730	0.7677	0.9165	0.1062
IBTK	0.8393	1.0744	1.0363	0.7160	0.9165	0.0844
ATG5	0.8956	0.9532	0.9990	0.8182	0.9165	0.0390
DCHS1	0.8841	0.9419	0.9918	0.8483	0.9165	0.0316
C9orf117	0.8738	0.9675	0.9912	0.8336	0.9165	0.0375
LOC647353	0.8486	1.0385	0.8840	0.8950	0.9165	0.0419
TBPL1	0.6174	1.2416	1.1992	0.6078	0.9165	0.1757
SNORA41	0.8870	1.0438	0.9303	0.8052	0.9166	0.0497

EMP3	0.8797	1.0566	0.9779	0.7521	0.9166	0.0657
CC2D1A	0.9139	1.0889	0.8905	0.7730	0.9166	0.0652
LOC646302	0.7284	1.1278	0.9285	0.8817	0.9166	0.0823
LOC727756	0.9208	0.9256	1.0335	0.7865	0.9166	0.0506
FAM103A1	0.8112	1.0667	1.0542	0.7344	0.9166	0.0845
LOC650869	0.9337	1.0067	0.9244	0.8016	0.9166	0.0425
PTPRN	0.8950	1.0001	0.8922	0.8793	0.9166	0.0280
MFSD1	0.7521	1.3158	0.9592	0.6394	0.9166	0.1486
LOC1001338	0.7289	1.0727	0.9946	0.8704	0.9167	0.0752
LOC1001338	0.7668	0.9577	1.0540	0.8883	0.9167	0.0604
IL22RA2	0.8462	0.9790	0.9416	0.8999	0.9167	0.0285
MACF1	0.8670	1.1368	0.8217	0.8416	0.9167	0.0739
ALKBH8	0.7474	1.1567	0.8688	0.8941	0.9167	0.0861
ANAPC13	0.8165	1.1877	0.9502	0.7126	0.9168	0.1026
TCF12	0.8627	1.1602	0.9070	0.7371	0.9168	0.0888
NS3BP	0.8183	1.0065	0.9779	0.8644	0.9168	0.0449
LOC728889	0.7991	1.0373	1.0358	0.7949	0.9168	0.0692
DEM1	0.7668	1.1862	0.9051	0.8090	0.9168	0.0944
LOC651609	0.9562	0.9709	0.9339	0.8062	0.9168	0.0376
FUT4	0.9422	0.9990	0.9461	0.7799	0.9168	0.0474
NOC4L	0.8237	1.1286	0.9329	0.7820	0.9168	0.0774
ZNF236	0.8370	1.1123	0.8354	0.8827	0.9168	0.0661
NBEAL2	0.9573	1.1264	0.7454	0.8382	0.9168	0.0822
FAM164A	0.7515	1.2168	0.9309	0.7682	0.9169	0.1079
MAPKAPK2	0.8659	1.1522	0.8973	0.7522	0.9169	0.0844
SRP68	0.7873	1.4146	0.8192	0.6466	0.9169	0.1701
LOC1001322	0.9030	1.0116	0.8302	0.9231	0.9170	0.0373
LONRF1	0.8475	1.2691	0.8584	0.6928	0.9170	0.1233
LOC390660	0.8409	1.0378	0.8957	0.8935	0.9170	0.0422
NUDT7	0.9255	1.1375	0.8645	0.7406	0.9170	0.0829
LOC1001342	0.8740	0.9972	0.9889	0.8080	0.9170	0.0460
ACE2	0.8208	1.1165	0.9209	0.8100	0.9170	0.0710
CXorf38	0.6940	1.1582	0.9966	0.8194	0.9170	0.1016
LOC644677	0.7721	1.0817	0.9443	0.8702	0.9171	0.0652
ZNF771	0.8461	1.0000	0.9745	0.8477	0.9171	0.0408
SNORA77	0.8823	1.0902	0.9019	0.7941	0.9171	0.0623
LOC1001293	0.8365	1.0725	0.8919	0.8675	0.9171	0.0530
PKNOX1	0.7891	1.2580	0.8854	0.7361	0.9171	0.1177
MYOD1	0.8905	1.0300	0.8741	0.8740	0.9171	0.0378
WDR23	0.8548	1.1731	0.9187	0.7220	0.9172	0.0946
RFPL4A	0.9150	1.0813	0.8427	0.8296	0.9172	0.0579
HDLBP	0.7363	1.1848	0.8787	0.8688	0.9172	0.0949
NBPF3	0.8563	1.0751	0.9251	0.8122	0.9172	0.0575
RWDD4A	0.7835	1.1861	0.9535	0.7456	0.9172	0.1004
SFTPD	0.9399	0.9626	0.9665	0.7997	0.9172	0.0396
EHHADH	0.7709	1.0884	0.9010	0.9086	0.9172	0.0652

RGPD2	0.8635	0.9614	1.0355	0.8085	0.9172	0.0505
SERPINB5	0.8692	1.1281	0.9320	0.7396	0.9172	0.0809
HS6ST1	0.9653	1.0616	0.8825	0.7596	0.9172	0.0640
TAF1D	0.8576	1.0041	1.0012	0.8060	0.9172	0.0504
C11orf66	0.9013	0.9938	0.9847	0.7891	0.9172	0.0475
LOC283392	0.8954	1.0225	0.9167	0.8343	0.9172	0.0392
C16orf62	0.9321	1.0483	0.9228	0.7658	0.9172	0.0580
LOC388796	0.6340	1.4420	0.8108	0.7823	0.9173	0.1791
IFNAR2	0.7868	1.0819	0.9999	0.8004	0.9173	0.0734
OR7E24	0.9852	0.9190	0.9172	0.8477	0.9173	0.0281
ETV6	0.8993	1.0786	0.8571	0.8341	0.9173	0.0554
ARL17P1	0.7854	1.0457	0.9273	0.9108	0.9173	0.0533
TRUB2	0.8820	1.1901	0.8971	0.6999	0.9173	0.1014
ZNF28	0.8134	1.0785	0.9528	0.8246	0.9173	0.0623
LOC654200	0.7805	1.0755	0.9910	0.8222	0.9173	0.0696
LOC1001322	0.8322	0.9450	1.0572	0.8348	0.9173	0.0535
LRG1	0.8703	1.0086	0.8817	0.9086	0.9173	0.0315
LOC643582	0.8957	0.9927	1.0124	0.7685	0.9173	0.0558
ATP6V1A	0.8873	1.1134	0.9774	0.6913	0.9173	0.0885
MYST3	0.8127	1.0972	1.0274	0.7321	0.9174	0.0865
LOC653487	0.9859	0.8703	1.0694	0.7439	0.9174	0.0708
LOC644884	0.8167	1.0414	1.0182	0.7932	0.9174	0.0652
CLRN2	0.9181	0.9600	0.9388	0.8526	0.9174	0.0232
C9orf129	0.8845	0.9648	0.9098	0.9105	0.9174	0.0169
HSF1	0.7929	1.1024	0.8835	0.8907	0.9174	0.0656
LOC440309	0.8772	0.9678	0.8964	0.9281	0.9174	0.0198
ARHGDIG	0.8467	1.0452	0.9497	0.8279	0.9174	0.0503
LOC391692	1.0112	0.8570	0.9217	0.8797	0.9174	0.0340
C6orf26	0.9080	0.9548	0.8974	0.9095	0.9174	0.0127
KLF7	0.9713	0.8657	0.9029	0.9300	0.9174	0.0223
ITCH	0.7732	1.1436	0.9309	0.8221	0.9175	0.0823
NFATC3	0.8982	1.0435	0.8867	0.8415	0.9175	0.0438
LOC1001285	0.9893	0.8261	0.9385	0.9159	0.9175	0.0341
HOXD11	0.8929	1.0483	0.8625	0.8662	0.9175	0.0441
LOC1001329	1.0212	0.8838	0.9845	0.7803	0.9175	0.0542
EIF5	0.7506	1.2518	0.9100	0.7576	0.9175	0.1173
LOC1001343	0.9531	0.9717	0.9295	0.8157	0.9175	0.0350
FAM122C	0.7863	0.9866	0.8452	1.0520	0.9175	0.0615
LOC644224	0.8001	0.9919	0.9518	0.9263	0.9175	0.0414
POFUT2	0.8968	1.1676	0.8295	0.7762	0.9175	0.0869
LOC1001303	0.8289	1.0157	1.0006	0.8249	0.9175	0.0524
LOC643237	0.8013	0.9821	1.0349	0.8519	0.9175	0.0546
LOC1001316	0.8547	0.9703	0.8871	0.9582	0.9176	0.0279
PEX13	0.9126	1.0013	1.0873	0.6690	0.9176	0.0902
LOC1001299	0.9712	0.8795	0.9852	0.8343	0.9176	0.0363
LOC652286	0.8696	0.9779	0.9837	0.8392	0.9176	0.0370

ELOVL2	0.7430	1.1191	0.9073	0.9010	0.9176	0.0772
MMP21	0.9233	1.0268	0.8839	0.8364	0.9176	0.0405
WDR55	0.7388	1.2530	0.8855	0.7932	0.9176	0.1158
VPS8	0.8802	1.1313	0.8474	0.8116	0.9176	0.0726
DDX11	0.8734	1.0396	0.9337	0.8238	0.9176	0.0465
DDX43	1.0206	1.0927	0.8141	0.7432	0.9176	0.0829
CYP1B1	0.8880	0.8729	1.0314	0.8784	0.9177	0.0380
ZNF283	0.6976	1.1543	1.0236	0.7953	0.9177	0.1043
SLC6A5	0.9822	0.9018	0.8823	0.9045	0.9177	0.0221
LOC652476	0.9961	0.7917	1.0080	0.8750	0.9177	0.0516
RNF128	0.8382	1.1027	0.8484	0.8816	0.9177	0.0623
MCFD2	0.9732	1.0846	0.8906	0.7227	0.9178	0.0762
SLU7	0.8463	1.0885	1.0216	0.7147	0.9178	0.0848
LOC1001291	0.8666	1.0504	1.0220	0.7322	0.9178	0.0739
FLJ44124	0.7800	1.1482	1.0010	0.7421	0.9178	0.0957
PEBP1	0.8926	0.9855	1.0846	0.7086	0.9178	0.0800
PSMB2	0.6790	1.5529	0.8243	0.6152	0.9178	0.2162
LOC643324	0.8259	1.0693	0.9201	0.8561	0.9178	0.0542
LOC1001346	0.8952	1.0013	0.9305	0.8445	0.9178	0.0329
LOC646307	0.8916	0.9390	0.9804	0.8606	0.9179	0.0263
FMOD	0.8897	0.9506	0.9630	0.8683	0.9179	0.0230
ARMCX5	0.8347	1.0262	1.1354	0.6755	0.9179	0.1019
LOC645743	0.8166	1.1411	0.9141	0.7998	0.9179	0.0785
SERF1A	0.8891	1.1288	0.9264	0.7274	0.9179	0.0825
ZNF263	0.8669	1.2094	0.8924	0.7031	0.9180	0.1058
LOC728850	0.8806	1.0182	0.9725	0.8006	0.9180	0.0485
MSH2	0.7718	1.0092	0.9529	0.9382	0.9180	0.0511
RPH3AL	0.8235	1.0502	0.9821	0.8163	0.9180	0.0584
LOC1001328	0.9282	1.0333	0.8510	0.8599	0.9181	0.0421
LOC402382	0.8207	1.0038	0.9715	0.8765	0.9181	0.0423
LOC1001325	0.9132	1.0276	0.9488	0.7830	0.9181	0.0510
MAN1C1	0.9371	0.9875	0.9688	0.7792	0.9182	0.0475
DDR2	0.9170	0.9770	0.9281	0.8505	0.9182	0.0261
LOC729451	0.9182	0.9295	0.9139	0.9111	0.9182	0.0040
LOC1001285	0.8475	1.0638	0.9909	0.7705	0.9182	0.0666
LOC652185	0.9857	0.9810	0.9505	0.7555	0.9182	0.0548
RADIL	0.9844	1.0411	0.7875	0.8597	0.9182	0.0577
LOC649984	0.8573	1.0365	0.9366	0.8426	0.9182	0.0445
FNIP1	0.8044	1.1800	0.9315	0.7570	0.9182	0.0947
GYG1	0.6508	1.4057	1.0313	0.5851	0.9182	0.1899
BAD	0.8686	1.1676	0.9203	0.7164	0.9182	0.0937
LOC441454	0.7492	1.0177	1.0254	0.8808	0.9183	0.0654
ZNHIT6	0.7969	1.1543	1.0048	0.7170	0.9183	0.0993
LOC731159	0.9420	0.8248	0.9718	0.9345	0.9183	0.0322
DDB2	0.9181	0.9566	0.9716	0.8267	0.9183	0.0325
LOC440348	0.9008	1.0313	0.9264	0.8146	0.9183	0.0446

NPC1L1	0.8774	0.9436	0.9928	0.8594	0.9183	0.0307
LOC1001342	0.9008	0.9763	0.9615	0.8346	0.9183	0.0323
EGLN2	0.8856	1.1955	0.8576	0.7346	0.9183	0.0980
DNAL1	0.7898	1.1312	0.9700	0.7824	0.9184	0.0832
UBASH3B	0.8393	1.0242	0.9701	0.8400	0.9184	0.0468
AATK	0.7993	1.0913	0.9739	0.8092	0.9184	0.0702
LOC1001330	0.7581	1.0116	0.9556	0.9484	0.9184	0.0553
ARNT	0.8965	1.0424	0.9064	0.8284	0.9184	0.0448
LOC645246	0.9143	0.9453	0.9652	0.8489	0.9185	0.0254
DAO	0.7828	1.0949	0.9158	0.8804	0.9185	0.0652
USP26	0.9178	1.0027	0.9324	0.8210	0.9185	0.0374
LOC1001312	0.7687	0.9042	1.0107	0.9904	0.9185	0.0550
ADAM17	0.8374	1.1262	0.8243	0.8861	0.9185	0.0705
WDR6	0.8570	1.2447	0.9384	0.6340	0.9185	0.1264
LOC1001294	0.8071	1.0692	0.9379	0.8599	0.9185	0.0570
LOC1001328	0.8112	1.0351	0.9493	0.8786	0.9185	0.0480
INSM1	0.8592	1.0494	0.9666	0.7990	0.9185	0.0557
LOC401074	0.8362	1.2427	0.9147	0.6807	0.9185	0.1185
C21orf91	0.7008	1.0713	1.0204	0.8817	0.9185	0.0829
LOC647176	0.8147	1.0423	0.9509	0.8662	0.9186	0.0499
SUPT3H	0.8321	1.0937	0.9462	0.8023	0.9186	0.0661
IL18R1	0.8799	1.0164	0.9857	0.7923	0.9186	0.0512
TMEM106A	0.8221	1.0562	0.9113	0.8847	0.9186	0.0495
LOC642243	0.8000	1.0541	1.0394	0.7808	0.9186	0.0742
MGC70863	0.8472	1.0707	0.8871	0.8693	0.9186	0.0514
YLPM1	0.9299	0.9377	1.0455	0.7613	0.9186	0.0587
CCDC43	0.8012	1.0742	1.0184	0.7806	0.9186	0.0747
LOC646567	0.6214	1.4452	0.9028	0.7052	0.9187	0.1851
RTDR1	0.8314	0.9963	0.9632	0.8838	0.9187	0.0375
MIR580	0.8035	1.0890	0.9855	0.7967	0.9187	0.0717
LOC653664	0.9618	0.9362	1.0281	0.7487	0.9187	0.0599
PUS7L	0.7970	1.0552	0.9799	0.8428	0.9187	0.0598
FAM164C	0.8141	1.1448	0.9590	0.7570	0.9187	0.0865
LOC729952	0.8383	0.9547	0.9984	0.8836	0.9187	0.0358
ANKHD1	0.8853	1.1872	0.8895	0.7131	0.9188	0.0985
LOC647166	0.8526	0.9891	0.9528	0.8806	0.9188	0.0315
SNORD125	0.7198	1.0657	1.0251	0.8647	0.9188	0.0793
C1GALT1	0.7959	0.9076	1.0875	0.8845	0.9189	0.0611
ZMYND10	0.9159	1.0218	0.8722	0.8657	0.9189	0.0361
C14orf21	0.8600	0.9869	0.9709	0.8579	0.9189	0.0348
LOC647979	0.9253	0.9783	0.9815	0.7906	0.9189	0.0447
LOC1001336	0.8017	0.9678	0.9737	0.9326	0.9190	0.0401
NFKBIE	0.8879	1.0890	0.8143	0.8845	0.9190	0.0592
LOC400707	0.8305	1.0691	1.0144	0.7619	0.9190	0.0731
MIR631	0.9546	0.9572	0.8724	0.8917	0.9190	0.0217
RCADH5	0.8618	1.0270	0.9588	0.8284	0.9190	0.0454

LOC728475	0.8572	1.1609	0.8474	0.8106	0.9190	0.0812
HIST1H4L	0.8209	1.0969	0.8887	0.8698	0.9190	0.0610
ZNF75A	0.9568	0.9362	0.9888	0.7944	0.9190	0.0429
LDLRAD3	0.8887	0.9643	0.9411	0.8822	0.9191	0.0200
TDRD1	0.8415	0.9521	0.9555	0.9273	0.9191	0.0266
NOTCH2NL	0.8193	1.0899	0.9322	0.8349	0.9191	0.0622
TMEM82	0.8926	1.0289	0.9248	0.8300	0.9191	0.0416
C21orf56	0.8031	1.2191	0.8545	0.7997	0.9191	0.1008
EXD2	0.8669	1.1651	0.9416	0.7030	0.9191	0.0959
LOC651249	0.8958	0.9706	0.9302	0.8800	0.9191	0.0201
FLJ41841	0.9168	1.0264	0.8608	0.8726	0.9191	0.0377
LOC728070	0.9324	1.0194	0.9426	0.7822	0.9192	0.0496
LOC1001323	0.8385	1.0869	0.9958	0.7554	0.9192	0.0749
LOC1001328	0.8518	1.0054	0.9680	0.8514	0.9192	0.0397
EPGN	0.9107	0.8903	1.0225	0.8533	0.9192	0.0364
TAF13	0.6510	1.1892	1.0355	0.8011	0.9192	0.1198
SNORA33	0.8167	0.9810	0.9231	0.9560	0.9192	0.0362
LOC401098	0.7647	0.9743	1.2013	0.7365	0.9192	0.1079
LRIT3	0.9094	1.0621	0.8833	0.8220	0.9192	0.0510
ACOX2	0.7479	1.2250	0.9556	0.7484	0.9192	0.1130
C9orf75	0.6902	1.2909	0.9175	0.7783	0.9193	0.1324
LOC644756	0.8421	1.0320	0.9978	0.8051	0.9193	0.0562
PLCB2	0.8709	0.9318	0.9951	0.8792	0.9193	0.0286
RNF149	0.7354	1.3168	0.9615	0.6634	0.9193	0.1469
C17orf78	0.9339	0.9058	0.9722	0.8653	0.9193	0.0226
LOC1001335	0.8439	0.9838	0.9700	0.8797	0.9193	0.0341
FLI1	0.7275	1.1263	1.0632	0.7604	0.9193	0.1023
FNDC3A	0.8516	1.0161	0.9863	0.8234	0.9193	0.0480
APOC1	0.7855	1.0800	0.8735	0.9384	0.9194	0.0620
LOC648645	0.8950	0.9442	1.0048	0.8334	0.9194	0.0364
MYADM	0.7333	1.2621	0.9499	0.7322	0.9194	0.1252
MCTS1	0.8006	1.0268	1.1877	0.6624	0.9194	0.1168
TNXA	0.9333	1.1178	0.9214	0.7051	0.9194	0.0844
FLJ36848	0.8878	1.0951	0.8179	0.8767	0.9194	0.0606
C14orf64	0.9284	0.9310	0.9722	0.8461	0.9194	0.0264
NBPF22P	0.8259	1.0398	0.9345	0.8774	0.9194	0.0458
FAM190B	0.7505	1.1279	0.9368	0.8627	0.9194	0.0793
HSPG2	0.8561	0.9394	0.9724	0.9099	0.9194	0.0247
HIST1H2BF	0.6585	1.3067	0.9976	0.7150	0.9195	0.1489
STC1	0.8952	0.9987	0.9246	0.8594	0.9195	0.0296
LOC1001307	0.9282	0.7942	1.0375	0.9180	0.9195	0.0497
TOR2A	0.7667	1.0897	1.0144	0.8070	0.9195	0.0785
CYYR1	0.8370	0.9567	0.9514	0.9328	0.9195	0.0280
LOC729088	0.9145	0.9529	0.8738	0.9368	0.9195	0.0172
ZNF439	0.8829	0.9617	0.9251	0.9084	0.9195	0.0165
LOC1001329	0.8569	0.8848	0.9656	0.9708	0.9195	0.0287

CDK5R1	0.8926	1.0936	0.9261	0.7657	0.9195	0.0675
KIAA1285	0.8039	1.2238	0.8613	0.7891	0.9195	0.1026
LOC1001342	0.7571	1.0516	1.0514	0.8182	0.9196	0.0772
LOC729985	0.8429	0.9117	1.1036	0.8200	0.9196	0.0644
LOC1001336	0.8694	0.9295	0.7856	1.0938	0.9196	0.0651
LOC652541	0.7717	1.0747	0.9951	0.8369	0.9196	0.0698
C7orf59	0.7989	1.1330	0.9289	0.8177	0.9196	0.0767
SMAD7	0.8332	1.1199	0.9423	0.7831	0.9196	0.0746
LOC654209	0.8298	0.9588	0.9577	0.9322	0.9196	0.0306
NBPF6	0.8338	0.9835	0.9716	0.8896	0.9196	0.0354
LOC650549	0.9881	0.9840	0.8916	0.8148	0.9196	0.0414
LOC643191	0.8775	1.0873	0.8332	0.8806	0.9196	0.0569
LOC727773	0.8595	1.0454	0.9529	0.8208	0.9197	0.0503
LOC652740	0.9978	0.9060	0.9007	0.8741	0.9197	0.0270
LOC643451	0.9185	0.9958	0.9823	0.7820	0.9197	0.0489
LOC650187	0.8110	0.9758	0.9726	0.9193	0.9197	0.0385
CRIPT	0.7572	1.3135	0.9202	0.6878	0.9197	0.1400
ZNF845	0.8133	1.2133	0.8953	0.7569	0.9197	0.1019
IQCF2	0.9723	0.9992	0.8946	0.8128	0.9197	0.0420
LOC650656	0.8674	1.0155	0.9568	0.8392	0.9197	0.0406
NCF2	0.8339	1.0055	0.9469	0.8927	0.9197	0.0367
C6orf218	0.8244	1.0227	0.8798	0.9523	0.9198	0.0431
KIAA0556	1.0306	1.1431	0.8014	0.7041	0.9198	0.1011
LOC641724	0.8276	0.9991	0.9846	0.8678	0.9198	0.0425
MIR32	0.8137	1.0800	0.9384	0.8471	0.9198	0.0596
ABI3	0.8213	1.1388	0.9370	0.7822	0.9198	0.0801
NAT9	0.7959	1.3292	0.8473	0.7070	0.9199	0.1395
LOC554235	0.9153	1.0398	0.8179	0.9065	0.9199	0.0456
ZNF77	0.7931	1.1615	0.9679	0.7570	0.9199	0.0928
MTM	0.8580	1.1853	0.9009	0.7353	0.9199	0.0952
HEATR7A	0.8796	1.0253	0.9311	0.8436	0.9199	0.0395
LOC645447	0.8864	1.0308	0.9480	0.8144	0.9199	0.0460
C14orf179	0.8434	1.2936	0.8886	0.6540	0.9199	0.1345
MLLT10	0.9223	1.1456	0.8393	0.7726	0.9200	0.0812
CDH16	0.9150	1.0321	0.8523	0.8804	0.9200	0.0395
MIR634	0.8642	1.0367	0.9217	0.8573	0.9200	0.0415
LOC646590	0.7857	0.9908	0.9721	0.9313	0.9200	0.0465
ZNF567	0.7604	1.2022	0.9389	0.7784	0.9200	0.1023
ZNF586	0.8659	1.1913	0.8796	0.7432	0.9200	0.0955
MOSPD1	0.8731	0.9101	0.9993	0.8976	0.9200	0.0275
ZNF772	0.8542	1.1082	1.0552	0.6625	0.9200	0.1018
LOC441506	0.6983	1.2162	1.1768	0.5887	0.9200	0.1614
ALKBH4	0.8628	1.1390	0.8164	0.8619	0.9200	0.0738
LOC1001340	0.9235	0.9685	0.9353	0.8529	0.9200	0.0243
TUBB2A	0.7980	1.1516	1.0454	0.6851	0.9200	0.1078
LOC93432	0.8334	1.0197	0.9578	0.8692	0.9200	0.0423

LOC653410	0.9483	0.8224	0.9836	0.9259	0.9201	0.0346
TTTY10	0.8779	0.9483	0.9387	0.9153	0.9201	0.0157
ZNF717	0.8614	1.0950	1.0708	0.6530	0.9201	0.1033
FAM71C	0.9493	0.9627	0.9973	0.7709	0.9201	0.0507
RBP7	0.7812	1.0340	1.0684	0.7968	0.9201	0.0761
SLC6A11	0.9860	0.9418	0.8948	0.8578	0.9201	0.0279
MED17	0.8898	1.0412	0.9190	0.8304	0.9201	0.0444
SRRM1L	0.8319	1.2478	0.8451	0.7555	0.9201	0.1110
MGC16384	0.8126	1.1168	0.8858	0.8653	0.9201	0.0674
ZNF768	0.8537	1.3247	0.8158	0.6864	0.9201	0.1395
LOC401387	0.8642	0.9797	0.9696	0.8671	0.9201	0.0315
LOC653094	0.9695	0.9456	0.9821	0.7834	0.9202	0.0462
MYL7	0.8365	1.0111	0.9605	0.8726	0.9202	0.0399
OSGIN1	0.8888	0.9904	0.9493	0.8522	0.9202	0.0308
LOC1001330	0.9457	0.9291	0.9343	0.8716	0.9202	0.0166
KDM5A	0.8941	0.9225	0.9901	0.8740	0.9202	0.0253
ZNF592	0.8468	1.0755	0.9614	0.7972	0.9202	0.0621
LRRC7	0.8573	1.0149	0.9894	0.8193	0.9202	0.0482
CIB2	0.9009	0.9858	0.9512	0.8431	0.9202	0.0311
LOC1001329	0.9384	1.0317	0.9620	0.7489	0.9202	0.0605
LOC1001284	0.9086	0.9818	1.0169	0.7737	0.9202	0.0538
LOC648974	0.8514	1.1061	0.9642	0.7594	0.9203	0.0748
MIR30B	0.8512	0.9932	1.0164	0.8203	0.9203	0.0494
LOC392459	0.8545	1.0408	0.9450	0.8409	0.9203	0.0463
LOC441436	0.8593	1.0961	0.8330	0.8929	0.9203	0.0599
LOC644538	0.9550	0.9833	0.9601	0.7830	0.9203	0.0462
LOC1001316	0.8617	0.9434	0.9454	0.9308	0.9203	0.0198
LOC643236	0.8829	0.9778	0.8636	0.9574	0.9204	0.0278
POLB	0.7807	1.2277	0.9805	0.6929	0.9204	0.1188
ZMYM3	0.9095	1.0671	0.8825	0.8226	0.9204	0.0521
MIR215	0.8626	0.9717	0.9995	0.8479	0.9204	0.0382
LOC649987	0.8474	1.0245	0.9123	0.8976	0.9204	0.0374
GABRE	0.8933	1.0187	0.8455	0.9242	0.9204	0.0365
RNU11	0.8849	1.1259	0.9273	0.7437	0.9205	0.0789
TMEM30A	0.9048	1.0263	0.9613	0.7894	0.9205	0.0502
DTD1	0.8590	1.3617	0.7650	0.6962	0.9205	0.1508
ATF1	0.9698	0.9785	0.9672	0.7663	0.9205	0.0514
LOC648972	0.7994	1.0170	0.9649	0.9006	0.9205	0.0468
NKD1	0.8849	0.9799	1.0503	0.7669	0.9205	0.0614
LOC653434	0.8255	1.1056	0.9300	0.8209	0.9205	0.0666
ACTR10	0.8447	1.1494	0.9370	0.7509	0.9205	0.0852
LOC401703	0.9375	0.9330	1.0525	0.7590	0.9205	0.0605
ACIN1	0.7818	1.2064	0.8990	0.7948	0.9205	0.0988
JMJD4	0.8633	1.1817	0.9126	0.7246	0.9205	0.0957
LOC646492	0.7018	1.1212	0.9498	0.9093	0.9205	0.0862
LOC649921	0.9115	0.9758	0.9338	0.8611	0.9206	0.0239

HSCB	0.8107	1.2125	0.8983	0.7607	0.9206	0.1014
THADA	0.8451	1.1496	0.9012	0.7865	0.9206	0.0798
HIST1H1E	0.8262	0.9786	0.9877	0.8900	0.9206	0.0384
RFNG	0.8633	1.0999	1.0077	0.7115	0.9206	0.0850
WHDC1L2	0.8926	1.0506	0.9082	0.8310	0.9206	0.0464
MIR1908	0.9067	0.9988	0.9118	0.8653	0.9207	0.0281
DKFZp779M(	0.8558	1.0530	0.9445	0.8294	0.9207	0.0505
DAPK2	0.9085	1.0672	0.9124	0.7948	0.9207	0.0559
FANCL	0.8173	1.1291	0.8945	0.8419	0.9207	0.0713
LOC654127	0.8022	0.9781	0.9845	0.9180	0.9207	0.0423
SPEG	0.9219	1.1202	0.8320	0.8088	0.9207	0.0708
LOC652627	0.7657	0.9930	0.9521	0.9721	0.9207	0.0523
C6orf61	0.7816	1.2062	0.8742	0.8210	0.9207	0.0970
NOTO	0.8535	0.9644	0.9956	0.8695	0.9208	0.0349
XKRX	0.8251	1.0692	0.9003	0.8885	0.9208	0.0522
ZNF121	0.8050	1.0425	1.0240	0.8116	0.9208	0.0651
LOC644734	0.8767	1.0113	0.9846	0.8105	0.9208	0.0469
GPD1L	0.7095	1.2648	0.9698	0.7390	0.9208	0.1286
LOC392215	0.8691	1.0175	0.9408	0.8557	0.9208	0.0373
SLC9A5	0.8717	1.0326	0.9824	0.7965	0.9208	0.0533
LOC1001287	0.7677	1.1010	0.9962	0.8184	0.9208	0.0775
OR2T34	0.8470	0.9736	0.9489	0.9139	0.9208	0.0275
MMP10	0.8183	1.0243	0.9950	0.8458	0.9209	0.0519
LOC644132	0.7698	1.2846	0.9137	0.7153	0.9209	0.1283
GFRA3	0.9561	0.9159	0.9180	0.8935	0.9209	0.0130
LOC652674	0.6863	0.9701	1.0000	1.0273	0.9209	0.0791
PPIAL4C	0.8395	1.0241	1.0129	0.8072	0.9209	0.0568
ANKRD13C	0.7183	1.0791	1.2123	0.6742	0.9210	0.1329
LOC388681	0.9148	0.9169	1.0413	0.8109	0.9210	0.0471
ATAD3C	0.8901	0.9238	1.0142	0.8557	0.9210	0.0340
DIS3L2	0.8473	1.1187	0.8754	0.8424	0.9210	0.0663
IRF9	0.8260	1.2762	0.8167	0.7651	0.9210	0.1192
SNORA4	0.8446	0.9746	1.0356	0.8293	0.9210	0.0502
SETD2	0.7968	1.2377	0.8877	0.7619	0.9210	0.1088
FAM167B	0.8834	1.0604	0.8370	0.9033	0.9210	0.0485
KRT78	0.9049	1.0599	0.8916	0.8279	0.9211	0.0492
LOC650185	0.9051	0.9723	0.9898	0.8172	0.9211	0.0392
LOC1000085	0.2929	1.1645	1.6061	0.6208	0.9211	0.2906
LOC650689	0.8826	0.9980	0.9898	0.8140	0.9211	0.0443
ZNF286A	0.8338	1.0964	0.8933	0.8608	0.9211	0.0597
NEUROG2	0.8253	0.9200	0.9664	0.9730	0.9211	0.0341
LOC729993	0.8507	1.0800	0.9966	0.7575	0.9212	0.0723
LOC642688	0.9096	0.9177	1.0346	0.8229	0.9212	0.0435
ACAP2	0.7575	1.2698	0.9365	0.7209	0.9212	0.1254
TUSC3	0.8470	1.1171	0.9060	0.8147	0.9212	0.0680
FLJ35409	0.8415	1.0102	0.9011	0.9321	0.9212	0.0351

ZIM2	0.8987	1.0393	0.9162	0.8306	0.9212	0.0435
PAIP2B	0.8540	1.0765	0.7791	0.9752	0.9212	0.0657
SGMS1	0.9400	1.2226	0.8066	0.7157	0.9212	0.1105
AP3B1	0.7777	1.1787	0.9438	0.7848	0.9213	0.0940
LOC642861	0.8824	1.0035	1.0001	0.7993	0.9213	0.0495
HUNK	0.8526	1.0380	0.9271	0.8676	0.9213	0.0421
KIAA1618	0.8404	1.1168	0.9577	0.7704	0.9213	0.0758
PODN	0.8452	1.0011	0.9500	0.8891	0.9214	0.0342
LOC344405	0.9118	1.0138	0.9359	0.8241	0.9214	0.0391
OTUB1	0.8591	1.1090	0.9000	0.8175	0.9214	0.0648
LOC731196	0.8666	1.0639	1.0093	0.7459	0.9214	0.0718
ZNF323	0.8767	0.9955	1.0125	0.8009	0.9214	0.0503
PTK7	0.8694	1.0619	0.9232	0.8311	0.9214	0.0505
CSAG1	0.8305	1.2932	0.8307	0.7313	0.9214	0.1261
NDUFS7	0.6818	1.2772	0.9406	0.7863	0.9214	0.1299
GBAP	0.8597	1.0201	0.9100	0.8961	0.9215	0.0345
TADA2A	0.8843	1.0126	0.9632	0.8257	0.9215	0.0414
CBX8	0.8937	1.1759	0.8215	0.7947	0.9215	0.0874
CFD	0.7358	1.1719	1.0343	0.7438	0.9215	0.1086
CLK2	0.8206	1.1892	0.9557	0.7203	0.9215	0.1015
OR7G2	0.9440	0.9863	0.9328	0.8228	0.9215	0.0349
ZC3H5	0.8474	1.2514	0.8365	0.7507	0.9215	0.1121
TTC30A	0.8671	1.2007	0.9138	0.7044	0.9215	0.1033
LOC643590	0.9275	0.9682	0.9335	0.8569	0.9215	0.0233
PDE4A	0.9702	1.0419	0.8637	0.8102	0.9215	0.0521
SCNM1	0.7200	1.3085	0.9355	0.7221	0.9215	0.1385
LOC730919	0.9748	0.9311	1.0000	0.7804	0.9215	0.0492
C5orf44	0.7046	1.2879	1.0464	0.6473	0.9216	0.1506
TRIM23	0.8006	1.1878	0.9099	0.7879	0.9216	0.0929
IFT122	0.8530	1.0390	0.9613	0.8330	0.9216	0.0482
C2CD4C	0.7844	1.0874	1.0057	0.8088	0.9216	0.0742
LOC1001286	0.8328	1.1107	0.9839	0.7590	0.9216	0.0785
NMS	0.9825	0.8543	0.9726	0.8770	0.9216	0.0327
LOC728627	0.9176	1.0158	0.9683	0.7847	0.9216	0.0498
ZNF747	0.9252	1.0685	0.9658	0.7270	0.9216	0.0715
ANRIL	0.9446	1.1646	0.8183	0.7590	0.9216	0.0898
HAUS5	0.8678	0.9758	0.9802	0.8628	0.9216	0.0326
CCDC112	0.8834	1.1376	0.8859	0.7799	0.9217	0.0761
KIR3DL3	0.9684	0.9599	0.9273	0.8312	0.9217	0.0314
LOC642154	0.9139	1.0682	0.8764	0.8285	0.9217	0.0518
LOC1001340	0.7995	0.9524	1.0279	0.9073	0.9218	0.0477
SNORA16A	0.7718	1.0150	1.0690	0.8312	0.9218	0.0713
LOC654159	0.9628	0.9714	0.9209	0.8321	0.9218	0.0319
CARTPT	0.8973	1.1067	0.9494	0.7337	0.9218	0.0769
MYOZ3	0.9244	0.8207	0.9796	0.9625	0.9218	0.0356
SLC12A7	0.8186	1.0027	0.9310	0.9350	0.9218	0.0381

TMEM137	0.7040	1.1316	0.9957	0.8560	0.9218	0.0919
C1orf189	0.8907	1.0123	0.9223	0.8622	0.9219	0.0325
LOC652776	0.9636	0.9340	0.8690	0.9208	0.9219	0.0198
LOC643159	0.8511	1.0198	0.9139	0.9027	0.9219	0.0354
TRAF7	0.8221	1.0867	0.9495	0.8292	0.9219	0.0622
ISOC1	0.6617	1.1662	1.1601	0.6996	0.9219	0.1395
RASSF1	0.8096	1.0936	0.9597	0.8246	0.9219	0.0665
LOC645586	0.7995	1.1548	0.8778	0.8555	0.9219	0.0793
LOC651553	0.9200	1.1123	0.8286	0.8267	0.9219	0.0671
FTL	0.7699	1.2917	0.9378	0.6882	0.9219	0.1338
LOC1001343	0.8672	1.0361	0.9283	0.8561	0.9219	0.0412
LAMP1	0.9139	1.2576	0.8824	0.6337	0.9219	0.1283
HTATIP2	0.8294	1.0744	0.8800	0.9038	0.9219	0.0531
C11orf46	0.7472	1.0546	1.1620	0.7239	0.9219	0.1099
RGPD3	0.9591	1.0072	0.9638	0.7576	0.9219	0.0558
NPHP1	0.8610	1.0475	0.9926	0.7866	0.9219	0.0597
LOC1001290	1.0478	1.1058	0.8084	0.7257	0.9219	0.0918
NRN1	0.8578	1.0932	0.9120	0.8251	0.9220	0.0598
GAPDHL6	0.8396	1.1259	0.8572	0.8653	0.9220	0.0682
LOC728477	0.9438	1.0591	0.7924	0.8927	0.9220	0.0555
BCAR3	0.8408	1.2459	0.8844	0.7169	0.9220	0.1136
KIAA1881	0.9128	0.9910	0.8721	0.9122	0.9220	0.0249
LOC728752	0.8540	1.0026	0.9995	0.8320	0.9220	0.0459
ERCC3	0.8450	1.1740	0.8402	0.8289	0.9220	0.0840
G6PC3	0.8299	1.2706	0.9300	0.6577	0.9220	0.1291
ULK2	0.8494	0.9803	1.0065	0.8520	0.9221	0.0415
OR4C15	0.9163	1.0146	0.9575	0.7998	0.9221	0.0455
FAM92A1	0.7477	0.9870	1.0265	0.9270	0.9221	0.0616
LOC642369	0.9408	1.0523	0.8019	0.8933	0.9221	0.0521
LOC731551	0.8863	0.9933	0.9390	0.8697	0.9221	0.0280
LOC652854	0.9104	0.9586	0.9158	0.9035	0.9221	0.0124
ZNF681	0.8763	0.9926	0.9531	0.8663	0.9221	0.0305
C10orf31	0.8035	1.0901	0.9270	0.8678	0.9221	0.0614
LOC1001336	0.8695	1.1167	0.9305	0.7717	0.9221	0.0727
LOC646674	0.7263	1.2261	0.9248	0.8112	0.9221	0.1092
TFAP2D	0.8758	1.0232	0.9402	0.8493	0.9221	0.0387
MGC40499	0.8067	1.0956	0.9110	0.8754	0.9222	0.0617
MYH4	0.9229	0.9902	0.9755	0.8000	0.9222	0.0432
VPS26	0.8125	0.9603	1.0321	0.8838	0.9222	0.0475
LOC1001316	0.9851	0.9523	0.8406	0.9107	0.9222	0.0312
AGBL3	0.8912	0.8802	0.9464	0.9710	0.9222	0.0218
ELAVL2	0.9398	0.9115	0.8994	0.9381	0.9222	0.0100
LOC647297	0.7974	0.9929	0.9487	0.9498	0.9222	0.0429
LOC730746	0.8065	1.1091	0.9139	0.8594	0.9222	0.0661
C10orf95	0.9461	0.9796	0.9223	0.8409	0.9222	0.0296
CLP1	0.7482	1.2828	0.9587	0.6994	0.9222	0.1327

FAM58B	0.8340	0.9196	1.0440	0.8913	0.9222	0.0443
LOC642112	0.8627	1.0297	0.9563	0.8404	0.9222	0.0437
LOC651296	1.0096	0.9087	0.9536	0.8172	0.9223	0.0406
TIGD4	0.7375	1.0621	0.9726	0.9169	0.9223	0.0685
FLJ13305	0.8705	0.9949	0.9800	0.8438	0.9223	0.0381
FAM71F2	0.8266	1.0743	1.0134	0.7749	0.9223	0.0720
CDK5R2	0.8547	1.0520	1.0025	0.7800	0.9223	0.0633
SLC1A4	0.8877	1.1120	0.8136	0.8758	0.9223	0.0653
LOC389722	0.8579	0.9000	0.9712	0.9601	0.9223	0.0266
COPS2	0.7238	1.1396	1.0169	0.8090	0.9223	0.0950
FLJ44342	0.7984	1.0528	0.9931	0.8451	0.9224	0.0601
SNORA5A	0.7693	1.0300	1.0361	0.8541	0.9224	0.0662
LOC388076	0.8059	0.9480	1.3689	0.5668	0.9224	0.1683
LRRC37B2	0.7899	1.3645	0.8528	0.6824	0.9224	0.1515
FERMT2	0.7817	1.2697	0.9441	0.6942	0.9224	0.1268
OSTCL	0.6955	1.1257	1.0222	0.8463	0.9224	0.0951
MYLPF	0.6711	1.1390	1.0035	0.8761	0.9224	0.0995
LOC642879	0.9288	0.9808	0.9523	0.8279	0.9224	0.0333
LOC649095	0.8046	1.1363	1.0254	0.7234	0.9225	0.0957
CSN1S2B	0.8809	1.0582	0.8782	0.8724	0.9225	0.0453
LOC1001318	0.7992	1.1088	1.0414	0.7404	0.9225	0.0900
SNORA11B	0.9974	0.9941	0.8280	0.8704	0.9225	0.0432
CLEC4F	0.9403	1.0588	0.9031	0.7876	0.9225	0.0559
HIPK3	0.8683	0.9952	0.9608	0.8656	0.9225	0.0328
LOC653533	0.8792	1.0226	0.9281	0.8600	0.9225	0.0363
OSBPL6	0.8777	1.0041	0.9527	0.8555	0.9225	0.0342
ECT2L	0.7528	1.1564	0.9272	0.8536	0.9225	0.0858
FCHO2	0.8515	0.9801	1.0113	0.8470	0.9225	0.0428
TBC1D8B	0.9201	1.0160	0.9269	0.8271	0.9225	0.0386
LOC649458	0.8922	0.9441	0.9766	0.8772	0.9225	0.0230
H1FNT	0.8169	0.9333	1.0939	0.8460	0.9225	0.0622
MRS2P2	0.8942	1.0500	1.0117	0.7344	0.9225	0.0709
MYOG	0.8823	1.0304	1.0198	0.7577	0.9225	0.0645
LOC653711	0.8765	0.9971	0.9671	0.8495	0.9225	0.0354
SLC6A19	0.9095	1.0181	0.9765	0.7862	0.9226	0.0507
LOC648066	0.8248	1.0349	0.9233	0.9072	0.9226	0.0432
LOC652588	0.8514	0.8948	1.0716	0.8725	0.9226	0.0505
LOC441907	0.8182	1.0482	1.0373	0.7866	0.9226	0.0697
LOC440341	0.9381	1.1335	0.8101	0.8087	0.9226	0.0766
FTHL17	0.8704	1.0809	0.9779	0.7612	0.9226	0.0689
LOC645843	0.9625	0.9792	0.9504	0.7982	0.9226	0.0419
PRKCI	0.8653	0.9156	1.1543	0.7552	0.9226	0.0842
PFDN4	0.9290	0.9342	1.0136	0.8136	0.9226	0.0412
LOC642561	0.9103	1.0641	0.9503	0.7657	0.9226	0.0616
LOC257054	0.8376	0.9756	0.9636	0.9137	0.9226	0.0314
ZNF828	0.8191	1.3372	0.8583	0.6759	0.9226	0.1436

LOC652335	0.9494	0.9165	0.9809	0.8437	0.9226	0.0294
DVL1	0.9099	1.0666	0.9061	0.8079	0.9226	0.0535
MTMR2	0.9221	1.1655	0.8653	0.7378	0.9227	0.0897
LOC646686	0.9392	0.8765	0.9579	0.9170	0.9227	0.0175
LOC644345	0.8542	1.0983	0.8398	0.8984	0.9227	0.0598
LOC647471	0.8544	1.0676	0.9501	0.8186	0.9227	0.0557
ABCC5	0.9004	1.1266	0.9057	0.7580	0.9227	0.0761
CH25H	0.8568	0.9527	1.0016	0.8796	0.9227	0.0333
ATPBD1B	0.8049	1.1752	0.9676	0.7431	0.9227	0.0966
MTUS1	0.8708	1.0868	0.8924	0.8408	0.9227	0.0557
CCDC78	0.8490	1.0436	0.9119	0.8865	0.9227	0.0423
RHEBL1	0.8827	1.0148	0.8933	0.9000	0.9227	0.0309
LOC647543	1.0377	0.9793	0.8236	0.8507	0.9228	0.0512
LOC650152	0.7911	1.1901	1.0442	0.6658	0.9228	0.1189
ECM2	0.8821	0.9669	0.9465	0.8957	0.9228	0.0202
CIAO1	0.7135	1.3557	0.9687	0.6534	0.9228	0.1597
LOXL3	0.8244	1.0483	0.9482	0.8704	0.9228	0.0490
LOC391044	0.8507	1.0849	0.8764	0.8795	0.9229	0.0544
LOC1001289	0.8703	1.0360	0.9899	0.7954	0.9229	0.0550
LOC1001319	0.9064	1.0167	0.8721	0.8964	0.9229	0.0321
GPATC4	0.7625	1.0625	0.8934	0.9732	0.9229	0.0636
C19orf30	0.7561	1.0981	1.0148	0.8227	0.9229	0.0801
LOC647363	0.7718	1.1344	1.0543	0.7312	0.9229	0.1007
LOC654340	0.9105	1.0169	0.9232	0.8412	0.9229	0.0361
PIP4K2C	0.9360	1.3144	0.7839	0.6576	0.9230	0.1423
LOC642321	0.8694	1.0346	0.9121	0.8759	0.9230	0.0384
FAM13A	0.8698	1.1850	0.9084	0.7288	0.9230	0.0955
GPR15	0.8881	1.0006	0.8728	0.9304	0.9230	0.0286
HSP90AB6P	0.8659	0.9400	0.9890	0.8971	0.9230	0.0267
IL20	0.8625	0.9951	0.9288	0.9056	0.9230	0.0277
LRRC31	0.8601	1.1046	0.9064	0.8209	0.9230	0.0630
LOC644350	0.8039	1.0146	1.0165	0.8572	0.9230	0.0545
LOC644799	0.8399	1.2580	0.9627	0.6315	0.9230	0.1309
NCRNA0015	0.8238	0.9785	1.0125	0.8773	0.9231	0.0438
ROBLD3	0.7690	1.3985	0.8077	0.7170	0.9231	0.1596
PHC2	0.8992	1.1005	0.8847	0.8078	0.9231	0.0624
SNORA28	0.8288	1.0575	1.0332	0.7729	0.9231	0.0717
LOC653563	0.9003	1.0538	0.9228	0.8156	0.9231	0.0493
GLB1L3	0.9188	0.9963	0.9046	0.8728	0.9231	0.0262
BZW1	0.8933	1.0070	0.9592	0.8330	0.9231	0.0380
NMUR1	0.8664	0.9535	0.9667	0.9059	0.9231	0.0230
ITIH5	0.7248	1.3217	0.9300	0.7160	0.9231	0.1418
LOC1001330	0.8242	1.0489	1.0105	0.8091	0.9232	0.0621
LOC283849	0.9543	0.9894	0.8874	0.8617	0.9232	0.0295
LOC400879	0.7180	1.2330	1.0106	0.7312	0.9232	0.1233
LOC653092	0.8823	1.0629	0.8775	0.8701	0.9232	0.0466

RNF112	0.7640	1.1728	0.9344	0.8216	0.9232	0.0904
F11	0.7073	1.0345	1.0613	0.8898	0.9232	0.0812
LOC653113	0.9269	1.0216	0.9568	0.7875	0.9232	0.0494
DKFZp434M1	0.9232	0.9932	0.8874	0.8891	0.9232	0.0247
TMEM76	0.8648	1.0611	0.9965	0.7706	0.9232	0.0652
PC	0.8848	1.0947	0.9322	0.7813	0.9233	0.0653
SLC9A6	0.8645	1.0994	0.9066	0.8227	0.9233	0.0612
CALR	0.8430	1.1682	0.9396	0.7423	0.9233	0.0910
PRKDC	0.8061	1.1787	0.9100	0.7984	0.9233	0.0888
DUX1	0.8710	1.0103	0.9774	0.8345	0.9233	0.0419
NBPF4	0.8537	0.9729	0.9535	0.9131	0.9233	0.0263
RNF182	0.8732	1.0242	0.9716	0.8242	0.9233	0.0455
MINA	0.8767	1.0479	0.9739	0.7948	0.9233	0.0554
LOC648814	0.8992	0.9430	1.0047	0.8464	0.9233	0.0336
TIGD1	0.8971	0.9614	0.9698	0.8649	0.9233	0.0253
LOC728537	0.8143	1.0036	0.9695	0.9059	0.9233	0.0416
ZSCAN22	0.8344	1.1668	0.8467	0.8454	0.9233	0.0812
LOC649908	0.8755	1.0952	0.8948	0.8280	0.9234	0.0590
MIR1178	0.8083	0.9914	1.0641	0.8296	0.9234	0.0622
SASH1	0.8763	1.2204	0.9764	0.6206	0.9234	0.1241
LOC387683	0.8241	1.0765	0.9240	0.8690	0.9234	0.0550
LOC126661	0.8839	0.9057	0.9308	0.9732	0.9234	0.0192
KRT83	0.9447	0.9651	0.9525	0.8315	0.9234	0.0309
LOC645363	0.8599	1.0623	0.8964	0.8751	0.9234	0.0469
MAP7D3	0.9277	0.9641	0.9877	0.8142	0.9234	0.0384
C21orf84	0.9455	0.8608	0.9739	0.9136	0.9235	0.0243
INPP5B	0.8552	1.0538	0.9369	0.8480	0.9235	0.0479
ARRB2	0.9085	1.0453	0.9132	0.8268	0.9235	0.0452
GPRIN1	0.7697	1.0051	0.9420	0.9771	0.9235	0.0529
LOC1001314	0.8341	1.0361	0.9754	0.8483	0.9235	0.0492
LOC653242	0.7243	1.1553	0.8992	0.9151	0.9235	0.0885
ZNF254	0.8799	1.0210	1.0379	0.7551	0.9235	0.0664
LOC728485	0.9163	1.0542	0.9067	0.8168	0.9235	0.0490
TMEM110	0.8187	1.1767	0.9290	0.7696	0.9235	0.0907
GSTO2	0.8237	1.0769	1.0471	0.7464	0.9235	0.0817
ALG10	0.8979	1.1268	0.9005	0.7689	0.9235	0.0744
LOC646829	0.8859	0.9751	0.9264	0.9067	0.9235	0.0191
LOC390705	0.8827	0.9258	0.9768	0.9088	0.9235	0.0199
LOC728689	0.8140	1.0810	0.9274	0.8720	0.9236	0.0573
ST7L	0.8058	1.1096	1.0092	0.7698	0.9236	0.0814
LOC650518	0.7469	1.1968	1.0212	0.7295	0.9236	0.1129
TMEM165	0.7582	1.3130	0.9209	0.7024	0.9236	0.1378
KRT18P28	1.0093	1.0278	0.9157	0.7418	0.9236	0.0654
PRICKLE3	0.8538	0.9362	0.9660	0.9385	0.9236	0.0243
LOC652331	0.8799	0.9054	1.0086	0.9007	0.9237	0.0288
CYMP	0.9458	1.0211	0.8542	0.8735	0.9237	0.0380

NOSIP	0.7924	1.1952	0.9159	0.7912	0.9237	0.0951
MIR875	0.8857	0.9819	0.9324	0.8947	0.9237	0.0219
CD68	0.7269	1.1025	1.0229	0.8426	0.9237	0.0852
ZNF486	0.7404	1.2909	0.9692	0.6945	0.9237	0.1363
CCR4	0.8606	1.0493	0.9428	0.8422	0.9237	0.0472
LOC729533	0.8706	1.0079	0.9077	0.9088	0.9237	0.0294
SLC12A9	0.7575	1.2626	0.9145	0.7604	0.9237	0.1188
LOC653895	0.7934	1.1955	0.9664	0.7397	0.9238	0.1027
C1orf102	0.8110	1.2306	0.8724	0.7810	0.9238	0.1040
LOC1001291	0.9381	0.9056	1.0234	0.8281	0.9238	0.0404
TRMT6	0.7987	1.0347	1.1317	0.7300	0.9238	0.0952
FLJ33360	0.8603	1.0042	0.9591	0.8716	0.9238	0.0347
RAB27A	0.8849	1.0798	0.9742	0.7564	0.9238	0.0686
LOC285941	0.9801	0.8528	0.9781	0.8843	0.9238	0.0325
FAM30A	0.9587	0.9904	0.9070	0.8393	0.9238	0.0330
MAN1B1	0.9422	1.2867	0.8458	0.6207	0.9238	0.1384
LOC651268	0.8455	1.0596	0.9704	0.8200	0.9239	0.0559
MIR543	0.8415	0.9959	1.0315	0.8265	0.9239	0.0525
HTRA1	0.7577	1.1234	1.0914	0.7230	0.9239	0.1064
MED1	0.7398	1.1819	1.0819	0.6922	0.9239	0.1222
EFEMP2	0.9594	0.9799	0.9162	0.8403	0.9239	0.0309
LOC388559	0.8845	1.0267	0.9487	0.8358	0.9239	0.0413
PLAGL2	0.9172	1.2211	0.7968	0.7608	0.9240	0.1045
PNKD	0.8815	1.1400	0.9351	0.7393	0.9240	0.0830
LOC402562	0.8280	1.0504	0.9096	0.9080	0.9240	0.0462
LOC440302	0.8890	1.0482	0.9494	0.8095	0.9240	0.0503
CCNDBP1	0.9743	1.1603	0.8441	0.7174	0.9240	0.0946
RUNDC2B	0.8277	1.0530	1.0091	0.8064	0.9240	0.0626
AIM1L	0.8912	0.9837	0.9215	0.8998	0.9240	0.0209
DENND4C	0.8780	1.2693	0.8574	0.6914	0.9240	0.1224
KIF4B	0.6981	1.0999	1.0018	0.8965	0.9241	0.0860
EDG1	0.8411	1.0326	1.0749	0.7477	0.9241	0.0777
LOC650724	0.8057	0.8680	1.1011	0.9214	0.9241	0.0636
FNDC4	0.8977	1.0312	0.8709	0.8966	0.9241	0.0362
TATDN3	0.7433	1.2961	0.9677	0.6894	0.9241	0.1379
CHRNA6	0.7887	0.9889	0.9309	0.9881	0.9241	0.0471
LOC1001296	0.8413	0.9479	1.0596	0.8477	0.9241	0.0513
PELI3	0.8013	1.0429	0.9759	0.8764	0.9241	0.0534
MFSD2	0.8547	1.0647	0.9281	0.8491	0.9241	0.0502
LOC1001327	0.8178	1.0064	1.1239	0.7485	0.9242	0.0860
FUT1	0.8275	1.1155	0.9167	0.8370	0.9242	0.0668
FLJ30058	0.8494	0.9751	0.9751	0.8972	0.9242	0.0309
TBL1X	0.8457	1.1210	0.9321	0.7980	0.9242	0.0712
LILRP2	0.8877	1.0551	0.9112	0.8429	0.9242	0.0459
CCR5	0.8873	0.9200	1.0996	0.7900	0.9242	0.0646
NRN1L	0.8125	1.0070	0.9375	0.9398	0.9242	0.0406

CHRNB3	0.9281	1.0002	0.8603	0.9083	0.9242	0.0290
LOC646278	0.8517	1.0577	0.9788	0.8087	0.9242	0.0573
LOC1001343	0.8057	1.0415	0.9772	0.8725	0.9242	0.0527
CTTNBP2NL	0.9510	0.9963	0.9559	0.7938	0.9242	0.0447
MUC21	0.7471	1.0525	0.9713	0.9261	0.9242	0.0646
DERL2	0.8391	1.2004	1.0608	0.5968	0.9243	0.1321
RPS26P10	0.8449	1.0443	0.9807	0.8271	0.9243	0.0527
NCRNA00081	0.9141	1.0572	0.9646	0.7612	0.9243	0.0619
AK2P2	0.8327	1.0362	0.9101	0.9181	0.9243	0.0420
MYF5	0.7903	1.0482	0.9581	0.9005	0.9243	0.0540
LOC652651	0.8805	1.0247	0.9484	0.8436	0.9243	0.0399
LOC644885	0.9478	0.8106	1.0626	0.8761	0.9243	0.0539
USP18	0.9346	1.0427	1.0541	0.6657	0.9243	0.0903
LOC1001326	0.8685	0.8881	1.0111	0.9296	0.9243	0.0316
SDHALP1	0.8474	1.1306	0.9102	0.8091	0.9243	0.0718
CC2D2A	0.9200	1.0878	0.8531	0.8365	0.9243	0.0574
HLA-A29.1	0.8589	1.1712	0.8673	0.8000	0.9243	0.0836
LOC130773	0.9171	1.1899	0.9120	0.6782	0.9243	0.1046
MED28	0.6441	1.3564	0.9757	0.7211	0.9243	0.1605
SHKBP1	0.9461	1.1038	0.8802	0.7672	0.9243	0.0703
LOC643384	0.7795	1.2880	0.8799	0.7498	0.9243	0.1244
RPGR	0.8793	1.0467	0.9458	0.8256	0.9243	0.0476
GADD45G	0.8167	1.1587	0.9561	0.7661	0.9244	0.0878
ARP11	0.7840	1.0682	1.0856	0.7600	0.9244	0.0882
CSPP1	0.8625	1.1824	0.8496	0.8032	0.9244	0.0869
MIR558	0.8289	1.0275	0.9900	0.8513	0.9244	0.0495
FGFBP3	0.8313	1.0631	0.9334	0.8699	0.9244	0.0508
TSPAN5	0.8262	1.0957	0.9639	0.8119	0.9245	0.0666
TMC1	0.7780	1.0096	0.9808	0.9294	0.9245	0.0516
CNNM2	0.8543	1.0338	0.9803	0.8295	0.9245	0.0492
GAN	0.8694	1.0106	0.9879	0.8302	0.9245	0.0441
LOC651302	0.7748	1.1188	0.9601	0.8443	0.9245	0.0752
LOC731444	0.8419	1.2179	0.8489	0.7894	0.9245	0.0987
ZSCAN2	0.8713	1.1584	0.8192	0.8491	0.9245	0.0787
CCS	0.7820	1.2292	0.8891	0.7977	0.9245	0.1043
CP110	0.8063	1.0875	1.0004	0.8039	0.9245	0.0712
KCTD9	0.9077	1.0127	0.9937	0.7840	0.9245	0.0521
ATXN3	0.8017	1.1845	0.8228	0.8891	0.9245	0.0886
NAP1L4	0.7262	1.3420	0.8940	0.7359	0.9245	0.1444
LOC647370	0.8073	1.1282	0.9740	0.7886	0.9245	0.0797
DGAT1	0.7922	1.1860	0.9588	0.7613	0.9246	0.0973
LOC1001328	0.8781	1.1318	0.9236	0.7647	0.9246	0.0767
SOAT1	0.9488	0.9729	1.0304	0.7462	0.9246	0.0619
LOC643435	0.9114	1.0199	0.9684	0.7986	0.9246	0.0475
PWWP2B	0.7798	1.2580	0.8669	0.7936	0.9246	0.1128
ARAF	0.6620	1.2391	1.0029	0.7943	0.9246	0.1261

LOC1001339	0.8537	0.9489	1.0141	0.8816	0.9246	0.0359
CSPG4LYP2	0.9567	0.9536	0.9446	0.8435	0.9246	0.0272
EXOC4	0.7257	1.2136	1.0452	0.7139	0.9246	0.1232
LCN15	0.9125	1.0757	0.8767	0.8335	0.9246	0.0529
MRC2	0.9474	1.0032	0.9724	0.7754	0.9246	0.0510
LOC642781	0.9015	1.1366	0.9813	0.6790	0.9246	0.0953
LOC647187	0.9791	1.0591	0.8627	0.7976	0.9246	0.0585
LOC729510	0.8936	0.9951	0.9330	0.8767	0.9246	0.0263
DHRS7B	0.7576	1.2740	1.0531	0.6138	0.9246	0.1481
CSNK2A2	0.7322	1.3712	0.9231	0.6719	0.9246	0.1582
LOC1001344	0.8895	0.9741	1.0247	0.8102	0.9246	0.0472
H1FX	0.9428	1.1796	0.8616	0.7145	0.9246	0.0972
EXPH5	0.6679	1.2804	0.9202	0.8301	0.9246	0.1296
LOC96610	0.7867	1.1270	1.0905	0.6944	0.9247	0.1082
NR3C1	0.8404	1.0432	0.9255	0.8896	0.9247	0.0432
LOC728444	1.0261	0.9501	0.8823	0.8401	0.9247	0.0407
PCDHB2	0.8571	1.1756	0.9544	0.7116	0.9247	0.0974
TSSK4	0.9033	1.0576	0.8910	0.8471	0.9248	0.0459
LOC645304	0.9109	1.0701	0.9243	0.7938	0.9248	0.0566
C6orf68	0.8220	1.0221	1.0147	0.8403	0.9248	0.0542
FBXL19	0.8884	0.9473	0.9518	0.9117	0.9248	0.0151
LOC339778	0.7494	1.0782	1.0008	0.8707	0.9248	0.0725
LOC1001292	0.9655	0.9251	0.9685	0.8402	0.9248	0.0299
LOC650399	0.9075	0.9580	1.0404	0.7934	0.9248	0.0517
LOC1001348	0.9311	0.9889	0.9391	0.8403	0.9248	0.0310
C20orf127	0.7895	1.0928	0.9769	0.8401	0.9248	0.0686
LOC728503	0.8632	1.0015	1.0187	0.8159	0.9248	0.0503
LOC654078	0.8715	0.9467	1.0322	0.8490	0.9248	0.0414
ZNF600	0.9182	0.9664	0.9146	0.9002	0.9249	0.0144
LOC642341	0.8845	0.9983	0.9767	0.8398	0.9249	0.0376
LOC346085	0.9385	1.0749	0.9422	0.7438	0.9249	0.0682
LOC646806	0.7801	0.9403	0.9998	0.9793	0.9249	0.0498
LRFN3	0.9042	1.1814	0.8372	0.7767	0.9249	0.0894
NBPF10	0.7822	1.3603	0.8204	0.7367	0.9249	0.1461
TMEM77	0.8737	1.0715	0.9962	0.7583	0.9249	0.0689
TAF10	0.7331	1.2018	1.0075	0.7573	0.9249	0.1112
LOC728809	0.6538	1.2241	1.0766	0.7451	0.9249	0.1349
RPL23AP53	0.7931	1.1519	0.9406	0.8140	0.9249	0.0824
EHD2	0.7422	1.1248	0.9702	0.8625	0.9249	0.0813
LOC652579	0.9289	0.9681	0.9421	0.8606	0.9249	0.0229
C8G	0.8356	0.9507	1.0700	0.8434	0.9249	0.0550
JDP2	1.0365	1.0273	0.8436	0.7923	0.9249	0.0627
LOC1001342	0.8570	1.0640	0.9013	0.8775	0.9250	0.0472
C9orf4	0.8928	0.9708	0.9798	0.8564	0.9250	0.0301
TXNDC8	0.7908	1.1130	0.9936	0.8025	0.9250	0.0780
UNC50	0.7412	1.3233	0.9059	0.7296	0.9250	0.1387

RARG	0.7998	1.1298	0.9355	0.8350	0.9250	0.0741
ICOS	0.7153	1.1019	1.0350	0.8479	0.9250	0.0882
LOC1001312	0.8652	1.0020	1.0442	0.7886	0.9250	0.0594
C2orf58	0.8653	1.0043	0.9371	0.8935	0.9250	0.0303
CCND3	0.7251	1.2235	0.9831	0.7685	0.9251	0.1144
OGFRL1	0.8010	1.0673	0.9660	0.8659	0.9251	0.0583
LOC648164	0.8610	1.0116	0.9893	0.8383	0.9251	0.0440
ZNF354B	0.8408	0.9839	0.9774	0.8982	0.9251	0.0342
FAM40B	0.8623	1.0413	0.9444	0.8524	0.9251	0.0439
FAM128B	0.9399	1.0694	1.0394	0.6517	0.9251	0.0952
FAM10A4	0.8071	1.0940	1.1437	0.6556	0.9251	0.1165
ECAT8	0.9219	0.9568	0.9822	0.8396	0.9251	0.0311
TRAF3IP1	0.8802	1.0881	0.9338	0.7984	0.9251	0.0610
LYL1	0.8072	1.0561	0.9649	0.8723	0.9251	0.0543
ATG9A	0.7913	1.1925	0.9537	0.7629	0.9251	0.0985
LOC651371	0.9389	0.9993	0.9208	0.8415	0.9251	0.0325
HGF	0.8506	1.0105	0.9297	0.9098	0.9251	0.0330
NAG18	0.8119	1.2145	0.8954	0.7787	0.9251	0.0995
LOC440558	0.8389	1.0809	0.9620	0.8188	0.9251	0.0608
LENG9	0.9352	0.9351	0.9911	0.8392	0.9252	0.0316
LOC1001309	0.7834	1.0457	1.0036	0.8680	0.9252	0.0606
NAP1L2	0.8943	1.0125	0.9340	0.8599	0.9252	0.0328
LOC731999	0.9168	1.1503	0.8270	0.8065	0.9252	0.0788
SNX11	0.8336	1.1975	1.0089	0.6608	0.9252	0.1153
LOC441005	0.9196	0.9739	0.9634	0.8439	0.9252	0.0295
MIR638	0.9560	0.9090	0.8879	0.9480	0.9252	0.0161
LOC158257	0.8772	1.0321	0.9786	0.8129	0.9252	0.0493
C12orf61	0.8972	1.1093	0.9052	0.7892	0.9252	0.0668
LOC1001343	0.8591	1.1003	0.9202	0.8214	0.9253	0.0618
LOC1001297	0.9312	1.0345	0.9685	0.7668	0.9253	0.0570
LOC387720	0.8669	0.9993	0.9911	0.8438	0.9253	0.0407
LOC645967	0.8867	1.0070	0.8349	0.9725	0.9253	0.0393
GHRH	0.8339	0.9676	1.0362	0.8633	0.9253	0.0468
CCDC67	0.9307	0.9477	0.9504	0.8724	0.9253	0.0182
TRPC5	0.9433	1.0334	0.8855	0.8390	0.9253	0.0419
CXorf36	0.7988	1.0651	1.0449	0.7924	0.9253	0.0750
C16orf47	0.8687	1.0379	0.9425	0.8521	0.9253	0.0424
LOC1000936	0.9019	1.0009	0.8648	0.9338	0.9253	0.0289
REEP6	0.8524	1.2230	0.9733	0.6526	0.9253	0.1192
SMYD5	0.8045	1.0232	1.0586	0.8149	0.9253	0.0672
PPP1R3E	0.8852	1.0848	0.8503	0.8811	0.9253	0.0537
LOC645504	0.8201	0.9852	1.0076	0.8886	0.9254	0.0436
LOC647666	0.9219	0.9685	0.8899	0.9213	0.9254	0.0162
TRAF6	0.8572	1.0779	0.9567	0.8096	0.9254	0.0594
OXCT1	0.9109	1.1110	0.9091	0.7706	0.9254	0.0700
LOC1001327	0.8135	1.0996	1.0028	0.7856	0.9254	0.0755

LOC1001305	1.0235	0.9794	0.9428	0.7559	0.9254	0.0589
RBMS2P	0.8729	1.1152	0.8859	0.8276	0.9254	0.0645
LOC1001330	0.8818	1.0594	0.8843	0.8761	0.9254	0.0447
ATP6AP2	0.7601	1.3168	0.9425	0.6822	0.9254	0.1414
LOC730323	0.7811	1.2612	0.9234	0.7360	0.9254	0.1188
SLC5A6	0.7592	1.4549	0.8555	0.6323	0.9255	0.1823
FAM47E	0.9107	1.1199	0.9186	0.7526	0.9255	0.0752
MGC42367	0.9238	0.9873	0.9443	0.8465	0.9255	0.0295
LOC642393	0.8807	1.0295	0.9988	0.7929	0.9255	0.0546
PDE4C	0.7995	1.2698	0.9605	0.6723	0.9255	0.1290
LOC1001343	0.8320	1.0847	0.9852	0.8001	0.9255	0.0667
SLC25A5	0.7905	1.2486	0.9994	0.6637	0.9255	0.1280
LOC652408	0.8182	1.0399	0.9904	0.8536	0.9255	0.0532
AGTPBP1	0.7980	1.1153	0.9381	0.8507	0.9256	0.0695
HIST2H4A	0.6533	1.3701	0.9964	0.6824	0.9256	0.1673
ZNF414	0.8331	1.1638	0.8420	0.8634	0.9256	0.0797
LOC727942	0.8878	0.9790	0.9678	0.8678	0.9256	0.0280
LOC1001305	0.9225	0.9610	0.9033	0.9157	0.9256	0.0124
LOC653882	0.8686	0.8739	1.0021	0.9578	0.9256	0.0327
FER	0.7742	1.1348	0.9488	0.8447	0.9256	0.0784
LOC1001301	0.9181	0.9621	0.9119	0.9105	0.9256	0.0123
LSM14B	0.7918	1.0776	0.9961	0.8370	0.9256	0.0670
XCL2	0.8651	0.9140	1.0074	0.9161	0.9256	0.0297
LOC644014	0.9059	1.0443	0.8387	0.9137	0.9257	0.0430
C9orf127	0.8465	1.3044	0.8149	0.7368	0.9257	0.1283
LOC123688	0.7801	1.0661	0.9920	0.8646	0.9257	0.0639
LOC221136	0.8782	0.8617	1.0231	0.9397	0.9257	0.0365
ARNTL2	0.9152	0.9329	0.8983	0.9564	0.9257	0.0124
LOC377711	0.9344	0.8999	0.9875	0.8809	0.9257	0.0234
CNTNAP1	0.9259	1.0596	0.9124	0.8050	0.9257	0.0522
LOC645291	0.8874	1.0484	0.9411	0.8259	0.9257	0.0472
LOC441734	0.8613	0.9928	0.9929	0.8559	0.9257	0.0388
HIT-40	0.7934	1.0554	1.0951	0.7590	0.9257	0.0870
LOC1001300	0.8838	1.1749	0.9855	0.6588	0.9258	0.1075
RAP2B	0.8915	1.0910	0.9296	0.7909	0.9258	0.0624
LOC646716	0.8512	1.0067	0.9382	0.9070	0.9258	0.0324
DCLRE1C	0.9297	0.9881	0.9413	0.8440	0.9258	0.0300
L1CAM	0.8869	1.0347	0.9628	0.8188	0.9258	0.0467
LOC642381	0.8407	1.0800	0.9652	0.8173	0.9258	0.0608
C8orf37	0.7486	1.2860	1.0057	0.6629	0.9258	0.1404
LOC132241	0.9412	1.0660	0.9217	0.7743	0.9258	0.0598
COL4A2	0.8996	0.8963	0.9422	0.9652	0.9258	0.0168
DFNB59	0.8251	1.0810	0.9055	0.8918	0.9258	0.0546
LOC1001347	0.8593	1.0315	0.9814	0.8312	0.9258	0.0480
LOC442232	0.7864	1.1260	1.1165	0.6745	0.9259	0.1151
C12orf57	0.9111	1.1462	1.0128	0.6334	0.9259	0.1087

SENP1	0.8095	1.2046	0.9560	0.7333	0.9259	0.1038
LOC642828	0.7736	1.0126	1.2288	0.6884	0.9259	0.1221
NKX3-1	0.7952	1.0053	1.2884	0.6146	0.9259	0.1448
LOC650566	0.7946	1.0352	1.0751	0.7988	0.9259	0.0750
HOXD4	0.8687	1.0284	0.8772	0.9294	0.9259	0.0367
AIM1	0.7568	1.1752	0.9647	0.8071	0.9259	0.0941
AP3M2	0.8670	1.2128	0.9090	0.7150	0.9259	0.1043
KRTAP20-3	0.9293	1.0719	0.8924	0.8102	0.9260	0.0546
HP	0.9338	0.9721	0.8992	0.8988	0.9260	0.0174
LOC650155	0.8421	0.9516	0.9957	0.9146	0.9260	0.0325
SLC12A8	0.7755	1.1865	0.9815	0.7605	0.9260	0.1004
LOC399888	0.8389	0.9769	1.0188	0.8694	0.9260	0.0428
SYF2	0.8165	1.2254	0.9303	0.7319	0.9260	0.1078
UBN2	0.8688	0.9880	0.9777	0.8697	0.9260	0.0329
LOC1001281	0.8681	0.9852	0.9722	0.8787	0.9260	0.0306
LOC1001304	0.8561	1.0773	0.9584	0.8125	0.9261	0.0590
SNORD103A	0.8346	1.0606	0.9518	0.8573	0.9261	0.0515
TGIF2LY	0.8216	1.0832	0.9200	0.8795	0.9261	0.0561
RBM41	0.7587	1.2071	0.9757	0.7627	0.9261	0.1065
LOC645663	0.7687	1.0862	0.9636	0.8859	0.9261	0.0667
LRRC39	0.8443	0.9804	1.0111	0.8685	0.9261	0.0410
C10orf140	0.7871	1.1117	0.9913	0.8144	0.9261	0.0767
LOC643912	0.9937	0.8355	0.8683	1.0069	0.9261	0.0435
KIAA1211	0.8869	0.9996	1.0230	0.7949	0.9261	0.0529
GUCY2E	0.7912	0.9512	1.0070	0.9551	0.9261	0.0467
LOC652400	0.8213	0.9030	1.1048	0.8754	0.9261	0.0619
LOC647360	0.8687	1.0084	0.9899	0.8376	0.9261	0.0428
MARK3	0.7547	1.2826	0.8471	0.8201	0.9261	0.1204
SPRR2F	0.8587	1.0711	0.9922	0.7826	0.9261	0.0649
ARPM1	0.7903	1.1664	0.8540	0.8939	0.9262	0.0829
LOC644152	0.8383	1.0351	0.9782	0.8531	0.9262	0.0480
LOC646439	0.7738	1.1629	0.9239	0.8441	0.9262	0.0847
LOC1001346	0.8996	0.9960	0.9300	0.8791	0.9262	0.0255
LOC1001337	0.8716	0.9976	1.0669	0.7686	0.9262	0.0663
FLJ20464	0.8344	0.9899	0.9722	0.9083	0.9262	0.0353
PCDHB7	0.8509	0.9757	0.9788	0.8994	0.9262	0.0311
SLN	0.8227	1.0380	0.9338	0.9103	0.9262	0.0443
SLFN12	0.9987	0.9744	0.8846	0.8471	0.9262	0.0360
PCSK5	0.8344	1.0079	1.0242	0.8384	0.9262	0.0520
LOC1001280	0.8306	1.1131	0.8863	0.8749	0.9262	0.0634
LOC1001318	0.9166	0.9764	0.9736	0.8384	0.9263	0.0324
KIAA0738	0.8276	0.9033	1.0610	0.9131	0.9263	0.0488
HNRNPA1L2	0.8153	1.0966	0.9658	0.8274	0.9263	0.0663
TNFRSF4	0.8670	0.9994	0.9962	0.8424	0.9263	0.0416
RAPGEFL1	0.9309	1.0245	0.9255	0.8242	0.9263	0.0409
ZNF391	0.9028	0.9884	0.9229	0.8913	0.9263	0.0217

AGFG1	0.9270	1.0084	0.9358	0.8341	0.9263	0.0357
RN7SK	0.4972	1.2942	1.1719	0.7420	0.9263	0.1857
CD164L2	0.9289	1.0085	0.8167	0.9514	0.9264	0.0402
LOC147650	0.9425	0.9844	0.9401	0.8385	0.9264	0.0310
FLJ33534	0.7340	1.0919	1.0402	0.8394	0.9264	0.0841
CXCL13	1.0047	1.0814	0.7596	0.8599	0.9264	0.0721
LOC647797	0.9435	0.9314	0.9344	0.8962	0.9264	0.0104
INSRR	0.8713	0.9432	0.9410	0.9501	0.9264	0.0185
C9orf45	0.9238	1.2737	0.7322	0.7759	0.9264	0.1228
HTN1	0.9004	0.9191	0.9857	0.9004	0.9264	0.0203
LOC728312	0.7957	1.1743	0.9105	0.8252	0.9264	0.0861
LOC1001343	0.8496	1.1210	1.0001	0.7350	0.9264	0.0846
P2RY1	0.8554	0.9857	0.9146	0.9500	0.9264	0.0278
ZNF417	0.8545	0.9877	0.9638	0.8997	0.9265	0.0303
PLD1	0.9160	1.0189	0.9381	0.8329	0.9265	0.0382
C17orf69	0.9256	1.1013	0.9330	0.7460	0.9265	0.0725
SLC6A13	0.8087	1.0662	0.9355	0.8955	0.9265	0.0536
CCDC136	0.9274	1.2001	0.8580	0.7203	0.9265	0.1009
SPACA4	0.8944	1.0025	0.9906	0.8185	0.9265	0.0434
SENP3	0.8925	1.0205	0.9290	0.8639	0.9265	0.0341
LOC128322	0.9201	1.0162	1.0033	0.7664	0.9265	0.0575
MIR933	0.8116	1.0487	0.9344	0.9115	0.9265	0.0487
TPP1	0.9793	1.1495	0.8283	0.7491	0.9265	0.0883
OR4D1	0.8599	0.9914	0.9923	0.8627	0.9266	0.0377
LOC1001319	0.9531	0.9075	0.9661	0.8796	0.9266	0.0201
MX1	0.8492	1.1813	0.8648	0.8110	0.9266	0.0857
ARID5A	0.9182	0.9934	0.9721	0.8228	0.9266	0.0381
VASH1	0.9251	1.0852	0.8612	0.8350	0.9266	0.0561
LOC653602	0.8960	1.0099	0.9803	0.8203	0.9266	0.0429
YES1	1.0201	0.9640	1.0441	0.6784	0.9267	0.0844
NFKBID	0.8673	1.0148	0.8897	0.9347	0.9267	0.0326
LOC644167	0.8002	1.0585	0.9892	0.8587	0.9267	0.0591
LOC442041	0.7945	1.1300	0.9188	0.8635	0.9267	0.0724
LOC644690	0.9227	0.9486	0.9275	0.9080	0.9267	0.0084
LOC1001341	0.8127	1.0659	0.9874	0.8408	0.9267	0.0602
LNP1	0.9472	1.1145	0.8655	0.7796	0.9267	0.0713
COQ5	0.8354	1.3415	0.8418	0.6883	0.9267	0.1427
C10orf51	0.8571	1.0499	0.9697	0.8302	0.9268	0.0510
SLC10A5	0.9559	0.9696	0.8886	0.8930	0.9268	0.0210
SH3BP2	0.7489	1.0688	1.0714	0.8179	0.9268	0.0839
LOC441066	0.9051	1.0704	0.9111	0.8204	0.9268	0.0522
STOX2	0.9525	1.0161	0.8550	0.8834	0.9268	0.0361
LOC729424	0.9567	1.0785	0.8555	0.8164	0.9268	0.0586
LOC647195	0.6403	1.1169	1.2119	0.7381	0.9268	0.1400
LOC647521	0.8140	1.0252	1.0201	0.8479	0.9268	0.0558
SLC22A5	0.7975	1.3389	0.8843	0.6865	0.9268	0.1432

LOC645783	0.8750	0.9319	1.0335	0.8668	0.9268	0.0384
IFT57	0.9548	0.8685	0.9891	0.8949	0.9268	0.0275
LOC652671	0.8289	0.9374	1.1100	0.8311	0.9268	0.0661
TSG1	0.7194	1.0343	1.0780	0.8756	0.9268	0.0817
SIRT5	0.8299	1.0923	0.9378	0.8473	0.9268	0.0600
LOC644735	0.8432	1.0911	0.9842	0.7888	0.9268	0.0685
LOC652256	0.9336	1.0983	0.7911	0.8844	0.9269	0.0644
RBM42	0.8741	1.0956	1.0206	0.7172	0.9269	0.0837
CAPN13	0.8290	1.1699	1.0034	0.7052	0.9269	0.1015
LOC642477	0.7179	1.3365	0.9336	0.7197	0.9269	0.1456
C6orf138	0.8353	1.0061	0.9811	0.8851	0.9269	0.0402
LOC390998	0.8428	1.0081	0.9545	0.9022	0.9269	0.0354
CTAG1A	0.8307	1.1385	0.9416	0.7968	0.9269	0.0770
LOC642280	0.9324	1.0249	0.8839	0.8664	0.9269	0.0355
LOC1001282	0.8687	0.9898	0.9070	0.9421	0.9269	0.0258
RPL8	0.7455	1.0651	1.0216	0.8754	0.9269	0.0728
LOC402232	0.8206	1.1688	0.9259	0.7923	0.9269	0.0856
PDPK1	0.8481	1.0872	0.9694	0.8030	0.9269	0.0639
LOC729879	0.8414	1.0693	0.9413	0.8558	0.9269	0.0523
LOC1001347	0.8959	1.0264	0.9718	0.8138	0.9270	0.0462
H2BFM	0.8514	1.0347	1.0285	0.7933	0.9270	0.0616
MIR567	0.9512	0.9280	0.9463	0.8824	0.9270	0.0157
LOC400095	0.7693	1.1733	0.9396	0.8260	0.9270	0.0894
COL27A1	0.8729	1.0237	0.9643	0.8473	0.9270	0.0409
LOC652834	0.8572	0.9745	1.0376	0.8389	0.9270	0.0475
LOC654103	0.8867	1.1487	0.9873	0.6855	0.9270	0.0969
CENPT	0.8090	1.2097	0.8745	0.8151	0.9271	0.0954
LOC649974	0.8676	1.0243	0.9855	0.8309	0.9271	0.0462
KLF3	0.7876	1.0744	1.0523	0.7941	0.9271	0.0788
FAM180A	0.8918	0.9057	1.0532	0.8578	0.9271	0.0432
IP6K1	0.8619	1.2067	0.9167	0.7231	0.9271	0.1017
BIRC2	0.7306	1.3152	0.9154	0.7472	0.9271	0.1359
CXCL1	0.7714	1.0979	1.0260	0.8132	0.9271	0.0797
ZNF576	0.7669	1.1084	0.9895	0.8437	0.9271	0.0760
TNNI1	0.8337	0.9919	0.9939	0.8891	0.9271	0.0396
FRMPD3	0.9513	1.0113	0.9066	0.8394	0.9272	0.0363
LOC389676	0.8618	0.9736	0.9174	0.9558	0.9272	0.0247
DNTTIP1	0.9208	1.2360	0.8475	0.7043	0.9272	0.1123
PCDH15	0.9537	0.9383	0.9537	0.8629	0.9272	0.0217
TMED7	0.7871	1.1527	1.0886	0.6804	0.9272	0.1145
SCARNA3	0.8298	1.0539	0.9651	0.8601	0.9272	0.0512
EPB41L4A	0.8438	1.1749	0.8089	0.8813	0.9272	0.0839
LOC729687	0.8866	1.0653	0.9979	0.7591	0.9272	0.0671
MIR526A2	0.8330	1.0360	1.0644	0.7755	0.9272	0.0722
LOC652044	0.8612	0.9184	0.9465	0.9830	0.9273	0.0257
MMAA	0.8094	1.1501	0.9464	0.8032	0.9273	0.0813

LOC644193	0.8283	0.9406	1.0136	0.9267	0.9273	0.0381
LOC1001282	0.8701	1.1067	0.9089	0.8235	0.9273	0.0623
LOC647083	0.8842	0.9921	0.9198	0.9132	0.9273	0.0229
CHMP1A	0.8851	1.3127	0.8132	0.6982	0.9273	0.1341
FEM1B	0.7769	1.1871	0.9578	0.7875	0.9273	0.0960
SV2A	0.9415	0.9295	0.9499	0.8885	0.9273	0.0136
MOBKL1A	0.8483	1.0612	0.9577	0.8422	0.9273	0.0519
LOC653983	0.8658	0.9962	1.0241	0.8234	0.9274	0.0489
MDP1	0.8860	1.0825	0.9795	0.7614	0.9274	0.0683
SLC38A9	0.7840	1.1870	0.9682	0.7703	0.9274	0.0976
LOC649926	0.8957	0.9703	1.0362	0.8072	0.9274	0.0493
PIGB	0.9062	1.2061	0.8394	0.7578	0.9274	0.0977
LOC728484	0.7390	1.0710	1.2405	0.6591	0.9274	0.1373
SNORA81	0.8675	1.0796	0.9724	0.7902	0.9274	0.0630
LOC1001290	0.8978	1.0436	0.8746	0.8936	0.9274	0.0391
CLU	0.8718	1.0208	0.9513	0.8658	0.9274	0.0367
SYNGR2	0.8944	1.0697	0.9661	0.7795	0.9274	0.0610
C6orf188	0.8185	1.0100	1.0315	0.8499	0.9275	0.0544
AHSA1	0.7085	1.4397	0.9449	0.6168	0.9275	0.1842
LOC652577	0.8799	0.9635	1.0181	0.8485	0.9275	0.0387
LOC731827	0.8432	0.9877	0.9890	0.8901	0.9275	0.0364
FAM40A	0.8469	1.0945	0.9608	0.8078	0.9275	0.0644
QSOX1	0.8572	1.1959	0.9419	0.7152	0.9275	0.1009
LOC646090	0.9339	0.8883	0.9703	0.9177	0.9276	0.0171
LOC645799	0.9428	1.0549	0.8782	0.8344	0.9276	0.0479
FAM46D	0.6679	1.2283	0.9458	0.8683	0.9276	0.1161
LOC654133	0.8266	0.9538	1.0102	0.9198	0.9276	0.0385
ELMOD1	0.8897	0.9651	1.0135	0.8422	0.9276	0.0382
FAM120A	0.8571	1.1323	0.9861	0.7350	0.9276	0.0854
LOC652513	0.7847	1.0309	0.9645	0.9303	0.9276	0.0520
PUS3	0.7006	1.2229	1.0805	0.7065	0.9276	0.1326
TUBA4	0.8786	0.9654	0.9173	0.9493	0.9276	0.0192
LOC1001286	0.8565	0.9704	1.0148	0.8689	0.9276	0.0387
LOC645620	0.9032	1.0915	0.8513	0.8648	0.9277	0.0557
LOC646187	0.8314	1.0180	0.9882	0.8731	0.9277	0.0448
TAF1A	0.8704	1.0476	0.9734	0.8193	0.9277	0.0512
LOC652869	0.8666	0.9032	0.9739	0.9670	0.9277	0.0258
LGR4	0.8571	1.1339	0.9336	0.7861	0.9277	0.0750
KIF27	0.8965	1.0663	0.8963	0.8517	0.9277	0.0474
PIH1D2	0.8063	1.0850	0.9959	0.8237	0.9277	0.0677
LOC653171	0.8858	1.2183	0.8680	0.7388	0.9277	0.1022
TDRKH	0.9850	0.9159	1.0117	0.7984	0.9278	0.0476
LOC647054	1.0018	0.9155	0.9193	0.8744	0.9278	0.0267
BLZF1	0.7787	1.0567	1.1449	0.7308	0.9278	0.1020
CTSC	0.8764	1.0953	0.9579	0.7815	0.9278	0.0665
CORO1B	0.8609	1.1934	0.9081	0.7487	0.9278	0.0946

LOC728344	0.8345	1.1095	0.9597	0.8074	0.9278	0.0691
LOC644500	0.8355	1.0225	0.9832	0.8699	0.9278	0.0446
FLJ26056	0.7963	1.0255	0.9677	0.9217	0.9278	0.0487
KIAA1967	0.9084	1.1637	0.9060	0.7332	0.9278	0.0887
LOC649057	0.8794	1.0352	0.9258	0.8708	0.9278	0.0378
LY86	0.9243	1.0106	0.9764	0.8000	0.9278	0.0462
MARCKSL1	0.7399	1.4768	0.7664	0.7282	0.9278	0.1832
LOC642644	0.8822	0.9889	0.9481	0.8921	0.9278	0.0250
FLJ44054	0.9267	0.9734	1.1015	0.7098	0.9279	0.0815
LTBP4	0.8188	1.1765	0.9069	0.8092	0.9279	0.0857
LOC644396	0.9490	0.9527	0.8948	0.9150	0.9279	0.0139
LOC728452	0.9642	1.0757	0.8313	0.8403	0.9279	0.0578
LOC644088	0.8052	0.9812	0.9811	0.9441	0.9279	0.0418
NCRNA0011!	0.9736	0.9535	0.9143	0.8701	0.9279	0.0229
C21orf126	0.7787	0.9693	1.0167	0.9468	0.9279	0.0518
FOXO6	0.8847	1.0121	0.9777	0.8370	0.9279	0.0405
DPPA2	0.8650	1.0592	0.9093	0.8781	0.9279	0.0447
LOC1001340	0.8929	1.0730	0.9252	0.8206	0.9279	0.0531
LOC652883	0.8980	1.0519	0.9378	0.8240	0.9279	0.0476
SIL1	0.8242	1.2651	0.8807	0.7417	0.9279	0.1160
PFN4	0.9391	0.9461	0.9667	0.8599	0.9279	0.0234
LOC729012	0.7712	1.0576	1.0347	0.8482	0.9279	0.0702
LOC644039	0.6988	1.2248	1.1954	0.5927	0.9279	0.1645
PRDM7	0.9349	0.9450	0.9328	0.8992	0.9279	0.0100
LOC1001291	0.8320	0.9338	1.0708	0.8752	0.9280	0.0520
ZMAT5	0.8574	1.2300	0.8649	0.7595	0.9280	0.1035
LOC1001335	0.7709	1.0144	1.0914	0.8352	0.9280	0.0750
OGFOD1	0.7370	1.3027	0.9591	0.7131	0.9280	0.1366
CAMLG	0.8397	1.2980	0.9862	0.5880	0.9280	0.1482
TBC1D19	0.8505	1.1806	0.8838	0.7970	0.9280	0.0861
LOC653100	0.9071	0.9949	0.9200	0.8900	0.9280	0.0231
KIR2DS2	0.7756	0.9435	1.2006	0.7922	0.9280	0.0984
ARHGEF4	0.9056	1.0106	0.9238	0.8721	0.9280	0.0295
CD82	0.8256	1.2593	0.9091	0.7180	0.9280	0.1172
PNPLA4	0.8478	0.9434	1.0721	0.8489	0.9280	0.0530
ANKRD27	0.8820	1.0238	1.0551	0.7513	0.9280	0.0699
ANKZF1	0.8934	1.1384	0.9249	0.7554	0.9280	0.0792
UBE3B	0.9271	1.0368	0.8982	0.8501	0.9281	0.0396
SETD1B	0.9158	1.0134	0.9328	0.8502	0.9281	0.0336
LOC256374	0.6790	1.0661	1.0635	0.9037	0.9281	0.0913
LOC651556	0.8459	1.0403	0.9503	0.8759	0.9281	0.0434
IQCF3	0.9132	0.9858	0.9483	0.8652	0.9281	0.0257
ANKLE1	0.9087	1.0313	0.9708	0.8017	0.9281	0.0490
RPL13AP3	0.9619	0.9957	0.8708	0.8841	0.9281	0.0302
KIAA1279	0.8344	1.1698	1.0178	0.6905	0.9281	0.1048
LOC1001339	0.6383	0.9251	1.1786	0.9707	0.9281	0.1113

LOC649179	0.9092	1.0285	0.8712	0.9037	0.9281	0.0345
PPAN-P2RY1	0.8545	1.0079	0.9361	0.9142	0.9282	0.0317
LOC401717	0.8510	0.9549	1.1120	0.7947	0.9282	0.0697
FLJ44006	0.8726	0.9972	0.9851	0.8578	0.9282	0.0366
LOC1001323	0.9006	1.1022	0.9067	0.8032	0.9282	0.0627
LOC654002	0.9371	1.0452	0.9664	0.7640	0.9282	0.0593
LOC645481	0.8280	1.0419	0.9321	0.9107	0.9282	0.0441
FBXO42	0.9987	1.0709	1.0346	0.6085	0.9282	0.1076
OR14J1	0.8889	1.0041	0.8761	0.9437	0.9282	0.0292
CASP7	0.7679	1.1909	1.0272	0.7269	0.9282	0.1099
LOC651403	0.9026	0.9071	0.9546	0.9485	0.9282	0.0136
MON1A	0.8742	1.1988	0.8344	0.8056	0.9282	0.0913
C10orf120	0.9035	1.0407	0.9776	0.7912	0.9282	0.0536
C12orf77	0.7522	0.9919	1.1074	0.8616	0.9283	0.0772
MYH2	0.9195	0.9548	0.9515	0.8872	0.9283	0.0158
C1orf111	0.8238	1.0466	0.9402	0.9025	0.9283	0.0463
SLC16A9	0.8860	1.1897	0.8839	0.7535	0.9283	0.0925
GIMAP2	0.8941	1.0308	1.0013	0.7871	0.9283	0.0555
REXO1	0.9095	1.0048	0.9163	0.8828	0.9284	0.0265
CCDC4	0.8683	1.0291	0.9470	0.8691	0.9284	0.0383
LOC644196	0.8664	0.9570	0.9932	0.8968	0.9284	0.0286
MOG	0.8374	1.2651	0.8895	0.7215	0.9284	0.1176
KAT5	0.9077	0.9748	1.0539	0.7771	0.9284	0.0586
LOC342541	0.9962	0.8798	0.9283	0.9092	0.9284	0.0247
LOC1001330	0.8981	0.8941	0.9582	0.9631	0.9284	0.0187
DUSP26	0.9032	0.9403	1.0185	0.8517	0.9284	0.0351
RNF113B	0.9231	1.1508	0.8087	0.8312	0.9284	0.0781
AMPH	0.9096	1.0362	0.9128	0.8551	0.9284	0.0383
LOC641710	0.7921	1.0431	0.9362	0.9425	0.9285	0.0516
LOC644116	0.9244	1.1118	0.9270	0.7507	0.9285	0.0737
IL18	0.7535	1.2648	0.9385	0.7571	0.9285	0.1201
OR2T29	0.7849	1.0059	1.0932	0.8300	0.9285	0.0727
DNAJB6	0.7181	1.2653	0.9467	0.7839	0.9285	0.1221
LOC653193	0.9008	0.9940	0.9682	0.8510	0.9285	0.0325
SLC35B1	0.7168	1.4338	0.8387	0.7248	0.9285	0.1707
LOC440353	0.8938	1.4185	0.6512	0.7507	0.9285	0.1708
ITGA10	0.9424	1.0404	0.7993	0.9321	0.9286	0.0495
SEC23B	0.8027	1.1997	1.0005	0.7114	0.9286	0.1087
DRG2	1.0385	1.0205	0.9345	0.7208	0.9286	0.0729
ZNF32	0.8214	1.3340	0.8565	0.7024	0.9286	0.1391
LOC729028	0.9861	0.8748	1.0274	0.8259	0.9286	0.0470
LEMD1	0.8089	1.0956	0.9533	0.8566	0.9286	0.0633
LOC651291	0.7910	1.0654	0.9941	0.8639	0.9286	0.0620
PRR18	0.8500	1.0131	0.9833	0.8679	0.9286	0.0408
SYT8	0.8606	0.9544	0.9936	0.9058	0.9286	0.0289
REPS2	0.7352	1.2479	0.9551	0.7762	0.9286	0.1167

ARRDC4	0.8372	1.1504	0.9304	0.7965	0.9286	0.0791
FOXC2	0.8694	1.0828	0.9291	0.8331	0.9286	0.0551
LOC441377	0.7432	0.9027	1.4636	0.6050	0.9286	0.1884
LOC1001327	0.8305	0.9544	1.0369	0.8928	0.9286	0.0441
LOC1001337	0.8777	1.0459	0.8169	0.9741	0.9286	0.0507
PTH2	0.8061	1.0602	0.9306	0.9176	0.9286	0.0520
ESCO2	0.8266	0.9724	1.0254	0.8904	0.9287	0.0439
MIR551A	0.9675	0.8675	0.9828	0.8969	0.9287	0.0277
SMAD2	0.9075	1.0258	0.9162	0.8654	0.9287	0.0342
DDX31	0.8050	1.1813	0.9360	0.7927	0.9287	0.0902
JMJD5	0.9724	0.9767	0.9269	0.8389	0.9287	0.0320
FAM90A10	0.9194	1.0462	0.9178	0.8317	0.9288	0.0442
MAP3K12	0.7869	1.1339	0.8828	0.9115	0.9288	0.0734
DNAH8	0.8829	1.0782	0.8595	0.8945	0.9288	0.0503
LOC651483	0.8300	1.0250	1.0129	0.8473	0.9288	0.0522
ZFATAS	0.8560	0.9911	1.0495	0.8186	0.9288	0.0547
ECAT1	0.9164	1.0491	1.0046	0.7453	0.9288	0.0671
FOXQ1	0.8816	0.9481	0.9506	0.9350	0.9288	0.0161
LOC643960	0.8013	1.0857	0.9900	0.8384	0.9288	0.0663
TMEM191C	0.8550	1.0131	0.9574	0.8899	0.9288	0.0352
CCDC7	0.8990	1.0451	0.8781	0.8932	0.9289	0.0390
NARFL	0.9756	1.1086	0.9157	0.7156	0.9289	0.0817
PIKFYVE	0.8765	1.0793	0.9642	0.7955	0.9289	0.0608
LOC1001316	0.8973	1.0456	0.9676	0.8051	0.9289	0.0512
KIAA1128	0.8625	1.1391	0.9534	0.7606	0.9289	0.0804
LOC729513	0.8498	0.9977	0.9911	0.8770	0.9289	0.0383
LOC646649	0.8646	1.0621	0.8653	0.9236	0.9289	0.0465
LOC441759	0.8749	0.8209	1.1305	0.8896	0.9290	0.0688
KCNS1	0.9661	0.9486	0.9465	0.8547	0.9290	0.0251
ATP2B4	0.8414	1.2508	0.8532	0.7706	0.9290	0.1088
FAM118A	0.9727	0.8945	0.8964	0.9524	0.9290	0.0198
PCDHB6	0.9292	0.9701	0.9206	0.8960	0.9290	0.0154
BFSP1	0.9044	0.9969	0.9246	0.8901	0.9290	0.0237
HLA-DPA1	0.8612	0.9871	1.0452	0.8225	0.9290	0.0523
LOC391556	0.8206	1.0340	0.9474	0.9140	0.9290	0.0441
LOC1001315	0.7984	1.0323	0.9448	0.9405	0.9290	0.0484
LOC1001301	0.9047	0.9169	0.9515	0.9430	0.9290	0.0110
RBMY1J	0.8853	1.0098	0.9432	0.8779	0.9290	0.0306
LOC644166	0.8714	0.9901	1.0528	0.8020	0.9290	0.0567
C1QTNF9B	0.9142	1.0883	0.9135	0.8002	0.9290	0.0595
LOC652153	0.8347	1.0771	0.9579	0.8466	0.9291	0.0566
GZMH	0.8009	1.0307	1.0156	0.8692	0.9291	0.0561
C8orf13	0.8294	0.9369	0.9569	0.9931	0.9291	0.0352
B3GALNT1	0.8763	0.9499	1.0241	0.8660	0.9291	0.0368
NCAPH	0.8491	1.0957	0.9120	0.8596	0.9291	0.0572
LOC731950	0.7414	1.3228	0.9004	0.7519	0.9291	0.1362

MRPL41	0.7439	1.3151	0.8915	0.7662	0.9291	0.1327
LOC1001315	0.8420	1.0174	0.8932	0.9640	0.9291	0.0386
TASP1	0.9567	1.1545	0.8188	0.7865	0.9291	0.0837
ARL16	0.8139	1.2498	0.9553	0.6976	0.9292	0.1192
IDI2	0.9525	0.9357	0.9730	0.8554	0.9292	0.0257
LOC728288	0.9241	1.0078	0.9167	0.8681	0.9292	0.0290
FAM132B	0.8597	0.9741	1.0576	0.8253	0.9292	0.0533
LOC650076	0.9573	0.9915	0.9501	0.8179	0.9292	0.0382
C2orf51	0.9989	0.9963	0.9500	0.7718	0.9292	0.0537
JUNB	0.8417	1.1534	0.9005	0.8214	0.9292	0.0766
LOC651131	0.9012	0.9381	0.9457	0.9319	0.9292	0.0098
MYCBPAP	0.9901	0.9770	0.8861	0.8639	0.9292	0.0318
LOC644363	0.7879	1.2450	1.0231	0.6609	0.9292	0.1292
LOC648496	0.9890	0.9651	0.9792	0.7838	0.9293	0.0487
PLA2G2F	0.9016	1.1063	0.9679	0.7412	0.9293	0.0758
RFPL4B	0.8931	1.0088	0.9799	0.8354	0.9293	0.0398
MIR511-2	0.8386	0.9981	1.0525	0.8279	0.9293	0.0566
PDIA3P	0.7611	1.1486	1.1346	0.6729	0.9293	0.1239
GFOD2	0.8451	1.2111	0.9604	0.7006	0.9293	0.1079
TEKT1	0.8672	1.0144	0.8934	0.9423	0.9293	0.0324
PARP14	0.8576	1.1375	0.9460	0.7763	0.9293	0.0776
IRF8	0.8283	1.0780	0.9827	0.8284	0.9293	0.0615
BRAF	0.7360	1.2280	0.9389	0.8145	0.9294	0.1080
CCDC46	0.8853	1.0523	0.9322	0.8476	0.9294	0.0445
RNF122	0.8466	1.1857	0.9730	0.7121	0.9294	0.1007
CRLS1	0.7147	1.3075	0.9449	0.7504	0.9294	0.1358
MIR1179	0.8156	1.0269	0.9375	0.9375	0.9294	0.0434
DGUOK	0.7196	1.2842	0.9868	0.7269	0.9294	0.1336
SR140	0.6500	1.4446	0.7991	0.8238	0.9294	0.1760
ZMYM4	0.8012	1.1973	0.9859	0.7331	0.9294	0.1040
MYBBP1A	0.8150	0.9904	1.0838	0.8284	0.9294	0.0651
ARHGEF12	0.9022	1.0485	0.9366	0.8303	0.9294	0.0455
MGC3771	0.8786	1.0720	0.9640	0.8030	0.9294	0.0578
LOC1001281	0.9057	0.9964	0.9478	0.8678	0.9294	0.0277
XRN1	0.9240	1.0845	0.9132	0.7961	0.9294	0.0592
STGC3	0.9066	0.9391	0.9969	0.8752	0.9294	0.0260
ZNF285B	0.8420	1.0992	0.9151	0.8615	0.9294	0.0587
LOC643273	0.9344	1.1405	0.8514	0.7915	0.9294	0.0762
FTSJD1	0.8501	1.1633	0.9870	0.7174	0.9295	0.0954
LOC650919	0.8375	0.9595	1.0442	0.8766	0.9295	0.0459
HGD	0.8141	1.1217	0.9214	0.8606	0.9295	0.0678
PODNL1	0.8737	0.9871	0.9790	0.8781	0.9295	0.0310
AIRE	0.7122	1.1917	1.0992	0.7148	0.9295	0.1261
PHEX	0.8929	0.9072	1.0288	0.8889	0.9295	0.0334
LOC653809	0.9052	0.9407	1.0102	0.8618	0.9295	0.0314
LOC643339	0.8821	1.2582	0.7970	0.7806	0.9295	0.1118

GIF	0.7671	1.0081	1.0232	0.9194	0.9295	0.0588
FEZ1	0.8439	1.0630	0.9852	0.8259	0.9295	0.0570
DND1	0.8998	1.0147	0.9963	0.8072	0.9295	0.0479
APBA2BP	0.8802	1.0998	0.9931	0.7451	0.9295	0.0761
CACNA2D4	0.9187	1.0145	0.9405	0.8444	0.9295	0.0350
POLR1D	0.7433	1.3406	0.9045	0.7297	0.9296	0.1427
TMBIM6	0.8930	1.2814	0.9122	0.6315	0.9296	0.1336
ZIC5	0.7759	0.9932	1.0555	0.8936	0.9296	0.0611
FLJ44048	0.9049	0.9579	1.0130	0.8425	0.9296	0.0364
C1orf192	0.7856	0.9680	1.0881	0.8766	0.9296	0.0646
KIAA1345	0.7330	1.2133	0.9984	0.7737	0.9296	0.1111
ZNF177	0.7709	1.1769	0.9032	0.8675	0.9296	0.0870
DNAH6	0.9095	1.0580	0.8371	0.9139	0.9296	0.0463
PARP8	0.8796	1.1324	0.9754	0.7311	0.9296	0.0842
TFAP4	0.8116	1.0725	0.9691	0.8653	0.9296	0.0578
MGC27345	0.9413	1.0864	0.8733	0.8175	0.9296	0.0581
IL25	0.8783	1.0123	1.0845	0.7434	0.9296	0.0754
LOC650162	0.8543	0.9973	0.9167	0.9502	0.9296	0.0301
LOC731542	0.7984	1.2856	0.9006	0.7340	0.9296	0.1235
FLJ46906	0.8203	1.1035	0.8729	0.9219	0.9297	0.0616
KRT6C	0.9042	0.9991	0.9831	0.8322	0.9297	0.0385
FLJ20850	0.9275	0.9212	1.0706	0.7994	0.9297	0.0555
TOP2B	0.7643	1.3164	0.8773	0.7607	0.9297	0.1317
ANKS3	0.8444	1.1243	0.8525	0.8976	0.9297	0.0659
MXRA8	0.9309	1.0681	0.9266	0.7932	0.9297	0.0561
PLSCR1	0.9126	1.0029	0.8672	0.9363	0.9297	0.0283
LOC1001312	0.9350	0.9649	0.9639	0.8551	0.9297	0.0258
LOC728326	0.8561	1.0384	0.9015	0.9229	0.9297	0.0388
DNMBP	0.9018	1.0385	0.8386	0.9402	0.9298	0.0419
DQX1	0.8464	1.0032	1.0223	0.8473	0.9298	0.0480
LOC645966	0.9082	1.0524	0.9309	0.8277	0.9298	0.0465
ZNF227	0.8625	1.0220	1.0623	0.7725	0.9298	0.0679
LOC650611	0.9508	0.9422	0.9198	0.9064	0.9298	0.0102
LOC648218	0.9347	0.9760	0.8605	0.9480	0.9298	0.0246
GTPBP8	0.7225	1.3099	0.9783	0.7086	0.9298	0.1410
USP48	0.8004	1.1759	0.9231	0.8200	0.9298	0.0863
LOC649841	0.9445	0.9660	0.9536	0.8553	0.9298	0.0252
PIP5K3	0.8434	1.0545	0.8961	0.9255	0.9299	0.0449
MPP1	0.7469	1.0993	0.9989	0.8744	0.9299	0.0764
MKS1	0.8357	1.0874	0.9133	0.8831	0.9299	0.0549
OR1L8	1.0865	0.9206	0.9472	0.7652	0.9299	0.0658
LOC1001291	0.8476	1.1209	0.8578	0.8933	0.9299	0.0644
LOC642966	0.7455	1.1584	1.0008	0.8149	0.9299	0.0933
LOC728558	0.8620	1.1386	0.8795	0.8394	0.9299	0.0701
LOC645355	0.8571	1.0994	0.8435	0.9196	0.9299	0.0589
FKBP1B	0.7049	0.9730	1.1152	0.9266	0.9299	0.0851

LOC441135	0.9072	1.1110	0.8457	0.8559	0.9299	0.0618
AGAP7	0.8338	1.0047	0.9694	0.9119	0.9299	0.0373
LOC642711	0.9788	0.9627	1.0289	0.7495	0.9300	0.0618
TAS2R14	0.8726	1.0031	0.9189	0.9252	0.9300	0.0271
TCF4	0.9301	1.0858	0.8848	0.8191	0.9300	0.0567
LOC645431	0.8141	1.0092	0.9699	0.9267	0.9300	0.0421
MGC12538	0.9686	1.0078	0.9375	0.8061	0.9300	0.0437
PTPN6	0.8176	1.2805	0.8171	0.8048	0.9300	0.1169
SOLH	0.9359	1.0538	0.8550	0.8754	0.9300	0.0447
LOC728353	0.9422	0.8919	1.0365	0.8495	0.9300	0.0402
MGST1	0.7548	1.2956	0.9681	0.7015	0.9300	0.1348
IL1RL2	0.7943	1.0469	0.9008	0.9782	0.9301	0.0542
MCL1	0.9011	1.2513	0.9395	0.6284	0.9301	0.1275
VWA5B1	0.8820	0.9181	1.0648	0.8553	0.9301	0.0467
C6orf89	0.8314	1.1562	0.9362	0.7965	0.9301	0.0810
SOHLH1	0.9901	0.9694	0.8162	0.9446	0.9301	0.0391
MIR532	0.8678	1.1218	0.9539	0.7768	0.9301	0.0734
OR52N5	0.8288	1.0467	0.9158	0.9291	0.9301	0.0448
F8A3	0.8651	1.1476	0.9493	0.7585	0.9301	0.0823
MBLAC1	1.0355	1.1498	0.7921	0.7430	0.9301	0.0972
LOC642965	0.8412	1.1325	1.0521	0.6947	0.9301	0.0996
LOC440607	0.8879	1.0578	0.9303	0.8445	0.9301	0.0460
LOC1001287	0.8337	0.9350	1.0423	0.9096	0.9301	0.0431
LOC1001288	0.8732	1.1292	0.8841	0.8341	0.9301	0.0672
FLJ13614	0.7940	1.1417	0.9185	0.8663	0.9301	0.0750
NDUFAF3	0.7460	1.2165	1.0406	0.7175	0.9301	0.1202
RPS10P3	0.8615	1.1180	1.0456	0.6954	0.9301	0.0951
PARS2	0.8695	1.1371	0.9555	0.7585	0.9301	0.0799
LOC653781	0.8402	1.0621	0.9188	0.8996	0.9301	0.0470
LOC647058	0.8788	0.9707	0.9408	0.9304	0.9302	0.0191
FLJ38377	0.8811	1.0608	0.9404	0.8385	0.9302	0.0483
FBF1	0.8962	0.9681	0.9428	0.9137	0.9302	0.0159
MYLK2	0.7524	1.1836	0.9338	0.8512	0.9302	0.0922
LOC642213	0.8599	1.0260	1.0214	0.8136	0.9302	0.0548
LRP4	0.9283	0.9284	0.9416	0.9228	0.9303	0.0040
LOC730134	0.8558	1.0157	0.9978	0.8518	0.9303	0.0443
LOC646149	0.8937	1.0277	0.9734	0.8263	0.9303	0.0443
FIBCD1	1.0211	0.8921	0.8793	0.9286	0.9303	0.0320
LOC649497	0.8733	0.8911	1.0549	0.9019	0.9303	0.0420
SNORD14A	0.8647	1.0671	0.9777	0.8116	0.9303	0.0573
LOC388181	0.9416	1.0255	0.9055	0.8486	0.9303	0.0371
RGS22	0.8621	1.0151	0.9961	0.8481	0.9303	0.0437
SPOPL	0.8343	1.0590	0.9693	0.8588	0.9303	0.0520
LOC1001289	0.9969	1.0357	0.8685	0.8204	0.9304	0.0512
TNFRSF10A	0.7398	1.1911	0.9794	0.8112	0.9304	0.1004
LOC1001336	0.8797	0.9946	0.9015	0.9458	0.9304	0.0254

TCP10	0.8815	1.0438	0.9599	0.8364	0.9304	0.0456
LOC1001305	0.8930	1.0126	0.9831	0.8330	0.9304	0.0413
TAS2R3	0.9185	1.0300	0.9264	0.8469	0.9304	0.0377
LOC647044	0.9441	1.0559	0.9124	0.8094	0.9304	0.0507
C18orf18	0.8242	0.9613	1.0471	0.8892	0.9305	0.0479
HCRT	0.8293	1.0653	0.9328	0.8944	0.9305	0.0498
FXYD5	0.8143	1.1303	0.8984	0.8789	0.9305	0.0690
C14orf174	0.8638	1.0591	0.9958	0.8032	0.9305	0.0588
LOC1001320	0.9762	0.9936	0.9055	0.8465	0.9305	0.0339
FLJ43860	0.8759	0.9588	0.9896	0.8977	0.9305	0.0264
HHLA2	0.8894	1.0037	0.9149	0.9139	0.9305	0.0251
CSAG3	0.8723	1.1316	1.0594	0.6587	0.9305	0.1058
LOC1001300	0.7238	1.0987	1.1205	0.7790	0.9305	0.1041
LOC653377	0.8798	1.0600	1.1386	0.6436	0.9305	0.1099
CSPG4LYP1	0.8224	1.0568	0.9970	0.8459	0.9305	0.0572
ATP10B	0.9167	0.9927	0.9740	0.8387	0.9305	0.0346
ACSBG1	0.9737	1.0888	0.8608	0.7988	0.9305	0.0640
OR10H5	0.9341	1.0584	0.9362	0.7935	0.9306	0.0542
FGF4	0.9084	0.9687	1.0178	0.8274	0.9306	0.0410
CNTN2	0.8812	1.1202	0.9037	0.8171	0.9306	0.0658
LOC283440	0.9184	0.8589	1.0511	0.8939	0.9306	0.0420
AP1G2	0.8921	1.1640	0.9117	0.7545	0.9306	0.0853
UBXN11	0.8868	1.0100	0.9529	0.8726	0.9306	0.0317
SNORA23	0.8239	1.0458	0.9874	0.8653	0.9306	0.0518
LOC643703	0.6079	1.1070	1.1072	0.9004	0.9306	0.1181
LOC727811	0.9280	0.9614	1.0516	0.7815	0.9306	0.0561
LOC729393	0.8569	1.0561	0.9140	0.8954	0.9306	0.0435
LOC1001327	0.8657	1.0327	0.9175	0.9066	0.9306	0.0358
ICAM1	0.8921	1.0162	0.9121	0.9021	0.9306	0.0288
RSF1	0.8426	1.0358	0.9040	0.9401	0.9306	0.0404
LOC646154	0.9249	0.9704	0.9527	0.8746	0.9306	0.0209
LOC653750	0.9318	0.9433	0.9101	0.9373	0.9306	0.0072
PIP4K2B	0.7847	1.1063	0.9887	0.8430	0.9306	0.0726
FAM108A1	0.7742	1.0552	1.0775	0.8157	0.9306	0.0789
NCOA5	0.8861	1.2169	0.8749	0.7448	0.9307	0.1007
NBPF15	0.8967	1.0470	0.9525	0.8264	0.9307	0.0466
OLFML2A	0.8003	1.0413	1.0130	0.8679	0.9307	0.0577
LOC440180	0.9397	0.8966	1.0053	0.8811	0.9307	0.0278
ZMAT4	0.9221	1.0431	0.9421	0.8154	0.9307	0.0467
OR5D13	0.8459	1.0227	0.9554	0.8987	0.9307	0.0380
BNIP2	0.7423	1.3624	0.9854	0.6327	0.9307	0.1617
HS3ST4	0.8193	1.0760	0.9443	0.8831	0.9307	0.0548
C8A	0.8633	0.9627	0.9648	0.9320	0.9307	0.0237
ZNF565	0.8903	1.1263	0.9272	0.7790	0.9307	0.0724
LOC649355	0.9354	0.9646	1.0358	0.7871	0.9307	0.0523
PRR19	0.8192	1.0610	1.0377	0.8049	0.9307	0.0687

LOC1001294	0.8892	1.0095	0.9947	0.8294	0.9307	0.0431
SNORD12	0.7846	1.0321	0.9550	0.9513	0.9307	0.0522
ZSCAN5A	0.7724	1.3106	0.8624	0.7775	0.9307	0.1283
SLC36A1	0.9565	0.9987	0.9010	0.8667	0.9307	0.0292
EXOC8	0.8525	0.9787	0.9828	0.9091	0.9308	0.0311
TGFBR3	0.8822	1.2170	0.9523	0.6717	0.9308	0.1125
ST7OT3	0.8931	1.0128	0.9582	0.8590	0.9308	0.0342
ZFR	0.9082	1.0784	1.0611	0.6755	0.9308	0.0933
LOC642132	0.9444	1.0705	0.9095	0.7988	0.9308	0.0560
CMPK2	0.9071	0.9576	0.9515	0.9070	0.9308	0.0138
TMEM180	0.8899	1.0984	0.9383	0.7967	0.9308	0.0631
KIAA1614	0.9728	0.8703	0.9970	0.8833	0.9309	0.0317
C20orf66	0.8936	1.0218	0.9406	0.8674	0.9309	0.0339
ICA1	0.8936	1.2854	0.8440	0.7004	0.9309	0.1251
POTE2	0.9209	1.0170	0.9316	0.8541	0.9309	0.0335
LOC728612	0.9488	1.0025	0.9550	0.8173	0.9309	0.0397
C1orf120	0.9414	1.0843	0.9193	0.7787	0.9309	0.0625
C11orf42	0.8973	1.1090	0.8556	0.8617	0.9309	0.0601
LOC642334	0.8381	1.0414	1.0244	0.8197	0.9309	0.0591
LOC653066	0.9210	1.0452	0.9399	0.8175	0.9309	0.0466
C10orf6	0.8018	1.1602	0.9072	0.8545	0.9309	0.0794
LOC644948	0.9513	1.0266	0.9049	0.8410	0.9309	0.0391
SMAD5	0.7993	1.1611	0.9856	0.7777	0.9309	0.0898
CTF1	0.9586	1.0022	0.9926	0.7705	0.9310	0.0543
LOC648639	1.0235	0.9368	0.8879	0.8757	0.9310	0.0336
GFI1B	0.8358	1.0491	0.9797	0.8592	0.9310	0.0505
C3orf71	0.9535	1.1608	0.9094	0.7002	0.9310	0.0944
MIR320A	0.9078	1.0651	0.9162	0.8349	0.9310	0.0483
CD207	0.9072	1.0554	0.9423	0.8190	0.9310	0.0489
SCRN3	0.8011	1.1544	0.9820	0.7865	0.9310	0.0867
C1QTNF4	0.8703	1.0158	0.9376	0.9003	0.9310	0.0315
LOC644679	0.8735	1.0357	1.0068	0.8081	0.9310	0.0541
C6orf58	0.9305	1.0506	1.0152	0.7278	0.9310	0.0723
ZNF528	0.8815	0.9105	1.0158	0.9164	0.9310	0.0293
DEPDC6	0.7896	1.1826	0.9478	0.8042	0.9310	0.0911
LOC1001286	0.9931	1.0215	0.9041	0.8054	0.9310	0.0488
LOC648620	0.8780	1.0933	0.9132	0.8397	0.9311	0.0561
C4orf23	0.9430	1.0967	0.9165	0.7682	0.9311	0.0673
LOC653325	0.9950	1.0575	0.7972	0.8748	0.9311	0.0586
MIER1	0.8652	1.1234	0.9584	0.7774	0.9311	0.0740
ANGEL2	0.8762	1.2088	0.9276	0.7119	0.9311	0.1034
BCDIN3D	0.8147	1.4706	0.8328	0.6064	0.9311	0.1870
CACNA1S	0.9053	1.1069	0.9583	0.7541	0.9312	0.0728
HARS2	0.9399	1.0985	0.9276	0.7586	0.9312	0.0694
BIN1	0.7704	1.2874	0.8802	0.7866	0.9312	0.1212
HAX1	0.8042	1.3337	0.9242	0.6625	0.9312	0.1445

MIR1323	0.8877	1.0166	0.9756	0.8447	0.9312	0.0394
LOC644736	0.8561	1.1750	0.8566	0.8370	0.9312	0.0814
SMN1	0.8190	1.0277	1.0729	0.8051	0.9312	0.0694
CSNK1D	0.8186	1.2263	0.9427	0.7372	0.9312	0.1071
LOC652034	0.9684	0.9433	0.9568	0.8562	0.9312	0.0255
KIAA0125	0.9052	0.9511	0.9994	0.8690	0.9312	0.0283
ZNF136	0.8377	1.0829	0.9679	0.8363	0.9312	0.0592
AARS	0.9616	1.2307	0.8348	0.6977	0.9312	0.1134
CT45A6	0.8439	1.0915	0.9488	0.8406	0.9312	0.0590
DENND2A	0.8063	1.0061	1.0237	0.8888	0.9312	0.0513
XKR4	0.9510	1.1133	0.8874	0.7732	0.9312	0.0710
MBTD1	0.7728	1.0905	1.0196	0.8420	0.9312	0.0743
DEFB103A	0.9785	0.9981	0.8969	0.8515	0.9312	0.0345
PDGFC	0.8496	1.0836	0.8892	0.9026	0.9312	0.0520
RPN1	0.7785	1.3087	1.0575	0.5803	0.9313	0.1594
NDOR1	0.8652	1.0401	0.9615	0.8582	0.9313	0.0433
LOC647466	0.8725	1.0070	0.9041	0.9414	0.9313	0.0289
FOXJ3	0.8414	1.1998	0.9126	0.7713	0.9313	0.0940
TAS2R13	0.8402	1.0124	1.0033	0.8692	0.9313	0.0446
LOC1001302	0.8833	0.9952	1.0180	0.8286	0.9313	0.0451
OR1M1	0.8477	1.0422	0.8969	0.9383	0.9313	0.0413
POLR2J2	0.8459	1.0375	1.0921	0.7498	0.9313	0.0803
LOC286157	0.8631	1.1561	0.9234	0.7827	0.9313	0.0803
LOC728183	0.8847	1.0856	0.9332	0.8217	0.9313	0.0563
B3GNT9	0.9239	1.0770	0.8884	0.8360	0.9313	0.0518
KCNK5	0.9155	1.0841	0.9640	0.7617	0.9313	0.0667
LOC642660	0.7405	1.0215	0.9867	0.9767	0.9313	0.0643
ZBTB37	0.8774	1.0372	0.9775	0.8332	0.9313	0.0464
FGFR4	0.9018	1.0480	0.9664	0.8093	0.9314	0.0505
LOC1001313	0.9013	0.9998	0.9204	0.9039	0.9314	0.0232
CDKL3	0.8197	1.1393	0.9431	0.8233	0.9314	0.0750
NCF1B	0.8428	1.0041	1.0083	0.8702	0.9314	0.0436
MIR1204	0.7442	1.0825	0.9466	0.9521	0.9314	0.0698
LOC387753	0.8340	1.0012	1.0325	0.8578	0.9314	0.0500
MIR581	1.0174	0.9641	0.9232	0.8208	0.9314	0.0416
LOC286002	0.9281	0.9560	0.9841	0.8573	0.9314	0.0272
LOC1001344	0.9882	0.9529	0.9222	0.8623	0.9314	0.0267
KIF19	0.8697	0.9673	0.9874	0.9013	0.9314	0.0276
TMEM183A	0.6998	1.2645	1.0384	0.7229	0.9314	0.1352
ZNF443	0.8214	1.1020	0.9533	0.8490	0.9314	0.0636
LOC728648	0.9753	0.9140	0.9379	0.8985	0.9314	0.0167
LOC1001314	0.8462	1.0210	0.9626	0.8960	0.9314	0.0382
LOC644222	0.8618	1.1176	0.9087	0.8376	0.9314	0.0638
FGF23	0.8173	1.1399	0.9355	0.8330	0.9314	0.0743
MINPP1	0.7836	1.0944	1.0772	0.7707	0.9315	0.0892
LOC1001299	0.8889	0.9965	0.9531	0.8875	0.9315	0.0265

LOC728241	0.8985	1.0298	0.9444	0.8531	0.9315	0.0377
TMEM183B	0.6999	1.2536	1.0335	0.7389	0.9315	0.1307
TALDO1	0.7311	1.2476	0.9773	0.7699	0.9315	0.1184
ZBTB11	0.9152	1.0209	0.9104	0.8795	0.9315	0.0308
LOC641823	0.8234	1.1441	0.9465	0.8120	0.9315	0.0771
LOC645552	0.9145	1.0165	1.0601	0.7350	0.9315	0.0723
PPA2	0.7865	1.2430	1.0329	0.6636	0.9315	0.1291
LOC645459	0.9016	1.0698	1.0126	0.7421	0.9315	0.0722
CEP27	0.7032	1.1750	1.0060	0.8419	0.9315	0.1020
SCG5	0.7948	1.0601	0.9289	0.9423	0.9315	0.0543
ANTXR1	0.8697	1.0960	0.9630	0.7974	0.9315	0.0645
GRK5	0.7350	1.1916	0.9659	0.8337	0.9315	0.0987
LOC651577	0.8086	1.0052	1.0650	0.8474	0.9315	0.0615
HOXB2	0.8302	0.9369	1.0234	0.9357	0.9316	0.0395
USP36	0.8285	1.1947	0.8946	0.8084	0.9316	0.0896
LOC440280	0.9531	0.9775	1.0313	0.7643	0.9316	0.0581
LOC645492	0.8943	1.0392	0.9713	0.8215	0.9316	0.0471
LRRC14B	0.9004	1.0663	0.8511	0.9086	0.9316	0.0467
LOC649971	0.8990	1.0331	0.9380	0.8563	0.9316	0.0377
LOC1001313	0.9297	0.9720	0.9526	0.8722	0.9316	0.0216
DCAF15	0.9288	1.0818	0.9020	0.8140	0.9316	0.0557
EMR4P	1.0019	0.9837	0.8913	0.8497	0.9316	0.0365
LOC647785	0.8414	0.9677	0.9727	0.9448	0.9316	0.0307
LOC644117	0.9705	0.9170	0.9806	0.8584	0.9317	0.0281
LOC647070	0.8412	1.0417	0.8723	0.9715	0.9317	0.0460
HSPA1A	0.9617	0.9586	0.9553	0.8511	0.9317	0.0269
LOC651208	0.8664	0.9739	1.0648	0.8215	0.9317	0.0547
LOC643094	0.8698	0.9864	0.9412	0.9295	0.9317	0.0240
ZBTB40	0.8910	1.0155	1.0078	0.8126	0.9317	0.0489
ACSF3	0.8596	1.0626	0.9354	0.8692	0.9317	0.0468
LOC651125	0.9697	0.9835	0.8744	0.8993	0.9317	0.0265
POLA	0.8720	1.0847	0.8426	0.9275	0.9317	0.0540
LOC728454	0.9032	0.9518	1.0000	0.8718	0.9317	0.0281
CBLN1	0.8995	0.9802	0.9833	0.8639	0.9318	0.0298
LOC731718	0.9575	1.0217	1.0376	0.7103	0.9318	0.0758
TC2N	0.8217	1.1897	1.0635	0.6522	0.9318	0.1205
LOC441453	0.8207	1.0961	0.9492	0.8612	0.9318	0.0610
SERPINH1	0.8791	1.0033	0.9635	0.8814	0.9318	0.0309
PRRX2	0.8751	0.9307	1.0074	0.9140	0.9318	0.0278
RPL7	0.7990	1.0733	1.2624	0.5925	0.9318	0.1478
AREGB	0.9278	0.9698	0.9467	0.8828	0.9318	0.0184
C11orf72	0.8561	1.0992	0.9638	0.8082	0.9318	0.0646
GAB4	0.8838	1.0714	0.9662	0.8060	0.9318	0.0569
LOC392197	0.9726	0.9459	0.8921	0.9168	0.9318	0.0175
FLVCR2	0.8153	1.2626	0.9246	0.7249	0.9318	0.1176
CCL19	0.9403	0.9916	0.9298	0.8657	0.9318	0.0259

KIAA0240	0.8535	1.0856	1.0000	0.7883	0.9319	0.0677
MRM1	0.8598	0.9689	1.0165	0.8823	0.9319	0.0367
LOC401895	0.8984	1.0635	0.8518	0.9138	0.9319	0.0458
RHBDF2	0.7834	1.1638	0.9748	0.8056	0.9319	0.0883
LOC647706	0.9148	1.0166	0.9756	0.8207	0.9319	0.0426
LOC652688	0.9334	0.8929	0.9769	0.9245	0.9319	0.0173
OCLN	0.8427	1.0858	0.9617	0.8376	0.9319	0.0588
LOC1001280	0.7651	1.1086	1.0345	0.8197	0.9319	0.0827
AFAP1L2	0.8494	1.0613	0.9032	0.9140	0.9320	0.0454
MIR187	0.7701	1.0478	1.0268	0.8832	0.9320	0.0652
LOC651280	0.8623	1.0025	0.9919	0.8712	0.9320	0.0378
GIYD1	0.8704	1.0857	1.0013	0.7705	0.9320	0.0697
LOC649362	0.9162	0.9417	0.9596	0.9104	0.9320	0.0114
LOC151121	0.9154	0.8959	1.0214	0.8951	0.9320	0.0302
LOC1001313	0.9299	0.9483	1.0221	0.8277	0.9320	0.0401
FKBP6	0.8701	1.0820	0.9579	0.8180	0.9320	0.0577
LOC402221	0.8376	1.1995	1.0723	0.6186	0.9320	0.1286
LOC647584	0.9080	0.8586	1.0123	0.9492	0.9320	0.0325
LOC1001333	0.8566	1.0616	0.9504	0.8594	0.9320	0.0484
LOC1001346	0.8913	1.0552	0.9685	0.8131	0.9320	0.0519
GPR87	0.8733	1.0368	0.9316	0.8865	0.9320	0.0371
LOC653362	0.7659	0.9493	1.1587	0.8542	0.9320	0.0843
PROK2	0.9456	0.9986	1.0284	0.7557	0.9321	0.0612
SP110	0.7738	1.2007	0.9924	0.7613	0.9321	0.1041
CUTA	0.8651	1.3222	0.8426	0.6984	0.9321	0.1352
CD80	0.8073	1.0532	0.9514	0.9164	0.9321	0.0507
MAGEH1	0.8572	1.0628	0.9229	0.8854	0.9321	0.0456
LOC643664	0.9373	1.0355	0.9370	0.8186	0.9321	0.0444
HIGD2B	0.9436	0.9429	0.9674	0.8745	0.9321	0.0200
WIPF3	1.0892	0.9170	0.8148	0.9075	0.9321	0.0572
LOC641808	0.8216	1.0605	1.0150	0.8313	0.9321	0.0617
NCRNA0016	0.9075	0.9816	0.9459	0.8935	0.9321	0.0199
LOC1001329	0.9264	1.0114	0.9494	0.8414	0.9321	0.0352
LOC131873	0.8897	1.0050	0.9474	0.8865	0.9321	0.0280
KIAA1529	0.9009	1.0141	1.0889	0.7247	0.9322	0.0792
HERC6	0.7592	1.3248	0.8922	0.7525	0.9322	0.1348
RELB	0.9014	0.9716	0.9868	0.8690	0.9322	0.0281
LOC1001331	0.8020	1.0443	0.9982	0.8842	0.9322	0.0549
KIAA1549	0.9087	1.0017	1.0429	0.7756	0.9322	0.0593
KARS	0.6173	1.2815	1.0911	0.7390	0.9322	0.1538
C6orf132	0.7979	1.1982	0.9947	0.7382	0.9322	0.1042
LOC729227	0.8786	1.0044	0.9517	0.8943	0.9323	0.0287
SPANXN3	0.8113	1.1372	0.9003	0.8803	0.9323	0.0709
FAM90A20	0.9115	1.0068	0.9979	0.8131	0.9323	0.0452
MAP3K10	0.8814	0.9429	1.0665	0.8385	0.9323	0.0496
N4BP2	0.6746	1.1750	1.0234	0.8562	0.9323	0.1078

SLC25A18	0.8032	1.0058	1.0079	0.9124	0.9323	0.0485
LOC441511	0.8338	1.1018	1.0607	0.7331	0.9324	0.0888
LOC728103	0.9284	0.9672	0.9873	0.8465	0.9324	0.0311
CYP51A1	0.8780	1.0180	1.0405	0.7929	0.9324	0.0588
S100A7	0.9116	0.9491	0.9849	0.8839	0.9324	0.0220
BCOR	0.8617	1.2945	0.8468	0.7265	0.9324	0.1244
LOC647456	0.8709	1.1553	0.8388	0.8646	0.9324	0.0746
ASS1	0.9453	1.1801	0.9005	0.7037	0.9324	0.0978
LOC440957	0.7622	1.2867	0.9539	0.7267	0.9324	0.1282
C4orf50	0.8912	1.1034	0.8752	0.8597	0.9324	0.0574
LOC1001320	0.9375	0.9682	1.0108	0.8132	0.9324	0.0425
LOC642646	1.0226	0.8981	0.9276	0.8814	0.9324	0.0315
DDX6	0.7682	1.1526	1.0765	0.7324	0.9324	0.1065
PLIN	0.9245	1.0311	0.8686	0.9055	0.9324	0.0349
C15orf38	0.8661	1.0870	1.0271	0.7494	0.9324	0.0768
NFKBIB	0.8617	1.0468	1.0008	0.8204	0.9324	0.0542
RAD50	0.9060	1.1118	0.8875	0.8245	0.9324	0.0623
TMEM174	1.0714	0.8943	0.9270	0.8371	0.9324	0.0499
ZNF557	0.7897	1.1684	0.9546	0.8170	0.9324	0.0865
LIFR	0.8453	1.1490	0.8904	0.8451	0.9325	0.0730
TMEM71	0.9016	0.9743	0.9490	0.9050	0.9325	0.0176
LOC645852	0.8989	1.0871	0.9304	0.8134	0.9325	0.0572
FAM21D	0.8494	1.0640	0.9842	0.8324	0.9325	0.0554
PDCD2L	0.7001	1.3252	0.9053	0.7993	0.9325	0.1375
MRPS25	0.7951	1.0099	1.0841	0.8407	0.9325	0.0685
LOC649428	0.8597	0.9589	0.9830	0.9284	0.9325	0.0267
LOC732441	0.9071	0.9582	0.9878	0.8769	0.9325	0.0249
LOC642773	0.8548	1.1520	0.9141	0.8091	0.9325	0.0763
NOD3	0.8663	0.9172	1.0110	0.9355	0.9325	0.0300
PCYT1B	0.9313	1.0358	1.0028	0.7601	0.9325	0.0615
C19orf71	0.7953	0.8978	1.0869	0.9501	0.9325	0.0607
LOC646808	0.8996	1.0470	0.9906	0.7929	0.9325	0.0556
USP29	0.7979	0.9970	1.0424	0.8930	0.9326	0.0547
NOVA1	0.8176	1.0722	0.9978	0.8427	0.9326	0.0613
LOC1001324	0.9437	1.0386	0.8898	0.8582	0.9326	0.0395
MIR764	0.9447	1.0901	0.8506	0.8451	0.9326	0.0573
MYO9A	0.8618	1.2667	0.8267	0.7751	0.9326	0.1128
CMTM3	0.8101	1.0152	0.9901	0.9150	0.9326	0.0460
LOC440459	0.8006	1.0781	1.0199	0.8318	0.9326	0.0685
LOC148709	0.8586	0.9591	1.0048	0.9079	0.9326	0.0316
LOC1001296	0.8718	1.0902	0.9442	0.8241	0.9326	0.0581
U2AF1	0.7868	1.2251	0.9703	0.7482	0.9326	0.1089
LOC606724	0.9111	0.9858	0.9512	0.8824	0.9326	0.0227
LOC646936	0.7966	1.1041	0.9501	0.8796	0.9326	0.0652
LOC1001291	0.8078	1.0687	0.9759	0.8780	0.9326	0.0570
LOC647357	0.8881	1.0166	0.9148	0.9109	0.9326	0.0286

HCP5	0.8558	1.1889	0.8797	0.8060	0.9326	0.0868
MIR523	0.9205	0.9744	1.0060	0.8296	0.9326	0.0386
LOC649923	0.8613	0.8512	1.0538	0.9643	0.9327	0.0478
FLJ38773	0.9526	0.9099	0.9875	0.8807	0.9327	0.0235
NDUFA13	0.7537	1.2685	0.9806	0.7278	0.9327	0.1255
FLJ40434	0.9020	0.9786	0.9621	0.8880	0.9327	0.0222
ASCL2	0.8185	0.8806	1.1213	0.9103	0.9327	0.0657
TTTY18	0.8392	0.9681	1.0289	0.8946	0.9327	0.0415
LOC162073	0.8487	1.0003	0.9657	0.9161	0.9327	0.0329
SLC4A1AP	0.8601	1.2482	0.9226	0.6999	0.9327	0.1151
CYP2A13	0.8164	0.9818	1.0518	0.8808	0.9327	0.0523
ERCC-00123	0.8857	0.9409	0.9850	0.9194	0.9327	0.0208
ACTA1	0.8442	0.9723	0.9849	0.9296	0.9327	0.0318
LOC643577	0.9590	0.9296	0.9417	0.9007	0.9327	0.0123
LOC442403	0.9568	0.9816	0.9426	0.8500	0.9327	0.0287
LOC650557	0.9031	0.9722	0.9723	0.8835	0.9328	0.0231
LOC1001307	0.8591	1.3249	0.8993	0.6477	0.9328	0.1419
CYP26B1	0.8993	1.0015	0.9689	0.8613	0.9328	0.0319
BTN3A1	0.8331	1.1006	0.9776	0.8197	0.9328	0.0664
LOC643763	0.9626	0.9605	0.9880	0.8200	0.9328	0.0381
MAMLD1	0.7350	1.1470	0.9132	0.9359	0.9328	0.0844
NSUN4	0.9034	1.1127	1.0371	0.6779	0.9328	0.0953
FAM187B	0.9522	0.9571	0.9002	0.9217	0.9328	0.0134
GLRA4	0.9169	1.0871	0.9267	0.8005	0.9328	0.0589
ZNF674	0.7407	1.3440	0.9330	0.7135	0.9328	0.1455
LOC642815	0.8284	1.1052	0.9726	0.8250	0.9328	0.0670
IQCB1	0.9074	1.1408	0.9316	0.7514	0.9328	0.0800
LOC647359	0.7733	1.1805	0.8580	0.9194	0.9328	0.0878
RBP5	0.8994	1.1098	0.8994	0.8225	0.9328	0.0617
PSMD13	0.7342	1.2653	1.0257	0.7060	0.9328	0.1323
TARS2	0.8315	1.0322	0.9984	0.8692	0.9328	0.0487
SLC35E4	0.9559	0.8967	1.0733	0.8054	0.9328	0.0561
LOC731714	0.9445	1.0820	0.8419	0.8630	0.9328	0.0544
PHF14	0.8211	1.2448	1.0292	0.6362	0.9328	0.1314
PCDHA2	0.8971	1.0421	1.0292	0.7629	0.9328	0.0654
LOC200726	0.8454	0.9847	1.0353	0.8660	0.9328	0.0459
ZNF187	0.8118	1.0901	1.0317	0.7979	0.9329	0.0749
LOC648394	0.8703	1.0735	0.8930	0.8946	0.9329	0.0472
LOC1001337	0.8553	1.0580	0.9786	0.8396	0.9329	0.0520
PSMD5	0.9726	0.9034	1.0214	0.8341	0.9329	0.0409
SP4	0.7894	1.1261	1.1165	0.6995	0.9329	0.1103
ZIC3	0.8094	1.0444	0.9677	0.9101	0.9329	0.0495
LOC1001336	0.8113	0.9976	1.0136	0.9090	0.9329	0.0466
ALDOA	0.7344	1.3515	0.9402	0.7055	0.9329	0.1490
KIAA1024	0.8906	1.0162	0.9272	0.8975	0.9329	0.0289
B3GNT7	0.8961	1.1260	0.9173	0.7923	0.9329	0.0699

SLA	0.9419	0.9990	0.9104	0.8804	0.9329	0.0254
CYP4F2	0.8661	1.0726	0.9053	0.8878	0.9329	0.0472
ZSCAN20	0.9428	0.9630	0.9550	0.8709	0.9329	0.0211
KRTAP21-1	0.8156	0.9679	1.0771	0.8712	0.9329	0.0574
TMEM44	1.0089	1.0594	0.8728	0.7907	0.9329	0.0617
LOC653787	0.8607	0.9865	0.9371	0.9476	0.9329	0.0263
TCF20	0.8082	1.1613	0.9327	0.8297	0.9329	0.0808
MED12L	0.8683	0.9718	1.0250	0.8667	0.9330	0.0393
LOC642962	0.9693	0.9806	0.9062	0.8757	0.9330	0.0251
C20orf111	0.8030	1.2013	1.0291	0.6985	0.9330	0.1130
CHIC1	0.8029	1.0934	0.9958	0.8399	0.9330	0.0679
CFC1B	0.8393	1.0747	0.9720	0.8459	0.9330	0.0562
LOC400696	0.8828	0.9952	1.0056	0.8483	0.9330	0.0396
C19orf46	0.8483	1.3700	0.8484	0.6652	0.9330	0.1519
LOC151174	0.9136	1.0470	0.9178	0.8537	0.9330	0.0407
LOC730995	0.9106	1.0296	0.9194	0.8723	0.9330	0.0338
DOCK1	0.9590	1.1092	0.8618	0.8021	0.9330	0.0670
LOC440928	0.8534	1.1609	1.0112	0.7066	0.9330	0.0982
PRSS48	0.9230	1.0061	0.9845	0.8184	0.9330	0.0420
LOC645636	0.8998	0.8747	1.0099	0.9477	0.9330	0.0298
LOC653211	1.0150	1.0480	0.8911	0.7781	0.9330	0.0617
TEX101	0.7840	0.9966	0.9531	0.9984	0.9330	0.0508
PHOX2B	0.8531	1.0311	1.0101	0.8378	0.9330	0.0508
SURF4	0.9099	1.3037	0.7722	0.7464	0.9330	0.1287
LOC730294	0.9267	0.9255	0.9939	0.8860	0.9330	0.0224
LOC643235	0.8690	1.1005	0.9189	0.8438	0.9331	0.0580
LOC652668	0.9016	1.0538	0.9703	0.8066	0.9331	0.0524
FLJ37307	0.7956	1.1013	0.9662	0.8692	0.9331	0.0661
RABL5	1.0884	1.1142	0.7987	0.7311	0.9331	0.0982
LOC1000085	0.5440	0.8096	1.2298	1.1490	0.9331	0.1585
LOC1001297	0.8453	1.0270	1.0979	0.7622	0.9331	0.0779
LOC652755	0.9024	1.2717	0.7433	0.8150	0.9331	0.1175
LOC728190	0.8447	1.0400	1.0539	0.7938	0.9331	0.0666
MTF2	0.7883	1.1744	1.1134	0.6565	0.9331	0.1252
ARRDC5	0.8771	1.0618	1.0104	0.7832	0.9331	0.0633
C20orf106	0.8924	1.0037	0.9820	0.8544	0.9331	0.0356
ARG99	0.9130	0.9759	0.9427	0.9009	0.9331	0.0167
LOC1001285	0.8462	1.0933	0.8959	0.8972	0.9331	0.0547
ZNF728	0.8293	0.9864	1.0125	0.9043	0.9331	0.0416
ACOT4	0.7750	1.1694	0.9144	0.8739	0.9332	0.0840
BRF1	0.9055	1.0448	0.9209	0.8615	0.9332	0.0393
LOC728844	0.8643	1.0327	0.9521	0.8838	0.9332	0.0381
ACSL4	0.8136	0.9947	1.0778	0.8467	0.9332	0.0622
SCN9A	0.9651	0.9921	0.9312	0.8444	0.9332	0.0321
LOC1001287	0.9342	1.0365	0.8924	0.8698	0.9332	0.0369
PLCD3	0.9356	1.0131	0.9401	0.8441	0.9332	0.0346

RPL39L	0.8851	1.0098	0.9678	0.8701	0.9332	0.0334
ASB2	0.8163	1.0909	0.8455	0.9802	0.9332	0.0635
TMEM182	0.8324	0.9671	1.0794	0.8540	0.9332	0.0570
SERPINB12	0.8998	0.9411	0.9489	0.9431	0.9332	0.0113
LOC649443	0.9147	0.9937	0.9145	0.9101	0.9332	0.0202
LOC1001304	0.8706	0.9631	0.9703	0.9290	0.9332	0.0228
NF2	0.8798	1.0139	0.9707	0.8686	0.9333	0.0353
LOC1001329	0.8376	1.0545	0.9490	0.8919	0.9333	0.0464
TGFBR2	0.8763	1.0559	0.9500	0.8509	0.9333	0.0460
LMX1B	0.8579	1.0225	0.9929	0.8599	0.9333	0.0434
KCNA4	0.8616	1.0776	0.9719	0.8222	0.9333	0.0576
PPP1R14D	0.8711	0.9517	1.0626	0.8479	0.9333	0.0485
LEKR1	0.8016	1.0743	1.0484	0.8091	0.9333	0.0741
LOC1001305	0.9147	1.0616	0.9137	0.8434	0.9334	0.0459
USP31	0.9019	1.0692	0.9143	0.8481	0.9334	0.0475
ZNF394	0.7670	1.0899	1.0624	0.8141	0.9334	0.0832
TRIM64	0.8566	1.0427	1.0156	0.8186	0.9334	0.0561
LILRA4	0.8621	1.0883	0.9104	0.8728	0.9334	0.0527
LOC645732	0.8755	1.0062	0.8671	0.9847	0.9334	0.0361
DDX51	0.7678	1.0896	1.0832	0.7929	0.9334	0.0885
MUC6	0.8433	0.9661	1.0073	0.9169	0.9334	0.0353
VPS4A	1.0077	1.0963	0.9659	0.6637	0.9334	0.0939
PRKAA2	0.9009	1.0287	0.9746	0.8294	0.9334	0.0435
TBC1D22B	0.7941	1.1216	0.9559	0.8620	0.9334	0.0710
LOC644113	0.8829	0.9857	1.0335	0.8316	0.9334	0.0463
LOC643197	0.8339	1.0625	0.9531	0.8841	0.9334	0.0495
LOC1001292	0.8976	0.9487	1.0354	0.8519	0.9334	0.0393
MIR181C	0.8762	1.0291	0.9867	0.8416	0.9334	0.0445
GlyBP	0.8595	1.0089	0.9800	0.8853	0.9334	0.0361
PMS2L3	0.9049	1.0128	0.9734	0.8425	0.9334	0.0376
SCARNA23	0.8463	1.0830	1.0144	0.7900	0.9334	0.0690
MGC3020	0.9328	0.9313	0.9646	0.9051	0.9334	0.0122
ZSWIM2	0.8034	1.0447	1.0128	0.8728	0.9334	0.0572
LOC647022	0.9659	0.8400	1.0523	0.8756	0.9334	0.0477
EGFL8	0.8484	0.9816	0.9495	0.9542	0.9335	0.0292
LOC390829	1.0053	0.9382	0.9479	0.8426	0.9335	0.0337
PAR5	0.7897	0.9867	1.1608	0.7968	0.9335	0.0884
LOC648364	0.8348	1.0382	0.9742	0.8867	0.9335	0.0452
NSL1	0.7233	1.1978	1.0650	0.7478	0.9335	0.1175
LOC220998	0.9102	0.8843	1.1055	0.8341	0.9335	0.0595
SNORD3D	0.9767	1.0043	0.9333	0.8199	0.9335	0.0406
RNF152	0.9065	0.9330	1.0311	0.8637	0.9335	0.0355
LOC1001288	0.8072	1.1003	1.0245	0.8023	0.9336	0.0760
ZNF555	0.8791	0.9543	1.0246	0.8763	0.9336	0.0353
C4orf36	0.8821	1.0627	0.9678	0.8216	0.9336	0.0525
LOC642573	0.9479	0.9475	1.0190	0.8200	0.9336	0.0414

C19orf67	0.8796	0.9580	1.0272	0.8695	0.9336	0.0369
LOC727752	0.8475	1.0613	0.9453	0.8802	0.9336	0.0472
LGALS7	0.7606	0.9565	1.0719	0.9453	0.9336	0.0644
C9orf163	0.7856	1.1794	0.9573	0.8121	0.9336	0.0902
NDST3	0.9482	0.9824	0.9262	0.8776	0.9336	0.0220
LOC339879	0.8467	1.0465	0.9585	0.8828	0.9336	0.0443
LOC1001324	0.8421	1.1023	0.9902	0.7998	0.9336	0.0695
P4HTM	0.8272	1.2071	0.9279	0.7722	0.9336	0.0967
C11orf45	0.9353	0.9684	1.0111	0.8196	0.9336	0.0410
COQ4	0.9888	1.0551	0.9126	0.7779	0.9336	0.0595
LOC651754	0.8223	1.0582	0.9893	0.8647	0.9336	0.0546
GCM1	0.8872	1.1005	0.9283	0.8185	0.9336	0.0600
LOC442206	0.8463	1.0315	1.0079	0.8487	0.9336	0.0499
PSG1	0.9140	0.9672	0.9929	0.8606	0.9337	0.0294
LOC729249	0.9434	0.9555	0.9699	0.8658	0.9337	0.0233
ZIM3	0.8182	1.0931	0.9823	0.8412	0.9337	0.0643
LTF	0.8505	0.9202	0.9923	0.9718	0.9337	0.0316
LOC732199	0.9259	0.9414	0.9951	0.8723	0.9337	0.0253
CD72	0.9077	1.0024	0.9051	0.9197	0.9337	0.0231
LOC644813	0.8365	1.0934	0.9512	0.8538	0.9337	0.0589
LOC643550	0.9596	0.9946	0.9809	0.7998	0.9337	0.0452
ZNF385D	0.9008	1.0424	0.9354	0.8564	0.9337	0.0396
TRPV5	0.8440	0.9784	1.0418	0.8709	0.9337	0.0462
LOC645445	0.9418	1.0251	0.9562	0.8120	0.9338	0.0445
GUK1	0.8346	1.3954	0.8425	0.6626	0.9338	0.1594
DOK5	0.8979	1.0315	0.9726	0.8331	0.9338	0.0433
LOC650407	0.9146	1.0914	0.9354	0.7937	0.9338	0.0611
C13orf25	0.8161	0.9612	1.0576	0.9003	0.9338	0.0509
SLC30A6	0.8405	1.2166	0.9297	0.7484	0.9338	0.1013
GCNT2	0.9294	1.1032	0.8694	0.8332	0.9338	0.0599
ADAM11	0.8268	1.0251	0.9804	0.9031	0.9338	0.0437
EDIL3	0.8686	0.9951	0.9843	0.8873	0.9338	0.0326
LOC650263	0.9838	0.9282	0.9727	0.8507	0.9338	0.0302
TRIP4	0.8259	1.2868	0.8704	0.7522	0.9338	0.1202
C8orf77	0.9386	1.0778	0.9436	0.7753	0.9338	0.0619
C11orf20	0.7446	1.1731	0.9787	0.8390	0.9338	0.0931
LOC387756	0.8927	1.0006	1.0855	0.7567	0.9338	0.0710
SNORA70C	0.8437	1.1647	0.9618	0.7652	0.9339	0.0869
C21orf63	0.8178	1.0707	1.0182	0.8288	0.9339	0.0648
FBXL13	0.8332	1.0864	0.9085	0.9072	0.9339	0.0538
ZNF141	0.8823	0.8902	1.0432	0.9198	0.9339	0.0373
LOC647802	0.9695	1.0456	0.8355	0.8849	0.9339	0.0464
KIAA0408	0.7018	1.0253	1.1894	0.8190	0.9339	0.1083
C1orf91	0.8770	1.0741	0.9687	0.8157	0.9339	0.0563
POLR2J	0.8634	1.2878	0.8899	0.6945	0.9339	0.1257
SIRPB2	0.9254	1.0317	0.9040	0.8745	0.9339	0.0342

PCGF1	0.8416	1.2349	0.8538	0.8055	0.9339	0.1008
MESDC1	0.7642	1.1182	0.9942	0.8591	0.9339	0.0775
ZNF365	0.8668	1.1218	0.9481	0.7990	0.9339	0.0697
LOC641999	0.8646	1.1164	0.9262	0.8286	0.9340	0.0641
SPANXC	0.8948	1.0786	0.9562	0.8062	0.9340	0.0572
LOC153684	0.8372	0.9518	0.9694	0.9774	0.9340	0.0327
LOC341378	0.9241	0.9582	0.9964	0.8572	0.9340	0.0295
LOC642497	0.7790	1.0519	1.0124	0.8926	0.9340	0.0618
GNGT2	0.9044	1.0756	0.8909	0.8650	0.9340	0.0479
WDR31	0.8508	1.0778	0.9471	0.8603	0.9340	0.0526
C1orf200	0.9183	0.9719	0.9681	0.8777	0.9340	0.0224
LOC340274	0.8152	1.2338	0.8956	0.7913	0.9340	0.1024
LOC440015	0.8846	1.0301	0.9864	0.8349	0.9340	0.0450
SNORD8	0.7901	1.0749	1.0118	0.8592	0.9340	0.0660
C1orf172	0.8739	1.2816	0.9495	0.6310	0.9340	0.1343
ERCC-00046	0.9300	0.9662	0.9617	0.8782	0.9340	0.0203
LOC645427	0.8476	0.9334	1.0062	0.9489	0.9340	0.0328
C12orf44	0.9615	1.1441	0.8472	0.7834	0.9340	0.0791
TRPA1	0.8827	1.0428	1.0404	0.7703	0.9341	0.0662
LOC647894	0.8562	1.0488	0.9786	0.8526	0.9341	0.0482
LOC1001336	0.8487	1.0301	0.9797	0.8778	0.9341	0.0426
EED	0.8864	1.0153	0.9911	0.8436	0.9341	0.0411
LOC1001344	0.9175	0.9826	0.9305	0.9058	0.9341	0.0169
C19orf15	0.8660	1.0472	0.9967	0.8266	0.9341	0.0524
LOC643853	0.8054	1.0661	1.0483	0.8167	0.9341	0.0712
C8orf30A	0.7945	1.0674	0.9839	0.8908	0.9341	0.0589
CAMK1G	0.9339	1.1100	0.8529	0.8398	0.9342	0.0622
LOC645166	0.8496	1.3702	0.8421	0.6749	0.9342	0.1508
LOC653496	0.8768	1.1092	0.9634	0.7873	0.9342	0.0685
LOC392221	0.8949	1.1066	0.8866	0.8486	0.9342	0.0584
FNTA	0.8297	1.2358	0.9827	0.6885	0.9342	0.1171
MIR942	0.9716	0.9620	0.9719	0.8312	0.9342	0.0344
MMP3	0.9447	0.9982	1.0061	0.7878	0.9342	0.0507
PF4	0.9405	1.0205	0.8754	0.9005	0.9342	0.0317
AGAP3	0.7842	1.1557	0.9583	0.8387	0.9342	0.0823
ANKRD5	0.8827	1.0749	0.9362	0.8431	0.9342	0.0506
TMEM189	0.8680	1.2913	0.8986	0.6791	0.9342	0.1285
LOC1001334	0.8784	1.0622	0.8921	0.9043	0.9342	0.0430
LOC1001338	0.7194	1.0586	1.0366	0.9224	0.9342	0.0776
NANOS3	0.8967	0.9953	1.0224	0.8227	0.9343	0.0460
LIPN	0.8665	0.9946	0.9218	0.9541	0.9343	0.0271
DCP1B	0.8485	1.0425	0.9521	0.8940	0.9343	0.0418
LOC653115	0.8849	0.9042	1.0360	0.9120	0.9343	0.0344
H2BFXP	0.9063	1.0316	0.9529	0.8464	0.9343	0.0391
DOCK11	0.8499	1.2235	0.9733	0.6904	0.9343	0.1125
HTR3A	0.8317	1.0864	0.9351	0.8841	0.9343	0.0549

LOC645450	0.9363	0.9946	0.9577	0.8487	0.9343	0.0310
LOC644836	0.7943	1.1486	0.9039	0.8906	0.9343	0.0755
LOC729225	0.9515	0.9874	0.9930	0.8055	0.9343	0.0439
GGA3	0.9015	1.1155	0.9186	0.8018	0.9343	0.0656
LOC729882	0.8071	1.0485	0.9551	0.9268	0.9344	0.0498
C10orf35	0.7471	1.2929	0.9856	0.7119	0.9344	0.1341
LOC646144	0.8215	1.0788	0.9971	0.8400	0.9344	0.0622
TAS2R43	0.8877	0.9898	0.9806	0.8794	0.9344	0.0295
OGDH	0.9352	1.1954	0.8759	0.7310	0.9344	0.0970
DLX2	0.9690	0.9908	0.9189	0.8588	0.9344	0.0293
C22orf30	0.7115	1.3256	0.9550	0.7455	0.9344	0.1411
LOC650412	0.7464	1.0373	1.0847	0.8691	0.9344	0.0779
THOC2	0.7841	1.2270	1.0449	0.6816	0.9344	0.1239
LOC645942	0.8663	1.2248	0.8315	0.8150	0.9344	0.0974
CXCL17	0.8596	0.9798	1.0132	0.8851	0.9344	0.0368
LOC647389	0.7946	1.0956	1.0304	0.8172	0.9344	0.0756
LOC389160	0.8343	0.9893	1.0524	0.8619	0.9344	0.0518
LOC391378	0.8542	1.0361	0.9763	0.8712	0.9344	0.0433
LOC1001298	1.2096	0.9842	1.0150	0.5290	0.9344	0.1441
LOC1001279	0.8851	1.0268	0.9781	0.8478	0.9345	0.0412
C6orf208	0.8261	1.0729	0.9655	0.8735	0.9345	0.0545
LOC648548	0.8869	0.9422	0.9161	0.9928	0.9345	0.0225
TECPR1	0.8531	1.0920	0.9617	0.8311	0.9345	0.0598
MDH1B	0.8394	1.0240	0.8948	0.9797	0.9345	0.0415
LOC391825	1.0210	1.0573	0.9145	0.7452	0.9345	0.0700
FAM120C	0.8343	0.9944	0.9372	0.9720	0.9345	0.0354
SETDB1	0.8939	1.3419	0.8081	0.6940	0.9345	0.1418
LOC650159	0.9175	1.1103	0.9258	0.7843	0.9345	0.0670
PEF1	0.7564	1.2381	0.9857	0.7581	0.9346	0.1146
PHOSPHO1	0.8565	1.1251	0.9001	0.8565	0.9346	0.0643
LOC643933	0.8818	1.1859	0.8689	0.8016	0.9346	0.0856
LOC401650	0.9589	1.0089	0.9671	0.8035	0.9346	0.0451
LOC646781	0.9672	0.9117	1.0002	0.8592	0.9346	0.0311
LOC1001311	0.9038	0.9671	0.9689	0.8986	0.9346	0.0193
LOC649848	0.9084	0.9636	1.0240	0.8424	0.9346	0.0388
LOC646135	0.9253	1.0485	1.0221	0.7425	0.9346	0.0693
SLC24A3	0.8589	1.0488	0.9514	0.8794	0.9346	0.0429
KRTAP8-1	0.8781	0.9782	0.9565	0.9257	0.9346	0.0217
LOC1001342	0.8658	1.0898	0.9112	0.8717	0.9346	0.0527
LOC344165	0.8474	0.9368	1.0408	0.9136	0.9346	0.0401
KCTD18	0.9757	1.1303	0.9472	0.6855	0.9347	0.0923
TCEAL2	0.8697	1.1457	0.8947	0.8287	0.9347	0.0716
LOC642412	0.7876	1.2430	0.9277	0.7805	0.9347	0.1082
C10orf131	0.8473	1.0165	1.0100	0.8650	0.9347	0.0455
ZNHIT3	0.8326	1.2285	0.9568	0.7209	0.9347	0.1091
LOC730101	0.9205	1.0518	0.9057	0.8608	0.9347	0.0410

C17orf72	0.8335	0.9978	0.9677	0.9399	0.9347	0.0357
CACNB4	0.8342	1.0328	0.9478	0.9242	0.9347	0.0408
LOC400298	0.9145	1.0766	0.9182	0.8297	0.9347	0.0515
ANK3	0.8102	1.2026	0.9821	0.7441	0.9347	0.1024
ZDHHC8	0.7763	1.4080	0.8264	0.7283	0.9347	0.1590
LOC1001327	0.9197	0.9873	0.9573	0.8749	0.9348	0.0243
VN1R5	0.8728	1.0348	0.9611	0.8705	0.9348	0.0395
LOC642521	0.8283	1.0520	0.9782	0.8807	0.9348	0.0499
MUC2	0.8718	1.0126	0.9157	0.9391	0.9348	0.0294
LOC646129	0.8686	1.0410	0.9282	0.9014	0.9348	0.0375
C15orf2	0.7638	1.0888	1.0096	0.8770	0.9348	0.0718
LOC729519	0.8114	1.0262	1.0238	0.8778	0.9348	0.0538
SHROOM3	1.0246	1.1688	0.8300	0.7160	0.9348	0.1007
ATAD1	0.7530	1.1561	1.0838	0.7464	0.9348	0.1079
TMEM72	0.9741	0.9476	0.9800	0.8376	0.9348	0.0332
SGIP1	0.8733	1.0017	0.9666	0.8978	0.9348	0.0298
FLJ32569	0.8569	1.0529	0.9757	0.8538	0.9349	0.0485
TTC12	0.8250	1.1281	1.0310	0.7554	0.9349	0.0870
ANKS6	0.8934	1.0474	0.9404	0.8581	0.9349	0.0411
FAM108B1	0.8217	1.1573	0.9867	0.7737	0.9349	0.0871
SNORD18C	0.8448	1.0104	0.9147	0.9696	0.9349	0.0359
KIF18A	0.8197	1.0010	1.1456	0.7732	0.9349	0.0857
SOCS2	0.9122	1.1840	0.9047	0.7386	0.9349	0.0922
CYP4X1	0.9465	1.0116	0.9181	0.8634	0.9349	0.0308
LOC729986	0.8670	0.9957	1.0156	0.8613	0.9349	0.0411
LOC644829	0.8555	1.0868	0.9486	0.8486	0.9349	0.0555
LOC641746	0.9209	1.0122	1.0112	0.7953	0.9349	0.0512
FLJ20397	0.9149	1.0290	0.9325	0.8632	0.9349	0.0347
LOC643131	0.9898	0.9418	0.8991	0.9089	0.9349	0.0204
SYS1	0.8369	1.3639	0.8448	0.6941	0.9349	0.1471
LOC650058	0.8957	0.9434	0.9789	0.9217	0.9350	0.0176
HHEX	0.9189	1.0540	0.9447	0.8223	0.9350	0.0476
LOC653186	0.8754	1.0208	0.9541	0.8896	0.9350	0.0334
OR13C5	0.9568	0.8714	1.0501	0.8616	0.9350	0.0439
LOC646513	0.8768	1.0315	0.9746	0.8569	0.9350	0.0412
PSMB1	0.7437	1.3383	1.0705	0.5874	0.9350	0.1679
PIK3CG	0.9021	1.0480	1.0278	0.7622	0.9350	0.0660
CD1E	0.8811	1.0003	1.0201	0.8386	0.9350	0.0445
DOK4	0.8591	1.0412	0.9553	0.8846	0.9350	0.0408
LOC1001297	0.9004	0.9452	0.9465	0.9481	0.9350	0.0116
IDO2	0.9309	0.9674	0.9243	0.9177	0.9350	0.0111
SRC	0.8934	1.1898	0.8136	0.8434	0.9351	0.0865
FER1L6	0.9896	0.9819	0.9229	0.8459	0.9351	0.0333
GALNT12	0.9463	1.0092	0.8990	0.8858	0.9351	0.0279
GAGE2C	0.9552	0.9001	1.0638	0.8211	0.9351	0.0510
LPCAT2	0.8953	1.0545	0.9613	0.8292	0.9351	0.0481

MIR509-2	0.9445	0.9764	0.9793	0.8403	0.9351	0.0326
LOC442448	0.9178	1.1252	0.9230	0.7745	0.9351	0.0721
RUFY3	0.8194	1.1394	0.9765	0.8051	0.9351	0.0784
C19orf18	0.9217	1.0544	0.9075	0.8569	0.9351	0.0421
ULK4	0.9070	1.0648	0.9761	0.7926	0.9351	0.0574
LOC652357	0.8319	1.0561	0.9997	0.8529	0.9351	0.0549
C20orf107	0.9169	0.9707	0.9866	0.8664	0.9351	0.0274
CORT	0.8680	1.0503	0.9681	0.8542	0.9351	0.0460
NSMCE2	0.7977	1.3305	0.8950	0.7174	0.9352	0.1367
GNG13	0.9313	0.9756	0.9592	0.8745	0.9352	0.0222
ATF6B	0.8812	0.9950	0.9230	0.9416	0.9352	0.0236
CA5B	0.8404	1.0737	0.9727	0.8539	0.9352	0.0549
LOC648834	0.8021	1.0689	1.0332	0.8366	0.9352	0.0677
HSD17B3	0.9665	1.0532	0.8598	0.8613	0.9352	0.0466
LOC641784	0.8658	1.0290	0.9753	0.8708	0.9352	0.0402
LINCR	0.7518	1.0282	1.0701	0.8908	0.9352	0.0721
C12orf67	0.9411	1.0416	0.8576	0.9006	0.9352	0.0393
LOC654080	0.8583	1.0634	0.9680	0.8513	0.9353	0.0504
HOMER1	0.7181	1.0517	1.1790	0.7922	0.9353	0.1082
TNKS2	0.9849	0.8035	1.0903	0.8624	0.9353	0.0640
ZNRF3	0.8572	1.1988	0.8771	0.8081	0.9353	0.0890
OR1E1	0.9315	0.9405	0.9811	0.8881	0.9353	0.0191
POLG	0.8718	1.2153	0.8781	0.7760	0.9353	0.0962
CPXM2	0.8621	0.9876	1.0039	0.8876	0.9353	0.0354
C20orf144	0.8559	0.9897	1.0093	0.8864	0.9353	0.0378
LOC651341	0.8721	1.0025	1.0277	0.8390	0.9353	0.0468
GYS1	0.8883	1.0668	0.9508	0.8354	0.9353	0.0498
LTB4R2	0.8838	0.9996	0.9727	0.8851	0.9353	0.0299
LOC728772	0.9002	1.0777	0.9724	0.7912	0.9354	0.0603
LOC730974	0.8845	1.0256	0.9801	0.8513	0.9354	0.0406
MYOHD1	0.8287	1.1013	0.8901	0.9215	0.9354	0.0586
LOC729335	0.9278	0.9438	0.9523	0.9177	0.9354	0.0078
MAGED4B	0.9407	1.1023	0.9134	0.7852	0.9354	0.0651
C2orf88	0.9004	1.0273	0.9112	0.9028	0.9354	0.0307
CCDC121	0.9459	1.0743	0.9401	0.7815	0.9354	0.0599
CGB8	0.9205	1.0605	0.9700	0.7908	0.9354	0.0563
PLAC1	0.8498	0.9759	1.0307	0.8853	0.9354	0.0414
LOC1001335	0.8518	1.0392	0.8726	0.9782	0.9354	0.0443
LOC650568	0.9261	0.9559	0.8499	1.0098	0.9354	0.0334
CELSR3	0.8904	1.0824	0.9213	0.8477	0.9354	0.0513
DVL3	0.9222	1.2274	0.7854	0.8068	0.9354	0.1018
LOC650841	0.9003	0.9738	1.0122	0.8554	0.9355	0.0354
LOC641705	0.8489	1.0605	0.9502	0.8822	0.9355	0.0467
LOC1001280	0.9305	1.0045	0.9020	0.9049	0.9355	0.0239
LRRTM2	0.8124	1.0375	0.9115	0.9804	0.9355	0.0484
C17orf51	0.9110	1.1121	0.9004	0.8184	0.9355	0.0624

OR52E6	0.8603	1.0445	0.8949	0.9422	0.9355	0.0400
LOC90499	0.9408	1.0824	0.8900	0.8287	0.9355	0.0541
BAG2	0.7743	1.1481	1.0120	0.8076	0.9355	0.0882
LOC645162	0.9679	1.0047	0.9711	0.7985	0.9355	0.0464
THRAP3	0.8370	1.3815	0.8532	0.6705	0.9355	0.1543
LOC642934	0.8419	1.1348	1.1457	0.6198	0.9356	0.1266
ZNF705A	0.8960	1.0212	0.9976	0.8275	0.9356	0.0451
AMN1	0.9086	1.0056	1.0042	0.8239	0.9356	0.0436
LOC1001300	0.9012	1.0467	0.8777	0.9167	0.9356	0.0379
BRI3	0.8238	1.4657	0.8005	0.6523	0.9356	0.1807
CPXM1	0.8968	1.0243	0.9839	0.8373	0.9356	0.0422
DKFZp434E1	1.0044	1.0437	0.9024	0.7918	0.9356	0.0564
GLI4	0.8298	1.1432	0.9662	0.8033	0.9356	0.0779
LOC1001316	0.8196	1.0113	1.1909	0.7206	0.9356	0.1043
LOC1001296	0.9022	0.9989	0.8815	0.9598	0.9356	0.0268
SAT2	0.9576	1.2350	0.8499	0.7000	0.9356	0.1129
KLK11	0.9609	0.9403	0.9419	0.8995	0.9356	0.0129
LOC647123	0.8332	0.9968	0.9458	0.9670	0.9357	0.0357
LOC652469	0.8990	0.9854	1.0370	0.8214	0.9357	0.0476
LOC645334	0.8730	1.0838	1.0124	0.7735	0.9357	0.0696
BTBD1	0.6933	1.3389	1.0460	0.6646	0.9357	0.1599
PRO0611	0.7661	1.0281	1.0040	0.9447	0.9357	0.0592
PIN1L	0.9344	0.9265	1.0133	0.8687	0.9357	0.0297
SYN2	0.8770	1.0080	0.9996	0.8583	0.9357	0.0395
LOC642883	0.9299	1.1659	0.8256	0.8216	0.9357	0.0807
LOC643624	0.9362	1.0758	0.9483	0.7827	0.9358	0.0600
MIR758	0.8915	1.0666	0.9234	0.8615	0.9358	0.0454
LOC652543	0.8083	1.1257	0.9385	0.8706	0.9358	0.0687
C20orf201	0.9358	0.9967	0.9622	0.8483	0.9358	0.0317
TESSP1	0.9822	0.9965	0.8973	0.8671	0.9358	0.0317
TEX15	0.7874	1.0326	0.9560	0.9671	0.9358	0.0523
BTN2A3	0.9010	1.0565	0.9466	0.8391	0.9358	0.0459
TUSC5	0.8358	1.0458	1.0586	0.8030	0.9358	0.0676
LOC727808	0.7279	1.4334	0.8745	0.7075	0.9358	0.1700
LOC1001332	0.9923	0.9908	0.9756	0.7846	0.9358	0.0505
LINGO1	0.9382	1.0621	0.9419	0.8011	0.9358	0.0533
LOC1001291	0.9261	0.9833	0.9773	0.8566	0.9358	0.0294
SDHAP2	0.8917	1.2670	0.8631	0.7216	0.9358	0.1165
FLJ34306	0.9069	1.0675	0.9680	0.8010	0.9359	0.0558
NKAP	0.9241	1.0986	0.9247	0.7962	0.9359	0.0621
LOC729204	0.8802	0.9806	0.8863	0.9965	0.9359	0.0306
LOC1001303	0.7944	1.0376	1.0435	0.8680	0.9359	0.0623
FOXR2	0.9288	0.9534	1.0290	0.8324	0.9359	0.0406
TGM4	0.8377	1.0482	1.0007	0.8570	0.9359	0.0522
BPIL2	0.9249	1.0329	0.9178	0.8680	0.9359	0.0347
NUDT21	0.7541	1.1939	1.0433	0.7524	0.9359	0.1099

RFK	0.8836	1.0669	1.0197	0.7735	0.9359	0.0666
PITPNM2	0.8733	1.2087	0.8585	0.8032	0.9359	0.0922
LOC344967	0.8875	0.9617	1.0502	0.8444	0.9359	0.0451
LOC220077	0.9317	0.9887	0.8960	0.9275	0.9359	0.0193
FLJ40113	0.8537	1.0772	0.9104	0.9025	0.9360	0.0487
FLJ35934	0.8880	1.0167	0.9476	0.8916	0.9360	0.0302
COL11A2	0.8863	1.0164	0.9882	0.8530	0.9360	0.0393
LOC728094	0.8826	0.9648	1.0214	0.8752	0.9360	0.0350
MPHOSPH10	0.7678	1.2751	0.9791	0.7219	0.9360	0.1261
LOC644689	0.8614	0.9936	0.9427	0.9462	0.9360	0.0274
STK16	0.8490	1.1845	0.8665	0.8441	0.9360	0.0830
ANKRD41	0.8652	1.0704	0.9639	0.8445	0.9360	0.0518
SNCAIP	0.8803	1.0053	0.9913	0.8671	0.9360	0.0362
LSM1	0.7061	1.3348	0.9706	0.7326	0.9360	0.1456
LOC729275	0.8267	1.0838	0.9443	0.8892	0.9360	0.0548
RAET1E	0.8394	1.0047	0.9979	0.9021	0.9360	0.0398
MIR1302-6	0.8723	1.0638	0.8682	0.9400	0.9361	0.0457
RABAC1	0.9403	1.1723	0.9904	0.6413	0.9361	0.1102
ETF1	0.8134	1.3297	0.9436	0.6577	0.9361	0.1436
LOC649580	0.7715	1.2152	0.8877	0.8700	0.9361	0.0965
CCL26	0.9145	0.9902	1.0197	0.8199	0.9361	0.0446
CDC2L1	0.9228	1.2443	0.8341	0.7433	0.9361	0.1091
EML5	0.9202	1.0184	0.9292	0.8766	0.9361	0.0298
GPATCH8	0.8593	1.1354	1.0045	0.7452	0.9361	0.0850
LOC1001342	0.9119	0.9898	0.9583	0.8846	0.9361	0.0235
IFRD2	0.8149	1.3417	0.9365	0.6515	0.9362	0.1473
CCDC132	0.8926	1.0233	0.9956	0.8332	0.9362	0.0444
DHX8	1.0008	1.0013	0.9168	0.8258	0.9362	0.0418
SCYL1BP1	0.8740	1.0807	0.9808	0.8091	0.9362	0.0598
GPX3	0.8542	1.0573	0.9518	0.8814	0.9362	0.0453
FAM82B	0.9643	0.9624	0.9173	0.9009	0.9362	0.0160
C7orf33	0.9204	0.9635	0.9416	0.9194	0.9362	0.0104
LOC1001331	0.8950	0.9893	0.9700	0.8906	0.9362	0.0254
LOC649227	0.8976	1.0049	1.0249	0.8175	0.9362	0.0485
LOC644251	0.8597	0.9938	1.0214	0.8701	0.9362	0.0416
NBPF8	0.8572	1.1025	0.9433	0.8421	0.9363	0.0597
SPTY2D1	0.6957	1.1437	1.1116	0.7942	0.9363	0.1125
LOC651794	0.9871	1.0424	0.8770	0.8387	0.9363	0.0473
PARN	0.8599	1.1958	0.8806	0.8088	0.9363	0.0878
MIR548H4	0.9324	0.9253	0.9653	0.9221	0.9363	0.0099
ISL1	0.9147	0.9696	1.0198	0.8411	0.9363	0.0383
C20orf26	0.7927	1.0177	1.0120	0.9228	0.9363	0.0526
LOC1001321	0.8139	1.0499	0.9183	0.9631	0.9363	0.0491
FAM133B	0.7607	1.2126	1.0523	0.7197	0.9363	0.1182
LOC649886	0.8823	1.0604	0.9552	0.8473	0.9363	0.0471
FSD1	0.8944	1.0549	0.9224	0.8735	0.9363	0.0408

AQP1	0.8207	1.1244	0.9389	0.8612	0.9363	0.0673
LOC644899	0.9449	0.8976	1.0065	0.8963	0.9363	0.0260
STRN	0.9841	1.1316	0.8360	0.7937	0.9363	0.0768
LOC645001	0.8667	1.1682	0.8699	0.8406	0.9364	0.0775
FLJ38717	0.8503	1.0602	0.9301	0.9049	0.9364	0.0445
C1orf95	0.9105	1.0671	0.9161	0.8519	0.9364	0.0459
LOC652274	0.8841	0.9847	0.9762	0.9007	0.9364	0.0257
BMP5	0.9843	1.1296	0.8533	0.7784	0.9364	0.0772
RAPGEF3	0.8616	1.0298	0.9572	0.8969	0.9364	0.0369
LOC1001279	0.8243	0.9649	1.0179	0.9386	0.9364	0.0409
KIAA1407	0.9008	1.0580	0.9196	0.8672	0.9364	0.0419
LOC644641	0.8200	1.0668	1.0358	0.8230	0.9364	0.0666
LOC642003	0.9177	1.0338	0.9237	0.8704	0.9364	0.0346
LOC643253	0.9144	1.1317	0.9326	0.7669	0.9364	0.0749
GPX4	0.7726	1.2865	1.0357	0.6509	0.9364	0.1417
PROKR2	0.9647	0.9976	0.9729	0.8105	0.9364	0.0425
LOC730427	0.8425	1.0557	0.9722	0.8754	0.9364	0.0484
SNORD91A	0.8563	1.0018	1.0479	0.8398	0.9364	0.0520
LOC1001330	0.8932	1.0420	0.9592	0.8514	0.9364	0.0416
LOC651694	0.8343	1.0078	0.9970	0.9067	0.9364	0.0409
IFLTD1	0.8831	1.0694	0.9603	0.8330	0.9364	0.0515
LOC651886	0.8966	1.0633	0.9155	0.8704	0.9364	0.0433
COMMD6	0.8018	1.2240	0.9788	0.7412	0.9365	0.1083
PLCH1	0.8576	1.0914	0.9670	0.8299	0.9365	0.0595
KCNQ3	0.8874	1.0049	0.9700	0.8836	0.9365	0.0303
LOC1001343	0.9506	0.9684	0.9193	0.9076	0.9365	0.0140
NIPAL1	0.8878	1.1137	0.9424	0.8020	0.9365	0.0658
MYBL1	0.7767	1.1222	0.8998	0.9471	0.9365	0.0716
LOC729209	0.8503	1.0666	1.0130	0.8160	0.9365	0.0611
NPLOC4	0.9203	1.2313	0.8519	0.7425	0.9365	0.1049
BBC3	0.8550	1.1879	0.9259	0.7772	0.9365	0.0891
MIR1266	0.9710	1.0468	0.8864	0.8418	0.9365	0.0455
HRH1	0.9302	1.1361	0.8790	0.8007	0.9365	0.0717
LOC647244	0.8864	0.9689	0.9243	0.9664	0.9365	0.0196
MIR30E	0.8999	1.0796	0.9624	0.8042	0.9365	0.0577
FAM126A	0.8764	1.0824	0.8665	0.9208	0.9365	0.0500
POLM	0.8628	1.0237	0.9972	0.8624	0.9365	0.0430
LOC730004	0.7430	1.0968	1.1693	0.7371	0.9365	0.1144
LOC653733	0.9307	1.0357	0.8464	0.9334	0.9365	0.0387
LOC644297	0.9693	0.9692	0.9961	0.8115	0.9366	0.0421
LOC1001296	0.8753	1.0022	0.9779	0.8909	0.9366	0.0314
LOC1001299	0.8885	1.1265	0.8176	0.9136	0.9366	0.0665
ZNF582	0.9189	1.0297	0.9197	0.8779	0.9366	0.0325
LOC649652	0.9680	0.9970	0.9352	0.8461	0.9366	0.0327
LOC648979	0.8972	0.9864	0.9407	0.9221	0.9366	0.0188
DOK3	1.0180	0.9039	0.8827	0.9418	0.9366	0.0298

LOC1001342	0.8388	1.1082	0.9448	0.8546	0.9366	0.0618
NUP43	0.8146	1.0198	1.1302	0.7819	0.9366	0.0833
LOC651974	0.8465	0.9176	1.0608	0.9217	0.9366	0.0449
LOC654780	0.9158	0.9757	0.9959	0.8592	0.9367	0.0309
KCNA2	0.9213	1.0141	0.9115	0.8996	0.9367	0.0262
MIR217	0.8943	0.9583	1.0012	0.8929	0.9367	0.0264
DMC1	0.7965	1.0860	1.0221	0.8422	0.9367	0.0696
PYROXD1	0.8493	0.9039	1.0877	0.9058	0.9367	0.0520
WHSC1L1	0.8740	1.0871	0.9437	0.8419	0.9367	0.0544
LOC442609	0.8361	0.9900	1.0639	0.8567	0.9367	0.0544
ALDH18A1	0.6583	1.4610	0.9356	0.6920	0.9367	0.1854
LOC649279	0.8104	1.1088	0.9515	0.8762	0.9367	0.0642
SNORA48	0.7358	1.1669	1.0086	0.8356	0.9367	0.0952
LOC1001313	0.9349	0.9550	0.9416	0.9154	0.9367	0.0082
IGSF10	0.9731	1.0490	0.8749	0.8499	0.9367	0.0459
DMRTC1	0.8892	0.9695	0.9843	0.9039	0.9367	0.0236
ZC3HAV1L	0.8461	1.0637	0.9376	0.8996	0.9367	0.0463
LOC653419	0.8912	1.0136	0.9732	0.8690	0.9367	0.0340
NLRP11	0.8073	0.9898	1.0705	0.8794	0.9367	0.0583
VPS25	0.8883	1.3177	0.9079	0.6332	0.9368	0.1416
SNORA71A	0.8341	1.0935	0.9784	0.8409	0.9368	0.0619
LOC652300	0.9166	0.9262	1.0102	0.8941	0.9368	0.0254
LOC1001334	0.9025	0.9480	1.0086	0.8880	0.9368	0.0271
LOC283143	0.9495	1.0068	0.9795	0.8114	0.9368	0.0434
FAM92B	1.0144	0.9848	0.8462	0.9017	0.9368	0.0385
GPR162	0.9317	1.0194	0.9335	0.8626	0.9368	0.0321
C1orf108	1.0041	0.9724	0.9311	0.8395	0.9368	0.0357
LOC641750	0.8684	0.9664	1.0006	0.9119	0.9368	0.0292
LOC644548	0.9086	0.9917	0.9613	0.8857	0.9368	0.0242
ARNTL	0.8264	1.1394	0.9897	0.7919	0.9368	0.0801
HISPPD2A	0.9885	1.0998	0.8740	0.7850	0.9368	0.0684
MIR554	0.8329	1.0059	1.0064	0.9022	0.9368	0.0424
INTS10	0.8739	1.3246	0.8967	0.6521	0.9368	0.1405
LOC648271	0.8610	0.9238	1.0098	0.9528	0.9368	0.0310
LOC1001334	0.9206	1.0342	0.9901	0.8024	0.9368	0.0506
ABCD3	0.8520	1.2311	0.8711	0.7933	0.9369	0.0995
LOC646456	0.8808	1.0958	0.9003	0.8706	0.9369	0.0533
SNORD3A	0.8730	1.0219	1.0705	0.7820	0.9369	0.0666
SCGBL	0.9760	0.9548	0.9400	0.8766	0.9369	0.0214
KBTBD7	0.8318	1.0142	1.1289	0.7725	0.9369	0.0821
RGS9BP	0.9019	1.1249	0.9506	0.7701	0.9369	0.0734
LOC1001280	0.9142	0.9079	0.9320	0.9935	0.9369	0.0195
LOC732275	0.8240	1.0109	1.0242	0.8885	0.9369	0.0485
CLECL1	0.8756	0.9824	0.9638	0.9257	0.9369	0.0236
CFHR2	0.9722	1.0325	0.9201	0.8228	0.9369	0.0444
MAF1	0.8892	1.2463	0.9154	0.6967	0.9369	0.1141

CNOT4	0.7941	1.1773	0.9984	0.7778	0.9369	0.0946
STK38L	0.8411	0.9849	1.0267	0.8950	0.9369	0.0421
LOC645322	0.9156	0.9427	1.0156	0.8738	0.9369	0.0298
ASB16	0.9598	0.9173	0.9428	0.9278	0.9369	0.0093
LOC642832	0.9929	0.9854	0.9396	0.8299	0.9369	0.0376
ANKRD20A1	0.9115	1.0895	0.9559	0.7910	0.9370	0.0616
TRIM16	0.9440	1.0280	0.9202	0.8558	0.9370	0.0356
LOC392781	0.8565	0.9700	1.0564	0.8651	0.9370	0.0474
LOC647710	0.7323	1.0999	0.9883	0.9275	0.9370	0.0770
LOC645405	0.9193	1.0109	0.9670	0.8509	0.9370	0.0343
POLH	0.9321	0.9723	1.0094	0.8344	0.9370	0.0377
RABIF	0.9227	1.0661	0.9896	0.7697	0.9370	0.0630
SPARC	0.9365	1.0444	0.9200	0.8473	0.9371	0.0407
HRB	0.7023	1.1535	1.0933	0.7991	0.9371	0.1101
ATP2C2	0.8623	1.2691	0.8404	0.7764	0.9371	0.1122
LOC729266	0.9159	0.9280	1.0518	0.8526	0.9371	0.0417
PRSS36	0.9317	1.0539	0.9562	0.8065	0.9371	0.0509
FLJ30428	0.8873	1.0682	0.9522	0.8407	0.9371	0.0493
ZNF267	0.8617	1.1838	0.9263	0.7765	0.9371	0.0878
LOC1001335	0.9257	0.9782	0.9554	0.8890	0.9371	0.0193
ZNF497	0.9117	1.0025	0.9763	0.8578	0.9371	0.0326
C19orf57	0.9280	1.0821	0.8951	0.8431	0.9371	0.0514
SLC16A11	0.8786	0.9070	0.9835	0.9793	0.9371	0.0262
TBC1D16	0.9331	1.0879	0.9461	0.7813	0.9371	0.0626
MAGIX	0.8443	0.9616	1.0101	0.9324	0.9371	0.0348
LOC729760	0.8612	1.1023	0.8929	0.8922	0.9371	0.0555
PCYT1A	0.9393	1.0485	0.9118	0.8489	0.9371	0.0417
KDR	0.8991	0.9989	0.9391	0.9114	0.9371	0.0222
MIR490	0.9606	0.9760	0.8572	0.9547	0.9371	0.0270
LOC644921	0.8140	1.0893	0.8788	0.9664	0.9371	0.0596
LOC646312	0.9253	1.0070	0.9515	0.8647	0.9371	0.0295
LOC645453	0.8626	1.0082	0.9793	0.8985	0.9371	0.0340
BP75	0.7687	1.2253	0.9452	0.8095	0.9372	0.1032
PCDHGB4	0.9723	1.0438	0.8477	0.8850	0.9372	0.0441
LOC730027	0.8541	1.1024	0.9040	0.8883	0.9372	0.0560
LOC347364	1.0386	1.0100	0.9062	0.7941	0.9372	0.0556
LOC652368	0.8762	0.9428	0.9816	0.9483	0.9372	0.0221
MIIP	0.8474	1.0939	0.9164	0.8912	0.9372	0.0541
LOC653580	0.9595	0.9960	0.8885	0.9048	0.9372	0.0248
C16orf65	0.7943	1.0557	1.0165	0.8825	0.9372	0.0604
NOS2	0.9666	1.0073	0.9312	0.8439	0.9372	0.0348
PGM2L1	0.8924	1.0840	1.0154	0.7572	0.9373	0.0719
LOC1001288	0.8955	0.9217	1.0281	0.9037	0.9373	0.0308
RAMP3	0.8635	0.9684	0.9637	0.9535	0.9373	0.0248
LOC338598	0.9323	0.9208	0.9628	0.9333	0.9373	0.0090
LOC728774	0.9070	0.9420	1.0818	0.8183	0.9373	0.0548

ZNF575	0.8488	0.9817	0.9860	0.9328	0.9373	0.0319
LOC645165	0.8261	1.1357	1.0092	0.7782	0.9373	0.0828
MKX	0.8549	1.0329	0.8982	0.9634	0.9373	0.0389
TMEM100	0.9735	1.1580	0.8380	0.7798	0.9373	0.0840
FLJ16331	0.9511	0.9020	1.0081	0.8881	0.9373	0.0272
C6orf153	0.7434	1.4097	0.9335	0.6629	0.9373	0.1674
LOC441251	0.8869	0.9620	1.1367	0.7639	0.9374	0.0780
ACP6	0.8348	1.3334	0.8194	0.7619	0.9374	0.1329
OR1C1	0.8045	1.1236	0.9735	0.8479	0.9374	0.0717
LOC729569	0.8710	1.1605	0.8636	0.8545	0.9374	0.0744
LOC1001290	0.9532	1.0389	0.9252	0.8323	0.9374	0.0426
LOC646223	0.7604	1.1963	1.0423	0.7508	0.9374	0.1096
LOC653206	0.8221	1.0628	0.9681	0.8969	0.9375	0.0513
SERPINB11	0.9403	0.9973	0.9310	0.8812	0.9375	0.0238
OR56A3	0.8902	0.9871	0.9462	0.9264	0.9375	0.0202
SIGLEC11	0.8221	1.0567	0.9483	0.9229	0.9375	0.0482
LOC653225	0.9276	1.0122	0.9172	0.8930	0.9375	0.0259
ALX1	0.6998	1.0952	1.1085	0.8464	0.9375	0.0995
DEPDC5	0.8500	1.0834	0.8951	0.9215	0.9375	0.0508
LOC644423	0.8578	1.1852	0.8739	0.8331	0.9375	0.0830
CLC	0.8285	1.0763	1.0245	0.8207	0.9375	0.0661
IL8RBP	0.9013	0.9777	0.9139	0.9571	0.9375	0.0180
ANP32C	0.8587	1.0307	1.0328	0.8279	0.9375	0.0548
KIF16B	0.9040	1.0832	0.9326	0.8302	0.9375	0.0531
HIF1A	0.7933	1.2411	1.0376	0.6781	0.9375	0.1259
LOC389203	0.7853	1.3090	0.9095	0.7463	0.9375	0.1286
LINGO3	0.8583	1.0655	1.0547	0.7717	0.9375	0.0730
LOC1001299	1.0275	1.0416	0.8911	0.7900	0.9375	0.0598
LOC1001328	0.8248	1.0996	0.9067	0.9191	0.9376	0.0579
C7orf42	0.8620	1.3123	0.9376	0.6385	0.9376	0.1401
HNRPC	0.8141	1.1012	1.1566	0.6785	0.9376	0.1144
AQP7P1	0.8462	1.0177	0.8800	1.0065	0.9376	0.0436
USP9Y	1.0354	0.9438	0.9798	0.7914	0.9376	0.0523
LOC344741	0.8556	1.0591	0.9812	0.8546	0.9376	0.0502
ATP11C	0.7695	1.1057	1.1402	0.7351	0.9376	0.1074
LOC729242	0.8755	0.9308	1.0198	0.9243	0.9376	0.0301
LOC643395	0.7831	1.0094	1.0270	0.9311	0.9376	0.0556
MFN2	0.7848	1.4477	0.8925	0.6254	0.9376	0.1787
LOC728408	0.8074	1.0790	1.0540	0.8103	0.9376	0.0746
LRRC46	0.9573	1.1088	0.8106	0.8739	0.9376	0.0645
THEM5	0.8733	1.0172	1.0244	0.8357	0.9376	0.0486
GNAL	0.8571	1.0615	0.9342	0.8978	0.9376	0.0442
LOC1001339	0.9089	0.9848	0.9714	0.8855	0.9376	0.0240
TMEM89	0.8571	1.1724	0.7999	0.9212	0.9376	0.0821
C6orf140	0.9152	1.0364	0.8980	0.9010	0.9376	0.0331
LOC389024	0.8320	1.1466	0.8683	0.9038	0.9377	0.0712

OPN4	0.8879	1.0547	0.9724	0.8357	0.9377	0.0481
LOC286135	0.9541	0.9890	1.0083	0.7993	0.9377	0.0475
LOC1001346	1.0553	0.9001	0.8435	0.9518	0.9377	0.0450
LOC1001290	0.9356	1.0059	0.9283	0.8810	0.9377	0.0257
C14orf72	0.9567	0.9221	1.0483	0.8238	0.9377	0.0464
LOC1001301	0.7945	1.0363	1.0633	0.8567	0.9377	0.0662
OR5K3	0.8914	1.0023	1.0006	0.8566	0.9377	0.0375
LOC643719	0.8637	1.0562	0.8908	0.9401	0.9377	0.0425
C11orf64	0.8882	1.0303	0.9730	0.8594	0.9377	0.0392
SAC	0.8064	1.1262	1.0154	0.8029	0.9377	0.0801
C10orf108	0.8828	1.0495	0.9970	0.8217	0.9377	0.0520
IL17C	0.9434	1.0976	0.8235	0.8866	0.9377	0.0586
C10orf73	0.9220	0.9519	1.0723	0.8048	0.9377	0.0550
LOC727879	0.8758	1.0220	1.0388	0.8143	0.9377	0.0551
C21orf128	0.8920	0.9543	1.0168	0.8879	0.9378	0.0304
OR8J3	0.9019	1.0665	0.9315	0.8512	0.9378	0.0460
SNORA19	0.9393	1.0741	0.9088	0.8289	0.9378	0.0511
CYB5R2	0.9453	0.9900	0.9626	0.8532	0.9378	0.0296
LOC646316	0.8018	1.0900	0.9991	0.8602	0.9378	0.0655
OR10A5	0.8992	1.0452	0.8589	0.9479	0.9378	0.0402
LOC441550	0.9360	1.0101	1.0920	0.7131	0.9378	0.0814
ALOX12B	0.8421	1.0123	1.0549	0.8420	0.9378	0.0560
C2orf52	0.8971	1.0738	0.9627	0.8176	0.9378	0.0542
YWHAB	0.7883	1.1737	1.0725	0.7168	0.9378	0.1099
ADAM12	0.9172	0.9973	0.9548	0.8820	0.9378	0.0248
UTP20	0.9268	1.0793	0.9082	0.8371	0.9378	0.0510
LOC727913	0.8098	1.0896	0.9658	0.8861	0.9378	0.0598
GOLPH3L	0.9737	1.0813	0.9887	0.7078	0.9378	0.0803
WNK3	0.8893	1.0200	0.9572	0.8849	0.9378	0.0320
WIPI1	0.8657	1.1005	0.9873	0.7978	0.9378	0.0669
LIX1L	0.8247	1.1620	0.9791	0.7855	0.9378	0.0856
LOC1001297	0.8654	0.9292	0.9961	0.9607	0.9378	0.0277
RAB11FIP4	0.8738	1.1195	0.9283	0.8298	0.9378	0.0638
RPL10A	0.7881	1.2176	0.9813	0.7644	0.9379	0.1051
OR11H1	0.9199	1.0618	0.9990	0.7709	0.9379	0.0628
LOC389740	0.8954	0.9057	1.0748	0.8756	0.9379	0.0461
LOC392501	0.8644	1.0256	1.0058	0.8557	0.9379	0.0451
LOC651319	0.9680	0.9516	0.9884	0.8435	0.9379	0.0324
NINL	0.8515	1.0528	0.9212	0.9261	0.9379	0.0419
ZBTB7B	0.9169	1.0122	1.0252	0.7973	0.9379	0.0527
LOC650295	0.9651	1.0859	0.8245	0.8762	0.9379	0.0572
LOC729766	0.8694	0.9890	0.9783	0.9150	0.9379	0.0281
POU4F1	0.9041	0.9940	0.9400	0.9137	0.9379	0.0202
RLN3	0.8445	0.9493	1.0487	0.9092	0.9379	0.0428
LOC650901	0.9369	1.0410	0.9047	0.8692	0.9379	0.0370
CAPN7	0.8544	1.1016	0.9355	0.8602	0.9379	0.0576

TDRD10	0.7733	1.0353	1.0116	0.9316	0.9379	0.0592
LOC644988	1.0225	1.1483	0.9375	0.6437	0.9380	0.1072
CYP4F8	0.9798	1.0161	0.8659	0.8901	0.9380	0.0358
LOC1001330	0.8429	1.0406	1.0169	0.8515	0.9380	0.0527
GCLC	1.0000	1.0166	0.9598	0.7757	0.9380	0.0554
LOC647854	0.9178	0.9282	1.0244	0.8816	0.9380	0.0305
ECM1	0.8805	1.0926	0.8901	0.8888	0.9380	0.0516
GALNS	0.7805	1.0464	1.1388	0.7863	0.9380	0.0912
GDEP	0.8512	0.9882	1.0085	0.9040	0.9380	0.0367
FCER2	0.8723	1.0189	0.9309	0.9300	0.9380	0.0302
LOC731109	0.8575	1.1571	1.0584	0.6791	0.9380	0.1064
NLRP4	0.8452	1.0043	1.0031	0.8996	0.9380	0.0395
TRIP12	0.8791	1.0209	1.1259	0.7261	0.9380	0.0869
CFP	0.8162	1.0545	1.0180	0.8634	0.9380	0.0580
SGK2	0.8721	1.0257	0.9846	0.8699	0.9381	0.0396
C1QL2	0.8183	1.1683	0.9026	0.8631	0.9381	0.0786
OSBP2	0.8817	1.0917	1.0093	0.7696	0.9381	0.0708
LOC127099	0.8655	0.9898	1.0256	0.8713	0.9381	0.0409
IRAK1BP1	0.9270	1.0352	0.9424	0.8477	0.9381	0.0385
LOC441495	0.8871	1.0488	0.9189	0.8976	0.9381	0.0375
GAS2L2	0.9437	1.0646	0.9764	0.7676	0.9381	0.0623
LOC650321	0.8479	1.0977	0.9200	0.8868	0.9381	0.0552
ZNF548	0.8725	1.1230	1.0044	0.7525	0.9381	0.0803
FAM25C	0.8683	1.0463	1.1044	0.7335	0.9381	0.0847
LOC642866	0.8855	1.0775	0.9438	0.8458	0.9381	0.0506
LOC388946	0.8504	0.9763	0.9972	0.9286	0.9381	0.0326
LOC646381	0.8174	1.0309	1.0718	0.8324	0.9381	0.0660
SERPINF2	0.9566	1.0296	0.9712	0.7952	0.9381	0.0502
LOC645410	0.9562	1.1303	0.8790	0.7870	0.9381	0.0728
LOC648377	0.8847	0.9956	1.0136	0.8587	0.9381	0.0389
LOC391013	0.8788	0.9978	1.0299	0.8460	0.9382	0.0447
KCNH3	0.9937	0.8975	0.9972	0.8643	0.9382	0.0338
PWRN1	0.9204	1.0092	0.9403	0.8828	0.9382	0.0265
C20orf4	0.8503	1.2549	0.9048	0.7426	0.9382	0.1108
LOC652889	0.8038	0.9766	1.0058	0.9666	0.9382	0.0456
CRHR1	0.9369	1.0147	0.8818	0.9194	0.9382	0.0280
LOC646011	0.8931	0.9162	1.1042	0.8393	0.9382	0.0576
LOC442329	0.9114	0.9511	0.9638	0.9265	0.9382	0.0118
C3orf24	0.9483	1.0215	0.8850	0.8980	0.9382	0.0310
LOC642000	0.8863	1.0428	1.0027	0.8210	0.9382	0.0513
LOC650538	0.9154	0.9531	1.1072	0.7771	0.9382	0.0679
C6orf186	0.9072	0.9754	1.0139	0.8564	0.9382	0.0351
MGC72104	0.8448	1.2865	0.9025	0.7192	0.9382	0.1222
LOC154449	0.9023	0.9986	0.9293	0.9228	0.9382	0.0209
C19orf75	0.7971	1.0302	1.0287	0.8970	0.9383	0.0565
SGCD	0.9368	1.0059	0.9620	0.8483	0.9383	0.0332

NKAIN4	0.9838	0.9148	0.9635	0.8909	0.9383	0.0214
SPG20	0.8342	1.1081	0.9007	0.9101	0.9383	0.0591
LOC1001348	0.7991	1.1382	1.0223	0.7935	0.9383	0.0853
LOC650562	0.8184	1.0439	0.9704	0.9204	0.9383	0.0473
LOC441120	0.8829	0.9608	0.9806	0.9289	0.9383	0.0213
LOC641761	1.0299	0.9848	0.9064	0.8320	0.9383	0.0437
LOC645037	0.8293	1.1763	0.9570	0.7906	0.9383	0.0869
LOC389786	0.8820	1.0215	0.9229	0.9268	0.9383	0.0295
PTP4A2	0.7921	1.2038	0.9621	0.7952	0.9383	0.0970
LOC642771	0.7311	1.2181	1.0017	0.8024	0.9383	0.1094
ELOVL3	0.9224	0.9503	0.9608	0.9199	0.9383	0.0102
LOC642738	0.8594	0.9772	1.1092	0.8075	0.9384	0.0671
LOC642181	0.8595	1.0221	1.0246	0.8472	0.9384	0.0491
MAPK14	0.8325	1.1246	0.9797	0.8166	0.9384	0.0721
LOC340096	0.8401	0.9812	1.0123	0.9199	0.9384	0.0380
LOC441811	0.8814	0.9747	1.0614	0.8360	0.9384	0.0502
LOC1001321	0.8631	1.0374	0.9734	0.8797	0.9384	0.0410
LOC401629	0.8527	1.0239	1.0062	0.8708	0.9384	0.0445
HACE1	0.7973	1.1609	0.9661	0.8293	0.9384	0.0827
LOC96597	0.9522	0.9265	1.0131	0.8619	0.9384	0.0313
LOC388743	0.9218	1.0274	0.9403	0.8642	0.9384	0.0338
LOC642749	0.8704	1.0954	0.9200	0.8679	0.9384	0.0537
SLC12A1	0.9758	0.9415	0.9395	0.8969	0.9384	0.0162
RNF6	0.8545	1.0523	1.0145	0.8325	0.9385	0.0556
NEURL4	0.8842	1.0896	0.8558	0.9243	0.9385	0.0523
ZNF676	0.9189	1.0378	0.9849	0.8123	0.9385	0.0486
NOD2	0.9127	0.9510	0.9724	0.9177	0.9385	0.0141
LOC643225	0.9759	1.0717	0.8420	0.8644	0.9385	0.0532
S100A1	0.7538	1.0348	1.0523	0.9132	0.9385	0.0689
LOC650241	0.7398	1.0890	0.9272	0.9980	0.9385	0.0740
LOC641798	0.7780	1.1741	1.0506	0.7513	0.9385	0.1036
FAM123B	0.8571	1.0691	0.9921	0.8357	0.9385	0.0556
LOC440926	0.8474	1.1632	1.0081	0.7355	0.9385	0.0935
LOC442208	0.9205	0.9653	1.0885	0.7798	0.9385	0.0637
ERCC-00104	0.9882	0.9707	0.9746	0.8207	0.9385	0.0395
LOC441632	0.8245	1.1284	0.9781	0.8231	0.9385	0.0730
MTHFSD	0.9644	1.0600	0.8959	0.8339	0.9386	0.0485
DNM2	0.9031	1.1563	0.9477	0.7472	0.9386	0.0843
FAM155A	0.8735	1.0932	0.9391	0.8485	0.9386	0.0550
PWWP2	0.8728	1.2200	0.8486	0.8130	0.9386	0.0946
LOC645411	0.8754	1.0169	1.0659	0.7962	0.9386	0.0623
CLTCL1	0.9033	1.0876	0.9137	0.8497	0.9386	0.0516
LOC1001347	0.8899	1.0397	1.0120	0.8129	0.9386	0.0531
SMCR7	0.9118	1.0853	0.9430	0.8144	0.9386	0.0560
LOC1001341	0.9632	1.0011	0.9586	0.8315	0.9386	0.0369
LOC1001333	0.9014	1.0914	0.9551	0.8065	0.9386	0.0595

CPNE6	0.8723	1.0132	0.9845	0.8846	0.9386	0.0353
LOC647908	0.9643	1.0015	0.9304	0.8584	0.9387	0.0304
LOC651745	0.7934	0.9976	1.0034	0.9603	0.9387	0.0494
TMEM104	0.9244	1.0713	0.9247	0.8344	0.9387	0.0490
TBC1D2	0.9716	1.0603	0.8612	0.8618	0.9387	0.0481
TAS2R39	0.8126	1.0748	1.0253	0.8421	0.9387	0.0653
TEAD3	0.8736	1.0496	0.9201	0.9116	0.9387	0.0383
TOMM20L	0.8708	1.1015	0.9628	0.8198	0.9387	0.0618
CCDC141	0.9007	1.0132	0.9781	0.8629	0.9387	0.0345
CD24	1.0322	1.0417	0.9364	0.7447	0.9387	0.0689
ZNF595	0.9255	0.9470	1.0557	0.8267	0.9387	0.0470
FAM7A1	0.7801	1.1028	1.0431	0.8289	0.9387	0.0791
LOC643982	0.9042	0.9717	0.9941	0.8851	0.9388	0.0262
C1QTNF6	0.8953	1.0040	0.9785	0.8773	0.9388	0.0309
TACR3	0.8701	1.1630	0.8167	0.9052	0.9388	0.0769
CCL3	0.8156	0.9583	1.0685	0.9126	0.9388	0.0525
FIGN	0.7699	1.0844	1.0426	0.8581	0.9388	0.0747
FLJ25169	0.9603	1.0427	0.9838	0.7683	0.9388	0.0594
LOC649192	0.8325	1.1287	0.9124	0.8815	0.9388	0.0654
LOC729854	0.9585	1.0273	0.8872	0.8822	0.9388	0.0343
MIRLET7F1	0.8478	1.0854	0.9753	0.8466	0.9388	0.0574
POM121L10I	0.8898	0.9523	1.0256	0.8874	0.9388	0.0326
IHPK3	0.8693	0.8763	1.2132	0.7963	0.9388	0.0933
ZMYND17	0.8236	0.9330	1.0184	0.9802	0.9388	0.0422
SEMA3D	0.8951	1.0409	0.9427	0.8766	0.9388	0.0368
LOC729663	0.9069	1.0276	0.9620	0.8588	0.9388	0.0363
RUNX3	0.8561	1.0550	0.9930	0.8511	0.9388	0.0508
NFKBIZ	0.9735	1.0665	0.8456	0.8697	0.9388	0.0508
FLJ21865	0.7469	1.1610	0.9287	0.9187	0.9388	0.0850
NOD1	0.8831	1.1668	0.9019	0.8036	0.9388	0.0789
LOC642176	0.9643	0.9848	0.8840	0.9223	0.9388	0.0225
LOC648287	0.9264	0.9671	0.9932	0.8687	0.9388	0.0271
LOC651575	0.8769	1.0057	1.1010	0.7718	0.9388	0.0722
CCDC96	0.7948	1.1429	1.0684	0.7494	0.9389	0.0979
LOC1001326	0.8630	1.0599	0.9723	0.8604	0.9389	0.0480
IFNAR1	0.9214	1.2538	0.8752	0.7052	0.9389	0.1148
LOC1001337	0.8572	1.0401	1.0831	0.7753	0.9389	0.0733
EMR2	0.8696	1.0466	0.9651	0.8745	0.9389	0.0421
LOC642469	0.9543	0.9746	0.9136	0.9133	0.9390	0.0153
LOC652655	0.9696	0.9926	0.9906	0.8030	0.9390	0.0456
C1orf64	0.8684	1.0085	1.0403	0.8388	0.9390	0.0501
NR2F1	0.7991	1.0301	1.0417	0.8850	0.9390	0.0587
LOC647998	0.9096	0.9823	1.0074	0.8567	0.9390	0.0344
LOC652563	1.0188	1.0474	0.8551	0.8347	0.9390	0.0548
PGF	0.7970	1.0343	0.9888	0.9359	0.9390	0.0514
RAET1K	0.8495	0.9898	1.0152	0.9016	0.9390	0.0385

C2orf89	0.9011	1.0391	0.9219	0.8940	0.9390	0.0339
C4BPB	0.9271	1.0625	0.8672	0.8992	0.9390	0.0429
LOC646561	0.8992	1.0959	0.9522	0.8088	0.9390	0.0601
LOC643187	0.9327	1.1418	0.9199	0.7616	0.9390	0.0780
LOC653060	0.9825	1.0315	0.8968	0.8454	0.9390	0.0418
SH3PXD2A	0.8774	1.0696	0.9348	0.8743	0.9390	0.0457
SEC61B	0.8592	1.1098	1.0897	0.6974	0.9390	0.0986
DFNA5	0.9105	1.1501	0.8635	0.8321	0.9390	0.0722
C6orf199	0.8491	1.0719	0.9483	0.8869	0.9390	0.0488
REG3G	0.9202	0.9803	0.9579	0.8979	0.9390	0.0185
LOC1001303	0.8571	1.0533	0.9557	0.8901	0.9391	0.0433
LCMT1	0.8518	1.3356	0.9035	0.6653	0.9391	0.1418
LOC731039	0.8747	1.1052	0.9070	0.8694	0.9391	0.0560
SEC1	0.8742	1.1054	1.0009	0.7759	0.9391	0.0721
T	0.8271	0.9821	1.0471	0.9001	0.9391	0.0479
ACHE	0.9165	0.9985	0.9436	0.8979	0.9391	0.0219
ANKRD38	0.8539	1.1094	0.9071	0.8862	0.9391	0.0578
LOC400708	0.9438	1.0392	0.8736	0.9000	0.9391	0.0364
LOC729421	0.7475	1.2592	0.9761	0.7739	0.9392	0.1183
LOC1001333	0.8924	1.0472	0.9374	0.8797	0.9392	0.0381
LOC651859	0.8287	1.0541	1.0145	0.8593	0.9392	0.0559
C17orf49	1.0113	1.2307	0.8623	0.6523	0.9392	0.1219
LOC652813	1.0004	1.0275	0.8766	0.8522	0.9392	0.0438
LOC643684	0.9574	1.0174	0.9153	0.8666	0.9392	0.0320
B3GALT6	0.9215	1.1852	0.9176	0.7324	0.9392	0.0931
LOC1000497	0.7837	1.0893	1.0225	0.8613	0.9392	0.0705
LOC650247	0.7969	1.1141	1.0403	0.8054	0.9392	0.0811
LOC375010	0.8872	0.9038	1.0121	0.9537	0.9392	0.0281
LOC648059	0.7050	1.0737	1.1211	0.8569	0.9392	0.0970
LOC652605	0.8694	0.9966	0.9573	0.9335	0.9392	0.0266
LOC650788	0.9113	1.0286	0.9441	0.8728	0.9392	0.0332
POU6F1	0.8697	1.0169	0.9574	0.9128	0.9392	0.0315
NBPF5	0.8974	1.0457	0.9582	0.8556	0.9392	0.0413
LOC1001327	0.9400	0.9761	0.9712	0.8696	0.9392	0.0245
SYT15	0.8238	1.0780	0.9899	0.8652	0.9392	0.0582
LOC1001340	0.8441	0.9842	1.0866	0.8419	0.9392	0.0594
FLJ42709	0.7948	1.0548	1.0389	0.8685	0.9393	0.0640
EXOC1	0.9110	1.1605	0.9489	0.7367	0.9393	0.0870
LOC1001294	0.8912	1.0818	0.8974	0.8868	0.9393	0.0475
LOC727833	0.8469	1.0057	0.9889	0.9157	0.9393	0.0365
FCGR2A	0.8744	1.0073	0.9714	0.9041	0.9393	0.0304
LOC441873	0.8960	1.1161	0.9302	0.8150	0.9393	0.0637
OR2T11	0.9389	0.8415	1.0576	0.9193	0.9393	0.0447
CNFN	0.9669	1.0956	0.9232	0.7717	0.9393	0.0668
LOC401845	0.9589	0.9170	1.0425	0.8390	0.9393	0.0424
LINS1	0.8608	1.0962	1.0198	0.7806	0.9393	0.0721

CPEB1	0.9261	0.9965	0.9167	0.9181	0.9393	0.0192
LOC647943	0.8753	1.0721	0.9330	0.8771	0.9394	0.0462
SLC39A7	0.8827	0.9945	0.9592	0.9210	0.9394	0.0241
PRRT3	0.7764	1.1779	1.0443	0.7589	0.9394	0.1029
LOC440577	0.9268	1.1040	0.8599	0.8668	0.9394	0.0569
LOC731157	0.8914	1.1140	0.9383	0.8138	0.9394	0.0636
AGXT	0.9028	1.1192	0.9483	0.7872	0.9394	0.0689
LOC1001336	0.8438	0.9437	0.9603	1.0097	0.9394	0.0348
LOC731312	0.9136	0.9394	1.0108	0.8938	0.9394	0.0256
LOC1001343	0.9723	1.0728	0.9413	0.7712	0.9394	0.0627
DSCR8	0.8603	0.9942	1.0061	0.8971	0.9394	0.0359
MIR453	0.8682	1.0310	0.9823	0.8761	0.9394	0.0401
RTP2	0.8962	1.0211	0.9949	0.8454	0.9394	0.0413
ITGB3	0.8986	0.9514	1.0354	0.8722	0.9394	0.0360
LOC646750	0.9164	0.8845	1.0942	0.8625	0.9394	0.0528
LOC642680	0.9171	0.9711	0.9326	0.9368	0.9394	0.0114
TMEM131	0.8009	1.3594	0.8623	0.7351	0.9394	0.1424
NXF5	0.8932	1.0497	0.9855	0.8294	0.9394	0.0488
KLHL22	0.8769	1.3427	0.7541	0.7841	0.9395	0.1369
AGXT2	0.8242	1.0257	1.0979	0.8101	0.9395	0.0722
KIF9	0.9388	1.0717	0.9646	0.7828	0.9395	0.0596
ODF3B	0.9049	1.0584	0.9644	0.8302	0.9395	0.0482
CTGLF3	0.7914	1.3868	0.8452	0.7345	0.9395	0.1508
MFSD6L	0.8575	1.2331	0.8821	0.7852	0.9395	0.1000
ZC3H7A	0.8544	1.2969	0.8332	0.7734	0.9395	0.1204
EFNB1	0.9042	1.0149	1.0193	0.8195	0.9395	0.0481
SMARCA5	0.7949	1.0824	1.1803	0.7004	0.9395	0.1142
KPTN	0.8240	1.0660	0.9971	0.8709	0.9395	0.0558
PDE6H	0.8383	0.9630	0.9665	0.9902	0.9395	0.0343
CCDC135	0.8729	1.0800	1.0325	0.7726	0.9395	0.0711
STIM2	0.7558	1.1507	1.0593	0.7923	0.9395	0.0976
LOC440518	0.8705	1.0819	0.9765	0.8293	0.9395	0.0567
GPR44	0.8152	1.0485	1.0112	0.8832	0.9395	0.0545
WFDC3	0.8986	1.0381	0.9535	0.8679	0.9395	0.0373
EVC	0.8861	1.1458	0.8817	0.8446	0.9395	0.0694
TGFBR1	0.9266	1.0174	0.9629	0.8513	0.9396	0.0348
RAB40A	0.9869	0.9938	0.9463	0.8313	0.9396	0.0376
LOC283584	0.8936	0.9934	0.9453	0.9259	0.9396	0.0209
LOC1001308	0.9404	0.9915	0.9548	0.8717	0.9396	0.0250
DIXDC1	0.8931	1.0284	0.9417	0.8951	0.9396	0.0317
LOC649169	0.8223	1.0459	1.0902	0.7999	0.9396	0.0749
FRMD3	0.8816	1.1192	0.9353	0.8223	0.9396	0.0642
A2BP1	0.8900	1.0490	0.9801	0.8393	0.9396	0.0467
LOC645688	0.7696	1.1186	1.1768	0.6934	0.9396	0.1217
CASC4	0.7720	1.3475	0.8591	0.7798	0.9396	0.1374
CAPN2	1.0259	1.0650	0.8847	0.7829	0.9396	0.0650

PLVAP	0.8892	1.0736	0.9490	0.8467	0.9396	0.0493
LOXHD1	0.7316	1.1515	0.9676	0.9078	0.9396	0.0866
LOC391817	0.9557	1.0715	0.8897	0.8417	0.9396	0.0498
PRAMEF12	0.9112	1.0997	0.9532	0.7945	0.9396	0.0630
LOC1001295	0.9461	0.9897	0.9625	0.8604	0.9397	0.0279
AMELX	0.9138	1.0066	0.9087	0.9295	0.9397	0.0227
STAC3	0.8691	1.0643	0.9645	0.8609	0.9397	0.0477
COL13A1	0.8009	1.0748	1.0032	0.8798	0.9397	0.0613
AIFM3	0.8682	1.0264	0.9708	0.8934	0.9397	0.0362
RGPD8	0.7488	1.2962	0.9561	0.7577	0.9397	0.1281
RMRP	0.8361	1.0162	1.0637	0.8428	0.9397	0.0587
TMPRSS11E	0.8737	1.0337	0.9392	0.9123	0.9397	0.0341
MIR206	0.9770	1.0590	0.9270	0.7958	0.9397	0.0551
LOC729081	0.8451	1.0642	0.9215	0.9282	0.9397	0.0456
LOC648763	0.7790	1.1252	0.9899	0.8648	0.9397	0.0755
WDR24	0.9020	1.3249	0.8103	0.7218	0.9397	0.1336
AQP6	0.8923	1.0175	0.9587	0.8905	0.9398	0.0304
ARHGEF1	0.9117	1.0878	0.9102	0.8493	0.9398	0.0514
LOC730392	0.7068	1.1396	1.0198	0.8930	0.9398	0.0926
NARF	0.9517	1.3349	0.7937	0.6788	0.9398	0.1431
LOC644424	0.9265	1.0297	1.0038	0.7990	0.9398	0.0518
SIK1	0.9400	0.9859	0.9076	0.9257	0.9398	0.0167
TBX6	0.8874	1.0864	0.8874	0.8979	0.9398	0.0489
RAB34	0.9008	1.0814	0.9960	0.7811	0.9398	0.0645
LOC728734	0.9114	1.1109	0.8443	0.8926	0.9398	0.0588
FBXO34	0.7709	1.3918	0.9503	0.6463	0.9398	0.1631
LOC646966	0.8103	1.0217	1.2374	0.6899	0.9398	0.1206
LRRC67	0.9012	1.0075	0.9826	0.8680	0.9398	0.0330
OR51E1	0.9171	1.0132	0.9488	0.8802	0.9398	0.0282
LOC653495	0.8838	1.0254	0.9670	0.8831	0.9398	0.0347
LOC652786	0.9016	0.9528	0.9428	0.9621	0.9398	0.0133
LOC646508	0.8518	1.1940	0.9857	0.7278	0.9398	0.0997
GCG	0.7094	1.1726	1.0582	0.8192	0.9398	0.1064
LOC642082	0.9834	1.2082	0.8291	0.7387	0.9399	0.1027
DNAJC19	0.7810	1.3530	0.9131	0.7123	0.9399	0.1439
C16orf30	0.9658	0.9349	0.9891	0.8696	0.9399	0.0259
LOC646653	0.9614	1.0111	0.9385	0.8484	0.9399	0.0340
LOC732146	0.6413	1.1401	1.0300	0.9481	0.9399	0.1070
LOC642269	0.8443	1.0582	1.0262	0.8308	0.9399	0.0595
MBD2	0.8765	0.9961	1.0212	0.8657	0.9399	0.0401
LOC646943	0.8788	1.1331	0.8958	0.8519	0.9399	0.0650
SLC38A8	0.8619	1.0469	0.9902	0.8606	0.9399	0.0469
MSGN1	0.8977	1.0203	0.9688	0.8728	0.9399	0.0336
AHCYL2	0.8846	1.2195	0.8593	0.7962	0.9399	0.0950
GP9	0.8866	0.9424	0.9855	0.9451	0.9399	0.0203
LOC121838	0.9455	1.0610	0.8859	0.8673	0.9399	0.0437

ARAP3	0.9668	1.0062	0.9511	0.8356	0.9399	0.0367
LOC645276	0.9219	0.9401	1.0290	0.8687	0.9399	0.0333
DCTN4	0.8343	1.2282	0.9814	0.7159	0.9399	0.1104
C12orf35	0.9186	1.0820	0.9681	0.7911	0.9399	0.0603
LOC339782	0.8306	1.0335	1.0428	0.8529	0.9399	0.0569
SIGLEC5	0.8075	0.9928	1.0436	0.9159	0.9400	0.0514
C19orf2	0.8357	1.1532	1.0724	0.6985	0.9400	0.1050
SFRP1	0.9179	1.0269	0.9371	0.8779	0.9400	0.0315
LOC727815	0.8830	1.0199	1.0078	0.8492	0.9400	0.0433
LOC1001292	0.9741	1.0611	0.8971	0.8277	0.9400	0.0502
LOC652894	0.9318	1.0423	0.9457	0.8403	0.9400	0.0413
RPS28	0.6445	1.0728	1.3794	0.6634	0.9400	0.1767
LOC402282	0.8675	1.0541	0.9582	0.8803	0.9400	0.0430
OFD1	0.8052	1.2994	0.8256	0.8299	0.9400	0.1199
LOC650861	0.8607	1.0678	0.9476	0.8841	0.9400	0.0464
BRI3P1	0.7166	1.2088	1.1527	0.6821	0.9400	0.1396
LOC652875	0.8334	0.9904	1.0442	0.8922	0.9400	0.0475
MTA3	0.8939	1.3189	0.9501	0.5973	0.9401	0.1481
LOC643226	0.8274	1.0363	1.0178	0.8787	0.9401	0.0514
GLI1	0.8385	1.0908	0.9462	0.8848	0.9401	0.0549
STXBP5	0.8806	1.0926	0.9981	0.7890	0.9401	0.0664
DKFZP564J1(	0.8042	1.0448	1.0028	0.9086	0.9401	0.0535
CHRM5	0.9881	0.9059	0.9270	0.9395	0.9401	0.0174
LOC648403	0.8342	1.1752	0.9224	0.8286	0.9401	0.0813
LOC440944	0.8617	1.0172	1.0222	0.8595	0.9401	0.0459
LOC649956	0.9633	0.9990	0.9429	0.8553	0.9401	0.0306
LOC650174	0.9050	0.9234	1.0942	0.8379	0.9401	0.0545
MPP2	0.8824	1.1814	0.9708	0.7259	0.9401	0.0950
LOC729409	0.9175	1.0740	0.9383	0.8307	0.9401	0.0503
TPO	0.9275	1.0143	0.9362	0.8826	0.9401	0.0274
OR10AG1	0.8581	1.0304	1.0158	0.8564	0.9402	0.0480
PSMA7	0.8525	1.0423	0.9461	0.9197	0.9402	0.0393
LOC642356	0.8175	1.0843	0.9577	0.9012	0.9402	0.0560
LOC1001300	0.9296	1.0213	0.9719	0.8379	0.9402	0.0389
LOC653333	0.7969	1.3044	0.8555	0.8039	0.9402	0.1221
LOC342918	0.9190	1.0442	0.8732	0.9242	0.9402	0.0365
LOC1001319	0.8563	1.0500	0.9877	0.8668	0.9402	0.0472
MYPN	0.8879	1.0896	0.9381	0.8452	0.9402	0.0533
LOC643149	0.8679	1.0641	0.9669	0.8620	0.9402	0.0478
MIR588	0.8186	0.9799	1.0048	0.9576	0.9402	0.0417
LOC729639	0.7604	1.0922	1.0911	0.8172	0.9402	0.0882
KRT4	0.8731	0.9407	0.9904	0.9567	0.9402	0.0247
SDPR	0.8826	1.0085	1.0131	0.8566	0.9402	0.0411
MYO10	0.8980	1.0671	1.0807	0.7150	0.9402	0.0858
LOC150763	0.8898	1.0340	1.0110	0.8261	0.9402	0.0495
LOC388885	0.7253	1.0280	1.0046	1.0030	0.9402	0.0719

TMPRSS12	0.8984	1.0989	0.9748	0.7889	0.9402	0.0652
LOC728278	0.8668	1.0797	1.0036	0.8109	0.9402	0.0616
EIF4E1B	0.9140	1.0409	1.0201	0.7860	0.9402	0.0584
SLC15A1	0.8557	1.0624	0.9582	0.8847	0.9402	0.0461
LRRK2	0.8859	0.9482	1.0475	0.8794	0.9402	0.0390
NRIP1	0.8398	1.2395	0.9415	0.7402	0.9403	0.1079
LOC1001326	0.6681	1.0763	1.4692	0.5474	0.9403	0.2095
BTN2A1	0.8853	1.2196	0.9897	0.6665	0.9403	0.1149
GBP2	0.7914	1.1837	0.9527	0.8333	0.9403	0.0880
C4orf16	0.9304	1.0273	0.9334	0.8701	0.9403	0.0324
RNF2	0.8943	0.9898	0.9326	0.9446	0.9403	0.0197
LOC401817	0.9381	1.0303	1.0228	0.7701	0.9403	0.0605
STH	0.8729	0.9350	0.9777	0.9757	0.9403	0.0245
INPP4A	0.8597	1.0510	0.9573	0.8932	0.9403	0.0421
LOC728637	0.8824	0.9969	1.0424	0.8396	0.9403	0.0475
FILIP1	0.8051	1.0867	0.9561	0.9133	0.9403	0.0582
FAM150B	0.8814	1.0120	0.9642	0.9038	0.9403	0.0296
LOC1001289	0.7311	1.0936	1.2577	0.6790	0.9403	0.1403
LOC1001286	0.9409	0.9749	0.8930	0.9527	0.9403	0.0173
GRIK3	0.8295	1.1177	0.9222	0.8920	0.9404	0.0622
SULT1A3	0.8956	1.1032	0.9323	0.8303	0.9404	0.0582
ANKRD7	0.9449	1.0518	0.9358	0.8291	0.9404	0.0455
LOC1001331	0.8007	1.1199	0.8410	0.9999	0.9404	0.0737
LOC649246	0.9782	1.0607	0.8990	0.8236	0.9404	0.0510
LOC645203	0.7688	1.0523	1.0942	0.8463	0.9404	0.0788
LOC1001312	0.9067	0.9991	0.9977	0.8581	0.9404	0.0349
SLC26A4	0.9498	1.0028	0.9414	0.8676	0.9404	0.0278
FLJ45721	0.9479	1.0786	0.8738	0.8614	0.9404	0.0499
WBSCR17	0.8547	1.0993	0.9610	0.8466	0.9404	0.0590
C11orf59	0.7936	1.2586	0.9613	0.7481	0.9404	0.1156
LOC643821	0.8903	1.0522	0.9691	0.8501	0.9404	0.0447
LOC1001327	0.8424	0.9512	1.0475	0.9205	0.9404	0.0424
LOC134466	0.7703	1.1197	1.0334	0.8384	0.9404	0.0817
ZGPAT	0.8348	1.1385	0.9656	0.8228	0.9404	0.0735
LOC650845	0.9244	0.9577	0.9926	0.8871	0.9404	0.0226
LOC645744	0.9241	0.9587	1.0025	0.8764	0.9404	0.0267
NEUROD2	0.8949	1.0343	0.9829	0.8497	0.9404	0.0417
LOC652456	0.6909	0.7750	1.1845	1.1114	0.9405	0.1219
FAM26A	0.9296	0.8925	0.9621	0.9777	0.9405	0.0189
SNORD18A	0.7741	1.1017	0.9913	0.8948	0.9405	0.0697
ALS2	0.7823	1.3314	0.8570	0.7913	0.9405	0.1314
PSKH2	0.9019	1.0016	0.9058	0.9525	0.9405	0.0234
LOC650852	1.0501	0.9630	0.8442	0.9046	0.9405	0.0439
LOC646422	0.9331	0.9131	0.9887	0.9270	0.9405	0.0166
KIAA1462	0.8533	1.0238	0.9235	0.9616	0.9405	0.0357
WDR38	0.8690	1.0462	0.9853	0.8616	0.9405	0.0452

PSG2	0.8858	1.0779	0.9216	0.8768	0.9405	0.0468
STOML3	0.8497	1.0638	0.8864	0.9622	0.9405	0.0473
TUBB2B	0.9659	0.9443	0.9511	0.9008	0.9405	0.0140
FAM153B	0.8868	1.0121	0.9280	0.9352	0.9405	0.0261
ELN	0.9473	0.9700	0.9409	0.9039	0.9405	0.0137
LOC729602	0.8931	0.9746	1.0189	0.8755	0.9405	0.0339
PRPF4B	0.8535	1.0711	0.9770	0.8607	0.9405	0.0519
MAZ	0.8515	1.1420	0.9496	0.8192	0.9405	0.0726
KIAA2010	0.7494	1.2561	0.9580	0.7987	0.9406	0.1142
LOC390975	0.8436	1.0569	1.0139	0.8479	0.9406	0.0555
GDPD5	0.9531	0.9808	1.0037	0.8247	0.9406	0.0400
UROD	0.8780	1.1488	1.0599	0.6755	0.9406	0.1048
IL21	0.8083	0.9803	1.0497	0.9240	0.9406	0.0510
LOC643634	0.8641	1.0919	0.9138	0.8926	0.9406	0.0514
LOC644037	0.8852	0.9637	1.0610	0.8525	0.9406	0.0464
LOC652071	0.8869	1.0436	1.0568	0.7751	0.9406	0.0673
LOC728116	0.8858	0.9854	0.9780	0.9133	0.9406	0.0244
LOC1001333	0.9010	1.0645	0.9810	0.8160	0.9406	0.0533
LOC440131	0.8716	1.0883	0.9268	0.8760	0.9406	0.0508
NCRNA0015[illegible]	0.8520	1.0364	0.9650	0.9094	0.9407	0.0394
IGSF9B	0.9113	1.0591	0.9311	0.8612	0.9407	0.0421
C20orf199	0.8400	1.2782	0.8747	0.7700	0.9407	0.1146
LOC645402	0.9651	1.0154	0.8576	0.9249	0.9407	0.0333
LOC728498	0.9516	0.9712	0.9909	0.8493	0.9407	0.0315
LOC643817	0.7934	0.9829	1.0327	0.9540	0.9407	0.0517
HCG4	0.9060	1.0462	1.0009	0.8099	0.9407	0.0525
MMP24	1.0153	1.0131	0.9716	0.7630	0.9408	0.0601
SLC6A18	0.8826	1.0817	0.9574	0.8414	0.9408	0.0528
FLJ36492	0.8737	0.9703	0.9989	0.9201	0.9408	0.0277
PPYR1	0.9119	0.9628	0.9773	0.9110	0.9408	0.0172
AMIGO2	0.7084	1.0328	1.1040	0.9178	0.9408	0.0864
LOC1001298	0.9531	1.0682	0.8956	0.8461	0.9408	0.0478
LOC650482	0.8211	1.1092	1.0340	0.7988	0.9408	0.0772
FLJ16126	0.9251	1.0363	0.9002	0.9015	0.9408	0.0323
LOC730833	0.8469	1.0188	0.9685	0.9289	0.9408	0.0363
LOC1001321	0.9276	1.0326	0.8750	0.9279	0.9408	0.0330
LCE2C	0.8017	0.9937	1.0222	0.9456	0.9408	0.0490
FLJ31945	0.9354	1.0252	0.9018	0.9009	0.9408	0.0293
PCBD2	0.9010	1.0820	0.9214	0.8589	0.9408	0.0488
OPN3	0.9495	1.0079	0.9396	0.8662	0.9408	0.0291
C19orf38	0.8230	1.1383	0.8390	0.9631	0.9408	0.0729
ATG16L1	0.9467	1.1943	0.8739	0.7485	0.9408	0.0939
LOC1001299	0.9055	1.0860	1.0375	0.7344	0.9408	0.0787
LOC653885	0.8696	1.0673	0.9642	0.8623	0.9408	0.0481
C14orf45	0.8767	1.0744	0.9990	0.8133	0.9409	0.0589
LOC643195	0.8552	1.2081	0.8635	0.8366	0.9409	0.0893

C1orf201	0.8125	0.9074	1.1358	0.9078	0.9409	0.0687
LOC654256	0.9251	1.0815	0.9561	0.8008	0.9409	0.0576
CPO	0.9088	1.0670	0.9290	0.8587	0.9409	0.0446
LOC651386	0.9067	0.9879	1.0181	0.8508	0.9409	0.0381
LOC641785	0.8395	1.1440	1.0170	0.7631	0.9409	0.0861
RNU1F1	0.9437	1.1380	0.9008	0.7812	0.9409	0.0742
ZNF205	0.9418	1.1037	0.9011	0.8170	0.9409	0.0602
MTNR1A	0.8203	1.0401	1.0040	0.8995	0.9409	0.0501
FAM5B	0.9594	1.0574	0.8942	0.8528	0.9410	0.0446
OR6M1	0.8982	0.9890	0.9733	0.9033	0.9410	0.0234
TDO2	0.8839	1.0407	0.9620	0.8773	0.9410	0.0384
DICER1	0.7915	1.1165	0.9923	0.8635	0.9410	0.0717
TTC7B	0.9557	1.0015	0.9270	0.8797	0.9410	0.0256
LOC643899	0.8366	0.9549	1.0606	0.9120	0.9410	0.0468
PSMB11	0.9152	0.8742	1.0103	0.9644	0.9410	0.0295
LOC647834	0.9494	1.1215	0.9248	0.7683	0.9410	0.0723
ZNF48	0.8987	1.0423	0.9787	0.8443	0.9410	0.0436
LOC646019	0.9002	0.9308	0.9895	0.9436	0.9410	0.0186
LOC652699	0.9463	1.0023	0.8302	0.9853	0.9410	0.0388
FLJ45983	0.8969	1.0477	0.8883	0.9312	0.9410	0.0368
LOC643509	0.9148	1.2714	0.9058	0.6722	0.9410	0.1236
ACBD5	0.8336	1.1372	0.9630	0.8303	0.9410	0.0723
MC4R	1.0061	0.8373	0.9605	0.9603	0.9410	0.0362
LOC391504	1.0020	0.9663	0.9680	0.8279	0.9411	0.0386
DHRS8	0.9102	0.9159	1.0347	0.9034	0.9411	0.0313
KIAA1107	1.0457	1.0483	0.8784	0.7918	0.9411	0.0637
DOC2A	0.8433	1.1157	0.9347	0.8705	0.9411	0.0613
NLRP6	0.9274	0.9494	0.9372	0.9502	0.9411	0.0054
C9orf36	0.8615	0.9885	1.0544	0.8599	0.9411	0.0483
LOC442388	0.8621	0.9775	1.1140	0.8107	0.9411	0.0674
COBL	0.9212	1.3944	0.8146	0.6341	0.9411	0.1623
SECTM1	0.9700	0.9383	0.9748	0.8812	0.9411	0.0215
OR4F16	0.9364	0.9614	0.9798	0.8867	0.9411	0.0202
TCL6	0.9421	1.0284	0.9525	0.8414	0.9411	0.0384
TDRG1	0.9035	1.0213	0.8819	0.9577	0.9411	0.0311
RNASE13	0.9513	1.0760	0.9928	0.7443	0.9411	0.0705
SEPSECS	0.8777	1.2036	0.9084	0.7747	0.9411	0.0920
EIF3B	0.8007	1.3525	0.9128	0.6985	0.9411	0.1439
LOC1001287	0.8909	0.9385	0.9960	0.9390	0.9411	0.0215
C10orf62	0.9536	1.0213	0.9336	0.8559	0.9411	0.0340
LOC643047	0.9019	1.0002	0.8545	1.0080	0.9412	0.0376
ANO3	0.8343	1.0392	0.9724	0.9188	0.9412	0.0433
APBB3	0.8244	1.2838	0.8953	0.7612	0.9412	0.1175
SNORD69	0.7691	1.2955	0.9530	0.7471	0.9412	0.1268
PARP3	0.9199	1.1170	0.9445	0.7833	0.9412	0.0685
TFAP2E	0.9486	1.0076	0.9873	0.8213	0.9412	0.0418

HEATR4	0.8615	1.0749	1.0078	0.8206	0.9412	0.0600
LOC1001333	0.9124	1.0692	0.8666	0.9166	0.9412	0.0441
LOC644790	0.6785	1.1566	1.1790	0.7508	0.9412	0.1317
SNORA20	0.8827	1.0256	1.0014	0.8551	0.9412	0.0424
AP4B1	0.8487	1.3099	0.9565	0.6498	0.9412	0.1383
SOCS5	0.8737	1.0908	1.0585	0.7419	0.9412	0.0818
TMEM151B	0.9224	1.0731	0.8679	0.9016	0.9412	0.0454
C12orf54	0.8132	0.9518	0.9766	1.0233	0.9412	0.0452
DEFB106A	0.8242	1.1247	0.8916	0.9245	0.9412	0.0646
SERPINB1	0.8528	0.9998	1.0156	0.8968	0.9412	0.0395
LOC651789	0.9571	0.9625	0.9360	0.9094	0.9412	0.0121
LOC1001316	0.9377	1.0674	0.8958	0.8641	0.9413	0.0447
KCNH2	0.9182	1.0509	0.9343	0.8616	0.9413	0.0397
SVIL	0.9156	1.0832	0.9439	0.8224	0.9413	0.0540
WFDC10B	0.8532	1.0310	0.9927	0.8880	0.9413	0.0421
LOC646438	0.8716	0.9725	1.0130	0.9079	0.9413	0.0317
RFWD2	0.8385	1.1872	1.0524	0.6869	0.9413	0.1111
MLLT3	0.7936	1.1210	0.9635	0.8870	0.9413	0.0692
LOC731075	0.9852	1.0104	0.9321	0.8375	0.9413	0.0383
RHOT1	0.7698	1.3084	0.9242	0.7627	0.9413	0.1279
LOC642387	0.9241	1.0101	0.9542	0.8768	0.9413	0.0279
LOC644165	0.8407	1.0705	0.9432	0.9109	0.9413	0.0481
LOC401447	0.8155	1.0226	0.9929	0.9342	0.9413	0.0458
LOC653349	0.9514	0.9902	0.9753	0.8484	0.9413	0.0320
C6orf146	0.9503	0.9880	0.9536	0.8733	0.9413	0.0242
SIPA1	0.9341	1.1526	0.8885	0.7901	0.9413	0.0766
LOC652831	0.8943	0.9955	1.0004	0.8750	0.9413	0.0330
LOC642160	0.9092	1.0212	0.9489	0.8860	0.9413	0.0296
RARA	0.8170	1.1832	0.9410	0.8241	0.9413	0.0855
C12orf55	1.0033	0.9423	0.9515	0.8684	0.9414	0.0278
LOC652871	0.9140	0.9733	0.9811	0.8971	0.9414	0.0210
AQP10	0.9543	0.9274	1.0029	0.8810	0.9414	0.0255
CNOT3	0.8867	1.1546	0.9364	0.7878	0.9414	0.0775
LOC1001342	0.9123	1.0523	0.9464	0.8545	0.9414	0.0416
LOC1001335	0.9226	0.9087	1.1308	0.8034	0.9414	0.0685
LOC441178	0.9276	1.0197	0.9209	0.8975	0.9414	0.0269
PARVG	0.8323	1.1271	1.0230	0.7833	0.9414	0.0806
LOC1001308	0.9317	0.9506	0.9423	0.9412	0.9414	0.0039
INTS2	0.8957	1.0910	1.0038	0.7753	0.9414	0.0683
TRIM42	0.8208	1.1283	0.9571	0.8596	0.9414	0.0686
C11orf55	0.8224	1.0529	1.0058	0.8847	0.9415	0.0532
TNRC4	0.8649	0.9986	0.9849	0.9175	0.9415	0.0311
LOC1001308	0.9994	0.9425	0.9752	0.8488	0.9415	0.0330
OR6P1	0.8197	1.0038	1.0607	0.8816	0.9415	0.0552
R3HCC1	1.0235	1.1205	0.9903	0.6316	0.9415	0.1069
RAD17	0.9729	1.1254	0.8210	0.8466	0.9415	0.0697

ZNF552	0.8429	0.9885	1.0929	0.8416	0.9415	0.0611
ST3GAL6	0.9790	1.0605	0.8999	0.8266	0.9415	0.0504
LOC647306	0.7578	1.0537	1.0236	0.9309	0.9415	0.0666
PHLDA1	0.7612	1.1626	0.9811	0.8612	0.9415	0.0863
ACBD3	0.8939	1.2025	0.9816	0.6881	0.9415	0.1065
LOC1001337	0.7943	0.8942	0.9272	1.1504	0.9415	0.0751
AHRR	0.9003	0.9585	0.9541	0.9534	0.9415	0.0138
LOC389634	0.9881	0.9943	0.9319	0.8519	0.9415	0.0330
BAG1	0.9077	1.0438	0.9328	0.8818	0.9416	0.0356
SNORD113-2	0.9524	1.0332	0.8117	0.9690	0.9416	0.0467
GAS6	0.8616	1.0750	0.9707	0.8591	0.9416	0.0515
LOC1001297	0.9332	0.9395	1.0297	0.8639	0.9416	0.0340
DPY19L2P1	0.8755	1.0765	0.9215	0.8929	0.9416	0.0460
HTR7P	0.8488	1.1174	0.8926	0.9076	0.9416	0.0599
HOXB5	0.8486	1.1938	0.8681	0.8559	0.9416	0.0841
LOC728147	0.8635	1.2156	0.8864	0.8010	0.9416	0.0931
LOC255620	0.8950	1.0216	0.9515	0.8984	0.9416	0.0296
NPW	1.0314	1.0381	0.9095	0.7876	0.9416	0.0592
MIR548P	0.9506	1.0234	1.0077	0.7849	0.9416	0.0545
RTP4	0.8536	0.9625	1.0277	0.9228	0.9416	0.0365
LOC1001328	1.0206	0.9150	0.9815	0.8495	0.9416	0.0376
CYorf15B	0.8169	1.0911	1.0085	0.8501	0.9416	0.0650
LOC651933	0.8527	1.0313	0.9546	0.9281	0.9416	0.0369
LOC729698	0.9183	1.0560	0.9874	0.8049	0.9416	0.0535
RCP9	0.9171	1.1406	0.9415	0.7674	0.9417	0.0767
LOC729668	0.8135	1.0965	1.0501	0.8066	0.9417	0.0766
LOC653536	0.8675	1.0207	1.0214	0.8571	0.9417	0.0459
LOC642276	0.9740	1.1258	0.8951	0.7717	0.9417	0.0742
LOC1001298	0.8437	1.1683	0.8216	0.9331	0.9417	0.0793
RFX6	0.8896	1.1065	0.9331	0.8375	0.9417	0.0583
MIR548M	0.9704	0.9868	0.9577	0.8518	0.9417	0.0306
LOC1001327	0.8013	1.1907	1.0563	0.7185	0.9417	0.1098
PRSS21	0.8472	1.0818	0.9526	0.8851	0.9417	0.0516
C1orf86	0.8511	1.2239	0.9522	0.7396	0.9417	0.1036
LOC441237	0.7936	1.0542	0.9916	0.9274	0.9417	0.0557
IL1B	0.9335	0.9722	0.9916	0.8695	0.9417	0.0269
FLJ38969	0.8645	0.9670	1.1102	0.8252	0.9417	0.0636
LOC728351	1.0879	0.9825	0.9109	0.7855	0.9417	0.0635
RNF216	0.7938	1.1490	1.0238	0.8003	0.9417	0.0874
LOC646537	0.9147	0.9944	1.0754	0.7824	0.9417	0.0624
CD1B	0.8286	1.1023	0.9959	0.8402	0.9418	0.0657
UNQ6975	0.8901	0.9324	1.0525	0.8920	0.9418	0.0382
TCTN2	0.9296	0.8882	1.0108	0.9384	0.9418	0.0255
SCARNA2	0.9723	0.9622	0.9593	0.8733	0.9418	0.0230
TMEM215	0.8357	1.0554	1.0240	0.8521	0.9418	0.0570
LOC196993	0.8410	1.1172	0.8965	0.9125	0.9418	0.0604

LOC652491	0.8460	1.1931	0.9368	0.7912	0.9418	0.0890
LOC285548	0.9630	1.0119	0.9890	0.8033	0.9418	0.0472
TPBG	0.8034	1.1756	0.9337	0.8545	0.9418	0.0824
ADAT3	0.7028	1.0574	1.2763	0.7308	0.9418	0.1375
ASTN1	0.8719	1.0016	0.9880	0.9057	0.9418	0.0315
ANGPT4	0.8880	0.9760	0.9597	0.9436	0.9418	0.0191
LOC389394	0.9661	1.0202	0.9622	0.8189	0.9418	0.0431
NUDT22	0.8761	1.1134	0.9538	0.8241	0.9418	0.0631
LOC653053	0.9131	0.9436	0.9313	0.9794	0.9419	0.0140
LOC1001281	1.0197	1.0294	0.8754	0.8429	0.9419	0.0482
LOC651315	1.0416	0.8493	0.9952	0.8813	0.9419	0.0457
LOC643453	0.8477	0.9524	1.0756	0.8917	0.9419	0.0495
LOC1001338	0.7842	1.0742	1.0607	0.8483	0.9419	0.0737
FBXL21	0.9051	1.1400	0.8892	0.8332	0.9419	0.0678
FBXL7	0.8764	1.0579	0.9595	0.8737	0.9419	0.0435
OR52E2	0.8608	0.9860	1.0823	0.8385	0.9419	0.0570
LOC400750	0.8738	1.0788	0.9383	0.8768	0.9419	0.0480
LOC201140	0.8370	1.0363	0.9863	0.9081	0.9419	0.0438
LOC441119	0.8739	1.0705	1.0011	0.8222	0.9419	0.0570
GOPC	0.9138	1.0747	1.0363	0.7429	0.9419	0.0747
RABGAP1L	0.8015	1.0598	1.0327	0.8738	0.9420	0.0622
LOC439992	0.9075	0.9923	0.9650	0.9031	0.9420	0.0219
FGFBP1	0.9053	0.9550	0.9685	0.9391	0.9420	0.0136
LOC643416	0.8777	1.0203	1.0176	0.8523	0.9420	0.0447
LOC441009	0.6768	1.1003	0.9922	0.9986	0.9420	0.0918
MSH5	0.8873	1.0793	0.9698	0.8316	0.9420	0.0539
LOC648706	0.8445	1.0959	0.9259	0.9016	0.9420	0.0541
SF4	0.9136	1.1220	0.9209	0.8114	0.9420	0.0650
IL17RD	0.8342	1.0953	1.0316	0.8070	0.9420	0.0715
CD70	0.8509	1.1581	0.9160	0.8429	0.9420	0.0739
ERCC-00157	0.9784	1.2000	0.7818	0.8079	0.9420	0.0964
URB1	0.9392	1.1400	0.8487	0.8402	0.9420	0.0697
MIR301A	0.8619	0.9760	1.0048	0.9254	0.9420	0.0313
NTSR2	0.8977	1.0068	0.9807	0.8829	0.9420	0.0305
LRRC38	0.7978	1.0972	1.0633	0.8098	0.9421	0.0801
ADAM32	0.8788	1.0627	0.9857	0.8409	0.9421	0.0506
LOC644714	0.8858	1.0076	0.9480	0.9268	0.9421	0.0254
TBN	1.0379	1.0219	0.8474	0.8611	0.9421	0.0509
LOC650008	0.9224	0.9836	1.0141	0.8482	0.9421	0.0366
MIR760	0.8349	1.0658	1.0838	0.7838	0.9421	0.0774
GAS5	0.8970	1.0783	0.9505	0.8426	0.9421	0.0504
LOC653226	0.8623	1.2342	0.9398	0.7320	0.9421	0.1064
IL22	0.8767	1.1055	0.9412	0.8449	0.9421	0.0580
LOC727963	0.8743	1.1038	0.9777	0.8126	0.9421	0.0638
LOC728064	0.8613	1.0467	0.9472	0.9133	0.9421	0.0391
HNRNPUL2	0.9086	1.3438	0.8353	0.6808	0.9421	0.1421

MYCN	0.9160	0.9573	0.9724	0.9228	0.9421	0.0136
LOC1001324	0.8958	1.0290	0.9704	0.8733	0.9421	0.0356
EREG	0.8512	1.1241	1.0098	0.7835	0.9421	0.0770
SEL1L	0.8597	1.1083	1.0098	0.7908	0.9421	0.0718
LALBA	0.9085	0.9269	1.0184	0.9148	0.9422	0.0257
LOC1001335	0.8552	1.0157	1.0176	0.8802	0.9422	0.0433
HS3ST3A1	0.8452	1.0223	0.9676	0.9337	0.9422	0.0371
MBNL1	0.8043	1.1505	1.0823	0.7316	0.9422	0.1026
IMPG2	0.9079	1.0175	1.0152	0.8281	0.9422	0.0458
LOC388276	0.8569	0.9530	1.0790	0.8800	0.9422	0.0500
LOC728866	0.9129	1.0265	0.9282	0.9014	0.9422	0.0286
MRPS10	0.8230	1.3504	0.9837	0.6118	0.9422	0.1559
NEIL1	0.7819	1.1716	0.9492	0.8662	0.9422	0.0837
LOC649143	0.8739	1.0593	0.9916	0.8440	0.9422	0.0504
MANBAL	0.8286	1.1407	0.9663	0.8332	0.9422	0.0735
B3GNT2	0.7016	1.3640	0.9570	0.7462	0.9422	0.1512
LOC388922	0.7913	1.0611	0.9824	0.9341	0.9422	0.0567
LOC653722	0.7975	1.0840	0.9294	0.9580	0.9422	0.0588
ZNF673	1.0301	1.0401	0.8876	0.8112	0.9422	0.0559
CCDC88A	0.9565	1.0546	0.8846	0.8732	0.9422	0.0417
LOC648980	0.9243	1.1984	0.9472	0.6991	0.9422	0.1021
MGC10981	0.9251	0.9786	0.9830	0.8821	0.9422	0.0240
LOC1001300	0.9183	0.9939	0.9418	0.9150	0.9422	0.0182
PLD4	0.8995	1.0318	1.0231	0.8145	0.9422	0.0522
SLC25A3	0.7761	1.2419	1.0157	0.7353	0.9423	0.1175
LOC654235	0.8947	1.1224	0.8894	0.8625	0.9423	0.0605
LOC727880	0.9078	0.9927	0.9426	0.9259	0.9423	0.0182
LOC728877	0.9684	1.3256	0.8723	0.6027	0.9423	0.1494
LOC441016	0.8735	1.1152	0.8986	0.8817	0.9423	0.0579
CCL27	0.9217	1.0088	0.9957	0.8429	0.9423	0.0383
HLA-DPB2	0.8120	1.0929	0.8912	0.9731	0.9423	0.0600
LOC442465	0.9796	1.0675	0.8920	0.8301	0.9423	0.0518
LOC642073	0.8847	0.9337	1.0667	0.8840	0.9423	0.0431
LOC541472	0.8430	1.0606	0.9839	0.8817	0.9423	0.0494
LOC1001289	0.8903	1.0623	0.9398	0.8768	0.9423	0.0422
LOC727797	0.7974	1.2409	0.9136	0.8174	0.9423	0.1027
LOC728153	0.9311	1.2342	0.8475	0.7566	0.9423	0.1036
LOC727860	0.8883	0.9450	0.9979	0.9382	0.9424	0.0224
TRIM5	0.8435	1.2573	0.8970	0.7716	0.9424	0.1081
OR5B17	0.9618	0.9885	0.9572	0.8619	0.9424	0.0277
LOC1001345	0.7321	1.0903	1.1531	0.7939	0.9424	0.1051
GPR111	0.9146	1.0060	1.0189	0.8300	0.9424	0.0441
MOXD2	0.8824	1.0880	0.9528	0.8464	0.9424	0.0533
LOC1001343	0.9570	1.0643	0.9831	0.7651	0.9424	0.0634
GPR107	0.9084	1.0449	1.0004	0.8157	0.9424	0.0509
LOC651680	0.9084	0.9951	1.0023	0.8637	0.9424	0.0338

LOC647819	0.8318	1.0567	1.0443	0.8368	0.9424	0.0625
CDC42EP3	0.7423	1.2348	0.9891	0.8033	0.9424	0.1107
HIST1H3I	0.8931	0.9984	0.8821	0.9960	0.9424	0.0317
MIR759	0.9177	0.9483	0.9444	0.9592	0.9424	0.0088
WNT16	0.8586	1.0447	0.9647	0.9017	0.9424	0.0404
SLIT1	0.9386	0.9744	0.9729	0.8838	0.9424	0.0212
CALN1	0.8830	1.0726	0.9946	0.8195	0.9424	0.0565
CCDC6	0.7402	1.2156	0.9275	0.8865	0.9425	0.0995
RIBC1	0.9117	1.0461	0.9485	0.8635	0.9425	0.0387
REG1P	0.7999	1.0744	0.9334	0.9622	0.9425	0.0564
LOC650706	0.9658	0.9951	0.9064	0.9027	0.9425	0.0227
CSGALNACT2	0.7052	1.1972	0.9672	0.9003	0.9425	0.1015
TNR	0.8885	1.0288	0.9516	0.9011	0.9425	0.0318
GPN2	0.6965	1.4031	0.9214	0.7491	0.9425	0.1609
LOC653464	0.8973	1.0188	0.9261	0.9279	0.9425	0.0264
ABCA8	0.9118	1.0467	0.9185	0.8932	0.9425	0.0351
TMF1	0.7910	1.1401	1.0000	0.8390	0.9425	0.0796
CCRL2	0.9031	0.9953	1.0651	0.8067	0.9425	0.0561
PRSS3	0.9882	0.9558	0.9230	0.9031	0.9425	0.0187
PLEKHH2	0.8781	0.9720	1.0051	0.9150	0.9425	0.0284
LOC1001281	0.8935	1.1222	0.9157	0.8389	0.9425	0.0620
MIR548F1	0.9390	0.9620	0.9968	0.8724	0.9426	0.0262
LOC654249	0.8848	0.9449	1.0537	0.8869	0.9426	0.0396
LOC646236	0.8397	1.1174	0.9668	0.8463	0.9426	0.0652
LOC1001318	0.8448	0.9930	0.9389	0.9936	0.9426	0.0350
MIR1913	0.9567	0.9519	0.8855	0.9763	0.9426	0.0198
ZCCHC7	0.8487	1.1338	1.0644	0.7234	0.9426	0.0950
TDRD5	1.0142	1.0661	0.8997	0.7903	0.9426	0.0615
CNO	0.7880	1.2408	1.0089	0.7327	0.9426	0.1159
LOC649318	0.9779	0.9309	0.9522	0.9093	0.9426	0.0147
SH3GL1	0.7499	1.1985	1.0054	0.8166	0.9426	0.1010
LOC643502	0.9259	1.0547	0.9285	0.8613	0.9426	0.0405
LOC644830	0.9111	1.0548	0.9476	0.8569	0.9426	0.0418
LOC652803	0.9221	1.0026	1.0615	0.7842	0.9426	0.0600
OR10H1	0.9142	1.0829	0.9948	0.7786	0.9426	0.0646
PKDCC	0.8192	1.0642	0.9754	0.9117	0.9426	0.0517
LOC391039	0.8645	1.0709	0.9879	0.8472	0.9426	0.0530
LOC1001285	0.8617	1.0249	0.9505	0.9334	0.9426	0.0335
LOC1001306	0.9282	1.0209	0.8640	0.9575	0.9426	0.0326
MFAP3L	0.8448	1.0682	0.9833	0.8743	0.9426	0.0514
FAM45B	1.0349	1.0527	0.8993	0.7838	0.9427	0.0631
IQCH	0.9155	1.0065	0.9687	0.8801	0.9427	0.0280
ZNF718	0.8662	1.1469	0.9560	0.8016	0.9427	0.0751
GUCY1B2	0.8763	1.0241	0.9571	0.9132	0.9427	0.0318
MIR572	0.8356	1.1108	0.9460	0.8785	0.9427	0.0605
COL4A4	0.7999	1.0371	0.9896	0.9442	0.9427	0.0513

XKR3	0.9362	0.9808	0.9750	0.8789	0.9427	0.0235
MIR372	0.9740	0.9521	0.9349	0.9099	0.9427	0.0135
LOC727987	0.9539	0.8888	1.0310	0.8972	0.9427	0.0328
LIN28	0.9007	0.9625	0.9909	0.9169	0.9428	0.0207
KIAA1109	1.0110	0.9512	0.9111	0.8978	0.9428	0.0254
LOC645138	0.7321	1.2301	1.2702	0.5387	0.9428	0.1820
MIR451	0.9591	0.8986	0.9026	1.0107	0.9428	0.0265
PTH1R	0.9029	1.0232	1.0047	0.8403	0.9428	0.0432
LOC285735	0.9659	0.9832	0.9838	0.8383	0.9428	0.0351
ZNF569	0.8864	0.9610	1.0385	0.8853	0.9428	0.0365
RPTOR	0.8388	1.0173	1.0680	0.8470	0.9428	0.0586
LOC642278	0.8924	1.0966	0.9035	0.8787	0.9428	0.0515
VPS11	0.9236	1.1080	0.8908	0.8488	0.9428	0.0572
LOC1001291	0.8885	1.0759	0.9620	0.8448	0.9428	0.0505
C2orf48	0.8310	1.0079	0.9860	0.9464	0.9428	0.0394
MED29	0.9169	1.2282	0.9166	0.7095	0.9428	0.1070
TARSL2	0.9264	1.2536	0.8284	0.7628	0.9428	0.1089
C3orf22	0.8453	1.0239	0.9910	0.9111	0.9428	0.0402
COX16	0.9149	1.0628	0.9394	0.8542	0.9428	0.0438
LOC1001295	0.9083	1.0170	1.0438	0.8023	0.9428	0.0553
ASB17	0.8922	0.9931	0.9525	0.9337	0.9428	0.0209
WDR35	0.8297	1.0798	1.0057	0.8562	0.9429	0.0599
STX6	0.8386	1.3659	0.8488	0.7182	0.9429	0.1441
SNORA34	0.8503	1.0605	0.9861	0.8746	0.9429	0.0491
CLDN4	0.9317	0.9741	0.9741	0.8915	0.9429	0.0198
FLT3	0.8362	1.2000	0.9253	0.8100	0.9429	0.0892
LOC729373	0.8690	0.9561	0.9985	0.9479	0.9429	0.0270
ENPP4	0.7766	1.2375	0.9889	0.7685	0.9429	0.1107
ARSI	0.9235	1.0332	0.9587	0.8563	0.9429	0.0368
LOC644204	0.9061	0.9555	1.0539	0.8561	0.9429	0.0422
LOC731419	0.8961	0.9793	1.0483	0.8480	0.9429	0.0444
DDI1	0.8591	1.0459	0.9339	0.9326	0.9429	0.0385
STMN4	0.8347	1.1131	0.9544	0.8693	0.9429	0.0621
KRT13	0.8625	1.0771	0.9498	0.8822	0.9429	0.0485
ID2	0.8667	1.1823	1.0341	0.6886	0.9429	0.1065
LOC644917	0.9867	1.0058	0.9557	0.8236	0.9429	0.0411
SPTBN1	0.8618	1.0183	1.0605	0.8311	0.9429	0.0567
LOC1001329	0.8587	1.0357	0.9700	0.9073	0.9429	0.0384
C19orf39	0.9221	1.0133	0.9051	0.9312	0.9429	0.0241
CTAGE6	0.8591	1.1060	1.0024	0.8043	0.9429	0.0685
LOC650298	0.8459	1.1096	1.0746	0.7417	0.9429	0.0890
TNFRSF10C	0.8438	0.9990	0.9188	1.0102	0.9429	0.0388
LOC1001319	0.8280	1.0740	0.9953	0.8745	0.9429	0.0561
C1orf125	0.8044	1.0805	0.9516	0.9353	0.9429	0.0565
LOC388900	0.8490	1.0885	0.9764	0.8580	0.9430	0.0565
FAM19A1	0.9782	1.0060	1.0768	0.7110	0.9430	0.0801

SLC10A7	0.8207	1.2843	0.9072	0.7597	0.9430	0.1177
RPL35	0.8515	0.9315	1.0658	0.9231	0.9430	0.0447
LOC1001322	0.9267	1.2919	0.7799	0.7735	0.9430	0.1216
KLHL33	0.9485	1.0276	0.9390	0.8569	0.9430	0.0349
DNM1L	0.7513	1.2306	1.0637	0.7264	0.9430	0.1228
WNK2	0.9339	1.0264	1.0209	0.7909	0.9430	0.0549
LOC647347	0.9993	0.9997	0.8989	0.8743	0.9430	0.0330
C5orf36	0.9078	1.0334	0.9813	0.8496	0.9430	0.0404
SNORD114-3	0.8087	1.0712	0.9869	0.9053	0.9430	0.0561
HMX2	0.9088	1.0437	0.9830	0.8367	0.9431	0.0449
GSTM5	0.9491	0.9963	0.9420	0.8849	0.9431	0.0228
LOC441792	0.9195	1.0448	0.9329	0.8751	0.9431	0.0361
LOC1001283	0.9435	1.0533	0.9272	0.8483	0.9431	0.0422
UBR3	0.8596	1.3827	0.7929	0.7371	0.9431	0.1487
LOC285577	0.8710	1.0461	0.9884	0.8669	0.9431	0.0444
LOC645333	0.8725	1.0490	1.0213	0.8295	0.9431	0.0542
VIT	0.9206	0.9963	0.9343	0.9212	0.9431	0.0180
LOC131185	0.9319	1.0044	0.9617	0.8745	0.9431	0.0273
LOC729324	0.7259	1.4044	0.9980	0.6442	0.9431	0.1714
LOC652882	0.9209	0.9020	0.9773	0.9723	0.9431	0.0187
CMYA5	0.8647	1.0102	1.0274	0.8701	0.9431	0.0439
RBP4	0.8676	1.0841	0.9817	0.8391	0.9431	0.0562
MT1P2	0.9253	0.9925	0.9928	0.8619	0.9431	0.0314
MGC51338	0.8975	0.9374	1.0135	0.9242	0.9431	0.0249
IGFN1	0.9468	0.9652	0.9810	0.8796	0.9431	0.0223
LOC1001332	0.9161	0.9583	1.0544	0.8438	0.9432	0.0440
TRNAU1AP	0.9919	1.0412	0.8832	0.8564	0.9432	0.0439
MIR221	0.8670	1.0144	1.0077	0.8836	0.9432	0.0394
LOC1001339	0.8941	1.0612	0.9085	0.9089	0.9432	0.0395
CALHM2	0.6923	1.2298	0.9878	0.8630	0.9432	0.1131
C14orf38	0.7610	1.0980	1.0262	0.8875	0.9432	0.0748
HIST1H4A	0.8791	1.0194	0.9553	0.9192	0.9432	0.0298
ABP1	0.9351	0.9537	0.9250	0.9591	0.9432	0.0080
CHRNA10	0.9112	1.0058	0.9291	0.9268	0.9432	0.0212
TTLL12	0.8252	1.2954	0.9237	0.7287	0.9432	0.1239
LOC1001320	1.0035	0.9731	0.9838	0.8125	0.9432	0.0440
LOC728037	1.1166	1.0572	0.8973	0.7018	0.9432	0.0929
APOL6	0.9240	1.0405	0.9696	0.8389	0.9432	0.0422
SIRPB1	0.8915	0.9785	0.9419	0.9610	0.9432	0.0188
LOC1001315	0.9525	1.0332	0.9057	0.8815	0.9433	0.0334
LOC646670	0.9724	0.9225	1.0445	0.8335	0.9433	0.0443
LOC649754	0.9211	1.0301	0.9289	0.8929	0.9433	0.0300
NLRP8	0.8711	0.9529	0.9853	0.9638	0.9433	0.0250
C20orf56	0.8487	0.9669	1.0548	0.9027	0.9433	0.0443
DIPAS	0.9239	1.0327	0.9861	0.8304	0.9433	0.0437
FAM48A	0.8792	1.0348	0.9614	0.8976	0.9433	0.0352

HBM	0.9448	0.9755	0.9407	0.9122	0.9433	0.0130
SDCCAG3L	0.8962	1.1972	0.8339	0.8458	0.9433	0.0857
LOC1001342	0.9002	1.0077	1.0041	0.8612	0.9433	0.0370
FAM118B	0.9508	1.1914	0.8991	0.7319	0.9433	0.0950
LOC727978	0.9800	1.0697	0.9460	0.7777	0.9433	0.0611
USO1	1.0157	1.0555	1.0154	0.6867	0.9433	0.0861
S100Z	0.9246	0.9605	0.9892	0.8990	0.9433	0.0198
LOC654117	0.9200	0.9899	1.0149	0.8486	0.9433	0.0374
COX7A2	0.8102	1.2528	0.9967	0.7137	0.9434	0.1187
LOC388117	0.8879	1.0162	0.9797	0.8896	0.9434	0.0324
LOC646821	0.8683	1.0773	0.9420	0.8858	0.9434	0.0473
HEMK1	0.8950	1.0549	1.0163	0.8073	0.9434	0.0567
SLC35A1	0.8736	1.1452	0.9033	0.8513	0.9434	0.0681
RPL26	0.8011	1.1507	1.0783	0.7434	0.9434	0.1006
LOC646317	0.9583	0.9737	1.0219	0.8196	0.9434	0.0434
LOC641801	0.9102	1.1183	0.9696	0.7755	0.9434	0.0710
LOC93349	0.9268	0.9895	0.9487	0.9085	0.9434	0.0174
OMP	0.9671	0.9699	0.9981	0.8385	0.9434	0.0357
ZAR1	0.8141	1.0867	0.9843	0.8885	0.9434	0.0591
FCRL5	0.8788	1.1416	0.9269	0.8264	0.9434	0.0692
LOC645465	0.8278	1.0638	1.0429	0.8392	0.9434	0.0636
LOC642141	0.7943	0.9870	1.0603	0.9321	0.9434	0.0562
LOC1001310	0.8963	1.1250	0.9018	0.8506	0.9434	0.0616
LOC1001294	0.8754	0.9730	1.0146	0.9107	0.9434	0.0311
ERCC-00062	0.8797	0.9755	0.9709	0.9477	0.9435	0.0221
AGL	0.8525	1.0773	1.0274	0.8166	0.9435	0.0641
TMEM85	0.8553	1.3398	0.8566	0.7222	0.9435	0.1358
PHYHD1	0.9243	1.0592	0.9945	0.7960	0.9435	0.0564
LOC646300	0.8891	1.0718	0.9279	0.8851	0.9435	0.0438
AP3M1	0.9076	1.2498	0.8918	0.7248	0.9435	0.1101
LOC1001337	0.9120	0.9430	1.0846	0.8344	0.9435	0.0523
ZBED5	0.9364	1.0977	0.9440	0.7959	0.9435	0.0616
DEDD2	0.7821	1.3381	0.8918	0.7620	0.9435	0.1346
LOC283432	0.8906	1.0496	0.8997	0.9342	0.9435	0.0366
LOC653418	0.8877	0.9896	1.0563	0.8404	0.9435	0.0488
LOC347292	0.9062	0.8868	1.2384	0.7426	0.9435	0.1049
ACTBL2	0.8687	1.0497	0.9712	0.8845	0.9435	0.0420
CXorf40A	0.8947	1.2639	0.9497	0.6658	0.9435	0.1232
ATP5EP2	0.8489	1.1227	1.0573	0.7454	0.9435	0.0882
LOC644922	0.9116	1.0156	0.9315	0.9155	0.9436	0.0244
LOC727762	0.9792	1.0188	0.9408	0.8354	0.9436	0.0394
LOC729806	0.9428	0.9832	0.9784	0.8698	0.9436	0.0262
LOC642935	0.9137	1.0409	0.9829	0.8368	0.9436	0.0441
LOC644105	0.9406	0.9910	1.0013	0.8415	0.9436	0.0365
SAMD4A	0.9613	0.9418	1.0408	0.8304	0.9436	0.0434
MIR181B2	0.8179	1.0081	1.0972	0.8512	0.9436	0.0659

OR10G9	0.9929	1.0734	0.8212	0.8869	0.9436	0.0559
THRB	0.8114	1.0312	0.9496	0.9822	0.9436	0.0471
VPRBP	0.8742	1.0956	0.9623	0.8424	0.9436	0.0567
LOC646388	1.0222	0.9929	0.9418	0.8176	0.9436	0.0452
LOC652687	0.9262	1.0288	0.9601	0.8594	0.9436	0.0353
AP1GBP1	0.8562	1.0424	0.9621	0.9139	0.9436	0.0394
LOC390084	0.9020	0.9957	1.0097	0.8671	0.9436	0.0350
TINAG	0.9091	1.1725	0.8907	0.8022	0.9436	0.0798
MIR409	0.9054	1.1082	0.8999	0.8610	0.9436	0.0557
MIR943	0.9090	0.9808	0.9959	0.8888	0.9436	0.0263
LOC644093	0.8941	1.0815	0.9201	0.8789	0.9437	0.0467
PCP4L1	0.8474	0.9829	1.0365	0.9078	0.9437	0.0415
CEP68	0.9220	1.1307	0.8908	0.8312	0.9437	0.0651
EPHA2	0.9189	0.9070	0.9527	0.9961	0.9437	0.0200
ACTB	0.8292	1.2600	0.9577	0.7279	0.9437	0.1154
LOC727937	0.9834	1.0099	0.9430	0.8384	0.9437	0.0377
LOC643018	0.9299	1.0317	0.8942	0.9190	0.9437	0.0303
LOC1001294	0.8933	1.0968	0.9368	0.8479	0.9437	0.0542
OR1G1	0.9814	0.9646	0.9438	0.8850	0.9437	0.0210
CSN1S1	0.8170	1.0065	1.0456	0.9057	0.9437	0.0515
BMP1	0.8588	1.0611	0.9949	0.8600	0.9437	0.0505
EGFL6	0.8810	1.0199	1.0018	0.8721	0.9437	0.0390
LOC391636	0.9089	0.9924	1.0035	0.8700	0.9437	0.0324
LOC344887	0.8639	1.0480	1.1389	0.7241	0.9437	0.0929
KLF2	0.9197	1.2218	0.9753	0.6580	0.9437	0.1157
DHRS12	0.9469	1.0763	0.9364	0.8153	0.9437	0.0533
VCL	0.8945	1.2861	0.9116	0.6827	0.9437	0.1254
LOC390547	0.8300	0.9860	0.9777	0.9812	0.9437	0.0379
MIR95	0.9033	1.0159	0.9143	0.9416	0.9437	0.0254
TTC9C	0.8675	1.2788	0.9198	0.7089	0.9437	0.1203
LOC644707	0.9409	0.9148	0.9710	0.9483	0.9438	0.0116
MIR338	0.9256	0.9607	0.9721	0.9166	0.9438	0.0134
C2CD4B	0.9764	1.0634	0.9035	0.8317	0.9438	0.0496
LOC644607	0.7922	0.9033	1.1224	0.9572	0.9438	0.0687
LOC645994	0.8957	0.9843	0.9671	0.9280	0.9438	0.0199
PGR	0.8177	1.0229	1.0116	0.9229	0.9438	0.0476
RAD18	0.8445	1.0054	1.0365	0.8888	0.9438	0.0459
NFASC	0.8681	1.0975	0.9887	0.8208	0.9438	0.0622
LOC1001319	0.9223	1.2148	0.8585	0.7797	0.9438	0.0949
FKBP7	0.9048	1.0482	0.9455	0.8767	0.9438	0.0376
GNG12	0.6171	1.4218	0.9918	0.7446	0.9438	0.1773
TMEM38A	0.9089	1.0331	0.9028	0.9306	0.9438	0.0303
SLC22A6	0.9493	1.0829	0.9057	0.8375	0.9438	0.0518
LOC1001340	0.9397	1.0451	0.9492	0.8413	0.9438	0.0416
NBL1	0.9040	0.9958	1.0182	0.8575	0.9438	0.0379
LOC1001278	0.9072	1.0200	0.9606	0.8876	0.9439	0.0297

ERCC-00079	0.9399	0.9926	0.9540	0.8890	0.9439	0.0214
C21orf54	0.8890	1.1228	0.9289	0.8348	0.9439	0.0627
LOC1001297	0.8877	1.1215	0.8807	0.8857	0.9439	0.0592
SLC47A2	0.8683	1.0206	0.9726	0.9141	0.9439	0.0333
AP1G1	0.8046	1.3084	0.9049	0.7577	0.9439	0.1253
RAD52	0.9234	0.9697	0.9782	0.9043	0.9439	0.0179
LOC653431	0.9133	1.0713	0.9880	0.8030	0.9439	0.0570
CLDN16	0.8684	1.0874	0.9492	0.8707	0.9439	0.0514
LOC646845	0.9460	1.0654	0.9431	0.8212	0.9439	0.0499
LOC1001328	0.9097	0.9631	1.0226	0.8803	0.9439	0.0313
LOC647480	0.8170	1.1223	0.9827	0.8536	0.9439	0.0693
YIPF5	0.8747	1.1162	0.8851	0.8999	0.9439	0.0576
LOC641699	0.9526	1.0493	0.9832	0.7908	0.9440	0.0549
ACAP3	0.8626	1.0858	0.9881	0.8394	0.9440	0.0575
LOC643248	0.8841	1.0641	0.9794	0.8483	0.9440	0.0487
SLC16A7	0.9193	1.0128	0.8979	0.9460	0.9440	0.0250
LOC732371	0.8793	1.0524	0.9665	0.8778	0.9440	0.0417
LOC401620	0.8499	1.0586	0.9436	0.9239	0.9440	0.0432
FOLR2	0.8218	1.1283	0.9501	0.8758	0.9440	0.0668
LOC1001299	0.9573	1.0710	0.9270	0.8207	0.9440	0.0515
LOC650087	0.9247	1.0430	0.9684	0.8400	0.9440	0.0424
CD300LB	0.8667	1.0269	0.9146	0.9679	0.9440	0.0345
U2AF1L4	0.8625	1.0425	1.0294	0.8417	0.9440	0.0533
RCOR1	0.8796	1.0352	0.8605	1.0009	0.9440	0.0435
LOC653562	0.9645	1.0181	0.9328	0.8609	0.9440	0.0328
PSENEN	0.7690	1.2584	0.9498	0.7990	0.9440	0.1120
LOC652628	0.8651	1.0976	0.8931	0.9204	0.9441	0.0524
MIR656	0.8258	0.9708	1.0735	0.9061	0.9441	0.0524
RASA3	0.8709	1.1776	0.7991	0.9286	0.9441	0.0822
LOC442711	0.8719	1.1286	0.8329	0.9430	0.9441	0.0656
NOS1	0.9926	0.9804	0.9257	0.8777	0.9441	0.0265
HLA-DPB1	0.8931	1.0091	0.9614	0.9127	0.9441	0.0260
PAK3	0.9462	0.9289	1.0214	0.8798	0.9441	0.0294
SLC18A1	0.8962	0.9918	1.0341	0.8543	0.9441	0.0416
PTPLAD2	0.9187	1.0571	1.0407	0.7600	0.9441	0.0687
LOC1001336	0.8356	1.0042	1.0808	0.8558	0.9441	0.0591
CCDC89	0.8856	1.0485	0.9926	0.8498	0.9441	0.0462
EFHC1	0.9129	0.9753	1.0049	0.8834	0.9441	0.0279
LOC652493	0.9612	0.9310	0.9499	0.9343	0.9441	0.0070
KLF15	0.8684	1.1573	0.9464	0.8044	0.9441	0.0768
ZNF646	0.9953	1.0535	0.9297	0.7979	0.9441	0.0549
LOC650339	0.8296	1.0362	1.0049	0.9057	0.9441	0.0472
SFRS14	0.9655	1.2128	0.8251	0.7731	0.9441	0.0984
LOC647082	0.9618	0.9924	0.9572	0.8651	0.9441	0.0275
TCEAL4	0.7963	1.2976	0.8902	0.7925	0.9441	0.1200
ANO9	0.9396	0.9893	0.9696	0.8780	0.9441	0.0243

GUCY1A2	0.9406	1.0172	0.9731	0.8456	0.9441	0.0364
CHCHD6	0.8767	1.1738	0.9700	0.7560	0.9441	0.0882
LOC401623	0.9797	1.0231	0.9601	0.8137	0.9442	0.0454
LOC645868	0.8534	0.9980	1.0615	0.8637	0.9442	0.0511
SAMD5	0.9359	0.9930	1.0110	0.8367	0.9442	0.0392
MX2	0.8958	1.0136	1.0584	0.8090	0.9442	0.0566
FLJ33544	0.8805	0.9542	0.9288	1.0133	0.9442	0.0276
HMX3	0.9238	1.0360	0.9512	0.8657	0.9442	0.0354
LOC1001336	0.8174	1.1146	0.9276	0.9173	0.9442	0.0620
LOC652757	0.8896	1.0947	0.8833	0.9093	0.9442	0.0505
CCDC88B	0.9316	1.0188	0.9845	0.8419	0.9442	0.0385
RRN3P2	0.8826	0.9436	1.0589	0.8918	0.9442	0.0405
MIR602	0.8225	1.0790	1.0250	0.8503	0.9442	0.0635
CALB1	0.9238	0.9464	0.9652	0.9416	0.9442	0.0085
LOC646688	0.7002	1.1568	1.1998	0.7201	0.9442	0.1355
TPH2	0.9130	1.0060	0.9936	0.8643	0.9442	0.0337
KCNE4	0.9935	0.9908	0.9607	0.8319	0.9442	0.0382
TDH	0.8881	1.0651	0.9424	0.8814	0.9442	0.0425
GDF10	0.8907	1.0818	0.9381	0.8664	0.9442	0.0482
LOC645995	0.9908	1.0871	0.8484	0.8507	0.9443	0.0581
LOC1001282	0.8359	1.0002	1.0503	0.8906	0.9443	0.0492
LOC645851	0.8583	1.0334	1.0755	0.8098	0.9443	0.0650
FOS	0.9120	0.9890	0.9487	0.9274	0.9443	0.0167
LOC646173	0.8649	1.0733	0.8862	0.9527	0.9443	0.0469
CRX	0.9905	0.9971	0.9483	0.8412	0.9443	0.0360
LOC442316	0.8536	1.0792	0.9399	0.9044	0.9443	0.0483
LOC729559	0.9270	1.0322	1.0227	0.7952	0.9443	0.0551
SIPA1L1	0.8273	1.1908	0.9624	0.7967	0.9443	0.0897
LOC652168	0.8970	0.8790	1.0421	0.9591	0.9443	0.0368
RIC8A	0.9427	1.2873	0.8890	0.6583	0.9443	0.1299
LOC1001295	0.8973	1.1112	0.9365	0.8324	0.9443	0.0596
LOC653541	0.9134	0.9812	0.9592	0.9235	0.9443	0.0157
LOC388931	0.8519	1.0790	0.8996	0.9468	0.9443	0.0489
DNAJC5G	0.8592	1.0274	0.9401	0.9508	0.9443	0.0344
KRTAP4-2	0.8813	1.0366	0.9314	0.9281	0.9443	0.0328
MIR370	0.8217	1.0351	1.0013	0.9193	0.9444	0.0476
LOC1001342	0.9342	1.0131	0.9209	0.9092	0.9444	0.0235
ZNF506	0.9579	0.9618	0.9012	0.9565	0.9444	0.0144
ZNF193	0.8919	1.0948	0.9506	0.8402	0.9444	0.0550
SNORD114-7	0.8427	1.0480	1.0229	0.8639	0.9444	0.0530
LOC1001282	0.8769	1.0640	0.9659	0.8707	0.9444	0.0454
HTR3C	0.9266	1.0433	0.9276	0.8801	0.9444	0.0348
EPB41L2	1.0170	0.9719	0.9834	0.8053	0.9444	0.0473
LOC1001324	0.8513	1.0900	0.9463	0.8900	0.9444	0.0523
SCN4A	0.8968	1.0668	0.9798	0.8343	0.9444	0.0505
LOC286238	0.9052	1.0043	0.9812	0.8869	0.9444	0.0286

JPH2	0.7985	1.1347	0.9950	0.8495	0.9444	0.0759
ERMP1	0.8558	1.1651	1.0685	0.6884	0.9444	0.1070
DDX3Y	0.8292	0.9242	1.1146	0.9098	0.9444	0.0604
LOC653157	0.9768	0.9832	0.9617	0.8560	0.9444	0.0298
LOC653978	0.9153	1.0402	1.0004	0.8220	0.9445	0.0484
LOC645241	0.8904	0.9899	0.9328	0.9648	0.9445	0.0215
EPS15	0.8838	1.3192	0.9374	0.6375	0.9445	0.1409
C20orf165	0.7939	1.0303	1.0141	0.9397	0.9445	0.0539
ZNF749	0.8324	1.1467	0.9246	0.8743	0.9445	0.0700
CYP11B2	0.9480	0.9698	0.9676	0.8926	0.9445	0.0180
LOC647337	1.0159	0.9516	1.0364	0.7741	0.9445	0.0596
LOC642809	0.9293	1.1092	0.9416	0.7980	0.9445	0.0638
TTC23L	0.9433	1.0236	0.9655	0.8456	0.9445	0.0371
LOC648001	0.9109	0.9244	1.0298	0.9129	0.9445	0.0286
LOC145820	0.9244	0.9793	0.9631	0.9112	0.9445	0.0160
LOC649891	1.0337	0.9201	0.8509	0.9733	0.9445	0.0389
SPSB2	0.9485	1.1846	0.8924	0.7525	0.9445	0.0900
LOC649773	0.8659	1.0473	1.0031	0.8617	0.9445	0.0475
GMCL1	0.7169	1.3472	0.9731	0.7409	0.9445	0.1461
FSTL3	0.9365	1.1739	0.9504	0.7173	0.9445	0.0932
EXOC3L	0.8578	1.0133	1.0412	0.8659	0.9445	0.0481
LOC648931	0.9610	1.0542	0.9091	0.8538	0.9445	0.0426
PLCD4	0.8781	1.1008	0.9213	0.8780	0.9446	0.0531
LOC388444	0.8677	1.0455	0.9877	0.8773	0.9446	0.0433
LOC729977	0.9614	0.9369	0.8842	0.9958	0.9446	0.0235
LOC646804	0.8015	1.0440	0.9790	0.9539	0.9446	0.0513
GCNT6	0.9420	1.0063	1.0083	0.8218	0.9446	0.0437
FOXO4	0.9779	1.1578	0.9335	0.7091	0.9446	0.0923
SLC22A4	0.9006	1.0013	0.9764	0.9001	0.9446	0.0261
LOC1001297	0.8199	1.1756	0.8707	0.9122	0.9446	0.0793
ANKRD17	0.7827	1.2335	0.9735	0.7887	0.9446	0.1060
LOC652761	0.8675	1.1069	0.9361	0.8680	0.9446	0.0564
LOC644981	0.9755	1.0537	0.9095	0.8398	0.9446	0.0457
GLRA2	0.9582	0.9821	0.9401	0.8981	0.9446	0.0177
LOC730461	0.8876	1.0321	1.0128	0.8461	0.9447	0.0459
MIR612	0.8961	0.9913	0.9934	0.8978	0.9447	0.0275
LOC1001325	0.6741	1.2329	0.9872	0.8844	0.9447	0.1161
PDCD1	0.8921	1.0497	1.0527	0.7842	0.9447	0.0653
CCDC84	0.9117	1.0386	0.9599	0.8685	0.9447	0.0364
LOC645039	0.9558	1.0705	0.9310	0.8214	0.9447	0.0511
LOC1001326	0.9644	0.9401	0.9967	0.8775	0.9447	0.0252
FLJ30719	0.9141	1.0839	0.9135	0.8672	0.9447	0.0477
LOC401410	0.9379	0.9549	0.9628	0.9231	0.9447	0.0089
FMR1	0.9334	1.0316	0.9120	0.9017	0.9447	0.0297
KIAA0947	0.9006	1.0212	0.9898	0.8671	0.9447	0.0364
LOC644893	0.9129	0.9061	0.9838	0.9759	0.9447	0.0204

CD200R1L	0.8906	1.1454	0.9073	0.8356	0.9447	0.0686
TMEM145	0.8786	1.1435	0.9469	0.8098	0.9447	0.0719
C2orf65	0.9648	1.0685	0.9133	0.8322	0.9447	0.0495
LOC389174	0.8056	1.1029	0.9858	0.8847	0.9447	0.0643
LOC1001279	0.9419	0.9587	1.0082	0.8701	0.9447	0.0286
ZFP64	0.8271	1.1732	0.9486	0.8302	0.9448	0.0812
SCARNA22	0.7613	1.1558	1.0283	0.8337	0.9448	0.0902
LOC152118	0.9368	0.8964	1.0896	0.8562	0.9448	0.0510
C20orf197	0.8344	1.0840	0.9822	0.8784	0.9448	0.0558
TPM2	0.9198	0.9897	1.0017	0.8679	0.9448	0.0313
C10orf105	0.8863	1.0373	1.0386	0.8169	0.9448	0.0556
LOC651118	0.9058	1.0125	0.9892	0.8716	0.9448	0.0334
LOC729652	0.8314	1.0028	1.0009	0.9440	0.9448	0.0402
PAMR1	0.8344	1.1040	0.9596	0.8812	0.9448	0.0590
LOC653673	0.8859	1.1060	0.8745	0.9127	0.9448	0.0543
LOC645183	0.9527	1.0380	0.9361	0.8523	0.9448	0.0381
LTB4R	0.9396	1.0241	1.0111	0.8044	0.9448	0.0503
LOC730920	0.9712	1.1291	0.9364	0.7426	0.9448	0.0794
LOC342933	0.8758	1.0247	0.9226	0.9562	0.9448	0.0313
MIR1246	0.8881	0.9269	1.0774	0.8868	0.9448	0.0452
TMEM22	0.9110	1.0101	0.8996	0.9586	0.9448	0.0253
TIMM8B	0.9744	1.0024	1.0957	0.7068	0.9448	0.0835
LOC648708	0.8905	1.0638	0.9650	0.8601	0.9448	0.0454
EIF5A2	0.9418	1.0077	0.9750	0.8548	0.9448	0.0329
LOC342931	0.7956	1.0009	1.0772	0.9057	0.9448	0.0609
LOC650851	0.9462	1.0932	0.8715	0.8685	0.9448	0.0526
TNFAIP2	0.8376	1.0314	1.0207	0.8897	0.9448	0.0481
TXNDC9	0.9631	1.0574	1.0946	0.6644	0.9449	0.0975
LOC1001284	0.8852	1.0108	0.9512	0.9322	0.9449	0.0260
LOC440368	0.9821	0.8772	1.0071	0.9130	0.9449	0.0301
C13orf16	0.7423	1.1438	0.9790	0.9144	0.9449	0.0830
RABGEF1	0.9252	1.1202	0.9265	0.8075	0.9449	0.0648
LOC652264	0.9368	1.0132	0.9318	0.8978	0.9449	0.0244
LOC651150	0.9222	0.9478	1.0123	0.8973	0.9449	0.0247
NEUROG1	0.8132	1.1257	1.0001	0.8406	0.9449	0.0730
PLP2	0.9116	0.9902	0.9722	0.9056	0.9449	0.0213
LOC728929	0.8176	1.0390	1.0490	0.8740	0.9449	0.0584
GNG8	0.8561	1.0899	0.9780	0.8556	0.9449	0.0563
C9orf66	0.8241	1.0801	0.9011	0.9743	0.9449	0.0545
LOC550643	0.8431	1.1839	0.9934	0.7593	0.9449	0.0932
LLPH	0.6845	1.4460	0.9345	0.7147	0.9449	0.1761
LOC649486	0.8042	0.9646	1.0850	0.9258	0.9449	0.0579
FAM90A14	0.7921	1.0612	0.9855	0.9409	0.9449	0.0567
LOC440268	0.8890	1.0014	1.0176	0.8717	0.9449	0.0376
ELP2	0.9076	1.1461	1.0017	0.7245	0.9449	0.0884
LOC647630	0.8583	1.0992	0.9256	0.8967	0.9450	0.0532

ENAM	0.8560	1.1024	0.9452	0.8762	0.9450	0.0559
C6orf167	0.8715	1.0698	1.0041	0.8344	0.9450	0.0553
LOC391269	0.8801	1.0571	0.9716	0.8711	0.9450	0.0437
DDX12	0.9199	1.0134	0.9495	0.8972	0.9450	0.0252
CHID1	0.8384	0.9867	0.9755	0.9793	0.9450	0.0356
PANX3	0.8746	1.0321	0.9436	0.9297	0.9450	0.0326
PMS1	0.9780	1.0466	0.8402	0.9152	0.9450	0.0440
MIR1245	0.8867	0.9780	0.9912	0.9242	0.9450	0.0242
MSI1	0.9365	1.0151	0.9601	0.8684	0.9450	0.0304
LOC728780	0.8739	1.0972	1.0009	0.8082	0.9450	0.0646
TRIML1	0.7611	1.1363	0.9671	0.9156	0.9450	0.0773
ALKBH6	0.7675	1.2323	1.0216	0.7589	0.9450	0.1135
LOC648398	0.9907	0.9617	0.9633	0.8646	0.9450	0.0276
MIR576	0.8522	1.0154	0.9724	0.9402	0.9451	0.0346
MIR1288	1.0013	0.8956	0.9988	0.8845	0.9451	0.0318
CHTF8	0.8660	1.1867	0.9058	0.8217	0.9451	0.0824
ARL3	0.9290	1.1320	0.9477	0.7715	0.9451	0.0738
G6PD	0.9703	1.1856	0.8788	0.7456	0.9451	0.0925
KIAA1804	0.8777	1.1101	0.9145	0.8781	0.9451	0.0557
LOC654350	0.8219	1.1572	0.9545	0.8468	0.9451	0.0763
MAGEB16	1.0240	0.9196	0.9565	0.8804	0.9451	0.0305
LOC286467	0.8474	1.0832	0.9723	0.8776	0.9451	0.0532
FHAD1	0.8559	1.0706	1.0080	0.8460	0.9451	0.0559
LOC1001304	0.8688	0.9612	1.0381	0.9124	0.9451	0.0363
LOC729277	0.8158	1.0150	1.0541	0.8957	0.9451	0.0547
LOC644276	0.7556	1.0252	1.1408	0.8589	0.9451	0.0857
LOC652554	0.8216	0.9781	0.9865	0.9943	0.9451	0.0413
DOCK2	0.8301	1.0786	1.1335	0.7384	0.9451	0.0954
LOC474170	0.8826	1.1168	0.8978	0.8834	0.9452	0.0573
LOC654230	0.9070	0.8782	0.9732	1.0223	0.9452	0.0325
KRT18P50	0.8592	0.9599	1.0310	0.9307	0.9452	0.0356
LOC127602	0.9185	1.0447	0.9549	0.8628	0.9452	0.0382
LOC1001328	0.8904	0.9933	1.0393	0.8579	0.9452	0.0426
SBF1	0.8361	1.2971	0.9400	0.7077	0.9452	0.1265
LOC1001344	0.8643	1.0118	0.9698	0.9350	0.9452	0.0312
LOC1001329	0.8895	1.0387	0.9706	0.8822	0.9452	0.0370
LOC644683	0.9701	0.9107	1.0485	0.8517	0.9453	0.0420
LOC392559	0.9100	0.9838	1.0350	0.8523	0.9453	0.0402
MAP3K3	0.8318	1.0994	0.9855	0.8644	0.9453	0.0611
CROP	0.8653	1.0131	1.0679	0.8348	0.9453	0.0565
LOC729562	0.8962	1.0378	0.9986	0.8485	0.9453	0.0439
DDX4	0.8907	1.0175	1.0432	0.8296	0.9453	0.0510
CEP170	0.9240	1.0702	0.9347	0.8522	0.9453	0.0455
MIR876	0.9820	0.9091	1.0432	0.8468	0.9453	0.0428
LOC648003	0.8560	0.9090	1.0049	1.0111	0.9453	0.0378
LOC647009	0.8466	1.0179	1.1072	0.8096	0.9453	0.0705

BCR	0.8211	1.1766	0.9464	0.8371	0.9453	0.0820
MIR421	0.8759	1.0531	0.9917	0.8605	0.9453	0.0463
ZYG11B	0.7207	1.3238	0.9325	0.8041	0.9453	0.1335
LOC389369	0.9384	0.9873	0.9930	0.8624	0.9453	0.0302
LOC643559	0.9095	1.0305	0.9192	0.9220	0.9453	0.0285
NEK11	0.8839	1.0622	1.0215	0.8136	0.9453	0.0582
MATR3	0.8811	1.2878	0.9220	0.6904	0.9453	0.1248
C18orf1	0.8483	1.0010	1.0143	0.9176	0.9453	0.0388
LOC653857	0.8588	1.0047	1.0680	0.8498	0.9453	0.0542
LOC644816	0.7752	1.2333	0.9748	0.7980	0.9453	0.1059
C3orf33	0.8681	1.0856	0.9493	0.8784	0.9453	0.0501
NEUROG3	0.9556	0.8407	1.0286	0.9564	0.9453	0.0389
LOC1001282	0.9299	1.0346	0.9658	0.8510	0.9453	0.0382
FIGNL2	0.8193	1.1346	1.0343	0.7931	0.9453	0.0831
MCF2	0.8447	1.0845	0.9280	0.9242	0.9453	0.0502
FLJ41200	0.8975	1.0865	1.0323	0.7650	0.9453	0.0721
GATA1	0.8636	1.1177	0.9408	0.8593	0.9453	0.0604
PYDC1	1.0309	0.9887	0.9902	0.7717	0.9454	0.0587
TMEM204	0.9506	0.9763	0.9413	0.9133	0.9454	0.0130
LOC649133	0.9284	1.0552	1.0117	0.7861	0.9454	0.0592
VSTM2B	0.9137	1.0936	0.9410	0.8332	0.9454	0.0545
C1orf152	0.9148	0.9444	1.0287	0.8937	0.9454	0.0296
LOC643566	0.8931	0.9995	0.9650	0.9240	0.9454	0.0233
PROL1	0.9521	1.0990	0.8951	0.8355	0.9454	0.0565
CHD5	0.9208	1.0464	0.9428	0.8718	0.9454	0.0368
CCDC59	0.7755	1.1221	1.1344	0.7496	0.9454	0.1057
GLTPD2	0.9377	1.0292	0.9584	0.8563	0.9454	0.0356
OTOP1	0.9647	1.0813	0.8854	0.8503	0.9454	0.0512
RAB33A	0.9215	0.9431	1.0437	0.8734	0.9454	0.0358
PSPHL	0.9396	0.9262	1.0278	0.8881	0.9454	0.0296
CNPY1	0.8741	1.0806	0.9902	0.8370	0.9455	0.0556
LOC643495	0.8632	1.0318	0.9945	0.8923	0.9455	0.0403
LOC647876	0.8350	1.1749	0.9299	0.8420	0.9455	0.0795
LOC1001338	0.8597	1.0799	0.9921	0.8501	0.9455	0.0553
TYMP	0.9184	1.1469	0.8750	0.8417	0.9455	0.0690
IL13RA2	0.9836	1.0857	0.9841	0.7286	0.9455	0.0762
LOC643240	0.8652	1.0431	0.9989	0.8747	0.9455	0.0446
LOC648792	1.0510	0.9161	0.9167	0.8982	0.9455	0.0354
LOC646496	0.9832	0.8102	1.0456	0.9429	0.9455	0.0498
LOC730990	0.8397	0.9983	1.0667	0.8774	0.9455	0.0527
RHBDD3	0.8864	1.2269	0.9196	0.7492	0.9455	0.1008
LOC728297	0.9079	1.0195	1.0029	0.8517	0.9455	0.0398
FAM75A3	0.8789	0.9473	1.0380	0.9180	0.9455	0.0339
PADI6	0.9009	0.9787	1.0059	0.8967	0.9455	0.0276
FBLN5	0.9206	1.0256	0.9663	0.8697	0.9455	0.0332
DHDDS	0.9087	1.1254	0.8972	0.8509	0.9455	0.0612

PDXDC1	0.9354	1.2878	0.8989	0.6601	0.9456	0.1294
FLJ10803	0.8792	1.0437	1.0293	0.8300	0.9456	0.0535
LOC646853	0.9200	1.0292	0.9851	0.8480	0.9456	0.0395
LOC401630	0.8782	1.0494	0.9654	0.8893	0.9456	0.0397
PVT1	0.7589	1.2053	1.0652	0.7529	0.9456	0.1132
LOC729609	0.8902	0.9339	0.9697	0.9886	0.9456	0.0217
LOC727827	0.8295	1.0472	1.0150	0.8906	0.9456	0.0513
PRSS12	0.9034	1.0164	0.9890	0.8736	0.9456	0.0340
HAVCR2	0.9016	0.9788	0.9956	0.9065	0.9456	0.0243
LOC648342	0.7768	1.0843	1.0212	0.9001	0.9456	0.0680
P11	0.9894	1.0151	0.9787	0.7993	0.9456	0.0494
LOC343927	0.8168	1.1170	0.9632	0.8855	0.9456	0.0645
GML	0.8346	1.0415	0.9889	0.9176	0.9456	0.0449
LOC1001328	0.8781	1.0431	1.0028	0.8586	0.9456	0.0456
LOC652750	0.9164	1.0057	1.0616	0.7990	0.9456	0.0573
LOC1001322	0.8842	0.9731	1.0217	0.9037	0.9457	0.0317
ZNF571	0.8867	1.1478	0.8900	0.8582	0.9457	0.0678
LOC1001285	0.9360	1.0627	0.9882	0.7959	0.9457	0.0563
LOC731787	0.9790	0.9645	1.0242	0.8151	0.9457	0.0454
LOC391025	0.8934	1.0771	0.9644	0.8479	0.9457	0.0499
LOC644361	0.9158	1.0809	0.9574	0.8287	0.9457	0.0525
LOC644530	0.9615	1.0136	0.9985	0.8092	0.9457	0.0468
RASL10A	0.9740	0.9189	0.9640	0.9259	0.9457	0.0137
GPX6	0.8258	1.0608	0.9812	0.9151	0.9457	0.0498
C21orf30	0.5200	1.3362	0.9703	0.9564	0.9457	0.1669
MOBK1B	0.8894	1.2789	0.8468	0.7679	0.9457	0.1139
DFNB31	0.9184	0.9951	1.0676	0.8019	0.9457	0.0568
PBX4	0.9057	0.9707	1.0339	0.8727	0.9457	0.0357
LOC728504	0.8294	1.0494	0.9303	0.9739	0.9458	0.0459
LOC652707	0.8719	1.1092	0.9059	0.8961	0.9458	0.0549
KCNJ1	0.8547	1.0208	1.0210	0.8865	0.9458	0.0439
HIP1R	0.8973	1.0661	0.9460	0.8737	0.9458	0.0428
LOC647444	0.8758	1.0989	1.0022	0.8062	0.9458	0.0652
LOC644727	0.9545	1.0462	0.9323	0.8501	0.9458	0.0403
EDN3	0.9005	0.9894	1.0125	0.8807	0.9458	0.0324
TBC1D15	0.7410	1.3217	1.0002	0.7203	0.9458	0.1405
ZNF521	0.9526	0.9967	0.9985	0.8353	0.9458	0.0383
LOC732058	0.9321	0.9846	1.0124	0.8542	0.9458	0.0348
LOC643454	0.8564	1.0419	1.0143	0.8706	0.9458	0.0479
HFE	0.9217	1.0518	0.9430	0.8668	0.9458	0.0388
LOC642287	0.8574	1.0325	0.9486	0.9447	0.9458	0.0358
RAP1A	0.8788	1.0871	1.0427	0.7748	0.9458	0.0725
C6orf107	1.0251	0.9828	0.8947	0.8808	0.9458	0.0348
LOC1001324	0.8447	1.0545	0.9523	0.9319	0.9458	0.0431
CUL7	0.7731	1.1121	1.1212	0.7769	0.9458	0.0987
LOC338799	0.9677	1.0029	0.8818	0.9311	0.9459	0.0259

LRRC61	0.8484	1.0419	1.0227	0.8705	0.9459	0.0503
LOC389672	0.7149	1.4027	1.0365	0.6293	0.9459	0.1757
STX5	0.8595	1.2510	0.9401	0.7329	0.9459	0.1103
C21orf77	0.9506	0.9872	0.9244	0.9213	0.9459	0.0153
C8orf86	0.9225	1.0666	0.9104	0.8841	0.9459	0.0410
CEP120	0.8831	1.0595	1.0253	0.8156	0.9459	0.0578
TMEM37	0.9283	0.9501	1.0140	0.8910	0.9459	0.0258
SPANXD	0.7884	1.1253	0.9742	0.8956	0.9459	0.0709
LOC652046	0.8907	1.1135	1.0179	0.7614	0.9459	0.0766
RP1	0.9527	1.0371	0.9293	0.8645	0.9459	0.0357
LOC1001328	0.9168	1.0671	0.9769	0.8228	0.9459	0.0514
ELOVL1	0.9564	1.2869	0.8490	0.6913	0.9459	0.1260
LOC641714	0.9068	1.0220	1.0255	0.8293	0.9459	0.0477
IKZF5	0.8308	1.1566	1.0380	0.7582	0.9459	0.0919
LOC390594	0.9426	1.0404	0.9371	0.8636	0.9459	0.0363
LOC649620	0.8473	1.0297	1.0522	0.8546	0.9459	0.0551
LOC1001311	0.8100	1.0093	1.0888	0.8756	0.9459	0.0631
CRSP6	0.9212	1.0752	0.8686	0.9187	0.9459	0.0448
LOC401093	0.9125	0.9748	0.9547	0.9419	0.9460	0.0130
ERCC-00086	0.8801	1.0958	0.9533	0.8546	0.9460	0.0541
PHACTR1	0.9017	1.0361	0.9845	0.8616	0.9460	0.0395
PRG4	0.9055	1.0758	0.9596	0.8430	0.9460	0.0494
LOC1001284	0.9637	0.9943	0.9784	0.8476	0.9460	0.0334
LOC729120	0.6263	1.4058	1.0368	0.7151	0.9460	0.1768
OR5T1	0.9069	1.0372	0.9117	0.9281	0.9460	0.0307
LOC1001309	0.8950	1.0587	0.9730	0.8573	0.9460	0.0446
CPB2	0.9129	1.0370	0.9839	0.8502	0.9460	0.0408
PMCHL2	0.8588	0.9978	0.9761	0.9512	0.9460	0.0306
MMS19	0.7602	1.2940	0.9796	0.7501	0.9460	0.1275
FCGR3B	0.7990	1.1013	1.0254	0.8583	0.9460	0.0705
DNAJB4	0.9454	0.9685	1.0385	0.8316	0.9460	0.0430
MGC5457	0.8429	1.0596	1.0711	0.8106	0.9460	0.0692
OR1K1	0.8825	0.9559	1.0445	0.9011	0.9460	0.0363
LOC651430	0.7918	1.0834	1.0087	0.9003	0.9460	0.0637
EMX1	0.8306	1.0259	1.0320	0.8956	0.9460	0.0497
EPM2A	0.9082	1.0789	0.9310	0.8662	0.9460	0.0463
NAPSA	0.9388	0.9422	0.9251	0.9781	0.9460	0.0113
FKSG49	1.0098	0.9697	0.9764	0.8283	0.9461	0.0402
LOC654164	0.9504	1.0098	1.0254	0.7986	0.9461	0.0517
PLEKHO1	0.7960	0.9822	1.0542	0.9518	0.9461	0.0544
POU3F4	0.8163	1.0370	1.0148	0.9161	0.9461	0.0506
HYAL2	0.9323	1.2102	0.8098	0.8320	0.9461	0.0920
ZNF638	1.0576	1.0591	0.8619	0.8057	0.9461	0.0658
LOC653513	0.9425	1.0339	0.9106	0.8973	0.9461	0.0308
LOC645202	0.8554	1.1094	0.9876	0.8320	0.9461	0.0643
DNAH1	0.8620	1.0690	0.9783	0.8750	0.9461	0.0485

LOC731881	0.9267	0.9780	0.9748	0.9049	0.9461	0.0181
IPW	0.9837	1.0755	0.9641	0.7611	0.9461	0.0663
PTPN9	0.8884	1.1383	0.9552	0.8026	0.9461	0.0713
FLJ43826	0.9222	1.0190	0.9400	0.9033	0.9461	0.0254
FLJ44477	0.9816	1.0517	0.9036	0.8476	0.9461	0.0446
FBXW12	0.9609	1.0660	0.8975	0.8601	0.9461	0.0450
PSCD4	0.9426	1.0493	0.8633	0.9294	0.9461	0.0385
ALDH1A2	0.8745	1.0550	0.9390	0.9161	0.9461	0.0387
TSG101	0.8897	1.2409	0.9658	0.6882	0.9461	0.1144
LOC390712	0.7804	0.9418	1.1479	0.9145	0.9461	0.0759
STRA6	0.8809	0.9948	1.0089	0.8999	0.9461	0.0325
YDD19	0.8895	0.8969	1.0782	0.9199	0.9461	0.0445
LOC1001344	0.8487	1.1193	0.9971	0.8195	0.9461	0.0696
LOC647855	0.8858	1.0029	1.0078	0.8880	0.9462	0.0342
LOC1001329	0.8798	1.0071	0.9767	0.9209	0.9462	0.0284
PACS1	0.9566	1.1453	0.8629	0.8198	0.9462	0.0722
TXNRD2	0.8666	1.2144	0.9550	0.7486	0.9462	0.0989
ZNF331	0.7885	1.1358	1.0898	0.7706	0.9462	0.0967
LOC729353	0.9135	0.9773	1.0096	0.8844	0.9462	0.0287
OR6C6	0.8592	1.0515	1.0395	0.8347	0.9462	0.0576
HDAC8	0.9202	1.1794	0.9198	0.7654	0.9462	0.0859
LOC1001310	0.8526	1.0919	0.9338	0.9065	0.9462	0.0514
ADAM2	0.7820	1.1803	0.9158	0.9066	0.9462	0.0838
PDE8A	0.9217	1.0744	0.9021	0.8867	0.9462	0.0433
CPT1C	0.8871	1.0460	0.9718	0.8800	0.9462	0.0393
LOC641741	0.8880	0.9987	0.9866	0.9116	0.9462	0.0274
LOC643836	0.9128	1.0621	0.9464	0.8637	0.9462	0.0422
LOC158301	0.9140	1.0606	0.9332	0.8772	0.9463	0.0398
LEFTY2	0.8867	1.1153	0.8711	0.9120	0.9463	0.0570
C19orf34	0.9676	1.0632	0.8617	0.8925	0.9463	0.0449
PRRT2	0.9838	0.9745	1.0548	0.7720	0.9463	0.0608
ZNF430	0.7527	1.2670	1.0987	0.6667	0.9463	0.1419
CXCL2	0.8914	1.0244	0.9549	0.9144	0.9463	0.0292
LTC4S	0.9484	0.9587	0.9311	0.9470	0.9463	0.0057
MRO	0.9397	1.0048	0.9214	0.9192	0.9463	0.0200
LOC650006	0.8665	1.0889	0.9148	0.9149	0.9463	0.0489
FOXK2	0.9542	0.9880	1.0306	0.8123	0.9463	0.0473
BAP1	1.0984	1.0659	0.8704	0.7504	0.9463	0.0824
ICF45	0.8234	1.1662	0.8752	0.9205	0.9463	0.0759
LOC648170	0.9642	1.0243	0.9615	0.8352	0.9463	0.0398
LOC730200	0.8561	1.0434	0.9630	0.9228	0.9463	0.0391
HLA-DMB	0.9231	1.1760	0.8755	0.8107	0.9463	0.0799
C8orf42	0.8883	1.1000	0.9477	0.8493	0.9463	0.0551
ZDHHC20	0.9953	1.0104	0.8836	0.8961	0.9463	0.0329
SRMS	0.9393	0.9594	0.9975	0.8892	0.9463	0.0225
LOC442181	0.8703	0.9716	1.0875	0.8560	0.9464	0.0536

LOC729051	0.9463	1.0033	0.9631	0.8728	0.9464	0.0273
PLA2G12B	0.9651	1.0491	0.8626	0.9087	0.9464	0.0401
SH3BGRL2	0.9816	1.1869	0.9286	0.6884	0.9464	0.1025
NUDT8	0.9971	0.9692	1.0161	0.8031	0.9464	0.0487
KIF1C	0.9420	1.0932	0.9615	0.7889	0.9464	0.0623
LOC644302	0.9011	1.0983	0.8612	0.9249	0.9464	0.0523
LOC1001279	0.9371	1.0578	0.8995	0.8912	0.9464	0.0385
LOC390507	0.8683	1.0971	1.0163	0.8039	0.9464	0.0671
HEPHL1	0.8490	1.1669	0.9930	0.7767	0.9464	0.0862
N-PAC	0.8938	1.2415	0.8922	0.7582	0.9464	0.1034
FBXL8	1.0261	0.9561	0.9491	0.8544	0.9464	0.0353
NXF2	0.8790	1.0375	0.9627	0.9065	0.9464	0.0350
CD1A	0.8965	0.9245	1.1776	0.7872	0.9464	0.0825
LOC649951	0.8078	0.9977	1.0106	0.9697	0.9464	0.0470
LOC401876	0.8548	1.0736	0.9792	0.8781	0.9464	0.0503
GCNT4	0.9109	1.0265	0.9208	0.9276	0.9465	0.0269
MOAP1	0.9207	1.2228	0.8556	0.7868	0.9465	0.0961
LOC283588	0.9408	1.0377	0.8996	0.9078	0.9465	0.0317
SNORD37	0.8650	1.0173	1.0514	0.8522	0.9465	0.0513
LOC342934	0.8695	1.0666	0.9672	0.8826	0.9465	0.0455
LOC392145	0.8391	1.1435	0.9220	0.8812	0.9465	0.0678
LHFPL4	0.9190	1.1186	0.9624	0.7858	0.9465	0.0686
LOC388955	0.9341	0.9875	1.0041	0.8602	0.9465	0.0324
LOC1001319	0.9015	1.0027	1.0195	0.8623	0.9465	0.0383
C12orf37	0.8959	1.1751	0.9087	0.8062	0.9465	0.0795
HDHD3	0.8376	1.1797	0.9670	0.8017	0.9465	0.0855
ZNF292	0.9222	1.0949	0.9140	0.8548	0.9465	0.0517
LOC139431	0.8630	1.0527	0.9898	0.8805	0.9465	0.0451
LOC650278	1.0449	0.9533	0.9062	0.8816	0.9465	0.0360
LOC1001309	0.9145	1.1623	0.8725	0.8367	0.9465	0.0737
LOC647488	0.9305	0.9346	0.9428	0.9781	0.9465	0.0109
INSC	0.9739	1.0068	0.9369	0.8683	0.9465	0.0297
KPRP	0.9355	0.9837	1.0220	0.8449	0.9465	0.0382
PDCD11	0.8814	1.0679	0.8969	0.9398	0.9465	0.0423
C8orf81	0.7887	1.0699	1.0292	0.8984	0.9465	0.0641
FLJ42393	1.0316	1.0377	0.8973	0.8196	0.9466	0.0533
LOC644242	0.8635	1.0685	0.9696	0.8847	0.9466	0.0466
LOC645043	0.8748	1.0266	0.9934	0.8915	0.9466	0.0374
ZNF680	0.8910	1.0793	1.0929	0.7231	0.9466	0.0876
PCTP	0.9600	0.9847	1.1408	0.7009	0.9466	0.0912
LOXL1	0.9185	1.0507	0.9379	0.8793	0.9466	0.0368
LOC652515	0.7884	1.0515	0.9515	0.9950	0.9466	0.0566
NYNRIN	0.9056	1.0605	0.9023	0.9181	0.9466	0.0381
LOC643055	0.8765	1.0276	0.9626	0.9198	0.9466	0.0322
C8ORFK32	0.9467	0.9647	0.9667	0.9084	0.9466	0.0135
LOC646784	0.9322	1.0195	1.0771	0.7577	0.9466	0.0697

DDX5	0.8773	1.1530	0.9516	0.8047	0.9466	0.0750
ANXA2	0.8125	1.0357	1.0126	0.9257	0.9466	0.0506
PRKY	0.8701	1.1274	0.9123	0.8768	0.9467	0.0610
LOC649025	0.8253	1.0394	0.9975	0.9244	0.9467	0.0469
LOC1001328	0.9647	1.1961	0.8842	0.7416	0.9467	0.0951
BEST1	0.9451	1.0523	0.9407	0.8487	0.9467	0.0416
LOC1001286	0.9028	1.0459	0.9643	0.8737	0.9467	0.0381
BIK	0.9591	1.0015	1.0433	0.7829	0.9467	0.0572
LOC642197	0.7282	1.1840	1.1423	0.7321	0.9467	0.1253
PCK1	0.9073	1.0410	0.9883	0.8501	0.9467	0.0423
PDIA2	0.9023	1.0658	0.9003	0.9183	0.9467	0.0399
LOC647726	0.9933	1.0326	0.9206	0.8402	0.9467	0.0424
LOC645217	0.9239	1.0499	0.9402	0.8728	0.9467	0.0373
TCEAL1	0.8614	1.2445	1.0125	0.6684	0.9467	0.1217
GAB1	0.8975	1.0760	0.8944	0.9189	0.9467	0.0435
LOC442261	0.9022	0.9221	1.0273	0.9352	0.9467	0.0277
C6orf174	0.9894	0.9515	0.9557	0.8901	0.9467	0.0207
LOC440337	0.8978	1.1131	0.9235	0.8524	0.9467	0.0574
C20orf179	0.9844	1.0194	0.9005	0.8826	0.9467	0.0328
OR2A4	0.8758	1.0539	1.0455	0.8116	0.9467	0.0609
FLJ20581	0.8121	1.0018	1.0128	0.9602	0.9467	0.0463
LOC650977	0.8726	1.0486	1.0202	0.8455	0.9467	0.0512
OR5R1	0.8389	1.1010	0.9532	0.8939	0.9467	0.0565
SLC45A1	0.8854	1.0328	1.0187	0.8501	0.9467	0.0463
DSCR5	0.7938	1.0247	1.0016	0.9669	0.9467	0.0524
MEF2C	0.9646	1.0186	0.9869	0.8169	0.9468	0.0447
LOC641896	0.9289	1.0509	1.0229	0.7844	0.9468	0.0601
ECE1	0.9390	1.1014	0.8916	0.8550	0.9468	0.0543
APIP	0.6831	1.3571	0.9096	0.8374	0.9468	0.1447
C17orf63	0.7165	1.2364	1.0470	0.7873	0.9468	0.1199
LOC644359	0.9477	1.0348	0.9497	0.8551	0.9468	0.0367
GARNL3	0.8932	1.1402	0.9234	0.8305	0.9468	0.0673
GPR3	0.8666	1.2117	0.7965	0.9125	0.9468	0.0914
LOC729558	0.9063	1.0752	0.9456	0.8602	0.9468	0.0462
LOC643038	1.0236	1.0021	0.8868	0.8748	0.9468	0.0384
FAM27A	0.8085	1.1348	0.9658	0.8782	0.9468	0.0704
MYB	0.8473	1.0248	1.0163	0.8990	0.9468	0.0439
NUDT12	0.9713	1.0195	1.0539	0.7427	0.9468	0.0701
LOC1001330	0.9576	0.9751	1.0077	0.8469	0.9468	0.0349
LOC729137	0.8109	1.1897	0.8668	0.9200	0.9468	0.0839
GPR120	0.8063	0.9875	1.0832	0.9105	0.9469	0.0587
UCP2	0.9473	1.0653	0.8973	0.8776	0.9469	0.0421
CDH20	0.7654	1.1741	0.9203	0.9276	0.9469	0.0845
PPP4R2	0.9629	0.9936	0.9378	0.8931	0.9469	0.0212
LOC1001323	0.8655	1.0812	0.9793	0.8615	0.9469	0.0524
PDZD4	0.9510	1.0231	0.9868	0.8266	0.9469	0.0427

LOC652708	0.9708	0.9057	1.0677	0.8434	0.9469	0.0479
IKBIP	0.8719	1.0991	0.9765	0.8401	0.9469	0.0585
AGRP	0.9590	0.9780	0.9898	0.8608	0.9469	0.0294
NCOA2	0.7731	1.1892	0.9885	0.8367	0.9469	0.0926
PFKL	0.9930	1.1395	0.8974	0.7576	0.9469	0.0804
LAPTM5	0.8081	0.9864	1.0447	0.9483	0.9469	0.0503
LOC1001318	0.8600	1.0842	0.9596	0.8837	0.9469	0.0505
KCNB1	0.9350	1.0454	0.9154	0.8919	0.9469	0.0340
MIR633	0.8951	0.9814	0.9836	0.9275	0.9469	0.0216
TRAPPC10	0.8281	1.0055	0.9876	0.9665	0.9469	0.0404
VEGFA	0.8659	1.0880	0.9575	0.8763	0.9469	0.0513
LOC647154	0.8566	1.0092	1.0055	0.9165	0.9469	0.0370
ADAD2	0.7442	1.0650	1.0397	0.9388	0.9469	0.0729
LOC645018	0.7296	1.2725	1.1268	0.6589	0.9469	0.1496
PDCL	0.8477	1.2025	0.9629	0.7747	0.9470	0.0936
LOC1001301	0.8897	1.0237	1.0861	0.7883	0.9470	0.0669
WDR59	0.8975	1.1741	0.9552	0.7611	0.9470	0.0859
LOC645685	0.9107	1.0633	0.9854	0.8285	0.9470	0.0503
CBLB	0.8448	1.2972	0.9059	0.7400	0.9470	0.1217
IGFL4	0.8981	0.9035	1.1217	0.8646	0.9470	0.0589
ZNF503	0.7883	1.1354	0.9429	0.9213	0.9470	0.0715
HAMP	0.9322	1.0912	0.8856	0.8789	0.9470	0.0495
CRB3	0.8696	1.2836	0.9085	0.7262	0.9470	0.1189
LOC199800	0.7962	1.1883	0.9333	0.8701	0.9470	0.0852
NUP214	0.8423	1.4460	0.8370	0.6627	0.9470	0.1715
C9orf14	0.7698	1.0351	1.0838	0.8993	0.9470	0.0708
LOC391081	0.8609	1.0622	0.9257	0.9392	0.9470	0.0420
NIPAL4	1.0039	0.8918	1.0075	0.8848	0.9470	0.0339
LOC1001303	0.9528	1.1261	0.8843	0.8249	0.9470	0.0651
LOC653707	0.9673	0.9468	0.9944	0.8795	0.9470	0.0245
C1orf63	0.8704	1.0680	0.9600	0.8896	0.9470	0.0447
LOC158381	0.8856	1.1349	0.8490	0.9187	0.9470	0.0642
ITGA2B	0.8006	1.0992	1.0250	0.8633	0.9470	0.0693
NQO1	0.8708	1.3625	0.8625	0.6923	0.9470	0.1445
RRAGA	0.8307	1.2036	0.9780	0.7758	0.9470	0.0956
LCN1L1	0.7945	1.0593	1.0364	0.8980	0.9470	0.0621
CCL3L1	0.9298	0.9664	0.9593	0.9327	0.9470	0.0093
UBR2	0.8540	1.1463	1.0135	0.7744	0.9470	0.0830
LOC729523	1.0234	1.0183	0.9323	0.8143	0.9471	0.0489
PILRB	0.8472	1.1256	0.9350	0.8806	0.9471	0.0622
MEGF10	0.9203	1.0477	0.9206	0.8997	0.9471	0.0339
RUNX1	0.8840	1.0730	0.9746	0.8568	0.9471	0.0490
LOC652821	0.8643	1.1239	0.9627	0.8375	0.9471	0.0648
OR4P4	0.8649	1.0274	1.0318	0.8643	0.9471	0.0476
LOC648064	0.8609	1.1257	1.0017	0.8000	0.9471	0.0730
LOC652762	0.8403	0.9690	1.0264	0.9527	0.9471	0.0390

MIR552	0.9417	1.0331	0.9463	0.8674	0.9471	0.0339
KDELR1	0.8785	1.2325	0.9275	0.7499	0.9471	0.1022
OR2B6	0.8153	1.0346	1.0196	0.9189	0.9471	0.0509
PCDHGB3	0.8473	1.0797	0.9908	0.8707	0.9471	0.0542
LOC728735	0.9593	0.9525	0.9458	0.9309	0.9471	0.0061
CYP2E1	0.8836	1.1846	0.9183	0.8020	0.9471	0.0828
GABRR3	0.8706	1.0203	0.9805	0.9171	0.9471	0.0332
FUNDC1	0.9716	1.0782	0.9377	0.8011	0.9471	0.0571
MIR582	0.9077	1.0289	0.9255	0.9264	0.9471	0.0276
TBC1D21	0.8947	1.0786	0.9305	0.8848	0.9471	0.0449
LOC731186	0.9107	1.0129	0.9183	0.9467	0.9472	0.0232
LOC728254	0.8337	1.0519	1.0044	0.8986	0.9472	0.0496
LOC641996	0.8989	1.0772	0.9359	0.8767	0.9472	0.0450
LOC1001335	1.1144	1.0670	0.8670	0.7404	0.9472	0.0873
TUBB8	0.9268	1.0390	0.9472	0.8756	0.9472	0.0341
HOXD10	0.9927	0.9956	0.9229	0.8776	0.9472	0.0286
LOC1001294	0.8111	1.0982	0.9568	0.9227	0.9472	0.0592
ANGPTL5	0.8756	1.0209	0.9729	0.9194	0.9472	0.0316
LOC1001344	0.9551	1.0931	0.8563	0.8844	0.9472	0.0529
C17orf55	0.9359	1.1324	0.8261	0.8944	0.9472	0.0658
FLJ34870	0.9013	0.9995	1.0129	0.8753	0.9472	0.0345
MGC27382	0.8340	1.0299	1.1055	0.8196	0.9472	0.0713
LOC645365	0.8326	1.0862	0.9552	0.9150	0.9473	0.0529
UPB1	0.8119	1.1307	0.9199	0.9265	0.9473	0.0665
LOC647122	0.8347	1.0692	1.0140	0.8711	0.9473	0.0561
BAALC	0.9348	1.0664	0.9671	0.8208	0.9473	0.0506
MYST1	0.8268	1.2464	0.8984	0.8174	0.9473	0.1013
SEC24C	0.8658	1.2278	0.8860	0.8095	0.9473	0.0949
NR4A2	0.9472	1.0161	0.9724	0.8534	0.9473	0.0344
SEC14L1	0.8763	1.0702	1.0039	0.8388	0.9473	0.0541
LOC1001329	0.9112	1.1241	0.9471	0.8067	0.9473	0.0660
LRSAM1	0.8327	1.2034	0.9975	0.7555	0.9473	0.0992
SCARNA7	0.8828	1.1042	0.9471	0.8551	0.9473	0.0557
SLITRK6	0.8337	0.9739	1.0688	0.9128	0.9473	0.0496
LOC728275	0.7339	1.1751	1.0155	0.8647	0.9473	0.0953
LOC120364	0.9392	1.0327	0.9185	0.8989	0.9473	0.0296
LOC652792	0.9926	1.0567	0.9096	0.8305	0.9473	0.0492
SLC4A9	0.8840	1.0267	0.9831	0.8956	0.9473	0.0345
CHAD	0.8741	1.0268	1.0604	0.8281	0.9473	0.0568
LOC652800	0.8003	1.2994	0.8396	0.8501	0.9473	0.1178
FUT10	0.9034	1.0738	0.9696	0.8425	0.9474	0.0495
CLDN24	0.8290	1.0609	1.0235	0.8760	0.9474	0.0561
GPR112	0.9138	1.0729	0.9267	0.8761	0.9474	0.0432
LOC644200	0.9145	1.0880	0.9230	0.8640	0.9474	0.0486
NR6A1	0.9186	1.0715	0.9936	0.8057	0.9474	0.0566
NAT8B	0.9323	0.9802	0.9416	0.9353	0.9474	0.0111

RLBP1	0.8840	1.1102	0.9655	0.8298	0.9474	0.0610
ATF2	0.7525	1.2101	1.0293	0.7976	0.9474	0.1065
MT1L	0.9120	1.0635	0.9545	0.8595	0.9474	0.0433
FITM2	0.8779	1.0493	0.9961	0.8663	0.9474	0.0449
LOC642229	0.8855	0.9981	1.0316	0.8744	0.9474	0.0396
LOC643895	0.9338	1.0226	0.9043	0.9289	0.9474	0.0259
LOC1001347	0.9467	0.9842	0.9163	0.9424	0.9474	0.0140
LOC1001332	0.8309	1.0778	0.8897	0.9912	0.9474	0.0546
FLJ32682	0.8698	1.0609	0.9398	0.9191	0.9474	0.0406
AKAP12	0.8791	1.0217	1.0082	0.8806	0.9474	0.0391
SNX19	1.0121	1.1517	0.9385	0.6873	0.9474	0.0973
LOC653180	0.8805	1.0353	0.9439	0.9299	0.9474	0.0323
LOC732150	1.0292	0.9726	0.9760	0.8118	0.9474	0.0470
LOC653528	0.8768	1.0076	1.0401	0.8651	0.9474	0.0447
LOC391810	0.9678	0.9664	1.0072	0.8484	0.9474	0.0343
LOC1001330	0.9084	1.0612	0.9683	0.8517	0.9474	0.0448
LOC729486	0.9294	1.0337	1.0073	0.8193	0.9474	0.0481
LAMP3	0.8772	1.0686	1.0395	0.8044	0.9474	0.0636
NKAIN2	0.9121	1.0204	0.9733	0.8839	0.9474	0.0307
LOC643234	0.8854	1.0136	0.9308	0.9599	0.9474	0.0269
DPF1	0.9174	1.0367	0.9519	0.8837	0.9474	0.0328
CCKBR	0.8325	1.0981	0.9787	0.8804	0.9474	0.0587
MGC12965	0.8954	1.0356	0.9488	0.9099	0.9474	0.0315
LOC642776	0.9383	1.0461	0.9797	0.8256	0.9475	0.0463
SCEL	0.8937	1.0182	1.0361	0.8418	0.9475	0.0474
LOC730118	0.8180	1.0132	1.0563	0.9024	0.9475	0.0540
STXBP4	0.8537	1.1105	1.0006	0.8251	0.9475	0.0666
HSPC072	0.8439	1.0031	1.0000	0.9429	0.9475	0.0372
IL17F	0.8149	1.0258	1.0567	0.8926	0.9475	0.0568
TSPAN16	0.9080	1.0990	0.9399	0.8430	0.9475	0.0544
C6orf160	0.8730	1.1932	1.0956	0.6281	0.9475	0.1258
LOC646580	0.8366	1.0095	1.0034	0.9404	0.9475	0.0401
LOC650457	0.8977	1.1336	0.9816	0.7772	0.9475	0.0749
FLJ35848	0.8919	1.0568	1.0104	0.8309	0.9475	0.0521
LOC1001328	0.9106	0.9795	1.0367	0.8633	0.9475	0.0381
DGCR6L	0.6703	1.3778	0.9662	0.7758	0.9475	0.1560
ATP6V1B2	0.7967	1.3187	0.9715	0.7033	0.9475	0.1356
LOC652694	0.9656	0.9702	0.8862	0.9681	0.9475	0.0205
GMEB2	0.8246	1.2580	0.9292	0.7782	0.9475	0.1082
NCOR1	0.8166	1.2932	0.9369	0.7434	0.9475	0.1219
LOC1001340	0.9496	1.0324	0.9178	0.8904	0.9475	0.0308
RLTPR	0.9116	1.0812	0.8604	0.9370	0.9475	0.0473
MC2R	0.9564	1.0410	0.9774	0.8154	0.9475	0.0476
LOC727828	0.9427	1.0096	0.9573	0.8805	0.9475	0.0266
EGR2	0.8867	1.1346	0.9367	0.8322	0.9476	0.0659
LOC1001341	1.0422	0.8725	0.9279	0.9476	0.9476	0.0353

LOC1001316	0.8474	1.0548	1.0063	0.8817	0.9476	0.0494
PFTK2	0.8999	1.0366	1.0057	0.8480	0.9476	0.0442
HSD17B13	0.9221	0.9582	0.9741	0.9359	0.9476	0.0116
FFAR3	0.9424	1.2338	0.8534	0.7607	0.9476	0.1024
LOC1001334	0.8808	0.9982	0.9703	0.9410	0.9476	0.0251
LOC401002	0.8830	1.0819	0.8689	0.9566	0.9476	0.0487
FLJ14107	0.8875	0.9941	1.0211	0.8877	0.9476	0.0351
LOC644667	0.8423	1.0985	0.9973	0.8523	0.9476	0.0615
LOC643605	0.9764	1.0509	0.9605	0.8027	0.9476	0.0522
LOC1001327	1.0333	1.0207	0.9637	0.7728	0.9476	0.0602
LOC728709	0.8570	1.0936	0.8670	0.9729	0.9476	0.0553
GALNT5	0.9303	1.1250	0.8953	0.8399	0.9476	0.0620
LOC652038	0.8743	1.0719	0.8904	0.9539	0.9476	0.0448
LOC652271	0.8870	1.0776	0.9420	0.8839	0.9476	0.0453
FAM55B	0.9480	0.9375	0.9915	0.9136	0.9476	0.0163
RGS20	0.9022	1.0330	0.9653	0.8900	0.9476	0.0329
LOC1001283	0.9939	0.9811	1.0576	0.7580	0.9476	0.0654
LOC653590	0.8535	1.1356	1.0247	0.7769	0.9477	0.0813
LOC1001283	0.9025	1.0740	0.9937	0.8204	0.9477	0.0550
SMCR5	0.9060	1.0242	1.0475	0.8130	0.9477	0.0545
PDE8B	0.9593	1.0808	0.8969	0.8537	0.9477	0.0494
LOC651738	0.8636	1.0817	0.9023	0.9431	0.9477	0.0475
LOC286367	0.9529	0.9518	0.9367	0.9492	0.9477	0.0037
GPR27	0.9216	0.9655	0.9792	0.9244	0.9477	0.0145
LOC727869	0.8809	1.1114	0.9859	0.8126	0.9477	0.0652
MAGEB1	0.9186	1.0314	0.9550	0.8856	0.9477	0.0313
LOC732428	0.8661	0.9672	0.9641	0.9933	0.9477	0.0280
C16orf54	0.8887	1.0192	1.0650	0.8179	0.9477	0.0571
CYP27C1	0.9549	1.0639	0.8453	0.9267	0.9477	0.0452
LOC1001297	0.9440	1.0377	0.9386	0.8706	0.9477	0.0343
SNORD114-1	0.8887	0.9817	1.0188	0.9015	0.9477	0.0314
DKFZP434I07	0.9401	1.2109	0.8075	0.8323	0.9477	0.0923
LOC652281	0.8598	1.2117	0.9162	0.8031	0.9477	0.0910
LOC1001338	1.0106	0.9481	0.9563	0.8759	0.9477	0.0277
LOC401317	0.9484	1.1019	0.8508	0.8898	0.9477	0.0552
C17orf71	0.9089	1.1221	1.0238	0.7361	0.9477	0.0829
LOC653669	0.9162	0.9605	0.9720	0.9422	0.9477	0.0122
LOC644852	0.8962	1.0291	0.9559	0.9096	0.9477	0.0300
DDX59	0.9455	1.0551	0.9489	0.8414	0.9477	0.0436
MIR506	0.9595	1.0339	0.9287	0.8687	0.9477	0.0344
RPTN	0.9056	1.0999	0.9199	0.8656	0.9478	0.0520
FPGS	0.8279	1.0845	1.0599	0.8187	0.9478	0.0720
LOC389963	0.8737	1.1284	0.9163	0.8727	0.9478	0.0611
F2RL2	0.8684	1.0254	0.9966	0.9007	0.9478	0.0376
LOC1001330	0.9847	0.9160	0.9963	0.8941	0.9478	0.0252
LOC400954	0.9375	1.0570	0.9278	0.8689	0.9478	0.0394

LOC644411	0.8795	1.0337	1.0077	0.8703	0.9478	0.0425
MGC4677	0.8289	1.1224	0.9917	0.8482	0.9478	0.0686
FLJ41856	0.8899	1.1006	0.9732	0.8275	0.9478	0.0590
GJD4	0.8491	1.0830	0.9895	0.8696	0.9478	0.0547
MIR204	0.9767	0.9608	1.0159	0.8378	0.9478	0.0384
LOC1001325	0.8831	1.0027	0.9593	0.9463	0.9478	0.0247
LOC652211	0.9099	0.9605	1.0423	0.8786	0.9478	0.0357
LOC645181	0.9451	1.0597	0.9825	0.8040	0.9478	0.0535
C1orf229	0.9299	1.0597	0.9048	0.8970	0.9478	0.0379
USP49	0.8414	1.2142	0.9396	0.7961	0.9478	0.0937
OR2T35	0.9598	0.9846	0.9748	0.8721	0.9478	0.0257
LOC1001313	0.9181	1.1119	0.9008	0.8606	0.9478	0.0560
PPP3CA	0.9362	1.3369	0.7827	0.7356	0.9479	0.1366
LOC647499	0.9254	1.0449	0.9180	0.9032	0.9479	0.0327
LOC652760	0.9042	1.0634	0.9241	0.8997	0.9479	0.0389
TAS1R1	0.9502	0.9309	1.0019	0.9085	0.9479	0.0199
FUZ	0.8792	1.0188	1.0771	0.8163	0.9479	0.0604
LOC1001281	0.9073	1.0757	0.9298	0.8786	0.9479	0.0439
LOC648761	0.9400	0.9884	0.9790	0.8841	0.9479	0.0237
TM4SF20	0.9987	0.9316	0.9734	0.8879	0.9479	0.0243
LOC1001315	0.8259	1.0446	1.0268	0.8943	0.9479	0.0527
SCARNA11	0.8743	1.1367	0.9626	0.8180	0.9479	0.0696
PDGFB	0.8659	1.1704	0.9863	0.7690	0.9479	0.0865
POU5F1	0.9360	1.0759	0.9772	0.8025	0.9479	0.0567
MAPK8	0.8452	1.1455	0.9947	0.8062	0.9479	0.0774
MBTPS1	0.8899	1.2752	0.9442	0.6823	0.9479	0.1228
LOC284648	0.9151	0.8929	0.9984	0.9853	0.9479	0.0259
LOC1001320	0.7594	0.9770	1.1201	0.9352	0.9479	0.0743
LOC728601	0.8674	1.0635	1.0268	0.8340	0.9479	0.0570
FLJ43980	0.8388	1.0335	1.0144	0.9050	0.9479	0.0461
LOC645126	0.9545	0.9836	0.9732	0.8805	0.9479	0.0233
LOC728347	0.8577	1.0678	0.9774	0.8888	0.9479	0.0473
LOC651647	0.9251	1.0174	0.8889	0.9604	0.9479	0.0274
LOC646836	0.8801	1.0797	1.0279	0.8041	0.9479	0.0639
REG1B	0.9400	1.1154	0.8223	0.9141	0.9480	0.0612
LOC1001319	0.8696	0.9101	1.2967	0.7154	0.9480	0.1236
SYN3	0.8549	0.9600	1.0686	0.9083	0.9480	0.0456
LOC642451	0.8155	1.0793	0.9953	0.9017	0.9480	0.0571
LOC728796	0.8730	1.0345	0.9730	0.9115	0.9480	0.0354
CRYBG3	0.8715	1.1544	0.9556	0.8103	0.9480	0.0750
LOC730631	0.8988	1.0241	0.9795	0.8897	0.9480	0.0324
LOC651659	0.9345	1.0152	0.9797	0.8625	0.9480	0.0329
LOC1001335	0.9446	1.0160	0.8813	0.9501	0.9480	0.0275
LOC643158	0.7955	1.0731	1.0019	0.9215	0.9480	0.0595
COL5A3	0.8274	1.0359	1.0032	0.9257	0.9480	0.0464
LOC729435	0.8383	1.1131	0.9975	0.8431	0.9480	0.0663

LOC644889	0.8377	1.1365	1.0792	0.7387	0.9480	0.0952
LOC390877	0.9406	0.9595	0.9997	0.8924	0.9480	0.0223
LOC285479	0.8755	1.0953	0.9657	0.8558	0.9481	0.0546
PLGLB2	0.8996	0.9852	1.0588	0.8486	0.9481	0.0464
LOC390956	0.8439	1.0306	1.0314	0.8864	0.9481	0.0486
CMAS	0.8843	1.3273	0.9007	0.6799	0.9481	0.1360
LOC1001320	0.9119	1.0452	1.0031	0.8321	0.9481	0.0476
LOC642344	0.9640	1.0239	1.0509	0.7536	0.9481	0.0673
LOC388312	0.8365	1.0269	1.0003	0.9286	0.9481	0.0426
ARMC5	0.9462	0.9926	0.9687	0.8850	0.9481	0.0231
LOC441073	0.8167	1.0349	1.2138	0.7269	0.9481	0.1097
LOC1001331	0.8450	1.1209	1.0554	0.7711	0.9481	0.0833
CDY1B	0.8349	1.0538	0.9836	0.9201	0.9481	0.0466
C20orf95	0.8986	1.0460	0.9560	0.8918	0.9481	0.0357
LOC442180	0.7548	1.1299	1.0405	0.8673	0.9481	0.0844
OR6C76	0.9270	0.9756	0.9237	0.9662	0.9481	0.0133
PLAC4	0.8976	0.9571	0.9858	0.9520	0.9481	0.0184
LOC1001298	0.8937	1.0156	1.0211	0.8621	0.9481	0.0411
LOC653197	0.8777	1.0437	0.9289	0.9423	0.9481	0.0348
LOC1001316	0.8078	1.0633	1.0087	0.9128	0.9481	0.0562
LOC642216	0.9776	0.9697	0.9605	0.8848	0.9482	0.0214
FIS	0.9356	0.9436	1.0541	0.8594	0.9482	0.0401
SAMD6	0.7965	1.0850	1.0103	0.9009	0.9482	0.0631
LOC649856	1.0000	1.0245	0.9700	0.7982	0.9482	0.0512
LOC728670	0.8021	1.0239	1.0521	0.9146	0.9482	0.0570
LOC643261	0.8181	1.0390	1.0430	0.8926	0.9482	0.0557
NLRX1	0.9221	1.2263	0.8810	0.7634	0.9482	0.0986
LOC441481	0.8033	1.2778	0.9303	0.7813	0.9482	0.1147
ADAT2	0.8334	1.1086	1.0375	0.8133	0.9482	0.0736
ELAVL1	0.9064	1.1840	0.8170	0.8854	0.9482	0.0809
GALNT3	0.8716	1.0894	0.9250	0.9068	0.9482	0.0484
C19orf73	0.8731	1.0566	0.9490	0.9141	0.9482	0.0393
ANXA7	0.8812	1.1536	0.9861	0.7720	0.9482	0.0812
LOC440345	0.8924	1.0732	0.9557	0.8716	0.9482	0.0453
LOC1001344	0.8766	0.9940	0.9996	0.9227	0.9482	0.0296
DRGX	0.8818	0.9551	1.0144	0.9417	0.9482	0.0272
LOC349114	0.8733	1.0950	0.9194	0.9052	0.9482	0.0499
PRAMEF17	0.9446	1.0298	0.9129	0.9056	0.9482	0.0285
ZNF366	0.9724	0.9840	0.9548	0.8819	0.9482	0.0229
GBP3	0.9043	0.9790	1.0662	0.8435	0.9482	0.0481
C1QTNF8	0.8589	1.0246	0.9901	0.9194	0.9483	0.0370
COL24A1	1.0041	0.9220	0.9495	0.9175	0.9483	0.0199
CD69	0.8844	1.0173	0.9823	0.9091	0.9483	0.0310
MIR548F3	0.8135	1.1057	0.9776	0.8963	0.9483	0.0623
NEK3	0.9132	1.0542	0.9892	0.8365	0.9483	0.0471
LGALS9B	0.8914	1.1049	0.9707	0.8262	0.9483	0.0600

MIR519B	0.8796	1.0250	1.0103	0.8783	0.9483	0.0402
LOC730232	0.8150	1.1258	0.9523	0.9001	0.9483	0.0656
C13orf28	0.9230	1.0653	0.9745	0.8304	0.9483	0.0491
ZNF184	0.8653	1.0992	0.9649	0.8638	0.9483	0.0556
A4GNT	1.0160	1.0059	0.9930	0.7783	0.9483	0.0569
NCRNA0015	0.7913	1.0581	1.1254	0.8185	0.9483	0.0841
FBXO41	0.9052	1.0295	1.0174	0.8412	0.9483	0.0454
ZNF451	0.8612	1.1219	1.0097	0.8006	0.9483	0.0726
IDH3A	0.6874	1.4990	0.9323	0.6747	0.9483	0.1929
FAM19A5	0.9058	1.2157	1.0542	0.6176	0.9483	0.1271
LOC1001318	0.8894	1.0881	1.0035	0.8124	0.9484	0.0609
LOC648691	0.8110	1.1217	0.9846	0.8761	0.9484	0.0680
SNORD94	0.8022	1.1560	1.0073	0.8278	0.9484	0.0829
LOC652562	0.9054	0.9467	1.0148	0.9266	0.9484	0.0237
LOC644305	0.9060	1.0964	0.8950	0.8961	0.9484	0.0494
PNMAL2	0.8064	1.1008	0.9847	0.9016	0.9484	0.0625
C3orf16	0.9446	0.9746	0.9633	0.9110	0.9484	0.0139
CCDC103	0.8404	1.0627	1.0807	0.8097	0.9484	0.0716
KCNA1	0.8672	1.0484	0.9734	0.9045	0.9484	0.0399
C12orf12	0.9049	1.0500	0.9764	0.8623	0.9484	0.0412
ING5	0.9109	1.0087	0.9868	0.8871	0.9484	0.0292
CLCA3	0.8373	1.0672	0.9294	0.9595	0.9484	0.0474
HECTD3	0.8005	1.1908	1.0133	0.7889	0.9484	0.0959
ARFRP1	0.8948	1.0931	0.8640	0.9417	0.9484	0.0508
PGLYRP3	0.9348	0.9637	1.0377	0.8575	0.9484	0.0372
ST8SIA4	0.8757	1.0697	0.9503	0.8980	0.9484	0.0434
LOC1001335	0.8694	0.9956	1.0348	0.8939	0.9484	0.0397
TGFB2	0.9672	1.0269	0.9439	0.8558	0.9484	0.0355
LOC1001338	0.8987	1.1114	0.8760	0.9075	0.9484	0.0547
ALDH8A1	0.8629	1.0597	0.9551	0.9160	0.9484	0.0416
NOVA2	0.8558	1.0270	1.0336	0.8774	0.9484	0.0475
LOC1001332	0.7902	1.0390	1.0942	0.8705	0.9484	0.0710
LOC285544	0.9516	1.0455	0.9412	0.8554	0.9484	0.0389
LOC643327	0.9227	0.9307	1.0933	0.8471	0.9484	0.0518
LOC400752	0.9844	0.8839	0.9791	0.9465	0.9485	0.0231
CPSF1	1.0337	1.1350	0.9107	0.7144	0.9485	0.0905
LOC641992	0.8888	1.1033	0.9904	0.8115	0.9485	0.0633
ATG7	0.8974	1.1369	1.0432	0.7164	0.9485	0.0917
MIR155HG	0.9475	0.9176	1.0546	0.8743	0.9485	0.0384
PLEKHG7	0.9614	1.0876	0.8928	0.8522	0.9485	0.0516
LOC1001286	0.9676	1.0191	0.9866	0.8206	0.9485	0.0439
LOC399715	0.9363	0.9819	0.9571	0.9186	0.9485	0.0136
LOC728939	0.8276	1.1054	0.9874	0.8736	0.9485	0.0622
PTPN20B	0.8960	1.0748	0.9968	0.8264	0.9485	0.0548
MIRLET7G	0.8224	0.9998	1.0669	0.9049	0.9485	0.0536
RASA4	0.8712	1.0253	0.9912	0.9064	0.9485	0.0359

HOXC12	0.9283	0.9247	1.1362	0.8048	0.9485	0.0688
LOC338756	0.9102	1.0217	1.0134	0.8488	0.9485	0.0418
SLC2A9	0.9182	1.0837	0.9479	0.8443	0.9485	0.0501
C16orf85	0.8760	1.1004	1.0651	0.7525	0.9485	0.0818
EPS8	0.8550	1.0503	0.9714	0.9173	0.9485	0.0414
LOC650369	0.8518	1.2413	0.9890	0.7121	0.9485	0.1128
LOC651177	0.9942	0.8887	1.0015	0.9098	0.9485	0.0288
LOC730268	0.8796	1.0493	1.0095	0.8558	0.9485	0.0476
PKDREJ	0.8948	0.9474	0.9646	0.9873	0.9486	0.0197
LOC1001334	0.9385	1.0067	0.9085	0.9406	0.9486	0.0207
ZNF568	0.8675	1.2481	0.9062	0.7724	0.9486	0.1037
LOC653657	0.9258	1.1227	0.8977	0.8482	0.9486	0.0602
FLJ35946	0.8979	1.1078	0.9000	0.8886	0.9486	0.0531
UAP1L1	0.9571	1.3438	0.9413	0.5521	0.9486	0.1616
TBC1D28	0.8401	1.0350	1.0650	0.8543	0.9486	0.0589
LOC727878	0.8489	1.1130	1.0306	0.8019	0.9486	0.0737
ZNF71	0.9153	1.0199	1.0584	0.8008	0.9486	0.0578
LOC1001285	0.9932	1.0168	0.9562	0.8282	0.9486	0.0420
MIR452	0.8702	0.9934	1.0313	0.8995	0.9486	0.0381
NOS1AP	0.8605	1.0786	1.0185	0.8370	0.9486	0.0592
LOC643520	0.8338	0.9627	1.0467	0.9513	0.9486	0.0438
CHML	0.8269	1.1209	0.9822	0.8645	0.9486	0.0663
LST1	0.9105	1.0672	0.9684	0.8484	0.9487	0.0465
LOC727927	0.8259	1.1092	0.9454	0.9141	0.9487	0.0592
P2RY8	1.0695	0.9652	0.9155	0.8444	0.9487	0.0473
LOC1001311	0.8444	1.0342	0.9976	0.9185	0.9487	0.0423
MIR223	0.9459	1.0549	0.9463	0.8476	0.9487	0.0424
LOC643359	0.9856	0.8826	0.9338	0.9927	0.9487	0.0256
CGA	0.9712	1.0158	0.9708	0.8369	0.9487	0.0387
LOC285929	0.9158	1.0595	1.0059	0.8135	0.9487	0.0539
C19orf63	0.8864	1.0028	1.0673	0.8381	0.9487	0.0525
LOC645052	0.9005	0.9979	0.9940	0.9024	0.9487	0.0273
SFRS5	0.8364	1.1398	0.9652	0.8534	0.9487	0.0698
LOC729369	0.8923	1.0381	1.0046	0.8598	0.9487	0.0430
FAM113B	0.9355	1.0292	0.9645	0.8657	0.9487	0.0339
GPAM	0.9271	1.1565	1.0062	0.7050	0.9487	0.0941
PRR22	0.9541	0.9553	0.8882	0.9973	0.9487	0.0225
LOC653515	0.9524	1.0824	0.9359	0.8241	0.9487	0.0529
LOC387647	0.7672	1.1224	1.0302	0.8751	0.9487	0.0792
LOC1001341	0.8758	1.2027	0.9204	0.7960	0.9487	0.0885
NHLRC1	0.8055	1.0761	0.9945	0.9188	0.9487	0.0575
LOC1001329	0.8682	1.0037	1.0210	0.9021	0.9487	0.0375
CFL1	0.7584	1.3001	0.9768	0.7597	0.9488	0.1279
PROCA1	0.8746	1.0315	1.0063	0.8826	0.9488	0.0408
LOC1001328	0.9271	1.0151	0.9668	0.8861	0.9488	0.0276
PCA3	0.9484	1.0167	0.9150	0.9150	0.9488	0.0240

LCE1C	0.9311	1.1022	0.9189	0.8428	0.9488	0.0547
LOC642169	0.9171	1.0210	1.0968	0.7602	0.9488	0.0728
UHRF1BP1L	0.8854	1.0577	1.0099	0.8422	0.9488	0.0508
BMP3	0.8665	1.0810	0.8765	0.9712	0.9488	0.0500
TTC21A	0.8879	1.1078	0.9972	0.8023	0.9488	0.0663
LY6G6F	0.8814	1.0177	0.9229	0.9732	0.9488	0.0297
FLJ45422	0.8780	1.1031	0.9188	0.8954	0.9488	0.0521
LANCL2	0.9263	1.2022	0.8679	0.7989	0.9488	0.0884
LOC650494	0.8604	1.1177	0.9165	0.9008	0.9488	0.0575
NCOR2	0.9130	1.2975	0.8824	0.7025	0.9488	0.1251
LOC652330	0.8564	1.0526	1.0517	0.8347	0.9488	0.0598
CYTL1	0.8913	0.9448	1.0005	0.9588	0.9488	0.0225
FAM169B	0.8890	1.0823	0.9598	0.8644	0.9489	0.0488
MAGEA1	0.8732	1.1097	1.0131	0.7995	0.9489	0.0695
LOC644012	0.8696	0.8592	1.1367	0.9302	0.9489	0.0645
FSIP2	0.9284	1.0100	0.9836	0.8736	0.9489	0.0303
OR5D14	0.8826	1.0862	1.0326	0.7942	0.9489	0.0672
LOC652119	0.8799	1.0690	0.9829	0.8637	0.9489	0.0480
EID3	0.8676	0.9907	0.9441	0.9932	0.9489	0.0294
LOC647050	0.7969	1.2305	0.9051	0.8631	0.9489	0.0965
LOC1001316	0.8436	1.0336	1.0268	0.8916	0.9489	0.0480
SNORD14B	0.8983	0.9268	1.0744	0.8961	0.9489	0.0424
NECAP1	0.8093	1.3508	0.9760	0.6596	0.9489	0.1487
NOM1	0.8812	1.0992	0.9571	0.8581	0.9489	0.0544
BCL6B	0.7993	1.1168	0.9930	0.8865	0.9489	0.0686
REXO1L5P	0.8725	1.0236	0.9661	0.9335	0.9489	0.0315
FAM13AOS	0.9275	1.1233	0.8489	0.8960	0.9489	0.0603
LOC643872	0.8122	0.9589	1.0572	0.9675	0.9489	0.0507
LOC1001302	1.0132	0.9688	0.9359	0.8778	0.9489	0.0285
LOC402483	0.8483	0.9990	1.0089	0.9396	0.9489	0.0369
CD97	0.8097	1.3788	0.9190	0.6883	0.9489	0.1508
RHOXF2	0.8605	0.9320	1.1134	0.8899	0.9490	0.0568
RNF10	0.9195	1.1399	1.0174	0.7190	0.9490	0.0889
FZD6	0.8196	1.1859	1.0085	0.7817	0.9490	0.0933
YY1	0.6837	1.3681	1.0006	0.7435	0.9490	0.1557
MIR27B	0.8637	1.0049	1.0752	0.8521	0.9490	0.0546
C10orf71	0.8365	1.0218	0.9655	0.9721	0.9490	0.0395
KCNIP3	0.9659	1.0097	0.9273	0.8930	0.9490	0.0251
ASPG	0.8796	1.1827	0.8466	0.8870	0.9490	0.0784
CNN1	0.9659	0.9736	1.0190	0.8373	0.9490	0.0390
LOC1001308	0.9630	0.9522	0.9849	0.8959	0.9490	0.0190
LOC150207	0.9168	0.9487	0.9866	0.9438	0.9490	0.0144
LOC642502	0.7607	1.1074	1.0740	0.8538	0.9490	0.0843
LOC653629	0.9344	1.0425	0.9944	0.8247	0.9490	0.0470
HIST1H2BH	0.8981	1.0319	0.9886	0.8774	0.9490	0.0367
LOC645721	0.9229	1.1382	0.9689	0.7661	0.9490	0.0766

C8orf74	0.9124	1.0528	0.9456	0.8853	0.9490	0.0367
RALGAPB	0.8724	1.2528	0.9695	0.7015	0.9490	0.1154
LOC643558	0.8853	1.0686	0.9753	0.8670	0.9491	0.0463
SNORD114-1	0.9111	0.9868	1.0100	0.8883	0.9491	0.0293
CXorf50	0.8574	1.0315	1.0257	0.8816	0.9491	0.0462
LOC652466	0.9248	1.0721	0.9292	0.8701	0.9491	0.0431
OR52E8	0.9133	1.0838	0.8827	0.9164	0.9491	0.0455
CADM4	0.8827	1.1827	0.9260	0.8050	0.9491	0.0818
PRMT8	0.8784	1.0533	0.9831	0.8815	0.9491	0.0424
HS6ST3	0.8109	1.0938	1.0018	0.8899	0.9491	0.0621
LOC1001345	0.8105	0.9442	1.1527	0.8889	0.9491	0.0732
RSPH10B2	0.9235	0.9999	0.9811	0.8919	0.9491	0.0250
HIST1H2BL	0.9653	1.0369	0.8965	0.8978	0.9491	0.0334
BCAR4	0.8209	1.0380	0.9584	0.9793	0.9491	0.0460
SERPINA4	0.9889	1.0041	0.9404	0.8631	0.9491	0.0317
LOC1001327	0.7219	1.2561	1.0904	0.7282	0.9491	0.1337
EFNA5	0.7392	1.3173	0.8642	0.8759	0.9491	0.1266
LOC1001292	0.9649	1.0159	0.9847	0.8311	0.9491	0.0407
LOC647041	0.9252	1.0540	0.9393	0.8781	0.9491	0.0373
SFTPA2	0.9474	0.9895	0.9585	0.9013	0.9491	0.0183
TNFRSF25	0.8780	0.9613	0.9860	0.9713	0.9491	0.0243
C9orf64	0.8646	1.2275	0.9022	0.8023	0.9492	0.0950
ZNF691	0.9095	1.0867	1.0575	0.7429	0.9492	0.0789
LOC653260	0.9495	0.8921	1.0866	0.8686	0.9492	0.0488
LOC729706	0.9214	1.0289	0.9470	0.8995	0.9492	0.0283
LOC652062	0.8636	1.0651	1.0152	0.8528	0.9492	0.0535
C20orf71	0.9216	1.0401	0.9669	0.8682	0.9492	0.0364
KIAA1328	0.9485	1.0826	0.9309	0.8348	0.9492	0.0510
TM6SF1	0.9812	0.9292	1.0013	0.8851	0.9492	0.0262
SLC9A7	0.9244	0.9822	1.0707	0.8195	0.9492	0.0527
LOC646813	0.9031	0.9447	1.0746	0.8744	0.9492	0.0442
MIR128-1	0.9866	0.9450	0.9658	0.8994	0.9492	0.0186
BAGE2	0.9381	1.0077	0.9461	0.9049	0.9492	0.0214
LOC642553	0.8638	1.1503	0.9393	0.8434	0.9492	0.0701
BARHL2	0.9514	1.0735	0.9226	0.8494	0.9492	0.0466
SPATA17	0.9056	0.9375	1.0241	0.9297	0.9492	0.0259
PIGS	1.0308	1.2250	0.8207	0.7204	0.9492	0.1124
PLEKHA8	0.9309	1.1232	0.9219	0.8208	0.9492	0.0631
FLJ46358	0.8237	1.0086	1.0791	0.8855	0.9492	0.0579
LOC645548	0.9465	1.0078	0.9277	0.9148	0.9492	0.0206
LOC649366	0.8462	1.0368	1.0148	0.8993	0.9492	0.0457
GPR156	0.9814	1.0763	0.8850	0.8543	0.9492	0.0503
LOC400682	0.8747	1.0770	0.9772	0.8681	0.9492	0.0494
NUS1	0.8665	1.1921	0.8920	0.8463	0.9492	0.0815
CLEC3A	0.9340	0.9331	0.9876	0.9423	0.9492	0.0130
TAS2R7	0.9259	0.9644	0.9862	0.9204	0.9492	0.0158

LOC648117	0.9612	1.0304	1.0211	0.7842	0.9492	0.0571
SMPD2	0.8530	1.1777	0.9587	0.8076	0.9492	0.0825
RP9P	0.8180	1.0269	1.0513	0.9008	0.9493	0.0548
FAM25A	0.8594	1.0479	1.0240	0.8657	0.9493	0.0503
LOC1001309	0.8331	1.0892	1.0239	0.8508	0.9493	0.0635
NCRNA0012(	1.0163	1.0846	0.9162	0.7800	0.9493	0.0662
C19orf61	0.8556	1.1514	0.8733	0.9168	0.9493	0.0686
LOC729742	0.7513	1.2266	1.0791	0.7401	0.9493	0.1214
SNORD32A	0.7604	1.2042	1.1029	0.7296	0.9493	0.1199
PPP1R3C	0.9554	0.9810	1.0276	0.8331	0.9493	0.0415
DHDH	0.9410	1.2117	1.0176	0.6269	0.9493	0.1216
LOC388022	0.8726	1.0708	0.9788	0.8750	0.9493	0.0475
SNORD121B	0.9251	0.9495	0.9915	0.9310	0.9493	0.0150
LOC1001296	0.8686	1.0054	1.0209	0.9023	0.9493	0.0376
FOXD4L5	0.8980	1.0211	1.0206	0.8575	0.9493	0.0421
LOC339209	0.9710	1.0089	0.9515	0.8658	0.9493	0.0303
GTF3C4	0.9019	1.0992	0.9794	0.8167	0.9493	0.0600
LOC643422	0.8163	1.1019	0.9619	0.9171	0.9493	0.0593
C9orf31	0.9731	0.9627	0.9613	0.9002	0.9493	0.0166
GOLGA8B	0.9197	1.0962	0.9180	0.8634	0.9493	0.0507
ANKRD36	0.8895	1.0689	0.9786	0.8604	0.9493	0.0471
LOC732425	0.8243	1.1901	1.0076	0.7755	0.9494	0.0945
CDC37L1	0.9418	1.0727	0.8858	0.8971	0.9494	0.0429
WT1	0.9182	0.9889	0.9691	0.9212	0.9494	0.0176
EFTUD1	0.8262	1.2330	1.0076	0.7307	0.9494	0.1106
LOC389465	0.8508	1.0353	1.0194	0.8921	0.9494	0.0459
ERCC-00060	0.9510	1.0866	0.9690	0.7909	0.9494	0.0608
ERBB2IP	0.8176	1.2295	0.8979	0.8526	0.9494	0.0948
LOC400197	0.9648	1.0757	0.8953	0.8618	0.9494	0.0472
LOC646938	0.8930	1.1105	0.9164	0.8777	0.9494	0.0543
LOC644499	0.9830	1.1119	0.9076	0.7953	0.9494	0.0665
RBP1	0.9374	0.9498	0.9810	0.9295	0.9494	0.0113
OR5D18	0.8571	1.0103	1.0586	0.8717	0.9494	0.0502
RBM23	0.7843	1.3467	0.9660	0.7008	0.9494	0.1435
LOC728942	0.7831	1.1831	0.9963	0.8352	0.9494	0.0901
LOC651301	0.8892	1.1056	0.9435	0.8594	0.9494	0.0549
TXNDC6	0.8707	1.1784	0.8800	0.8687	0.9494	0.0764
FSTL4	0.9293	1.0783	0.9302	0.8599	0.9495	0.0460
UBQLNL	0.9921	0.9298	1.0076	0.8683	0.9495	0.0318
LOC649101	0.9439	1.0129	0.9915	0.8496	0.9495	0.0363
SNORA53	0.8884	1.0553	0.8945	0.9597	0.9495	0.0388
FAM9A	0.9944	1.0506	0.9342	0.8187	0.9495	0.0497
C5AR1	0.8805	1.0140	1.0467	0.8567	0.9495	0.0474
CNTF	0.8844	1.0767	0.9546	0.8822	0.9495	0.0456
OPA3	0.8845	1.0617	0.9697	0.8820	0.9495	0.0426
LOC440122	0.9538	1.1100	0.9674	0.7666	0.9495	0.0705

UNQ5830	0.8682	0.9756	1.0895	0.8645	0.9495	0.0533
LOC652216	0.9090	1.0061	0.9271	0.9558	0.9495	0.0212
LOC390561	0.9193	1.0436	1.0351	0.7998	0.9495	0.0574
ZNF542	0.9795	0.9921	0.9780	0.8483	0.9495	0.0339
C8ORFK36	0.8422	1.0553	1.0117	0.8888	0.9495	0.0502
LOC389333	0.9469	1.0802	0.8945	0.8763	0.9495	0.0461
LOC653340	0.9396	0.9648	0.9240	0.9696	0.9495	0.0108
HOXD8	0.8572	1.0930	0.9704	0.8774	0.9495	0.0538
MED4	0.7607	1.1906	1.0081	0.8387	0.9495	0.0955
C1orf183	0.8656	1.0537	1.0401	0.8387	0.9495	0.0566
GUCA1A	0.7913	1.0669	1.0177	0.9221	0.9495	0.0607
MIR647	0.9244	1.0470	0.9363	0.8904	0.9495	0.0339
ZAN	0.8457	1.0046	1.0068	0.9411	0.9495	0.0378
CHN1	0.8719	1.0388	1.0277	0.8598	0.9495	0.0485
LOC399978	0.8084	1.1470	0.9985	0.8443	0.9496	0.0777
LOC651729	0.9087	1.0842	0.9200	0.8853	0.9496	0.0455
LOC1001292	0.9306	1.0361	0.9459	0.8856	0.9496	0.0316
LOC1001325	0.7793	1.0834	1.1152	0.8204	0.9496	0.0871
LOC642602	0.9149	1.0842	0.9636	0.8358	0.9496	0.0520
LOC1001337	0.8404	0.9776	1.0617	0.9188	0.9496	0.0467
MRGPRX4	0.9811	1.0419	0.9417	0.8338	0.9496	0.0438
MYCBP	0.9166	1.0849	0.9488	0.8483	0.9496	0.0497
RNASE6	0.9300	1.0716	0.9650	0.8320	0.9497	0.0495
LCK	0.8945	1.0583	0.9832	0.8626	0.9497	0.0443
RASGEF1A	0.8743	0.9285	1.1169	0.8789	0.9497	0.0571
LOC647448	0.8921	1.0234	1.0466	0.8366	0.9497	0.0508
POLL	0.9672	1.0562	0.9569	0.8184	0.9497	0.0491
DC36	0.8633	0.9984	0.9552	0.9818	0.9497	0.0301
QRICH2	0.8385	1.0719	1.0604	0.8279	0.9497	0.0673
LOC1001283[illegible]	1.0530	1.0584	0.9209	0.7664	0.9497	0.0689
OR1D4	0.9081	1.0219	0.9945	0.8742	0.9497	0.0350
LOC642685	0.9013	0.9604	1.0269	0.9101	0.9497	0.0289
CTAG2	0.9430	1.0856	0.9536	0.8166	0.9497	0.0550
LOC644860	0.9559	0.9935	0.9991	0.8503	0.9497	0.0345
PDZK1P1	0.9503	1.0742	0.9204	0.8539	0.9497	0.0461
SFRP4	0.9368	1.0653	0.9725	0.8244	0.9497	0.0498
TTTY23	0.8985	1.0103	1.0585	0.8316	0.9497	0.0517
CSH1	0.8662	1.0930	0.9138	0.9258	0.9497	0.0495
PSME4	0.8789	1.1964	0.9349	0.7887	0.9497	0.0876
FAM24A	0.8734	1.0075	1.0453	0.8727	0.9497	0.0449
LOC642185	0.9213	0.9739	1.0318	0.8719	0.9497	0.0344
FBXO40	0.8623	1.0152	0.9936	0.9278	0.9497	0.0345
LOC402665	0.9091	1.0210	0.9367	0.9321	0.9497	0.0245
LOC1001306[illegible]	0.9285	1.0060	0.9977	0.8668	0.9497	0.0326
LOC1001329	0.9407	0.8919	1.1002	0.8662	0.9497	0.0525
LOC648111	0.8516	1.0153	1.0067	0.9254	0.9497	0.0385

SERPINA10	0.8616	1.1801	1.0008	0.7564	0.9497	0.0917
LOC645139	0.9528	0.9390	0.8777	1.0295	0.9498	0.0312
LOC1001299	0.8475	1.0620	0.9973	0.8923	0.9498	0.0488
ZNF547	0.8944	1.0060	0.9553	0.9434	0.9498	0.0229
LOC641367	0.8824	1.0547	0.9752	0.8869	0.9498	0.0410
APBB2	0.8541	1.1051	1.0168	0.8231	0.9498	0.0670
KRTAP10-1	0.8378	1.1228	1.0105	0.8281	0.9498	0.0713
HCFC1	0.8231	1.1071	1.0957	0.7733	0.9498	0.0881
ADRA1A	0.8906	0.9930	0.9873	0.9284	0.9498	0.0246
OR14C36	0.8620	1.0811	0.9775	0.8786	0.9498	0.0506
TRIM7	0.9192	1.1029	0.9507	0.8265	0.9498	0.0574
LOC541473	0.9624	1.1161	0.8833	0.8374	0.9498	0.0612
PSG4	0.8571	1.1242	0.9319	0.8860	0.9498	0.0601
ADAMTS8	0.8709	1.1048	0.9673	0.8563	0.9498	0.0572
TGOLN2	0.8039	1.2043	0.9584	0.8327	0.9498	0.0912
CD209	0.8582	1.0818	0.9987	0.8606	0.9498	0.0549
DOCK3	0.8498	1.0143	1.1254	0.8099	0.9498	0.0733
SNORA71D	0.9473	1.0509	0.9595	0.8417	0.9498	0.0428
ADORA3	0.8979	1.0485	0.9451	0.9080	0.9498	0.0344
CSF2RA	0.9965	1.1008	0.9845	0.7176	0.9498	0.0817
SNORD83A	0.8970	1.0256	0.9384	0.9385	0.9499	0.0271
TRAF3IP3	0.9210	1.0470	0.9595	0.8719	0.9499	0.0370
LOC729251	0.8525	0.9453	1.1499	0.8518	0.9499	0.0702
LOC645458	0.9876	1.0672	0.9550	0.7897	0.9499	0.0584
OAS1	0.9094	1.0400	0.9810	0.8692	0.9499	0.0379
NUBPL	0.7184	1.2609	1.0396	0.7806	0.9499	0.1248
CLDN22	0.8681	0.8988	1.0132	1.0195	0.9499	0.0389
LCN2	0.8030	1.0791	1.0385	0.8790	0.9499	0.0653
APBA1	0.8424	1.0340	1.0319	0.8913	0.9499	0.0490
SYT2	0.7919	1.1115	0.9363	0.9600	0.9499	0.0654
MIR642	0.9035	0.9971	0.9471	0.9520	0.9499	0.0191
SDC1	0.8310	1.2061	1.0155	0.7470	0.9499	0.1022
RLF	0.9837	1.0802	1.0089	0.7269	0.9499	0.0771
KRTAP1-5	0.9916	1.0225	0.8992	0.8865	0.9499	0.0337
NR1D1	0.8907	0.9348	1.0105	0.9637	0.9499	0.0252
HNF4G	0.9343	0.9933	1.0110	0.8612	0.9500	0.0338
LOC642399	0.9207	1.0012	0.9406	0.9374	0.9500	0.0176
CCDC9	0.9385	1.0830	0.9468	0.8315	0.9500	0.0515
LOC401305	1.0152	1.0457	0.9567	0.7822	0.9500	0.0589
BCAN	0.8777	1.0738	0.9923	0.8561	0.9500	0.0510
EWSR1	0.8891	1.2793	0.8868	0.7448	0.9500	0.1148
ZNF500	1.0550	1.0905	0.9021	0.7523	0.9500	0.0775
PRELP	0.8379	1.0990	0.9461	0.9169	0.9500	0.0547
SNORD85	0.9283	0.9989	1.0023	0.8704	0.9500	0.0315
MIR888	0.8234	1.0740	0.9446	0.9580	0.9500	0.0512
UBE2DNL	0.8566	1.0678	1.0221	0.8534	0.9500	0.0556

EXOC6	0.9280	1.1115	0.8823	0.8782	0.9500	0.0550
MIR205	0.8261	0.9230	1.0747	0.9761	0.9500	0.0519
IFIT3	0.8952	1.0857	1.0102	0.8089	0.9500	0.0612
PRG1	0.9627	0.9515	1.0214	0.8644	0.9500	0.0324
CT47A7	0.9614	0.9575	0.9880	0.8931	0.9500	0.0201
C19orf69	0.9121	1.0125	1.0182	0.8572	0.9500	0.0394
MMP12	0.8471	1.0066	1.0527	0.8937	0.9500	0.0479
C1QL3	0.8995	1.0494	1.0043	0.8470	0.9500	0.0465
LOC644694	0.8382	1.1040	0.9770	0.8809	0.9500	0.0590
LOC1001318	0.9263	0.9871	1.0376	0.8492	0.9500	0.0406
C19orf20	0.9157	1.0662	0.9667	0.8514	0.9500	0.0454
LOC730535	0.9199	0.9335	1.0387	0.9081	0.9500	0.0300
CD300LG	0.9107	1.0992	0.9317	0.8586	0.9500	0.0520
DES	0.9778	0.9287	0.9696	0.9241	0.9500	0.0138
LOC649324	0.9154	1.0966	0.9095	0.8787	0.9500	0.0495
LOC728971	0.8964	1.0575	0.9939	0.8524	0.9500	0.0464
LOC1001327	0.9077	1.0681	1.0049	0.8194	0.9501	0.0546
LOC647910	0.8149	1.0440	0.9752	0.9661	0.9501	0.0483
SNTG2	0.9049	1.0724	0.9873	0.8356	0.9501	0.0512
OLFM1	0.8627	1.0112	1.0542	0.8721	0.9501	0.0486
SNORD33	0.6062	1.2509	1.1097	0.8335	0.9501	0.1437
MIR1206	0.9089	1.0430	0.9195	0.9288	0.9501	0.0312
REPS1	0.8215	1.0096	1.0155	0.9537	0.9501	0.0451
KRT35	1.0702	0.9384	0.9042	0.8874	0.9501	0.0414
C4orf22	0.8716	0.9202	1.0181	0.9904	0.9501	0.0333
KIAA0802	0.7910	1.2302	1.0045	0.7746	0.9501	0.1070
EML2	0.8104	1.1879	0.9187	0.8834	0.9501	0.0824
CRISP2	0.8473	1.0000	1.0186	0.9344	0.9501	0.0387
ARHGEF10L	0.8860	1.0566	0.9661	0.8917	0.9501	0.0399
IL8RB	0.9395	1.0144	1.0066	0.8399	0.9501	0.0404
TMEM191A	0.9577	1.0335	1.0004	0.8089	0.9501	0.0496
LOC1001283	0.8927	1.0237	0.9780	0.9060	0.9501	0.0309
GNS	0.8641	1.2754	0.9081	0.7528	0.9501	0.1132
PKN3	1.0328	0.9571	0.9307	0.8798	0.9501	0.0319
LOC1001314	0.9568	0.9820	0.9274	0.9342	0.9501	0.0123
LOC388080	0.9139	1.0077	0.9956	0.8834	0.9501	0.0305
FLJ20518	0.9365	1.0199	0.9716	0.8725	0.9501	0.0310
DKFZp779B1	0.8926	0.9660	1.0661	0.8758	0.9502	0.0433
CRCT1	1.0091	1.0104	0.9130	0.8680	0.9502	0.0356
LOC652646	0.9298	0.9521	1.0284	0.8904	0.9502	0.0290
SH3BP5	0.8964	1.0127	0.9870	0.9045	0.9502	0.0292
ATXN3L	0.9564	1.0028	0.9516	0.8898	0.9502	0.0232
LOC1001309	0.9845	1.0240	0.9148	0.8774	0.9502	0.0331
IFRG15	0.7746	1.2574	0.9518	0.8169	0.9502	0.1091
C17orf103	0.8518	0.9866	1.0334	0.9290	0.9502	0.0391
PMPCB	0.7651	1.4230	0.9566	0.6561	0.9502	0.1694

MRPL10	0.8601	1.0393	1.1190	0.7825	0.9502	0.0778
LOC286444	0.8608	1.0901	1.0846	0.7655	0.9502	0.0815
LOC647138	0.8830	1.0328	0.9405	0.9447	0.9502	0.0309
KLRD1	0.9264	1.0012	0.9987	0.8746	0.9502	0.0306
LOC648742	0.7387	1.1371	1.0874	0.8378	0.9502	0.0962
DTX4	0.8665	1.0762	0.9619	0.8964	0.9502	0.0465
MAP1A	0.8708	1.1389	0.9431	0.8482	0.9503	0.0661
LOC646915	0.8738	1.0286	0.8979	1.0008	0.9503	0.0379
LOC648041	0.8827	1.0777	1.0135	0.8272	0.9503	0.0577
LOC646515	0.8656	1.0583	0.9296	0.9476	0.9503	0.0401
LOC150356	0.7055	0.9789	1.1617	0.9550	0.9503	0.0937
KCNK18	0.8495	0.9644	1.0405	0.9467	0.9503	0.0393
LOC646445	0.8066	1.0749	1.0432	0.8764	0.9503	0.0647
LOC648894	1.0668	1.0246	0.9518	0.7580	0.9503	0.0684
FAM5C	0.9521	1.0701	0.9282	0.8509	0.9503	0.0454
JAKMIP1	0.8852	1.0073	0.9845	0.9243	0.9503	0.0279
FGA	0.8962	1.0644	0.9725	0.8682	0.9503	0.0439
LOC646848	0.8109	1.0734	1.0406	0.8764	0.9503	0.0634
ZBTB8OS	0.9055	1.0656	1.0623	0.7678	0.9503	0.0714
RPS23	0.7805	1.2210	1.0818	0.7180	0.9503	0.1202
LOC1001288	0.8669	0.9736	1.0642	0.8967	0.9503	0.0441
BRD7P3	0.8910	1.0605	1.0172	0.8327	0.9503	0.0532
CLEC2B	0.7779	1.0817	1.0951	0.8467	0.9503	0.0810
FUBP1	0.7892	1.0737	1.1270	0.8115	0.9503	0.0874
LOC728044	0.8200	1.0234	1.0301	0.9279	0.9504	0.0493
LOC647134	1.0867	0.9876	0.9374	0.7898	0.9504	0.0619
LOC644738	0.7991	0.9905	1.1504	0.8615	0.9504	0.0777
C11orf84	0.9905	1.0099	1.0006	0.8005	0.9504	0.0501
LOC646596	0.9531	0.9810	0.9442	0.9232	0.9504	0.0120
SPHAR	0.9260	1.0764	0.9088	0.8902	0.9504	0.0427
LOC647208	0.9061	0.9995	1.0382	0.8577	0.9504	0.0415
DNAJB3	0.8742	0.9879	0.9877	0.9518	0.9504	0.0268
C6orf10	0.9261	0.9630	0.9685	0.9440	0.9504	0.0097
ZNF720	0.9720	1.0505	0.8685	0.9107	0.9504	0.0396
KCNC4	0.9212	1.0382	0.9693	0.8729	0.9504	0.0353
LOC727837	0.9061	1.0240	0.9335	0.9381	0.9504	0.0255
LOC727938	0.8454	0.9137	1.1800	0.8627	0.9504	0.0779
ZNF404	0.8185	1.1133	0.9742	0.8957	0.9504	0.0629
LOC650975	0.8968	1.1030	0.9406	0.8613	0.9504	0.0534
LOC1001298	0.9559	0.9886	1.0151	0.8421	0.9504	0.0381
GPR146	0.8596	1.0413	0.9865	0.9143	0.9504	0.0399
LOC641704	0.8282	1.0802	0.9741	0.9193	0.9504	0.0527
CACNB2	0.8984	1.0223	0.9935	0.8876	0.9504	0.0338
LOC1001316	0.8632	1.1166	1.0192	0.8028	0.9504	0.0717
CEACAM5	0.9697	1.0670	0.9400	0.8250	0.9504	0.0498
C21orf96	0.8649	1.0118	1.0682	0.8569	0.9505	0.0530

LOC1001345	0.8741	1.1305	0.9498	0.8475	0.9505	0.0638
DPPA4	0.8791	1.0440	0.9631	0.9158	0.9505	0.0356
LOC652070	0.8753	1.0442	0.9939	0.8887	0.9505	0.0409
MAP3K5	0.7932	1.0040	1.1123	0.8926	0.9505	0.0690
MTMR6	0.9387	0.9705	1.0895	0.8034	0.9505	0.0588
PHOX2A	0.9088	0.9787	0.9719	0.9428	0.9505	0.0159
LOC388153	0.9701	0.9286	0.9714	0.9320	0.9505	0.0117
LOC729776	1.0394	1.1912	0.9316	0.6399	0.9505	0.1164
LOC284009	0.9152	1.0278	0.8997	0.9596	0.9505	0.0287
LOC642345	0.9903	0.9995	0.9144	0.8980	0.9505	0.0259
LOC646615	0.8745	1.0222	0.9507	0.9548	0.9505	0.0302
CHCHD9	0.8814	1.1688	1.0913	0.6607	0.9506	0.1141
ZNF777	0.8755	1.3263	0.9251	0.6753	0.9506	0.1364
SLC5A10	0.8602	1.0653	1.0249	0.8518	0.9506	0.0552
ZNF689	0.8025	1.3555	0.9025	0.7418	0.9506	0.1390
C6orf201	0.8717	1.1031	1.0227	0.8046	0.9506	0.0683
MGC20983	0.8891	1.0271	1.0319	0.8542	0.9506	0.0461
LUZP6	0.7043	1.1610	0.9950	0.9421	0.9506	0.0944
LOC285550	0.9217	1.1153	0.9623	0.8031	0.9506	0.0645
LOC1001330	0.8878	0.9796	1.0854	0.8496	0.9506	0.0526
CHRNB1	0.9658	1.0441	0.9986	0.7939	0.9506	0.0546
MC1R	0.9537	1.0636	0.9244	0.8607	0.9506	0.0424
LZTS1	0.8627	1.0968	0.9581	0.8848	0.9506	0.0528
LPCAT4	0.8739	1.0280	0.8846	1.0160	0.9506	0.0413
NPC1	0.6356	1.3927	1.0669	0.7073	0.9506	0.1750
USP32	0.8563	1.1342	0.9477	0.8642	0.9506	0.0646
LOC728975	0.9060	1.0420	0.9594	0.8952	0.9506	0.0335
LOC643900	0.9496	1.0313	1.0567	0.7649	0.9506	0.0660
LOC728340	0.8541	0.9795	1.0362	0.9327	0.9506	0.0385
COPZ2	0.9395	0.9600	0.9487	0.9544	0.9506	0.0044
KIAA1009	0.8472	1.1411	1.0224	0.7918	0.9506	0.0803
LOC645513	0.9677	1.0167	0.9794	0.8388	0.9506	0.0387
METT5D1	0.8615	1.2273	0.9200	0.7938	0.9506	0.0957
ARHGAP18	0.9737	1.0502	0.9099	0.8688	0.9507	0.0396
FLJ36701	0.8919	1.0093	1.0543	0.8472	0.9507	0.0486
LHX4	0.9103	1.0503	1.0445	0.7976	0.9507	0.0604
SCARNA16	0.8479	1.0195	1.0702	0.8651	0.9507	0.0555
LOC647225	0.9994	1.0141	0.8911	0.8981	0.9507	0.0325
POM121L1P	0.8265	1.1547	0.9312	0.8903	0.9507	0.0713
CA7	0.9250	0.9679	1.0053	0.9046	0.9507	0.0225
ATP2B3	0.9392	1.0021	0.9833	0.8782	0.9507	0.0275
LOC644326	0.9719	1.0222	0.9387	0.8699	0.9507	0.0319
FLJ14166	0.8404	1.0995	1.0176	0.8453	0.9507	0.0645
LOC652640	0.8769	0.9704	1.0513	0.9041	0.9507	0.0389
LOC1001329	0.9199	0.9702	0.9018	1.0110	0.9507	0.0248
LOC728089	0.8418	1.0244	0.9632	0.9734	0.9507	0.0387

SYT9	0.9595	1.0183	0.9667	0.8584	0.9507	0.0334
AOC2	0.9620	1.0236	0.9833	0.8339	0.9507	0.0410
LOC221442	0.8039	1.0239	0.9875	0.9876	0.9507	0.0497
LOC1001341	0.8863	0.9978	0.9771	0.9417	0.9507	0.0244
LOC642147	0.8156	0.9897	1.0895	0.9080	0.9507	0.0584
LOC641929	0.9488	1.0051	1.0012	0.8478	0.9507	0.0366
C17orf70	0.8527	1.4579	0.8196	0.6727	0.9507	0.1735
LOC644225	0.8954	0.9258	1.0031	0.9786	0.9507	0.0245
MIR513A2	0.9249	1.0024	0.9539	0.9217	0.9507	0.0187
IFI30	0.9472	1.0511	0.9700	0.8346	0.9507	0.0447
LOC646554	0.8519	0.9750	1.0139	0.9622	0.9507	0.0347
DCLK1	0.9496	0.9867	0.9861	0.8807	0.9507	0.0249
LOC648704	0.8895	1.0157	1.0062	0.8915	0.9507	0.0348
FAM176A	0.9268	1.0458	0.9678	0.8626	0.9508	0.0384
COL7A1	1.0275	1.0146	0.9508	0.8101	0.9508	0.0498
LOC642995	0.8916	1.0394	0.9358	0.9363	0.9508	0.0313
LOC644732	0.8373	1.1138	0.9052	0.9468	0.9508	0.0589
SMC1A	0.9381	0.9892	0.9550	0.9207	0.9508	0.0146
C1orf158	0.9074	1.1151	0.8681	0.9125	0.9508	0.0557
NPAS2	0.9096	1.0926	0.9394	0.8616	0.9508	0.0499
RGMA	1.0184	0.9819	0.8650	0.9378	0.9508	0.0330
LOC647015	1.0113	0.9793	1.0201	0.7924	0.9508	0.0535
RPL36AL	0.7771	1.3521	1.0210	0.6530	0.9508	0.1541
IFIT5	0.8683	1.2022	0.8841	0.8487	0.9508	0.0841
LOC1001302	0.9548	1.0431	0.9672	0.8380	0.9508	0.0424
EIF6	0.8207	1.3575	0.9999	0.6251	0.9508	0.1557
LOC649182	0.8788	1.0371	0.9246	0.9627	0.9508	0.0335
SNORA70B	0.8782	1.0601	1.0038	0.8611	0.9508	0.0484
ZNF192	0.8934	1.1131	0.9913	0.8055	0.9508	0.0661
DNAJC25	0.8525	1.2850	1.0320	0.6338	0.9508	0.1380
LOC652204	0.8679	0.9691	1.0042	0.9621	0.9508	0.0291
P2RY14	0.8823	1.0130	0.9969	0.9110	0.9508	0.0320
KIAA0754	0.9200	0.9701	0.9308	0.9825	0.9508	0.0151
LOC253724	0.9426	0.9990	1.0144	0.8474	0.9508	0.0378
MTMR10	0.8167	1.4471	0.8854	0.6542	0.9508	0.1724
LOC646566	0.9142	1.0538	1.0025	0.8329	0.9509	0.0487
LOC646074	0.9786	1.0691	0.9018	0.8540	0.9509	0.0470
LOC643757	0.8932	0.9929	0.9384	0.9789	0.9509	0.0224
LOC642515	0.9294	1.0408	1.0023	0.8310	0.9509	0.0461
LOC728783	0.9711	1.0046	0.9478	0.8801	0.9509	0.0263
LOC1001343	0.8603	1.0960	0.9334	0.9138	0.9509	0.0508
LOC644994	0.8560	0.9895	1.0350	0.9230	0.9509	0.0391
SNORD42B	0.9069	1.0986	0.9968	0.8012	0.9509	0.0634
CASP5	0.8943	1.0332	1.0053	0.8707	0.9509	0.0402
TMEM90A	0.9742	1.0182	0.9054	0.9058	0.9509	0.0277
BTLA	0.9384	1.1089	0.8760	0.8802	0.9509	0.0546

MIR718	1.0369	0.9559	0.9494	0.8613	0.9509	0.0359
LOC339562	0.8551	1.0160	0.9880	0.9444	0.9509	0.0352
SOX5	0.9974	0.9507	0.9791	0.8764	0.9509	0.0266
CTNND1	0.8626	1.1108	0.9545	0.8757	0.9509	0.0570
ERCC-00057	1.0461	0.9559	0.9861	0.8154	0.9509	0.0489
GJA9	0.9612	1.0677	0.9709	0.8039	0.9509	0.0546
LRRC43	0.9258	1.0588	0.9463	0.8728	0.9509	0.0391
FLJ43879	0.9491	1.0272	0.9362	0.8912	0.9509	0.0283
LOC642450	0.9380	0.9726	1.0444	0.8488	0.9509	0.0406
PGLYRP4	0.8832	1.1599	0.9094	0.8513	0.9509	0.0707
PRKD3	0.8528	1.1873	0.9693	0.7945	0.9510	0.0868
KRT18P26	0.9163	1.0392	0.9608	0.8875	0.9510	0.0331
LOC1001346	0.9705	1.0284	0.8827	0.9222	0.9510	0.0314
LATS1	0.9231	1.0377	0.9451	0.8978	0.9510	0.0305
SCN1B	0.9195	1.0508	0.9938	0.8397	0.9510	0.0458
LOC644003	0.8973	1.0274	1.0027	0.8764	0.9510	0.0376
LOC652880	0.9489	0.9693	0.9770	0.9087	0.9510	0.0153
TTTY17A	0.8405	1.0545	1.0039	0.9050	0.9510	0.0482
CD247	0.8646	1.0280	1.0242	0.8872	0.9510	0.0436
LOC647468	0.9165	0.9627	1.0589	0.8658	0.9510	0.0411
FBXW8	0.9386	1.0641	0.9367	0.8646	0.9510	0.0415
BPIL1	0.8676	1.0020	0.9921	0.9424	0.9510	0.0307
LOC642505	0.9114	0.9956	0.9956	0.9015	0.9510	0.0258
USP45	0.9167	1.0518	0.9599	0.8758	0.9510	0.0377
LOC653353	0.8906	0.9819	1.0669	0.8647	0.9510	0.0461
MYO15B	0.8819	0.9893	0.9533	0.9797	0.9510	0.0243
RNU6ATAC	0.7468	1.1994	1.0485	0.8095	0.9510	0.1052
LOC650323	0.9941	0.9489	0.9114	0.9498	0.9511	0.0169
CDC20B	1.0433	1.0161	0.8664	0.8785	0.9511	0.0458
GDPD2	0.8694	1.0993	0.9216	0.9142	0.9511	0.0507
LOC643995	0.8166	1.0869	1.0022	0.8987	0.9511	0.0591
LOC439945	0.9780	1.0195	0.9383	0.8686	0.9511	0.0321
HECW2	0.8848	1.1246	0.8902	0.9050	0.9512	0.0580
LOC653216	0.9149	1.0433	1.0122	0.8342	0.9512	0.0476
KIAA1026	0.8682	1.0466	0.9991	0.8908	0.9512	0.0428
OR8B12	1.0224	1.0857	0.9038	0.7928	0.9512	0.0649
SLAMF9	0.8417	1.1605	0.9118	0.8907	0.9512	0.0713
GOLIM4	0.7993	1.2519	0.9127	0.8408	0.9512	0.1030
CYB5R4	0.7954	1.2473	1.0381	0.7239	0.9512	0.1194
LMAN2	0.7563	1.2420	0.8617	0.9447	0.9512	0.1043
PAQR6	0.9791	0.9903	0.9773	0.8581	0.9512	0.0312
LOC339902	0.8865	0.9980	0.9552	0.9651	0.9512	0.0234
LOC649683	0.9228	1.0592	0.9617	0.8611	0.9512	0.0415
LOC646782	0.9081	1.0597	0.9921	0.8449	0.9512	0.0471
ATP6V1E2	0.9449	1.1812	0.9608	0.7180	0.9512	0.0946
C10orf50	0.8641	1.0211	0.9958	0.9239	0.9512	0.0356

LOC730964	0.8810	1.0358	0.9589	0.9292	0.9512	0.0324
LOC645210	0.9891	1.0084	0.9010	0.9064	0.9512	0.0277
POLR2B	0.7373	1.2563	1.0872	0.7242	0.9512	0.1319
RNF169	0.8242	1.2277	0.7460	1.0071	0.9512	0.1072
SNORD73A	0.9629	1.0647	0.9226	0.8548	0.9513	0.0439
GPT	0.8939	1.1082	0.9942	0.8087	0.9513	0.0646
LOC1001310	0.8568	1.0227	0.9941	0.9315	0.9513	0.0368
SPEN	0.7905	1.3697	0.9457	0.6992	0.9513	0.1485
LOC651167	0.8316	1.0419	1.0576	0.8739	0.9513	0.0576
TLR9	0.8269	1.0580	1.0273	0.8930	0.9513	0.0548
LOC1001323	0.6013	1.0505	1.1240	1.0293	0.9513	0.1184
PTPRN2	1.0189	0.9367	0.9532	0.8963	0.9513	0.0255
MIR587	0.9515	0.9110	0.9838	0.9590	0.9513	0.0151
C20orf173	0.9626	1.0714	0.9301	0.8411	0.9513	0.0475
MIR7-3	0.8999	1.0389	0.9849	0.8815	0.9513	0.0369
TNKS	0.8735	1.0787	0.9180	0.9350	0.9513	0.0444
GPA33	0.8759	1.0132	1.0390	0.8772	0.9513	0.0435
SYVN1	0.8876	1.3039	0.8862	0.7276	0.9513	0.1234
LOC653424	0.8506	1.0653	0.9579	0.9315	0.9513	0.0443
LOC646085	0.9505	0.9476	0.9429	0.9643	0.9513	0.0046
LOC728519	0.8530	1.2383	0.9086	0.8055	0.9513	0.0979
GOLGA7B	0.8821	1.0510	1.0247	0.8475	0.9513	0.0507
ZNF335	0.9593	1.0998	0.9242	0.8221	0.9513	0.0574
SCGN	0.8802	1.0583	1.0108	0.8560	0.9513	0.0493
OTP	0.8596	1.0390	0.9970	0.9098	0.9513	0.0407
LOC646908	0.8684	1.1360	0.9884	0.8126	0.9514	0.0717
SNORA78	0.8445	1.0405	0.9106	1.0098	0.9514	0.0451
MIR648	0.8491	1.0206	0.9998	0.9361	0.9514	0.0385
TMEM16C	0.8358	1.0410	0.9947	0.9340	0.9514	0.0443
MAGOHB	0.9412	0.9219	0.9709	0.9715	0.9514	0.0121
ACAP1	0.8755	1.0298	0.9428	0.9575	0.9514	0.0317
HLA-G	0.9367	1.0393	1.0126	0.8171	0.9514	0.0498
LOC391771	0.9356	0.9877	0.9746	0.9077	0.9514	0.0183
ZFP92	0.8610	1.0493	0.9947	0.9006	0.9514	0.0430
A26C3	0.9143	1.0932	1.0013	0.7970	0.9514	0.0631
LOC648874	0.8779	1.1333	0.9237	0.8708	0.9514	0.0617
ZNF3	0.8780	1.1624	0.9632	0.8020	0.9514	0.0777
LOC442366	0.9074	1.1038	0.9511	0.8433	0.9514	0.0554
C1orf118	0.8401	1.0389	0.9980	0.9287	0.9514	0.0435
PCDHGA1	0.9090	1.0363	0.9286	0.9318	0.9514	0.0288
RPIA	0.8378	1.0944	1.1345	0.7391	0.9514	0.0966
LOC728417	0.8521	1.1268	0.9685	0.8584	0.9514	0.0643
ZC3H15	0.9246	1.1716	1.1005	0.6091	0.9514	0.1254
ZNF658	0.8737	1.0926	0.9917	0.8478	0.9515	0.0565
LOC647003	0.9619	1.0339	0.9468	0.8632	0.9515	0.0350
GNAI1	0.9563	1.0936	0.9208	0.8352	0.9515	0.0538

LOC729319	0.9135	0.9488	1.0165	0.9271	0.9515	0.0229
LOC1001282	0.9095	1.0819	0.8435	0.9710	0.9515	0.0507
LOC644319	0.9054	1.0273	1.0128	0.8605	0.9515	0.0407
CPNE9	0.9550	1.0406	0.9943	0.8160	0.9515	0.0484
RHBDL3	0.9268	0.9908	0.9575	0.9309	0.9515	0.0148
RABGGTA	0.9188	1.2363	0.8655	0.7853	0.9515	0.0988
LOC644288	0.8817	0.9512	1.0134	0.9597	0.9515	0.0271
KLF8	0.9246	1.0777	0.9601	0.8436	0.9515	0.0486
ACTRT2	0.9382	0.8966	1.0273	0.9439	0.9515	0.0274
PLEK2	0.9304	1.0417	1.0064	0.8275	0.9515	0.0474
CD177	0.8821	1.0319	0.9838	0.9084	0.9515	0.0344
LOC392262	0.8603	1.0924	0.9457	0.9078	0.9515	0.0501
IQCG	0.8469	1.1379	0.9834	0.8380	0.9515	0.0705
MAB21L1	0.8332	1.0812	1.0344	0.8573	0.9515	0.0623
C20orf133	0.9282	0.9972	0.9918	0.8890	0.9515	0.0261
AKAP3	0.8902	1.0281	1.0003	0.8875	0.9515	0.0366
CMTM1	0.9330	1.0087	0.9528	0.9116	0.9515	0.0208
LOC1001348	0.8643	1.0676	1.0154	0.8588	0.9515	0.0530
GABRA5	0.8764	1.0651	1.0198	0.8449	0.9515	0.0537
CASQ1	0.8736	1.1319	0.9808	0.8199	0.9515	0.0688
LOC642938	0.9184	1.0622	0.9402	0.8855	0.9516	0.0385
LOC642761	0.8432	1.0835	0.9906	0.8889	0.9516	0.0537
ATXN1	0.9117	1.1025	1.0191	0.7729	0.9516	0.0712
LOC284276	0.9445	1.0698	0.8714	0.9206	0.9516	0.0422
TAS2R60	0.8650	1.0290	1.0353	0.8769	0.9516	0.0466
LOC729396	0.9305	0.9946	1.0194	0.8618	0.9516	0.0353
CASC2	0.8309	1.0988	1.0247	0.8518	0.9516	0.0655
LOC1001290	0.9638	0.9557	1.0221	0.8646	0.9516	0.0325
LOC645476	0.8751	1.0515	0.9894	0.8903	0.9516	0.0419
COL21A1	0.9245	0.9682	1.0850	0.8287	0.9516	0.0532
HOXC11	0.9246	1.0296	0.9499	0.9023	0.9516	0.0278
LOC1001332	0.9264	0.9691	1.0047	0.9061	0.9516	0.0220
NPNT	0.9499	1.1142	1.0142	0.7281	0.9516	0.0818
ERCC-00040	0.8446	1.0040	1.1336	0.8243	0.9516	0.0728
CATSPER4	0.9110	1.1569	0.9058	0.8328	0.9516	0.0707
LOC1001290	0.8403	1.1625	0.9592	0.8445	0.9516	0.0755
LOC154822	0.8584	1.0316	0.9930	0.9235	0.9516	0.0383
LOC442423	0.8694	1.1404	0.9507	0.8461	0.9516	0.0668
ZNF137	0.8285	1.0928	1.0349	0.8503	0.9516	0.0660
RFFL	0.9186	1.1809	0.9283	0.7788	0.9516	0.0837
LOC644800	0.8715	0.9534	1.0221	0.9596	0.9516	0.0309
FCER1A	0.9851	0.9643	1.1096	0.7476	0.9517	0.0752
SHOX	0.8873	1.0289	0.9555	0.9349	0.9517	0.0294
TTTY4B	0.8952	1.0198	0.9479	0.9438	0.9517	0.0257
LOC374973	0.8316	1.1115	0.9490	0.9146	0.9517	0.0587
LOC391045	0.8223	1.1213	0.9470	0.9161	0.9517	0.0625

FAIM2	0.9145	1.0859	0.9627	0.8437	0.9517	0.0510
LOC1001285	0.8324	1.0164	0.9950	0.9630	0.9517	0.0412
LOC648200	0.8657	1.0642	1.0158	0.8611	0.9517	0.0519
FGF17	0.8834	1.0549	1.0014	0.8669	0.9517	0.0456
LOC728664	0.7878	1.0827	1.0395	0.8968	0.9517	0.0676
FLJ44076	0.9064	1.0899	0.9318	0.8786	0.9517	0.0473
COL25A1	0.9083	1.0547	0.9730	0.8708	0.9517	0.0403
LOC650257	0.9310	1.0505	0.9364	0.8888	0.9517	0.0346
KLHL4	0.9380	0.9744	0.9776	0.9169	0.9517	0.0147
KIF18B	0.8587	1.0445	1.0259	0.8778	0.9517	0.0485
SPAG4	1.0326	1.0767	0.8884	0.8091	0.9517	0.0623
OR13D1	0.9057	1.0595	0.9354	0.9062	0.9517	0.0366
DCAF12L2	0.9991	1.0655	0.9631	0.7792	0.9517	0.0613
LOC389120	0.9537	0.9403	1.0423	0.8706	0.9517	0.0353
RAB3GAP1	0.8794	1.2094	0.9696	0.7485	0.9517	0.0971
LOC1001298	0.8895	1.0792	0.9690	0.8693	0.9517	0.0476
MIR181A2	0.9268	1.0465	0.9802	0.8534	0.9517	0.0409
LOC440277	1.0002	0.8740	1.0675	0.8653	0.9517	0.0494
GIMAP6	0.9516	1.0206	0.9836	0.8512	0.9517	0.0364
CXorf30	0.9502	1.0314	0.9454	0.8800	0.9517	0.0310
ACCN2	0.8569	1.1505	0.9554	0.8442	0.9517	0.0708
MIR545	0.9382	0.9971	1.0032	0.8684	0.9517	0.0314
LOC641787	0.9558	0.9812	0.9189	0.9511	0.9518	0.0128
SDC4P	0.9000	1.0713	0.9386	0.8972	0.9518	0.0409
SNORD123	0.9556	1.0409	0.9565	0.8540	0.9518	0.0382
LOC648283	0.9355	1.1350	0.9146	0.8221	0.9518	0.0659
DERL3	0.9270	1.0767	1.0046	0.7989	0.9518	0.0594
LOC731682	0.9559	1.0135	0.9410	0.8967	0.9518	0.0241
SUPT5H	0.9520	1.2035	0.8824	0.7693	0.9518	0.0920
GPR158L1	0.8465	1.0270	1.0340	0.8996	0.9518	0.0467
LOC1001284	0.8875	0.9774	1.0742	0.8681	0.9518	0.0472
FAM41AY2	1.0100	0.9967	0.9794	0.8210	0.9518	0.0441
FAM160B2	0.8956	1.1346	1.0446	0.7324	0.9518	0.0882
LOC649214	0.9291	0.8895	1.0804	0.9083	0.9518	0.0436
AMAC1L3	1.0000	1.0160	0.9277	0.8636	0.9518	0.0351
LOC390564	0.9347	1.0736	0.9966	0.8024	0.9518	0.0573
PCDHGA2	0.8878	1.0321	0.9895	0.8980	0.9518	0.0352
LOC1001328	0.8487	1.0662	1.0394	0.8529	0.9518	0.0586
LOC391334	0.9119	1.0844	1.0103	0.8007	0.9518	0.0615
UBTF	0.9527	1.1131	0.9671	0.7744	0.9518	0.0694
LOC1001279	0.9210	0.9302	1.0490	0.9073	0.9519	0.0327
LOC400836	0.8599	1.0494	1.0765	0.8216	0.9519	0.0649
LOC728162	0.9737	0.9559	1.0467	0.8311	0.9519	0.0448
SPATA19	0.9104	1.0024	0.9200	0.9746	0.9519	0.0220
LOC730379	0.9576	1.0509	0.8827	0.9163	0.9519	0.0364
LOC647949	1.0235	0.9648	0.9690	0.8503	0.9519	0.0364

ATP8B4	0.8987	1.2420	0.9135	0.7533	0.9519	0.1032
LOC392481	0.8869	1.0251	0.9982	0.8974	0.9519	0.0350
PRPS1	0.8948	1.2740	1.0203	0.6186	0.9519	0.1363
RAB2B	0.8438	1.2217	0.9569	0.7852	0.9519	0.0967
C9orf144	0.9194	0.9440	1.0309	0.9134	0.9519	0.0271
LOC1001311	0.9118	1.0572	0.8560	0.9827	0.9519	0.0436
TCF1	0.8653	1.0051	0.9328	1.0045	0.9519	0.0335
MS4A14	0.8685	1.0924	0.9598	0.8869	0.9519	0.0508
IL20RB	0.9155	1.0925	0.9133	0.8863	0.9519	0.0473
LOC643897	0.8089	1.0744	1.0062	0.9182	0.9519	0.0574
LOC1001285	0.7274	1.3901	1.0285	0.6618	0.9519	0.1664
TMEM103	0.8732	0.9771	1.0099	0.9476	0.9519	0.0292
FLJ22662	0.9629	1.0743	0.9312	0.8393	0.9519	0.0485
TM7SF4	0.9406	1.0176	0.9942	0.8553	0.9519	0.0360
LOC654252	0.9196	1.0010	0.9767	0.9104	0.9519	0.0220
LOC647451	0.8917	1.0779	1.0087	0.8294	0.9519	0.0561
UVRAG	0.8542	1.2434	0.9388	0.7713	0.9519	0.1030
LOC284948	0.8398	1.0003	0.9366	1.0311	0.9520	0.0423
RALGPS1	0.8732	1.3144	0.8718	0.7485	0.9520	0.1243
GPR82	0.9278	1.0425	1.0220	0.8156	0.9520	0.0519
DSCR9	0.8977	1.0191	1.0368	0.8543	0.9520	0.0449
LOC352909	0.9000	1.0631	0.9628	0.8821	0.9520	0.0409
DHRS7C	0.8958	1.0678	1.0167	0.8276	0.9520	0.0549
KRTAP9-8	0.8827	1.0938	1.0060	0.8254	0.9520	0.0605
LOC730060	0.9202	0.9610	1.0618	0.8649	0.9520	0.0416
KATNAL1	0.9062	1.0882	0.9559	0.8578	0.9520	0.0496
OR3A2	0.9676	1.0080	0.8955	0.9370	0.9520	0.0238
MDFIC	0.8644	1.0627	0.9786	0.9023	0.9520	0.0439
SLC9A11	0.9594	0.9546	0.9837	0.9103	0.9520	0.0153
LOC731486	1.0041	0.9729	0.9657	0.8655	0.9520	0.0300
GRIA3	0.8851	1.0745	0.9789	0.8697	0.9520	0.0474
LOC648069	0.8614	1.1218	0.9414	0.8834	0.9520	0.0591
RBM19	0.9183	1.1035	0.8895	0.8968	0.9520	0.0509
ALKBH3	0.9467	1.2434	0.9447	0.6734	0.9520	0.1164
JOSD2	0.8626	1.1220	0.9609	0.8626	0.9520	0.0612
SALL3	0.9183	1.0157	0.9745	0.8997	0.9520	0.0265
FAM100B	0.9079	1.0811	0.8959	0.9232	0.9520	0.0434
LOC442446	0.8660	0.9780	1.0143	0.9500	0.9520	0.0316
UGP2	0.8664	1.2645	0.9226	0.7548	0.9521	0.1098
ARID3C	0.9221	1.0737	0.9846	0.8279	0.9521	0.0518
FOXH1	0.8367	1.1358	0.9986	0.8370	0.9521	0.0722
CHRNG	0.8809	1.0052	1.0236	0.8985	0.9521	0.0364
FLJ41733	0.8978	1.0133	0.9650	0.9322	0.9521	0.0246
SELE	0.9161	1.0710	0.9622	0.8591	0.9521	0.0449
LOC1001330	0.9545	1.0395	0.9098	0.9044	0.9521	0.0312
LCTL	0.7983	1.1033	0.9753	0.9314	0.9521	0.0629

LOC643686	0.7752	1.1018	1.1107	0.8206	0.9521	0.0895
LOC642118	0.9784	1.0588	0.8691	0.9020	0.9521	0.0423
LOC1001322	0.8910	1.0955	0.9675	0.8543	0.9521	0.0533
LOC389830	0.9066	1.0212	0.9851	0.8955	0.9521	0.0305
BCAS3	0.8831	1.0082	0.9500	0.9671	0.9521	0.0260
ACSF2	0.9917	1.0526	1.0191	0.7449	0.9521	0.0702
LOC644267	0.9320	1.0352	0.9854	0.8557	0.9521	0.0384
SERPINA11	0.9853	0.9719	0.9538	0.8974	0.9521	0.0193
LOC646377	0.8626	1.1196	0.9274	0.8988	0.9521	0.0574
C13orf30	0.9102	0.9723	1.0044	0.9215	0.9521	0.0221
DKK3	0.9180	1.0329	0.9810	0.8766	0.9521	0.0344
THRA	0.8318	1.1163	0.9821	0.8783	0.9521	0.0631
CSNK1A1P	0.9106	0.9727	1.0279	0.8973	0.9521	0.0301
LOC1001327	0.9040	1.0173	1.0080	0.8792	0.9521	0.0354
LOC1001337	0.9941	1.0613	1.0028	0.7504	0.9521	0.0689
VTCN1	0.9085	1.0515	0.9168	0.9318	0.9521	0.0335
LOC1001300	0.9399	1.0761	0.8894	0.9031	0.9522	0.0427
METTL2B	0.8433	1.0726	0.9757	0.9171	0.9522	0.0484
RIN3	0.9042	1.0104	0.9577	0.9365	0.9522	0.0223
C20orf94	0.7893	1.0993	1.1114	0.8087	0.9522	0.0886
MIER3	0.7458	1.1901	0.9964	0.8765	0.9522	0.0944
NT5E	1.0053	0.9234	0.9388	0.9413	0.9522	0.0181
AMZ2	0.7519	1.3739	0.9697	0.7133	0.9522	0.1515
LY6H	0.8720	1.1013	0.9512	0.8845	0.9522	0.0526
TTTY5	0.8975	1.0544	0.9067	0.9502	0.9522	0.0360
H3F3A	0.8519	1.2860	0.9910	0.6799	0.9522	0.1282
ZNF710	0.9122	1.0198	0.9758	0.9010	0.9522	0.0279
SHF	0.8784	1.0978	0.9713	0.8613	0.9522	0.0542
UNQ1940	0.9653	1.0930	0.8894	0.8613	0.9522	0.0518
C6orf130	0.8680	1.3616	0.8278	0.7515	0.9522	0.1386
LOC644744	0.9314	1.0494	0.8849	0.9433	0.9522	0.0347
LOC440031	0.9702	1.0826	0.9480	0.8081	0.9522	0.0564
ANKRD30B	0.7657	1.3425	0.9937	0.7071	0.9522	0.1440
SNORD45B	0.9402	0.9793	0.9652	0.9244	0.9522	0.0123
PDE2A	0.9685	0.9651	1.0160	0.8594	0.9523	0.0331
CCNT2	0.7773	1.2052	0.9752	0.8514	0.9523	0.0937
C2orf19	0.8900	0.9680	1.1084	0.8427	0.9523	0.0581
LOC286411	0.9118	1.0674	0.9810	0.8489	0.9523	0.0469
LOC651894	0.7568	1.1768	1.1068	0.7687	0.9523	0.1104
KIF17	0.9313	1.0512	0.9562	0.8705	0.9523	0.0376
UPK3B	0.8906	1.0640	0.9765	0.8780	0.9523	0.0432
KLHL11	0.8033	1.0337	1.0429	0.9293	0.9523	0.0559
LOC653816	0.9288	0.9993	1.0005	0.8806	0.9523	0.0292
ZNF174	0.9456	1.1409	0.9728	0.7499	0.9523	0.0801
PDDC1	0.9001	1.1491	0.9346	0.8253	0.9523	0.0695
LOC651919	0.8290	0.9937	1.0338	0.9527	0.9523	0.0443

TAS2R41	1.0032	0.9217	1.0493	0.8350	0.9523	0.0472
MIR548J	0.9668	0.9784	0.9797	0.8844	0.9523	0.0228
ME3	0.8967	1.0295	1.0175	0.8656	0.9523	0.0416
MSL3L2	0.8382	0.9847	1.0311	0.9553	0.9523	0.0411
LOC644075	0.8891	1.0443	1.0723	0.8035	0.9523	0.0639
ODC1	0.7933	1.4237	0.9253	0.6670	0.9523	0.1657
C22orf37	0.9470	1.0498	0.9557	0.8569	0.9523	0.0394
MESDC2	0.9122	1.0892	0.9529	0.8550	0.9523	0.0498
LOC1001311	0.7919	1.2669	0.9966	0.7539	0.9523	0.1176
LOC652737	0.9534	0.9918	1.0035	0.8607	0.9523	0.0324
C6orf123	0.9308	0.9988	0.9935	0.8862	0.9523	0.0269
CDS1	0.7215	1.2908	1.0982	0.6989	0.9523	0.1453
LOC1001286	0.9619	0.9692	0.9814	0.8969	0.9524	0.0189
LOC1001340	0.9328	1.0427	0.9560	0.8780	0.9524	0.0343
SNORD113-3	0.8599	1.0670	1.0234	0.8592	0.9524	0.0543
LOC643626	0.9382	0.9448	0.9700	0.9565	0.9524	0.0070
AFAR3	0.9508	0.9648	1.0087	0.8853	0.9524	0.0255
LOC729157	0.8351	1.2005	0.9208	0.8531	0.9524	0.0847
SOX18	0.8858	1.0958	1.0008	0.8272	0.9524	0.0599
LOC644925	0.8234	1.1397	0.9716	0.8749	0.9524	0.0696
LOC1001335	0.8574	1.0508	1.0558	0.8456	0.9524	0.0583
MYLK	0.9316	1.0604	0.9457	0.8719	0.9524	0.0394
CD4	0.9563	1.0306	0.9093	0.9133	0.9524	0.0282
GCC2	0.8329	1.0837	1.0347	0.8583	0.9524	0.0627
LOC650620	0.9117	1.1194	0.9505	0.8282	0.9524	0.0612
LOC652876	0.9778	1.1008	0.8867	0.8443	0.9524	0.0568
OR4F3	0.9581	0.9869	0.9762	0.8885	0.9524	0.0221
LOC642460	0.9496	0.9464	0.9964	0.9174	0.9524	0.0163
LOC729020	0.9275	1.0262	0.9778	0.8782	0.9524	0.0319
ADCY7	0.7759	1.2868	0.9244	0.8226	0.9524	0.1157
TSPY1	0.9183	1.0625	0.9331	0.8959	0.9524	0.0375
PRDM2	0.9073	1.0161	1.0350	0.8514	0.9524	0.0439
AIFM1	0.8766	1.3399	0.9491	0.6441	0.9524	0.1446
LOC91461	0.9139	0.9203	0.9646	1.0110	0.9525	0.0225
LOC644963	0.9536	0.9763	0.9968	0.8832	0.9525	0.0247
C1orf55	0.8238	1.2983	0.9805	0.7072	0.9525	0.1282
KRTAP12-3	0.8710	1.0571	1.0138	0.8679	0.9525	0.0487
TRIM3	0.9163	1.0415	0.9712	0.8809	0.9525	0.0350
KRT18P19	0.9548	0.9504	0.9681	0.9365	0.9525	0.0065
LOC441996	0.8725	1.1447	0.9619	0.8308	0.9525	0.0697
LOC1001279	0.9202	0.9944	1.0277	0.8677	0.9525	0.0361
ADAM18	0.9217	1.0583	0.9768	0.8532	0.9525	0.0434
ACOX1	0.8498	1.2060	0.9899	0.7644	0.9525	0.0964
DNCL1	0.7453	1.1876	1.0978	0.7793	0.9525	0.1115
LOC645812	0.7966	1.1196	0.9946	0.8991	0.9525	0.0688
LOC647704	0.9592	1.0722	0.9558	0.8229	0.9525	0.0510

LOC646731	0.9586	0.9853	1.0877	0.7785	0.9525	0.0643
C14orf125	0.7857	1.0602	1.0284	0.9359	0.9525	0.0615
SPATA4	0.7905	1.1787	0.9852	0.8558	0.9525	0.0855
LOC1001335	0.8773	1.0160	1.0046	0.9122	0.9525	0.0342
LOC642161	0.8197	0.9877	1.0608	0.9420	0.9525	0.0506
RPS19BP1	0.8234	1.3186	0.9415	0.7268	0.9526	0.1297
HSPA6	0.9797	1.0747	0.9704	0.7855	0.9526	0.0605
LOC730159	0.9633	1.0730	0.9017	0.8723	0.9526	0.0444
BAGE3	0.9258	1.0110	0.9895	0.8840	0.9526	0.0292
LOC1001307	0.9004	0.9974	1.0017	0.9107	0.9526	0.0272
LOC440059	0.8864	1.0667	0.9475	0.9097	0.9526	0.0401
LOC643563	0.8837	1.0742	1.0092	0.8432	0.9526	0.0538
LOC651868	0.9250	1.0053	0.9760	0.9040	0.9526	0.0232
EYA4	0.9041	1.0003	1.0369	0.8691	0.9526	0.0395
LOC643699	0.8982	1.1609	0.8786	0.8728	0.9526	0.0696
LOC647707	0.8935	1.0647	1.0331	0.8191	0.9526	0.0580
FAM22B	0.8956	1.0286	1.0252	0.8611	0.9526	0.0435
LOC1001286	0.9592	1.0104	0.9156	0.9252	0.9526	0.0214
LOC645882	0.8805	1.1347	0.9634	0.8318	0.9526	0.0665
UBE4A	0.7344	1.1761	1.1708	0.7291	0.9526	0.1275
MOBKL1B	0.8505	1.2101	1.0307	0.7193	0.9526	0.1069
LOC641977	0.8524	0.9433	1.0370	0.9778	0.9526	0.0386
LOC649593	0.9196	0.9862	1.0048	0.9000	0.9526	0.0253
LOC642563	0.8347	1.1161	0.9689	0.8910	0.9527	0.0610
PRAMEF8	0.9005	1.1033	0.9835	0.8233	0.9527	0.0599
LOC1001011	0.8530	1.1211	0.9765	0.8600	0.9527	0.0629
TRIML2	0.9358	0.9672	1.0047	0.9030	0.9527	0.0218
LOC645339	0.9071	1.0640	0.9684	0.8712	0.9527	0.0422
PPP1R1A	0.8193	1.0795	1.0028	0.9091	0.9527	0.0565
CYP3A43	0.9253	0.9512	1.0217	0.9125	0.9527	0.0244
DERPC	0.8350	1.2844	0.9467	0.7447	0.9527	0.1180
OR9K2	0.9654	0.9884	0.9631	0.8939	0.9527	0.0204
GRB7	0.9179	1.0798	0.9521	0.8611	0.9527	0.0463
P704P	0.9455	0.9357	1.0138	0.9158	0.9527	0.0213
MMP9	0.8717	1.0126	1.0430	0.8835	0.9527	0.0439
ZNF619	0.8315	0.9887	1.0380	0.9525	0.9527	0.0440
LOC644819	0.8952	1.0330	0.9514	0.9312	0.9527	0.0292
LOC1001321	0.8560	1.0708	1.0164	0.8677	0.9527	0.0537
LTA4H	0.7799	1.4073	0.9466	0.6770	0.9527	0.1614
FLJ10213	0.9793	1.1003	0.9072	0.8240	0.9527	0.0585
ANKRD34A	0.7732	0.9372	1.2823	0.8181	0.9527	0.1152
GPR133	0.8503	0.9797	1.0728	0.9081	0.9527	0.0480
CDX2	0.8613	1.1237	0.8886	0.9374	0.9527	0.0591
LOC647591	0.9509	1.0023	1.0033	0.8544	0.9527	0.0350
FLJ40288	0.8141	1.0423	1.0440	0.9106	0.9527	0.0558
DOT1L	0.9422	1.1334	0.9834	0.7519	0.9527	0.0785

SNORD38B	0.8519	1.0378	1.0630	0.8582	0.9527	0.0566
MIR1277	0.8865	1.0454	1.0142	0.8649	0.9527	0.0452
LOC648470	0.9568	1.0747	0.9793	0.8002	0.9527	0.0569
C9orf102	0.9450	1.1466	0.9540	0.7654	0.9527	0.0778
LOC1001303	0.9358	0.8962	1.0192	0.9599	0.9528	0.0258
OR2A14	0.8844	1.0477	1.0475	0.8315	0.9528	0.0558
ZNF268	0.8997	1.1019	0.9711	0.8383	0.9528	0.0566
LOC1001323	0.9190	1.1339	0.9124	0.8458	0.9528	0.0626
KIF21B	0.9852	1.0403	0.9512	0.8343	0.9528	0.0435
LOC1001320	0.9674	0.9273	0.9960	0.9205	0.9528	0.0177
LOC402100	0.9809	0.9720	0.9477	0.9106	0.9528	0.0157
MDM2	0.8170	1.1010	1.0056	0.8876	0.9528	0.0629
LOC643951	0.9922	0.9464	0.9755	0.8970	0.9528	0.0209
RANBP9	0.8393	1.0865	0.9895	0.8958	0.9528	0.0543
NOTUM	0.9532	1.0849	0.8764	0.8967	0.9528	0.0469
LRAT	1.0249	0.9626	0.9947	0.8289	0.9528	0.0432
LOC653937	0.8206	1.1268	0.9715	0.8922	0.9528	0.0657
LOC150383	0.8442	1.0973	0.9737	0.8961	0.9528	0.0550
LOC646123	0.8569	1.0106	1.0684	0.8753	0.9528	0.0516
PTPN7	0.9210	0.9803	1.0386	0.8712	0.9528	0.0363
LOC647778	0.8860	1.2015	0.8939	0.8299	0.9528	0.0841
FGF21	0.8708	1.1326	1.0373	0.7706	0.9528	0.0813
OR51V1	1.0320	0.9527	0.9195	0.9072	0.9528	0.0281
LOC730036	0.8999	1.0235	0.9626	0.9253	0.9528	0.0269
FLJ37201	0.8641	1.1041	0.9697	0.8734	0.9528	0.0558
FOLH1B	0.9450	1.0678	0.9416	0.8569	0.9528	0.0434
LOC1001294	0.8985	1.0303	1.0112	0.8713	0.9528	0.0398
LOC391282	0.9620	1.0104	1.0131	0.8258	0.9528	0.0439
FYB	0.8671	1.0545	0.9623	0.9275	0.9528	0.0392
LOC1001284	0.9119	1.0628	1.0136	0.8232	0.9529	0.0534
LOC651991	0.7784	1.1171	0.9925	0.9234	0.9529	0.0706
LOC646835	0.8944	1.0707	0.9062	0.9401	0.9529	0.0405
KRT10	0.9834	1.2761	0.8889	0.6630	0.9529	0.1270
RNF32	0.8763	1.0422	1.0097	0.8833	0.9529	0.0427
LOC651520	0.9518	0.9806	1.0011	0.8780	0.9529	0.0269
ANK1	0.8791	1.0706	0.9834	0.8784	0.9529	0.0464
LIG3	0.8713	1.2192	0.8617	0.8593	0.9529	0.0888
WBP4	0.7582	1.2348	0.9417	0.8768	0.9529	0.1014
LOC1001291	0.9248	1.0384	0.9943	0.8541	0.9529	0.0404
RTN4RL1	1.0458	1.0168	0.9059	0.8431	0.9529	0.0474
GABPB2	0.7909	1.3695	0.8832	0.7679	0.9529	0.1411
SIGLEC8	0.8885	1.1145	0.9262	0.8823	0.9529	0.0547
FLJ40296	0.9574	1.0072	0.9784	0.8687	0.9529	0.0299
LOC126536	1.0166	1.0217	0.8747	0.8987	0.9529	0.0386
LOC730419	0.9536	0.9803	1.0227	0.8550	0.9529	0.0356
LOC644628	0.9316	0.9397	0.9805	0.9598	0.9529	0.0110

MPST	0.8683	1.3099	0.8864	0.7471	0.9529	0.1229
F13A1	0.8791	1.0402	0.9902	0.9022	0.9529	0.0377
FAM181B	0.9558	1.0376	0.9201	0.8982	0.9529	0.0306
CIDECP	0.9699	0.9953	0.9801	0.8663	0.9529	0.0293
LOC727735	0.8602	1.0577	0.9900	0.9039	0.9529	0.0441
ZNF488	0.8787	1.0934	0.9295	0.9102	0.9529	0.0480
ACSM4	0.8826	1.0745	0.9776	0.8770	0.9529	0.0466
LOC649397	0.9298	1.1185	0.8850	0.8784	0.9529	0.0564
LOC1001314	0.8024	1.0726	0.9996	0.9372	0.9529	0.0573
LOC1001305	0.6914	1.2186	1.0367	0.8651	0.9530	0.1132
PAX1	0.9542	1.1102	0.8549	0.8926	0.9530	0.0563
LOC651213	0.9264	1.0866	0.9398	0.8591	0.9530	0.0479
FAM78B	0.8637	1.0223	1.0368	0.8890	0.9530	0.0446
LOC641772	0.9115	1.0458	0.9913	0.8633	0.9530	0.0407
CEP250	0.9051	1.1070	0.9619	0.8379	0.9530	0.0572
BET3L	1.0234	1.0404	0.9079	0.8403	0.9530	0.0477
LOX	0.9522	1.0694	0.8804	0.9100	0.9530	0.0415
LOC730099	0.8598	1.1588	0.9202	0.8733	0.9530	0.0698
C17orf65	0.8930	1.0675	1.0183	0.8332	0.9530	0.0543
MYO3B	0.8201	0.9957	1.1787	0.8175	0.9530	0.0860
LOC648044	0.8950	1.0360	1.0372	0.8437	0.9530	0.0494
TREML4	0.9355	0.9716	0.9699	0.9351	0.9530	0.0102
LOC651289	0.9497	0.9636	1.0273	0.8715	0.9530	0.0320
LOC1001330	0.9069	1.0312	1.0354	0.8387	0.9530	0.0484
LOC645804	0.8699	0.9970	1.0446	0.9005	0.9530	0.0408
LOC642864	0.8702	1.0667	1.0606	0.8146	0.9530	0.0649
CEACAM4	1.0032	1.0534	0.9833	0.7722	0.9530	0.0621
LOC391209	0.9481	1.0123	0.9592	0.8925	0.9530	0.0246
PAPOLG	0.8963	1.0060	1.0310	0.8789	0.9530	0.0383
AP1S3	0.8646	1.0987	0.9710	0.8778	0.9530	0.0540
SSX1	0.9490	1.0585	0.9055	0.8991	0.9530	0.0369
MYCT1	0.9278	0.9842	1.0314	0.8687	0.9530	0.0352
MGC33948	0.7471	1.1522	0.9889	0.9240	0.9530	0.0838
UNCX	0.9289	1.0841	0.9224	0.8768	0.9530	0.0452
SNORD87	1.0146	0.9501	0.9014	0.9461	0.9530	0.0233
LOC730029	0.8999	1.2453	1.0304	0.6367	0.9531	0.1272
AGBL1	0.9404	0.9674	0.9570	0.9474	0.9531	0.0059
LOC388820	0.9728	1.0291	0.9306	0.8798	0.9531	0.0317
LOC149950	0.8997	1.1337	0.8852	0.8936	0.9531	0.0603
ZNF675	0.9002	1.0466	1.0088	0.8566	0.9531	0.0447
MEOX1	0.8711	1.1414	0.9244	0.8755	0.9531	0.0639
CCDC114	0.9899	0.9193	0.9720	0.9312	0.9531	0.0167
CGB2	0.8369	1.0670	0.9565	0.9520	0.9531	0.0470
LOC647104	0.8372	1.1867	0.9864	0.8022	0.9531	0.0875
HIST1H3A	0.8885	1.0960	0.9188	0.9092	0.9531	0.0480
FAM110C	0.8616	1.0322	0.9672	0.9515	0.9531	0.0351

FRRS1	1.0033	1.1902	0.9398	0.6791	0.9531	0.1057
ALS2CR11	0.9737	1.0413	0.9016	0.8958	0.9531	0.0343
LOC653570	0.9439	1.0001	0.9999	0.8685	0.9531	0.0312
SLC27A3	0.8702	1.2781	0.8182	0.8460	0.9531	0.1088
RICTOR	0.9133	1.1434	0.9325	0.8234	0.9531	0.0677
SLC45A2	0.8579	1.0180	1.0174	0.9192	0.9531	0.0393
C1orf81	0.7457	1.0838	1.0152	0.9677	0.9531	0.0731
LOC338829	0.9369	1.0222	1.0197	0.8338	0.9531	0.0445
ELA2A	0.9360	1.0429	0.9314	0.9023	0.9532	0.0308
SNORD58B	0.8859	0.9133	1.0127	1.0007	0.9532	0.0315
LOC1001305	0.7897	1.1368	0.9742	0.9120	0.9532	0.0722
TRIM9	0.7955	1.1817	0.9768	0.8586	0.9532	0.0850
LOC652768	0.8986	1.0357	1.0585	0.8199	0.9532	0.0568
LOC1001307	0.9066	1.0033	1.0129	0.8900	0.9532	0.0319
LOC647060	0.9559	1.0830	0.8779	0.8960	0.9532	0.0464
TREH	0.9319	0.9527	0.9644	0.9638	0.9532	0.0076
OLFML1	0.9625	1.0439	0.8403	0.9661	0.9532	0.0421
LOC730050	0.8761	1.1329	0.9737	0.8301	0.9532	0.0670
BMF	0.9879	1.0367	0.8442	0.9440	0.9532	0.0410
LOC442113	0.9029	1.0353	0.9421	0.9326	0.9532	0.0286
LOC643089	0.9886	0.9103	0.9814	0.9325	0.9532	0.0190
LRRC52	0.9149	1.1074	0.9962	0.7943	0.9532	0.0660
CYP2C19	0.9412	1.0913	1.0234	0.7570	0.9532	0.0722
NHEDC1	0.8802	1.0327	1.0083	0.8916	0.9532	0.0392
LOC1001309	0.8313	1.0613	0.9685	0.9517	0.9532	0.0472
ATP2B2	0.9174	0.9911	1.0282	0.8763	0.9532	0.0345
RPL34	0.7955	1.3577	0.9715	0.6883	0.9532	0.1469
LOC653411	0.9058	1.0660	1.0113	0.8299	0.9533	0.0529
LOC650553	0.9277	1.0137	1.0256	0.8460	0.9533	0.0419
C1orf188	0.9320	1.0567	0.9219	0.9025	0.9533	0.0350
LOC653907	0.7973	1.0482	1.0334	0.9341	0.9533	0.0578
HPSE2	0.9565	1.0623	0.9937	0.8005	0.9533	0.0554
SCARA5	0.8913	1.0886	0.9664	0.8668	0.9533	0.0498
RGPD4	0.9523	0.9720	0.9857	0.9031	0.9533	0.0181
PKD2L2	0.9301	1.0392	0.9551	0.8887	0.9533	0.0317
LOC652802	0.8531	1.0755	0.9979	0.8867	0.9533	0.0511
NCKAP1L	0.9576	1.0032	1.0055	0.8468	0.9533	0.0372
TAS2R49	0.9217	0.9417	1.1248	0.8248	0.9533	0.0626
ZNF660	0.8622	0.9916	0.9514	1.0079	0.9533	0.0326
SERAC1	0.8587	1.0050	1.0320	0.9174	0.9533	0.0399
CASKIN1	0.9043	0.9679	0.9851	0.9558	0.9533	0.0174
LOC391722	0.8994	1.0334	0.9948	0.8855	0.9533	0.0361
GNAT1	0.8809	1.0679	0.9581	0.9063	0.9533	0.0414
LOC647435	1.0139	0.8845	1.0049	0.9100	0.9533	0.0328
LOC728849	0.8400	1.0320	1.0104	0.9308	0.9533	0.0436
EYS	0.8321	1.0425	1.0932	0.8454	0.9533	0.0670

TCF21	0.9298	1.0547	0.9732	0.8555	0.9533	0.0416
LOC389813	0.9254	1.0147	1.0572	0.8159	0.9533	0.0534
TET2	0.9351	0.9716	1.0082	0.8983	0.9533	0.0236
SLC43A3	0.9125	1.0127	1.0338	0.8543	0.9533	0.0423
CTSE	0.8970	0.9693	0.9911	0.9558	0.9533	0.0201
LOC642895	0.9324	0.9627	1.0087	0.9094	0.9533	0.0214
C1orf100	0.9149	1.0823	0.9483	0.8678	0.9533	0.0461
MIR125A	0.9286	1.1217	0.9387	0.8243	0.9533	0.0618
LOC729506	0.9572	0.9764	0.9668	0.9128	0.9533	0.0140
LOC1001318	0.9594	1.0967	0.8500	0.9073	0.9533	0.0528
LOC654088	0.9014	1.0571	0.9721	0.8827	0.9533	0.0396
CT47A6	0.9205	1.0161	0.9845	0.8922	0.9533	0.0285
HIST1H2AI	0.8022	1.1770	1.1388	0.6953	0.9533	0.1204
TRIM75	0.8281	0.9755	1.0079	1.0019	0.9533	0.0423
QSER1	0.9131	1.0016	0.9956	0.9030	0.9533	0.0262
C17orf87	0.8737	1.1558	0.9240	0.8600	0.9534	0.0689
PALM	0.9502	1.1583	0.9162	0.7887	0.9534	0.0766
AGTR1	0.8723	1.0535	0.9852	0.9025	0.9534	0.0410
TRAF5	0.8963	1.0454	0.9710	0.9007	0.9534	0.0351
ASPA	0.8743	1.0090	0.9352	0.9950	0.9534	0.0308
LOC440366	0.9026	1.1066	0.9365	0.8679	0.9534	0.0529
KIAA1324	0.9594	1.0013	0.9053	0.9475	0.9534	0.0198
LOC646008	0.9011	0.9286	1.1279	0.8560	0.9534	0.0601
LOC646374	0.9999	1.0966	0.8378	0.8793	0.9534	0.0588
LOC652771	0.8456	1.0901	0.9752	0.9028	0.9534	0.0527
LOC652126	0.8749	1.0658	0.9850	0.8880	0.9534	0.0448
RAB1A	0.6612	1.4607	0.9574	0.7344	0.9534	0.1805
MS4A1	0.9086	1.0461	0.9178	0.9413	0.9534	0.0316
LOC651353	0.8635	1.0616	1.0504	0.8382	0.9534	0.0595
CCBL2	0.8105	1.1867	1.0937	0.7229	0.9534	0.1109
TRAPPC9	0.9489	1.0766	0.9470	0.8412	0.9534	0.0482
LOC1001323	0.9823	0.9819	0.9178	0.9318	0.9534	0.0168
CD151	0.8517	1.2183	0.9839	0.7599	0.9534	0.0995
FLJ32310	0.8438	1.0536	1.0317	0.8846	0.9534	0.0524
JRKL	0.8865	1.0444	0.9779	0.9051	0.9535	0.0362
LOC1001301	0.9135	0.9808	0.9777	0.9419	0.9535	0.0160
ZNF688	0.8305	1.2058	0.9633	0.8142	0.9535	0.0905
LOC650410	0.9518	1.1305	0.8272	0.9043	0.9535	0.0643
DCD	0.9437	1.0273	0.9357	0.9071	0.9535	0.0258
C1orf157	0.9205	1.1276	0.9953	0.7705	0.9535	0.0745
ASB14	0.8158	0.9947	1.0816	0.9217	0.9535	0.0563
EPS8L2	0.8820	1.0554	0.9894	0.8871	0.9535	0.0420
IL6ST	0.8280	1.0690	1.0267	0.8902	0.9535	0.0566
INTS6	0.8648	1.1905	0.9700	0.7887	0.9535	0.0873
UBE2NL	0.9345	1.0978	0.9006	0.8811	0.9535	0.0493
LOC646996	0.6721	1.3714	1.0252	0.7453	0.9535	0.1587

IL9R	0.9447	1.0100	0.9985	0.8608	0.9535	0.0340
C10orf104	0.9796	1.0411	1.0631	0.7301	0.9535	0.0765
GTSCR1	0.9607	0.9333	1.0919	0.8281	0.9535	0.0543
LOC730184	0.9297	1.0351	0.9797	0.8696	0.9535	0.0353
AVIL	0.8508	1.0680	0.9731	0.9222	0.9535	0.0457
LOC442676	0.9073	1.0538	0.9909	0.8621	0.9535	0.0428
TRAPPC4	0.8297	1.4885	0.8215	0.6744	0.9535	0.1818
LOC440864	0.8775	1.0025	1.0072	0.9269	0.9535	0.0313
LOC1001328	0.9086	1.1078	0.9550	0.8427	0.9535	0.0563
C1orf70	0.8852	1.1128	1.0021	0.8141	0.9535	0.0657
RGAG1	0.8776	0.9894	0.9973	0.9498	0.9535	0.0274
LOC1001315	0.9956	1.0320	0.9900	0.7966	0.9535	0.0532
C9orf25	0.9117	0.9950	0.8677	1.0397	0.9535	0.0390
ZNF133	0.9088	1.1339	0.9622	0.8093	0.9535	0.0680
FLJ20920	0.9246	1.1344	0.9480	0.8072	0.9535	0.0677
GTF2H1	0.8970	1.0572	0.9914	0.8686	0.9535	0.0434
LOC646851	0.8752	1.0320	0.9522	0.9548	0.9536	0.0320
LOC1001334	0.8679	1.0659	1.0920	0.7885	0.9536	0.0744
OR13C3	0.9452	1.0726	0.9118	0.8847	0.9536	0.0416
F9	0.7669	1.0964	1.0745	0.8765	0.9536	0.0795
LAMB1	0.8969	1.1194	1.0270	0.7710	0.9536	0.0761
LOC647188	0.9760	1.0876	0.8552	0.8955	0.9536	0.0512
FLJ44653	0.9292	1.0065	1.0378	0.8407	0.9536	0.0440
LOC730254	0.8671	1.1227	0.9565	0.8680	0.9536	0.0601
PRAMEF7	0.9027	1.0054	1.0255	0.8807	0.9536	0.0362
LOC1001335	0.9121	1.0391	1.0072	0.8559	0.9536	0.0423
LOC643648	0.9276	0.9971	0.9987	0.8910	0.9536	0.0266
ZNF277	0.7632	1.4209	1.0136	0.6168	0.9536	0.1760
NPVF	0.9217	0.9217	1.0158	0.9552	0.9536	0.0222
SAP130	0.9331	1.3366	0.7988	0.7459	0.9536	0.1336
PRAMEF5	0.8836	1.0541	1.0446	0.8321	0.9536	0.0563
KIAA0319	0.9339	1.0321	0.8995	0.9489	0.9536	0.0281
ERCC-00099	0.8632	1.1228	0.9896	0.8388	0.9536	0.0654
LOC648447	0.8845	1.0383	1.0029	0.8888	0.9536	0.0393
GPRASP1	0.8903	0.9728	1.0144	0.9369	0.9536	0.0264
LOC641721	1.0056	1.0121	0.9051	0.8916	0.9536	0.0321
LOC731957	0.9419	1.0295	0.9291	0.9139	0.9536	0.0259
LOC648099	0.8179	1.0639	1.1807	0.7520	0.9536	0.1011
LOC1001343	1.0196	1.0074	0.9325	0.8550	0.9536	0.0381
KCNC3	0.9198	1.0860	0.8797	0.9290	0.9536	0.0454
LOC729189	0.9617	0.9402	0.9988	0.9139	0.9536	0.0179
SPACA5	0.8856	1.0137	1.0146	0.9007	0.9536	0.0351
SULT1B1	0.9011	1.0406	0.9305	0.9423	0.9536	0.0303
PAQR7	0.8874	1.1523	0.9285	0.8464	0.9537	0.0683
UGT2B4	0.8790	1.0810	0.9732	0.8815	0.9537	0.0478
LIPG	0.9027	1.1059	0.9475	0.8586	0.9537	0.0539

FLJ37543	0.9017	1.2510	0.8286	0.8334	0.9537	0.1005
LOC642346	0.9011	1.0040	1.0530	0.8566	0.9537	0.0453
LOC643774	0.9247	0.9743	1.0539	0.8618	0.9537	0.0406
PTOV1	0.8230	1.4553	0.8295	0.7069	0.9537	0.1696
NRF1	0.9037	1.0021	1.0275	0.8814	0.9537	0.0360
FLJ10781	0.8295	1.0482	0.9904	0.9468	0.9537	0.0463
ACTR8	0.8842	1.1718	1.0354	0.7233	0.9537	0.0967
FAM84B	0.8629	1.2283	1.0063	0.7173	0.9537	0.1089
LOC652676	1.0180	1.1331	0.7588	0.9049	0.9537	0.0799
NDUFB6	0.8475	1.2689	0.9651	0.7333	0.9537	0.1152
WIT-1	0.9779	1.0643	0.9190	0.8536	0.9537	0.0448
YPEL5	0.9477	1.1500	0.9540	0.7632	0.9537	0.0790
HOXC13	1.0489	1.2335	0.7221	0.8105	0.9537	0.1160
TTC4	0.7730	1.3730	0.9897	0.6792	0.9537	0.1542
C8orf15	1.0113	1.0601	0.9779	0.7656	0.9537	0.0649
LOC728973	0.8589	1.0125	1.2441	0.6994	0.9537	0.1160
LOC1001337	0.9337	0.9940	0.9960	0.8913	0.9537	0.0253
DLEU7	0.8903	1.0344	1.0314	0.8589	0.9537	0.0461
LOC1001318	0.8691	1.0758	0.9658	0.9043	0.9537	0.0453
RYK	0.8199	1.3072	0.9551	0.7328	0.9538	0.1264
LOC642223	0.9348	1.0855	0.9582	0.8367	0.9538	0.0512
LOC645969	0.7995	1.2047	0.9878	0.8231	0.9538	0.0935
OR51D1	0.8740	1.0548	0.9388	0.9474	0.9538	0.0375
LOC649768	1.0363	1.0299	0.9482	0.8007	0.9538	0.0548
DDR1	0.9182	1.2018	0.9339	0.7612	0.9538	0.0914
LOC644589	0.9454	1.0103	1.0257	0.8338	0.9538	0.0436
LOC440132	0.9663	0.8790	0.9687	1.0011	0.9538	0.0262
LOC649174	0.8116	1.0950	1.0433	0.8652	0.9538	0.0683
LOC440925	0.8663	1.0639	0.9888	0.8962	0.9538	0.0450
RNASE8	0.7989	1.0542	1.0544	0.9077	0.9538	0.0621
MIR2115	0.8731	1.1222	0.9373	0.8826	0.9538	0.0579
LOC649379	0.8998	0.9898	1.0904	0.8351	0.9538	0.0555
OR10G4	0.8688	1.1141	0.9368	0.8956	0.9538	0.0552
LOC730031	0.8836	1.1045	0.9446	0.8826	0.9538	0.0523
LOC1001334	0.9613	1.0385	0.9465	0.8690	0.9538	0.0347
FAM167A	0.8511	1.0791	1.0275	0.8575	0.9538	0.0584
C1orf9	0.8790	1.0417	1.0052	0.8892	0.9538	0.0410
GRHL3	0.9853	1.0398	0.9324	0.8578	0.9538	0.0388
SNORA5B	0.8048	1.1350	0.9889	0.8865	0.9538	0.0712
SLCO3A1	0.8713	1.0614	0.9903	0.8923	0.9538	0.0443
SNORA11E	0.8771	1.2305	0.9290	0.7786	0.9538	0.0974
NR0B2	0.9133	0.9525	1.0591	0.8905	0.9538	0.0373
GSTM1	0.9309	1.0420	0.9191	0.9233	0.9538	0.0295
GJB5	0.9280	0.8819	1.0122	0.9933	0.9538	0.0300
LOC253573	0.9193	1.1447	0.9579	0.7934	0.9538	0.0727
BVES	0.9264	1.0786	0.9670	0.8434	0.9538	0.0489

SLC22A11	0.8830	1.0918	0.9359	0.9046	0.9538	0.0472
LOC1001335	0.9127	1.0114	0.9818	0.9094	0.9538	0.0254
ZC3H13	0.9569	0.9309	0.9918	0.9358	0.9539	0.0139
ZFYVE9	0.8183	1.1554	0.9911	0.8507	0.9539	0.0769
LOC728789	0.9311	0.9788	0.9187	0.9869	0.9539	0.0170
LOC643937	0.9290	1.1973	0.8642	0.8249	0.9539	0.0839
LOC648615	1.0334	1.0699	0.8093	0.9029	0.9539	0.0601
PDCL3	0.7235	1.3744	1.0190	0.6988	0.9539	0.1579
LOC652663	0.9729	0.9815	0.9852	0.8759	0.9539	0.0261
C10orf4	0.9134	1.0260	1.0008	0.8754	0.9539	0.0356
LOC345643	0.9890	1.0470	0.9271	0.8525	0.9539	0.0417
TncRNA	0.9954	1.0781	0.8590	0.8831	0.9539	0.0510
LOC648460	0.9498	0.9955	0.9491	0.9212	0.9539	0.0154
MSI2	0.7669	1.2759	0.9345	0.8383	0.9539	0.1127
RCSD1	0.8382	1.0333	1.1075	0.8366	0.9539	0.0690
C21orf89	0.9459	0.9644	1.0310	0.8745	0.9539	0.0322
FLJ45340	0.8933	1.0725	0.9517	0.8982	0.9539	0.0417
LOC646997	0.9571	1.0265	1.0066	0.8255	0.9539	0.0452
GDAP2	0.7819	1.1429	1.0539	0.8370	0.9539	0.0861
BEX2	0.8545	1.4456	0.8419	0.6737	0.9539	0.1690
LOC647786	0.9784	1.0038	1.0368	0.7968	0.9539	0.0537
C8orf58	0.8300	1.0254	1.0513	0.9091	0.9539	0.0516
LOC1001326	0.9050	1.1079	0.9326	0.8702	0.9539	0.0529
CPA3	0.9601	1.1057	0.8571	0.8929	0.9540	0.0549
ASCL5	0.8162	1.0783	1.0046	0.9167	0.9540	0.0566
ASAH2C	0.8952	1.1329	1.0536	0.7341	0.9540	0.0884
ING4	0.9346	1.0525	0.9282	0.9005	0.9540	0.0337
LOC653658	0.8888	0.9401	1.3813	0.6056	0.9540	0.1603
SCN10A	0.8956	1.0791	0.9809	0.8602	0.9540	0.0488
TTC9B	0.9408	1.0806	0.8872	0.9073	0.9540	0.0436
SNORD113-6	0.8626	1.1604	0.9015	0.8915	0.9540	0.0693
C9orf78	0.7047	1.3699	1.0640	0.6773	0.9540	0.1643
LOC442442	0.9004	1.0571	1.0482	0.8102	0.9540	0.0599
PRR20B	0.8386	1.0550	1.0702	0.8521	0.9540	0.0628
PRDM1	0.8983	1.0291	1.0047	0.8838	0.9540	0.0368
LOC652734	0.9033	1.0525	0.9795	0.8807	0.9540	0.0390
LOC644897	0.8523	1.0539	1.0731	0.8366	0.9540	0.0634
LOC1001330	0.8766	0.9930	1.0018	0.9446	0.9540	0.0287
LOC442535	0.8495	1.0685	1.0040	0.8940	0.9540	0.0501
LOC650236	0.9427	1.0803	0.9272	0.8658	0.9540	0.0453
LOC645777	0.8293	1.0950	0.9754	0.9163	0.9540	0.0558
BCAP31	0.7958	1.4307	0.9277	0.6618	0.9540	0.1679
CXXC4	0.9618	1.0848	0.9159	0.8535	0.9540	0.0489
LOC1001342	0.8943	0.9729	0.9493	0.9995	0.9540	0.0224
LOC729970	0.9251	0.9258	1.1578	0.8073	0.9540	0.0734
LOC651614	0.9113	1.1022	0.9671	0.8354	0.9540	0.0563

LOC645671	0.9633	1.0428	0.9669	0.8431	0.9540	0.0413
ADAMTS14	0.9432	1.0140	0.9828	0.8761	0.9540	0.0297
LOC440795	0.9409	1.1461	0.9360	0.7930	0.9540	0.0726
MIER2	0.8274	1.0982	1.0156	0.8749	0.9540	0.0625
LOC649383	0.8333	0.9888	1.0761	0.9179	0.9540	0.0516
HSPCAL3	0.9114	1.0585	0.9520	0.8944	0.9540	0.0368
LOC647533	0.8879	0.9976	1.0206	0.9100	0.9541	0.0325
FAM74A3	0.8887	1.0675	0.9738	0.8861	0.9541	0.0430
FLJ25328	0.8790	1.1064	0.9787	0.8521	0.9541	0.0576
MAP3K7IP3	0.8465	1.1255	1.0103	0.8339	0.9541	0.0699
LOC387934	0.7880	1.0371	1.1422	0.8489	0.9541	0.0821
LOC643377	0.9897	0.9831	1.0144	0.8292	0.9541	0.0422
HBQ1	0.8578	1.1646	0.8581	0.9358	0.9541	0.0725
PTCHD2	0.9321	1.0361	0.9396	0.9086	0.9541	0.0281
LOC645487	0.8846	1.0341	1.0118	0.8859	0.9541	0.0400
HS3ST2	0.8921	1.0466	0.9651	0.9126	0.9541	0.0345
LOC645084	0.9270	1.0924	0.8530	0.9440	0.9541	0.0501
LOC441864	0.8462	1.2394	0.8801	0.8507	0.9541	0.0954
PRKCZ	0.9196	1.1873	0.9212	0.7885	0.9541	0.0837
LOC199897	0.9041	1.1833	0.9011	0.8280	0.9541	0.0784
FLJ44005	0.8028	1.0494	1.0377	0.9266	0.9541	0.0575
LOC643293	0.9747	1.0127	0.9849	0.8442	0.9541	0.0375
ABCC6P2	1.0307	1.1383	0.8957	0.7519	0.9541	0.0837
LOC439985	0.8511	0.9981	0.9985	0.9689	0.9541	0.0350
LOC119358	0.9076	1.0356	0.9340	0.9394	0.9541	0.0280
LOC651809	0.8609	1.0085	0.9806	0.9666	0.9541	0.0323
EIF4E	0.9282	1.0405	0.9637	0.8843	0.9541	0.0330
CLYBL	0.7658	1.2532	1.0715	0.7261	0.9542	0.1261
PTGES	0.8747	0.9909	1.0281	0.9229	0.9542	0.0343
LOC339788	0.9243	1.0077	0.9666	0.9180	0.9542	0.0208
LOC653234	0.9443	1.1021	0.8656	0.9047	0.9542	0.0519
MIR579	0.8688	1.0486	0.9969	0.9025	0.9542	0.0415
CRLF1	0.8951	1.0623	1.0124	0.8469	0.9542	0.0501
SNORD12C	0.8539	0.9484	1.1121	0.9024	0.9542	0.0560
LOC644809	0.8161	1.0914	1.0134	0.8959	0.9542	0.0611
LOC654118	0.8646	1.0273	1.0776	0.8473	0.9542	0.0577
LOC1001305	0.9163	1.0021	0.9945	0.9038	0.9542	0.0257
LOC163233	0.8937	1.0586	0.9780	0.8865	0.9542	0.0405
CSK	0.8641	1.4883	0.8509	0.6136	0.9542	0.1871
LOC727765	0.9454	1.0469	0.9466	0.8780	0.9542	0.0348
CUZD1	0.8674	1.0738	0.9705	0.9051	0.9542	0.0452
AKR1E2	0.9108	1.0981	0.9298	0.8781	0.9542	0.0491
IL15RA	0.8839	1.0419	0.9798	0.9112	0.9542	0.0355
LETMD1	0.8214	1.3905	0.9168	0.6883	0.9542	0.1528
LOC1001287	0.8147	1.0183	1.0430	0.9408	0.9542	0.0513
BEST4	0.9709	1.0501	0.9961	0.7998	0.9542	0.0541

LOC647013	0.9492	0.9267	1.0095	0.9316	0.9542	0.0190
LOC644187	0.8667	0.9721	1.0715	0.9066	0.9542	0.0447
APLF	0.9620	1.0370	0.9256	0.8924	0.9543	0.0310
LRRC59	0.8365	1.0349	0.9824	0.9632	0.9543	0.0421
FCGR2B	0.8407	1.1416	1.0255	0.8093	0.9543	0.0786
TXNL4A	0.8065	1.1883	1.0109	0.8114	0.9543	0.0914
EMB	0.8480	1.0329	0.9998	0.9364	0.9543	0.0407
ELAC1	0.8651	1.0724	1.0560	0.8236	0.9543	0.0641
S100A7A	0.9107	0.9791	1.0747	0.8527	0.9543	0.0477
TAAR3	0.8332	0.9906	1.0086	0.9848	0.9543	0.0407
RBPMS	0.9535	1.0233	0.9758	0.8645	0.9543	0.0333
ZNF347	0.9215	1.0700	0.9740	0.8518	0.9543	0.0460
ARL8A	0.8043	1.1372	0.9898	0.8860	0.9543	0.0718
GNA15	0.8692	0.9745	0.9886	0.9850	0.9543	0.0285
LOC653632	0.9421	1.0085	0.9119	0.9548	0.9543	0.0202
LOC646818	0.8531	1.0137	1.0370	0.9136	0.9543	0.0431
LOC654046	0.9179	1.0000	0.9862	0.9132	0.9543	0.0226
LOC132203	0.9428	1.0090	0.9889	0.8765	0.9543	0.0294
NFKBIL1	0.9051	1.1026	0.8950	0.9147	0.9543	0.0496
LOC388755	0.8847	0.9432	1.1086	0.8809	0.9543	0.0534
C3orf51	1.0053	0.9911	0.9451	0.8758	0.9544	0.0292
IGFALS	0.8098	1.0647	1.0702	0.8727	0.9544	0.0666
RASL11A	0.9408	1.0391	1.0398	0.7978	0.9544	0.0571
FAM193B	0.8760	1.0692	0.9829	0.8894	0.9544	0.0451
PRR8	0.7964	1.0805	0.9671	0.9736	0.9544	0.0587
RGS2	0.8722	0.9756	1.0579	0.9118	0.9544	0.0406
LOC646449	0.9870	1.0236	0.9628	0.8442	0.9544	0.0388
ABCC2	0.8832	1.0075	0.9796	0.9472	0.9544	0.0267
LOC646914	0.9299	1.0944	0.9875	0.8058	0.9544	0.0601
CDGAP	0.9809	0.9843	0.9453	0.9070	0.9544	0.0181
C10orf93	0.9202	1.0563	0.9834	0.8577	0.9544	0.0426
CSN3	0.9817	1.0242	0.9457	0.8660	0.9544	0.0335
HYLS1	0.8397	1.1540	1.0101	0.8138	0.9544	0.0795
FAM20A	0.9330	1.0036	1.0206	0.8605	0.9544	0.0366
LOC642530	0.9325	1.0245	0.9842	0.8765	0.9544	0.0321
ZNF385C	0.6738	1.0814	0.9949	1.0676	0.9544	0.0954
ERP44	0.9257	1.0263	0.9299	0.9358	0.9544	0.0241
LOC730284	0.8857	1.1776	0.8915	0.8629	0.9544	0.0747
APOL3	0.8703	1.1075	0.9855	0.8544	0.9544	0.0588
LOC619207	0.8597	1.0535	1.0013	0.9033	0.9544	0.0443
LOC646428	0.9880	0.9978	0.9386	0.8934	0.9544	0.0241
FLJ44861	0.8472	1.0209	1.0817	0.8680	0.9545	0.0574
IFI44L	0.9445	1.0553	0.9759	0.8421	0.9545	0.0441
LOC645722	0.9388	1.0146	1.0276	0.8368	0.9545	0.0438
GALNTL2	0.9276	0.9564	0.9978	0.9362	0.9545	0.0156
LOC642946	0.9510	1.1014	0.9489	0.8167	0.9545	0.0582

ESRRB	0.8252	1.0320	1.0077	0.9531	0.9545	0.0461
LOC442570	0.8158	1.0569	0.9542	0.9911	0.9545	0.0509
LOC650496	0.9429	0.9869	0.9820	0.9064	0.9545	0.0188
PPP3R1	0.7896	1.3727	0.8809	0.7748	0.9545	0.1414
LOC1001317[illegible]	0.9541	1.0776	0.9963	0.7900	0.9545	0.0605
LOC1001309	0.8590	1.1730	0.9971	0.7889	0.9545	0.0847
SUSD3	0.9528	0.9601	1.0279	0.8774	0.9545	0.0308
NPY6R	0.9726	0.9554	0.9631	0.9271	0.9545	0.0098
SFRS11	0.9194	1.0421	1.0814	0.7754	0.9546	0.0690
JAZF1	0.8187	1.1341	1.0139	0.8515	0.9546	0.0735
LOC728290	0.9217	0.9369	0.9767	0.9829	0.9546	0.0149
ATXN2L	0.9085	1.0779	0.9351	0.8967	0.9546	0.0419
MIR218-2	0.9271	1.0815	0.9050	0.9047	0.9546	0.0426
PRHOXNB	0.9296	1.0031	0.9788	0.9069	0.9546	0.0221
HRASLS5	0.9769	1.0115	1.0351	0.7948	0.9546	0.0546
AFAP1L1	0.8731	1.0347	1.0730	0.8375	0.9546	0.0583
SMOX	0.8943	1.0004	1.0504	0.8733	0.9546	0.0424
LBH	0.8970	1.0985	0.9352	0.8877	0.9546	0.0491
T1560	0.7786	1.0939	1.0235	0.9225	0.9546	0.0684
LOC728318	0.9820	0.9786	0.9420	0.9157	0.9546	0.0158
PSG3	0.9721	0.9919	0.9509	0.9035	0.9546	0.0190
BECN1L1	0.9592	0.9740	0.9888	0.8964	0.9546	0.0203
LOC1001335[illegible]	0.8373	1.0606	0.9951	0.9253	0.9546	0.0479
POP5	0.8117	1.2144	1.0043	0.7879	0.9546	0.0992
LOC388438	0.9212	0.9989	1.0094	0.8889	0.9546	0.0294
C16orf56	0.8832	1.3621	0.8381	0.7349	0.9546	0.1393
LOC727748	0.8214	1.0883	0.9872	0.9215	0.9546	0.0561
LOC652288	1.0164	1.0309	0.9580	0.8132	0.9546	0.0497
LOC1001323	0.8986	1.0239	1.0056	0.8904	0.9546	0.0349
CES1	0.9095	1.0666	0.9323	0.9100	0.9546	0.0377
LOC644300	0.9342	1.0465	0.9400	0.8979	0.9546	0.0320
GPIHBP1	0.9579	1.0007	0.9847	0.8754	0.9546	0.0279
KRT18P17	0.9092	1.1171	0.9643	0.8280	0.9546	0.0610
LOC730243	0.7982	1.1033	1.0250	0.8920	0.9546	0.0680
LIME1	0.7743	1.2297	0.8300	0.9846	0.9546	0.1019
LOC651091	0.8957	1.0263	0.9366	0.9601	0.9547	0.0273
PRAMEF3	0.9282	1.0356	0.9533	0.9015	0.9547	0.0290
RND2	1.0393	0.9530	1.0204	0.8060	0.9547	0.0529
KCNMB3	0.9024	1.0520	0.9533	0.9110	0.9547	0.0343
LOC730078	0.8665	1.0096	1.0489	0.8936	0.9547	0.0442
LOC642434	0.9149	0.9744	1.0484	0.8809	0.9547	0.0367
LOC643695	0.8851	0.9596	1.0941	0.8799	0.9547	0.0499
CRYGC	0.9500	0.9831	0.9444	0.9411	0.9547	0.0097
LOC645869	0.8913	0.9964	1.0316	0.8995	0.9547	0.0350
PPCDC	0.9395	1.0443	1.0454	0.7896	0.9547	0.0604
LOC648608	0.9602	1.0104	1.0707	0.7775	0.9547	0.0632

CHMP7	0.8657	1.1847	0.8983	0.8701	0.9547	0.0770
LRRC16	0.9587	1.0534	0.9905	0.8162	0.9547	0.0502
LOC339742	0.9241	1.0197	1.0280	0.8470	0.9547	0.0430
LOC440551	0.9795	1.0601	0.8955	0.8837	0.9547	0.0411
USP16	0.8460	1.2174	1.0846	0.6708	0.9547	0.1219
FLJ22536	0.8687	1.1095	0.9685	0.8721	0.9547	0.0565
LOC402693	0.9365	1.0123	1.0092	0.8608	0.9547	0.0359
SNX33	0.8338	1.0179	1.0686	0.8987	0.9547	0.0538
LOC643220	0.8850	1.0404	1.0456	0.8480	0.9547	0.0515
MIR596	0.9434	1.0141	0.9832	0.8783	0.9547	0.0293
LOC1001343	0.9041	1.0977	0.9233	0.8939	0.9547	0.0480
FADS6	0.9152	1.0301	0.9111	0.9626	0.9547	0.0277
POMT1	0.8770	1.2123	0.9344	0.7953	0.9547	0.0905
LOC144678	0.9384	1.1190	0.9501	0.8116	0.9547	0.0631
ARSG	0.9377	0.8987	1.0527	0.9299	0.9548	0.0337
MIRLET7D	1.0529	0.9774	0.9670	0.8217	0.9548	0.0483
LOC220429	0.8412	0.9535	1.0507	0.9737	0.9548	0.0433
MGC34800	0.8436	1.0206	0.9651	0.9898	0.9548	0.0388
SCAF1	0.8860	0.9983	0.9809	0.9539	0.9548	0.0247
FRMD7	0.8462	1.0310	1.0632	0.8787	0.9548	0.0541
LOC652424	0.7596	1.0686	1.0788	0.9121	0.9548	0.0754
IKZF3	0.8026	1.1883	0.9834	0.8449	0.9548	0.0869
MEF2A	0.8761	1.0317	0.9685	0.9428	0.9548	0.0322
LOC1001342	0.8479	1.0826	0.9843	0.9044	0.9548	0.0510
POM121	0.9291	1.0865	0.9354	0.8683	0.9548	0.0464
NLRP1	0.9128	1.0162	1.0189	0.8713	0.9548	0.0372
HBEGF	0.7819	1.2642	0.9422	0.8309	0.9548	0.1084
LOC1001288	0.8819	1.0532	1.0583	0.8258	0.9548	0.0594
PWWP2A	0.9827	1.1214	1.0050	0.7101	0.9548	0.0870
PHF11	0.9044	1.0024	0.9770	0.9354	0.9548	0.0218
PGBD2	0.8129	1.1151	1.0643	0.8270	0.9548	0.0786
LOC649500	0.9699	1.0010	0.9160	0.9325	0.9548	0.0191
KNDC1	0.9102	1.0116	1.0459	0.8517	0.9548	0.0449
OR8B3	0.9446	1.0215	0.9891	0.8642	0.9549	0.0341
LOC1001299	0.8822	1.0272	0.9996	0.9104	0.9549	0.0348
MUC3A	0.9028	0.9559	1.0028	0.9580	0.9549	0.0205
MAGEB18	0.8936	1.0500	1.0355	0.8403	0.9549	0.0520
DAPL1	0.7746	1.1426	1.0343	0.8679	0.9549	0.0825
ARHGAP1	0.9153	1.2437	0.8648	0.7957	0.9549	0.0994
LOC388948	0.9439	1.0246	0.9708	0.8802	0.9549	0.0300
LOC647995	0.8474	1.1117	0.9740	0.8865	0.9549	0.0586
OR10A7	0.7893	1.1131	0.8979	1.0192	0.9549	0.0706
SLC5A8	0.9232	1.1299	0.9175	0.8489	0.9549	0.0607
POTEC	1.0013	1.0173	0.9135	0.8875	0.9549	0.0320
LOC647413	0.9070	0.9891	0.9981	0.9254	0.9549	0.0227
DSG4	0.8925	0.9910	0.9831	0.9530	0.9549	0.0224

LOC1001318	0.9933	1.0203	1.0421	0.7641	0.9549	0.0644
MIR591	0.9539	1.0194	1.0410	0.8055	0.9549	0.0531
PTGDS	0.9509	1.0523	0.9187	0.8979	0.9549	0.0342
LOC732300	1.0204	0.9292	0.9987	0.8716	0.9550	0.0339
LOC731602	0.8324	1.0736	1.1004	0.8135	0.9550	0.0765
LOC1001324	0.9229	1.0613	0.9921	0.8436	0.9550	0.0466
LOC727832	0.9418	1.0197	0.9493	0.9091	0.9550	0.0233
RPE65	0.8704	1.0970	1.0658	0.7868	0.9550	0.0752
LOC653412	0.9867	0.9369	0.9765	0.9199	0.9550	0.0159
LOC642471	0.8482	1.0334	0.9393	0.9990	0.9550	0.0406
C2orf78	0.9490	1.1597	0.7760	0.9354	0.9550	0.0787
LOC730375	0.8935	0.9891	0.9959	0.9416	0.9550	0.0238
REG1A	0.9686	0.9814	1.0568	0.8133	0.9550	0.0511
RALGPS2	0.9386	1.0399	0.9805	0.8612	0.9550	0.0376
SPTBN4	0.9269	1.1314	0.9118	0.8500	0.9550	0.0611
CFB	1.0056	1.0271	1.0076	0.7799	0.9551	0.0586
TMEM40	0.8610	1.0667	0.9550	0.9376	0.9551	0.0425
LOC1001328	0.7773	1.0686	1.1142	0.8602	0.9551	0.0811
LOC645705	1.0226	0.9668	1.0122	0.8187	0.9551	0.0470
TMEM233	0.8809	1.0446	1.0017	0.8933	0.9551	0.0403
WASH2P	0.8835	1.1789	0.8356	0.9224	0.9551	0.0767
RBMS3	0.8983	1.0366	1.0054	0.8801	0.9551	0.0388
C14orf149	1.0132	1.0698	1.0116	0.7258	0.9551	0.0776
LOC400145	0.9622	1.0167	0.9856	0.8559	0.9551	0.0349
DAZ4	0.9388	1.0915	0.9678	0.8223	0.9551	0.0553
DUB3	0.8156	0.9919	1.0977	0.9153	0.9551	0.0597
ARPC5	0.9761	1.0003	1.3202	0.5240	0.9551	0.1637
CES4	0.8861	0.9977	0.9690	0.9678	0.9551	0.0240
POU3F3	0.8330	1.0597	0.9858	0.9421	0.9551	0.0474
SLC16A2	0.9184	1.0199	0.9265	0.9558	0.9551	0.0230
OR7D2	0.9175	0.9823	0.9906	0.9302	0.9551	0.0183
RIPPLY2	0.9147	1.0175	0.9634	0.9251	0.9551	0.0233
OR5AP2	0.9490	1.0449	0.9724	0.8543	0.9551	0.0393
LOC1001319	0.9021	1.0813	0.9678	0.8693	0.9552	0.0468
TP53TG3	0.8772	1.2132	1.0340	0.6962	0.9552	0.1103
HDAC10	0.9010	1.0594	1.0473	0.8129	0.9552	0.0595
BEND6	0.8757	1.0834	0.9600	0.9015	0.9552	0.0462
LOC1001285	0.8845	1.1082	1.0346	0.7934	0.9552	0.0712
CCL23	0.8618	1.0287	1.0241	0.9060	0.9552	0.0421
LOC644403	0.8878	0.9138	1.0643	0.9548	0.9552	0.0389
LOC389370	0.8263	1.0141	0.9802	1.0000	0.9552	0.0435
LOC339766	0.8810	1.0569	1.0344	0.8484	0.9552	0.0529
LOC648749	0.9738	0.9999	0.9235	0.9235	0.9552	0.0191
LOC653559	0.8628	1.0359	0.9632	0.9588	0.9552	0.0355
IL6	0.9536	1.0183	0.9359	0.9129	0.9552	0.0226
UFSP2	0.8605	1.2279	1.0042	0.7281	0.9552	0.1070

LOC648963	0.9158	1.1680	0.9380	0.7990	0.9552	0.0772
C8orf4	0.8913	1.0496	0.9775	0.9024	0.9552	0.0368
LOC389936	0.9311	0.9323	1.0267	0.9308	0.9552	0.0238
NKX6-3	0.9274	1.0323	0.9490	0.9122	0.9552	0.0268
LOC651116	0.9042	1.0368	0.9375	0.9423	0.9552	0.0285
LOC389267	0.8490	1.1083	0.9330	0.9306	0.9552	0.0546
PAK2	0.6183	1.2611	1.2252	0.7163	0.9552	0.1676
LOC642743	0.9462	0.9482	1.0211	0.9054	0.9552	0.0241
CCDC87	0.8686	1.1038	1.0850	0.7635	0.9552	0.0833
LOC647783	0.7869	0.9832	1.0532	0.9976	0.9552	0.0581
FLJ44606	0.9345	1.1678	1.0043	0.7143	0.9552	0.0940
CLEC1B	0.9817	1.0082	0.9470	0.8841	0.9552	0.0268
MIR196A1	0.8978	1.0749	1.0065	0.8417	0.9552	0.0525
SLIT3	0.8978	1.0783	0.9278	0.9170	0.9552	0.0415
ADCYAP1	0.9516	1.0489	1.0159	0.8047	0.9553	0.0541
RNF166	0.9735	0.9724	1.0156	0.8595	0.9553	0.0335
MIR497	0.9618	1.0728	0.9322	0.8543	0.9553	0.0453
LIG4	0.9231	1.0512	0.9988	0.8480	0.9553	0.0444
ZDHHC11	0.9220	1.1431	0.9150	0.8409	0.9553	0.0652
TFIP11	0.7915	1.3737	0.9889	0.6669	0.9553	0.1544
LOC731231	0.8500	1.1226	1.0004	0.8481	0.9553	0.0662
LOC150051	0.8919	1.0348	1.0221	0.8723	0.9553	0.0425
LOC440995	0.9331	0.9499	1.0268	0.9113	0.9553	0.0251
CACNA1H	0.9713	1.1014	0.9105	0.8381	0.9553	0.0558
LOC1001288	0.9559	1.0641	0.9579	0.8433	0.9553	0.0451
BAI2	0.8314	1.0244	1.0408	0.9246	0.9553	0.0486
LOC651140	0.7978	1.0916	1.0920	0.8398	0.9553	0.0793
ESPNP	0.9108	0.9551	1.0096	0.9457	0.9553	0.0204
NAT11	0.8555	1.1095	1.0096	0.8466	0.9553	0.0636
LOC442726	0.9215	0.9435	1.0483	0.9080	0.9553	0.0318
KRTAP22-1	0.8888	1.0242	0.9653	0.9430	0.9553	0.0280
ZNF234	0.7863	1.1666	1.0035	0.8651	0.9553	0.0835
DGKQ	0.7633	1.3125	0.9822	0.7634	0.9553	0.1297
C10orf129	0.8856	1.1634	0.9435	0.8288	0.9553	0.0732
NPR3	0.8168	1.0124	1.0559	0.9363	0.9553	0.0524
WBSCR28	0.9504	0.9572	1.0295	0.8843	0.9554	0.0297
OXCT2	0.8977	1.0011	0.9791	0.9436	0.9554	0.0226
LOC652048	0.9464	1.0400	0.9222	0.9128	0.9554	0.0291
LOC126767	0.8428	1.1497	1.0230	0.8060	0.9554	0.0803
LOC1001339	0.8956	1.1093	0.9462	0.8703	0.9554	0.0537
DNAH14	0.9434	0.9387	1.0712	0.8682	0.9554	0.0423
TRIM14	0.9389	1.0393	0.9408	0.9026	0.9554	0.0293
SNORA42	0.7997	1.0731	0.9704	0.9784	0.9554	0.0569
ATP2A1	0.9193	1.0287	1.0065	0.8671	0.9554	0.0377
PPIG	0.9108	1.1995	0.9173	0.7940	0.9554	0.0862
MAGEB2	0.9258	1.0511	1.0351	0.8096	0.9554	0.0560

LOC1001332	0.8568	1.1163	0.9823	0.8662	0.9554	0.0608
GTPBP10	0.9163	1.1484	1.0232	0.7338	0.9554	0.0878
LIX1	0.9702	0.9433	1.0217	0.8865	0.9554	0.0281
TTTY6B	0.9769	0.9968	1.0133	0.8348	0.9554	0.0409
C21orf2	0.9192	1.0847	0.9849	0.8330	0.9554	0.0531
TTRAP	0.8848	1.0588	1.0136	0.8646	0.9554	0.0477
LOC1001281	0.8992	1.0202	1.0359	0.8665	0.9554	0.0426
LOC641697	0.8704	0.9277	1.0240	0.9997	0.9554	0.0349
LOC652548	0.8979	1.0914	1.0175	0.8150	0.9554	0.0615
LOC389842	0.9179	0.9596	1.0645	0.8798	0.9555	0.0398
SAMD3	0.9160	1.0427	0.9615	0.9017	0.9555	0.0317
MIR590	0.9496	0.9264	1.0118	0.9341	0.9555	0.0194
SLC39A1	0.6647	1.5381	0.9087	0.7104	0.9555	0.2013
GLIS2	1.0244	0.9385	0.9735	0.8855	0.9555	0.0292
CRB1	0.9537	0.9503	1.0387	0.8791	0.9555	0.0326
SNRPD1	0.7674	0.9998	1.2055	0.8493	0.9555	0.0962
SLC25A2	0.8922	1.0179	1.0577	0.8542	0.9555	0.0488
LOC642592	0.9773	0.8984	1.0444	0.9019	0.9555	0.0348
ZNF34	0.8836	1.1099	0.9534	0.8752	0.9555	0.0544
FFAR1	0.9073	1.1504	0.9339	0.8304	0.9555	0.0686
SPRYD3	0.9257	1.2049	0.9446	0.7469	0.9555	0.0943
CIR1	0.8171	1.0652	1.1697	0.7701	0.9555	0.0964
ERC1	0.9378	1.1058	0.9937	0.7849	0.9555	0.0667
OR1L3	0.9757	1.1153	0.8900	0.8412	0.9555	0.0601
U58	0.8621	1.0738	0.9425	0.9437	0.9555	0.0438
CLTC	0.8763	1.0879	1.0906	0.7674	0.9555	0.0803
LOC441687	0.9195	1.0209	0.9494	0.9323	0.9555	0.0226
NCRNA0016(	0.8599	1.0664	1.0983	0.7975	0.9555	0.0746
NUMBL	0.8352	1.1031	1.0654	0.8185	0.9556	0.0748
GK2	0.8556	1.0290	0.9811	0.9565	0.9556	0.0366
DEFB123	1.0100	1.0169	0.9559	0.8395	0.9556	0.0410
LOC649618	0.9304	0.9614	0.9813	0.9492	0.9556	0.0107
CEP290	0.9433	1.0485	0.9315	0.8990	0.9556	0.0324
KCTD17	0.9311	1.0823	0.8755	0.9334	0.9556	0.0443
LOC1001285	0.9106	1.0651	0.9875	0.8592	0.9556	0.0450
EIF2AK3	0.9170	1.1183	0.9373	0.8497	0.9556	0.0574
LOC202181	0.9518	1.0377	1.0178	0.8150	0.9556	0.0503
MIR324	0.9669	0.9122	0.9479	0.9953	0.9556	0.0174
C20orf46	0.9078	1.0020	0.9986	0.9140	0.9556	0.0259
LOC644528	0.8022	1.1326	0.9829	0.9048	0.9556	0.0696
RBBP6	0.9556	1.0812	0.9767	0.8089	0.9556	0.0561
LOC1001330	1.0165	1.0196	0.8971	0.8892	0.9556	0.0361
LOC727791	0.9226	1.1309	0.9321	0.8369	0.9556	0.0622
PCDHGA11	0.9350	0.9943	0.9512	0.9421	0.9556	0.0133
LOC401115	0.8236	1.1966	1.1180	0.6843	0.9556	0.1209
SPINT2	0.7472	1.2537	1.0163	0.8054	0.9556	0.1149

MIR34A	0.8737	0.9804	1.0348	0.9336	0.9556	0.0342
CNTNAP3B	0.8758	1.0643	1.0054	0.8770	0.9556	0.0473
CCR9	0.9481	1.0387	0.9270	0.9087	0.9556	0.0288
LOC401357	0.8582	1.0588	0.9834	0.9223	0.9556	0.0428
LOC650612	0.8348	1.0328	1.0521	0.9029	0.9556	0.0522
NR2E3	0.9646	1.0125	0.9233	0.9222	0.9557	0.0214
LOC1001320	0.8973	1.0386	1.0185	0.8682	0.9557	0.0427
LOC648684	0.9027	1.0717	1.0155	0.8328	0.9557	0.0540
TMEM67	0.8323	1.1165	0.9736	0.9003	0.9557	0.0609
FLJ44290	0.9672	0.9170	1.0798	0.8587	0.9557	0.0469
CHADL	0.9207	1.0190	0.9482	0.9348	0.9557	0.0218
LOC440829	0.9562	1.0876	0.9002	0.8788	0.9557	0.0469
LCA5	0.7791	1.0551	1.0864	0.9022	0.9557	0.0713
GZF1	0.9059	1.1306	0.9769	0.8095	0.9557	0.0676
AP2A2	1.0227	1.1310	0.8063	0.8629	0.9557	0.0742
LOC283953	0.8070	1.1065	1.0447	0.8647	0.9557	0.0713
HLCS	0.8997	1.0791	1.0075	0.8366	0.9557	0.0542
EMILIN1	0.9627	0.9871	0.9387	0.9345	0.9557	0.0121
LOC641726	1.0001	1.0259	0.9675	0.8294	0.9557	0.0438
LOC283767	0.9174	1.0724	0.9446	0.8885	0.9557	0.0405
LOC729966	0.9242	1.0195	0.9630	0.9162	0.9557	0.0236
LOC641938	0.8259	1.0579	1.0325	0.9066	0.9558	0.0545
APLP2	0.9431	1.1950	0.8969	0.7880	0.9558	0.0861
LOC730344	0.9518	1.0126	0.9316	0.9271	0.9558	0.0197
USH3A	0.9767	0.9821	0.9536	0.9107	0.9558	0.0162
NLRP3	0.8736	1.0119	1.0724	0.8653	0.9558	0.0514
MIR106B	0.8007	1.0430	1.0508	0.9286	0.9558	0.0588
SNORA2B	0.8639	0.9895	1.0263	0.9434	0.9558	0.0350
GPR84	0.8682	0.9953	0.9989	0.9607	0.9558	0.0305
LOC1001333	0.7556	1.2660	1.0823	0.7193	0.9558	0.1317
LOC1001340	0.8114	1.0637	1.0894	0.8588	0.9558	0.0706
CEND1	0.7467	1.1531	1.0011	0.9223	0.9558	0.0846
LOC653472	0.8938	1.0455	1.0338	0.8501	0.9558	0.0493
MIR569	0.9656	0.9989	1.0235	0.8352	0.9558	0.0419
CRELD2	0.8260	1.3157	0.9238	0.7578	0.9558	0.1247
LOC1001297	0.9695	1.1239	0.9333	0.7964	0.9558	0.0673
RAD1	0.8309	1.0608	1.0681	0.8634	0.9558	0.0631
MIR139	0.8435	1.0387	1.0863	0.8548	0.9558	0.0624
LOC415056	0.8452	1.0699	1.0410	0.8672	0.9558	0.0580
AADACL1	0.7849	1.2679	1.0146	0.7559	0.9558	0.1190
LOC646869	0.9593	0.9292	1.0460	0.8888	0.9558	0.0334
HTR2A	0.9645	1.0681	0.8779	0.9128	0.9558	0.0414
LOC642621	0.9306	1.0094	1.0689	0.8145	0.9558	0.0550
C9orf79	0.8807	1.1438	0.9439	0.8549	0.9558	0.0654
LOC644916	0.8246	1.0327	0.9893	0.9767	0.9558	0.0454
SSX4	0.8945	1.0957	0.9393	0.8939	0.9558	0.0478

UFM1	0.9021	1.1781	0.9941	0.7491	0.9558	0.0897
ZNF492	0.9362	1.0213	0.9334	0.9325	0.9558	0.0218
LOC283824	0.9229	1.1015	0.9957	0.8033	0.9559	0.0627
SFTPA1B	0.8686	1.0824	0.9666	0.9058	0.9559	0.0468
C19orf45	0.8423	1.1824	0.8738	0.9250	0.9559	0.0774
INVS	0.9377	1.0980	0.9591	0.8287	0.9559	0.0553
FLJ25037	1.0231	1.0349	0.9697	0.7957	0.9559	0.0552
PTP4A3	0.9145	1.0754	1.0199	0.8137	0.9559	0.0580
LOC652790	0.9608	1.0602	0.9573	0.8452	0.9559	0.0439
LTK	0.9077	1.0152	1.0085	0.8921	0.9559	0.0325
FLJ45455	0.9324	1.0615	0.9117	0.9179	0.9559	0.0355
ZCCHC12	0.9302	1.0814	1.0623	0.7497	0.9559	0.0765
LOC642548	0.7846	1.2709	0.9182	0.8499	0.9559	0.1085
NEU1	1.0551	1.2441	0.8463	0.6782	0.9559	0.1232
LOC730051	0.7754	1.2114	1.0061	0.8308	0.9559	0.0983
LOC730173	0.8525	1.2277	0.9095	0.8339	0.9559	0.0920
KIAA0492	0.9594	0.9866	1.0050	0.8728	0.9559	0.0292
LOC653198	0.8251	0.9795	1.0985	0.9207	0.9559	0.0572
LOC1001325	0.7948	1.1479	0.9819	0.8992	0.9559	0.0746
ADHFE1	0.9737	1.0380	0.9537	0.8584	0.9559	0.0371
NPBWR1	0.8408	1.0802	0.9646	0.9382	0.9559	0.0492
TTTY1	0.9310	1.1474	0.9725	0.7730	0.9560	0.0769
LOC1001283	0.9770	1.0322	0.9887	0.8259	0.9560	0.0449
FRMD1	0.8867	1.0533	0.9577	0.9262	0.9560	0.0355
LOC402198	0.9478	1.0231	0.9167	0.9363	0.9560	0.0233
NR4A3	0.9266	1.0535	0.9628	0.8810	0.9560	0.0366
BPI	1.0051	1.0389	0.9550	0.8250	0.9560	0.0470
LOC1001335	0.8110	1.1336	1.0115	0.8678	0.9560	0.0727
CCL22	0.9346	0.9808	1.0026	0.9061	0.9560	0.0219
LOC401677	1.0484	1.0730	0.9574	0.7452	0.9560	0.0745
LOC389791	0.8514	1.0016	0.9890	0.9820	0.9560	0.0351
TTTY2	0.8822	1.0877	0.9322	0.9220	0.9560	0.0452
ST8SIA6	0.9083	0.9742	1.0277	0.9138	0.9560	0.0282
PHKG1	0.9038	1.0581	0.9789	0.8833	0.9560	0.0397
LOC390378	0.8167	1.0606	1.0543	0.8925	0.9560	0.0606
LOC647965	0.9333	1.0028	1.0484	0.8398	0.9561	0.0454
LOC652292	1.0289	1.0239	0.9085	0.8629	0.9561	0.0417
LOC1001324	0.9144	1.0691	0.9552	0.8855	0.9561	0.0403
LOC646691	0.9661	1.0269	0.9562	0.8750	0.9561	0.0312
PPM1J	0.8255	1.0866	0.9926	0.9197	0.9561	0.0553
LOC200261	0.9289	0.9952	1.0606	0.8396	0.9561	0.0472
LOC1001341	0.9752	0.9796	1.0146	0.8550	0.9561	0.0348
LOC1001320	0.8668	1.0296	0.9588	0.9692	0.9561	0.0336
C21orf123	0.8957	1.0561	0.9405	0.9321	0.9561	0.0347
TRDN	0.8732	1.0819	0.9419	0.9275	0.9561	0.0445
FLJ22531	0.8985	1.0657	0.9884	0.8719	0.9561	0.0442

LOC653801	0.8849	1.0497	0.9786	0.9112	0.9561	0.0369
LY75	0.8625	1.1270	0.9228	0.9122	0.9561	0.0585
LOC1001283	0.9511	1.0312	1.0720	0.7701	0.9561	0.0669
CLK4	0.9571	1.1477	0.9783	0.7413	0.9561	0.0833
RHOJ	0.9368	1.0250	0.9637	0.8991	0.9561	0.0265
LOC1001279	0.8312	1.1353	0.9615	0.8965	0.9561	0.0654
LOC649128	0.9116	1.1260	0.9277	0.8592	0.9561	0.0585
FAM75A7	0.8817	1.0215	1.0165	0.9048	0.9561	0.0366
LOC1001337	0.7859	1.1696	0.9666	0.9025	0.9561	0.0804
MGC25181	0.8808	1.0534	0.9729	0.9174	0.9561	0.0376
ZNF536	0.9256	0.9647	1.0224	0.9119	0.9561	0.0248
LOC653855	0.9522	1.1406	0.8871	0.8447	0.9561	0.0654
C19orf31	0.8280	1.3820	0.8691	0.7455	0.9562	0.1443
LOC1001279	0.8576	1.0080	0.9355	1.0235	0.9562	0.0380
ACTL7B	0.8675	1.0971	0.8520	1.0081	0.9562	0.0587
LOC1001296	0.8820	1.0508	1.0559	0.8360	0.9562	0.0569
LOC1001341	0.8293	1.1141	0.9462	0.9351	0.9562	0.0589
FLJ42177	0.8843	1.1143	0.9468	0.8792	0.9562	0.0549
NCRNA0005	0.8947	1.0441	0.9621	0.9239	0.9562	0.0324
LOC1001301	0.9115	1.0787	0.9439	0.8906	0.9562	0.0423
LOC653981	0.8876	1.1888	0.9221	0.8263	0.9562	0.0800
LOC402457	0.8017	1.0522	1.0693	0.9015	0.9562	0.0638
LOC285033	0.8979	1.0537	0.9210	0.9523	0.9562	0.0344
LOC643145	0.9632	1.0434	0.9575	0.8607	0.9562	0.0374
MORN5	0.8989	1.0083	1.0702	0.8475	0.9562	0.0507
SNORD18B	0.8569	1.0998	0.9549	0.9132	0.9562	0.0519
LOC647810	0.9445	1.0528	0.9619	0.8656	0.9562	0.0384
CELA2B	0.8552	1.1768	0.8997	0.8932	0.9562	0.0742
LOC653706	0.8948	1.1209	0.9427	0.8666	0.9562	0.0571
ARMC8	0.8713	1.1452	0.9723	0.8362	0.9562	0.0693
LOC653203	0.9130	1.0085	0.9729	0.9306	0.9563	0.0215
LOC644931	1.0710	1.3133	0.7082	0.7325	0.9563	0.1450
FLJ21767	0.8989	1.0304	1.0029	0.8928	0.9563	0.0353
LOC649107	0.9427	1.0261	1.0182	0.8380	0.9563	0.0437
ARL6	0.9209	1.0588	0.9754	0.8699	0.9563	0.0404
RTF1	0.9343	1.0388	0.9938	0.8583	0.9563	0.0391
KLK6	0.9110	1.0381	1.0227	0.8533	0.9563	0.0445
GPX2	0.8119	1.1363	1.0381	0.8388	0.9563	0.0784
LOC648253	0.9775	1.0291	0.9601	0.8584	0.9563	0.0358
UBXN10	0.8695	1.0863	0.9446	0.9247	0.9563	0.0462
SYNPO2L	0.9807	0.9498	0.9980	0.8967	0.9563	0.0222
LOC338579	0.8246	1.0445	1.0583	0.8979	0.9563	0.0570
PITPNA	0.8463	1.2530	1.0282	0.6976	0.9563	0.1198
ATP13A2	0.9334	1.1810	0.8504	0.8604	0.9563	0.0771
C3orf63	0.8559	1.1234	0.8642	0.9818	0.9563	0.0627
LOC650030	0.8894	1.0982	0.9441	0.8937	0.9563	0.0489

BACH1	0.8370	1.1204	1.0032	0.8646	0.9563	0.0657
PACSIN2	0.9299	1.2956	0.8838	0.7160	0.9563	0.1221
LOC652765	0.8678	1.1224	0.9222	0.9129	0.9563	0.0566
OR13H1	0.9354	1.0131	0.9845	0.8925	0.9564	0.0267
CRISP3	0.8987	0.9679	0.9436	1.0152	0.9564	0.0243
LOC388915	0.9088	1.0756	0.9285	0.9125	0.9564	0.0400
LOC23117	0.8523	1.3482	0.8034	0.8215	0.9564	0.1310
IL15	0.8378	1.0829	1.0114	0.8934	0.9564	0.0556
OMG	0.8636	1.0439	1.0759	0.8421	0.9564	0.0603
LOC643093	0.9083	1.0458	1.0178	0.8536	0.9564	0.0453
SUSD4	0.9468	1.1048	0.9904	0.7835	0.9564	0.0666
CRYBB2	0.9009	1.0393	1.0087	0.8766	0.9564	0.0398
FLJ35379	0.8942	0.9745	1.0290	0.9279	0.9564	0.0293
LOC648699	0.8009	1.0964	1.0848	0.8434	0.9564	0.0780
LOC647191	0.9182	0.9686	1.0733	0.8655	0.9564	0.0443
LOC729595	0.9195	1.0232	0.9429	0.9399	0.9564	0.0229
LOC643100	0.9307	0.8821	0.9756	1.0372	0.9564	0.0330
LOC1001279	0.9114	1.0414	0.9164	0.9565	0.9564	0.0301
ATP6AP1	0.7791	1.3179	0.9955	0.7332	0.9564	0.1334
LOC284297	0.8531	1.0985	0.9892	0.8849	0.9564	0.0556
TNN	0.9623	0.9976	0.9761	0.8897	0.9564	0.0234
ZNF526	0.9385	1.1038	0.9754	0.8082	0.9564	0.0608
LOC641806	0.9490	1.0285	0.9881	0.8602	0.9564	0.0359
RASGRF1	0.9326	1.0231	1.0472	0.8229	0.9564	0.0509
LOC401533	0.8695	0.9645	1.0704	0.9213	0.9564	0.0427
BTBD17	0.9236	0.9983	1.0072	0.8967	0.9564	0.0273
MIR493	0.8848	1.0081	1.0766	0.8563	0.9565	0.0518
SLCO6A1	0.8973	0.9988	1.0565	0.8732	0.9565	0.0430
CREB5	0.8941	1.1236	0.9275	0.8807	0.9565	0.0566
LYPD4	0.7731	1.0295	1.0817	0.9416	0.9565	0.0676
OR8H3	0.9024	1.0670	0.9549	0.9016	0.9565	0.0389
LOC652209	0.9465	1.0803	0.9640	0.8351	0.9565	0.0502
FCN3	0.9572	1.0707	0.9317	0.8663	0.9565	0.0426
LOC645391	0.8247	1.0646	1.0231	0.9135	0.9565	0.0543
HSD3B2	0.8619	1.1174	0.9739	0.8727	0.9565	0.0593
LOC1001291	0.7860	0.9677	1.1355	0.9367	0.9565	0.0717
STAG3	0.8478	1.1058	1.0153	0.8570	0.9565	0.0629
LOC727778	0.8881	0.9309	1.1459	0.8611	0.9565	0.0648
LOC652860	0.8548	1.0771	0.9939	0.9002	0.9565	0.0495
MIR548I4	0.9798	1.0157	0.9635	0.8669	0.9565	0.0318
LOC400221	0.9950	1.0164	0.9509	0.8638	0.9565	0.0338
HELZ	0.8963	1.2783	0.8650	0.7864	0.9565	0.1097
LOC494141	0.8263	1.0763	1.0819	0.8415	0.9565	0.0709
LOC653800	0.8908	0.9514	1.0914	0.8925	0.9565	0.0471
IL32	0.8370	1.0931	1.0189	0.8770	0.9565	0.0599
LOC1001310	0.9264	1.0214	0.9703	0.9080	0.9565	0.0253

LAMB3	0.9422	1.0224	0.9944	0.8671	0.9565	0.0341
LOC1001311	0.9093	1.0457	0.9601	0.9110	0.9565	0.0320
LOC644782	0.9330	0.9998	1.0310	0.8623	0.9565	0.0375
KRT25	0.9987	0.9464	1.0198	0.8614	0.9565	0.0353
EIF3IP1	0.9605	1.0141	1.0091	0.8425	0.9565	0.0399
MIR519E	0.9785	0.9515	0.9571	0.9391	0.9565	0.0082
ZDHHC21	0.8849	1.0268	1.1418	0.7727	0.9565	0.0807
HIST1H2AK	0.9529	1.1241	0.9412	0.8080	0.9566	0.0648
LOC646160	0.9533	1.0213	1.0947	0.7570	0.9566	0.0725
LOC1001308	0.9724	1.0207	0.9931	0.8401	0.9566	0.0401
LOC731042	0.9664	1.0523	1.0027	0.8049	0.9566	0.0535
PSMA4	0.7334	1.5073	0.9897	0.5959	0.9566	0.2009
LOC643325	0.9983	0.9947	0.9614	0.8719	0.9566	0.0294
LOC729327	0.8810	0.9954	1.0348	0.9152	0.9566	0.0354
LOC728895	0.9096	0.9708	1.0053	0.9406	0.9566	0.0205
COX8C	0.9875	0.9930	0.9054	0.9405	0.9566	0.0207
GFRA4	0.9463	0.9892	1.0063	0.8846	0.9566	0.0271
LOC651914	0.8802	0.9669	0.9777	1.0016	0.9566	0.0265
LOC649317	0.8423	0.9904	1.0594	0.9344	0.9566	0.0459
S1PR4	0.9733	0.9990	1.0416	0.8126	0.9566	0.0500
ANKK1	0.9040	1.0850	0.9613	0.8762	0.9566	0.0463
RTTN	0.7993	1.1614	1.0958	0.7699	0.9566	0.1004
LOC654260	0.9215	1.0283	0.9776	0.8990	0.9566	0.0290
LOC1001287	0.8568	1.0659	0.9848	0.9189	0.9566	0.0448
LOC652742	0.9443	1.0174	1.0120	0.8528	0.9566	0.0384
POTEE	0.7689	1.1871	1.1006	0.7700	0.9566	0.1095
SNORD114-2	0.8468	1.0921	0.9344	0.9533	0.9566	0.0508
GRAP2	0.9407	0.9853	0.9342	0.9664	0.9566	0.0118
LOC652582	0.8816	0.9872	0.9879	0.9700	0.9567	0.0254
LOC649212	0.9234	0.9655	1.0415	0.8963	0.9567	0.0317
LOC644978	0.7685	1.1695	0.9725	0.9162	0.9567	0.0830
MORF4	0.8534	1.1007	1.0236	0.8491	0.9567	0.0629
LOC645785	0.9128	1.0928	1.0399	0.7812	0.9567	0.0696
LOC391764	0.8741	1.0480	0.9349	0.9697	0.9567	0.0363
UNK	1.1008	0.9353	0.8785	0.9121	0.9567	0.0494
MIR302F	0.9037	0.9605	1.0245	0.9380	0.9567	0.0254
ITPRIPL2	0.8920	1.0041	0.9482	0.9824	0.9567	0.0244
LOC652680	0.9994	1.0190	0.8845	0.9238	0.9567	0.0316
TTC39B	0.9040	1.0929	0.9294	0.9004	0.9567	0.0459
LOC441920	0.9031	1.0923	1.0097	0.8217	0.9567	0.0594
POR	0.9082	1.1881	0.9496	0.7809	0.9567	0.0851
PP14571	0.7630	1.1785	0.8857	0.9996	0.9567	0.0883
TGFB1	0.9628	0.8945	1.0466	0.9229	0.9567	0.0331
LOC649613	0.9090	1.1287	0.8371	0.9520	0.9567	0.0620
LOC1001330	0.9569	0.9784	1.0375	0.8540	0.9567	0.0382
OR1I1	0.8462	1.0571	1.0102	0.9133	0.9567	0.0475

LOC729021	0.8873	1.0060	0.9915	0.9420	0.9567	0.0269
CPSF4L	0.8546	1.0986	1.0102	0.8634	0.9567	0.0593
ERCC-00108	0.8620	1.0529	0.9855	0.9265	0.9567	0.0408
LOC643009	1.0361	0.8547	1.0729	0.8631	0.9567	0.0570
PPP2R2B	0.8930	1.1226	1.0013	0.8099	0.9567	0.0678
LRRN3	0.9312	1.0451	1.0141	0.8365	0.9567	0.0467
GAB2	0.9215	0.9936	1.0140	0.8979	0.9567	0.0279
LOC730387	0.8339	1.0847	0.9732	0.9351	0.9567	0.0518
KSR2	0.9164	1.0485	1.0010	0.8610	0.9567	0.0420
LOC388965	0.8603	1.0629	1.0037	0.9000	0.9567	0.0465
MIR657	0.7816	1.0503	0.9971	0.9979	0.9567	0.0597
CNKSR2	0.9432	1.0934	0.9090	0.8813	0.9567	0.0473
MIR302E	0.9489	1.0805	0.9587	0.8389	0.9567	0.0494
ING1	0.9246	1.0195	1.0068	0.8761	0.9567	0.0341
LOC643878	0.7050	1.0522	1.0753	0.9945	0.9567	0.0856
LOC390735	0.9211	1.0523	1.0835	0.7701	0.9567	0.0715
LOC390345	0.6732	1.2856	1.2031	0.6651	0.9567	0.1669
LOC649946	0.7666	1.1335	1.2965	0.6304	0.9568	0.1553
FAM22C	1.0038	0.9417	0.9597	0.9219	0.9568	0.0175
CHAT	0.9830	1.0250	0.9311	0.8879	0.9568	0.0299
LOC440570	0.9932	1.0387	0.9181	0.8770	0.9568	0.0364
LOC729399	0.8924	1.1041	0.9077	0.9228	0.9568	0.0495
LOC729832	0.9741	1.0554	1.0004	0.7972	0.9568	0.0558
WEE2	0.7957	1.1277	1.0224	0.8813	0.9568	0.0737
S100A2	1.0408	0.9827	0.8941	0.9095	0.9568	0.0340
LOC375748	0.9289	1.0151	1.0416	0.8414	0.9568	0.0454
LYPD5	0.9238	1.0542	0.9769	0.8723	0.9568	0.0389
NUPL1	0.8606	1.0818	0.9283	0.9564	0.9568	0.0463
MIR874	0.9746	1.0223	0.9599	0.8703	0.9568	0.0318
RGL4	0.8544	1.0262	1.0447	0.9018	0.9568	0.0466
LOC653114	0.9657	1.0438	0.9630	0.8547	0.9568	0.0389
GOLGA8G	0.9357	1.0464	0.9961	0.8490	0.9568	0.0425
LOC648752	0.8345	0.9782	1.0934	0.9212	0.9568	0.0543
AKAP14	0.9279	1.0744	0.9258	0.8992	0.9568	0.0397
LOC729792	0.8944	0.9820	0.9697	0.9813	0.9568	0.0210
LOC654113	0.8856	1.0173	1.0299	0.8945	0.9568	0.0387
LVRN	0.8540	1.0028	1.0093	0.9612	0.9568	0.0359
APOBEC4	0.9014	0.9813	1.1006	0.8441	0.9568	0.0556
LOC728823	0.8068	1.0510	1.3517	0.6178	0.9568	0.1587
LOC1001324	0.9511	1.0409	0.9839	0.8515	0.9568	0.0397
ZNF587	0.9248	0.9858	1.0142	0.9026	0.9568	0.0260
SEC24A	0.8852	1.0639	1.0264	0.8519	0.9568	0.0520
PRKCB1	0.9674	0.9937	1.0159	0.8504	0.9568	0.0368
C7orf11	0.7892	1.3759	0.8745	0.7879	0.9568	0.1411
C4orf38	0.9566	1.0980	0.9187	0.8541	0.9569	0.0516
NCRNA0009	0.9057	0.9684	0.9836	0.9697	0.9569	0.0174

RNF151	0.8680	1.0491	1.0174	0.8930	0.9569	0.0449
MGC13098	0.9444	1.0245	0.9406	0.9180	0.9569	0.0233
LOC649975	0.8458	0.9726	1.1046	0.9043	0.9569	0.0557
FBXO39	0.9407	1.0478	0.8832	0.9558	0.9569	0.0341
LOC652392	1.0032	1.0344	0.9970	0.7929	0.9569	0.0553
PDE1A	0.8832	1.0221	1.0121	0.9101	0.9569	0.0353
UBE2B	0.9015	1.0938	0.9550	0.8771	0.9569	0.0485
LOC642930	0.8978	1.0131	0.9420	0.9745	0.9569	0.0245
RELT	0.9172	1.1005	0.9318	0.8780	0.9569	0.0492
ADAM21	0.9148	1.0243	0.9093	0.9792	0.9569	0.0275
PCOLCE2	0.8835	1.0231	0.9637	0.9572	0.9569	0.0286
OGN	0.9068	1.0684	1.0173	0.8351	0.9569	0.0528
TRIM55	0.9286	1.0285	0.9429	0.9275	0.9569	0.0241
TOX2	0.9251	1.1220	0.9151	0.8654	0.9569	0.0566
SYT1	0.9539	0.9750	0.8951	1.0037	0.9569	0.0230
IKBKAP	0.8915	1.0183	1.0098	0.9080	0.9569	0.0332
MTFMT	0.7613	1.2747	1.0619	0.7297	0.9569	0.1297
CNGA3	0.9314	1.0352	0.9920	0.8691	0.9569	0.0362
LOC644865	0.9915	0.9678	0.9983	0.8699	0.9569	0.0297
CTGLF5	0.8357	1.0338	1.0651	0.8930	0.9569	0.0551
LGI4	0.9040	1.0736	0.9024	0.9477	0.9569	0.0403
LOC642726	0.8910	1.1424	0.9455	0.8489	0.9569	0.0649
SPRR1B	0.8171	1.1525	0.8794	0.9787	0.9569	0.0732
LOC645079	0.8894	1.1175	0.8439	0.9770	0.9569	0.0602
LOH12CR1	0.8705	1.0566	1.0276	0.8730	0.9569	0.0495
JMJD1C	0.8816	1.2380	0.9592	0.7489	0.9569	0.1033
LOC730100	0.9194	0.9931	1.0803	0.8351	0.9570	0.0523
C3orf10	0.9213	1.4789	0.8515	0.5760	0.9570	0.1893
C1orf215	0.8617	1.0243	1.0534	0.8885	0.9570	0.0479
C1orf186	0.9387	1.0185	0.9979	0.8728	0.9570	0.0328
LOC642121	0.9535	0.9742	1.0557	0.8445	0.9570	0.0435
OFCC1	0.8793	1.0894	1.0313	0.8279	0.9570	0.0617
LOC643955	0.8570	1.0549	1.0212	0.8948	0.9570	0.0479
LOC641841	0.8393	1.1359	0.9091	0.9436	0.9570	0.0635
OR4F17	0.9288	1.0537	0.9058	0.9397	0.9570	0.0330
LOC442153	0.8295	1.0728	1.1613	0.7644	0.9570	0.0951
SULF1	0.8265	1.0199	1.0783	0.9033	0.9570	0.0567
CLLU1OS	0.8822	0.9675	1.0762	0.9021	0.9570	0.0437
LOC389816	0.7917	1.2653	1.0249	0.7462	0.9570	0.1195
LOC648362	0.9364	0.9865	0.9938	0.9113	0.9570	0.0199
TAF3	0.8894	1.0621	0.9658	0.9107	0.9570	0.0386
FAM75C1	0.8488	1.1027	1.0194	0.8571	0.9570	0.0624
SLC5A3	0.8854	1.0602	0.9384	0.9440	0.9570	0.0369
LOC650883	0.9549	0.9761	0.9479	0.9491	0.9570	0.0065
LOC1001281	0.9107	1.0004	1.0273	0.8896	0.9570	0.0336
MIR365-1	0.9583	0.9384	0.8931	1.0382	0.9570	0.0303

LOC1001331	0.7474	1.4783	0.8796	0.7228	0.9570	0.1771
OR10AD1	0.9913	0.9490	0.9907	0.8970	0.9570	0.0223
LOC729313	0.9008	0.9727	1.0694	0.8851	0.9570	0.0420
LOC440349	0.8138	1.1023	1.0564	0.8555	0.9570	0.0718
LOC1001334	0.8619	1.0471	1.0677	0.8514	0.9570	0.0581
IL2RA	0.8918	0.9992	1.0119	0.9253	0.9570	0.0289
YIPF7	0.8684	1.0653	1.0118	0.8827	0.9570	0.0484
LOC388923	0.8346	1.1525	0.9247	0.9164	0.9570	0.0682
LAMC2	0.8644	1.0952	1.0050	0.8636	0.9570	0.0568
LOC729754	0.9749	1.0117	0.9807	0.8608	0.9570	0.0331
LOC651979	0.9189	1.1277	0.9774	0.8042	0.9570	0.0673
CHST5	0.9373	1.0585	0.9650	0.8674	0.9571	0.0396
AP1M2	0.8592	1.3043	0.8721	0.7926	0.9571	0.1170
LOC1001345	0.9433	0.9873	1.0615	0.8362	0.9571	0.0471
LOC645580	0.9254	1.0814	0.8854	0.9361	0.9571	0.0428
PLSCR2	0.9000	1.0344	1.0412	0.8527	0.9571	0.0476
CASP9	0.9592	1.0437	0.9886	0.8367	0.9571	0.0438
MIR1229	0.8296	1.0342	0.9881	0.9765	0.9571	0.0443
LOC339047	0.9440	0.9982	0.9903	0.8958	0.9571	0.0237
MAP9	0.9038	0.9818	1.0588	0.8840	0.9571	0.0399
HLA-DOA	0.8869	1.0327	1.0006	0.9082	0.9571	0.0353
C17orf39	0.9272	1.1771	0.9048	0.8193	0.9571	0.0769
TLR10	0.9268	0.9926	0.9579	0.9511	0.9571	0.0136
MLANA	1.0317	1.0092	0.9364	0.8511	0.9571	0.0408
FOXF2	1.0024	0.9699	1.0394	0.8167	0.9571	0.0489
LOC729408	0.9019	1.0805	0.9818	0.8641	0.9571	0.0479
LOC652764	0.8766	0.9927	0.9875	0.9716	0.9571	0.0272
TSTD2	0.8005	1.1800	1.0869	0.7610	0.9571	0.1039
LOC652519	0.8953	1.0005	1.0242	0.9084	0.9571	0.0324
C11orf47	0.9175	1.0534	0.9495	0.9081	0.9571	0.0333
SNORA36A	0.9005	1.1053	0.9797	0.8430	0.9571	0.0568
LOC1001285	0.9112	1.1194	0.9784	0.8195	0.9571	0.0631
GBGT1	0.9060	1.0708	0.9727	0.8790	0.9571	0.0427
NALCN	0.8782	1.0223	1.0447	0.8833	0.9571	0.0443
DLEU2L	0.9439	0.9969	0.9781	0.9096	0.9571	0.0193
SNORD56B	0.8334	1.0255	1.0026	0.9670	0.9571	0.0430
LOC1001323	0.9317	1.0515	0.9951	0.8502	0.9571	0.0432
ANTXRL	0.8597	1.1550	0.9665	0.8474	0.9571	0.0712
ABL1	0.8549	1.2631	0.9581	0.7525	0.9571	0.1103
MOCS3	0.8353	1.2896	0.9872	0.7165	0.9571	0.1239
WDR57	0.6150	1.4929	0.9442	0.7766	0.9572	0.1908
MS4A2	0.9023	1.1655	0.9554	0.8055	0.9572	0.0761
BNC2	0.8879	1.0292	1.0130	0.8985	0.9572	0.0371
ZNF840	0.9037	1.0435	1.0058	0.8756	0.9572	0.0401
LOC654096	0.8991	1.0006	1.0064	0.9226	0.9572	0.0272
EMP1	0.9444	0.9621	0.9995	0.9227	0.9572	0.0163

TFCP2	0.9080	1.1303	1.0394	0.7510	0.9572	0.0825
ZNF471	0.9190	1.1410	0.9269	0.8418	0.9572	0.0642
B3GALNT2	0.6241	1.3449	1.1421	0.7176	0.9572	0.1715
ZNF697	0.7894	1.2952	0.9453	0.7989	0.9572	0.1182
MOGAT3	0.9503	0.9352	1.0228	0.9205	0.9572	0.0227
OR8J1	0.9381	1.0159	1.0111	0.8637	0.9572	0.0359
LOC644491	0.8577	0.9648	1.0098	0.9966	0.9572	0.0345
ST6GAL1	0.8929	1.0641	0.9705	0.9014	0.9572	0.0396
LOC1001347	0.8760	1.0765	0.9255	0.9509	0.9572	0.0427
RAP1BL	0.9613	1.0803	0.9700	0.8172	0.9572	0.0540
LOC652287	0.9307	0.8806	1.0620	0.9557	0.9572	0.0382
LOC389669	0.8897	0.9937	1.0548	0.8908	0.9572	0.0406
COL22A1	0.9419	0.9864	1.0547	0.8459	0.9572	0.0438
PLAC8	0.9125	1.0587	0.9829	0.8749	0.9572	0.0406
LOC646446	0.8531	1.0478	1.0340	0.8940	0.9572	0.0491
LOC652644	0.9638	0.9163	1.0634	0.8854	0.9573	0.0389
LOC375323	0.8734	1.0588	0.9906	0.9062	0.9573	0.0419
TPSAB1	0.7667	1.1536	1.0520	0.8567	0.9573	0.0885
RUNX2	0.9415	1.0001	0.9836	0.9038	0.9573	0.0217
ZSCAN4	0.9077	1.0626	0.9755	0.8832	0.9573	0.0402
THEX1	0.9562	1.0283	0.9806	0.8640	0.9573	0.0345
RIG	0.9506	1.0517	0.9569	0.8699	0.9573	0.0372
LOC653061	0.8753	1.0808	0.9799	0.8932	0.9573	0.0471
LOC727820	0.8040	1.1720	0.9362	0.9169	0.9573	0.0773
WNT9B	0.8001	1.0557	1.0687	0.9047	0.9573	0.0643
LOC729815	0.8770	1.0048	1.0724	0.8749	0.9573	0.0489
SAFB	0.6466	1.6030	0.8479	0.7318	0.9573	0.2191
LOC727962	0.9201	0.9776	1.0344	0.8972	0.9573	0.0308
GM2A	0.9249	1.0936	0.9728	0.8380	0.9573	0.0533
HINT1	0.8137	1.2412	1.0005	0.7739	0.9573	0.1067
ATM	0.8348	1.1739	0.9848	0.8358	0.9573	0.0803
LOC642767	0.8380	1.1038	1.0012	0.8864	0.9573	0.0596
WNT9A	0.8933	1.0897	0.9634	0.8830	0.9573	0.0476
GPX5	0.9569	1.0374	0.9288	0.9063	0.9574	0.0286
C19orf29	0.8873	1.0983	0.9749	0.8690	0.9574	0.0524
CCDC126	0.9939	0.9919	1.0377	0.8060	0.9574	0.0515
SLC16A8	0.8767	1.0275	1.0588	0.8664	0.9574	0.0500
LOC644979	0.9241	1.0727	1.0187	0.8139	0.9574	0.0568
LOC1001296	0.8765	1.0728	0.9622	0.9180	0.9574	0.0423
MAS1L	0.9966	1.0469	0.9482	0.8378	0.9574	0.0447
LOC646146	0.9071	1.0714	0.9853	0.8657	0.9574	0.0454
LOC391037	0.9089	0.9986	1.0170	0.9050	0.9574	0.0294
TMED8	0.8852	1.0575	0.9783	0.9086	0.9574	0.0388
GAS1	0.9373	1.0936	1.0108	0.7879	0.9574	0.0649
AQR	0.9282	1.2289	0.9044	0.7680	0.9574	0.0972
LOC255167	0.9042	1.0734	1.0834	0.7686	0.9574	0.0752

LOC643403	0.9406	0.9720	1.0475	0.8695	0.9574	0.0369
IL2	0.7995	1.1296	1.1022	0.7983	0.9574	0.0917
LOC126520	0.9065	1.0142	1.0067	0.9022	0.9574	0.0307
LOC1001322	0.9319	0.9723	1.0366	0.8888	0.9574	0.0314
LOC652647	0.8303	0.9997	1.0561	0.9437	0.9574	0.0482
GRID2	0.9696	0.9626	0.9988	0.8987	0.9574	0.0211
CDKN2BAS	0.8305	1.0855	1.0378	0.8760	0.9574	0.0616
SNORD32B	0.9190	1.0987	1.0379	0.7741	0.9574	0.0716
MAPKSP1	0.8906	1.0744	0.9829	0.8818	0.9574	0.0452
MCC	0.8417	1.1738	0.9114	0.9028	0.9574	0.0738
FAM19A3	0.9432	1.0612	0.9544	0.8709	0.9574	0.0392
LOC647328	0.9017	1.0316	1.0041	0.8924	0.9574	0.0354
LOC643424	1.0023	1.0123	0.9781	0.8371	0.9574	0.0408
PLCG2	0.9147	1.1156	0.9750	0.8245	0.9574	0.0611
LOC730024	0.9977	1.0625	0.9645	0.8051	0.9574	0.0547
ZNF280B	0.8529	1.0710	1.0216	0.8843	0.9574	0.0527
GMPR	0.9110	1.1819	0.8338	0.9032	0.9575	0.0768
ASB18	0.9320	1.0859	0.9158	0.8962	0.9575	0.0434
CCKAR	0.9176	0.9900	1.0025	0.9198	0.9575	0.0225
GUCY2F	0.9812	1.0076	0.9818	0.8593	0.9575	0.0333
LOC1001329	0.8735	1.1365	0.8966	0.9233	0.9575	0.0605
GPX8	0.9821	1.0910	0.9178	0.8390	0.9575	0.0533
NXF3	1.0247	0.9565	0.9840	0.8647	0.9575	0.0340
CEP350	0.7270	1.3930	0.8611	0.8488	0.9575	0.1483
CWC22	0.9792	1.0218	0.9753	0.8536	0.9575	0.0362
LOC645090	0.9059	1.0936	1.0989	0.7316	0.9575	0.0877
OR2T6	0.9141	1.0240	0.9991	0.8928	0.9575	0.0319
LOC645302	0.8577	1.1216	1.0033	0.8474	0.9575	0.0653
LOC1001329	0.8364	1.1338	0.9285	0.9313	0.9575	0.0628
SLC23A3	0.8692	1.0580	0.9390	0.9638	0.9575	0.0390
ZNF556	0.7769	1.1151	1.0638	0.8742	0.9575	0.0794
PCIF1	0.8905	1.0688	0.9980	0.8728	0.9575	0.0463
LOC339970	0.9913	1.1747	0.8348	0.8292	0.9575	0.0816
LOC653178	0.7713	1.0884	1.0649	0.9054	0.9575	0.0742
LOC1001326	0.8743	1.0943	0.9945	0.8670	0.9575	0.0542
LOC1001327	0.8981	1.0937	0.9764	0.8620	0.9575	0.0513
LOC647286	0.9446	1.0506	0.9672	0.8678	0.9575	0.0376
CDC14B	0.8799	1.2245	0.9335	0.7922	0.9575	0.0936
EEA1	0.8625	1.0735	1.0464	0.8478	0.9576	0.0595
ABCC3	0.9629	1.0843	0.8945	0.8886	0.9576	0.0455
LOC651219	0.9145	0.9878	1.0312	0.8967	0.9576	0.0315
FLJ90036	0.8109	1.1278	1.0108	0.8808	0.9576	0.0702
CEP97	0.8872	1.0478	1.0293	0.8659	0.9576	0.0471
ROBO1	0.9082	1.0000	1.0250	0.8971	0.9576	0.0322
LYPD6	0.9214	0.9645	1.0207	0.9237	0.9576	0.0232
ZNF782	0.8816	1.0174	1.0545	0.8768	0.9576	0.0459

LOC348840	0.9676	1.0716	0.9683	0.8229	0.9576	0.0511
LOC442064	0.9670	1.0912	0.9726	0.7995	0.9576	0.0600
DCHS2	0.9797	1.0459	0.9366	0.8681	0.9576	0.0373
ATRN	1.0050	1.1720	0.8922	0.7612	0.9576	0.0871
MIR944	0.9408	1.0204	0.9895	0.8796	0.9576	0.0307
C15orf51	0.8279	1.0336	1.0321	0.9367	0.9576	0.0488
LOC729603	0.7946	1.3119	1.0175	0.7063	0.9576	0.1350
LOC646194	0.7949	1.2368	0.9846	0.8142	0.9576	0.1023
LOC1001335	0.9720	0.9462	1.1251	0.7871	0.9576	0.0692
LOC652904	0.8354	1.1050	0.9655	0.9244	0.9576	0.0561
LOC647920	0.9628	1.0351	0.9822	0.8504	0.9576	0.0389
HIST1H2AB	0.9227	0.9507	1.0306	0.9265	0.9576	0.0251
KCNQ1OT1	0.9142	0.9418	1.1345	0.8401	0.9576	0.0627
C13orf7	0.9851	0.9493	0.9166	0.9796	0.9576	0.0158
TFAP2B	0.8653	1.0600	1.0177	0.8876	0.9576	0.0479
LOC728763	0.8452	1.0611	1.0420	0.8823	0.9576	0.0549
LOC648733	0.8794	1.0523	1.0371	0.8618	0.9576	0.0505
LOC642903	0.9187	1.0042	1.0827	0.8250	0.9577	0.0555
LOC1001319	0.8454	1.0746	0.9810	0.9296	0.9577	0.0480
LEFTY1	0.9092	1.0987	0.9971	0.8257	0.9577	0.0586
TMC3	0.8948	1.0811	0.8917	0.9632	0.9577	0.0443
LOC1001303	0.9099	1.0661	0.9664	0.8884	0.9577	0.0397
PWRN2	0.9857	1.0902	0.8768	0.8782	0.9577	0.0510
FAM122A	0.8037	1.2927	0.9161	0.8184	0.9577	0.1144
LOC1001343	0.9404	1.0820	0.9006	0.9078	0.9577	0.0423
FAM65C	0.8485	1.1310	0.9424	0.9090	0.9577	0.0609
LOC1001310	0.8739	1.0496	0.9109	0.9965	0.9577	0.0399
ZFP112	0.9773	1.1180	0.9411	0.7945	0.9577	0.0665
LOC729259	0.8734	1.1510	1.0233	0.7832	0.9577	0.0812
TAF4B	0.9504	0.9527	1.0169	0.9109	0.9577	0.0220
MAP3K13	0.7783	1.1001	1.1417	0.8107	0.9577	0.0948
YY1AP1	0.9083	1.3773	0.9021	0.6432	0.9577	0.1529
CTHRC1	0.9129	0.9999	1.0512	0.8670	0.9577	0.0416
LOC1001337	0.8830	1.0450	1.0640	0.8389	0.9577	0.0567
TNNT2	0.9439	1.0580	0.9422	0.8868	0.9577	0.0360
CTAGE5	0.9088	1.0713	0.9789	0.8719	0.9577	0.0439
LOC1001333	0.9481	1.0498	0.9561	0.8771	0.9577	0.0354
LOC1001292	0.8902	0.9553	1.0275	0.9581	0.9577	0.0280
AKT2	0.8853	1.0128	1.0523	0.8806	0.9578	0.0439
LOC651850	0.8742	0.9466	1.0416	0.9687	0.9578	0.0345
ALK	0.8548	1.1106	0.9392	0.9265	0.9578	0.0542
LOC729350	0.9139	1.0530	0.9812	0.8830	0.9578	0.0378
LOC642249	0.9313	1.0161	1.0346	0.8491	0.9578	0.0426
CTSL3	0.8290	1.1284	0.9844	0.8894	0.9578	0.0653
LOC652490	0.8714	1.0696	1.0542	0.8360	0.9578	0.0606
PRDM14	0.9244	0.9767	1.0154	0.9147	0.9578	0.0235

CDH24	0.9647	1.0968	0.9237	0.8459	0.9578	0.0525
LOC653576	1.0726	0.8653	0.9843	0.9091	0.9578	0.0455
KRT20	0.9456	1.0867	0.9356	0.8632	0.9578	0.0467
LOC729954	0.9373	1.0337	1.0030	0.8572	0.9578	0.0391
LOC283663	0.9595	1.0937	0.9579	0.8202	0.9578	0.0558
SPN	0.9329	1.0024	0.9742	0.9216	0.9578	0.0187
FGF16	0.8676	1.0435	1.0519	0.8682	0.9578	0.0519
PNMA5	0.9528	1.0238	0.9576	0.8971	0.9578	0.0259
FLJ14100	0.8828	1.0903	0.9875	0.8707	0.9578	0.0514
LOC220594	0.8898	1.0292	0.9774	0.9349	0.9578	0.0298
CRY1	0.9032	1.2196	0.9731	0.7354	0.9578	0.1005
LOC732162	0.9478	0.9872	0.9689	0.9273	0.9578	0.0130
DISC2	0.8755	1.0326	1.0486	0.8746	0.9578	0.0479
SH3BGR	0.9169	1.1192	0.9717	0.8235	0.9578	0.0619
POM121L2	0.7942	1.0827	1.0867	0.8678	0.9578	0.0748
SPAG1	0.8617	1.1144	0.9879	0.8673	0.9578	0.0598
LOC149224	0.8484	1.0703	1.0963	0.8164	0.9578	0.0729
GBP1	0.9415	0.9933	0.9723	0.9243	0.9579	0.0154
OR10A3	0.8831	0.9847	1.0432	0.9204	0.9579	0.0354
LOC439914	0.9611	1.0643	1.1312	0.6749	0.9579	0.1006
LOC729494	0.8920	1.0554	0.9400	0.9441	0.9579	0.0346
LOC644843	0.9910	1.0611	0.9429	0.8365	0.9579	0.0472
LOC200493	0.9626	1.0383	0.9536	0.8770	0.9579	0.0330
LOC647841	0.6869	1.1078	1.0332	1.0036	0.9579	0.0930
NECAB1	0.9604	1.0263	1.0842	0.7607	0.9579	0.0704
LOC729173	0.9625	1.0660	0.9315	0.8716	0.9579	0.0407
AIM2	0.9198	0.9984	0.9752	0.9382	0.9579	0.0177
RAMP2	0.8376	1.0663	0.9752	0.9524	0.9579	0.0470
PRSS27	0.9972	1.0177	0.9677	0.8490	0.9579	0.0377
ZNF585B	0.8516	1.1152	0.9947	0.8701	0.9579	0.0613
LOC391358	0.9402	1.0321	0.9693	0.8901	0.9579	0.0297
MIR601	0.9016	1.0708	0.9299	0.9294	0.9579	0.0382
PABPC1L2B	0.9056	1.0214	0.9875	0.9172	0.9579	0.0278
AADACL2	0.9640	0.9950	0.9340	0.9387	0.9579	0.0140
LOC651643	0.8217	1.1466	1.0075	0.8559	0.9579	0.0747
MIR614	0.8854	1.0874	1.0475	0.8114	0.9579	0.0655
KIAA1600	0.8107	1.1730	1.1039	0.7442	0.9579	0.1060
LOC647308	0.8818	1.0299	1.0227	0.8974	0.9580	0.0396
LOC285407	0.9092	1.1264	0.9909	0.8053	0.9580	0.0678
LOC653461	0.9638	1.0047	0.9460	0.9174	0.9580	0.0183
L1TD1	0.8745	1.0702	0.9930	0.8942	0.9580	0.0455
ZSCAN5B	0.8675	1.0424	1.0002	0.9219	0.9580	0.0391
LOC653663	0.8868	1.0376	1.0140	0.8935	0.9580	0.0395
UNG2	0.9493	1.0605	0.9086	0.9135	0.9580	0.0354
OLIG3	0.9354	1.0833	0.9610	0.8523	0.9580	0.0478
DLL1	0.9404	1.1259	0.9041	0.8616	0.9580	0.0582

LOC643640	0.9232	1.0267	0.9539	0.9282	0.9580	0.0239
IL17A	0.8775	1.0662	0.9766	0.9117	0.9580	0.0415
LOC1001296	0.9127	0.9309	1.1041	0.8842	0.9580	0.0496
LOC644268	0.9390	1.0858	0.9887	0.8186	0.9580	0.0556
LOC1001321	0.8237	1.0691	1.0037	0.9356	0.9580	0.0524
ACTG2	0.8667	1.0449	1.1105	0.8100	0.9580	0.0713
LOC340094	0.8674	0.9550	1.0655	0.9442	0.9580	0.0408
LOC647206	1.0223	0.8584	1.0801	0.8713	0.9580	0.0552
XAGE2B	0.8690	1.1972	0.8830	0.8829	0.9580	0.0798
ATRNL1	0.7981	1.1396	0.9966	0.8978	0.9580	0.0728
LOC644415	0.8528	1.1119	0.9470	0.9204	0.9580	0.0550
LOC389458	0.9236	1.0857	0.9319	0.8911	0.9580	0.0435
LOC400590	0.9345	1.0742	0.9754	0.8481	0.9580	0.0469
C3orf74	0.8504	1.1036	1.0402	0.8379	0.9581	0.0671
ALG12	0.9294	1.0826	0.9676	0.8526	0.9581	0.0479
AIG1	0.7500	1.1365	1.2181	0.7276	0.9581	0.1278
INSIG2	0.7846	1.4508	0.9837	0.6132	0.9581	0.1809
IQSEC3	0.9040	1.1392	0.9295	0.8595	0.9581	0.0621
TEX261	0.8919	1.2494	1.0366	0.6544	0.9581	0.1250
LOC649676	0.8001	1.1551	1.0195	0.8577	0.9581	0.0804
LOC727997	0.9175	0.9400	1.1299	0.8449	0.9581	0.0608
LOC387927	0.8906	1.1630	0.9846	0.7942	0.9581	0.0786
CEMP1	0.8641	1.0406	0.9886	0.9392	0.9581	0.0376
MARCKS	0.9773	1.1075	1.0300	0.7177	0.9581	0.0845
LOC442582	0.7605	1.1042	1.0829	0.8848	0.9581	0.0823
GJB1	0.8349	1.0540	1.0268	0.9167	0.9581	0.0507
DIRAS3	0.9568	1.0317	0.9856	0.8583	0.9581	0.0367
SIAH3	0.8907	1.0640	1.0199	0.8579	0.9581	0.0497
DOCK5	0.9856	1.0159	0.9627	0.8682	0.9581	0.0319
LOC653355	0.8777	1.1220	0.9841	0.8488	0.9581	0.0619
LOC1001345	0.9262	1.0330	1.0231	0.8503	0.9581	0.0433
LOC649060	0.9350	0.9767	1.0804	0.8405	0.9581	0.0497
C20orf51	0.9023	1.0603	0.9099	0.9601	0.9581	0.0364
TAS2R9	0.9222	1.1408	0.9076	0.8620	0.9581	0.0622
HS3ST6	0.9197	1.0042	1.0697	0.8390	0.9581	0.0502
ZNF364	0.7749	1.2638	0.9499	0.8440	0.9581	0.1080
LOC642826	0.9437	1.0348	1.0208	0.8333	0.9581	0.0462
OR13G1	0.9159	0.9521	1.0541	0.9106	0.9582	0.0333
LOC1001338	0.8896	1.1430	0.9058	0.8942	0.9582	0.0617
LOC643844	0.9035	1.0850	1.0297	0.8145	0.9582	0.0611
LOC1001308	0.8602	1.1182	0.9837	0.8705	0.9582	0.0602
LOC1001334	0.8997	1.0362	0.9928	0.9040	0.9582	0.0337
ZNF273	0.8643	1.0083	1.0115	0.9486	0.9582	0.0345
ANKRD43	0.9900	1.0069	1.0191	0.8167	0.9582	0.0475
LOC642628	0.8510	1.1591	0.9498	0.8729	0.9582	0.0702
LOC644447	0.8311	1.0705	0.9653	0.9659	0.9582	0.0491

LOC647028	0.9706	1.0105	1.0031	0.8486	0.9582	0.0376
LOC729856	0.9756	1.0217	0.9197	0.9158	0.9582	0.0252
ATP8B3	0.9793	1.0015	0.9377	0.9144	0.9582	0.0197
LOC728991	0.8745	1.0403	1.0222	0.8959	0.9582	0.0425
LOC648039	0.8280	1.0342	0.9662	1.0046	0.9582	0.0456
HEATR6	0.8861	1.3239	0.8424	0.7805	0.9582	0.1238
SMCHD1	0.9026	1.0905	1.0143	0.8254	0.9582	0.0587
PAX4	0.8671	1.1353	0.9321	0.8984	0.9582	0.0605
ADAM8	0.9392	1.0004	1.0237	0.8696	0.9582	0.0345
VEGFC	0.9735	1.0156	0.9983	0.8455	0.9582	0.0386
MAGEB5	1.0567	0.7835	0.9900	1.0027	0.9582	0.0600
LOC728199	0.9121	1.0163	1.0422	0.8623	0.9582	0.0426
LOC651410	1.0207	0.9404	0.9481	0.9237	0.9582	0.0214
CTGLF1	1.0005	1.0089	1.0149	0.8088	0.9582	0.0499
LRRC24	0.9449	1.0669	1.0503	0.7708	0.9582	0.0681
LOC441931	0.8942	1.0672	1.0776	0.7941	0.9583	0.0690
LOC390669	0.8941	1.0226	0.9768	0.9395	0.9583	0.0273
WDR61	0.8293	1.3260	0.9705	0.7072	0.9583	0.1339
LOC647197	0.9582	0.9947	0.9788	0.9014	0.9583	0.0204
PRKAG1	0.9058	1.2797	0.9468	0.7008	0.9583	0.1199
LOC1001322	0.8005	1.1082	1.0009	0.9235	0.9583	0.0648
LOC643142	0.9990	1.0125	0.9200	0.9016	0.9583	0.0278
CD248	0.8436	1.0190	1.0419	0.9287	0.9583	0.0454
PER4	0.9622	1.0310	0.9841	0.8559	0.9583	0.0370
FGFR1OP2	0.9298	1.1504	0.9655	0.7875	0.9583	0.0747
LOC51149	0.9733	1.0714	0.9826	0.8059	0.9583	0.0554
BCAT1	0.8375	1.0578	1.0693	0.8687	0.9583	0.0611
FEZF2	0.8592	1.0687	1.0680	0.8375	0.9583	0.0637
ZNF862	0.9192	1.1906	0.9289	0.7947	0.9583	0.0832
TIMP3	0.9811	0.9992	0.9265	0.9265	0.9583	0.0187
C1orf147	0.8741	1.1937	0.9728	0.7928	0.9583	0.0867
KLK14	0.8953	1.0433	1.0022	0.8927	0.9584	0.0381
LOC649327	0.9856	0.9969	0.9602	0.8907	0.9584	0.0238
ERAP2	0.8510	1.1949	0.9324	0.8552	0.9584	0.0810
LOC729485	0.8719	1.0920	0.9401	0.9295	0.9584	0.0470
FAM172A	0.8502	1.1892	1.0119	0.7822	0.9584	0.0908
LOC651957	0.8712	1.0888	1.0993	0.7742	0.9584	0.0808
WFIKKN2	1.0096	0.9739	0.9863	0.8636	0.9584	0.0324
TMPRSS4	0.9301	1.0523	0.9898	0.8613	0.9584	0.0409
ZDHHC1	0.9353	1.0905	0.9426	0.8651	0.9584	0.0474
LOC729389	0.9862	1.0812	0.8916	0.8745	0.9584	0.0477
ALPL	0.9357	1.0086	0.9858	0.9033	0.9584	0.0238
CLEC4GP1	0.9931	1.0195	0.9483	0.8727	0.9584	0.0321
ACOT12	0.8799	1.0247	1.0425	0.8865	0.9584	0.0436
ZRSR2	0.9728	1.0256	0.9593	0.8758	0.9584	0.0310
S100A3	0.9250	1.0403	0.9206	0.9477	0.9584	0.0279

LOC1001328	0.9369	0.9529	0.9991	0.9446	0.9584	0.0140
CELSR1	0.9512	1.0592	0.9258	0.8974	0.9584	0.0354
IGHMBP2	0.8498	1.1392	0.9456	0.8990	0.9584	0.0634
LOC649583	0.8651	1.0613	1.0390	0.8682	0.9584	0.0532
LOC1001314	0.9119	1.0359	0.9250	0.9608	0.9584	0.0278
OAS3	0.8909	1.0351	0.9695	0.9381	0.9584	0.0302
LOC649389	0.9522	1.0432	0.9228	0.9155	0.9584	0.0293
YAP1	0.9603	1.0100	1.1394	0.7239	0.9584	0.0868
PRKAR2A	0.7518	1.4092	0.8706	0.8022	0.9584	0.1522
LOC1001317	0.9338	1.0483	0.9600	0.8915	0.9584	0.0331
KIAA0773	0.7478	1.0915	1.0167	0.9778	0.9584	0.0741
LOC440291	1.0462	1.0308	0.9078	0.8490	0.9585	0.0478
CC2D1B	0.8650	1.0801	0.9495	0.9393	0.9585	0.0447
ANAPC4	0.8273	1.3785	0.9617	0.6664	0.9585	0.1525
C11orf36	0.9370	1.1813	0.8303	0.8853	0.9585	0.0774
LOC647450	0.8454	1.0685	1.0388	0.8811	0.9585	0.0558
LOC653326	0.9080	1.0078	1.0053	0.9127	0.9585	0.0278
LOC1001345	0.9163	1.1716	0.8538	0.8922	0.9585	0.0722
SNORD107	0.8723	0.9603	1.0557	0.9456	0.9585	0.0377
LOC1001323	0.7907	1.0513	1.0372	0.9547	0.9585	0.0599
GABBR2	0.8479	1.0576	1.0022	0.9263	0.9585	0.0456
GPR35	0.8426	1.0007	1.0115	0.9790	0.9585	0.0392
LOC388476	0.9224	1.1215	0.9834	0.8066	0.9585	0.0656
LOC387745	0.9125	1.2160	0.9405	0.7649	0.9585	0.0941
LOC647753	0.9021	1.0642	0.9901	0.8775	0.9585	0.0427
LOC728308	0.9447	1.0914	0.9562	0.8416	0.9585	0.0513
CFC1	0.7776	1.0548	0.9884	1.0133	0.9585	0.0618
PTPRJ	0.9286	1.1041	0.9538	0.8475	0.9585	0.0536
LOC652298	0.9398	1.0218	0.9474	0.9250	0.9585	0.0216
OR2S2	0.9463	1.0469	0.9962	0.8446	0.9585	0.0432
LOC647989	0.9118	0.9596	1.0146	0.9482	0.9585	0.0213
LOC646357	0.9796	0.9847	1.0251	0.8447	0.9585	0.0393
TAS2R10	0.8636	1.0864	0.9547	0.9295	0.9585	0.0467
MS4A6E	0.9107	1.0115	1.0412	0.8707	0.9585	0.0405
LOC648358	0.9732	1.0206	0.9757	0.8645	0.9585	0.0332
TLR3	0.9275	1.0180	1.0137	0.8749	0.9585	0.0348
ERCC-00058	0.8504	1.1496	0.9171	0.9170	0.9585	0.0656
RREB1	0.8874	1.1582	0.9765	0.8121	0.9585	0.0745
LOC133874	0.8688	1.1273	1.0554	0.7827	0.9585	0.0800
CPLX1	0.9221	1.2384	0.9502	0.7235	0.9586	0.1060
IFNA1	0.8996	0.9898	1.0679	0.8769	0.9586	0.0438
ELFN1	0.9863	1.0202	1.0056	0.8222	0.9586	0.0460
OR52B6	0.9665	1.0696	0.9675	0.8307	0.9586	0.0490
LOC652215	0.8513	0.9639	1.0285	0.9906	0.9586	0.0381
C14orf177	0.8982	1.0234	0.9681	0.9446	0.9586	0.0260
AGTR2	0.7959	1.1030	1.0329	0.9025	0.9586	0.0683

LOC646256	0.8903	1.1419	0.9338	0.8684	0.9586	0.0626
IDI1	0.8979	1.0774	1.1893	0.6698	0.9586	0.1134
LOC653056	0.9441	1.0390	0.9477	0.9036	0.9586	0.0286
LOC652113	0.8861	1.0489	1.0848	0.8147	0.9586	0.0646
KIAA2022	0.8449	1.0584	0.9948	0.9364	0.9586	0.0454
ZNF157	0.9503	1.0115	0.9448	0.9279	0.9586	0.0183
PRB2	0.8974	1.0403	1.0114	0.8853	0.9586	0.0393
IQCD	1.0309	1.0016	1.0082	0.7938	0.9586	0.0553
LOC649910	0.8777	1.1019	1.0015	0.8534	0.9587	0.0577
LOC1001321	0.8379	1.0689	1.0263	0.9016	0.9587	0.0537
TFPI2	0.9041	1.0621	1.0416	0.8268	0.9587	0.0562
TM4SF19	0.9500	1.0531	0.9607	0.8709	0.9587	0.0373
LOC652850	1.1499	1.0460	0.8005	0.8383	0.9587	0.0835
FLJ26850	0.9019	1.0435	1.0504	0.8388	0.9587	0.0526
PSMA6	0.7826	1.2562	0.9976	0.7984	0.9587	0.1106
LOC644008	0.9171	1.1217	0.9233	0.8727	0.9587	0.0555
LOC653423	0.8938	1.0113	1.0596	0.8700	0.9587	0.0457
LAT1-3TM	0.9190	1.0522	0.9507	0.9129	0.9587	0.0322
LOC642037	0.9128	1.0635	0.9998	0.8587	0.9587	0.0454
BPGM	0.9199	1.1296	0.9860	0.7992	0.9587	0.0689
LOC1001284	0.9888	0.9311	0.9724	0.9425	0.9587	0.0133
LOC647741	0.8497	1.0557	1.0019	0.9275	0.9587	0.0448
HIST1H2AD	0.9289	1.0914	0.9005	0.9140	0.9587	0.0446
FAM163B	0.8499	1.0143	0.9359	1.0349	0.9587	0.0421
LOC651936	0.8549	1.0561	1.0505	0.8734	0.9587	0.0547
LOC648132	0.8765	0.9705	1.0224	0.9655	0.9587	0.0303
OR6A2	0.9323	0.9701	1.0151	0.9174	0.9587	0.0218
LYSMD3	0.8734	1.0513	1.0654	0.8449	0.9587	0.0579
ERCC-00111	0.9026	1.1481	0.9382	0.8460	0.9587	0.0659
SFI1	0.9680	0.9323	1.0110	0.9237	0.9587	0.0199
GLYCTK	0.7854	1.0524	1.1048	0.8923	0.9587	0.0734
ASB15	0.9523	0.9772	1.0110	0.8945	0.9587	0.0246
LOC390213	0.9289	0.9599	1.0732	0.8729	0.9587	0.0422
LOC647566	0.9577	1.0163	1.0271	0.8338	0.9587	0.0443
LOC729438	0.8828	1.1112	0.9890	0.8520	0.9588	0.0587
ENG	0.9705	1.0167	0.9853	0.8624	0.9588	0.0335
CNTLN	0.9802	1.1539	0.8418	0.8591	0.9588	0.0720
C2orf70	0.9073	0.9748	0.9858	0.9671	0.9588	0.0176
LOC1001292	0.8374	1.0387	1.0235	0.9354	0.9588	0.0464
IFI27	0.9451	1.1042	0.9966	0.7892	0.9588	0.0655
LOC346950	0.7029	1.0426	1.3024	0.7871	0.9588	0.1354
MORC1	0.8562	1.1102	1.0512	0.8175	0.9588	0.0718
OR2T1	0.9483	1.0374	0.9091	0.9404	0.9588	0.0275
FAM49A	0.8389	1.1577	1.0330	0.8056	0.9588	0.0831
LOC643809	0.9704	1.0750	0.8942	0.8955	0.9588	0.0426
POLI	0.8680	1.0817	0.9467	0.9388	0.9588	0.0446

LOC651493	0.8580	1.0411	0.9861	0.9500	0.9588	0.0385
LOC1001342	0.9549	0.9986	1.0077	0.8740	0.9588	0.0305
LOC648921	0.9900	1.1643	0.9293	0.7516	0.9588	0.0851
RBM46	0.8928	1.1294	0.8972	0.9159	0.9588	0.0571
LOC645563	0.7617	1.1568	0.9853	0.9315	0.9588	0.0814
JMJD2C	0.8767	1.0593	0.9420	0.9573	0.9588	0.0378
BTNL8	0.8719	1.1086	0.9464	0.9084	0.9588	0.0522
PDZD11	0.8448	1.0893	1.0077	0.8935	0.9588	0.0553
PFDN2	0.8870	1.2641	0.8824	0.8018	0.9588	0.1036
LOC651122	0.9295	1.0153	1.0126	0.8780	0.9588	0.0335
CROCCL2	0.9137	0.9532	0.9752	0.9932	0.9588	0.0171
ASPRV1	0.9416	1.0126	1.0254	0.8557	0.9588	0.0390
SIGLEC15	0.8519	1.1058	0.8934	0.9843	0.9588	0.0562
OR10K2	0.8870	1.0533	1.0084	0.8867	0.9588	0.0426
ADRBK2	0.8938	1.0596	0.8957	0.9863	0.9588	0.0399
LOC649813	0.9415	0.9899	0.9680	0.9361	0.9589	0.0125
ZNF416	0.8733	1.1297	0.9931	0.8393	0.9589	0.0658
FGF18	0.7931	1.2662	0.9400	0.8362	0.9589	0.1070
COX11	0.8863	1.0220	0.9925	0.9346	0.9589	0.0302
C6orf145	0.9594	0.9804	0.9949	0.9008	0.9589	0.0207
LOC1001296	0.9053	1.0319	0.9793	0.9190	0.9589	0.0292
LOC1001294	0.9514	1.1427	0.9814	0.7600	0.9589	0.0785
HRH3	0.9074	1.0162	1.0569	0.8551	0.9589	0.0468
LOC1001333	0.8502	0.9919	1.0602	0.9333	0.9589	0.0446
OPN1MW	0.9083	1.0461	0.9929	0.8883	0.9589	0.0369
LOC731158	0.9453	1.0998	0.9430	0.8475	0.9589	0.0522
MYH8	0.8271	1.0237	1.0011	0.9838	0.9589	0.0447
LLGL2	0.9002	1.0676	0.9602	0.9077	0.9589	0.0386
LYPD1	0.8756	1.0793	1.0007	0.8801	0.9589	0.0495
PLCL4	0.8991	1.0731	1.0168	0.8468	0.9589	0.0521
GALM	1.0229	1.1184	0.9412	0.7532	0.9589	0.0776
LACRT	0.9498	0.9833	0.9146	0.9881	0.9589	0.0171
LOC389342	0.8440	1.0770	1.0430	0.8718	0.9589	0.0590
ERCC2	0.8315	1.1780	1.0009	0.8253	0.9589	0.0836
LOC642133	0.8421	1.0719	0.9938	0.9279	0.9589	0.0488
LPL	0.8279	0.9794	1.0418	0.9867	0.9589	0.0458
LOC652164	0.8447	0.9896	1.1532	0.8483	0.9589	0.0730
ERCC-00154	0.9114	1.0238	1.0409	0.8598	0.9589	0.0438
LOC284672	0.9618	0.9877	0.9738	0.9125	0.9590	0.0164
LOC651404	0.9687	0.9489	0.9933	0.9249	0.9590	0.0145
MIR592	0.9734	1.0689	0.8645	0.9291	0.9590	0.0429
ABI1	0.8316	1.1346	1.0390	0.8306	0.9590	0.0763
LOC1001282	0.8637	1.0237	1.0020	0.9465	0.9590	0.0357
ALDH3B1	0.9273	1.0650	0.9588	0.8848	0.9590	0.0385
LOC441239	0.9258	1.0407	1.0538	0.8155	0.9590	0.0558
LOC441874	0.9620	0.9589	1.0969	0.8181	0.9590	0.0569

AMD1	0.7017	1.4367	1.0233	0.6742	0.9590	0.1779
NPHS1	0.9050	0.9510	1.0611	0.9189	0.9590	0.0354
REG4	0.9374	0.9747	1.0427	0.8811	0.9590	0.0339
LOC650695	0.9061	0.9782	1.0403	0.9114	0.9590	0.0317
ALDOC	0.8324	1.3829	0.8642	0.7564	0.9590	0.1431
PTPRB	0.9314	1.1001	0.8930	0.9114	0.9590	0.0477
SLC22A7	0.9460	1.0262	0.9703	0.8936	0.9590	0.0275
DEFB130	0.9866	0.9823	0.9598	0.9073	0.9590	0.0182
XAGE5	0.9300	0.9968	1.0065	0.9028	0.9590	0.0253
SLAMF1	0.9370	0.9963	0.9239	0.9788	0.9590	0.0171
KRTAP10-11	0.8691	1.0349	1.1178	0.8142	0.9590	0.0707
SPATA21	0.9444	1.0253	0.9586	0.9078	0.9590	0.0246
LOC439936	0.8137	1.0850	1.0420	0.8954	0.9590	0.0632
SLC13A4	0.9522	1.0321	0.9621	0.8898	0.9590	0.0291
LOC648302	0.9763	1.0007	0.9041	0.9549	0.9590	0.0206
LOC728343	0.8887	1.0793	0.9796	0.8886	0.9590	0.0455
MEPE	0.9824	0.9189	1.0128	0.9221	0.9590	0.0231
LOC727992	0.9952	0.9523	0.9838	0.9049	0.9590	0.0202
LOC731085	0.9538	1.0121	0.9482	0.9221	0.9591	0.0190
LOC441442	1.0124	1.0127	0.9705	0.8406	0.9591	0.0407
LOC1001336	0.9413	1.1270	0.9353	0.8327	0.9591	0.0613
GIP	0.9544	0.9735	1.0629	0.8454	0.9591	0.0446
SSX4B	0.9586	1.0329	0.9375	0.9074	0.9591	0.0267
PM20D2	0.8693	1.0804	1.0238	0.8628	0.9591	0.0549
LOC388526	0.9187	0.9753	0.9742	0.9683	0.9591	0.0136
SERPINC1	0.9627	1.0557	0.8956	0.9224	0.9591	0.0350
LOC651324	0.9042	1.0285	0.9804	0.9234	0.9591	0.0282
LOC644578	0.8673	0.9668	1.0700	0.9323	0.9591	0.0423
C3orf45	0.8946	0.9945	1.0954	0.8520	0.9591	0.0544
FLJ13224	0.9360	1.1084	0.9588	0.8332	0.9591	0.0568
LOC1001314	0.9787	1.0550	0.9888	0.8140	0.9591	0.0512
LOC1001331	0.9177	1.1163	0.9171	0.8854	0.9591	0.0529
LOC400163	0.8929	1.1069	0.9738	0.8629	0.9591	0.0546
LOC1001339	0.8823	1.0790	0.9608	0.9145	0.9591	0.0431
LOC647131	1.0032	1.0552	0.9079	0.8703	0.9591	0.0425
PPBPL2	0.8800	1.1519	0.9620	0.8427	0.9591	0.0689
LOC645012	0.8432	1.0328	1.0559	0.9046	0.9591	0.0510
PPP2CB	0.7543	1.4045	0.9705	0.7073	0.9591	0.1591
FRAS1	0.9337	1.0527	0.9821	0.8680	0.9592	0.0390
LOC1001347	0.8505	1.1443	1.0035	0.8383	0.9592	0.0723
LOC284577	0.8939	1.1617	0.9537	0.8275	0.9592	0.0723
CBFA2T3	0.8900	1.0249	1.0026	0.9194	0.9592	0.0324
ITGA9	0.8294	1.1148	0.9121	0.9805	0.9592	0.0604
LOC644128	0.8826	1.2082	0.9422	0.8037	0.9592	0.0877
LOC440104	0.8962	1.0048	0.9839	0.9519	0.9592	0.0236
KRT85	0.9230	1.0427	1.0260	0.8451	0.9592	0.0463

EMR4	0.9261	1.0679	0.9562	0.8867	0.9592	0.0389
LOC346702	0.8541	1.1029	1.0015	0.8784	0.9592	0.0577
LOC1001290	0.8670	1.0265	1.0265	0.9169	0.9592	0.0402
C5orf56	0.8693	0.9830	1.0543	0.9303	0.9592	0.0393
FAM9C	0.9105	1.0602	0.9573	0.9089	0.9592	0.0355
LOC641744	0.9304	0.9938	0.9955	0.9172	0.9592	0.0206
TRAK1	0.8757	1.2007	0.9067	0.8538	0.9592	0.0812
MYCL1	0.9534	1.0364	0.9704	0.8767	0.9592	0.0328
LOC1001304	0.8048	1.1802	0.9322	0.9198	0.9592	0.0790
LOC644241	1.0069	0.9380	1.0217	0.8703	0.9592	0.0348
OR2G3	0.9255	0.9579	1.0523	0.9014	0.9593	0.0331
LOC648841	0.9354	0.9875	1.0475	0.8667	0.9593	0.0384
OSCAR	0.8339	1.0937	0.9486	0.9609	0.9593	0.0532
ZNF516	0.8554	1.0699	1.0269	0.8849	0.9593	0.0525
XAGE2	0.9354	1.0473	1.0192	0.8352	0.9593	0.0477
LIN28B	0.9195	1.0683	0.9861	0.8631	0.9593	0.0442
C3orf65	0.8757	0.9528	1.0455	0.9631	0.9593	0.0347
C9orf128	0.9052	0.9904	1.0653	0.8763	0.9593	0.0428
LOC729484	0.9411	0.9759	0.9614	0.9587	0.9593	0.0071
LOC653451	0.9861	1.0330	0.9805	0.8375	0.9593	0.0423
ARL4D	0.9426	1.0036	0.9873	0.9037	0.9593	0.0226
KCMF1	1.0140	1.0443	0.8939	0.8850	0.9593	0.0409
LOC642106	0.8258	1.0659	0.9629	0.9826	0.9593	0.0498
FAM171B	0.9470	1.1135	0.8832	0.8935	0.9593	0.0533
PEX26	0.9015	1.1507	0.9411	0.8439	0.9593	0.0669
NKG7	0.8594	1.1048	0.9675	0.9056	0.9593	0.0533
LOC1001312	0.9679	1.0294	1.0079	0.8321	0.9593	0.0443
SNORA72	0.8802	1.0643	0.9828	0.9100	0.9593	0.0411
LOC1001317	0.9440	1.0355	1.0414	0.8164	0.9593	0.0526
C1orf113	0.9267	0.9836	0.9732	0.9538	0.9593	0.0125
LOC728613	0.9058	1.0851	1.0173	0.8292	0.9593	0.0570
LOC727948	0.9057	1.0750	0.9893	0.8673	0.9594	0.0462
MIR203	0.9041	1.0543	0.9398	0.9392	0.9594	0.0327
LOC440970	0.8584	1.1013	0.9950	0.8828	0.9594	0.0559
LOC732229	0.8417	1.0227	1.0668	0.9063	0.9594	0.0518
FLNC	0.9935	1.0460	0.9169	0.8812	0.9594	0.0372
SERPINA13	0.9405	1.0248	1.0184	0.8539	0.9594	0.0400
NDUFC1	0.9269	1.1641	1.0617	0.6848	0.9594	0.1036
TUBGCP3	0.9330	0.9070	1.0684	0.9292	0.9594	0.0368
C1orf117	0.8640	1.0867	0.9911	0.8958	0.9594	0.0503
LOC647046	0.9002	1.0223	0.9603	0.9549	0.9594	0.0250
DKFZp761O2	0.9727	1.0338	0.9368	0.8945	0.9594	0.0295
WDTC1	0.8999	1.0498	1.0082	0.8798	0.9594	0.0413
LOC650898	0.8961	1.0732	0.9544	0.9140	0.9594	0.0398
PRRX1	0.8962	1.0282	0.9771	0.9363	0.9595	0.0282
LOC642464	0.9254	1.0916	0.9587	0.8622	0.9595	0.0484

C10orf114	0.8955	1.0587	1.0208	0.8629	0.9595	0.0475
LOC646720	1.0017	1.0537	0.9443	0.8382	0.9595	0.0462
LOC653127	0.9040	1.0949	1.0137	0.8254	0.9595	0.0594
LOC144438	0.8456	1.1549	1.0569	0.7805	0.9595	0.0879
LOC729238	0.9582	1.0024	1.0214	0.8559	0.9595	0.0370
TRIM31	0.9169	1.1457	1.0143	0.7611	0.9595	0.0811
GSDMA	0.9494	1.0987	0.9758	0.8141	0.9595	0.0584
LOC1001328	0.8467	1.0837	0.9941	0.9134	0.9595	0.0512
TPM4	0.7875	1.3152	0.8740	0.8613	0.9595	0.1201
LOC1001312	0.9276	1.0142	1.0517	0.8445	0.9595	0.0463
LOC652203	0.8840	1.0616	1.0175	0.8750	0.9595	0.0471
EDNRB	0.9261	1.0374	1.0003	0.8743	0.9595	0.0366
LSP1	0.9519	1.0464	1.0495	0.7903	0.9595	0.0608
LOC641802	0.8796	1.1491	0.8733	0.9362	0.9595	0.0647
CYCSP52	0.8747	1.1046	0.9237	0.9351	0.9595	0.0501
ZNF354A	0.8976	1.0167	0.9976	0.9263	0.9595	0.0284
LOC219347	0.9008	1.0626	0.9857	0.8891	0.9595	0.0405
LOC401218	0.8915	1.2872	0.8792	0.7803	0.9595	0.1120
ZNF41	0.8839	1.0546	1.0368	0.8630	0.9596	0.0500
C17orf38	0.9743	1.1027	0.9074	0.8539	0.9596	0.0537
LOC645958	0.9659	0.9637	1.0647	0.8440	0.9596	0.0452
LOC651390	0.9271	1.1484	0.9357	0.8271	0.9596	0.0676
SLC6A6	0.8050	1.0742	0.9497	1.0094	0.9596	0.0574
ATR	0.8396	1.0350	1.0221	0.9416	0.9596	0.0450
LOC1001301	0.9006	1.0519	0.9952	0.8906	0.9596	0.0388
EVI1	0.7773	1.2104	0.8961	0.9545	0.9596	0.0914
SNORD28	0.9744	0.9650	1.0335	0.8655	0.9596	0.0348
LGI1	0.9167	1.0398	1.0162	0.8658	0.9596	0.0411
MIR640	0.8510	0.9467	1.0717	0.9691	0.9596	0.0453
LOC730005	0.9548	0.9795	1.0201	0.8840	0.9596	0.0286
IFNA16	0.9028	0.9501	1.0453	0.9404	0.9596	0.0303
SH3RF2	0.8407	1.0202	1.0194	0.9583	0.9596	0.0422
EEF1B2	0.7580	1.2705	1.0747	0.7353	0.9596	0.1294
OSBPL7	0.8794	1.0671	1.0774	0.8147	0.9596	0.0664
FLJ11292	0.8426	1.0248	1.0460	0.9252	0.9596	0.0471
GGA1	0.7811	1.1658	1.0541	0.8375	0.9596	0.0905
LOC731932	0.9159	1.0452	0.9800	0.8975	0.9597	0.0336
C19orf44	0.8854	1.1196	1.0044	0.8292	0.9597	0.0646
LOC729492	0.8841	1.0848	0.9472	0.9225	0.9597	0.0437
GPR116	0.9300	1.0208	0.9516	0.9362	0.9597	0.0209
PDE1C	0.9970	0.9818	1.0502	0.8098	0.9597	0.0521
LOC1001342	0.9623	1.0451	1.0039	0.8273	0.9597	0.0472
ADAM28	0.9144	1.0943	0.9709	0.8590	0.9597	0.0504
LOC652571	1.0636	0.9391	0.8648	0.9712	0.9597	0.0412
LOC652594	0.8459	1.0093	1.1117	0.8718	0.9597	0.0621
WFDC5	0.9204	1.0734	0.9989	0.8460	0.9597	0.0491

PSORS1C2	0.9284	1.0868	0.9437	0.8799	0.9597	0.0445
NINJ2	0.9208	0.9805	1.0356	0.9019	0.9597	0.0304
KCNK4	0.8898	1.1062	1.0294	0.8133	0.9597	0.0662
RNF207	0.8380	1.2501	0.8358	0.9149	0.9597	0.0985
SCARNA4	0.8891	1.0882	0.9998	0.8618	0.9597	0.0522
LOC646437	0.9513	1.0619	1.0100	0.8156	0.9597	0.0531
LOC1001343	0.8673	1.0351	0.9705	0.9660	0.9597	0.0346
TBC1D1	0.8540	1.1796	0.9994	0.8059	0.9597	0.0840
MIR29A	0.8783	1.0161	1.0794	0.8651	0.9597	0.0525
LOC1001292	0.9074	0.9907	0.9978	0.9430	0.9597	0.0213
IL3	0.9192	1.0331	0.9467	0.9399	0.9597	0.0251
FAM59B	0.9276	1.0742	0.8889	0.9483	0.9597	0.0401
EIF1AD	0.8475	1.0839	1.0255	0.8821	0.9597	0.0566
GPRC5A	0.9733	1.0260	1.0027	0.8370	0.9597	0.0423
SDHC	0.8156	1.3977	0.8721	0.7535	0.9597	0.1480
LOC1001282	0.8314	1.0726	1.1423	0.7926	0.9597	0.0868
LOC440896	0.8584	1.0898	1.0248	0.8660	0.9597	0.0579
LOC1001282	0.9189	1.0139	0.9590	0.9471	0.9597	0.0199
SETD4	0.9229	1.0922	0.9597	0.8642	0.9598	0.0483
COL11A1	0.9142	1.0996	0.9483	0.8769	0.9598	0.0488
TCBA1	0.8790	1.0867	0.9191	0.9544	0.9598	0.0450
C9orf131	0.9382	1.0735	0.9394	0.8880	0.9598	0.0398
MGC57359	0.8717	1.0473	1.0347	0.8855	0.9598	0.0470
SCYL2	0.9611	1.2391	1.0217	0.6172	0.9598	0.1288
LOC554223	0.9266	1.0541	1.0048	0.8536	0.9598	0.0441
DUSP19	0.6881	1.2340	1.1385	0.7785	0.9598	0.1335
LOC442319	0.9534	1.0383	0.9024	0.9450	0.9598	0.0284
LOC643913	0.9111	1.1329	1.0456	0.7495	0.9598	0.0836
PDE7A	0.8634	1.0983	1.0620	0.8155	0.9598	0.0706
OSBPL8	0.6495	1.3239	1.2566	0.6092	0.9598	0.1915
MOBP	0.8820	1.0386	1.0013	0.9173	0.9598	0.0363
LOC643767	0.8828	1.0124	1.1032	0.8407	0.9598	0.0602
RASSF4	0.8608	1.0900	0.9484	0.9401	0.9598	0.0477
RGNEF	0.9423	1.0550	0.9816	0.8604	0.9598	0.0405
SSX9	0.9073	1.0397	1.0002	0.8921	0.9598	0.0358
REXO1L3P	0.9822	0.9735	0.9839	0.8996	0.9598	0.0202
ROCK1	0.8603	1.0799	1.1093	0.7897	0.9598	0.0794
LOC646066	0.8727	1.0237	1.0694	0.8735	0.9598	0.0509
LOC1001305	0.8917	1.1232	0.9384	0.8859	0.9598	0.0557
LOC651401	0.8912	1.1048	1.0189	0.8245	0.9598	0.0629
LOC400793	0.8065	1.0909	1.0241	0.9178	0.9598	0.0623
LOC645029	0.8929	1.1003	0.9529	0.8931	0.9598	0.0489
C11orf53	0.8960	1.0536	1.0089	0.8809	0.9598	0.0423
MIST	0.9338	1.0444	0.9451	0.9161	0.9598	0.0288
DMGDH	0.9681	0.9955	1.0087	0.8671	0.9599	0.0321
MT4	0.8958	0.9984	1.0953	0.8499	0.9599	0.0548

LOC647649	0.8963	1.1139	1.0389	0.7903	0.9599	0.0723
CLDN10	0.9184	1.0819	0.9956	0.8436	0.9599	0.0512
LOC1001307	0.9581	1.0080	0.8849	0.9885	0.9599	0.0270
BTNL2	0.9388	1.0976	0.9028	0.9001	0.9599	0.0468
LOC650465	0.8840	1.0936	1.0060	0.8558	0.9599	0.0552
EXOC2	0.8713	1.1192	1.0031	0.8459	0.9599	0.0633
VWA3B	0.8032	1.1197	0.9756	0.9410	0.9599	0.0650
SLC6A17	0.8880	1.0838	0.9965	0.8712	0.9599	0.0498
N4BP2L1	0.9349	0.9736	1.0467	0.8843	0.9599	0.0342
LOC653149	0.8528	1.1658	0.9302	0.8908	0.9599	0.0704
LOC649509	0.8117	1.0843	1.1270	0.8165	0.9599	0.0846
LOC91561	0.8996	1.0702	1.0836	0.7862	0.9599	0.0715
RAX	1.0037	1.0031	0.9846	0.8480	0.9599	0.0375
SLC35F3	0.9325	1.0172	0.9906	0.8992	0.9599	0.0269
LOC201651	0.9380	1.0304	0.9761	0.8951	0.9599	0.0287
LOC644479	0.9977	0.9692	1.0056	0.8671	0.9599	0.0319
LOC1001316	0.7933	1.1517	1.0155	0.8792	0.9599	0.0786
LOC148766	0.9581	1.0531	0.9873	0.8411	0.9599	0.0443
LOC649425	0.9982	0.9475	1.0140	0.8799	0.9599	0.0302
LOC1001339	1.0137	0.9848	0.9990	0.8421	0.9599	0.0397
TIGD2	0.7787	1.2603	0.9633	0.8373	0.9599	0.1073
LOC121456	0.8174	1.0555	1.0974	0.8694	0.9599	0.0686
LOC649063	0.8555	1.0773	1.0088	0.8981	0.9599	0.0508
LOC652350	0.8247	1.1615	0.9831	0.8705	0.9599	0.0750
CHST6	0.9764	1.0931	0.8934	0.8767	0.9599	0.0495
ZNF740	0.9399	1.0203	1.0420	0.8375	0.9599	0.0463
LOC646780	0.9395	0.8741	1.0825	0.9436	0.9599	0.0438
F3	0.8972	1.0183	1.0268	0.8976	0.9600	0.0362
LOC392506	0.9125	1.0752	0.9777	0.8745	0.9600	0.0439
MAG	0.9512	1.0382	0.9745	0.8761	0.9600	0.0335
SOX30	0.8583	0.9858	1.0404	0.9554	0.9600	0.0382
OOEP	0.8270	1.1363	0.9102	0.9664	0.9600	0.0654
GKN1	0.8368	1.0617	0.9980	0.9435	0.9600	0.0476
ZNF878	0.9311	1.0296	0.9466	0.9329	0.9600	0.0234
TNNI3K	0.8183	1.0689	1.0116	0.9413	0.9600	0.0540
FHIT	0.9159	1.0258	1.0269	0.8716	0.9600	0.0393
MTTP	0.9107	1.0358	1.0444	0.8492	0.9600	0.0479
CAMK1D	0.9325	1.0577	0.9472	0.9028	0.9600	0.0338
BOLL	0.9156	1.0286	0.9733	0.9227	0.9600	0.0262
PSG8	0.9437	1.0415	0.9624	0.8926	0.9600	0.0309
LOC651951	0.9338	1.1027	0.9317	0.8719	0.9600	0.0497
LOC653463	0.8690	1.0574	0.9746	0.9393	0.9601	0.0392
NLGN4Y	0.9978	1.0505	0.9821	0.8099	0.9601	0.0521
IMPA1	0.8250	1.2105	1.1471	0.6576	0.9601	0.1315
VANGL1	0.7759	1.1641	1.0707	0.8295	0.9601	0.0935
IQSEC2	1.0705	1.0208	0.9617	0.7872	0.9601	0.0618

MIR30C1	0.9243	0.9694	1.0212	0.9255	0.9601	0.0229
OR5H1	0.8897	0.9601	0.9981	0.9924	0.9601	0.0249
KIAA1609	0.9667	1.0365	0.9790	0.8582	0.9601	0.0372
LOC1001309	0.8736	1.0128	1.0618	0.8922	0.9601	0.0458
HOXB3	0.8751	1.1737	0.9839	0.8077	0.9601	0.0799
AMZ1	0.9083	1.1292	0.9285	0.8745	0.9601	0.0574
LOC729059	0.9649	1.0446	0.9824	0.8484	0.9601	0.0410
KCNA7	0.8651	1.1738	0.9245	0.8770	0.9601	0.0724
LOC1001318	0.8985	0.9686	1.0009	0.9725	0.9601	0.0218
LOC647366	0.9907	1.0655	0.9603	0.8240	0.9601	0.0505
LOC440296	0.7878	0.9860	1.0893	0.9774	0.9601	0.0628
COL28	0.9185	0.9283	1.0552	0.9386	0.9601	0.0320
LOC285804	0.8990	1.0719	0.9839	0.8857	0.9601	0.0431
LOC1001291	0.8839	1.1152	0.9927	0.8487	0.9602	0.0601
LOC652127	0.9336	1.0206	1.0893	0.7971	0.9602	0.0630
LOC650463	0.8312	1.0046	1.0179	0.9868	0.9602	0.0434
LOC339192	0.8772	1.0525	0.9955	0.9155	0.9602	0.0394
GPBAR1	0.9084	0.9878	1.0466	0.8979	0.9602	0.0351
TFR2	0.9272	1.0726	0.8728	0.9680	0.9602	0.0423
TEX13B	0.9670	1.0897	0.9250	0.8590	0.9602	0.0486
LOC648399	0.8058	1.0499	1.0329	0.9523	0.9602	0.0557
SMEK2	0.8211	1.0910	1.2109	0.7178	0.9602	0.1148
KRTAP7-1	0.9400	0.9347	1.0687	0.8974	0.9602	0.0374
H2AFB1	0.9280	1.0798	1.0249	0.8081	0.9602	0.0596
INE2	0.8436	0.9789	0.9826	1.0358	0.9602	0.0410
LOC1001283	0.9272	1.0988	0.8835	0.9314	0.9602	0.0474
LOC401648	0.8724	1.0726	0.9790	0.9168	0.9602	0.0434
LMOD2	0.9119	1.0228	1.0248	0.8813	0.9602	0.0373
FAM178A	0.9392	1.0555	0.9843	0.8619	0.9602	0.0406
PLN	0.8870	1.0911	1.0221	0.8407	0.9602	0.0582
LOC1001336	0.6525	1.3509	1.0824	0.7551	0.9602	0.1592
PRSS16	0.9073	1.2338	0.8720	0.8279	0.9602	0.0926
OR1J4	0.8284	1.1253	0.9493	0.9379	0.9602	0.0614
GGT8P	0.8637	1.0538	1.0628	0.8606	0.9602	0.0567
LOC389631	0.8923	1.0986	1.0217	0.8283	0.9602	0.0612
LOC649340	1.0093	1.0636	0.8911	0.8770	0.9602	0.0455
LOC1001323	0.9092	1.0235	1.0324	0.8760	0.9602	0.0397
LOC653729	0.8677	1.0163	1.0389	0.9181	0.9602	0.0405
ODF4	0.8679	0.9920	1.0336	0.9475	0.9603	0.0354
C21orf87	0.9228	1.0677	0.9770	0.8736	0.9603	0.0416
ACSM2B	0.8834	0.9676	1.0286	0.9614	0.9603	0.0298
OPRL1	0.9182	1.1452	0.9300	0.8477	0.9603	0.0643
NUDT10	0.9322	1.0181	0.9728	0.9179	0.9603	0.0225
SP140L	0.8541	1.0257	1.0637	0.8976	0.9603	0.0501
SYT14L	0.8548	1.0747	1.0566	0.8550	0.9603	0.0609
LOC644227	0.9596	1.0425	0.9845	0.8546	0.9603	0.0393

LOC441546	0.9237	0.9592	0.9858	0.9724	0.9603	0.0133
BHMT2	0.9747	0.9884	0.9547	0.9233	0.9603	0.0141
LOC649198	0.9106	1.0508	0.9399	0.9398	0.9603	0.0310
LOC1001302	0.9245	1.0396	0.9969	0.8801	0.9603	0.0358
ZNF597	0.7933	1.1327	1.0032	0.9120	0.9603	0.0718
FAM75A2	0.9799	1.0646	0.9214	0.8754	0.9603	0.0408
LOC649584	0.8616	1.0576	0.9366	0.9854	0.9603	0.0412
GLIS3	0.9184	1.0320	0.9896	0.9013	0.9603	0.0306
LOC651044	0.9344	0.9827	1.0248	0.8994	0.9603	0.0275
PHF5A	0.7520	1.3913	0.9264	0.7715	0.9603	0.1489
NFIC	0.8552	1.1547	1.0117	0.8197	0.9603	0.0771
LOC645757	0.8890	1.0127	1.0403	0.8992	0.9603	0.0387
SPACA1	0.9106	1.0553	1.0276	0.8477	0.9603	0.0489
LOC729891	0.8463	1.0528	1.0784	0.8638	0.9603	0.0611
PCOTH	1.0187	1.0352	0.9295	0.8580	0.9603	0.0413
KIAA0649	0.9330	1.2797	0.7748	0.8540	0.9603	0.1112
CST1	0.8829	1.0931	0.9315	0.9340	0.9604	0.0458
CPN2	0.9126	1.0654	1.0656	0.7979	0.9604	0.0651
WBSCR19	0.8470	1.1086	0.9913	0.8946	0.9604	0.0578
ADAM30	0.9001	0.9760	1.0453	0.9201	0.9604	0.0325
LOC729314	0.9608	1.0822	0.9724	0.8261	0.9604	0.0525
LOC1001324	1.0529	0.9325	1.0164	0.8396	0.9604	0.0475
SP3	0.9583	1.0565	1.0986	0.7280	0.9604	0.0828
LOC1001285	0.9456	1.0560	0.9597	0.8801	0.9604	0.0363
DNAH12L	0.9778	1.0243	0.9233	0.9160	0.9604	0.0254
MN1	0.9322	1.0450	0.9955	0.8688	0.9604	0.0383
TEKT3	0.8925	1.0797	0.9704	0.8989	0.9604	0.0435
NY-REN-7	0.9394	1.0413	0.9823	0.8786	0.9604	0.0343
DKFZp451M2	0.9251	1.1466	0.9324	0.8374	0.9604	0.0657
LOC389827	0.8550	1.1285	0.9890	0.8689	0.9604	0.0636
MAGEB10	0.9087	1.0159	0.9926	0.9243	0.9604	0.0260
FSTL5	0.9904	1.0218	0.9300	0.8995	0.9604	0.0278
HLA-DRB5	0.8651	1.0568	1.0284	0.8912	0.9604	0.0481
CIITA	0.9329	1.0069	1.0460	0.8557	0.9604	0.0420
LOC648240	0.9089	1.0751	0.9586	0.8990	0.9604	0.0404
WIF1	0.9407	1.0796	0.9910	0.8303	0.9604	0.0520
FBXO43	0.9227	1.0275	1.0265	0.8648	0.9604	0.0402
LOC401934	0.8932	1.0503	0.9266	0.9716	0.9604	0.0340
LOC651186	0.8926	1.0752	0.9775	0.8963	0.9604	0.0430
C2orf42	0.9146	1.1533	1.0037	0.7701	0.9604	0.0803
FLJ45300	0.9908	1.0735	0.9390	0.8384	0.9604	0.0492
PPIE	0.7643	1.3734	0.9594	0.7446	0.9604	0.1460
NPAS4	0.9606	1.0963	0.9645	0.8203	0.9604	0.0564
LOC641987	0.9155	1.0480	1.0517	0.8266	0.9604	0.0547
MIR548H1	0.9696	0.9876	0.9383	0.9462	0.9604	0.0112
LOC652404	0.9547	1.0548	1.0565	0.7757	0.9604	0.0660

FOXN3	0.9417	1.0378	0.9919	0.8703	0.9604	0.0359
LOC389901	0.8082	1.1373	1.0993	0.7970	0.9604	0.0915
FAM90A12	0.9728	1.0924	0.8843	0.8925	0.9605	0.0483
OR11H6	0.9109	1.0835	1.0421	0.8054	0.9605	0.0634
COL2A1	0.9088	1.0633	0.9757	0.8941	0.9605	0.0386
LOC643660	0.8546	1.0322	1.0298	0.9253	0.9605	0.0432
KCNQ2	0.8740	1.0765	0.9910	0.9004	0.9605	0.0461
HNMT	0.9271	1.0071	0.9904	0.9173	0.9605	0.0225
SPATA3	0.8781	1.0998	0.9842	0.8799	0.9605	0.0526
LOC1001312	0.8092	1.0570	0.9817	0.9940	0.9605	0.0531
CRHBP	0.9095	1.0012	1.0449	0.8864	0.9605	0.0375
LOC1001321	0.9939	1.0042	0.9225	0.9214	0.9605	0.0224
LOC651231	0.9857	1.0391	0.9826	0.8346	0.9605	0.0439
TFDP3	0.8689	1.0032	0.9906	0.9793	0.9605	0.0309
LOC650822	0.9448	1.0094	1.0832	0.8046	0.9605	0.0592
ELA1	0.9691	0.9729	0.9610	0.9390	0.9605	0.0076
ZNF341	0.8263	1.2255	0.9779	0.8123	0.9605	0.0960
PAIP1	0.9985	1.1067	0.9456	0.7913	0.9605	0.0656
LOC644091	0.8931	1.2113	1.0939	0.6438	0.9605	0.1243
LOC1001324	0.9501	1.0329	0.9627	0.8964	0.9605	0.0281
KIAA0748	0.9434	1.1367	0.8727	0.8892	0.9605	0.0606
LOC648590	0.9054	1.1046	0.9721	0.8601	0.9605	0.0532
C2orf90	0.9025	0.9653	1.0256	0.9488	0.9605	0.0254
MIR924	0.9350	1.0531	0.9992	0.8548	0.9605	0.0427
ZNF202	0.8316	1.2266	0.8938	0.8900	0.9605	0.0898
RORB	0.9470	1.0692	0.9766	0.8493	0.9605	0.0453
AMY2A	0.9146	1.0921	1.0217	0.8138	0.9605	0.0610
RAC2	0.8640	1.0802	0.9989	0.8991	0.9605	0.0491
NOS2A	0.9094	1.0872	0.9731	0.8725	0.9605	0.0471
LOC647993	0.8992	1.0831	1.0074	0.8525	0.9606	0.0522
LOC729197	0.9505	1.0359	0.9644	0.8914	0.9606	0.0297
LOC1001325	0.8827	1.1369	0.9457	0.8769	0.9606	0.0608
RNF138P1	0.9311	1.0298	0.9942	0.8872	0.9606	0.0319
ADAM19	0.9680	1.0044	0.9699	0.9000	0.9606	0.0219
RABL2B	0.9307	1.2314	0.9134	0.7667	0.9606	0.0975
MIR520H	1.0031	1.0177	0.9986	0.8229	0.9606	0.0461
PRLR	0.9105	1.0347	1.0192	0.8779	0.9606	0.0390
EDA2R	0.9418	0.9655	0.9625	0.9724	0.9606	0.0066
CMA1	0.8756	1.0786	1.1114	0.7767	0.9606	0.0805
MAGEE2	0.8953	1.0163	0.9888	0.9419	0.9606	0.0266
LOC728334	0.8880	0.9837	1.0133	0.9573	0.9606	0.0268
LOC650256	0.8947	1.0947	1.0027	0.8504	0.9606	0.0550
C2orf50	0.9781	0.9951	0.9778	0.8914	0.9606	0.0234
PAH	0.8755	1.0824	1.0096	0.8750	0.9606	0.0515
LOC1001314	1.0005	1.0588	0.9446	0.8386	0.9606	0.0469
NLRP12	0.8555	1.1030	0.9567	0.9273	0.9606	0.0520

PRKAR2B	0.8691	1.0525	0.9976	0.9234	0.9606	0.0404
FGF3	0.9135	0.9689	1.0385	0.9215	0.9606	0.0287
LOC728900	0.8619	1.0848	1.0035	0.8922	0.9606	0.0514
SOCS6	0.9115	1.0468	1.0765	0.8075	0.9606	0.0624
GLRB	0.9071	1.0071	1.0171	0.9112	0.9606	0.0298
LOC643012	0.9504	0.9523	1.0161	0.9237	0.9606	0.0196
TOP1	0.8485	1.1866	1.0158	0.7916	0.9606	0.0891
SPIN2B	0.8714	1.2046	0.9393	0.8272	0.9606	0.0845
LOC441344	0.8925	1.0216	0.9830	0.9454	0.9606	0.0275
MIR184	0.9654	1.0504	0.9713	0.8554	0.9606	0.0401
SH3YL1	0.8161	1.0871	1.0509	0.8885	0.9606	0.0647
IL1RL1	0.8955	1.0872	1.0059	0.8540	0.9606	0.0530
C10orf85	0.9111	1.1018	0.9782	0.8515	0.9606	0.0537
LOC644710	0.8701	1.0103	1.0271	0.9351	0.9607	0.0362
ZNF704	0.9486	1.0064	1.0264	0.8612	0.9607	0.0370
FREM2	0.8635	1.0187	1.0550	0.9055	0.9607	0.0454
C9orf37	0.9108	1.0359	1.1153	0.7807	0.9607	0.0733
MIR450A2	0.9633	0.9540	1.0414	0.8839	0.9607	0.0322
SCARNA8	0.8842	0.9842	1.0420	0.9322	0.9607	0.0339
LOC1001316	0.7990	1.0979	0.9415	1.0043	0.9607	0.0627
SLC35C1	0.8444	1.4949	0.8148	0.6885	0.9607	0.1813
LOC121792	0.8633	1.1364	0.9007	0.9423	0.9607	0.0607
SLC39A2	0.8797	1.0208	0.9734	0.9688	0.9607	0.0294
MIR770	0.8387	1.0680	0.9696	0.9664	0.9607	0.0470
MYBPC3	0.8858	0.9074	1.0730	0.9766	0.9607	0.0422
LOC642953	0.9269	0.9511	1.0266	0.9381	0.9607	0.0225
LOC645454	0.8432	1.1436	1.0034	0.8525	0.9607	0.0712
TTLL2	0.9345	1.0120	1.0231	0.8732	0.9607	0.0352
LOC391777	0.8710	1.2076	1.0754	0.6887	0.9607	0.1141
SEZ6L	0.9317	0.9649	1.0131	0.9331	0.9607	0.0191
TSHZ3	1.0032	1.1397	0.9252	0.7748	0.9607	0.0762
GPSM3	0.8552	1.1353	0.8906	0.9617	0.9607	0.0623
KIAA0565	0.9148	1.0259	1.0016	0.9006	0.9607	0.0312
HCG8	0.9149	1.0238	1.0744	0.8297	0.9607	0.0549
LOC1001339	0.9966	0.9753	1.0185	0.8525	0.9607	0.0371
LOC1001304	0.8934	1.0375	1.0011	0.9109	0.9607	0.0348
MIR202	0.9905	0.9607	1.0430	0.8486	0.9607	0.0411
LOC643866	0.9147	1.0293	0.9914	0.9075	0.9607	0.0297
RAB12	0.9123	1.1143	0.9182	0.8982	0.9607	0.0513
LOC1001309	0.8488	1.0608	1.0280	0.9053	0.9607	0.0501
GPR172B	0.8329	0.9987	1.0417	0.9696	0.9607	0.0451
LOC1001312	0.8943	1.0618	0.9813	0.9057	0.9607	0.0388
ZNF420	0.8633	1.0901	1.0192	0.8704	0.9607	0.0561
MFAP2	0.9053	1.0544	1.0210	0.8623	0.9607	0.0458
HOXB6	0.8256	1.1105	1.0476	0.8592	0.9608	0.0698
LOC730109	0.9286	1.1153	0.9972	0.8019	0.9608	0.0655

MGC13168	0.9727	1.0587	0.9286	0.8830	0.9608	0.0374
C15orf33	0.9452	1.0490	1.0536	0.7952	0.9608	0.0606
LOC1001302	0.9290	1.0307	0.9611	0.9222	0.9608	0.0248
EVPL	0.9291	1.3144	0.8489	0.7507	0.9608	0.1234
PPIL4	0.9178	1.0042	0.9452	0.9759	0.9608	0.0187
LOC653598	0.9367	0.9388	1.0468	0.9208	0.9608	0.0290
SLFN13	0.8167	1.0182	1.0558	0.9524	0.9608	0.0526
LOC649294	0.9109	1.0503	1.0301	0.8518	0.9608	0.0476
KRTAP10-2	0.9453	0.9694	1.0525	0.8760	0.9608	0.0364
NELL1	0.9046	1.0015	1.0478	0.8892	0.9608	0.0382
HSD17B8	0.9537	1.3156	0.9174	0.6565	0.9608	0.1355
LOC642372	0.8575	1.1399	0.9888	0.8570	0.9608	0.0673
MGC13057	0.8802	0.9817	1.0596	0.9218	0.9608	0.0390
D21S2056E	0.8554	1.0457	1.0329	0.9093	0.9608	0.0467
C14orf56	0.8811	0.9726	1.0656	0.9240	0.9608	0.0396
LOC653145	0.8741	1.0619	1.0144	0.8929	0.9608	0.0458
LOC645195	0.9303	0.9446	1.0775	0.8909	0.9608	0.0405
C1orf101	0.9922	1.1457	0.8298	0.8756	0.9608	0.0705
C14orf128	0.9147	1.0501	0.9939	0.8847	0.9608	0.0376
LOC1001012	1.0008	0.9577	0.9758	0.9092	0.9609	0.0194
LOC645574	0.8665	1.1714	0.9933	0.8122	0.9609	0.0798
TGM6	0.9240	1.0211	1.0520	0.8464	0.9609	0.0469
MIA	0.9841	1.0524	0.9905	0.8164	0.9609	0.0506
SMCR8	0.9341	0.9954	1.0369	0.8770	0.9609	0.0350
LOC1001308	0.8973	0.9761	0.9919	0.9781	0.9609	0.0215
GRIP2	0.9084	1.0131	1.0012	0.9208	0.9609	0.0270
PAX9	0.8465	1.1309	1.0502	0.8159	0.9609	0.0769
MGC48637	0.8990	1.0448	1.0318	0.8679	0.9609	0.0452
BLID	0.8994	0.9930	1.0836	0.8675	0.9609	0.0488
LOC1001294	0.7030	1.3497	0.9575	0.8333	0.9609	0.1396
LGSN	0.9349	1.0550	1.0122	0.8414	0.9609	0.0469
LOC1001334	0.9081	0.9964	1.0740	0.8650	0.9609	0.0466
LOC646048	0.8584	1.0154	0.9969	0.9728	0.9609	0.0353
MAGEF1	0.9266	1.3457	0.8244	0.7470	0.9609	0.1334
LOC1001281	0.8950	0.9967	1.0082	0.9437	0.9609	0.0261
LDB1	0.9074	1.1258	0.8634	0.9470	0.9609	0.0576
LOC646111	0.9933	1.0878	0.8776	0.8849	0.9609	0.0499
LOC649209	0.8580	1.1767	1.0928	0.7161	0.9609	0.1059
UST	0.8661	1.1156	0.9626	0.8994	0.9609	0.0553
C10orf21	0.8793	1.1263	1.0364	0.8017	0.9609	0.0736
LOC643296	0.9095	1.0305	0.9654	0.9383	0.9609	0.0258
DAZ1	0.8531	1.0757	1.0031	0.9118	0.9609	0.0492
LOC1001319	0.9115	1.0529	0.9965	0.8828	0.9609	0.0390
SNORD88C	0.8328	1.1785	0.9170	0.9154	0.9609	0.0751
RAB43	0.9382	1.0819	0.9939	0.8297	0.9609	0.0528
LOC1001314	0.9698	1.0276	0.9777	0.8687	0.9609	0.0333

FAM62C	0.8980	1.0568	0.9815	0.9075	0.9609	0.0370
LOC391766	0.9294	1.0307	0.9523	0.9314	0.9609	0.0238
LOC729517	0.9707	1.0683	0.8625	0.9423	0.9609	0.0425
CHORDC1	0.9310	1.2162	1.0014	0.6952	0.9609	0.1073
HTR3E	0.8745	0.9747	1.0312	0.9634	0.9609	0.0324
KRTAP4-7	0.8829	0.9717	1.1072	0.8819	0.9609	0.0531
ZSCAN21	0.9789	1.1515	0.9633	0.7501	0.9609	0.0822
AP4S1	0.9641	1.0711	0.9712	0.8373	0.9609	0.0479
MIPOL1	0.8025	1.2756	1.1138	0.6519	0.9609	0.1423
ZNF100	0.9578	1.0457	0.9762	0.8641	0.9609	0.0374
LOC644512	0.9441	1.0671	1.0632	0.7695	0.9609	0.0699
MC3R	0.9802	1.0492	0.9215	0.8928	0.9610	0.0346
LOC728493	0.8540	0.9828	1.0952	0.9119	0.9610	0.0519
KRTAP21-3	0.9626	0.9710	0.9764	0.9337	0.9610	0.0095
MFAP5	0.9338	1.0712	0.9832	0.8556	0.9610	0.0452
ZFR2	0.9310	1.1970	0.9247	0.7912	0.9610	0.0850
LOC133491	0.9348	1.0028	1.0035	0.9028	0.9610	0.0252
HERC4	0.9114	1.2601	0.8832	0.7892	0.9610	0.1031
TMCO2	0.9370	1.0818	0.9372	0.8879	0.9610	0.0419
SLC10A6	0.9252	1.0315	0.9825	0.9048	0.9610	0.0287
FLJ41327	0.9002	1.0887	1.0049	0.8501	0.9610	0.0534
ICOSLG	0.8191	0.9935	1.1415	0.8898	0.9610	0.0700
CD38	0.8960	1.0120	1.0249	0.9110	0.9610	0.0334
GABRG1	0.9222	0.9973	0.9907	0.9338	0.9610	0.0192
THTPA	0.8988	1.1016	0.9718	0.8718	0.9610	0.0514
LOC644070	0.9235	0.9873	1.0445	0.8888	0.9610	0.0345
UBR4	0.8576	1.4193	0.8319	0.7352	0.9610	0.1550
LOC144383	0.9502	0.9869	1.0571	0.8499	0.9610	0.0432
C17orf46	0.9776	1.1112	0.9271	0.8283	0.9610	0.0589
LOC728175	0.8770	1.1204	0.9404	0.9064	0.9610	0.0547
LOC642843	0.9265	1.0143	1.0388	0.8647	0.9611	0.0402
DNAH3	0.8835	1.0694	1.0402	0.8511	0.9611	0.0548
LOC1001299	0.9204	1.0687	0.8791	0.9760	0.9611	0.0410
ATRX	0.8784	1.1387	1.0115	0.8156	0.9611	0.0719
PTPRD	0.9338	1.0155	0.9524	0.9426	0.9611	0.0185
PAQR8	0.8794	0.9954	1.0789	0.8905	0.9611	0.0472
SYNPO2	0.8833	1.0724	0.9998	0.8887	0.9611	0.0458
ZNF284	0.8356	1.0918	1.0822	0.8347	0.9611	0.0727
LOC643449	0.9311	0.9602	1.0731	0.8799	0.9611	0.0409
POTE14	0.8288	1.1081	0.9896	0.9178	0.9611	0.0590
LOC730755	0.8613	1.0268	0.9194	1.0369	0.9611	0.0426
INHBA	0.9321	1.0519	0.9627	0.8977	0.9611	0.0331
LOC645627	0.8647	1.0187	1.0317	0.9293	0.9611	0.0394
LOC647281	0.9163	0.9260	1.0676	0.9345	0.9611	0.0357
PMS2L1	0.8595	1.1196	0.9844	0.8810	0.9611	0.0594
LOC1001319	0.8657	1.0761	1.0656	0.8370	0.9611	0.0637

MAP7D2	0.8363	1.0807	1.0524	0.8750	0.9611	0.0617
PRM3	0.8769	1.1155	0.9906	0.8615	0.9611	0.0590
C9orf126	0.9296	0.9767	1.1167	0.8215	0.9611	0.0612
POLR2J4	0.8973	1.0580	1.0108	0.8785	0.9611	0.0435
LOC1001290	0.8539	1.0984	1.0298	0.8625	0.9611	0.0611
ERCC-00012	0.8816	1.0414	0.9891	0.9324	0.9611	0.0346
NXPH3	0.9078	1.0062	1.0667	0.8639	0.9611	0.0461
P2RY2	0.9030	1.0383	0.9764	0.9269	0.9611	0.0299
C14orf37	0.9173	1.0752	0.9769	0.8752	0.9611	0.0434
SNAR-I	0.9739	1.0556	0.9485	0.8667	0.9611	0.0389
LOC642797	0.9038	1.1040	0.9368	0.9000	0.9612	0.0483
EPB41	0.8857	1.1251	0.9703	0.8635	0.9612	0.0593
LOC649023	0.9104	1.0824	0.9943	0.8575	0.9612	0.0493
MIR765	0.8382	0.9763	1.0512	0.9789	0.9612	0.0445
GRIN2B	0.8774	1.0443	0.9825	0.9405	0.9612	0.0351
WDR85	0.9059	1.1317	1.0158	0.7913	0.9612	0.0730
LOC340515	1.0013	0.9744	0.9494	0.9196	0.9612	0.0174
LOC642918	0.9763	0.9976	0.9683	0.9025	0.9612	0.0205
LOC203235	0.9927	1.0239	0.9374	0.8908	0.9612	0.0295
LOC644635	0.9332	1.0556	1.0413	0.8147	0.9612	0.0559
RSPH9	0.8846	1.0792	0.9627	0.9183	0.9612	0.0425
P2RX3	0.9642	1.0097	1.0337	0.8373	0.9612	0.0437
RCN2	0.7256	1.3321	1.0110	0.7762	0.9612	0.1384
UBE2U	0.8358	1.0732	1.0000	0.9358	0.9612	0.0503
SLC26A9	0.9499	1.0157	0.9848	0.8945	0.9612	0.0260
MAL	0.8704	1.1013	0.9598	0.9134	0.9612	0.0501
THAP5	0.9203	1.0274	0.9525	0.9447	0.9612	0.0231
OR2G2	0.8486	0.9547	1.0355	1.0061	0.9612	0.0411
LOC1001332	0.9819	1.1372	1.0461	0.6797	0.9612	0.0991
WDR16	0.9429	1.0376	1.0056	0.8589	0.9612	0.0394
OR13C4	0.8779	0.9060	1.1913	0.8698	0.9612	0.0771
EPR1	0.8873	1.0526	1.0630	0.8421	0.9613	0.0565
LOC387856	0.9295	0.9999	0.9749	0.9408	0.9613	0.0161
GPR61	0.8793	1.2044	0.9254	0.8359	0.9613	0.0831
SNORD114-2	0.9290	0.9728	1.0235	0.9198	0.9613	0.0237
CLSPN	0.8327	1.0643	0.9956	0.9524	0.9613	0.0487
FLJ25404	0.9183	1.0367	0.9977	0.8924	0.9613	0.0337
LOC727732	0.8918	1.2776	0.8949	0.7808	0.9613	0.1087
GPR77	0.9790	1.0204	0.8895	0.9562	0.9613	0.0274
C19orf53	0.7811	1.2457	1.0015	0.8168	0.9613	0.1064
MUC12	0.8924	1.0568	1.0283	0.8676	0.9613	0.0476
C1D	0.8973	1.0125	0.9924	0.9428	0.9613	0.0259
MIR548Q	0.8653	1.1542	1.0309	0.7948	0.9613	0.0811
GMIP	0.9580	1.1301	0.9974	0.7596	0.9613	0.0766
ARMCX4	0.9743	1.0109	1.0081	0.8519	0.9613	0.0374
COMMD8	0.7658	1.2723	1.0852	0.7219	0.9613	0.1315

LOC732075	0.8784	1.0409	1.0607	0.8651	0.9613	0.0519
LOC647439	0.8855	1.2119	0.9094	0.8385	0.9613	0.0848
ENO2	0.9169	1.3180	0.8026	0.8077	0.9613	0.1218
SNORD35A	0.8011	1.1277	1.0882	0.8282	0.9613	0.0852
C5orf39	0.8219	1.1522	1.0229	0.8484	0.9613	0.0777
SLC25A30	0.7610	1.2686	1.0641	0.7516	0.9613	0.1255
LOC641959	0.9151	1.1071	0.9819	0.8412	0.9613	0.0564
C10orf39	0.9596	1.1725	0.9101	0.8032	0.9613	0.0776
CSF3	0.8805	1.0445	0.9945	0.9258	0.9613	0.0363
IL17B	0.9147	1.0482	1.0371	0.8454	0.9613	0.0491
LOC651905	1.0504	0.9985	0.9671	0.8294	0.9613	0.0472
ANPEP	0.9319	1.1385	0.9759	0.7991	0.9613	0.0700
UBE2D4	0.8498	1.2885	1.0472	0.6599	0.9614	0.1347
ZNF562	0.7854	1.2676	1.0039	0.7884	0.9614	0.1142
SNORA11D	1.0222	1.0253	0.9164	0.8816	0.9614	0.0367
INTS7	1.0011	0.9753	1.1046	0.7644	0.9614	0.0713
LOC388237	0.8347	1.1128	1.0062	0.8917	0.9614	0.0618
LOC340549	0.9750	1.1657	0.9738	0.7309	0.9614	0.0891
MGC35030	0.8640	1.0371	1.0271	0.9173	0.9614	0.0423
MIR15A	0.8870	1.0822	0.9692	0.9071	0.9614	0.0439
OR3A4	0.8760	1.0706	0.9018	0.9971	0.9614	0.0448
PCDH12	0.9050	1.1379	0.9349	0.8678	0.9614	0.0604
LOC645489	1.0350	1.1395	0.9617	0.7093	0.9614	0.0916
LOC649747	0.8785	1.0316	1.1493	0.7861	0.9614	0.0805
MPL	0.9871	1.0227	0.9750	0.8608	0.9614	0.0350
LOC647534	0.8906	1.2057	1.0104	0.7389	0.9614	0.0986
LOC1001340	0.8866	1.1086	1.0745	0.7759	0.9614	0.0788
EXTL2	0.8502	1.3720	0.9112	0.7123	0.9614	0.1430
FBXW10	0.8232	1.0579	1.0302	0.9343	0.9614	0.0531
LOC387763	0.9043	1.0394	1.0414	0.8607	0.9614	0.0464
SKP1A	0.8726	1.3083	0.8813	0.7836	0.9614	0.1177
LOC148003	0.9814	1.0756	0.9310	0.8578	0.9614	0.0457
NLRP9	0.8380	1.0698	0.9194	1.0186	0.9614	0.0517
LOC732139	0.7993	1.2436	0.8888	0.9141	0.9614	0.0972
LOC646982	0.9136	1.0591	1.0395	0.8336	0.9615	0.0534
NBAS	0.8516	1.2232	0.9582	0.8129	0.9615	0.0925
ZNF452	0.8818	1.0723	1.0207	0.8710	0.9615	0.0503
GNGT1	0.9668	0.9916	1.0241	0.8634	0.9615	0.0347
LOC653796	0.9585	1.1054	0.8882	0.8939	0.9615	0.0505
PH-4	0.9766	0.9888	0.9756	0.9051	0.9615	0.0191
C16orf82	0.8797	1.0429	1.0624	0.8609	0.9615	0.0529
RAB40C	0.9381	1.3007	0.8636	0.7435	0.9615	0.1200
LOC646891	0.9916	1.0907	0.8848	0.8789	0.9615	0.0503
LRRC14	0.8090	1.2698	0.9688	0.7985	0.9615	0.1099
CGREF1	0.9693	1.0039	0.9493	0.9235	0.9615	0.0170
LOC645818	0.8931	1.1098	1.0583	0.7849	0.9615	0.0748

LOC1001326	0.9025	1.0982	0.9272	0.9181	0.9615	0.0459
LOC652324	0.7622	1.2583	0.9853	0.8403	0.9615	0.1092
PRO1853	0.8783	1.1035	0.9973	0.8671	0.9615	0.0557
DNAJB9	1.0506	1.2411	0.9279	0.6265	0.9615	0.1289
LOC389983	0.8940	1.1232	0.9680	0.8609	0.9615	0.0584
LOC643189	0.9112	1.0260	0.9990	0.9100	0.9615	0.0299
LOC648898	0.9127	1.0404	1.0158	0.8773	0.9615	0.0394
LOC1001324	0.8939	1.1549	0.9098	0.8876	0.9616	0.0646
LOC649978	0.9075	1.0728	0.9239	0.9420	0.9616	0.0377
LOC644567	0.9929	0.9781	0.9060	0.9693	0.9616	0.0192
KRT37	0.9386	0.9215	1.1128	0.8734	0.9616	0.0523
IGFBP6	0.8808	1.2006	0.9713	0.7936	0.9616	0.0876
ZBTB1	0.8859	1.1148	0.9102	0.9355	0.9616	0.0521
LOC652587	0.9381	1.0124	1.0427	0.8530	0.9616	0.0423
LOC1001283	0.9078	1.0893	0.9843	0.8650	0.9616	0.0492
LOC1001325	0.9060	1.0473	1.0170	0.8760	0.9616	0.0417
LOC1001300	0.9769	1.1138	0.9033	0.8524	0.9616	0.0568
MRGPRE	0.9299	1.0476	0.9511	0.9177	0.9616	0.0295
RPL14	0.7888	1.1279	1.3110	0.6187	0.9616	0.1574
C20orf151	0.9790	0.9393	1.1305	0.7975	0.9616	0.0685
FLJ42875	0.9185	1.0702	0.9861	0.8716	0.9616	0.0432
LOC728626	0.8125	1.0681	1.0481	0.9178	0.9616	0.0598
ADAMTSL4	0.9078	1.0498	1.0057	0.8832	0.9616	0.0395
SNORD113-7	0.8355	1.1429	0.9080	0.9601	0.9616	0.0656
LOC730510	0.9807	1.0098	0.9742	0.8818	0.9616	0.0277
LOC649800	0.8917	1.0139	1.0094	0.9314	0.9616	0.0300
FCGBP	0.8765	1.1107	0.9857	0.8735	0.9616	0.0561
LOC641926	0.9683	1.0637	0.9057	0.9088	0.9616	0.0370
C13orf18	0.9519	0.9695	1.0401	0.8850	0.9616	0.0319
LOC285733	0.9771	0.9994	0.9658	0.9042	0.9616	0.0204
LOC388401	0.9015	0.9978	1.0360	0.9112	0.9616	0.0329
C11orf67	0.9717	1.1720	0.9803	0.7226	0.9616	0.0921
STAG2	0.9301	1.2668	0.9461	0.7036	0.9617	0.1158
ARFGAP2	0.9089	1.1082	0.9999	0.8296	0.9617	0.0600
PTPDC1	0.8700	1.1112	1.0253	0.8401	0.9617	0.0643
GP2	1.0183	1.0240	0.9402	0.8641	0.9617	0.0377
RAB20	0.8304	1.2186	0.9933	0.8043	0.9617	0.0953
LOC643584	0.9579	1.0796	0.9752	0.8340	0.9617	0.0503
FRMD5	1.0060	1.0186	0.9394	0.8826	0.9617	0.0316
PIWIL2	0.9337	1.0385	1.0070	0.8675	0.9617	0.0383
LOC1001334	0.9209	1.1653	0.9019	0.8587	0.9617	0.0691
LOC284395	1.0176	0.9974	0.9420	0.8897	0.9617	0.0288
WDR40A	0.8513	1.2678	0.9398	0.7879	0.9617	0.1067
C12orf53	0.9304	1.0419	0.9816	0.8928	0.9617	0.0323
LOC651546	0.8973	1.1819	0.9497	0.8179	0.9617	0.0783
USP9X	0.8993	1.2151	0.9361	0.7963	0.9617	0.0895

LOC1001307	0.9251	0.9588	1.0064	0.9565	0.9617	0.0168
MMRN1	0.9190	1.0729	0.9830	0.8720	0.9617	0.0435
BPY2B	0.8482	1.1105	1.0791	0.8091	0.9617	0.0775
LOC644120	0.8889	0.9863	0.9843	0.9874	0.9617	0.0243
TMCC3	0.8988	1.0745	1.0064	0.8673	0.9617	0.0480
LOC285205	1.0080	0.9363	0.9419	0.9606	0.9617	0.0163
ADPRHL1	0.9559	1.1404	0.9918	0.7588	0.9617	0.0786
PRH2	0.9436	1.0198	0.8613	1.0221	0.9617	0.0381
APOA2	0.9707	1.0097	0.9749	0.8916	0.9617	0.0250
LOC647310	1.0175	0.9921	1.0159	0.8214	0.9617	0.0471
GALNT7	0.9205	1.0568	0.9647	0.9050	0.9617	0.0341
CXCL11	0.9251	1.0880	0.9711	0.8629	0.9617	0.0476
GCGR	0.8444	1.0130	1.1185	0.8712	0.9617	0.0640
LOC651007	0.8820	1.0776	0.9739	0.9134	0.9618	0.0431
OR2C3	0.8941	1.0791	1.0456	0.8282	0.9618	0.0600
LOC1001342	0.9014	1.1147	0.9309	0.9001	0.9618	0.0515
MUCDHL	1.0094	1.0185	0.8816	0.9375	0.9618	0.0323
BCL2A1	0.9057	1.0106	1.1000	0.8308	0.9618	0.0590
LOC1001343	0.8638	1.0919	1.0323	0.8591	0.9618	0.0592
LOC440292	0.8612	1.1202	0.9517	0.9141	0.9618	0.0560
C6orf182	0.8910	1.0631	1.0659	0.8271	0.9618	0.0607
AGRN	0.9989	1.2795	0.8484	0.7202	0.9618	0.1203
LOC642453	0.8836	0.9588	1.0781	0.9266	0.9618	0.0417
CSTA	0.9105	0.9416	1.0541	0.9409	0.9618	0.0316
C1QA	1.0069	0.9526	0.9717	0.9159	0.9618	0.0190
R3HDML	0.9933	0.9756	1.0265	0.8518	0.9618	0.0382
UBE1C	0.8828	1.0824	1.0651	0.8168	0.9618	0.0661
SV2B	0.8707	1.1452	0.8957	0.9356	0.9618	0.0626
CLCNKA	0.9288	1.0879	0.9672	0.8632	0.9618	0.0472
LOC1001299	0.9555	0.9771	1.0272	0.8874	0.9618	0.0290
FAM171A1	0.8082	1.3429	0.9534	0.7428	0.9618	0.1344
LOC644474	1.0527	0.9551	0.9728	0.8666	0.9618	0.0382
SYT5	0.9834	1.1128	0.9921	0.7589	0.9618	0.0738
ZEB2	0.9633	0.9749	1.0138	0.8952	0.9618	0.0247
RHOBTB2	0.8016	1.2858	0.9745	0.7854	0.9618	0.1162
TMEM217	0.8791	1.1020	0.9802	0.8860	0.9618	0.0521
PITX2	0.8656	1.0751	0.9977	0.9089	0.9618	0.0467
LOC646560	1.0081	0.9424	1.0030	0.8939	0.9618	0.0271
LOC653346	0.8476	1.0470	1.0106	0.9421	0.9618	0.0439
ZNF474	0.9569	1.0844	0.9334	0.8727	0.9618	0.0445
PTER	0.9294	1.0886	0.9909	0.8384	0.9618	0.0526
LOC1001322	0.8529	1.0212	0.9784	0.9949	0.9618	0.0374
FLJ42842	0.7834	1.1491	0.9328	0.9821	0.9618	0.0754
GLT6D1	0.8859	1.0238	1.1080	0.8297	0.9619	0.0635
LOC1001305	0.8669	1.0429	1.0786	0.8590	0.9619	0.0576
LOC728060	0.8873	1.1067	1.0653	0.7881	0.9619	0.0750

NUDT4	0.8931	1.1090	0.9776	0.8678	0.9619	0.0544
LOC652857	0.9601	1.0893	0.9155	0.8826	0.9619	0.0454
LOC643152	0.9086	1.1034	0.9674	0.8681	0.9619	0.0514
A1BG	0.9415	1.0823	0.9983	0.8254	0.9619	0.0539
CRYGD	0.9118	1.0244	0.9619	0.9494	0.9619	0.0234
PCDH24	0.9692	1.1836	0.9275	0.7672	0.9619	0.0858
OR4K5	0.8853	1.0860	0.9919	0.8845	0.9619	0.0484
LOC653249	0.8969	1.0533	1.0207	0.8768	0.9619	0.0440
ADARB2	0.9042	1.0923	0.9496	0.9016	0.9619	0.0448
SYPL2	0.9276	0.9896	1.0295	0.9011	0.9619	0.0292
MIB1	0.9004	1.0445	1.0267	0.8761	0.9619	0.0430
LOC727977	0.8966	1.0435	1.0500	0.8577	0.9619	0.0496
DCP2	0.8972	1.2355	0.9869	0.7282	0.9619	0.1058
LOC644669	0.9398	1.0910	0.9259	0.8911	0.9620	0.0442
SNORD58A	0.7713	1.1213	1.0508	0.9044	0.9620	0.0780
C17orf61	0.7901	1.1513	1.1775	0.7289	0.9620	0.1177
RYBP	0.7524	1.3928	0.9352	0.7674	0.9620	0.1495
DPYSL3	0.9421	0.9345	1.0304	0.9410	0.9620	0.0229
LOC643977	0.9978	0.9969	0.9827	0.8705	0.9620	0.0307
CALR3	0.9671	1.0011	1.0471	0.8326	0.9620	0.0461
GNRHR2	0.8983	1.1219	0.8885	0.9393	0.9620	0.0544
OLFML3	0.8582	1.0297	0.9953	0.9648	0.9620	0.0371
LOC643608	0.8930	0.9368	0.9891	1.0290	0.9620	0.0298
LOC651704	0.9785	1.0022	1.0510	0.8162	0.9620	0.0509
OR3A1	0.8706	1.0596	0.9810	0.9367	0.9620	0.0397
SEMA3B	0.9406	1.0381	0.9648	0.9044	0.9620	0.0282
TMPRSS5	0.9739	1.1572	0.9200	0.7969	0.9620	0.0749
SNORD36B	0.9302	1.1278	0.9120	0.8781	0.9620	0.0563
MGC26647	0.9048	1.0552	0.9388	0.9492	0.9620	0.0325
MORN4	0.9284	1.1217	1.0121	0.7858	0.9620	0.0708
OR2T8	0.8775	1.0337	0.9963	0.9405	0.9620	0.0341
LOC731898	0.9735	1.0559	0.9390	0.8797	0.9620	0.0368
LOC388177	0.9189	1.0187	1.0368	0.8736	0.9620	0.0392
FOXD4L4	1.0084	0.9533	0.9832	0.9031	0.9620	0.0226
LOC642405	0.9227	1.0037	1.0137	0.9080	0.9620	0.0272
RNY4	0.7734	1.0655	1.1233	0.8861	0.9621	0.0807
LOC1001296	0.9700	1.0419	0.9111	0.9252	0.9621	0.0294
TMC2	0.9600	0.9633	1.0285	0.8964	0.9621	0.0270
CCDC97	0.8343	1.3195	0.9741	0.7204	0.9621	0.1299
DKKL1	0.9152	0.9943	0.9943	0.9445	0.9621	0.0195
MYO7A	0.9531	1.0491	0.9688	0.8773	0.9621	0.0352
LOC646023	0.7735	0.9361	1.1165	1.0222	0.9621	0.0729
NCOA7	0.8596	1.3485	0.9367	0.7035	0.9621	0.1376
C19orf29OS	1.0259	0.9830	0.9096	0.9299	0.9621	0.0263
LOC652566	0.8072	1.1547	1.0434	0.8431	0.9621	0.0826
LOC648659	1.0255	1.0768	1.0078	0.7383	0.9621	0.0760

LOC338739	0.9472	1.0617	0.9302	0.9094	0.9621	0.0341
MSX1	0.8743	1.3262	0.9568	0.6912	0.9621	0.1334
MBOAT4	1.0374	0.9618	1.0093	0.8400	0.9621	0.0436
FLJ45032	0.9629	1.0764	0.9588	0.8503	0.9621	0.0462
CNTFR	0.9672	0.9873	0.9947	0.8993	0.9621	0.0217
COL28A1	0.8336	1.1038	1.0269	0.8842	0.9621	0.0625
GPR143	0.9247	1.0964	1.0064	0.8210	0.9621	0.0587
LOC1001286	0.9335	1.0217	1.0201	0.8732	0.9621	0.0361
PPY	0.9061	1.1360	0.7916	1.0149	0.9621	0.0737
MEX3A	0.9168	1.2737	0.8522	0.8058	0.9621	0.1063
ZNF418	0.7959	1.1561	1.1102	0.7864	0.9621	0.0992
LOC651709	0.8377	1.2131	1.0037	0.7940	0.9621	0.0951
MIR449C	0.9461	1.0324	0.9848	0.8852	0.9621	0.0311
ANO1	0.8336	1.0608	1.0927	0.8615	0.9622	0.0667
USP12	0.7971	1.1320	1.0642	0.8554	0.9622	0.0806
PRL	0.9792	1.0724	0.9161	0.8810	0.9622	0.0420
LOC649242	0.8621	1.0619	1.0100	0.9147	0.9622	0.0452
LOC653817	0.9043	1.0491	1.0386	0.8567	0.9622	0.0482
HRNBP3	0.9333	0.9758	1.1104	0.8293	0.9622	0.0582
ENOX1	0.8649	1.2681	0.8489	0.8669	0.9622	0.1021
LOC1001338	0.8934	1.0251	1.0104	0.9197	0.9622	0.0327
LOC649823	0.9151	0.9620	1.1312	0.8404	0.9622	0.0617
EPB49	0.9388	1.0289	1.0959	0.7852	0.9622	0.0672
LOC730322	0.8978	1.0916	1.0114	0.8479	0.9622	0.0551
DEADC1	0.9031	1.0975	0.9585	0.8896	0.9622	0.0475
LOC728205	0.9122	0.9318	1.1171	0.8876	0.9622	0.0524
RHOXF1	1.0328	0.9866	0.8920	0.9373	0.9622	0.0305
PYY	0.9383	1.0469	0.9891	0.8745	0.9622	0.0367
OR10J1	0.9109	1.0520	0.9610	0.9249	0.9622	0.0317
MIR320C1	0.8701	1.0011	0.9936	0.9840	0.9622	0.0309
MORN3	0.9071	1.0294	1.1075	0.8049	0.9622	0.0667
CBWD6	1.0710	0.8267	1.1099	0.8412	0.9622	0.0745
LOC730153	0.9914	0.9917	0.9719	0.8939	0.9622	0.0232
RASSF9	0.8260	1.0990	1.0067	0.9172	0.9622	0.0586
LOC339799	0.9177	0.9882	0.9449	0.9981	0.9622	0.0188
LOC729162	0.9497	1.0543	1.0513	0.7937	0.9622	0.0612
UNQ9370	0.7152	1.2607	0.9644	0.9087	0.9622	0.1129
LOC1001343	0.9292	1.1126	0.8921	0.9152	0.9622	0.0507
PLTP	0.8355	1.1203	1.0120	0.8812	0.9622	0.0646
LOC649067	0.9899	1.0200	0.8910	0.9481	0.9623	0.0280
TRIM73	0.9140	1.0719	1.0273	0.8358	0.9623	0.0537
C6orf103	0.8867	1.0387	1.0431	0.8805	0.9623	0.0454
LOC728590	0.7333	1.1661	1.3368	0.6128	0.9623	0.1723
HSPC268	0.7787	1.1475	1.1730	0.7498	0.9623	0.1146
LOC392452	0.9392	0.9336	0.9945	0.9817	0.9623	0.0152
LOC1001310	1.0075	0.9627	0.9086	0.9703	0.9623	0.0204

LIPC	0.8802	1.0983	0.9765	0.8942	0.9623	0.0501
C10orf67	1.0106	1.1330	0.9469	0.7586	0.9623	0.0781
PENK	0.8534	1.0427	1.0647	0.8883	0.9623	0.0534
C1orf51	0.9344	1.0539	0.9488	0.9121	0.9623	0.0315
SPANXB2	0.9046	1.2242	0.9872	0.7332	0.9623	0.1021
LCE3D	0.9364	1.0372	0.9958	0.8798	0.9623	0.0344
ATP6V1G3	0.8453	1.0645	1.0202	0.9192	0.9623	0.0495
LOC729839	0.8969	1.0725	0.9739	0.9059	0.9623	0.0406
ANKRD54	0.8412	1.3900	0.8450	0.7731	0.9623	0.1435
LOC254028	0.9319	1.0639	0.9402	0.9132	0.9623	0.0343
GABBR1	0.9235	1.1169	0.9488	0.8600	0.9623	0.0548
SH2D3A	0.9164	1.0665	0.9509	0.9155	0.9623	0.0357
GUSB	0.7895	1.4345	0.9317	0.6935	0.9623	0.1648
CKMT1A	0.8641	1.3340	0.9985	0.6526	0.9623	0.1429
FAM176B	0.9251	1.1774	0.9306	0.8161	0.9623	0.0764
LOC653051	0.9788	1.0537	0.9396	0.8773	0.9623	0.0369
PXMP4	0.9193	1.0493	0.9976	0.8832	0.9623	0.0376
LOC647135	0.9251	1.0975	1.0080	0.8187	0.9623	0.0594
LOC729858	0.9395	1.0389	1.0211	0.8499	0.9623	0.0433
FAM21B	0.9334	0.9974	1.0401	0.8784	0.9623	0.0355
CD48	0.9139	1.0104	1.0195	0.9055	0.9623	0.0305
LRRIQ3	0.8877	1.0880	0.9843	0.8894	0.9623	0.0476
TRPV2	0.8549	1.1009	1.0501	0.8436	0.9624	0.0662
LOC645351	0.8416	1.2226	0.9017	0.8835	0.9624	0.0877
LOC1001326	0.9718	1.0491	0.9855	0.8431	0.9624	0.0432
ANXA8L2	0.9344	1.0042	1.0446	0.8662	0.9624	0.0393
PDE4B	0.9489	1.0024	1.0044	0.8937	0.9624	0.0262
LOC1001315	1.0070	1.0665	0.9441	0.8319	0.9624	0.0502
LOC649203	0.8793	1.0313	1.0409	0.8981	0.9624	0.0428
LOC644532	0.8479	0.9949	1.1055	0.9011	0.9624	0.0566
LOC1001300	1.0309	0.9410	1.0147	0.8629	0.9624	0.0385
TMEM173	0.9112	1.1461	0.9888	0.8035	0.9624	0.0721
CCDC82	0.9458	1.0148	0.9887	0.9002	0.9624	0.0251
HM13	0.7090	1.3822	0.9814	0.7769	0.9624	0.1514
LOC648050	0.8100	1.0178	1.1080	0.9138	0.9624	0.0644
LOC642214	0.8328	1.1299	1.0226	0.8643	0.9624	0.0696
ACE	0.9623	1.0313	1.0040	0.8519	0.9624	0.0395
HSD17B12	0.8375	1.1342	1.1876	0.6904	0.9624	0.1189
LOC653039	0.9236	0.9444	0.9607	1.0210	0.9624	0.0210
HSPC047	0.8984	1.0525	1.0233	0.8755	0.9624	0.0442
LOC645822	0.8824	1.1420	0.9961	0.8292	0.9624	0.0692
PSTPIP2	0.8846	1.0489	0.9925	0.9237	0.9624	0.0364
LOC728677	0.8881	1.0274	1.0129	0.9213	0.9624	0.0341
LOC1001305	0.9550	0.9957	0.9938	0.9053	0.9624	0.0212
FAM90A5	0.9030	1.0889	0.9265	0.9314	0.9624	0.0426
LOC1001325	0.9165	1.0361	1.0254	0.8718	0.9624	0.0405

CXADRP2	0.9997	0.9808	0.9518	0.9175	0.9624	0.0179
SAMSN1	0.8994	0.9836	1.0315	0.9353	0.9624	0.0288
LOC731050	0.9318	1.0122	1.0526	0.8532	0.9624	0.0442
LOC1001301	0.8398	1.1253	1.0285	0.8563	0.9625	0.0690
CD84	0.9484	1.0898	0.9009	0.9107	0.9625	0.0437
PTPN18	0.8511	1.1235	0.9607	0.9146	0.9625	0.0582
LOC387790	0.7777	1.1415	0.8735	1.0572	0.9625	0.0832
TEK	0.9631	1.0725	0.9766	0.8378	0.9625	0.0482
ZNF287	0.9224	1.0241	1.0536	0.8499	0.9625	0.0469
MAFF	0.8788	1.0370	1.0656	0.8686	0.9625	0.0516
FLJ46154	0.9141	1.0421	1.0285	0.8653	0.9625	0.0433
LOC1001289	0.9342	1.0976	0.9769	0.8413	0.9625	0.0532
C1QC	0.9671	1.0639	0.9622	0.8568	0.9625	0.0423
LOC730152	0.9118	1.0146	0.9152	1.0084	0.9625	0.0283
SNORD10	1.0329	1.0256	0.9859	0.8056	0.9625	0.0533
COX18	0.9783	1.0745	0.9426	0.8547	0.9625	0.0455
LOC643336	0.7990	1.3275	0.9946	0.7289	0.9625	0.1340
LOC729828	0.9219	1.0046	0.9752	0.9483	0.9625	0.0178
DYDC2	1.0472	1.0404	0.8980	0.8646	0.9625	0.0474
LOC1001011	0.8593	1.0332	0.9798	0.9778	0.9625	0.0367
RAB27B	0.8763	1.0604	1.0116	0.9018	0.9625	0.0439
LOC1001290	0.9230	1.1074	1.0403	0.7794	0.9625	0.0720
POTEG	0.9178	1.1268	0.9106	0.8950	0.9625	0.0549
LOC286239	1.0755	1.0198	0.9107	0.8441	0.9625	0.0523
ATP6V0A4	0.8545	1.0402	1.0233	0.9322	0.9625	0.0431
LOC284232	0.9181	1.0439	0.9657	0.9224	0.9625	0.0292
LOC400950	0.9327	1.0415	0.9643	0.9116	0.9625	0.0285
LOC439957	0.8471	1.0814	1.0013	0.9204	0.9625	0.0506
IL20RA	0.8931	1.1368	0.8602	0.9601	0.9625	0.0617
LOC1001339	0.9027	0.9876	0.9555	1.0045	0.9626	0.0224
GUCA2B	0.9826	1.0000	0.9959	0.8718	0.9626	0.0305
CYP3A4	0.9802	1.0091	1.0433	0.8177	0.9626	0.0500
MIR616	0.9245	1.0154	1.0303	0.8801	0.9626	0.0361
KRTAP4-11	0.9470	0.9598	1.0001	0.9434	0.9626	0.0130
RPLP0	0.8641	1.1685	1.0092	0.8086	0.9626	0.0806
LOC344423	0.9474	1.0449	0.9210	0.9371	0.9626	0.0280
OR10K1	0.9738	0.9909	0.9924	0.8933	0.9626	0.0235
SCCPDH	0.9353	1.2628	0.9106	0.7417	0.9626	0.1089
SYCP1	0.9593	1.0042	0.9730	0.9138	0.9626	0.0188
LOC652111	0.8745	1.0294	1.0419	0.9045	0.9626	0.0427
LOC729175	0.9186	1.0803	1.0380	0.8136	0.9626	0.0603
LOC646276	0.9089	1.1385	0.9622	0.8408	0.9626	0.0637
KRT6B	0.9559	1.0898	0.9144	0.8904	0.9626	0.0445
LOC1001313	0.8521	1.1634	0.9572	0.8777	0.9626	0.0706
C20orf79	1.0192	0.9899	0.9803	0.8610	0.9626	0.0348
PARD6A	0.9164	1.2843	0.9111	0.7386	0.9626	0.1149

LOC651441	0.9058	1.0560	0.9562	0.9325	0.9626	0.0328
LOC653894	0.8365	1.0227	1.1400	0.8513	0.9626	0.0727
RSPO1	0.9025	1.0675	1.0730	0.8075	0.9626	0.0651
LOC652282	0.8211	1.1247	0.9735	0.9312	0.9626	0.0629
ARL14	0.8792	1.0139	1.0092	0.9482	0.9626	0.0316
LOC643039	0.8309	1.1404	0.9914	0.8878	0.9626	0.0679
PDC	0.8925	1.0783	0.9614	0.9184	0.9626	0.0411
DYRK3	0.9084	1.0689	0.9899	0.8834	0.9626	0.0421
LOC731915	0.9619	1.0080	1.1198	0.7609	0.9627	0.0750
LOC441655	0.8290	1.0567	0.9879	0.9770	0.9627	0.0479
LOC1001326	0.9234	0.9976	1.0479	0.8817	0.9627	0.0372
SRGN	0.9124	1.0513	1.0060	0.8809	0.9627	0.0397
CXorf48	0.9321	1.0252	0.9996	0.8938	0.9627	0.0302
LOC400743	0.9422	1.1679	0.9542	0.7864	0.9627	0.0784
AIP	0.7412	1.4281	0.9151	0.7663	0.9627	0.1598
OR1F2P	0.9638	1.0210	0.9599	0.9060	0.9627	0.0235
DYNC1I1	1.0005	0.9082	1.0747	0.8674	0.9627	0.0466
LOC142937	0.8960	1.1530	0.9086	0.8932	0.9627	0.0635
LOC648600	0.9050	1.0763	0.9894	0.8800	0.9627	0.0445
C2orf14	0.9464	1.0444	0.9977	0.8622	0.9627	0.0390
LRFN5	0.8914	1.0428	0.9931	0.9236	0.9627	0.0341
BIRC8	0.9346	1.0365	0.9722	0.9075	0.9627	0.0279
LOC652184	0.9140	1.0752	0.9864	0.8752	0.9627	0.0440
LOC643672	0.8265	1.1696	0.9272	0.9276	0.9627	0.0729
LOC389906	0.9453	1.0796	0.9940	0.8320	0.9627	0.0517
LOC1001296	0.8939	1.0287	1.0175	0.9109	0.9627	0.0351
GTF2A1L	0.8827	1.0739	1.0169	0.8775	0.9627	0.0491
ZFYVE1	0.9259	1.1171	1.0086	0.7994	0.9627	0.0671
LOC651562	0.8440	1.1067	1.0106	0.8897	0.9628	0.0595
LOC652846	1.0213	1.0282	0.9234	0.8782	0.9628	0.0370
SLC4A8	0.9125	1.0281	1.0547	0.8558	0.9628	0.0472
FGF7	0.8594	1.0549	0.9367	1.0001	0.9628	0.0421
CALHM3	0.8553	1.0667	1.0839	0.8452	0.9628	0.0651
LOC642585	0.8960	1.0418	1.0328	0.8805	0.9628	0.0432
NKX6-1	0.8627	0.9773	1.0600	0.9511	0.9628	0.0406
OR7E156P	0.8881	1.0795	0.9810	0.9026	0.9628	0.0439
ZNF839	1.0625	1.1469	0.8540	0.7878	0.9628	0.0848
LOC392435	0.9371	1.0551	0.9928	0.8662	0.9628	0.0402
SERPINI2	0.9876	1.0388	0.9702	0.8546	0.9628	0.0389
RNF5P1	0.9170	1.2529	0.9079	0.7734	0.9628	0.1021
C14orf48	0.8650	1.1247	0.9796	0.8819	0.9628	0.0596
LOC1001343	0.8443	0.9726	1.0194	1.0150	0.9628	0.0409
OCLM	0.8936	1.1354	0.9430	0.8793	0.9628	0.0591
LOC728820	0.6486	1.2646	1.2828	0.6552	0.9628	0.1795
LOC644886	0.9982	1.0780	0.9536	0.8214	0.9628	0.0537
GPR21	1.0179	1.0355	0.9463	0.8517	0.9628	0.0418

C10orf107	0.9872	0.9710	0.9904	0.9029	0.9628	0.0204
ZNF514	0.9011	1.0567	1.0270	0.8665	0.9628	0.0466
LOC642653	0.8968	0.9441	0.9872	1.0233	0.9628	0.0273
LOC118945	0.8483	1.0936	1.0256	0.8840	0.9628	0.0580
LOC1001335	0.8517	1.0351	1.0824	0.8822	0.9628	0.0566
OR51T1	0.9111	0.9607	1.1108	0.8687	0.9628	0.0528
SERINC5	0.9133	1.0512	0.9760	0.9110	0.9629	0.0331
CYTIP	0.8537	1.1086	0.9999	0.8892	0.9629	0.0577
C4orf47	0.8693	1.0120	1.0879	0.8823	0.9629	0.0527
FLJ45994	1.0259	0.9342	0.9974	0.8939	0.9629	0.0299
GNAT3	0.9469	1.0037	0.9567	0.9441	0.9629	0.0139
LOC643146	0.9112	1.0353	0.9687	0.9363	0.9629	0.0269
LOC647655	0.9321	1.0306	0.9941	0.8946	0.9629	0.0305
CFHR3	0.8844	1.0723	1.0115	0.8833	0.9629	0.0473
C1orf74	0.9102	1.1710	1.0205	0.7498	0.9629	0.0889
LOC644974	0.9689	0.9521	1.0248	0.9057	0.9629	0.0246
LOC1001307	0.9779	0.9006	1.0404	0.9326	0.9629	0.0303
MIR148A	0.9211	0.9855	1.0370	0.9079	0.9629	0.0300
OR2B3P	0.8537	1.0588	1.0301	0.9090	0.9629	0.0488
LOC149837	0.9309	1.0563	1.0167	0.8477	0.9629	0.0465
AHDC1	0.9723	1.0707	0.8961	0.9124	0.9629	0.0395
LOC650885	0.9136	1.0777	0.9530	0.9072	0.9629	0.0396
ARL9	0.9384	0.9837	1.0206	0.9088	0.9629	0.0247
GPR150	0.8339	1.1298	1.0003	0.8877	0.9629	0.0655
SEBOX	0.9767	1.0377	0.9686	0.8686	0.9629	0.0350
LOC1001321	0.9085	1.1526	0.9383	0.8522	0.9629	0.0657
LOC440905	0.8909	1.0535	0.9603	0.9469	0.9629	0.0337
HAO2	0.9620	1.0303	0.9832	0.8762	0.9629	0.0322
PAK6	0.9247	1.0452	0.9899	0.8918	0.9629	0.0342
LOC644856	0.8964	0.9792	0.9474	1.0286	0.9629	0.0278
TRIAP1	0.8362	1.2043	1.0645	0.7466	0.9629	0.1047
LOC1001316	0.9064	1.0458	1.0000	0.8994	0.9629	0.0359
IMMP2L	0.8779	1.2979	0.9683	0.7075	0.9629	0.1241
ADAR	0.8728	1.2916	0.9776	0.7097	0.9629	0.1226
SRPK3	0.9310	0.9932	0.9885	0.9390	0.9629	0.0162
HIST1H2AL	0.9526	1.0109	1.0355	0.8526	0.9629	0.0407
C14orf169	0.9384	1.0909	0.9268	0.8956	0.9629	0.0436
LOC1001307	0.9798	1.0706	0.9372	0.8641	0.9629	0.0431
LOC729570	0.9248	1.2069	0.9135	0.8065	0.9629	0.0856
GNB4	0.8789	1.1207	0.9871	0.8652	0.9629	0.0592
ALDH1L2	0.9121	0.9947	1.0354	0.9096	0.9629	0.0312
LOC400858	0.8376	1.0856	0.9836	0.9449	0.9629	0.0512
LOC643959	1.0189	0.9492	0.9847	0.8991	0.9629	0.0256
LOC1001306	0.8868	1.0728	0.9697	0.9225	0.9629	0.0404
ADAMTS15	0.8966	1.1458	0.9445	0.8649	0.9629	0.0631
LOC1001295	0.9937	1.0848	0.9423	0.8310	0.9630	0.0529

DLL4	0.8943	1.1003	0.8950	0.9622	0.9630	0.0485
LOC728836	1.0367	0.9831	0.8873	0.9448	0.9630	0.0315
SCARNA5	0.9668	0.9977	0.9710	0.9164	0.9630	0.0170
ACVRL1	0.8689	1.0814	1.0172	0.8844	0.9630	0.0516
ZMYM2	0.7769	1.4321	0.9579	0.6851	0.9630	0.1663
LOC728970	0.9553	0.9580	1.0387	0.8999	0.9630	0.0286
PTPRU	0.7637	1.2007	0.9925	0.8950	0.9630	0.0921
LOC644311	0.8935	1.0481	0.9958	0.9146	0.9630	0.0359
AKR1B1	0.8916	1.0641	1.0548	0.8415	0.9630	0.0566
IGFBPL1	0.9204	1.0150	1.0303	0.8863	0.9630	0.0353
FRYL	0.9003	1.1326	0.9771	0.8419	0.9630	0.0629
LOC727836	0.9962	1.0753	0.9772	0.8033	0.9630	0.0573
CSTB	0.8209	1.2223	0.9779	0.8309	0.9630	0.0936
MIR137	0.9436	1.0627	0.9213	0.9245	0.9630	0.0336
RHOBTB1	0.9191	1.1040	0.9855	0.8434	0.9630	0.0552
EMILIN2	0.9606	1.0175	1.0405	0.8334	0.9630	0.0463
LOC653537	0.9348	1.0014	0.9815	0.9343	0.9630	0.0169
C1QTNF2	0.9469	1.0436	0.9436	0.9180	0.9630	0.0276
LOC1001292	0.8850	1.1256	1.0116	0.8298	0.9630	0.0662
ETV4	0.8972	1.0194	1.0554	0.8800	0.9630	0.0437
LOC642335	0.9161	1.0102	0.9908	0.9350	0.9630	0.0223
LOC646890	0.9811	1.0351	0.9422	0.8936	0.9630	0.0300
PCDHB1	0.8411	1.0381	1.0039	0.9691	0.9630	0.0430
MGC34829	0.8513	1.0646	0.9719	0.9644	0.9630	0.0437
LOC729780	0.9209	1.0453	1.1200	0.7659	0.9630	0.0775
OSGIN2	0.7906	1.3022	0.9756	0.7838	0.9630	0.1215
LRRC6	0.9652	1.0755	0.9237	0.8878	0.9630	0.0407
MUC5B	0.8871	1.1619	0.9645	0.8387	0.9630	0.0712
LOC1001311	0.8831	1.0736	0.9527	0.9428	0.9631	0.0399
TBL1Y	0.9373	1.0526	1.0406	0.8217	0.9631	0.0537
LOC164380	0.9889	1.0775	0.9304	0.8554	0.9631	0.0469
ZNF169	0.8618	1.0437	0.9980	0.9488	0.9631	0.0389
LOC652161	0.9794	0.9811	0.9736	0.9181	0.9631	0.0151
KCNT1	0.9084	1.0492	1.0341	0.8605	0.9631	0.0465
LOC1001292	0.7444	1.1049	1.0599	0.9430	0.9631	0.0805
LOC642105	0.9110	1.0034	1.0610	0.8768	0.9631	0.0422
LOC731670	0.9189	1.0334	0.9853	0.9147	0.9631	0.0285
LOC649248	0.9767	1.0507	0.9712	0.8537	0.9631	0.0407
TBX22	0.8961	1.1164	0.9198	0.9200	0.9631	0.0514
LOC136288	0.8910	1.0862	0.9877	0.8874	0.9631	0.0472
LOC644122	0.9154	1.0584	0.9745	0.9040	0.9631	0.0353
VPS45	0.9371	1.4411	0.8137	0.6604	0.9631	0.1691
LOC116412	0.9528	1.0105	1.0349	0.8542	0.9631	0.0402
NPY5R	0.8730	1.1830	0.9536	0.8428	0.9631	0.0769
HTA	1.0276	1.0687	0.9072	0.8488	0.9631	0.0512
LOC441347	0.9295	1.0705	1.0171	0.8352	0.9631	0.0516

LOC642961	0.9252	1.0298	1.0403	0.8572	0.9631	0.0438
SNORD1C	0.7622	1.0511	1.1107	0.9284	0.9631	0.0770
CDC2L2	0.8933	1.2230	0.9487	0.7874	0.9631	0.0929
LOC1001282	0.8739	1.0371	1.0751	0.8664	0.9631	0.0543
USF2	1.0255	1.1614	0.9476	0.7180	0.9631	0.0929
HIST1H3G	0.7956	1.1268	0.9949	0.9352	0.9631	0.0687
LRRC30	0.9364	0.9125	1.0023	1.0014	0.9631	0.0229
LOC647400	0.9431	1.0595	1.0544	0.7956	0.9631	0.0620
KRTAP9-3	0.8942	0.9733	1.0377	0.9475	0.9632	0.0298
LOC391004	0.9285	0.9700	1.0281	0.9262	0.9632	0.0239
LOC644057	0.9706	0.9352	1.0125	0.9344	0.9632	0.0185
LOC643623	0.8757	1.0799	1.0319	0.8652	0.9632	0.0545
SOX11	0.9592	1.0990	0.8799	0.9146	0.9632	0.0481
LOC730121	1.0782	1.0392	0.9312	0.8041	0.9632	0.0615
FBP1	0.8931	1.1310	0.9432	0.8854	0.9632	0.0574
RGS8	0.8850	1.0958	0.8786	0.9933	0.9632	0.0515
COL6A3	0.9225	1.0954	0.9437	0.8912	0.9632	0.0454
SYCE1	0.9167	1.1164	0.9469	0.8728	0.9632	0.0533
VAMP4	0.8719	1.1511	1.0324	0.7975	0.9632	0.0795
PPP4R1L	0.8903	1.0917	0.9700	0.9009	0.9632	0.0463
RPS12	0.9342	0.9773	1.0525	0.8888	0.9632	0.0348
IL1F6	0.9182	1.0502	1.0141	0.8704	0.9632	0.0416
LOC652540	0.8910	1.0462	0.9618	0.9540	0.9632	0.0319
LOC642616	0.9316	0.9966	0.9742	0.9505	0.9632	0.0141
STK33	0.8802	1.2423	0.9033	0.8272	0.9632	0.0944
GBP6	0.9394	1.0426	0.9357	0.9352	0.9632	0.0265
LOC646956	0.9680	1.0378	0.9846	0.8625	0.9632	0.0367
GLB1L	0.8352	1.2044	0.9574	0.8559	0.9632	0.0847
MIR550-2	0.8909	1.1356	0.9027	0.9236	0.9632	0.0579
ID2B	0.9390	1.1127	1.0632	0.7381	0.9632	0.0835
LOC1001294	0.9099	1.0367	0.9910	0.9154	0.9632	0.0307
SLC25A32	1.0066	1.0467	0.9362	0.8634	0.9632	0.0404
C1orf226	0.8821	0.9981	1.0640	0.9088	0.9632	0.0417
BRPF3	0.8444	1.4830	0.8815	0.6441	0.9633	0.1809
LOC646262	0.7967	1.1085	1.0140	0.9339	0.9633	0.0660
SIGLEC1	0.9369	1.0648	0.9356	0.9156	0.9633	0.0342
TSC2	0.8235	1.5158	0.9118	0.6018	0.9633	0.1954
FLJ45121	0.8856	1.0136	1.0462	0.9076	0.9633	0.0393
SIGLEC6	0.8929	1.0158	1.0066	0.9378	0.9633	0.0292
MAGI2	0.9483	0.9719	1.0728	0.8601	0.9633	0.0437
LOC645964	1.0186	0.9936	1.0069	0.8340	0.9633	0.0434
UBTFL2	0.9615	1.1221	0.9198	0.8496	0.9633	0.0578
MIR374A	0.9685	1.0796	0.9096	0.8955	0.9633	0.0419
S100G	0.8648	1.0394	1.0680	0.8809	0.9633	0.0526
LCP2	0.8996	1.1359	1.0183	0.7994	0.9633	0.0729
PAGE1	0.8717	1.1952	0.9743	0.8120	0.9633	0.0842

LOC649851	0.9405	1.0531	1.0442	0.8154	0.9633	0.0555
CENPJ	0.9067	1.0865	1.0234	0.8367	0.9633	0.0563
KIAA1202	0.9619	1.0883	0.8595	0.9436	0.9633	0.0472
PLXNA4	0.8819	1.0882	0.9937	0.8894	0.9633	0.0488
KCNQ4	0.8714	1.0575	1.0226	0.9017	0.9633	0.0453
LOC650834	0.9482	0.9870	1.0660	0.8521	0.9633	0.0444
SLC36A2	1.0330	1.0605	0.9026	0.8572	0.9633	0.0494
LOC642109	0.9320	1.0050	1.0839	0.8324	0.9633	0.0535
NDUFB11	0.7514	1.3259	0.9998	0.7762	0.9634	0.1331
LOC1001302	0.8589	1.0680	1.0754	0.8510	0.9634	0.0626
MAP4	0.9154	1.1410	0.9482	0.8489	0.9634	0.0627
LOC401561	0.8246	1.2733	0.9247	0.8308	0.9634	0.1058
LPPR1	0.8878	1.1528	0.9672	0.8456	0.9634	0.0680
MBIP	0.8920	1.1521	1.0723	0.7371	0.9634	0.0930
PALM2	0.9107	1.1127	0.9863	0.8438	0.9634	0.0577
LOC645196	1.0471	0.9867	0.8813	0.9384	0.9634	0.0353
DIO2	0.8613	1.0847	0.9961	0.9114	0.9634	0.0491
LOC649784	0.9569	1.0069	1.0203	0.8694	0.9634	0.0342
TSHB	0.9336	0.9806	0.9842	0.9552	0.9634	0.0118
SIT1	1.0356	1.0609	0.9123	0.8447	0.9634	0.0512
LOC643879	0.8198	1.0237	0.9211	1.0889	0.9634	0.0590
LOC643935	0.8814	1.0616	1.0388	0.8718	0.9634	0.0504
LOC1001311	1.0089	1.0239	1.0125	0.8082	0.9634	0.0518
CSRNP3	0.9090	1.0795	1.0298	0.8352	0.9634	0.0557
HABP2	0.8757	1.0193	1.0885	0.8702	0.9634	0.0541
RAB33B	0.8946	1.0542	1.1596	0.7452	0.9634	0.0909
LPHN3	0.9111	1.0218	1.0390	0.8817	0.9634	0.0393
RNF157	0.8692	1.0837	1.0149	0.8858	0.9634	0.0516
LOC646353	0.8921	1.0487	0.9424	0.9704	0.9634	0.0327
LOC728910	0.9247	1.1004	0.9399	0.8886	0.9634	0.0469
LRRC19	0.9293	1.0357	1.0295	0.8592	0.9634	0.0424
HIST2H4B	0.6985	1.4391	1.0519	0.6642	0.9634	0.1811
LOC1001446	1.0264	1.0342	0.9204	0.8728	0.9634	0.0398
TUBD1	0.8374	1.1649	1.0300	0.8216	0.9635	0.0822
SAA1	0.9495	1.0846	0.9431	0.8766	0.9635	0.0436
GJB3	0.8780	1.1269	0.9316	0.9172	0.9635	0.0557
LOC220416	0.8092	1.0350	1.0749	0.9347	0.9635	0.0593
LOC388002	0.9532	1.0448	0.9891	0.8668	0.9635	0.0373
C4orf26	0.9466	1.1253	0.9464	0.8355	0.9635	0.0600
SLC12A5	0.9352	1.0523	0.9896	0.8768	0.9635	0.0375
TMTC1	0.8850	1.1088	1.0320	0.8281	0.9635	0.0647
C12orf69	0.9647	1.0685	0.9464	0.8743	0.9635	0.0401
LOC653680	0.9278	1.0823	0.9203	0.9236	0.9635	0.0396
CTXN3	0.8953	1.0934	0.9318	0.9334	0.9635	0.0442
LOC652231	0.8556	1.0676	0.9879	0.9429	0.9635	0.0443
C1orf180	0.8983	1.0467	1.0972	0.8118	0.9635	0.0659

ARHGEF11	0.8775	1.1380	1.0462	0.7923	0.9635	0.0785
RTEL1	0.8492	1.1277	1.0567	0.8204	0.9635	0.0759
LOC388532	0.6598	1.1419	1.3451	0.7071	0.9635	0.1672
ERCC-00004	0.8936	1.0060	1.0601	0.8943	0.9635	0.0416
SAGE1	0.9093	0.9690	0.9929	0.9828	0.9635	0.0187
LIMD2	0.9316	0.9722	0.9716	0.9786	0.9635	0.0107
LOC643814	0.9685	0.9319	1.0158	0.9379	0.9635	0.0192
LOC440742	0.9599	1.0900	0.9830	0.8211	0.9635	0.0553
LOC646766	0.9291	1.1856	0.9303	0.8090	0.9635	0.0793
KRTAP23-1	0.9177	1.0107	1.0338	0.8918	0.9635	0.0346
LOC400352	0.8981	1.0580	1.0427	0.8553	0.9635	0.0510
ST18	0.8169	1.1252	1.0261	0.8859	0.9635	0.0693
LOC653734	0.8572	1.0496	1.0454	0.9019	0.9635	0.0493
WFDC10A	0.9387	0.9838	0.9700	0.9616	0.9635	0.0095
LOC643320	0.9631	0.9667	1.0320	0.8924	0.9635	0.0285
LOC644958	0.9873	1.0608	0.9490	0.8570	0.9635	0.0424
LOC644059	0.8555	1.0339	1.0851	0.8797	0.9635	0.0566
KIAA1274	0.8716	1.1429	1.0098	0.8300	0.9635	0.0711
LOC652102	0.9324	0.9908	0.9831	0.9479	0.9635	0.0140
LOC399936	0.9211	1.0684	0.9860	0.8787	0.9636	0.0413
C13orf33	0.9241	1.0005	1.0058	0.9238	0.9636	0.0229
LOC653941	0.9328	1.1340	0.9998	0.7877	0.9636	0.0720
ZNF655	0.9609	1.0812	0.9371	0.8751	0.9636	0.0432
DMXL1	0.8255	1.1176	0.9807	0.9305	0.9636	0.0607
LOC346887	0.8472	1.1149	1.0269	0.8654	0.9636	0.0646
PLA2G12A	0.8751	1.1468	1.0450	0.7874	0.9636	0.0812
FNDC5	0.9741	0.9911	1.0159	0.8732	0.9636	0.0313
SYNC1	0.9470	1.0448	1.0154	0.8472	0.9636	0.0439
PLD5	0.8660	1.0522	1.0932	0.8429	0.9636	0.0637
LOC642869	0.9262	1.2062	0.9455	0.7765	0.9636	0.0893
LOC642187	0.9389	1.0193	1.0052	0.8910	0.9636	0.0299
LOC1001341	0.9036	1.0875	1.0581	0.8051	0.9636	0.0665
NOL6	0.9829	1.1805	0.9715	0.7194	0.9636	0.0945
OR8D1	0.9168	1.1345	0.9896	0.8135	0.9636	0.0675
AMOTL1	0.9861	1.0997	0.9986	0.7700	0.9636	0.0694
LOC338667	0.9460	1.1229	0.9970	0.7886	0.9636	0.0692
MIR548I2	0.9106	1.0680	1.0051	0.8709	0.9636	0.0447
LOC652879	0.8817	1.0575	0.9916	0.9238	0.9636	0.0386
LOC644024	0.8425	1.0789	1.0716	0.8615	0.9636	0.0646
LOC649186	0.9507	1.0360	1.0331	0.8349	0.9636	0.0473
SUNC1	0.9392	1.0534	0.9927	0.8693	0.9637	0.0391
ERCC-00061	0.9364	1.0313	0.9677	0.9191	0.9637	0.0247
RAPGEF4	0.8488	1.0778	1.0991	0.8289	0.9637	0.0723
LOC652383	0.9302	1.1224	0.9003	0.9017	0.9637	0.0534
LOC126860	0.9291	1.1022	0.9407	0.8827	0.9637	0.0478
LOC646572	0.8864	0.9684	1.0712	0.9288	0.9637	0.0395

LOC1001340	0.8762	1.0156	1.0404	0.9224	0.9637	0.0387
LOC728934	0.9030	1.1686	0.9280	0.8551	0.9637	0.0700
LOC642795	0.8534	1.0008	1.1168	0.8837	0.9637	0.0601
GALP	0.8944	1.0424	0.9918	0.9261	0.9637	0.0332
LOC645093	0.9275	0.9656	1.0192	0.9424	0.9637	0.0201
LOC644191	0.8334	1.2943	1.0743	0.6527	0.9637	0.1400
LOC728105	0.8841	1.2008	0.8727	0.8972	0.9637	0.0792
TMEM220	0.9212	1.0028	1.0478	0.8830	0.9637	0.0376
INTU	0.8381	1.1792	1.0270	0.8105	0.9637	0.0864
LOC727851	0.9860	0.9858	0.9981	0.8850	0.9637	0.0264
LOC728951	0.8978	1.0717	0.9707	0.9146	0.9637	0.0392
FLJ36031	0.8940	1.0007	1.0452	0.9150	0.9637	0.0356
LOC651825	0.9513	1.0749	0.9464	0.8823	0.9637	0.0403
CASP12	0.9509	0.9725	0.9908	0.9407	0.9637	0.0112
C1orf182	0.8567	1.2764	0.9465	0.7754	0.9637	0.1099
CABP2	0.9518	1.0362	1.0521	0.8148	0.9637	0.0543
LOC645893	0.9841	1.0204	0.9464	0.9042	0.9637	0.0250
LOC642365	0.8999	1.0306	1.0581	0.8665	0.9637	0.0474
NR1I3	0.9178	0.9784	1.0089	0.9499	0.9637	0.0195
MAGEC1	0.8814	1.0554	1.0234	0.8947	0.9638	0.0443
FADS3	1.0294	1.0880	1.0152	0.7224	0.9638	0.0820
LOC728468	0.9981	1.0039	0.9527	0.9004	0.9638	0.0240
LOC652373	0.9702	0.9435	1.0189	0.9225	0.9638	0.0208
LOC647384	0.8857	1.0934	1.0022	0.8739	0.9638	0.0520
LOC1001313	0.8606	1.0620	0.9236	1.0090	0.9638	0.0447
C10orf84	0.9859	1.1306	0.9139	0.8249	0.9638	0.0646
LOC642673	0.9137	1.0096	1.0232	0.9088	0.9638	0.0305
FAM125B	0.8402	1.2069	0.9149	0.8933	0.9638	0.0825
AGAP2	0.9320	1.0285	1.0171	0.8777	0.9638	0.0359
NTF5	0.9282	1.0601	0.9449	0.9221	0.9638	0.0324
C8orf49	0.8874	1.0776	1.0128	0.8775	0.9638	0.0488
LOC652620	0.8408	1.0347	0.9778	1.0020	0.9638	0.0426
LOC440456	0.8746	1.0344	0.9880	0.9583	0.9638	0.0336
CYP3A5	0.9800	1.0026	1.0070	0.8657	0.9638	0.0332
LOC400120	0.8168	1.0912	1.1116	0.8358	0.9638	0.0796
LOC651293	0.9356	0.9744	1.0497	0.8957	0.9638	0.0328
RNU2-1	0.9259	1.0189	0.9472	0.9634	0.9638	0.0199
LOC346157	0.9065	1.0341	1.1182	0.7965	0.9638	0.0707
LOC652886	1.0076	0.9740	1.0018	0.8720	0.9638	0.0315
C6orf12	0.8735	1.0598	0.9962	0.9260	0.9639	0.0407
CREB3L3	0.9578	1.1125	0.8546	0.9305	0.9639	0.0541
DEFA6	0.8550	1.0157	1.0303	0.9544	0.9639	0.0398
IL21R	0.9045	1.0865	0.9772	0.8873	0.9639	0.0453
SH2B1	1.0593	0.9677	0.9659	0.8625	0.9639	0.0402
SLC26A2	0.9265	1.0051	1.0834	0.8405	0.9639	0.0521
LOC1001347	0.9106	1.0929	0.9624	0.8896	0.9639	0.0456

PLEKHA4	0.9461	1.0213	1.0323	0.8558	0.9639	0.0408
RXFP3	0.9589	1.0095	1.0212	0.8658	0.9639	0.0354
MGAM	0.8603	1.0365	1.0010	0.9578	0.9639	0.0381
LOC652833	0.9800	0.9391	0.9923	0.9442	0.9639	0.0131
FOXL1	0.9993	0.9862	1.0369	0.8332	0.9639	0.0449
OR4F4	0.9111	1.1114	0.9728	0.8604	0.9639	0.0543
CSN1S2A	0.8963	1.1291	0.8920	0.9382	0.9639	0.0560
LOC727751	0.8023	1.1335	1.0480	0.8718	0.9639	0.0766
C9orf144B	0.8884	0.9596	1.0988	0.9089	0.9639	0.0474
LOC1001331	0.8104	1.0261	1.1171	0.9020	0.9639	0.0675
GPR83	0.8370	1.0867	0.9567	0.9753	0.9639	0.0511
LOC1001304	0.9114	1.0676	0.9509	0.9258	0.9639	0.0355
CST4	0.8940	1.0186	1.0371	0.9061	0.9640	0.0372
FLJ20184	0.9460	1.0973	0.9721	0.8405	0.9640	0.0528
LOC642620	1.0555	0.9404	1.0039	0.8560	0.9640	0.0430
LOC646396	0.8970	1.0616	1.0494	0.8479	0.9640	0.0538
YSK4	0.8933	1.0812	0.9548	0.9266	0.9640	0.0410
LOC1001308	0.9330	0.9822	1.0456	0.8952	0.9640	0.0325
SPACA3	0.8234	0.9864	1.0284	1.0178	0.9640	0.0477
LOC645293	1.0133	1.0436	0.9333	0.8658	0.9640	0.0402
TTTY13	0.9062	1.0150	0.9569	0.9778	0.9640	0.0227
LOC730704	0.8762	1.1005	1.0795	0.7998	0.9640	0.0745
LOC642633	1.0262	1.0421	0.9850	0.8027	0.9640	0.0551
FAM183B	0.9967	1.0345	0.9729	0.8519	0.9640	0.0395
LOC647723	0.9141	0.9820	1.0100	0.9499	0.9640	0.0207
RPS6KB2	0.9022	1.2589	0.9824	0.7126	0.9640	0.1134
KCNK2	0.8749	1.0177	1.0233	0.9402	0.9640	0.0352
DIO3	0.9515	1.0740	1.0190	0.8116	0.9640	0.0567
MGC26718	0.9844	1.0280	0.9367	0.9070	0.9640	0.0266
MIR624	0.8386	1.1078	1.0230	0.8867	0.9640	0.0618
CGGBP1	0.8660	1.3016	0.9405	0.7479	0.9640	0.1193
MUT	0.8800	1.3782	0.8903	0.7075	0.9640	0.1443
KIAA1276	0.8759	1.0970	1.0034	0.8798	0.9640	0.0533
SNORA50	0.8854	1.0025	1.0222	0.9460	0.9640	0.0308
C2orf66	0.8848	1.0077	1.0461	0.9176	0.9640	0.0377
CHST10	1.0051	1.1359	0.8586	0.8564	0.9640	0.0670
C6orf114	0.9049	1.1110	0.9727	0.8675	0.9640	0.0536
LOC642403	0.8710	1.0353	1.0231	0.9268	0.9640	0.0394
LOC1001335	0.8657	1.1106	1.0346	0.8454	0.9640	0.0647
HSPB7	0.8400	1.0870	1.0573	0.8719	0.9641	0.0630
KRTAP4-4	0.9712	1.0130	0.9824	0.8896	0.9641	0.0263
TRIM15	0.9298	1.0638	0.9512	0.9115	0.9641	0.0342
LOC729310	0.8583	1.0667	1.0187	0.9126	0.9641	0.0477
BICC1	0.9382	1.0643	0.9894	0.8644	0.9641	0.0421
NES	0.9201	1.0853	1.0299	0.8211	0.9641	0.0587
MAGED2	0.9070	1.1471	1.0003	0.8018	0.9641	0.0733

CALU	0.8591	1.3377	1.0010	0.6586	0.9641	0.1430
BTK	0.8283	1.1598	0.9650	0.9033	0.9641	0.0710
FAM41C	0.9910	0.9634	0.9620	0.9400	0.9641	0.0105
C10orf112	0.9647	1.1167	0.9655	0.8095	0.9641	0.0627
PDE4DIP	0.9340	1.0182	0.9870	0.9173	0.9641	0.0234
TMEM190	0.8802	0.9788	1.0459	0.9515	0.9641	0.0343
LOC644482	0.9668	0.9287	1.1655	0.7954	0.9641	0.0765
ARHGAP30	0.9077	1.0324	0.9894	0.9270	0.9641	0.0287
PARP11	0.9466	1.1115	0.9379	0.8605	0.9641	0.0528
LOC642568	0.9807	1.0661	0.9118	0.8979	0.9641	0.0385
ARID1B	0.8840	1.0211	1.0527	0.8987	0.9641	0.0426
TMEM102	0.9424	1.0896	0.9377	0.8867	0.9641	0.0437
RAB10	0.8250	1.2707	0.9941	0.7666	0.9641	0.1130
LOC643981	0.8979	1.0537	0.9425	0.9624	0.9641	0.0328
GPR110	0.9214	1.0709	0.9798	0.8844	0.9641	0.0407
LOC440320	0.9178	1.0295	1.0164	0.8929	0.9641	0.0344
LOC1001295	0.8910	1.0747	1.0651	0.8258	0.9641	0.0625
A3GALT2	0.9550	1.0527	0.9566	0.8923	0.9642	0.0331
ALKBH5	0.8654	1.2638	1.0071	0.7203	0.9642	0.1158
LOC730382	0.9205	0.9691	1.1787	0.7884	0.9642	0.0811
SNORA54	0.8392	1.0387	1.0742	0.9046	0.9642	0.0554
LOC400347	0.9443	1.1033	0.9294	0.8796	0.9642	0.0484
SNORD55	0.8047	1.2174	1.0770	0.7575	0.9642	0.1099
MIR222	0.9251	1.0009	0.9892	0.9415	0.9642	0.0183
MMP8	0.8667	1.0474	1.0308	0.9118	0.9642	0.0443
MIR373	1.0487	0.9737	1.0556	0.7787	0.9642	0.0646
LOC644391	0.9456	0.9611	1.0548	0.8953	0.9642	0.0333
PIWIL1	0.9752	1.0385	0.9326	0.9104	0.9642	0.0282
EFCAB6	0.9009	1.0475	1.0029	0.9054	0.9642	0.0364
ZNF498	0.9398	1.0730	0.9412	0.9028	0.9642	0.0373
LOC652290	0.9344	1.1181	1.0046	0.7996	0.9642	0.0666
LOC1001335	0.9328	0.9789	1.0101	0.9350	0.9642	0.0186
LOC1001296	0.9733	1.0562	0.8871	0.9402	0.9642	0.0354
LOC652191	0.9197	1.0407	1.0062	0.8903	0.9642	0.0354
LOC642580	0.9208	1.1496	0.9645	0.8218	0.9642	0.0686
SCRN1	0.8921	1.0500	0.9342	0.9806	0.9642	0.0338
LOC1001308	1.0444	1.0572	0.9470	0.8084	0.9642	0.0575
LCE3B	0.9707	0.9258	1.0282	0.9322	0.9642	0.0235
LOC729371	0.9240	1.1197	0.9584	0.8549	0.9642	0.0561
MIR577	0.8202	1.1694	1.0584	0.8090	0.9642	0.0893
DUSP6	0.8317	1.0519	1.0788	0.8945	0.9642	0.0600
LOC642974	0.9568	1.0905	0.9619	0.8477	0.9642	0.0496
GRM7	0.9335	0.9921	1.0292	0.9022	0.9642	0.0286
LOC1001338	0.8670	1.1615	1.0157	0.8128	0.9642	0.0785
LOC645416	0.8937	1.0990	1.0697	0.7945	0.9642	0.0725
FAM7A2	0.9531	1.0295	0.9879	0.8866	0.9642	0.0302

LOC1001331	1.0123	1.0356	1.0407	0.7683	0.9642	0.0656
IL18BP	0.9004	1.0744	1.0004	0.8817	0.9642	0.0450
LOC645914	0.9992	0.8710	1.1223	0.8645	0.9643	0.0611
LOC644705	0.9704	1.0882	0.9723	0.8261	0.9643	0.0536
CASS4	0.8490	1.1424	0.9764	0.8893	0.9643	0.0651
ELP2P	1.0672	1.0000	0.9608	0.8291	0.9643	0.0501
CIZ1	0.9214	1.0519	0.9661	0.9177	0.9643	0.0312
MIR548A3	0.9597	1.0728	0.9810	0.8436	0.9643	0.0471
LOC729370	0.9213	1.1225	0.9476	0.8657	0.9643	0.0554
TPPP2	0.9367	1.0035	0.9601	0.9567	0.9643	0.0141
LOC1001293	0.8618	1.0772	1.0007	0.9174	0.9643	0.0472
VPS53	0.8853	1.0864	1.0145	0.8709	0.9643	0.0520
CCL28	0.8709	1.0936	1.0079	0.8847	0.9643	0.0530
YJEFN3	0.9608	1.0321	0.9846	0.8797	0.9643	0.0319
LOC440786	0.7506	1.1593	1.0070	0.9403	0.9643	0.0847
LOC400986	0.9629	1.0219	0.9981	0.8744	0.9643	0.0323
CLCA2	0.8143	1.0425	1.0680	0.9325	0.9643	0.0580
MIR548E	1.0023	1.0308	1.0217	0.8025	0.9643	0.0543
AICDA	0.9432	1.0841	0.9413	0.8887	0.9643	0.0419
LOC1001307	0.9628	1.0652	0.9811	0.8482	0.9643	0.0447
THAP9	0.9108	0.9649	1.0881	0.8935	0.9643	0.0440
HIST1H4I	0.9909	1.1401	1.0391	0.6873	0.9643	0.0974
LOC1001339	0.9320	1.0385	0.9705	0.9163	0.9643	0.0272
CATSPER3	0.8813	1.1557	0.9159	0.9045	0.9643	0.0642
MIR1280	0.8629	1.0509	0.9950	0.9486	0.9644	0.0398
NKX6-2	0.8416	1.0673	1.0411	0.9075	0.9644	0.0538
LOC1001292	0.9042	0.9649	1.1294	0.8589	0.9644	0.0591
FMN2	0.9602	1.0210	1.0251	0.8512	0.9644	0.0405
LOC728395	0.8890	1.1024	1.0348	0.8312	0.9644	0.0629
LOC652260	0.9434	1.0968	0.9159	0.9014	0.9644	0.0450
APOA4	0.9233	1.0899	0.9577	0.8867	0.9644	0.0443
OR2A5	0.8454	1.0472	1.0417	0.9232	0.9644	0.0489
LOC1001347	0.9888	1.0033	0.9958	0.8695	0.9644	0.0317
LOC727789	0.8608	1.0583	0.9820	0.9565	0.9644	0.0407
LOC644291	0.9208	1.0214	1.0286	0.8867	0.9644	0.0357
TAS2R5	0.9130	1.0408	0.9945	0.9093	0.9644	0.0322
ZNF79	0.8360	1.1369	1.1201	0.7645	0.9644	0.0959
ZNF662	0.9780	1.0921	0.8938	0.8936	0.9644	0.0470
LOC1001300	0.9973	1.0684	0.9651	0.8268	0.9644	0.0507
LOC729004	0.8507	1.0763	1.0792	0.8513	0.9644	0.0655
PCDHGA8	0.9719	0.9220	1.1126	0.8512	0.9644	0.0552
IL1F8	0.9354	1.0799	0.9800	0.8623	0.9644	0.0455
CCDC102B	0.9980	1.1090	0.9506	0.8000	0.9644	0.0641
LOC642804	0.7924	1.1518	0.9701	0.9434	0.9644	0.0737
FLJ37453	0.9203	1.0185	1.0165	0.9022	0.9644	0.0309
MUC7	0.9111	1.0941	0.9486	0.9038	0.9644	0.0443

LOC1001331	0.8994	0.9672	0.9683	1.0228	0.9644	0.0253
LOC285444	0.8751	1.0981	0.9341	0.9504	0.9644	0.0474
LOC392288	0.9635	1.0347	0.9720	0.8874	0.9644	0.0302
LOC127150	0.8686	1.1371	1.0578	0.7943	0.9644	0.0799
LOC440992	0.9066	1.1444	0.9305	0.8763	0.9644	0.0610
LOC649765	0.9378	1.1383	0.8390	0.9427	0.9644	0.0627
LOC651029	0.9133	1.1443	0.9696	0.8306	0.9644	0.0664
ANKRD36B	0.9229	1.1815	0.9182	0.8352	0.9645	0.0751
LOC652252	0.9469	1.0186	1.0652	0.8271	0.9645	0.0518
ZNF8	0.9656	1.0049	0.9663	0.9210	0.9645	0.0171
ZNF438	0.9757	1.1275	0.9328	0.8219	0.9645	0.0633
SNORD93	0.9287	1.0232	1.0668	0.8391	0.9645	0.0508
F10	0.9150	1.0683	0.9436	0.9310	0.9645	0.0351
LOC649738	0.9025	1.0356	0.9406	0.9792	0.9645	0.0284
CT45A1	0.9334	1.0728	0.9408	0.9109	0.9645	0.0367
KLRG2	0.9405	0.9685	1.0729	0.8760	0.9645	0.0410
EIF4E2	0.7485	1.4688	0.9323	0.7083	0.9645	0.1750
LOC643356	0.9665	1.0697	0.9226	0.8991	0.9645	0.0377
PACS2	0.9605	1.1449	0.9135	0.8391	0.9645	0.0651
AUTS2	1.0677	0.9509	1.1206	0.7188	0.9645	0.0892
FAM39DP	0.8880	1.2759	0.9199	0.7742	0.9645	0.1084
LOC1001331	0.7971	1.0448	1.0483	0.9679	0.9645	0.0588
ZNF778	0.9293	1.0546	1.0206	0.8536	0.9645	0.0455
IL11	1.0373	1.0764	0.9144	0.8300	0.9645	0.0566
LOC649200	0.9170	1.0209	0.9482	0.9720	0.9645	0.0219
KRT79	0.9122	1.0919	1.0225	0.8315	0.9645	0.0577
LOC729154	0.8620	1.0510	0.9454	0.9997	0.9645	0.0404
LOC1001288	0.9537	1.0635	0.9903	0.8507	0.9645	0.0443
SEC11B	0.9714	1.0182	0.9368	0.9317	0.9645	0.0199
AP3S1	0.8312	1.3745	0.9118	0.7407	0.9646	0.1410
FLJ46300	0.8454	1.0629	1.0193	0.9307	0.9646	0.0483
LOC648222	0.9871	1.0297	0.9528	0.8886	0.9646	0.0298
MBD5	0.9082	1.1407	0.9472	0.8621	0.9646	0.0612
LOC1001286	0.8684	1.0996	1.0004	0.8897	0.9646	0.0535
LOC728401	0.9346	0.9859	1.0223	0.9155	0.9646	0.0243
C6orf148	0.7826	1.4040	0.8948	0.7769	0.9646	0.1490
PZP	0.9455	1.0807	0.9122	0.9198	0.9646	0.0394
GRM1	0.9208	1.0022	1.0482	0.8871	0.9646	0.0369
MMP13	0.8884	1.1119	1.0218	0.8362	0.9646	0.0627
NHEJ1	0.9053	1.0019	0.9832	0.9679	0.9646	0.0210
PDK4	0.9746	1.0579	0.9373	0.8885	0.9646	0.0357
FARSB	0.8573	1.0579	1.0508	0.8924	0.9646	0.0523
LOC731797	0.8739	1.0341	1.0229	0.9275	0.9646	0.0386
LAYN	0.8955	1.1253	0.9003	0.9374	0.9646	0.0544
C1orf194	0.9439	1.0000	1.0170	0.8975	0.9646	0.0273
LOC729915	0.8614	1.0183	0.9964	0.9823	0.9646	0.0352

PRDM15	0.9312	0.9838	0.9419	1.0014	0.9646	0.0167
LOC728473	0.9731	1.0804	0.8930	0.9119	0.9646	0.0422
SNX32	0.7646	1.0311	1.0586	1.0042	0.9646	0.0676
SEMA6B	0.9355	0.9419	1.0616	0.9194	0.9646	0.0327
MIR138-1	0.9077	1.0522	0.9332	0.9654	0.9646	0.0315
LOC650887	1.0832	1.0260	0.9400	0.8093	0.9646	0.0595
USP4	0.8903	1.1592	1.0000	0.8091	0.9646	0.0757
LOC652156	0.9251	1.0300	0.9774	0.9259	0.9646	0.0250
LOC642981	0.8405	1.0942	1.0287	0.8951	0.9646	0.0585
FBLN1	0.8838	1.0965	0.9658	0.9125	0.9646	0.0471
USP50	0.8694	1.0241	1.0314	0.9338	0.9647	0.0388
TRIM60	0.9164	0.9939	1.0139	0.9344	0.9647	0.0233
LOC644143	0.8869	1.0168	1.0232	0.9318	0.9647	0.0333
SPP2	0.9208	1.0788	0.9895	0.8696	0.9647	0.0453
C14orf70	0.8676	1.0315	1.0899	0.8698	0.9647	0.0567
LOC647262	0.9548	0.9718	0.9968	0.9353	0.9647	0.0130
C14orf139	0.9701	0.9911	0.9963	0.9012	0.9647	0.0219
LOC642583	0.9582	1.0606	1.0008	0.8391	0.9647	0.0468
TAS2R4	0.9129	0.9864	1.0280	0.9316	0.9647	0.0262
C10orf92	0.9284	1.0873	0.9834	0.8597	0.9647	0.0481
LOC642361	0.8782	1.2375	0.9330	0.8101	0.9647	0.0944
DAB2IP	0.8629	1.1244	1.0014	0.8701	0.9647	0.0620
LOC728912	0.9328	0.9866	1.0219	0.9177	0.9647	0.0241
LOC643749	1.0525	0.8794	0.9603	0.9668	0.9647	0.0354
FBXL14	0.8644	1.1031	1.0269	0.8645	0.9647	0.0600
LOC647224	1.0051	1.0930	0.9189	0.8418	0.9647	0.0542
LOC1001289	0.8586	1.1023	1.0368	0.8613	0.9647	0.0620
LOC644814	0.9131	1.0740	0.9476	0.9242	0.9647	0.0371
FAM55D	0.9170	1.0558	0.9813	0.9049	0.9647	0.0347
LOC644957	0.9210	1.0660	1.0346	0.8374	0.9647	0.0526
FOXP3	0.9495	1.0067	1.0517	0.8511	0.9648	0.0433
FAM26D	0.9873	0.8372	1.0523	0.9822	0.9648	0.0454
LOC391655	0.9606	1.0058	1.0791	0.8135	0.9648	0.0560
TMC7	0.9859	1.0178	0.9936	0.8617	0.9648	0.0350
LOC1001343	0.9117	1.0664	1.0212	0.8597	0.9648	0.0478
LOC1001326	0.8530	1.0630	1.0376	0.9055	0.9648	0.0508
PTPRV	0.8859	0.9710	1.0085	0.9936	0.9648	0.0274
CARD9	0.9461	1.0776	0.9472	0.8883	0.9648	0.0400
STK31	0.9387	1.0477	1.0133	0.8595	0.9648	0.0418
TTN	0.8784	1.0409	1.0044	0.9355	0.9648	0.0361
ATP11A	0.9730	1.0212	0.9669	0.8980	0.9648	0.0254
MGC40168	0.9247	1.0439	0.9305	0.9601	0.9648	0.0275
LOC283874	0.8861	1.0333	1.0095	0.9303	0.9648	0.0342
LOC1001331	0.9158	1.0086	0.8695	1.0652	0.9648	0.0442
DAND5	0.8510	1.0147	0.9364	1.0571	0.9648	0.0454
LOC1001296	0.8903	1.0484	1.0390	0.8816	0.9648	0.0456

DNASE1L1	0.8753	1.1286	1.0382	0.8172	0.9648	0.0719
C20orf152	0.9444	1.0904	0.9861	0.8385	0.9648	0.0521
LOC651017	0.9998	1.0099	1.0304	0.8192	0.9648	0.0490
LOC642780	0.9350	1.0615	0.9771	0.8857	0.9648	0.0372
LOC283683	0.8493	1.0562	1.0766	0.8772	0.9648	0.0591
LOC728615	0.8696	1.0568	1.0100	0.9229	0.9648	0.0422
ACRC	0.9562	0.9976	1.1172	0.7883	0.9648	0.0680
APOL1	0.8608	1.0254	1.0291	0.9441	0.9649	0.0398
LOC1001343	0.7219	0.8780	1.1819	1.0776	0.9649	0.1026
ACADL	0.9025	1.0627	0.9684	0.9258	0.9649	0.0353
MGC15705	0.9086	1.0103	1.0895	0.8511	0.9649	0.0530
SEC22B	0.8412	1.3468	0.8855	0.7860	0.9649	0.1289
LTBP1	0.9295	0.9587	1.0395	0.9318	0.9649	0.0258
GCET2	0.8877	1.1533	0.9759	0.8426	0.9649	0.0686
LOC647582	0.9672	1.0258	1.0324	0.8341	0.9649	0.0460
MMP14	0.8764	0.9672	1.0202	0.9957	0.9649	0.0314
LOC389156	0.5282	1.2391	1.4203	0.6718	0.9649	0.2159
LOC1001314	0.9343	1.0800	0.9935	0.8518	0.9649	0.0481
LOC731002	0.9210	1.0417	0.9879	0.9089	0.9649	0.0309
RTP1	0.9309	1.1159	0.9096	0.9032	0.9649	0.0507
APOBEC1	0.8705	1.0579	0.9853	0.9458	0.9649	0.0391
PCDHA9	0.8750	1.0822	1.0009	0.9015	0.9649	0.0476
NPHS2	0.9777	1.0992	0.9702	0.8125	0.9649	0.0588
AMY2B	0.9609	1.0572	0.9595	0.8821	0.9649	0.0358
FAM48B1	0.9288	1.0726	0.9713	0.8870	0.9649	0.0398
RGS7BP	0.8803	0.9781	1.0476	0.9536	0.9649	0.0345
LOC728393	0.8609	1.0497	1.0236	0.9254	0.9649	0.0438
SLC16A13	0.8802	1.1685	0.9753	0.8357	0.9649	0.0738
LOC652200	0.9650	0.9971	1.0192	0.8783	0.9649	0.0309
LOC644739	0.9526	1.1411	0.9610	0.8050	0.9649	0.0688
LOC728150	0.9637	0.9633	1.0401	0.8926	0.9649	0.0301
SEC11A	0.7757	1.3787	0.9218	0.7835	0.9649	0.1419
LYZL6	0.9560	1.1860	0.8401	0.8777	0.9649	0.0775
C1orf146	0.9792	1.0521	0.9930	0.8354	0.9649	0.0460
RAET1G	0.9383	1.0613	1.0104	0.8498	0.9650	0.0459
ENKUR	0.9497	0.9606	1.0516	0.8979	0.9650	0.0320
PM20D1	1.0355	1.0844	0.9076	0.8324	0.9650	0.0578
C12orf29	0.8055	1.1530	1.1842	0.7172	0.9650	0.1191
LOC440900	0.8987	1.0333	1.1078	0.8201	0.9650	0.0648
LOC1001328	0.8799	1.0648	0.9581	0.9570	0.9650	0.0380
PAGE2	0.9832	1.0345	0.9535	0.8887	0.9650	0.0304
DMD	0.9174	1.0941	0.9692	0.8792	0.9650	0.0468
RYR1	0.8725	1.0250	1.0410	0.9214	0.9650	0.0406
KCNH8	0.8498	1.1409	1.0262	0.8430	0.9650	0.0724
MIR122	0.8471	1.0909	1.0200	0.9019	0.9650	0.0553
LOC653924	0.8619	1.0555	0.9378	1.0047	0.9650	0.0420

MIR1324	0.9557	1.0833	0.9208	0.9002	0.9650	0.0411
LOC1001287	0.8967	1.0328	1.1073	0.8232	0.9650	0.0643
ILDR1	0.9730	1.0663	0.9526	0.8681	0.9650	0.0407
LOC1001309	0.8452	1.0428	1.0627	0.9092	0.9650	0.0525
LOC642692	0.9219	1.1799	0.8939	0.8643	0.9650	0.0726
LOC652492	0.8752	1.0725	1.0528	0.8595	0.9650	0.0566
SLC26A5	0.8710	1.0999	0.9891	0.9001	0.9650	0.0515
GFOD1	0.7654	1.4345	0.9187	0.7414	0.9650	0.1614
MIR635	1.0150	1.1135	0.7336	0.9980	0.9650	0.0812
LOC642188	0.8778	0.9678	1.0894	0.9250	0.9650	0.0454
C20orf96	0.9058	1.0968	0.8816	0.9758	0.9650	0.0483
SNRNP35	0.9154	1.1628	0.9548	0.8271	0.9650	0.0711
BMP2K	0.9300	1.0124	1.0341	0.8836	0.9650	0.0352
MIR939	0.9075	1.0305	1.0267	0.8954	0.9650	0.0368
LOC728217	0.9384	1.0193	0.9815	0.9209	0.9650	0.0221
LOC375295	0.9653	1.0327	0.9683	0.8937	0.9650	0.0284
OR4D9	0.9044	1.0085	1.0687	0.8785	0.9650	0.0445
LOC648089	0.8886	1.0689	0.9987	0.9040	0.9651	0.0423
PTCHD3	0.9266	1.0837	0.9898	0.8601	0.9651	0.0476
CDRT15L2	0.8710	1.1723	0.9527	0.8642	0.9651	0.0720
SCN2B	1.0413	1.0192	0.9427	0.8570	0.9651	0.0418
FLJ16369	0.8683	1.0394	1.1103	0.8423	0.9651	0.0652
KIAA1383	0.9303	1.0976	0.9813	0.8511	0.9651	0.0517
LOC642468	0.9035	1.1134	1.0110	0.8324	0.9651	0.0616
TGM7	0.8946	0.9604	1.0825	0.9228	0.9651	0.0414
LOC729590	0.9100	0.9905	0.9893	0.9705	0.9651	0.0189
GTF2IRD2	0.9998	1.0333	0.9189	0.9085	0.9651	0.0305
LOC728624	0.9394	1.0096	0.9863	0.9250	0.9651	0.0198
SNORD127	0.8665	1.0187	0.9814	0.9938	0.9651	0.0338
DEFB104A	0.8363	1.1089	0.9876	0.9276	0.9651	0.0571
LOC1001286	0.8810	1.0344	1.0374	0.9076	0.9651	0.0412
LOC653689	0.9748	1.0551	1.0083	0.8223	0.9651	0.0504
LOC644011	0.8603	1.1489	0.9886	0.8627	0.9651	0.0682
ACCS	0.8502	1.0288	1.0678	0.9137	0.9651	0.0504
LOC727863	0.9448	1.0428	0.9571	0.9158	0.9651	0.0273
CBLC	0.9617	1.0906	0.9487	0.8594	0.9651	0.0476
VCAM1	0.9399	1.0848	0.9197	0.9161	0.9651	0.0402
S100A5	0.8799	1.1345	0.9294	0.9167	0.9651	0.0574
SNHG11	0.7656	1.1646	1.0603	0.8699	0.9651	0.0903
MPG	0.9225	1.0715	0.9725	0.8940	0.9651	0.0390
FLJ21986	0.9048	1.0738	1.0184	0.8634	0.9651	0.0488
TRIM29	0.9391	1.0282	1.0250	0.8682	0.9651	0.0383
LOC651530	0.9111	1.1118	0.9403	0.8974	0.9651	0.0497
RNF31	0.8524	1.3321	0.9847	0.6914	0.9651	0.1362
PRPF18	0.8601	1.0537	1.1497	0.7970	0.9651	0.0823
C17orf73	0.8713	1.2244	0.9396	0.8253	0.9651	0.0895

C14orf183	0.8916	1.1160	0.9700	0.8829	0.9651	0.0540
HBZ	0.9501	1.1151	0.9680	0.8274	0.9652	0.0589
LOC1001336	0.9672	1.0520	0.9426	0.8988	0.9652	0.0322
ERCC-00024	0.8750	0.9947	1.0383	0.9526	0.9652	0.0348
LOC1001284	0.9528	0.9944	0.9584	0.9550	0.9652	0.0098
SSR3	0.7857	1.3015	0.9453	0.8281	0.9652	0.1171
LOC729467	0.9858	1.0299	1.0040	0.8410	0.9652	0.0424
LOC649030	0.9475	1.0081	1.0209	0.8842	0.9652	0.0314
RPS26L	0.8509	1.1922	1.1221	0.6954	0.9652	0.1162
STK17A	0.8542	1.1036	1.1048	0.7980	0.9652	0.0811
GPX7	0.9677	1.0336	0.9607	0.8988	0.9652	0.0276
OR2Y1	0.9510	1.0099	0.9754	0.9245	0.9652	0.0182
UBXD7	0.9083	1.1423	0.9173	0.8929	0.9652	0.0592
LOC652597	1.0093	1.0811	0.9335	0.8368	0.9652	0.0523
SNX17	0.7332	1.4522	0.8949	0.7805	0.9652	0.1659
UBTFL5	0.9370	0.9695	1.1256	0.8287	0.9652	0.0614
OR2W3	0.7800	1.1214	1.0576	0.9017	0.9652	0.0771
ZNF468	0.8725	1.0415	1.0718	0.8750	0.9652	0.0532
ZNF43	0.9022	1.0966	0.9644	0.8977	0.9652	0.0464
CPNE7	0.8910	1.0245	1.0411	0.9043	0.9652	0.0392
CUL9	0.9225	1.1198	1.0102	0.8084	0.9652	0.0660
RAB6C	0.9530	1.0933	0.9010	0.9135	0.9652	0.0441
CCDC108	0.9241	1.0753	0.9790	0.8824	0.9652	0.0417
LOC441416	0.8429	1.0807	1.0735	0.8638	0.9652	0.0647
FBXO27	0.9633	1.0079	0.9824	0.9072	0.9652	0.0214
LOC1001293	0.9489	1.0067	1.0051	0.9002	0.9652	0.0255
LOC730385	0.9623	0.9628	0.9749	0.9609	0.9652	0.0033
LOC389118	0.9281	1.0293	0.9544	0.9492	0.9652	0.0221
LOC642996	0.9456	1.0073	1.0041	0.9039	0.9652	0.0249
LECT2	0.9302	0.9948	0.9796	0.9563	0.9652	0.0141
USP2	0.8838	1.1953	0.9573	0.8245	0.9652	0.0814
LOC732443	0.8599	1.1207	1.0931	0.7874	0.9652	0.0833
ITGB8	0.9312	1.0494	0.9740	0.9064	0.9653	0.0313
NLRP7	0.9254	1.0617	1.0388	0.8352	0.9653	0.0526
NKAIN3	1.0031	1.1229	0.9094	0.8255	0.9653	0.0639
KCNMB2	0.8834	1.0287	1.0373	0.9117	0.9653	0.0396
LOC441773	0.8463	1.0865	1.0424	0.8858	0.9653	0.0585
C11orf70	0.9384	1.0816	0.9696	0.8716	0.9653	0.0438
LOC643884	0.9723	1.0872	0.9670	0.8347	0.9653	0.0516
LOC729218	0.9717	1.0505	1.0153	0.8237	0.9653	0.0499
LOC645951	0.9127	1.1926	0.9341	0.8217	0.9653	0.0796
LOC401433	0.9568	1.0602	0.9848	0.8593	0.9653	0.0415
FAM127B	0.8702	1.3010	0.9229	0.7671	0.9653	0.1165
C1QTNF5	0.9787	1.0868	0.9245	0.8712	0.9653	0.0461
TSHZ1	0.9849	1.2779	0.9244	0.6740	0.9653	0.1240
LOC641815	0.9846	1.0035	1.0083	0.8648	0.9653	0.0339

LOC1001315	0.9142	1.0586	1.0133	0.8750	0.9653	0.0426
LOC642252	0.8958	1.1012	0.9516	0.9126	0.9653	0.0468
RBM11	0.9280	1.0436	0.9773	0.9124	0.9653	0.0295
LOC1001339	0.9223	1.0873	0.9156	0.9360	0.9653	0.0409
FGF9	1.0105	0.9805	0.9748	0.8955	0.9653	0.0246
NFKBIL2	0.9100	1.0843	0.9835	0.8834	0.9653	0.0450
LOC653641	1.0189	1.0410	0.9073	0.8940	0.9653	0.0377
LOC1001285	0.9224	1.0335	1.0118	0.8936	0.9653	0.0339
LOC146439	0.7029	1.2592	1.0804	0.8189	0.9653	0.1258
PPP3CC	0.8785	1.2804	0.9688	0.7336	0.9653	0.1157
FLJ42289	0.8680	1.1218	1.0039	0.8677	0.9653	0.0612
LOC1001309	0.8102	1.0035	1.0880	0.9597	0.9653	0.0582
INPP5A	0.9576	1.1732	0.9711	0.7594	0.9653	0.0845
ZFAT	0.9457	1.0252	1.0030	0.8875	0.9654	0.0309
ERCC-00071	0.8796	1.1023	0.9654	0.9141	0.9654	0.0489
UGT1A7	0.9619	1.0300	1.0460	0.8235	0.9654	0.0507
LOC644413	0.9531	1.0365	0.9123	0.9595	0.9654	0.0259
LOC645708	0.9019	1.0891	1.0208	0.8496	0.9654	0.0546
GPR85	0.9057	1.0637	0.9230	0.9691	0.9654	0.0354
FAM87A	0.8633	1.0640	1.1329	0.8013	0.9654	0.0791
IQSEC1	0.8684	1.2929	0.8468	0.8534	0.9654	0.1093
FMR1NB	0.8541	1.0144	1.0082	0.9848	0.9654	0.0376
FIS1	0.9077	1.2560	0.9935	0.7044	0.9654	0.1143
LOC1001318	0.8249	1.1054	1.0582	0.8731	0.9654	0.0686
LOC650799	0.9423	1.0595	0.9886	0.8711	0.9654	0.0396
LOC388474	0.8879	1.1915	0.9928	0.7895	0.9654	0.0860
STYX	0.8633	1.1249	1.0071	0.8663	0.9654	0.0629
FETUB	0.9870	1.1157	0.9040	0.8550	0.9654	0.0570
DEFA3	0.8694	1.0839	1.0130	0.8953	0.9654	0.0504
PANK4	1.1017	1.0862	0.9336	0.7402	0.9654	0.0841
KL	0.9563	1.0315	0.8957	0.9782	0.9654	0.0281
LOC643943	0.8407	1.1078	1.0323	0.8810	0.9654	0.0629
KRT84	0.8161	1.1528	1.0419	0.8509	0.9654	0.0798
MIR129-1	0.8941	1.0556	1.0236	0.8885	0.9654	0.0433
ARL8B	0.9285	1.1801	1.1530	0.6001	0.9654	0.1342
NDUFS4	0.8572	1.2034	1.0861	0.7150	0.9654	0.1102
LOC646574	0.9124	1.0214	1.0337	0.8942	0.9654	0.0361
LOC646604	0.9787	0.9257	1.0063	0.9510	0.9654	0.0174
LOC645879	0.9667	1.0623	0.9919	0.8409	0.9655	0.0462
ACMSD	0.8970	1.0788	1.0615	0.8245	0.9655	0.0623
CD79A	0.8517	1.0754	1.0914	0.8433	0.9655	0.0682
DIRAS1	0.9093	1.0669	1.0196	0.8661	0.9655	0.0468
LOC1001327	0.9621	1.0388	1.0130	0.8479	0.9655	0.0423
MIR1276	0.9181	1.0855	0.9217	0.9365	0.9655	0.0402
LOC1001302	0.9903	1.0356	0.9566	0.8794	0.9655	0.0329
TCF15	0.9010	1.0199	1.0499	0.8911	0.9655	0.0406

LOC651896	0.9198	1.0739	0.9056	0.9625	0.9655	0.0381
LOC648934	0.8620	1.1252	0.8862	0.9885	0.9655	0.0599
ZNF491	0.9153	1.0943	0.9305	0.9218	0.9655	0.0431
SNORA15	1.0249	0.9689	0.9438	0.9243	0.9655	0.0218
LOC126235	0.9459	1.0310	0.9757	0.9094	0.9655	0.0257
LOC360030	0.9190	1.0720	0.9617	0.9094	0.9655	0.0373
MIR199A2	0.8221	1.1048	1.0348	0.9004	0.9655	0.0639
LOC651536	0.9124	1.0779	1.0497	0.8221	0.9655	0.0599
SCARNA14	0.8584	1.0836	1.0194	0.9006	0.9655	0.0521
PCDHA7	0.9631	1.0464	0.9839	0.8687	0.9655	0.0368
LOC391241	1.0190	1.0542	0.9196	0.8693	0.9655	0.0429
IFFO2	0.9047	1.0779	1.0262	0.8532	0.9655	0.0521
LOC644135	1.0208	1.0855	0.9122	0.8436	0.9655	0.0541
ANP32E	0.8639	1.1041	0.9810	0.9131	0.9655	0.0521
KIR2DL5B	0.9168	1.0220	1.0108	0.9125	0.9655	0.0295
TECPR2	0.9387	1.0298	0.9735	0.9202	0.9655	0.0241
LOC1001337	0.8437	0.9802	1.0656	0.9727	0.9655	0.0458
LOC728348	0.8697	1.0799	1.0486	0.8639	0.9655	0.0574
PARP15	0.8719	1.0473	1.0259	0.9171	0.9655	0.0423
FBXO24	0.9320	1.0620	0.9288	0.9395	0.9655	0.0322
LOC1001323	0.9107	1.0792	0.9862	0.8861	0.9655	0.0435
LGR6	0.9507	1.0625	0.9425	0.9065	0.9656	0.0337
LOC1001280	0.9198	0.9914	1.0650	0.8860	0.9656	0.0398
LOC728054	0.9567	1.0403	0.9777	0.8875	0.9656	0.0315
LOC650296	0.9832	1.0832	0.9654	0.8304	0.9656	0.0520
LOC1001325	0.8948	0.9872	1.0420	0.9382	0.9656	0.0317
LOC646067	0.9303	1.1627	0.9146	0.8547	0.9656	0.0677
LOC647646	0.9691	1.0745	0.9677	0.8509	0.9656	0.0457
LOC401222	0.9146	1.0437	1.0127	0.8913	0.9656	0.0370
DNAJC28	0.9482	1.0646	1.0331	0.8164	0.9656	0.0555
PMS2CL	0.9062	1.1108	1.0425	0.8027	0.9656	0.0690
GPR31	0.9508	1.0428	0.9902	0.8785	0.9656	0.0346
LRMP	0.8682	1.0057	1.0365	0.9520	0.9656	0.0369
LOC646458	0.9536	1.0150	0.9891	0.9046	0.9656	0.0239
LOC1001320	0.9199	1.0672	0.9709	0.9043	0.9656	0.0367
C1orf150	0.9149	0.9435	1.0772	0.9268	0.9656	0.0377
IRAK3	0.9308	0.9832	1.0278	0.9207	0.9656	0.0248
LOC1001302	1.0193	0.9769	0.8623	1.0040	0.9656	0.0355
SNORD117	0.8828	0.9929	1.0755	0.9113	0.9656	0.0434
SORBS1	0.9055	1.1000	0.9608	0.8962	0.9656	0.0470
DUSP15	0.9110	1.0585	0.9973	0.8956	0.9656	0.0382
PRSS7	0.8234	1.0337	1.0273	0.9781	0.9656	0.0490
LOC400804	0.9569	1.0323	0.9499	0.9233	0.9656	0.0234
SERPINB7	0.9462	1.0289	1.0082	0.8792	0.9656	0.0337
LOC730841	0.8578	1.0526	1.0702	0.8820	0.9656	0.0556
TGS1	0.9634	1.0381	0.9560	0.9052	0.9656	0.0274

CACNG1	0.9855	1.0865	0.9598	0.8307	0.9656	0.0526
MIRLET7E	0.8466	1.0773	0.9973	0.9415	0.9656	0.0485
LOC653696	1.0068	1.0622	1.0431	0.7505	0.9657	0.0726
LOC650638	0.8440	1.0290	1.1384	0.8512	0.9657	0.0717
PLGLA	0.9557	1.0639	0.9870	0.8560	0.9657	0.0430
LOC641943	0.9001	1.1009	1.0067	0.8549	0.9657	0.0552
TULP2	0.9649	1.1006	0.8865	0.9107	0.9657	0.0479
LOC652463	0.8263	1.1614	1.0079	0.8670	0.9657	0.0760
KLHL6	0.8839	1.0941	0.9188	0.9658	0.9657	0.0460
GIPC3	0.9579	1.0707	0.9713	0.8627	0.9657	0.0426
LOC648354	0.9570	0.9691	0.9833	0.9533	0.9657	0.0068
MPP7	0.8604	1.1085	1.0439	0.8499	0.9657	0.0652
CACNB1	0.9546	1.0639	1.0064	0.8377	0.9657	0.0481
LOC402643	0.9658	1.0926	0.9964	0.8079	0.9657	0.0591
LOC1001326	0.9985	1.0407	0.9606	0.8629	0.9657	0.0380
C21orf49	0.8874	1.0808	0.9844	0.9101	0.9657	0.0436
PRKACA	0.9766	1.0894	0.9411	0.8556	0.9657	0.0484
RTN2	0.9360	1.0629	0.9298	0.9340	0.9657	0.0324
CHST15	0.9761	1.1532	0.9275	0.8060	0.9657	0.0720
LOC1001311	0.8853	1.0390	0.9515	0.9869	0.9657	0.0322
FN3K	1.0264	1.0918	0.8803	0.8643	0.9657	0.0556
MIR525	0.9119	1.0358	0.9690	0.9461	0.9657	0.0262
FAM115C	0.9673	1.0429	0.9514	0.9012	0.9657	0.0294
CLCF1	0.9658	1.0707	0.9808	0.8455	0.9657	0.0463
UBN1	0.9900	1.2925	0.8853	0.6950	0.9657	0.1249
LOC401252	0.9047	1.0327	1.0068	0.9186	0.9657	0.0318
AQP4	0.9510	1.0398	1.0270	0.8451	0.9657	0.0447
KRTAP20-1	1.0400	1.0338	0.9938	0.7954	0.9657	0.0577
TREX2	0.8791	1.1630	0.9993	0.8214	0.9657	0.0755
OR4C13	1.0036	0.9348	1.0789	0.8456	0.9657	0.0497
LOC642473	0.8624	1.0053	1.0889	0.9063	0.9657	0.0508
KCTD19	0.9565	0.9777	1.0004	0.9282	0.9657	0.0154
LOC643082	0.8316	1.2145	0.9374	0.8794	0.9657	0.0857
GRID1	0.8962	0.9144	1.0986	0.9538	0.9657	0.0459
CDA	0.9836	1.0287	0.9824	0.8683	0.9657	0.0342
GUCA1B	0.9565	1.0491	0.9301	0.9272	0.9657	0.0286
FLJ30375	0.9327	1.0769	0.9151	0.9382	0.9657	0.0374
CLEC3B	0.9331	1.0220	1.0689	0.8390	0.9658	0.0508
LENG8	0.9114	1.1106	0.9562	0.8849	0.9658	0.0505
LOC645320	0.9668	1.0348	0.9992	0.8623	0.9658	0.0372
ARSJ	0.8891	1.0887	0.9908	0.8945	0.9658	0.0472
ZNF509	0.9101	1.1132	0.9994	0.8404	0.9658	0.0589
LOC440248	0.9036	0.9937	1.1431	0.8227	0.9658	0.0686
PRSS42	0.9839	1.0291	0.9237	0.9265	0.9658	0.0253
LOC642111	0.9266	0.9873	1.0398	0.9094	0.9658	0.0298
LOC644623	0.9252	1.0737	0.9574	0.9069	0.9658	0.0375

LOC728743	0.9114	1.1087	1.0568	0.7861	0.9658	0.0730
OMD	0.9833	1.0447	0.9695	0.8656	0.9658	0.0372
LOC646132	0.9153	1.0268	1.0892	0.8318	0.9658	0.0574
LOC400958	0.9400	1.1170	0.8901	0.9162	0.9658	0.0514
PCDHB19P	0.9980	1.0271	0.8258	1.0123	0.9658	0.0470
ADAMTS20	0.9671	1.0349	0.9926	0.8686	0.9658	0.0353
SNORD116-2	1.0097	0.9760	1.0797	0.7977	0.9658	0.0600
DPY19L2P2	0.8741	1.0462	1.0703	0.8726	0.9658	0.0536
LOC134145	0.7756	1.1783	1.0393	0.8701	0.9658	0.0894
LOC653107	0.9048	1.1198	0.9644	0.8743	0.9658	0.0546
LOC401498	0.8957	1.1111	1.0458	0.8106	0.9658	0.0686
LOC1001332	0.8946	1.0861	0.9954	0.8872	0.9658	0.0471
SDR16C5	0.9973	1.0094	0.8832	0.9734	0.9658	0.0285
ZPBP2	0.9174	1.0575	1.0065	0.8819	0.9658	0.0403
LOC729786	0.9192	1.1188	1.0634	0.7619	0.9658	0.0799
BFSP2	0.9427	1.0530	0.9661	0.9015	0.9658	0.0320
ATG16L2	1.0425	1.0528	1.0299	0.7382	0.9658	0.0760
LOC1001322	0.9518	1.0867	0.9720	0.8528	0.9658	0.0480
IRF2	0.9615	1.0625	0.9901	0.8493	0.9658	0.0443
LOC1001339	0.9282	1.0662	0.9326	0.9363	0.9658	0.0335
SEC14L2	1.0586	1.0370	0.9303	0.8376	0.9659	0.0511
LOC642316	1.0486	1.0305	0.8341	0.9502	0.9659	0.0488
PDLIM4	1.0313	0.9435	1.0676	0.8210	0.9659	0.0549
LOC1001330	0.9019	1.0565	0.9670	0.9381	0.9659	0.0330
PRAMEF22	0.9575	1.0269	1.0273	0.8517	0.9659	0.0414
LOC642546	0.8874	0.9576	1.0616	0.9568	0.9659	0.0359
IKBKE	0.9698	1.1442	0.9834	0.7661	0.9659	0.0775
DGKZ	0.8993	1.0650	1.0004	0.8988	0.9659	0.0408
LOC1001328	1.0422	0.9980	0.9788	0.8446	0.9659	0.0426
TDRD9	0.9544	1.0627	0.9517	0.8948	0.9659	0.0351
RGN	0.9404	1.0179	1.0047	0.9007	0.9659	0.0276
LOC1001340	0.9200	1.0474	1.0654	0.8310	0.9659	0.0554
LOC642398	0.9039	1.0496	1.0404	0.8698	0.9659	0.0462
MIR376A1	0.8820	1.2013	0.9286	0.8518	0.9659	0.0800
LOC647243	0.8757	0.9510	1.0068	1.0302	0.9659	0.0344
LOC401397	0.8463	1.1923	1.0936	0.7316	0.9659	0.1068
COL5A1	0.8453	1.0620	1.0209	0.9355	0.9659	0.0481
LOC644598	0.8954	1.1002	1.0159	0.8522	0.9659	0.0566
OR1J2	0.9382	1.0531	1.0926	0.7799	0.9660	0.0701
AQP7P2	0.9344	1.0738	0.9922	0.8635	0.9660	0.0445
RBMY3AP	0.9502	1.0202	0.9974	0.8961	0.9660	0.0275
LOC650037	0.8985	1.0241	1.1087	0.8326	0.9660	0.0620
WDR45L	0.8442	1.3775	0.8509	0.7913	0.9660	0.1378
LOC729866	0.8808	1.0704	0.9938	0.9189	0.9660	0.0420
ZFP42	0.8970	0.9806	1.0112	0.9751	0.9660	0.0243
RN7SL1	0.8313	1.2968	1.0155	0.7203	0.9660	0.1260

ANKRD45	0.9968	1.0312	0.9858	0.8501	0.9660	0.0398
SCN11A	0.9018	1.0443	1.0688	0.8491	0.9660	0.0536
ZNF397	0.8228	1.2008	0.9494	0.8911	0.9660	0.0824
KDM1B	0.8823	1.0339	1.0796	0.8682	0.9660	0.0533
LOC1001310	0.8476	1.1037	1.0304	0.8824	0.9660	0.0606
OR2AK2	0.8720	0.9547	1.0256	1.0117	0.9660	0.0349
LOC730011	0.8714	1.0198	1.0430	0.9299	0.9660	0.0399
KIRREL	0.9013	0.9999	1.0922	0.8707	0.9660	0.0503
LOC653686	0.9537	1.0350	0.9718	0.9036	0.9660	0.0271
LOC641950	0.9328	1.1034	0.9884	0.8395	0.9660	0.0551
PIWIL3	0.9631	0.9927	1.0184	0.8899	0.9660	0.0278
LOC730045	0.8952	1.1007	0.9962	0.8720	0.9660	0.0524
FMNL3	0.9907	0.9882	0.9603	0.9250	0.9660	0.0153
AVPR2	0.9051	1.0899	1.0751	0.7941	0.9660	0.0710
LOC731414	0.8154	1.0994	1.0786	0.8707	0.9660	0.0720
NKX1-1	0.9037	1.0210	1.0049	0.9346	0.9660	0.0280
FLJ41562	0.9262	1.1308	0.9814	0.8258	0.9661	0.0637
MIR1471	0.9280	0.9991	1.0149	0.9222	0.9661	0.0239
LOC1001326	0.9261	1.0005	1.0191	0.9186	0.9661	0.0256
LOC285484	0.9021	1.0891	1.0449	0.8282	0.9661	0.0608
LOC1001331	0.9405	1.0558	0.9738	0.8942	0.9661	0.0341
ZNF846	1.0054	1.1139	0.9422	0.8028	0.9661	0.0650
CSF2RB	1.0406	0.9528	0.9651	0.9058	0.9661	0.0279
LOC347376	0.7389	1.0513	1.2484	0.8257	0.9661	0.1148
LOC652677	0.9381	1.0171	1.0388	0.8704	0.9661	0.0385
LOC651467	1.0376	0.9750	0.8945	0.9572	0.9661	0.0294
LOC653601	1.0298	1.0160	0.9199	0.8988	0.9661	0.0332
LOC90113	1.0139	0.9941	1.0353	0.8210	0.9661	0.0491
GPN1	0.8229	1.5561	0.9008	0.5846	0.9661	0.2079
LOC649103	0.8343	1.2472	0.9651	0.8178	0.9661	0.0993
LOC541471	0.7754	1.2088	1.0764	0.8037	0.9661	0.1056
LOC643983	1.0289	1.0131	0.9396	0.8828	0.9661	0.0339
LOC143188	0.9681	1.0367	1.0676	0.7920	0.9661	0.0617
KRTAP19-3	0.9158	1.1092	0.9305	0.9090	0.9661	0.0479
LOC653934	0.8281	1.1710	0.9498	0.9154	0.9661	0.0729
SFTPC	0.8766	1.0603	1.0186	0.9089	0.9661	0.0437
LOC645153	0.9140	1.0928	0.9580	0.8996	0.9661	0.0440
HOXB1	0.8533	1.0834	1.0511	0.8767	0.9661	0.0589
LOC642148	0.9371	0.9902	1.1018	0.8354	0.9661	0.0555
LOC644670	0.8915	1.1530	1.0134	0.8066	0.9661	0.0754
LRRTM1	0.9369	1.0755	0.9661	0.8860	0.9661	0.0400
LOC1001308	0.9239	1.0568	0.9915	0.8923	0.9661	0.0366
ABCA6	0.9910	1.0501	0.9442	0.8793	0.9661	0.0362
LOC730049	0.9958	0.9950	1.0195	0.8543	0.9662	0.0377
LOC647283	0.8854	1.0687	0.9708	0.9397	0.9662	0.0385
NAT2	0.8165	1.1129	1.0595	0.8756	0.9662	0.0712

CFLAR	0.7325	1.2068	1.0990	0.8264	0.9662	0.1117
BRDT	0.8702	1.1410	1.0763	0.7771	0.9662	0.0854
LOC90925	0.9652	1.0480	0.9293	0.9221	0.9662	0.0289
CXorf55	1.0362	1.0773	0.9443	0.8069	0.9662	0.0599
LOC642594	1.0266	1.0341	0.8973	0.9068	0.9662	0.0371
DAZL	0.9566	1.0248	0.9699	0.9134	0.9662	0.0229
DKFZP686C2	0.9505	0.9411	0.9945	0.9785	0.9662	0.0123
C18orf12	0.8777	1.0586	1.0061	0.9223	0.9662	0.0407
MT1B	0.9022	1.0161	1.0561	0.8904	0.9662	0.0412
MITF	0.8496	1.1056	1.0793	0.8302	0.9662	0.0732
FBXL6	0.8155	1.2668	1.0163	0.7663	0.9662	0.1138
LOC401164	0.9807	1.0775	0.9618	0.8448	0.9662	0.0477
LOC730273	0.8481	1.1986	0.9864	0.8319	0.9662	0.0849
TNNI3	0.9648	0.9836	1.0023	0.9141	0.9662	0.0190
CCL4L1	1.0656	0.9941	0.9612	0.8440	0.9662	0.0462
LOC1001293	1.0118	1.0800	0.9480	0.8251	0.9662	0.0542
LOC648815	0.9000	1.0331	1.0250	0.9069	0.9662	0.0363
LOC440261	0.9152	1.0132	1.0759	0.8607	0.9662	0.0483
COL3A1	0.9225	1.1442	1.0844	0.7139	0.9662	0.0963
TMLHE	0.9215	1.0814	1.0086	0.8535	0.9662	0.0498
RANGRF	0.9787	1.0609	0.8930	0.9323	0.9662	0.0361
HIST1H4G	0.8993	1.0329	1.0064	0.9264	0.9662	0.0318
LOC391073	0.9735	1.1073	0.9089	0.8752	0.9662	0.0513
CLIC2	0.8880	1.1117	0.9785	0.8869	0.9662	0.0530
DTYMK	1.0373	1.0354	0.9088	0.8834	0.9662	0.0408
TRH	0.9077	1.1130	0.9392	0.9051	0.9662	0.0495
LOC650599	0.8714	1.0455	0.9630	0.9851	0.9662	0.0361
CCR8	0.9231	0.9757	1.0407	0.9254	0.9662	0.0276
LOC1001280	0.8971	1.0973	1.0281	0.8425	0.9662	0.0585
C5orf62	0.9544	0.8946	1.0696	0.9464	0.9663	0.0369
TLL2	0.8953	1.0635	0.9994	0.9068	0.9663	0.0399
ARHGAP9	0.8719	1.1372	0.9308	0.9252	0.9663	0.0585
MAGEA2B	0.9135	1.1547	0.9009	0.8960	0.9663	0.0629
GSDMB	0.9043	1.1012	0.9707	0.8889	0.9663	0.0483
LOC642975	0.8086	1.2011	1.1907	0.6648	0.9663	0.1358
TRPM2	0.8672	1.0531	1.0807	0.8641	0.9663	0.0584
LOC727839	0.8322	1.0826	1.0451	0.9053	0.9663	0.0588
LOC652848	0.9855	1.0178	0.9698	0.8922	0.9663	0.0266
C5orf38	0.8710	1.0323	1.0561	0.9058	0.9663	0.0458
CDH12	0.8628	0.9507	1.1189	0.9329	0.9663	0.0543
ARMC9	0.8353	1.1220	0.9896	0.9183	0.9663	0.0607
CCDC79	0.9319	0.9849	1.0154	0.9329	0.9663	0.0205
LOC390595	0.9120	1.0480	1.0178	0.8874	0.9663	0.0393
LOC650020	0.9901	1.1520	0.9797	0.7435	0.9663	0.0841
LOC653581	0.8369	1.1980	0.9868	0.8435	0.9663	0.0846
HYAL1	0.9202	1.0205	1.0387	0.8858	0.9663	0.0374

ZNF844	0.9423	1.0527	1.0684	0.8018	0.9663	0.0616
KIF25	0.9392	0.9984	1.0405	0.8871	0.9663	0.0336
SHANK1	0.7394	1.0421	1.1365	0.9473	0.9663	0.0849
LOC642821	0.8996	1.0383	0.9989	0.9285	0.9663	0.0318
CTRC	0.9511	0.9828	1.0438	0.8877	0.9663	0.0325
BCORL2	0.8780	0.9806	1.0873	0.9195	0.9663	0.0455
MIR298	0.9605	1.0735	0.8726	0.9587	0.9663	0.0412
ARGFXP2	0.9308	1.0349	0.9833	0.9164	0.9663	0.0270
ANXA2P2	0.8095	1.3672	0.9481	0.7406	0.9663	0.1404
WHSC2	0.7667	1.3077	0.9726	0.8183	0.9663	0.1219
DNAJC1	0.7353	1.3389	1.0532	0.7379	0.9663	0.1449
SCARA3	0.9310	1.0894	0.9806	0.8644	0.9663	0.0474
ZNF630	0.8971	1.1153	0.9532	0.8999	0.9664	0.0513
LIPK	0.9635	1.0298	0.9624	0.9097	0.9664	0.0246
LOC642104	0.9622	1.0241	1.0347	0.8444	0.9664	0.0437
C15orf21	0.8893	1.1374	0.9804	0.8583	0.9664	0.0626
KRT3	0.9044	1.1096	0.9996	0.8517	0.9664	0.0567
ZNF808	1.0028	0.9992	1.0177	0.8457	0.9664	0.0404
LOC728716	0.9512	1.0577	0.9353	0.9212	0.9664	0.0311
LOC642119	0.9661	1.0041	0.9563	0.9390	0.9664	0.0138
LOC654128	0.9463	0.9708	1.0193	0.9291	0.9664	0.0196
NGF	0.9261	1.0805	0.9324	0.9265	0.9664	0.0381
IL8	0.9193	1.0864	1.0153	0.8445	0.9664	0.0531
TNFRSF9	0.9624	1.0892	0.9852	0.8287	0.9664	0.0535
LOC653507	0.8651	1.1275	0.9864	0.8865	0.9664	0.0599
PIGL	0.7894	1.1823	0.9252	0.9686	0.9664	0.0815
SNORD116-2	0.8989	1.1200	0.9203	0.9263	0.9664	0.0515
LOC644019	0.8586	1.0997	0.9894	0.9179	0.9664	0.0519
OR2D3	0.7755	1.0668	1.1316	0.8917	0.9664	0.0813
C6orf225	0.9752	1.0440	0.9777	0.8686	0.9664	0.0363
TRIM8	0.7952	1.2831	1.0766	0.7107	0.9664	0.1314
EXTL1	0.8719	1.0679	1.0075	0.9184	0.9664	0.0440
LOC649771	0.8810	0.9765	1.0245	0.9836	0.9664	0.0304
LOC648982	0.9961	1.0128	0.9756	0.8811	0.9664	0.0294
CXorf21	0.9036	1.0903	0.9720	0.8997	0.9664	0.0445
LOC642718	0.9513	1.1251	0.9029	0.8864	0.9664	0.0547
MIR383	0.9483	1.0288	1.0057	0.8829	0.9664	0.0326
LOC642588	0.9201	1.0494	1.0351	0.8612	0.9664	0.0455
DEFB119	0.9244	1.1142	0.9315	0.8958	0.9665	0.0498
C21orf90	0.8409	1.1127	0.9244	0.9877	0.9665	0.0573
LOC729179	0.8041	1.1659	1.0347	0.8611	0.9665	0.0826
LGALS7B	0.9805	1.0740	0.9073	0.9039	0.9665	0.0400
LOC1001283	0.9521	1.0870	1.0083	0.8184	0.9665	0.0566
IL1F7	0.9353	0.9633	0.9801	0.9872	0.9665	0.0115
MIR27A	1.0518	0.9655	1.0133	0.8353	0.9665	0.0472
OR4B1	0.9147	1.0500	0.9924	0.9088	0.9665	0.0337

ZPBP	0.9019	1.0280	1.0112	0.9248	0.9665	0.0312
LOC644362	0.9950	0.9917	1.0063	0.8728	0.9665	0.0314
GOLGA8E	0.9038	1.0635	0.9835	0.9151	0.9665	0.0368
LOC652808	0.8588	1.1048	0.9924	0.9099	0.9665	0.0537
TCF23	0.8730	1.1365	1.0358	0.8207	0.9665	0.0729
LOC1001331	0.9181	0.9246	1.1042	0.9191	0.9665	0.0459
ZNF92	0.9183	1.0933	0.9720	0.8823	0.9665	0.0461
SIAH2	0.9003	1.3064	0.9217	0.7376	0.9665	0.1205
BBS5	0.9290	1.0148	0.9082	1.0139	0.9665	0.0280
OR52E4	0.9636	0.9442	1.0299	0.9283	0.9665	0.0223
LOC1001283	0.9499	1.0620	1.0002	0.8539	0.9665	0.0440
CRNN	0.8999	1.1316	0.9829	0.8517	0.9665	0.0613
AKAP2	0.8863	1.0916	1.0000	0.8882	0.9665	0.0494
BAHD1	0.8634	1.0613	1.0148	0.9266	0.9665	0.0443
RYR2	0.9352	1.0631	0.9904	0.8773	0.9665	0.0396
LOC1001298	0.8056	1.1326	1.1426	0.7854	0.9665	0.0989
LOC645177	0.8873	1.1175	0.9914	0.8700	0.9665	0.0570
OR12D2	1.0083	1.0136	0.9997	0.8446	0.9665	0.0408
CXorf65	0.8742	1.1184	0.9882	0.8853	0.9665	0.0568
LCORL	0.9220	1.0759	0.9939	0.8743	0.9665	0.0440
PLA2G10	0.9002	1.1192	0.9431	0.9037	0.9665	0.0518
LOC731431	0.8949	1.0681	1.0410	0.8622	0.9666	0.0515
QARS	0.8351	1.3483	0.9038	0.7790	0.9666	0.1298
DKFZP434A0	0.9968	0.9853	1.0369	0.8472	0.9666	0.0413
LOC146909	0.9237	1.1289	1.0253	0.7884	0.9666	0.0727
LOC646680	0.9557	0.9299	1.0336	0.9471	0.9666	0.0230
FAM186B	0.9049	1.0321	1.0577	0.8716	0.9666	0.0460
SPZ1	0.8558	1.0938	1.0156	0.9011	0.9666	0.0541
STON2	0.8800	1.0832	0.9679	0.9353	0.9666	0.0429
TICAM2	0.8753	1.1868	0.9181	0.8862	0.9666	0.0740
SPDYA	0.9609	1.0991	0.9461	0.8602	0.9666	0.0494
NEK10	0.9432	1.0734	0.9717	0.8780	0.9666	0.0407
DBF4	0.9208	1.0752	1.0697	0.8007	0.9666	0.0659
CIDEC	0.8563	1.1264	0.9734	0.9102	0.9666	0.0584
AWAT2	0.9462	0.9311	0.9915	0.9975	0.9666	0.0165
LOC649167	0.9377	1.1087	0.9658	0.8542	0.9666	0.0530
HEYL	1.0338	0.9506	0.9433	0.9388	0.9666	0.0225
PDE1B	0.9200	1.0607	0.9953	0.8905	0.9666	0.0383
BSND	0.9595	1.0394	0.8994	0.9682	0.9666	0.0287
LOC1001340	0.9931	1.0300	1.0405	0.8029	0.9666	0.0555
OR11G2	0.9368	1.0057	1.0663	0.8578	0.9666	0.0449
C3orf41	0.9349	1.0121	0.9757	0.9439	0.9666	0.0175
LOC646026	0.8833	1.0218	1.0213	0.9402	0.9666	0.0338
LOC1001337	0.8738	0.9928	1.1047	0.8953	0.9666	0.0528
ZZEF1	0.8433	1.2186	0.9757	0.8290	0.9666	0.0902
LOC647285	0.7298	1.2706	1.1981	0.6681	0.9667	0.1558

LOC646748	1.0242	1.0338	0.9693	0.8394	0.9667	0.0447
LOC727901	0.9087	1.0741	0.9076	0.9762	0.9667	0.0392
LOC652906	1.0118	0.9776	0.9429	0.9343	0.9667	0.0177
MIR521-2	0.8320	1.0608	0.9839	0.9899	0.9667	0.0482
SLC4A5	0.9245	1.0484	1.0178	0.8760	0.9667	0.0401
LPIN3	0.8211	1.2273	0.9599	0.8583	0.9667	0.0917
LOC653935	0.9352	1.0117	1.0544	0.8654	0.9667	0.0418
DNASE1L2	0.9494	1.0624	1.0045	0.8504	0.9667	0.0451
CPNE2	0.9398	0.9267	1.1480	0.8522	0.9667	0.0635
LOC729666	0.9050	1.0513	0.9932	0.9172	0.9667	0.0343
COL4A3	0.9553	1.0187	0.9661	0.9266	0.9667	0.0193
LOC653653	0.8797	1.0486	0.9485	0.9899	0.9667	0.0355
LOC343515	0.9380	1.0674	0.9863	0.8750	0.9667	0.0406
LOC644266	0.8726	1.0586	1.0630	0.8725	0.9667	0.0544
LOC1001303	0.8955	1.1661	0.9049	0.9002	0.9667	0.0665
LOC286187	0.9853	0.9625	0.9936	0.9253	0.9667	0.0153
LOC1001306	0.9437	1.0385	0.9587	0.9259	0.9667	0.0249
LOC653337	0.9435	1.0332	1.0321	0.8578	0.9667	0.0419
LOC1001289	0.9047	1.0268	1.0660	0.8693	0.9667	0.0473
LOC652777	0.9794	0.9955	1.0478	0.8441	0.9667	0.0434
RETN	1.0333	1.0545	0.9647	0.8143	0.9667	0.0543
LOC643883	0.9275	1.0814	1.0408	0.8170	0.9667	0.0596
LOC391763	0.8710	1.0917	0.9383	0.9658	0.9667	0.0462
CYGB	0.9206	1.1126	0.9183	0.9153	0.9667	0.0487
TRIM40	0.8341	1.0839	1.0791	0.8697	0.9667	0.0667
LOC653352	0.9276	1.0148	1.0070	0.9174	0.9667	0.0256
LOC1001293	0.7653	1.2745	0.8907	0.9364	0.9667	0.1088
LOC1001281	0.8632	1.1187	1.0861	0.7990	0.9667	0.0797
LOC652003	0.9231	1.1082	1.0572	0.7785	0.9667	0.0739
KRTAP4-5	0.8597	1.0678	1.0469	0.8926	0.9667	0.0529
CALHM1	0.9294	1.0084	1.0661	0.8631	0.9667	0.0445
LOC1001309	0.8772	1.1233	0.9644	0.9020	0.9667	0.0553
WFDC11	1.0441	0.9829	1.0563	0.7837	0.9667	0.0631
MFHAS1	0.8234	1.2333	0.9346	0.8757	0.9667	0.0917
LOC1001308	0.9266	1.0110	1.0410	0.8883	0.9667	0.0356
MIR98	1.0225	0.9449	1.0205	0.8791	0.9667	0.0343
CA5A	0.8810	1.0867	1.0014	0.8979	0.9667	0.0480
C14orf180	0.9330	1.0551	0.9819	0.8970	0.9668	0.0342
LOC375190	0.9333	1.0513	1.0401	0.8423	0.9668	0.0493
LOC731631	0.9361	1.0539	0.9658	0.9112	0.9668	0.0311
ASB11	0.9872	1.0172	1.0005	0.8622	0.9668	0.0354
FAM161A	0.7709	1.1300	0.9781	0.9881	0.9668	0.0739
MIR23B	0.7988	1.0339	1.1413	0.8930	0.9668	0.0756
CRYGA	0.8796	1.1302	0.9890	0.8683	0.9668	0.0609
LOC388327	0.9302	1.1171	0.9538	0.8661	0.9668	0.0534
NACC1	0.9030	1.1884	0.9811	0.7946	0.9668	0.0832

LOC642968	0.9222	1.0326	0.9942	0.9181	0.9668	0.0281
LOC728403	0.8736	1.1162	0.9263	0.9511	0.9668	0.0524
NTN3	0.9019	1.0646	1.0123	0.8884	0.9668	0.0428
MIR192	0.8736	1.0382	1.0060	0.9495	0.9668	0.0361
MEGF6	1.0361	1.0786	0.9846	0.7679	0.9668	0.0690
LOC392364	0.9128	0.9565	1.0587	0.9392	0.9668	0.0319
LOC653766	0.9339	1.0740	0.9921	0.8673	0.9668	0.0439
LOC339862	0.9386	1.0727	0.8930	0.9629	0.9668	0.0382
FLJ30092	0.9272	1.0534	0.9857	0.9010	0.9668	0.0338
INHBE	0.9169	1.1632	0.9242	0.8631	0.9668	0.0669
LRP2BP	0.9566	1.0620	0.9905	0.8582	0.9668	0.0423
EPHB3	0.9588	1.1551	0.8863	0.8671	0.9668	0.0658
INSL3	0.9305	1.0434	0.9808	0.9126	0.9668	0.0293
C10orf37	0.9081	1.0160	1.0326	0.9107	0.9668	0.0333
ACP5	0.9401	0.9434	1.0617	0.9222	0.9668	0.0320
CLEC10A	0.9563	1.0548	0.9663	0.8899	0.9668	0.0339
GAS7	0.9411	1.0309	0.9870	0.9084	0.9669	0.0267
FUNDC2	0.9494	1.0555	1.0118	0.8508	0.9669	0.0444
H2AFB2	0.9535	1.1146	0.9691	0.8303	0.9669	0.0582
C10orf75	0.9243	1.0395	1.0055	0.8982	0.9669	0.0333
MYPOP	0.9107	1.1261	1.0482	0.7826	0.9669	0.0759
OR8G2	0.9175	1.1400	0.8958	0.9141	0.9669	0.0579
C6orf150	0.9389	1.0906	0.9663	0.8717	0.9669	0.0458
LOC652438	0.8973	1.0580	1.0218	0.8904	0.9669	0.0428
LRRC37A4	0.9638	1.1045	0.9412	0.8580	0.9669	0.0512
LOC642047	0.9121	1.0066	1.0449	0.9039	0.9669	0.0349
LOC653238	0.9458	1.0578	0.9647	0.8992	0.9669	0.0333
SNORD92	0.9754	1.0674	0.9257	0.8991	0.9669	0.0370
HOMER3	0.7861	1.2273	0.9793	0.8749	0.9669	0.0954
LOC649044	0.9130	1.1235	0.9340	0.8972	0.9669	0.0527
BDNF	0.9072	1.0952	0.9756	0.8897	0.9669	0.0466
DNAJC6	0.9511	0.9540	1.0239	0.9386	0.9669	0.0193
RAB13	0.7372	1.2419	1.0483	0.8403	0.9669	0.1122
LOC727882	0.9950	0.9885	0.9426	0.9417	0.9669	0.0144
LOC654163	0.8659	1.0911	1.0876	0.8232	0.9669	0.0712
PRDM5	0.8791	1.0800	1.0077	0.9010	0.9669	0.0470
LOC1001290	0.9235	1.0591	1.1193	0.7659	0.9669	0.0785
OSR1	0.9851	1.0869	1.0367	0.7591	0.9669	0.0723
PDZRN3	0.9343	1.0637	1.0234	0.8464	0.9670	0.0484
SPRR2E	1.0092	1.0279	0.9549	0.8758	0.9670	0.0341
RPRC1	0.9044	1.4203	0.8833	0.6598	0.9670	0.1609
LOC441728	0.9127	1.0746	0.9682	0.9124	0.9670	0.0382
LOC1001321	0.9689	1.0808	0.9270	0.8912	0.9670	0.0411
SP100	0.9179	1.0226	1.0294	0.8980	0.9670	0.0344
KLC2	0.8788	1.1966	0.9673	0.8253	0.9670	0.0820
LOC646257	0.9763	1.0399	0.9299	0.9219	0.9670	0.0271

LOC388553	0.8356	1.1197	1.0182	0.8945	0.9670	0.0636
SULT4A1	0.9286	1.0564	1.0431	0.8400	0.9670	0.0511
LOC645212	0.8572	1.2882	0.9703	0.7523	0.9670	0.1159
LOC150759	0.8394	1.0405	1.0594	0.9287	0.9670	0.0514
LOC650268	0.8458	1.2583	0.9456	0.8184	0.9670	0.1009
ATP6V0B	0.8526	1.2416	1.1079	0.6659	0.9670	0.1288
LOC1001313	0.9381	0.9742	1.0360	0.9198	0.9670	0.0256
SNORA13	0.8545	1.1125	1.0341	0.8670	0.9670	0.0635
LOC1001303	0.9590	1.1295	0.9830	0.7965	0.9670	0.0682
SULF2	0.9205	1.0559	0.9961	0.8955	0.9670	0.0366
LOC651861	0.9141	1.0670	1.0248	0.8622	0.9670	0.0475
LOC387924	0.9717	1.0898	0.9757	0.8309	0.9670	0.0530
NCRNA0017	0.9172	1.1046	1.0112	0.8351	0.9670	0.0583
TMCO5A	0.9896	1.0584	0.9745	0.8456	0.9670	0.0444
SCGB1D1	0.9652	1.0262	0.9755	0.9012	0.9670	0.0257
LOC1001317	0.9116	1.1293	0.9871	0.8402	0.9670	0.0619
ZEB1	0.9608	1.0577	0.9347	0.9150	0.9671	0.0316
COG7	0.9114	1.1758	1.0064	0.7746	0.9671	0.0843
LOC1001288	0.7942	1.4123	0.9235	0.7383	0.9671	0.1534
LOC643962	0.9552	1.0598	0.9895	0.8638	0.9671	0.0407
IL18RAP	0.8503	1.0595	1.0449	0.9137	0.9671	0.0509
SNORD114-1	0.8852	1.0926	1.0432	0.8473	0.9671	0.0596
C9orf72	0.9259	1.1521	1.0021	0.7882	0.9671	0.0759
GNAO1	0.8961	1.0122	1.0703	0.8897	0.9671	0.0445
C6orf79	0.9940	1.0105	0.9027	0.9611	0.9671	0.0238
LOC442251	0.9974	0.9435	1.0253	0.9021	0.9671	0.0275
LOC730456	0.9202	1.1062	1.0180	0.8240	0.9671	0.0610
LOC644280	0.9375	1.0772	1.0107	0.8429	0.9671	0.0503
COG5	0.8983	1.1503	1.0169	0.8029	0.9671	0.0751
WBP5	0.8007	1.4130	0.8855	0.7693	0.9671	0.1506
RAB38	0.9325	1.1430	0.9622	0.8308	0.9671	0.0650
KRTAP10-12	0.7344	1.2177	1.0689	0.8474	0.9671	0.1087
LOC347475	0.8845	1.0767	0.9530	0.9543	0.9671	0.0400
OR52K2	0.8599	1.0635	1.0174	0.9277	0.9671	0.0455
SNORD78	0.8818	1.0859	0.9874	0.9134	0.9671	0.0454
GALNT11	0.8699	1.3463	0.8996	0.7527	0.9671	0.1303
CAPN8	0.8852	1.0324	1.0131	0.9378	0.9671	0.0341
TMEM150B	0.8691	1.1061	1.0017	0.8916	0.9671	0.0546
DGCR6	0.7867	1.3359	1.0228	0.7230	0.9671	0.1388
SYTL4	0.9460	1.1302	1.0076	0.7847	0.9671	0.0718
LOC285500	0.8802	1.0232	1.0676	0.8976	0.9671	0.0462
ITM2A	0.8749	1.0837	1.0013	0.9086	0.9671	0.0471
ADAMTS16	0.8976	1.0472	0.9813	0.9425	0.9671	0.0317
LOC1001303	0.8809	1.0483	1.0333	0.9061	0.9671	0.0430
TRERF1	0.8723	1.1504	0.9778	0.8681	0.9671	0.0661
C3orf62	0.8792	1.1564	1.0346	0.7984	0.9671	0.0799

LOC440687	1.0036	0.9979	0.9675	0.8996	0.9671	0.0239
PSMD7	0.7720	1.3139	1.0057	0.7770	0.9671	0.1278
LOC1001304	0.8967	1.0512	1.0075	0.9132	0.9671	0.0372
LOC1001331	0.7764	1.1715	1.0591	0.8616	0.9672	0.0903
GPR50	0.9225	0.9714	1.0107	0.9640	0.9672	0.0181
LOC647585	0.9524	1.0695	0.9089	0.9378	0.9672	0.0353
KIF1B	0.8693	1.3204	0.9214	0.7576	0.9672	0.1226
KRT222	0.8698	1.0643	1.0984	0.8362	0.9672	0.0666
KCNE3	0.9281	1.1454	1.0014	0.7939	0.9672	0.0733
SYNJ2	0.8774	1.0010	0.9806	1.0097	0.9672	0.0305
BRCA2	0.8848	1.0916	1.0751	0.8173	0.9672	0.0685
LOC728760	0.8855	1.0041	1.0231	0.9560	0.9672	0.0307
TMEM151	0.8636	1.2435	0.9712	0.7904	0.9672	0.0993
NGLY1	0.9095	1.1727	1.1172	0.6694	0.9672	0.1143
C12orf26	0.9092	1.1100	1.0226	0.8271	0.9672	0.0622
LOC1001343	0.8416	1.0641	1.0163	0.9469	0.9672	0.0483
MIR302A	0.9323	1.1631	0.8682	0.9053	0.9672	0.0666
ZNF37A	0.8566	1.1753	0.9670	0.8700	0.9672	0.0736
LOC283398	0.8944	1.0108	1.0508	0.9128	0.9672	0.0378
KCNK10	0.9267	1.0707	1.0118	0.8597	0.9672	0.0465
LOC1001287	0.8345	1.0757	1.0310	0.9278	0.9672	0.0540
SIM2	0.8916	1.1879	0.9378	0.8517	0.9672	0.0756
LOC729269	0.8806	1.1150	0.8952	0.9781	0.9672	0.0537
TRAF3	0.8619	1.0657	1.0159	0.9254	0.9672	0.0456
LOC644038	1.0189	1.0152	0.9694	0.8655	0.9672	0.0357
POMT2	0.9369	1.2684	0.8189	0.8448	0.9672	0.1035
DUSP5P	0.9548	1.1482	0.8986	0.8674	0.9672	0.0630
LOC1001280	0.7980	1.1083	1.2135	0.7493	0.9672	0.1143
LOC645971	0.9539	1.1003	0.9528	0.8620	0.9673	0.0493
LOC730978	0.9334	1.0901	0.9848	0.8608	0.9673	0.0482
PRG2	0.9126	0.9984	0.9370	1.0211	0.9673	0.0255
FUK	1.0075	1.2625	0.8342	0.7648	0.9673	0.1109
CDH22	0.8922	1.1528	0.9694	0.8548	0.9673	0.0663
LOC647747	1.0231	1.1441	1.0194	0.6826	0.9673	0.0992
VCAN	1.0033	0.9743	0.9563	0.9354	0.9673	0.0144
LOC653853	0.9918	0.9978	0.9965	0.8831	0.9673	0.0281
ZNF610	1.0357	0.9487	0.9962	0.8886	0.9673	0.0317
GYPC	0.9001	1.0686	0.9808	0.9198	0.9673	0.0379
LOC645520	0.8612	1.0421	0.9759	0.9900	0.9673	0.0381
PMCH	0.7655	1.1366	1.0767	0.8905	0.9673	0.0853
LOC1001317	1.0068	1.0924	0.9955	0.7746	0.9673	0.0678
KIAA1033	0.9400	1.0695	1.0398	0.8200	0.9673	0.0564
LOC441812	0.9814	1.0432	0.8614	0.9833	0.9673	0.0381
LOC1001315	0.9241	1.0034	1.0137	0.9281	0.9673	0.0239
ZCCHC16	0.8804	1.0431	0.9950	0.9509	0.9673	0.0346
CCDC72	0.9359	1.3086	0.9017	0.7232	0.9673	0.1229

CDRT4	0.9188	1.1817	0.9243	0.8445	0.9673	0.0737
KCNF1	0.9021	1.0385	1.1056	0.8233	0.9674	0.0640
LOC641972	1.1167	1.1011	0.9266	0.7252	0.9674	0.0915
HAR1A	0.9448	1.0991	0.8538	0.9719	0.9674	0.0506
LOC149086	0.9458	1.0851	0.9062	0.9324	0.9674	0.0401
ZNF793	0.9422	1.0805	1.1020	0.7448	0.9674	0.0822
LOC647535	0.8806	1.0077	1.0069	0.9743	0.9674	0.0299
LOC199882	0.9620	1.0639	1.0092	0.8343	0.9674	0.0490
LOC647971	0.9414	1.0203	1.1334	0.7744	0.9674	0.0754
BRIP1	0.9287	1.0327	0.9309	0.9772	0.9674	0.0245
LOC730015	0.8519	1.0839	1.1045	0.8292	0.9674	0.0735
LOC651741	0.8069	1.1052	1.0510	0.9065	0.9674	0.0680
LOC652226	0.8612	1.0276	1.0848	0.8959	0.9674	0.0531
LOC730130	0.9217	0.9665	1.0306	0.9509	0.9674	0.0230
LOC646717	0.9383	1.0609	0.8876	0.9828	0.9674	0.0368
FLJ43093	0.8994	1.0781	1.0593	0.8328	0.9674	0.0602
ERCC-00074	0.9459	1.0902	0.9599	0.8737	0.9674	0.0451
LOC653380	0.8987	1.0362	1.0324	0.9023	0.9674	0.0386
LOC653695	0.8930	1.2168	0.9235	0.8362	0.9674	0.0851
LOC643680	0.9462	0.9953	0.9308	0.9974	0.9674	0.0170
MEX3C	0.7699	1.2564	1.1098	0.7336	0.9674	0.1283
JMJD1A	1.0770	1.0136	1.0365	0.7424	0.9674	0.0761
LOC728111	0.9351	1.0167	1.0808	0.8370	0.9674	0.0527
TBX18	0.9484	1.0656	0.9847	0.8710	0.9674	0.0404
FLJ00312	0.8872	1.0824	1.0079	0.8922	0.9674	0.0474
LOC1001301	0.7634	1.2255	1.0380	0.8427	0.9674	0.1036
NDUFA7	0.7844	1.3570	0.9441	0.7842	0.9674	0.1352
MGC70870	0.9696	1.0697	0.9606	0.8697	0.9674	0.0409
LOC728707	0.8747	1.1262	0.9706	0.8982	0.9674	0.0567
LOC645557	0.8587	1.0867	0.9963	0.9280	0.9674	0.0487
POLR2J3	0.7844	1.1457	1.2174	0.7222	0.9674	0.1251
LOC647520	0.8964	1.1722	0.9231	0.8780	0.9674	0.0689
LOC729897	0.8606	0.9880	1.1092	0.9119	0.9674	0.0540
FAM22G	0.9503	1.0268	1.0028	0.8900	0.9674	0.0304
LOC653436	0.9323	1.1832	0.9502	0.8041	0.9674	0.0789
RASAL2	0.8967	1.0813	0.9944	0.8973	0.9674	0.0444
SPINK9	0.9476	1.0631	1.0038	0.8553	0.9675	0.0442
PRLH	0.9974	1.0323	0.9519	0.8883	0.9675	0.0311
LOC652608	0.8516	1.0861	1.1584	0.7738	0.9675	0.0919
GFRAL	0.8935	1.1006	0.9915	0.8842	0.9675	0.0506
CCDC70	0.8589	0.9508	1.0868	0.9734	0.9675	0.0468
HPR	0.9387	1.1896	0.9676	0.7740	0.9675	0.0854
LOC652696	0.8456	1.0494	1.0203	0.9546	0.9675	0.0452
WDR63	0.9430	1.0809	0.9664	0.8796	0.9675	0.0420
C10orf91	0.9343	1.0304	0.9740	0.9313	0.9675	0.0231
LOC729444	0.8978	1.0189	1.0468	0.9065	0.9675	0.0382

RNF17	0.9364	1.0426	1.0104	0.8805	0.9675	0.0365
MIR1200	0.9185	1.0772	0.9134	0.9609	0.9675	0.0381
VWCE	0.8519	1.0809	0.9594	0.9778	0.9675	0.0469
LOC644201	0.9902	1.0920	0.9388	0.8490	0.9675	0.0507
FAM168A	1.0298	0.9610	0.9995	0.8797	0.9675	0.0325
LOC643482	0.8988	1.1423	0.9521	0.8768	0.9675	0.0604
LOC1001321	0.8139	1.2010	0.9762	0.8788	0.9675	0.0847
LOC1001328	1.0639	0.9643	0.9091	0.9328	0.9675	0.0340
LOC1001302	0.9645	1.0088	0.9974	0.8993	0.9675	0.0246
LOC731228	0.9289	1.0676	0.9985	0.8751	0.9675	0.0418
ERCC-00131	0.8574	1.0152	1.0636	0.9338	0.9675	0.0454
LOC729096	0.8644	1.0374	0.9322	1.0361	0.9675	0.0423
LAT	0.9038	1.0846	0.9771	0.9046	0.9675	0.0426
TXNDC3	0.9306	0.9150	1.1272	0.8973	0.9675	0.0537
NAV2	0.8627	1.0927	1.0524	0.8624	0.9675	0.0612
LOC1001346	0.8923	1.0431	1.0612	0.8735	0.9675	0.0491
TF	0.8719	1.1562	0.9369	0.9052	0.9675	0.0643
OR13A1	0.9033	0.9553	1.0517	0.9598	0.9675	0.0308
CXorf27	0.9158	1.0260	1.0070	0.9214	0.9675	0.0285
OR1N1	1.0315	0.9583	0.9958	0.8846	0.9675	0.0314
HYPB	0.8897	0.9973	1.0206	0.9626	0.9676	0.0286
BREA2	0.9129	1.0655	1.0250	0.8668	0.9676	0.0466
SLC39A5	0.9001	1.0315	1.0716	0.8671	0.9676	0.0496
LOC728904	0.8656	1.0563	0.9197	1.0286	0.9676	0.0450
LOC1001339	0.7900	1.2066	1.0193	0.8544	0.9676	0.0932
CYP2W1	0.8752	1.0308	1.0404	0.9239	0.9676	0.0406
LOC645930	0.9858	1.0289	1.0210	0.8346	0.9676	0.0453
LOC652341	1.0246	0.9735	0.9567	0.9155	0.9676	0.0226
DCDC5	0.8794	1.0712	1.0239	0.8959	0.9676	0.0473
SPIC	0.9897	1.0538	0.9491	0.8778	0.9676	0.0369
LOC652344	0.9766	1.0512	0.9323	0.9103	0.9676	0.0311
LOC651552	0.9962	1.0574	0.9780	0.8388	0.9676	0.0462
LOC728361	0.9030	1.0367	1.0136	0.9172	0.9676	0.0337
SLMAP	0.8787	1.2963	0.9594	0.7360	0.9676	0.1189
LOC152845	0.8920	1.0383	1.0268	0.9134	0.9676	0.0378
ZFP1	0.8957	1.1189	1.0214	0.8344	0.9676	0.0637
LGI3	0.9739	1.0296	0.9135	0.9535	0.9676	0.0242
LOC1001281	0.9863	0.9344	1.1164	0.8335	0.9676	0.0589
NLF2	0.9901	1.0123	0.9830	0.8852	0.9676	0.0282
LOC642950	0.9788	1.0850	0.9324	0.8743	0.9676	0.0446
CACNG7	0.9294	1.0965	0.9774	0.8673	0.9676	0.0485
FLJ16734	0.9285	1.1180	0.9962	0.8279	0.9676	0.0609
PIK3R5	0.8560	1.1694	0.9969	0.8482	0.9676	0.0754
LOC642655	1.1763	0.8756	0.9472	0.8714	0.9677	0.0717
LOC653303	1.0042	0.9764	1.0463	0.8438	0.9677	0.0437
THSD7A	0.8705	1.0307	1.0744	0.8950	0.9677	0.0501

LOC220729	0.8967	1.0291	1.0735	0.8714	0.9677	0.0494
EPC2	0.9812	1.0913	0.9091	0.8891	0.9677	0.0457
MYO5B	0.9986	1.0044	1.0566	0.8110	0.9677	0.0538
TRAPPC6B	0.9284	1.1037	1.1078	0.7307	0.9677	0.0894
LOC641743	0.8760	1.0055	1.0954	0.8938	0.9677	0.0513
LOC643040	0.9407	1.0314	1.0235	0.8750	0.9677	0.0371
MLXIPL	0.8671	1.2038	0.9427	0.8570	0.9677	0.0810
CABP5	0.9812	1.0133	1.0821	0.7941	0.9677	0.0616
MS4A6A	0.8988	1.0593	1.0086	0.9040	0.9677	0.0397
LOC1001318	0.9192	1.0715	1.0305	0.8495	0.9677	0.0509
CROCCL1	0.9618	1.0204	0.9926	0.8959	0.9677	0.0268
FCRL2	0.9187	1.0987	0.9740	0.8792	0.9677	0.0478
LOC389768	0.8695	1.0804	1.0480	0.8729	0.9677	0.0561
MIR149	0.8568	1.0475	1.0436	0.9227	0.9677	0.0469
LOC728285	0.9361	1.0363	0.9939	0.9044	0.9677	0.0294
ARL15	0.7899	1.2937	1.0208	0.7664	0.9677	0.1229
PDZK1IP1	1.0612	0.9644	1.0279	0.8172	0.9677	0.0540
OTX1	0.8585	1.0843	1.0253	0.9026	0.9677	0.0525
LOC642245	1.0891	0.9500	1.0013	0.8304	0.9677	0.0540
CHST9	0.9772	0.9931	1.0412	0.8594	0.9677	0.0386
COX6A2	0.9861	1.0447	0.9833	0.8567	0.9677	0.0396
DZIP1	0.9324	1.0044	1.0445	0.8895	0.9677	0.0349
THUMPD3	0.9508	1.1019	1.0848	0.7335	0.9677	0.0851
LOC554175	0.9450	1.0979	0.9323	0.8956	0.9677	0.0446
LOC652317	0.8354	1.1000	1.0522	0.8833	0.9677	0.0641
ZNF530	0.8748	1.1007	0.9272	0.9683	0.9677	0.0483
LOC728748	0.8433	1.2002	0.9610	0.8665	0.9677	0.0816
LOC1001316	0.9082	1.0675	1.0375	0.8577	0.9677	0.0504
LOC647935	0.9081	0.9915	0.8901	1.0813	0.9677	0.0438
INGX	0.8902	1.0844	0.9933	0.9030	0.9677	0.0452
TDGF1	0.9163	0.9815	1.0904	0.8828	0.9678	0.0457
HOXA3	0.9352	1.0768	0.9615	0.8975	0.9678	0.0386
LOC644094	0.8882	1.0465	1.0260	0.9103	0.9678	0.0400
ZNF780B	0.9211	1.0479	0.9857	0.9163	0.9678	0.0311
LOC729744	0.9211	1.0613	1.0447	0.8440	0.9678	0.0518
LOC389102	0.8772	1.1901	0.9088	0.8950	0.9678	0.0744
FLJ39660	0.8651	1.1344	1.0585	0.8131	0.9678	0.0766
LOC646282	0.9413	1.0666	1.0091	0.8541	0.9678	0.0457
LOC651158	0.8513	0.9798	1.1126	0.9274	0.9678	0.0550
CNTNAP5	0.9265	1.0380	0.9723	0.9343	0.9678	0.0255
IFNA5	1.0297	0.9919	0.9657	0.8839	0.9678	0.0309
C6orf124	0.9605	1.0575	1.0317	0.8215	0.9678	0.0529
LOC641810	0.8957	1.0329	1.0581	0.8844	0.9678	0.0452
LOC137107	0.9822	1.0127	1.0699	0.8064	0.9678	0.0568
LOC732402	0.9961	1.0110	0.9417	0.9224	0.9678	0.0212
DEGS2	0.8705	1.0807	0.9764	0.9436	0.9678	0.0437

LOC642083	0.9094	1.0291	1.1088	0.8239	0.9678	0.0631
PRAMEF14	0.9205	1.1082	1.0046	0.8380	0.9678	0.0578
TRY1	0.9455	1.1001	1.0313	0.7944	0.9678	0.0659
LOC645101	0.9252	1.0928	0.9746	0.8787	0.9678	0.0460
LOC729476	0.9047	1.0217	0.9595	0.9854	0.9678	0.0246
LOC645313	0.8215	1.0842	1.1080	0.8576	0.9678	0.0746
PCDHAC2	0.9167	0.9864	1.0394	0.9289	0.9678	0.0283
LOC649542	0.9053	1.0721	0.9425	0.9515	0.9678	0.0362
LOC649937	0.9453	1.0648	0.9722	0.8891	0.9678	0.0367
LOC645254	0.9248	0.9495	1.0231	0.9741	0.9679	0.0210
LOC389662	0.7720	1.1124	1.1592	0.8278	0.9679	0.0981
SERTAD4	0.9229	1.0373	0.9714	0.9399	0.9679	0.0252
GLIPR2	0.8770	1.3933	0.9048	0.6964	0.9679	0.1491
LOC641693	0.8361	1.0846	0.9965	0.9542	0.9679	0.0516
FOXL2	0.8941	1.0146	0.9797	0.9830	0.9679	0.0258
LOC1001299	0.8977	1.0673	0.9621	0.9444	0.9679	0.0358
KIAA1660	0.9118	1.0768	0.9223	0.9607	0.9679	0.0378
TIGD3	0.9535	1.1471	0.9734	0.7975	0.9679	0.0715
KLHDC9	0.9693	1.2588	0.8664	0.7769	0.9679	0.1047
KLRC3	0.8971	1.0653	1.0248	0.8844	0.9679	0.0454
CXorf18	0.8847	1.0115	1.0607	0.9146	0.9679	0.0411
C16orf86	1.0348	1.0053	0.9814	0.8500	0.9679	0.0408
PDE4D	0.9172	1.2391	0.9450	0.7703	0.9679	0.0982
LOC646452	0.9772	1.0692	0.9681	0.8571	0.9679	0.0434
LOC642325	1.0278	1.0744	0.9855	0.7839	0.9679	0.0640
ELAVL4	0.9578	1.0572	0.9003	0.9563	0.9679	0.0326
AOX1	0.9782	1.0109	0.9837	0.8988	0.9679	0.0241
SNORD24	1.0110	1.0313	0.9270	0.9023	0.9679	0.0314
LY6G5B	0.9857	1.0074	0.9897	0.8887	0.9679	0.0268
OR2W1	0.9061	1.1270	0.9342	0.9043	0.9679	0.0535
LOC729568	0.9363	1.0139	0.9975	0.9239	0.9679	0.0222
MGC16291	1.0248	1.1227	0.9059	0.8183	0.9679	0.0667
CASP10	0.9553	1.0634	0.9627	0.8903	0.9679	0.0357
LOC645679	0.8918	1.1069	0.9641	0.9089	0.9679	0.0488
LOC441228	0.8322	1.1129	0.9754	0.9513	0.9679	0.0576
SNORD19	0.9444	0.9773	1.0095	0.9406	0.9679	0.0161
LOC1001288	0.8957	1.0397	0.9916	0.9447	0.9679	0.0309
MIR331	0.8279	0.9815	1.0733	0.9891	0.9680	0.0511
APOBEC3D	0.9508	1.0789	1.0029	0.8393	0.9680	0.0503
LOC647856	0.7298	1.2687	1.2137	0.6596	0.9680	0.1588
LOC1001330	0.9513	1.0022	0.9483	0.9700	0.9680	0.0124
RPESP	0.9642	1.0599	0.9388	0.9091	0.9680	0.0326
LOC644686	0.8653	1.1441	0.9751	0.8874	0.9680	0.0633
CRSP2	0.9221	1.0748	0.9950	0.8800	0.9680	0.0428
TPCN2	0.9515	1.1297	0.9461	0.8446	0.9680	0.0593
FAM71E2	0.9515	1.1210	0.9245	0.8749	0.9680	0.0534

LOC645116	0.8464	1.0286	1.0505	0.9465	0.9680	0.0463
LOC342900	0.9021	1.0708	1.0214	0.8777	0.9680	0.0465
LOC1001327	0.9254	0.9396	1.1288	0.8781	0.9680	0.0552
IGSF1	0.8817	1.0727	0.9757	0.9419	0.9680	0.0400
LOC730145	0.8926	1.0305	1.0142	0.9347	0.9680	0.0327
LOC653651	0.8903	1.0155	1.0424	0.9238	0.9680	0.0363
GLT8D2	0.9055	1.0727	0.9610	0.9328	0.9680	0.0367
CDK7	0.8678	1.3614	0.9510	0.6918	0.9680	0.1418
PRR20E	0.9264	0.9931	1.0219	0.9307	0.9680	0.0236
SPTLC2	0.8649	1.1613	0.9591	0.8867	0.9680	0.0675
LOC1001279	1.0101	1.0046	1.0250	0.8324	0.9680	0.0454
LOC727872	0.9536	1.0419	1.0117	0.8649	0.9680	0.0390
ATP1A3	0.9911	1.0858	0.9967	0.7984	0.9680	0.0606
CDC42EP1	0.8866	1.0596	1.0174	0.9085	0.9680	0.0418
LOC645325	0.8837	1.0430	0.9636	0.9818	0.9680	0.0328
C9orf3	0.9063	1.1014	1.0070	0.8575	0.9680	0.0543
CEACAM1	0.9096	1.0493	1.0165	0.8967	0.9680	0.0381
LOC645508	0.9348	1.1664	0.8594	0.9115	0.9680	0.0680
LOC1001338	0.8537	1.2022	0.9964	0.8200	0.9680	0.0869
LOC647681	0.8929	1.1527	1.0876	0.7390	0.9681	0.0942
LOC1001319	0.8390	1.1052	0.9595	0.9686	0.9681	0.0544
UBE2Z	0.8716	1.3751	0.8685	0.7570	0.9681	0.1383
LOC400713	0.8955	1.1345	1.0046	0.8378	0.9681	0.0654
OR9Q1	0.9714	1.0160	0.8946	0.9902	0.9681	0.0261
RAB11B	0.9285	1.2235	0.9367	0.7836	0.9681	0.0921
LOC1001281	0.8920	1.0557	0.9999	0.9247	0.9681	0.0369
LOC649878	0.8652	0.9865	1.0524	0.9682	0.9681	0.0388
LOC1001304	0.9295	1.0627	1.0260	0.8542	0.9681	0.0472
CALCRL	0.9598	1.0177	0.9587	0.9362	0.9681	0.0174
CDY2B	1.0117	1.0376	0.9786	0.8446	0.9681	0.0429
LOC651881	0.8833	1.1097	1.0060	0.8734	0.9681	0.0560
LOC642257	0.9567	1.0878	0.9792	0.8487	0.9681	0.0490
ERCC-00067	0.9067	1.0944	0.9802	0.8912	0.9681	0.0464
PPM2C	0.9015	1.0664	1.0797	0.8249	0.9681	0.0626
UBE2S	0.9502	1.1497	0.9502	0.8225	0.9681	0.0676
FAM18A	0.8805	1.0086	1.1387	0.8448	0.9681	0.0668
FAM18B2	0.8025	1.1971	1.1077	0.7653	0.9681	0.1082
LOC642639	0.9262	1.0236	0.9654	0.9573	0.9681	0.0203
FLJ30679	0.8459	1.0711	1.0431	0.9125	0.9681	0.0534
CCDC61	0.9225	1.1239	0.9500	0.8761	0.9681	0.0541
DUSP13	0.8862	1.0390	1.0517	0.8957	0.9681	0.0447
LOC1001343	0.9073	0.9808	1.0173	0.9671	0.9681	0.0229
LOC647246	0.8839	1.1462	1.0164	0.8261	0.9681	0.0715
KRTAP5-2	0.8547	1.0094	1.1267	0.8818	0.9681	0.0627
OR5B21	0.8683	1.0529	1.0921	0.8593	0.9682	0.0608
MUC1	0.8797	1.1276	0.9621	0.9033	0.9682	0.0559

LOC343705	0.9161	1.1164	0.9794	0.8607	0.9682	0.0551
KIAA1975	0.9701	1.0279	0.8999	0.9748	0.9682	0.0263
FUT9	0.9798	1.0292	0.9356	0.9281	0.9682	0.0233
PTN	0.9129	1.1245	0.8816	0.9536	0.9682	0.0542
LOC1001315	0.9001	1.1277	0.9670	0.8779	0.9682	0.0564
CLEC9A	0.9367	0.9538	0.9764	1.0057	0.9682	0.0149
CAMK4	0.9166	1.0240	1.0221	0.9100	0.9682	0.0317
ADCY8	0.9340	1.0384	1.0723	0.8280	0.9682	0.0552
MGC27348	1.0076	0.9788	0.9587	0.9277	0.9682	0.0168
LOC643175	0.9387	1.0582	1.0358	0.8400	0.9682	0.0500
LOC727910	0.8503	1.0319	1.0644	0.9262	0.9682	0.0491
LOC642731	0.9943	1.0370	0.9749	0.8666	0.9682	0.0363
ACSS3	0.9266	1.1027	0.9555	0.8879	0.9682	0.0469
C9orf29	0.8686	1.0201	1.0443	0.9398	0.9682	0.0400
LOC440181	0.8852	1.1548	1.0145	0.8183	0.9682	0.0743
BGLAP	0.8507	0.9902	1.0471	0.9848	0.9682	0.0416
MIR873	0.9215	0.9527	1.0722	0.9264	0.9682	0.0353
AMBRA1	0.8407	1.0594	1.0182	0.9545	0.9682	0.0477
CLIC6	0.8561	1.0574	1.0607	0.8987	0.9682	0.0532
LOC647275	0.9329	1.1178	0.9774	0.8447	0.9682	0.0570
LOC1001311	0.8856	1.0931	1.0190	0.8752	0.9682	0.0530
LOC1001342	0.8945	1.0625	0.9546	0.9613	0.9682	0.0348
ARAP2	0.9100	1.0818	0.9839	0.8973	0.9682	0.0424
CFI	0.9340	0.9979	1.1362	0.8049	0.9682	0.0689
LOC641912	0.9873	1.0551	1.0701	0.7605	0.9682	0.0716
SNORA47	0.9572	0.9920	0.9877	0.9361	0.9682	0.0132
SKIP	0.8591	1.1076	0.9792	0.9272	0.9683	0.0525
LOC644682	0.9543	1.0125	1.0153	0.8909	0.9683	0.0294
LOC652767	0.9429	0.9759	1.0105	0.9438	0.9683	0.0160
LOC1001315	0.9738	0.9638	0.9954	0.9400	0.9683	0.0115
OR2T5	0.9593	1.0678	0.9524	0.8935	0.9683	0.0363
MIR301B	0.9086	1.1454	0.9499	0.8691	0.9683	0.0613
SNORD79	0.9346	1.1260	0.9931	0.8193	0.9683	0.0638
TSPAN11	0.8280	1.1587	1.0026	0.8838	0.9683	0.0732
MIR937	0.9603	0.9919	0.9586	0.9622	0.9683	0.0079
RUTBC2	0.8767	0.9971	0.9865	1.0128	0.9683	0.0310
APRIN	1.0200	1.0604	0.9987	0.7941	0.9683	0.0595
RPL26L1	0.8799	1.2409	1.0160	0.7364	0.9683	0.1073
FLJ37078	0.9049	1.1203	1.0496	0.7984	0.9683	0.0722
FAM90A9	0.9474	1.0619	1.0048	0.8591	0.9683	0.0433
MGC16121	0.8849	1.1540	0.9433	0.8911	0.9683	0.0633
TJP3	1.0533	1.1313	0.9527	0.7360	0.9683	0.0856
LOC643266	0.9948	1.0130	1.0016	0.8639	0.9683	0.0350
TTC29	0.9493	1.1419	0.9615	0.8205	0.9683	0.0661
MIR516A1	0.9163	1.0180	0.9944	0.9445	0.9683	0.0231
LOC643002	0.9121	0.9500	1.1038	0.9073	0.9683	0.0462

LOC648926	0.9340	1.1116	0.8896	0.9380	0.9683	0.0490
C19orf21	0.9407	1.1108	1.0051	0.8166	0.9683	0.0615
CD200	0.9224	1.0267	1.0003	0.9239	0.9683	0.0266
C7orf58	0.9385	1.0693	1.0074	0.8581	0.9683	0.0454
LOC650103	0.9236	0.9499	1.0234	0.9763	0.9683	0.0213
ILVBL	0.8083	1.3417	0.9623	0.7610	0.9683	0.1317
LOC388161	0.9022	1.0310	0.9829	0.9572	0.9683	0.0268
TREML2P	0.8877	1.0580	0.9754	0.9523	0.9683	0.0352
FLJ44379	0.9257	1.0646	0.9989	0.8841	0.9683	0.0399
PCDH10	0.9149	1.1630	0.9478	0.8477	0.9683	0.0681
ZFYVE27	0.9109	1.1956	0.9472	0.8198	0.9683	0.0803
RFX3	0.9171	1.2176	0.8941	0.8446	0.9683	0.0845
ADAM3A	0.9182	1.1008	0.9926	0.8618	0.9683	0.0516
SLC25A45	0.9780	1.1455	0.9371	0.8128	0.9683	0.0687
SCTR	1.0070	1.0744	0.8913	0.9007	0.9683	0.0440
SNORD15B	0.8452	1.0532	1.0483	0.9267	0.9684	0.0504
SNORA14A	0.9081	1.0274	0.9516	0.9863	0.9684	0.0254
DLG1	0.9891	1.1502	0.8517	0.8823	0.9684	0.0674
IPF1	0.9386	1.0697	0.9597	0.9055	0.9684	0.0356
STAB1	0.9068	1.0546	1.0665	0.8456	0.9684	0.0547
MCART2	0.9436	1.0739	0.9799	0.8761	0.9684	0.0412
LOC1001309	0.9008	1.0397	1.0165	0.9165	0.9684	0.0350
MPPED1	0.8388	1.0703	0.9970	0.9673	0.9684	0.0483
LOC148203	0.8992	1.0136	1.0285	0.9322	0.9684	0.0313
LOC644680	0.9063	0.9995	1.0492	0.9185	0.9684	0.0340
SNORA38	0.8810	1.1584	0.9200	0.9140	0.9684	0.0639
OR11H4	0.8947	1.0595	0.9760	0.9433	0.9684	0.0347
LOC644887	0.9273	0.9992	0.9867	0.9603	0.9684	0.0159
HSFX1	0.8900	1.0763	0.9283	0.9790	0.9684	0.0403
ITGA11	0.8761	1.0541	1.1107	0.8326	0.9684	0.0674
RANBP3	0.9064	1.2774	0.8962	0.7936	0.9684	0.1061
FLJ46361	0.8628	1.0813	1.0407	0.8888	0.9684	0.0543
CD74	0.9387	1.0314	1.0346	0.8689	0.9684	0.0399
LOC641942	1.0419	1.0106	0.9167	0.9044	0.9684	0.0341
LOC646004	0.9107	0.9960	1.1352	0.8318	0.9684	0.0649
INCA1	0.8848	1.0676	1.0926	0.8285	0.9684	0.0657
LOC442434	0.9152	1.0201	0.9535	0.9848	0.9684	0.0224
LOC645582	0.9147	1.0879	1.0106	0.8604	0.9684	0.0505
LOC1001011	1.0011	1.0210	0.9700	0.8815	0.9684	0.0308
C16orf87	0.8089	1.3994	0.9198	0.7456	0.9684	0.1481
THEG	0.8759	1.1411	0.9468	0.9098	0.9684	0.0594
LOC652645	0.9666	1.0533	1.0045	0.8493	0.9684	0.0435
LOC646310	0.8749	1.0436	1.1024	0.8529	0.9684	0.0617
TRPC4	0.7676	1.0370	1.0960	0.9730	0.9684	0.0715
LOC647506	0.9871	0.9596	1.0797	0.8473	0.9684	0.0479
MYL3	0.8267	1.0316	0.9789	1.0366	0.9684	0.0490

PBEF1	0.8798	1.1657	0.9761	0.8522	0.9684	0.0709
MYO9B	0.8678	1.2494	0.8817	0.8748	0.9684	0.0937
PCDHGA7	0.9595	1.0619	0.9673	0.8850	0.9684	0.0363
C6orf134	0.9464	1.0203	1.0181	0.8890	0.9684	0.0316
C14orf166B	0.8892	1.0858	0.9723	0.9265	0.9684	0.0427
LOC401589	0.9226	1.0762	1.0044	0.8705	0.9684	0.0453
LOC728052	1.0015	1.0338	0.9441	0.8944	0.9685	0.0309
FTHL19	0.8862	1.0659	1.0562	0.8655	0.9685	0.0537
FAM66E	0.8682	1.0443	1.0240	0.9372	0.9685	0.0407
FLJ22269	0.9328	0.9789	1.0764	0.8857	0.9685	0.0407
MS4A5	0.8416	1.1477	0.9847	0.8998	0.9685	0.0666
DHX57	0.9324	1.1634	0.9317	0.8463	0.9685	0.0681
PATZ1	0.9238	1.1234	0.9162	0.9105	0.9685	0.0517
FLJ43859	0.8667	1.0898	1.0147	0.9026	0.9685	0.0513
LOC1001315	0.8785	1.0531	1.0261	0.9161	0.9685	0.0421
LOC644621	0.9560	0.9624	1.1166	0.8390	0.9685	0.0569
ZNF761	0.9099	1.2180	0.9341	0.8119	0.9685	0.0873
ZNF658B	0.9041	1.1290	0.9628	0.8781	0.9685	0.0563
LOC729626	0.8081	0.9922	1.0356	1.0381	0.9685	0.0545
C12orf33	0.9126	0.9977	1.0350	0.9287	0.9685	0.0289
LOC441488	0.9419	0.9503	1.0298	0.9519	0.9685	0.0205
FABP2	0.9595	0.9567	1.0404	0.9174	0.9685	0.0258
LOC644658	1.0126	1.0513	1.0054	0.8047	0.9685	0.0555
SNAG1	1.0227	1.0489	0.9427	0.8597	0.9685	0.0427
LOC649191	0.9656	1.0178	0.9427	0.9480	0.9685	0.0171
LOC728143	0.9471	1.0151	1.0041	0.9078	0.9685	0.0251
PXDNL	0.9787	1.0527	0.9199	0.9228	0.9685	0.0311
LOC1001313	0.7962	1.0159	1.0989	0.9631	0.9685	0.0639
LOC1001310	1.0045	0.9770	1.0076	0.8850	0.9685	0.0287
LOC284274	0.9592	1.0535	0.9742	0.8872	0.9685	0.0341
LOC727866	0.9319	1.0189	1.0165	0.9068	0.9685	0.0289
LRRC16B	0.8813	1.0585	1.0277	0.9066	0.9685	0.0438
LOC644569	0.8925	1.0665	1.0415	0.8736	0.9685	0.0498
LOC401860	0.9857	1.0463	0.9386	0.9035	0.9685	0.0309
FOXB1	0.9861	0.9789	1.0086	0.9005	0.9685	0.0235
TREML3	0.9105	1.0012	1.0263	0.9361	0.9685	0.0271
OR52B4	0.9455	1.0640	0.9374	0.9273	0.9685	0.0320
TGM1	0.8556	1.1038	1.0410	0.8738	0.9686	0.0614
LOC347487	0.9218	1.0094	1.1021	0.8410	0.9686	0.0562
MGC11082	0.9068	1.0681	1.0218	0.8775	0.9686	0.0455
LOC643388	0.8999	1.0586	1.0731	0.8427	0.9686	0.0574
LOC1001342	0.9481	0.9740	0.9947	0.9575	0.9686	0.0102
LOC653059	0.8632	1.0926	0.9800	0.9385	0.9686	0.0479
LOC646887	0.9749	0.9593	1.0567	0.8834	0.9686	0.0355
NTM	0.9416	1.0306	0.9992	0.9030	0.9686	0.0286
LOC399491	0.9453	1.1021	0.9902	0.8368	0.9686	0.0549

LOC648657	0.9002	1.0773	1.0669	0.8300	0.9686	0.0615
PRM2	0.9419	0.9715	1.0672	0.8938	0.9686	0.0366
C20orf132	0.9133	1.0096	1.0055	0.9460	0.9686	0.0235
DGCR5	0.9748	1.0425	1.0370	0.8201	0.9686	0.0518
C5orf45	0.9305	1.1196	0.9688	0.8555	0.9686	0.0556
LOC728522	0.9241	1.0731	0.9908	0.8865	0.9686	0.0410
VANGL2	1.0571	1.1249	0.8980	0.7945	0.9686	0.0750
LOC390846	1.0075	0.9989	0.9802	0.8880	0.9686	0.0275
MAGED4	0.8188	1.0490	1.0332	0.9734	0.9686	0.0525
LOC647213	0.9757	0.9956	0.9519	0.9513	0.9686	0.0106
LOC651515	0.8758	1.1073	0.9790	0.9125	0.9686	0.0509
CDON	0.9030	1.0316	1.0130	0.9269	0.9686	0.0316
CPA1	0.9177	1.0419	1.0208	0.8940	0.9686	0.0368
GPRIN3	0.9059	1.0769	1.0345	0.8571	0.9686	0.0520
LOC652251	0.9430	1.1590	0.9314	0.8411	0.9686	0.0674
LOC442668	0.9717	1.0610	0.8973	0.9446	0.9686	0.0344
C9orf135	0.8645	1.1397	0.9916	0.8787	0.9686	0.0637
FRK	1.0543	0.9382	0.9236	0.9584	0.9686	0.0294
C9orf50	0.9066	1.0986	0.9435	0.9260	0.9687	0.0440
LOC391132	0.9355	1.0787	1.0315	0.8289	0.9687	0.0553
ST5	0.9554	1.1272	0.9136	0.8785	0.9687	0.0551
VCPIP1	0.9016	1.1584	0.9527	0.8619	0.9687	0.0659
LOC646403	0.9181	1.0653	0.9530	0.9383	0.9687	0.0330
TERT	0.9156	1.0204	0.9761	0.9626	0.9687	0.0216
LOC1001338	0.8565	1.0742	1.0798	0.8642	0.9687	0.0626
PGBD5	0.9162	0.9646	1.0319	0.9620	0.9687	0.0238
STAT4	0.8933	1.1104	0.9157	0.9553	0.9687	0.0489
LOC1001307	0.9314	1.0949	0.9198	0.9286	0.9687	0.0422
DNM3	0.8934	1.0713	1.0085	0.9015	0.9687	0.0431
KPNA1	0.7472	1.3428	1.0343	0.7505	0.9687	0.1417
PRO0132	0.9033	1.0735	0.8901	1.0079	0.9687	0.0438
NRXN2	0.9195	1.1166	0.9644	0.8743	0.9687	0.0526
MIR140	0.9942	0.9677	1.0251	0.8878	0.9687	0.0294
SLC16A4	0.9454	1.0471	0.9604	0.9220	0.9687	0.0273
SLCO2B1	0.9253	1.0367	1.0451	0.8677	0.9687	0.0433
FBXL12	0.9359	1.2727	0.9006	0.7656	0.9687	0.1078
CDX4	1.0387	0.9540	0.9889	0.8933	0.9687	0.0306
LOC728170	0.8738	1.0923	0.9903	0.9184	0.9687	0.0477
FLJ32252	0.8959	0.9837	1.0360	0.9593	0.9687	0.0291
LOC1001329	1.0188	1.0221	0.9742	0.8598	0.9687	0.0379
FGL2	0.8162	1.1319	1.0895	0.8373	0.9687	0.0825
LOC1001294	0.9207	1.0180	1.0093	0.9270	0.9687	0.0260
ISLR2	0.9379	1.1128	1.0399	0.7844	0.9688	0.0712
ETV3	0.8440	1.2097	1.0622	0.7591	0.9688	0.1026
FLJ37396	0.9315	1.1266	1.0031	0.8138	0.9688	0.0655
LOC404266	0.9481	0.9744	1.0148	0.9377	0.9688	0.0172

LOC1001342	0.9302	1.0468	1.0065	0.8916	0.9688	0.0353
SIX3	0.8438	1.0498	1.0536	0.9278	0.9688	0.0509
LOC1001303	0.9112	1.0839	0.9348	0.9452	0.9688	0.0390
LOC400831	0.9877	0.9633	1.0016	0.9226	0.9688	0.0173
C6orf81	1.0021	1.0626	0.9901	0.8203	0.9688	0.0520
TLX3	0.9731	1.1509	0.8672	0.8840	0.9688	0.0650
ADRB3	0.9719	1.0201	0.9900	0.8931	0.9688	0.0271
SNORD67	0.7950	1.1437	1.0861	0.8504	0.9688	0.0859
IL7	0.9232	1.0239	1.0123	0.9159	0.9688	0.0286
LOC643922	0.8692	1.1437	1.0392	0.8231	0.9688	0.0746
LOC1001317	0.9338	1.0886	1.0145	0.8383	0.9688	0.0538
FAM20C	0.9037	1.0718	0.9606	0.9392	0.9688	0.0363
CT45-2	0.9228	1.0127	0.9662	0.9736	0.9688	0.0184
LOC1001011	0.8749	1.0440	1.0653	0.8911	0.9688	0.0499
LOC391124	0.9450	1.1130	0.9761	0.8413	0.9688	0.0560
SNORD51	0.9147	1.0761	1.0152	0.8694	0.9688	0.0470
GRM3	0.9368	1.0716	1.0058	0.8612	0.9688	0.0452
FLJ44216	0.9854	1.0344	0.9248	0.9307	0.9688	0.0258
LOC647634	0.9283	1.0626	0.9812	0.9034	0.9689	0.0352
WDR52	0.8960	1.0675	1.0405	0.8714	0.9689	0.0497
LOC392617	0.9462	1.1487	0.9742	0.8063	0.9689	0.0703
IL12A	0.8425	1.1211	1.0347	0.8771	0.9689	0.0658
C14orf118	0.9392	1.0940	1.0118	0.8305	0.9689	0.0559
LRP1B	0.9300	1.0298	1.0516	0.8640	0.9689	0.0438
LOC652678	1.0399	0.9653	0.9908	0.8794	0.9689	0.0336
FSCN3	0.8537	1.0774	1.0275	0.9168	0.9689	0.0510
C6orf204	0.8984	1.0895	1.0136	0.8740	0.9689	0.0504
PNPO	0.7693	1.3719	1.0226	0.7117	0.9689	0.1504
MEG8	1.0077	1.0660	1.0593	0.7425	0.9689	0.0766
WDFY3	0.8646	1.1455	0.9693	0.8962	0.9689	0.0628
ZNF280A	0.9005	1.0882	0.9777	0.9091	0.9689	0.0434
LOC1001318	1.0165	1.0163	1.0190	0.8237	0.9689	0.0484
FLJ35785	0.9327	1.1044	0.9704	0.8680	0.9689	0.0499
LOC652730	0.9370	0.9968	1.0747	0.8670	0.9689	0.0441
MYH3	0.9736	1.0921	0.9638	0.8460	0.9689	0.0503
ROM1	0.8697	1.1609	1.0769	0.7680	0.9689	0.0907
LOC388795	0.8833	1.0716	1.0031	0.9175	0.9689	0.0425
C20orf166	0.8943	1.0184	1.0392	0.9237	0.9689	0.0354
LOC651309	0.9172	0.9571	1.0305	0.9708	0.9689	0.0235
LOC651133	0.9343	1.0568	1.0444	0.8401	0.9689	0.0510
CBWD5	0.8902	1.2809	1.0267	0.6778	0.9689	0.1264
OR2J3	0.8761	1.0576	1.0447	0.8972	0.9689	0.0478
ADAP1	0.9542	1.1159	0.8761	0.9295	0.9689	0.0516
OPCML	0.9172	0.9998	1.0216	0.9371	0.9689	0.0249
TAGLN3	0.9188	1.1538	0.9272	0.8759	0.9689	0.0627
LOC401622	0.9077	1.0799	0.9711	0.9171	0.9689	0.0395

CALML6	0.9780	1.0667	0.9681	0.8629	0.9689	0.0417
LOC642198	0.9230	1.0260	1.0370	0.8898	0.9689	0.0368
HOXB7	0.8552	1.1237	0.9987	0.8982	0.9689	0.0597
LOC284215	0.9720	0.9685	1.0146	0.9208	0.9690	0.0192
FLJ13197	0.8809	1.0694	1.0192	0.9064	0.9690	0.0450
LOC1001290	0.9126	1.0439	1.0360	0.8833	0.9690	0.0415
FN1	0.9612	1.0355	0.9696	0.9096	0.9690	0.0258
FLJ43763	0.9830	0.9864	0.9579	0.9486	0.9690	0.0093
TPD52L3	0.9857	1.0520	0.9771	0.8612	0.9690	0.0396
STXBP1	1.0708	1.0871	0.9162	0.8018	0.9690	0.0677
LOC1001299	0.9690	1.1202	0.9357	0.8510	0.9690	0.0562
LOC653687	0.9009	1.1287	0.9752	0.8710	0.9690	0.0576
ANXA8L1	0.8782	1.0435	1.0823	0.8719	0.9690	0.0548
MIR1537	0.9044	1.0922	0.9981	0.8813	0.9690	0.0482
SLC34A3	0.9947	1.0002	0.9883	0.8928	0.9690	0.0255
CD36	0.8997	0.9806	1.0688	0.9269	0.9690	0.0373
SPATA22	0.9505	0.9805	1.0073	0.9377	0.9690	0.0156
LOC391033	0.8996	1.1101	0.9903	0.8760	0.9690	0.0531
LOC644768	0.8900	1.0392	1.0672	0.8796	0.9690	0.0490
LOC1001324	0.8330	1.1183	1.0892	0.8355	0.9690	0.0780
C1orf138	0.9374	1.1963	0.9034	0.8389	0.9690	0.0785
LOC648279	0.9572	1.0576	0.9844	0.8769	0.9690	0.0373
ITGA8	0.9334	1.0342	1.0183	0.8901	0.9690	0.0344
GAP43	0.9593	1.0349	0.8888	0.9930	0.9690	0.0309
ETHE1	0.8680	1.1836	0.9510	0.8734	0.9690	0.0740
LGTN	0.9117	1.1816	0.9904	0.7924	0.9690	0.0817
GAB3	0.8963	1.1058	1.0297	0.8443	0.9690	0.0600
TRIM35	0.8861	1.1230	1.0186	0.8484	0.9690	0.0630
PPP1R14A	1.0693	1.0392	0.8854	0.8823	0.9690	0.0496
SYCN	0.8648	1.0286	1.0361	0.9467	0.9690	0.0402
ZNF781	0.9222	1.0901	1.0074	0.8564	0.9690	0.0508
OR5M9	0.9944	0.9885	0.9967	0.8966	0.9690	0.0242
JAM2	1.0029	1.0267	0.9697	0.8768	0.9690	0.0329
LOC642475	0.9946	1.0451	0.9611	0.8754	0.9690	0.0357
ADH6	0.9241	1.0840	1.0167	0.8513	0.9690	0.0511
C17orf47	0.9024	1.0763	0.9987	0.8989	0.9690	0.0426
ADAMTS3	0.9757	1.0151	0.9061	0.9793	0.9691	0.0228
LOC1001330	0.9567	1.1259	0.9505	0.8432	0.9691	0.0584
LOC650062	0.9259	1.0411	0.9974	0.9119	0.9691	0.0304
C14orf19	0.9224	1.0047	1.0560	0.8932	0.9691	0.0374
MERTK	0.9604	1.1342	0.9545	0.8272	0.9691	0.0630
LRRC41	0.9038	1.5089	0.7487	0.7150	0.9691	0.1846
ERC2	0.9368	1.1110	0.9608	0.8677	0.9691	0.0513
PARL	0.8112	1.3567	1.0506	0.6578	0.9691	0.1524
LOC1001331	0.7698	1.3832	0.9847	0.7385	0.9691	0.1485
ACSL6	0.9489	1.0237	0.9835	0.9203	0.9691	0.0223

OR10P1	0.9316	0.9751	1.0788	0.8908	0.9691	0.0404
LOC642380	0.8982	1.0204	1.0155	0.9422	0.9691	0.0296
TRIM46	0.8657	1.1655	0.9778	0.8674	0.9691	0.0705
BHLHE22	0.9191	1.0023	1.0052	0.9499	0.9691	0.0210
LOC649120	0.9069	1.0302	0.9864	0.9529	0.9691	0.0261
LOC1001345	0.9677	0.9596	1.0876	0.8615	0.9691	0.0463
MSL1	0.8396	1.2225	1.0539	0.7604	0.9691	0.1048
GDF1	0.9100	1.0675	0.9742	0.9248	0.9691	0.0356
MPP4	0.8794	1.0949	0.9558	0.9464	0.9691	0.0453
HPD	1.0118	1.0692	1.0007	0.7947	0.9691	0.0601
SYMPK	1.0589	1.0471	0.9366	0.8339	0.9691	0.0528
ARHGAP22	0.9546	0.9865	1.0334	0.9020	0.9691	0.0276
TMIE	0.8861	1.0331	1.0312	0.9261	0.9691	0.0373
MIR361	0.8526	1.1502	0.9955	0.8782	0.9691	0.0679
LOC644587	0.9308	1.0428	0.9776	0.9253	0.9691	0.0272
LOC644838	0.9122	1.0112	1.0538	0.8994	0.9691	0.0377
LOC649086	0.8699	1.0736	1.0243	0.9088	0.9691	0.0478
MIR30C2	0.9373	1.0232	1.0114	0.9047	0.9692	0.0287
KCNQ1	0.9014	1.0792	0.9976	0.8985	0.9692	0.0433
KRTAP3-2	0.9436	1.0107	1.0331	0.8892	0.9692	0.0327
PRAMEF4	0.9472	1.0876	1.0109	0.8311	0.9692	0.0542
CFTR	0.8005	1.1619	0.9532	0.9611	0.9692	0.0741
LOC400388	0.9089	1.1246	0.9519	0.8913	0.9692	0.0533
LOC1001330	0.8941	1.1275	0.9301	0.9250	0.9692	0.0534
PLCL1	0.8646	1.0797	0.9806	0.9519	0.9692	0.0443
LOC649896	1.0002	1.0403	0.9276	0.9087	0.9692	0.0308
LOC1001325	0.9738	1.0296	1.0020	0.8714	0.9692	0.0345
CKMT2	0.9564	1.0217	0.9739	0.9248	0.9692	0.0202
KIF3C	0.9766	1.0784	0.9448	0.8770	0.9692	0.0419
FAM23B	0.9387	0.9859	0.9467	1.0056	0.9692	0.0159
COL29A1	0.9137	1.0545	0.9689	0.9396	0.9692	0.0306
CEBPE	0.8850	1.0190	1.0375	0.9355	0.9692	0.0358
XIRP1	0.8759	1.1671	0.9773	0.8565	0.9692	0.0711
LOC650739	0.8531	1.0541	1.0885	0.8812	0.9692	0.0596
G0S2	1.0098	0.9686	1.0435	0.8550	0.9692	0.0410
IIP45	0.9433	1.0146	1.0192	0.8998	0.9692	0.0289
MIR302D	0.8802	0.9731	1.0149	1.0087	0.9692	0.0311
LOC729356	0.9108	1.1105	0.9839	0.8718	0.9692	0.0525
LOC442382	0.8281	1.0338	1.0405	0.9746	0.9692	0.0493
ACVR1B	0.9029	1.2855	0.9293	0.7592	0.9692	0.1119
LOC653146	0.8408	1.1927	0.9838	0.8596	0.9692	0.0810
LOC647849	0.9475	1.0317	0.9615	0.9362	0.9692	0.0214
MIR1910	1.0975	0.9530	1.0309	0.7956	0.9692	0.0650
LOC652698	0.9276	1.0760	1.0178	0.8556	0.9692	0.0486
LOC388114	0.8578	1.1185	0.9800	0.9206	0.9693	0.0557
DRD1	0.8527	1.1817	0.9656	0.8771	0.9693	0.0748

PTCRA	0.9505	1.1051	0.9913	0.8301	0.9693	0.0568
LOC390748	0.9322	1.0662	1.0329	0.8459	0.9693	0.0500
UBL4A	0.8211	1.3405	1.0544	0.6611	0.9693	0.1478
LOC649415	0.9168	1.0116	1.1147	0.8340	0.9693	0.0606
FAM106A	0.9274	1.0852	0.9859	0.8787	0.9693	0.0444
SMTNL2	0.9317	1.1327	0.9477	0.8651	0.9693	0.0573
LOC644373	0.8597	1.0563	1.1404	0.8208	0.9693	0.0769
LMO3	0.8890	1.1475	0.9616	0.8792	0.9693	0.0622
FLJ40411	0.9337	1.0166	1.0058	0.9212	0.9693	0.0244
CD8B	0.9245	1.0644	0.9809	0.9074	0.9693	0.0354
DPY19L2	0.9114	1.0119	1.0259	0.9281	0.9693	0.0290
ATP5F1	0.8266	1.2956	1.0073	0.7477	0.9693	0.1216
LOC339535	0.8378	1.1111	1.0620	0.8663	0.9693	0.0687
LOC1001337	1.0071	0.9844	0.9975	0.8883	0.9693	0.0274
LOC653471	1.0447	1.0820	0.9154	0.8352	0.9693	0.0572
KIAA0853	0.9032	0.9373	0.9949	1.0419	0.9693	0.0307
RNF19A	0.8388	1.1357	1.1355	0.7673	0.9693	0.0971
C11orf35	0.9078	1.1811	0.9591	0.8293	0.9693	0.0755
PFKFB1	0.9060	1.0229	1.0227	0.9258	0.9693	0.0311
LOC653194	1.0318	1.0024	0.9714	0.8718	0.9693	0.0348
YAF2	0.9373	1.0980	1.0096	0.8325	0.9693	0.0562
VPS72	0.8356	1.2862	0.9233	0.8322	0.9694	0.1077
GLUD2	0.7791	1.0816	1.0713	0.9454	0.9694	0.0706
TMEM35	0.9042	1.0593	1.0093	0.9046	0.9694	0.0389
LOC652662	0.9510	1.0282	1.0232	0.8750	0.9694	0.0361
IL26	0.9172	1.0856	1.0547	0.8199	0.9694	0.0618
ZNF333	0.9157	1.0480	0.9512	0.9627	0.9694	0.0280
LOC647130	1.0606	0.9490	0.9112	0.9567	0.9694	0.0320
LOC1001303	0.8511	1.1015	1.0615	0.8634	0.9694	0.0653
C21orf74	0.9632	1.0792	1.0188	0.8163	0.9694	0.0563
CLPS	0.8251	1.0685	0.9965	0.9874	0.9694	0.0514
DRD3	0.9061	1.1629	0.9652	0.8433	0.9694	0.0691
LOC392242	0.9138	0.9356	1.0171	1.0110	0.9694	0.0262
LOC729642	0.9807	1.0866	0.9498	0.8605	0.9694	0.0467
LOC646990	0.9285	1.1518	0.9999	0.7974	0.9694	0.0738
LOC123855	0.8593	1.0491	0.9911	0.9782	0.9694	0.0398
FGR	0.9177	1.0542	1.0225	0.8831	0.9694	0.0410
GPR135	0.8714	1.0573	1.0279	0.9210	0.9694	0.0439
LOC729456	0.9445	1.0434	1.0381	0.8517	0.9694	0.0453
LOC645687	0.9636	0.9568	1.0588	0.8984	0.9694	0.0332
WDR87	0.8981	1.0439	1.0050	0.9307	0.9694	0.0334
LOC440981	0.9210	1.1373	0.9058	0.9135	0.9694	0.0560
C14orf102	0.9272	1.3541	0.8565	0.7399	0.9694	0.1339
KGFLP1	0.9336	1.1501	0.9601	0.8338	0.9694	0.0661
DEFB122	0.8863	1.0870	0.9713	0.9331	0.9694	0.0429
MYLK4	0.9441	1.0919	0.9792	0.8624	0.9694	0.0476

PGAP1	0.9022	1.1087	0.9494	0.9175	0.9694	0.0474
KRT28	0.9373	1.0709	0.9932	0.8763	0.9694	0.0414
SPINK4	0.9070	1.0669	0.9770	0.9268	0.9694	0.0357
LOC1001279	0.8794	1.0400	1.0380	0.9204	0.9694	0.0410
LOC644100	0.9085	1.1149	0.9982	0.8561	0.9694	0.0567
SCRT1	0.8618	1.0856	0.9843	0.9460	0.9694	0.0464
CCDC28A	0.8436	1.2805	0.9943	0.7594	0.9694	0.1145
DAZ3	0.8439	1.1082	0.9818	0.9438	0.9694	0.0546
TMEM154	0.9110	1.0744	0.9664	0.9259	0.9694	0.0369
HOXA10	0.9075	1.0870	1.0567	0.8266	0.9694	0.0617
TBC1D3C	0.9447	1.1361	0.9518	0.8453	0.9695	0.0606
APCDD1L	0.8666	1.0937	1.0340	0.8836	0.9695	0.0560
ZNF624	0.9282	1.1337	0.9098	0.9061	0.9695	0.0550
MIR1183	0.9458	1.0202	1.0647	0.8472	0.9695	0.0476
LOC1001280	0.8282	1.1659	0.9923	0.8914	0.9695	0.0737
C9orf62	1.0386	1.0425	1.0041	0.7926	0.9695	0.0596
LOC729905	0.9002	1.1075	1.0642	0.8060	0.9695	0.0705
WHAMM	0.8239	1.3110	0.9696	0.7735	0.9695	0.1212
MCM3AP	0.9253	1.3386	0.8938	0.7202	0.9695	0.1310
LOC1001290	0.8616	1.1283	1.0119	0.8762	0.9695	0.0628
LOC644477	0.9772	1.0179	0.9807	0.9023	0.9695	0.0242
LOC644496	0.9201	1.0534	1.0046	0.8998	0.9695	0.0360
NEDD8	0.7573	1.3185	0.9955	0.8067	0.9695	0.1272
LOC646366	0.9669	1.0491	0.9534	0.9086	0.9695	0.0293
LILRB4	0.8647	1.1049	0.9930	0.9154	0.9695	0.0523
FLJ32790	0.8532	1.0519	1.0706	0.9024	0.9695	0.0540
LELP1	0.8949	0.9468	1.0559	0.9804	0.9695	0.0337
LOC285453	0.8606	1.0727	1.0401	0.9047	0.9695	0.0514
ITGA3	0.8484	1.1470	1.0288	0.8538	0.9695	0.0725
ZNF469	0.9188	1.0471	0.9866	0.9256	0.9695	0.0300
SMC6	1.0591	0.9747	0.9380	0.9063	0.9695	0.0330
UROC1	0.9426	1.0892	1.0004	0.8459	0.9695	0.0511
LOC650965	0.8400	1.0578	1.0254	0.9549	0.9695	0.0482
MIR454	0.9573	1.1132	0.9603	0.8473	0.9695	0.0546
LOC643690	0.9996	1.0795	1.0070	0.7921	0.9695	0.0618
MIR483	0.9437	1.0249	1.0236	0.8859	0.9695	0.0337
LOC1001338	0.8380	1.2389	0.9741	0.8271	0.9695	0.0958
LOC134505	0.9462	1.0528	0.9796	0.8995	0.9695	0.0323
SAMD12	1.0296	0.9848	0.9600	0.9038	0.9695	0.0262
MEFV	0.9833	1.0483	0.9543	0.8923	0.9696	0.0324
LOC643154	0.8015	1.0604	1.0379	0.9784	0.9696	0.0586
MIR196B	0.9493	1.0668	1.0642	0.7979	0.9696	0.0634
LOC1001286	0.8775	1.1767	0.9151	0.9091	0.9696	0.0695
MIR16-2	0.9911	1.0133	0.9854	0.8885	0.9696	0.0277
LOC642606	0.9635	1.0831	1.0068	0.8249	0.9696	0.0542
ALOX5	0.9499	1.0223	0.9773	0.9288	0.9696	0.0202

C14orf115	0.8698	1.0482	1.0328	0.9275	0.9696	0.0427
CDH19	0.9457	1.0342	0.9465	0.9518	0.9696	0.0216
LOC439965	0.9589	1.1504	0.9327	0.8363	0.9696	0.0658
SMOC2	0.9608	1.0031	0.9902	0.9242	0.9696	0.0175
LOC730235	1.0418	1.0820	0.9312	0.8234	0.9696	0.0582
LOC652436	0.8606	1.0628	1.0420	0.9130	0.9696	0.0492
LOC1001323	0.8720	1.1020	0.9699	0.9345	0.9696	0.0485
LOC727994	0.8715	1.0933	1.0063	0.9073	0.9696	0.0501
LOC1001313	0.8974	1.0761	1.0252	0.8796	0.9696	0.0481
LOC651357	0.8400	1.0703	1.1021	0.8659	0.9696	0.0678
S100A8	0.9456	1.0581	1.0193	0.8555	0.9696	0.0446
LOC1001336	0.8808	1.0913	0.9869	0.9195	0.9696	0.0461
SULT1C4	0.9604	1.0684	0.9969	0.8530	0.9696	0.0449
LOC642978	0.8771	1.1646	0.9541	0.8827	0.9696	0.0673
PDE5A	0.9354	1.0527	0.9645	0.9259	0.9696	0.0289
COL6A6	0.9308	1.0133	1.0269	0.9075	0.9696	0.0297
LOC442283	1.0549	0.9359	0.9645	0.9234	0.9697	0.0297
RASGRP1	0.9307	1.0872	0.9153	0.9454	0.9697	0.0397
UNC119	0.8189	1.3320	0.9647	0.7630	0.9697	0.1280
TMEM200C	0.9626	0.9815	1.0071	0.9275	0.9697	0.0167
LOC285556	0.9229	1.1900	1.0136	0.7521	0.9697	0.0913
LOC643486	0.8908	0.9928	1.0404	0.9547	0.9697	0.0316
GPR124	0.8580	1.1061	0.9005	1.0140	0.9697	0.0561
LOC644246	0.9875	1.0202	0.9794	0.8916	0.9697	0.0275
LOC729153	0.9572	1.0458	1.0037	0.8720	0.9697	0.0372
MINK1	0.9062	1.1276	0.8819	0.9631	0.9697	0.0553
SSBP3	0.8930	1.2475	0.9829	0.7553	0.9697	0.1038
LOC388630	0.9311	1.0661	0.9701	0.9115	0.9697	0.0344
C6orf155	0.8995	1.0502	1.1579	0.7711	0.9697	0.0848
ST7OT2	0.8621	1.0719	1.0235	0.9213	0.9697	0.0477
LOC643345	0.8986	0.9709	1.0141	0.9951	0.9697	0.0253
LOC649587	0.8932	1.0921	0.9620	0.9315	0.9697	0.0432
AGBL4	0.9219	0.9905	0.9755	0.9908	0.9697	0.0163
LOC1001329	1.0256	1.0470	0.9131	0.8930	0.9697	0.0389
COL10A1	0.9227	1.0327	1.0164	0.9069	0.9697	0.0320
LOC647843	0.9956	1.0510	0.9609	0.8713	0.9697	0.0377
C9orf171	0.9080	0.9823	1.0902	0.8984	0.9697	0.0443
GDA	0.9281	1.0893	0.9907	0.8708	0.9697	0.0468
LOC652246	0.8900	1.0813	0.9728	0.9348	0.9697	0.0409
SPINLW1	0.9258	1.0860	0.9469	0.9203	0.9697	0.0392
PPM1L	0.9537	1.0629	1.0131	0.8492	0.9697	0.0460
SMAD6	0.9991	1.0910	0.8785	0.9102	0.9697	0.0478
FAM163A	0.9931	1.1186	0.9646	0.8025	0.9697	0.0650
THSD1P	0.9633	1.1081	0.9243	0.8832	0.9697	0.0489
PSKH1	0.8700	1.2290	0.9847	0.7952	0.9697	0.0948
PTPRQ	0.7421	1.1704	1.1215	0.8449	0.9697	0.1044

LOC1001327	0.8797	1.1304	1.0118	0.8570	0.9697	0.0635
NR1H4	0.9542	1.0795	0.9296	0.9156	0.9697	0.0375
SLC39A12	0.9053	1.0616	0.9600	0.9520	0.9697	0.0329
ADAMTS6	0.8885	1.0454	1.0068	0.9382	0.9697	0.0350
LOC647156	0.8985	1.1069	0.9725	0.9011	0.9697	0.0488
TTC15	0.9429	1.1313	0.9929	0.8119	0.9697	0.0660
LOC1001297	0.9223	1.0769	0.9198	0.9599	0.9697	0.0369
KRTDAP	0.9297	1.0307	0.9617	0.9568	0.9697	0.0215
PPP2R1A	0.8223	1.4544	0.8862	0.7162	0.9698	0.1653
CEP57	0.9399	1.1388	1.0151	0.7852	0.9698	0.0739
LOC642360	1.0420	0.9599	0.9913	0.8858	0.9698	0.0327
PDLIM3	0.9491	1.0878	0.9861	0.8561	0.9698	0.0479
SNX31	0.9448	1.0264	1.0267	0.8812	0.9698	0.0353
AGR3	0.8912	1.0951	0.9891	0.9037	0.9698	0.0471
TNFRSF14	0.9721	1.0906	1.0476	0.7689	0.9698	0.0713
INE1	0.9957	1.1386	0.8856	0.8593	0.9698	0.0635
KRTAP3-1	0.9572	1.0037	1.0142	0.9041	0.9698	0.0252
LOC728586	0.9023	1.0123	1.0576	0.9070	0.9698	0.0387
LOC729261	0.9063	1.0504	0.9642	0.9583	0.9698	0.0298
PRICKLE2	0.9742	1.0687	1.0166	0.8198	0.9698	0.0536
AGPAT6	0.9042	1.2412	0.8936	0.8403	0.9698	0.0915
USP42	0.8595	1.3209	0.9807	0.7182	0.9698	0.1287
LOC729400	0.9793	0.9532	0.9943	0.9525	0.9698	0.0103
LOC643807	0.9024	1.0428	1.0515	0.8827	0.9698	0.0449
LOC1001290	0.9848	0.9841	1.0203	0.8901	0.9698	0.0279
OTUD6A	0.9595	0.9996	1.0183	0.9020	0.9698	0.0257
OR4N2	0.9822	0.9735	1.0484	0.8752	0.9698	0.0357
HAL	0.8852	1.0539	1.0856	0.8548	0.9698	0.0584
LOC646198	0.8871	1.1793	0.9813	0.8317	0.9699	0.0763
NBR1	0.8373	1.1491	1.0562	0.8368	0.9699	0.0790
LOC646361	0.9806	1.0104	1.0312	0.8572	0.9699	0.0389
RUNX1T1	0.9299	1.0686	1.0053	0.8756	0.9699	0.0423
LOC732172	0.9166	1.0828	0.9212	0.9589	0.9699	0.0388
ZNF665	0.9133	0.9928	0.9814	0.9920	0.9699	0.0190
GPR114	0.9227	1.0729	0.9736	0.9102	0.9699	0.0370
LOC115648	0.9648	1.1326	1.0582	0.7239	0.9699	0.0889
LOC1001305	0.9097	1.0264	1.0874	0.8560	0.9699	0.0529
LOC1001326	0.8907	1.0734	0.9938	0.9216	0.9699	0.0407
LOC652685	0.7496	1.4332	1.0645	0.6322	0.9699	0.1794
LOC391592	0.9438	1.0438	0.9712	0.9208	0.9699	0.0267
LOC651495	0.9862	1.0076	0.9585	0.9272	0.9699	0.0174
C16orf67	1.0117	1.0549	0.9594	0.8535	0.9699	0.0434
LOC643621	0.9598	0.9514	1.0482	0.9202	0.9699	0.0275
ERCC-00035	0.8915	1.0955	1.0486	0.8439	0.9699	0.0605
FLJ11827	0.8746	1.1560	1.0135	0.8355	0.9699	0.0729
LOC653521	0.8063	1.1409	1.0744	0.8580	0.9699	0.0814

DEFB107A	0.9067	1.1428	1.0010	0.8291	0.9699	0.0675
LOC642277	0.9575	0.9960	1.0356	0.8904	0.9699	0.0309
SCHIP1	0.9845	1.0591	0.9587	0.8773	0.9699	0.0375
LOC401730	0.8679	1.0903	0.9869	0.9345	0.9699	0.0469
LOC1001343	1.0305	1.0171	0.9697	0.8624	0.9699	0.0382
PIBF1	0.8992	1.1043	0.9263	0.9499	0.9699	0.0460
ANKRD20A3	0.8426	1.0656	1.0583	0.9132	0.9699	0.0551
RIMBP3B	0.9912	0.9373	1.0214	0.9298	0.9699	0.0219
FLJ46309	0.8354	1.2126	1.1150	0.7168	0.9699	0.1162
ACCN4	0.9591	1.0376	0.9614	0.9217	0.9699	0.0243
SNORA26	0.9324	1.0882	1.0210	0.8381	0.9699	0.0543
CPSF3L	0.9217	1.1887	0.9923	0.7770	0.9699	0.0856
VOPP1	0.8126	1.2838	0.9073	0.8761	0.9699	0.1065
DNAH9	0.9603	0.9927	1.0179	0.9090	0.9699	0.0235
ADAM33	0.8691	1.1266	0.9745	0.9096	0.9699	0.0566
LOC654056	0.8455	0.9979	0.9791	1.0572	0.9699	0.0447
LOC402057	0.8498	1.2386	0.9991	0.7923	0.9699	0.0996
ICAM4	0.8250	1.0867	1.0097	0.9584	0.9700	0.0550
LOC390714	0.8720	1.0769	1.0383	0.8927	0.9700	0.0514
LOC729417	0.9065	1.1298	1.0368	0.8068	0.9700	0.0711
KRT31	0.8794	1.1283	0.9975	0.8747	0.9700	0.0599
ZNF826	0.9252	1.0771	1.0053	0.8724	0.9700	0.0450
LOC646625	0.9563	1.0814	0.9170	0.9252	0.9700	0.0381
LOC645253	0.9770	1.1532	0.8043	0.9453	0.9700	0.0717
GEN1	0.9479	1.0494	1.0080	0.8747	0.9700	0.0380
WNK4	0.9592	1.0684	0.9396	0.9127	0.9700	0.0342
TPK1	0.9036	1.1104	1.0331	0.8329	0.9700	0.0625
SNORA29	0.9326	1.0939	0.9194	0.9341	0.9700	0.0414
LOC1001335	0.8041	1.3091	0.8621	0.9047	0.9700	0.1149
DPP6	0.9289	1.0745	0.9747	0.9019	0.9700	0.0379
LOC731236	0.8834	1.1448	0.9970	0.8548	0.9700	0.0658
OTUB2	0.9363	1.0561	0.9727	0.9149	0.9700	0.0311
CPNE4	0.9628	1.0360	0.9959	0.8854	0.9700	0.0319
LOC653441	0.9441	1.0616	0.9889	0.8854	0.9700	0.0372
MCHR1	0.9716	1.0943	0.9252	0.8890	0.9700	0.0447
EBF1	0.9523	1.0716	0.9521	0.9040	0.9700	0.0357
FOSL2	0.8816	1.1751	1.0495	0.7739	0.9700	0.0888
PRELID2	0.9611	1.0213	1.0313	0.8663	0.9700	0.0379
HORMAD1	1.0136	1.0002	0.9952	0.8711	0.9700	0.0332
MIR541	0.9695	1.0690	0.9170	0.9246	0.9700	0.0350
LOC391747	0.8843	1.1093	1.0178	0.8687	0.9700	0.0572
LOC1001315	0.9089	1.0098	1.0476	0.9139	0.9700	0.0347
NMT1	1.0236	1.2125	0.9398	0.7043	0.9700	0.1054
LOC732393	0.9207	1.0601	1.0316	0.8678	0.9700	0.0454
C4orf21	0.9381	0.9948	1.0078	0.9395	0.9701	0.0182
IQCJ	0.9636	1.0721	0.9775	0.8671	0.9701	0.0419

C15orf32	0.9162	1.0198	1.0338	0.9104	0.9701	0.0329
DUPD1	0.8265	1.0605	1.1526	0.8407	0.9701	0.0810
LOC652664	0.9232	1.0801	0.9909	0.8861	0.9701	0.0426
FLJ45256	0.8839	1.0651	1.0826	0.8487	0.9701	0.0604
CDH10	0.7945	1.1283	1.0092	0.9484	0.9701	0.0694
LOC441956	0.9308	1.0288	1.0245	0.8962	0.9701	0.0334
LOC1001296	0.8900	1.1549	0.9722	0.8633	0.9701	0.0658
LOC1001340	0.8198	1.2827	0.8726	0.9053	0.9701	0.1057
LOC1001289	0.9721	1.1037	0.9450	0.8596	0.9701	0.0506
LOC645730	0.9146	0.9678	1.0160	0.9820	0.9701	0.0211
LOC643006	0.9244	1.0860	1.0501	0.8199	0.9701	0.0609
LOC158376	1.0069	1.0017	0.8926	0.9792	0.9701	0.0265
TES	0.9989	1.0836	0.9668	0.8311	0.9701	0.0525
IVL	0.8852	1.1439	0.9158	0.9356	0.9701	0.0588
PHOSPHO2	0.8660	1.1635	1.0528	0.7981	0.9701	0.0840
TMEM231	0.9628	1.2083	0.9062	0.8031	0.9701	0.0860
NIPSNAP3B	1.0026	0.9445	1.0251	0.9082	0.9701	0.0267
LOC642570	0.9753	1.1312	0.9854	0.7885	0.9701	0.0702
CYP26A1	0.9428	1.0201	0.9958	0.9218	0.9701	0.0228
AGBL2	0.8417	1.1412	0.9533	0.9445	0.9701	0.0624
LOC646867	0.9437	0.9922	1.0288	0.9159	0.9701	0.0251
LOC1001322	0.9072	1.0757	1.0105	0.8872	0.9702	0.0444
LOC650373	0.9178	1.0306	1.0464	0.8858	0.9702	0.0401
LOC652585	0.8915	1.0424	0.9769	0.9698	0.9702	0.0309
LOC1001323	0.9420	1.2057	0.8510	0.8820	0.9702	0.0808
GUCA1C	0.8728	1.1901	0.9137	0.9040	0.9702	0.0738
PPP1R3A	0.8732	1.1571	0.9716	0.8789	0.9702	0.0663
LOC727753	0.9300	1.1417	1.0920	0.7169	0.9702	0.0958
PLAUR	0.9479	1.0653	0.9941	0.8734	0.9702	0.0403
DKFZp667F0	0.8949	1.0329	1.0139	0.9390	0.9702	0.0322
LOC649635	0.9899	1.0522	1.0134	0.8253	0.9702	0.0500
SNORD114-1	0.8724	1.1314	0.9475	0.9294	0.9702	0.0561
LOC647042	0.9072	1.1330	0.9494	0.8912	0.9702	0.0556
LY6G5C	0.9113	1.0297	0.9786	0.9611	0.9702	0.0244
LYN	0.7457	1.2753	1.0693	0.7905	0.9702	0.1244
PCDHA10	0.9097	0.9834	1.0271	0.9606	0.9702	0.0244
USP35	0.8756	1.1151	0.9521	0.9380	0.9702	0.0511
LOC646562	0.8897	1.0093	1.1169	0.8649	0.9702	0.0582
LOC646334	0.8654	1.0864	1.0085	0.9206	0.9702	0.0487
LOC646200	0.7082	1.3750	1.1431	0.6546	0.9702	0.1737
APOL4	0.9384	1.0848	0.9245	0.9330	0.9702	0.0383
C18orf16	0.8445	0.9923	1.1095	0.9346	0.9702	0.0555
DNM1	0.9032	1.0765	0.9892	0.9120	0.9702	0.0403
WDR70	0.7144	1.3257	1.1247	0.7161	0.9702	0.1528
LOC729756	0.8663	1.1043	0.9831	0.9272	0.9702	0.0507
RBMY1B	0.9952	0.9396	0.9857	0.9604	0.9702	0.0126

OR2M1P	0.8611	1.0699	1.0207	0.9292	0.9702	0.0466
ERCC-00014	0.9104	1.0150	1.0730	0.8826	0.9702	0.0446
BPESC1	0.9585	1.1977	0.9169	0.8079	0.9702	0.0822
ITGAX	0.9184	1.0519	0.9746	0.9361	0.9702	0.0296
P2RX7	0.9489	1.0744	0.9924	0.8653	0.9702	0.0436
LOC390880	0.9593	0.9490	0.9924	0.9803	0.9702	0.0099
MGC29506	0.8672	1.0856	1.0434	0.8849	0.9703	0.0552
C21orf81	0.8191	1.0508	1.0682	0.9429	0.9703	0.0575
CACNA1E	0.8292	1.1137	1.0533	0.8848	0.9703	0.0675
LOC1001292	0.8563	1.1406	0.9731	0.9111	0.9703	0.0616
LOC1001291	0.8424	1.0623	1.0412	0.9352	0.9703	0.0509
CRLF2	0.9764	1.0492	0.9862	0.8693	0.9703	0.0373
LOC652881	0.8823	1.0297	1.0501	0.9190	0.9703	0.0411
SUV420H1	0.8441	1.3180	0.9287	0.7903	0.9703	0.1194
CHI3L1	0.9252	1.0515	0.9684	0.9361	0.9703	0.0286
LOC440956	0.8719	1.1463	1.0781	0.7849	0.9703	0.0850
LOC644951	0.9292	1.0500	1.0072	0.8948	0.9703	0.0355
PDPN	0.9038	1.1308	0.9336	0.9130	0.9703	0.0539
EIF4A1	0.8055	1.2239	1.0620	0.7899	0.9703	0.1050
LOC1001283	0.9988	0.9674	1.0322	0.8829	0.9703	0.0320
OR10Z1	0.9465	1.0798	0.9542	0.9007	0.9703	0.0383
LOC1001292	0.9289	1.0858	1.0184	0.8481	0.9703	0.0519
PAPPA2	0.9662	1.0112	0.9648	0.9391	0.9703	0.0150
ALPI	0.8857	1.1910	0.9548	0.8498	0.9703	0.0767
LOC643904	0.8918	0.9608	1.0894	0.9394	0.9703	0.0422
NAIP	0.9449	1.0491	0.9930	0.8943	0.9703	0.0331
SPATA1	0.9754	0.9881	0.9532	0.9648	0.9703	0.0074
LOC649395	0.9249	1.0626	0.9872	0.9067	0.9703	0.0353
LOC643768	0.8607	1.0809	1.0613	0.8785	0.9703	0.0584
LOC255649	0.9035	1.0431	0.9794	0.9553	0.9703	0.0290
MYLK3	0.8970	1.0968	1.0273	0.8603	0.9703	0.0553
LOC1001288	0.9780	1.0345	0.9638	0.9051	0.9703	0.0266
IPMK	1.0164	1.1843	0.8775	0.8033	0.9703	0.0839
HTR6	0.9994	1.1105	0.9305	0.8410	0.9704	0.0569
HTRA3	0.8975	1.1091	0.9897	0.8852	0.9704	0.0518
LOC649270	0.9248	1.0762	1.0327	0.8476	0.9704	0.0518
SYNM	0.9610	1.1559	0.9388	0.8257	0.9704	0.0686
LOC642120	0.8696	1.0031	1.1213	0.8874	0.9704	0.0584
NTF4	0.9390	0.9900	1.0153	0.9372	0.9704	0.0194
LOC727970	0.8908	1.1263	1.0311	0.8333	0.9704	0.0665
LOC642178	0.8716	1.0577	0.9651	0.9871	0.9704	0.0384
WISP1	0.9406	1.0714	0.9782	0.8912	0.9704	0.0381
ABCA11	0.8202	1.3464	0.9833	0.7316	0.9704	0.1358
LOC653876	0.8429	1.1851	0.9767	0.8768	0.9704	0.0770
NPR2	0.9327	0.9907	1.0300	0.9282	0.9704	0.0244
USH2A	0.9362	1.0654	0.9842	0.8957	0.9704	0.0365

ISLR	0.9128	1.0459	1.0139	0.9089	0.9704	0.0350
LOC645200	0.9601	1.0101	1.0080	0.9033	0.9704	0.0252
MIR515-2	1.0117	0.9522	1.0374	0.8803	0.9704	0.0349
RHAG	0.9513	1.0276	0.9954	0.9073	0.9704	0.0262
CDR1	0.9549	1.0442	0.9797	0.9028	0.9704	0.0294
RNF208	0.8941	1.1139	0.9388	0.9348	0.9704	0.0489
IGSF11	0.9265	1.0357	0.9711	0.9483	0.9704	0.0236
LOC554226	0.9341	1.0243	1.0517	0.8715	0.9704	0.0415
LOC647713	0.9487	0.9790	1.0259	0.9280	0.9704	0.0213
KLHL34	0.9116	1.0277	1.0821	0.8603	0.9704	0.0511
GDF2	0.9915	0.9872	1.0027	0.9003	0.9704	0.0236
ZFY	0.9627	1.0933	0.9529	0.8728	0.9704	0.0456
LOC730347	0.9159	1.0405	1.0420	0.8834	0.9704	0.0414
LOC1001310	0.9349	0.9896	1.0556	0.9016	0.9704	0.0337
LGALS13	0.9396	1.0521	1.0543	0.8358	0.9704	0.0523
FATE1	0.8820	1.1291	1.0199	0.8508	0.9704	0.0644
LOC1001283	0.9640	0.9598	1.0371	0.9208	0.9704	0.0243
LOC399898	0.9818	1.0270	1.0049	0.8680	0.9704	0.0354
LOC643556	0.8876	1.0902	1.0469	0.8570	0.9705	0.0577
B3GALT2	0.9415	1.0618	0.9263	0.9521	0.9705	0.0309
LOC643702	0.9217	1.0307	1.0335	0.8958	0.9705	0.0360
MAGEA5	0.9126	0.9752	1.0505	0.9434	0.9705	0.0296
PKIG	0.9499	1.0808	0.9000	0.9511	0.9705	0.0387
RIMKLA	0.8890	1.1496	0.8867	0.9566	0.9705	0.0619
ERH	0.6818	1.5062	1.0236	0.6703	0.9705	0.1965
LOC642595	1.0207	1.0973	0.9617	0.8022	0.9705	0.0626
CRHR2	0.9100	1.0852	0.9956	0.8911	0.9705	0.0445
LOC727838	0.9749	0.9909	1.0517	0.8643	0.9705	0.0391
NDUFC2	0.9802	1.0818	1.0545	0.7654	0.9705	0.0716
LOC644209	0.9393	1.0530	0.9874	0.9023	0.9705	0.0326
OR2T12	0.9550	1.0151	1.0335	0.8783	0.9705	0.0350
MT1JP	0.8868	1.0959	0.9446	0.9548	0.9705	0.0444
PLB1	0.9019	1.0462	1.0502	0.8837	0.9705	0.0450
MIR2053	0.9770	1.0331	1.0011	0.8708	0.9705	0.0352
GTPBP5	0.8189	1.1457	1.0600	0.8574	0.9705	0.0788
NEU2	0.9422	1.0091	0.9513	0.9794	0.9705	0.0151
LOC1001343	0.9552	1.0156	1.0441	0.8671	0.9705	0.0391
MS4A13	0.9191	1.0545	0.9422	0.9661	0.9705	0.0296
LOC646055	0.8631	1.0946	0.9526	0.9717	0.9705	0.0477
LOC1001302	0.9521	1.0706	0.9885	0.8709	0.9705	0.0414
UNC45B	0.9312	1.0507	0.9735	0.9268	0.9705	0.0287
CYP2F1	0.9176	1.1228	0.9437	0.8981	0.9705	0.0516
GALT	0.8681	1.3241	0.9515	0.7385	0.9706	0.1257
LOC441644	0.9009	1.0288	1.0334	0.9192	0.9706	0.0351
LOC652353	0.8861	1.0777	0.9485	0.9699	0.9706	0.0399
LOC653354	0.8763	1.3205	0.9189	0.7666	0.9706	0.1210

ABO	1.0074	1.0305	0.9124	0.9320	0.9706	0.0286
CYLD	0.9970	1.0862	0.9902	0.8090	0.9706	0.0581
LOC1001313	0.9447	1.1196	0.9952	0.8228	0.9706	0.0615
LOC1001313	0.8867	1.0640	0.9726	0.9590	0.9706	0.0364
MIR181B1	0.8969	1.0864	0.9406	0.9584	0.9706	0.0407
DMWD	0.7998	1.1999	1.0309	0.8517	0.9706	0.0911
CCDC48	0.9584	1.1489	1.0107	0.7644	0.9706	0.0796
LOC400099	0.9191	1.0276	0.9544	0.9812	0.9706	0.0229
GTF2F1	0.9229	1.1858	0.9134	0.8603	0.9706	0.0730
SPATA16	0.8322	1.1372	0.9482	0.9647	0.9706	0.0629
LOC652667	0.9959	1.0259	1.0394	0.8212	0.9706	0.0506
MRAP	0.8963	1.1108	1.0118	0.8635	0.9706	0.0565
SPRY2	0.9869	1.0304	0.9625	0.9026	0.9706	0.0266
PDXDC2	0.9017	1.1477	0.9756	0.8574	0.9706	0.0639
LOC442240	0.9029	1.0650	0.9794	0.9351	0.9706	0.0352
LOC390424	0.9172	1.0461	0.9898	0.9294	0.9706	0.0297
LOC644335	0.9415	1.1757	0.9351	0.8302	0.9706	0.0730
HAP1	0.9137	1.0747	1.0169	0.8772	0.9706	0.0456
UBE2CBP	0.9336	1.1015	0.9690	0.8784	0.9706	0.0474
GPR155	0.8744	1.0927	1.0181	0.8973	0.9706	0.0515
LOC220115	0.9155	1.0986	0.9881	0.8803	0.9706	0.0482
KDELC2	0.9757	0.9879	1.0170	0.9020	0.9706	0.0245
LOC644248	0.8452	1.0814	1.0314	0.9246	0.9706	0.0531
LOC440820	1.0485	1.0117	0.9065	0.9158	0.9706	0.0352
LOC260339	0.9428	1.0887	1.0071	0.8439	0.9706	0.0517
MAB21L2	0.9457	1.0307	0.9465	0.9596	0.9706	0.0203
PTRF	0.7649	1.3797	0.9381	0.7999	0.9706	0.1414
LOC390806	0.9361	1.0241	1.0621	0.8603	0.9706	0.0453
PTPRCAP	0.9410	1.0115	0.9487	0.9814	0.9706	0.0162
DYX1C1	0.9047	1.0299	1.0692	0.8789	0.9706	0.0465
MIR637	0.9462	1.0914	0.9994	0.8456	0.9706	0.0513
FLJ42133	0.9160	1.0233	1.0299	0.9134	0.9706	0.0323
MIR23A	0.9366	1.0774	0.9396	0.9290	0.9707	0.0357
SLC16A12	0.7894	1.3974	0.9632	0.7326	0.9707	0.1505
KRT18P42	0.9488	1.0374	1.0724	0.8240	0.9707	0.0554
ERCC-00022	0.9555	1.0390	0.9696	0.9186	0.9707	0.0252
PREP	0.7449	1.4520	0.9324	0.7534	0.9707	0.1662
LOC1001302	0.9731	1.0432	0.9442	0.9222	0.9707	0.0263
OR52A5	0.9429	1.0561	0.9882	0.8955	0.9707	0.0342
CLDN14	0.8763	1.1250	1.0472	0.8342	0.9707	0.0690
C3orf57	0.8750	1.0736	1.0236	0.9106	0.9707	0.0467
FLJ39080	0.9422	1.0641	1.0059	0.8706	0.9707	0.0416
ZNF487	0.9569	1.0716	0.9907	0.8636	0.9707	0.0430
LOC731040	0.9520	1.1215	0.9276	0.8817	0.9707	0.0523
INPP5F	0.8769	1.2069	0.9185	0.8806	0.9707	0.0793
LOC442142	0.9992	1.0343	0.8988	0.9505	0.9707	0.0295

PORCN	0.8843	1.0983	0.9795	0.9208	0.9707	0.0468
PKLR	0.9615	1.0060	1.0620	0.8534	0.9707	0.0442
LOC1001309	0.9628	1.0606	1.0015	0.8580	0.9707	0.0426
LOC1001308	0.8672	1.1776	0.9829	0.8553	0.9707	0.0747
ALDH7A1	0.8618	1.1111	1.0362	0.8738	0.9707	0.0614
NBPF1	0.8594	1.0665	1.0821	0.8751	0.9707	0.0599
LOC1001317	1.0200	0.9265	1.0886	0.8478	0.9707	0.0527
SCG2	0.9645	1.0947	1.0225	0.8013	0.9708	0.0624
CCDC144NL	0.9883	1.0911	0.8201	0.9835	0.9708	0.0560
LOC1001324	0.9730	0.9984	0.9832	0.9284	0.9708	0.0150
LOC650961	0.9380	1.0948	0.9191	0.9312	0.9708	0.0415
LILRA5	0.9928	1.0727	0.8954	0.9222	0.9708	0.0397
ACAN	0.8261	1.0502	0.9908	1.0161	0.9708	0.0497
HSPB3	1.0304	1.1172	0.8420	0.8935	0.9708	0.0630
LOC646926	0.9637	1.0590	0.9397	0.9206	0.9708	0.0307
LOC649396	0.8788	1.0909	1.0059	0.9075	0.9708	0.0484
PTGDR	0.8607	1.0497	1.0551	0.9177	0.9708	0.0485
LOC728316	0.9969	1.1733	0.8585	0.8545	0.9708	0.0752
LOC729080	1.0180	1.0400	0.9342	0.8910	0.9708	0.0350
PTPRS	0.8978	1.0746	0.9629	0.9480	0.9708	0.0373
FLJ44112	0.8802	1.1077	1.0077	0.8876	0.9708	0.0542
LOC1001318	0.9865	1.2545	0.8986	0.7436	0.9708	0.1071
ZSWIM5	0.9856	1.0814	0.9813	0.8350	0.9708	0.0508
LOC206227	0.8952	1.0836	0.9793	0.9251	0.9708	0.0414
LOC1001328	0.8964	1.0856	1.0497	0.8516	0.9708	0.0571
LOC285359	0.9052	1.0205	1.0191	0.9384	0.9708	0.0291
FAM153C	0.9443	0.9865	1.0068	0.9456	0.9708	0.0155
LOC644421	0.9773	1.0497	0.9645	0.8918	0.9708	0.0323
LOC442308	0.9437	1.0516	1.0091	0.8789	0.9708	0.0378
PPID	0.9346	1.0416	1.0168	0.8903	0.9708	0.0353
DPP10	0.9703	1.0299	0.9896	0.8934	0.9708	0.0286
ZNF683	0.9254	1.0779	0.9755	0.9045	0.9708	0.0387
TCTE3	0.9599	0.9826	1.0212	0.9196	0.9708	0.0213
FLJ32011	0.8345	1.0016	1.1405	0.9067	0.9708	0.0661
ARPC2	0.8016	1.2376	1.0598	0.7844	0.9708	0.1090
KRT39	0.9638	1.0437	0.9908	0.8850	0.9708	0.0331
LOC399959	0.9819	0.9804	0.9759	0.9451	0.9708	0.0087
LOC1001323	0.8383	1.1345	1.0659	0.8447	0.9708	0.0760
RP11-49G10.	0.9290	1.2223	0.8546	0.8775	0.9709	0.0853
LOC1001324	0.9244	1.0439	0.9823	0.9327	0.9709	0.0275
HAAO	0.9803	1.0995	0.9840	0.8196	0.9709	0.0575
EMILIN3	0.9175	1.0332	0.9854	0.9473	0.9709	0.0250
LOC1001329	0.8667	1.1204	0.9798	0.9167	0.9709	0.0549
LOC646779	1.1120	0.9556	0.9628	0.8532	0.9709	0.0533
LOC646509	0.9321	1.1539	1.0579	0.7396	0.9709	0.0895
BAGE	0.8916	1.1708	0.9805	0.8406	0.9709	0.0726

PLD6	1.0120	0.9951	0.9633	0.9132	0.9709	0.0217
MGAT3	0.9510	0.9864	0.9725	0.9738	0.9709	0.0073
LOC729212	0.9062	1.1310	0.9727	0.8736	0.9709	0.0572
EMCN	0.9737	1.0279	0.9992	0.8828	0.9709	0.0314
LOC1001328	0.8797	0.9895	0.9975	1.0170	0.9709	0.0309
LIN7C	0.8685	1.2261	1.0693	0.7198	0.9709	0.1112
MRAS	0.7358	1.1474	1.1108	0.8897	0.9709	0.0969
ERCC-00163	0.8714	1.1343	0.9482	0.9298	0.9709	0.0569
FBLN7	0.9094	1.1706	0.9977	0.8059	0.9709	0.0772
ITIH4	0.9320	1.0643	0.9496	0.9378	0.9709	0.0314
POU5F2	1.0134	0.9957	1.0046	0.8700	0.9709	0.0338
LOC391135	0.8788	0.9982	1.0793	0.9275	0.9709	0.0437
PIP5KL1	0.9846	0.9698	1.0486	0.8808	0.9709	0.0346
C3orf36	1.0293	1.1061	0.9530	0.7955	0.9710	0.0663
LOC728694	0.8977	1.1551	0.9023	0.9287	0.9710	0.0618
DEFB127	0.9446	0.9283	1.1108	0.9001	0.9710	0.0475
CD200R1	0.9000	1.0312	1.0708	0.8818	0.9710	0.0471
LOC645485	0.9473	1.0168	1.0178	0.9019	0.9710	0.0283
GSTM3	0.9455	1.1118	0.9883	0.8383	0.9710	0.0566
CDC14C	0.7938	1.0867	1.1222	0.8812	0.9710	0.0794
LOC344328	0.8965	1.0871	0.9713	0.9291	0.9710	0.0416
SLFN11	0.8142	1.0612	1.0979	0.9107	0.9710	0.0661
LOC389386	0.9290	1.1909	0.9598	0.8042	0.9710	0.0807
MLF1	0.8768	1.1693	0.9415	0.8964	0.9710	0.0675
LOC652575	0.9369	1.0868	1.0423	0.8180	0.9710	0.0599
SND1	0.8598	1.4323	0.9225	0.6694	0.9710	0.1629
MIR562	0.9030	1.0432	1.0055	0.9323	0.9710	0.0323
CACNA1F	0.9548	1.0650	0.9595	0.9047	0.9710	0.0337
CSMD2	0.8587	1.0995	0.9905	0.9354	0.9710	0.0506
LOC1001335	0.9518	1.0777	0.9550	0.8996	0.9710	0.0378
LOC642520	0.8777	1.0752	1.0070	0.9242	0.9710	0.0438
SLC35E2	1.0042	1.1187	0.9763	0.7849	0.9710	0.0693
SNORD114-4	0.9569	1.1133	0.9544	0.8594	0.9710	0.0526
C3orf14	0.9032	1.0488	0.9944	0.9377	0.9710	0.0320
ADAM5	0.8819	1.0612	1.0250	0.9160	0.9710	0.0429
HELB	0.8727	1.1252	0.9134	0.9730	0.9710	0.0554
LOC647215	0.9564	1.1287	1.0150	0.7840	0.9710	0.0719
LOC400509	0.8868	1.1676	0.9655	0.8643	0.9710	0.0690
HIGD1B	0.9752	0.9583	0.9547	0.9960	0.9710	0.0094
PDLIM2	0.9472	1.0596	1.0435	0.8339	0.9711	0.0520
SHISA3	0.8963	1.1447	1.0077	0.8356	0.9711	0.0680
LOC644519	0.9200	0.9470	1.0960	0.9213	0.9711	0.0421
LCN8	0.9334	1.0591	0.9572	0.9345	0.9711	0.0298
LOC641982	0.9330	1.0743	1.0482	0.8288	0.9711	0.0565
MRGPRX2	0.9329	1.1044	0.9411	0.9059	0.9711	0.0451
LOC283682	0.9763	1.0629	0.9295	0.9157	0.9711	0.0332

LOC643331	0.8565	1.1332	1.0379	0.8567	0.9711	0.0689
KLF16	1.0365	0.9870	0.9623	0.8985	0.9711	0.0287
DNAJC30	0.7976	1.2895	0.9849	0.8124	0.9711	0.1143
MTMR7	0.9685	1.1068	0.9233	0.8859	0.9711	0.0483
SUMO1	1.0054	1.0186	0.9812	0.8792	0.9711	0.0316
ZNF502	0.9890	1.1087	0.9835	0.8033	0.9711	0.0630
LOC648629	0.9492	1.1067	0.9634	0.8652	0.9711	0.0501
KRT2	0.9691	0.9595	1.0543	0.9016	0.9711	0.0315
LOC651454	1.0214	1.0227	0.9550	0.8855	0.9711	0.0326
NHS	0.9472	1.0657	0.9854	0.8862	0.9711	0.0376
LOC644619	0.9045	1.1804	0.9885	0.8112	0.9711	0.0786
WDR1	0.8997	1.3276	1.0082	0.6491	0.9711	0.1406
LOC651115	0.8574	1.1593	0.9659	0.9020	0.9712	0.0666
NT5C1B	0.9490	1.0965	0.9769	0.8623	0.9712	0.0484
ABCC9	0.9143	1.1287	0.9955	0.8462	0.9712	0.0607
SNORD11	0.9957	1.0876	0.9988	0.8026	0.9712	0.0601
C3orf42	0.9190	1.0661	0.9550	0.9445	0.9712	0.0325
LOC1001328	0.8760	1.0206	1.0101	0.9780	0.9712	0.0330
NAPSB	0.9657	0.9490	1.0833	0.8866	0.9712	0.0411
A1CF	0.9054	1.0555	0.9999	0.9240	0.9712	0.0347
C2orf55	0.9172	1.0153	0.9998	0.9524	0.9712	0.0224
PAX3	0.9174	1.0006	1.0563	0.9103	0.9712	0.0350
LOC646825	0.8417	1.0234	0.9924	1.0272	0.9712	0.0438
MIR154	0.9768	1.0785	0.9780	0.8514	0.9712	0.0465
LRRC8C	0.9803	0.9601	1.0007	0.9437	0.9712	0.0124
CYP2S1	0.9051	1.0483	0.9771	0.9543	0.9712	0.0298
SMTN	1.0083	1.0879	0.9525	0.8361	0.9712	0.0529
LOC1001315	0.8881	1.0510	0.9776	0.9681	0.9712	0.0333
ERMN	0.9513	1.0209	0.9280	0.9846	0.9712	0.0202
AHSP	0.8517	1.1461	1.0379	0.8492	0.9712	0.0732
LOC388503	0.9640	0.9814	1.0300	0.9094	0.9712	0.0249
LOC728847	0.8638	1.0492	1.0692	0.9026	0.9712	0.0516
VHLL	0.9309	1.0001	1.0004	0.9535	0.9712	0.0174
LOC650147	0.9062	1.0340	1.0605	0.8842	0.9712	0.0444
SNED1	0.9784	1.0519	0.9511	0.9035	0.9712	0.0310
LOC1001332	0.9579	1.1080	0.9220	0.8970	0.9712	0.0473
CD300E	0.9200	1.1607	0.9750	0.8292	0.9712	0.0700
MGC16025	0.9612	1.0432	0.9380	0.9426	0.9712	0.0245
NPY1R	1.0228	1.0638	0.8823	0.9160	0.9712	0.0430
SH2D7	0.9783	1.0561	1.0017	0.8489	0.9712	0.0439
C10orf72	0.8950	1.1064	0.9540	0.9296	0.9712	0.0466
LOC645032	0.9566	1.0283	0.9695	0.9307	0.9713	0.0207
MIR1265	0.9195	1.0961	0.9370	0.9325	0.9713	0.0418
LOC1001301	1.0315	1.0268	0.9486	0.8781	0.9713	0.0364
PTGR2	0.8829	1.2006	0.9340	0.8675	0.9713	0.0778
LOC729260	0.8589	0.9429	1.1164	0.9669	0.9713	0.0536

LOC645681	0.8767	0.9878	1.1262	0.8944	0.9713	0.0571
LOC648256	0.9747	1.0855	0.9903	0.8346	0.9713	0.0517
PHF20L1	0.9503	1.1517	0.9130	0.8700	0.9713	0.0624
SMAD9	0.9614	0.9871	1.0652	0.8715	0.9713	0.0399
NBPF9	0.9613	1.0979	0.9580	0.8679	0.9713	0.0474
RASIP1	0.8068	1.1263	1.1491	0.8029	0.9713	0.0962
MIR2278	0.7707	1.2270	1.0224	0.8650	0.9713	0.0998
LOC645941	1.0003	1.0989	0.8812	0.9047	0.9713	0.0497
CNTN1	0.9729	1.0835	0.9422	0.8866	0.9713	0.0414
KLK4	0.7515	1.3248	0.9825	0.8263	0.9713	0.1273
TRO	0.8888	1.0967	1.0356	0.8640	0.9713	0.0564
CAV3	0.9490	1.0054	0.9876	0.9433	0.9713	0.0150
LOC1001341	0.8826	1.0802	1.0253	0.8971	0.9713	0.0484
LOC643576	1.0589	0.9940	0.9950	0.8374	0.9713	0.0472
ISM2	0.9194	1.1932	0.8674	0.9053	0.9713	0.0748
MAP1D	0.9474	1.1287	0.9656	0.8435	0.9713	0.0590
FAM151B	0.8353	1.2324	1.0021	0.8154	0.9713	0.0966
KCNJ3	0.9095	1.0150	1.0492	0.9114	0.9713	0.0358
LOC730074	0.8088	1.3467	1.0439	0.6859	0.9713	0.1455
RASL10B	1.0474	1.0358	0.9189	0.8832	0.9713	0.0413
AQP7	0.9475	1.0097	0.9650	0.9631	0.9713	0.0134
HDC	0.9169	0.9940	1.0731	0.9013	0.9713	0.0395
LOC642017	0.7284	1.3380	0.7806	1.0384	0.9713	0.1397
CCDC63	0.8915	1.1049	0.9828	0.9061	0.9713	0.0488
LOC729078	0.9972	1.0200	1.0268	0.8414	0.9713	0.0438
CASP8AP2	0.8882	1.0755	1.0150	0.9067	0.9713	0.0446
OSBPL11	0.9030	1.1070	0.9876	0.8878	0.9713	0.0503
KIAA1024L	0.8624	1.1160	0.9294	0.9775	0.9714	0.0537
LRRC8E	0.8440	1.0389	1.1553	0.8471	0.9714	0.0764
LOC1001296	0.9659	1.0424	0.9159	0.9613	0.9714	0.0262
LOC651463	0.9077	1.0787	0.9793	0.9196	0.9714	0.0391
LIPE	0.9537	1.0689	0.9512	0.9116	0.9714	0.0339
MIR125B2	0.8426	1.0958	1.1313	0.8157	0.9714	0.0826
FSHB	0.9184	1.1115	0.9595	0.8961	0.9714	0.0485
SUSD1	0.9318	0.9931	1.0610	0.8996	0.9714	0.0356
TMSL3	0.9970	1.0904	1.0726	0.7255	0.9714	0.0844
SNORD111	0.9698	1.0260	1.0031	0.8865	0.9714	0.0305
PYGO1	0.8710	1.0460	1.0037	0.9649	0.9714	0.0373
SNORD71	0.9187	0.9889	1.0396	0.9383	0.9714	0.0271
LOC391656	0.8917	1.1265	1.2124	0.6550	0.9714	0.1254
LOC644155	0.8701	1.1206	0.9241	0.9708	0.9714	0.0538
CC2D2B	0.9260	1.0037	1.0148	0.9411	0.9714	0.0222
LOC653539	0.9004	1.0687	0.9888	0.9276	0.9714	0.0373
LOC644536	0.8510	1.1331	1.0125	0.8890	0.9714	0.0640
LOC643932	1.1017	1.1100	0.9031	0.7708	0.9714	0.0822
SLAMF8	0.9186	1.1509	0.9122	0.9040	0.9714	0.0599

LOC440061	0.8691	1.0720	1.0458	0.8988	0.9714	0.0512
LOC399937	0.9185	1.0629	1.0233	0.8809	0.9714	0.0429
SCXA	0.9021	1.1849	0.9480	0.8507	0.9714	0.0739
FAM115B	0.8412	1.1231	1.0339	0.8875	0.9714	0.0651
KBTBD12	0.8705	1.0186	1.0698	0.9269	0.9714	0.0448
LOC727914	0.8955	1.1564	1.0024	0.8313	0.9714	0.0711
SETD7	0.8731	1.1066	1.0794	0.8266	0.9714	0.0711
ZNF197	0.9093	1.1708	1.0069	0.7988	0.9715	0.0789
LOC730818	0.8333	1.0904	1.0460	0.9161	0.9715	0.0591
LOC647542	0.9602	1.0033	0.9767	0.9457	0.9715	0.0124
FAM26E	0.8168	1.2019	0.9785	0.8887	0.9715	0.0836
OR6F1	0.9118	1.0989	1.0410	0.8342	0.9715	0.0602
FCGRT	0.9378	1.0442	1.0296	0.8742	0.9715	0.0401
SSTR3	0.9508	1.0831	0.9405	0.9115	0.9715	0.0381
DCXR	0.8274	1.2878	1.0079	0.7629	0.9715	0.1175
LOC644150	0.9790	1.0087	1.0372	0.8611	0.9715	0.0387
LOC644982	0.9441	1.0113	0.9910	0.9396	0.9715	0.0176
TRMT2A	0.8706	1.1118	1.0453	0.8582	0.9715	0.0633
LOC645551	0.9423	1.0469	0.9861	0.9107	0.9715	0.0295
LOC402715	1.0317	1.1224	0.8945	0.8374	0.9715	0.0647
HLX	0.8749	1.0977	1.1023	0.8111	0.9715	0.0753
LOC728364	0.9509	1.0525	1.0073	0.8753	0.9715	0.0382
LOC1001328	0.9414	1.0698	1.0877	0.7871	0.9715	0.0696
SRRM5	0.8795	1.1018	1.0548	0.8499	0.9715	0.0627
LOC283029	1.0304	1.0109	0.9908	0.8540	0.9715	0.0400
AGAP11	1.0134	0.9526	1.0073	0.9128	0.9715	0.0239
LOC653588	0.9061	1.1351	1.0283	0.8165	0.9715	0.0697
LOC1001317	0.7330	1.1283	1.0341	0.9907	0.9715	0.0845
LOC1001295	0.9279	1.0074	1.0487	0.9020	0.9715	0.0341
DGKG	1.0021	1.0157	0.9771	0.8912	0.9715	0.0279
CYP2U1	0.9850	1.0916	0.9988	0.8108	0.9715	0.0586
ORM2	1.1790	0.9986	0.9140	0.7945	0.9715	0.0808
AGER	0.9688	1.0727	0.9749	0.8697	0.9715	0.0415
LOC441052	0.9415	1.0652	0.9180	0.9614	0.9715	0.0325
PCBP2	0.7920	1.3042	0.9994	0.7906	0.9715	0.1213
ZBTB6	0.9355	1.1621	0.9839	0.8047	0.9715	0.0739
LOC653148	0.7815	1.0658	1.0595	0.9793	0.9716	0.0663
CCT8L1	0.9530	1.1046	0.9784	0.8502	0.9716	0.0523
TIMP4	1.0237	1.0002	0.9452	0.9172	0.9716	0.0245
LOC650823	0.9042	1.0196	1.0287	0.9337	0.9716	0.0310
TMEM84	0.9425	0.9541	0.9961	0.9935	0.9716	0.0136
LOC442572	0.9893	1.0950	0.9485	0.8534	0.9716	0.0500
LOC1001302	0.9213	1.1557	0.9721	0.8372	0.9716	0.0674
MIR623	0.8110	1.1992	0.9950	0.8811	0.9716	0.0848
LOC1001331	0.9829	1.0385	0.9274	0.9375	0.9716	0.0254
LOC344167	0.9137	1.1640	0.9551	0.8534	0.9716	0.0675

GPR45	0.9949	0.9423	1.0533	0.8958	0.9716	0.0339
ACSS1	0.9984	0.9997	1.1095	0.7787	0.9716	0.0694
LOC648025	0.9590	0.9565	1.0723	0.8986	0.9716	0.0363
C1orf133	1.0188	1.0452	0.9439	0.8785	0.9716	0.0377
FLJ16793	0.9010	1.1689	0.9038	0.9126	0.9716	0.0658
SCARNA12	0.9049	1.1520	1.0123	0.8172	0.9716	0.0722
SELO	0.8780	1.2182	1.0115	0.7787	0.9716	0.0950
LOC653596	0.9702	1.0474	0.9738	0.8951	0.9716	0.0311
UCN2	0.9584	1.0114	1.0472	0.8694	0.9716	0.0386
ERCC-00145	0.9970	1.0083	0.9559	0.9252	0.9716	0.0191
GTSF1L	0.8967	1.0861	0.9405	0.9632	0.9716	0.0406
LOC441752	0.9951	1.0087	1.0208	0.8619	0.9716	0.0369
CD33	0.8977	1.0880	0.9484	0.9524	0.9716	0.0408
MFSD7	1.0778	1.0386	0.9501	0.8201	0.9716	0.0572
WFIKKN1	0.9208	1.0238	0.9740	0.9680	0.9716	0.0211
LOC649990	0.9056	1.2415	0.9550	0.7844	0.9716	0.0968
SH3TC2	0.9668	1.0574	0.9483	0.9140	0.9716	0.0306
SNORA36B	0.8499	1.0020	1.0434	0.9912	0.9716	0.0421
RPL27	0.8669	1.0205	1.1286	0.8705	0.9716	0.0634
LOC1001338	0.8688	1.0867	1.0682	0.8629	0.9716	0.0612
LOC728773	0.9286	1.0617	1.0333	0.8630	0.9716	0.0462
FGG	0.8855	1.0215	1.0411	0.9385	0.9716	0.0363
FLJ36644	0.8661	1.0597	1.0548	0.9061	0.9717	0.0501
LOC1001342	0.9924	1.0393	1.0121	0.8429	0.9717	0.0440
SERHL	0.9506	1.0203	1.0367	0.8790	0.9717	0.0361
LOC642093	0.9186	1.0005	1.0938	0.8737	0.9717	0.0485
C6orf223	0.9018	1.0079	1.0356	0.9413	0.9717	0.0306
MLL4	1.0124	1.2101	0.7884	0.8758	0.9717	0.0919
LOC729127	0.9156	1.1255	0.9776	0.8681	0.9717	0.0559
APOL2	0.9219	1.2081	0.9448	0.8120	0.9717	0.0840
LOC646145	0.7914	1.0834	1.0800	0.9319	0.9717	0.0697
LOC650744	0.9124	1.0359	1.0353	0.9032	0.9717	0.0369
LOC402207	0.9662	1.0122	1.0617	0.8467	0.9717	0.0460
MIR644	0.8856	1.0547	1.0063	0.9401	0.9717	0.0371
LOC732226	0.8849	1.0125	0.9739	1.0154	0.9717	0.0304
HUS1	0.8185	1.1405	1.0358	0.8921	0.9717	0.0721
GJB4	0.8607	1.2192	0.9542	0.8527	0.9717	0.0857
LOC731895	0.8511	1.1253	1.0549	0.8556	0.9717	0.0698
TMEM101	0.7416	1.3935	1.0311	0.7206	0.9717	0.1574
LOC729141	0.8500	1.0207	1.0921	0.9241	0.9717	0.0532
PEX5	0.7565	1.4460	0.9736	0.7108	0.9717	0.1682
CEP164	0.8692	1.1261	0.9417	0.9499	0.9717	0.0546
OR13C2	0.9687	1.0146	0.9644	0.9393	0.9717	0.0157
LOC644090	1.0480	1.0510	0.9148	0.8731	0.9717	0.0457
PSORS1C1	0.8743	1.0005	1.1815	0.8307	0.9717	0.0786
ZFP41	0.9142	1.0104	1.0334	0.9288	0.9717	0.0295

LOC1001338	0.8361	1.0706	1.0328	0.9475	0.9717	0.0520
LOC139116	0.9947	1.0295	1.0093	0.8533	0.9717	0.0401
LOC644711	0.9181	1.1290	0.9929	0.8469	0.9717	0.0603
ZNF99	0.8467	1.1406	1.0605	0.8392	0.9717	0.0762
PSG5	0.9365	1.0641	0.9141	0.9722	0.9717	0.0330
SNORA38B	0.8903	0.9502	1.0656	0.9809	0.9717	0.0365
TJP2	0.8446	1.1801	0.9841	0.8782	0.9717	0.0755
LOC643133	0.9622	1.1295	1.0132	0.7821	0.9717	0.0722
SF3B14	0.9100	1.3636	0.9766	0.6368	0.9717	0.1499
IL17RA	0.9526	1.1552	0.9417	0.8375	0.9718	0.0664
FAM22E	0.9227	1.0160	1.0430	0.9054	0.9718	0.0340
RUFY2	0.8951	1.1032	1.0059	0.8828	0.9718	0.0518
LOC650881	0.8448	1.0201	1.0506	0.9716	0.9718	0.0453
LOC401480	0.9186	0.9701	1.0708	0.9276	0.9718	0.0349
TM6SF2	0.9638	1.0522	0.9607	0.9104	0.9718	0.0295
LOC1001290	0.8918	1.0471	1.0089	0.9393	0.9718	0.0348
MYO3A	0.9752	1.0440	0.9494	0.9185	0.9718	0.0267
RBBP5	0.8269	1.2376	1.0233	0.7994	0.9718	0.1017
CKAP4	0.8762	1.4352	0.8614	0.7144	0.9718	0.1587
CDADC1	0.9149	1.0366	1.0341	0.9017	0.9718	0.0368
TAL1	1.0369	1.0255	0.9549	0.8699	0.9718	0.0385
LOC643599	0.8643	1.1139	1.0460	0.8630	0.9718	0.0639
COCH	0.9683	1.0824	0.9966	0.8400	0.9718	0.0502
LOC646208	0.9539	0.9954	1.0832	0.8548	0.9718	0.0474
MGC23270	0.9272	1.0372	1.0024	0.9204	0.9718	0.0286
MEGF11	0.7985	1.0683	1.0525	0.9679	0.9718	0.0618
LOC645662	1.0630	0.9859	0.9314	0.9070	0.9718	0.0346
MICALL2	1.0107	0.9924	1.0067	0.8775	0.9718	0.0317
COMMD10	0.9030	1.2534	1.0286	0.7023	0.9718	0.1154
LOR	0.8727	1.1267	0.9673	0.9206	0.9718	0.0551
PHF21B	0.9841	0.9751	0.9727	0.9554	0.9718	0.0060
LOC646304	0.9233	1.1171	0.9516	0.8954	0.9719	0.0498
ZNF407	0.9334	1.1032	0.9710	0.8798	0.9719	0.0476
LOC440334	0.9748	0.9831	0.9703	0.9592	0.9719	0.0050
CCDC11	0.9204	1.0275	1.0503	0.8892	0.9719	0.0395
GRAMD1B	0.8483	1.0664	1.0001	0.9726	0.9719	0.0457
LOC149620	0.8407	1.1566	1.0169	0.8733	0.9719	0.0725
LOC646332	1.0410	1.0742	0.9080	0.8642	0.9719	0.0508
MTSS1L	1.0407	1.0918	0.8844	0.8706	0.9719	0.0556
MIR126	0.9384	1.0365	0.9710	0.9416	0.9719	0.0228
RNASEN	0.7597	1.4509	0.9944	0.6825	0.9719	0.1729
LOC732156	0.9829	1.0977	0.9832	0.8237	0.9719	0.0563
HSF2BP	0.8272	1.0182	1.1535	0.8887	0.9719	0.0724
LOC728728	0.9741	1.0463	0.9680	0.8991	0.9719	0.0301
KLC3	0.8906	1.0429	1.0708	0.8832	0.9719	0.0494
LOC645349	1.0043	1.0261	0.9920	0.8651	0.9719	0.0363

LOC646243	0.9130	1.0355	1.0731	0.8660	0.9719	0.0491
IL17D	0.8476	1.1016	0.9822	0.9561	0.9719	0.0521
C6orf54	0.9042	1.1701	0.9406	0.8726	0.9719	0.0675
LOC647038	0.9284	1.0006	1.0923	0.8662	0.9719	0.0486
TENC1	0.9316	1.0744	1.0095	0.8721	0.9719	0.0443
LOC652865	0.8703	1.0385	0.9838	0.9950	0.9719	0.0359
HOXA6	0.8129	1.1645	0.9959	0.9142	0.9719	0.0743
TTC13	0.7815	1.3787	0.9970	0.7304	0.9719	0.1474
LOC644964	0.8866	0.9935	1.0313	0.9762	0.9719	0.0307
PAX7	1.0214	1.0464	0.9522	0.8676	0.9719	0.0401
FLJ45831	0.8557	1.0218	1.0401	0.9701	0.9719	0.0415
SYNJ2BP	0.8160	1.2874	1.0085	0.7759	0.9719	0.1168
FGD5	0.9091	1.0786	0.9622	0.9379	0.9719	0.0372
STAB2	0.9211	1.0094	1.0280	0.9293	0.9719	0.0273
HTR1A	0.9871	1.0169	1.0284	0.8554	0.9719	0.0398
HTR5A	0.9334	1.0037	1.0696	0.8810	0.9719	0.0411
MGC42630	0.8563	1.1126	0.9268	0.9920	0.9719	0.0544
LOC645946	0.9491	0.9833	1.0043	0.9511	0.9719	0.0133
LOC1001309	0.8938	1.1179	0.9420	0.9341	0.9719	0.0498
MIR377	0.8833	1.0821	1.0551	0.8673	0.9719	0.0562
LOC440287	1.0059	1.1191	0.8614	0.9014	0.9720	0.0577
CXorf24	0.8997	1.1069	1.0777	0.8035	0.9720	0.0724
MIR1286	1.0136	1.0538	0.9945	0.8260	0.9720	0.0502
LOC732272	0.8784	1.0786	0.9756	0.9553	0.9720	0.0413
KIAA1377	1.0287	1.0376	0.9841	0.8374	0.9720	0.0464
PCDHB16	0.9567	1.0390	0.9985	0.8937	0.9720	0.0310
SUZ12P	0.9020	1.0780	1.0214	0.8865	0.9720	0.0465
MIR628	0.9838	1.0227	1.0714	0.8099	0.9720	0.0569
LOC729199	0.9373	1.0562	0.9636	0.9308	0.9720	0.0290
LOC1001335	1.0354	0.9960	0.9679	0.8886	0.9720	0.0311
USP15	0.9441	1.1375	1.0641	0.7422	0.9720	0.0863
EGFL10	0.9808	1.0150	0.9818	0.9103	0.9720	0.0220
LOC729025	0.7728	1.1018	1.0710	0.9423	0.9720	0.0748
IRAK4	0.9387	1.1594	0.9492	0.8407	0.9720	0.0671
SLC8A2	0.8955	1.0302	1.0470	0.9154	0.9720	0.0388
LOC401398	0.9567	1.1683	0.9871	0.7759	0.9720	0.0803
TXNDC14	0.8137	1.3281	1.0826	0.6636	0.9720	0.1470
LOC1001315	0.8640	1.0901	1.0307	0.9033	0.9720	0.0531
LOC1001304	0.9564	1.1214	0.9589	0.8514	0.9720	0.0557
KIF13A	0.9714	1.0687	0.8866	0.9613	0.9720	0.0374
XK	0.9210	1.0014	1.0335	0.9321	0.9720	0.0272
LOC1001314	0.7866	1.1260	1.1191	0.8564	0.9720	0.0881
AZU1	0.9751	0.9531	1.0116	0.9484	0.9720	0.0144
LOC1001327	0.7890	1.2327	1.1951	0.6713	0.9720	0.1419
SNORD72	0.9664	1.1016	0.9468	0.8733	0.9720	0.0476
FSHR	0.9781	1.0175	0.9662	0.9263	0.9720	0.0188

SMEK1	0.8435	1.0827	1.0342	0.9278	0.9720	0.0537
SBSN	0.9910	1.0405	0.9623	0.8944	0.9720	0.0305
RILPL2	0.8373	1.4145	0.9293	0.7070	0.9720	0.1544
CD3E	0.9754	1.0881	0.9311	0.8935	0.9720	0.0422
FAM63B	0.9479	0.9734	1.0903	0.8766	0.9720	0.0444
TNMD	0.9008	0.9945	0.9228	1.0701	0.9721	0.0383
MIR216A	0.9510	1.0759	0.9694	0.8919	0.9721	0.0383
MYLC2PL	0.9488	1.0480	0.9370	0.9545	0.9721	0.0256
NMD3	0.9065	1.2137	1.0652	0.7028	0.9721	0.1095
LOC401296	0.8775	1.1414	0.9611	0.9081	0.9721	0.0590
LOC643825	0.8794	1.0274	1.0398	0.9416	0.9721	0.0378
CLDN6	0.9493	0.9554	1.0406	0.9429	0.9721	0.0230
RGS18	0.9153	0.9847	0.9920	0.9963	0.9721	0.0191
STAR	0.9052	1.0902	0.9651	0.9277	0.9721	0.0413
LOC729948	0.8885	1.1242	0.9682	0.9074	0.9721	0.0535
OR7C2	0.8829	1.1933	0.9623	0.8498	0.9721	0.0774
CHRM4	0.9342	1.0531	0.9852	0.9158	0.9721	0.0307
LOC1001304	0.9169	1.0044	1.0444	0.9227	0.9721	0.0313
LOC646713	0.8574	1.0197	1.1214	0.8899	0.9721	0.0609
FEZF1	1.0249	1.0442	0.9472	0.8720	0.9721	0.0394
HTATSF1	0.9989	1.0239	1.0520	0.8136	0.9721	0.0539
ATN1	0.9499	1.1354	0.9609	0.8422	0.9721	0.0607
C21orf99	0.8845	1.1218	1.0361	0.8461	0.9721	0.0646
LOC1001341	0.8663	1.0682	1.0412	0.9128	0.9721	0.0489
LOC651558	0.8523	1.1393	1.0048	0.8920	0.9721	0.0644
POTEB	0.9503	1.0041	1.0267	0.9073	0.9721	0.0269
CTRB1	0.9635	0.9804	1.1021	0.8425	0.9721	0.0531
LUC7L	0.8823	1.1866	0.9969	0.8227	0.9721	0.0801
VCP	0.7838	1.3294	1.0046	0.7707	0.9721	0.1306
MIR613	0.9289	0.9923	0.9967	0.9707	0.9721	0.0155
FLJ90680	0.9618	1.1087	0.9446	0.8735	0.9721	0.0494
OR5K2	0.9344	0.9716	1.0403	0.9423	0.9721	0.0241
LOC1001316	0.9595	1.0695	1.0023	0.8572	0.9721	0.0445
RPS6KA3	0.8851	1.1310	1.0151	0.8575	0.9722	0.0631
OR1F1	0.8617	1.0435	1.0581	0.9252	0.9722	0.0473
TSPAN17	0.8789	1.2951	0.9607	0.7540	0.9722	0.1157
LOC646879	0.8662	1.1145	1.0488	0.8592	0.9722	0.0646
LOC649162	0.8189	1.0283	1.1192	0.9222	0.9722	0.0650
LOC388572	0.9494	1.0475	0.9797	0.9121	0.9722	0.0287
ITGBL1	0.8631	1.1230	1.0034	0.8993	0.9722	0.0584
NR4A1	0.9732	1.0649	0.9668	0.8838	0.9722	0.0370
LCE1B	0.9273	1.1533	0.9784	0.8297	0.9722	0.0678
KCTD4	0.9755	0.9518	0.9907	0.9708	0.9722	0.0080
BCAM	0.9371	1.0608	0.9590	0.9318	0.9722	0.0301
MIR499	0.9272	0.9795	1.0292	0.9529	0.9722	0.0218
LOC1001338	0.9827	0.9731	1.0247	0.9083	0.9722	0.0241

CART1	0.9434	1.0559	1.0520	0.8375	0.9722	0.0519
LOC652704	0.9000	1.0953	0.9893	0.9043	0.9722	0.0459
LOC440525	0.7486	1.0990	1.1356	0.9056	0.9722	0.0900
HPGDS	0.9548	1.1305	0.9318	0.8717	0.9722	0.0556
LOC650543	1.0008	1.0555	0.9165	0.9160	0.9722	0.0342
FLJ45684	0.8919	1.0650	1.0451	0.8869	0.9722	0.0480
IL5RA	1.0216	1.0458	1.0028	0.8187	0.9722	0.0519
LOC729759	0.9847	1.0250	0.9663	0.9129	0.9722	0.0233
C16orf71	1.0247	1.0261	1.0203	0.8179	0.9722	0.0515
LOC1001290	0.8799	1.0584	1.0122	0.9384	0.9722	0.0395
LOC646207	0.9880	0.9177	1.0690	0.9143	0.9722	0.0364
IGFBP7	0.8674	1.1135	1.0156	0.8924	0.9722	0.0572
LOC646903	0.9291	0.9517	1.0355	0.9726	0.9723	0.0229
CCL3L3	1.0406	0.9861	0.9083	0.9539	0.9723	0.0278
FAM26C	0.9185	1.0235	1.0098	0.9373	0.9723	0.0261
SPRN	0.8177	1.2364	0.9635	0.8716	0.9723	0.0930
FLJ12078	0.9121	1.2410	0.8887	0.8473	0.9723	0.0906
BTRC	0.9025	1.1016	1.0128	0.8722	0.9723	0.0526
SNORA74A	0.8497	1.0928	1.0282	0.9184	0.9723	0.0545
LOC646576	0.9178	0.9703	1.0334	0.9676	0.9723	0.0237
IMMP1L	0.8988	1.0275	1.0585	0.9043	0.9723	0.0413
LOC650770	0.9744	1.0051	0.9826	0.9272	0.9723	0.0164
TTC22	0.9338	1.1147	1.0171	0.8237	0.9723	0.0618
FLJ32810	0.9882	1.0288	0.9678	0.9043	0.9723	0.0260
LOC642506	0.9851	0.9675	1.0289	0.9077	0.9723	0.0251
OR2L13	0.8542	1.0674	1.0417	0.9261	0.9723	0.0500
SPAG11B	0.8962	1.0684	0.9938	0.9309	0.9723	0.0379
CCNB3	0.9275	1.0743	1.0014	0.8862	0.9723	0.0415
ERO1LB	0.8915	1.2746	0.9127	0.8105	0.9723	0.1031
DAD1L	0.9034	1.0883	0.9943	0.9034	0.9723	0.0442
LOC644952	0.9066	0.9913	1.0131	0.9783	0.9724	0.0231
PYHIN1	0.9407	1.0293	1.0249	0.8945	0.9724	0.0330
PRF1	0.8342	1.1486	0.9756	0.9310	0.9724	0.0657
OR5M10	0.8612	1.1558	1.0110	0.8614	0.9724	0.0706
LOC728470	0.8499	0.9705	1.0819	0.9871	0.9724	0.0476
LOC652565	0.9058	1.0570	1.0356	0.8911	0.9724	0.0430
ZFP14	0.9420	1.1055	0.9732	0.8688	0.9724	0.0495
LOC339674	0.9637	0.9998	0.9947	0.9313	0.9724	0.0159
LOC645590	0.8987	1.0481	1.0521	0.8906	0.9724	0.0449
LOC650759	0.8445	1.1736	0.9991	0.8724	0.9724	0.0750
C7orf66	0.9512	1.0903	0.9571	0.8910	0.9724	0.0420
LOC338805	0.9674	1.0252	1.0380	0.8590	0.9724	0.0408
FAM124A	0.9174	1.0625	1.0404	0.8693	0.9724	0.0469
OTOR	0.9210	1.0459	1.0053	0.9174	0.9724	0.0318
SALL2	0.9334	1.2711	0.8866	0.7985	0.9724	0.1034
LOC1001346	0.9827	1.0881	0.9712	0.8477	0.9724	0.0492

LOC646864	1.0045	1.0675	0.9239	0.8938	0.9724	0.0394
LOC1001332	0.8688	1.0077	1.0455	0.9676	0.9724	0.0380
LOC390205	0.8765	1.0640	1.0115	0.9378	0.9724	0.0411
LOC649330	0.8392	1.1170	0.9663	0.9672	0.9724	0.0568
WASL	0.9012	1.3092	0.9464	0.7328	0.9724	0.1213
LOC345051	0.8010	1.1058	1.0551	0.9279	0.9724	0.0683
LOC728224	0.9164	1.0714	1.0474	0.8545	0.9724	0.0520
FLJ22447	0.8999	1.0487	1.0471	0.8941	0.9724	0.0436
PMCHL1	0.8783	1.1835	0.8899	0.9380	0.9724	0.0715
IL29	0.9325	1.0143	1.0347	0.9082	0.9724	0.0308
LOC731082	0.8583	0.9434	1.1045	0.9837	0.9724	0.0512
LOC1001303	0.8100	1.0753	1.0758	0.9287	0.9724	0.0643
LOC1001282	0.9209	1.0792	1.0013	0.8884	0.9724	0.0428
LOC649456	0.9699	0.9989	0.9571	0.9639	0.9725	0.0092
RAB11FIP1	0.8704	1.3109	0.9551	0.7534	0.9725	0.1202
LOC1001280	0.9398	1.0268	0.9782	0.9451	0.9725	0.0200
NEUROD1	0.9143	1.0686	0.9845	0.9225	0.9725	0.0357
CPLX2	0.8885	1.0710	0.9908	0.9395	0.9725	0.0389
LOC729231	0.9366	1.0577	0.9864	0.9092	0.9725	0.0326
LOC650810	0.9841	1.0312	0.9923	0.8823	0.9725	0.0318
LOC651113	0.9442	1.1923	0.8935	0.8599	0.9725	0.0753
LOC652258	0.9604	1.0214	0.9904	0.9178	0.9725	0.0221
LOC1001328	0.9191	1.0415	0.9889	0.9405	0.9725	0.0272
EHD4	0.8913	1.4046	0.9489	0.6452	0.9725	0.1584
HSF4	0.8970	1.1446	1.0199	0.8286	0.9725	0.0697
LOC728946	0.9110	1.0377	1.0363	0.9049	0.9725	0.0373
DGCR14	0.8925	1.2260	0.8960	0.8755	0.9725	0.0846
PCDHA6	0.9087	1.0090	1.0594	0.9129	0.9725	0.0371
LOC644098	0.8086	1.0392	1.0560	0.9863	0.9725	0.0566
MIR210	0.9366	1.0591	1.0083	0.8862	0.9725	0.0382
EPHB1	0.9320	1.0597	1.0026	0.8959	0.9725	0.0365
RXRB	0.8814	1.3350	0.9163	0.7574	0.9725	0.1255
TNFRSF11A	0.8802	1.1313	0.9747	0.9039	0.9725	0.0566
MFSD2B	0.9907	1.0180	0.9512	0.9303	0.9725	0.0196
SOX7	0.8810	1.0059	1.0871	0.9162	0.9725	0.0463
LOC390282	0.9459	1.0616	0.9952	0.8875	0.9725	0.0370
LOC646933	0.9218	1.1592	1.0451	0.7640	0.9725	0.0847
LOC286094	0.9941	1.0033	0.8791	1.0138	0.9726	0.0314
PRKCQ	0.9016	1.1044	1.0053	0.8790	0.9726	0.0518
CDC16	0.8055	1.3771	0.9358	0.7719	0.9726	0.1394
ZNF22	0.8814	1.0293	1.0836	0.8959	0.9726	0.0498
LOC387762	0.9367	1.0602	1.0116	0.8818	0.9726	0.0395
PACAP	0.9081	1.0131	1.0811	0.8880	0.9726	0.0454
KRT126P	0.9321	1.1085	0.9867	0.8630	0.9726	0.0519
LOC649804	0.9070	0.9367	1.0629	0.9836	0.9726	0.0340
FUSSEL18	0.9062	1.0934	0.9857	0.9051	0.9726	0.0445

PLCE1	0.9189	0.9969	1.0672	0.9074	0.9726	0.0373
MIR887	0.9421	0.9939	0.9985	0.9559	0.9726	0.0140
RAB7B	0.9727	1.0155	0.9422	0.9600	0.9726	0.0156
NFIL3	0.8695	1.1516	1.1239	0.7455	0.9726	0.0988
CD14	0.8936	1.0972	0.9592	0.9404	0.9726	0.0438
RERE	0.9163	1.1642	0.9175	0.8925	0.9726	0.0641
LOC653582	0.9571	1.0878	0.9461	0.8994	0.9726	0.0404
ESF1	0.9014	1.0155	1.0553	0.9184	0.9726	0.0373
WDR78	0.9145	1.0469	1.0267	0.9025	0.9726	0.0374
LOC1001338	0.8150	1.0932	1.1078	0.8745	0.9726	0.0749
GDF5	0.8732	1.1010	1.0071	0.9093	0.9727	0.0513
MATN3	0.8646	1.0886	0.9661	0.9714	0.9727	0.0458
C3orf38	0.7647	1.3564	1.0966	0.6730	0.9727	0.1570
LOC650879	0.8404	1.1999	0.9075	0.9428	0.9727	0.0787
ITGAM	0.8515	1.1223	1.1082	0.8088	0.9727	0.0828
ANKFN1	0.9970	1.0072	0.9708	0.9158	0.9727	0.0205
ZNF687	0.9057	0.9905	1.0377	0.9569	0.9727	0.0278
C1orf132	0.9119	1.1208	0.8697	0.9884	0.9727	0.0552
BDH1	0.8685	1.1124	1.0598	0.8500	0.9727	0.0665
ANKRD28	0.9202	1.2384	0.9405	0.7916	0.9727	0.0945
MIR1321	0.9211	1.1575	0.9918	0.8204	0.9727	0.0709
AVPR1B	0.9580	1.0484	0.9587	0.9258	0.9727	0.0264
LOC642838	0.9043	1.1432	1.0130	0.8303	0.9727	0.0681
LOC1001338	0.9533	1.0083	1.0355	0.8937	0.9727	0.0314
RGS3	0.9064	1.0823	1.0410	0.8612	0.9727	0.0529
KRAS	0.8796	1.2325	0.9809	0.7978	0.9727	0.0944
LOC1001322	0.8887	1.0173	1.0990	0.8860	0.9727	0.0521
MIR518E	0.9451	1.0773	0.9088	0.9597	0.9727	0.0365
SPHK1	0.8589	1.1309	1.0449	0.8562	0.9727	0.0688
PCDHGA9	0.9144	1.0263	1.0929	0.8575	0.9727	0.0532
MLNR	0.9288	1.0572	1.0257	0.8793	0.9727	0.0414
MUC4	0.8979	1.0909	1.0138	0.8884	0.9728	0.0486
C6orf100	0.9360	1.0324	0.9697	0.9529	0.9728	0.0210
GSTA4	0.9214	1.1939	1.0737	0.7020	0.9728	0.1061
PLXNB1	1.0645	1.2507	0.8195	0.7563	0.9728	0.1140
SCUBE2	0.8688	1.0397	1.0987	0.8839	0.9728	0.0570
ZNF645	1.0488	1.0961	0.8344	0.9118	0.9728	0.0604
IL7R	0.9344	1.1299	0.9715	0.8553	0.9728	0.0577
RUSC2	0.8548	1.2768	0.9100	0.8495	0.9728	0.1023
KLK12	0.9852	0.9804	1.0199	0.9056	0.9728	0.0241
LOC653591	0.9058	1.0716	1.1221	0.7917	0.9728	0.0760
C14orf182	0.9045	1.0262	1.0189	0.9416	0.9728	0.0298
OR8K3	0.8852	0.9912	1.0632	0.9516	0.9728	0.0372
LOC644420	0.9513	1.0403	1.0549	0.8447	0.9728	0.0484
LOC642032	0.9253	1.1761	0.9191	0.8707	0.9728	0.0688
SLC15A3	0.9533	1.0032	1.0684	0.8662	0.9728	0.0426

LOC1001282	0.9240	1.0197	1.0470	0.9004	0.9728	0.0357
LOC646097	0.9135	0.9820	1.0148	0.9809	0.9728	0.0213
FAM83C	0.9238	1.0785	0.9758	0.9131	0.9728	0.0378
LOC1001340	0.9375	1.0748	0.9807	0.8982	0.9728	0.0379
SPRED3	0.9496	1.0065	0.9927	0.9424	0.9728	0.0158
NXF2B	0.8574	1.0472	1.0635	0.9232	0.9728	0.0496
LOC1001315	0.8803	1.0585	1.0409	0.9116	0.9728	0.0450
DHX32	0.8777	1.3421	0.9402	0.7314	0.9728	0.1306
UNC13D	1.0339	0.9509	0.9632	0.9434	0.9728	0.0207
LOC647020	1.0043	1.0506	0.9349	0.9015	0.9728	0.0336
LOC1001304	0.8976	1.1788	0.9496	0.8654	0.9728	0.0708
LOC148413	0.9303	1.3116	0.9465	0.7029	0.9728	0.1259
NEK1	0.8014	1.1631	1.2255	0.7013	0.9729	0.1301
IGJ	0.9352	1.0617	0.9566	0.9380	0.9729	0.0300
CERCAM	0.9330	0.9958	1.0006	0.9619	0.9729	0.0158
LOC643344	0.9027	1.0630	0.9491	0.9767	0.9729	0.0337
OR4D11	0.8857	1.0501	0.9671	0.9885	0.9729	0.0340
ZNF599	0.8894	1.0414	0.9938	0.9668	0.9729	0.0318
LOC653930	1.0172	1.0072	1.0197	0.8474	0.9729	0.0419
HMGN3	0.9669	1.0540	1.0190	0.8515	0.9729	0.0442
GSC2	1.0317	0.9908	1.0243	0.8445	0.9729	0.0437
LOC646280	0.9078	1.1569	0.9466	0.8801	0.9729	0.0628
C1orf198	0.8865	1.3674	0.9147	0.7229	0.9729	0.1381
SYDE1	0.9167	1.0958	0.9585	0.9204	0.9729	0.0421
HEMGN	0.9200	1.0670	0.9583	0.9462	0.9729	0.0324
LOC653104	0.9672	1.0035	1.0518	0.8690	0.9729	0.0387
LOC1001335	0.9966	0.9976	0.9356	0.9617	0.9729	0.0150
SLC7A4	0.9343	1.0752	0.9995	0.8825	0.9729	0.0417
DCLK3	0.8692	1.1057	1.0991	0.8175	0.9729	0.0755
FLJ44450	0.9641	1.0798	0.9942	0.8534	0.9729	0.0468
DEFA4	0.9239	1.1622	0.9637	0.8417	0.9729	0.0680
LOC1001287	0.9436	1.1010	0.9478	0.8992	0.9729	0.0441
GTF2A2	0.9241	1.3785	0.9780	0.6109	0.9729	0.1576
SNORD26	0.8597	1.0114	1.0093	1.0112	0.9729	0.0377
OR2A9P	0.9294	1.0617	1.0760	0.8247	0.9729	0.0594
LOC642909	0.8985	1.0856	1.0700	0.8376	0.9729	0.0619
KU-MEL-3	0.8600	0.9995	1.0820	0.9502	0.9729	0.0464
GPR32	0.8310	1.0369	1.0738	0.9499	0.9729	0.0539
MYH7	0.9916	1.0714	0.9172	0.9115	0.9729	0.0376
LOC648955	0.9966	0.9111	1.0905	0.8936	0.9729	0.0452
FLJ44881	0.9002	1.0462	1.0402	0.9051	0.9729	0.0406
MIR2052	0.9639	1.0183	1.0478	0.8618	0.9729	0.0409
MCOLN1	0.9151	1.3315	0.9638	0.6813	0.9729	0.1345
LOC644010	0.9209	1.0607	1.0651	0.8451	0.9729	0.0542
ABCA9	0.9303	1.1018	0.9796	0.8801	0.9729	0.0475
LOC643328	1.1188	1.0157	0.9028	0.8545	0.9729	0.0592

LOC649407	0.9316	1.0712	1.0234	0.8655	0.9729	0.0460
C1orf65	1.0075	1.0483	0.9309	0.9052	0.9730	0.0332
TCOF1	0.9128	1.0396	1.0068	0.9326	0.9730	0.0301
LDB3	0.9339	1.0663	1.0275	0.8642	0.9730	0.0457
SERPINA3	1.0094	1.0420	0.9040	0.9366	0.9730	0.0319
OR6X1	0.9409	1.0730	1.0345	0.8436	0.9730	0.0513
POLR2A	0.9124	1.1945	1.0039	0.7813	0.9730	0.0868
LIPL4	0.9355	1.1263	0.9479	0.8824	0.9730	0.0530
MACC1	0.8797	1.1484	0.9942	0.8698	0.9730	0.0649
C2orf53	0.9630	1.0600	0.9785	0.8906	0.9730	0.0347
LOC652669	0.9248	1.2160	0.9277	0.8237	0.9730	0.0845
CETN1	0.9108	1.1428	1.0216	0.8169	0.9730	0.0704
KEL	0.9396	1.0059	1.0277	0.9189	0.9730	0.0260
FCAR	0.8856	1.1196	1.0179	0.8690	0.9730	0.0591
LOC730076	0.8868	1.0229	1.0763	0.9061	0.9730	0.0457
LOC642669	1.0515	1.0078	1.0006	0.8322	0.9730	0.0483
NOTCH2	0.9067	1.0648	1.0406	0.8800	0.9730	0.0466
LOC644841	0.8693	0.9594	1.1105	0.9530	0.9730	0.0502
LOC390937	0.8623	1.0631	1.0712	0.8956	0.9730	0.0548
LOC646179	0.9809	0.9011	1.1212	0.8890	0.9730	0.0534
NDEL1	0.8418	1.3839	0.9130	0.7535	0.9730	0.1408
LOC441806	0.9069	0.9994	1.0769	0.9090	0.9730	0.0408
OR4K15	0.9440	1.0551	0.9883	0.9048	0.9730	0.0322
C20orf118	0.9495	1.0386	1.0097	0.8945	0.9731	0.0321
MMP27	0.9957	1.0548	0.8944	0.9473	0.9731	0.0342
OR4M2	0.9847	1.0415	0.9701	0.8959	0.9731	0.0300
LOC644397	0.9328	0.9806	0.9708	1.0081	0.9731	0.0156
LOC644005	0.9622	1.1108	0.8960	0.9233	0.9731	0.0479
FIT1	0.9633	1.0129	1.0262	0.8900	0.9731	0.0308
MIR342	0.8812	1.0681	1.0745	0.8685	0.9731	0.0568
LOC1001328	0.9008	1.0075	1.0562	0.9278	0.9731	0.0358
TTBK2	0.9196	1.0023	1.0880	0.8824	0.9731	0.0458
SART1	0.9786	1.0773	0.8911	0.9454	0.9731	0.0391
EFHA2	0.8451	1.1321	1.0509	0.8643	0.9731	0.0704
LOC1001288	0.9808	1.0574	0.9889	0.8653	0.9731	0.0398
LOC649495	0.9228	1.0732	1.0228	0.8737	0.9731	0.0456
TCF2	0.8820	1.1118	1.0138	0.8849	0.9731	0.0555
SSX7	0.9078	1.1396	0.9017	0.9435	0.9731	0.0562
PROKR1	0.9191	1.0598	0.9574	0.9561	0.9731	0.0302
TEAD1	0.9802	1.0694	0.9728	0.8701	0.9731	0.0408
C11orf63	0.8934	1.0644	1.0683	0.8663	0.9731	0.0541
LOC1001307	1.0275	1.0175	0.9994	0.8480	0.9731	0.0421
HHCM	0.9523	1.0864	0.9949	0.8589	0.9731	0.0472
LOC1001329	0.9072	1.0885	0.9856	0.9111	0.9731	0.0425
SPRR4	1.0221	1.0381	0.9665	0.8659	0.9731	0.0389
LOC645752	0.8769	1.1346	1.0001	0.8808	0.9731	0.0610

LOC1001301	1.0271	1.0724	0.9714	0.8216	0.9731	0.0546
ATXN2	0.7571	1.4065	0.9938	0.7352	0.9731	0.1559
LOC1001302	0.8764	1.0540	1.0166	0.9456	0.9731	0.0393
LOC646699	0.8452	1.0944	1.0559	0.8970	0.9731	0.0604
WNT7A	1.0167	1.0038	1.0708	0.8014	0.9731	0.0591
MIR1227	0.9283	0.9781	1.0149	0.9713	0.9731	0.0178
SLC25A29	0.8961	1.1720	0.9877	0.8368	0.9731	0.0732
PRKAB2	0.9022	1.1980	1.0041	0.7883	0.9732	0.0869
APOH	0.8952	1.0648	1.0228	0.9098	0.9732	0.0418
FLJ40330	0.8664	1.1453	0.9841	0.8968	0.9732	0.0626
MYF6	0.9494	1.0496	1.0444	0.8492	0.9732	0.0473
LOC1001337	0.8885	1.1569	1.0289	0.8184	0.9732	0.0753
LOC646481	1.0116	1.0335	0.9966	0.8510	0.9732	0.0414
LOC1001320	0.9473	1.1568	1.0056	0.7830	0.9732	0.0772
LOC1001284	0.9759	1.0304	1.0030	0.8834	0.9732	0.0319
LOC729255	0.8374	1.1571	1.2144	0.6838	0.9732	0.1272
PLGLB1	0.9273	1.0154	1.0422	0.9078	0.9732	0.0328
BPIL3	0.8926	1.1240	1.0212	0.8550	0.9732	0.0616
ATAD2B	0.7599	1.1498	1.0391	0.9440	0.9732	0.0826
FLJ10661	0.8831	1.0804	1.0558	0.8734	0.9732	0.0551
LOC285423	0.9838	0.9311	1.0839	0.8940	0.9732	0.0413
TAC1	0.8596	1.0715	1.0064	0.9553	0.9732	0.0447
C21orf58	0.9480	1.1764	0.8715	0.8969	0.9732	0.0696
LOC1001327	1.1392	0.9570	0.9272	0.8695	0.9732	0.0582
MIR885	0.9255	0.9795	0.9811	1.0067	0.9732	0.0171
SLC9A3R2	1.0019	0.9442	1.0314	0.9153	0.9732	0.0265
ESRP2	0.9875	1.0058	1.0394	0.8601	0.9732	0.0392
B3GNT5	1.0008	1.1137	0.9781	0.8002	0.9732	0.0648
DRD5	0.9231	1.0609	1.0354	0.8736	0.9732	0.0447
NNAT	0.8694	1.0633	1.0383	0.9220	0.9732	0.0463
C4A	0.9084	1.1200	1.0559	0.8086	0.9732	0.0705
POU2F2	0.9186	1.0069	1.0478	0.9197	0.9732	0.0323
PSG10	0.8691	1.1130	1.0320	0.8788	0.9732	0.0597
LOC1001331	0.8793	1.0600	1.0428	0.9108	0.9732	0.0457
ABCG5	0.8936	1.1119	1.0575	0.8300	0.9732	0.0666
LOC645739	0.9352	0.9410	1.0943	0.9224	0.9732	0.0406
MCF2L2	0.9173	1.0337	0.9946	0.9473	0.9732	0.0257
PHACTR3	0.9728	1.0032	1.0476	0.8693	0.9732	0.0379
ZNF572	1.0075	0.9874	1.0096	0.8885	0.9732	0.0287
NDUFB2	0.9198	1.2671	0.9886	0.7174	0.9732	0.1136
DACH2	0.9698	1.0389	0.9641	0.9202	0.9732	0.0245
LOC1001285	0.8970	1.0145	1.0301	0.9513	0.9732	0.0306
LOC1001315	0.7872	1.0580	1.0500	0.9978	0.9732	0.0634
OR5AC2	0.8507	1.1091	1.0157	0.9175	0.9733	0.0566
AADACL3	0.9226	1.0212	1.0560	0.8933	0.9733	0.0388
PRY2	0.8692	1.0686	1.0361	0.9193	0.9733	0.0472

CRMP1	0.9389	1.0583	0.9483	0.9476	0.9733	0.0284
LOC654123	0.8937	1.0931	1.0692	0.8372	0.9733	0.0635
LOC644175	0.9566	1.0349	1.0078	0.8939	0.9733	0.0310
LOC654116	0.8312	1.2776	0.9111	0.8733	0.9733	0.1027
LOC645963	0.9341	1.0109	1.0126	0.9356	0.9733	0.0222
GRIK5	1.0000	1.1121	0.9489	0.8322	0.9733	0.0581
ARL11	0.9641	1.1047	0.9364	0.8880	0.9733	0.0465
DBC1	1.0868	0.9707	0.9945	0.8413	0.9733	0.0506
LOC147670	0.8457	1.0345	1.1304	0.8826	0.9733	0.0664
LECT1	0.8997	1.0854	1.0180	0.8902	0.9733	0.0473
KCNQ5	0.9197	1.0575	1.0489	0.8671	0.9733	0.0474
LOC652641	1.0550	0.9081	0.9601	0.9701	0.9733	0.0304
ERG	0.9221	1.1435	0.9521	0.8756	0.9733	0.0589
ZNF703	0.9571	1.0572	0.9698	0.9092	0.9733	0.0309
LOC652097	0.9013	0.9513	1.1318	0.9089	0.9733	0.0540
ZSWIM3	0.9215	1.1298	0.9501	0.8920	0.9733	0.0535
CD27	0.9798	1.0604	1.0224	0.8307	0.9733	0.0503
HCG3	0.9776	1.0435	0.9925	0.8798	0.9733	0.0342
CHST8	0.9708	1.1537	0.9810	0.7879	0.9733	0.0747
MIR889	0.9243	1.1072	0.9534	0.9084	0.9733	0.0456
LOC1001295	0.9501	1.0911	0.8588	0.9933	0.9733	0.0482
KIAA1919	0.9967	1.0516	1.0159	0.8291	0.9733	0.0494
BRF2	0.7694	1.3982	1.0756	0.6502	0.9733	0.1676
LOC643219	0.9916	1.0579	0.9630	0.8809	0.9733	0.0367
TNFAIP6	0.9306	1.0824	0.9510	0.9294	0.9733	0.0367
PRKCB	0.9295	1.0789	1.1028	0.7822	0.9734	0.0744
LOC1001344	1.0306	0.9407	1.0779	0.8442	0.9734	0.0516
SNORA56	0.9501	1.0679	0.9917	0.8837	0.9734	0.0386
PTPN4	1.0097	1.1685	0.9600	0.7553	0.9734	0.0852
C2orf3	0.8535	1.1685	1.0150	0.8564	0.9734	0.0752
DEXI	0.9243	1.2617	1.1009	0.6065	0.9734	0.1404
MT1E	0.8752	1.0886	1.0284	0.9013	0.9734	0.0509
LOC1001316	0.9585	0.9706	1.0417	0.9226	0.9734	0.0250
LOC643365	1.0312	1.0109	0.9372	0.9142	0.9734	0.0282
LOC284379	0.9150	1.0558	1.0182	0.9046	0.9734	0.0376
LOC541469	0.9704	0.9976	1.0495	0.8761	0.9734	0.0364
ZNF304	0.9081	1.1534	1.0924	0.7396	0.9734	0.0937
GNRHR	0.9315	1.0377	1.0488	0.8756	0.9734	0.0420
LOC1001289	0.7484	1.1498	1.1357	0.8598	0.9734	0.1004
LOC731954	0.9531	1.1247	0.9487	0.8671	0.9734	0.0542
NR5A1	0.9625	1.0242	1.0338	0.8731	0.9734	0.0370
LOC1001289	1.0711	1.0426	0.9661	0.8139	0.9734	0.0576
LOC646084	0.9733	1.0241	1.0282	0.8681	0.9734	0.0373
LOC441763	0.4214	1.1535	1.6167	0.7021	0.9734	0.2621
PUM1	0.7971	1.4104	0.9282	0.7580	0.9734	0.1501
LOC642425	0.8918	1.1348	1.0664	0.8008	0.9734	0.0770

LOC728675	1.0102	0.9723	0.9688	0.9425	0.9734	0.0139
tAKR	0.9424	1.0351	1.0129	0.9033	0.9734	0.0306
UGT2A3	0.9615	1.0842	0.9769	0.8713	0.9735	0.0437
LOC728423	1.0282	1.0234	0.9977	0.8446	0.9735	0.0435
RPL7A	0.7641	1.3308	1.1340	0.6650	0.9735	0.1561
LOC644173	0.9780	1.1018	0.9222	0.8918	0.9735	0.0464
LOC646183	0.9867	0.9731	1.0888	0.8453	0.9735	0.0499
FAM83G	0.9465	0.9735	1.0153	0.9585	0.9735	0.0150
LOC730132	0.8629	1.0573	1.0879	0.8858	0.9735	0.0578
MYL2	0.8934	1.0696	1.0131	0.9178	0.9735	0.0412
LOC1001336	0.9100	1.1250	1.0052	0.8538	0.9735	0.0594
LRRC27	1.0842	1.0280	0.9137	0.8681	0.9735	0.0499
LOC646746	0.9397	1.0284	1.0262	0.8996	0.9735	0.0321
MATK	1.0015	1.0947	0.8980	0.8998	0.9735	0.0471
LOC652774	1.0254	0.9888	1.0032	0.8765	0.9735	0.0332
ATP13A5	0.8677	1.0908	1.0247	0.9108	0.9735	0.0513
GPR34	0.9123	1.0697	1.0158	0.8962	0.9735	0.0416
MEG3	0.9158	1.0504	0.9967	0.9311	0.9735	0.0311
LOC648476	0.9277	1.0466	1.0184	0.9014	0.9735	0.0349
MIR130A	0.9583	1.0226	1.0574	0.8558	0.9735	0.0443
C16orf68	0.8333	1.3029	0.9496	0.8082	0.9735	0.1140
LOC645364	0.9212	1.0875	0.9470	0.9384	0.9735	0.0384
LOC1001310	0.9527	1.1704	0.9747	0.7964	0.9735	0.0767
C1orf99	0.8538	1.0966	1.0873	0.8564	0.9735	0.0684
MIR514-2	1.1154	0.8775	1.0619	0.8393	0.9735	0.0678
TRIM16L	0.8855	1.0209	1.0573	0.9305	0.9735	0.0396
FLJ34503	0.9639	1.0129	0.9803	0.9371	0.9735	0.0159
HBD	0.8122	1.0873	1.0492	0.9455	0.9736	0.0616
RANBP17	0.9405	1.0183	1.0598	0.8757	0.9736	0.0409
GJA4	0.8607	0.9503	1.2105	0.8728	0.9736	0.0814
C19orf35	0.9321	1.1001	0.9767	0.8854	0.9736	0.0461
LOC727918	0.8692	1.0320	1.1004	0.8928	0.9736	0.0555
FAM131B	0.9453	1.0062	1.0528	0.8900	0.9736	0.0355
LOC342892	0.7923	1.1620	1.0099	0.9302	0.9736	0.0772
ZNF609	0.8061	1.2861	0.9936	0.8085	0.9736	0.1131
PCDH9	0.9076	1.0185	1.0094	0.9589	0.9736	0.0256
C4orf19	0.9214	1.1502	1.0509	0.7719	0.9736	0.0819
LOC441843	0.9575	1.0243	0.9956	0.9170	0.9736	0.0233
FOXE3	0.9641	1.0714	0.9957	0.8632	0.9736	0.0431
LOC441089	0.7710	1.4471	0.9385	0.7378	0.9736	0.1638
OR4F21	0.9794	1.0254	1.0139	0.8758	0.9736	0.0340
HLA-DOB	0.8848	1.0879	1.0340	0.8877	0.9736	0.0516
LOC653717	0.9043	0.9810	1.0181	0.9911	0.9736	0.0244
MYL6	0.8344	1.3197	1.0061	0.7343	0.9736	0.1283
LOC649999	1.0333	1.2007	0.9449	0.7156	0.9736	0.1010
SETD1A	0.9241	1.2570	0.9241	0.7894	0.9736	0.0996

LOC1001286	0.8506	1.0620	1.0262	0.9558	0.9736	0.0466
LOC1001312	0.8683	1.0822	0.9815	0.9626	0.9736	0.0438
LOC728813	0.9250	1.0095	1.0741	0.8859	0.9736	0.0423
LOC1001323	0.9245	1.0415	1.0435	0.8850	0.9736	0.0406
ACVR2B	0.9224	1.1022	0.9767	0.8933	0.9736	0.0462
SPON2	0.8245	1.4151	0.8855	0.7694	0.9736	0.1490
LOC1001321	0.9018	1.1059	0.9573	0.9295	0.9736	0.0455
OTC	0.8954	1.0746	1.0341	0.8906	0.9737	0.0473
TTTY17B	0.8460	1.0668	1.0445	0.9374	0.9737	0.0511
LOC1001330	0.8804	1.0516	1.0170	0.9456	0.9737	0.0381
OTOP3	1.0036	1.0195	0.9856	0.8860	0.9737	0.0300
SLC38A11	0.9522	1.0614	1.0314	0.8497	0.9737	0.0473
ATP12A	1.0315	1.0796	0.9481	0.8355	0.9737	0.0535
DMRT2	0.9931	1.0409	0.9731	0.8876	0.9737	0.0320
LOC653891	1.0260	1.0368	1.0285	0.8034	0.9737	0.0568
SYNPR	0.8910	0.9655	1.0941	0.9441	0.9737	0.0431
ATHL1	0.9752	1.0517	0.9771	0.8907	0.9737	0.0329
MIR518F	0.8741	1.0504	0.9746	0.9957	0.9737	0.0368
LOC650520	0.9486	1.0392	0.9775	0.9295	0.9737	0.0240
CCDC144A	0.9257	1.1123	0.9542	0.9026	0.9737	0.0474
LOC727945	0.9198	1.0414	1.0193	0.9143	0.9737	0.0330
NACA2	1.1204	1.0307	0.8499	0.8938	0.9737	0.0622
OVOL1	0.8925	1.0577	1.0116	0.9330	0.9737	0.0374
LOC1001336	0.8479	1.2647	1.0409	0.7414	0.9737	0.1151
IFNE	0.9909	1.1219	0.8726	0.9095	0.9737	0.0552
LOC652094	0.9131	0.8427	1.0707	1.0683	0.9737	0.0571
FAM107B	0.8713	1.3860	1.0119	0.6256	0.9737	0.1589
EMX2	0.8719	1.0944	1.0201	0.9084	0.9737	0.0511
LOC643318	0.9788	1.0157	1.0555	0.8448	0.9737	0.0457
LOC727868	0.9101	1.1090	1.0025	0.8732	0.9737	0.0527
LOC653324	0.9904	0.9856	1.0016	0.9172	0.9737	0.0191
NRG1	0.9478	1.0402	0.9943	0.9127	0.9737	0.0277
RBMY1E	0.9519	1.0932	0.9556	0.8943	0.9737	0.0422
HNRNPUL1	0.9309	1.1338	1.1007	0.7296	0.9738	0.0927
TMEM156	0.8840	1.0538	1.1120	0.8451	0.9738	0.0646
WNT11	0.9468	1.0662	0.9558	0.9262	0.9738	0.0314
MIR1255B1	0.9547	1.0790	0.9828	0.8785	0.9738	0.0414
LOC653254	0.8833	1.1599	1.0216	0.8301	0.9738	0.0740
LOC1001290	0.9610	1.0266	0.9936	0.9139	0.9738	0.0240
AGAP1	0.9960	1.0544	1.0031	0.8415	0.9738	0.0460
KRTAP9-5	0.9420	1.1108	0.9921	0.8502	0.9738	0.0543
LOC649416	0.9570	1.1159	0.9236	0.8985	0.9738	0.0489
NPTX1	0.8553	1.0881	0.9945	0.9571	0.9738	0.0482
LOC729121	0.8697	1.0494	1.0606	0.9154	0.9738	0.0479
DCUN1D2	0.9420	1.1052	0.9847	0.8632	0.9738	0.0505
LOC1001337	0.8862	1.1503	0.9809	0.8777	0.9738	0.0633

FAM169A	0.9838	1.0531	1.0163	0.8419	0.9738	0.0462
LOC646897	0.8671	1.0516	1.0171	0.9593	0.9738	0.0404
LOC654032	0.9769	1.0440	0.9561	0.9181	0.9738	0.0264
LOC641820	0.8765	1.1792	1.0148	0.8246	0.9738	0.0794
AQP5	1.0115	0.9249	0.9772	0.9816	0.9738	0.0180
IFNA6	0.9379	1.0529	0.9404	0.9640	0.9738	0.0270
LOC1001302	1.0863	0.9205	1.0083	0.8800	0.9738	0.0461
LOC642980	0.9102	1.1134	0.9963	0.8754	0.9738	0.0530
LOC643596	0.9134	1.0809	1.0105	0.8905	0.9738	0.0442
BEST2	0.9458	1.0045	1.0936	0.8513	0.9738	0.0509
GRAP	0.9628	1.0548	1.0147	0.8629	0.9738	0.0415
LOC647958	0.8857	1.0605	1.0630	0.8860	0.9738	0.0508
COX4I2	0.9278	1.0634	0.9842	0.9199	0.9738	0.0331
LOC653555	0.9498	1.0164	1.0909	0.8381	0.9738	0.0536
LOC646034	0.9405	1.1471	0.9710	0.8367	0.9738	0.0645
P76	0.8897	1.1075	1.0220	0.8762	0.9738	0.0554
LOC642384	0.8467	1.1358	0.9923	0.9205	0.9738	0.0616
LOC650363	0.9016	1.1203	0.9539	0.9195	0.9738	0.0500
GUCY2C	0.9001	1.0731	0.9935	0.9286	0.9738	0.0384
LOC653648	0.9318	1.0987	1.0004	0.8644	0.9738	0.0500
LOC440776	1.0530	1.0963	0.9940	0.7521	0.9739	0.0768
LOC1001306	0.7687	1.1076	1.0229	0.9962	0.9739	0.0724
HES7	0.9741	1.0134	1.0154	0.8925	0.9739	0.0287
IL31	0.9155	1.2053	0.9484	0.8262	0.9739	0.0814
LOC1001318	0.9236	1.0177	1.0751	0.8791	0.9739	0.0444
RPP38	0.8413	1.2408	1.0205	0.7928	0.9739	0.1016
APOM	0.9852	1.1262	0.9177	0.8663	0.9739	0.0563
C21orf130	0.8545	1.0687	1.0421	0.9301	0.9739	0.0498
LOC647174	0.8103	1.0665	1.1618	0.8568	0.9739	0.0839
LASS3	0.9696	1.1112	0.9256	0.8892	0.9739	0.0486
C3orf52	0.9666	1.0921	0.9749	0.8619	0.9739	0.0470
RPGRIP1	0.9476	1.1067	0.9289	0.9124	0.9739	0.0449
ZNF608	0.9608	1.1077	0.9703	0.8567	0.9739	0.0515
LOC1001319	0.9217	1.0565	1.0152	0.9023	0.9739	0.0370
MUSTN1	0.9420	1.0219	1.0379	0.8938	0.9739	0.0340
PABPC3	0.9079	1.1105	1.1326	0.7446	0.9739	0.0916
LOC649452	0.9436	1.0138	0.9790	0.9592	0.9739	0.0151
LOC645418	0.8210	1.0308	1.1317	0.9121	0.9739	0.0679
LOC643123	0.9239	1.1239	1.0380	0.8098	0.9739	0.0683
LOC1001344	0.8487	1.1124	1.0346	0.9000	0.9739	0.0606
KRTAP2-2	0.9899	1.0707	0.9416	0.8934	0.9739	0.0378
MIR584	0.8608	1.0808	1.1447	0.8093	0.9739	0.0819
LOC1001326	0.9210	0.9682	1.0386	0.9679	0.9739	0.0243
LOC648169	0.9987	0.9859	1.0312	0.8799	0.9739	0.0327
ABHD14B	0.8278	1.2599	1.0225	0.7855	0.9739	0.1084
LIPJ	0.9227	1.0662	1.0372	0.8696	0.9739	0.0466

LOC643926	0.8524	1.1694	0.9854	0.8886	0.9739	0.0710
LOC647479	0.8953	1.0657	1.0275	0.9073	0.9739	0.0427
LOC650325	0.8010	1.1202	1.0592	0.9154	0.9739	0.0719
TPCN1	0.8378	1.0071	1.0432	1.0078	0.9739	0.0462
CABP7	0.8540	1.1386	0.9359	0.9674	0.9740	0.0599
CDO1	0.8925	1.1222	1.0078	0.8733	0.9740	0.0577
LOC1001316	0.9963	1.1153	0.9402	0.8440	0.9740	0.0567
TCEANC	0.9794	1.2079	0.9776	0.7310	0.9740	0.0974
LOC399940	0.9461	1.1267	0.9796	0.8434	0.9740	0.0586
LOC650528	0.8164	1.0639	1.0446	0.9710	0.9740	0.0562
LOC642714	0.8974	1.1377	0.9921	0.8687	0.9740	0.0606
LOC648040	0.8413	1.0279	1.0302	0.9964	0.9740	0.0449
PRPS1L1	0.9306	1.0506	1.0364	0.8782	0.9740	0.0417
C17orf57	0.8892	1.0583	0.9755	0.9728	0.9740	0.0345
WDR64	0.9456	1.0694	0.9622	0.9187	0.9740	0.0330
ABCC11	0.9535	1.0775	1.0245	0.8405	0.9740	0.0512
RTN4RL2	1.0739	1.0281	0.8863	0.9077	0.9740	0.0456
LOC730546	0.9588	1.0442	0.9924	0.9005	0.9740	0.0301
SALL1	0.9878	1.0214	0.9918	0.8949	0.9740	0.0274
GPR109A	1.0313	0.9915	0.9660	0.9072	0.9740	0.0260
MVK	1.0715	1.1630	0.9153	0.7461	0.9740	0.0916
C17orf98	0.8751	1.1801	0.9326	0.9082	0.9740	0.0697
LOC642591	0.8870	1.0633	0.9296	1.0161	0.9740	0.0401
PLEKHG4	0.8812	1.0763	1.1027	0.8359	0.9740	0.0675
LOC1001315	0.8149	1.1893	0.9353	0.9566	0.9740	0.0782
LOC643869	0.9566	1.0030	1.0111	0.9253	0.9740	0.0202
LOC653574	0.8633	1.0977	0.9535	0.9816	0.9740	0.0483
LOC1001283	0.9233	1.0580	1.0147	0.9003	0.9740	0.0373
MIR138-2	0.8649	1.0438	1.0643	0.9232	0.9740	0.0479
LOC340069	0.8268	1.1449	1.0732	0.8513	0.9741	0.0795
LOC653625	0.7889	1.2308	0.9235	0.9530	0.9741	0.0928
LOC647862	1.0006	0.9453	1.0507	0.8996	0.9741	0.0329
PDZD3	0.9323	1.0709	1.0153	0.8777	0.9741	0.0429
DUOX1	0.9477	1.0275	1.0356	0.8854	0.9741	0.0356
B3GALT1	0.9740	1.0758	0.9704	0.8761	0.9741	0.0408
LOC646363	0.9202	1.0671	0.9506	0.9584	0.9741	0.0321
MYO1B	0.8754	1.1848	1.0176	0.8185	0.9741	0.0818
RPL14L	0.6992	1.3176	1.1460	0.7334	0.9741	0.1530
PRAMEF20	0.9287	1.0814	0.9672	0.9190	0.9741	0.0372
C22orf23	1.0123	1.0944	0.9588	0.8309	0.9741	0.0553
OR52K1	0.8936	1.0801	1.0262	0.8965	0.9741	0.0470
AQP2	0.8665	1.1532	0.9979	0.8788	0.9741	0.0666
LOC645135	0.8649	1.0962	0.9586	0.9767	0.9741	0.0475
WHAMML1	0.9140	1.0577	0.9953	0.9294	0.9741	0.0330
LOC1001319	0.9439	1.1484	0.9518	0.8524	0.9741	0.0623
SUSD5	0.9071	1.0938	0.9138	0.9817	0.9741	0.0433

LOC645306	1.0465	1.0355	0.9536	0.8608	0.9741	0.0431
LOC1001282	0.9511	1.0725	0.9709	0.9020	0.9741	0.0358
LOC645651	0.9021	1.0863	1.0711	0.8369	0.9741	0.0619
PI4KA	0.9981	1.1092	0.9753	0.8139	0.9741	0.0609
ABAT	0.9103	1.1836	1.0605	0.7421	0.9741	0.0954
UPK1B	0.8258	0.9999	1.0690	1.0017	0.9741	0.0520
AMMECR1L	0.9787	1.1696	1.0214	0.7269	0.9741	0.0920
LOC402279	0.9303	1.0446	0.9668	0.9548	0.9741	0.0247
CLIP3	0.9212	1.1760	0.9516	0.8477	0.9741	0.0707
RAB9B	0.8849	1.0562	1.1073	0.8482	0.9741	0.0634
LOC1001306	0.9883	1.0564	1.0313	0.8206	0.9741	0.0531
NDST4	0.9222	1.0080	0.9921	0.9743	0.9742	0.0186
LOC1001290	0.8707	1.0235	1.0963	0.9061	0.9742	0.0522
LOC641905	0.8924	1.1792	0.9664	0.8586	0.9742	0.0720
LOC1001331	0.9065	1.2009	0.9497	0.8395	0.9742	0.0789
LOC441420	0.9231	1.0521	0.9750	0.9465	0.9742	0.0281
A2M	0.9824	1.0438	0.9553	0.9153	0.9742	0.0270
LOC646569	0.8835	1.1178	1.0849	0.8104	0.9742	0.0752
MAGEB4	0.8909	1.0709	1.0057	0.9293	0.9742	0.0401
LOC643444	0.9017	1.0822	1.0504	0.8624	0.9742	0.0542
SNORD15A	0.9348	1.0480	1.0275	0.8865	0.9742	0.0382
LOC648665	0.9275	1.1068	0.9863	0.8761	0.9742	0.0496
LOC645565	0.9631	1.0222	1.0295	0.8819	0.9742	0.0341
LOC1001337	0.9310	1.0539	0.9309	0.9809	0.9742	0.0291
LOC644611	0.9331	1.0880	1.0439	0.8318	0.9742	0.0576
SCNN1B	0.9057	1.0656	1.0316	0.8939	0.9742	0.0436
MIR325	0.9263	1.0560	0.9552	0.9592	0.9742	0.0282
ODZ2	0.9211	1.1110	0.9087	0.9560	0.9742	0.0467
OR51F1	0.9097	1.0545	1.0224	0.9102	0.9742	0.0377
ALKBH1	0.8628	1.3148	0.9864	0.7328	0.9742	0.1248
MC5R	0.9443	1.1349	0.9070	0.9106	0.9742	0.0542
LOC645974	0.8734	1.1352	0.9758	0.9124	0.9742	0.0577
LOC51145	0.9193	1.0719	1.0239	0.8817	0.9742	0.0443
ERCC-00096	0.9713	0.9559	1.0208	0.9488	0.9742	0.0162
LOC647008	0.9163	1.0925	0.9966	0.8915	0.9742	0.0454
CBWD2	0.9572	1.0187	1.0204	0.9006	0.9742	0.0286
MIR557	0.8188	1.2280	0.9880	0.8621	0.9742	0.0919
LRTM2	0.9244	1.1849	0.9006	0.8871	0.9742	0.0706
MAGEA8	0.9562	1.0667	1.0303	0.8437	0.9742	0.0492
TBC1D29	0.8791	1.0096	1.0592	0.9491	0.9742	0.0389
LOC643692	0.9149	0.9882	1.0555	0.9383	0.9742	0.0311
LOC1001338	0.9839	1.1035	0.8741	0.9354	0.9742	0.0486
FLJ41170	0.8322	1.1431	0.9451	0.9765	0.9742	0.0642
NLRC3	1.0395	1.0784	1.0065	0.7726	0.9742	0.0688
LOC338651	0.9917	1.0650	0.9771	0.8632	0.9743	0.0417
LOC1001309	0.9479	1.0008	1.0247	0.9236	0.9743	0.0233

LOC145845	1.0051	1.0517	1.0222	0.8181	0.9743	0.0529
TRIM69	1.0118	1.0375	0.9718	0.8760	0.9743	0.0354
LOC392583	0.9642	0.9617	1.0744	0.8968	0.9743	0.0368
LOC643203	0.9175	1.0810	0.9848	0.9138	0.9743	0.0391
SLC26A6	1.0002	1.1944	0.8642	0.8383	0.9743	0.0815
LOC388814	0.9253	0.9959	1.0212	0.9547	0.9743	0.0213
PIK3CA	0.9540	0.9718	1.0682	0.9031	0.9743	0.0345
LOC650253	0.9402	1.0179	0.9884	0.9506	0.9743	0.0178
NFYA	1.0528	1.1244	0.8602	0.8598	0.9743	0.0676
PGA3	0.9105	1.1058	0.9927	0.8882	0.9743	0.0492
LOC729426	0.8159	1.1220	1.0678	0.8914	0.9743	0.0722
ARPC1B	1.0177	1.2137	0.8835	0.7823	0.9743	0.0932
MCHR2	0.9992	1.0545	0.9164	0.9270	0.9743	0.0325
BTG4	0.9632	0.9634	1.0726	0.8980	0.9743	0.0362
LOC642538	0.9926	0.9948	0.9485	0.9613	0.9743	0.0115
LOC202459	1.0086	1.0656	1.0142	0.8088	0.9743	0.0566
SLC10A4	0.9490	1.0666	0.9537	0.9279	0.9743	0.0313
LOC286076	0.9062	1.0291	1.0000	0.9619	0.9743	0.0265
CD300C	0.8849	1.0254	1.0110	0.9760	0.9743	0.0316
LOC652451	0.9237	1.0763	0.9070	0.9904	0.9743	0.0385
LOC1001330	0.8311	0.9827	1.1598	0.9238	0.9743	0.0692
CAV1	0.9820	1.0677	0.9356	0.9121	0.9743	0.0343
FLJ10088	1.0215	1.0064	0.9571	0.9124	0.9743	0.0248
C14orf53	0.7668	1.1377	1.1178	0.8752	0.9743	0.0914
NAP1L6	0.9211	1.1671	0.8746	0.9345	0.9743	0.0655
ZNF804B	0.8508	1.0385	1.0382	0.9699	0.9744	0.0442
LOC1001280	0.9491	0.9922	1.0743	0.8818	0.9744	0.0403
LOC149069	0.9194	1.1071	0.9653	0.9056	0.9744	0.0460
MIR524	0.9449	0.9596	1.0061	0.9868	0.9744	0.0137
NOTCH4	0.9272	1.0306	1.0008	0.9388	0.9744	0.0247
RASAL1	0.8927	1.1345	1.0226	0.8476	0.9744	0.0650
LOC651040	0.9056	1.1255	0.9783	0.8880	0.9744	0.0540
STARD9	0.8804	1.1170	0.9946	0.9055	0.9744	0.0535
GOSR2	0.8728	1.1853	1.0427	0.7967	0.9744	0.0871
CPA6	0.9114	1.0262	1.0239	0.9361	0.9744	0.0297
GPAT2	0.9593	1.0906	1.0144	0.8332	0.9744	0.0542
PRAME	0.8708	1.3934	0.9019	0.7315	0.9744	0.1445
MMP19	1.0040	0.9767	0.9795	0.9374	0.9744	0.0138
TRPV4	0.8766	1.0539	1.0375	0.9295	0.9744	0.0427
EFEMP1	0.8757	1.0607	1.0212	0.9400	0.9744	0.0414
LOC1001318	0.9550	1.0618	1.0065	0.8742	0.9744	0.0399
C9orf139	1.0089	0.9934	0.9631	0.9322	0.9744	0.0170
MIR199A1	0.8927	1.1466	1.0353	0.8230	0.9744	0.0724
PPP1R1B	0.9270	1.0463	1.0027	0.9216	0.9744	0.0303
LOC1001302	0.9048	1.0952	1.0955	0.8022	0.9744	0.0729
KRT16	0.9636	1.0591	0.9959	0.8791	0.9744	0.0375

PAX8	0.9085	1.1674	0.9431	0.8786	0.9744	0.0657
LOC642741	0.9761	0.9778	1.0999	0.8439	0.9744	0.0523
CAMKK1	0.9611	1.0650	0.9433	0.9283	0.9744	0.0309
LOC284701	0.8904	1.1036	0.9980	0.9058	0.9744	0.0492
LOC648876	0.9690	1.1894	0.9793	0.7601	0.9744	0.0876
MIR425	0.9432	1.0610	1.0993	0.7942	0.9744	0.0687
PDAP1	0.9657	1.1066	1.0464	0.7791	0.9744	0.0712
LOC649139	0.9617	1.1476	0.9815	0.8070	0.9744	0.0697
MIR654	0.9612	1.0477	0.9903	0.8986	0.9745	0.0310
LOC650985	0.9403	1.1399	1.0571	0.7606	0.9745	0.0822
SERPINB3	0.8953	1.0403	1.0825	0.8798	0.9745	0.0510
EGF	0.8704	1.1271	1.0598	0.8406	0.9745	0.0703
LOC151658	0.9089	1.0106	1.0359	0.9426	0.9745	0.0294
OIT3	0.8739	1.1331	0.9894	0.9015	0.9745	0.0583
OR5F1	0.8635	1.0926	0.9815	0.9602	0.9745	0.0470
GRXCR2	0.9363	1.0598	0.9314	0.9705	0.9745	0.0297
LOC646529	0.9332	1.1051	0.9866	0.8731	0.9745	0.0493
LOC1001297	0.9522	0.9975	0.9849	0.9634	0.9745	0.0102
IGSF6	0.9515	1.0166	1.0193	0.9106	0.9745	0.0264
LOC644262	0.9828	1.0024	1.0654	0.8474	0.9745	0.0459
LOC1001337	0.9685	1.0708	0.9906	0.8682	0.9745	0.0417
LOC730087	0.8906	1.0353	1.0998	0.8723	0.9745	0.0554
ERCC-00165	0.9777	1.0563	1.0895	0.7744	0.9745	0.0707
LOC647374	0.9526	0.9706	1.0871	0.8877	0.9745	0.0415
LOC728819	0.9690	1.0654	0.9561	0.9077	0.9745	0.0330
LOC649641	0.9182	1.0520	1.0386	0.8893	0.9745	0.0414
ERCC-00075	0.9838	1.0731	0.9960	0.8452	0.9745	0.0474
DKFZp686J05	0.9727	1.0483	1.0041	0.8730	0.9745	0.0372
OR2A7	0.8803	1.0616	0.9836	0.9726	0.9745	0.0371
LOC651506	0.8121	1.1100	1.0669	0.9091	0.9745	0.0693
MIR100	0.9450	1.0717	0.9389	0.9426	0.9745	0.0324
ANKRD23	0.8700	1.1083	1.0020	0.9179	0.9745	0.0523
SLC4A11	0.9557	1.1576	0.9806	0.8042	0.9745	0.0724
LOC1001342	1.0134	1.0720	0.9013	0.9115	0.9745	0.0412
NRK	0.9149	1.2010	0.9292	0.8530	0.9745	0.0773
MAP6	0.9677	1.0390	1.0422	0.8493	0.9745	0.0452
LOC643533	0.9145	1.0431	1.0410	0.8996	0.9745	0.0391
NRAP	0.9344	1.0124	1.0371	0.9143	0.9745	0.0297
LOC644347	0.9728	1.0845	0.9393	0.9016	0.9745	0.0394
LOC729956	0.9987	1.1178	0.8968	0.8849	0.9746	0.0541
LOC647099	0.9600	1.2308	0.9681	0.7394	0.9746	0.1005
MLC1	0.8991	1.0389	1.0442	0.9160	0.9746	0.0389
PRKCE	0.9416	1.1540	0.9278	0.8748	0.9746	0.0615
LOC728919	0.8859	0.9833	1.0953	0.9338	0.9746	0.0449
LOC389852	0.9236	1.0538	0.9823	0.9386	0.9746	0.0292
HMP19	0.9020	1.0871	1.0306	0.8787	0.9746	0.0502

LOC1001325	0.9091	1.1468	1.0055	0.8369	0.9746	0.0670
LOC653587	0.9502	1.1392	0.9957	0.8133	0.9746	0.0672
LOC728599	0.8754	1.1269	0.9976	0.8985	0.9746	0.0573
LOC651898	0.9380	0.9985	1.0741	0.8877	0.9746	0.0402
MTMR1	0.9213	1.0894	0.9525	0.9352	0.9746	0.0388
ARNT2	1.0124	1.1438	0.9328	0.8094	0.9746	0.0702
EPO	0.9297	1.0111	0.9839	0.9736	0.9746	0.0169
LOC285588	0.8351	1.1260	1.0463	0.8909	0.9746	0.0674
LNX1	0.9678	1.1586	0.8975	0.8745	0.9746	0.0645
TNNI2	0.9810	0.9654	0.9926	0.9594	0.9746	0.0075
PLA2G3	0.8863	1.0994	0.9166	0.9961	0.9746	0.0476
GPR97	0.9255	1.0523	0.9810	0.9396	0.9746	0.0284
LOC138864	0.8865	1.0908	0.9746	0.9465	0.9746	0.0429
LOC348926	0.8735	1.1071	1.0839	0.8339	0.9746	0.0704
LOC644436	0.9532	0.9663	1.0180	0.9609	0.9746	0.0147
CXorf45	0.9281	1.2105	0.9037	0.8561	0.9746	0.0800
LOC643693	0.9789	1.1338	0.9101	0.8757	0.9746	0.0572
LOC643903	0.9009	1.0790	1.0163	0.9023	0.9746	0.0441
KCNH4	0.9822	1.0839	0.9079	0.9246	0.9746	0.0397
OR10C1	0.8412	1.0170	1.1069	0.9333	0.9746	0.0569
LOC652673	0.8546	1.1404	1.0180	0.8855	0.9746	0.0656
LOC642354	0.9430	1.1320	0.9760	0.8475	0.9746	0.0591
C7orf65	0.9456	1.1295	0.9531	0.8703	0.9746	0.0549
HCG18	0.9775	1.1049	0.9885	0.8276	0.9746	0.0568
SEC15L2	0.9363	1.0867	0.9851	0.8904	0.9746	0.0421
FAM90A13	0.9310	1.0828	0.9849	0.8998	0.9746	0.0401
TTTY7	0.8935	1.0509	1.0653	0.8889	0.9746	0.0483
LOC643911	0.8626	1.0539	1.3292	0.6529	0.9746	0.1438
LOC729069	0.8930	1.0240	1.0215	0.9600	0.9746	0.0310
LOC729349	0.8658	1.1063	1.1214	0.8051	0.9746	0.0814
LOC1001299	0.9345	1.0126	1.0428	0.9087	0.9746	0.0317
LOC652342	0.9501	1.0876	0.9418	0.9191	0.9747	0.0382
LOC402716	0.9194	1.0471	1.0786	0.8536	0.9747	0.0530
LOC652578	0.9623	1.0024	1.0111	0.9230	0.9747	0.0203
LOC389328	1.0100	1.0087	0.9724	0.9077	0.9747	0.0240
NCRNA0016[illegible]	0.9493	1.1870	0.8856	0.8769	0.9747	0.0726
LOC284288	0.8456	1.0172	1.0995	0.9364	0.9747	0.0544
FMNL2	0.9652	1.0687	1.0080	0.8569	0.9747	0.0446
P2RX2	0.9207	1.0736	1.0250	0.8795	0.9747	0.0450
NIT1	0.8093	0.9915	1.1379	0.9601	0.9747	0.0674
MIR1208	0.9263	1.0746	1.0497	0.8481	0.9747	0.0532
LOC728379	0.8996	1.0830	0.9854	0.9307	0.9747	0.0402
LOC642367	0.8937	1.2031	0.9664	0.8355	0.9747	0.0807
LOC653684	0.9327	0.9693	1.0207	0.9761	0.9747	0.0180
LOC646209	0.9109	1.0414	1.0557	0.8909	0.9747	0.0429
LOC388907	0.9243	1.0476	1.0477	0.8792	0.9747	0.0431

AURKC	0.9135	1.0638	1.0296	0.8920	0.9747	0.0424
LOC650227	0.9049	1.1139	0.9233	0.9566	0.9747	0.0476
LOC441896	0.8910	1.0688	1.0640	0.8751	0.9747	0.0530
LOC647558	0.9604	1.0168	1.0456	0.8761	0.9747	0.0374
LOC651051	0.9276	0.9463	1.0847	0.9403	0.9747	0.0369
HOXB8	0.9930	0.9636	1.0068	0.9355	0.9747	0.0159
BSPH1	0.9254	1.1515	0.9687	0.8534	0.9747	0.0635
TMPRSS7	0.9518	1.0668	1.0385	0.8419	0.9747	0.0506
LOC728481	0.8436	1.0595	1.3909	0.6050	0.9747	0.1669
LOC647911	1.0130	1.0466	0.9321	0.9072	0.9747	0.0329
EDA	0.8877	1.0731	1.0152	0.9231	0.9747	0.0424
CCDC54	0.9919	1.0158	0.9705	0.9208	0.9748	0.0202
MIR1236	0.9191	1.0421	1.0120	0.9258	0.9748	0.0308
MGC39372	0.9690	1.0572	0.9487	0.9242	0.9748	0.0289
LOC1001285	0.9524	1.0185	1.0174	0.9108	0.9748	0.0263
WBP2NL	0.9498	1.0449	1.0014	0.9029	0.9748	0.0308
ABCA12	0.9752	1.0248	0.9869	0.9121	0.9748	0.0234
LOC1001291	0.8763	0.9784	1.0314	1.0130	0.9748	0.0346
OR10J5	0.9365	1.1558	0.9598	0.8470	0.9748	0.0651
MIR583	0.8974	1.1449	0.9338	0.9230	0.9748	0.0572
C21orf121	0.9383	0.9625	1.0090	0.9893	0.9748	0.0154
HEPACAM2	0.9013	1.1282	0.9611	0.9085	0.9748	0.0528
LOC645690	0.9166	1.0061	1.0908	0.8856	0.9748	0.0464
LOC651362	0.8326	1.1547	1.0358	0.8761	0.9748	0.0742
LOC729312	0.8712	1.1675	0.9450	0.9154	0.9748	0.0660
LRP2	0.9014	0.9973	1.0377	0.9628	0.9748	0.0289
LOC1001305	0.9903	0.9903	1.0143	0.9043	0.9748	0.0242
NEB	0.9364	1.0557	1.0283	0.8789	0.9748	0.0409
LOC728592	0.8931	1.1317	0.9298	0.9447	0.9748	0.0534
LOC648790	0.9410	1.0935	1.0207	0.8440	0.9748	0.0536
TBC1D3G	0.8915	1.2168	0.9013	0.8897	0.9748	0.0807
SLC35E3	0.8208	1.2679	1.0882	0.7223	0.9748	0.1246
LOC649442	0.9376	0.9348	1.0034	1.0235	0.9748	0.0227
DCST2	1.0183	1.0278	0.9346	0.9186	0.9748	0.0281
LOC388344	0.8893	1.0633	1.0603	0.8864	0.9748	0.0502
LOC1001323	0.9444	1.1031	0.9795	0.8722	0.9748	0.0483
NTS	1.0012	1.0538	1.0256	0.8187	0.9748	0.0531
HDAC9	0.9419	1.0401	0.9980	0.9193	0.9748	0.0273
LOC1001344	0.8563	1.0614	0.9908	0.9908	0.9748	0.0429
LOC647571	0.9490	1.0616	0.9876	0.9011	0.9748	0.0339
ANKRD6	0.9187	1.1234	0.9697	0.8875	0.9748	0.0523
KCNAB2	0.8647	1.1700	0.9661	0.8984	0.9748	0.0684
HEXA	0.9564	1.0748	0.9797	0.8885	0.9748	0.0385
MSLNL	0.9544	1.0293	1.0697	0.8460	0.9748	0.0491
LSR	0.9209	1.1655	0.9918	0.8212	0.9748	0.0726
RP13-360B22	0.9729	0.9512	1.0109	0.9644	0.9748	0.0128

LOC1001324	0.9156	1.1918	0.9502	0.8418	0.9749	0.0758
NPAS3	0.9421	1.0323	1.0527	0.8723	0.9749	0.0418
LOC1001297	0.9308	1.0941	1.0205	0.8539	0.9749	0.0523
PLEKHG6	0.9637	1.0341	1.0432	0.8585	0.9749	0.0427
LOC729159	0.9135	1.0563	1.0212	0.9084	0.9749	0.0376
ROPN1	0.8904	1.1002	0.9887	0.9202	0.9749	0.0466
GP5	0.9238	1.0102	1.0297	0.9357	0.9749	0.0264
LOC1001333	0.9308	0.9835	1.0819	0.9033	0.9749	0.0394
USP51	0.9945	1.0307	1.0112	0.8630	0.9749	0.0380
LOC440402	0.9069	1.1054	0.9273	0.9600	0.9749	0.0448
ZNF426	0.8451	1.2597	1.0368	0.7580	0.9749	0.1114
MYL9	0.9653	1.0338	1.0131	0.8873	0.9749	0.0325
LOC649414	0.8896	1.0559	1.0459	0.9082	0.9749	0.0441
PNLDC1	0.9083	1.0065	1.0473	0.9374	0.9749	0.0317
RNF219	0.8312	1.3529	0.8730	0.8424	0.9749	0.1263
LOC648710	0.9029	1.0483	1.0496	0.8987	0.9749	0.0428
LOC401980	0.9038	1.1075	1.0127	0.8756	0.9749	0.0532
SLC25A17	0.8443	1.3159	1.0730	0.6663	0.9749	0.1409
LOC1001302	0.9283	0.9594	1.0854	0.9265	0.9749	0.0376
C17orf37	1.0241	0.9388	1.1051	0.8316	0.9749	0.0586
LOC339843	0.9787	1.0786	1.0576	0.7848	0.9749	0.0669
DULLARD	0.9365	1.4482	0.7902	0.7249	0.9749	0.1638
CD180	0.9172	1.1276	1.0261	0.8289	0.9749	0.0649
LOC728148	0.9273	0.9727	1.1255	0.8742	0.9749	0.0541
GATAD2B	0.8957	1.1129	1.0671	0.8241	0.9749	0.0686
LOC653486	0.9452	1.0750	1.0895	0.7902	0.9749	0.0696
KIF12	0.8587	1.0824	1.0548	0.9038	0.9749	0.0551
MIR146A	0.9405	0.9387	1.0165	1.0042	0.9750	0.0206
LOC1001310	0.8886	1.0097	1.0517	0.9498	0.9750	0.0356
KCNIP1	0.9528	1.0301	0.9701	0.9469	0.9750	0.0190
GPR18	0.9495	0.9786	1.0922	0.8795	0.9750	0.0443
LOC649493	1.0038	1.1181	0.9006	0.8774	0.9750	0.0551
NT5DC4	0.9188	1.0537	1.0435	0.8838	0.9750	0.0432
FLJ45202	1.0154	1.1701	0.8974	0.8170	0.9750	0.0767
LOC255130	0.9860	1.0936	0.9351	0.8852	0.9750	0.0446
NOTCH3	0.8647	1.3835	0.8584	0.7934	0.9750	0.1371
LOC1001328	1.0207	0.9358	1.0462	0.8973	0.9750	0.0350
IGFL2	0.8935	1.0494	1.0916	0.8653	0.9750	0.0561
LOC1001280	0.9374	1.0477	1.0110	0.9039	0.9750	0.0330
HCFC2	1.0304	1.0535	1.0704	0.7457	0.9750	0.0769
LOC653520	0.9273	1.0602	0.9917	0.9208	0.9750	0.0326
CCDC22	0.9831	1.1994	0.9421	0.7753	0.9750	0.0873
IKZF2	0.9377	1.1031	0.9445	0.9147	0.9750	0.0432
LOC1001323	0.9143	1.0741	1.0342	0.8775	0.9750	0.0470
LOC1001325	0.8818	1.1081	1.0206	0.8895	0.9750	0.0546
MAFA	0.9469	0.9220	1.1094	0.9218	0.9750	0.0452

TPSG1	0.8901	1.0324	1.0502	0.9273	0.9750	0.0392
NTN1	0.9180	1.1075	0.9687	0.9059	0.9750	0.0462
FAM7A3	0.8730	1.0962	1.0074	0.9235	0.9750	0.0490
YIPF1	0.9447	1.3172	0.9192	0.7190	0.9750	0.1247
HERC2P4	0.8512	1.1047	1.0253	0.9189	0.9750	0.0561
LOC727909	0.9054	1.1119	0.9605	0.9223	0.9750	0.0471
GPR109B	0.9508	1.0568	1.0015	0.8911	0.9750	0.0354
LOC440836	0.9255	1.0935	0.9983	0.8829	0.9750	0.0461
C11orf87	0.8615	1.1115	0.8669	1.0603	0.9750	0.0648
LOC649341	0.8570	1.1399	0.9451	0.9582	0.9751	0.0594
DNTTIP2	1.0047	1.0559	0.9489	0.8907	0.9751	0.0356
PSD	0.7686	1.1608	1.0811	0.8897	0.9751	0.0893
SLC25A34	0.7976	1.2130	1.0704	0.8192	0.9751	0.1006
TCEB1P3	0.8969	1.1721	0.9550	0.8762	0.9751	0.0678
LOC646093	0.8479	1.1448	1.0456	0.8619	0.9751	0.0723
LOC1001335	0.8818	1.0078	1.0253	0.9854	0.9751	0.0321
LOC1001307	0.8902	1.0543	1.0655	0.8902	0.9751	0.0490
TP63	0.9536	1.0616	0.9924	0.8927	0.9751	0.0354
EDN2	1.0109	1.1368	0.9571	0.7954	0.9751	0.0707
LOC727958	0.9206	1.0809	1.0339	0.8649	0.9751	0.0498
LOC652327	0.8602	1.1691	1.0295	0.8415	0.9751	0.0773
TWIST2	0.9268	1.0652	0.9838	0.9245	0.9751	0.0330
SST	0.9759	1.0921	1.0550	0.7775	0.9751	0.0702
ZNF238	0.9271	1.0237	1.0673	0.8824	0.9751	0.0426
LOC643428	0.9802	1.0382	0.9375	0.9446	0.9751	0.0230
REN	1.0140	0.9308	0.9677	0.9879	0.9751	0.0176
CTGLF7	0.8815	1.1688	1.0963	0.7538	0.9751	0.0957
LOC401068	0.8487	1.0545	1.0079	0.9894	0.9751	0.0443
APBB1	0.9199	1.0014	1.0247	0.9545	0.9751	0.0235
LOC283028	0.9841	1.0182	1.0011	0.8970	0.9751	0.0269
CILP2	0.8652	1.1681	1.0110	0.8562	0.9751	0.0735
LOC646575	0.9228	0.9481	0.9910	1.0386	0.9751	0.0254
AGMAT	0.9468	1.0241	0.9899	0.9397	0.9751	0.0197
C6orf222	1.0343	0.9710	1.0417	0.8535	0.9751	0.0435
C2	0.8635	1.0218	1.0198	0.9954	0.9751	0.0377
FXYD1	0.9151	1.2065	0.9819	0.7970	0.9751	0.0861
LOC652725	1.0416	1.0390	0.9212	0.8988	0.9751	0.0379
LOC645227	0.9132	1.0323	1.0110	0.9441	0.9751	0.0279
LOC651714	0.9446	1.0217	1.0191	0.9152	0.9752	0.0268
CNR2	0.9226	1.0799	0.9708	0.9273	0.9752	0.0366
C10orf88	0.8530	1.3455	0.9635	0.7386	0.9752	0.1317
PML	0.9212	1.0758	1.0074	0.8963	0.9752	0.0411
GORASP1	1.1473	1.2611	0.8697	0.6225	0.9752	0.1434
NOL10	0.9497	1.1555	0.9664	0.8291	0.9752	0.0674
CSRP3	0.9554	1.0057	1.0650	0.8745	0.9752	0.0404
EFNA3	0.9786	1.1274	0.9326	0.8619	0.9752	0.0561

LOC730926	0.9093	1.0455	1.0581	0.8876	0.9752	0.0446
ACOXL	0.8342	1.1330	1.0244	0.9090	0.9752	0.0656
FCAMR	0.8995	1.0969	0.9951	0.9092	0.9752	0.0459
PLA2G4C	0.9327	1.0011	1.0305	0.9364	0.9752	0.0242
LOC1001289	0.8645	1.1031	1.0362	0.8969	0.9752	0.0566
LOC651073	0.8978	1.1221	1.0139	0.8669	0.9752	0.0583
LOC1001326	0.8638	1.2122	0.8485	0.9762	0.9752	0.0840
ZXDA	0.9319	1.0474	1.0073	0.9141	0.9752	0.0314
LOC1001320	1.0222	0.9591	1.0582	0.8612	0.9752	0.0432
LOC642960	0.9728	1.1277	0.9158	0.8844	0.9752	0.0540
LOC1001304	0.8479	1.2156	1.1037	0.7335	0.9752	0.1114
TSPAN2	0.9442	0.9948	1.0813	0.8805	0.9752	0.0424
OR1D5	0.9275	1.0704	1.0411	0.8619	0.9752	0.0487
LOC642373	0.9548	1.0317	0.9900	0.9244	0.9752	0.0231
CACNG4	1.0122	1.2792	0.8448	0.7646	0.9752	0.1137
EPB41L4B	0.9099	1.0857	0.9583	0.9470	0.9752	0.0382
LOC284100	0.9389	1.0069	1.0256	0.9294	0.9752	0.0241
LOC729390	0.9288	1.0399	0.9941	0.9380	0.9752	0.0259
MIR380	0.9432	1.0125	1.0452	0.9000	0.9752	0.0329
FLJ32867	0.9668	1.0935	0.9050	0.9356	0.9752	0.0414
ALOX12P2	0.9202	1.1234	0.9788	0.8784	0.9752	0.0535
LOC643102	1.0098	1.0162	1.0347	0.8402	0.9752	0.0453
LOC400456	0.8856	1.0645	1.0226	0.9282	0.9752	0.0413
MED30	0.9384	1.2247	0.9101	0.8277	0.9752	0.0864
FLJ46321	0.9475	1.0717	0.9910	0.8907	0.9752	0.0381
SLC24A5	0.9388	0.9818	1.0309	0.9494	0.9752	0.0207
HCCA2	0.8871	1.2461	0.9416	0.8261	0.9752	0.0933
LOC389904	1.0016	1.0562	1.0232	0.8199	0.9752	0.0530
LOC728129	0.9123	1.1499	1.0027	0.8360	0.9752	0.0674
NLRC4	0.9324	1.1498	0.9966	0.8223	0.9753	0.0684
TEX11	0.9477	1.0664	0.9787	0.9084	0.9753	0.0336
OPRS1	0.9129	1.0305	1.0570	0.9007	0.9753	0.0400
MIRLET7A1	0.8879	1.0327	0.9866	0.9938	0.9753	0.0308
LOC643107	0.8802	1.0854	0.9784	0.9571	0.9753	0.0423
LOC1001344	0.9609	1.0327	1.0828	0.8247	0.9753	0.0561
LOC1001289	0.9360	1.0465	1.0027	0.9159	0.9753	0.0301
LOC653759	0.9182	1.1040	1.0123	0.8667	0.9753	0.0524
SLC4A10	1.0426	1.0265	0.9358	0.8962	0.9753	0.0353
LOC1001285	0.9595	1.0384	1.0115	0.8917	0.9753	0.0323
CFHR4	0.8996	1.0607	1.0057	0.9352	0.9753	0.0360
ZDHHC11B	0.9622	1.0601	0.8737	1.0051	0.9753	0.0393
LOC1001291	1.0512	1.0715	0.9014	0.8770	0.9753	0.0501
LOC648415	0.8770	1.1766	0.9611	0.8865	0.9753	0.0697
LOC651286	0.9980	1.1040	0.9314	0.8678	0.9753	0.0505
PIP5K2A	0.9074	1.0851	1.0964	0.8124	0.9753	0.0694
GOLGA6A	0.9562	1.1010	0.9410	0.9031	0.9753	0.0433

LOC649184	0.9170	1.0736	0.9996	0.9112	0.9753	0.0385
OPRD1	0.8605	1.0899	1.0295	0.9215	0.9753	0.0518
FYN	0.8828	1.0993	1.0037	0.9156	0.9753	0.0486
LOC642693	1.0837	0.9497	1.0645	0.8036	0.9753	0.0644
LOC644348	0.9536	0.9250	1.1219	0.9010	0.9754	0.0500
GPBP1	0.8516	1.3327	0.9621	0.7550	0.9754	0.1264
UBA52	0.9265	1.0804	0.9890	0.9055	0.9754	0.0392
TPSD1	0.9978	1.0320	0.9946	0.8770	0.9754	0.0339
SOSTDC1	0.8926	1.0672	1.0064	0.9353	0.9754	0.0386
OR1D2	0.9270	1.1293	0.9486	0.8966	0.9754	0.0524
COX19	0.9874	1.2001	0.9482	0.7659	0.9754	0.0891
LOC652624	0.6218	1.2036	1.4452	0.6308	0.9754	0.2075
CCL8	0.9876	1.0799	0.9416	0.8924	0.9754	0.0399
LOC649540	0.9968	1.2343	0.9094	0.7610	0.9754	0.0991
LOC1001304	0.9323	1.1546	0.8945	0.9201	0.9754	0.0602
TAS2R19	0.8857	1.0291	1.1170	0.8697	0.9754	0.0593
NCRNA0011	0.9130	1.1151	1.0551	0.8183	0.9754	0.0674
LOC647716	0.9227	1.0449	1.0414	0.8925	0.9754	0.0396
PEX12	0.9670	1.0455	1.0191	0.8699	0.9754	0.0388
LOC729810	1.0549	1.0183	1.0911	0.7373	0.9754	0.0808
TMC5	0.9471	1.0089	1.0201	0.9255	0.9754	0.0231
LOC1001317	0.9408	1.0671	1.0073	0.8864	0.9754	0.0393
GON4L	0.9655	1.0214	1.0530	0.8616	0.9754	0.0420
CCDC144B	0.8508	1.1321	0.9867	0.9321	0.9754	0.0592
CA14	0.8615	1.1105	1.0509	0.8789	0.9754	0.0621
LOC284912	0.8399	1.0847	1.0398	0.9373	0.9754	0.0547
ANK2	0.9146	1.0955	0.9764	0.9152	0.9754	0.0426
CASP14	1.0273	0.9461	1.0422	0.8860	0.9754	0.0365
EPHA8	0.9575	1.0806	0.9962	0.8674	0.9754	0.0442
DCDC1	0.8358	1.1031	1.0928	0.8700	0.9754	0.0711
CNBP	0.6927	1.4502	0.9669	0.7919	0.9754	0.1681
LOC642443	0.8340	1.2215	1.0454	0.8008	0.9754	0.0983
LOC644086	0.9190	1.0222	1.0138	0.9468	0.9754	0.0253
HDGFL1	0.9111	1.0053	1.1085	0.8768	0.9754	0.0520
FAM178B	1.0069	1.0979	0.9116	0.8855	0.9754	0.0484
CACNG6	0.9645	1.1200	0.9554	0.8619	0.9754	0.0535
LOC645006	0.9231	1.0807	1.0371	0.8608	0.9754	0.0506
N6AMT2	0.8440	1.1820	1.0582	0.8177	0.9754	0.0874
C21orf42	0.9937	1.0953	0.9926	0.8202	0.9754	0.0571
LOC284685	0.9727	1.0362	0.9893	0.9037	0.9754	0.0274
LOC651287	0.8829	1.0556	1.0212	0.9421	0.9755	0.0389
SSTR5	1.0124	0.9391	0.9988	0.9515	0.9755	0.0178
PMFBP1	0.9389	1.0546	1.0739	0.8344	0.9755	0.0557
SLC2A13	0.9750	0.9898	1.0058	0.9312	0.9755	0.0160
MIR496	0.9367	1.0081	1.0240	0.9331	0.9755	0.0236
MIRLET7A2	0.9493	0.9989	1.0288	0.9249	0.9755	0.0235

LRP3	0.9414	1.3393	0.8464	0.7747	0.9755	0.1260
CALML5	0.9261	1.0907	1.0333	0.8516	0.9755	0.0535
SNORD91B	0.9134	1.0658	0.9978	0.9248	0.9755	0.0354
LPPR5	0.9437	1.0154	1.0292	0.9136	0.9755	0.0279
FKBP1P1	0.8364	1.2136	0.9657	0.8862	0.9755	0.0837
LOC650657	0.8223	1.0611	1.0150	1.0035	0.9755	0.0526
LOC642622	1.0157	1.0526	0.9283	0.9052	0.9755	0.0350
LOC644779	0.9122	1.0846	0.9753	0.9297	0.9755	0.0387
CRYBB3	0.9123	0.8865	1.1013	1.0018	0.9755	0.0487
C1QTNF7	1.0289	0.9735	0.9893	0.9103	0.9755	0.0247
EXOD1	1.0209	1.0260	0.9620	0.8931	0.9755	0.0311
TMCO6	0.9636	1.0944	0.9938	0.8501	0.9755	0.0503
SNX20	0.9407	1.0657	0.9802	0.9155	0.9755	0.0329
CCNYL3	0.9552	1.0692	1.0072	0.8704	0.9755	0.0421
LOC253039	0.8978	1.1788	0.9627	0.8627	0.9755	0.0709
FLJ43870	0.9644	1.0341	1.0171	0.8865	0.9755	0.0332
C8B	0.9469	1.0821	0.9051	0.9678	0.9755	0.0379
AASDH	0.8784	1.1235	1.0506	0.8496	0.9755	0.0663
ICA1L	0.9233	1.1077	0.9847	0.8863	0.9755	0.0485
FLJ36157	0.9767	1.1022	0.9437	0.8794	0.9755	0.0468
SATL1	0.9869	1.1403	0.9685	0.8064	0.9755	0.0683
FGF12	0.9378	1.0594	0.9623	0.9426	0.9755	0.0285
LOC727935	0.9079	1.0727	0.9679	0.9535	0.9755	0.0348
APBA3	1.0125	1.2330	0.8609	0.7957	0.9755	0.0971
LCE1D	0.9374	1.0265	1.0684	0.8698	0.9755	0.0446
MIR1264	0.8703	1.0503	1.0760	0.9054	0.9755	0.0514
LOC652043	0.9825	0.9011	1.0865	0.9319	0.9755	0.0406
GNB3	0.8987	1.0458	1.0936	0.8641	0.9755	0.0557
DSPP	0.8522	1.0848	1.0065	0.9586	0.9755	0.0486
LOC641518	0.9664	1.0408	0.9394	0.9555	0.9755	0.0225
DPEP3	0.9666	1.0838	0.9506	0.9012	0.9755	0.0387
SERPINB9	0.8303	1.0978	1.0262	0.9479	0.9755	0.0573
DKFZP434B0	0.9713	1.0820	0.9926	0.8565	0.9756	0.0464
SYNC	0.9433	0.9815	1.0953	0.8822	0.9756	0.0449
EIF5AL1	0.8604	1.1000	1.0530	0.8889	0.9756	0.0593
C21orf125	0.9951	1.0669	0.9549	0.8855	0.9756	0.0379
TNNT3	0.9743	1.0725	0.9172	0.9384	0.9756	0.0344
FUT7	1.0383	1.1057	0.9241	0.8343	0.9756	0.0602
LOC643313	0.9345	1.0166	1.0180	0.9333	0.9756	0.0241
LOC642684	0.8976	1.0955	1.0301	0.8792	0.9756	0.0522
CACNA1G	0.9379	1.0487	1.0220	0.8938	0.9756	0.0361
LOC1001306	0.9632	1.1145	0.9436	0.8811	0.9756	0.0495
LOC649094	0.9281	1.0641	0.9661	0.9441	0.9756	0.0305
LOC643993	0.9334	1.0596	1.0630	0.8464	0.9756	0.0526
LOC646971	0.9763	1.0697	1.0754	0.7810	0.9756	0.0687
CADPS	0.9650	1.0598	1.0079	0.8698	0.9756	0.0402

LOC1001342	0.9512	1.1076	0.9759	0.8678	0.9756	0.0497
SERP2	0.8553	1.1197	0.9700	0.9575	0.9756	0.0545
LOC649745	0.9895	1.0636	0.9857	0.8638	0.9756	0.0414
OR6W1P	0.9706	1.0988	0.9513	0.8819	0.9756	0.0452
LOC645141	1.0641	0.9331	1.0602	0.8452	0.9756	0.0531
LOC643580	0.9064	1.0853	1.0205	0.8904	0.9756	0.0466
LOC653040	0.8999	1.0846	1.0163	0.9018	0.9756	0.0454
LOC648152	0.9839	1.0350	0.9946	0.8891	0.9757	0.0309
LOC1001348	0.7589	1.0675	1.1329	0.9433	0.9757	0.0823
DCDC2	1.0071	1.0463	0.9558	0.8934	0.9757	0.0331
LOC642671	0.9768	1.0591	0.9442	0.9226	0.9757	0.0300
DNM1P35	0.9145	1.0133	1.0108	0.9640	0.9757	0.0233
SEMA6D	0.9485	1.0308	0.9895	0.9338	0.9757	0.0218
LOC402670	0.9741	0.9465	1.0370	0.9450	0.9757	0.0215
TEX28	0.9660	0.9889	0.9454	1.0025	0.9757	0.0126
LOC1001309	0.9848	1.0639	0.9777	0.8763	0.9757	0.0385
LOC123876	0.9037	1.0552	1.0264	0.9175	0.9757	0.0381
LOC643147	0.9018	1.0659	1.0304	0.9046	0.9757	0.0425
RPS18	0.8506	1.1005	1.1882	0.7636	0.9757	0.1006
ATOX1	0.8333	1.4222	0.9274	0.7199	0.9757	0.1548
NPR1	0.8954	1.0772	1.1048	0.8255	0.9757	0.0683
CYP2C18	0.8427	1.0946	1.0328	0.9328	0.9757	0.0555
LOC1001297	0.7533	1.1435	1.0207	0.9854	0.9757	0.0815
LOC441127	0.8814	1.0641	1.0123	0.9452	0.9757	0.0398
CASP1	0.9314	1.0656	0.9892	0.9168	0.9757	0.0338
LOC646324	0.9363	0.9576	1.1148	0.8943	0.9757	0.0482
ANKRD34B	0.9245	1.0658	0.9752	0.9375	0.9757	0.0319
CPNE3	0.7789	1.3581	1.0242	0.7418	0.9758	0.1420
LEAP2	0.9123	1.1046	0.9840	0.9021	0.9758	0.0467
LOC1001307	0.9636	1.0081	1.0124	0.9189	0.9758	0.0219
BMP2KL	0.9591	1.0869	0.9992	0.8578	0.9758	0.0475
S100A10	0.8648	1.1634	0.9989	0.8760	0.9758	0.0695
C1orf84	0.8852	1.1228	1.0033	0.8918	0.9758	0.0560
LOC401957	0.8996	1.1062	1.0711	0.8261	0.9758	0.0673
LOC646706	0.9469	1.1340	0.9649	0.8573	0.9758	0.0578
POLR1A	0.9646	1.0976	1.0326	0.8082	0.9758	0.0621
LOC1001319	0.8922	1.0480	0.9931	0.9699	0.9758	0.0323
PLK5P	0.8583	1.0239	1.0526	0.9683	0.9758	0.0429
KRTAP5-8	0.9270	0.9749	1.0468	0.9544	0.9758	0.0256
LOC653394	0.9570	1.0821	0.9222	0.9418	0.9758	0.0361
LOC648278	0.9561	1.1035	0.9065	0.9371	0.9758	0.0438
FLJ39739	0.8985	1.0388	1.0484	0.9175	0.9758	0.0394
EPHA5	0.9736	1.0272	1.0089	0.8935	0.9758	0.0296
NF1	0.8840	1.1087	1.0231	0.8874	0.9758	0.0549
LOC1001337	0.9281	1.0944	0.9755	0.9051	0.9758	0.0422
LOC646346	0.9281	1.0774	1.0663	0.8314	0.9758	0.0589

XRCC4	0.8721	1.0833	1.0123	0.9354	0.9758	0.0459
SYT6	0.9244	0.9935	1.0932	0.8922	0.9758	0.0445
CCDC37	0.9299	1.0823	0.9509	0.9401	0.9758	0.0357
LOC649940	0.9934	0.9543	1.0013	0.9543	0.9758	0.0125
LOC442597	1.1257	0.9302	0.9624	0.8850	0.9758	0.0524
LOC1001282	0.9153	1.0675	1.0325	0.8879	0.9758	0.0438
LOC729798	0.9270	1.1893	1.0165	0.7706	0.9758	0.0874
CSHL1	0.9449	1.1006	1.0191	0.8387	0.9758	0.0557
TMEM202	0.9207	1.0898	0.9292	0.9636	0.9758	0.0391
MMP28	0.9118	1.0706	1.0059	0.9150	0.9758	0.0384
LOC1001306	0.8923	1.1085	1.0074	0.8952	0.9758	0.0517
NPPB	0.8394	1.0226	1.1553	0.8861	0.9758	0.0713
LOC1001312	0.8792	1.1615	0.9492	0.9134	0.9758	0.0635
LOC401463	0.9614	1.0651	0.9967	0.8802	0.9758	0.0385
ANXA5	0.6354	1.5103	1.0424	0.7152	0.9758	0.1987
BRWD3	0.9022	1.0703	1.0143	0.9166	0.9758	0.0401
C22orf33	0.8907	1.1618	0.9687	0.8822	0.9759	0.0650
MGC15763	0.9215	1.2276	0.8949	0.8594	0.9759	0.0849
LOC342531	0.8945	1.0254	1.0761	0.9074	0.9759	0.0445
LOC645197	0.9078	1.0946	1.0442	0.8568	0.9759	0.0560
C8orf62	0.9709	0.9857	1.0460	0.9008	0.9759	0.0298
HCN3	0.8919	0.9941	1.1245	0.8929	0.9759	0.0550
SLC25A33	0.9565	1.0627	0.9545	0.9298	0.9759	0.0296
GABPAP	0.9661	1.0753	0.9665	0.8956	0.9759	0.0371
LOC647919	0.9323	1.1322	1.0309	0.8081	0.9759	0.0692
IL16	0.8652	1.0094	1.0981	0.9309	0.9759	0.0503
LOC1001301	0.9300	1.1405	0.9569	0.8762	0.9759	0.0574
MTUS2	0.8926	1.0945	1.0290	0.8875	0.9759	0.0513
AMPD2	0.9423	1.0667	0.9609	0.9336	0.9759	0.0308
MACROD2	0.9643	1.0694	1.0122	0.8576	0.9759	0.0449
LOC1001339	0.9935	1.0540	0.8910	0.9651	0.9759	0.0338
LOC649501	0.9012	1.1256	0.9843	0.8925	0.9759	0.0540
LOC644666	0.8903	1.0649	1.0631	0.8854	0.9759	0.0509
LOC653753	0.9406	1.0216	0.9897	0.9518	0.9759	0.0185
LOC441296	0.9975	1.0725	1.0351	0.7985	0.9759	0.0611
LOC646932	0.8708	1.0946	1.0177	0.9205	0.9759	0.0500
LOC649935	0.9626	1.0457	1.0599	0.8355	0.9759	0.0515
LOC653105	0.9618	1.0544	0.9739	0.9135	0.9759	0.0292
ERCC-00073	0.8493	1.0718	1.0366	0.9460	0.9759	0.0498
CYBB	0.8751	1.1016	0.9804	0.9466	0.9759	0.0473
TIMP1	0.9945	1.0097	1.0209	0.8786	0.9759	0.0329
KDM2B	1.0027	1.1688	0.8934	0.8389	0.9759	0.0727
LOC440067	0.9312	1.0342	0.9111	1.0274	0.9759	0.0319
LDHC	0.9226	1.0742	1.0096	0.8974	0.9760	0.0406
LOC650038	0.8873	1.0431	0.9863	0.9871	0.9760	0.0324
ACTRT1	0.8157	1.2281	0.9724	0.8876	0.9760	0.0899

CDK5	1.0463	1.2662	0.8805	0.7108	0.9760	0.1185
LOC1001344	0.9242	1.0149	1.0209	0.9438	0.9760	0.0246
LOC653458	0.9031	1.0410	0.9820	0.9777	0.9760	0.0282
PKD1L2	0.9189	1.0658	1.0176	0.9017	0.9760	0.0393
LOC646434	0.8853	1.2137	0.9236	0.8812	0.9760	0.0798
MAP3K2	0.8457	1.0884	1.1074	0.8624	0.9760	0.0706
GLYCAM1	1.0174	1.0532	0.9461	0.8873	0.9760	0.0370
LOC652870	0.9463	1.0301	0.9126	1.0150	0.9760	0.0279
LOC654220	0.9305	1.0516	0.9074	1.0145	0.9760	0.0341
SORCS1	0.9571	1.0878	0.9543	0.9047	0.9760	0.0392
ELSPBP1	0.9126	1.0580	1.0946	0.8388	0.9760	0.0603
LOC1001317	0.9458	1.0177	1.0573	0.8833	0.9760	0.0386
LOC653547	1.0467	1.0201	0.9882	0.8490	0.9760	0.0440
NANOS1	0.9002	1.0895	1.0660	0.8483	0.9760	0.0599
COL6A2	0.9560	1.0323	1.0604	0.8554	0.9760	0.0459
GABRG2	0.9021	1.1030	0.9717	0.9272	0.9760	0.0447
PRR4	0.8369	1.2394	0.9802	0.8476	0.9760	0.0936
DKFZP781G0	0.8997	1.0532	1.0412	0.9099	0.9760	0.0412
EXOC3L2	0.9930	1.0424	0.9316	0.9371	0.9760	0.0261
OR5C1	0.9537	1.1389	0.9226	0.8889	0.9760	0.0559
ITGAV	0.7581	1.2849	1.1527	0.7085	0.9760	0.1431
LOC1001299	0.9487	1.1081	0.9821	0.8653	0.9760	0.0504
LOC1001288	0.8971	1.0583	1.0517	0.8970	0.9760	0.0456
REL	0.9632	0.9834	1.0777	0.8799	0.9760	0.0406
PCID2	0.7375	1.4789	0.8841	0.8037	0.9761	0.1703
LOC646021	0.9153	1.1000	0.9455	0.9435	0.9761	0.0419
LOC646043	0.9720	1.2267	0.9062	0.7994	0.9761	0.0908
RGPD5	0.9351	1.1215	0.9626	0.8851	0.9761	0.0511
LOC1001346	0.9595	1.1571	0.8582	0.9296	0.9761	0.0640
LOC644767	0.8572	1.0333	1.0406	0.9732	0.9761	0.0424
LOC645188	0.9727	1.0257	0.9838	0.9222	0.9761	0.0213
LOC642408	0.9904	1.0628	1.0419	0.8093	0.9761	0.0577
C3orf58	0.9809	1.0571	1.0659	0.8005	0.9761	0.0616
LOC646667	0.8810	1.0046	1.0260	0.9927	0.9761	0.0324
BRSK1	0.9286	1.1918	0.8972	0.8868	0.9761	0.0724
LOC728572	0.8559	0.9574	1.1737	0.9174	0.9761	0.0691
LOC647211	0.9472	1.1491	0.9719	0.8361	0.9761	0.0648
LOC641851	0.9224	0.9600	1.1206	0.9015	0.9761	0.0497
LOC729941	0.9588	1.1350	0.9653	0.8453	0.9761	0.0597
ANKRD2	0.8871	1.0032	1.1097	0.9044	0.9761	0.0514
HAND1	0.9551	0.9516	1.0051	0.9926	0.9761	0.0134
FLJ14186	0.9130	1.0619	0.9687	0.9609	0.9761	0.0311
LOC646870	0.9520	1.1383	0.8738	0.9404	0.9761	0.0567
GPR158	0.9530	1.0785	0.9768	0.8962	0.9761	0.0381
C6orf184	0.9310	1.0591	0.9759	0.9384	0.9761	0.0293
SH3GL2	0.9441	1.0354	0.9639	0.9610	0.9761	0.0203

FBN3	0.9004	1.0683	1.0283	0.9075	0.9761	0.0425
LOC642759	1.0166	1.0222	0.8832	0.9825	0.9761	0.0322
KCNJ10	0.8811	1.1258	0.8425	1.0551	0.9761	0.0680
LOC1001342	0.8712	1.1784	0.9090	0.9460	0.9761	0.0691
UNC93B1	0.8258	1.2865	1.0520	0.7402	0.9761	0.1226
LOC1001283	0.9575	1.1715	1.0447	0.7309	0.9761	0.0928
MIR1185-1	0.8698	1.1676	1.0000	0.8672	0.9761	0.0709
LOC728530	0.8079	1.1287	1.0314	0.9366	0.9762	0.0684
LOC728901	0.9109	0.9872	1.0584	0.9481	0.9762	0.0315
LOC729397	0.9705	1.0141	1.0131	0.9069	0.9762	0.0252
C3orf60	0.9248	1.1040	1.0243	0.8516	0.9762	0.0554
LOC729601	0.9465	1.0650	0.9913	0.9019	0.9762	0.0348
APOC3	0.9233	1.0485	1.0261	0.9067	0.9762	0.0358
CLEC1A	0.9762	1.0498	0.9781	0.9006	0.9762	0.0305
PRODH2	0.8612	1.0779	1.0794	0.8863	0.9762	0.0594
LOC389257	0.9795	1.0458	0.8353	1.0440	0.9762	0.0494
LILRA6	0.9574	1.0343	0.9991	0.9139	0.9762	0.0260
MYL12A	0.8472	1.1885	1.0895	0.7795	0.9762	0.0972
LOC643847	0.9159	1.0407	1.0332	0.9149	0.9762	0.0351
MAML2	0.9467	1.0548	0.9583	0.9450	0.9762	0.0264
LOC400578	0.9480	1.0856	0.9964	0.8747	0.9762	0.0442
LOC1001318	0.9798	1.1324	1.0049	0.7877	0.9762	0.0712
BCL2L14	0.9321	1.0701	0.9579	0.9447	0.9762	0.0318
RSPO3	0.9161	1.0714	1.0377	0.8796	0.9762	0.0464
LOC391746	0.9569	1.1469	0.9479	0.8532	0.9762	0.0615
ASGR2	0.9353	1.1171	0.9310	0.9215	0.9762	0.0471
LOC162632	0.9215	1.0754	1.0048	0.9032	0.9762	0.0398
OVOS2	0.9508	1.0445	1.0212	0.8884	0.9762	0.0354
ELFN2	0.9059	1.1113	0.9821	0.9056	0.9762	0.0485
MGC87042	0.8846	1.3016	1.0181	0.7005	0.9762	0.1265
LOC1001299	0.8871	1.1411	0.9945	0.8822	0.9762	0.0608
LOC642645	0.8989	0.9655	1.0588	0.9817	0.9762	0.0328
LOC1001290	1.0438	1.1317	0.8729	0.8566	0.9762	0.0669
XRRA1	0.9915	0.9851	1.0689	0.8594	0.9762	0.0433
MEIS2	0.9726	1.0507	0.9491	0.9326	0.9763	0.0261
EXOC6B	0.9317	1.0372	0.9925	0.9435	0.9763	0.0242
CHRDL2	0.8618	1.1100	1.0573	0.8759	0.9763	0.0630
LOC654259	0.8330	1.1391	1.0638	0.8692	0.9763	0.0742
FRMPD2L1	0.8953	1.1069	0.9973	0.9056	0.9763	0.0492
SPANXF1	0.8913	1.1105	0.9707	0.9325	0.9763	0.0476
PNLIPRP3	0.8891	1.0222	1.0270	0.9668	0.9763	0.0321
LOC1001344	0.8969	1.0334	1.0687	0.9062	0.9763	0.0438
MAGEC3	0.9563	1.0443	0.9704	0.9342	0.9763	0.0238
NFIX	0.9135	1.2440	0.9926	0.7551	0.9763	0.1020
LOC649762	0.8592	1.0814	0.9876	0.9770	0.9763	0.0455
LOC1001339	0.8753	1.0879	0.9345	1.0075	0.9763	0.0460

NKPD1	0.9223	1.0135	1.0835	0.8859	0.9763	0.0447
LOC643130	0.9300	1.1423	0.9621	0.8708	0.9763	0.0585
LOC374768	0.8108	1.0325	1.0996	0.9624	0.9763	0.0619
SDCCAG8	0.9496	1.1061	0.9770	0.8726	0.9763	0.0486
SERPINA2	0.9425	1.0219	1.0060	0.9349	0.9763	0.0220
LOC653904	0.9228	1.1348	0.8964	0.9513	0.9763	0.0540
ZNF705D	0.9436	1.1327	0.9404	0.8886	0.9763	0.0536
EPHB6	0.8977	1.1450	1.0058	0.8569	0.9763	0.0644
LOC1001322	0.8857	1.1480	0.9624	0.9092	0.9763	0.0594
LOC723805	0.9018	1.1878	0.9717	0.8440	0.9763	0.0752
CAGE1	0.9450	1.0350	1.0360	0.8893	0.9763	0.0360
BRAP	0.9390	1.0689	0.9351	0.9624	0.9763	0.0314
OR6C65	0.8732	1.0106	1.0009	1.0206	0.9763	0.0346
GPRC5D	0.9634	1.1050	1.0251	0.8119	0.9763	0.0620
LGR8	0.9606	0.9596	1.0082	0.9770	0.9764	0.0113
ANGPTL4	0.9784	1.0925	0.9846	0.8500	0.9764	0.0496
LOC653252	0.9485	1.0449	0.9526	0.9594	0.9764	0.0230
RBMY2FP	0.9306	1.1037	1.0525	0.8186	0.9764	0.0639
RG9MTD2	0.9150	1.1711	1.0170	0.8024	0.9764	0.0783
ALKBH7	0.7158	1.3296	1.0751	0.7850	0.9764	0.1411
FAM123A	0.8822	1.0734	0.9934	0.9565	0.9764	0.0398
FLJ44790	0.9153	1.1734	0.9225	0.8943	0.9764	0.0659
PTGS1	0.9198	1.1149	1.0671	0.8038	0.9764	0.0709
FTCD	0.9726	1.0763	0.9545	0.9022	0.9764	0.0365
LOC285047	0.9587	0.9905	1.0825	0.8739	0.9764	0.0431
THSD3	0.8885	1.1268	1.0094	0.8810	0.9764	0.0581
TSSC4	0.8584	1.3854	0.8906	0.7712	0.9764	0.1386
FAM78A	0.8818	1.0093	1.0939	0.9206	0.9764	0.0474
SNORA30	0.9313	1.0218	1.0351	0.9173	0.9764	0.0303
NUFIP2	0.7849	1.3193	1.0611	0.7404	0.9764	0.1345
FKBP14	0.8718	1.2786	0.9663	0.7889	0.9764	0.1070
LOC642574	0.8948	1.0396	1.0401	0.9313	0.9764	0.0374
ECHS1	0.7088	1.4079	1.0943	0.6948	0.9764	0.1710
LOC729837	0.8608	1.0953	1.0175	0.9322	0.9764	0.0509
LOC649722	0.9430	1.0791	0.9863	0.8973	0.9764	0.0388
LOC728317	0.9955	1.0367	0.9974	0.8761	0.9764	0.0348
ADCYAP1R1	0.8881	0.9672	1.0869	0.9636	0.9764	0.0411
LOC1001326	0.9206	1.0436	1.1159	0.8257	0.9765	0.0644
MYEOV	0.9416	1.0822	0.9999	0.8822	0.9765	0.0427
LOC644846	0.9517	1.0584	0.9705	0.9252	0.9765	0.0289
LOC645408	1.0940	0.9561	0.9151	0.9407	0.9765	0.0401
MGC40170	1.0432	1.1109	0.8810	0.8707	0.9765	0.0598
CPB1	0.9107	1.1342	0.9983	0.8627	0.9765	0.0596
HBS1L	0.9467	1.0969	1.0096	0.8527	0.9765	0.0515
TPSB2	0.9544	1.1539	0.8481	0.9495	0.9765	0.0640
FUT3	0.9712	1.1189	0.8767	0.9391	0.9765	0.0514

PDX1	0.9270	1.0077	1.1564	0.8148	0.9765	0.0719
KNCN	0.8644	1.0918	1.0412	0.9086	0.9765	0.0537
C16orf90	0.9066	1.0598	0.9840	0.9556	0.9765	0.0320
LOC643813	1.0209	0.9891	1.0201	0.8758	0.9765	0.0344
FLJ40453	0.9232	1.0340	1.0587	0.8900	0.9765	0.0412
LOC647447	0.9326	1.0341	1.0211	0.9182	0.9765	0.0298
DENND4A	0.9565	1.1428	0.9651	0.8416	0.9765	0.0622
LOC653272	0.9123	1.1680	0.9361	0.8896	0.9765	0.0645
LOC642969	1.0190	1.0572	1.0201	0.8097	0.9765	0.0563
FLJ42957	0.9889	0.9877	1.0211	0.9082	0.9765	0.0240
SNTB1	0.9631	1.0522	0.9479	0.9429	0.9765	0.0256
NCRNA0018[illegible]	0.9396	1.0538	0.9907	0.9219	0.9765	0.0296
LOC1001313[illegible]	0.8500	1.1035	1.0674	0.8851	0.9765	0.0637
RAD23A	0.8926	1.2845	1.0670	0.6620	0.9765	0.1320
DEFB133	1.0319	0.9938	0.9461	0.9343	0.9765	0.0225
HOXA2	0.9054	1.1096	0.9980	0.8930	0.9765	0.0502
IGLL1	0.9246	1.0257	1.0678	0.8881	0.9765	0.0421
SACS	0.8937	1.1164	1.0399	0.8561	0.9765	0.0612
LOC651758	1.0189	1.1465	0.9194	0.8214	0.9765	0.0695
PSMD12	0.8580	1.3177	1.0186	0.7119	0.9766	0.1298
LOC728924	0.9596	1.1271	1.0247	0.7949	0.9766	0.0697
LOC727916	0.9709	1.0551	0.9711	0.9091	0.9766	0.0300
MED13L	0.9922	1.1157	0.9751	0.8234	0.9766	0.0599
MYH16	0.9124	1.0561	1.0082	0.9295	0.9766	0.0337
KRTAP17-1	0.9352	0.9859	1.0474	0.9378	0.9766	0.0263
LOC1001322[illegible]	0.9291	1.1187	0.9166	0.9419	0.9766	0.0477
LOC401087	0.9525	1.2197	0.9313	0.8028	0.9766	0.0875
FLJ43080	0.8635	1.1068	1.0366	0.8994	0.9766	0.0572
LOC645289	0.9881	1.0876	0.9861	0.8446	0.9766	0.0500
C7orf45	0.9073	1.0934	0.9787	0.9270	0.9766	0.0417
LOC654208	0.9415	0.9914	1.0443	0.9292	0.9766	0.0263
LOC1001307[illegible]	0.8930	1.2725	1.0415	0.6995	0.9766	0.1209
C6orf195	0.8658	1.0686	1.0153	0.9569	0.9766	0.0434
GSTA3	0.9426	1.0980	0.9736	0.8923	0.9766	0.0438
LAIR2	0.8755	1.0107	1.0392	0.9811	0.9766	0.0357
SH2D1A	0.9147	1.1058	1.0503	0.8357	0.9766	0.0618
LOC643985	0.9155	1.0621	1.1161	0.8129	0.9766	0.0691
LOC345222	0.9939	1.0643	1.1270	0.7214	0.9766	0.0893
LOC653136	0.9370	1.0807	0.9473	0.9416	0.9767	0.0347
LOC646119	0.9409	1.0344	1.0237	0.9076	0.9767	0.0311
C1orf151	0.8229	1.1772	1.0389	0.8677	0.9767	0.0814
RIMBP3C	0.9102	1.0293	1.0135	0.9537	0.9767	0.0275
LOC654023	0.9919	1.0958	0.9059	0.9131	0.9767	0.0442
MGC4294	0.9421	1.0457	0.9824	0.9365	0.9767	0.0252
LOC648827	0.9872	1.0270	0.9817	0.9109	0.9767	0.0241
LOC727951	0.7960	1.1212	1.0230	0.9665	0.9767	0.0682

LOC389787	0.7868	1.0670	1.4121	0.6408	0.9767	0.1699
LOC1001303	0.9225	1.1694	0.8784	0.9365	0.9767	0.0654
LOC642164	0.8933	1.1069	0.9768	0.9297	0.9767	0.0467
ALPK3	1.0486	1.0204	0.9582	0.8796	0.9767	0.0375
LOC646934	0.8930	1.0550	1.0454	0.9133	0.9767	0.0427
LOC1001325	0.8620	1.0233	1.2873	0.7342	0.9767	0.1192
FRY	0.9676	1.0562	0.9801	0.9028	0.9767	0.0315
GPR123	0.9518	1.0550	0.9818	0.9182	0.9767	0.0292
LOC647541	0.8793	1.1288	0.9974	0.9012	0.9767	0.0568
LOC1001332	0.8522	1.1649	1.1221	0.7676	0.9767	0.0982
CYP7B1	0.9614	1.0322	1.0110	0.9022	0.9767	0.0289
LOC651012	1.0222	0.9192	0.9617	1.0037	0.9767	0.0230
LOC389442	1.0442	1.0414	0.9809	0.8403	0.9767	0.0478
MFSD4	0.9682	1.0942	1.0185	0.8259	0.9767	0.0566
DDX19-DDX1	0.9512	1.1567	0.8400	0.9589	0.9767	0.0659
LOC645393	0.9768	0.9639	1.1142	0.8520	0.9767	0.0537
C3orf43	0.9845	1.0876	0.9966	0.8382	0.9767	0.0516
LOC731823	0.9375	1.0446	0.9396	0.9852	0.9767	0.0252
CCDC105	0.9494	1.0632	1.0094	0.8850	0.9767	0.0384
NBEAL1	0.8778	1.2034	1.0218	0.8040	0.9767	0.0881
LOC1001310	0.9248	1.0801	1.0556	0.8463	0.9767	0.0552
FLJ25076	0.9603	1.0660	1.0280	0.8527	0.9767	0.0468
RP1L1	0.9606	1.0866	0.9738	0.8859	0.9767	0.0414
ARHGAP4	0.9265	1.1993	0.9565	0.8246	0.9767	0.0794
LOC255809	0.8286	1.1000	0.9822	0.9961	0.9767	0.0559
LOC642419	0.8944	1.1721	0.9601	0.8803	0.9767	0.0674
LOC644253	0.9358	1.0027	0.9981	0.9703	0.9767	0.0154
LOC642853	0.9596	1.1489	0.8463	0.9521	0.9767	0.0630
OR6N2	0.9876	0.9787	0.9790	0.9617	0.9767	0.0054
LOC255313	0.8815	0.9776	1.0422	1.0057	0.9767	0.0344
TSPAN32	0.9433	1.0698	0.9685	0.9255	0.9767	0.0322
OR56B4	0.9472	0.9962	1.0696	0.8940	0.9768	0.0373
PARP4	0.8750	1.4047	0.9676	0.6597	0.9768	0.1566
HYDIN	0.9502	1.0488	1.0059	0.9022	0.9768	0.0320
C2orf27A	1.0198	1.0014	0.9959	0.8899	0.9768	0.0294
CAPN10	0.9513	1.0693	1.0181	0.8683	0.9768	0.0435
ZNF663	0.9321	1.0124	1.0556	0.9069	0.9768	0.0346
LOC284688	0.8648	1.0799	1.0456	0.9167	0.9768	0.0513
LOC1001320	0.8817	1.0834	1.0440	0.8980	0.9768	0.0509
ESAM	0.9557	1.0010	1.0379	0.9125	0.9768	0.0272
LOC730496	0.8545	1.1372	1.0938	0.8217	0.9768	0.0808
LOC653899	0.9938	0.9715	0.9887	0.9532	0.9768	0.0092
MIR220C	0.8832	1.0019	1.0760	0.9460	0.9768	0.0410
HSFY2	0.9597	1.0537	1.0011	0.8927	0.9768	0.0340
CD163	0.9006	1.0753	0.9925	0.9387	0.9768	0.0379
GALNT17	0.9265	0.9591	0.9747	1.0470	0.9768	0.0255

PPP4R4	0.9376	1.0344	1.0224	0.9128	0.9768	0.0303
OR4L1	0.9171	1.0426	1.0221	0.9254	0.9768	0.0324
SSX6	0.9164	1.0440	1.0509	0.8959	0.9768	0.0410
LOC646303	0.9219	1.0662	1.0257	0.8934	0.9768	0.0412
COLQ	0.9081	1.0871	1.0340	0.8781	0.9768	0.0499
FLJ34690	0.9532	0.9749	1.0677	0.9114	0.9768	0.0330
ALS2CR16	0.9140	1.0271	0.9756	0.9906	0.9768	0.0236
CLTA	0.8554	1.3698	0.9086	0.7735	0.9768	0.1339
LOC728142	0.9439	1.1755	0.9950	0.7928	0.9768	0.0789
LOC643594	0.9393	0.9248	1.1005	0.9427	0.9768	0.0414
SPIB	0.8768	1.1473	1.0422	0.8412	0.9768	0.0717
ERCC-00069	1.0739	1.0615	0.8870	0.8850	0.9768	0.0525
LOC653387	0.9382	1.0430	1.0048	0.9215	0.9768	0.0284
GATM	0.9328	1.0624	0.9570	0.9551	0.9768	0.0290
KIAA1984	0.9870	1.1270	0.9495	0.8439	0.9768	0.0585
LOC643341	0.9704	0.9919	0.9742	0.9709	0.9768	0.0051
OR13F1	0.9541	1.0252	1.0078	0.9203	0.9769	0.0241
MIR152	0.8408	1.1038	1.0890	0.8738	0.9769	0.0694
MIR7-2	0.9175	1.0353	0.9782	0.9764	0.9769	0.0241
GNLY	0.8857	1.0598	1.0603	0.9016	0.9769	0.0482
LOC1001305	0.9931	1.0238	0.9726	0.9179	0.9769	0.0223
FLJ46446	1.0909	0.9608	0.9695	0.8862	0.9769	0.0424
LOC442270	0.9691	1.1511	0.9651	0.8221	0.9769	0.0674
SYCE1L	0.9479	1.0782	1.0072	0.8742	0.9769	0.0434
CLK2P	0.9489	1.1761	0.9135	0.8690	0.9769	0.0684
LOC653493	0.8640	1.0187	1.0332	0.9917	0.9769	0.0386
NOX4	0.9073	1.0800	1.0118	0.9085	0.9769	0.0422
PRRT1	0.8752	1.0798	1.0976	0.8551	0.9769	0.0648
MPP3	1.0638	1.0001	0.9725	0.8712	0.9769	0.0401
SCGB2A2	1.0003	0.9968	1.0228	0.8877	0.9769	0.0303
LOC390251	0.9519	1.0007	0.9926	0.9626	0.9769	0.0117
CRNKL1	0.9057	1.1854	0.9221	0.8945	0.9769	0.0697
LOC728211	0.8828	1.1486	0.9637	0.9126	0.9769	0.0596
IL12RB1	0.9508	1.0986	1.0283	0.8300	0.9769	0.0575
KIAA1210	0.9106	1.1574	0.9629	0.8768	0.9769	0.0627
N4BP3	0.9696	1.0686	1.0113	0.8582	0.9769	0.0445
LPCAT1	1.0207	1.2081	1.0225	0.6563	0.9769	0.1156
LOC400965	0.9018	1.1209	1.0743	0.8107	0.9769	0.0727
LOC644489	0.8791	1.1597	0.9351	0.9338	0.9769	0.0623
P2RY6	0.9298	1.0277	1.0190	0.9312	0.9769	0.0269
LOC644602	0.9253	0.9444	1.0637	0.9743	0.9769	0.0306
LOC653037	0.9652	1.0845	0.9592	0.8989	0.9769	0.0389
OTOA	0.9221	1.0359	1.0091	0.9407	0.9769	0.0271
LOC650687	1.0274	0.9750	1.0270	0.8784	0.9769	0.0351
LOC1001295	0.9217	1.0490	1.0197	0.9175	0.9770	0.0337
LOC440422	1.0032	1.0350	1.0268	0.8428	0.9770	0.0452

RMST	0.8985	1.0958	1.0563	0.8574	0.9770	0.0584
LOC1001299	0.9783	1.0202	1.0574	0.8520	0.9770	0.0447
LOC1001334	0.9772	1.0678	1.0450	0.8179	0.9770	0.0564
C1orf83	1.0237	1.0957	0.9689	0.8196	0.9770	0.0585
LOC642351	0.9188	1.0063	1.1156	0.8672	0.9770	0.0544
LOC643206	0.8671	1.1740	0.8876	0.9792	0.9770	0.0700
CALY	0.9320	1.1541	0.9457	0.8761	0.9770	0.0609
LOC1001342	1.0386	0.9811	1.0204	0.8678	0.9770	0.0383
LOC1001321	0.9128	1.0498	1.0597	0.8857	0.9770	0.0453
PNPLA8	0.9117	1.1474	1.0782	0.7706	0.9770	0.0847
LOC728765	0.9758	1.0102	1.0177	0.9044	0.9770	0.0259
TLE4	0.9750	1.0584	0.9579	0.9167	0.9770	0.0298
LOC727781	0.9341	1.0418	0.9822	0.9499	0.9770	0.0238
LOC652712	0.9008	1.1489	1.0223	0.8360	0.9770	0.0691
STK3	0.7575	1.3628	1.1029	0.6848	0.9770	0.1577
MIR99B	0.9368	1.0977	1.0242	0.8493	0.9770	0.0538
LOC648277	0.9485	1.1794	0.9658	0.8144	0.9770	0.0755
LOC1001287	0.9508	1.0720	0.9878	0.8975	0.9770	0.0367
LOC729679	0.6214	1.3888	1.1432	0.7547	0.9770	0.1763
LOC1001315	0.9232	1.0186	1.0934	0.8729	0.9770	0.0492
SHC2	0.8912	1.0969	1.0101	0.9099	0.9770	0.0477
LCN6	0.9374	1.1044	0.9082	0.9581	0.9770	0.0437
LOC339760	0.9186	1.1476	1.0522	0.7898	0.9770	0.0781
PRKD2	1.0347	1.0751	1.0260	0.7723	0.9770	0.0691
LOC644928	0.8363	1.2286	1.1658	0.6775	0.9770	0.1318
ERCC-00077	0.9752	0.9833	1.0561	0.8936	0.9770	0.0332
HIST1H4J	0.9820	0.9280	1.1116	0.8866	0.9770	0.0489
LOC1001311	0.8699	1.1647	0.9628	0.9108	0.9770	0.0654
OR7D4	1.0458	0.8969	1.0314	0.9341	0.9771	0.0365
CLEC4C	0.8930	1.0609	1.0562	0.8982	0.9771	0.0471
LOC645282	0.9521	1.1534	0.9695	0.8332	0.9771	0.0661
FDXACB1	0.9610	1.1129	0.9947	0.8397	0.9771	0.0562
LOC440438	0.9159	1.0793	0.9457	0.9673	0.9771	0.0357
LOC645027	0.9769	1.0069	1.0312	0.8933	0.9771	0.0300
SNORA11C	0.9015	1.1105	0.9860	0.9103	0.9771	0.0484
CAPN9	1.0011	1.0842	0.9612	0.8618	0.9771	0.0462
LOC729150	0.9100	0.9314	1.0118	1.0552	0.9771	0.0340
C14orf1	1.0618	0.9853	0.9566	0.9046	0.9771	0.0328
SYT10	0.9568	0.9313	1.0287	0.9915	0.9771	0.0212
LOC1001291	0.8131	1.0757	1.0021	1.0174	0.9771	0.0569
APEG1	0.7708	1.0902	1.0122	1.0351	0.9771	0.0707
IFNA13	0.9313	1.0508	1.0085	0.9178	0.9771	0.0317
LOC652441	0.9750	1.0242	1.0467	0.8625	0.9771	0.0410
RGL3	0.9794	1.1356	0.9677	0.8257	0.9771	0.0633
LOC1001308	0.9462	1.0137	1.0221	0.9265	0.9771	0.0240
ASCL1	0.9707	0.9828	1.0660	0.8889	0.9771	0.0362

SIN3A	0.7547	1.4790	0.8931	0.7816	0.9771	0.1700
TTC8	0.7175	1.5138	0.8622	0.8150	0.9771	0.1814
ITGB4	0.9484	1.0461	1.0136	0.9004	0.9771	0.0327
LOC644624	0.9362	1.1136	0.9467	0.9120	0.9771	0.0461
PIK3C3	1.0033	1.0423	1.1728	0.6901	0.9771	0.1023
ENTPD8	0.9316	1.1225	0.9921	0.8623	0.9771	0.0552
LOC1001321	0.9343	1.0991	0.9658	0.9093	0.9771	0.0423
CPLX4	0.8948	1.1315	0.9707	0.9116	0.9771	0.0540
PSMB9	0.9806	1.0516	0.9943	0.8821	0.9771	0.0352
SLC25A41	0.8945	1.0396	1.0651	0.9095	0.9772	0.0438
AMPD3	0.9372	1.0697	0.9961	0.9056	0.9772	0.0361
MIR632	0.8552	1.2047	1.0334	0.8154	0.9772	0.0894
LOC652405	0.8381	1.0352	1.1278	0.9076	0.9772	0.0647
C9orf38	0.9072	1.0789	1.0123	0.9102	0.9772	0.0418
LOC729177	0.8172	1.1196	1.0787	0.8931	0.9772	0.0726
RNU12	0.9324	1.0339	0.9923	0.9501	0.9772	0.0227
LOC645040	0.9574	0.9466	1.0481	0.9567	0.9772	0.0238
PTPN20A	0.8382	1.1099	1.0699	0.8907	0.9772	0.0665
ZNF594	0.8555	1.0709	1.0092	0.9731	0.9772	0.0453
DCAMKL3	0.8791	1.1004	0.9915	0.9377	0.9772	0.0471
C16orf38	0.9258	1.1112	1.0119	0.8599	0.9772	0.0544
WNT8B	0.9763	1.0221	1.0026	0.9076	0.9772	0.0250
LOC728933	0.9964	0.9949	1.0433	0.8741	0.9772	0.0362
PHF8	0.9510	1.0133	0.9440	1.0005	0.9772	0.0174
PRKCSH	0.8119	1.2930	1.0316	0.7722	0.9772	0.1197
FAM71A	0.9620	1.0505	1.0550	0.8413	0.9772	0.0501
LOC440338	0.9994	1.0008	1.0036	0.9050	0.9772	0.0241
APOBEC3F	0.9118	1.2064	1.0352	0.7554	0.9772	0.0955
OR52I2	0.8889	1.0033	1.1131	0.9035	0.9772	0.0520
LOC202134	0.9741	1.0473	1.0010	0.8865	0.9772	0.0338
LOC641788	0.8681	1.1269	1.0124	0.9015	0.9772	0.0587
GSG1L	0.9460	0.9782	0.9899	0.9947	0.9772	0.0110
LOC650341	0.9027	1.0491	1.0439	0.9133	0.9772	0.0400
LOC645983	0.9613	1.0248	0.9711	0.9517	0.9772	0.0164
LOC1001324	1.0083	0.9872	1.1336	0.7798	0.9772	0.0733
LOC1001341	0.9148	1.1506	0.9616	0.8819	0.9772	0.0601
COMP	0.9552	1.0801	0.9665	0.9071	0.9772	0.0366
LOC387941	0.9998	1.1144	0.9744	0.8204	0.9772	0.0605
MAGEA9B	0.8522	1.0943	1.0807	0.8818	0.9772	0.0640
DYRK1A	0.8873	1.1708	1.1499	0.7011	0.9773	0.1124
FABP4	0.9150	1.0966	0.9550	0.9426	0.9773	0.0406
LOC646447	0.9022	1.0544	1.0719	0.8806	0.9773	0.0499
OR2A2	0.9763	1.0321	0.9799	0.9208	0.9773	0.0227
LOC646012	0.9598	1.1857	0.8922	0.8713	0.9773	0.0720
C4orf33	0.9472	1.1262	1.1157	0.7199	0.9773	0.0951
PCMTD2	0.8095	1.4396	0.8561	0.8040	0.9773	0.1545

LOC727759	0.9670	1.1552	1.0892	0.6977	0.9773	0.1010
LOC647212	0.9590	1.0571	0.9741	0.9190	0.9773	0.0290
GPHB5	0.9530	1.0880	1.0228	0.8453	0.9773	0.0519
MIR382	0.8762	1.1823	0.9522	0.8985	0.9773	0.0702
CPM	0.9152	1.1029	0.9418	0.9492	0.9773	0.0425
LOC1001337	0.9886	0.9826	0.9814	0.9566	0.9773	0.0071
PRR20C	0.8471	1.1430	1.0755	0.8436	0.9773	0.0774
CYP21A2	0.9424	1.1225	0.9271	0.9172	0.9773	0.0487
HDAC7	0.9916	0.9219	1.1267	0.8690	0.9773	0.0558
SPON1	0.8096	1.1211	0.9642	1.0143	0.9773	0.0648
PKP1	0.9437	1.0666	1.0433	0.8557	0.9773	0.0485
LOC1001294	0.9314	1.1499	0.9625	0.8653	0.9773	0.0610
WDR40B	0.9245	1.1056	0.9902	0.8889	0.9773	0.0476
MXRA7	0.9611	1.0939	1.0131	0.8411	0.9773	0.0530
LOC1001292	0.9223	1.2162	1.0232	0.7475	0.9773	0.0979
KIAA0087	0.8978	1.0498	1.0171	0.9446	0.9773	0.0344
TMEM25	0.8471	1.0293	1.2191	0.8137	0.9773	0.0935
MIR182	1.0023	0.9933	1.0017	0.9120	0.9773	0.0219
LOC647065	0.9337	1.0630	1.0183	0.8943	0.9773	0.0385
LOC1001339	0.9635	1.0746	1.0448	0.8264	0.9773	0.0555
KATNAL2	0.9482	1.0802	0.9641	0.9168	0.9773	0.0357
LOC1001309	0.9490	1.1047	0.9623	0.8933	0.9773	0.0450
LOC1001327	0.9008	1.1644	0.9473	0.8967	0.9773	0.0634
LOC645649	0.9019	1.1455	1.0537	0.8083	0.9773	0.0755
LOC650761	0.9412	1.1162	0.9463	0.9056	0.9773	0.0472
CDH6	1.0017	0.9787	0.9951	0.9339	0.9773	0.0153
LOC643534	0.8951	1.1127	1.0172	0.8844	0.9773	0.0542
PRAMEF15	0.9315	1.0929	1.0269	0.8582	0.9774	0.0517
LOC647371	0.8910	1.1834	0.9640	0.8711	0.9774	0.0715
SIDT1	0.9488	1.0332	1.0355	0.8919	0.9774	0.0349
LOC651610	1.1464	1.1058	0.8689	0.7884	0.9774	0.0878
CRYBA2	0.8705	1.0325	1.0995	0.9070	0.9774	0.0535
LOC653583	0.8813	1.1607	0.9500	0.9174	0.9774	0.0627
ZNF419	1.0017	1.4261	0.8048	0.6769	0.9774	0.1638
PRKCDBP	0.9194	1.0160	1.0429	0.9312	0.9774	0.0307
LOC728905	0.8305	1.1003	1.0816	0.8971	0.9774	0.0671
AIFM2	0.8287	1.1294	1.0289	0.9225	0.9774	0.0651
MT1IP	0.8132	1.1260	1.0692	0.9012	0.9774	0.0726
GABRA1	0.9215	1.0110	1.0905	0.8865	0.9774	0.0459
LOC651731	1.0160	0.9981	1.0281	0.8672	0.9774	0.0372
LOC727941	0.8778	1.0377	1.0439	0.9501	0.9774	0.0395
OXT	0.9357	0.9964	1.0460	0.9315	0.9774	0.0272
MAP2K5	0.8326	1.2315	1.0318	0.8137	0.9774	0.0980
LOC644124	0.8427	1.0553	1.0686	0.9430	0.9774	0.0530
TNIK	0.9497	1.0608	0.9563	0.9428	0.9774	0.0279
LOC647651	0.9053	1.1174	1.0084	0.8785	0.9774	0.0544

LOC732377	1.0144	1.0959	0.8970	0.9023	0.9774	0.0479
LYPD6B	0.9177	1.1596	0.9842	0.8481	0.9774	0.0668
MGC16075	0.9418	0.9628	1.0641	0.9410	0.9774	0.0293
GPATCH3	0.9041	1.1082	1.0033	0.8941	0.9774	0.0501
LOC390354	0.7046	1.2316	1.3614	0.6120	0.9774	0.1871
LOC1001326	0.9250	1.1590	0.9334	0.8923	0.9774	0.0612
RPRM	1.0549	0.9985	0.9457	0.9107	0.9774	0.0315
OR52N4	0.9277	1.0857	0.9299	0.9664	0.9774	0.0372
CCL15	0.8955	1.0745	1.0042	0.9355	0.9774	0.0394
LOC645164	0.9049	1.0378	1.0490	0.9180	0.9774	0.0383
LOC646951	0.9634	0.9855	1.0296	0.9312	0.9774	0.0207
CR2	0.8495	1.0370	1.0921	0.9312	0.9774	0.0542
C1orf38	0.7793	1.0340	1.0921	1.0044	0.9774	0.0685
OR3A3	0.9186	1.1262	1.0034	0.8617	0.9775	0.0575
SLC26A10	0.9079	1.1369	0.9624	0.9027	0.9775	0.0548
LOC1001325	0.9252	1.0008	1.0566	0.9272	0.9775	0.0317
ZNF626	0.8739	1.0839	1.0287	0.9233	0.9775	0.0480
LOC652000	0.9359	1.0263	1.0416	0.9060	0.9775	0.0333
LOC441282	0.9775	1.1222	0.8553	0.9549	0.9775	0.0551
LOC731377	0.9973	0.9169	1.0586	0.9370	0.9775	0.0320
LOC650195	0.8800	1.0705	1.0731	0.8862	0.9775	0.0545
UNC13A	0.9537	1.0091	1.0333	0.9139	0.9775	0.0270
LOC1001317	1.0008	1.0326	0.9856	0.8909	0.9775	0.0305
OR8G1	0.9648	0.9677	1.1144	0.8631	0.9775	0.0517
SPEM1	0.9064	1.1574	1.0294	0.8169	0.9775	0.0741
LOC646493	0.9650	1.0448	1.0053	0.8949	0.9775	0.0320
CDYL	0.8512	1.1987	1.0208	0.8393	0.9775	0.0846
AMN	1.0034	1.0167	1.0361	0.8539	0.9775	0.0417
LOC1001318	0.9039	1.1026	0.9387	0.9648	0.9775	0.0435
LOC387804	0.8954	1.1172	1.0104	0.8871	0.9775	0.0544
LOC131909	0.9512	1.0144	1.0076	0.9368	0.9775	0.0196
LOC652163	0.8936	1.0472	1.0806	0.8886	0.9775	0.0504
FAM90A2P	0.9317	1.0849	1.0134	0.8802	0.9775	0.0451
KLK9	0.9249	1.0766	1.0499	0.8587	0.9775	0.0516
LOC390211	0.9465	1.0745	1.0079	0.8814	0.9775	0.0414
HHIPL2	0.9658	1.0861	0.9509	0.9074	0.9775	0.0382
LOC643792	0.8589	1.1107	1.0491	0.8915	0.9775	0.0608
PCDHB9	0.8137	1.1146	1.0796	0.9022	0.9775	0.0717
LOC653240	0.9066	1.1130	0.9922	0.8984	0.9776	0.0499
C11orf41	0.9184	1.0554	1.0155	0.9210	0.9776	0.0344
FOXA1	0.9008	1.3129	1.0019	0.6946	0.9776	0.1288
LOC652150	0.9347	1.0299	1.0438	0.9019	0.9776	0.0350
LOC731789	1.0293	1.1228	0.9834	0.7747	0.9776	0.0736
LOC1001324	1.0266	1.0374	0.9035	0.9428	0.9776	0.0325
LOC1001318	0.8920	1.0576	1.0887	0.8721	0.9776	0.0557
FOLR4	0.9412	1.0742	0.9848	0.9100	0.9776	0.0357

PILRA	0.8725	1.1031	1.0618	0.8730	0.9776	0.0611
LOC653262	0.9124	1.0777	0.9782	0.9420	0.9776	0.0360
CAMK2A	0.8447	1.1465	0.9917	0.9275	0.9776	0.0638
FLJ44635	1.0570	1.0223	0.9583	0.8727	0.9776	0.0405
LOC644153	0.9387	1.1666	0.9649	0.8401	0.9776	0.0685
SCARNA27	0.8466	1.0838	1.0864	0.8936	0.9776	0.0628
LOC401233	0.9532	0.9674	1.0773	0.9125	0.9776	0.0352
TRAF1	0.8903	1.1671	0.9751	0.8779	0.9776	0.0668
GRIP1	0.9725	1.0532	0.9726	0.9121	0.9776	0.0290
FSD2	0.9509	1.0080	0.9869	0.9647	0.9776	0.0125
LOC647696	0.9792	1.0208	1.0075	0.9030	0.9776	0.0264
UBXN6	0.8436	1.3281	1.0294	0.7095	0.9776	0.1340
MADD	0.9070	1.2136	0.9937	0.7963	0.9776	0.0884
LOC649702	0.9069	1.0725	1.0084	0.9227	0.9776	0.0387
LOC1001293	0.8348	1.0540	1.0986	0.9232	0.9776	0.0605
DEFB108B	0.8511	1.1782	1.0051	0.8761	0.9776	0.0749
SKI	0.9203	1.2155	0.8005	0.9743	0.9776	0.0872
SPATA18	0.8426	1.1958	1.0731	0.7991	0.9776	0.0944
ZNF19	0.8761	1.1253	0.9957	0.9135	0.9777	0.0552
LOC401805	0.8516	1.2691	0.9794	0.8106	0.9777	0.1036
SNORA74B	0.9587	1.0380	1.0242	0.8897	0.9777	0.0340
OR10J3	0.8217	1.1440	1.0599	0.8851	0.9777	0.0749
SPINK6	1.0313	1.0061	0.9624	0.9109	0.9777	0.0264
LOC1001292	0.9997	1.0143	1.0070	0.8897	0.9777	0.0295
LOC1001334	1.0177	1.0189	0.9894	0.8847	0.9777	0.0317
SEPHS2	0.9815	1.2078	1.0263	0.6952	0.9777	0.1061
ANKRD31	1.0642	1.0178	0.8700	0.9588	0.9777	0.0419
TMEM198	0.9258	1.0503	1.0432	0.8915	0.9777	0.0405
LOC646046	0.9283	1.1247	0.9619	0.8959	0.9777	0.0508
FLCN	0.9440	1.1747	0.9727	0.8193	0.9777	0.0736
FLJ39639	1.0217	1.1253	0.8741	0.8897	0.9777	0.0593
ZMAT1	0.9268	1.0241	0.9922	0.9677	0.9777	0.0205
LOC729683	0.8210	0.9274	1.2533	0.9092	0.9777	0.0948
LOC653321	0.9482	1.0221	1.1325	0.8081	0.9777	0.0680
LOC390856	0.7971	1.0636	1.0935	0.9567	0.9777	0.0670
LOC1001334	1.0235	0.9830	0.9977	0.9067	0.9777	0.0251
TBC1D7	0.9089	1.2856	0.9912	0.7252	0.9777	0.1167
LOC1001347	0.8855	1.0472	1.0940	0.8843	0.9777	0.0544
LOC652968	1.0634	1.1907	0.9398	0.7170	0.9777	0.1009
LOC648213	0.9802	0.9841	1.0179	0.9287	0.9777	0.0184
HIST1H1T	1.0019	1.0626	0.9960	0.8505	0.9777	0.0450
LOC643712	1.0493	1.1312	0.8755	0.8549	0.9777	0.0672
RBM43	0.9142	1.0728	0.9934	0.9305	0.9778	0.0360
LOC441242	0.8866	1.0452	1.0841	0.8952	0.9778	0.0508
LOC642702	0.8338	1.0928	1.0391	0.9453	0.9778	0.0569
LOC644873	0.9687	1.1216	0.9320	0.8889	0.9778	0.0506

CDH7	0.9165	1.0889	0.9915	0.9142	0.9778	0.0412
LOC647677	1.0129	1.0035	1.0178	0.8768	0.9778	0.0338
SLC6A20	0.9787	1.0100	1.0510	0.8714	0.9778	0.0384
LOC1001290	0.9242	1.0397	1.0450	0.9021	0.9778	0.0376
UBD	0.9427	1.0433	0.9911	0.9340	0.9778	0.0252
OR6Y1	0.9864	1.0044	0.9962	0.9241	0.9778	0.0183
LOC645176	0.9664	1.0451	0.9669	0.9328	0.9778	0.0238
SNORD59A	0.9888	1.0677	0.9380	0.9167	0.9778	0.0336
LOC389422	1.0131	1.0164	1.0225	0.8593	0.9778	0.0396
HSP90B3P	0.9590	0.9881	1.0316	0.9324	0.9778	0.0212
LOC650577	0.9372	1.1306	0.9450	0.8984	0.9778	0.0519
SPANXB1	0.8983	1.0249	1.0668	0.9211	0.9778	0.0405
APOBEC3C	0.9334	1.0641	1.0421	0.8716	0.9778	0.0455
LOC643090	0.9415	1.0569	0.9894	0.9234	0.9778	0.0298
KCNK12	0.9474	1.0071	1.1370	0.8198	0.9778	0.0659
LRRK1	0.8829	1.0553	1.0268	0.9463	0.9778	0.0392
POF1B	0.8501	1.0316	1.0933	0.9364	0.9778	0.0534
LRAP	0.7161	1.0035	1.3805	0.8112	0.9778	0.1469
LOC647169	0.8965	1.2111	0.9469	0.8568	0.9778	0.0799
PF4V1	0.9643	1.1316	0.9951	0.8204	0.9778	0.0638
FGFR2	0.8900	1.0465	1.0436	0.9312	0.9778	0.0397
LOC650005	1.0440	1.0252	0.9185	0.9236	0.9778	0.0330
PIWIL4	0.8846	1.0728	1.0763	0.8777	0.9778	0.0559
EIF2C4	0.9739	1.0750	1.0169	0.8457	0.9778	0.0487
SCN3B	0.9741	1.0127	1.0044	0.9203	0.9779	0.0209
CCR10	0.9420	1.0719	1.0108	0.8867	0.9779	0.0403
AMBN	0.9435	1.0011	0.9936	0.9733	0.9779	0.0129
LOC730185	0.9757	0.9863	1.0887	0.8608	0.9779	0.0466
LOC393078	0.9177	1.0222	1.0933	0.8785	0.9779	0.0490
LOC642397	0.8991	1.0866	1.0243	0.9017	0.9779	0.0465
CCNG2	0.8913	1.3170	1.0867	0.6166	0.9779	0.1486
TRIM54	0.9582	1.0797	0.9890	0.8847	0.9779	0.0404
RAB14	0.9702	1.0855	0.9759	0.8801	0.9779	0.0421
LOC729296	0.9128	1.0704	0.9833	0.9451	0.9779	0.0340
TCF3	1.0112	1.1076	0.8821	0.9108	0.9779	0.0513
SLCO2A1	1.0447	1.0008	1.0015	0.8647	0.9779	0.0391
FOXP2	0.9056	1.1266	1.0097	0.8698	0.9779	0.0578
FLJ41423	0.9646	1.0495	0.9635	0.9341	0.9779	0.0249
RAB9P1	1.0386	1.0088	0.9994	0.8649	0.9779	0.0386
LOC1001327	0.8564	1.1185	1.0291	0.9076	0.9779	0.0592
ARL13A	1.0075	1.0831	0.9963	0.8248	0.9779	0.0545
LOC650853	0.9261	1.0602	1.0525	0.8730	0.9779	0.0466
LOC648173	0.8503	1.0242	1.0222	1.0151	0.9779	0.0426
FLJ36116	1.0557	1.0367	0.9902	0.8291	0.9779	0.0515
TEPP	0.9122	1.0983	1.0050	0.8962	0.9779	0.0468
GJA3	0.9869	1.0420	1.0075	0.8755	0.9779	0.0360

LOC649857	0.9365	1.0639	1.0333	0.8781	0.9779	0.0429
LOC641948	0.7955	1.1276	1.1165	0.8722	0.9779	0.0847
LOC650405	0.9449	1.0710	0.9971	0.8989	0.9780	0.0369
LOC643637	0.9050	1.0711	1.0828	0.8529	0.9780	0.0582
LOC138652	0.9704	0.9786	1.1182	0.8446	0.9780	0.0559
OR5AY1	0.9494	1.0170	1.0168	0.9287	0.9780	0.0229
MGC39606	0.8939	1.1375	1.0170	0.8635	0.9780	0.0627
LOC392335	1.0001	1.0864	0.9670	0.8585	0.9780	0.0471
LOC651022	0.9771	1.0733	0.9367	0.9249	0.9780	0.0337
LOC1001296	0.9536	1.0638	0.9897	0.9049	0.9780	0.0335
STS	1.0902	0.9399	0.9683	0.9136	0.9780	0.0390
LOC652715	0.8983	1.0230	1.1510	0.8396	0.9780	0.0692
LOC644655	0.9944	1.0169	1.0195	0.8812	0.9780	0.0327
SPI1	0.9824	1.0568	0.9672	0.9055	0.9780	0.0311
SIGLECP3	0.9849	1.0120	1.0123	0.9027	0.9780	0.0259
TTTY14	0.8730	1.0677	1.0311	0.9402	0.9780	0.0441
EFHB	0.9459	1.0345	1.0332	0.8984	0.9780	0.0337
TM9SF2	0.7538	1.1767	1.2623	0.7192	0.9780	0.1407
CLRN3	0.8874	1.1495	1.0566	0.8185	0.9780	0.0759
LOC391429	0.8589	0.9230	1.1434	0.9867	0.9780	0.0610
TCL1B	0.9320	1.0427	0.9769	0.9604	0.9780	0.0235
LOC648552	0.9444	1.0141	1.0536	0.9000	0.9780	0.0345
STOM	0.9088	1.2096	0.9393	0.8544	0.9780	0.0792
HAUS6	0.9122	1.0748	1.0591	0.8660	0.9780	0.0523
LOC1001290	0.9585	1.0382	0.9850	0.9303	0.9780	0.0230
LOC644171	0.9966	0.9512	0.9460	1.0183	0.9780	0.0176
LOC441617	1.0314	0.8818	1.0496	0.9492	0.9780	0.0388
EDARADD	0.9928	0.9985	0.9918	0.9290	0.9780	0.0164
LOC1001284	0.9742	1.0816	0.9774	0.8789	0.9780	0.0414
TRIM34	0.8989	1.0745	1.0217	0.9171	0.9780	0.0420
MIR1269	0.9833	1.0788	0.9946	0.8554	0.9780	0.0461
LOC1001290	0.8976	1.0495	1.0693	0.8957	0.9780	0.0472
LOC644265	0.8557	1.2004	1.0473	0.8088	0.9780	0.0903
GLYAT	0.9212	1.0973	1.0111	0.8826	0.9781	0.0480
MIR1205	0.8831	1.0720	1.0191	0.9380	0.9781	0.0420
LOC391169	0.9273	1.0397	0.9829	0.9622	0.9781	0.0235
AK5	0.9030	1.1265	0.9506	0.9322	0.9781	0.0505
PGAM2	0.9811	0.9583	1.0151	0.9577	0.9781	0.0135
EPB42	0.9659	1.0925	0.8969	0.9570	0.9781	0.0411
LOC1001313	0.9286	0.9640	1.1106	0.9091	0.9781	0.0456
LOC1001326	0.9809	0.9914	1.0869	0.8530	0.9781	0.0480
LOC283332	0.8415	1.1129	1.0619	0.8960	0.9781	0.0649
TEX13A	0.9896	1.0369	0.9677	0.9181	0.9781	0.0247
LOC731511	0.9675	0.9406	1.0427	0.9615	0.9781	0.0223
LOC648863	0.9548	1.1446	0.9453	0.8676	0.9781	0.0588
FAM98C	0.9586	1.3120	0.9136	0.7281	0.9781	0.1220

ASNA1	0.8581	1.1135	0.8677	1.0730	0.9781	0.0670
BCL2L11	0.9621	0.9849	1.0494	0.9160	0.9781	0.0277
PPFIA4	0.9788	1.1126	0.9524	0.8686	0.9781	0.0506
TAS2R8	0.9756	0.9694	1.0816	0.8859	0.9781	0.0401
LOC650436	0.9204	1.0260	1.0268	0.9391	0.9781	0.0282
LOC649596	0.8765	1.1735	0.9761	0.8864	0.9781	0.0689
PCTK3	0.9011	1.1102	0.9853	0.9159	0.9781	0.0477
LOC641515	1.0263	1.0175	0.9542	0.9145	0.9781	0.0266
LOC728362	0.9781	1.0270	1.0442	0.8633	0.9781	0.0408
CTAGE4	0.9215	1.0188	1.0223	0.9499	0.9781	0.0252
LOC650846	0.8733	1.0818	1.0992	0.8582	0.9781	0.0650
CST5	0.9156	1.0774	1.0100	0.9096	0.9781	0.0403
C21orf88	0.9311	1.0865	1.0062	0.8887	0.9781	0.0435
PLEK	0.9037	1.1097	0.9425	0.9566	0.9781	0.0453
EVX1	0.9480	0.9884	1.0274	0.9488	0.9781	0.0190
LOC649527	0.9481	1.0250	0.9365	1.0029	0.9781	0.0213
KCTD14	0.9587	1.0809	0.9518	0.9213	0.9781	0.0352
C3orf27	0.9941	0.9954	0.9902	0.9329	0.9781	0.0151
SCAND2	0.9641	1.1123	0.9649	0.8713	0.9782	0.0498
RFX4	0.9371	1.0800	0.9927	0.9029	0.9782	0.0387
LOC653650	0.9734	1.0771	0.9761	0.8861	0.9782	0.0390
CPXCR1	0.9637	1.0105	1.0187	0.9197	0.9782	0.0229
EFCBP1	0.9120	1.0739	0.9184	1.0083	0.9782	0.0388
GRHL1	0.9194	1.0876	1.0062	0.8994	0.9782	0.0432
BMS1	0.7187	1.4862	0.9432	0.7646	0.9782	0.1761
C16orf73	0.9439	1.0220	1.0272	0.9196	0.9782	0.0273
RGS11	0.9113	1.0421	1.0012	0.9581	0.9782	0.0281
PLEKHG1	1.0046	1.0430	0.9957	0.8694	0.9782	0.0377
LOC402116	0.9393	1.0626	0.9827	0.9282	0.9782	0.0305
CES3	0.9870	1.0692	0.9209	0.9356	0.9782	0.0335
LRRC66	0.9011	1.0872	1.0684	0.8560	0.9782	0.0584
MMP25	0.9046	1.1919	0.9082	0.9081	0.9782	0.0712
ERCC-00016	0.9684	1.1115	0.9448	0.8880	0.9782	0.0475
BCAR1	1.0312	1.1612	0.9010	0.8194	0.9782	0.0750
SMARCA1	1.0166	1.0269	1.0407	0.8286	0.9782	0.0501
TTC33	0.9287	1.0951	1.0973	0.7916	0.9782	0.0737
LOC150185	0.8362	1.1802	0.9747	0.9218	0.9782	0.0731
MRAP2	0.9365	1.0851	0.9357	0.9555	0.9782	0.0359
LOC727894	0.8602	1.1235	1.0265	0.9027	0.9782	0.0599
CHI3L2	0.9655	1.0035	1.0485	0.8953	0.9782	0.0324
DNHD1	1.0559	1.0418	1.0123	0.8029	0.9782	0.0591
SHISA5	0.8798	1.2093	1.0952	0.7287	0.9782	0.1076
LOC1001286	0.9177	1.0741	1.0133	0.9079	0.9782	0.0398
OCM	0.8437	1.1162	1.0320	0.9211	0.9782	0.0601
CCDC24	0.8432	1.4854	0.8622	0.7222	0.9782	0.1719
LOC1001291	1.0398	1.0511	0.9692	0.8529	0.9783	0.0455

LOC646473	0.9740	1.0119	1.0480	0.8792	0.9783	0.0363
BMP8A	1.0074	1.0154	1.0187	0.8716	0.9783	0.0356
LOC730066	0.8222	1.1575	1.0458	0.8875	0.9783	0.0760
LOC646906	0.9446	0.9941	1.0610	0.9134	0.9783	0.0322
LOC1001334	0.8405	1.1218	1.0654	0.8854	0.9783	0.0682
LOC732455	1.0487	0.9614	1.0304	0.8726	0.9783	0.0399
TIE1	0.8358	1.1336	1.0659	0.8779	0.9783	0.0720
LOC1001301	0.9151	1.0883	0.9862	0.9235	0.9783	0.0400
ERCC-00168	1.1111	1.0130	0.9381	0.8509	0.9783	0.0553
C21orf62	0.8431	1.1216	0.9522	0.9963	0.9783	0.0576
LOC1001331	0.8762	1.0291	1.0412	0.9667	0.9783	0.0377
MIR132	1.0078	0.9881	1.0131	0.9042	0.9783	0.0253
ZNF23	0.9082	1.1427	1.0671	0.7952	0.9783	0.0782
LOC440918	0.8182	1.0851	1.1219	0.8880	0.9783	0.0741
COMMD9	0.9594	1.1700	1.0918	0.6922	0.9783	0.1048
SRD5A2	0.9372	1.1454	0.9000	0.9306	0.9783	0.0563
FCRL3	0.8850	1.0630	1.0422	0.9230	0.9783	0.0438
KCNG3	1.0255	1.1275	0.9676	0.7926	0.9783	0.0702
LOC650943	0.8738	1.1410	0.9999	0.8985	0.9783	0.0607
HISPPD1	0.9207	1.0033	1.1220	0.8672	0.9783	0.0555
MIR487A	0.9283	1.0293	1.0066	0.9491	0.9783	0.0237
ZBTB26	0.8582	1.1302	1.0237	0.9011	0.9783	0.0616
TCN2	0.7956	1.1255	1.1078	0.8844	0.9783	0.0820
SNORD7	0.8712	1.0454	1.0883	0.9084	0.9783	0.0524
FGF2	0.9303	1.0880	1.0107	0.8843	0.9783	0.0449
LOC143678	0.9787	1.1447	0.9471	0.8428	0.9783	0.0626
LOC442147	0.8923	1.1365	0.9986	0.8860	0.9783	0.0587
CNRIP1	0.8740	1.0762	1.0109	0.9523	0.9783	0.0430
LOC647955	0.9437	1.0911	0.9608	0.9178	0.9783	0.0386
OR1J1	0.8989	1.1168	1.0421	0.8556	0.9783	0.0610
LOC1001300	0.9332	1.0452	1.0781	0.8568	0.9783	0.0510
AMHR2	0.9553	1.2020	1.0253	0.7308	0.9783	0.0975
C1orf167	0.9409	1.1318	0.9417	0.8990	0.9783	0.0521
SLC17A2	0.8776	1.0594	1.0359	0.9405	0.9784	0.0423
CDH29	0.8420	1.3526	0.8863	0.8326	0.9784	0.1253
LOC651834	0.9410	0.8720	1.0206	1.0799	0.9784	0.0455
ANKS4B	1.0050	0.9221	1.0073	0.9791	0.9784	0.0198
LOC399753	0.9616	1.0389	1.0227	0.8903	0.9784	0.0338
C10orf128	0.9413	1.0167	1.0686	0.8869	0.9784	0.0402
MAST1	0.9925	1.2169	0.9213	0.7828	0.9784	0.0906
LOC148137	0.9110	1.1139	0.9850	0.9036	0.9784	0.0488
C7orf64	0.9487	0.9868	1.0841	0.8940	0.9784	0.0400
TRIM43	0.9279	1.0474	1.0924	0.8459	0.9784	0.0562
ACAD11	0.8682	1.2338	1.0389	0.7727	0.9784	0.1014
LOC644525	0.7938	1.2492	1.0114	0.8592	0.9784	0.1011
C8orf71	0.9371	1.0124	1.0651	0.8990	0.9784	0.0373

SPANXN2	1.0107	1.0311	1.0589	0.8129	0.9784	0.0560
WISP3	0.8913	1.0689	1.0306	0.9228	0.9784	0.0424
ZNF620	0.8651	0.9910	1.0430	1.0146	0.9784	0.0392
SFXN2	0.9508	1.1509	0.9864	0.8256	0.9784	0.0670
TREX1	0.9840	1.0802	0.9486	0.9009	0.9784	0.0379
CFHR5	0.8521	1.2064	0.9273	0.9280	0.9784	0.0781
LOC1001299	0.9334	1.1676	0.9296	0.8832	0.9784	0.0641
MAGEB6B	0.9724	1.1017	0.9469	0.8927	0.9784	0.0443
C14orf23	0.9978	1.0358	0.9672	0.9129	0.9784	0.0260
GFRA1	0.9220	1.0843	1.0025	0.9049	0.9784	0.0412
QKI	0.8493	1.1761	1.0004	0.8880	0.9784	0.0733
LOC642248	0.9111	1.0997	1.0304	0.8726	0.9784	0.0526
LOC729338	0.9996	1.1775	0.8330	0.9036	0.9784	0.0746
HOXD1	0.9940	0.9592	0.9604	1.0003	0.9784	0.0109
PCSK4	0.9065	1.0131	1.1147	0.8795	0.9784	0.0538
LOC255326	0.9267	1.1451	0.9310	0.9109	0.9785	0.0557
OCA2	0.9136	1.0876	0.9986	0.9141	0.9785	0.0415
LOC116437	0.9054	1.0749	1.0196	0.9140	0.9785	0.0413
LOC1001312	0.8440	1.1106	1.1413	0.8181	0.9785	0.0855
CTSG	0.8468	1.0512	1.0765	0.9394	0.9785	0.0530
LOC645956	0.8371	1.1713	0.9582	0.9474	0.9785	0.0698
LOC642775	0.9612	0.9487	1.0428	0.9613	0.9785	0.0217
ACTL6B	0.8367	1.1540	0.9142	1.0091	0.9785	0.0683
PCSK6	0.9498	1.0898	1.0131	0.8615	0.9785	0.0484
PRICKLE1	1.0010	0.9995	0.9887	0.9250	0.9785	0.0181
LOC730234	1.0033	1.0573	0.9056	0.9479	0.9785	0.0330
LOC1001294	0.9041	1.1447	0.9573	0.9081	0.9785	0.0567
KREMEN1	0.8949	1.1310	0.9945	0.8938	0.9785	0.0560
LOC654203	0.9318	1.0435	0.9963	0.9426	0.9785	0.0258
LOC1001291	0.9345	0.9904	1.0364	0.9528	0.9785	0.0225
LOC401286	0.9357	1.0662	1.0131	0.8991	0.9785	0.0377
ZNF713	0.8978	1.1917	1.0302	0.7945	0.9785	0.0859
LOC654000	0.9236	1.1539	0.9747	0.8619	0.9785	0.0628
LOXL2	0.9498	1.0880	0.9346	0.9418	0.9785	0.0366
ALDOB	0.8236	1.2262	1.0105	0.8539	0.9785	0.0921
LOC647264	0.8854	1.0531	1.0051	0.9706	0.9785	0.0354
SYTL1	1.0572	1.1105	0.8845	0.8621	0.9786	0.0619
LOC729566	1.0185	1.0310	0.9468	0.9179	0.9786	0.0275
LOC652784	0.9275	1.0223	1.0727	0.8917	0.9786	0.0418
MIR1302-7	0.9638	1.0834	0.9562	0.9107	0.9786	0.0369
LOC1001297	1.0000	0.9764	1.0508	0.8870	0.9786	0.0342
MGC13005	0.8904	1.0508	1.0415	0.9315	0.9786	0.0400
LRRN4CL	0.9105	1.0425	0.9927	0.9685	0.9786	0.0274
KRTAP10-10	0.8894	1.1114	1.0029	0.9105	0.9786	0.0507
LOC400011	0.9468	1.0464	1.0571	0.8641	0.9786	0.0455
LOC392232	1.0156	0.9975	0.9981	0.9032	0.9786	0.0255

LOC1001289	0.9263	1.0693	1.0218	0.8970	0.9786	0.0403
LOC642006	0.9386	1.0356	0.9965	0.9438	0.9786	0.0231
PROC	1.0208	1.0212	0.8853	0.9871	0.9786	0.0321
SEMA7A	0.9541	1.0608	1.0316	0.8680	0.9786	0.0432
PLCL2	0.8852	1.1969	1.0357	0.7967	0.9786	0.0879
MBL2	0.9432	1.0628	0.9447	0.9638	0.9786	0.0285
TMCC2	0.8644	1.0683	1.0108	0.9711	0.9786	0.0430
TNFSF12	0.9098	1.0942	0.9513	0.9592	0.9786	0.0400
PDE6A	0.8670	1.1005	1.0474	0.8997	0.9786	0.0565
FBLIM1	0.9633	1.0146	1.0074	0.9293	0.9786	0.0200
LOC654222	0.9231	1.0515	1.1165	0.8234	0.9786	0.0655
LOC652523	0.9057	0.9829	1.0462	0.9798	0.9786	0.0287
LOC644554	0.9049	1.1359	1.0466	0.8271	0.9786	0.0694
LOC727796	0.8467	1.1421	1.0308	0.8950	0.9786	0.0670
LOC642263	0.9415	1.0753	1.0559	0.8418	0.9787	0.0543
C6orf165	0.8717	1.0854	0.9971	0.9605	0.9787	0.0443
MYO1A	0.8900	1.0957	1.0327	0.8963	0.9787	0.0510
LOC399988	0.8980	1.3255	0.9899	0.7012	0.9787	0.1304
LOC729289	0.8743	1.0801	0.9833	0.9770	0.9787	0.0420
LOC730079	0.8147	1.1524	1.0032	0.9443	0.9787	0.0700
BEND2	0.9808	1.1233	0.9470	0.8636	0.9787	0.0541
FLJ46210	0.9051	1.1074	0.9931	0.9090	0.9787	0.0475
MIR643	0.9333	1.1204	0.9327	0.9283	0.9787	0.0473
APAF1	0.7953	1.2163	1.0055	0.8976	0.9787	0.0901
RBMS1	0.9397	1.0449	0.9888	0.9413	0.9787	0.0248
LOC649417	0.9340	1.0865	0.9413	0.9530	0.9787	0.0361
LOC645390	0.9815	1.0132	1.0200	0.9002	0.9787	0.0275
LOC283481	0.8697	0.9826	1.1020	0.9604	0.9787	0.0478
LOC728369	0.9651	1.0749	0.9480	0.9269	0.9787	0.0330
LOC401431	0.8499	1.3348	0.8612	0.8689	0.9787	0.1188
LOC1001327	0.8250	0.9311	1.2305	0.9282	0.9787	0.0875
GJB6	0.9820	1.0220	1.0222	0.8886	0.9787	0.0315
LPPR4	0.9692	1.0399	0.9864	0.9194	0.9787	0.0249
C1orf114	0.9249	1.0251	0.9958	0.9691	0.9787	0.0213
LOC222967	0.9100	1.0627	1.0439	0.8983	0.9787	0.0433
LOC1001334	0.9831	1.0050	1.0092	0.9175	0.9787	0.0212
SNAI3	0.9075	1.0339	1.0951	0.8784	0.9787	0.0514
LOC1001294	0.9056	1.0020	1.0526	0.9547	0.9787	0.0315
LOC644973	0.9240	1.2652	0.8909	0.8348	0.9787	0.0973
HIST1H2AJ	0.9658	1.0949	1.0407	0.8135	0.9787	0.0611
LOC646331	1.0235	1.0428	0.8730	0.9757	0.9787	0.0380
LOC647661	0.8904	1.1359	1.0171	0.8715	0.9787	0.0615
HOXA11AS	0.9330	1.0745	0.9662	0.9412	0.9787	0.0327
LOC729008	0.8811	1.2258	0.9034	0.9048	0.9787	0.0825
LOC649979	0.9456	1.0813	1.0069	0.8812	0.9787	0.0427
PHF7	0.9702	1.0921	0.9801	0.8726	0.9787	0.0449

MYO15A	0.9632	1.1147	0.9080	0.9291	0.9788	0.0467
LOC729497	0.9496	1.0746	1.0208	0.8700	0.9788	0.0444
SERPINA6	0.8710	1.0658	1.0079	0.9704	0.9788	0.0409
LOC646036	0.9442	1.0671	0.9210	0.9828	0.9788	0.0321
LOC647570	0.9476	1.0150	1.0948	0.8577	0.9788	0.0503
SLAMF6	0.9638	1.0724	0.9145	0.9645	0.9788	0.0333
GOLGA1	1.0238	1.1367	1.0227	0.7320	0.9788	0.0865
LOC646503	0.9776	0.9046	1.1392	0.8939	0.9788	0.0566
LOC642350	0.9805	0.9757	1.0186	0.9404	0.9788	0.0160
LOC1001313	0.9101	1.1603	0.9634	0.8814	0.9788	0.0628
SPEF2	0.9627	1.1083	0.9471	0.8971	0.9788	0.0454
LOC644021	1.0120	0.9923	1.0301	0.8808	0.9788	0.0336
LOC1001309	0.9509	1.0109	1.0149	0.9385	0.9788	0.0199
LOC653564	0.8394	1.0281	1.0888	0.9590	0.9788	0.0535
ADAM21P	0.9846	1.0608	1.0173	0.8526	0.9788	0.0449
LOC646946	0.9483	1.0858	0.9597	0.9216	0.9788	0.0365
ESM1	0.8704	1.1729	0.9483	0.9237	0.9788	0.0667
OR7A10	0.9411	1.0914	0.9968	0.8861	0.9788	0.0438
PRO1768	0.9178	1.0171	1.1027	0.8778	0.9789	0.0506
LOC155100	0.9370	1.0614	1.0112	0.9059	0.9789	0.0353
LOC731438	0.9277	1.0916	0.9772	0.9190	0.9789	0.0397
PLXNC1	0.9259	1.1809	1.0317	0.7770	0.9789	0.0852
GFI1	0.9376	1.1697	0.8835	0.9247	0.9789	0.0646
ZBTB45	0.8802	1.3138	0.9068	0.8146	0.9789	0.1133
JAK3	0.9164	1.0500	1.0205	0.9286	0.9789	0.0332
SNORD113-8	0.9528	1.0532	0.9772	0.9323	0.9789	0.0264
LOC644725	0.9619	1.1484	1.0121	0.7932	0.9789	0.0734
SNORD4B	0.9771	1.1073	0.9543	0.8768	0.9789	0.0479
PIGR	0.9430	1.1058	0.9824	0.8843	0.9789	0.0469
PSG6	0.9226	1.0747	0.9906	0.9276	0.9789	0.0355
LOC1001284	0.9697	1.0914	0.9861	0.8684	0.9789	0.0457
RNF139	0.8245	1.1355	1.0148	0.9408	0.9789	0.0652
LOC1001302	0.9677	1.1948	0.9302	0.8229	0.9789	0.0782
TGFB1I1	0.9397	1.0185	1.0625	0.8949	0.9789	0.0378
GRIN2A	0.8022	1.1713	0.9993	0.9429	0.9789	0.0764
MYO18A	0.9187	1.0798	1.0659	0.8512	0.9789	0.0560
VSNL1	0.9436	1.0874	1.0218	0.8628	0.9789	0.0486
PPT2	0.9192	1.1535	0.9764	0.8666	0.9789	0.0624
FLJ42953	0.8991	1.0156	0.9949	1.0060	0.9789	0.0269
TSPY3	0.9312	1.0612	0.9571	0.9663	0.9789	0.0284
LOC729704	0.9468	1.0590	0.9919	0.9180	0.9789	0.0307
FLG2	0.9017	0.9555	1.1058	0.9527	0.9789	0.0441
WHDC1L1	0.8846	1.0239	1.0961	0.9111	0.9789	0.0494
LOC441582	0.8767	1.1111	1.0837	0.8443	0.9789	0.0689
LOC442606	0.9101	1.0424	1.0028	0.9605	0.9789	0.0284
LOC1001302	0.8850	1.0785	0.9883	0.9640	0.9789	0.0398

ATP2A3	0.9338	1.0891	0.9356	0.9573	0.9789	0.0371
C3orf25	0.9112	1.1735	0.9216	0.9095	0.9790	0.0649
MIR411	0.9130	1.0362	1.0765	0.8902	0.9790	0.0457
LOC1001346	0.9930	0.9958	1.0637	0.8633	0.9790	0.0419
LOC645038	0.8695	1.0886	1.0324	0.9254	0.9790	0.0498
TMEM194B	1.0058	1.0200	1.0370	0.8530	0.9790	0.0425
LOC260340	0.9384	1.1081	1.0239	0.8455	0.9790	0.0564
SDC3	0.9140	1.0924	0.9937	0.9158	0.9790	0.0421
LOC648432	0.9035	1.1120	1.0698	0.8306	0.9790	0.0669
HSP90AB4P	0.9636	1.0293	1.0560	0.8670	0.9790	0.0421
LOC1001336	1.0079	1.1260	0.9165	0.8655	0.9790	0.0572
LOC651128	0.8727	1.1051	1.0533	0.8848	0.9790	0.0589
MMP26	0.8816	1.0463	1.0343	0.9538	0.9790	0.0384
LOC1001312	0.9379	1.0870	0.9615	0.9295	0.9790	0.0366
LOC1001319	0.9240	1.1414	0.9545	0.8962	0.9790	0.0554
ZNF140	0.9830	1.1100	1.0245	0.7985	0.9790	0.0657
KIFC3	1.0292	0.9716	1.1123	0.8030	0.9790	0.0654
GTF3C1	0.8206	1.3098	0.8344	0.9513	0.9790	0.1141
LOC729973	0.9104	1.0980	1.0669	0.8408	0.9790	0.0617
EHMT1	0.9317	1.1166	0.9799	0.8879	0.9790	0.0496
LOC727755	0.8824	1.1958	0.8913	0.9466	0.9790	0.0736
MIR424	1.0313	1.0117	0.9892	0.8838	0.9790	0.0329
KRT8P15	0.9231	1.0203	1.0624	0.9103	0.9790	0.0371
C20orf195	0.9461	0.9752	1.0047	0.9902	0.9790	0.0125
LOC1001329	0.8909	1.0853	1.0631	0.8768	0.9790	0.0552
MEIG1	0.9622	1.0344	1.0235	0.8961	0.9790	0.0319
CT45A4	0.9210	1.0275	1.0496	0.9180	0.9790	0.0347
LOC391730	1.0309	1.0281	0.9825	0.8746	0.9790	0.0365
LOC340239	1.0031	1.0309	0.9751	0.9072	0.9790	0.0265
GLI2	0.9353	0.9768	1.0504	0.9536	0.9791	0.0253
INHBB	0.9234	1.0758	0.9356	0.9813	0.9791	0.0346
ZSCAN23	0.9337	1.0886	0.9957	0.8983	0.9791	0.0417
LOC389765	0.7729	1.1129	1.2689	0.7615	0.9791	0.1264
CRYBB1	0.8154	1.2009	0.9553	0.9448	0.9791	0.0805
PTCH1	0.9740	1.0332	1.0375	0.8716	0.9791	0.0386
AMAC1L2	0.9861	1.0484	0.9694	0.9124	0.9791	0.0280
LOC729645	0.9642	1.0685	0.9966	0.8871	0.9791	0.0376
LOC1001339	0.8868	1.0371	1.0663	0.9260	0.9791	0.0431
OR4K2	0.9208	1.0614	1.0040	0.9301	0.9791	0.0331
LOC653879	0.8705	1.1242	1.0225	0.8992	0.9791	0.0585
EFR3B	0.7723	1.2266	1.0151	0.9023	0.9791	0.0963
C7orf31	0.8748	0.9828	1.1347	0.9241	0.9791	0.0564
SOX4	0.8997	1.3055	1.0191	0.6922	0.9791	0.1280
KLHL32	0.9361	1.0919	0.9996	0.8888	0.9791	0.0439
LOC648473	1.0506	1.0910	0.8816	0.8932	0.9791	0.0536
LOC653550	0.9228	1.0304	1.0747	0.8885	0.9791	0.0439

MIR507	0.9823	0.9779	0.9455	1.0108	0.9791	0.0134
LOC729445	0.9735	1.0508	0.9745	0.9177	0.9791	0.0273
INHA	0.9376	1.0384	1.0257	0.9148	0.9791	0.0310
FRMD4A	0.9370	0.9484	1.1544	0.8768	0.9791	0.0605
LOC644880	0.9503	1.1182	1.0792	0.7688	0.9791	0.0787
FLJ40712	0.9372	1.1497	1.0346	0.7951	0.9791	0.0752
LOC652703	0.8591	1.0697	1.1419	0.8458	0.9791	0.0747
TBC1D3	0.9373	1.0558	0.9663	0.9572	0.9792	0.0263
LOC645401	1.0515	1.0102	0.9692	0.8857	0.9792	0.0354
LEPREL2	0.9460	1.0207	1.0006	0.9494	0.9792	0.0186
LOC1001279	0.9172	1.0952	0.9990	0.9053	0.9792	0.0439
LOC730517	0.9345	1.0564	1.0436	0.8821	0.9792	0.0424
LOC646070	1.0237	0.9998	0.9760	0.9172	0.9792	0.0228
OR2M7	1.0594	0.9970	0.9484	0.9120	0.9792	0.0319
OPRM1	0.9447	1.0701	0.9767	0.9253	0.9792	0.0321
FLJ10489	0.9219	1.0716	1.0678	0.8555	0.9792	0.0540
DNER	0.9408	1.0895	0.9638	0.9227	0.9792	0.0377
NANOG	0.9563	1.0772	1.0076	0.8757	0.9792	0.0425
LOC728649	0.7795	1.0795	1.0577	1.0002	0.9792	0.0687
LRRC33	0.8746	1.0514	0.9920	0.9989	0.9792	0.0373
CLDN18	0.9743	1.0454	0.9759	0.9213	0.9792	0.0254
PNMAL1	0.9452	1.0115	1.0501	0.9102	0.9792	0.0316
ABCD4	0.9737	1.0309	1.0381	0.8741	0.9792	0.0379
HAND2	0.8708	1.1284	1.0269	0.8908	0.9792	0.0606
ERCC-00039	1.0497	1.0721	0.9158	0.8793	0.9792	0.0480
BAGE4	0.9631	1.0597	1.0050	0.8891	0.9792	0.0360
FAM96B	0.7615	1.4207	0.9594	0.7753	0.9792	0.1539
LOC1001311	0.8881	1.1062	1.0259	0.8967	0.9792	0.0528
LOC1001283	0.8918	1.1524	1.0196	0.8532	0.9792	0.0678
NFAT5	0.8365	1.3579	0.8864	0.8361	0.9792	0.1268
ZNF229	1.0065	1.0306	1.0029	0.8769	0.9792	0.0346
LOC285634	0.9195	1.0632	1.0216	0.9128	0.9793	0.0375
LOC653075	0.9472	1.0719	1.0270	0.8710	0.9793	0.0444
LOC441847	0.9141	1.0703	1.0090	0.9237	0.9793	0.0371
SELV	0.8098	1.1156	1.0786	0.9130	0.9793	0.0716
LOC652618	0.9568	1.0392	1.0167	0.9045	0.9793	0.0304
MGC22014	0.9033	1.0671	1.0628	0.8839	0.9793	0.0496
PPARGC1A	0.9392	1.0842	0.9942	0.8996	0.9793	0.0400
FAM90A16	0.9659	1.0747	1.0407	0.8358	0.9793	0.0530
LOC645261	1.0059	1.0465	0.9538	0.9109	0.9793	0.0297
LOC1001331	0.9078	1.2280	0.9907	0.7907	0.9793	0.0925
LTBP2	0.9436	1.0355	1.1055	0.8325	0.9793	0.0591
LOC1001304	0.9867	1.0973	0.9642	0.8690	0.9793	0.0469
LOC440421	0.9999	1.1376	0.8629	0.9168	0.9793	0.0598
MIR485	0.9575	0.9699	1.0733	0.9166	0.9793	0.0333
DYSFIP1	0.9941	1.0602	0.9571	0.9059	0.9793	0.0325

FLJ11783	1.0018	1.0797	0.9437	0.8921	0.9793	0.0403
MSX2	0.8191	1.1121	1.0500	0.9361	0.9793	0.0647
LOC728157	0.9166	1.1293	1.0780	0.7934	0.9793	0.0768
LOC642830	0.9197	1.0699	1.0350	0.8927	0.9793	0.0432
LOC1001301	0.8980	1.0405	1.0557	0.9231	0.9793	0.0402
ACADS	0.9824	1.2435	0.9563	0.7352	0.9794	0.1040
MIR146B	0.9526	1.0593	1.0010	0.9045	0.9794	0.0331
VAMP1	0.8881	1.0759	1.0669	0.8865	0.9794	0.0532
ADIG	0.9648	1.0554	0.9480	0.9493	0.9794	0.0256
PACRGL	0.9860	1.0688	0.9483	0.9144	0.9794	0.0332
LY9	0.8981	1.0637	0.9813	0.9744	0.9794	0.0338
DIRAS2	0.9332	1.0936	1.0095	0.8812	0.9794	0.0463
KRT86	0.8800	1.0197	1.1198	0.8980	0.9794	0.0561
RHBG	1.0170	1.0763	1.0243	0.7999	0.9794	0.0613
LOC643075	0.9576	1.0238	1.0181	0.9180	0.9794	0.0254
C12orf27	0.9170	1.0811	1.0649	0.8545	0.9794	0.0557
LOC1001283	0.8781	1.0772	1.0724	0.8898	0.9794	0.0552
LOC650203	0.9639	1.1157	1.0486	0.7894	0.9794	0.0705
LHX6	0.9898	1.0331	1.0271	0.8675	0.9794	0.0385
FLJ41047	0.9961	1.0932	0.9327	0.8956	0.9794	0.0432
FLJ42986	0.9383	1.0086	0.9968	0.9739	0.9794	0.0155
MIR1263	0.8494	1.0892	1.0265	0.9525	0.9794	0.0515
LOC643696	0.9519	0.9731	1.0147	0.9779	0.9794	0.0131
LOC651195	0.8770	1.0891	1.1142	0.8374	0.9794	0.0712
LOC391574	0.9694	1.0134	1.0053	0.9296	0.9794	0.0192
CARD18	0.9417	1.0591	1.0248	0.8921	0.9794	0.0381
FLJ10357	0.9114	1.0806	1.0140	0.9118	0.9794	0.0415
ABRA	1.0191	1.0872	0.9451	0.8664	0.9794	0.0476
FAM44A	0.8551	1.1989	0.9832	0.8806	0.9795	0.0782
GEM	0.9733	1.0582	0.9992	0.8871	0.9795	0.0355
PDGFA	0.8700	1.1005	1.0165	0.9308	0.9795	0.0503
LOC732043	0.8743	1.1609	0.9833	0.8994	0.9795	0.0648
TCP11L2	0.8327	1.1281	1.1021	0.8550	0.9795	0.0786
LOC644473	0.9798	1.1530	0.9533	0.8319	0.9795	0.0662
MS4A3	0.9067	1.0870	1.0172	0.9071	0.9795	0.0443
LOC91664	0.9237	1.1100	0.8997	0.9845	0.9795	0.0470
USP30	0.8252	1.3864	0.8799	0.8264	0.9795	0.1362
C6orf15	1.0254	0.9473	1.0305	0.9147	0.9795	0.0288
LOC1001341	0.8805	1.1149	1.0099	0.9127	0.9795	0.0528
LOC388906	1.0026	1.0821	0.9731	0.8603	0.9795	0.0459
LOC642812	0.8640	1.1370	0.9632	0.9538	0.9795	0.0570
EVC2	1.0225	0.9999	0.9755	0.9202	0.9795	0.0220
LOC642597	0.8878	1.1277	1.0436	0.8589	0.9795	0.0639
LOC1001315	0.9047	1.0713	1.0483	0.8937	0.9795	0.0467
C20orf91	0.9918	1.0168	0.9724	0.9370	0.9795	0.0168
DPT	0.8550	1.1248	1.0942	0.8442	0.9795	0.0753

LOC644599	0.9454	1.1719	0.9524	0.8484	0.9795	0.0684
LOC1001284	1.0203	1.0931	0.9123	0.8925	0.9795	0.0471
TBXA2R	0.9363	1.0443	1.0303	0.9073	0.9795	0.0340
LOC646627	0.9029	1.0829	1.0587	0.8736	0.9795	0.0533
LOC651314	0.8961	1.1202	0.9498	0.9522	0.9796	0.0486
LOC1001306	0.8560	1.0908	1.0367	0.9348	0.9796	0.0524
STK10	0.9529	1.0399	1.0013	0.9241	0.9796	0.0256
MYL4	0.9258	1.0736	1.0729	0.8460	0.9796	0.0565
MAGEA4	0.9376	1.1726	0.9346	0.8733	0.9796	0.0660
UQCRB	0.8604	1.3436	1.0452	0.6690	0.9796	0.1436
LOC1001317	0.9213	1.0062	1.0311	0.9597	0.9796	0.0244
SLC34A2	0.9727	1.0544	0.9401	0.9511	0.9796	0.0258
VIM	0.7939	1.2333	1.0477	0.8434	0.9796	0.1008
LOC643386	0.9064	1.0112	1.0406	0.9601	0.9796	0.0295
LOC1001335	0.8954	1.0572	1.0265	0.9392	0.9796	0.0376
KLHL25	0.9619	1.0884	1.0610	0.8070	0.9796	0.0636
OR5J2	0.8881	1.0570	0.9840	0.9892	0.9796	0.0347
SNORA46	0.9678	1.1080	0.9946	0.8480	0.9796	0.0534
ABCC6	0.9114	1.0608	1.0584	0.8879	0.9796	0.0464
LOC1001295	0.9647	1.0266	0.9900	0.9371	0.9796	0.0190
LOC1001332	0.8955	1.0939	1.0037	0.9253	0.9796	0.0444
CDH23	0.9525	1.0977	0.9725	0.8957	0.9796	0.0426
OR1E2	0.9567	1.0326	1.0258	0.9034	0.9796	0.0306
LOC728450	0.9361	1.0052	1.0271	0.9500	0.9796	0.0218
OR4N5	0.9422	1.1054	1.0235	0.8474	0.9796	0.0553
GNA12	0.9334	1.1803	0.9644	0.8404	0.9796	0.0719
LOC651398	0.9378	1.1172	1.0599	0.8036	0.9796	0.0696
C15orf40	0.8178	1.1796	1.0626	0.8585	0.9796	0.0855
LBA1	0.8556	1.0855	1.0776	0.8998	0.9796	0.0596
LOC643072	0.8698	1.1479	0.9948	0.9060	0.9796	0.0619
LOC1001307	0.8752	1.1007	1.0062	0.9364	0.9797	0.0484
ERCC4	0.8387	1.1082	1.0311	0.9407	0.9797	0.0581
FAM3B	0.9006	1.0826	1.1009	0.8345	0.9797	0.0662
LOC653971	0.8699	1.0762	1.0879	0.8848	0.9797	0.0592
TLR7	1.0078	1.0634	0.9987	0.8488	0.9797	0.0459
PTTG2	0.9265	1.1541	0.9604	0.8778	0.9797	0.0605
MGC45800	1.0851	1.0001	0.9595	0.8741	0.9797	0.0438
NLGN3	0.9747	1.1670	0.9011	0.8759	0.9797	0.0659
LOC728711	1.0091	1.0379	0.9839	0.8879	0.9797	0.0325
LOC729032	0.9776	1.0881	0.9700	0.8831	0.9797	0.0420
OR5W2	0.9799	1.0032	0.9988	0.9369	0.9797	0.0152
MYADML	0.8800	1.1917	0.9738	0.8733	0.9797	0.0743
DGAT2L6	0.9386	1.0404	1.0548	0.8850	0.9797	0.0408
ERCC-00120	0.9168	1.1234	0.9428	0.9358	0.9797	0.0482
GNA14	1.0004	1.0150	0.9780	0.9255	0.9797	0.0196
UNC45A	1.0447	1.2878	0.9307	0.6556	0.9797	0.1312

SAA2	0.9609	1.0640	1.0597	0.8342	0.9797	0.0540
P2RX5	0.9262	1.0909	0.9961	0.9057	0.9797	0.0418
NCDN	0.8894	1.1804	1.0604	0.7888	0.9797	0.0873
LOC641860	0.9924	1.0467	0.9843	0.8956	0.9797	0.0313
UNC93A	0.9967	1.0618	0.9367	0.9237	0.9797	0.0316
FAM120B	0.9617	1.3609	0.8271	0.7692	0.9797	0.1333
RGS6	0.8538	1.1167	0.9836	0.9648	0.9797	0.0539
LOC650128	0.8959	1.1869	1.0030	0.8331	0.9797	0.0774
NRG3	0.8743	1.1108	0.9980	0.9358	0.9797	0.0505
LOC651747	0.8970	1.1123	0.9839	0.9258	0.9797	0.0478
POU4F3	0.9558	1.0639	0.9794	0.9200	0.9798	0.0306
ANKAR	0.8572	1.0296	1.0600	0.9722	0.9798	0.0447
L3MBTL2	0.9114	1.2697	0.9887	0.7492	0.9798	0.1088
MDGA2	0.9318	1.0326	1.0448	0.9098	0.9798	0.0344
LOC649299	0.8382	1.0972	1.0796	0.9040	0.9798	0.0642
LOC651746	0.8954	1.1416	0.9477	0.9343	0.9798	0.0551
RABL2A	0.8824	1.1051	0.9708	0.9607	0.9798	0.0462
LOC643378	1.0078	1.0328	0.9998	0.8787	0.9798	0.0344
HHLA1	0.9821	1.0503	0.9933	0.8934	0.9798	0.0324
OR8K1	1.0175	1.0155	0.8888	0.9974	0.9798	0.0307
TNRC9	1.0146	1.0244	1.1048	0.7754	0.9798	0.0711
LOC387646	0.8867	1.0549	1.0644	0.9132	0.9798	0.0465
ARVP6125	0.9253	1.0930	0.9767	0.9241	0.9798	0.0397
GIMAP8	1.0093	1.0008	1.0747	0.8343	0.9798	0.0512
OR2B2	0.7685	1.1633	1.1036	0.8836	0.9798	0.0926
KRTAP19-7	0.9756	1.0506	0.9759	0.9171	0.9798	0.0273
OR8H2	0.9286	1.0461	1.0315	0.9130	0.9798	0.0343
UPK1A	0.9212	1.0620	1.0091	0.9268	0.9798	0.0340
LOC1001292	0.9525	1.1186	1.0209	0.8273	0.9798	0.0612
PCBP4	0.9229	1.1195	0.9887	0.8881	0.9798	0.0510
RBM20	0.9588	1.0393	1.0244	0.8967	0.9798	0.0328
VIL1	0.9133	1.0404	1.0525	0.9131	0.9798	0.0385
GDF6	0.9124	1.0055	1.0760	0.9254	0.9798	0.0381
AIF1	0.9334	1.1626	0.9970	0.8263	0.9798	0.0704
MICALL1	0.9587	1.1439	1.0098	0.8069	0.9798	0.0696
C9orf150	1.0593	0.9829	0.9998	0.8773	0.9798	0.0379
HERC2P2	0.9396	1.0655	1.0113	0.9029	0.9798	0.0364
OR6S1	0.8956	1.0314	1.0800	0.9124	0.9798	0.0450
LOC728807	0.9355	0.9791	1.0571	0.9478	0.9799	0.0273
SSTR4	0.9587	1.0648	0.9937	0.9023	0.9799	0.0340
LOC1001316	0.9456	1.0341	1.0363	0.9034	0.9799	0.0331
NLRP13	0.9904	0.9932	1.0624	0.8735	0.9799	0.0392
LOC648585	0.9862	1.0251	1.0142	0.8940	0.9799	0.0298
OPN1SW	0.8676	0.9739	1.1297	0.9483	0.9799	0.0548
WFDC12	0.9257	1.0892	1.0347	0.8698	0.9799	0.0500
LOC646769	0.9402	1.1224	1.0218	0.8351	0.9799	0.0610

TTBK1	0.8896	1.1059	1.1179	0.8061	0.9799	0.0782
CBWD1	0.9393	1.0782	0.9762	0.9259	0.9799	0.0344
KCNIP4	0.9344	1.0788	0.9962	0.9101	0.9799	0.0376
LOC727899	0.8519	1.0617	1.1220	0.8839	0.9799	0.0661
LOC441340	0.9039	1.0028	1.1460	0.8668	0.9799	0.0624
LOC1001348	0.9291	1.1008	1.0317	0.8580	0.9799	0.0538
LOC728419	0.9171	1.0045	1.0652	0.9327	0.9799	0.0342
LOC644457	0.8498	1.0853	0.9937	0.9908	0.9799	0.0486
GH2	0.9148	1.0689	1.0527	0.8832	0.9799	0.0473
CR1	0.9463	1.0563	0.9794	0.9375	0.9799	0.0270
ETV3L	0.9635	1.0917	0.9396	0.9248	0.9799	0.0381
MIR181A1	0.9038	0.9457	1.0661	1.0040	0.9799	0.0353
LOC643045	0.8739	1.0693	1.0636	0.9130	0.9799	0.0506
KIR2DS1	0.9537	1.0493	0.9952	0.9215	0.9799	0.0276
TMSB10	0.8205	1.2166	1.0410	0.8416	0.9799	0.0932
SNORD121A	0.9327	1.1005	0.9236	0.9630	0.9799	0.0411
FAM19A2	0.9187	1.1243	0.9631	0.9136	0.9799	0.0494
LOC645992	1.0433	1.0800	0.9194	0.8771	0.9799	0.0485
COLEC11	0.9362	1.0974	1.0414	0.8448	0.9799	0.0561
ASXL3	0.9561	1.0599	0.9730	0.9308	0.9799	0.0280
PDS5A	0.8930	1.3909	0.8929	0.7430	0.9799	0.1415
LOC652195	1.0068	1.0404	1.1605	0.7121	0.9799	0.0952
HIST1H1A	0.7974	1.2625	1.0442	0.8156	0.9800	0.1096
LOC646709	0.9560	1.1360	1.0563	0.7715	0.9800	0.0786
KIAA1245	0.9338	1.0150	1.0420	0.9291	0.9800	0.0286
HTR1B	0.9263	1.1208	0.8817	0.9910	0.9800	0.0520
TTTY9A	1.0003	1.0152	1.0144	0.8900	0.9800	0.0302
LOC653569	0.8855	1.1631	1.0238	0.8473	0.9800	0.0719
SLC22A20	1.0120	1.0518	1.0526	0.8034	0.9800	0.0596
LIN7A	0.7304	1.3313	1.1709	0.6872	0.9800	0.1602
KIAA0888	0.9086	1.0850	1.0247	0.9017	0.9800	0.0450
PIGH	0.8226	1.2045	1.2075	0.6853	0.9800	0.1335
TMED6	0.8740	1.0249	1.0160	1.0050	0.9800	0.0356
BOC	0.8892	1.0534	1.0319	0.9454	0.9800	0.0382
LOC645218	0.9183	1.1119	0.9856	0.9041	0.9800	0.0474
MAP4K1	0.9297	1.0851	0.9826	0.9225	0.9800	0.0375
psiTPTE22	0.9135	1.0176	1.1018	0.8871	0.9800	0.0494
TRPV1	0.9416	1.0365	1.0040	0.9379	0.9800	0.0242
IGF1R	1.0639	1.0952	0.9157	0.8453	0.9800	0.0596
KIAA0467	0.9450	0.9896	1.0624	0.9230	0.9800	0.0308
LOC642222	0.8874	1.2431	0.9096	0.8800	0.9800	0.0879
CDC2L5	0.8502	1.2230	1.0001	0.8468	0.9800	0.0885
LOC391092	0.9249	1.1152	1.0133	0.8666	0.9800	0.0542
LOC645745	0.9550	0.9838	1.0562	0.9250	0.9800	0.0281
LOC402199	0.9861	1.0593	0.9943	0.8805	0.9800	0.0370
APOE	0.9334	1.0245	1.0814	0.8809	0.9800	0.0450

LOC650914	0.8810	1.0635	1.0578	0.9179	0.9800	0.0472
MAK	1.0000	1.2014	0.8823	0.8364	0.9800	0.0814
KCNJ8	0.8210	1.1704	1.0479	0.8809	0.9800	0.0796
GALNT9	0.9402	1.0828	1.0192	0.8780	0.9800	0.0448
AKT3	0.9105	1.0821	1.0448	0.8828	0.9800	0.0491
LOC648667	0.9442	0.9885	1.0476	0.9399	0.9800	0.0251
MIR1293	0.9801	1.1561	0.9287	0.8553	0.9800	0.0640
LOC650034	0.8638	1.1750	0.9989	0.8824	0.9800	0.0715
VTRNA1-1	0.9410	0.9137	1.2760	0.7896	0.9801	0.1040
VSTM2	1.0021	1.0058	0.9698	0.9425	0.9801	0.0149
LOC392264	0.9244	1.1087	0.9478	0.9394	0.9801	0.0431
MIR548N	0.9325	0.9780	1.0644	0.9454	0.9801	0.0297
PHLDB2	0.9956	1.1014	0.9211	0.9022	0.9801	0.0452
HBB	0.9880	1.0375	0.9439	0.9509	0.9801	0.0215
LOC653338	0.8902	1.0479	1.0750	0.9073	0.9801	0.0474
LOC647941	0.8561	1.1313	0.9679	0.9652	0.9801	0.0567
FABP9	0.9683	1.0658	1.0217	0.8647	0.9801	0.0433
LOC643033	0.8788	1.0836	1.0426	0.9156	0.9801	0.0492
LOC644388	0.9667	0.9151	1.0657	0.9730	0.9801	0.0313
SLC3A1	0.9319	1.0986	0.9769	0.9132	0.9801	0.0417
LOC90342	0.9488	1.0670	1.0369	0.8679	0.9801	0.0451
LOC1001324	1.0997	1.0528	0.9393	0.8287	0.9801	0.0607
H1FOO	0.9731	1.0974	1.0071	0.8430	0.9801	0.0527
C12orf8	0.9327	1.0847	1.0550	0.8482	0.9801	0.0549
LOC285346	1.0234	1.1453	0.9177	0.8342	0.9801	0.0673
SLA2	0.9245	1.1109	0.9739	0.9113	0.9801	0.0456
DKFZp686K1	0.8470	1.1933	1.0047	0.8756	0.9802	0.0789
LOC653806	0.8861	1.1579	0.9626	0.9140	0.9802	0.0613
LOC644286	0.9035	1.1024	0.9309	0.9838	0.9802	0.0440
LOC1001294	0.9401	1.1172	1.0433	0.8200	0.9802	0.0646
MIR26A2	0.9505	1.0416	1.0198	0.9088	0.9802	0.0307
LOC644380	0.9087	1.0327	1.0822	0.8971	0.9802	0.0458
C21orf129	1.0585	0.9824	0.9758	0.9041	0.9802	0.0315
PLAU	0.8207	0.9880	1.1585	0.9534	0.9802	0.0695
TMEM133	1.0022	1.0143	1.0151	0.8891	0.9802	0.0305
FABP5L3	0.9689	1.0126	0.9788	0.9605	0.9802	0.0114
ATP1B4	0.9448	1.0454	1.0673	0.8633	0.9802	0.0472
IRF4	0.9307	1.0504	1.0886	0.8511	0.9802	0.0546
LOC644068	1.0000	1.0061	0.9486	0.9660	0.9802	0.0137
LOC731106	0.9427	1.0510	1.0046	0.9225	0.9802	0.0294
SLC25A31	0.8857	1.0989	1.0848	0.8514	0.9802	0.0649
NAB2	0.8525	1.4923	0.9632	0.6128	0.9802	0.1857
NT5C1A	0.9122	0.9736	0.9895	1.0455	0.9802	0.0274
ONECUT3	0.9531	0.9222	1.0391	1.0065	0.9802	0.0262
LOC146481	0.9110	1.0192	1.0555	0.9351	0.9802	0.0342
C6orf203	0.9818	1.0443	1.0622	0.8326	0.9802	0.0521

LOC727960	0.9458	1.1173	0.9679	0.8899	0.9802	0.0486
LOC1001309	0.9126	1.1217	0.9681	0.9186	0.9802	0.0488
LOC729920	0.8878	1.1224	0.9913	0.9194	0.9802	0.0521
LOC644715	0.8622	1.2429	0.9913	0.8246	0.9802	0.0945
LOC1001332	0.9133	1.0140	1.0234	0.9703	0.9802	0.0251
LOC389362	0.9994	1.1526	0.9982	0.7708	0.9802	0.0787
LOC133993	0.9270	1.0652	0.9900	0.9388	0.9802	0.0314
LOC646664	0.9397	0.9880	1.1182	0.8752	0.9803	0.0514
LOC727858	0.9075	1.1024	1.0154	0.8957	0.9803	0.0488
LOC646103	0.9735	1.0670	1.0731	0.8075	0.9803	0.0619
MTMR8	1.0084	0.9986	1.0230	0.8911	0.9803	0.0302
LLGL1	0.9759	1.4550	0.7290	0.7612	0.9803	0.1675
LOC387940	1.0142	1.0228	1.0220	0.8620	0.9803	0.0395
ZNF432	0.8856	1.2021	0.9388	0.8946	0.9803	0.0749
CTRB2	0.8872	1.0539	0.9739	1.0061	0.9803	0.0351
LOC1001339	0.9535	1.0244	0.9928	0.9505	0.9803	0.0176
DPPA3	0.9166	1.1243	1.0368	0.8434	0.9803	0.0624
LOC644412	0.9764	1.0070	1.0539	0.8838	0.9803	0.0359
LOC1001295	0.8973	1.0197	1.0967	0.9075	0.9803	0.0477
MIR598	0.8957	1.1779	0.9375	0.9101	0.9803	0.0664
LOC644285	0.9634	1.0188	0.9723	0.9668	0.9803	0.0130
CUGBP2	0.9004	1.0311	1.0112	0.9787	0.9803	0.0288
ATXN7	0.9174	1.0804	1.1057	0.8179	0.9803	0.0684
LENG1	0.8207	1.1831	0.9679	0.9497	0.9803	0.0751
RPL23AP13	0.9528	1.0481	1.1325	0.7880	0.9804	0.0739
C18orf2	0.8786	1.0835	1.0366	0.9227	0.9804	0.0478
ECD	0.7909	1.2513	1.1308	0.7484	0.9804	0.1244
TLR4	0.9787	1.1012	0.9307	0.9108	0.9804	0.0427
MIR1202	0.9397	1.0394	1.0123	0.9301	0.9804	0.0269
RCVRN	0.8433	1.0993	0.9831	0.9956	0.9804	0.0526
LOC647181	0.9470	1.1024	0.9537	0.9184	0.9804	0.0414
LOC651603	0.8935	0.9989	1.0936	0.9355	0.9804	0.0435
APOC4	0.8655	1.0724	1.0228	0.9608	0.9804	0.0446
LOC1001347	0.9175	1.1424	0.8174	1.0443	0.9804	0.0712
CLNK	0.9065	1.1906	0.9295	0.8950	0.9804	0.0704
PBLD	0.9168	1.1067	1.0058	0.8923	0.9804	0.0487
LOC652659	0.8895	1.1050	1.0189	0.9083	0.9804	0.0504
KIAA1644	0.8671	1.2319	0.9270	0.8957	0.9804	0.0847
TCEB3CL	0.8941	1.0810	1.0015	0.9451	0.9804	0.0401
TBC1D3E	0.8931	1.0986	1.0309	0.8990	0.9804	0.0506
HTN3	1.0014	1.1421	0.9064	0.8717	0.9804	0.0605
SPP1	0.8843	1.0845	1.1164	0.8366	0.9804	0.0703
LOC644516	1.0029	1.0525	0.9886	0.8778	0.9804	0.0368
LOC641719	0.9053	1.1276	1.0095	0.8795	0.9804	0.0565
LOC1001294	0.8957	1.1047	1.0349	0.8866	0.9805	0.0535
RCL1	0.7944	1.5104	0.9844	0.6327	0.9805	0.1907

KRT33A	0.9434	1.1367	0.9628	0.8790	0.9805	0.0551
LOC649917	0.9472	1.0810	0.9848	0.9089	0.9805	0.0369
SNTN	1.0065	1.0134	0.9810	0.9210	0.9805	0.0210
IFNA4	0.8899	1.1102	1.0269	0.8948	0.9805	0.0536
HAVCR1	0.9500	1.1211	0.9198	0.9310	0.9805	0.0473
C13orf31	1.0091	1.1404	0.9083	0.8642	0.9805	0.0613
SPOCK3	0.9528	1.1046	1.0331	0.8315	0.9805	0.0586
OR5AN1	1.0115	1.1184	0.8993	0.8928	0.9805	0.0534
C4orf40	0.9144	1.0239	1.0582	0.9254	0.9805	0.0357
SPNS3	0.8506	1.0457	1.0474	0.9783	0.9805	0.0462
LOC645277	0.9481	1.0842	1.0589	0.8308	0.9805	0.0580
ENPP2	0.9507	1.0340	1.0117	0.9256	0.9805	0.0254
DSC1	0.8801	1.1184	1.0731	0.8504	0.9805	0.0674
KIR2DL5A	0.9595	1.0414	1.0001	0.9210	0.9805	0.0259
C17orf62	0.9490	1.2640	0.9422	0.7668	0.9805	0.1035
NDRG4	0.9398	1.0362	1.0338	0.9123	0.9805	0.0319
PDGFRA	0.9910	0.9940	0.9886	0.9486	0.9805	0.0107
SOCS1	0.9253	1.1107	1.0342	0.8518	0.9805	0.0573
RASSF3	0.6499	1.3324	1.0715	0.8683	0.9805	0.1455
TRAF4	0.9288	1.2111	0.9975	0.7847	0.9805	0.0887
LOC389857	1.0405	1.0659	0.8927	0.9231	0.9805	0.0427
CXCR5	0.9214	1.1004	1.0012	0.8991	0.9805	0.0456
LOC642168	0.9129	0.9983	1.0624	0.9485	0.9805	0.0324
NT5DC3	0.7421	1.4766	0.9919	0.7115	0.9805	0.1769
ADAMTSL1	0.9462	1.0652	1.0395	0.8713	0.9805	0.0445
KGFLP2	0.7899	1.0130	1.1503	0.9690	0.9805	0.0743
KIF2B	0.8562	1.0672	1.0266	0.9723	0.9805	0.0458
LAMC3	1.0418	1.0470	0.9924	0.8410	0.9805	0.0481
ZNF682	0.8241	1.2859	1.0266	0.7856	0.9805	0.1147
LOC643201	0.8873	1.0659	1.0277	0.9412	0.9806	0.0406
HIST1H2BB	0.9423	1.1305	0.9652	0.8842	0.9806	0.0528
LOC1001341	0.9234	1.1286	1.0300	0.8403	0.9806	0.0628
SNORD22	0.9104	1.0631	0.9845	0.9643	0.9806	0.0316
LOC401072	0.9578	1.0679	0.9954	0.9011	0.9806	0.0350
LOC283523	0.9720	1.0699	0.9893	0.8911	0.9806	0.0367
LOC643441	0.9539	1.1065	0.9727	0.8892	0.9806	0.0456
PDE11A	0.9344	1.0434	1.0439	0.9007	0.9806	0.0371
NIPAL2	0.9375	1.1467	0.9969	0.8413	0.9806	0.0640
LOC642859	0.9920	1.0504	0.9553	0.9247	0.9806	0.0270
FXYD4	0.9031	1.0598	1.0002	0.9592	0.9806	0.0330
MIR92A2	0.9403	1.0486	1.0639	0.8696	0.9806	0.0461
RNF148	0.9389	1.1163	0.8758	0.9914	0.9806	0.0510
OR8G5	0.9586	1.0927	1.0538	0.8172	0.9806	0.0613
LOC390372	0.9979	1.1538	0.9139	0.8568	0.9806	0.0646
LOC644968	1.0018	1.0031	1.1251	0.7925	0.9806	0.0691
C6orf156	0.9860	1.0711	1.0665	0.7989	0.9806	0.0636

LOC727818	0.7890	1.1066	1.2513	0.7755	0.9806	0.1183
HARS	0.8923	1.4028	0.9563	0.6711	0.9806	0.1534
LOC1001296	1.0558	1.0603	0.9939	0.8125	0.9806	0.0580
TMEM213	0.8783	1.0311	1.0281	0.9850	0.9806	0.0357
FABP12	0.9457	1.0270	1.0219	0.9279	0.9806	0.0256
RC3H1	0.8866	1.1259	1.1638	0.7462	0.9806	0.0993
LOC643210	0.8374	1.2196	0.9612	0.9044	0.9806	0.0836
GPR119	1.0240	0.9254	1.0450	0.9283	0.9806	0.0314
KBTBD9	0.8985	1.1690	0.9638	0.8914	0.9807	0.0649
KCNG2	0.9919	0.9928	0.9910	0.9469	0.9807	0.0113
BMPR1A	1.0303	1.2358	0.9166	0.7399	0.9807	0.1039
ANXA3	0.9884	1.0654	1.0178	0.8510	0.9807	0.0460
LOC649941	0.9954	1.0104	1.0004	0.9164	0.9807	0.0217
C5orf48	0.8984	1.0631	1.0058	0.9554	0.9807	0.0352
GABRR1	1.0090	0.9488	0.9953	0.9695	0.9807	0.0134
LOC647323	1.0229	0.9083	1.0122	0.9793	0.9807	0.0258
MIR559	0.9596	1.0456	1.0163	0.9011	0.9807	0.0320
GPR65	1.0052	1.0654	0.9382	0.9138	0.9807	0.0342
LOC1001285	0.8379	1.2775	0.9469	0.8604	0.9807	0.1017
PPAP2C	0.9826	1.2846	0.9227	0.7328	0.9807	0.1145
FAM48B2	0.9232	1.0774	1.0110	0.9111	0.9807	0.0392
BMP7	0.9235	1.1249	0.9974	0.8770	0.9807	0.0541
LOC731656	0.9506	1.0610	1.0451	0.8659	0.9807	0.0454
LOC1001337	0.9128	1.1240	1.0194	0.8665	0.9807	0.0575
FLJ33387	0.9249	1.0693	1.1108	0.8178	0.9807	0.0674
TMEM150A	0.9149	1.3095	0.9328	0.7656	0.9807	0.1158
ELMO1	0.9702	1.0780	0.9813	0.8933	0.9807	0.0379
GSTA2	1.0024	1.0746	0.9176	0.9282	0.9807	0.0365
LOC644322	0.9396	1.1083	1.0355	0.8394	0.9807	0.0584
LOC653073	0.9606	1.0221	1.0125	0.9277	0.9807	0.0222
SPATA7	0.8867	1.1996	1.0317	0.8049	0.9807	0.0867
LOC1001324	0.9498	1.0962	0.9914	0.8856	0.9807	0.0442
TRIM63	0.9734	1.0235	1.0267	0.8993	0.9807	0.0298
CTAG1B	0.9342	1.0697	1.0235	0.8955	0.9807	0.0400
LOC644950	0.7610	1.1766	1.0462	0.9392	0.9807	0.0879
LOC651848	0.9306	1.1256	0.9730	0.8938	0.9807	0.0509
S100A9	1.0848	1.0126	1.0095	0.8161	0.9808	0.0576
LOC651285	0.9544	1.0877	0.9954	0.8855	0.9808	0.0422
LOC1001345	0.9664	1.0897	1.0129	0.8541	0.9808	0.0493
TESSP5	0.8581	1.0646	1.1290	0.8712	0.9808	0.0683
LOC652291	0.8966	1.1242	1.0043	0.8979	0.9808	0.0541
CSDE1	0.6935	1.4447	1.0326	0.7523	0.9808	0.1714
LOC1001305	0.9526	1.0764	1.0234	0.8708	0.9808	0.0446
C15orf49	1.0386	1.0614	1.0372	0.7859	0.9808	0.0652
LOC651763	1.0433	0.9987	0.9704	0.9107	0.9808	0.0278
LOC1001348	0.9174	1.0922	0.9965	0.9171	0.9808	0.0416

PRAMEF18	1.0870	1.0865	0.9951	0.7546	0.9808	0.0784
LOC349196	0.8650	1.1201	1.0260	0.9121	0.9808	0.0574
LOC391322	1.0016	1.0658	1.0637	0.7922	0.9808	0.0646
FAM83A	0.9676	0.9938	1.0511	0.9107	0.9808	0.0292
LOC441196	0.9297	1.0498	1.0021	0.9417	0.9808	0.0279
FLJ46380	0.8904	1.1882	1.0129	0.8319	0.9808	0.0787
KRTAP19-2	0.9543	1.0713	1.0228	0.8749	0.9808	0.0427
LOC727747	0.8887	1.1849	0.9974	0.8523	0.9808	0.0747
MIR488	0.7615	1.1546	1.1851	0.8221	0.9808	0.1100
LOC728127	0.8693	1.1391	1.0756	0.8394	0.9809	0.0744
PROSC	0.7805	1.3683	0.9383	0.8363	0.9809	0.1332
MIR563	1.0240	1.0733	0.9063	0.9199	0.9809	0.0405
C1orf222	0.9075	1.1025	0.9821	0.9314	0.9809	0.0434
CT47A1	0.9502	1.0609	1.0359	0.8765	0.9809	0.0421
PATL2	0.9639	1.0698	0.9661	0.9237	0.9809	0.0312
CAST	0.9258	1.1339	1.0777	0.7861	0.9809	0.0784
PARD6B	0.9511	0.9349	1.1283	0.9092	0.9809	0.0499
C4orf49	0.9864	1.1067	0.9691	0.8613	0.9809	0.0503
LOC728653	1.0801	1.0778	0.8910	0.8747	0.9809	0.0567
LOC647588	0.9188	1.1665	0.9091	0.9292	0.9809	0.0620
CSPG4	0.9204	1.1159	1.0560	0.8313	0.9809	0.0645
LOC1001292	0.9425	0.9792	1.0416	0.9603	0.9809	0.0216
LOC389316	0.8514	1.0995	0.9398	1.0329	0.9809	0.0542
VPS13B	0.9439	1.0675	1.0103	0.9019	0.9809	0.0365
AGPAT1	0.9276	1.1931	1.0136	0.7893	0.9809	0.0845
B3GAT2	0.9415	1.0746	0.9715	0.9361	0.9809	0.0322
BACE2	0.9445	1.0735	0.9610	0.9446	0.9809	0.0311
TTTY9B	0.9461	1.1042	0.9788	0.8946	0.9809	0.0446
TRIM22	0.8697	1.1393	0.9885	0.9262	0.9809	0.0581
LOC1001327	0.9256	1.1580	0.9886	0.8516	0.9809	0.0653
LOC643824	0.8570	1.1689	0.9476	0.9503	0.9809	0.0663
GZMM	0.9685	0.9670	1.0402	0.9480	0.9809	0.0203
CYP4B1	0.9458	1.0406	0.9928	0.9446	0.9809	0.0228
FBXL18	0.8844	1.3234	0.9378	0.7782	0.9809	0.1189
TCTEX1D4	0.9673	0.9963	0.9866	0.9736	0.9809	0.0065
LOC1001285	0.9918	1.0272	0.9983	0.9066	0.9810	0.0260
TCEAL5	0.9300	1.1826	0.9834	0.8277	0.9810	0.0746
SYT12	0.9163	1.0659	0.9820	0.9597	0.9810	0.0314
USH1G	0.8698	1.0468	1.1660	0.8412	0.9810	0.0766
NAGS	0.9390	1.1525	0.9601	0.8722	0.9810	0.0602
LOC442454	0.7722	1.2430	1.2785	0.6301	0.9810	0.1643
GCKR	0.8468	1.0422	1.0759	0.9591	0.9810	0.0510
LOC731992	0.9683	1.0349	0.9824	0.9384	0.9810	0.0202
GAD2	0.9055	1.0902	1.0029	0.9253	0.9810	0.0420
LOC649091	0.8614	1.0186	1.0232	1.0208	0.9810	0.0399
RPP14	0.9544	1.1688	1.0005	0.8002	0.9810	0.0759

ZNF354C	0.9699	1.0980	0.9740	0.8820	0.9810	0.0444
TSLP	0.9088	1.0246	1.1117	0.8790	0.9810	0.0537
RSAD2	0.9301	1.1171	1.0819	0.7950	0.9810	0.0741
LOC650968	0.8949	1.1406	0.9906	0.8979	0.9810	0.0577
POGZ	0.9397	1.1109	1.0192	0.8542	0.9810	0.0549
LOC729960	0.9225	1.0822	1.0186	0.9007	0.9810	0.0424
FLJ37644	0.9132	1.0213	1.0389	0.9506	0.9810	0.0296
LOC651732	0.9226	0.9611	1.0912	0.9491	0.9810	0.0376
MFRP	0.9247	1.1023	1.0582	0.8389	0.9810	0.0606
TMPRSS11D	0.8897	1.0783	1.0475	0.9086	0.9810	0.0478
LOC642902	0.9168	1.0856	1.0500	0.8716	0.9810	0.0515
TBX2	0.8978	1.1460	0.9865	0.8937	0.9810	0.0590
LRTM1	0.9492	1.0310	1.0768	0.8671	0.9810	0.0462
CNGB1	0.8848	1.1030	1.0681	0.8681	0.9810	0.0609
ENTPD2	0.9038	1.1580	0.9745	0.8878	0.9810	0.0619
OR5T3	0.9381	1.0286	1.0452	0.9122	0.9810	0.0329
C15orf27	0.8561	1.1222	0.9665	0.9793	0.9810	0.0546
ZACN	0.8825	1.1460	0.9595	0.9362	0.9810	0.0573
LOC1002332	1.0287	1.1036	1.0147	0.7772	0.9810	0.0707
SH2D1B	1.0569	1.0106	1.0158	0.8407	0.9810	0.0479
LOC643140	0.9551	1.1405	0.9870	0.8415	0.9810	0.0617
C9orf130	0.9298	1.1389	0.9798	0.8757	0.9811	0.0568
MICAL3	0.8771	1.1290	1.0141	0.9040	0.9811	0.0575
DNAH10	0.9384	1.0367	1.0983	0.8509	0.9811	0.0545
OTOP2	0.9693	1.0283	0.9629	0.9638	0.9811	0.0158
EGLN3	0.8439	1.1569	1.0098	0.9136	0.9811	0.0678
TGFBI	1.0046	1.0941	1.0202	0.8053	0.9811	0.0618
CYP2D7P1	0.9432	1.1054	0.9605	0.9152	0.9811	0.0425
CCIN	0.9041	1.1494	0.9611	0.9097	0.9811	0.0575
LOC728855	0.9724	1.2508	1.0033	0.6978	0.9811	0.1131
LOC653210	0.9512	1.0467	1.0221	0.9044	0.9811	0.0326
TUBB1	0.9452	1.1456	1.0089	0.8245	0.9811	0.0669
LOC1001318	0.9953	1.0376	1.0192	0.8722	0.9811	0.0373
GNAZ	0.9176	1.1080	0.9932	0.9056	0.9811	0.0465
LOC647988	0.9282	1.1468	0.9975	0.8519	0.9811	0.0627
SNORD108	0.8709	1.0781	0.9907	0.9846	0.9811	0.0425
SLITRK4	0.9821	0.9604	1.0206	0.9612	0.9811	0.0141
C18orf22	0.8891	1.2450	1.0849	0.7053	0.9811	0.1172
LOC1001284	0.8689	1.0103	1.0770	0.9682	0.9811	0.0436
TRMT2B	0.8213	1.2049	1.0303	0.8679	0.9811	0.0870
LOC1001302	0.9507	1.0319	1.0240	0.9178	0.9811	0.0279
C15orf5	0.9880	1.0643	1.0567	0.8154	0.9811	0.0578
LOC647891	0.9156	1.0868	1.0147	0.9073	0.9811	0.0429
EVI2B	1.0301	1.0804	0.9288	0.8851	0.9811	0.0449
LOC731548	0.9809	1.0267	1.0328	0.8841	0.9811	0.0344
MIR200A	0.9237	1.0233	1.0003	0.9772	0.9811	0.0213

LOC646448	0.9149	1.1106	1.0254	0.8736	0.9811	0.0538
PREX2	0.9328	1.0900	0.9998	0.9019	0.9811	0.0417
C4orf7	1.0296	0.9751	0.9983	0.9215	0.9811	0.0228
RNF141	0.9156	1.1495	0.9969	0.8625	0.9811	0.0626
TUBAL3	0.9599	1.0948	0.9897	0.8801	0.9811	0.0444
GAPT	0.8758	1.0923	0.9977	0.9587	0.9811	0.0449
LOH3CR2A	1.0400	1.0647	0.9437	0.8760	0.9811	0.0437
LOC645336	0.8589	1.0901	1.0603	0.9152	0.9811	0.0559
TRIM33	0.8670	1.2918	0.9338	0.8319	0.9811	0.1057
OR4A47	0.8632	1.0903	1.0820	0.8890	0.9811	0.0609
C1orf68	0.8668	1.0779	1.0681	0.9118	0.9811	0.0539
C1orf141	0.9227	1.0706	0.9908	0.9404	0.9811	0.0331
SNCB	0.9390	1.0820	0.9905	0.9131	0.9811	0.0373
AFG3L1	0.9001	1.1166	0.9894	0.9184	0.9811	0.0491
KRTAP9-4	0.9764	1.0391	0.9831	0.9260	0.9812	0.0231
C20orf160	0.9177	1.1237	0.9826	0.9006	0.9812	0.0507
LOC1001307	0.8656	1.0888	0.9578	1.0126	0.9812	0.0470
FLJ43681	0.7933	1.1234	1.4783	0.5296	0.9812	0.2055
LOC651965	1.0646	1.0037	0.9172	0.9392	0.9812	0.0333
MLN	0.8896	1.0241	1.0060	1.0050	0.9812	0.0309
SESN2	0.8826	1.3767	0.8362	0.8293	0.9812	0.1324
LOC1001285	0.9065	1.1094	1.0473	0.8615	0.9812	0.0583
LOC642635	0.9321	1.1230	1.0037	0.8659	0.9812	0.0550
LOC391703	0.9620	1.0326	1.0866	0.8436	0.9812	0.0525
TRIM58	1.0531	1.0184	0.9584	0.8950	0.9812	0.0348
C3orf17	0.9327	1.1007	1.0063	0.8851	0.9812	0.0470
MAPK8IP3	1.0216	1.0700	0.9238	0.9094	0.9812	0.0387
C12orf68	0.8296	1.1784	1.0032	0.9136	0.9812	0.0747
LOC641835	0.8852	1.0284	1.0084	1.0030	0.9812	0.0325
MIR597	0.9245	1.0868	0.9823	0.9313	0.9812	0.0375
OR5T2	0.9419	1.0444	0.9635	0.9750	0.9812	0.0221
KCNK6	1.0070	1.0459	0.9856	0.8865	0.9812	0.0340
NDUFA3	0.8878	1.1807	1.0503	0.8062	0.9812	0.0836
LOC652814	0.8786	0.9793	1.0679	0.9992	0.9812	0.0391
PPAPDC3	0.9122	1.0498	0.9946	0.9683	0.9812	0.0286
LOC651075	0.9070	1.1369	0.9711	0.9100	0.9812	0.0539
TBX4	0.9108	1.0361	1.0686	0.9094	0.9812	0.0416
LOC653468	0.9799	1.0460	0.9854	0.9137	0.9812	0.0270
PTGS2	0.9810	1.0955	0.9231	0.9254	0.9812	0.0404
CHD7	0.8106	1.1685	1.0577	0.8882	0.9813	0.0810
LOC441171	1.0044	1.1314	0.9432	0.8461	0.9813	0.0597
NRXN3	0.9101	1.0964	1.0044	0.9142	0.9813	0.0441
GGCX	0.8747	1.1707	1.0872	0.7925	0.9813	0.0885
C10orf136	0.8626	1.1445	0.9921	0.9259	0.9813	0.0605
LOC653737	0.8265	1.1356	1.2711	0.6919	0.9813	0.1340
ADPRH	0.8516	1.1810	1.0021	0.8904	0.9813	0.0738

LOC1001332	1.0153	0.9744	1.0714	0.8640	0.9813	0.0439
HSPB6	0.9778	1.0721	0.9482	0.9271	0.9813	0.0320
LOC1001328	0.9147	1.1410	0.9473	0.9222	0.9813	0.0537
LOC643363	1.0479	1.0544	0.8899	0.9330	0.9813	0.0413
C1orf127	0.9605	1.0998	0.9384	0.9265	0.9813	0.0401
TPPP3	0.9129	1.0914	1.0400	0.8810	0.9813	0.0502
SKIV2L	0.9421	1.2019	1.0320	0.7492	0.9813	0.0943
CYP2D6	1.0003	1.0730	0.9747	0.8772	0.9813	0.0405
LOC1001316	1.0462	1.0259	0.9947	0.8585	0.9813	0.0423
LOC1001305	0.9307	1.0763	0.9441	0.9742	0.9813	0.0329
TYW1B	0.8534	1.3226	0.9561	0.7933	0.9813	0.1186
COL8A2	0.9132	1.1594	0.9790	0.8737	0.9813	0.0632
WIZ	1.0236	1.2332	0.8176	0.8509	0.9813	0.0953
CCNB1IP1	0.8593	1.4414	0.9127	0.7118	0.9813	0.1591
LOC646970	0.9277	1.0936	1.0932	0.8108	0.9813	0.0690
MIR1247	0.9429	1.0686	0.9275	0.9863	0.9813	0.0316
LOC653280	1.0019	1.1444	0.9413	0.8378	0.9813	0.0640
MCCD1	0.9223	1.1684	0.9320	0.9027	0.9813	0.0626
GRPR	0.9457	1.1149	1.0015	0.8633	0.9813	0.0528
LOC650747	0.7794	1.1651	1.0875	0.8934	0.9813	0.0883
LOC652630	0.8974	1.1781	1.0379	0.8120	0.9814	0.0804
FAM9B	0.9503	1.1054	0.9415	0.9282	0.9814	0.0416
ABCC6P1	0.9554	1.0631	1.0222	0.8847	0.9814	0.0391
KIAA1826	0.8273	1.3326	1.0043	0.7613	0.9814	0.1278
LOC728597	0.8894	1.1162	0.9933	0.9265	0.9814	0.0498
LOC389124	1.0222	1.1422	0.9102	0.8509	0.9814	0.0643
LOC730242	0.9623	1.1229	0.9154	0.9250	0.9814	0.0482
LOC646881	0.9776	1.0352	1.0829	0.8299	0.9814	0.0549
TMCO3	0.8962	1.3683	0.8737	0.7874	0.9814	0.1311
LOC731779	0.8387	1.0958	1.1638	0.8272	0.9814	0.0868
C1orf126	0.8853	1.0236	1.0171	0.9996	0.9814	0.0324
RAP1B	0.8983	1.0802	1.0458	0.9013	0.9814	0.0476
LOC641858	0.9507	1.0550	1.0382	0.8817	0.9814	0.0403
LOC653789	0.8949	1.0329	1.0817	0.9163	0.9814	0.0451
SERINC4	0.8879	1.0067	1.0923	0.9387	0.9814	0.0443
LOC1001289	0.9919	1.0969	0.9191	0.9178	0.9814	0.0422
C20orf11	0.9013	1.3211	0.9386	0.7647	0.9814	0.1192
LOC649352	0.9792	1.0690	0.9711	0.9063	0.9814	0.0335
LOC1001320	0.8742	1.0128	1.0134	1.0253	0.9814	0.0358
POPDC3	0.9335	1.0292	1.0378	0.9251	0.9814	0.0302
LOC1001307	0.8928	0.9859	1.0395	1.0075	0.9814	0.0315
G6PC	0.8419	1.1092	1.0844	0.8902	0.9814	0.0675
LOC728185	0.9225	1.1369	0.9657	0.9005	0.9814	0.0536
GTSF1	0.9102	1.1571	0.8670	0.9913	0.9814	0.0640
ASH2L	0.8908	1.4415	0.9327	0.6608	0.9814	0.1646
C20orf77	0.9757	1.0902	1.0079	0.8518	0.9814	0.0495

LOC646734	0.9655	0.9967	1.0772	0.8864	0.9814	0.0395
TMEM171	0.9303	1.0651	0.9489	0.9815	0.9814	0.0298
LOC400174	0.8612	1.2037	0.9511	0.9098	0.9815	0.0763
ZSCAN10	0.9868	1.0781	1.0074	0.8535	0.9815	0.0469
MYL1	0.9157	1.0994	0.9952	0.9157	0.9815	0.0435
LOC651675	0.9628	0.9893	1.0348	0.9389	0.9815	0.0205
LOC649944	0.9281	0.9927	1.1060	0.8992	0.9815	0.0459
LOC1001295	1.0043	1.0637	1.0272	0.8307	0.9815	0.0517
CYP26C1	0.9088	1.1151	0.9701	0.9319	0.9815	0.0463
LOC1001329	0.9171	1.1076	0.9707	0.9306	0.9815	0.0435
VWDE	0.9124	1.0356	1.1090	0.8690	0.9815	0.0552
QPCT	1.0164	1.0163	1.0439	0.8493	0.9815	0.0445
LOC646665	0.9693	1.0360	1.0177	0.9031	0.9815	0.0297
PDE6C	0.8858	1.0614	1.0321	0.9467	0.9815	0.0401
ZSCAN29	0.9364	1.0929	1.0788	0.8179	0.9815	0.0650
UGT1A8	0.9349	1.1012	1.0401	0.8497	0.9815	0.0558
TTC36	0.9675	0.9797	1.0897	0.8892	0.9815	0.0413
P2RX1	0.8519	1.1568	0.9852	0.9321	0.9815	0.0645
MAPK9	0.9311	1.3215	0.9320	0.7414	0.9815	0.1219
LOC729995	0.9893	1.0867	0.9612	0.8889	0.9815	0.0409
LY6G6C	0.9531	1.0430	1.0448	0.8852	0.9815	0.0386
ZNF549	0.6906	1.3552	1.0123	0.8679	0.9815	0.1409
SOAT2	0.8212	1.1800	1.0117	0.9132	0.9815	0.0767
LOC150577	0.9773	1.0358	1.0809	0.8321	0.9815	0.0541
CPZ	0.9194	1.0556	1.0145	0.9366	0.9815	0.0322
PISRT1	0.9574	1.0485	0.9865	0.9337	0.9815	0.0248
SNORA80	0.7509	1.3144	1.0290	0.8319	0.9815	0.1254
OR10R2	0.9767	1.0609	0.9977	0.8908	0.9815	0.0351
LOC387686	0.9103	1.1660	1.0310	0.8189	0.9815	0.0753
LOC1001338	1.0369	1.0415	0.8897	0.9581	0.9816	0.0361
TCL1A	0.9614	1.0125	0.9893	0.9630	0.9816	0.0121
TSSK1B	0.9118	1.0742	1.1004	0.8397	0.9816	0.0631
SRGAP2	0.9359	1.2315	0.8918	0.8671	0.9816	0.0845
LOC647568	0.8236	1.1594	1.0849	0.8583	0.9816	0.0829
LOC647251	1.0135	1.0162	1.1000	0.7967	0.9816	0.0648
FOXG1	0.9392	1.2360	0.8341	0.9171	0.9816	0.0878
CES8	0.9068	0.9975	1.0027	1.0193	0.9816	0.0253
KRT8P9	0.9429	1.1668	1.0414	0.7753	0.9816	0.0826
SAG	0.9803	0.9567	1.0067	0.9828	0.9816	0.0102
ABCA13	0.8862	1.0688	1.0545	0.9169	0.9816	0.0467
LOC728046	0.8569	1.1856	0.9529	0.9310	0.9816	0.0710
NDUFS6	0.9168	1.0802	1.0431	0.8863	0.9816	0.0472
LOC391609	1.0129	1.0421	0.9607	0.9108	0.9816	0.0290
LOC1001282	0.9193	1.1230	0.9965	0.8877	0.9816	0.0524
RCBTB2	0.9658	1.0151	1.0459	0.8996	0.9816	0.0319
LOC645558	1.0137	1.0549	0.9878	0.8701	0.9816	0.0396

NDUFA5	1.0050	1.0410	0.9986	0.8820	0.9816	0.0345
LOC647527	0.9059	1.1663	0.9895	0.8649	0.9816	0.0668
LOC256483	1.0102	1.0439	0.9479	0.9246	0.9816	0.0275
LOC728792	0.9835	1.1078	0.9210	0.9144	0.9816	0.0448
NBPF11	0.8812	1.2042	0.9806	0.8606	0.9816	0.0787
LOC392275	0.9039	1.0346	1.0114	0.9767	0.9816	0.0285
ADIPOQ	0.9562	1.0079	0.9700	0.9925	0.9817	0.0115
LOC651820	0.9714	0.9915	1.0250	0.9388	0.9817	0.0181
FLJ40606	0.8064	1.1033	1.1255	0.8914	0.9817	0.0787
LOC652011	0.9407	1.0468	1.0392	0.9000	0.9817	0.0364
CD7	0.9661	1.0437	1.0245	0.8923	0.9817	0.0340
LOC643948	1.0167	1.0217	0.9784	0.9099	0.9817	0.0258
LOC144817	0.9882	1.0382	0.8734	1.0269	0.9817	0.0376
CXCR6	1.0176	1.0167	0.9177	0.9748	0.9817	0.0235
PCDH1	0.9944	1.1056	1.0059	0.8209	0.9817	0.0591
S100A16	0.8489	1.1175	0.9760	0.9843	0.9817	0.0549
FAM90A6P	0.9522	1.0902	0.9560	0.9284	0.9817	0.0367
HRC	0.8911	1.0962	1.0269	0.9126	0.9817	0.0484
L3MBTL3	0.9050	1.1349	1.0848	0.8021	0.9817	0.0776
LOC732111	0.8907	1.0533	1.0035	0.9794	0.9817	0.0340
C9orf53	0.9449	1.0769	1.0222	0.8828	0.9817	0.0426
LTA	0.7158	1.1820	1.1591	0.8699	0.9817	0.1136
NBPF16	0.9710	0.9960	1.0620	0.8979	0.9817	0.0339
ZNF596	0.9466	0.9891	1.1182	0.8730	0.9817	0.0514
FCHO1	0.8526	1.1647	1.0045	0.9051	0.9817	0.0687
ERBB3	0.8165	1.3828	1.0083	0.7193	0.9817	0.1466
LOC1001338	1.0189	0.9925	0.9859	0.9297	0.9817	0.0188
BTF3L1	1.0312	1.0409	0.9661	0.8887	0.9817	0.0352
VAPB	0.9111	1.0363	1.0240	0.9554	0.9817	0.0295
GALNTL1	0.9143	1.1078	1.0693	0.8355	0.9817	0.0642
OR14A16	1.0092	0.9768	1.0124	0.9285	0.9817	0.0195
LOC727896	0.9554	1.0628	1.0354	0.8734	0.9817	0.0427
LOC649557	0.9748	0.9958	1.0710	0.8855	0.9817	0.0382
ANKMY2	0.9322	1.1776	1.0668	0.7503	0.9817	0.0920
MORC4	0.9878	1.1346	0.9046	0.8999	0.9817	0.0548
MGC52498	0.9237	1.1473	0.9154	0.9406	0.9817	0.0554
LOC1001336	0.9798	1.1523	0.9503	0.8445	0.9817	0.0638
LOC647869	0.9092	1.1677	0.9223	0.9278	0.9817	0.0621
ROR1	1.0002	1.2127	0.9306	0.7835	0.9817	0.0893
LOC650517	0.9074	1.1028	1.0363	0.8805	0.9817	0.0527
HNRNPCL1	0.9646	1.0353	1.0229	0.9042	0.9818	0.0301
ZFC3H1	0.9189	1.1734	1.0067	0.8280	0.9818	0.0736
PTK9	0.8508	1.2442	0.9575	0.8745	0.9818	0.0904
OR6Q1	0.9869	1.0594	1.0190	0.8617	0.9818	0.0427
LOC442474	0.8452	1.1864	0.9897	0.9057	0.9818	0.0744
AKNAD1	0.8758	1.1463	1.0074	0.8976	0.9818	0.0619

TNFRSF11B	0.9098	1.0999	1.0602	0.8571	0.9818	0.0583
FLJ11795	0.9980	1.0302	1.0471	0.8518	0.9818	0.0445
LOC729113	1.0514	0.9140	0.9749	0.9868	0.9818	0.0282
LOC642698	0.8108	1.1614	1.0272	0.9277	0.9818	0.0744
ANXA2P1	0.9268	0.9878	1.0875	0.9250	0.9818	0.0381
LOC644408	0.9948	1.0953	0.9933	0.8438	0.9818	0.0518
ASB12	0.9408	1.0912	0.9461	0.9490	0.9818	0.0365
LOC731852	0.9015	1.1389	0.9386	0.9482	0.9818	0.0533
LOC1001285	0.9255	1.1564	0.9212	0.9240	0.9818	0.0582
ZNF709	0.9081	1.0805	1.0334	0.9052	0.9818	0.0444
LOC643432	0.9243	1.0143	1.0437	0.9449	0.9818	0.0282
SGTB	0.9866	1.0400	1.0729	0.8277	0.9818	0.0544
LOC729195	0.8594	1.0822	0.9786	1.0071	0.9818	0.0463
LOC401640	0.6672	1.1738	1.4419	0.6444	0.9818	0.1961
NKX2-1	0.9794	1.0641	0.9976	0.8863	0.9818	0.0367
CACNA1A	0.8920	1.1046	1.0277	0.9030	0.9818	0.0512
C21orf7	0.9429	1.1371	0.9875	0.8598	0.9818	0.0581
LOC254398	0.9496	1.1137	0.9804	0.8836	0.9818	0.0484
LYPD2	0.9284	1.0408	1.0669	0.8911	0.9818	0.0426
MGC46336	0.9594	1.1401	0.9307	0.8971	0.9818	0.0543
LOC642772	0.8590	1.1514	1.0197	0.8972	0.9818	0.0661
ANXA2P3	0.8312	1.1010	1.0050	0.9901	0.9818	0.0559
LOC654010	0.9808	1.0983	0.9470	0.9013	0.9818	0.0421
SUV420H2	0.9009	1.1277	1.0040	0.8949	0.9818	0.0547
TAS2R31	0.9807	1.0890	1.0039	0.8539	0.9818	0.0486
XAGE-4	0.9384	1.0775	0.9861	0.9255	0.9818	0.0344
LOC646861	0.9573	1.0292	0.9882	0.9527	0.9819	0.0176
LOC729863	0.9309	1.0288	1.0044	0.9633	0.9819	0.0217
LOC647836	1.0133	1.0666	1.0736	0.7739	0.9819	0.0706
SOX14	0.9290	1.0261	1.0493	0.9230	0.9819	0.0326
LOC652690	0.8503	1.0736	1.0433	0.9603	0.9819	0.0500
LOC1001339	0.9713	0.9882	1.0526	0.9154	0.9819	0.0283
LOC1001293	0.6896	1.0496	1.4526	0.7356	0.9819	0.1761
LOC652747	0.8367	1.1370	1.0073	0.9465	0.9819	0.0626
LOC644079	0.8992	1.0953	0.9897	0.9432	0.9819	0.0421
C20orf70	1.0091	1.1047	0.8698	0.9439	0.9819	0.0498
UOX	0.9357	1.0818	1.0542	0.8558	0.9819	0.0526
FLJ31401	0.9349	1.0826	0.9727	0.9374	0.9819	0.0346
DUOXA2	0.8840	1.1039	0.9766	0.9630	0.9819	0.0455
SLC5A11	0.9660	1.0428	1.0644	0.8542	0.9819	0.0475
OLFM3	0.9659	1.0674	0.9071	0.9872	0.9819	0.0332
SNORA21	0.7868	1.2682	0.9487	0.9239	0.9819	0.1019
LYZL1	0.8350	1.0597	1.1043	0.9285	0.9819	0.0616
ATF7IP	0.7926	1.1233	1.0964	0.9153	0.9819	0.0782
LOC652416	0.9475	1.0056	1.0260	0.9486	0.9819	0.0200
LOC643060	0.9664	1.0260	1.0391	0.8962	0.9819	0.0327

CPA4	0.9185	1.0302	0.9859	0.9930	0.9819	0.0233
LOC1001308	0.9506	1.1120	0.9719	0.8931	0.9819	0.0465
ATL3	0.6229	1.2976	1.1868	0.8204	0.9819	0.1572
LOC642127	0.9935	1.0104	1.0115	0.9122	0.9819	0.0236
LCE1A	0.8492	1.1112	1.0340	0.9333	0.9819	0.0573
LOC286177	0.9212	1.0385	1.0789	0.8890	0.9819	0.0456
WNT2B	0.9285	1.1120	1.0081	0.8791	0.9819	0.0508
HES3	0.9693	1.0458	1.0022	0.9104	0.9819	0.0285
TBL1XR1	0.7690	1.5829	0.8801	0.6957	0.9819	0.2039
MIR487B	0.9553	0.9717	1.1087	0.8920	0.9819	0.0456
METTL14	0.8254	1.2688	1.0189	0.8146	0.9819	0.1065
LOC1001294	0.9536	1.0670	0.9873	0.9198	0.9819	0.0315
VTRNA1-2	0.8984	0.9736	1.1623	0.8934	0.9819	0.0629
LOC729950	0.9200	1.0375	1.0992	0.8711	0.9819	0.0524
SCIN	1.0270	1.0809	0.9265	0.8934	0.9820	0.0435
OSM	0.9734	1.0168	0.9433	0.9943	0.9820	0.0156
DST	0.9604	1.1054	0.9846	0.8775	0.9820	0.0471
LCE3A	0.8746	1.0954	1.0729	0.8849	0.9820	0.0592
FRG2	0.9761	1.0910	0.9508	0.9100	0.9820	0.0388
S100A7L2	0.8949	1.0466	1.0442	0.9421	0.9820	0.0379
FLJ35258	0.9258	1.1057	1.0205	0.8758	0.9820	0.0510
LOC645208	0.8744	0.9664	1.0337	1.0534	0.9820	0.0404
FLJ32658	1.0089	0.9926	0.9858	0.9406	0.9820	0.0146
LOC649432	0.8707	1.1324	1.0085	0.9163	0.9820	0.0577
LOC389888	0.9164	1.1433	0.9515	0.9167	0.9820	0.0544
MS4A7	0.9622	1.0521	1.0422	0.8715	0.9820	0.0420
FAM159A	0.8795	1.1470	0.9855	0.9159	0.9820	0.0593
SUCLG2	0.8776	1.1883	1.2028	0.6593	0.9820	0.1311
GOT1L1	0.9016	1.1322	1.0284	0.8657	0.9820	0.0611
KCNJ9	0.9946	1.0715	0.9804	0.8815	0.9820	0.0390
KRT9	0.9486	1.0468	1.0530	0.8795	0.9820	0.0417
ZNF711	0.9680	0.9772	1.0166	0.9661	0.9820	0.0118
HOXC5	0.9340	1.1166	0.8776	0.9998	0.9820	0.0513
KLHL14	0.9600	0.9904	1.0343	0.9433	0.9820	0.0200
LOC647035	1.0053	1.0042	0.9584	0.9601	0.9820	0.0131
FAM151A	0.9624	1.1162	0.9996	0.8498	0.9820	0.0549
KRT17P3	0.9239	1.0914	1.0080	0.9048	0.9820	0.0428
SHE	0.9480	1.0629	0.9460	0.9712	0.9820	0.0275
STON1	0.9197	1.0811	1.0098	0.9176	0.9820	0.0394
CYP7A1	0.8796	1.0812	1.0242	0.9431	0.9820	0.0444
LMX1A	0.9657	1.0164	1.0252	0.9208	0.9820	0.0243
LOC387723	0.9816	0.9429	0.9423	1.0615	0.9820	0.0280
LOC729433	0.9584	1.0249	1.0865	0.8583	0.9820	0.0489
LOC387693	1.0220	1.0728	0.9605	0.8729	0.9820	0.0430
BICD1	0.9184	1.1590	1.0449	0.8058	0.9820	0.0766
ZNF14	0.8785	1.2669	1.0772	0.7056	0.9820	0.1216

LOC646931	0.8833	1.0263	1.0417	0.9769	0.9821	0.0357
MALAT1	0.9119	1.1064	0.9844	0.9254	0.9821	0.0443
KIAA1571	0.7662	1.2572	1.0357	0.8691	0.9821	0.1072
PRKG2	1.0009	1.0087	0.9667	0.9520	0.9821	0.0135
LOC730811	0.9298	1.0469	1.1231	0.8284	0.9821	0.0648
NECAP2	0.9827	1.2406	0.8383	0.8667	0.9821	0.0917
LOC729278	0.9523	0.9468	0.9723	1.0569	0.9821	0.0255
GZMK	0.7940	1.0763	1.1039	0.9540	0.9821	0.0706
MIR1284	0.9298	1.0432	0.9827	0.9726	0.9821	0.0234
LOC390259	0.8880	1.1101	0.9806	0.9497	0.9821	0.0468
LOC647006	0.8892	1.1234	0.9122	1.0036	0.9821	0.0532
CCDC150	0.9952	1.0082	0.9596	0.9655	0.9821	0.0117
PLAC1L	0.9606	1.0635	1.0551	0.8492	0.9821	0.0501
DIDO1	0.9075	1.1428	0.9427	0.9353	0.9821	0.0541
UGT2A1	0.8653	1.1416	1.0245	0.8971	0.9821	0.0633
PLP1	0.8727	1.1597	1.0272	0.8688	0.9821	0.0698
TP53AIP1	0.9516	1.0015	1.0231	0.9523	0.9821	0.0180
LOC1001299	0.8883	1.1305	1.0969	0.8128	0.9821	0.0778
LOC650212	0.9345	1.0222	1.0269	0.9449	0.9821	0.0246
LOC643718	0.9014	1.1286	1.0129	0.8856	0.9822	0.0564
PTPRC	0.9856	1.0649	0.9762	0.9019	0.9822	0.0334
GDNF	0.9291	1.0300	1.1021	0.8675	0.9822	0.0522
LOC652499	0.9302	0.9599	1.0692	0.9694	0.9822	0.0302
CARD16	0.9204	1.0644	1.0414	0.9025	0.9822	0.0413
LDLRAD2	0.8629	1.1109	1.0135	0.9413	0.9822	0.0528
ZNF334	0.9831	1.1297	0.9852	0.8307	0.9822	0.0610
LOC729374	0.8489	1.1672	0.9667	0.9459	0.9822	0.0668
DOK7	0.9900	0.9599	1.0126	0.9662	0.9822	0.0120
LOC728956	0.8800	1.0918	1.0170	0.9399	0.9822	0.0461
LOC1001340	0.9173	1.0367	0.9924	0.9822	0.9822	0.0246
CCDC27	0.8663	1.2325	0.9014	0.9286	0.9822	0.0844
C20orf62	0.8838	1.0982	1.0330	0.9137	0.9822	0.0503
RPS24	0.8042	1.2813	1.0203	0.8230	0.9822	0.1110
TMEM132C	0.9912	1.1034	0.9444	0.8898	0.9822	0.0454
MIR142	0.9547	1.1355	0.9007	0.9379	0.9822	0.0523
GGT5	0.8935	1.1043	1.0311	0.8999	0.9822	0.0516
LOC728081	0.9063	1.1201	1.0498	0.8527	0.9822	0.0620
KIAA0040	0.9592	1.0480	0.9864	0.9352	0.9822	0.0243
LOC642244	0.8481	1.1435	0.9796	0.9576	0.9822	0.0610
GH1	0.9501	1.1185	0.9636	0.8966	0.9822	0.0477
LDHAL6B	0.9162	1.0380	1.0601	0.9146	0.9822	0.0388
TREM1	0.9988	0.9922	0.9944	0.9435	0.9822	0.0130
ZDBF2	0.9356	1.0423	1.0237	0.9273	0.9822	0.0296
LOC652453	0.9799	1.0941	1.0346	0.8204	0.9822	0.0588
LOC646214	0.7738	1.2391	1.3013	0.6147	0.9822	0.1699
ERCC-00084	0.8938	1.0498	1.0677	0.9176	0.9822	0.0446

PCDHGB5	0.9863	1.0409	0.9314	0.9704	0.9822	0.0227
LOC1001311	1.0141	0.9951	1.0052	0.9145	0.9822	0.0229
SNORD115-4	0.9747	1.1245	0.9223	0.9074	0.9822	0.0496
H6PD	0.9747	1.0758	1.0231	0.8555	0.9823	0.0470
SKIL	0.9730	1.0382	1.0544	0.8635	0.9823	0.0433
LOC730110	0.9597	1.0009	1.0481	0.9204	0.9823	0.0274
LOC650628	0.8762	1.1412	0.9734	0.9383	0.9823	0.0567
C19orf22	0.9179	1.2374	0.9941	0.7797	0.9823	0.0959
LOC728611	0.9020	1.1318	1.0211	0.8742	0.9823	0.0592
SULT2B1	0.9506	1.1648	0.9387	0.8749	0.9823	0.0631
SMC1B	0.9507	0.9031	1.1376	0.9378	0.9823	0.0528
SDR42E1	0.8659	1.0704	1.0642	0.9286	0.9823	0.0507
ZNF843	0.8876	1.0681	0.9961	0.9773	0.9823	0.0371
CLIP2	1.0178	1.0418	0.9658	0.9037	0.9823	0.0306
SERPINB13	0.9717	1.0476	0.9544	0.9555	0.9823	0.0221
MRC1	0.9903	1.0994	1.0281	0.8115	0.9823	0.0613
PRO0628	0.9965	1.0490	0.9816	0.9021	0.9823	0.0304
IFNA21	0.9484	1.0716	0.9890	0.9203	0.9823	0.0329
PCDHA1	0.9525	1.0245	1.0586	0.8937	0.9823	0.0369
ELK3	0.9467	1.1128	0.9941	0.8756	0.9823	0.0499
MMS19L	0.8338	1.4809	0.8817	0.7330	0.9823	0.1690
ADRA2C	0.9392	1.0953	0.9757	0.9191	0.9823	0.0395
LOC653741	0.9478	1.0195	1.0577	0.9043	0.9823	0.0346
BTBD9	0.9490	1.1236	0.9695	0.8873	0.9823	0.0502
LOC642539	0.9467	1.0479	1.0632	0.8716	0.9823	0.0451
TMEM53	0.8085	1.3739	1.0443	0.7027	0.9823	0.1488
MIR1470	1.0108	0.9966	0.9901	0.9319	0.9823	0.0173
FLJ14082	0.9773	1.1770	0.9810	0.7941	0.9824	0.0782
TNC	0.9193	1.2645	1.0086	0.7370	0.9824	0.1097
LOC388210	0.8617	1.1145	1.1262	0.8269	0.9824	0.0800
IRF2BP1	0.8876	1.1355	1.0958	0.8106	0.9824	0.0790
CILP	0.9969	1.0728	0.9943	0.8654	0.9824	0.0430
NLRC5	0.8999	1.1123	1.0109	0.9063	0.9824	0.0502
LOC728449	0.9950	1.0663	1.0575	0.8107	0.9824	0.0594
LOC151457	0.9263	1.0916	1.0434	0.8681	0.9824	0.0515
CTGLF6	0.9542	1.1059	0.9699	0.8994	0.9824	0.0439
LOC647625	0.9267	1.0858	0.9934	0.9235	0.9824	0.0381
RIMBP2	0.9413	1.0772	0.9612	0.9498	0.9824	0.0319
DAZ2	0.9262	1.0884	1.0405	0.8744	0.9824	0.0495
LOC729254	0.8698	1.0883	1.0496	0.9218	0.9824	0.0517
C20orf38	0.9100	1.0867	1.0062	0.9265	0.9824	0.0406
FLJ42418	1.0055	1.1105	0.9880	0.8255	0.9824	0.0589
ERCC-00078	1.0330	1.0868	0.9494	0.8603	0.9824	0.0495
LOC730196	0.9312	0.9915	1.0832	0.9236	0.9824	0.0369
HCN2	0.9110	1.0540	1.0044	0.9602	0.9824	0.0306
LOC643687	0.8438	1.1613	1.0063	0.9182	0.9824	0.0682

LBP	0.8647	1.1468	0.9450	0.9731	0.9824	0.0594
OR51B5	0.9156	1.1099	0.9588	0.9452	0.9824	0.0435
ASB10	0.8949	1.0101	1.0331	0.9915	0.9824	0.0304
NCRNA0009?	0.8768	1.0864	1.1548	0.8115	0.9824	0.0821
CD1C	0.9484	0.9826	1.1352	0.8634	0.9824	0.0568
CCT8L2	0.9860	0.9831	1.0030	0.9576	0.9824	0.0094
LOC731283	0.9388	1.0153	1.0790	0.8966	0.9824	0.0405
PCDP1	0.8713	1.1301	1.0025	0.9257	0.9824	0.0561
LOC284890	0.9054	1.0822	1.0136	0.9284	0.9824	0.0406
OTUD5	0.7698	1.1902	1.0232	0.9465	0.9824	0.0872
LOC1001320	0.9896	1.0388	1.0895	0.8117	0.9824	0.0605
PTPRO	0.8954	1.1137	1.0085	0.9121	0.9824	0.0504
LRRC16A	0.9481	1.1196	1.0589	0.8031	0.9824	0.0695
SSX2	0.9686	1.0680	1.0003	0.8928	0.9824	0.0364
PBX1	0.8950	1.3011	0.9802	0.7534	0.9824	0.1161
KRTAP27-1	0.9451	1.0710	1.0385	0.8751	0.9824	0.0446
PMS2L4	0.7357	1.4411	0.9640	0.7889	0.9824	0.1605
C9orf122	0.9067	1.0917	1.0800	0.8513	0.9824	0.0608
FLJ32784	0.9899	1.0629	1.0167	0.8601	0.9824	0.0435
FLJ90231	0.9303	1.0145	1.0441	0.9408	0.9824	0.0278
LOC653512	0.9748	1.0099	1.0422	0.9029	0.9824	0.0299
LOC732107	0.9925	1.0354	0.9801	0.9217	0.9824	0.0235
LOC1001278	0.9169	1.2118	0.9278	0.8732	0.9824	0.0774
LOC642707	0.9084	1.0796	1.0115	0.9304	0.9824	0.0392
LOC645661	0.9436	1.0131	1.0635	0.9097	0.9825	0.0345
SNORD113-4	0.9865	1.1827	0.9005	0.8602	0.9825	0.0718
LOC728494	0.9095	1.1061	1.0579	0.8564	0.9825	0.0593
LOC1001285	0.9849	0.9842	1.0142	0.9465	0.9825	0.0139
ATP8A2	0.9702	1.0620	0.9939	0.9038	0.9825	0.0326
C7orf57	1.0015	0.9923	1.0360	0.9001	0.9825	0.0290
LOC653086	0.9569	1.1335	1.0241	0.8155	0.9825	0.0665
SFN	0.9204	1.0191	1.0773	0.9131	0.9825	0.0398
TBC1D3F	0.8423	1.2285	1.0213	0.8379	0.9825	0.0925
PLCB1	1.0265	1.0863	1.0179	0.7993	0.9825	0.0629
C6orf118	0.9327	1.0638	1.0727	0.8607	0.9825	0.0517
PAX6	0.9859	1.0214	1.0111	0.9116	0.9825	0.0248
XKR5	0.7868	1.0425	1.1488	0.9519	0.9825	0.0766
ARID4A	0.9027	1.1705	1.0511	0.8056	0.9825	0.0805
ERCC-00109	0.8644	1.0096	1.0839	0.9721	0.9825	0.0457
LOC1001318	0.9187	1.0873	0.9988	0.9251	0.9825	0.0394
LOC728692	0.8965	1.0626	1.0259	0.9450	0.9825	0.0378
LOC649406	1.0220	0.9184	0.9976	0.9920	0.9825	0.0224
MIR489	1.0052	1.0201	1.0176	0.8871	0.9825	0.0320
PRB1	0.9212	1.0399	1.0279	0.9411	0.9825	0.0300
STK35	0.9019	1.4048	0.9076	0.7157	0.9825	0.1477
LOC1001299	0.9396	1.0576	0.9897	0.9431	0.9825	0.0275

LOC284837	1.0942	1.0267	0.8695	0.9397	0.9825	0.0492
LOC1001287	0.8746	0.9103	1.0737	1.0715	0.9825	0.0525
LOC645529	0.8607	1.0794	1.0381	0.9520	0.9826	0.0485
RPL30	0.9164	1.1326	1.0468	0.8345	0.9826	0.0664
LOC728380	1.0617	0.9214	0.9631	0.9840	0.9826	0.0294
LOC732424	1.0074	1.0708	0.9441	0.9079	0.9826	0.0359
CD52	1.0255	1.1695	0.8978	0.8375	0.9826	0.0736
LOC727922	0.8574	1.0959	1.0870	0.8900	0.9826	0.0632
IER3IP1	0.9182	1.0400	1.2182	0.7539	0.9826	0.0980
ERCC-00097	0.8920	1.1898	0.9248	0.9238	0.9826	0.0695
MGC62100	0.9126	1.1170	1.0580	0.8427	0.9826	0.0634
LOC645231	0.9253	1.0422	1.0530	0.9098	0.9826	0.0377
BAIAP3	0.9634	1.0023	1.0629	0.9017	0.9826	0.0339
CERKL	0.9705	1.0425	0.9831	0.9344	0.9826	0.0225
HIST1H2BN	0.9257	1.0908	1.0136	0.9004	0.9826	0.0435
LOC388910	0.9839	1.0811	0.9711	0.8943	0.9826	0.0383
C1orf185	0.9758	1.0148	0.9224	1.0174	0.9826	0.0222
LOC652512	1.0119	0.9950	0.9661	0.9574	0.9826	0.0126
LOC642452	0.8789	1.1245	0.9905	0.9365	0.9826	0.0525
RNU4-1	0.8213	1.1876	1.0132	0.9084	0.9826	0.0788
MIOX	0.9479	0.9618	1.0363	0.9845	0.9826	0.0194
SAMHD1	0.8272	1.0522	1.1395	0.9117	0.9826	0.0699
GRIA1	1.0806	1.0277	0.9492	0.8730	0.9826	0.0454
LOC651345	0.9977	1.0561	0.9827	0.8940	0.9826	0.0335
CKM	0.8879	1.0844	0.9932	0.9651	0.9826	0.0406
LOC1001345	1.0004	1.0798	1.0751	0.7753	0.9826	0.0715
BTBD8	0.9259	1.0814	1.0121	0.9112	0.9827	0.0397
DSCAML1	0.8963	1.1256	0.9648	0.9439	0.9827	0.0498
MIR549	0.9189	1.0426	0.9927	0.9764	0.9827	0.0255
MIR568	0.9546	1.1466	0.8511	0.9783	0.9827	0.0612
ARL10	0.9739	1.0786	0.9213	0.9568	0.9827	0.0338
LOC652804	1.0186	1.0557	1.0211	0.8354	0.9827	0.0498
CACNA1D	0.8024	1.1680	1.0833	0.8769	0.9827	0.0857
ACACB	1.0010	1.2480	0.8326	0.8490	0.9827	0.0962
GATA3	0.9406	1.0933	1.0475	0.8492	0.9827	0.0548
HIVEP3	0.8242	1.0832	1.0782	0.9451	0.9827	0.0618
LOC1001305	0.9731	1.0176	1.0980	0.8420	0.9827	0.0535
CLTB	0.8794	1.2182	1.0251	0.8081	0.9827	0.0906
LOC643222	0.8614	1.0914	1.0432	0.9348	0.9827	0.0520
LOC440804	0.9573	1.1119	0.9752	0.8865	0.9827	0.0471
SH2D5	0.8725	1.1144	0.9894	0.9546	0.9827	0.0503
HSPA2	0.9226	1.0934	0.9991	0.9158	0.9827	0.0414
MOXD1	0.9641	1.0309	1.0050	0.9309	0.9827	0.0221
LOC400301	1.0224	1.0483	0.9922	0.8679	0.9827	0.0399
LOC1001287	0.9217	1.0991	0.9576	0.9525	0.9827	0.0396
LOC653887	0.8691	1.1570	1.0302	0.8746	0.9827	0.0691

OLFML2B	0.9441	1.0666	0.9622	0.9580	0.9827	0.0282
LOC401525	0.8969	1.1565	0.9734	0.9042	0.9827	0.0604
SNORD21	0.8753	1.0933	1.1077	0.8546	0.9827	0.0682
SPNS2	0.8495	1.1946	0.9639	0.9229	0.9827	0.0745
LOC1001310	0.9177	1.1119	1.0504	0.8510	0.9828	0.0598
LOC401720	0.9253	1.2003	0.8836	0.9217	0.9828	0.0731
LOC402360	0.8545	1.0876	1.0431	0.9458	0.9828	0.0520
LOC648823	0.8575	1.0927	1.0335	0.9473	0.9828	0.0513
FLRT3	0.9468	1.0604	0.9973	0.9265	0.9828	0.0299
USP28	0.9528	1.0357	1.0153	0.9273	0.9828	0.0255
ORAI2	0.8861	1.1975	0.9525	0.8949	0.9828	0.0731
LOC440910	0.9652	1.0451	0.9805	0.9403	0.9828	0.0224
PRDM9	0.8750	1.1791	0.9533	0.9237	0.9828	0.0674
LOC1001308	0.9770	1.1091	0.9613	0.8837	0.9828	0.0468
TNF	0.9231	1.0866	1.0594	0.8619	0.9828	0.0539
XAGE1C	0.9505	1.0690	1.1621	0.7495	0.9828	0.0890
SPO11	0.9033	1.0932	0.9710	0.9636	0.9828	0.0398
SEMA4C	0.9334	1.1244	0.9702	0.9032	0.9828	0.0492
CCDC134	0.8402	1.1826	1.0479	0.8605	0.9828	0.0814
LOC649313	0.8897	1.0847	0.9934	0.9633	0.9828	0.0404
LOC728005	0.9288	1.0067	1.0666	0.9291	0.9828	0.0334
MIR339	1.0076	1.0179	1.0239	0.8818	0.9828	0.0338
FAM159B	0.9417	1.0414	1.0986	0.8496	0.9828	0.0550
ABCB4	0.8720	1.0654	1.0373	0.9566	0.9828	0.0435
LOC152742	0.9708	1.1027	1.0078	0.8500	0.9828	0.0523
OBSL1	0.9036	0.9492	1.0065	1.0720	0.9828	0.0364
MIR668	0.9565	1.1519	1.0179	0.8050	0.9828	0.0719
PRR20D	0.9869	1.0729	0.9282	0.9433	0.9828	0.0325
TXNRD3IT1	0.8938	1.1350	0.9968	0.9057	0.9828	0.0557
ZNF330	0.7154	1.3925	1.0799	0.7436	0.9828	0.1597
FOXI3	0.8709	1.1441	1.0018	0.9146	0.9828	0.0602
LOC730908	0.9168	1.1039	1.0832	0.8276	0.9828	0.0666
HSFYP1	0.8804	1.0581	1.0751	0.9178	0.9829	0.0491
TP53I11	1.0019	1.0851	1.0883	0.7562	0.9829	0.0782
GAS2L1	0.9224	1.1280	1.0267	0.8543	0.9829	0.0600
LOC647592	0.9827	1.0284	1.0115	0.9088	0.9829	0.0264
SPATS1	0.9774	1.0067	1.0259	0.9215	0.9829	0.0228
FLJ14213	0.8888	1.1228	0.9852	0.9346	0.9829	0.0506
CCDC52	0.8792	1.1369	1.0039	0.9115	0.9829	0.0577
LOC650942	1.0060	0.9978	1.0022	0.9255	0.9829	0.0192
LOC652652	0.9550	1.0503	1.0416	0.8846	0.9829	0.0392
LOC1001333	0.9161	1.1377	0.9889	0.8889	0.9829	0.0558
CCDC152	0.9684	1.0062	1.0575	0.8995	0.9829	0.0333
LOC1001346	0.9383	0.9972	1.1123	0.8839	0.9829	0.0489
KIR3DL2	0.9572	1.0385	1.0760	0.8600	0.9829	0.0479
SNORD53	0.9784	1.0363	1.0028	0.9141	0.9829	0.0258

RHPN2	0.9024	1.4658	0.9856	0.5778	0.9829	0.1834
RORA	0.9702	1.0424	1.0365	0.8826	0.9829	0.0372
PRAMEF21	0.9817	1.1163	0.9929	0.8407	0.9829	0.0564
LOC1001316	0.9091	1.0434	0.9989	0.9803	0.9829	0.0279
GPR173	0.9223	1.0407	1.0614	0.9073	0.9829	0.0397
STAC2	1.0755	1.0069	0.9633	0.8861	0.9829	0.0397
LOC1001279	0.9038	1.0807	1.0570	0.8904	0.9830	0.0499
DEFB134	0.9921	1.1169	1.0311	0.7917	0.9830	0.0689
KLF1	0.8822	1.1961	0.9591	0.8945	0.9830	0.0730
LOC645268	0.9788	1.0751	1.0484	0.8296	0.9830	0.0550
LOC440563	0.9936	0.9354	1.0111	0.9917	0.9830	0.0164
LOC648456	0.9171	1.0997	0.9917	0.9235	0.9830	0.0424
POU5F1P1	1.0111	0.9971	1.0461	0.8777	0.9830	0.0366
ERCC-00148	0.9148	1.0193	1.0153	0.9825	0.9830	0.0242
FOXI1	0.9271	1.0690	0.9916	0.9443	0.9830	0.0318
MIR1979	0.9151	1.0486	1.0195	0.9487	0.9830	0.0309
LOC650238	0.7157	1.1911	0.9643	1.0608	0.9830	0.1005
PRPF31	0.9119	1.2582	1.0202	0.7417	0.9830	0.1082
ZNF114	0.9287	1.1672	1.0144	0.8217	0.9830	0.0730
LOC650239	0.9798	1.0632	0.9802	0.9089	0.9830	0.0315
HUS1B	0.9458	1.1430	0.9074	0.9359	0.9830	0.0539
LOC116349	0.9103	1.1584	1.0231	0.8403	0.9830	0.0695
LOC1001345	0.8766	1.0551	1.0958	0.9047	0.9830	0.0543
STELLAR	0.9733	1.0634	1.0050	0.8905	0.9830	0.0361
GIYD2	0.8135	1.1460	1.0237	0.9490	0.9831	0.0696
PTX3	0.9255	1.0775	1.0009	0.9283	0.9831	0.0360
LOC646190	0.8467	1.0441	1.0556	0.9860	0.9831	0.0480
KDSR	0.9757	1.1966	0.8620	0.8979	0.9831	0.0750
LOC1001293	1.0125	0.9891	1.0147	0.9160	0.9831	0.0231
LOC1001295	0.9069	1.0983	0.9909	0.9363	0.9831	0.0422
BIRC1	0.9336	1.1317	0.9676	0.8996	0.9831	0.0514
TRR1	0.9269	1.1278	0.9966	0.8811	0.9831	0.0537
C17orf82	0.9508	1.1598	1.0264	0.7954	0.9831	0.0760
POSTN	0.9423	1.0565	0.9829	0.9507	0.9831	0.0260
NR1I2	0.9421	1.0405	1.0661	0.8837	0.9831	0.0426
LOC643342	0.9227	1.1205	1.0216	0.8676	0.9831	0.0558
MIR376C	0.9157	1.1009	0.9532	0.9627	0.9831	0.0406
LOC390367	0.8881	1.0781	1.0279	0.9384	0.9831	0.0429
CLDN12	0.8075	1.3572	1.0176	0.7501	0.9831	0.1373
LOC1001338	0.9421	1.1628	0.9160	0.9116	0.9831	0.0603
CLEC2A	1.0074	1.0124	1.0753	0.8374	0.9831	0.0510
MRGPRG	0.8418	1.1165	1.0282	0.9461	0.9831	0.0586
C3orf72	0.9597	1.0466	0.9760	0.9502	0.9831	0.0218
LOC642210	0.9500	1.0642	0.9876	0.9308	0.9831	0.0295
PRR17	0.9612	1.1701	0.9094	0.8918	0.9831	0.0640
SNORA32	0.9747	1.1318	0.9428	0.8833	0.9831	0.0531

ADCY4	0.9069	1.1200	0.9480	0.9577	0.9831	0.0469
CCL7	0.9214	1.0447	1.0787	0.8877	0.9832	0.0464
LOC642446	0.9073	1.1335	1.0100	0.8818	0.9832	0.0573
PCDHA13	0.9618	1.0122	1.0059	0.9527	0.9832	0.0151
SLC17A5	0.8535	1.0902	1.0526	0.9363	0.9832	0.0542
LOC391359	0.9236	0.9825	1.1135	0.9131	0.9832	0.0461
SLC7A9	0.8940	1.1275	1.0161	0.8951	0.9832	0.0560
LOC1001302	0.8814	1.1418	1.0184	0.8911	0.9832	0.0614
STEAP4	0.9550	1.0832	1.0116	0.8828	0.9832	0.0425
GDF7	0.9307	1.0682	1.0577	0.8762	0.9832	0.0474
LOC652898	1.0407	1.0753	0.9886	0.8281	0.9832	0.0547
GGTL3	0.9762	1.0903	0.9502	0.9161	0.9832	0.0378
RPL3	0.9039	1.2224	1.0854	0.7210	0.9832	0.1090
MIR555	0.8840	1.0854	0.9640	0.9995	0.9832	0.0418
C10orf53	0.9077	1.0622	1.0356	0.9273	0.9832	0.0386
LOC729198	0.9187	1.1180	0.9771	0.9190	0.9832	0.0470
LOC1001299	0.9530	1.0166	1.0215	0.9418	0.9832	0.0208
PRODH	0.9670	0.9721	1.0802	0.9136	0.9832	0.0349
ROBO4	0.9618	1.0401	1.0309	0.9001	0.9832	0.0327
CA8	0.9583	1.0628	0.9880	0.9238	0.9832	0.0296
LYRM5	0.9325	1.0289	1.2596	0.7119	0.9832	0.1135
MRPL49	0.9697	1.1567	1.0575	0.7490	0.9832	0.0869
C22orf31	1.0479	1.0619	0.9601	0.8629	0.9832	0.0460
RPS11	0.9294	1.0448	1.0862	0.8725	0.9832	0.0496
PSMD9	0.9960	1.0135	1.0559	0.8675	0.9832	0.0406
XKR7	0.9383	0.9953	1.0877	0.9115	0.9832	0.0390
KLHL31	0.9697	1.0489	0.9248	0.9894	0.9832	0.0257
KLRB1	0.8798	1.0761	1.0264	0.9506	0.9832	0.0431
LOC131055	0.9853	0.9548	1.1240	0.8689	0.9832	0.0530
LOC646585	0.8297	1.2662	1.0329	0.8042	0.9832	0.1073
LOC1001282	0.7992	1.0672	1.2880	0.7785	0.9832	0.1210
ARMCX2	0.9648	0.9451	1.0547	0.9684	0.9832	0.0244
LOC642480	0.9857	0.9533	1.0593	0.9346	0.9832	0.0275
LOC650488	0.9295	1.0568	1.0856	0.8610	0.9832	0.0530
PON1	0.9417	1.1061	0.9215	0.9637	0.9832	0.0419
KRT71	0.9722	1.0136	0.9714	0.9757	0.9832	0.0102
LOC653437	1.0896	1.0388	0.9787	0.8259	0.9833	0.0571
DKFZP434L18	0.9533	1.1418	0.9644	0.8736	0.9833	0.0566
LOC651453	0.9209	1.1533	1.1727	0.6861	0.9833	0.1144
LOC1001326	0.9636	1.0423	1.0767	0.8504	0.9833	0.0502
LOC439994	0.8725	1.1026	1.1324	0.8256	0.9833	0.0783
PRAF2	0.8518	1.2175	1.0455	0.8183	0.9833	0.0928
PCDHA3	0.9456	1.0812	0.9980	0.9083	0.9833	0.0375
LOC1001307	0.9897	1.0161	1.0343	0.8930	0.9833	0.0314
FLJ20209	0.8415	1.0286	1.1488	0.9143	0.9833	0.0673
MFAP4	1.0450	1.0247	1.0382	0.8252	0.9833	0.0529

LOC440491	0.9521	1.1031	0.9569	0.9210	0.9833	0.0407
CISH	0.9347	1.0947	1.0019	0.9019	0.9833	0.0426
LOC1001282	0.8748	1.0707	0.9862	1.0015	0.9833	0.0406
FAM153A	0.8949	1.0990	1.0706	0.8686	0.9833	0.0591
SEC61G	0.8993	1.2045	1.0638	0.7654	0.9833	0.0957
CCNJL	0.8868	1.0701	1.0565	0.9196	0.9833	0.0468
C19orf59	0.9120	1.1647	1.0195	0.8370	0.9833	0.0711
LOC650429	0.8978	1.0283	1.0544	0.9526	0.9833	0.0358
NEU4	0.9689	1.0705	1.0002	0.8936	0.9833	0.0367
RSPH3	0.9417	1.1564	1.0727	0.7623	0.9833	0.0859
TNNC2	0.9543	1.0166	1.0678	0.8945	0.9833	0.0376
ANO4	0.9483	1.0640	1.0061	0.9149	0.9833	0.0328
XCR1	0.8403	1.0288	1.1027	0.9614	0.9833	0.0557
LOC649817	0.8858	1.1132	1.0818	0.8525	0.9833	0.0666
PTPN14	1.0204	1.0595	0.9518	0.9017	0.9833	0.0352
TMEM87A	0.7735	1.3648	1.0851	0.7099	0.9833	0.1513
LOC441268	0.9531	1.1654	0.9548	0.8600	0.9833	0.0646
LOC1001299	0.9280	1.1454	1.0708	0.7891	0.9833	0.0789
HSPA8	0.8194	1.2923	1.0820	0.7397	0.9833	0.1263
BLK	0.9184	1.0589	1.0586	0.8975	0.9833	0.0437
FLJ44186	0.8864	1.0399	1.0876	0.9195	0.9834	0.0479
KLKBL4	0.8695	1.0312	1.0623	0.9705	0.9834	0.0425
LOC402617	0.8615	1.1438	0.9581	0.9701	0.9834	0.0587
C3orf47	0.8806	1.1362	0.9831	0.9335	0.9834	0.0551
KCNE2	0.9759	1.0896	0.9602	0.9078	0.9834	0.0383
LOC642799	0.9608	1.0608	1.0215	0.8904	0.9834	0.0372
BRS3	0.8463	1.0495	1.0929	0.9448	0.9834	0.0553
LOC644806	0.9052	1.0545	1.0145	0.9593	0.9834	0.0326
FLJ35424	0.9331	1.0052	1.0327	0.9626	0.9834	0.0221
ST6GALNAC2	0.9647	1.1768	1.0279	0.7641	0.9834	0.0855
LOC729003	0.8851	1.0842	1.0997	0.8646	0.9834	0.0629
LOC650849	0.8781	1.0541	1.0561	0.9452	0.9834	0.0436
SMC5	0.8447	1.1015	1.0786	0.9088	0.9834	0.0631
P2RY10	0.8875	1.0787	0.9990	0.9683	0.9834	0.0395
FBXO25	0.9490	1.0794	1.0620	0.8431	0.9834	0.0550
MST1R	0.9131	1.0802	1.0115	0.9288	0.9834	0.0388
MRGPRD	0.9708	1.0474	0.9996	0.9158	0.9834	0.0275
LOC646156	0.8245	1.0497	1.0576	1.0019	0.9834	0.0544
OTOS	0.9971	1.0809	0.9534	0.9022	0.9834	0.0379
CXCL5	0.9313	1.1107	1.0351	0.8566	0.9834	0.0560
C11orf39	1.0208	1.0432	1.0137	0.8560	0.9834	0.0429
KCNJ4	0.8967	1.1051	1.1046	0.8275	0.9834	0.0715
LOC729191	0.9103	1.1234	1.0149	0.8852	0.9834	0.0545
IQUB	0.9056	1.1895	0.8750	0.9636	0.9834	0.0711
LOC642423	0.9049	1.1772	0.9217	0.9300	0.9834	0.0648
MURC	0.9537	1.0296	0.9910	0.9594	0.9834	0.0174

LOC643396	0.9142	0.9988	1.0989	0.9218	0.9834	0.0430
ALDH3A1	0.9718	1.1248	1.0114	0.8258	0.9834	0.0617
LOC648237	0.9594	1.0292	1.0410	0.9041	0.9834	0.0320
NEGR1	0.9785	1.0744	1.0277	0.8532	0.9834	0.0476
LOC645033	0.9319	1.0059	1.0736	0.9225	0.9834	0.0354
CLMN	0.9295	1.1653	0.9565	0.8824	0.9835	0.0625
FLJ40194	0.8949	1.2262	0.9683	0.8443	0.9835	0.0848
PSMD8	0.8039	1.4745	0.9920	0.6635	0.9835	0.1770
LOC652148	0.9964	1.1084	0.9792	0.8499	0.9835	0.0530
PNPLA1	1.0304	1.0043	1.0551	0.8441	0.9835	0.0476
RPL27A	0.9085	1.0983	1.0243	0.9026	0.9835	0.0474
OR5M11	0.9620	1.0423	1.0371	0.8924	0.9835	0.0355
LOC440993	0.9448	1.1112	0.9679	0.9100	0.9835	0.0442
LOC440386	0.9905	1.1259	0.9250	0.8926	0.9835	0.0516
OR51G1	0.9382	1.0934	0.9902	0.9120	0.9835	0.0401
EIF4E3	0.8686	1.2639	1.0597	0.7417	0.9835	0.1141
USP20	0.9805	1.0095	1.0593	0.8846	0.9835	0.0368
LOC729591	0.9277	1.1189	0.9750	0.9123	0.9835	0.0471
LOC648223	0.8830	1.1761	0.9623	0.9126	0.9835	0.0662
HOXD13	0.9385	0.9961	0.9812	1.0182	0.9835	0.0168
RTBDN	0.9810	1.0094	1.0104	0.9332	0.9835	0.0181
PEX6	1.0112	1.0480	0.9908	0.8840	0.9835	0.0352
PSEN1	1.0266	1.1135	1.0181	0.7757	0.9835	0.0725
TMEM159	0.9374	1.2122	1.0119	0.7726	0.9835	0.0912
SHD	0.9272	1.0473	0.9958	0.9638	0.9835	0.0255
CHL1	0.9531	1.0850	0.9724	0.9236	0.9835	0.0353
KIF26A	0.8479	1.3114	0.9694	0.8054	0.9835	0.1147
TIPARP	0.8482	1.3143	0.9226	0.8490	0.9835	0.1116
RRAD	0.9670	1.0252	1.0087	0.9332	0.9835	0.0208
OR8H1	0.9162	1.0638	1.0491	0.9049	0.9835	0.0423
ZNF641	0.9477	1.1227	1.0481	0.8155	0.9835	0.0665
LOC729534	0.9411	1.1121	1.0301	0.8507	0.9835	0.0564
LOC1001330	0.8647	0.8059	1.1852	1.0783	0.9835	0.0891
LOC442546	0.9833	1.1772	1.0509	0.7228	0.9835	0.0958
TSSK2	1.0262	1.0222	0.9597	0.9261	0.9835	0.0245
LOC728516	1.0089	0.9919	1.1020	0.8313	0.9835	0.0562
LOC649422	0.9387	1.0896	1.0467	0.8591	0.9835	0.0522
LOC1001291	0.8628	1.0343	1.0937	0.9433	0.9835	0.0507
DEFA1B	0.9639	1.0579	1.0530	0.8594	0.9835	0.0467
LOC1001325	0.8771	1.0052	1.0634	0.9885	0.9835	0.0390
HFE2	0.9117	1.0750	1.0189	0.9286	0.9835	0.0385
LOC646905	0.9270	1.0562	1.0431	0.9079	0.9835	0.0384
SLC44A5	0.9148	1.1434	1.0519	0.8241	0.9835	0.0709
LOC1001299	1.0056	0.9948	1.0144	0.9193	0.9836	0.0218
LOC1001319	1.0103	1.0351	1.0311	0.8577	0.9836	0.0423
C11orf60	0.9310	1.2662	0.9215	0.8154	0.9836	0.0978

LOC728544	0.9518	0.9424	1.1411	0.8989	0.9836	0.0538
EYA1	0.9143	1.0489	0.9929	0.9782	0.9836	0.0277
SPARCL1	0.9511	1.0752	1.0040	0.9039	0.9836	0.0368
LOC645448	0.9663	1.1027	0.9926	0.8726	0.9836	0.0473
C5orf42	0.9053	1.0722	1.0173	0.9395	0.9836	0.0377
OR11H12	0.9123	1.0908	1.0447	0.8865	0.9836	0.0498
NAALADL1	0.9929	1.0482	0.9959	0.8973	0.9836	0.0314
LRRC37B	0.9310	1.1044	1.0358	0.8632	0.9836	0.0537
TLR1	0.8942	1.1163	1.0082	0.9157	0.9836	0.0507
LOC727847	0.9043	1.0913	1.0236	0.9151	0.9836	0.0449
LOC730275	0.9600	0.9921	0.9939	0.9884	0.9836	0.0080
FGFR1	1.0060	0.9955	1.0226	0.9103	0.9836	0.0251
PTGFRN	0.9510	1.2798	0.9242	0.7794	0.9836	0.1057
LOC441737	0.9214	0.9852	1.0386	0.9892	0.9836	0.0240
IGFBP1	0.9242	1.0805	0.9979	0.9319	0.9836	0.0363
DDAH1	1.0714	1.2070	1.0342	0.6219	0.9836	0.1261
LOC1001311	0.9900	1.0544	0.9210	0.9691	0.9836	0.0277
KIR2DL2	0.9017	1.0747	1.0558	0.9023	0.9836	0.0473
FLJ46552	1.0577	1.1195	1.0646	0.6928	0.9836	0.0979
LRRC15	1.0419	1.1017	0.9304	0.8606	0.9836	0.0542
LOC652787	0.9326	1.0373	1.0439	0.9207	0.9836	0.0330
SEMA3A	1.0047	1.0811	1.0217	0.8272	0.9836	0.0547
ABLIM2	1.0288	1.0214	1.0322	0.8522	0.9837	0.0439
FLJ16165	0.8735	1.1263	1.0746	0.8602	0.9837	0.0683
PANX1	0.9223	1.1502	1.0970	0.7652	0.9837	0.0876
LOC642659	0.9524	0.9416	1.0908	0.9498	0.9837	0.0358
NPS	0.9723	0.9552	1.0538	0.9533	0.9837	0.0238
OR4D2	0.9454	1.0544	1.0564	0.8785	0.9837	0.0436
LOC652505	0.9314	1.1929	0.9107	0.8997	0.9837	0.0700
LOC154860	0.9057	1.2145	0.9311	0.8834	0.9837	0.0776
TRIM74	1.0186	1.0115	1.0095	0.8951	0.9837	0.0296
LOC642736	0.8695	1.0310	1.1813	0.8530	0.9837	0.0771
LOC1001293	0.8846	1.0971	0.9929	0.9602	0.9837	0.0441
SUV39H2	0.8547	1.2343	0.9169	0.9288	0.9837	0.0851
LOC652599	1.0054	1.0103	1.0017	0.9174	0.9837	0.0222
TELO2	1.3862	0.9229	0.8007	0.8251	0.9837	0.1367
FAM86B2	0.9821	0.9988	1.0057	0.9481	0.9837	0.0128
P2RY4	0.8938	1.1206	0.9882	0.9323	0.9837	0.0496
KRT18P44	0.9053	1.0842	1.0065	0.9389	0.9837	0.0396
LOC1001294	0.9702	1.0017	1.0631	0.8999	0.9837	0.0339
C3orf54	0.9596	1.1224	0.9117	0.9413	0.9837	0.0473
HIPK1	0.9616	1.0134	1.0618	0.8982	0.9837	0.0351
KLRK1	0.8385	1.2631	1.0015	0.8318	0.9837	0.1011
LOC646855	0.8434	1.1319	1.0867	0.8730	0.9837	0.0733
UAP1	0.8899	1.1714	1.2680	0.6056	0.9837	0.1494
LOC121006	0.9301	1.0579	1.0144	0.9326	0.9837	0.0315

SYT16	0.7730	1.3131	1.0349	0.8140	0.9838	0.1239
LOC1001321	0.8270	1.0746	1.0862	0.9472	0.9838	0.0610
LOC646268	0.9128	1.1359	1.0146	0.8718	0.9838	0.0589
MEIS1	0.7728	1.1245	1.1642	0.8735	0.9838	0.0953
LOC650721	0.9147	1.0654	1.0338	0.9212	0.9838	0.0386
BATF3	0.8803	1.1241	1.0315	0.8992	0.9838	0.0576
GPR125	0.8913	1.1509	0.9698	0.9232	0.9838	0.0580
RBM8A	0.8395	1.2011	1.0168	0.8777	0.9838	0.0818
RASGEF1B	0.9247	0.9904	1.1292	0.8908	0.9838	0.0527
NYX	0.9978	0.9605	1.1461	0.8307	0.9838	0.0649
SEC16B	0.9590	1.0842	0.9450	0.9469	0.9838	0.0336
LOC646109	1.0481	1.0162	0.9437	0.9272	0.9838	0.0289
FAM82A1	0.9532	0.9732	1.1354	0.8734	0.9838	0.0549
LOC645805	0.9556	0.9971	1.0964	0.8861	0.9838	0.0440
LOC652470	0.9557	1.1125	0.9296	0.9374	0.9838	0.0432
LOC401220	0.8714	1.0667	1.0497	0.9474	0.9838	0.0458
LOC646654	0.9051	1.0298	1.0453	0.9551	0.9838	0.0328
FZD10	0.9937	1.1284	0.9327	0.8804	0.9838	0.0535
LOC641955	0.9040	1.0526	1.0051	0.9735	0.9838	0.0312
FCRL4	1.0022	0.9867	1.0147	0.9317	0.9838	0.0183
CHRNA9	0.9463	1.0779	1.0379	0.8732	0.9838	0.0460
SLC9A4	0.9763	1.0815	0.9753	0.9022	0.9838	0.0369
LOC642265	0.9140	1.2108	0.9187	0.8918	0.9838	0.0759
LOC646405	0.8983	1.1005	1.0263	0.9101	0.9838	0.0484
MGC10646	0.9692	1.0795	0.9885	0.8981	0.9838	0.0373
KLHDC7A	0.8808	1.1187	1.0389	0.8970	0.9838	0.0573
LOC645172	0.9093	1.0122	1.1486	0.8652	0.9839	0.0630
HR	0.9903	1.0257	1.0405	0.8789	0.9839	0.0365
LOC652076	0.8731	1.1942	1.0881	0.7800	0.9839	0.0953
BCORL1	0.8848	1.2106	0.8351	1.0048	0.9839	0.0836
PDE10A	0.9507	0.9827	1.1053	0.8968	0.9839	0.0442
MKRN3	0.9323	1.2597	0.9373	0.8062	0.9839	0.0968
LOC401914	0.9766	1.0399	0.9919	0.9270	0.9839	0.0233
LOC652142	0.8387	1.1858	0.9549	0.9560	0.9839	0.0727
COL8A1	0.9817	1.0939	0.9749	0.8850	0.9839	0.0428
TBX5	0.9709	1.1009	0.9509	0.9127	0.9839	0.0408
SHANK2	0.9916	1.0638	1.0147	0.8654	0.9839	0.0423
LOC728250	0.9827	0.9750	1.0001	0.9778	0.9839	0.0056
DSCAM	0.8227	1.1889	1.0069	0.9170	0.9839	0.0780
SDHAP3	0.9416	1.1281	0.9783	0.8875	0.9839	0.0516
FCGR1C	0.9637	1.0652	0.9003	1.0064	0.9839	0.0348
LOC642447	0.8923	1.0953	1.0041	0.9438	0.9839	0.0436
LOC651849	0.8825	1.1247	0.9365	0.9919	0.9839	0.0520
MIR423	0.8955	1.1224	1.0202	0.8975	0.9839	0.0546
DOK2	0.9614	1.1525	0.9704	0.8513	0.9839	0.0624
LOC731837	1.0152	1.0978	1.0015	0.8211	0.9839	0.0583

LOC648500	0.8802	1.0339	1.0990	0.9225	0.9839	0.0502
PNPLA2	1.0595	1.1118	1.0565	0.7079	0.9839	0.0929
LOC730994	0.8793	1.0803	1.0040	0.9721	0.9839	0.0416
SH3RF3	0.9345	1.1090	1.0424	0.8498	0.9839	0.0574
LOC1001327	0.9161	1.1906	1.0362	0.7929	0.9839	0.0849
LOC1001305	0.9495	1.0192	1.0905	0.8765	0.9839	0.0459
ANXA13	0.9252	1.0779	0.9525	0.9802	0.9839	0.0333
TGM2	0.9340	1.0783	1.0241	0.8993	0.9839	0.0410
WARS2	0.9614	1.0589	1.0380	0.8774	0.9839	0.0412
LOC642220	0.9879	1.0889	0.9392	0.9197	0.9839	0.0378
MIR651	0.8555	1.1279	1.0672	0.8852	0.9840	0.0670
LOC644891	0.9993	1.1602	0.8793	0.8970	0.9840	0.0644
LOC653527	0.8224	1.0505	1.0662	0.9968	0.9840	0.0559
LOC1001283	0.9155	1.0828	1.0478	0.8899	0.9840	0.0478
MT3	0.8601	1.1444	1.0564	0.8750	0.9840	0.0696
RALY	0.9398	1.2513	1.0101	0.7347	0.9840	0.1066
GPR62	0.9970	1.0347	0.9535	0.9508	0.9840	0.0199
LOC1001288	0.9630	1.0896	0.9590	0.9243	0.9840	0.0363
ELAVL3	0.9457	1.0964	1.0781	0.8157	0.9840	0.0654
DEFB1	0.9255	1.0205	1.1224	0.8675	0.9840	0.0559
LOC728831	0.9616	1.1189	0.9540	0.9014	0.9840	0.0469
SLC25A24	0.8314	1.2541	0.9721	0.8784	0.9840	0.0947
ZNF461	0.9524	1.1696	0.9795	0.8344	0.9840	0.0694
LOC1001283	0.7752	1.1833	1.1502	0.8273	0.9840	0.1063
C8orf47	1.0238	1.0782	1.0008	0.8332	0.9840	0.0528
LOC92497	0.8928	1.1622	0.9347	0.9463	0.9840	0.0605
SLC38A6	0.9128	1.0205	1.0613	0.9414	0.9840	0.0344
LOC644064	0.9077	1.1562	0.8798	0.9923	0.9840	0.0622
LRRC44	1.0077	0.9992	0.9712	0.9580	0.9840	0.0117
MAP2K7	0.9036	1.1302	1.0363	0.8659	0.9840	0.0609
SCN4B	0.9266	1.1986	0.9241	0.8868	0.9840	0.0721
XPNPEP3	0.9318	1.1857	1.1262	0.6924	0.9840	0.1113
CSN2	0.8925	1.1194	1.0418	0.8823	0.9840	0.0580
INMT	0.9613	1.0292	0.9953	0.9502	0.9840	0.0179
FLJ12334	0.9140	1.0323	0.9869	1.0029	0.9840	0.0252
GLIS1	0.9588	1.0382	0.9566	0.9825	0.9840	0.0190
ALS2CR12	0.9962	1.2158	0.9920	0.7321	0.9840	0.0989
LOC653616	0.8857	1.1428	0.8769	1.0307	0.9840	0.0636
OR2T10	1.0159	0.9825	0.9760	0.9617	0.9840	0.0115
GC	0.8578	1.0772	1.1108	0.8903	0.9840	0.0642
LOC1001317	0.9069	1.0635	1.0159	0.9499	0.9841	0.0347
TXLNB	0.9858	1.1149	0.9454	0.8901	0.9841	0.0478
C1orf88	0.9407	1.0406	1.0733	0.8817	0.9841	0.0443
UNC13C	0.9430	1.0613	1.0350	0.8970	0.9841	0.0386
LOC730291	0.9868	0.9849	1.0144	0.9502	0.9841	0.0132
MMP23A	0.9130	1.0430	1.0279	0.9524	0.9841	0.0309

ALPP	0.8182	1.3786	0.9654	0.7741	0.9841	0.1377
F2	0.9185	1.0365	0.9826	0.9988	0.9841	0.0246
UGT2A2	0.9360	0.9720	1.0589	0.9694	0.9841	0.0262
ANKS1B	0.9506	1.1033	0.9330	0.9494	0.9841	0.0399
TSPY2	0.9526	1.1390	0.8857	0.9591	0.9841	0.0542
PRB3	0.8774	1.1745	1.0777	0.8069	0.9841	0.0855
LOC442229	0.9056	1.0821	1.0225	0.9263	0.9841	0.0414
SLC26A8	0.9712	1.0663	1.0175	0.8814	0.9841	0.0393
ANAPC10	0.7357	1.2866	1.1231	0.7911	0.9841	0.1322
SLC17A4	0.9288	1.0729	1.2269	0.7079	0.9841	0.1104
LOC648672	0.9081	1.1156	1.0790	0.8338	0.9841	0.0675
KCNH5	0.9630	1.0833	1.0018	0.8884	0.9841	0.0406
ADSSL1	0.8610	1.2167	0.9783	0.8805	0.9841	0.0817
ASPN	0.9556	0.9854	1.0666	0.9289	0.9841	0.0298
XKR9	0.9248	1.1102	0.9981	0.9035	0.9841	0.0466
LOC642921	0.8610	1.0559	1.0520	0.9676	0.9841	0.0458
LOC1001317	0.9204	1.0288	1.0622	0.9252	0.9841	0.0361
LOC441034	0.9328	1.0925	1.0939	0.8174	0.9841	0.0672
MIR1282	0.8978	0.9931	0.9139	1.1318	0.9841	0.0534
SLC36A3	0.9730	1.0344	1.0111	0.9182	0.9841	0.0254
CA6	0.9928	1.0957	0.9594	0.8887	0.9842	0.0430
LOC1001334	0.9097	1.2078	0.9038	0.9154	0.9842	0.0746
SHC3	1.0574	1.0341	0.9544	0.8908	0.9842	0.0381
SPINT4	0.9411	1.0945	0.9627	0.9384	0.9842	0.0372
G6PC2	0.9485	1.0427	0.9959	0.9496	0.9842	0.0224
DDX25	0.9627	1.0275	1.0371	0.9095	0.9842	0.0299
CYP4Z1	0.9232	1.0757	1.0119	0.9259	0.9842	0.0368
STX1A	1.0169	1.1429	0.9558	0.8212	0.9842	0.0669
FCGR3A	0.8848	1.1191	1.0604	0.8725	0.9842	0.0622
LOC652745	0.9010	1.1228	1.0431	0.8699	0.9842	0.0596
LOC1001312	0.9439	1.0121	1.0150	0.9659	0.9842	0.0175
PQLC2	0.9671	1.0708	0.9873	0.9116	0.9842	0.0330
SH3TC1	1.0423	1.0848	0.9923	0.8174	0.9842	0.0587
ZBED2	0.9515	1.0000	1.0332	0.9521	0.9842	0.0199
C4orf12	0.9329	1.0616	1.0185	0.9238	0.9842	0.0335
ADIPOR2	0.8842	1.3574	0.9844	0.7109	0.9842	0.1366
CCR2	0.9229	1.0812	1.0232	0.9096	0.9842	0.0411
SNORD116-1	0.9226	1.1522	0.9984	0.8637	0.9842	0.0624
LCA5L	0.9426	1.0938	1.0012	0.8992	0.9842	0.0421
POMC	0.9036	1.0898	0.9989	0.9446	0.9842	0.0402
LOC653366	0.9812	1.0978	1.0228	0.8352	0.9842	0.0552
SERPINA1	0.9495	1.0248	1.0524	0.9102	0.9842	0.0329
LRCH2	0.9906	1.0585	0.9494	0.9384	0.9842	0.0272
GOSR1	0.8495	1.2020	1.0620	0.8235	0.9842	0.0901
LOC647493	0.8943	1.0249	1.0906	0.9273	0.9843	0.0450
KHDRBS2	0.9709	1.0477	0.9979	0.9206	0.9843	0.0265

ROS1	0.9343	0.9536	1.1233	0.9258	0.9843	0.0467
LOC644593	0.9491	1.3616	0.8084	0.8178	0.9843	0.1298
GRRP1	0.8910	1.0660	1.0530	0.9270	0.9843	0.0441
FLJ37512	0.9361	1.0325	1.1228	0.8457	0.9843	0.0599
LOC1001295	0.9518	0.9682	1.0567	0.9603	0.9843	0.0244
LOC650036	1.0191	1.0859	0.9462	0.8859	0.9843	0.0435
ARHGAP6	0.8897	1.2074	0.9988	0.8412	0.9843	0.0813
LOC92017	0.9678	1.1402	0.9626	0.8665	0.9843	0.0570
MIR494	1.0454	1.0522	0.9877	0.8519	0.9843	0.0464
FCGR1B	0.9587	1.0582	1.0293	0.8909	0.9843	0.0375
MIR1-2	0.9235	1.1167	0.9878	0.9092	0.9843	0.0473
LOC391859	0.9482	1.1849	0.8991	0.9050	0.9843	0.0678
ALPK1	0.9068	1.0916	1.0677	0.8711	0.9843	0.0557
ESP33	0.9195	1.0107	1.0373	0.9698	0.9843	0.0257
LOC642939	0.9433	1.1529	0.8993	0.9417	0.9843	0.0571
LOC647025	1.0248	1.1508	0.8716	0.8900	0.9843	0.0652
LOC1001309	0.9948	0.9575	1.0376	0.9473	0.9843	0.0205
LOC644954	0.8531	1.1127	0.9834	0.9880	0.9843	0.0530
LOC222699	0.9169	1.1786	0.8891	0.9526	0.9843	0.0660
LOC648405	1.0372	1.0307	1.0004	0.8690	0.9843	0.0393
MBOAT2	0.8907	1.3204	1.0111	0.7151	0.9843	0.1274
MPZL3	0.7811	1.3650	0.9469	0.8444	0.9843	0.1314
ODZ1	0.9164	1.0760	0.9953	0.9496	0.9843	0.0346
LOC653554	1.0530	0.9782	1.0299	0.8762	0.9843	0.0393
COL23A1	0.9319	1.0188	0.9914	0.9951	0.9843	0.0185
FAM175A	0.9490	1.1446	1.0963	0.7474	0.9843	0.0893
ZNF623	0.9803	1.1330	0.9463	0.8778	0.9843	0.0539
SEMA3E	0.8042	1.1024	1.2462	0.7845	0.9843	0.1136
ENTHD1	0.9510	1.0918	0.9946	0.8999	0.9843	0.0407
OR5K4	1.0622	1.0907	0.9360	0.8485	0.9843	0.0564
OR6K6	0.8761	1.0174	1.2088	0.8351	0.9843	0.0844
LOC283553	1.0366	1.1233	0.9791	0.7984	0.9843	0.0687
LOC645537	0.9497	1.1120	0.9357	0.9401	0.9843	0.0426
RYR3	1.0580	1.0238	0.9843	0.8714	0.9844	0.0405
LOC643901	1.0398	1.0615	0.9803	0.8559	0.9844	0.0462
LOC1001341	0.9932	1.0061	0.9858	0.9523	0.9844	0.0115
ZNF80	0.9015	1.1523	0.9975	0.8861	0.9844	0.0612
LOC1001283	0.9137	1.1085	1.0294	0.8859	0.9844	0.0517
PLA2G1B	0.9653	1.1248	1.0396	0.8077	0.9844	0.0673
SLC41A2	0.8801	1.3643	0.9531	0.7400	0.9844	0.1341
KCNN4	0.9205	1.1522	0.9958	0.8691	0.9844	0.0617
PYY2	0.9724	1.0186	1.0426	0.9039	0.9844	0.0305
LAMB4	0.8705	1.1509	0.9321	0.9839	0.9844	0.0602
LOC1001311	0.9623	1.1003	0.9636	0.9113	0.9844	0.0405
LHFPL3	0.9569	1.0172	1.0305	0.9330	0.9844	0.0234
TIMM13	1.0434	1.1031	0.9177	0.8735	0.9844	0.0535

LOC651196	0.7474	1.1671	0.9925	1.0306	0.9844	0.0874
PSEN2	0.9902	1.1946	1.0945	0.6583	0.9844	0.1164
S1PR1	0.9259	0.9927	1.0705	0.9485	0.9844	0.0319
NKX2-5	0.9207	1.0976	0.9604	0.9589	0.9844	0.0388
PCDHA5	0.9012	1.1022	1.0253	0.9090	0.9844	0.0484
BIRC7	0.9154	1.0770	1.0123	0.9330	0.9844	0.0374
ZNF628	0.9291	1.0416	0.8887	1.0784	0.9844	0.0450
LOC649812	0.9285	1.0273	1.0967	0.8852	0.9844	0.0478
OR1L1	0.8636	1.0908	1.1362	0.8471	0.9844	0.0752
DUOXA1	1.0411	1.1075	0.9655	0.8235	0.9844	0.0610
IL9	1.0163	0.9712	1.0273	0.9230	0.9844	0.0238
FGFR3	0.9188	1.1691	1.0194	0.8304	0.9844	0.0727
PTH	0.9215	1.0663	1.0577	0.8922	0.9844	0.0452
OR51F2	1.0011	1.0509	0.9969	0.8889	0.9844	0.0341
MUC15	0.8826	1.2018	0.9234	0.9299	0.9844	0.0732
LOC642987	0.9853	0.7998	1.0728	1.0798	0.9844	0.0652
LOC151300	0.8752	1.1489	1.0125	0.9013	0.9844	0.0624
LOC729878	0.9357	1.1171	0.9799	0.9051	0.9844	0.0468
ANGPTL3	0.9622	1.0947	0.9956	0.8854	0.9844	0.0434
TAF9L	0.8988	1.0861	1.3509	0.6020	0.9845	0.1577
LOC1001334	0.9952	1.0591	1.0133	0.8702	0.9845	0.0404
C17orf67	0.9077	1.1414	0.9724	0.9163	0.9845	0.0542
SLC25A23	0.8346	1.2795	1.0617	0.7620	0.9845	0.1173
CECR1	0.8868	1.1316	1.0209	0.8986	0.9845	0.0577
LOC1001314	0.8251	1.1969	1.0808	0.8351	0.9845	0.0923
OR5V1	0.8940	1.0797	0.9935	0.9706	0.9845	0.0382
LOC645701	0.8251	1.0937	1.0473	0.9718	0.9845	0.0588
GYPA	0.8803	1.1175	0.9718	0.9683	0.9845	0.0491
KRTAP12-1	0.8172	1.2339	1.0479	0.8390	0.9845	0.0981
LOC654101	0.9458	1.0144	1.0797	0.8981	0.9845	0.0397
ZFAND2A	0.9218	1.3593	0.9479	0.7091	0.9845	0.1359
LOC652009	0.9786	1.0574	0.9493	0.9527	0.9845	0.0252
HSGT1	0.9721	1.2515	0.9180	0.7964	0.9845	0.0963
PRPF39	0.8936	1.1161	1.0993	0.8290	0.9845	0.0724
MIR548L	0.9832	0.9960	1.0093	0.9495	0.9845	0.0128
SIRT3	0.9973	0.9928	1.0267	0.9213	0.9845	0.0224
TMEM115	0.9256	1.2872	0.9302	0.7951	0.9845	0.1056
RBMY1A3P	0.9098	1.1252	0.9861	0.9170	0.9845	0.0499
LOC651008	0.9227	1.0493	1.0724	0.8937	0.9845	0.0447
ERVK6	0.9160	1.0060	1.0383	0.9779	0.9845	0.0260
LRCH1	0.9706	0.9994	1.0585	0.9096	0.9845	0.0310
LOC645330	0.9129	1.0969	1.0194	0.9089	0.9845	0.0454
RNY5	0.9098	1.1578	1.0826	0.7879	0.9846	0.0836
OR56A4	0.9713	1.0813	0.9059	0.9798	0.9846	0.0362
RAB41	1.0079	1.0094	1.0248	0.8961	0.9846	0.0297
MIR212	0.8685	1.1669	1.0890	0.8138	0.9846	0.0851

IGDCC3	0.9685	1.0441	1.0120	0.9136	0.9846	0.0283
LOC284352	0.8723	1.1152	1.0298	0.9210	0.9846	0.0546
LOC1001267	0.9189	1.0773	1.0118	0.9303	0.9846	0.0372
DUXA	0.9664	1.0768	1.0153	0.8798	0.9846	0.0416
PCDHGB1	0.9074	1.1243	1.0050	0.9016	0.9846	0.0523
MBNL3	0.9765	1.0502	1.0209	0.8907	0.9846	0.0348
LOC728465	0.8347	1.2251	1.0813	0.7972	0.9846	0.1020
CCDC107	0.9447	1.2340	0.9092	0.8505	0.9846	0.0854
C10orf82	0.9539	1.0757	1.0190	0.8898	0.9846	0.0402
LOC728518	1.0072	1.0398	0.9443	0.9471	0.9846	0.0234
LOC1001281	0.9486	1.0770	0.9971	0.9157	0.9846	0.0350
PLEKHG5	0.9511	1.0406	1.0388	0.9080	0.9846	0.0330
ZNF534	0.9720	1.0252	1.0033	0.9379	0.9846	0.0190
LOC643669	1.0002	1.0732	1.0212	0.8439	0.9846	0.0494
TNFAIP3	0.8762	1.1629	1.0372	0.8622	0.9846	0.0715
BHLHB4	0.9073	1.1701	1.0041	0.8570	0.9846	0.0689
LOC1001293	0.8877	1.1254	1.0281	0.8973	0.9846	0.0568
SOX17	1.0160	1.0221	0.9879	0.9125	0.9846	0.0252
DNAJB8	1.0033	1.0039	0.9493	0.9821	0.9846	0.0128
LOC441709	0.9861	1.0059	1.0232	0.9233	0.9846	0.0218
KLF12	0.9642	1.0643	1.0126	0.8975	0.9846	0.0355
NHLRC4	0.8089	1.1996	1.0249	0.9052	0.9846	0.0842
TNK1	0.9240	0.9927	0.9993	1.0226	0.9846	0.0212
UFC1	0.8028	1.4304	1.0857	0.6197	0.9846	0.1768
SNORA5C	0.8653	1.1668	0.9937	0.9128	0.9847	0.0662
SLC24A4	0.8987	1.0808	0.9855	0.9736	0.9847	0.0374
GDAP1	0.7925	1.2240	1.0796	0.8425	0.9847	0.1014
LOC1001322	0.9316	1.1162	1.0350	0.8559	0.9847	0.0572
CYP17A1	0.9905	1.0882	0.9505	0.9095	0.9847	0.0383
CSMD3	0.9745	1.0630	0.9820	0.9191	0.9847	0.0297
LOC1001319	0.9322	1.1496	1.0045	0.8524	0.9847	0.0631
MIR769	0.9997	1.0534	1.0127	0.8728	0.9847	0.0390
TSPAN4	0.8291	1.2794	1.0024	0.8278	0.9847	0.1065
LOC1001333	0.8451	1.1293	1.0894	0.8749	0.9847	0.0727
LOC647247	1.0010	0.9654	0.9996	0.9728	0.9847	0.0091
MRGPRF	0.9751	1.0520	0.9869	0.9247	0.9847	0.0262
LOC645070	0.9799	1.0943	0.9789	0.8856	0.9847	0.0427
LOC642922	0.9039	1.0669	1.0782	0.8897	0.9847	0.0509
LOC647844	0.9536	1.0023	0.9973	0.9856	0.9847	0.0110
LOC652160	1.2004	0.9997	0.9832	0.7556	0.9847	0.0909
C20orf54	0.9542	1.1198	0.9857	0.8792	0.9847	0.0502
MIR29B1	0.9217	1.1375	1.0842	0.7955	0.9847	0.0780
FCRL6	0.9649	1.0191	1.0798	0.8751	0.9847	0.0434
RDH5	1.0283	1.1178	0.9188	0.8740	0.9847	0.0549
LOC650755	0.9369	0.9974	1.0034	1.0012	0.9847	0.0160
LOC389633	1.0039	1.1351	0.9992	0.8007	0.9847	0.0689

OR2F1	0.8322	1.0264	1.1340	0.9463	0.9847	0.0637
FOXC1	0.8016	1.1445	1.0753	0.9175	0.9847	0.0773
LOC727722	0.9760	1.1170	0.9359	0.9101	0.9847	0.0461
LOC643120	0.9106	1.0623	1.0021	0.9640	0.9847	0.0319
LOC653498	0.8717	1.1153	1.0194	0.9325	0.9847	0.0530
LOC1001294	0.9277	1.0358	1.0035	0.9719	0.9847	0.0230
TCEAL7	0.8852	1.0458	1.0512	0.9568	0.9848	0.0396
CACNG3	0.9595	1.0392	1.0660	0.8743	0.9848	0.0432
LOC131054	0.8739	1.0412	0.9825	1.0414	0.9848	0.0395
IL13	1.0055	1.0578	1.0431	0.8327	0.9848	0.0519
BPY2C	0.9643	1.0655	0.9815	0.9278	0.9848	0.0291
FDXR	0.8980	1.1597	0.9732	0.9080	0.9848	0.0607
LOC646296	0.9538	1.1583	0.9836	0.8433	0.9848	0.0652
LOC57399	0.9066	1.0716	1.0456	0.9152	0.9848	0.0430
OTOF	0.9238	1.0257	1.0377	0.9519	0.9848	0.0278
ABCB5	0.9821	0.9768	1.0154	0.9648	0.9848	0.0108
LOC644112	0.9587	1.0693	0.9889	0.9223	0.9848	0.0313
SNORD50A	0.9168	0.9650	1.1423	0.9151	0.9848	0.0538
LOC401007	0.9562	1.0293	1.0682	0.8855	0.9848	0.0404
LOC644215	0.9398	1.1055	0.9446	0.9493	0.9848	0.0403
LOC1001246	0.9255	1.0394	1.1159	0.8584	0.9848	0.0575
ITPK1	0.9032	1.1619	1.0542	0.8200	0.9848	0.0764
MIR665	0.8807	1.2903	0.9604	0.8078	0.9848	0.1065
C9orf24	0.9353	1.1146	0.9777	0.9117	0.9848	0.0454
TLR6	0.9545	1.0780	1.0034	0.9033	0.9848	0.0372
LOC1001278	0.9352	1.0770	1.0047	0.9224	0.9848	0.0356
LOC730194	1.0177	0.9780	0.9268	1.0167	0.9848	0.0214
OR51L1	0.9462	0.9575	0.9808	1.0548	0.9848	0.0244
LOC644912	0.9469	1.0246	1.0661	0.9017	0.9848	0.0371
ADAMTSL2	0.8635	1.1041	1.0021	0.9697	0.9848	0.0496
LOC389671	1.0611	1.0377	0.9086	0.9319	0.9848	0.0379
CAPNS2	0.9423	1.0995	0.8608	1.0367	0.9848	0.0525
ITGB6	0.9717	1.0745	0.9989	0.8943	0.9848	0.0372
LOC644563	0.9543	1.3046	1.0247	0.6558	0.9848	0.1332
LOC643246	0.8594	1.1549	0.9029	1.0222	0.9848	0.0663
FLJ44451	0.9030	1.1522	1.0381	0.8461	0.9848	0.0688
TTC21B	0.9647	1.1161	1.0070	0.8516	0.9849	0.0547
ZBED4	0.9663	1.2966	0.8762	0.8004	0.9849	0.1093
LOC1001328	0.9318	1.1516	0.9673	0.8888	0.9849	0.0578
COL4A1	0.9283	1.0745	1.0145	0.9222	0.9849	0.0366
UBLCP1	0.9904	1.0601	1.1361	0.7528	0.9849	0.0829
TMPRSS11A	0.9485	1.0742	1.0341	0.8827	0.9849	0.0430
LOC643213	0.9136	1.0505	1.0098	0.9656	0.9849	0.0294
CCDC149	0.9343	1.1637	0.8163	1.0252	0.9849	0.0734
LOC1001312	0.9958	1.0715	0.9477	0.9245	0.9849	0.0325
LOC644231	0.8646	1.0272	1.0839	0.9639	0.9849	0.0470

LOC646581	0.9174	1.0576	1.0030	0.9616	0.9849	0.0299
LOC732434	0.8718	0.9526	1.1446	0.9706	0.9849	0.0574
FBXO22OS	0.9059	1.0369	1.0864	0.9104	0.9849	0.0455
FAM181A	0.9887	1.0819	0.9411	0.9280	0.9849	0.0349
LOC651568	0.8536	1.1350	1.0615	0.8895	0.9849	0.0675
LOC651986	0.9504	1.0105	1.0268	0.9519	0.9849	0.0198
TTLL9	0.8954	1.0951	0.9854	0.9638	0.9849	0.0414
C4orf28	0.8046	1.2096	1.0853	0.8400	0.9849	0.0975
PAQR9	1.0221	1.0738	0.9958	0.8480	0.9849	0.0484
LOC644427	0.9917	1.0017	1.0381	0.9081	0.9849	0.0275
PLSCR5	0.9945	1.1000	0.9861	0.8591	0.9849	0.0493
TAAR1	0.9333	1.0804	0.9878	0.9381	0.9849	0.0341
LOC257396	0.6854	1.2985	1.1844	0.7713	0.9849	0.1510
LOC646299	0.9518	1.0503	1.0702	0.8673	0.9849	0.0470
YTHDF3	0.9631	1.1684	1.0608	0.7474	0.9849	0.0896
LOC1001327	0.9503	1.0085	1.0187	0.9622	0.9849	0.0169
LOC650787	0.9757	1.0395	1.0589	0.8656	0.9849	0.0436
IZUMO1	0.8943	1.0252	0.9288	1.0914	0.9849	0.0450
HRSP12	0.8361	1.4278	0.9690	0.7069	0.9849	0.1570
LOC644504	0.9142	0.9995	1.0300	0.9960	0.9849	0.0248
CNTNAP4	0.9255	1.0339	0.9720	1.0082	0.9849	0.0235
SNAI1	1.0169	1.0117	1.0000	0.9111	0.9849	0.0249
TEX12	0.9501	1.0421	1.1420	0.8056	0.9849	0.0715
SOX2OT	0.8947	0.9682	1.0842	0.9926	0.9849	0.0391
LOC648054	0.9566	1.0727	1.0865	0.8240	0.9849	0.0610
LOC440225	0.9593	1.0581	0.9850	0.9374	0.9849	0.0262
LOC645048	0.9884	1.0589	0.9686	0.9238	0.9849	0.0281
PPARA	0.9877	1.0656	0.9719	0.9146	0.9849	0.0311
LOC1001289	0.9071	1.0793	0.9696	0.9838	0.9849	0.0356
FLJ45974	0.8448	1.0983	1.1652	0.8314	0.9849	0.0859
FLJ41352	0.9598	1.0880	0.9622	0.9298	0.9849	0.0351
C1QTNF1	0.8853	1.0461	1.0265	0.9819	0.9850	0.0358
PCDHGB8P	1.0188	1.1097	0.9053	0.9060	0.9850	0.0494
SNX18	0.8678	1.1606	0.9957	0.9158	0.9850	0.0642
ANKRD20B	0.9779	1.0829	0.9916	0.8875	0.9850	0.0400
HSD11B1	0.9279	1.0627	1.0279	0.9213	0.9850	0.0356
OR2A42	0.9297	1.1255	0.9771	0.9076	0.9850	0.0490
AP2S1	0.9756	1.2017	0.9854	0.7772	0.9850	0.0867
EXD3	0.9435	1.0510	0.9812	0.9642	0.9850	0.0233
LOC642933	0.9743	1.0408	1.0572	0.8676	0.9850	0.0430
LOC153328	0.9661	1.0901	1.0611	0.8226	0.9850	0.0603
LOC442057	0.8639	1.0676	0.9569	1.0516	0.9850	0.0472
LOC152084	1.0236	1.0005	1.0517	0.8641	0.9850	0.0416
CYS1	0.9605	1.1418	0.9921	0.8455	0.9850	0.0610
LOC643662	0.8850	1.0236	1.0907	0.9407	0.9850	0.0453
DEPDC4	1.0000	1.0761	0.9672	0.8967	0.9850	0.0372

HOXD9	0.8521	1.1482	0.9728	0.9668	0.9850	0.0611
SLC23A2	0.9162	1.1107	0.9932	0.9199	0.9850	0.0455
LOC729378	0.8781	1.0718	0.9658	1.0243	0.9850	0.0417
ZBTB32	0.8529	1.1094	1.0763	0.9014	0.9850	0.0634
LOC643402	1.0023	1.0186	1.0158	0.9033	0.9850	0.0275
SYCP3	0.9868	1.0606	1.0449	0.8478	0.9850	0.0484
OR6B3	0.9408	1.0731	1.0848	0.8414	0.9850	0.0580
OR2T2	0.9283	1.1448	0.9855	0.8815	0.9850	0.0573
LOC650716	0.8920	1.1694	0.9987	0.8799	0.9850	0.0670
SLC6A15	0.9455	1.0155	1.0428	0.9363	0.9850	0.0261
CCDC127	0.9516	1.2351	0.9636	0.7899	0.9850	0.0923
GPR78	0.8634	1.1224	1.0753	0.8790	0.9850	0.0665
TMEM196	0.9226	1.1445	0.9002	0.9728	0.9850	0.0553
OR10A6	0.8899	1.1484	0.9761	0.9258	0.9850	0.0572
LOC441376	0.9436	1.0998	1.0086	0.8882	0.9850	0.0455
CD5L	0.8545	1.2809	0.9131	0.8916	0.9850	0.0994
SEC14L5	0.9866	1.0979	0.9273	0.9284	0.9851	0.0401
PLXNA2	0.9841	1.0401	1.0210	0.8949	0.9851	0.0322
PCDH11X	0.9573	1.0836	0.9798	0.9195	0.9851	0.0351
LOC1001336	1.0348	1.0523	0.9582	0.8949	0.9851	0.0363
CROCC	0.9007	1.0856	1.0431	0.9108	0.9851	0.0466
CYP2B6	0.9232	1.0809	0.9651	0.9711	0.9851	0.0337
LOC641298	0.9668	1.0419	1.0140	0.9175	0.9851	0.0273
LOC645931	0.9706	0.9851	1.0534	0.9311	0.9851	0.0255
CLDN9	0.9640	1.0509	0.9663	0.9591	0.9851	0.0220
LOC646195	1.0123	1.1017	0.9957	0.8306	0.9851	0.0565
ATP2B1	0.9223	1.1001	1.0598	0.8581	0.9851	0.0569
LOC440575	0.9073	1.1200	1.0814	0.8316	0.9851	0.0690
FER1L5	1.0116	1.0456	1.0066	0.8766	0.9851	0.0372
PCP4	0.9351	1.0113	1.1025	0.8915	0.9851	0.0463
MIR125B1	0.9772	0.9999	1.0173	0.9460	0.9851	0.0154
RBMY1D	0.9873	1.0447	1.0757	0.8327	0.9851	0.0540
MFI2	0.9513	1.0398	1.0391	0.9103	0.9851	0.0325
LOC644229	0.9187	1.1036	1.0397	0.8784	0.9851	0.0523
LOC648541	0.9279	1.0785	0.9579	0.9761	0.9851	0.0327
HIST1H4F	1.0058	1.0620	0.9433	0.9294	0.9851	0.0305
LOC1001296	0.9224	1.0816	1.0547	0.8818	0.9851	0.0490
ERCC-00033	1.0353	1.0598	0.9514	0.8940	0.9851	0.0382
LOC1001317	0.9444	1.0804	0.9886	0.9271	0.9851	0.0343
ACOT8	0.9448	1.3112	0.8905	0.7939	0.9851	0.1131
LOC728066	1.0251	1.0652	0.9289	0.9213	0.9851	0.0356
CCL4	0.8711	1.0614	1.0854	0.9226	0.9851	0.0523
LOC644466	1.0122	1.0587	1.0030	0.8665	0.9851	0.0414
MCF2L	0.8520	1.1562	1.0331	0.8992	0.9851	0.0687
LOC730993	1.0329	1.0917	0.9593	0.8566	0.9851	0.0507
C3AR1	0.9125	1.0346	1.0643	0.9291	0.9851	0.0378

IL2RB	0.8484	1.0324	1.1135	0.9463	0.9851	0.0569
SLC28A1	0.9955	1.0732	0.9904	0.8814	0.9851	0.0394
ERCC-00013	1.0084	0.9896	1.0631	0.8795	0.9851	0.0385
OR52R1	0.9295	1.0618	0.9831	0.9662	0.9851	0.0279
LOC646373	0.8547	1.1122	1.0782	0.8954	0.9852	0.0645
CYBA	0.8606	1.1217	0.9447	1.0137	0.9852	0.0552
LYST	0.8779	1.0891	1.0900	0.8836	0.9852	0.0603
LOC401875	0.9549	1.0983	0.9898	0.8976	0.9852	0.0422
SFXN3	0.7204	1.3579	1.0544	0.8080	0.9852	0.1430
SMOC1	0.9780	1.0181	1.0196	0.9249	0.9852	0.0223
MIR191	0.9796	1.0893	1.0068	0.8650	0.9852	0.0463
CSTL1	0.9524	1.0556	1.0185	0.9141	0.9852	0.0319
LOC645634	0.9084	0.9706	1.0701	0.9915	0.9852	0.0334
PLEKHA5	0.9225	1.0331	1.0230	0.9622	0.9852	0.0261
ERAP1	1.0184	1.1914	0.9041	0.8268	0.9852	0.0792
FBXO36	1.0490	1.0742	1.0004	0.8172	0.9852	0.0581
LOC283340	0.9927	0.9205	1.0654	0.9622	0.9852	0.0306
RNASE10	0.8634	1.1359	1.0718	0.8697	0.9852	0.0698
STX18	0.8783	1.2225	0.9778	0.8623	0.9852	0.0831
LOC1001317	0.9390	1.1365	0.9924	0.8729	0.9852	0.0560
KRTAP10-9	0.9453	1.1469	0.9830	0.8657	0.9852	0.0592
SH2B2	0.9114	1.0719	1.0656	0.8919	0.9852	0.0484
SMA3	0.9563	0.9324	1.0350	1.0171	0.9852	0.0243
IPP	0.9664	1.4196	0.8200	0.7349	0.9852	0.1525
CGB1	0.9096	1.1286	1.0531	0.8496	0.9852	0.0641
FLJ44385	0.9530	1.0615	1.0032	0.9232	0.9852	0.0303
LOC727821	0.8962	1.0038	1.2289	0.8121	0.9852	0.0902
LOC1001342	0.8594	1.1137	0.9537	1.0142	0.9852	0.0534
KIR3DS1	0.9023	1.0387	1.0692	0.9306	0.9852	0.0406
FAM71B	0.8543	1.0167	1.1239	0.9460	0.9852	0.0569
LOC728073	0.9617	1.0899	1.0235	0.8659	0.9853	0.0476
DUX3	0.8550	1.2254	1.0120	0.8486	0.9853	0.0885
LOC643256	1.0529	0.9711	1.0256	0.8914	0.9853	0.0356
C15orf48	0.9517	1.0493	1.0015	0.9384	0.9853	0.0253
LOC728846	0.9844	1.0761	0.9820	0.8985	0.9853	0.0363
LOC643275	0.9608	1.0113	1.0319	0.9371	0.9853	0.0219
LOC641983	0.9728	1.1190	0.9779	0.8714	0.9853	0.0509
LOC653866	0.9417	1.0104	1.0845	0.9044	0.9853	0.0397
LOC1001291	0.9354	0.9953	1.1248	0.8856	0.9853	0.0516
MIR766	0.9171	1.0859	1.0712	0.8669	0.9853	0.0549
LOC647034	0.9106	1.0845	0.9518	0.9942	0.9853	0.0372
MS4A10	0.9222	1.0171	1.0652	0.9366	0.9853	0.0339
TMEM128	0.8689	1.0911	1.0887	0.8925	0.9853	0.0606
MIR512-2	0.9005	1.1650	1.0387	0.8370	0.9853	0.0732
FLJ44817	0.9748	0.9886	0.9649	1.0129	0.9853	0.0104
OR6C3	0.9804	1.0915	0.9948	0.8745	0.9853	0.0444

LOC644507	1.0093	1.0186	0.9750	0.9383	0.9853	0.0183
LOC646241	0.8406	1.0266	1.1057	0.9684	0.9853	0.0559
FABP7	0.9775	1.1306	0.9566	0.8765	0.9853	0.0531
LOC649199	0.9405	0.9932	1.0059	1.0017	0.9853	0.0152
LOC643326	0.9401	1.0508	1.0368	0.9136	0.9853	0.0343
LOC728412	0.9087	1.2215	0.9942	0.8170	0.9853	0.0866
FLJ36032	0.9521	1.0507	1.0595	0.8790	0.9853	0.0430
ZYG11A	0.9352	1.0625	0.9835	0.9602	0.9853	0.0275
PALM2-AKAF	0.9858	0.9966	1.0449	0.9140	0.9853	0.0270
FAM53B	1.0323	1.1129	0.9799	0.8163	0.9853	0.0626
SNAP91	1.0221	1.0402	0.9988	0.8803	0.9854	0.0360
FMN1	0.9705	1.0614	0.9541	0.9554	0.9854	0.0256
KIAA1755	0.9133	1.1386	0.9875	0.9021	0.9854	0.0545
LOC653799	0.9556	1.1062	0.9982	0.8814	0.9854	0.0469
PSMD10	0.7969	1.4597	0.9457	0.7392	0.9854	0.1640
KCNJ5	0.9330	1.1038	0.9927	0.9120	0.9854	0.0430
LOC1001301	0.8759	1.0621	1.1564	0.8470	0.9854	0.0743
LOC1001283	0.8442	1.1144	1.0215	0.9613	0.9854	0.0566
NCRNA0011	1.0147	1.0142	0.9549	0.9578	0.9854	0.0168
FBXO9	0.8812	1.2046	1.0231	0.8327	0.9854	0.0835
HCK	0.9700	1.1445	0.8734	0.9537	0.9854	0.0571
POTEH	0.9422	1.0346	1.0314	0.9334	0.9854	0.0275
LOC651907	0.8499	1.1564	0.9806	0.9547	0.9854	0.0636
LOC652845	0.9133	1.0437	1.0921	0.8925	0.9854	0.0488
LOC642623	0.9178	1.0397	1.0209	0.9633	0.9854	0.0278
LOC283755	0.9679	1.0417	1.0444	0.8877	0.9854	0.0371
LOC442015	0.9129	1.1578	0.9811	0.8900	0.9854	0.0606
CST11	0.9291	1.1269	0.9758	0.9099	0.9854	0.0492
LOC728002	0.9227	1.0811	1.0944	0.8435	0.9854	0.0613
DBX1	1.0159	1.0530	0.9783	0.8945	0.9854	0.0339
SAR1P3	0.9042	1.2572	0.9715	0.8088	0.9854	0.0965
LOC342293	0.9726	1.0077	1.0120	0.9493	0.9854	0.0149
LOC1001288	0.9711	1.0790	1.0243	0.8673	0.9854	0.0451
LOC1001309	0.9639	1.1076	0.9510	0.9193	0.9855	0.0418
C1orf137	0.9324	1.0260	1.0466	0.9368	0.9855	0.0297
KIAA1957	1.0010	1.1128	0.9774	0.8506	0.9855	0.0538
LOC1001324	0.9005	1.0386	1.0388	0.9640	0.9855	0.0334
MYCNOS	0.8950	1.0958	0.9387	1.0123	0.9855	0.0440
LOC729291	0.9604	1.0770	1.0195	0.8848	0.9855	0.0411
LYZ	0.9582	1.0661	1.0040	0.9136	0.9855	0.0326
LOC127011	0.9309	1.0941	1.0364	0.8805	0.9855	0.0486
LOC653794	0.8935	1.1315	1.0388	0.8781	0.9855	0.0607
KLHDC7B	0.9744	1.0735	0.9833	0.9107	0.9855	0.0335
LOC285141	0.9303	1.1041	0.9493	0.9583	0.9855	0.0400
CCDC129	0.9627	1.0838	0.9679	0.9275	0.9855	0.0340
LOC642808	0.8357	1.1595	1.0235	0.9233	0.9855	0.0696

LOC650387	0.8938	1.1132	1.0953	0.8396	0.9855	0.0696
LOC1001311	1.0276	0.9995	0.9977	0.9172	0.9855	0.0238
LOC646900	0.8527	1.1479	1.2826	0.6588	0.9855	0.1411
ABCC10	1.0227	1.2576	0.9167	0.7451	0.9855	0.1072
FGF1	0.9222	1.0767	1.0181	0.9250	0.9855	0.0377
KALRN	0.9274	1.0930	1.0041	0.9176	0.9855	0.0407
LOC642889	0.9697	0.9848	1.1435	0.8441	0.9855	0.0614
ACR	0.9843	1.1270	0.9934	0.8374	0.9855	0.0592
IL12RB2	0.9808	1.0063	0.9955	0.9595	0.9855	0.0101
LOC388160	1.0341	1.0418	1.0198	0.8465	0.9855	0.0466
USP53	0.8979	1.0774	1.0970	0.8698	0.9855	0.0591
PLGLA1	0.9050	1.1171	0.9964	0.9236	0.9855	0.0481
CCDC40	0.9252	1.0276	1.0191	0.9702	0.9855	0.0238
MYH7B	0.9035	1.0938	1.0002	0.9446	0.9855	0.0412
LOC376693	0.9439	1.0122	1.1052	0.8808	0.9855	0.0481
PRDM16	0.9427	1.0704	1.0150	0.9141	0.9855	0.0354
LOC645535	0.9387	1.1537	0.9004	0.9494	0.9855	0.0570
MTNR1B	0.9027	1.1551	0.9366	0.9479	0.9856	0.0573
C11orf30	0.9811	1.0943	1.0102	0.8567	0.9856	0.0492
LOC729770	0.9501	1.0465	0.9848	0.9609	0.9856	0.0216
LOC1001305	0.9456	1.0354	1.0616	0.8996	0.9856	0.0379
C5	0.9613	1.0922	1.0635	0.8252	0.9856	0.0604
LOC644343	0.9378	1.1488	0.9569	0.8988	0.9856	0.0557
LOC729739	0.9729	1.0675	1.0164	0.8854	0.9856	0.0386
TMEM200A	0.9184	1.0641	1.0550	0.9048	0.9856	0.0428
LOC652346	1.0148	1.0645	1.0063	0.8567	0.9856	0.0448
NXNL2	0.8825	1.1299	1.0041	0.9258	0.9856	0.0543
LOC644055	0.8903	1.0574	1.0215	0.9732	0.9856	0.0361
SAMD9L	0.8805	1.0113	1.1257	0.9249	0.9856	0.0540
MIR410	0.9369	1.0144	0.9861	1.0049	0.9856	0.0173
EIF3H	0.6947	1.5711	1.0310	0.6455	0.9856	0.2131
TSP50	0.9688	1.1025	0.9194	0.9517	0.9856	0.0403
LOC647049	0.9946	1.0590	1.0180	0.8708	0.9856	0.0405
LOC647219	0.8586	1.1121	1.0622	0.9095	0.9856	0.0604
PTK6	0.9119	1.0812	1.0065	0.9428	0.9856	0.0375
LOC1001347	0.9897	1.0191	0.9869	0.9466	0.9856	0.0149
AVP	0.9873	1.1105	0.9088	0.9358	0.9856	0.0447
LOC652606	0.9277	1.0876	0.9425	0.9846	0.9856	0.0361
AKR1CL1	0.8604	1.1543	1.0036	0.9241	0.9856	0.0634
LOC730476	1.0018	1.0776	0.9781	0.8850	0.9856	0.0397
DCX	0.8889	1.0170	1.0233	1.0133	0.9856	0.0323
KIAA1704	0.8861	1.0710	1.0199	0.9655	0.9856	0.0396
TFEC	0.9178	1.0526	1.0062	0.9660	0.9856	0.0287
LOC653877	0.9419	1.0137	0.9339	1.0531	0.9856	0.0288
RSHL1	0.8653	1.0067	1.1171	0.9534	0.9856	0.0527
RASGRF2	0.9639	1.1079	0.9503	0.9204	0.9856	0.0418

WDR49	0.9466	1.1172	0.9655	0.9133	0.9856	0.0452
LOC642173	0.9134	1.0448	1.0854	0.8990	0.9856	0.0467
PRKCG	0.9984	1.1481	0.8779	0.9183	0.9857	0.0596
SSTR1	0.8702	1.0470	1.0629	0.9624	0.9857	0.0444
LOC441505	0.8615	1.1126	1.0400	0.9284	0.9857	0.0561
CXorf52	0.9609	1.0317	0.9917	0.9584	0.9857	0.0171
LOC643322	0.9642	1.0928	0.9982	0.8875	0.9857	0.0425
KCNG4	0.8891	1.1158	0.9967	0.9412	0.9857	0.0486
OR52L1	0.8271	1.0962	0.9991	1.0203	0.9857	0.0568
ENTPD5	0.8828	1.3513	0.8901	0.8186	0.9857	0.1229
LOC643334	0.9036	1.1229	0.9842	0.9321	0.9857	0.0487
LOC389523	0.8838	1.0494	1.0052	1.0044	0.9857	0.0356
LOC654147	1.0109	1.0359	0.9744	0.9216	0.9857	0.0248
LOC650686	0.9917	1.1055	0.9278	0.9178	0.9857	0.0432
GDF3	0.9490	1.1168	0.9332	0.9438	0.9857	0.0438
LOC1001343	0.8970	1.1378	1.0023	0.9057	0.9857	0.0560
INADL	0.9575	1.2013	1.0020	0.7819	0.9857	0.0862
SNORD70	0.8766	1.0590	1.0405	0.9669	0.9857	0.0415
LOC1001300	0.9494	1.1193	0.9985	0.8757	0.9857	0.0512
C8orf45	0.8986	1.1212	1.1074	0.8156	0.9857	0.0762
MIR510	0.8361	1.0850	1.0157	1.0061	0.9857	0.0529
AMIGO1	0.9282	1.0833	1.0202	0.9112	0.9857	0.0404
TH	0.9264	1.0583	1.0235	0.9348	0.9857	0.0327
TNNT1	0.9352	1.1188	1.0544	0.8346	0.9857	0.0631
LOC1001295	1.0111	1.0123	1.0421	0.8776	0.9858	0.0368
TMPRSS11F	0.9300	1.0491	1.0592	0.9047	0.9858	0.0399
GBX2	0.9088	1.1221	1.0191	0.8931	0.9858	0.0534
NSD1	0.9184	1.1960	0.9398	0.8889	0.9858	0.0709
CUX1	0.9156	1.1726	0.9804	0.8745	0.9858	0.0660
LOC152663	1.0428	1.0233	1.0284	0.8486	0.9858	0.0459
LOC728341	0.9465	1.0172	1.0239	0.9555	0.9858	0.0202
IL33	0.9752	1.0521	0.9676	0.9482	0.9858	0.0228
CNGA4	0.9473	1.0498	1.0886	0.8574	0.9858	0.0521
LOC652859	0.9133	0.9984	1.0191	1.0123	0.9858	0.0245
MED21	0.9967	1.0016	1.0213	0.9236	0.9858	0.0214
LOC730411	0.9620	1.0168	1.0305	0.9338	0.9858	0.0228
LOC651059	0.8125	1.1269	1.0826	0.9212	0.9858	0.0727
LOC730924	0.8757	1.0737	1.0740	0.9198	0.9858	0.0516
CXCR1	0.9121	1.2042	0.9962	0.8308	0.9858	0.0803
LOC642072	0.9047	0.9818	1.0897	0.9671	0.9858	0.0384
GJA1	0.8636	1.0549	1.1409	0.8839	0.9858	0.0672
SPRR2B	0.9805	0.9963	1.0683	0.8982	0.9858	0.0349
MIR920	1.0351	0.9869	1.0234	0.8979	0.9858	0.0311
DUSP7	0.8238	1.0861	1.0906	0.9428	0.9858	0.0640
TMEM31	0.8842	1.1154	1.0558	0.8879	0.9858	0.0589
NR0B1	0.9129	1.0325	1.0302	0.9678	0.9858	0.0286

C12orf42	0.9320	1.0896	0.9998	0.9220	0.9858	0.0387
SAMD14	0.9686	1.0891	0.9726	0.9131	0.9858	0.0370
TPST1	0.8358	1.4149	0.9434	0.7492	0.9858	0.1484
LOC647865	0.9318	1.1610	1.0089	0.8416	0.9858	0.0677
LOC1001296	0.9114	1.1475	0.9376	0.9470	0.9858	0.0544
LOC1001295	1.0242	1.0312	1.0043	0.8836	0.9858	0.0345
PHKA2	0.9552	1.2887	0.9541	0.7455	0.9859	0.1123
LOC1001320	0.9559	1.0730	1.0301	0.8845	0.9859	0.0416
ETS2	0.8917	1.1985	1.0264	0.8269	0.9859	0.0822
PCDHGA5	0.9521	1.0321	1.0344	0.9248	0.9859	0.0279
LOC338586	1.0404	1.0115	1.0877	0.8039	0.9859	0.0627
LOC644066	0.8783	0.9586	1.1300	0.9765	0.9859	0.0526
LOC650803	0.9641	1.4113	0.8046	0.7635	0.9859	0.1483
LOC646348	0.9091	1.3249	0.8310	0.8787	0.9859	0.1141
DENND2D	0.9192	1.0753	1.0115	0.9376	0.9859	0.0359
DYTN	0.9575	1.1013	0.9730	0.9118	0.9859	0.0406
PLA2G2E	0.9460	1.1215	0.9947	0.8814	0.9859	0.0508
LOC641765	0.9061	1.1250	0.9638	0.9487	0.9859	0.0480
LOC1001325	0.9128	1.1338	1.0319	0.8652	0.9859	0.0605
LOC1001340	0.9974	1.0556	0.9768	0.9138	0.9859	0.0292
CLEC4D	0.9476	0.9994	1.0491	0.9475	0.9859	0.0244
LOC647274	1.0183	1.0584	0.9766	0.8903	0.9859	0.0360
LOC1001283	0.9384	1.1192	0.9794	0.9067	0.9859	0.0469
CD164	0.9647	1.1371	1.0551	0.7868	0.9859	0.0751
MOCS2	0.6898	1.3115	1.0931	0.8493	0.9859	0.1366
LOC442590	0.9243	1.0738	1.0408	0.9048	0.9859	0.0420
LOC728543	0.9596	1.0649	1.0350	0.8842	0.9859	0.0405
LOC1001314	0.8807	1.2032	0.9480	0.9120	0.9859	0.0737
USP25	1.0155	1.0178	0.9978	0.9127	0.9860	0.0248
MIR384	0.9451	1.1388	0.9438	0.9161	0.9860	0.0514
LOC728853	0.8494	1.1151	1.0882	0.8911	0.9860	0.0676
LOC645602	0.9304	1.0325	1.1050	0.8759	0.9860	0.0513
LOC730032	0.9150	1.1006	0.9539	0.9744	0.9860	0.0401
DSG1	0.8908	1.1488	1.0419	0.8623	0.9860	0.0671
ZNF716	0.9073	1.0794	1.0380	0.9191	0.9860	0.0429
TRIP6	0.9254	1.1666	1.0567	0.7951	0.9860	0.0805
FIGLA	0.9749	1.1144	0.9437	0.9109	0.9860	0.0448
RANBP3L	1.0358	1.1790	0.9442	0.7849	0.9860	0.0826
CSDC2	0.9466	1.1250	0.9665	0.9058	0.9860	0.0480
LOC1001293	0.9097	1.1120	1.0694	0.8529	0.9860	0.0622
MAGI3	0.9207	1.0634	1.0581	0.9017	0.9860	0.0433
DEFB109P1	0.8996	1.1036	1.0416	0.8991	0.9860	0.0516
KRTAP20-2	0.9138	1.0383	1.0377	0.9542	0.9860	0.0311
LOC1001334	0.9313	1.0190	1.0481	0.9456	0.9860	0.0282
WNT1	0.8518	1.0925	1.0822	0.9175	0.9860	0.0601
HRH2	1.0251	0.9847	1.0048	0.9294	0.9860	0.0206

LOC646191	0.9037	1.0634	0.9853	0.9918	0.9860	0.0327
CLSTN2	0.9818	1.0987	1.0238	0.8398	0.9860	0.0544
LOC340221	0.9910	1.0529	1.0360	0.8643	0.9860	0.0426
LOC1001300	1.0074	1.0103	0.9634	0.9631	0.9860	0.0132
WFDC8	0.9637	1.0738	1.0249	0.8817	0.9860	0.0414
LOC652322	0.7446	1.2529	1.1660	0.7806	0.9860	0.1304
DPEP1	0.9289	1.1637	0.9870	0.8646	0.9860	0.0643
CICK0721Q.1	0.9232	1.0857	1.0278	0.9075	0.9860	0.0426
ERCC1	0.8078	1.3037	1.0639	0.7687	0.9861	0.1245
PSMB8	0.9275	1.0634	1.0583	0.8951	0.9861	0.0437
BBS9	0.9392	1.1939	1.0208	0.7902	0.9861	0.0841
LOC92973	0.9979	1.1571	0.9539	0.8354	0.9861	0.0665
KRTAP19-1	1.0257	1.0394	1.0304	0.8487	0.9861	0.0459
ACAD9	0.9383	1.3942	0.9260	0.6857	0.9861	0.1480
KRT38	0.9627	1.0391	0.9957	0.9468	0.9861	0.0204
LOC729076	0.8372	1.2616	0.9986	0.8470	0.9861	0.0990
LOC1001317	1.0174	1.0070	1.0197	0.9001	0.9861	0.0288
CIB4	1.1047	0.9474	1.0282	0.8640	0.9861	0.0518
LOC646584	0.9169	1.0731	0.9424	1.0119	0.9861	0.0353
TTC37	0.8637	1.2838	1.0046	0.7922	0.9861	0.1086
LOC643194	0.9261	1.0966	1.0596	0.8620	0.9861	0.0552
LOC1001341	0.8364	1.1369	1.0660	0.9052	0.9861	0.0696
LOC652155	0.9235	1.1066	0.9999	0.9144	0.9861	0.0445
ZNF836	0.9008	1.0907	1.0327	0.9202	0.9861	0.0454
LOC440243	0.9296	1.0476	1.0541	0.9131	0.9861	0.0375
EFCAB5	0.9216	1.0362	1.0204	0.9664	0.9861	0.0262
LOC652993	1.0226	1.0765	0.9796	0.8658	0.9861	0.0447
CXorf59	0.9700	1.1658	0.9471	0.8617	0.9861	0.0643
LOC402538	1.0609	1.0014	0.9279	0.9542	0.9861	0.0292
RBMY1A1	0.9414	1.0193	1.0500	0.9338	0.9861	0.0287
OR5AR1	1.0101	1.0526	0.9809	0.9011	0.9862	0.0320
SNX21	0.9832	1.0226	1.0250	0.9139	0.9862	0.0259
LOC644328	0.9591	1.1000	0.9827	0.9029	0.9862	0.0415
GTF2IRD1	0.9689	1.2622	0.9443	0.7693	0.9862	0.1022
RKHD1	0.8744	1.0085	1.0479	1.0139	0.9862	0.0383
AXL	0.9601	1.1194	1.0085	0.8567	0.9862	0.0545
ABCC13	0.9561	1.0513	1.0141	0.9232	0.9862	0.0287
ITIH3	0.9563	1.0950	0.9586	0.9348	0.9862	0.0367
LOC1001292	0.9500	1.1181	1.0158	0.8609	0.9862	0.0542
LOC1001319	0.9203	1.1017	1.0939	0.8289	0.9862	0.0671
HOXB9	1.0184	1.0314	1.0049	0.8901	0.9862	0.0325
LOC650311	0.9639	0.9994	1.0416	0.9399	0.9862	0.0221
LOC1001314	0.7384	1.3068	1.1303	0.7694	0.9862	0.1390
VAT1L	0.9311	1.1314	0.9846	0.8977	0.9862	0.0516
LOC1000342	0.9394	1.0571	1.0080	0.9403	0.9862	0.0286
LOC644785	1.0139	1.0881	0.9443	0.8985	0.9862	0.0414

LOC1001318	0.9174	1.1216	1.0029	0.9029	0.9862	0.0502
TCP11	0.8744	1.0305	1.0570	0.9829	0.9862	0.0403
LOC653204	0.9661	1.0781	1.0261	0.8745	0.9862	0.0437
ROPN1B	0.8946	1.0891	1.0568	0.9043	0.9862	0.0506
OR2T3	0.9280	1.1944	0.9387	0.8837	0.9862	0.0704
LOC643670	0.9121	1.0237	1.0641	0.9450	0.9862	0.0349
VSIG1	0.9540	1.0878	1.0264	0.8767	0.9862	0.0456
LOC392979	0.9184	1.0681	0.9825	0.9759	0.9862	0.0309
MGC16703	0.8948	1.3596	0.8825	0.8081	0.9862	0.1259
TFAP2A	0.9016	1.0958	1.1099	0.8377	0.9862	0.0686
ACTL8	0.9737	1.0020	1.0505	0.9188	0.9862	0.0275
GANC	0.9410	1.1186	0.9575	0.9279	0.9862	0.0445
MMD2	0.8760	1.1012	0.9382	1.0296	0.9862	0.0496
TMEM206	0.9941	1.1392	1.0327	0.7789	0.9863	0.0756
TACC2	0.9174	1.3570	0.9328	0.7378	0.9863	0.1313
ZNF650	0.8008	1.2813	0.9763	0.8867	0.9863	0.1047
CHST4	1.0344	1.0751	0.9392	0.8963	0.9863	0.0413
LOC649723	0.9292	1.1309	0.9639	0.9210	0.9863	0.0491
LOC647055	0.9001	1.0191	1.1357	0.8901	0.9863	0.0578
LOC129870	0.9959	1.0518	1.0678	0.8296	0.9863	0.0545
CHMP4B	0.8981	1.3039	1.0453	0.6977	0.9863	0.1276
SORCS3	1.0015	1.0380	1.0276	0.8779	0.9863	0.0369
NEK9	0.8588	1.1737	0.9557	0.9568	0.9863	0.0666
FAM98B	0.9330	1.1917	0.9327	0.8877	0.9863	0.0693
LOC1001342	0.9815	1.0772	0.9303	0.9562	0.9863	0.0320
CAPN6	0.9901	1.1452	0.9595	0.8503	0.9863	0.0609
SNORD3C	0.9188	1.2932	0.8477	0.8855	0.9863	0.1033
LOC1001282	0.9267	1.0136	1.0129	0.9919	0.9863	0.0205
DTNB	1.1046	1.0888	0.9942	0.7575	0.9863	0.0801
TYRP1	0.8861	1.1733	1.0677	0.8180	0.9863	0.0816
FILIP1L	0.9408	1.0290	0.9883	0.9871	0.9863	0.0180
LOC644789	0.9458	1.0515	0.9592	0.9887	0.9863	0.0235
LOC643138	1.0188	1.1443	0.8510	0.9311	0.9863	0.0628
ZRANB1	0.8552	1.2603	1.0841	0.7456	0.9863	0.1154
LOC401847	0.9452	1.0763	1.0742	0.8496	0.9863	0.0549
LOC641995	0.9296	1.0150	1.0609	0.9398	0.9863	0.0313
KIAA2026	0.9246	1.2061	0.9650	0.8495	0.9863	0.0771
LOC400299	0.9483	1.1184	1.0192	0.8593	0.9863	0.0549
LHX3	0.9698	1.0647	1.0091	0.9017	0.9863	0.0343
ZCCHC13	0.9863	1.0393	1.0273	0.8923	0.9863	0.0333
LOC644264	0.8786	1.1926	1.0020	0.8720	0.9863	0.0750
LOC1001342	0.8832	1.1600	0.9596	0.9425	0.9863	0.0602
LOC1001318	1.0011	1.0167	1.0461	0.8815	0.9863	0.0362
VSX1	0.9921	1.0719	0.9692	0.9121	0.9863	0.0331
HOXA9	0.8817	1.1070	0.9425	1.0141	0.9863	0.0485
RIPPLY1	0.9980	1.0190	1.0744	0.8539	0.9863	0.0470

LOC646335	0.9575	1.0418	0.9948	0.9512	0.9863	0.0208
LOC648840	0.9661	0.9986	1.0253	0.9553	0.9863	0.0159
C16orf81	0.8804	1.0634	0.9757	1.0259	0.9863	0.0396
LOC649374	0.9457	0.9307	1.0651	1.0038	0.9863	0.0306
BRD7P2	0.8820	1.4418	0.8796	0.7419	0.9863	0.1553
GDPD4	0.9419	1.0953	1.0661	0.8421	0.9863	0.0585
LOC653184	0.9732	1.0167	1.0348	0.9207	0.9864	0.0254
LOC650274	0.9278	1.1197	0.9579	0.9400	0.9864	0.0449
REXO1L2P	0.9659	1.0971	1.0115	0.8710	0.9864	0.0471
LOC728086	0.8806	1.1037	1.0419	0.9193	0.9864	0.0521
DHX58	0.9045	1.0786	1.0875	0.8749	0.9864	0.0562
RGL2	1.0026	1.1163	0.9706	0.8560	0.9864	0.0535
LRRC4B	1.0215	1.0044	0.9249	0.9947	0.9864	0.0212
ARL6IP4	0.8682	1.3229	0.9630	0.7915	0.9864	0.1175
LOC642509	0.9703	1.1360	0.9465	0.8927	0.9864	0.0525
LOC647080	0.9578	1.0925	1.1159	0.7793	0.9864	0.0773
LOC648680	1.0829	1.0964	0.9496	0.8168	0.9864	0.0655
VGLL1	0.9409	1.0522	1.0918	0.8607	0.9864	0.0527
RGSL2	0.8207	1.1984	1.0934	0.8331	0.9864	0.0946
PCF11	1.0386	1.2098	0.9056	0.7916	0.9864	0.0900
LOC139735	1.0551	1.0367	0.9288	0.9250	0.9864	0.0346
KCNA5	0.9420	1.0894	1.0412	0.8729	0.9864	0.0487
LOC1001323	0.9086	1.1406	1.0244	0.8720	0.9864	0.0608
ABHD9	0.9315	0.9587	1.0424	1.0130	0.9864	0.0252
FGF13	0.9714	1.0638	1.0107	0.8998	0.9864	0.0345
LOC732446	0.9455	1.0890	1.0418	0.8694	0.9864	0.0491
WDSOF1	0.9409	1.0881	0.9843	0.9324	0.9864	0.0357
TTTY21	1.0114	1.0908	1.0044	0.8392	0.9864	0.0528
MIR548G	1.0375	1.0777	0.9619	0.8687	0.9864	0.0460
LOC730163	0.9037	1.0348	1.0874	0.9200	0.9865	0.0445
DOC2B	1.0021	1.0629	0.9688	0.9119	0.9865	0.0316
CCDC122	0.9833	1.0912	0.9766	0.8948	0.9865	0.0403
HIST1H4D	0.9045	1.0499	1.0177	0.9738	0.9865	0.0314
LOC728687	0.8876	1.0907	1.0749	0.8926	0.9865	0.0557
LOC653544	0.9270	1.0797	1.0892	0.8500	0.9865	0.0588
LOC161527	0.9278	1.1197	1.0005	0.8980	0.9865	0.0493
ERCC-00134	0.9466	1.1053	0.9720	0.9220	0.9865	0.0409
GPR1	0.9664	1.1205	1.0459	0.8132	0.9865	0.0658
PVRL1	0.9513	1.0939	1.0302	0.8706	0.9865	0.0484
DOPEY1	0.9228	1.2238	1.0057	0.7936	0.9865	0.0904
TDRD12	0.9656	1.0239	1.0416	0.9150	0.9865	0.0288
CP	1.0017	1.0833	1.0359	0.8251	0.9865	0.0563
LOC647873	0.9344	1.1622	0.8906	0.9588	0.9865	0.0602
ULBP3	0.8746	1.1108	0.9366	1.0241	0.9865	0.0515
LOC149478	0.9615	1.0559	1.0297	0.8989	0.9865	0.0353
LOC652276	0.8673	1.1361	0.9946	0.9481	0.9865	0.0564

MIR938	0.8169	1.0573	1.1106	0.9612	0.9865	0.0644
CXorf42	0.9579	1.0934	0.9449	0.9498	0.9865	0.0357
NKX2-6	0.9108	1.1697	0.8987	0.9669	0.9865	0.0628
CYorf14	0.8818	1.0706	1.0073	0.9864	0.9865	0.0392
LOC652679	1.0033	1.1009	0.9609	0.8810	0.9865	0.0458
CCDC155	0.9234	1.0959	1.0602	0.8667	0.9865	0.0546
IAPP	1.0186	0.9728	0.9940	0.9609	0.9865	0.0127
TCF25	0.8673	1.4269	0.9924	0.6596	0.9866	0.1620
CACNA2D3	0.9771	1.0582	1.0354	0.8756	0.9866	0.0408
BHLHB2	0.8523	1.3782	0.8698	0.8459	0.9866	0.1306
C6orf205	0.9089	1.0695	1.0506	0.9172	0.9866	0.0427
NCCRP1	0.9409	1.0335	1.0433	0.9286	0.9866	0.0301
MIR645	0.9618	1.1773	0.9559	0.8512	0.9866	0.0685
LOC729885	0.9524	1.1324	1.0338	0.8276	0.9866	0.0645
MIR194-2	0.9907	1.1113	1.0619	0.7824	0.9866	0.0724
LOC442028	0.9581	1.0470	1.0518	0.8894	0.9866	0.0389
LOC343384	0.9330	1.0417	1.0012	0.9704	0.9866	0.0231
SNORA66	0.8936	1.0944	1.0687	0.8896	0.9866	0.0551
C12orf23	0.9274	1.1404	1.1169	0.7617	0.9866	0.0888
SCRIB	0.9162	1.2420	1.0001	0.7880	0.9866	0.0957
ANUBL1	0.7807	1.1303	1.0428	0.9926	0.9866	0.0743
PCDHGA6	0.9866	1.0809	0.9854	0.8935	0.9866	0.0382
PLS1	0.8621	1.2035	1.1480	0.7328	0.9866	0.1129
SFTPA1	0.9255	1.0153	1.1062	0.8994	0.9866	0.0470
FOXA2	0.9606	1.0070	1.0357	0.9430	0.9866	0.0212
ARRDC3	0.8678	1.3123	1.0512	0.7151	0.9866	0.1285
EXD1	0.9614	1.0717	1.0222	0.8912	0.9866	0.0390
LOC648133	1.0153	1.0230	0.9721	0.9361	0.9866	0.0202
HBA2	0.9743	1.0255	0.9460	1.0007	0.9866	0.0171
LOC391475	0.9326	1.0687	0.9956	0.9497	0.9866	0.0304
LOC1001309	0.9535	1.1338	0.9193	0.9398	0.9866	0.0496
LOC1001329	0.9238	1.1215	0.9422	0.9590	0.9866	0.0455
LOC648943	0.9494	1.0492	0.9890	0.9589	0.9866	0.0225
KRT18P30	0.9597	1.0616	1.0965	0.8287	0.9866	0.0601
TTTY11	0.8564	1.2110	0.9577	0.9214	0.9866	0.0777
GRIK2	0.9709	1.0653	0.9864	0.9239	0.9866	0.0294
LILRA3	0.9443	1.0241	1.0453	0.9328	0.9866	0.0282
RPS6KL1	0.8640	1.1294	1.0500	0.9032	0.9866	0.0622
MOBKL2B	0.9210	1.0361	1.0440	0.9454	0.9866	0.0313
OTUD7A	0.9157	1.1138	1.0660	0.8511	0.9866	0.0618
LOC1001317	0.8917	1.0943	1.0931	0.8674	0.9866	0.0620
MIR892B	0.8625	1.0548	1.0736	0.9557	0.9867	0.0488
IFNE1	1.0055	1.0744	0.9974	0.8693	0.9867	0.0428
ZP4	0.9115	1.1073	0.9505	0.9773	0.9867	0.0424
LOC647460	0.9355	1.0613	1.0313	0.9185	0.9867	0.0351
C3orf20	0.9907	1.0399	1.0599	0.8562	0.9867	0.0459

CLDN5	0.9592	1.0067	1.0520	0.9288	0.9867	0.0270
LOC388458	0.9478	1.1659	0.9965	0.8365	0.9867	0.0685
FLJ42102	0.9727	1.0692	0.9839	0.9209	0.9867	0.0307
LOC642005	0.9981	1.1570	0.9941	0.7974	0.9867	0.0736
LOC1001289	0.9276	1.1138	1.0271	0.8781	0.9867	0.0525
KIAA1271	0.8590	1.1718	1.0323	0.8836	0.9867	0.0726
KCNC2	0.9583	1.0602	1.0409	0.8873	0.9867	0.0398
LOC1001279	0.9935	1.0910	0.9885	0.8737	0.9867	0.0445
LOC653162	0.9765	1.1290	0.9138	0.9275	0.9867	0.0493
LOC1001341	0.8996	1.1076	1.0256	0.9139	0.9867	0.0492
PLXNA4B	0.9192	1.0334	1.0022	0.9920	0.9867	0.0242
LOC730805	0.9351	1.0543	0.9652	0.9922	0.9867	0.0254
LOC645545	1.0248	0.9496	1.0907	0.8816	0.9867	0.0454
C20orf103	0.9052	1.0892	1.0172	0.9352	0.9867	0.0416
LOC650933	0.8666	1.1768	1.0362	0.8672	0.9867	0.0749
ARRDC1	0.9873	1.2241	0.9729	0.7625	0.9867	0.0943
LOC731909	0.8965	1.0903	1.0346	0.9254	0.9867	0.0456
NUDT9P1	0.9146	1.0174	1.0706	0.9442	0.9867	0.0353
LOC729348	0.8564	1.2663	0.8908	0.9333	0.9867	0.0945
PAX5	0.9587	1.0389	1.0850	0.8642	0.9867	0.0485
ALMS1	1.0439	1.2269	0.8313	0.8447	0.9867	0.0937
LY96	0.9942	1.0647	0.9845	0.9034	0.9867	0.0330
PCDHA11	0.9569	1.1446	0.9255	0.9198	0.9867	0.0533
BRUNOL4	0.9352	1.0988	1.0027	0.9101	0.9867	0.0422
SPACA5B	0.9124	1.0876	1.0129	0.9339	0.9867	0.0400
KMO	0.9880	1.0075	0.9851	0.9664	0.9867	0.0084
NCAM1	0.9545	1.0807	0.9838	0.9279	0.9867	0.0334
CCDC113	0.9842	1.1165	1.0593	0.7869	0.9867	0.0719
HRCT1	0.9723	1.0476	0.9837	0.9433	0.9867	0.0220
LOC653968	0.9151	1.0768	1.0646	0.8904	0.9867	0.0488
LOC339804	0.9720	1.1358	0.9229	0.9163	0.9867	0.0512
LOC731308	1.0137	1.0145	1.0801	0.8388	0.9867	0.0517
SPATC1	0.9403	1.1229	0.9660	0.9178	0.9868	0.0464
MIR548B	0.9003	1.1483	0.9695	0.9289	0.9868	0.0557
MIR224	0.9566	1.0326	1.0443	0.9135	0.9868	0.0312
TNRC6C	0.8969	1.1045	1.0639	0.8817	0.9868	0.0570
L3MBTL4	0.8643	1.1518	1.0318	0.8993	0.9868	0.0658
LOC151234	0.9364	1.0604	1.0834	0.8669	0.9868	0.0513
EPB41L1	0.9645	1.0957	1.0052	0.8817	0.9868	0.0445
LOC649385	0.9304	1.1197	0.8579	1.0390	0.9868	0.0579
CMAH	0.9499	1.1402	0.9619	0.8950	0.9868	0.0532
MYEF2	0.9113	1.0144	0.9982	1.0231	0.9868	0.0257
MT1H	0.9559	1.0725	1.0283	0.8905	0.9868	0.0401
LOC643085	0.9525	1.1295	1.0352	0.8300	0.9868	0.0636
LOC728045	0.9763	1.0422	1.0866	0.8421	0.9868	0.0533
LOC650392	0.9338	0.9992	1.0479	0.9663	0.9868	0.0243

EPS8L3	0.9753	1.0107	1.0182	0.9431	0.9868	0.0173
LOC340228	1.0444	1.0947	0.9899	0.8182	0.9868	0.0601
LOC644342	0.9121	1.0152	1.0948	0.9251	0.9868	0.0427
LOC652355	0.8908	1.1118	1.0192	0.9255	0.9868	0.0497
HIRA	0.9129	1.2199	0.9638	0.8508	0.9868	0.0810
FXYD7	1.0732	0.9199	1.0363	0.9178	0.9868	0.0399
LOC644802	0.9102	1.1035	0.9463	0.9873	0.9868	0.0420
LOC1001284	0.9909	1.0718	1.0279	0.8566	0.9868	0.0464
HCG22	0.9970	0.9236	1.0080	1.0187	0.9868	0.0215
HTR2B	0.9590	0.9797	1.0897	0.9189	0.9868	0.0365
LOC644717	1.0154	1.0971	0.9561	0.8787	0.9868	0.0462
KRTAP13-1	0.9305	1.0764	1.0255	0.9150	0.9868	0.0386
LGR5	0.9511	1.1509	1.0093	0.8361	0.9868	0.0655
LOC653735	0.9630	1.0380	1.0816	0.8647	0.9868	0.0475
AVPR1A	0.9032	1.0980	0.9961	0.9500	0.9868	0.0416
LOC401537	0.7979	1.3939	1.0217	0.7338	0.9868	0.1491
DNHL1	0.9840	1.0668	0.9378	0.9587	0.9869	0.0283
SLC22A9	0.9811	1.0580	1.0499	0.8585	0.9869	0.0461
LOC642514	0.9752	1.1093	1.0499	0.8131	0.9869	0.0641
LOC651807	0.9113	1.1122	0.9882	0.9358	0.9869	0.0447
KLKB1	1.0260	1.0793	0.9316	0.9106	0.9869	0.0397
MIR450B	0.9177	1.0423	1.0834	0.9041	0.9869	0.0448
LOC646365	0.9431	1.0963	1.0172	0.8909	0.9869	0.0447
CAB39	0.9483	1.2757	0.9101	0.8135	0.9869	0.1004
LOC645004	0.8910	1.1031	1.0767	0.8767	0.9869	0.0598
TAS1R2	0.9888	0.9946	1.0381	0.9260	0.9869	0.0231
RARRES1	0.9145	1.1006	1.0267	0.9058	0.9869	0.0469
CSPG5	1.1033	1.2326	0.9244	0.6873	0.9869	0.1182
LOC642110	0.9797	1.0838	0.9049	0.9791	0.9869	0.0368
LOC389895	1.0771	1.0422	0.9567	0.8715	0.9869	0.0460
ARHGAP8	0.9366	1.1294	1.0144	0.8672	0.9869	0.0562
KANK1	0.9505	1.0601	1.0255	0.9115	0.9869	0.0340
RGMB	0.8969	1.1640	1.0291	0.8576	0.9869	0.0695
MFAP3	0.7996	1.3335	1.0249	0.7896	0.9869	0.1277
LOC643132	0.9699	0.9865	1.0007	0.9906	0.9869	0.0064
LOC1001318	0.9952	0.9505	1.0376	0.9644	0.9869	0.0193
LOC729479	0.9915	1.0411	0.9703	0.9448	0.9869	0.0204
MIR330	0.9190	1.0190	1.0965	0.9132	0.9869	0.0439
LOC646778	0.9693	1.1507	0.9745	0.8532	0.9869	0.0613
OR8A1	0.8932	1.1636	1.0102	0.8808	0.9869	0.0657
MYST2	0.9360	1.2402	0.9580	0.8137	0.9870	0.0902
FCGR2C	0.9852	0.9991	0.9834	0.9801	0.9870	0.0042
LOC730063	0.8932	1.0780	1.0009	0.9758	0.9870	0.0381
LOC1001285	0.9103	1.0786	1.0518	0.9072	0.9870	0.0455
SLC9A3	0.8847	1.0359	1.0701	0.9572	0.9870	0.0415
IL34	0.8342	1.0576	1.0820	0.9741	0.9870	0.0559

LOC730376	0.9007	1.1929	0.9875	0.8668	0.9870	0.0732
SNORA36C	0.9554	1.1219	1.0378	0.8327	0.9870	0.0616
MUC13	0.9498	1.0872	1.0215	0.8895	0.9870	0.0429
ZNF642	1.0221	1.1108	0.9603	0.8547	0.9870	0.0538
LOC1001318	0.8509	1.0652	1.0995	0.9323	0.9870	0.0579
KCNJ12	0.9463	1.0426	0.9980	0.9610	0.9870	0.0215
SNORA58	0.8152	1.1547	1.0472	0.9309	0.9870	0.0733
LOC729681	0.9542	1.0271	1.0345	0.9322	0.9870	0.0257
TMEM16A	0.9932	1.0705	0.9729	0.9114	0.9870	0.0328
LOC650668	0.9337	1.0915	1.0345	0.8883	0.9870	0.0463
HSPBAP1	0.8894	1.3106	0.9144	0.8336	0.9870	0.1092
FLJ42291	1.0159	0.9933	1.0666	0.8723	0.9870	0.0412
MIR1261	1.0090	1.0405	0.9805	0.9181	0.9870	0.0260
ERBB4	0.9377	1.1454	0.9927	0.8723	0.9870	0.0583
LOC730038	0.9168	1.0937	1.0380	0.8995	0.9870	0.0471
LOC646096	1.0165	1.0733	0.9392	0.9190	0.9870	0.0356
LOC400963	0.9207	1.1481	0.9411	0.9383	0.9870	0.0539
LOC1001342	1.0161	1.0053	1.0438	0.8829	0.9870	0.0356
KRTAP5-1	0.9159	1.1757	1.0599	0.7967	0.9871	0.0827
LOC1001289	0.9408	1.0943	1.0113	0.9018	0.9871	0.0423
FGF20	0.9985	1.0194	1.0119	0.9186	0.9871	0.0232
LOC649604	0.9555	1.2220	0.9666	0.8041	0.9871	0.0866
FAM90A18	1.0447	1.0418	0.9549	0.9069	0.9871	0.0339
LOC649489	0.9393	1.0147	1.0051	0.9892	0.9871	0.0168
LOC1001312	0.9472	1.0598	1.0157	0.9256	0.9871	0.0309
GPR128	0.8877	1.1206	1.0482	0.8918	0.9871	0.0581
SYNE1	0.9368	1.0584	1.0407	0.9124	0.9871	0.0366
CCRL1	0.9744	1.0812	1.0243	0.8685	0.9871	0.0451
LOC652557	0.9843	1.0146	1.0115	0.9379	0.9871	0.0177
LOC651565	0.8904	1.1880	0.9998	0.8703	0.9871	0.0728
TRIM17	0.9508	1.1559	0.9560	0.8857	0.9871	0.0585
LOC1001343	1.0416	1.0747	0.9541	0.8780	0.9871	0.0444
LOC650330	0.9059	1.1112	0.9649	0.9664	0.9871	0.0437
LOC1001341	1.0168	1.0239	1.0529	0.8548	0.9871	0.0448
LOC388578	0.9693	1.0273	1.1146	0.8374	0.9871	0.0582
TNP1	0.9039	1.1639	1.0233	0.8573	0.9871	0.0685
LOC440258	0.8835	1.0576	1.0223	0.9850	0.9871	0.0376
VAV1	0.8719	1.1635	0.9548	0.9582	0.9871	0.0621
MIR653	0.9658	1.0242	1.1011	0.8574	0.9871	0.0514
CYP4Z2P	0.8986	1.0967	1.0403	0.9130	0.9871	0.0484
LOC647288	0.8851	1.1255	1.0665	0.8715	0.9871	0.0640
LOC728802	0.9339	1.0704	1.0138	0.9305	0.9871	0.0338
LOC646630	0.9779	1.2389	1.0368	0.6950	0.9871	0.1123
LOC1001295	0.9520	1.0641	1.0518	0.8808	0.9872	0.0434
GABRB2	0.9863	1.0789	1.0248	0.8585	0.9872	0.0469
SPSB1	0.8980	1.0783	0.9802	0.9922	0.9872	0.0369

PCCB	0.8091	1.3396	1.0257	0.7743	0.9872	0.1300
TSPAN3	0.6587	1.4317	1.1290	0.7293	0.9872	0.1808
TSPAN18	0.9410	1.1220	0.9612	0.9244	0.9872	0.0456
RPL31	0.8442	1.0949	1.1924	0.8172	0.9872	0.0927
LOC653635	0.8499	1.2429	0.9560	0.8999	0.9872	0.0880
SCARF1	0.9757	1.0616	1.0232	0.8882	0.9872	0.0374
MUC3B	0.9291	1.0385	1.0669	0.9142	0.9872	0.0384
LOC645446	0.8786	1.0501	1.1078	0.9122	0.9872	0.0547
LOC641989	0.9935	1.1221	0.9456	0.8875	0.9872	0.0499
SEC14L3	0.8530	1.0405	1.0957	0.9596	0.9872	0.0527
MYBPHL	1.0132	1.0685	0.9594	0.9076	0.9872	0.0346
GPR152	0.9889	1.2180	0.9344	0.8074	0.9872	0.0858
LOC647529	0.9000	1.0815	1.0770	0.8902	0.9872	0.0532
LOC731366	1.0313	1.0418	0.9769	0.8987	0.9872	0.0327
LCE1E	0.9599	1.0457	1.0408	0.9024	0.9872	0.0344
LEP	0.9213	1.1180	1.0025	0.9069	0.9872	0.0484
FTH1	0.8883	1.1253	1.2217	0.7136	0.9872	0.1150
LOC647537	0.9299	1.0207	1.0443	0.9540	0.9872	0.0270
LOC647012	1.0032	1.1229	0.9581	0.8647	0.9872	0.0536
MIR548K	0.9065	1.1179	0.9970	0.9275	0.9872	0.0477
FAM108A2	0.8051	1.4797	0.9391	0.7249	0.9872	0.1700
NPBWR2	1.0141	1.0788	0.9334	0.9226	0.9872	0.0367
LOC642295	0.9494	1.0284	1.0707	0.9005	0.9872	0.0383
MVP	0.9086	1.2261	1.0315	0.7827	0.9872	0.0944
LGALS9	0.9405	1.0692	0.9699	0.9694	0.9872	0.0282
OMA1	0.8233	1.3558	1.0593	0.7107	0.9872	0.1427
SLC2A7	0.8999	1.0594	0.9607	1.0291	0.9873	0.0357
LOC648745	0.9850	1.0566	1.0321	0.8754	0.9873	0.0401
SELPLG	1.0572	1.0894	0.9231	0.8794	0.9873	0.0509
MIR107	0.9399	1.0477	1.0918	0.8697	0.9873	0.0505
LOC644197	0.9719	1.1706	0.9110	0.8955	0.9873	0.0633
MIR363	1.0204	1.0554	0.9774	0.8959	0.9873	0.0344
USP17L5	0.8855	1.0371	0.9659	1.0607	0.9873	0.0395
BAI1	0.9209	1.0642	1.0296	0.9344	0.9873	0.0353
LOC642237	0.9777	0.9918	1.0705	0.9091	0.9873	0.0331
RNU4-2	0.6984	1.1963	1.2175	0.8369	0.9873	0.1300
LOC401354	0.8633	1.1837	1.0340	0.8682	0.9873	0.0765
LOC642305	0.9368	1.0686	1.0214	0.9224	0.9873	0.0348
LOC345537	1.0389	1.0613	0.9758	0.8733	0.9873	0.0421
LOC1001296	0.9075	1.1199	0.9998	0.9220	0.9873	0.0486
LOC1001343	0.9269	1.1409	0.9449	0.9365	0.9873	0.0513
TTPA	0.9004	1.0408	1.0856	0.9224	0.9873	0.0450
LOC345630	0.9883	1.0854	0.9030	0.9725	0.9873	0.0376
SNORD105	0.9632	0.9943	1.1227	0.8690	0.9873	0.0524
RHBDL1	0.9536	0.9755	1.1350	0.8851	0.9873	0.0529
CDH4	0.8844	1.0404	1.0853	0.9391	0.9873	0.0460

EMR1	0.9291	1.1063	1.0012	0.9126	0.9873	0.0441
CDC14A	0.9221	1.1355	1.0090	0.8827	0.9873	0.0560
C4orf51	0.9194	0.9529	1.1447	0.9323	0.9873	0.0529
TTC35	0.8260	1.1673	1.2051	0.7510	0.9873	0.1161
PGM5	0.9544	1.0243	1.0483	0.9223	0.9873	0.0294
LOC1001340	0.9637	1.0095	1.0253	0.9509	0.9873	0.0178
GIMAP7	0.8892	1.1461	1.0122	0.9019	0.9874	0.0597
LOC653609	1.1423	1.0104	0.9130	0.8838	0.9874	0.0583
ERCC-00098	0.9660	1.0750	0.9504	0.9580	0.9874	0.0294
KLF14	0.9290	1.0594	1.0327	0.9283	0.9874	0.0343
C11orf57	1.0272	1.1404	0.9747	0.8071	0.9874	0.0693
PDGFRB	0.9492	1.1424	1.0283	0.8295	0.9874	0.0659
LOC649092	1.0121	1.0654	0.9822	0.8898	0.9874	0.0368
AMDHD1	1.0144	1.1516	0.8529	0.9306	0.9874	0.0639
CXCL6	0.9355	1.0615	1.0258	0.9267	0.9874	0.0333
UNQ9433	0.9633	1.0564	0.9607	0.9691	0.9874	0.0231
LOC647513	0.9191	1.1639	1.0437	0.8229	0.9874	0.0742
B3GALT3	0.9672	1.0949	0.9944	0.8930	0.9874	0.0418
PRDM4	0.8337	1.4020	0.9171	0.7968	0.9874	0.1405
DEPDC7	0.8858	1.0794	1.0794	0.9051	0.9874	0.0532
KIAA0889	0.9095	1.0849	1.0139	0.9413	0.9874	0.0392
LOC1001334	0.9057	1.1433	0.9454	0.9553	0.9874	0.0530
LOC652656	0.9415	1.0261	1.0995	0.8826	0.9874	0.0476
GALC	0.9289	1.0468	1.0230	0.9509	0.9874	0.0282
LOC1001334	1.0629	0.9453	0.9947	0.9467	0.9874	0.0277
ACAT2	1.0280	1.2042	0.9828	0.7347	0.9874	0.0968
LOC654264	0.9516	1.0113	1.0126	0.9742	0.9874	0.0149
VGLL3	0.8836	1.1758	1.0065	0.8838	0.9874	0.0692
LOC653165	0.9316	1.0815	1.0232	0.9133	0.9874	0.0395
LOC1001314	0.9323	1.0434	1.0294	0.9446	0.9874	0.0285
LOC645981	1.0244	0.9814	1.0200	0.9240	0.9874	0.0232
PRNT	0.9421	1.0529	1.1067	0.8481	0.9875	0.0577
LOC653140	0.9013	1.0736	1.1047	0.8702	0.9875	0.0594
LOC654187	1.0348	1.0533	0.9697	0.8922	0.9875	0.0365
SLC22A16	0.9479	1.0690	0.9534	0.9796	0.9875	0.0281
ERMAP	0.9733	1.1448	1.0087	0.8231	0.9875	0.0661
LOC646155	0.9627	1.0147	1.0876	0.8851	0.9875	0.0427
LOC730686	0.9425	1.1062	1.0161	0.8851	0.9875	0.0478
XKRY2	0.9031	1.0881	0.9988	0.9599	0.9875	0.0389
LOC339240	0.9610	1.0523	1.0374	0.8993	0.9875	0.0356
LOC1001312	0.9721	1.0830	1.0646	0.8303	0.9875	0.0577
SULT1C3	1.0525	1.0691	0.9695	0.8588	0.9875	0.0481
LOC650708	0.9362	1.0128	1.0564	0.9445	0.9875	0.0287
LOC728291	0.9574	1.0962	1.0215	0.8749	0.9875	0.0471
ANKRD42	0.9258	1.1126	1.0034	0.9083	0.9875	0.0465
SLC28A3	0.9010	1.0526	1.0212	0.9753	0.9875	0.0329

LOC1001347	0.9467	1.0872	1.1039	0.8122	0.9875	0.0682
LOC648148	0.8819	1.0651	1.0480	0.9551	0.9875	0.0427
CRP	0.9410	1.1196	0.9512	0.9384	0.9875	0.0441
MGC40069	0.9234	0.8934	1.1535	0.9799	0.9875	0.0582
C2orf71	0.9243	1.1220	1.0200	0.8838	0.9875	0.0531
UNC5B	1.0104	1.0883	0.9287	0.9228	0.9875	0.0391
LOC1001311	1.0248	0.9628	1.0391	0.9235	0.9876	0.0270
KRTAP12-4	0.9420	1.0827	1.0193	0.9062	0.9876	0.0395
LOC648604	1.0046	1.1913	0.8736	0.8807	0.9876	0.0743
LOC1001310	1.0093	1.0292	1.0647	0.8471	0.9876	0.0482
FLJ32575	0.9259	1.0082	1.0686	0.9475	0.9876	0.0322
RPL31P10	0.8598	1.0904	1.0888	0.9113	0.9876	0.0598
HSD17B7P2	0.9852	1.1260	1.1224	0.7167	0.9876	0.0961
LOC648123	0.9931	1.1065	0.9792	0.8716	0.9876	0.0480
MPO	0.9097	1.1747	0.9610	0.9049	0.9876	0.0636
ZNF155	0.9548	1.1515	0.9704	0.8737	0.9876	0.0586
B3GAT1	0.8881	1.0771	1.0648	0.9203	0.9876	0.0486
LOC644188	0.9674	0.9240	1.2246	0.8343	0.9876	0.0837
FCRL1	0.8521	1.1775	1.0973	0.8234	0.9876	0.0882
SLC24A1	0.9289	1.0801	1.0546	0.8867	0.9876	0.0471
ERCC-00137	0.8555	1.1433	1.0557	0.8958	0.9876	0.0676
C21orf131	0.8736	1.0735	1.0346	0.9687	0.9876	0.0437
LOC1001889	0.9760	1.0425	1.0748	0.8570	0.9876	0.0481
ZSCAN1	1.0314	1.0832	1.0453	0.7905	0.9876	0.0666
MBD1	0.9707	1.1896	0.9692	0.8209	0.9876	0.0760
ZNF767	0.8860	1.2078	0.9671	0.8895	0.9876	0.0758
UBOX5	0.8883	1.2513	1.0449	0.7659	0.9876	0.1048
XKRY	0.9786	1.0770	1.0248	0.8699	0.9876	0.0441
DMRT1	1.0175	1.0386	1.0199	0.8744	0.9876	0.0380
CHRM2	0.9806	1.0582	1.0058	0.9058	0.9876	0.0317
LOC652086	0.9926	1.0045	0.9979	0.9555	0.9876	0.0110
LOC1001279	1.0082	1.0864	0.9793	0.8766	0.9876	0.0434
OBSCN	0.9578	1.1618	0.9009	0.9301	0.9876	0.0592
USP44	0.9801	1.0685	0.9769	0.9250	0.9876	0.0298
FLJ35880	0.9415	1.1470	1.0174	0.8447	0.9876	0.0638
LOC654165	0.8709	1.0860	1.0593	0.9343	0.9876	0.0511
RASGRP4	1.0088	1.0744	0.9678	0.8996	0.9876	0.0366
LOC642629	0.8559	1.1291	1.0810	0.8847	0.9876	0.0687
RS1	0.9841	1.1486	0.9058	0.9120	0.9876	0.0565
LOC648217	1.0186	0.9584	1.0805	0.8930	0.9877	0.0402
CD34	0.9065	1.0385	1.0722	0.9334	0.9877	0.0401
MIR196A2	0.8948	1.0213	1.1117	0.9228	0.9877	0.0495
WFDC9	0.9418	1.0175	0.9957	0.9957	0.9877	0.0161
STOX1	0.9527	1.0987	0.9908	0.9084	0.9877	0.0407
SLC6A1	1.0187	1.0660	1.0070	0.8589	0.9877	0.0448
APOB	0.9169	1.0513	1.0791	0.9034	0.9877	0.0452

LOC730743	0.9336	1.0543	1.0313	0.9315	0.9877	0.0322
SLC22A8	0.9630	1.0304	1.0291	0.9282	0.9877	0.0253
THPO	0.9728	1.0491	1.0230	0.9059	0.9877	0.0315
CORO6	0.9186	1.1783	1.0627	0.7911	0.9877	0.0843
LOC1001336	0.9697	1.1582	0.9697	0.8531	0.9877	0.0631
LOC643783	1.0204	1.1265	0.9826	0.8212	0.9877	0.0633
ABCB8	0.9122	1.0261	1.0972	0.9153	0.9877	0.0451
LOC1001303	0.9977	1.0166	1.0380	0.8985	0.9877	0.0309
RGS21	0.9373	1.3111	0.9004	0.8019	0.9877	0.1115
TET3	0.9214	1.1327	0.9877	0.9090	0.9877	0.0513
LOC654020	0.8383	1.0689	1.0520	0.9915	0.9877	0.0525
LOC341346	0.8972	1.0298	1.0025	1.0213	0.9877	0.0307
RBAK	1.0120	1.1612	0.9210	0.8566	0.9877	0.0660
CD37	0.9515	1.1361	0.9791	0.8841	0.9877	0.0533
C1orf89	0.7982	1.1935	1.1152	0.8440	0.9877	0.0980
LOC1001290	0.8867	1.1187	1.0594	0.8862	0.9877	0.0597
EPS8L1	0.9102	1.1712	0.9811	0.8884	0.9877	0.0643
LOC402110	0.9791	1.1583	1.0298	0.7838	0.9877	0.0778
OR2M2	1.0246	1.0013	0.9988	0.9262	0.9877	0.0213
LOC651772	0.8873	1.2231	1.0314	0.8092	0.9878	0.0910
PNMA6B	0.9381	1.1217	0.9064	0.9848	0.9878	0.0475
LOC644144	0.9588	1.2340	0.9690	0.7892	0.9878	0.0919
LOC727842	0.9610	0.9951	1.0932	0.9018	0.9878	0.0401
LOC642578	0.9117	1.1402	1.0291	0.8700	0.9878	0.0610
C16orf72	0.9174	1.3404	1.0217	0.6715	0.9878	0.1386
STAT5A	0.8516	1.2341	1.0056	0.8598	0.9878	0.0894
LOC648490	0.9997	1.2110	0.8912	0.8493	0.9878	0.0809
LOC1001321	0.9754	1.1111	1.0426	0.8220	0.9878	0.0618
LGALS9C	0.9433	1.0959	0.9302	0.9818	0.9878	0.0377
SPAG8	0.9365	1.1352	0.9981	0.8812	0.9878	0.0546
CLCN1	0.9198	1.1724	1.0043	0.8547	0.9878	0.0687
C17orf66	0.8936	1.1069	0.9821	0.9686	0.9878	0.0442
MIR548F5	0.9377	1.0736	1.0091	0.9307	0.9878	0.0336
LOC731074	0.9822	1.2334	0.9536	0.7821	0.9878	0.0930
FMO5	1.0240	1.1849	0.9121	0.8302	0.9878	0.0768
LOC1001342	0.9296	1.1456	1.0304	0.8457	0.9878	0.0647
KLHDC1	0.9286	1.0415	0.9805	1.0007	0.9878	0.0235
NKX1-2	0.8971	1.0513	0.9921	1.0109	0.9878	0.0327
PKD1P1	0.8618	1.0822	1.1410	0.8664	0.9878	0.0725
LOC1001302	0.9154	1.1277	1.0159	0.8923	0.9878	0.0538
MMAB	1.0794	1.2367	0.8220	0.8133	0.9878	0.1034
MYT1L	0.9826	1.0306	1.0358	0.9024	0.9878	0.0309
MSTP9	0.9907	1.0130	0.9749	0.9729	0.9879	0.0093
LOC643401	1.0065	1.0439	0.9891	0.9120	0.9879	0.0278
DNMT3A	0.9622	1.0638	1.0159	0.9095	0.9879	0.0334
LOC440503	0.8600	1.2613	0.9466	0.8837	0.9879	0.0929

NFIA	0.8093	1.2684	1.1423	0.7315	0.9879	0.1292
TAS2R40	1.0288	1.0387	0.9645	0.9195	0.9879	0.0281
LOC653163	0.7995	1.1909	1.0350	0.9260	0.9879	0.0830
TBC1D22A	0.9244	1.2180	1.0068	0.8023	0.9879	0.0875
OXER1	0.9217	1.0879	0.9101	1.0318	0.9879	0.0432
LOC1001322	0.8065	1.1251	1.0588	0.9611	0.9879	0.0692
LOC647269	0.9361	1.0203	1.0526	0.9426	0.9879	0.0288
KY	0.9513	1.0980	1.0242	0.8781	0.9879	0.0473
SIK2	0.9390	1.1289	1.0396	0.8441	0.9879	0.0617
LOC400942	1.0279	1.0701	0.9671	0.8864	0.9879	0.0399
CD40LG	0.8847	1.0317	1.0343	1.0009	0.9879	0.0352
LOC653605	0.8795	0.9970	1.1137	0.9613	0.9879	0.0486
ADAMTS18	0.9161	1.1580	1.0483	0.8293	0.9879	0.0724
C4BPA	0.9228	0.9800	1.0592	0.9895	0.9879	0.0280
LOC1001310	0.9879	1.0350	0.9700	0.9587	0.9879	0.0168
ASB3	0.8798	1.3120	0.9584	0.8014	0.9879	0.1127
CD109	0.9590	1.1596	0.9058	0.9273	0.9879	0.0583
LOC643397	0.9410	1.1038	1.0286	0.8782	0.9879	0.0494
MYOM3	0.9514	1.0547	1.0496	0.8961	0.9879	0.0388
SNCA	0.9276	1.0845	1.0523	0.8873	0.9879	0.0476
DUSP4	0.8827	1.1421	1.0072	0.9198	0.9879	0.0576
CCDC116	0.9492	1.0655	1.1061	0.8310	0.9879	0.0620
TMEM38B	0.8427	1.4685	0.9351	0.7055	0.9879	0.1670
ST8SIA3	1.0015	1.0143	1.0559	0.8801	0.9879	0.0378
LOC643998	0.9632	1.1261	1.0171	0.8455	0.9879	0.0583
BCL9L	1.0745	1.1989	0.9206	0.7578	0.9880	0.0955
DCAF4L1	0.9074	1.0718	1.0309	0.9417	0.9880	0.0382
AKR7L	0.9653	1.0636	0.9751	0.9478	0.9880	0.0258
SLC10A1	0.9989	1.0480	0.9645	0.9406	0.9880	0.0233
LOC642914	1.0764	1.0335	0.9548	0.8872	0.9880	0.0420
SPTB	0.9146	1.0512	1.0404	0.9457	0.9880	0.0340
LOC647718	0.9968	1.0949	1.0155	0.8447	0.9880	0.0523
ST6GALNAC5	1.0275	0.9609	1.0354	0.9280	0.9880	0.0261
LRIT2	0.9139	1.0779	1.0866	0.8735	0.9880	0.0551
SPHKAP	1.0122	1.0326	1.0121	0.8950	0.9880	0.0313
C16orf69	0.9492	1.1086	1.0052	0.8889	0.9880	0.0467
LOC649210	0.8576	1.2066	1.0047	0.8830	0.9880	0.0796
NTRK3	0.8814	1.0611	1.0617	0.9477	0.9880	0.0445
RNF180	0.9070	1.0938	1.0642	0.8869	0.9880	0.0531
FAM194A	1.0207	1.0569	0.9423	0.9321	0.9880	0.0303
GALNTL6	0.9077	0.9984	1.0546	0.9913	0.9880	0.0303
C14orf176	0.7230	1.0939	1.1188	1.0163	0.9880	0.0910
GDPD3	0.9746	1.0377	1.0346	0.9050	0.9880	0.0312
LOC1001290	0.9972	1.0441	1.0261	0.8846	0.9880	0.0358
LOC340529	0.9654	1.0784	0.9892	0.9190	0.9880	0.0335
GDAP1L1	0.9971	1.0751	0.9485	0.9314	0.9880	0.0322

LOC649686	0.8513	1.1298	1.0199	0.9510	0.9880	0.0586
PCNP	0.8668	1.3423	1.0548	0.6882	0.9880	0.1398
FSCB	1.0062	1.0217	1.1206	0.8035	0.9880	0.0665
LOC1001310	0.9709	1.0504	1.0382	0.8925	0.9880	0.0363
AMPD1	0.9400	1.1212	0.9916	0.8992	0.9880	0.0483
STARD4	0.9926	1.0352	1.0076	0.9167	0.9880	0.0253
TMEM169	1.0396	1.1458	0.8699	0.8968	0.9880	0.0644
LOC645263	0.8758	1.1293	1.0712	0.8758	0.9880	0.0659
LOC644472	0.9418	1.1925	0.9727	0.8452	0.9880	0.0733
FRG2B	0.9644	1.0878	0.9907	0.9092	0.9880	0.0373
ZNF70	1.0148	1.0743	1.0145	0.8485	0.9880	0.0486
SNORA52	0.9196	1.0372	0.9881	1.0073	0.9880	0.0250
LOC729777	0.9649	0.9445	1.0892	0.9535	0.9880	0.0340
AHSG	0.9908	1.1049	0.8885	0.9681	0.9880	0.0447
LOC652238	1.0603	0.9503	1.0346	0.9070	0.9881	0.0358
BMPR1B	1.0327	1.1270	0.9673	0.8252	0.9881	0.0634
SNORD126	0.9731	1.0172	0.9952	0.9667	0.9881	0.0115
MGC119295	0.9719	1.0952	1.0895	0.7957	0.9881	0.0701
LOC649320	0.9994	0.9736	0.9936	0.9857	0.9881	0.0056
CYP2C8	0.9733	1.1048	1.0259	0.8484	0.9881	0.0538
LOC644172	0.8685	1.2225	1.0609	0.8004	0.9881	0.0957
MOGAT1	0.9629	1.1348	0.8889	0.9656	0.9881	0.0520
RNF12	0.9693	1.0712	1.0229	0.8890	0.9881	0.0390
PRAM1	0.8948	1.0637	1.0575	0.9364	0.9881	0.0427
ASAM	0.9197	1.1197	1.0052	0.9079	0.9881	0.0489
ODF3L1	0.9244	1.1461	0.9626	0.9193	0.9881	0.0535
LOC645870	0.8809	1.0998	1.0905	0.8813	0.9881	0.0618
KIAA0692	0.9102	1.1170	0.9977	0.9275	0.9881	0.0469
LOC644591	0.9733	1.1063	0.9473	0.9256	0.9881	0.0406
KLHL29	0.8439	1.2813	0.9713	0.8560	0.9881	0.1018
RAXL1	0.8971	1.2716	0.9366	0.8473	0.9881	0.0962
CNTN4	0.8777	1.1536	1.0029	0.9182	0.9881	0.0610
LOC653227	0.9805	1.1512	0.9738	0.8471	0.9881	0.0624
FLJ37673	1.0066	1.0568	1.0021	0.8871	0.9881	0.0359
LOC1001284	1.0166	1.0383	0.9886	0.9092	0.9881	0.0282
LOC1001339	0.9125	1.1096	1.0561	0.8744	0.9881	0.0563
AMIGO3	0.9951	1.2269	0.8681	0.8625	0.9882	0.0853
CHR415SYT	0.9552	1.0269	1.1205	0.8500	0.9882	0.0572
VAMP3	1.0820	1.1046	1.1104	0.6556	0.9882	0.1110
C1orf210	0.9362	1.1197	0.9837	0.9131	0.9882	0.0462
COX7A1	1.0515	1.0380	0.9756	0.8875	0.9882	0.0374
KRTAP2-1	0.9130	1.0980	0.9813	0.9603	0.9882	0.0393
LOC1001291	0.9682	1.0543	1.0227	0.9075	0.9882	0.0322
C18orf51	0.9809	1.1103	0.9277	0.9338	0.9882	0.0424
GAFA3	0.9747	1.0078	1.0750	0.8953	0.9882	0.0373
LOC646999	0.9777	1.0789	0.9931	0.9031	0.9882	0.0361

LOC644450	0.8207	1.1829	1.0313	0.9179	0.9882	0.0779
FOXS1	0.9559	1.1280	0.9467	0.9222	0.9882	0.0471
SNORD98	0.8935	1.0642	1.0771	0.9180	0.9882	0.0479
SYCP2	0.9799	0.9916	1.0629	0.9185	0.9882	0.0296
APOBEC3G	0.9249	1.0769	1.0369	0.9142	0.9882	0.0405
RGR	0.9170	1.1309	1.0079	0.8971	0.9882	0.0533
ASB7	0.9165	1.1374	1.0006	0.8984	0.9882	0.0545
MS4A4A	0.9241	1.0403	1.0414	0.9470	0.9882	0.0308
ANKRD19	0.9146	1.0764	1.0655	0.8965	0.9882	0.0479
WNT8A	0.9074	1.0469	1.0773	0.9213	0.9882	0.0432
ANO7	0.9951	1.0690	0.9896	0.8993	0.9882	0.0347
CCDC146	0.9308	1.0528	1.0259	0.9436	0.9882	0.0301
C1orf140	0.9772	1.0409	1.0532	0.8817	0.9883	0.0392
CLEC6A	1.0154	1.0640	1.0253	0.8483	0.9883	0.0478
LOC645960	0.9489	0.9803	1.0327	0.9912	0.9883	0.0173
HIST1H2BM	0.9723	1.0871	1.0476	0.8460	0.9883	0.0531
LOC727757	0.9651	1.1796	0.9411	0.8672	0.9883	0.0671
WBP11P1	0.9812	1.0856	1.0138	0.8725	0.9883	0.0443
OR10T2	0.9510	1.0706	1.0555	0.8759	0.9883	0.0459
HOXB4	0.9086	1.0704	1.1507	0.8234	0.9883	0.0745
LOC342346	0.9221	1.2205	0.9807	0.8298	0.9883	0.0834
KIAA1370	0.9315	1.3754	0.9215	0.7247	0.9883	0.1375
OR10Q1	0.9576	1.0274	1.0737	0.8944	0.9883	0.0394
GSC	0.9419	1.1920	0.9522	0.8670	0.9883	0.0705
ODF3L2	0.9023	1.0799	1.0627	0.9083	0.9883	0.0481
LOC645986	0.8772	1.0543	1.1196	0.9021	0.9883	0.0587
LOC642904	0.9489	1.0759	1.0674	0.8611	0.9883	0.0514
FAM154B	0.9381	1.1053	1.0381	0.8718	0.9883	0.0519
SIGLEC7	0.9436	1.0812	0.9972	0.9313	0.9883	0.0341
LOC647946	0.9682	1.0866	1.0408	0.8577	0.9883	0.0499
GPR179	0.8877	1.2020	1.0293	0.8344	0.9883	0.0822
LOC440748	0.8792	1.1944	1.0232	0.8565	0.9883	0.0780
TM4SF5	1.0199	0.9841	1.0152	0.9342	0.9883	0.0197
LOC646360	0.9779	1.1301	1.0297	0.8157	0.9883	0.0657
LOC401137	0.9845	1.1153	0.9534	0.9002	0.9884	0.0458
ZFPM2	0.9533	1.1068	0.9652	0.9282	0.9884	0.0402
DLX3	0.8825	1.1970	0.9380	0.9360	0.9884	0.0707
LOC644883	0.9383	1.1027	1.0337	0.8787	0.9884	0.0497
EVX2	1.0148	1.1103	0.9700	0.8585	0.9884	0.0523
BRDG1	0.9274	1.0119	1.0070	1.0071	0.9884	0.0203
LOC154761	0.8874	1.1561	1.0034	0.9066	0.9884	0.0614
SNCG	0.9516	1.0601	1.0253	0.9165	0.9884	0.0330
LOC387873	1.0192	1.0132	1.0581	0.8630	0.9884	0.0430
LOC728047	0.9049	1.0771	1.0667	0.9048	0.9884	0.0483
MFSD9	0.9664	1.0851	1.0069	0.8953	0.9884	0.0396
TTLL6	0.8572	1.2029	1.0968	0.7967	0.9884	0.0965

IL3RA	1.0601	1.0053	1.0070	0.8812	0.9884	0.0379
LOC283887	0.8997	1.0987	0.9962	0.9591	0.9884	0.0418
TMEM205	0.7811	1.4726	1.0043	0.6957	0.9884	0.1740
ZNF132	0.8833	1.1674	1.0890	0.8140	0.9884	0.0835
DNAHL1	0.9242	1.0757	0.9813	0.9724	0.9884	0.0317
C2CD4D	0.9732	1.1098	0.9950	0.8758	0.9884	0.0480
NCF1C	0.8366	1.1959	1.0073	0.9139	0.9884	0.0774
SHOX2	0.9179	1.1599	1.0120	0.8639	0.9884	0.0648
HIC1	0.8947	0.9352	1.1684	0.9555	0.9884	0.0613
LOC1001323	0.9279	1.0146	1.0379	0.9734	0.9884	0.0242
PAPPA	1.0049	1.0508	1.0456	0.8524	0.9884	0.0465
UBE2K	0.9295	1.1364	0.9977	0.8901	0.9884	0.0541
FAM76A	0.9436	1.0776	1.0940	0.8387	0.9884	0.0602
BSX	0.9930	1.0676	0.9289	0.9643	0.9884	0.0294
LOC442132	0.9270	0.9959	1.1872	0.8437	0.9884	0.0732
ZNF66	0.9601	1.0457	1.0438	0.9042	0.9884	0.0345
PER1	1.0506	1.0525	1.0205	0.8302	0.9885	0.0533
LOC401268	0.9427	1.1092	0.9639	0.9380	0.9885	0.0406
MIR1197	0.8953	1.1037	1.0495	0.9054	0.9885	0.0521
LOC649160	0.9173	1.0630	1.0109	0.9627	0.9885	0.0313
SNORD54	0.9571	1.1009	0.9990	0.8968	0.9885	0.0430
LOC644558	0.9212	1.0067	1.1474	0.8786	0.9885	0.0593
LOC642348	0.8573	1.0817	1.0916	0.9233	0.9885	0.0583
LOC646871	1.0619	1.0614	0.9172	0.9135	0.9885	0.0422
FAM90A11P	1.0632	1.0123	1.0117	0.8668	0.9885	0.0423
PRPH	0.9996	1.1253	0.9882	0.8408	0.9885	0.0582
C4orf15	0.9011	1.1004	1.0102	0.9422	0.9885	0.0436
LOC646110	0.9456	1.1467	1.0182	0.8434	0.9885	0.0638
LRRC37A3	0.9754	1.0579	1.0301	0.8906	0.9885	0.0369
FAM81B	1.0225	1.0959	0.9539	0.8816	0.9885	0.0459
LOC649707	0.9855	1.0979	0.9410	0.9296	0.9885	0.0384
CACNG8	0.8797	1.0818	1.0547	0.9378	0.9885	0.0479
KRTAP10-7	1.0128	0.9946	0.9942	0.9524	0.9885	0.0128
HSPB9	0.9308	1.1088	1.0275	0.8869	0.9885	0.0497
FLJ30594	0.9638	1.0845	1.0297	0.8759	0.9885	0.0449
CCDC62	0.9246	1.1104	1.0101	0.9089	0.9885	0.0463
LOC645932	0.9150	1.0928	1.0272	0.9190	0.9885	0.0434
VENTXP7	0.9821	1.0930	0.9930	0.8860	0.9885	0.0423
FAM75A6	0.9766	1.0908	1.0072	0.8795	0.9885	0.0436
LOC1001330	0.9790	1.0737	0.9719	0.9295	0.9885	0.0304
OR51B6	0.9303	1.0753	1.0376	0.9108	0.9885	0.0402
MYOC	0.9192	1.1463	0.9485	0.9402	0.9885	0.0529
LOC1001290	0.9357	1.0519	0.9896	0.9769	0.9885	0.0240
KIAA0363	0.9175	1.0453	1.0562	0.9352	0.9885	0.0362
C1orf213	0.9332	1.1273	1.0093	0.8844	0.9885	0.0529
PKD1L1	0.9314	1.1097	1.0222	0.8909	0.9886	0.0488

XIAP	0.9584	1.1160	0.9639	0.9159	0.9886	0.0438
WIT1	0.9240	1.0590	1.0514	0.9198	0.9886	0.0385
LOC649752	0.9953	1.0920	0.9784	0.8886	0.9886	0.0417
AADACL4	0.8404	1.0896	0.9952	1.0291	0.9886	0.0531
LOC1001315	0.9207	1.0514	1.0262	0.9560	0.9886	0.0303
ZRANB3	0.9540	1.0985	1.0124	0.8895	0.9886	0.0444
LOC1001330	1.0097	1.1222	1.0539	0.7686	0.9886	0.0769
ATG9B	0.9314	1.0359	1.0236	0.9636	0.9886	0.0248
LOC650010	0.9381	1.1642	0.9788	0.8733	0.9886	0.0624
KRTAP15-1	0.9417	1.0501	1.0412	0.9213	0.9886	0.0333
LOC441070	1.1202	1.0368	0.9491	0.8484	0.9886	0.0584
KCNIP2	0.8935	1.0551	1.0467	0.9591	0.9886	0.0384
LOC442204	1.0001	1.1089	0.9509	0.8946	0.9886	0.0455
LOC647234	0.9819	1.1250	0.9587	0.8889	0.9886	0.0496
LOC1001304	0.9717	1.0583	1.1037	0.8208	0.9886	0.0623
LOC643311	1.2674	0.8742	0.8566	0.9564	0.9886	0.0954
LOC642383	1.0081	0.9787	1.0138	0.9539	0.9886	0.0139
LOC1001308	0.9729	1.1819	0.9379	0.8618	0.9886	0.0685
C2CD4A	0.9949	1.0040	1.0444	0.9113	0.9886	0.0279
LOC1001305	0.9877	1.0974	1.0926	0.7769	0.9886	0.0750
DDN	0.9278	1.0663	1.0316	0.9289	0.9887	0.0355
LOC402160	0.8560	1.1067	1.0376	0.9543	0.9887	0.0541
CHRNA1	0.9799	1.0835	1.0033	0.8879	0.9887	0.0402
MGC39584	1.0316	0.9888	0.9996	0.9347	0.9887	0.0202
C17orf76	0.8687	1.1259	0.9954	0.9646	0.9887	0.0531
HEY2	0.9499	1.1052	1.0200	0.8796	0.9887	0.0483
BTNL3	0.9608	1.0606	1.0575	0.8758	0.9887	0.0442
ABCA4	0.8881	1.1051	0.9960	0.9655	0.9887	0.0449
LOC646507	0.9618	1.0245	1.0177	0.9508	0.9887	0.0189
TRAPPC1	0.8873	1.2814	1.0549	0.7311	0.9887	0.1179
LOC730360	0.9956	1.0198	0.9829	0.9565	0.9887	0.0132
FANCA	0.9509	1.0863	1.0039	0.9137	0.9887	0.0374
LOC1001325	0.9295	1.0102	1.0865	0.9286	0.9887	0.0378
LOC388565	1.0277	0.9911	1.0147	0.9213	0.9887	0.0237
MTERFD3	0.9422	1.1280	0.9990	0.8857	0.9887	0.0519
MGC34821	0.9128	1.0341	1.0286	0.9793	0.9887	0.0281
C5orf20	0.9368	1.1274	1.0263	0.8643	0.9887	0.0569
OR5H2	0.9154	1.0807	1.0495	0.9093	0.9887	0.0446
FLJ46836	0.8536	1.0778	1.0486	0.9749	0.9887	0.0500
PURG	0.9261	1.2158	0.9733	0.8397	0.9887	0.0806
TNFAIP8L3	1.0140	1.1591	0.9761	0.8058	0.9887	0.0726
SMR3B	1.0207	1.0057	1.1366	0.7920	0.9888	0.0718
OLR1	0.9727	1.0852	0.9967	0.9005	0.9888	0.0381
HDAC11	0.9234	1.3294	0.9409	0.7613	0.9888	0.1205
PCDH8	0.8817	1.0709	1.0793	0.9232	0.9888	0.0506
DUSP14	0.6818	1.4903	1.0371	0.7459	0.9888	0.1842

LOC645088	0.9379	1.0887	1.0004	0.9281	0.9888	0.0370
GNRH2	0.9471	1.0225	1.0272	0.9584	0.9888	0.0210
LOC1001280	0.8921	1.0296	1.0127	1.0207	0.9888	0.0324
LASS6	1.0813	1.2834	0.9434	0.6470	0.9888	0.1336
XPA	0.9505	1.1273	1.1484	0.7289	0.9888	0.0973
EIF2S1	1.0358	1.1547	1.0267	0.7379	0.9888	0.0886
LOC647748	0.9579	1.1046	0.9629	0.9298	0.9888	0.0393
TTTY8B	0.9667	1.0567	1.0384	0.8934	0.9888	0.0373
MADCAM1	0.9471	0.9842	0.9716	1.0523	0.9888	0.0225
PROX2	0.9445	1.0470	1.0532	0.9105	0.9888	0.0361
FCER1G	0.9287	1.0723	1.0776	0.8767	0.9888	0.0509
ST8SIA5	0.9734	1.0334	0.9699	0.9786	0.9888	0.0150
LOC1001314	0.9918	0.9760	1.0849	0.9026	0.9888	0.0374
KIAA1772	0.9340	0.9870	0.9786	1.0557	0.9888	0.0251
LOC646708	0.8524	1.1651	1.0480	0.8898	0.9888	0.0725
STAP1	1.0298	0.9396	1.0580	0.9280	0.9888	0.0324
TSNARE1	0.9666	1.0786	1.0204	0.8897	0.9888	0.0402
ARG1	0.8881	1.0356	1.1432	0.8884	0.9888	0.0621
LOC256880	0.9237	1.0804	1.0280	0.9232	0.9888	0.0392
LOC400891	1.0344	1.0446	1.0140	0.8624	0.9888	0.0426
PWP2	0.8223	1.0899	1.0814	0.9618	0.9888	0.0627
LOC1001315	0.9302	1.1639	0.9840	0.8772	0.9888	0.0623
MIR603	0.9653	1.0926	0.9674	0.9301	0.9888	0.0356
LOC1001327	0.9867	0.9427	1.1521	0.8739	0.9888	0.0591
GLDN	1.0371	1.0681	0.9568	0.8933	0.9888	0.0395
LOC730684	1.0622	1.0176	0.9756	0.9001	0.9888	0.0345
LOC1001344	0.9821	0.9438	1.0626	0.9669	0.9888	0.0258
FAM47A	0.9932	1.0666	1.0039	0.8917	0.9889	0.0362
SYTL5	0.8744	1.2401	0.9613	0.8796	0.9889	0.0861
FAM57B	0.8117	1.1010	1.0201	1.0226	0.9889	0.0620
LOC653676	0.9088	1.0643	1.0220	0.9604	0.9889	0.0342
LOC441551	1.0105	1.0125	0.9527	0.9798	0.9889	0.0142
ERCC-00059	0.9389	1.0002	1.0885	0.9279	0.9889	0.0368
LOC646470	1.0119	1.0494	0.9997	0.8944	0.9889	0.0332
C4orf35	0.8786	1.0815	1.0984	0.8969	0.9889	0.0586
TTTY8	0.9357	1.0837	0.9823	0.9538	0.9889	0.0330
TNS1	0.9084	1.1019	0.9270	1.0183	0.9889	0.0447
LOC647868	0.9253	1.0447	1.0134	0.9721	0.9889	0.0259
LOC400581	0.9332	1.1395	1.0356	0.8472	0.9889	0.0633
LOC653088	0.9855	1.0518	1.0800	0.8383	0.9889	0.0540
LOC728528	0.9979	1.1177	0.9573	0.8827	0.9889	0.0491
ZNF484	0.8780	1.2936	0.7963	0.9876	0.9889	0.1089
HEATR7B2	0.9599	1.0044	1.0703	0.9211	0.9889	0.0320
METTL12	0.9101	1.2072	1.0306	0.8078	0.9889	0.0858
ERCC-00095	0.8892	1.0481	1.0378	0.9808	0.9889	0.0364
MMP20	0.9619	1.0792	1.0056	0.9091	0.9890	0.0360

ADCY2	0.8585	1.1132	1.0692	0.9150	0.9890	0.0608
GDF5OS	0.9279	1.0540	1.0570	0.9170	0.9890	0.0385
DLK2	0.9050	1.1276	1.0343	0.8889	0.9890	0.0565
LOC1001347	0.9376	1.0893	0.9675	0.9614	0.9890	0.0341
LOC595101	0.9326	1.0061	0.9895	1.0276	0.9890	0.0203
LOC388242	0.9341	1.1268	1.0284	0.8665	0.9890	0.0567
LOC442020	0.7794	1.1367	1.0635	0.9763	0.9890	0.0772
TM4SF4	0.9608	1.0364	0.9974	0.9613	0.9890	0.0180
LOC1001280	0.9486	1.0539	1.1129	0.8406	0.9890	0.0600
PPP1R15B	0.9797	1.0786	0.9894	0.9082	0.9890	0.0349
CDKL2	0.9197	1.1010	1.0164	0.9188	0.9890	0.0438
CCDC35	0.8300	1.1460	0.9751	1.0049	0.9890	0.0648
ARHGAP21	0.9188	1.3280	0.9360	0.7733	0.9890	0.1188
MYO1G	1.0048	1.1976	0.9368	0.8168	0.9890	0.0797
FAM12B	0.9927	1.1039	1.0095	0.8498	0.9890	0.0525
MIR573	1.0066	1.0652	0.9907	0.8936	0.9890	0.0356
NCRNA0011¢	0.8009	1.1254	1.0554	0.9744	0.9890	0.0699
KRTAP5-6	1.1175	0.9584	1.0107	0.8694	0.9890	0.0518
AACSL	0.9636	1.1319	1.0287	0.8319	0.9890	0.0628
RHCE	0.9452	1.0946	1.0029	0.9133	0.9890	0.0398
GRASP	0.8729	1.1316	0.9678	0.9838	0.9890	0.0535
LOC1001344	1.0246	1.0261	1.0093	0.8962	0.9890	0.0312
BBS7	0.9093	1.2790	1.0343	0.7335	0.9890	0.1147
LOC730804	1.0037	1.0351	1.0136	0.9038	0.9890	0.0292
CATSPERB	0.9973	1.0506	0.9834	0.9249	0.9890	0.0258
DNHD2	0.9973	1.0608	0.9464	0.9517	0.9891	0.0265
MIR33B	0.9688	1.1275	1.0049	0.8550	0.9891	0.0561
PPP1R3B	0.9874	1.0432	1.0650	0.8606	0.9891	0.0458
ELA3A	0.9823	1.0955	0.9824	0.8960	0.9891	0.0409
POU3F2	0.9935	1.0811	0.9396	0.9421	0.9891	0.0331
LOC652661	0.8626	1.1868	0.9844	0.9225	0.9891	0.0704
MIR630	1.0624	1.0542	0.9991	0.8406	0.9891	0.0514
FLJ35740	0.8825	1.1052	0.9964	0.9720	0.9891	0.0458
DBX2	0.9765	1.1638	0.9362	0.8798	0.9891	0.0615
RASGEF1C	0.9103	1.1066	1.0801	0.8592	0.9891	0.0614
C14orf148	0.9810	1.0830	1.0165	0.8759	0.9891	0.0432
FAM188B	0.7638	1.2016	1.0879	0.9031	0.9891	0.0970
BMP2	0.9943	1.0821	1.0107	0.8693	0.9891	0.0443
ZNF648	0.8515	1.2120	1.0330	0.8600	0.9891	0.0852
LOC152578	1.0592	1.0156	1.0125	0.8692	0.9891	0.0414
HFM1	0.9715	1.1030	0.9629	0.9191	0.9891	0.0397
RSPO2	0.9303	1.1735	0.9551	0.8976	0.9891	0.0626
LOC643445	0.9871	1.0705	1.0242	0.8747	0.9891	0.0418
LOC1001322	0.9615	1.0301	1.0084	0.9566	0.9891	0.0179
GRINL1A	0.9159	1.2086	0.9869	0.8452	0.9891	0.0787
C14orf39	0.8945	1.1564	0.9746	0.9311	0.9892	0.0581

LOC1001278	1.0050	1.0891	0.9546	0.9079	0.9892	0.0388
ACCN1	0.9644	1.0159	1.0209	0.9554	0.9892	0.0170
LOC652775	0.9593	1.1386	1.0055	0.8532	0.9892	0.0591
PLA2G4B	0.9349	1.3086	0.8796	0.8336	0.9892	0.1085
C3orf32	1.0117	1.0749	0.9352	0.9349	0.9892	0.0338
FLJ10996	0.8863	1.1281	1.0691	0.8732	0.9892	0.0644
LOC1001282	0.8907	1.0939	1.0462	0.9260	0.9892	0.0482
LOC441762	1.0410	1.0025	0.9501	0.9631	0.9892	0.0206
DACH1	0.9100	1.0719	1.0058	0.9689	0.9892	0.0339
LOC1001318	0.9168	1.1173	1.0255	0.8971	0.9892	0.0512
LOC653158	1.0296	1.1882	0.9202	0.8188	0.9892	0.0791
LOC1001327	0.9249	1.1064	1.0641	0.8613	0.9892	0.0576
MIR1272	0.9509	1.0259	1.0635	0.9164	0.9892	0.0337
LOC654092	1.0439	1.0103	1.0344	0.8683	0.9892	0.0409
GAPDHS	0.9832	0.9960	1.0478	0.9297	0.9892	0.0242
SLC16A1	0.8500	1.1803	0.9721	0.9544	0.9892	0.0692
LOC645797	0.9502	0.9887	1.0229	0.9950	0.9892	0.0150
ABCF3	0.9737	1.2447	0.9398	0.7987	0.9892	0.0932
LOC1001307	0.8270	1.1598	1.0977	0.8724	0.9892	0.0821
LOC1001319	0.8700	1.0990	1.0148	0.9730	0.9892	0.0476
FLJ42258	0.9278	1.1052	0.9737	0.9502	0.9892	0.0398
FRG1B	1.0077	1.0449	1.0413	0.8630	0.9892	0.0429
OR4M1	0.9457	1.0842	1.0031	0.9239	0.9892	0.0358
KCNA6	0.9292	1.1351	0.9559	0.9367	0.9892	0.0490
MIR636	0.9912	1.0561	1.0632	0.8465	0.9892	0.0503
AKR1C1	0.8847	1.1558	1.0627	0.8537	0.9892	0.0721
LOC652448	0.9193	1.0799	1.0244	0.9334	0.9892	0.0381
IL1A	0.9229	1.0238	1.0431	0.9671	0.9892	0.0274
TRAPPC2	0.9505	1.1194	1.0752	0.8119	0.9892	0.0691
OR2A20P	0.9174	1.1076	1.0110	0.9210	0.9892	0.0450
LOC1001345	0.8603	1.1544	0.9602	0.9821	0.9892	0.0611
LOC1001283	0.9120	0.9988	1.0843	0.9620	0.9893	0.0363
NPCDR1	0.8596	1.1789	0.9782	0.9404	0.9893	0.0679
DPF3	0.9543	1.0626	1.0623	0.8779	0.9893	0.0450
PATE1	0.8743	1.1000	1.0616	0.9211	0.9893	0.0543
LOC653050	0.9504	1.1052	0.9820	0.9194	0.9893	0.0407
MED12	0.8539	1.1394	0.9579	1.0059	0.9893	0.0592
OSTbeta	0.9758	1.0585	0.9482	0.9746	0.9893	0.0239
LOC653886	0.8923	0.9997	1.1689	0.8962	0.9893	0.0648
LOC645441	1.0386	1.0544	0.9963	0.8679	0.9893	0.0423
LOC646700	0.9380	1.1791	0.9651	0.8749	0.9893	0.0660
Gcom1	0.9485	1.0478	1.0437	0.9171	0.9893	0.0332
C14orf73	0.9057	1.0544	1.0178	0.9792	0.9893	0.0318
C11orf21	1.0213	1.0155	1.1278	0.7926	0.9893	0.0704
LOC642608	0.8706	1.1064	1.1517	0.8285	0.9893	0.0817
PCDHA12	0.9542	1.0921	1.0419	0.8690	0.9893	0.0492

FABP3	0.9276	1.0458	1.0551	0.9288	0.9893	0.0353
LOC644006	0.8978	1.0605	1.0781	0.9208	0.9893	0.0466
ERCC-00085	0.9300	1.0252	1.0369	0.9652	0.9893	0.0253
C20orf123	0.9707	1.1982	0.8413	0.9471	0.9893	0.0751
LOC1001294	0.8672	1.2316	0.9572	0.9013	0.9893	0.0829
LOC392188	1.0366	0.8812	1.0992	0.9402	0.9893	0.0487
LOC283514	0.8847	1.3096	0.9193	0.8437	0.9893	0.1079
SNORD97	0.8986	1.0704	1.0433	0.9450	0.9893	0.0405
KIR2DS5	0.8981	1.0147	1.0420	1.0025	0.9893	0.0315
CDY1	0.9436	1.0917	0.9957	0.9264	0.9893	0.0372
TTC19	0.9239	1.4396	0.9977	0.5962	0.9893	0.1736
LOC387761	0.9076	1.1410	1.0616	0.8472	0.9893	0.0678
SLC22A2	0.9214	1.1212	0.9929	0.9218	0.9894	0.0471
LOC653129	0.9911	1.0981	1.0328	0.8354	0.9894	0.0558
LOC340527	0.8991	1.1026	1.0361	0.9197	0.9894	0.0483
LOC654069	0.9244	1.2199	0.9282	0.8848	0.9894	0.0775
LOC646320	0.8833	1.0795	1.0722	0.9225	0.9894	0.0506
KRT23	1.0255	0.9622	1.0418	0.9279	0.9894	0.0267
LOC645790	0.9933	1.0360	1.0976	0.8305	0.9894	0.0571
RASA2	0.9151	1.0672	1.0033	0.9718	0.9894	0.0317
GRIPAP1	0.7770	1.2426	1.0515	0.8864	0.9894	0.1015
BHMT	0.9184	1.0564	1.0660	0.9168	0.9894	0.0415
PAGE4	0.9416	1.0439	1.0604	0.9116	0.9894	0.0369
PHYHIPL	0.9434	1.0897	0.9788	0.9457	0.9894	0.0344
LOC1001292	0.9237	1.1008	1.1085	0.8245	0.9894	0.0696
LOC652479	0.9866	1.0561	1.0072	0.9077	0.9894	0.0309
C3orf48	0.9188	1.0749	0.9877	0.9763	0.9894	0.0322
LOC347544	0.7253	1.4976	1.0277	0.7070	0.9894	0.1847
KRTAP22-2	0.9615	1.0438	1.0178	0.9346	0.9894	0.0251
FGD4	0.9003	1.1617	1.0175	0.8781	0.9894	0.0651
ERCC-00130	0.9667	1.0823	1.0399	0.8689	0.9894	0.0467
RPL37	0.9507	1.1327	0.9377	0.9367	0.9894	0.0479
LOC441799	1.0004	0.9871	1.0368	0.9335	0.9894	0.0214
LOC338963	0.9125	1.2056	1.0590	0.7806	0.9894	0.0918
LCE3C	0.9336	1.1625	0.9897	0.8718	0.9894	0.0625
LOC1001344	0.9007	1.1649	0.9560	0.9362	0.9894	0.0596
RNASE3	0.9892	1.0578	1.0057	0.9052	0.9895	0.0317
PGM2	0.7368	1.4329	1.0000	0.7881	0.9895	0.1584
HS2ST1	0.8776	1.2093	0.9896	0.8813	0.9895	0.0777
HOXC10	0.9282	1.0398	1.0985	0.8914	0.9895	0.0481
ALLC	0.9452	1.0465	1.0402	0.9260	0.9895	0.0314
LOC651112	0.8997	1.1003	1.0828	0.8751	0.9895	0.0593
LOC648220	0.9714	1.0530	0.9747	0.9588	0.9895	0.0214
LOC400403	0.9280	1.1054	0.9984	0.9261	0.9895	0.0421
HBA1	0.9857	0.9945	1.0559	0.9218	0.9895	0.0274
CD1D	0.9219	1.0611	1.0262	0.9488	0.9895	0.0325

CAPN3	0.9546	1.0762	1.0010	0.9262	0.9895	0.0328
LOC1001302	0.9826	1.0994	1.0456	0.8304	0.9895	0.0581
NDUFA11	0.8458	1.4515	0.9611	0.6997	0.9895	0.1630
MAPK7	1.0143	1.1595	0.9744	0.8098	0.9895	0.0719
SPAG11A	0.9638	1.0683	1.0248	0.9013	0.9895	0.0364
SIRT6	1.1084	1.1041	0.9918	0.7539	0.9895	0.0831
LOC1001316	0.8931	1.0741	1.0171	0.9738	0.9895	0.0381
CAV2	0.9298	1.1396	0.9820	0.9068	0.9895	0.0524
WTIP	0.9509	1.1012	1.0315	0.8746	0.9895	0.0491
UACA	0.9507	1.1772	0.9631	0.8671	0.9895	0.0661
ATF7	0.9250	1.0819	1.0385	0.9128	0.9895	0.0418
LOC644047	0.9289	1.0898	1.0161	0.9234	0.9895	0.0396
HAS2AS	0.9178	1.1881	0.9678	0.8844	0.9895	0.0684
BMPR2	0.9783	1.2004	1.0495	0.7300	0.9895	0.0981
LOC1001315	0.9339	1.0666	1.0407	0.9171	0.9896	0.0375
CPA5	0.9898	1.0550	1.0096	0.9037	0.9896	0.0317
ANXA6	0.8855	1.2440	0.9841	0.8447	0.9896	0.0897
LOC643927	1.0326	1.0663	0.9465	0.9129	0.9896	0.0359
LOC730839	0.9406	1.0877	1.0339	0.8961	0.9896	0.0435
CORO7	1.0521	0.9293	1.1268	0.8501	0.9896	0.0618
LOC439951	1.0190	1.0729	0.9755	0.8909	0.9896	0.0384
LOC729870	1.0069	1.0791	1.0312	0.8410	0.9896	0.0517
MSRB3	0.9548	1.0860	1.0160	0.9015	0.9896	0.0397
LOC1001301	0.9845	1.0500	1.0568	0.8670	0.9896	0.0440
LOC389151	0.9209	1.0968	0.9808	0.9598	0.9896	0.0378
SEMA5A	0.9712	1.0410	1.0735	0.8726	0.9896	0.0444
CXADRP3	0.9774	1.2168	0.9123	0.8519	0.9896	0.0800
GCK	0.9281	1.0575	1.0766	0.8963	0.9896	0.0454
LOC650433	0.9152	1.1886	0.9976	0.8570	0.9896	0.0723
OR8K5	1.0236	1.0048	1.0978	0.8323	0.9896	0.0562
ZNF513	1.0349	1.1770	0.9856	0.7610	0.9896	0.0863
LOC643647	0.8906	1.1553	1.0571	0.8555	0.9896	0.0706
LOC730826	0.9125	1.1461	1.0159	0.8839	0.9896	0.0594
HAR1B	0.8813	1.1170	1.0250	0.9351	0.9896	0.0518
SDS	0.9825	1.1392	0.9841	0.8527	0.9896	0.0586
MIR1-1	0.9803	1.0922	0.9343	0.9518	0.9896	0.0355
LOC441682	0.9549	1.1040	1.0172	0.8826	0.9896	0.0470
LOC647343	1.0004	1.0042	0.9874	0.9665	0.9896	0.0085
MGC39821	0.9693	0.9966	1.0827	0.9100	0.9897	0.0359
NR2E1	0.8482	1.1701	1.0930	0.8474	0.9897	0.0834
FAM173B	0.8710	1.3743	0.9605	0.7528	0.9897	0.1351
LOC646754	0.9279	1.1152	1.0563	0.8592	0.9897	0.0585
LOC390858	0.9329	1.0417	1.0599	0.9242	0.9897	0.0355
LOC728897	0.9294	1.0249	1.0049	0.9995	0.9897	0.0208
LOC728219	0.9464	1.1599	0.9993	0.8530	0.9897	0.0643
MAGEC2	0.9654	1.1037	1.0591	0.8305	0.9897	0.0604

C21orf67	0.8766	1.1677	0.9700	0.9443	0.9897	0.0625
LOC1001311	0.9637	1.0597	0.9994	0.9359	0.9897	0.0267
UPP2	1.0136	1.1257	0.9863	0.8332	0.9897	0.0603
LOC643619	0.8712	1.0368	1.1161	0.9346	0.9897	0.0542
SBK2	0.9500	1.1274	0.9651	0.9163	0.9897	0.0470
PAPD5	0.8238	1.3512	0.9896	0.7942	0.9897	0.1279
KLK8	0.9622	1.0817	0.9463	0.9685	0.9897	0.0310
MIR195	1.0331	1.0305	1.0088	0.8865	0.9897	0.0348
ZNF776	0.9753	1.1800	0.9209	0.8826	0.9897	0.0662
PNLIPRP1	0.8378	1.1328	1.1320	0.8562	0.9897	0.0825
AJAP1	0.9545	1.0217	1.0579	0.9247	0.9897	0.0305
FGFBP2	0.9656	1.1414	1.0329	0.8190	0.9897	0.0674
KLRF1	1.0075	1.0227	1.0522	0.8765	0.9897	0.0389
LOC220686	1.0153	1.1443	0.9778	0.8215	0.9897	0.0665
LOC652713	0.9921	1.2239	0.8886	0.8542	0.9897	0.0834
LOC400807	0.9319	1.0809	1.0238	0.9222	0.9897	0.0380
IFNG	0.8761	1.0419	1.1163	0.9246	0.9897	0.0547
C11orf34	0.9291	1.0610	1.0899	0.8790	0.9897	0.0509
CXorf58	0.9649	1.0993	0.9904	0.9044	0.9898	0.0407
LOC645186	0.8908	1.1221	0.9554	0.9907	0.9898	0.0487
LOC649524	0.9234	1.0777	1.1671	0.7909	0.9898	0.0832
LOC643412	1.0162	1.1315	0.9256	0.8858	0.9898	0.0546
HAS2	0.9611	1.0866	1.0138	0.8976	0.9898	0.0401
MATN4	1.0046	1.0085	1.0319	0.9141	0.9898	0.0259
LOC650930	0.8977	1.2024	0.9678	0.8912	0.9898	0.0730
LOC649447	0.8701	1.1419	1.0876	0.8594	0.9898	0.0731
LOC649905	0.9760	1.0746	1.0368	0.8717	0.9898	0.0443
LOC1001315	1.0788	0.9627	1.0590	0.8585	0.9898	0.0506
PDK1	0.9689	1.1741	0.8944	0.9217	0.9898	0.0633
GRM5	0.8418	1.1276	1.1127	0.8770	0.9898	0.0757
GPR12	1.0595	0.9671	0.9861	0.9465	0.9898	0.0246
LOC1001338	0.9832	1.0750	0.9930	0.9080	0.9898	0.0342
CLEC4A	0.9086	1.1069	1.0393	0.9044	0.9898	0.0500
TMEM57	1.0516	1.0037	1.0859	0.8181	0.9898	0.0597
PLA2R1	0.9501	1.1254	0.9979	0.8858	0.9898	0.0507
CCL14	0.9367	1.0307	1.0121	0.9797	0.9898	0.0206
LOC730909	1.0074	1.0146	0.9878	0.9494	0.9898	0.0146
LOC728276	0.9336	1.1074	0.9822	0.9361	0.9898	0.0408
IFT172	0.9577	1.1673	0.9234	0.9108	0.9898	0.0600
C12orf65	0.9616	1.1610	1.0990	0.7377	0.9898	0.0938
IGSF2	0.9356	1.1612	0.8936	0.9689	0.9898	0.0592
CRCP	0.9503	1.1253	1.0201	0.8637	0.9898	0.0553
MCOLN3	0.9124	1.0768	1.0528	0.9172	0.9898	0.0436
MIRLET7I	0.9766	1.0847	0.9884	0.9096	0.9898	0.0361
SNORA35	1.0142	1.0012	1.0111	0.9329	0.9898	0.0192
MIR517B	1.0080	0.9963	1.0486	0.9065	0.9898	0.0300

APEH	0.8372	1.4403	0.9215	0.7603	0.9898	0.1537
HIST1H2BI	1.0281	1.0130	0.9903	0.9280	0.9898	0.0220
DNAH11	0.9899	1.0252	1.0579	0.8863	0.9898	0.0372
FAM47B	0.9426	1.0660	1.0747	0.8761	0.9899	0.0485
UPK2	1.0047	1.0627	1.0445	0.8475	0.9899	0.0490
KLHL10	0.9736	1.0745	1.0293	0.8821	0.9899	0.0414
LOC650418	0.9469	1.1117	1.0672	0.8336	0.9899	0.0626
TG	0.8822	1.0122	1.0512	1.0138	0.9899	0.0370
KCTD11	0.8629	1.2449	0.9696	0.8821	0.9899	0.0881
STARD13	0.9817	1.0569	1.0108	0.9101	0.9899	0.0308
GPC3	1.0461	1.0177	0.9937	0.9020	0.9899	0.0312
LOC1001325	1.0797	1.0250	1.0255	0.8292	0.9899	0.0551
MGC39545	1.0110	1.0097	1.0023	0.9365	0.9899	0.0179
LOC1001335	0.8675	1.0724	1.1012	0.9184	0.9899	0.0572
MS4A8B	0.8990	1.0857	0.9320	1.0428	0.9899	0.0443
ZBTB44	0.7878	1.1708	1.2236	0.7772	0.9899	0.1202
LOC641978	0.9368	1.2162	0.9138	0.8927	0.9899	0.0760
IFNA8	0.9512	1.0862	1.0461	0.8760	0.9899	0.0474
FLJ20309	0.9624	1.0601	1.0898	0.8472	0.9899	0.0548
TAGAP	0.9949	1.0592	1.0214	0.8840	0.9899	0.0377
LOC643896	0.9307	1.1165	1.0200	0.8924	0.9899	0.0500
AMICA1	0.9675	1.0215	1.0081	0.9624	0.9899	0.0147
LOC402269	0.9283	1.0723	1.0332	0.9257	0.9899	0.0372
FOXR1	0.8855	1.0619	1.0298	0.9824	0.9899	0.0385
LOC284751	0.9741	1.0513	0.9888	0.9454	0.9899	0.0224
NKAPL	0.9227	1.1132	0.9895	0.9342	0.9899	0.0436
LOC728388	0.9514	0.9773	1.0278	1.0032	0.9899	0.0165
LOC1001284	0.9298	0.9887	1.1329	0.9083	0.9899	0.0506
OR8U9	0.9767	1.0468	1.0323	0.9039	0.9899	0.0324
COX4I1	0.8728	1.3514	1.0305	0.7050	0.9899	0.1376
LOC728065	0.9455	1.1251	0.9452	0.9439	0.9899	0.0451
EPHA10	0.9322	1.1076	0.9886	0.9313	0.9899	0.0415
LOC389000	0.9540	1.1185	0.9627	0.9245	0.9899	0.0436
LOC642611	0.8723	1.0556	1.0144	1.0174	0.9899	0.0403
P2RY13	0.9418	1.0611	1.0388	0.9180	0.9899	0.0353
LOC1001340	0.9693	1.1010	1.0452	0.8442	0.9899	0.0556
LOC653253	1.0123	1.0899	0.9109	0.9467	0.9899	0.0394
LOC1001297	0.9662	1.0900	0.9700	0.9335	0.9899	0.0344
LOC653905	1.0363	1.0630	1.0378	0.8225	0.9899	0.0561
LOC730092	0.9793	1.0274	1.0565	0.8966	0.9899	0.0350
CNTD1	0.9493	1.1547	0.9689	0.8869	0.9899	0.0576
EBF3	0.8945	1.1626	1.0333	0.8694	0.9899	0.0679
SREBF2	0.8313	1.2842	1.0544	0.7899	0.9899	0.1140
LOC1001338	0.9381	1.0455	0.9830	0.9932	0.9899	0.0220
LOC1001298	0.9324	1.0611	1.0659	0.9004	0.9899	0.0430
OR51I2	0.8901	0.9377	1.1603	0.9716	0.9899	0.0592

FAM139A	1.0240	1.0005	1.0070	0.9283	0.9899	0.0212
LASS5	0.9472	1.3394	0.8511	0.8221	0.9899	0.1195
C6orf126	0.9151	1.0491	1.0658	0.9298	0.9900	0.0392
LOC1001312	0.9821	1.0126	1.0349	0.9303	0.9900	0.0226
LOC402634	0.9304	1.0669	1.1373	0.8252	0.9900	0.0697
LOC730262	0.9212	1.0598	1.0562	0.9226	0.9900	0.0393
C11orf65	0.8692	1.2136	1.0318	0.8452	0.9900	0.0853
IL24	0.9884	1.0837	0.9728	0.9150	0.9900	0.0350
LOC644733	0.8931	1.1144	1.1048	0.8475	0.9900	0.0697
LOC644360	0.8645	1.3415	0.9682	0.7857	0.9900	0.1230
LOC1001334	1.0522	0.9926	1.0004	0.9147	0.9900	0.0284
PRX	0.9061	1.0907	1.0346	0.9285	0.9900	0.0437
CD96	0.9631	1.1021	0.9720	0.9227	0.9900	0.0389
GCM2	0.9602	1.0620	1.0070	0.9309	0.9900	0.0287
PCDHGC5	0.9409	1.1089	1.0169	0.8932	0.9900	0.0471
LOC653560	0.9886	1.0690	1.0826	0.8198	0.9900	0.0604
DCC	0.9358	1.0839	1.0908	0.8495	0.9900	0.0589
LOC646471	0.9360	1.0525	1.0667	0.9048	0.9900	0.0408
LOC644145	0.8679	1.2059	0.9586	0.9277	0.9900	0.0744
LOC645955	0.9632	1.0218	0.9323	1.0427	0.9900	0.0256
GPR81	0.9479	1.1252	0.9707	0.9163	0.9900	0.0464
HSP90AB2P	0.9461	1.0818	1.0320	0.9003	0.9900	0.0410
LOC647149	0.9807	1.1014	0.9615	0.9165	0.9900	0.0395
MAPK4	0.9586	1.1030	0.9944	0.9040	0.9900	0.0420
LOC1001315	0.8579	1.2269	0.9629	0.9125	0.9900	0.0818
HEPH	0.9539	1.0381	1.0353	0.9329	0.9900	0.0273
LOC647307	0.8140	1.1748	1.2923	0.6791	0.9900	0.1453
TPRG1	0.9663	1.0474	1.0313	0.9152	0.9901	0.0305
LOC1001324	0.9757	1.0879	0.9784	0.9182	0.9901	0.0354
SPAG16	0.9144	1.0581	1.0709	0.9168	0.9901	0.0431
CEACAM16	0.9879	1.0571	0.9933	0.9220	0.9901	0.0276
AK1	0.7706	1.3017	1.1884	0.6996	0.9901	0.1497
LOC1001332	1.0072	1.0993	0.9084	0.9454	0.9901	0.0417
NECAB3	0.8799	1.4146	0.9524	0.7133	0.9901	0.1501
SNORD41	1.0238	1.0243	1.0051	0.9071	0.9901	0.0280
LY6D	0.9590	1.0850	1.0421	0.8742	0.9901	0.0466
ARSF	0.9569	1.0170	1.0590	0.9274	0.9901	0.0296
MIRLET7C	0.9074	1.1063	0.9681	0.9786	0.9901	0.0418
OR5L2	0.9495	0.9777	1.0187	1.0145	0.9901	0.0164
LOC1001329	0.9231	1.0935	1.0016	0.9422	0.9901	0.0383
GALR1	0.9404	1.0976	1.0959	0.8265	0.9901	0.0658
TRIM13	0.8976	1.1210	1.0699	0.8719	0.9901	0.0619
LOC440595	0.8857	1.1948	1.0126	0.8674	0.9901	0.0755
AOC3	0.9299	1.1166	1.0751	0.8388	0.9901	0.0644
LOC1001329	0.8985	1.1836	0.9872	0.8911	0.9901	0.0681
LOC401778	0.9588	1.1409	0.9359	0.9249	0.9901	0.0507

LOC646009	0.8823	1.0473	1.0688	0.9622	0.9901	0.0427
SNORD45C	0.9090	1.0466	1.0420	0.9628	0.9901	0.0332
LOC730417	0.9435	1.1165	0.9794	0.9211	0.9901	0.0438
MYO1H	0.9242	1.1728	0.9713	0.8921	0.9901	0.0630
LOC387820	0.8574	1.2793	0.9935	0.8303	0.9901	0.1028
MYBL2	1.0012	1.1262	0.9220	0.9110	0.9901	0.0496
LOC651635	0.9851	1.1462	0.9241	0.9051	0.9901	0.0548
CNIH3	0.8847	1.0715	1.0481	0.9562	0.9901	0.0431
TMEM95	0.9574	1.0012	1.0193	0.9827	0.9901	0.0132
LOC652799	0.9253	1.0905	1.0211	0.9236	0.9901	0.0405
OR1S2	1.0331	0.9727	1.0249	0.9299	0.9901	0.0241
LOC652856	0.9606	1.0069	1.0785	0.9146	0.9901	0.0350
LOC642504	0.9092	1.1521	0.9384	0.9609	0.9902	0.0550
FKBP8	0.8996	1.1872	1.0520	0.8218	0.9902	0.0812
LOC731282	0.8519	1.1049	0.9449	1.0589	0.9902	0.0570
AADAC	0.9973	1.0825	0.9780	0.9029	0.9902	0.0369
FLJ45513	0.9738	1.0063	0.9830	0.9975	0.9902	0.0073
LOC1001328	0.9361	1.0324	1.0359	0.9563	0.9902	0.0257
MIR1258	0.8653	1.0671	1.1809	0.8474	0.9902	0.0808
RCOR2	1.0916	1.1318	0.8956	0.8417	0.9902	0.0715
MIR539	0.8581	1.1176	1.0130	0.9721	0.9902	0.0537
ERCC-00003	0.9411	1.0792	1.0128	0.9276	0.9902	0.0351
CXorf20	0.9844	1.1066	0.9523	0.9174	0.9902	0.0412
LOC652529	0.9599	1.0309	1.0602	0.9098	0.9902	0.0341
CCDC140	0.9982	1.0573	1.0122	0.8931	0.9902	0.0347
SMG6	1.0101	1.1671	0.8919	0.8917	0.9902	0.0652
FMO6P	0.9404	1.1858	0.9619	0.8727	0.9902	0.0679
CYP24A1	0.9361	1.0257	1.0303	0.9688	0.9902	0.0228
COL19A1	0.9797	1.0163	1.0563	0.9086	0.9902	0.0314
TRAPPC5	0.8514	1.3325	0.9741	0.8029	0.9902	0.1196
LOC649217	1.0127	1.0369	1.0586	0.8526	0.9902	0.0468
LOC642413	0.9879	0.9955	1.0806	0.8968	0.9902	0.0376
LOC158825	0.9826	1.0520	0.9406	0.9857	0.9902	0.0230
LOC652049	0.8578	1.1065	1.0413	0.9553	0.9902	0.0539
SLC35F4	0.9323	1.1154	1.0046	0.9086	0.9902	0.0465
F5	0.9356	1.1386	0.9664	0.9204	0.9902	0.0504
LRRC70	0.9418	1.0738	1.0438	0.9016	0.9902	0.0409
LTB	0.9490	1.1272	0.9997	0.8851	0.9902	0.0513
LOC729385	0.9776	1.0592	0.9477	0.9765	0.9902	0.0240
LOC645908	0.9179	1.1425	1.0457	0.8549	0.9902	0.0644
LOC441424	1.0116	1.1139	0.9110	0.9245	0.9902	0.0469
RNF186	0.7564	1.0394	1.0863	1.0789	0.9903	0.0786
PRR10	0.8628	1.1080	1.0626	0.9275	0.9903	0.0572
C1orf14	0.9077	1.0690	1.0364	0.9479	0.9903	0.0376
LOC1001291	0.9045	1.0723	1.0974	0.8867	0.9903	0.0550
TPRXL	0.9815	0.9731	1.0701	0.9363	0.9903	0.0284

LOC730389	0.9258	1.0493	1.0457	0.9402	0.9903	0.0332
MIR1302-3	0.9480	1.0125	0.9913	1.0093	0.9903	0.0148
LOC442249	0.9660	1.1062	0.9912	0.8977	0.9903	0.0434
FBXO48	0.8977	1.0773	1.0356	0.9505	0.9903	0.0406
MIR93	0.9836	1.0796	0.9171	0.9808	0.9903	0.0335
LOC641947	0.8708	1.1254	1.1032	0.8617	0.9903	0.0718
MIR649	0.9271	1.1494	0.9529	0.9317	0.9903	0.0533
LOC643355	0.8485	1.0704	1.0443	0.9980	0.9903	0.0496
MIR346	0.9248	1.0907	1.0831	0.8626	0.9903	0.0572
MIR1251	0.9151	1.0885	0.9796	0.9780	0.9903	0.0360
TNFSF18	0.9151	0.9800	1.0366	1.0295	0.9903	0.0281
NRXN1	0.9603	1.0796	0.9984	0.9229	0.9903	0.0335
LOC647742	0.9656	1.0913	1.0001	0.9041	0.9903	0.0391
NHSL2	0.8879	1.1370	1.0155	0.9208	0.9903	0.0559
MIR1224	0.9106	1.1043	1.0288	0.9176	0.9903	0.0466
TMEM14E	0.9881	1.0529	0.9475	0.9728	0.9903	0.0225
FOXJ1	0.8726	1.0948	1.0843	0.9096	0.9903	0.0578
FAM160A1	0.8861	1.1669	1.0360	0.8723	0.9903	0.0695
C6orf57	0.7830	1.3290	1.0920	0.7574	0.9903	0.1361
S100B	0.9627	1.0360	0.9505	1.0121	0.9903	0.0202
ZNF235	1.0090	1.0518	1.0618	0.8389	0.9903	0.0518
CFHR1	0.8620	1.1876	1.0308	0.8811	0.9903	0.0758
LOC641990	0.8976	1.0375	1.1086	0.9177	0.9904	0.0501
C13orf26	0.9069	1.0566	1.1306	0.8674	0.9904	0.0620
CPT2	0.7765	1.3894	0.9895	0.8060	0.9904	0.1411
ZNF251	0.9822	1.1484	0.9269	0.9040	0.9904	0.0552
LOC648480	0.8192	1.0187	1.2314	0.8922	0.9904	0.0903
LOC647621	0.9444	1.1003	1.0406	0.8762	0.9904	0.0498
UNC5D	0.9637	1.0762	1.0640	0.8577	0.9904	0.0509
LOC643125	0.9211	1.1044	1.0557	0.8804	0.9904	0.0534
RHCG	0.9242	1.1313	0.9755	0.9306	0.9904	0.0483
C1orf87	0.9663	1.1311	0.9928	0.8713	0.9904	0.0537
LOC391205	0.9478	1.1321	0.9861	0.8955	0.9904	0.0508
APLNR	0.9473	1.1030	1.0059	0.9053	0.9904	0.0428
CCL20	0.8167	1.1188	1.1351	0.8910	0.9904	0.0804
FAM197Y2	0.9237	1.0795	1.0716	0.8868	0.9904	0.0498
LOC1001302	1.0178	1.0015	1.0491	0.8932	0.9904	0.0339
LOC728936	0.8449	1.1465	0.9748	0.9954	0.9904	0.0618
GCNT1	0.9043	1.2019	1.1229	0.7326	0.9904	0.1065
MLLT1	0.9307	1.1127	1.0561	0.8621	0.9904	0.0572
TMEM151A	0.9435	1.0902	1.0016	0.9264	0.9904	0.0369
FLJ46020	0.9831	1.0841	0.9340	0.9605	0.9904	0.0328
LOC728701	0.9387	0.9996	1.0392	0.9842	0.9904	0.0208
PAPSS1	0.9239	1.3025	0.9381	0.7972	0.9904	0.1087
LOC653257	1.0102	0.9589	1.0416	0.9510	0.9904	0.0215
THSD1	1.0172	1.0250	0.9649	0.9547	0.9904	0.0179

LOC648744	0.9994	1.1964	0.9323	0.8336	0.9904	0.0766
MIR744	1.0420	1.1412	0.8494	0.9291	0.9904	0.0639
KLK13	0.9489	1.0631	1.1061	0.8437	0.9904	0.0591
C1orf94	0.9133	1.1673	0.9665	0.9148	0.9905	0.0602
AZI2	0.9385	1.2254	1.0706	0.7274	0.9905	0.1055
LOC644668	0.9226	1.0919	1.0385	0.9089	0.9905	0.0446
NDST2	1.0019	1.1677	1.0178	0.7745	0.9905	0.0811
HSPA7	0.9193	1.1758	0.9075	0.9594	0.9905	0.0628
LOC648803	0.8901	1.0752	0.9577	1.0388	0.9905	0.0415
MIR10A	0.8883	1.0928	0.9696	1.0113	0.9905	0.0426
LOC1001326	0.9309	1.0978	0.9652	0.9680	0.9905	0.0368
KIAA1920	1.0202	0.9877	1.0477	0.9063	0.9905	0.0306
DPYSL5	0.8389	1.1060	1.1202	0.8968	0.9905	0.0718
LOC647579	0.9503	1.0146	1.0836	0.9135	0.9905	0.0374
ERCC-00164	1.0357	0.9965	1.0558	0.8740	0.9905	0.0408
ADAM6	0.9094	1.1705	1.0470	0.8351	0.9905	0.0743
LOC1001312	0.8782	1.1060	1.0229	0.9550	0.9905	0.0485
OR52I1	1.0222	1.1078	0.8812	0.9509	0.9905	0.0485
ELMO2	1.0453	1.1994	0.9406	0.7768	0.9905	0.0889
CEACAM19	0.9183	1.1124	0.9748	0.9567	0.9905	0.0423
TTC6	0.9422	1.1419	1.0044	0.8737	0.9905	0.0571
LOC643936	0.9066	1.0988	1.0853	0.8714	0.9905	0.0591
RBPJL	1.0462	1.0051	0.9692	0.9417	0.9905	0.0226
HCRTR1	0.9640	1.1218	1.0106	0.8658	0.9906	0.0531
OXNAD1	0.9616	1.0156	1.0899	0.8951	0.9906	0.0413
LOC728533	0.9321	1.4094	1.0286	0.5920	0.9906	0.1681
MIR181D	0.9619	0.9311	1.0237	1.0456	0.9906	0.0266
TRIM71	0.9824	1.0691	0.9888	0.9220	0.9906	0.0302
C9orf172	1.0174	1.0394	0.9702	0.9352	0.9906	0.0234
LOC642374	0.9646	1.1182	0.9889	0.8906	0.9906	0.0474
LOC1001345	0.9690	1.1229	1.0000	0.8705	0.9906	0.0520
TBPL2	1.0210	0.9792	1.0494	0.9127	0.9906	0.0297
KLHL2	0.9026	1.0962	1.1684	0.7951	0.9906	0.0860
LOC1001339	0.9486	1.0510	1.0761	0.8867	0.9906	0.0443
NRBP2	0.8612	1.2359	1.0683	0.7970	0.9906	0.1002
LOC343052	0.9215	1.0937	1.1038	0.8434	0.9906	0.0645
SLC40A1	0.9453	1.1026	1.0438	0.8707	0.9906	0.0515
DUX2	0.9514	1.0530	0.9586	0.9995	0.9906	0.0233
LOC644339	0.8621	1.0796	1.0886	0.9321	0.9906	0.0559
PDZD2	0.9959	1.1084	0.9937	0.8646	0.9906	0.0498
GXYLT2	0.9085	1.0237	1.0246	1.0058	0.9906	0.0277
OR2AE1	0.9570	1.0411	1.0218	0.9427	0.9906	0.0241
LOC648830	0.9487	1.0806	1.0311	0.9021	0.9906	0.0401
LOC645137	0.9623	1.0334	1.0947	0.8722	0.9906	0.0479
CCL17	0.9057	1.1071	1.0127	0.9371	0.9907	0.0448
MIR609	0.8838	1.1313	1.0593	0.8882	0.9907	0.0622

STAC	0.9711	1.0722	0.9301	0.9892	0.9907	0.0299
SKAP1	0.9764	1.0782	1.0267	0.8813	0.9907	0.0420
LOC649159	0.9534	1.1197	0.9720	0.9176	0.9907	0.0445
GPR149	0.9137	1.1237	1.0559	0.8694	0.9907	0.0596
HERC5	0.8479	1.3963	0.9916	0.7270	0.9907	0.1456
ERCC-00028	1.0188	1.0020	0.9908	0.9511	0.9907	0.0144
LOC1001299	0.9806	1.1119	1.0343	0.8360	0.9907	0.0582
ZNF764	1.2002	0.9269	1.0399	0.7957	0.9907	0.0858
IGLL3	0.9224	1.1264	1.0262	0.8878	0.9907	0.0539
PCDHGC4	0.9755	1.0868	1.0242	0.8763	0.9907	0.0444
DDTL	1.0019	1.1233	0.9456	0.8921	0.9907	0.0496
SLC13A1	0.9492	1.0129	1.0597	0.9411	0.9907	0.0281
FABP5	0.9287	1.1674	0.9309	0.9358	0.9907	0.0589
LOC643286	0.9355	1.1150	1.0225	0.8899	0.9907	0.0497
LOC1001325	0.6797	1.1159	1.3998	0.7675	0.9907	0.1657
LOC652259	0.8710	1.0891	1.1076	0.8952	0.9907	0.0624
FLJ20712	0.8664	1.2362	0.9416	0.9186	0.9907	0.0833
LOC654194	0.8330	1.2830	1.1008	0.7461	0.9907	0.1232
LOC646841	0.9306	1.0854	1.0927	0.8543	0.9908	0.0589
LOC441736	0.9636	1.0548	0.9995	0.9451	0.9908	0.0242
FLJ16779	0.9848	1.0617	0.9901	0.9265	0.9908	0.0277
DLX1	0.9376	1.1138	1.0294	0.8822	0.9908	0.0510
JAM3	0.9080	1.1858	1.0154	0.8540	0.9908	0.0731
LOC644341	0.9997	0.9355	1.0602	0.9677	0.9908	0.0266
C10orf113	0.9409	1.0117	1.0733	0.9373	0.9908	0.0324
LOC644331	0.7591	1.1632	1.1286	0.9123	0.9908	0.0951
SEMA4G	0.8819	1.0940	1.0847	0.9026	0.9908	0.0571
C1orf187	0.9325	1.0286	1.0235	0.9786	0.9908	0.0225
SOX21	1.0102	1.0623	1.0253	0.8654	0.9908	0.0432
LOC1001292	0.9808	1.0409	1.0280	0.9135	0.9908	0.0288
LOC284412	0.9159	1.1013	0.9761	0.9700	0.9908	0.0392
ATP1A2	0.9018	1.0504	1.0164	0.9946	0.9908	0.0318
OR51I1	0.9295	1.0686	1.0270	0.9381	0.9908	0.0340
CRYGS	0.9694	1.0454	0.9757	0.9728	0.9908	0.0182
PRSS22	1.0775	0.9942	1.0123	0.8792	0.9908	0.0413
LOC647276	0.9012	1.1264	1.0700	0.8656	0.9908	0.0635
MIR544	0.8846	1.0766	0.9946	1.0075	0.9908	0.0397
LOC650874	0.9238	1.0431	0.9968	0.9996	0.9908	0.0247
DDX53	0.9490	0.9797	1.0237	1.0109	0.9908	0.0167
LRIT1	0.9255	1.0979	0.9831	0.9568	0.9908	0.0376
GPR52	0.9051	1.1250	1.0283	0.9050	0.9908	0.0533
GRM4	0.9281	1.0020	1.0772	0.9560	0.9908	0.0326
PTHLH	0.9953	1.1044	1.0019	0.8619	0.9909	0.0497
KIAA0261	0.8554	1.3597	0.9337	0.8146	0.9909	0.1254
SLC22A12	0.9956	0.9614	1.0054	1.0009	0.9909	0.0100
SERPINE3	0.8774	1.0937	1.0564	0.9359	0.9909	0.0506

LPO	0.9422	1.0066	1.0130	1.0016	0.9909	0.0164
PRTN3	0.9776	1.0746	1.0272	0.8840	0.9909	0.0408
KC6	0.9200	1.1484	1.0200	0.8750	0.9909	0.0606
VTN	0.9832	1.0559	1.0958	0.8286	0.9909	0.0589
MIR802	0.9087	1.0548	0.9755	1.0246	0.9909	0.0319
MIR365-2	0.9533	1.0614	0.9932	0.9556	0.9909	0.0252
TCTEX1D1	0.9796	1.0153	1.0143	0.9544	0.9909	0.0147
IER5	0.9192	1.3292	0.9138	0.8015	0.9909	0.1160
FLJ23356	0.9490	1.1830	0.8986	0.9330	0.9909	0.0649
LOC392447	0.8412	1.1532	0.9794	0.9899	0.9909	0.0638
LOC729700	0.9860	1.0772	1.0096	0.8909	0.9909	0.0385
CTNNA3	0.9795	1.0508	1.0169	0.9165	0.9909	0.0288
FLJ21839	1.0380	0.9662	1.0562	0.9034	0.9909	0.0351
LOC1001300	0.9599	1.2026	0.9703	0.8310	0.9909	0.0773
NUAK2	0.8309	1.3096	0.9945	0.8288	0.9909	0.1131
LOC641994	0.9368	1.1340	1.0443	0.8487	0.9909	0.0622
OR5D16	0.9038	1.1245	1.0161	0.9194	0.9910	0.0510
LOC1001325	0.9478	1.1000	0.9920	0.9242	0.9910	0.0390
ARX	1.0405	1.1295	0.9767	0.8172	0.9910	0.0658
USH1C	0.9648	1.0564	1.0159	0.9268	0.9910	0.0285
LOC641716	0.9362	0.9782	1.0876	0.9620	0.9910	0.0333
MUSK	1.0090	1.1196	0.9808	0.8545	0.9910	0.0545
LOC645278	0.9187	1.0686	1.0889	0.8877	0.9910	0.0512
LOC283867	0.8944	1.1794	0.9910	0.8992	0.9910	0.0666
LOC641914	1.0260	1.0586	1.0664	0.8130	0.9910	0.0600
LOC729394	0.9227	1.0797	1.0643	0.8973	0.9910	0.0472
LOC650120	0.9619	1.1238	0.9605	0.9179	0.9910	0.0454
ITGAL	0.9744	1.0230	1.0834	0.8833	0.9910	0.0423
TMPRSS3	0.9717	1.1242	0.9816	0.8865	0.9910	0.0493
TMEM91	0.9943	1.1269	0.8940	0.9488	0.9910	0.0497
LOC732416	0.8115	1.0988	1.0553	0.9986	0.9910	0.0633
UBTD2	1.0329	1.1522	1.0133	0.7657	0.9910	0.0811
TP73L	0.9392	1.0969	0.9747	0.9533	0.9910	0.0360
LOC400419	0.9662	1.0075	0.9752	1.0152	0.9910	0.0120
LOC1001338	0.9964	1.0761	0.9775	0.9142	0.9910	0.0333
LOC646128	0.8739	1.0847	1.1231	0.8825	0.9910	0.0657
LOC553137	1.0118	1.0378	1.0094	0.9051	0.9910	0.0294
RASGRP3	1.0186	0.9962	1.0021	0.9473	0.9910	0.0154
LOC644749	0.9261	1.0702	1.0130	0.9549	0.9911	0.0320
KIN	0.9733	1.0557	0.9884	0.9468	0.9911	0.0232
SULT1C2	0.8852	1.1160	1.0343	0.9287	0.9911	0.0521
GGT3	0.9905	1.0723	0.9668	0.9347	0.9911	0.0294
LOC1001339	0.9139	1.0622	1.1105	0.8777	0.9911	0.0564
LOC649757	0.9681	1.0539	1.0681	0.8742	0.9911	0.0448
C21orf93	0.9010	1.0126	1.1541	0.8966	0.9911	0.0606
VMD2L3	0.8854	1.1151	1.0687	0.8952	0.9911	0.0590

FXYD2	0.9456	1.0675	1.0060	0.9452	0.9911	0.0292
KIAA1875	1.0149	1.1166	1.0207	0.8122	0.9911	0.0640
LOC650262	0.9116	1.0645	1.1213	0.8669	0.9911	0.0606
CRH	0.9764	1.0657	1.0203	0.9020	0.9911	0.0348
LOC643748	0.9293	1.0752	1.0327	0.9272	0.9911	0.0373
SNORD20	0.8689	1.1212	1.0421	0.9322	0.9911	0.0562
C6orf35	1.0139	1.0182	1.0735	0.8588	0.9911	0.0461
NUDT4P1	0.9205	1.0894	1.0071	0.9474	0.9911	0.0374
LOC644760	0.8690	1.1778	1.0628	0.8549	0.9911	0.0782
LOC651380	0.9364	1.2929	0.9823	0.7529	0.9911	0.1121
LOC650995	0.9766	1.2257	0.9359	0.8263	0.9911	0.0844
ZNF180	0.9233	1.0962	0.9655	0.9794	0.9911	0.0370
LOC1001335	0.9223	1.1785	0.8384	1.0254	0.9911	0.0732
MIR891B	0.9105	1.0657	1.0356	0.9528	0.9911	0.0360
LOC648521	0.9270	1.0249	1.0697	0.9429	0.9912	0.0339
ETV5	0.8912	1.1417	1.0118	0.9199	0.9912	0.0564
LOC642675	1.0141	1.1579	0.9512	0.8414	0.9912	0.0661
ADH4	0.9248	1.1100	0.9964	0.9336	0.9912	0.0427
MSH3	0.8685	1.2127	1.1546	0.7289	0.9912	0.1153
C3orf35	0.9627	1.1149	0.9806	0.9066	0.9912	0.0441
LOC653917	1.0057	1.0714	1.0632	0.8245	0.9912	0.0575
LOC645515	0.9359	1.2371	1.0938	0.6980	0.9912	0.1155
CD28	0.9050	1.0056	1.0698	0.9844	0.9912	0.0340
SLC16A5	0.9820	1.0446	1.0292	0.9091	0.9912	0.0305
ARMCX6	0.8585	1.3452	1.0403	0.7208	0.9912	0.1349
LOC253482	1.0120	1.1067	0.9375	0.9087	0.9912	0.0442
GIGYF2	0.8258	1.1292	1.0821	0.9278	0.9912	0.0699
LOC642943	0.8776	1.0139	1.1047	0.9687	0.9912	0.0473
MGAT4C	0.9566	1.0143	1.0703	0.9237	0.9912	0.0323
RAB3C	0.8989	1.0673	1.0116	0.9872	0.9912	0.0350
LOC644023	0.8998	1.0634	1.0958	0.9059	0.9912	0.0515
DKK1	0.9279	1.1336	1.0107	0.8928	0.9913	0.0535
LOC649853	0.9278	1.1963	0.9653	0.8756	0.9913	0.0708
OR4S2	0.9451	1.0699	1.0677	0.8824	0.9913	0.0466
LOC645287	1.0596	1.0261	0.9668	0.9125	0.9913	0.0325
LOC653047	0.9594	1.1103	0.9944	0.9010	0.9913	0.0441
CDH5	0.9377	1.0887	1.0358	0.9029	0.9913	0.0430
FLJ34969	0.8741	1.0898	1.1437	0.8575	0.9913	0.0734
C10orf79	0.9050	1.0705	1.0884	0.9013	0.9913	0.0510
PLEKHG4B	0.9699	1.1073	0.9804	0.9076	0.9913	0.0419
RFTN1	0.9186	1.0540	1.0303	0.9621	0.9913	0.0311
IGF1	0.9316	1.0837	1.0391	0.9108	0.9913	0.0417
LOC643371	0.8998	1.0465	1.0472	0.9716	0.9913	0.0353
AKAP11	0.8651	1.2250	1.0527	0.8223	0.9913	0.0926
LOC654114	0.9780	1.2285	1.0586	0.7001	0.9913	0.1102
LOC286478	0.8990	1.1208	1.0381	0.9072	0.9913	0.0537

TNFSF13B	0.8948	1.2482	0.9545	0.8677	0.9913	0.0875
LOC645652	0.9060	1.1401	1.0339	0.8851	0.9913	0.0595
HLA-DMA	0.8639	1.2417	1.0851	0.7744	0.9913	0.1060
EFCAB2	0.9291	1.1467	0.9471	0.9422	0.9913	0.0520
UCP3	0.9546	1.1578	0.9607	0.8921	0.9913	0.0576
PPP1R2P3	1.0127	0.9643	0.9718	1.0164	0.9913	0.0135
LOC1001329	0.9518	1.1094	1.0571	0.8469	0.9913	0.0582
ARSH	0.8777	1.1221	1.0478	0.9177	0.9913	0.0567
LOC648816	1.0139	1.0339	1.0352	0.8823	0.9913	0.0367
SNORA31	0.9407	1.1022	1.0196	0.9028	0.9913	0.0443
LOC653751	0.8707	1.0346	1.0103	1.0497	0.9913	0.0410
LOC647942	0.9778	1.0880	0.9217	0.9779	0.9913	0.0348
LOC646805	0.9029	1.0900	1.0575	0.9150	0.9913	0.0481
KRT18P40	0.9446	1.1001	1.0028	0.9178	0.9913	0.0404
MGC23284	0.9875	1.1730	0.9896	0.8152	0.9913	0.0730
HMCN1	0.9857	0.9973	0.9802	1.0022	0.9914	0.0051
SDC4	0.9444	1.2581	0.8989	0.8639	0.9914	0.0904
NPY2R	0.9549	1.0569	1.0205	0.9331	0.9914	0.0287
LOC401052	0.9468	1.2184	1.0064	0.7938	0.9914	0.0879
SNORD114-8	1.0294	1.0874	0.9470	0.9015	0.9914	0.0415
LOC1001302	0.9140	1.0952	1.0367	0.9196	0.9914	0.0447
PRTG	0.9434	1.0727	1.0336	0.9157	0.9914	0.0370
LOC1001332	0.8797	1.2831	0.9978	0.8049	0.9914	0.1050
LOC1001292	0.9139	1.1109	1.1506	0.7901	0.9914	0.0847
PCSK1	0.8862	1.0803	1.0471	0.9519	0.9914	0.0444
TAF8	1.0081	1.0874	0.9719	0.8980	0.9914	0.0394
BARX2	0.8873	1.1583	1.0373	0.8826	0.9914	0.0662
ZNF678	0.9846	1.0471	1.0339	0.9000	0.9914	0.0333
LCE2A	0.9064	1.0973	0.9871	0.9748	0.9914	0.0395
LOC647536	0.8972	1.0672	1.0888	0.9124	0.9914	0.0503
LOC650024	0.9481	1.0427	1.0851	0.8896	0.9914	0.0444
ZNF167	0.9635	1.0720	1.0540	0.8760	0.9914	0.0452
CAPS2	0.8172	1.2468	1.0408	0.8607	0.9914	0.0980
FLJ16323	0.8583	1.1367	1.0309	0.9396	0.9914	0.0599
ZNF560	0.9221	0.9853	1.1823	0.8759	0.9914	0.0675
MIR297	0.9358	1.0979	0.9934	0.9384	0.9914	0.0379
LOC728034	0.9008	1.1528	1.0755	0.8364	0.9914	0.0738
PPEF2	0.9462	1.0508	1.0592	0.9093	0.9914	0.0375
ACSM1	0.9493	1.0486	1.0266	0.9410	0.9914	0.0271
PXK	0.9694	1.1413	0.9914	0.8635	0.9914	0.0572
TBR1	0.9316	1.0925	1.0110	0.9305	0.9914	0.0386
LOC120376	0.9539	1.1493	0.9580	0.9045	0.9914	0.0540
LOC441601	0.9535	0.9685	1.1117	0.9319	0.9914	0.0408
LINGO2	0.9741	1.0203	1.0187	0.9525	0.9914	0.0168
DEFB109	0.8805	1.1545	0.9880	0.9427	0.9914	0.0587
LOC646266	0.9366	1.0538	1.0794	0.8960	0.9914	0.0445

LOC1001329	0.9962	0.9825	1.0870	0.9000	0.9914	0.0383
LOC440125	0.8789	1.0694	1.0516	0.9658	0.9914	0.0438
NTN5	1.0477	1.0991	0.9348	0.8841	0.9914	0.0496
LOC652733	0.8980	1.0738	1.1100	0.8839	0.9914	0.0586
MGC15634	0.9186	1.1881	0.9793	0.8798	0.9914	0.0687
GAFA1	0.9850	1.0787	0.9927	0.9093	0.9914	0.0346
LOC1001319	0.9364	1.0820	0.9895	0.9578	0.9914	0.0321
POU1F1	0.9592	1.0489	1.0116	0.9461	0.9914	0.0238
COX6A1	1.0172	1.1941	1.1151	0.6395	0.9914	0.1228
LOC652781	0.9786	1.0685	1.0176	0.9012	0.9915	0.0353
PNPLA7	0.9181	1.3036	0.9652	0.7789	0.9915	0.1113
LOC285620	0.9583	1.0682	1.0661	0.8733	0.9915	0.0470
LOC642134	0.9747	1.0963	0.9923	0.9026	0.9915	0.0400
C10orf59	0.9667	1.1027	1.0369	0.8596	0.9915	0.0520
TBC1D10C	0.9183	1.0281	1.0186	1.0010	0.9915	0.0250
TMOD4	0.9192	1.1640	0.9053	0.9775	0.9915	0.0596
CRIP3	0.9175	1.1272	0.9631	0.9581	0.9915	0.0464
KIAA2018	0.9654	1.0842	1.0599	0.8565	0.9915	0.0518
EFCAB4B	0.9176	1.1348	1.0862	0.8275	0.9915	0.0718
TEX9	0.9255	1.0857	1.1143	0.8405	0.9915	0.0653
ADAMTSL3	0.9576	0.9379	1.0997	0.9709	0.9915	0.0367
LOC645294	1.0183	1.0532	0.9479	0.9467	0.9915	0.0265
TBC1D3H	0.9831	1.0479	1.0144	0.9208	0.9915	0.0270
PCDH7	0.9772	1.0103	1.0380	0.9407	0.9915	0.0210
FLJ35776	0.9387	1.1351	1.0001	0.8922	0.9915	0.0527
HESX1	0.9269	1.0846	1.0051	0.9495	0.9915	0.0351
SNORA27	0.7274	1.1913	1.1052	0.9423	0.9916	0.1021
LOC728961	0.9449	1.0176	1.0052	0.9985	0.9916	0.0161
LOC729835	0.9579	1.0554	1.0256	0.9273	0.9916	0.0296
TRPM3	0.9257	1.0785	1.0355	0.9266	0.9916	0.0388
SLC26A1	0.9452	1.0759	1.0345	0.9108	0.9916	0.0383
OR8D2	0.8852	1.0026	0.9684	1.1101	0.9916	0.0466
GHRHR	0.9386	1.1015	1.0196	0.9065	0.9916	0.0437
FLJ41821	0.9503	1.0504	1.0514	0.9142	0.9916	0.0350
LOC1001294	0.8769	1.2530	1.0895	0.7470	0.9916	0.1121
LOC652495	0.9270	1.0293	1.0617	0.9483	0.9916	0.0321
LOC1001282	0.9194	1.0853	1.0948	0.8668	0.9916	0.0579
JCLN	0.9744	1.0651	1.0027	0.9243	0.9916	0.0294
SH3MD4	0.9109	1.0882	0.9948	0.9725	0.9916	0.0367
ZFP57	0.9913	1.1341	0.9471	0.8939	0.9916	0.0515
STK32A	0.9652	0.9814	1.0129	1.0069	0.9916	0.0111
LOC641753	0.9344	1.0756	1.0304	0.9261	0.9916	0.0367
LOC649293	0.9999	1.1132	1.0059	0.8476	0.9916	0.0546
TMEM75	0.9163	1.0206	1.0957	0.9340	0.9916	0.0415
AP4E1	1.0012	1.2819	0.9165	0.7670	0.9916	0.1082
ZNF382	0.8991	1.1047	1.0652	0.8975	0.9916	0.0545

LOC642948	0.9783	1.0476	1.0494	0.8913	0.9916	0.0373
ITPRIPL1	0.9515	1.0796	1.0367	0.8988	0.9917	0.0408
MIR663B	0.9423	1.0318	1.1112	0.8813	0.9917	0.0504
GPD2	0.9744	1.0781	0.9795	0.9347	0.9917	0.0305
LOC646082	0.9158	1.0596	1.0560	0.9353	0.9917	0.0384
LOC651751	0.8638	1.1988	0.9798	0.9243	0.9917	0.0730
OR1Q1	1.0244	1.1134	0.9480	0.8809	0.9917	0.0501
LOC340888	0.9032	1.0783	1.0639	0.9213	0.9917	0.0461
HTR7	0.9359	1.1216	1.0111	0.8981	0.9917	0.0493
PVALB	0.9542	1.1695	0.9492	0.8939	0.9917	0.0608
LOC728257	0.9487	1.1125	1.0091	0.8966	0.9917	0.0464
LOC728003	1.0380	0.9908	1.0360	0.9020	0.9917	0.0318
SIRT4	0.9921	1.1573	0.9848	0.8327	0.9917	0.0663
TSGA13	0.9220	1.0263	1.1146	0.9039	0.9917	0.0490
SLC22A15	0.9671	1.0772	1.0593	0.8633	0.9917	0.0491
LOC642250	0.9524	1.0561	1.0773	0.8811	0.9917	0.0459
LOC1001345	1.0507	1.0547	1.0302	0.8313	0.9917	0.0537
HIST1H2AM	0.8554	1.4194	0.9355	0.7567	0.9917	0.1472
LOC1001279	0.9688	1.0430	1.0434	0.9117	0.9917	0.0319
LOC651102	0.9238	1.0748	1.0604	0.9079	0.9917	0.0440
MIR504	0.9613	1.0356	1.0655	0.9045	0.9917	0.0364
ATP4B	0.9365	1.0717	1.0566	0.9022	0.9917	0.0425
LOC644663	0.9754	1.0595	1.0612	0.8708	0.9917	0.0450
LOC649504	0.9850	1.0543	0.9806	0.9471	0.9917	0.0225
ISX	0.9828	1.1504	0.9429	0.8909	0.9917	0.0561
SNX24	0.9886	1.0910	1.0308	0.8566	0.9918	0.0497
LOC645871	0.9904	1.0375	0.9996	0.9395	0.9918	0.0202
CYP4F3	0.9447	1.0382	1.0983	0.8859	0.9918	0.0474
AOAH	0.9620	1.1400	0.9615	0.9036	0.9918	0.0513
FLJ27465	0.9480	1.0718	1.0381	0.9092	0.9918	0.0379
AKR1C2	0.9467	1.0588	1.0269	0.9347	0.9918	0.0303
PEX3	0.9214	1.0744	1.0053	0.9660	0.9918	0.0324
CHST11	0.9695	1.0642	1.1957	0.7378	0.9918	0.0965
HYI	0.8856	1.1088	1.0138	0.9590	0.9918	0.0470
LOC644574	0.8172	1.1963	1.0248	0.9288	0.9918	0.0803
LOC729898	0.9317	1.0976	0.9995	0.9384	0.9918	0.0384
LOC731017	0.9880	1.1444	0.9939	0.8408	0.9918	0.0620
KIAA0100	1.0193	1.1417	1.0943	0.7119	0.9918	0.0966
LOC645066	0.9060	1.0736	1.0400	0.9476	0.9918	0.0391
PKD2	0.9991	1.1209	1.0142	0.8330	0.9918	0.0595
LPAR4	0.8978	1.1910	0.9901	0.8884	0.9918	0.0703
KIR3DP1	0.9328	1.0759	0.9444	1.0141	0.9918	0.0333
C13orf35	0.9970	1.0737	1.0234	0.8732	0.9918	0.0426
DPRX	0.9484	0.9885	1.0286	1.0019	0.9918	0.0167
RNASE9	0.8293	1.1488	1.0650	0.9242	0.9918	0.0713
LOC650422	0.9268	1.1570	0.9487	0.9349	0.9918	0.0552

RECK	0.9890	1.1453	0.9767	0.8563	0.9918	0.0593
C17orf74	0.9070	1.1779	0.9545	0.9279	0.9918	0.0628
MIR96	0.9347	1.0255	1.0382	0.9689	0.9918	0.0243
C7	0.9632	1.0740	0.9438	0.9864	0.9918	0.0287
LOC400464	0.8595	1.0556	1.0844	0.9678	0.9918	0.0506
LOC1001325	0.9762	1.0896	1.0470	0.8546	0.9919	0.0514
FPR2	0.9318	1.0420	1.0293	0.9644	0.9919	0.0263
LOC653510	0.9257	1.0299	1.1369	0.8749	0.9919	0.0581
SNORD114-1	0.9662	0.9750	1.1170	0.9092	0.9919	0.0442
EN1	0.9271	0.9962	1.0983	0.9459	0.9919	0.0383
ST7OT4	0.9833	1.0938	0.9701	0.9202	0.9919	0.0366
LOC645585	0.9176	1.0706	0.9369	1.0425	0.9919	0.0380
SCARNA1	1.0158	1.0325	1.0770	0.8422	0.9919	0.0515
MYEOV2	1.0302	1.1285	0.9775	0.8313	0.9919	0.0620
KLRC2	0.9646	1.0848	1.0023	0.9158	0.9919	0.0357
UTF1	1.0759	0.9823	1.0131	0.8962	0.9919	0.0374
LOC1001310	0.9233	1.0529	1.0054	0.9860	0.9919	0.0268
LOC1001289	0.9507	1.0963	1.0278	0.8928	0.9919	0.0445
GAL3ST1	0.9235	1.0781	1.0086	0.9573	0.9919	0.0336
LOC645128	0.8912	1.2458	0.9967	0.8338	0.9919	0.0911
CCDC64B	0.9821	1.0772	0.9946	0.9136	0.9919	0.0336
STEAP1	0.8597	1.3808	1.0302	0.6968	0.9919	0.1464
MIR548I1	0.9407	1.0962	1.0249	0.9057	0.9919	0.0428
LOC284371	1.0160	1.1249	0.9909	0.8358	0.9919	0.0596
CSF1R	0.9056	1.0153	1.0715	0.9753	0.9919	0.0349
GPR37L1	0.9336	1.1008	0.9950	0.9383	0.9919	0.0389
CYP19A1	0.9040	1.0981	1.0153	0.9503	0.9919	0.0421
CASRL1	0.9693	1.0176	1.0307	0.9500	0.9919	0.0192
ZNF148	0.7838	1.5526	0.9817	0.6496	0.9919	0.1990
CCL2	0.9222	1.1157	1.0664	0.8634	0.9919	0.0593
LOC729040	0.9874	1.0842	0.9240	0.9721	0.9919	0.0336
OLFM2	1.0031	1.0557	1.0542	0.8548	0.9919	0.0473
LIM2	0.9934	1.1963	0.9882	0.7898	0.9919	0.0830
LOC728022	0.9470	1.1301	0.9782	0.9125	0.9919	0.0480
FRMPD2	0.9785	1.0052	1.0679	0.9163	0.9919	0.0314
AKAP4	0.9919	1.1237	1.0055	0.8467	0.9920	0.0567
GHSR	0.9252	1.0818	1.0539	0.9070	0.9920	0.0443
GPR141	0.8490	1.1086	1.0882	0.9222	0.9920	0.0634
SCNN1D	0.9022	1.2515	0.9228	0.8913	0.9920	0.0868
LOC1001290	0.9009	1.1238	1.0278	0.9155	0.9920	0.0523
LOC646960	1.0323	1.0085	1.0317	0.8954	0.9920	0.0327
FLJ46838	0.8710	1.0180	1.1355	0.9436	0.9920	0.0565
LOC645307	0.8911	1.0702	1.1079	0.8988	0.9920	0.0566
MLX	0.9300	1.4306	0.8961	0.7112	0.9920	0.1539
LOC1001328	0.9997	1.0233	0.9630	0.9820	0.9920	0.0128
LOC648245	0.9608	1.1606	0.9801	0.8664	0.9920	0.0615

LOC1001280	0.9574	1.0996	1.0040	0.9069	0.9920	0.0410
LOC643734	0.9768	1.1120	0.9962	0.8830	0.9920	0.0470
ZNF253	0.9831	1.0989	0.9806	0.9054	0.9920	0.0399
LOC1001319	0.8848	1.0858	1.0310	0.9664	0.9920	0.0433
ERCC-00031	0.9228	1.0974	1.0207	0.9273	0.9920	0.0418
HPYR1	1.0636	1.0878	1.0002	0.8164	0.9920	0.0614
LOC731969	0.8917	1.1364	0.9575	0.9826	0.9920	0.0518
LOC1001282	0.9044	1.1474	0.9706	0.9458	0.9920	0.0535
SNORA51	0.9688	1.0490	1.0393	0.9111	0.9920	0.0324
DMKN	0.9630	1.0140	1.0555	0.9356	0.9920	0.0267
LOC1001289	0.8746	1.0350	1.0846	0.9739	0.9921	0.0452
PAOX	0.8867	1.1645	1.0573	0.8597	0.9921	0.0722
LOC648718	1.0035	1.0434	1.1477	0.7736	0.9921	0.0789
KIAA0317	0.9203	1.0911	0.9942	0.9627	0.9921	0.0363
LOC1001281	0.8864	1.2022	1.1105	0.7690	0.9921	0.0996
LYRM1	0.9304	1.4241	0.9554	0.6583	0.9921	0.1590
LOC1001295	0.9269	1.0291	1.1166	0.8957	0.9921	0.0503
CSF3R	0.9030	1.0809	1.0628	0.9215	0.9921	0.0464
VENTXP1	0.9489	1.1131	1.0272	0.8792	0.9921	0.0504
LOC652533	1.0094	1.1301	0.9907	0.8380	0.9921	0.0599
NMNAT3	0.8852	1.2053	1.0375	0.8402	0.9921	0.0827
ZNF90	0.9537	1.1744	0.9525	0.8876	0.9921	0.0627
MIR617	0.8130	1.0970	1.0977	0.9606	0.9921	0.0678
PREX1	0.8972	1.1346	1.0373	0.8992	0.9921	0.0577
LOC729065	0.9812	1.1841	0.9731	0.8300	0.9921	0.0728
LOC1001337	0.8828	1.0781	1.1519	0.8555	0.9921	0.0728
SNORD66	0.9775	1.0977	1.0134	0.8798	0.9921	0.0451
LPAR5	1.0353	0.9557	0.9998	0.9775	0.9921	0.0170
ADAMTS2	0.9666	1.0597	1.0060	0.9360	0.9921	0.0267
DYNC2LI1	0.9280	1.2942	0.9138	0.8325	0.9921	0.1029
EFCAB7	0.9344	1.1737	1.0739	0.7864	0.9921	0.0843
LOC1001283	0.9502	1.0789	0.9414	0.9979	0.9921	0.0315
LOC729501	0.9810	1.0543	1.0278	0.9054	0.9921	0.0327
DKFZp434I1C	1.0302	1.1230	1.0128	0.8024	0.9921	0.0677
YIPF4	0.9250	1.1309	1.1478	0.7648	0.9921	0.0911
BARHL1	0.8430	1.1876	1.0316	0.9062	0.9921	0.0760
CFH	0.9438	1.1048	1.0182	0.9017	0.9921	0.0446
IL4	0.9728	1.0426	1.0413	0.9118	0.9921	0.0313
NBPF7	0.9762	1.0949	0.9939	0.9035	0.9921	0.0394
MIR320B1	0.9532	1.0347	0.9863	0.9944	0.9921	0.0168
LOC649661	0.8668	1.1978	1.0750	0.8289	0.9921	0.0873
LOC642656	0.9536	1.0753	1.0249	0.9148	0.9921	0.0359
NCRNA0009	1.0024	1.0537	1.0147	0.8977	0.9921	0.0333
MMP23B	0.8570	1.0739	1.0988	0.9389	0.9921	0.0571
LOC389748	0.9561	1.0300	1.0571	0.9253	0.9921	0.0309
LOC1001312	0.9662	1.1054	1.0493	0.8477	0.9921	0.0560

LOC643369	1.0250	1.0298	1.0046	0.9092	0.9922	0.0282
EZH1	0.9514	1.1750	0.9723	0.8700	0.9922	0.0648
FRMPD2L2	0.9299	1.0834	1.0115	0.9439	0.9922	0.0352
ASAH2	0.9859	1.1212	0.9462	0.9154	0.9922	0.0454
LOC1001285	0.9214	1.1528	1.0437	0.8508	0.9922	0.0667
LRRN4	0.9260	1.0626	1.0208	0.9594	0.9922	0.0306
FBP2	0.9040	1.0480	1.0015	1.0152	0.9922	0.0310
FLJ45537	0.9860	1.1213	0.9430	0.9184	0.9922	0.0452
SRA1	0.9786	1.2399	0.9610	0.7892	0.9922	0.0930
VNN3	0.9519	1.0529	1.0585	0.9054	0.9922	0.0379
MED18	0.9493	1.0446	1.1521	0.8228	0.9922	0.0700
CAPN11	0.8486	1.0816	1.0316	1.0070	0.9922	0.0503
LOC644168	0.9638	1.2516	0.9060	0.8475	0.9922	0.0896
LOC1001308	0.9065	1.0185	1.0471	0.9968	0.9922	0.0304
FCHSD1	0.9199	1.1104	1.0752	0.8634	0.9922	0.0596
SAA4	0.8916	1.0324	1.0405	1.0043	0.9922	0.0344
LOC390414	1.0240	0.9611	1.0319	0.9519	0.9922	0.0208
PRSS38	0.9666	1.0702	1.0235	0.9087	0.9922	0.0350
LOC341689	0.9811	1.0446	1.0542	0.8890	0.9922	0.0380
OR5M8	0.9725	1.0501	0.9997	0.9466	0.9922	0.0221
FRZB	0.9570	1.0943	1.0163	0.9013	0.9922	0.0413
BCL11A	0.9146	1.0995	1.0121	0.9428	0.9922	0.0412
ITK	0.8985	1.0684	1.0555	0.9466	0.9922	0.0415
TGIF2LX	0.8771	1.1171	1.0852	0.8896	0.9922	0.0633
ZC3H7B	0.8981	1.2069	0.9697	0.8942	0.9922	0.0736
MIR604	0.8064	1.1937	1.0470	0.9219	0.9923	0.0832
FDPSL2A	0.8951	1.1111	0.9069	1.0559	0.9923	0.0539
FLJ38379	0.9601	1.0412	1.0121	0.9557	0.9923	0.0207
AGFG2	0.9084	1.2610	1.0160	0.7837	0.9923	0.1014
TRIP11	1.0424	1.3338	0.8349	0.7580	0.9923	0.1287
LOC729919	0.9041	1.1232	1.0267	0.9150	0.9923	0.0517
LOC392713	0.9308	1.1101	0.9970	0.9313	0.9923	0.0422
C1orf49	0.9898	1.1047	0.9344	0.9402	0.9923	0.0395
SF1	0.9467	1.0427	1.1264	0.8533	0.9923	0.0591
LOC653667	0.9425	1.0501	1.0492	0.9273	0.9923	0.0333
PCSK2	1.0099	1.0624	0.9636	0.9332	0.9923	0.0282
FLJ30851	0.9974	1.0563	0.9866	0.9288	0.9923	0.0261
LOC1001317	1.0058	1.0107	1.1168	0.8359	0.9923	0.0581
LOC731102	1.0502	0.9625	0.9977	0.9588	0.9923	0.0212
ADC	0.9667	1.1791	0.8798	0.9436	0.9923	0.0649
OR10G8	0.9252	1.0077	1.0779	0.9583	0.9923	0.0332
SLC22A14	0.9808	1.0602	0.9159	1.0123	0.9923	0.0302
HELT	0.9271	1.1534	1.0527	0.8360	0.9923	0.0697
SIGLEC16	0.8890	1.1107	1.0599	0.9096	0.9923	0.0549
LOC1001304	0.9403	1.1438	0.9554	0.9298	0.9923	0.0508
LOC646799	0.9807	1.0396	1.0714	0.8775	0.9923	0.0426

HERPUD2	0.9080	1.1534	1.0787	0.8292	0.9923	0.0748
THNSL2	0.9948	1.2766	1.0204	0.6775	0.9923	0.1227
MASP1	0.9872	1.0267	1.0898	0.8656	0.9923	0.0472
SPRED2	0.9287	1.1204	1.0447	0.8756	0.9923	0.0554
LOC1001293	0.9319	1.0716	1.0021	0.9637	0.9923	0.0301
COPB2	0.8302	1.3974	0.9764	0.7654	0.9923	0.1420
LOC642293	0.9599	1.1209	0.9046	0.9840	0.9923	0.0460
C11orf80	0.9157	1.3620	0.9662	0.7256	0.9924	0.1337
B3GALT5	0.9474	1.0680	1.0028	0.9513	0.9924	0.0282
PLG	0.9089	1.1316	1.0622	0.8666	0.9924	0.0626
LOC1001344	0.9014	1.1974	0.9970	0.8736	0.9924	0.0733
LOC650749	0.9107	1.1351	1.0327	0.8910	0.9924	0.0570
WWTR1	0.8721	1.1141	1.0613	0.9220	0.9924	0.0570
LOC651619	0.9887	1.2401	0.9132	0.8275	0.9924	0.0889
NACAD	0.9337	1.1424	1.0385	0.8548	0.9924	0.0626
GNG2	0.9103	1.0526	1.0572	0.9494	0.9924	0.0370
FAM23A	1.0353	1.0048	0.9773	0.9520	0.9924	0.0179
LOC645626	0.9059	1.0834	1.0346	0.9456	0.9924	0.0406
LOC1001288	0.9138	1.1399	1.0336	0.8823	0.9924	0.0590
LOC644386	1.0137	1.0226	1.0392	0.8940	0.9924	0.0332
LOC1001342	0.9134	1.1336	0.9798	0.9428	0.9924	0.0490
LOC342994	0.9359	1.0575	1.0602	0.9161	0.9924	0.0386
LOC646383	0.8821	1.1342	1.0510	0.9023	0.9924	0.0604
OR11A1	0.9644	1.0520	0.9675	0.9857	0.9924	0.0204
LOC646498	0.9531	1.0742	1.0391	0.9033	0.9924	0.0391
WDR3	0.9860	1.0007	1.0752	0.9077	0.9924	0.0343
LDHAL6A	0.7665	1.2537	0.9681	0.9813	0.9924	0.1000
TAAR9	0.9676	1.0655	1.0279	0.9087	0.9924	0.0344
ARHGEF6	0.9906	1.1330	1.0033	0.8427	0.9924	0.0594
KRTAP21-2	1.0135	0.9858	1.0826	0.8878	0.9924	0.0404
LOC651723	1.0448	1.0267	0.9152	0.9830	0.9924	0.0288
LOC88523	0.8509	1.3743	0.9763	0.7682	0.9924	0.1343
LOC285074	0.9249	1.3723	0.8847	0.7878	0.9924	0.1299
ZDHHC14	0.8766	1.2588	1.0992	0.7351	0.9924	0.1162
LOC643368	0.9149	1.1813	1.0088	0.8648	0.9924	0.0697
LOC728539	0.9610	1.0209	1.0432	0.9447	0.9924	0.0236
ZAP70	1.0005	1.0127	0.9474	1.0092	0.9924	0.0152
LOC647190	0.9082	1.2301	1.0078	0.8238	0.9924	0.0877
DKFZP564C1	0.8921	1.1360	0.9947	0.9469	0.9925	0.0522
DKFZp686A1	0.9791	0.9721	1.0966	0.9220	0.9925	0.0370
SNX22	0.8569	1.1696	1.2858	0.6575	0.9925	0.1438
PRG3	0.9928	1.0935	0.9726	0.9109	0.9925	0.0379
CNTN5	0.9549	1.0627	1.1004	0.8518	0.9925	0.0561
WWP1	0.8586	1.4184	1.0647	0.6281	0.9925	0.1677
MIR1203	0.9634	1.0506	1.0065	0.9493	0.9925	0.0229
BAIAP2	1.0316	1.1839	1.0214	0.7330	0.9925	0.0941

LOC441018	0.9911	1.0987	0.9620	0.9181	0.9925	0.0384
C4orf11	1.0025	1.0858	1.0933	0.7884	0.9925	0.0711
RUNDC3B	0.9001	1.0107	1.1121	0.9471	0.9925	0.0459
CCDC36	0.9185	1.1954	1.0353	0.8208	0.9925	0.0806
SLC7A14	0.9826	0.9639	1.1033	0.9202	0.9925	0.0392
MIR585	0.8825	1.1847	0.9921	0.9107	0.9925	0.0682
INA	0.9638	1.1532	0.9793	0.8738	0.9925	0.0584
LOC650796	0.8212	1.1950	1.1128	0.8410	0.9925	0.0948
LOC1001322	0.9786	1.0041	1.0873	0.9000	0.9925	0.0386
LOC653526	0.9666	1.1061	1.0149	0.8826	0.9925	0.0467
LOC442381	0.9962	1.0834	0.9547	0.9359	0.9925	0.0328
OR10G7	0.9337	1.0754	1.0075	0.9536	0.9925	0.0317
COX11P	0.8790	1.2470	0.9682	0.8761	0.9925	0.0875
LOC1001309	0.8556	1.2010	1.0267	0.8869	0.9925	0.0788
SLC10A2	0.9438	1.0373	1.0586	0.9305	0.9926	0.0324
LOC401280	0.8972	1.2029	0.9645	0.9056	0.9926	0.0717
LOC440041	0.9026	1.1457	1.0222	0.8997	0.9926	0.0585
KCNQ1DN	0.9760	1.0494	0.9856	0.9593	0.9926	0.0197
PLA2G4E	1.0099	1.0373	1.0466	0.8764	0.9926	0.0395
C17orf85	0.8269	1.3545	1.0014	0.7875	0.9926	0.1293
LOC155060	1.0217	1.0639	0.9840	0.9006	0.9926	0.0347
DGCR9	0.9061	1.1360	0.9836	0.9447	0.9926	0.0503
OR4D10	0.9905	1.0362	1.0018	0.9419	0.9926	0.0195
LOC641908	0.9473	1.1772	0.8933	0.9525	0.9926	0.0630
OR2H1	0.9485	1.1303	1.0234	0.8682	0.9926	0.0558
LOC651872	0.9532	1.0772	1.0664	0.8736	0.9926	0.0486
LOC644092	0.9405	1.0610	1.0269	0.9420	0.9926	0.0305
AAA1	0.9406	1.0594	1.0345	0.9358	0.9926	0.0318
PSG9	0.9280	1.1965	0.9174	0.9285	0.9926	0.0680
CHIT1	1.0090	0.9923	0.9983	0.9708	0.9926	0.0080
C12orf59	0.9523	1.0107	1.1697	0.8377	0.9926	0.0691
ARTN	0.9470	1.1296	1.0214	0.8724	0.9926	0.0549
LOC728212	1.1105	1.0861	0.8899	0.8840	0.9926	0.0612
CA5BP	0.9897	1.1518	0.9313	0.8976	0.9926	0.0564
FLJ33708	0.9740	1.0007	1.0766	0.9193	0.9926	0.0327
RAB19	0.9194	1.1127	0.9810	0.9573	0.9926	0.0420
C10orf64	0.9985	0.9861	1.0491	0.9367	0.9926	0.0231
TAS1R3	0.9625	1.1156	1.0552	0.8372	0.9926	0.0606
LOC649185	0.8951	1.0836	1.0778	0.9140	0.9926	0.0510
TMEM132D	0.9699	1.1364	0.9655	0.8987	0.9926	0.0506
DPYS	0.9813	1.0444	1.0280	0.9168	0.9926	0.0286
LOC643841	0.9353	1.0564	1.0736	0.9053	0.9926	0.0424
RAB37	0.9704	1.0804	1.0299	0.8899	0.9926	0.0410
MEI1	0.9502	1.1381	1.0031	0.8792	0.9927	0.0547
GRIA4	0.9084	1.1084	1.0643	0.8894	0.9927	0.0550
GPR148	0.9842	1.0771	1.0046	0.9046	0.9927	0.0355

OASL	0.9758	1.0928	1.0224	0.8796	0.9927	0.0447
APOF	1.1146	1.0198	1.0746	0.7616	0.9927	0.0794
CDC42EP5	1.0042	0.9704	1.0140	0.9821	0.9927	0.0100
C1orf75	0.8961	1.1393	1.0739	0.8614	0.9927	0.0675
ACSBG2	0.9729	1.0800	1.0009	0.9169	0.9927	0.0340
TYW1	0.9370	1.4481	0.9432	0.6425	0.9927	0.1672
HIST1H2AH	0.9428	1.1318	1.0030	0.8932	0.9927	0.0515
MAT1A	0.8897	1.0116	1.1505	0.9189	0.9927	0.0587
LOC284296	0.9545	1.1476	0.9748	0.8938	0.9927	0.0544
LOC284861	0.9321	1.0272	1.0819	0.9296	0.9927	0.0374
B4GALNT3	0.9024	1.2091	0.9914	0.8679	0.9927	0.0767
LOC650794	0.9345	1.1054	1.0350	0.8959	0.9927	0.0477
ORMDL2	0.9569	1.1937	1.1197	0.7006	0.9927	0.1092
NEUROD4	0.9611	1.0198	1.0044	0.9855	0.9927	0.0127
PRM1	0.8950	1.1318	1.0082	0.9359	0.9927	0.0519
DRD4	0.9579	1.1443	1.0393	0.8294	0.9927	0.0665
TMPRSS11B	0.7944	1.0258	1.1402	1.0104	0.9927	0.0722
LOC1001283	0.9656	0.9843	1.0508	0.9702	0.9927	0.0198
TTTY19	0.9261	1.0852	1.0367	0.9230	0.9927	0.0406
OR6K2	0.9824	1.1229	0.9339	0.9317	0.9927	0.0449
ANKH	0.8998	1.1095	0.9824	0.9793	0.9927	0.0433
C2orf83	0.9596	1.0671	1.0302	0.9140	0.9927	0.0344
KRTAP6-3	1.0033	1.0491	1.0122	0.9064	0.9927	0.0304
LOC1001330	0.9510	1.0153	1.1156	0.8891	0.9928	0.0484
VAC14	0.9696	1.1045	1.0376	0.8594	0.9928	0.0523
RTL1	0.9324	1.0037	1.0319	1.0030	0.9928	0.0212
LOC644299	0.8447	1.0742	1.0967	0.9555	0.9928	0.0583
IFI27L2	0.8550	1.1681	1.0771	0.8709	0.9928	0.0773
MGC12760	0.9255	1.0984	1.0547	0.8926	0.9928	0.0496
LOC651381	1.0017	1.0251	1.0107	0.9338	0.9928	0.0203
BCL2L10	0.9438	1.0335	1.0912	0.9027	0.9928	0.0427
KIAA1648	0.9080	1.0360	1.0069	1.0204	0.9928	0.0289
MIR1274A	1.0669	1.0371	0.9809	0.8864	0.9928	0.0397
MIR890	0.9275	1.0884	0.9843	0.9711	0.9928	0.0341
LOC653883	0.9044	1.1528	1.0212	0.8930	0.9928	0.0607
KIAA0284	0.8794	1.2407	0.9797	0.8716	0.9928	0.0862
TRPC2	0.9153	1.1159	1.0441	0.8961	0.9928	0.0526
RFX2	0.9861	1.0937	1.0041	0.8874	0.9928	0.0423
C6orf94	0.9658	1.0162	1.0182	0.9711	0.9928	0.0141
LOC644907	0.7661	1.2352	1.3541	0.6161	0.9929	0.1786
GVIN1	0.9190	1.1230	0.9569	0.9725	0.9929	0.0448
NME2P1	0.9126	1.1701	1.0242	0.8645	0.9929	0.0679
LOC1001311	0.9983	1.0415	1.1219	0.8097	0.9929	0.0662
PXT1	0.9458	1.1407	0.9805	0.9045	0.9929	0.0517
MMP15	0.9530	1.2938	0.9354	0.7893	0.9929	0.1068
DENND1B	0.9216	1.0738	1.0426	0.9336	0.9929	0.0383

SCXB	1.0166	1.0413	1.0208	0.8929	0.9929	0.0338
LOC1001299	0.9822	1.0535	1.0313	0.9045	0.9929	0.0330
LOC646862	0.9458	1.1380	0.9955	0.8923	0.9929	0.0527
LOC644949	1.0078	1.0131	0.9935	0.9572	0.9929	0.0126
LOC651952	0.8709	1.1386	1.0163	0.9458	0.9929	0.0569
LOC728683	0.8903	1.0687	1.1135	0.8991	0.9929	0.0575
NCRNA0015	0.9341	1.0792	1.0592	0.8992	0.9929	0.0448
AGPAT4	0.9808	1.0232	1.0368	0.9309	0.9929	0.0239
LOC441771	1.0008	1.0575	0.9369	0.9764	0.9929	0.0252
CD300A	0.9802	1.0359	1.0761	0.8794	0.9929	0.0426
PROZ	0.9486	1.0701	1.0690	0.8839	0.9929	0.0462
LOC387895	0.9017	1.0326	1.1711	0.8663	0.9929	0.0693
POTED	1.0421	1.0087	0.9749	0.9461	0.9929	0.0208
HHIP	0.9146	1.0368	1.0347	0.9856	0.9929	0.0287
LOC131691	0.9498	1.1185	0.9639	0.9395	0.9929	0.0421
IL12B	0.9246	1.1336	0.9783	0.9353	0.9929	0.0483
ELMO3	0.9516	1.2753	0.8780	0.8669	0.9929	0.0960
LOC729284	0.8019	1.1680	1.0620	0.9399	0.9930	0.0789
PCDH21	0.8103	1.1483	1.1021	0.9111	0.9930	0.0796
RNU105B	0.9232	1.1763	1.0059	0.8664	0.9930	0.0675
FAM22A	0.9513	1.0706	1.0140	0.9359	0.9930	0.0309
LOC401206	0.9107	1.0539	1.1108	0.8964	0.9930	0.0530
LOC400796	0.9237	1.1208	1.0237	0.9037	0.9930	0.0500
KRT77	1.0615	1.0800	0.9311	0.8994	0.9930	0.0455
TULP1	0.9949	0.9955	1.0435	0.9381	0.9930	0.0216
FKSG29	1.0186	1.0717	0.9822	0.8995	0.9930	0.0362
SCML4	0.9926	1.0966	0.9136	0.9692	0.9930	0.0383
EGFLAM	0.9987	1.0466	0.9753	0.9514	0.9930	0.0203
TEDDM1	0.9625	1.0862	1.0126	0.9107	0.9930	0.0374
C3orf49	1.0206	1.0235	0.9967	0.9312	0.9930	0.0215
LOC121981	0.9911	1.0865	1.0770	0.8174	0.9930	0.0624
SLCO1A2	0.9803	1.0837	1.0237	0.8844	0.9930	0.0419
LOC440297	0.8169	1.0528	1.1394	0.9629	0.9930	0.0689
DUX4	0.9081	1.0815	1.0966	0.8857	0.9930	0.0557
LOC644183	0.8855	1.1416	1.0603	0.8848	0.9930	0.0645
LOC652637	0.9776	0.9109	1.1315	0.9521	0.9930	0.0482
SLC35E1	0.9679	1.2027	1.0600	0.7416	0.9930	0.0967
C6orf78	0.9595	1.1170	0.9771	0.9186	0.9930	0.0431
LOC285016	1.0272	1.0026	1.0004	0.9419	0.9930	0.0181
LOC641819	0.9182	1.1398	1.0308	0.8833	0.9930	0.0582
LOC341651	0.9726	1.0377	1.0089	0.9529	0.9930	0.0189
SPATA5	0.9527	1.0316	1.0580	0.9298	0.9930	0.0307
CD40	0.9313	1.0657	1.0482	0.9270	0.9930	0.0371
SNORD116-4	0.9360	1.0252	1.0696	0.9414	0.9930	0.0327
RPPH1	0.9270	1.0135	1.0932	0.9385	0.9931	0.0385
LRRC8D	0.9006	1.3791	0.9439	0.7485	0.9931	0.1353

LOC642626	0.8838	1.0907	0.9925	1.0053	0.9931	0.0424
PRY	0.8176	1.0685	1.1079	0.9783	0.9931	0.0645
SRD5A2L2	0.9594	1.1020	1.0270	0.8839	0.9931	0.0466
PLAG1	0.9418	1.0769	1.0370	0.9167	0.9931	0.0381
KCTD16	1.0107	1.0094	1.0477	0.9045	0.9931	0.0308
LOC1001316	0.8918	1.1160	1.1084	0.8561	0.9931	0.0692
LOC647881	0.9653	1.1288	0.9767	0.9015	0.9931	0.0482
LOC646106	0.9759	1.1500	0.9016	0.9449	0.9931	0.0545
PMS2L11	0.9694	1.1631	0.9745	0.8654	0.9931	0.0620
LOC642785	0.9286	1.2028	0.9367	0.9043	0.9931	0.0702
GTPBP2	0.9827	1.1236	1.0475	0.8185	0.9931	0.0649
GLIPR1L2	0.9304	1.0058	1.0520	0.9843	0.9931	0.0252
MIR367	0.8850	1.1283	1.0498	0.9095	0.9931	0.0578
GRIA2	0.9323	0.9627	1.1192	0.9583	0.9931	0.0425
LOC647683	0.9242	1.1445	0.9750	0.9288	0.9931	0.0517
LOC254559	0.9732	1.1063	0.9940	0.8989	0.9931	0.0429
LOC1001296	1.0345	1.0382	1.0707	0.8292	0.9931	0.0552
SLCO1C1	0.9290	1.0154	1.0546	0.9735	0.9931	0.0270
LOC644365	0.9699	1.0593	1.0599	0.8835	0.9931	0.0422
SELI	0.9040	1.3996	0.9624	0.7065	0.9931	0.1461
TAL2	0.9625	1.1788	0.9367	0.8947	0.9932	0.0634
TAAR5	0.9288	1.0378	0.9857	1.0203	0.9932	0.0240
CHGB	0.9942	1.0933	1.0003	0.8849	0.9932	0.0426
PIP5K1A	0.8849	1.1364	1.0706	0.8808	0.9932	0.0651
LOC643950	0.9707	0.9763	1.0126	1.0131	0.9932	0.0114
LOC646628	0.8587	1.2035	0.9912	0.9193	0.9932	0.0752
LOC1001307	0.9554	1.0756	1.0403	0.9013	0.9932	0.0397
LOC442162	0.9869	1.0806	1.0802	0.8251	0.9932	0.0602
MYOM2	0.9130	1.1470	1.0397	0.8730	0.9932	0.0624
BCAS1	1.0098	1.1140	0.9854	0.8635	0.9932	0.0514
LOC643272	0.9923	0.9310	1.1095	0.9400	0.9932	0.0410
C10orf30	0.9791	1.0771	0.9808	0.9358	0.9932	0.0298
LOC1001347	0.9899	1.0431	1.0098	0.9299	0.9932	0.0238
LOC646793	0.8458	1.2514	1.0087	0.8670	0.9932	0.0933
ZNF770	0.9464	1.1778	1.0896	0.7590	0.9932	0.0915
GPR137	0.9006	1.3324	0.9794	0.7604	0.9932	0.1218
LOC646916	0.8817	1.2147	1.0100	0.8665	0.9932	0.0805
LOC646279	0.8614	1.1063	1.0448	0.9603	0.9932	0.0532
QTRT1	0.9664	1.0920	1.0410	0.8735	0.9932	0.0475
LOC1001297	0.9161	1.1652	0.9837	0.9079	0.9932	0.0598
OTOG	0.9612	1.0452	1.1373	0.8293	0.9932	0.0654
RHOXF2B	0.9176	1.1376	0.9911	0.9267	0.9933	0.0508
LOC732435	0.9897	0.9984	1.0500	0.9349	0.9933	0.0236
KRTAP26-1	0.9422	1.0162	1.0771	0.9375	0.9933	0.0332
OR4A5	0.9219	1.1277	1.0286	0.8949	0.9933	0.0533
PRAMEF2	0.8797	1.1357	1.0348	0.9228	0.9933	0.0576

BMPER	0.9171	1.0720	1.0855	0.8985	0.9933	0.0496
CATSPER1	0.9513	1.1156	1.0186	0.8875	0.9933	0.0488
FST	1.0205	1.0059	1.0288	0.9178	0.9933	0.0256
LOC392440	1.0134	1.0486	1.1365	0.7745	0.9933	0.0774
LOC732160	0.9377	1.1644	0.9088	0.9622	0.9933	0.0581
SUGT1	0.8911	1.2046	1.0368	0.8406	0.9933	0.0818
AFARP1	0.8610	1.1320	1.0768	0.9033	0.9933	0.0657
DKFZp686D0	1.0144	1.0918	1.0155	0.8516	0.9933	0.0506
LOC440706	0.9866	1.0800	1.0480	0.8586	0.9933	0.0489
GUCY2G	0.9207	1.1974	1.0499	0.8051	0.9933	0.0844
LOC399706	0.9053	1.1333	1.0133	0.9214	0.9933	0.0524
LOC649351	1.0072	1.1214	0.9663	0.8784	0.9933	0.0504
LOC645442	1.0131	1.1560	0.9380	0.8662	0.9933	0.0620
KIAA1239	0.8693	1.0662	1.1192	0.9187	0.9933	0.0592
LOC1001303	0.9765	1.0382	1.0704	0.8883	0.9933	0.0401
LOC1001279	1.0180	1.0559	1.0116	0.8878	0.9933	0.0365
ITIH5L	0.9832	1.0280	1.1601	0.8020	0.9933	0.0740
COLEC10	0.8667	1.1733	1.0646	0.8688	0.9933	0.0758
SPANXA1	0.9634	1.0429	1.0732	0.8939	0.9933	0.0404
FLJ12529	0.9226	1.1367	0.9901	0.9239	0.9933	0.0503
PSTPIP1	0.9901	1.0174	0.9942	0.9717	0.9934	0.0094
LOC643738	0.9620	1.0309	1.0307	0.9499	0.9934	0.0218
UMOD	0.9210	1.1134	1.0075	0.9316	0.9934	0.0444
LOC648453	1.0040	0.9585	1.0834	0.9276	0.9934	0.0339
SFTA3	0.9505	0.9877	1.0733	0.9619	0.9934	0.0278
LOC643475	1.0324	1.0188	1.0127	0.9097	0.9934	0.0282
SPINK7	0.9870	1.0310	1.1004	0.8551	0.9934	0.0517
TRIM10	0.9887	1.0561	1.0194	0.9092	0.9934	0.0312
FAP	0.8619	0.9635	1.1326	1.0155	0.9934	0.0563
LOC441257	0.9439	1.1094	0.9639	0.9563	0.9934	0.0389
ZNF81	0.8899	1.1015	1.1093	0.8728	0.9934	0.0648
KBTBD3	0.8481	1.1005	1.1422	0.8827	0.9934	0.0747
RPRD1B	0.8979	1.2105	1.1238	0.7413	0.9934	0.1068
LOC649075	0.9765	1.0577	1.0149	0.9245	0.9934	0.0283
IWS1	0.9376	1.4092	0.8781	0.7487	0.9934	0.1441
CCL21	0.8582	0.9542	1.1382	1.0230	0.9934	0.0589
LOC1001328	0.9445	1.0676	0.9962	0.9653	0.9934	0.0269
WDR21C	0.9147	1.1364	1.0372	0.8853	0.9934	0.0579
SLCO1B3	0.9860	1.0370	1.0288	0.9218	0.9934	0.0264
FLJ37587	0.9214	1.0164	1.0463	0.9896	0.9934	0.0267
LOC255275	0.9487	1.1644	0.9635	0.8972	0.9934	0.0587
CREG1	0.8704	1.3433	1.0493	0.7106	0.9934	0.1356
CRYAA	0.9837	1.0838	0.9488	0.9574	0.9934	0.0310
LOC388813	0.9262	1.0546	1.0511	0.9418	0.9934	0.0345
LOC729830	0.9965	1.0817	0.9190	0.9765	0.9934	0.0337
FREM1	0.9690	1.1670	0.8459	0.9919	0.9934	0.0661

SUMO1P1	1.0197	1.0469	1.0079	0.8994	0.9935	0.0324
CARD17	0.9734	1.0933	1.0761	0.8310	0.9935	0.0603
LOC1001331	0.9114	1.0536	1.0362	0.9727	0.9935	0.0324
OR52H1	0.9381	1.1171	1.0099	0.9088	0.9935	0.0464
MRGPRX3	0.9515	1.1074	0.9635	0.9515	0.9935	0.0381
LOC1001303	0.9601	1.0650	0.9683	0.9805	0.9935	0.0242
MIR548O	0.9117	1.0157	1.1680	0.8785	0.9935	0.0651
OR51S1	0.9674	1.1759	0.9660	0.8647	0.9935	0.0654
MGAT5	0.9494	1.0046	1.0688	0.9512	0.9935	0.0282
LOC650534	0.9732	1.0411	1.0860	0.8737	0.9935	0.0462
KIF26B	0.9272	1.0891	1.0229	0.9349	0.9935	0.0386
LOC1001313	0.8157	1.3320	1.1498	0.6765	0.9935	0.1503
LOC649282	0.9651	1.1378	1.0332	0.8380	0.9935	0.0628
ZNF607	0.9907	1.1553	0.9918	0.8363	0.9935	0.0651
DAB1	0.9730	0.9990	1.0300	0.9721	0.9935	0.0137
LOC730085	0.9823	1.0501	1.0182	0.9235	0.9935	0.0271
LOC645438	0.9222	1.1640	0.9669	0.9210	0.9935	0.0578
RETNLB	0.9536	1.1130	0.9868	0.9206	0.9935	0.0421
MLL2	0.9095	1.1244	1.1049	0.8354	0.9935	0.0717
LOC1001337	0.9952	1.0812	1.0577	0.8399	0.9935	0.0543
MSL2	0.9301	1.1355	1.0051	0.9035	0.9935	0.0520
LOC647987	0.9619	1.0971	1.0549	0.8602	0.9935	0.0527
LOC1001295	0.9819	1.0868	0.9986	0.9068	0.9935	0.0369
LOC650261	0.8340	1.0986	1.0214	1.0201	0.9936	0.0563
LOC1001346	1.0713	1.0468	0.9432	0.9129	0.9936	0.0386
LOC1001288	1.0130	1.0438	0.9802	0.9372	0.9936	0.0228
DDI2	0.9550	1.1329	0.9567	0.9297	0.9936	0.0468
LOC283585	0.9899	1.0371	1.0896	0.8576	0.9936	0.0497
C11orf40	0.9221	1.1283	0.9849	0.9389	0.9936	0.0468
CACNG5	0.9616	1.0455	1.0205	0.9467	0.9936	0.0235
LOC133185	0.8921	1.1586	0.9833	0.9403	0.9936	0.0581
INSM2	0.9925	0.9555	1.0667	0.9595	0.9936	0.0258
SP7	0.9800	1.0558	1.0188	0.9197	0.9936	0.0291
KPNA7	0.9681	1.0889	1.0545	0.8627	0.9936	0.0505
LOC284441	0.9247	1.1837	1.0319	0.8339	0.9936	0.0752
FLJ90086	0.8519	1.2581	1.0557	0.8085	0.9936	0.1033
LOC650840	0.7868	1.3076	0.9205	0.9594	0.9936	0.1110
ENPP6	0.9354	1.1086	1.0518	0.8786	0.9936	0.0526
LOC1001279	0.8503	1.1632	1.1295	0.8313	0.9936	0.0885
LOC442211	0.9346	1.1409	1.0258	0.8731	0.9936	0.0583
ACVR1	0.8363	1.2286	1.2009	0.7085	0.9936	0.1304
HLA-DRB1	0.9499	1.0465	1.0254	0.9525	0.9936	0.0248
ALDH9A1	0.9737	1.2574	1.0743	0.6691	0.9936	0.1231
MIR18B	0.9179	1.0642	1.0166	0.9758	0.9936	0.0310
MAP1LC3B2	0.9231	1.0835	1.0469	0.9209	0.9936	0.0420
CSF2	0.9461	1.1263	1.0588	0.8433	0.9936	0.0624

PGA5	0.9713	1.0518	1.0614	0.8899	0.9936	0.0400
OR10H3	0.9129	1.0889	1.0238	0.9488	0.9936	0.0393
LOC653117	0.8944	1.1016	1.0274	0.9510	0.9936	0.0452
CCL11	0.9047	1.1330	1.0977	0.8392	0.9936	0.0719
ELANE	1.0355	1.0621	0.9792	0.8978	0.9936	0.0363
DEFB113	0.9069	1.1171	1.0331	0.9174	0.9936	0.0501
LOC388279	0.9484	1.0821	0.9873	0.9568	0.9937	0.0306
TRIM39	1.0008	1.2448	0.9746	0.7544	0.9937	0.1003
FLJ44060	1.0016	1.0502	0.9852	0.9376	0.9937	0.0232
LOC648754	0.9535	1.0465	1.0464	0.9283	0.9937	0.0309
C14orf49	0.8872	1.1104	1.0394	0.9377	0.9937	0.0501
LOC642313	0.9292	1.1053	1.0054	0.9348	0.9937	0.0410
LOC652887	0.8975	1.2266	1.0128	0.8379	0.9937	0.0857
LOC654091	0.9570	1.1155	1.0315	0.8708	0.9937	0.0522
DMRT3	0.9651	1.0554	0.9594	0.9949	0.9937	0.0220
ERCC-00143	0.9850	1.0403	1.0315	0.9180	0.9937	0.0280
GPC1	0.9561	1.0930	1.1294	0.7962	0.9937	0.0756
DEFB117	1.0056	1.0720	0.9465	0.9507	0.9937	0.0294
MIR548A2	0.8510	1.2231	1.1083	0.7925	0.9937	0.1027
LOC648366	0.8344	1.1818	1.0316	0.9271	0.9937	0.0745
KCNAB1	0.9469	1.1341	1.0071	0.8867	0.9937	0.0529
ZNF300	0.9395	1.1764	1.0379	0.8212	0.9937	0.0753
LOC653089	1.0120	1.0352	0.9744	0.9532	0.9937	0.0184
LOC389332	0.9613	1.1034	1.0421	0.8680	0.9937	0.0510
TSNAX	0.7512	1.5859	0.9873	0.6506	0.9937	0.2096
BHLHB3	0.9410	1.1277	1.0227	0.8835	0.9937	0.0530
ODF1	0.9651	1.0604	1.0351	0.9144	0.9937	0.0333
ZNF76	0.9617	1.2245	0.9081	0.8807	0.9937	0.0787
LOC651730	0.9262	1.0732	1.0087	0.9668	0.9937	0.0314
ATP6V1C2	0.9758	1.1547	0.9583	0.8862	0.9937	0.0571
OR14I1	1.0242	1.0323	0.9936	0.9249	0.9937	0.0244
LOC643801	0.8822	1.0546	1.1253	0.9129	0.9937	0.0577
LOC731742	0.8818	1.1733	1.0161	0.9037	0.9938	0.0667
LOC149448	0.9905	1.1229	0.9518	0.9100	0.9938	0.0461
LRRC69	0.8754	1.2517	0.9490	0.8989	0.9938	0.0873
SCN2A	0.9558	1.0568	1.0304	0.9320	0.9938	0.0297
PPP1R12C	1.0771	1.0609	0.8776	0.9595	0.9938	0.0467
HMGB3L1	0.9200	1.0867	0.9822	0.9862	0.9938	0.0345
FAM47C	0.8622	1.1206	1.0999	0.8923	0.9938	0.0677
LOC1001344	0.9604	1.0266	1.0329	0.9552	0.9938	0.0208
LOC441124	1.0112	1.1189	0.9192	0.9258	0.9938	0.0467
LOC650713	0.9111	1.1507	1.0780	0.8353	0.9938	0.0728
LOC653514	0.9422	1.2001	0.9495	0.8834	0.9938	0.0704
LOC1001343	0.9632	1.0112	1.1007	0.9001	0.9938	0.0423
GP6	0.9315	1.0786	1.0788	0.8864	0.9938	0.0499
LOC1001334	0.9795	0.9749	1.0928	0.9280	0.9938	0.0350

ANKRD11	0.8977	1.3891	0.8906	0.7978	0.9938	0.1337
LOC729454	0.9571	1.0680	1.0665	0.8837	0.9938	0.0450
RTP3	0.9981	1.0938	0.9518	0.9315	0.9938	0.0361
LOC732387	1.0101	1.1391	0.9500	0.8761	0.9938	0.0556
CHRM3	0.7279	1.2305	1.2779	0.7391	0.9938	0.1506
F7	0.9215	1.1618	0.9592	0.9327	0.9938	0.0566
LOC400955	0.9428	1.0043	1.0962	0.9321	0.9938	0.0376
PPP1R1C	0.9632	1.0660	1.0035	0.9427	0.9938	0.0272
TLX2	0.9186	1.1328	0.9880	0.9359	0.9938	0.0486
SLC24A6	0.9638	1.2933	0.9437	0.7747	0.9938	0.1085
LOC390192	0.9307	1.1178	1.0012	0.9257	0.9939	0.0448
LOC440030	0.9906	0.9890	1.0453	0.9505	0.9939	0.0195
IMP5	0.9550	1.2156	0.9203	0.8847	0.9939	0.0753
ESX1	0.9068	1.0799	1.0603	0.9285	0.9939	0.0444
ELMOD2	0.7532	1.3093	1.2064	0.7067	0.9939	0.1541
TAS2R42	0.9682	1.0813	1.0227	0.9034	0.9939	0.0380
ITPKC	0.9956	1.1303	0.9422	0.9075	0.9939	0.0489
LOC1001344	0.9647	0.9786	1.0929	0.9394	0.9939	0.0340
ENPEP	0.9885	1.0679	0.9806	0.9386	0.9939	0.0270
TBC1D26	0.9129	1.1166	0.9817	0.9644	0.9939	0.0434
IGFL1	0.9133	1.0549	1.1115	0.8960	0.9939	0.0529
FLJ20718	0.7697	1.4799	1.0048	0.7213	0.9939	0.1734
CCDC73	1.0218	1.1374	0.9140	0.9025	0.9939	0.0548
SPAM1	1.0013	0.9924	0.9870	0.9950	0.9939	0.0030
LOC1001301	1.0306	1.0388	1.0021	0.9043	0.9939	0.0309
TMEM136	0.7488	1.4386	1.1203	0.6680	0.9939	0.1780
LOC644277	0.8928	1.0345	1.0896	0.9588	0.9939	0.0431
SOCS3	0.9746	1.0565	1.0227	0.9219	0.9939	0.0293
ANKRD18A	0.9304	1.0864	1.0325	0.9264	0.9939	0.0394
ARHGAP28	0.9940	1.2119	1.0030	0.7669	0.9939	0.0909
LOC643581	0.9465	1.0663	1.0472	0.9158	0.9939	0.0370
GATA6	0.9041	1.0735	1.0319	0.9663	0.9939	0.0372
LOC389730	0.9239	1.0228	1.0767	0.9524	0.9940	0.0345
BPY2	1.0049	1.1122	0.9372	0.9216	0.9940	0.0434
LOC1001312	1.0209	1.0340	0.9905	0.9304	0.9940	0.0230
BCL3	0.9473	1.2470	0.9571	0.8244	0.9940	0.0896
LOC1001312	0.9759	1.0467	0.9874	0.9658	0.9940	0.0181
LOC649660	0.8742	1.1054	1.1289	0.8672	0.9940	0.0713
TRIM32	0.7839	1.4185	1.0718	0.7017	0.9940	0.1622
OR5A2	0.9219	0.9430	1.1864	0.9246	0.9940	0.0643
CDY2A	0.9816	1.0649	0.9300	0.9994	0.9940	0.0279
LOC729545	0.9666	1.0690	1.1413	0.7991	0.9940	0.0742
CORIN	0.9506	1.1097	1.0071	0.9086	0.9940	0.0435
HLA-DQB1	0.8696	1.1286	1.0630	0.9148	0.9940	0.0610
LOC653636	0.9537	1.2186	0.9194	0.8844	0.9940	0.0762
TP73	0.9502	1.1390	1.0161	0.8708	0.9940	0.0567

COPG2IT1	0.8355	1.0815	1.1111	0.9479	0.9940	0.0636
RPL29P2	0.9634	1.0933	1.0130	0.9064	0.9940	0.0396
STAMBP	0.9233	1.3266	1.0045	0.7217	0.9940	0.1258
LOC1001301	1.0147	1.0228	0.9920	0.9466	0.9940	0.0171
LOC645899	1.0210	1.1063	0.9987	0.8501	0.9940	0.0533
LOC729387	1.0218	1.0069	1.0401	0.9073	0.9940	0.0297
LOC729862	0.9070	1.0865	1.0363	0.9463	0.9940	0.0410
LOC1001325	0.9382	1.1168	1.0651	0.8561	0.9940	0.0593
SMTNL1	0.9949	1.0610	0.9665	0.9537	0.9940	0.0239
GDPD1	0.8395	1.3684	0.9923	0.7759	0.9940	0.1328
NDP	0.9527	1.2240	0.9774	0.8221	0.9940	0.0839
UGT1A10	0.9331	1.0841	1.0425	0.9164	0.9940	0.0410
LOC389036	1.1019	1.0179	0.9639	0.8924	0.9940	0.0442
ZNF409	0.8557	1.1097	1.0458	0.9651	0.9940	0.0548
LOC645755	0.8862	1.1100	1.0279	0.9520	0.9940	0.0483
FOXE1	0.9271	1.0677	1.0882	0.8932	0.9940	0.0491
TMEM232	0.8666	1.1244	0.9572	1.0280	0.9941	0.0546
SCGB3A1	0.9485	1.1563	0.9001	0.9714	0.9941	0.0561
CENTB2	0.8869	1.1784	1.0663	0.8447	0.9941	0.0780
LOC441768	0.9139	1.0927	1.0537	0.9160	0.9941	0.0464
LOC390483	0.9352	1.0788	1.0340	0.9283	0.9941	0.0372
LOC644644	0.7681	1.2158	0.9822	1.0101	0.9941	0.0916
LOC1001340	1.0063	1.0036	1.0385	0.9279	0.9941	0.0234
C16orf50	0.9551	1.0586	1.1009	0.8617	0.9941	0.0537
SERPINA12	0.9217	1.1463	0.9510	0.9573	0.9941	0.0513
LOC1001344	0.9414	1.0740	1.0349	0.9261	0.9941	0.0359
KCNB2	1.0181	1.0350	1.0753	0.8479	0.9941	0.0502
COL9A3	0.9719	1.0454	1.0066	0.9525	0.9941	0.0204
RAPSN	0.9914	1.1110	0.9226	0.9513	0.9941	0.0414
LOC646892	0.8912	1.0948	1.0715	0.9189	0.9941	0.0519
C3orf46	0.9869	1.0232	1.0120	0.9544	0.9941	0.0153
IFNA17	0.9511	1.0566	1.0098	0.9589	0.9941	0.0246
TAS2R30	1.0209	0.9626	1.0640	0.9290	0.9941	0.0300
BMP10	1.0322	0.9718	1.0249	0.9477	0.9941	0.0205
LOC730198	0.9463	1.0174	1.1082	0.9047	0.9941	0.0446
TLX1	0.9147	1.1301	1.0335	0.8982	0.9941	0.0544
C11orf85	0.8546	1.1729	1.0154	0.9336	0.9942	0.0680
LOC642130	0.9153	1.0170	1.0007	1.0438	0.9942	0.0278
LOC1001289	0.9759	1.0309	1.0845	0.8854	0.9942	0.0425
C17orf54	0.8761	1.1401	0.9861	0.9744	0.9942	0.0545
ABCD2	0.9589	1.0371	1.0275	0.9532	0.9942	0.0221
ABL2	0.9392	1.0880	1.0182	0.9314	0.9942	0.0369
F11R	0.9853	1.0653	1.0450	0.8811	0.9942	0.0413
LOC283804	0.9539	1.0728	0.9779	0.9722	0.9942	0.0267
LOC650498	0.9183	1.0021	1.0390	1.0174	0.9942	0.0264
UMODL1	0.8717	1.0808	1.0270	0.9972	0.9942	0.0443

FAM75A4	0.9387	1.0862	1.0676	0.8844	0.9942	0.0491
C10orf27	0.9565	1.1119	0.9960	0.9125	0.9942	0.0428
OR6C68	0.9508	1.1180	1.0351	0.8729	0.9942	0.0529
LHCGR	0.9489	0.9972	1.0975	0.9333	0.9942	0.0370
LOC388122	0.8387	1.2582	1.0752	0.8047	0.9942	0.1066
FLJ46109	0.9395	1.1364	1.0122	0.8887	0.9942	0.0538
TSPYL2	0.8760	1.2382	1.0232	0.8394	0.9942	0.0905
SNORD19B	0.9954	1.0656	0.9981	0.9177	0.9942	0.0302
USP40	0.8938	1.2071	1.0615	0.8144	0.9942	0.0877
LOC648859	0.9292	1.0952	0.9786	0.9739	0.9942	0.0354
LOC651101	0.9947	1.0922	1.0327	0.8572	0.9942	0.0499
LOC1001310	0.9101	1.2006	1.0368	0.8294	0.9942	0.0810
REG3A	0.8971	1.1505	1.0882	0.8411	0.9942	0.0742
LOC653269	1.0133	1.0905	0.9023	0.9708	0.9942	0.0394
LOC1001320	0.9195	1.1040	0.9829	0.9705	0.9942	0.0391
LOC1001308	0.9897	1.0681	1.0272	0.8920	0.9942	0.0376
SNORA69	0.8667	1.1324	1.0450	0.9329	0.9942	0.0589
LOC644207	1.0053	1.0457	0.9843	0.9416	0.9942	0.0217
PFN2	0.8437	1.2913	1.2304	0.6116	0.9942	0.1615
SUMO4	0.9756	1.0393	0.9886	0.9736	0.9943	0.0154
TNFRSF6B	0.9166	1.0430	1.0310	0.9864	0.9943	0.0286
LOC729332	0.9188	1.0984	1.0737	0.8862	0.9943	0.0536
LOC1001292	0.9440	1.0803	1.0841	0.8687	0.9943	0.0530
LOC652379	0.9424	1.0663	1.1340	0.8343	0.9943	0.0664
LOC1001278	0.9517	1.0823	1.0051	0.9380	0.9943	0.0327
MAS1	1.0836	1.0241	0.9932	0.8762	0.9943	0.0436
CRTAC1	0.8923	1.0550	1.0721	0.9577	0.9943	0.0423
LOC646795	1.0100	1.0128	1.0511	0.9032	0.9943	0.0318
LOC402066	0.9773	1.1560	0.9146	0.9292	0.9943	0.0555
C9orf106	0.8777	1.1115	1.0246	0.9634	0.9943	0.0493
LOC649775	1.0072	0.9959	1.0629	0.9113	0.9943	0.0313
GPR182	0.7789	1.3610	1.0121	0.8253	0.9943	0.1322
MEP1A	0.9150	1.0353	1.1101	0.9168	0.9943	0.0478
LOC646589	0.9491	1.0629	1.0510	0.9143	0.9943	0.0369
LOC388996	0.9717	1.0020	1.0622	0.9414	0.9943	0.0258
JAKMIP3	0.9835	0.8981	1.1664	0.9293	0.9943	0.0600
LOC1001320	0.8635	1.1461	1.0930	0.8747	0.9943	0.0731
LOC1001343	0.8954	1.0579	1.0682	0.9559	0.9943	0.0416
LOC645099	1.0116	1.0328	1.0823	0.8508	0.9943	0.0501
LOC644387	1.0275	1.0375	0.9834	0.9290	0.9943	0.0247
LCE1F	0.9010	1.0873	0.9330	1.0561	0.9944	0.0456
DAPK1	0.9203	1.0608	1.0686	0.9277	0.9944	0.0407
LRIG3	0.9139	1.1747	1.0456	0.8433	0.9944	0.0733
FOXF1	0.9489	1.2273	0.9867	0.8146	0.9944	0.0860
LOC347411	0.9139	1.1497	0.9122	1.0018	0.9944	0.0558
LOC1001320	0.9959	1.1015	1.0303	0.8499	0.9944	0.0530

PKHD1	0.9160	1.0429	1.0987	0.9201	0.9944	0.0456
MIR1289-1	0.9577	1.1395	1.0278	0.8526	0.9944	0.0603
LOC648738	0.9810	1.1283	1.0285	0.8398	0.9944	0.0600
PRKACG	1.0788	0.9750	0.9796	0.9443	0.9944	0.0292
MIR141	0.9815	0.9784	1.0108	1.0071	0.9944	0.0084
IL1RAPL2	0.9469	1.1124	1.0083	0.9101	0.9944	0.0442
LOC729944	0.9700	1.1306	1.0479	0.8292	0.9944	0.0641
PCDH18	0.9267	1.0874	1.0992	0.8645	0.9945	0.0585
B4GALNT4	1.0929	1.1750	0.8918	0.8181	0.9945	0.0836
LOC730351	0.9327	1.1155	1.0404	0.8892	0.9945	0.0514
LOC649136	0.9236	1.1881	0.9845	0.8816	0.9945	0.0679
OLA1	0.9565	1.1078	1.0228	0.8909	0.9945	0.0464
DOCK10	1.0030	1.1684	0.9749	0.8316	0.9945	0.0691
DNTT	0.9523	1.0456	1.0482	0.9319	0.9945	0.0305
AIPL1	0.9659	1.1137	0.9298	0.9685	0.9945	0.0407
LOC1001333	0.9881	1.0267	1.0125	0.9506	0.9945	0.0166
DOCK4	0.9147	1.0785	1.0549	0.9299	0.9945	0.0421
LOC646582	1.0074	1.1531	0.9398	0.8776	0.9945	0.0591
XLKD1	0.9044	1.1500	0.9840	0.9396	0.9945	0.0543
MYOF	0.9018	1.1220	1.1161	0.8381	0.9945	0.0731
GAGE1	0.9536	1.1170	0.9788	0.9286	0.9945	0.0421
LOC221981	0.9033	1.1228	1.0642	0.8878	0.9945	0.0585
LOC1001299	0.9111	1.0779	1.0525	0.9366	0.9945	0.0415
CASP4	0.9034	1.1714	1.1176	0.7858	0.9945	0.0905
LOC650177	0.8476	1.0618	1.0923	0.9764	0.9945	0.0548
PAPLN	0.8567	1.0639	1.1394	0.9181	0.9945	0.0650
SMU1	0.7775	1.4470	0.9852	0.7684	0.9945	0.1589
ASB5	0.9694	1.0658	1.0566	0.8862	0.9945	0.0421
LOC732372	0.8920	1.0841	1.0607	0.9412	0.9945	0.0463
LOC643116	1.0779	0.9602	1.0952	0.8448	0.9945	0.0582
LOC728181	0.9142	1.2166	0.9985	0.8489	0.9945	0.0801
KRT75	0.9512	1.0154	1.0963	0.9153	0.9945	0.0397
DNAJA4	0.9187	1.1185	1.0403	0.9007	0.9945	0.0517
ANKRD13D	0.9448	1.1171	0.9864	0.9299	0.9945	0.0426
CAMTA2	0.9330	1.1891	0.9725	0.8836	0.9945	0.0674
MIR595	0.9800	1.0680	1.0073	0.9230	0.9945	0.0301
LOC1001323	1.0245	1.0907	1.0028	0.8602	0.9945	0.0485
SNORD114-3	0.8762	1.0408	1.1349	0.9263	0.9946	0.0581
SPHK2	0.8726	1.4543	0.9188	0.7325	0.9946	0.1583
MLLT4	0.9610	1.0450	1.0172	0.9551	0.9946	0.0219
LOC1001343	0.9891	1.0037	1.0865	0.8990	0.9946	0.0384
LOC644573	0.8827	1.2319	1.0072	0.8566	0.9946	0.0857
CDC27	0.9167	1.1045	1.1139	0.8433	0.9946	0.0679
LOC390427	0.9046	1.1034	1.0168	0.9535	0.9946	0.0429
MOSPD2	0.9307	1.1466	1.0868	0.8143	0.9946	0.0754
LOC645947	0.9351	1.0526	1.0760	0.9146	0.9946	0.0408

ZBTB12	0.8847	1.1345	1.0204	0.9387	0.9946	0.0544
SLC17A6	0.9612	1.0353	1.0372	0.9447	0.9946	0.0243
SFTA1P	0.9438	1.0026	1.0794	0.9525	0.9946	0.0311
HECTD1	0.8752	1.5942	0.8802	0.6287	0.9946	0.2083
FLJ41481	0.8832	1.1880	1.0073	0.9000	0.9946	0.0701
PLA2G6	1.0052	1.1203	0.9853	0.8676	0.9946	0.0517
C7orf63	1.0220	1.3195	0.9443	0.6926	0.9946	0.1291
PAK7	0.9729	1.0496	1.0379	0.9181	0.9946	0.0306
LOC1001288	1.0746	0.9852	0.9831	0.9355	0.9946	0.0290
OTOL1	1.0091	1.1587	0.9092	0.9014	0.9946	0.0599
LOC651503	0.8476	1.1442	0.9960	0.9907	0.9946	0.0606
RPL3L	0.9749	1.1267	1.0784	0.7986	0.9946	0.0726
SIGLEC10	0.9890	1.1413	0.9319	0.9164	0.9946	0.0513
TIMD4	0.9833	1.0728	0.9614	0.9610	0.9946	0.0266
MIMT1	0.9003	1.2127	0.9916	0.8739	0.9946	0.0769
KCNJ11	0.8999	1.0425	1.0680	0.9681	0.9946	0.0380
LRRC48	0.9465	1.1436	1.0090	0.8794	0.9946	0.0563
BCL2L15	0.9416	1.0081	1.0723	0.9566	0.9946	0.0296
LOC338328	0.9162	1.0391	1.0762	0.9471	0.9947	0.0377
LOC646588	0.9235	1.0940	1.0573	0.9038	0.9947	0.0475
LOC642377	1.0213	1.0663	0.9742	0.9168	0.9947	0.0320
LOC646976	0.8878	1.0663	1.1107	0.9139	0.9947	0.0552
RPL32	1.0261	1.1894	1.0074	0.7558	0.9947	0.0895
MIR548H3	0.9168	1.0469	1.0637	0.9513	0.9947	0.0359
SSX3	0.8693	1.1156	1.0089	0.9849	0.9947	0.0505
RNF126P1	1.0474	1.0837	0.9771	0.8705	0.9947	0.0469
CLEC18C	0.9490	1.0030	1.0063	1.0204	0.9947	0.0157
HPX	1.0148	1.1235	0.9183	0.9222	0.9947	0.0484
LOC648981	0.9654	1.0495	1.0009	0.9629	0.9947	0.0202
ZNF790	0.9048	0.9329	1.1158	1.0252	0.9947	0.0479
ZC3H12D	0.9133	1.1580	1.0523	0.8552	0.9947	0.0684
CACNG2	0.9946	1.0105	1.0939	0.8798	0.9947	0.0441
LOC652401	0.9230	1.0465	1.0146	0.9946	0.9947	0.0262
ANKRD21	0.9804	1.1243	0.9695	0.9045	0.9947	0.0463
LOC646330	0.9315	1.2626	0.9906	0.7941	0.9947	0.0983
NLRP2	0.9681	1.4898	0.8013	0.7197	0.9947	0.1729
TGM5	0.9470	1.1074	1.0137	0.9108	0.9947	0.0432
LOC440233	0.9386	0.9667	1.1895	0.8841	0.9947	0.0672
CRB2	0.8411	1.1480	0.9941	0.9956	0.9947	0.0626
LOC643857	0.9701	1.1039	1.0362	0.8686	0.9947	0.0501
LOC651808	1.0206	1.1449	0.9188	0.8946	0.9947	0.0570
CREB3L1	0.8637	1.1213	1.0907	0.9031	0.9947	0.0650
PLA2G2C	1.0326	1.0322	1.0737	0.8405	0.9947	0.0523
CAMP	0.9527	0.9529	1.1296	0.9438	0.9947	0.0450
CPT1B	0.9181	1.0338	1.1456	0.8815	0.9948	0.0599
MIR2113	0.9642	1.0844	1.0508	0.8796	0.9948	0.0460

C12orf36	0.9899	1.0287	1.0941	0.8663	0.9948	0.0479
TCEAL6	0.9565	1.0544	1.0253	0.9428	0.9948	0.0268
LOC1001307	0.9458	1.0831	1.0252	0.9249	0.9948	0.0365
TMEM27	1.0299	1.1460	1.0155	0.7876	0.9948	0.0750
GEFT	0.9161	1.1182	0.9456	0.9992	0.9948	0.0446
LOC642816	0.9121	1.0690	1.0432	0.9548	0.9948	0.0368
LOC644908	1.0031	1.1510	0.8755	0.9495	0.9948	0.0583
RBMY1F	0.8928	1.0201	1.0597	1.0064	0.9948	0.0358
LOC649436	0.9105	1.1093	1.0123	0.9471	0.9948	0.0436
LOC1001294	0.9389	1.0423	0.9919	1.0061	0.9948	0.0215
LOC653118	0.8117	1.1325	1.1487	0.8864	0.9948	0.0856
CSNK1G3	0.7311	1.4905	1.0421	0.7155	0.9948	0.1815
ZNRF2	0.9515	1.1142	1.0284	0.8851	0.9948	0.0494
C1QTNF3	1.0489	1.0152	1.0380	0.8771	0.9948	0.0399
BNC1	0.9347	1.0874	1.0291	0.9281	0.9948	0.0385
PRCD	0.8629	1.1668	1.0228	0.9267	0.9948	0.0661
FAM90A15	1.0023	1.0057	1.0391	0.9322	0.9948	0.0225
MGC24125	0.9161	1.0444	1.0874	0.9314	0.9948	0.0421
C8orf44	0.8676	1.2143	1.0218	0.8758	0.9948	0.0813
FAM124B	0.9286	1.0824	0.9836	0.9849	0.9949	0.0320
HLA-F	0.9415	1.0788	1.1575	0.8016	0.9949	0.0784
TRIM6-TRIM3	0.9054	1.0917	1.0455	0.9368	0.9949	0.0441
LOC652140	0.8707	1.1814	0.9769	0.9505	0.9949	0.0661
LOC1001299	0.9794	1.0822	1.0610	0.8569	0.9949	0.0510
LOC728457	0.9434	1.3400	0.9089	0.7872	0.9949	0.1198
C21orf136	0.9110	1.1151	0.9902	0.9633	0.9949	0.0433
LOC641964	0.9321	1.1150	1.0320	0.9005	0.9949	0.0489
RFTN2	0.8879	1.0792	1.0656	0.9469	0.9949	0.0464
VAX1	0.9384	1.0930	0.9925	0.9557	0.9949	0.0346
PTPRH	0.9796	1.0905	1.0575	0.8519	0.9949	0.0530
GSTA1	0.9820	1.1417	1.0245	0.8315	0.9949	0.0641
SLC30A2	1.0188	1.0449	0.9918	0.9241	0.9949	0.0260
KLHL13	0.8972	1.0688	1.0646	0.9490	0.9949	0.0428
LOC400553	1.0090	1.0456	0.9589	0.9661	0.9949	0.0202
LANCL3	0.9440	1.1261	1.0182	0.8912	0.9949	0.0509
DLX6	0.9386	1.1607	0.9733	0.9070	0.9949	0.0569
MSMP	0.9838	1.0763	1.0251	0.8944	0.9949	0.0385
TUBB6	0.9512	1.1210	1.0136	0.8939	0.9949	0.0486
MIR124-2	0.9775	1.0520	1.0509	0.8993	0.9949	0.0363
WISP2	1.1147	0.9910	0.9928	0.8813	0.9949	0.0477
MIR498	0.9775	1.0718	0.9768	0.9535	0.9949	0.0262
LOC728758	0.9674	1.2136	0.9552	0.8435	0.9949	0.0780
LGALS4	1.0649	1.1201	0.9312	0.8636	0.9949	0.0591
DCN	0.9609	1.0990	1.0027	0.9172	0.9949	0.0388
LOC643733	0.8642	1.2124	1.0246	0.8786	0.9949	0.0810
PLAGL1	0.9550	1.0661	0.9871	0.9716	0.9949	0.0246

ANKRD44	0.8842	1.0768	1.1032	0.9157	0.9950	0.0555
DBF4B	1.0065	1.0544	0.9877	0.9314	0.9950	0.0254
LOC650180	0.9327	1.0672	1.0364	0.9436	0.9950	0.0335
MIR2114	0.9785	0.9906	1.1083	0.9024	0.9950	0.0425
CALML3	1.0394	0.9895	0.9963	0.9547	0.9950	0.0174
LOC644613	1.0268	1.1932	0.8255	0.9344	0.9950	0.0778
HRASLS	0.9125	1.0094	1.1856	0.8724	0.9950	0.0697
ANO2	0.8901	1.1631	1.0191	0.9076	0.9950	0.0629
LOC652586	0.9200	1.1802	1.0034	0.8764	0.9950	0.0671
LOC642219	0.8908	1.0791	1.0007	1.0093	0.9950	0.0389
DKFZp686E2	0.8650	1.1126	0.9841	1.0182	0.9950	0.0511
LOC653807	0.9108	1.1358	1.0705	0.8628	0.9950	0.0646
KIF5A	0.9368	1.0476	1.0718	0.9239	0.9950	0.0378
KIR2DS4	1.0369	1.0709	0.9770	0.8953	0.9950	0.0385
SLC5A4	1.0277	1.0419	0.9872	0.9232	0.9950	0.0266
LOC642537	1.0209	1.0788	0.9700	0.9103	0.9950	0.0359
LOC729143	0.9556	1.1784	1.0505	0.7955	0.9950	0.0807
LOC1001289	0.9701	1.1046	0.9654	0.9400	0.9950	0.0371
LOC284064	0.9187	1.0451	1.0488	0.9675	0.9950	0.0316
LOC642267	0.9717	1.0650	1.0231	0.9203	0.9950	0.0314
DLEC1	0.9501	1.0796	1.0200	0.9304	0.9950	0.0341
MAGEE1	0.9807	1.1665	1.0476	0.7853	0.9950	0.0798
LOC1001311	0.7591	1.1479	1.1922	0.8810	0.9950	0.1044
CDH18	0.9459	1.1448	1.0437	0.8458	0.9950	0.0642
CNTD2	1.0746	1.0374	0.9874	0.8807	0.9950	0.0421
B4GALNT2	1.0618	1.0400	0.9233	0.9551	0.9951	0.0332
C14orf119	0.8617	1.1469	1.0021	0.9695	0.9951	0.0589
LOC727857	1.0229	1.0080	0.9923	0.9571	0.9951	0.0141
LRRC25	0.9460	1.1098	1.0284	0.8961	0.9951	0.0470
FAM3D	0.9483	1.0850	1.0413	0.9058	0.9951	0.0412
MBD3L1	0.9150	1.1559	0.9720	0.9374	0.9951	0.0549
LOC401915	0.9214	1.1668	1.0609	0.8312	0.9951	0.0742
C4orf37	0.9168	1.0756	1.0290	0.9590	0.9951	0.0354
STRC	0.9662	1.0610	1.0023	0.9510	0.9951	0.0245
MIR652	0.9712	1.0189	1.0273	0.9631	0.9951	0.0163
OR7C1	0.9562	1.0555	1.0442	0.9246	0.9951	0.0323
RSPH10B	1.0211	1.0538	0.9862	0.9193	0.9951	0.0288
LOC652816	0.9044	1.0305	1.0734	0.9721	0.9951	0.0367
TRIM36	0.9314	1.1718	0.9968	0.8805	0.9951	0.0635
SNORD27	0.9057	1.1689	1.0044	0.9016	0.9951	0.0626
KIR2DS3	1.0023	1.0782	0.9866	0.9135	0.9951	0.0338
LOC1001344	0.8677	1.2068	1.0569	0.8493	0.9951	0.0847
ACY3	0.9688	1.0136	1.0665	0.9316	0.9951	0.0291
LOC441642	0.9655	1.1208	1.0921	0.8022	0.9951	0.0726
GPKOW	0.9072	1.0148	1.1937	0.8650	0.9952	0.0733
MIR495	0.9619	1.1055	1.0140	0.8993	0.9952	0.0436

LOC1001279	0.9709	1.0727	0.9859	0.9512	0.9952	0.0268
SRGAP1	0.9534	1.1757	0.9982	0.8534	0.9952	0.0674
ESR2	1.0155	1.0831	0.9987	0.8834	0.9952	0.0415
PROX1	0.9570	1.0465	1.0580	0.9192	0.9952	0.0339
LOC728080	0.9606	1.0747	1.0519	0.8935	0.9952	0.0419
SPINT3	0.9299	1.0615	1.0996	0.8898	0.9952	0.0505
LOC1001325	0.8494	1.2392	0.9962	0.8959	0.9952	0.0869
C14orf144	0.9240	1.0868	1.0454	0.9245	0.9952	0.0418
AKAP8L	0.8392	1.2891	1.0596	0.7929	0.9952	0.1139
SFRS16	1.0547	0.9987	1.0250	0.9024	0.9952	0.0330
LOC1001308	0.9453	1.0427	1.1165	0.8763	0.9952	0.0529
LOC647108	0.9928	1.0246	1.0257	0.9377	0.9952	0.0206
LOC727846	0.8811	1.1444	1.0395	0.9158	0.9952	0.0602
LOC642166	0.8671	1.0637	1.0639	0.9861	0.9952	0.0465
FHL1	0.9716	1.1651	0.9089	0.9353	0.9952	0.0581
UNC5A	0.9718	1.1609	0.9388	0.9093	0.9952	0.0567
LOC1001321	0.9081	1.1184	1.0270	0.9274	0.9952	0.0486
LOC643121	0.9571	1.1975	0.9350	0.8913	0.9952	0.0688
TMOD1	0.9132	1.2299	0.9907	0.8471	0.9952	0.0836
KRT33B	0.8885	0.9959	1.1097	0.9867	0.9952	0.0452
LOC650427	0.8433	0.9978	1.1681	0.9717	0.9952	0.0668
SPATA8	1.0198	0.9971	0.9161	1.0480	0.9952	0.0284
LOC642338	0.9353	1.1552	0.9558	0.9347	0.9953	0.0535
GALNTL5	0.9733	1.1108	1.0500	0.8469	0.9953	0.0569
MIR214	0.9863	1.1611	0.9746	0.8591	0.9953	0.0623
NEBL	0.9327	1.1481	1.0563	0.8440	0.9953	0.0670
SPRR2A	0.9871	1.0925	1.0144	0.8871	0.9953	0.0424
LOC653244	0.9489	1.0540	1.0613	0.9170	0.9953	0.0366
ORAI3	1.0470	1.0976	1.0539	0.7826	0.9953	0.0718
LOC651961	1.0443	1.0357	0.9767	0.9244	0.9953	0.0280
LOC1001332	0.9902	1.0538	1.0176	0.9195	0.9953	0.0284
RXFP2	0.9674	0.9852	1.1068	0.9218	0.9953	0.0395
LOC650905	1.0357	0.9906	1.0843	0.8705	0.9953	0.0458
TMEM16J	0.9754	1.0053	1.0521	0.9484	0.9953	0.0222
LOC642490	0.8959	1.1621	0.9563	0.9669	0.9953	0.0577
LOC642730	1.0170	1.0001	1.0558	0.9084	0.9953	0.0312
CHN2	0.9018	1.0966	1.0268	0.9560	0.9953	0.0424
LOC1001339	0.9850	1.0958	1.0218	0.8787	0.9953	0.0452
SIX6	0.9182	1.1299	1.0663	0.8670	0.9953	0.0616
LACE1	0.8402	1.1138	1.1132	0.9141	0.9953	0.0699
C6orf59	0.9923	1.1048	0.9827	0.9016	0.9953	0.0418
LOC1001289	1.0102	1.0452	1.0002	0.9256	0.9953	0.0252
LOC650594	0.9665	1.0684	1.0217	0.9247	0.9953	0.0314
KLK5	0.9805	1.0534	1.0116	0.9359	0.9953	0.0248
PLCZ1	0.9482	1.0744	1.0307	0.9281	0.9953	0.0345
LOC645078	0.9007	1.1415	1.0034	0.9358	0.9953	0.0532

CORO2B	0.9607	1.0526	1.0774	0.8907	0.9953	0.0430
ZNF358	0.9101	1.3658	0.8883	0.8171	0.9953	0.1251
SSPN	0.9937	1.0776	1.0105	0.8997	0.9954	0.0367
OCM2	0.9198	1.0162	1.1623	0.8830	0.9954	0.0623
LOC652175	1.0098	1.1905	0.9833	0.7979	0.9954	0.0803
PPP2R2D	0.8400	1.3227	1.0554	0.7633	0.9954	0.1254
LOC1001286	0.9801	1.0648	1.0046	0.9320	0.9954	0.0276
C7orf52	0.9363	1.1091	1.0365	0.8996	0.9954	0.0477
LOC441246	0.8297	1.3076	1.0661	0.7781	0.9954	0.1215
LOC1001300	0.9200	1.1122	1.1393	0.8100	0.9954	0.0787
ACTN3	0.9381	1.1659	0.9990	0.8786	0.9954	0.0619
P2RY12	0.9380	1.0818	1.0615	0.9003	0.9954	0.0449
LOC643529	0.9737	1.1358	0.9348	0.9373	0.9954	0.0476
TPR	0.9539	1.3069	0.8816	0.8392	0.9954	0.1065
LOC652534	1.0062	1.0440	1.0336	0.8978	0.9954	0.0335
MDFI	0.8634	1.2494	0.9215	0.9473	0.9954	0.0865
SLC26A7	1.0562	0.9940	1.0175	0.9139	0.9954	0.0300
LOC1001315	0.9587	1.0816	1.0553	0.8860	0.9954	0.0450
LOC643697	0.9428	1.0485	0.9918	0.9985	0.9954	0.0216
DEFB104B	0.9074	0.9769	1.0914	1.0060	0.9954	0.0381
C17orf28	1.0739	1.1539	0.9750	0.7789	0.9954	0.0809
OR2T33	0.9536	1.2151	0.9739	0.8391	0.9954	0.0790
LOC1001336	0.9213	1.0213	1.0185	1.0206	0.9954	0.0247
DEFB129	0.9712	1.0480	1.0381	0.9245	0.9954	0.0292
LOC391767	0.9467	1.1701	1.0477	0.8173	0.9954	0.0749
LOC643171	0.9335	1.0780	1.0805	0.8898	0.9954	0.0492
LOC387701	0.9005	1.1349	0.9851	0.9613	0.9954	0.0498
LOC648494	0.8799	1.2149	1.0821	0.8049	0.9955	0.0937
LOC283155	1.0181	1.0419	1.0087	0.9132	0.9955	0.0283
FANK1	0.9418	1.1864	1.0023	0.8513	0.9955	0.0708
TMEM229B	0.8958	1.2115	0.9471	0.9275	0.9955	0.0728
MIR486	0.9920	1.0755	0.9407	0.9737	0.9955	0.0287
DMPK	0.9970	1.1002	1.0044	0.8803	0.9955	0.0450
LOC651344	1.0013	0.9478	1.0160	1.0168	0.9955	0.0163
TAF7L	0.9302	1.0333	1.0991	0.9193	0.9955	0.0430
GINS1	0.9674	1.1534	0.8816	0.9796	0.9955	0.0570
LOC1001342	0.9221	1.0914	1.0587	0.9096	0.9955	0.0465
GALNT14	0.8617	1.2398	1.0499	0.8305	0.9955	0.0948
DKFZP586I14	0.9576	1.2130	0.9550	0.8564	0.9955	0.0762
LOC648230	0.9226	1.1575	1.0355	0.8663	0.9955	0.0644
LOC283278	1.0845	1.0270	0.9912	0.8793	0.9955	0.0432
ZNF449	0.8666	1.1845	1.0206	0.9103	0.9955	0.0708
LOC648997	0.9694	1.0262	0.9816	1.0048	0.9955	0.0126
ZCCHC2	0.9828	1.1043	1.0364	0.8586	0.9955	0.0520
LOC728654	0.9927	1.0497	1.0248	0.9148	0.9955	0.0293
LOC652460	1.0707	1.0366	0.9705	0.9043	0.9955	0.0368

CNKSR1	0.9129	1.1501	0.9843	0.9348	0.9955	0.0536
CCDC8	0.8923	1.0680	1.1493	0.8725	0.9955	0.0675
LOC1001294	0.9000	1.0732	1.0818	0.9271	0.9955	0.0477
CYBRD1	0.9781	0.9837	1.1061	0.9142	0.9955	0.0401
LOC1001315	0.9421	1.0694	1.0264	0.9443	0.9955	0.0315
LOC390933	0.9678	1.1572	0.9839	0.8732	0.9955	0.0592
LOC652633	0.8574	1.2158	1.1096	0.7994	0.9955	0.0996
LOC1001296	0.9098	1.0964	1.1485	0.8274	0.9955	0.0759
PLAC2	0.9378	1.1114	0.9661	0.9670	0.9956	0.0392
ZNF181	0.9964	1.0606	1.0518	0.8734	0.9956	0.0431
LOC1001284	0.9242	1.0797	1.0451	0.9332	0.9956	0.0393
CASP8	0.9030	1.0743	1.0858	0.9192	0.9956	0.0489
INSL6	0.9984	1.0970	1.0015	0.8854	0.9956	0.0433
C8orf54	0.9345	1.1610	0.9386	0.9481	0.9956	0.0552
UTS2D	0.9571	1.1303	0.9984	0.8965	0.9956	0.0495
GGTA1	0.9742	1.1099	1.0174	0.8808	0.9956	0.0476
ERP27	0.9583	1.1623	0.9999	0.8617	0.9956	0.0627
LOC1001301	0.9266	1.0428	1.0503	0.9626	0.9956	0.0304
LOC642550	1.0261	1.0230	1.0207	0.9125	0.9956	0.0277
LOC1001343	0.9424	1.0475	1.0945	0.8980	0.9956	0.0455
LOC401351	1.0132	1.0652	0.9981	0.9059	0.9956	0.0332
C7orf38	0.8570	1.2994	1.1455	0.6805	0.9956	0.1394
LOC643747	1.0195	1.0317	1.0684	0.8629	0.9956	0.0454
MET	0.8631	1.1989	0.9440	0.9764	0.9956	0.0718
PGK2	1.0061	1.0296	1.0033	0.9435	0.9956	0.0184
KLC4	0.9696	1.0870	1.0559	0.8699	0.9956	0.0487
FAM160B1	0.7796	1.3767	1.1410	0.6853	0.9956	0.1606
TPTE2	0.9083	1.0833	1.0519	0.9390	0.9956	0.0425
LOC648127	0.8830	1.1373	1.1181	0.8442	0.9956	0.0767
ATP10D	0.9787	0.9520	1.1208	0.9311	0.9956	0.0428
LOC730378	0.9911	1.0566	1.0345	0.9003	0.9956	0.0346
XAGE1E	0.8861	1.2441	1.0113	0.8410	0.9956	0.0903
LOC646442	0.9462	0.9658	1.2209	0.8496	0.9956	0.0793
LOC650280	0.9490	1.1253	0.9518	0.9565	0.9956	0.0433
LOC728660	0.8328	1.0878	1.0663	0.9957	0.9956	0.0577
LOC653584	1.0103	1.0521	1.0205	0.8997	0.9957	0.0332
ATL1	1.0049	1.0572	0.9722	0.9482	0.9957	0.0236
SLC4A1	0.8927	1.1351	1.0161	0.9387	0.9957	0.0530
ZNF815	0.8625	1.2280	1.0391	0.8530	0.9957	0.0885
YPEL4	0.9534	1.1147	1.0009	0.9136	0.9957	0.0435
C10orf11	0.9033	1.1070	0.9994	0.9729	0.9957	0.0423
LMO2	0.8964	1.1709	0.9627	0.9527	0.9957	0.0602
CCNI	0.8329	1.2261	1.1275	0.7962	0.9957	0.1068
LOC728117	0.9221	1.2577	1.0004	0.8025	0.9957	0.0964
LOC441032	0.7495	1.0943	1.2917	0.8472	0.9957	0.1225
BAAT	0.9109	0.9975	1.0732	1.0013	0.9957	0.0332

C22orf27	0.8872	1.3571	0.9572	0.7813	0.9957	0.1258
GATA4	0.9707	1.0262	1.1018	0.8842	0.9957	0.0459
ONECUT1	0.9730	1.0962	1.0295	0.8842	0.9957	0.0449
USP22	0.9293	1.2899	0.9506	0.8132	0.9957	0.1026
LOC440226	0.8772	1.1581	1.0914	0.8562	0.9957	0.0759
LOC641975	0.8267	0.9549	1.2417	0.9596	0.9957	0.0876
LOC652015	1.0552	1.0547	0.9914	0.8815	0.9957	0.0409
CYP20A1	0.8693	1.2478	1.1046	0.7611	0.9957	0.1105
IDO1	0.9799	1.0722	1.0135	0.9173	0.9957	0.0324
LOC653232	0.9047	1.1209	1.1563	0.8010	0.9957	0.0855
SNORD100	0.8377	1.0167	1.1150	1.0135	0.9957	0.0577
KPNA5	0.8322	1.1414	1.1234	0.8860	0.9958	0.0797
FLJ40039	0.9492	1.1149	1.0716	0.8474	0.9958	0.0606
JPH3	0.9745	1.1006	0.9088	0.9992	0.9958	0.0398
LOC1001347	0.8702	1.0686	1.1060	0.9382	0.9958	0.0552
LOC641834	0.9317	1.0586	0.9905	1.0022	0.9958	0.0260
AFF2	0.9597	1.1754	0.9893	0.8587	0.9958	0.0661
KIF7	0.9489	1.1278	0.9369	0.9695	0.9958	0.0445
TDRD6	0.8058	1.1653	1.0805	0.9315	0.9958	0.0797
SUGT1P	0.9818	1.1922	1.0373	0.7718	0.9958	0.0869
PCDHGA3	1.0128	0.9816	1.0533	0.9354	0.9958	0.0249
LOC651468	0.9764	1.0626	1.0019	0.9423	0.9958	0.0254
ZFP95	0.9070	1.2195	0.9781	0.8786	0.9958	0.0775
LOC647257	1.0025	1.1276	0.9389	0.9143	0.9958	0.0477
LOC653674	0.8907	1.1428	1.0953	0.8546	0.9958	0.0722
CCDC142	0.9451	1.0953	0.9675	0.9755	0.9958	0.0338
MGLL	0.9372	1.1471	1.0152	0.8839	0.9958	0.0572
CIDEA	0.9590	1.0797	1.0402	0.9045	0.9958	0.0395
LOC728725	0.9450	1.0437	1.1110	0.8837	0.9959	0.0506
LOC1001296	0.9041	1.0694	1.1107	0.8992	0.9959	0.0551
LOC1001293	1.0055	1.0671	0.9928	0.9181	0.9959	0.0306
CCDC68	0.9845	1.1424	0.9726	0.8840	0.9959	0.0537
LOC1001321	0.8915	1.1510	1.0048	0.9361	0.9959	0.0567
LOC652843	1.0052	1.0377	0.9937	0.9469	0.9959	0.0188
METTL10	0.9340	1.1628	1.0400	0.8467	0.9959	0.0683
LOC647678	0.9963	1.1524	0.9884	0.8465	0.9959	0.0625
GPM6A	0.9196	1.0892	1.0415	0.9332	0.9959	0.0414
LOC650390	0.8480	1.1897	1.0475	0.8983	0.9959	0.0773
LOC1001336	0.9822	1.0805	1.0780	0.8429	0.9959	0.0559
GPLD1	0.9519	1.0376	1.0900	0.9041	0.9959	0.0418
LOC648249	0.8058	1.5374	0.9832	0.6572	0.9959	0.1924
TTC39A	0.8546	1.0323	1.0904	1.0063	0.9959	0.0503
LOC728302	0.8680	1.0503	1.1838	0.8814	0.9959	0.0751
IRAK2	0.9137	1.1830	1.1957	0.6912	0.9959	0.1206
LOC1001286	0.8759	1.1662	1.0162	0.9253	0.9959	0.0638
LOC1001317	0.8961	1.0279	1.1177	0.9419	0.9959	0.0489

BRD4	0.9018	1.0994	1.0286	0.9538	0.9959	0.0432
LOC400036	0.9502	1.0961	0.9963	0.9412	0.9959	0.0355
MYOCD	0.9942	1.0314	1.0237	0.9344	0.9959	0.0220
LOC646201	0.9484	1.1000	1.0109	0.9245	0.9959	0.0392
LOC651860	0.9132	1.0190	1.0644	0.9871	0.9959	0.0318
LOC1001308	0.9508	1.0930	1.0746	0.8654	0.9959	0.0538
ATP1A4	0.9480	1.0397	1.0405	0.9555	0.9959	0.0255
FLJ33996	1.0239	1.1807	0.9465	0.8326	0.9960	0.0730
KIRREL3	0.9407	1.1054	0.9985	0.9392	0.9960	0.0390
LOC1001342	0.9504	1.0882	0.9992	0.9461	0.9960	0.0330
TNFSF11	0.9567	1.0694	1.0277	0.9301	0.9960	0.0320
LOC652616	0.8630	1.0431	1.0375	1.0403	0.9960	0.0443
LOC646269	1.0204	0.9785	1.0259	0.9590	0.9960	0.0163
LOC285095	1.0023	1.0700	1.0088	0.9028	0.9960	0.0346
LOC642954	0.8332	1.0801	1.1933	0.8773	0.9960	0.0849
LOC648998	0.8228	1.0790	1.1103	0.9718	0.9960	0.0649
LOC389832	0.8818	1.1341	1.0937	0.8744	0.9960	0.0686
GPR68	1.0064	1.1651	0.9563	0.8561	0.9960	0.0645
FAM90A17	0.8536	1.1175	1.0069	1.0058	0.9960	0.0542
FAM38B	0.9144	1.1862	1.0373	0.8460	0.9960	0.0748
SPESP1	0.9392	1.0821	1.0101	0.9526	0.9960	0.0326
CNBD1	1.0100	1.0235	1.0161	0.9345	0.9960	0.0207
CLRN1OS	0.9796	0.9880	1.0532	0.9632	0.9960	0.0198
TAS2R1	0.9343	1.1413	1.0120	0.8964	0.9960	0.0541
DNMT3L	0.9383	1.0495	1.0584	0.9380	0.9960	0.0335
LOC644035	0.9059	1.0221	1.0249	1.0312	0.9960	0.0301
LOC648502	0.9361	1.0725	1.0725	0.9031	0.9960	0.0447
ZNF85	0.9867	0.9797	1.0783	0.9394	0.9960	0.0293
CASKIN2	0.8142	1.1098	1.1378	0.9223	0.9960	0.0772
CLDN19	0.8798	1.1116	0.9956	0.9972	0.9960	0.0473
LOC1001334	1.0212	1.4728	0.8649	0.6253	0.9960	0.1786
SCG3	0.9330	1.0749	1.0173	0.9590	0.9961	0.0316
DEFB115	1.0412	1.0251	1.0269	0.8910	0.9961	0.0352
SPAG4L	0.9104	1.0921	0.9895	0.9922	0.9961	0.0372
LOC1001334	0.9635	1.2388	0.9638	0.8182	0.9961	0.0879
LOC653392	1.0272	1.0917	1.0423	0.8231	0.9961	0.0593
LOC650140	1.0955	0.9369	1.0637	0.8883	0.9961	0.0496
SPRY4	0.9608	1.1233	1.0194	0.8808	0.9961	0.0510
LOC648689	0.8732	1.0990	1.0514	0.9608	0.9961	0.0500
LEPR	0.9595	1.1295	1.0028	0.8926	0.9961	0.0499
FLJ37228	0.8275	1.1798	1.0211	0.9559	0.9961	0.0733
LOC643231	0.9958	1.1184	1.0083	0.8619	0.9961	0.0525
LOC641733	0.9193	1.1386	0.9906	0.9360	0.9961	0.0499
LOC651079	0.9818	1.0901	1.0281	0.8844	0.9961	0.0433
LIPH	1.0128	1.1980	0.9795	0.7940	0.9961	0.0828
SCYL3	0.9174	1.1579	1.1339	0.7753	0.9961	0.0913

LOC392787	0.9124	1.2077	0.9773	0.8871	0.9961	0.0730
ITIH2	0.8507	1.0899	1.1188	0.9251	0.9961	0.0646
LOC144481	0.9988	1.1029	0.9810	0.9018	0.9961	0.0414
FLT4	0.9583	1.0645	1.0901	0.8716	0.9961	0.0504
MIR218-1	0.9124	1.2102	0.9738	0.8881	0.9961	0.0736
SPATA12	1.0268	1.0835	0.8815	0.9928	0.9962	0.0426
LOC1001292	0.9813	1.1782	1.0613	0.7639	0.9962	0.0873
LOC652849	0.9543	1.0648	1.0085	0.9571	0.9962	0.0261
LOC643169	0.9211	1.0627	1.0491	0.9517	0.9962	0.0352
CDRT1	0.8956	1.1143	1.0474	0.9274	0.9962	0.0512
FAM71F1	1.0269	1.0219	0.9608	0.9752	0.9962	0.0166
LOC644468	0.8477	1.0960	1.1082	0.9329	0.9962	0.0636
EBI3	1.0143	1.0560	1.0122	0.9023	0.9962	0.0329
TMSB4Y	0.9373	1.0521	1.0476	0.9478	0.9962	0.0311
ANKRD20A2	0.9601	0.9963	1.1228	0.9056	0.9962	0.0461
LOC653174	1.0253	1.0584	1.0075	0.8937	0.9962	0.0358
AGXT2L1	0.9942	1.0562	0.9948	0.9396	0.9962	0.0238
SEC61A1	0.9343	1.4267	0.9669	0.6569	0.9962	0.1595
PPP1R16B	0.8569	1.1008	1.0977	0.9294	0.9962	0.0613
LOC650329	1.0111	1.0497	1.0106	0.9134	0.9962	0.0291
LOC651752	0.8601	1.1866	0.9810	0.9571	0.9962	0.0686
POLR3H	0.9062	1.4102	0.9381	0.7303	0.9962	0.1454
ZNF221	0.9194	1.1197	1.0392	0.9066	0.9962	0.0508
LOC646342	0.9402	1.0931	1.0020	0.9496	0.9962	0.0350
LOC729196	0.9832	1.0520	1.1513	0.7983	0.9962	0.0744
CLDN2	0.9675	1.0273	1.0403	0.9498	0.9962	0.0221
KIAA1409	0.9433	1.0798	1.0568	0.9049	0.9962	0.0426
LOC643070	0.9245	1.0410	1.0610	0.9585	0.9962	0.0326
SFRS13B	1.0043	1.0865	0.9490	0.9453	0.9963	0.0330
FLJ40448	0.9603	1.0559	1.0482	0.9206	0.9963	0.0333
MIR941-2	0.8488	1.1043	1.0261	1.0058	0.9963	0.0535
LOC387931	1.0085	0.9799	1.0605	0.9361	0.9963	0.0261
LOC399744	0.9700	1.1372	0.9395	0.9384	0.9963	0.0475
PROM1	0.9208	1.0004	1.1218	0.9421	0.9963	0.0451
RPS10	0.8841	1.1511	1.0304	0.9193	0.9963	0.0603
ASTL	0.7956	1.1919	0.9945	1.0031	0.9963	0.0809
SPATA6	0.9298	1.1482	1.0078	0.8993	0.9963	0.0556
IGF2BP1	0.9825	1.1721	0.9768	0.8537	0.9963	0.0657
LOC642044	0.8975	1.0291	1.0894	0.9693	0.9963	0.0411
NTRK2	0.9291	1.1606	0.9804	0.9151	0.9963	0.0565
LOC730415	0.9397	1.1188	1.0116	0.9151	0.9963	0.0457
LOC728027	0.8434	1.1160	1.0929	0.9329	0.9963	0.0652
LOC132707	1.0198	1.1184	1.0180	0.8290	0.9963	0.0605
LOC1001323	1.0047	1.0519	1.0317	0.8970	0.9963	0.0345
MVD	0.8891	1.1420	1.0944	0.8597	0.9963	0.0713
TCF7	0.9820	1.0532	1.0042	0.9459	0.9963	0.0224

SLC12A4	0.8764	1.1692	1.0320	0.9078	0.9963	0.0667
LOC730412	0.8471	1.1199	1.0387	0.9796	0.9963	0.0575
FHL2	0.9349	1.1152	1.0277	0.9075	0.9963	0.0472
LOC643531	0.8615	1.2792	1.0622	0.7825	0.9963	0.1111
LOC1001332	0.9571	1.1633	0.9702	0.8948	0.9963	0.0580
GSDM1	0.8979	0.9680	1.1410	0.9785	0.9963	0.0514
MIR186	0.9140	0.9237	1.0816	1.0661	0.9963	0.0449
ARHGAP23	0.9733	1.0637	1.0285	0.9199	0.9963	0.0316
FLJ25439	0.9921	1.1572	0.9075	0.9286	0.9964	0.0566
OR6B1	0.9597	0.9818	1.0431	1.0008	0.9964	0.0177
SLC35D3	0.9564	1.0226	1.0399	0.9666	0.9964	0.0205
REEP2	0.9803	1.0706	0.9570	0.9776	0.9964	0.0253
LOC643015	0.9819	1.0534	1.0408	0.9093	0.9964	0.0329
IP6K3	0.9756	1.0956	0.9345	0.9799	0.9964	0.0346
LOC643429	0.9481	1.1500	1.0537	0.8337	0.9964	0.0681
PDILT	1.0754	0.9869	1.0004	0.9227	0.9964	0.0313
HBE1	0.9634	1.1235	0.9907	0.9078	0.9964	0.0457
GJA10	0.9513	1.0487	1.1017	0.8838	0.9964	0.0488
SLC7A13	0.9145	1.1499	0.9989	0.9221	0.9964	0.0546
CCL25	0.9980	1.0606	1.0107	0.9162	0.9964	0.0299
PCDHB3	1.0586	1.0730	0.9432	0.9108	0.9964	0.0407
C3orf19	0.9342	1.3365	0.9268	0.7882	0.9964	0.1182
LOC1001331	1.0679	1.0893	0.9708	0.8576	0.9964	0.0530
SNORA71B	0.9423	1.0577	1.0997	0.8858	0.9964	0.0496
MPRIP	0.9871	1.2790	0.9302	0.7892	0.9964	0.1030
LOC401097	1.0281	1.0914	0.9841	0.8821	0.9964	0.0440
LOC652837	0.9758	1.0781	1.0268	0.9051	0.9964	0.0369
C11orf86	0.8682	1.1752	1.0436	0.8987	0.9964	0.0708
ELOVL4	0.9619	1.0877	0.9538	0.9824	0.9964	0.0310
LOC728658	0.9196	1.0773	1.0574	0.9314	0.9964	0.0412
GTF2IRD2B	1.0039	1.3174	0.9438	0.7207	0.9964	0.1231
LOC1001338	0.8226	1.1192	1.1366	0.9074	0.9964	0.0779
SCNN1G	0.9680	1.0575	1.0192	0.9410	0.9964	0.0260
KRTAP5-7	0.9498	1.1108	0.9869	0.9383	0.9965	0.0395
LOC646350	0.9728	1.0541	1.0178	0.9412	0.9965	0.0248
MAPK8IP2	0.9890	1.0701	0.9996	0.9271	0.9965	0.0293
LOC650737	0.8007	1.2950	1.0275	0.8626	0.9965	0.1104
LOC1001314	0.9089	1.0773	1.0783	0.9213	0.9965	0.0470
LOC728531	0.9304	1.0493	1.0292	0.9769	0.9965	0.0268
PSCDBP	0.9909	1.1347	0.9818	0.8785	0.9965	0.0526
FAM90A1	0.8849	1.1069	1.0537	0.9404	0.9965	0.0509
LBX2	1.0084	1.1163	1.0044	0.8569	0.9965	0.0533
TSPAN15	1.0522	1.2264	0.9064	0.8010	0.9965	0.0923
ZCCHC4	0.9466	1.0580	1.0426	0.9387	0.9965	0.0313
LOC644944	0.8245	1.1023	1.0509	1.0084	0.9965	0.0605
LOC648822	0.9309	1.1009	1.0956	0.8587	0.9965	0.0606

ADAMTS17	0.9071	1.1493	1.0049	0.9247	0.9965	0.0552
OR52D1	1.0337	1.0375	0.9580	0.9568	0.9965	0.0226
MSX2P1	0.9507	1.0706	1.0557	0.9091	0.9965	0.0395
ATP6V1G1	0.9557	1.1606	1.2501	0.6196	0.9965	0.1399
TMEM225	1.0019	1.1389	0.9990	0.8463	0.9965	0.0598
MGC52000	0.9843	1.3350	0.8741	0.7926	0.9965	0.1195
MLL3	0.9108	1.1872	1.0674	0.8207	0.9965	0.0815
LOC643283	0.9992	1.0093	1.0327	0.9449	0.9965	0.0186
PCDHB11	0.9571	1.1599	1.0464	0.8227	0.9965	0.0713
MUM1	1.0069	1.2717	0.9175	0.7900	0.9965	0.1019
JMJD7	0.9518	1.2535	1.0151	0.7657	0.9965	0.1007
HSF5	0.9747	1.0991	0.9618	0.9506	0.9965	0.0345
LOC728566	0.9045	1.2459	1.0205	0.8154	0.9966	0.0931
LOC442060	0.9352	1.1150	1.0329	0.9032	0.9966	0.0482
LOC650014	0.8540	0.9954	1.1170	1.0199	0.9966	0.0543
LOC391352	0.9696	1.0233	1.1118	0.8816	0.9966	0.0483
LOC1001346	0.9608	1.0756	1.0494	0.9005	0.9966	0.0403
CEACAM8	0.9977	1.0465	1.0278	0.9144	0.9966	0.0292
JPH4	0.8589	1.0389	1.1909	0.8977	0.9966	0.0755
LOC728657	0.9163	1.1322	0.9666	0.9714	0.9966	0.0469
NMBR	0.8751	1.0959	1.0992	0.9163	0.9966	0.0589
RNF212	0.8432	1.1341	1.1147	0.8944	0.9966	0.0746
PCM1	0.9749	1.2077	1.0500	0.7540	0.9966	0.0943
LOC732343	0.9091	1.1602	0.9605	0.9566	0.9966	0.0558
LOC650285	0.9728	1.0059	1.0420	0.9658	0.9966	0.0175
C8orf46	0.8919	1.1208	0.9668	1.0070	0.9966	0.0478
FLJ35816	0.9205	0.9636	1.1027	0.9998	0.9966	0.0389
LOC1001334	0.9336	1.0981	0.9399	1.0151	0.9966	0.0385
RPS15	0.9193	1.1889	1.0221	0.8563	0.9966	0.0726
LOC650200	0.9123	1.0918	1.1066	0.8759	0.9966	0.0597
LOC653081	1.0341	1.0655	0.9501	0.9369	0.9967	0.0315
DGKI	0.9960	1.0583	1.0095	0.9229	0.9967	0.0280
LOC730102	1.0224	1.0188	1.0067	0.9387	0.9967	0.0196
ALAS2	0.9765	0.9998	1.0617	0.9487	0.9967	0.0241
LOC643855	1.0157	0.9961	1.0855	0.8894	0.9967	0.0406
CYP3A7	0.8133	1.1228	1.0929	0.9577	0.9967	0.0709
FLJ13611	0.9278	0.9605	1.1853	0.9131	0.9967	0.0636
TAAR6	0.9973	1.0486	1.0433	0.8975	0.9967	0.0350
RBM33	1.0725	1.2430	0.8264	0.8449	0.9967	0.0994
KRT7	0.8899	1.0600	1.1291	0.9078	0.9967	0.0583
NCR2	0.9387	1.0395	1.0905	0.9180	0.9967	0.0410
RASSF10	0.9573	1.0072	1.0333	0.9890	0.9967	0.0160
GSG1	0.9315	1.1279	1.0315	0.8959	0.9967	0.0523
LOC1001290	0.9303	1.0895	1.0151	0.9519	0.9967	0.0358
LYNX1	0.8572	1.0834	1.0863	0.9599	0.9967	0.0551
LOC1001346	0.9477	1.0280	1.0085	1.0026	0.9967	0.0172

LOC645464	0.9323	1.1760	0.9709	0.9076	0.9967	0.0612
VNN2	0.9531	1.1001	1.0629	0.8706	0.9967	0.0523
BTBD10	0.8457	1.2161	1.0328	0.8923	0.9967	0.0832
LOC645718	0.9511	1.0320	1.0955	0.9082	0.9967	0.0417
LOC650074	0.9707	1.0698	1.0530	0.8933	0.9967	0.0407
MSLN	1.0295	1.1111	1.0509	0.7953	0.9967	0.0693
LOC652222	0.9579	1.2508	0.9686	0.8096	0.9967	0.0921
LOC644586	0.9281	1.1524	1.0319	0.8746	0.9967	0.0613
LOC729793	1.0167	1.0384	1.0113	0.9205	0.9967	0.0261
ATPIF1	0.9950	1.2520	1.0666	0.6734	0.9967	0.1206
SSTR2	0.8829	1.1161	1.0791	0.9089	0.9967	0.0589
TTTY12	0.9130	1.1714	1.0412	0.8614	0.9968	0.0694
IRF5	0.8801	1.1063	1.1279	0.8728	0.9968	0.0696
LOC1001308	0.9770	1.1086	1.0586	0.8429	0.9968	0.0580
DYSF	0.9697	1.0659	1.0133	0.9382	0.9968	0.0277
LOC730329	0.9017	1.0608	1.0770	0.9475	0.9968	0.0428
CCDC13	0.9388	1.1269	0.9562	0.9651	0.9968	0.0437
LOC652779	0.8685	1.1732	1.0534	0.8920	0.9968	0.0718
ZCCHC10	0.9751	1.0586	0.9921	0.9612	0.9968	0.0216
LOC441869	1.0978	1.0245	0.9651	0.8996	0.9968	0.0422
LOC1001328	1.0190	1.0851	1.0109	0.8722	0.9968	0.0447
KRTAP4-12	1.0102	1.1104	1.0372	0.8294	0.9968	0.0597
TIAM1	0.9508	1.0641	1.0694	0.9029	0.9968	0.0416
LOC648365	1.0235	1.1277	0.9987	0.8373	0.9968	0.0600
LOC653483	1.0080	1.0874	0.9970	0.8948	0.9968	0.0395
FBXO17	0.9232	1.1252	1.0299	0.9090	0.9968	0.0506
SLCO1B1	0.8903	1.1163	1.0375	0.9432	0.9968	0.0501
LOC390671	1.0266	1.1038	0.9920	0.8648	0.9968	0.0498
PPIL6	1.0402	1.0574	1.0172	0.8725	0.9968	0.0423
FAM170A	0.8593	1.0412	1.1111	0.9757	0.9968	0.0535
GJD3	0.9939	1.1233	1.0402	0.8299	0.9968	0.0618
GSTM4	0.9011	1.0992	0.9964	0.9906	0.9968	0.0405
LOC442299	1.0104	1.0451	1.0675	0.8644	0.9968	0.0457
LOC147710	0.9203	1.1810	0.9376	0.9484	0.9968	0.0617
TNFSF15	0.7968	1.2111	1.1332	0.8464	0.9969	0.1029
YWHAE	1.0504	1.3775	0.8237	0.7358	0.9969	0.1431
CA3	0.9300	1.0651	0.9960	0.9963	0.9969	0.0276
LOC649137	0.9551	1.0301	1.1228	0.8794	0.9969	0.0520
LOC729978	0.8047	1.7852	0.6746	0.7230	0.9969	0.2641
LOC729732	1.0048	1.1185	0.9938	0.8705	0.9969	0.0507
LOC646482	0.8799	1.1512	1.0543	0.9023	0.9969	0.0644
LOC1001336	1.0154	1.0772	1.0606	0.8345	0.9969	0.0557
FABP6	0.8994	1.1276	0.9993	0.9613	0.9969	0.0482
UGT1A6	0.9698	1.0666	1.0061	0.9453	0.9969	0.0264
NPFF	0.9317	1.2077	1.0022	0.8461	0.9969	0.0772
LOC729715	0.9429	1.1927	0.9565	0.8957	0.9969	0.0665

FAM128A	1.0858	1.1180	1.0495	0.7344	0.9969	0.0886
FGF11	0.9875	1.0702	1.0666	0.8635	0.9970	0.0484
LOC650632	0.9387	0.9934	1.0668	0.9890	0.9970	0.0264
MIR508	0.8855	1.1951	0.9803	0.9270	0.9970	0.0688
OR6B2	0.9418	1.0732	1.0544	0.9185	0.9970	0.0391
SNX9	1.0026	0.9961	1.0596	0.9296	0.9970	0.0266
DDHD1	0.8987	1.1009	1.0937	0.8947	0.9970	0.0579
LOC653830	0.9294	1.0559	1.0882	0.9144	0.9970	0.0439
OR4C6	0.8912	1.1031	1.0612	0.9324	0.9970	0.0506
LOC730713	0.9939	1.0161	1.0781	0.8999	0.9970	0.0369
LOC642441	1.0525	1.0361	0.9707	0.9286	0.9970	0.0288
HMX1	0.9621	1.0999	0.9557	0.9704	0.9970	0.0344
C2orf32	0.8588	1.1056	1.0417	0.9819	0.9970	0.0525
MIR574	1.0763	1.0700	0.8925	0.9491	0.9970	0.0455
LOC1001293	1.0022	1.0620	1.0407	0.8832	0.9970	0.0399
MIR1275	0.9048	1.1938	0.9927	0.8968	0.9970	0.0691
C14orf105	0.9507	1.1100	1.0175	0.9099	0.9970	0.0437
DLX6AS	0.9207	1.1379	0.9907	0.9389	0.9970	0.0492
LOC728179	0.9397	1.0606	1.0791	0.9088	0.9970	0.0427
LOC1001301	0.9371	1.0628	0.9792	1.0090	0.9970	0.0264
LOC650767	1.0183	1.0622	1.0042	0.9034	0.9970	0.0336
OR2J2	0.8025	1.0607	1.1584	0.9665	0.9970	0.0758
LOC284620	1.0161	1.0032	1.1180	0.8509	0.9970	0.0551
ZNF208	0.9754	1.0576	1.0034	0.9518	0.9970	0.0228
C18orf20	0.9408	1.0534	1.1440	0.8501	0.9971	0.0642
LOC647461	0.9770	1.0651	1.0498	0.8964	0.9971	0.0387
LOC729731	0.8886	1.0889	1.0848	0.9259	0.9971	0.0524
LOC1001294	0.9331	1.0571	1.0889	0.9091	0.9971	0.0446
LOC643595	0.9532	1.0920	1.0480	0.8950	0.9971	0.0447
HIGD1C	0.9442	1.1271	1.0293	0.8876	0.9971	0.0522
VPS36	0.9487	1.1042	1.0738	0.8617	0.9971	0.0563
MIR147	0.8805	1.1560	1.0498	0.9021	0.9971	0.0650
SNORA49	0.9888	1.0970	0.9768	0.9258	0.9971	0.0360
PXN	0.8570	1.1043	1.0672	0.9599	0.9971	0.0558
KCNH7	0.9441	1.0973	1.0094	0.9375	0.9971	0.0371
OR2K2	1.0215	1.0627	1.0373	0.8669	0.9971	0.0442
PCDH11Y	0.9615	1.0768	1.0665	0.8836	0.9971	0.0459
DGCR10	1.0166	1.0443	1.0304	0.8972	0.9971	0.0338
OPALIN	0.9916	0.9768	1.0399	0.9802	0.9971	0.0146
LOC651311	0.9394	1.1282	1.0758	0.8451	0.9971	0.0644
LOC1001339	1.0556	1.0210	1.0136	0.8984	0.9971	0.0342
LOC1001301	0.9607	1.1018	1.0333	0.8928	0.9971	0.0452
CNDP1	0.9170	1.0568	1.0723	0.9426	0.9971	0.0394
LOC402677	1.0132	1.1717	1.0439	0.7598	0.9972	0.0862
OTX2	0.9703	1.1355	0.9576	0.9252	0.9972	0.0471
IKBKG	0.9840	1.2698	0.9289	0.8059	0.9972	0.0982

ADAD1	0.9095	1.1593	1.0422	0.8776	0.9972	0.0647
C6orf25	0.9600	1.0930	1.0058	0.9300	0.9972	0.0355
LOC652789	1.0097	1.2193	0.9514	0.8083	0.9972	0.0853
C2orf82	0.8955	1.2275	1.0423	0.8234	0.9972	0.0893
LOC1001323	0.9736	1.0732	1.0073	0.9345	0.9972	0.0294
LOC647199	1.0429	1.0668	0.9076	0.9714	0.9972	0.0361
ZNF233	1.0608	1.0093	1.0077	0.9109	0.9972	0.0313
SNORD6	0.9877	1.1563	0.9660	0.8787	0.9972	0.0580
TNIP1	0.8383	1.3443	0.9668	0.8394	0.9972	0.1196
CYP4F11	0.8540	1.1612	1.0939	0.8798	0.9972	0.0767
LOC728114	1.0073	1.0694	1.0467	0.8655	0.9972	0.0458
LOC650684	0.9421	1.1153	1.0449	0.8866	0.9972	0.0512
LOC1001309	0.9199	1.1452	1.0369	0.8869	0.9972	0.0589
CRTC1	1.0620	1.0900	0.9699	0.8670	0.9972	0.0504
LOC644508	0.9453	1.2096	0.9758	0.8582	0.9972	0.0751
SNORD60	1.0393	1.0823	0.9631	0.9042	0.9972	0.0396
LOC651968	1.0083	1.0449	1.0175	0.9183	0.9972	0.0274
GRP	0.9643	1.0782	1.0465	0.8999	0.9972	0.0403
LOC650860	0.8907	1.2628	1.0323	0.8032	0.9972	0.1003
SNORD102	0.9839	1.1275	1.0166	0.8609	0.9972	0.0549
TMEM176B	0.9054	1.1190	1.0473	0.9174	0.9972	0.0518
LOC728622	1.0195	1.0538	1.0557	0.8599	0.9972	0.0465
ZNF785	0.9069	1.0705	1.1377	0.8739	0.9972	0.0636
LOC1001300	0.9126	1.0800	1.1139	0.8825	0.9973	0.0583
KIT	0.9263	1.0586	1.0218	0.9823	0.9973	0.0283
SLC6A4	0.8952	1.0417	1.0448	1.0075	0.9973	0.0351
NPFFR1	0.9576	1.2352	0.9931	0.8032	0.9973	0.0894
PURB	0.8592	1.3969	0.9597	0.7734	0.9973	0.1385
ZNF17	0.9264	1.2361	1.0351	0.7916	0.9973	0.0939
CUBN	1.0257	1.0450	1.0482	0.8703	0.9973	0.0426
CAND2	0.9631	1.0335	1.0788	0.9138	0.9973	0.0366
LOC652684	0.9121	1.1383	1.0448	0.8940	0.9973	0.0578
LOC729176	0.9484	1.0929	1.1114	0.8367	0.9973	0.0648
UBASH3A	0.9134	1.0670	1.0478	0.9611	0.9973	0.0362
LOC650314	0.9062	1.0993	1.0119	0.9719	0.9973	0.0404
LOC729737	1.0134	1.0516	0.8805	1.0438	0.9973	0.0398
LOC652349	0.9322	1.1144	1.0109	0.9318	0.9973	0.0432
LOC642355	0.9064	1.1635	1.0220	0.8975	0.9973	0.0622
LOC257039	0.9970	1.1040	0.9914	0.8970	0.9973	0.0423
RERGL	0.8783	1.1350	1.0788	0.8973	0.9973	0.0644
CTSS	0.9375	0.9866	1.0936	0.9718	0.9973	0.0337
SLMO2	1.1028	1.0435	0.9577	0.8853	0.9974	0.0478
CABP1	0.9547	1.0611	1.0723	0.9013	0.9974	0.0416
ACOT6	0.8954	1.1048	1.0034	0.9858	0.9974	0.0429
LOC1001304	0.9395	1.0989	0.9134	1.0376	0.9974	0.0431
DNAH17	0.9544	0.9647	1.1054	0.9650	0.9974	0.0361

LOC1000096	0.8560	1.2065	1.1229	0.8042	0.9974	0.0987
GNE	1.0598	1.1250	0.9798	0.8249	0.9974	0.0647
TMEM26	0.8723	1.2101	0.9555	0.9516	0.9974	0.0735
NOL4	1.0547	1.0406	1.0237	0.8706	0.9974	0.0427
C16orf10	0.8815	1.2311	1.0008	0.8762	0.9974	0.0830
VAMP5	1.0755	1.0530	0.9716	0.8895	0.9974	0.0423
CEACAM7	0.9643	1.0511	1.0817	0.8925	0.9974	0.0429
LOC644898	0.9965	1.1575	0.9902	0.8455	0.9974	0.0637
LOC642833	1.0416	1.2561	0.8148	0.8772	0.9974	0.0986
CYB5D2	0.9443	1.6586	0.8063	0.5805	0.9974	0.2328
LOC148430	0.9567	1.0666	1.1443	0.8221	0.9974	0.0700
LOC645312	0.8822	1.0964	1.0591	0.9521	0.9974	0.0491
LOC402217	1.0199	1.0647	0.9446	0.9605	0.9974	0.0277
H2BFWT	0.9005	1.0327	1.0963	0.9602	0.9974	0.0426
NHLH2	0.9093	1.0310	1.1094	0.9400	0.9974	0.0454
MYOZ1	0.9425	1.0479	1.0813	0.9181	0.9974	0.0397
LOC441378	1.0342	1.0435	0.9561	0.9559	0.9974	0.0240
LOC146053	0.9521	1.0854	1.0085	0.9438	0.9974	0.0327
REP15	0.8781	1.1079	1.0870	0.9168	0.9974	0.0584
LOC650013	1.0052	1.0853	0.9257	0.9736	0.9974	0.0335
CTSW	0.9323	1.0571	1.0357	0.9647	0.9975	0.0294
LOC442560	1.0257	1.1181	1.0491	0.7970	0.9975	0.0696
LOC647961	0.9686	1.0504	1.0590	0.9119	0.9975	0.0351
LOC644769	0.9836	1.1404	0.9989	0.8670	0.9975	0.0560
ZBTB34	0.9416	1.2783	0.9843	0.7857	0.9975	0.1029
LOC1001294	0.9545	0.9567	1.0828	0.9959	0.9975	0.0300
DIO1	0.9087	1.2565	1.0259	0.7988	0.9975	0.0980
MAPK15	0.8981	1.0974	0.9636	1.0308	0.9975	0.0429
ZNF98	0.8978	1.0780	1.0948	0.9194	0.9975	0.0516
CDKL1	0.9120	1.1571	0.9858	0.9351	0.9975	0.0554
KLRG1	0.9099	1.0757	1.0626	0.9418	0.9975	0.0420
LOC727849	0.8407	1.1697	1.1169	0.8628	0.9975	0.0850
LOC648758	0.9171	1.1521	1.0681	0.8527	0.9975	0.0685
YTHDC1	0.7866	1.3533	1.0658	0.7843	0.9975	0.1358
FLJ27354	0.9736	1.1017	0.9976	0.9171	0.9975	0.0386
PRKAB1	1.0185	1.2909	0.9505	0.7302	0.9975	0.1155
LOC1001304	0.8766	1.1231	1.0181	0.9723	0.9975	0.0512
LCNL1	0.8740	1.1038	0.9634	1.0489	0.9975	0.0503
LOC1001319	0.9929	1.1763	0.9053	0.9156	0.9975	0.0627
MIR326	0.8476	1.2015	1.0523	0.8887	0.9975	0.0811
LOC390578	0.8887	1.1700	1.1338	0.7976	0.9975	0.0913
MAMDC4	0.9389	1.1013	0.9811	0.9688	0.9975	0.0357
MGC71805	0.8847	1.2015	0.9813	0.9227	0.9975	0.0708
GIMAP1	0.9511	1.0954	1.0099	0.9338	0.9975	0.0365
LOC647947	0.9325	1.1681	0.9867	0.9029	0.9975	0.0594
C15orf62	1.0827	1.1399	0.9373	0.8303	0.9975	0.0702

TIMM50	0.9911	1.0782	1.0647	0.8562	0.9975	0.0509
HLA-DQA1	0.9126	1.0923	1.0922	0.8930	0.9975	0.0548
CPLX3	1.0410	1.0307	1.0363	0.8822	0.9976	0.0385
LOC1001305	0.9477	1.1332	0.9926	0.9167	0.9976	0.0478
LIMS3	0.8929	1.1036	1.0618	0.9321	0.9976	0.0505
PSG11	0.9183	1.1316	1.0187	0.9217	0.9976	0.0504
SPG3A	1.0449	1.1230	0.9475	0.8749	0.9976	0.0544
LOC1001325	0.9675	1.0755	1.0579	0.8895	0.9976	0.0431
SLC6A12	0.9687	1.0390	1.0414	0.9412	0.9976	0.0252
LOC1001348	0.8350	1.2995	0.9030	0.9528	0.9976	0.1035
ADAMTS19	1.0545	0.9295	1.0698	0.9365	0.9976	0.0374
SMAD4	0.8990	1.2947	1.0610	0.7357	0.9976	0.1192
HSH2D	0.9996	1.2008	0.9464	0.8435	0.9976	0.0751
OLIG2	0.9174	1.1928	1.0221	0.8581	0.9976	0.0734
LOC441698	0.8893	1.1453	0.9685	0.9872	0.9976	0.0536
LOC651137	0.8841	1.1551	1.0109	0.9404	0.9976	0.0585
PITPNM3	1.0170	1.0106	1.0085	0.9543	0.9976	0.0145
KLHL30	0.9874	1.0149	1.0814	0.9068	0.9976	0.0361
LOC1001330	0.9296	1.0652	1.0137	0.9819	0.9976	0.0284
LOC402635	1.0461	1.0898	0.9550	0.8996	0.9976	0.0431
LOC729826	0.9456	1.1479	0.9610	0.9360	0.9976	0.0503
OR1L4	0.9601	1.0618	1.0653	0.9033	0.9976	0.0398
CXCL3	0.9491	1.0336	1.0850	0.9228	0.9976	0.0375
XGPY2	1.0047	1.0952	1.0233	0.8674	0.9976	0.0476
PTGER3	0.9459	1.0780	1.0721	0.8946	0.9976	0.0459
AKAP9	0.9352	1.0957	1.0087	0.9509	0.9976	0.0363
C7orf69	0.9606	1.1270	1.0430	0.8599	0.9976	0.0571
LOC644250	0.8431	1.2853	1.0607	0.8014	0.9976	0.1115
ILDR2	0.9919	1.0615	1.0236	0.9136	0.9976	0.0314
ADAMTS9	0.9764	1.0777	1.0275	0.9089	0.9976	0.0361
LOC653715	0.9401	1.0298	1.0002	1.0205	0.9976	0.0202
MFNG	0.9001	1.1477	1.0651	0.8777	0.9976	0.0652
DENND1C	0.9718	1.1640	1.0666	0.7881	0.9976	0.0801
FBXL4	0.9988	1.0697	1.0021	0.9201	0.9977	0.0306
SPOCD1	0.9398	1.0687	1.0781	0.9041	0.9977	0.0444
LOC1001342	0.9323	1.1018	1.0524	0.9041	0.9977	0.0473
LOC642312	0.9135	1.1410	1.0927	0.8434	0.9977	0.0710
MIR134	0.9048	1.1467	0.9286	1.0105	0.9977	0.0546
LOC1001298	1.0295	1.0445	1.0245	0.8922	0.9977	0.0354
SLC16A6	0.9303	1.1957	1.0812	0.7834	0.9977	0.0897
DSG3	1.0194	1.0754	0.9391	0.9568	0.9977	0.0311
LOC1001293	0.9659	1.1388	1.0562	0.8298	0.9977	0.0662
USHBP1	0.9898	1.0613	1.0013	0.9383	0.9977	0.0252
LOC649337	0.9607	1.0306	1.0288	0.9706	0.9977	0.0186
LOC201175	0.9243	1.1750	0.9761	0.9154	0.9977	0.0606
C3orf30	0.9435	1.0490	1.0959	0.9024	0.9977	0.0450

LOC441584	0.9903	0.9515	1.2039	0.8451	0.9977	0.0753
NIPBL	0.9153	1.2732	0.9968	0.8056	0.9977	0.0998
LOC647210	0.9007	1.2022	1.0392	0.8488	0.9977	0.0791
OC90	0.9155	1.0672	1.1355	0.8727	0.9977	0.0621
CREG2	0.9894	1.1258	0.9887	0.8870	0.9977	0.0490
IFNA14	1.0022	1.0496	1.0210	0.9181	0.9977	0.0283
ARVCF	1.0000	1.1884	0.9271	0.8754	0.9977	0.0685
STARD5	0.8878	1.2141	1.0626	0.8264	0.9977	0.0878
CASP6	0.9721	1.1030	1.0680	0.8479	0.9977	0.0571
OR12D3	0.9262	1.0969	1.1016	0.8663	0.9977	0.0599
FLJ27365	1.1378	1.1599	0.9260	0.7673	0.9977	0.0932
CDAN1	0.9163	1.2579	0.9928	0.8240	0.9977	0.0933
LOC644695	0.9838	1.0770	1.0340	0.8962	0.9978	0.0388
STS-1	0.8804	1.0473	1.0505	1.0128	0.9978	0.0400
OR4X2	0.9911	1.0403	1.0407	0.9190	0.9978	0.0287
FNDC1	1.0472	1.0674	0.9632	0.9133	0.9978	0.0361
LOC728764	1.0262	1.0468	0.9884	0.9296	0.9978	0.0257
LOC441179	0.9147	1.0472	1.0831	0.9460	0.9978	0.0401
NRP2	0.9550	1.0488	1.0166	0.9708	0.9978	0.0214
FLJ36777	0.9053	1.0000	1.1278	0.9580	0.9978	0.0475
SLC4A3	1.0203	1.1404	0.9807	0.8498	0.9978	0.0599
ANO5	1.0470	1.0737	0.9800	0.8904	0.9978	0.0409
MTPAP	0.9537	1.1149	1.0365	0.8861	0.9978	0.0497
LOC643227	1.0429	0.9375	1.0674	0.9435	0.9978	0.0335
PRAMEF10	0.9025	1.0989	1.0204	0.9694	0.9978	0.0414
ZNF510	0.8805	1.1658	1.0496	0.8953	0.9978	0.0678
ZNF517	0.9330	1.1586	1.0337	0.8659	0.9978	0.0637
LOC730249	0.9624	1.0786	1.0408	0.9094	0.9978	0.0381
PYDC2	0.9391	1.0675	1.0494	0.9352	0.9978	0.0352
ZNF807	0.9531	1.0671	1.1153	0.8558	0.9978	0.0583
LOC1001314	1.0989	0.9452	0.9857	0.9615	0.9978	0.0347
KIF6	0.9901	1.1181	0.8512	1.0318	0.9978	0.0557
MGC10701	0.8871	1.1349	1.0195	0.9498	0.9978	0.0531
C3	0.9676	1.1220	1.0560	0.8457	0.9978	0.0598
LOC283152	1.0250	1.0104	1.0531	0.9029	0.9978	0.0329
LOC646607	0.8646	1.0677	1.1039	0.9553	0.9979	0.0545
LOC730256	0.8957	1.1513	1.1552	0.7891	0.9979	0.0923
MIR2276	1.0243	0.9124	1.0321	1.0227	0.9979	0.0286
MIR940	0.9007	1.1239	1.0397	0.9272	0.9979	0.0517
LOC645321	0.9499	1.0458	1.0312	0.9646	0.9979	0.0238
LOC1001337	0.9765	1.0167	1.0329	0.9654	0.9979	0.0160
PDRG1	0.8043	1.4868	0.9620	0.7384	0.9979	0.1696
FABP5L7	0.9610	1.0891	0.9683	0.9731	0.9979	0.0305
LOC650631	1.0112	1.0691	0.9575	0.9537	0.9979	0.0271
LOC1001322	0.8579	1.1821	1.0288	0.9228	0.9979	0.0708
CENTD1	0.8748	1.1120	1.0847	0.9201	0.9979	0.0590

WIPF1	0.9465	1.0638	1.0058	0.9754	0.9979	0.0251
LOC649963	0.9446	1.1886	1.0032	0.8552	0.9979	0.0705
RNF190	1.0252	1.0252	1.0234	0.9178	0.9979	0.0267
ATCAY	0.8899	1.1327	1.0864	0.8825	0.9979	0.0652
DMBT1	0.9422	1.1080	1.0098	0.9316	0.9979	0.0406
ADAMTS10	1.0792	1.0986	0.9581	0.8557	0.9979	0.0567
LOC648605	0.8893	1.1399	1.0621	0.9003	0.9979	0.0617
LOC646519	0.9214	1.1051	1.0221	0.9430	0.9979	0.0418
MUPCDH	0.9109	1.1351	1.0548	0.8909	0.9979	0.0585
DHCR7	0.8788	1.4881	0.9597	0.6651	0.9979	0.1748
LOC648138	1.0026	1.0510	1.0669	0.8712	0.9979	0.0444
SIX2	0.9274	1.0122	1.2022	0.8499	0.9979	0.0757
LOC1001322	0.9634	1.0741	1.0217	0.9326	0.9979	0.0314
RFPL1S	0.8479	1.1277	1.0338	0.9823	0.9979	0.0584
LOC728196	0.9507	1.0879	0.9701	0.9830	0.9979	0.0307
LOC647650	0.9080	1.1585	1.0733	0.8519	0.9979	0.0712
LOC653746	0.9886	1.1241	0.9250	0.9540	0.9979	0.0440
NPM2	0.9349	1.0620	1.1198	0.8750	0.9979	0.0563
LOC1001280	0.9254	1.0613	1.0029	1.0022	0.9979	0.0279
LOC728767	0.9807	1.0108	1.0356	0.9647	0.9979	0.0158
FAM35A	0.9804	1.1907	1.0181	0.8025	0.9979	0.0796
LOC390387	0.9226	1.0733	1.0463	0.9496	0.9980	0.0366
LOC729061	0.9173	1.1520	0.9795	0.9431	0.9980	0.0529
LOC650217	1.1025	1.0424	1.0054	0.8417	0.9980	0.0558
LOC121296	0.9419	1.0850	1.0466	0.9184	0.9980	0.0402
HBBP1	0.9526	1.0557	1.1072	0.8764	0.9980	0.0517
KCND1	0.8747	1.1246	1.0376	0.9551	0.9980	0.0537
APOBEC3A	0.9353	1.1010	1.0359	0.9198	0.9980	0.0429
ERAS	0.9661	1.1166	1.0375	0.8718	0.9980	0.0521
NRG2	0.9069	1.1000	1.0644	0.9207	0.9980	0.0492
LOC92154	0.9926	1.1171	0.9611	0.9213	0.9980	0.0423
MGAT5B	0.9771	1.0443	1.0749	0.8957	0.9980	0.0397
RB1	0.8081	1.4216	0.9670	0.7953	0.9980	0.1465
LOC1001336	0.9486	1.0297	1.0557	0.9581	0.9980	0.0264
MEGF8	0.9390	1.1035	1.0667	0.8829	0.9980	0.0521
C8orf12	0.9169	1.1407	1.0078	0.9267	0.9980	0.0517
LOC728088	0.9129	1.1147	1.0255	0.9390	0.9980	0.0457
LOC644999	1.0593	1.2231	0.9236	0.7862	0.9980	0.0935
BBX	0.8643	1.4526	0.9835	0.6918	0.9981	0.1629
CAPN14	0.9462	1.2112	0.9819	0.8530	0.9981	0.0761
LOC283116	0.9756	1.0814	1.0320	0.9032	0.9981	0.0383
RUFY4	0.9624	1.0706	1.0528	0.9065	0.9981	0.0386
ROBO2	0.9407	1.1052	1.0310	0.9154	0.9981	0.0435
LOC646260	0.9322	1.0721	1.0345	0.9535	0.9981	0.0331
LOC1001281	0.8191	1.2284	1.0214	0.9234	0.9981	0.0872
FMO1	1.0328	0.8893	1.0684	1.0018	0.9981	0.0387

TRIM72	1.0840	0.9946	0.8295	1.0842	0.9981	0.0600
LOC653123	1.0750	1.0059	1.0221	0.8893	0.9981	0.0392
LOC653764	0.9496	1.1386	1.0680	0.8362	0.9981	0.0666
LOC646047	0.8951	1.0932	1.0466	0.9576	0.9981	0.0444
ADD2	0.9749	1.0892	1.0369	0.8915	0.9981	0.0425
LOC644390	0.9416	1.1128	1.1023	0.8358	0.9981	0.0668
TSN	0.9675	1.0814	1.0160	0.9276	0.9981	0.0331
FBXO44	0.9447	1.0998	1.0097	0.9384	0.9981	0.0375
MIR548C	0.8671	1.1373	1.0160	0.9721	0.9981	0.0559
LOC392522	0.9125	1.0489	1.1511	0.8801	0.9981	0.0627
NCF4	0.9920	1.0501	1.0172	0.9334	0.9981	0.0247
MIR575	0.9568	1.0954	1.0307	0.9098	0.9981	0.0409
UCMA	0.8861	1.0879	1.0684	0.9502	0.9982	0.0482
PDCL2	0.9205	1.0928	0.9855	0.9938	0.9982	0.0355
LOC645373	0.9858	1.0427	1.0423	0.9219	0.9982	0.0287
KRTAP4-3	0.9841	1.0783	1.0607	0.8695	0.9982	0.0475
IL28A	0.9954	1.0434	1.0802	0.8737	0.9982	0.0450
IL11RA	0.9791	1.1376	1.0039	0.8721	0.9982	0.0546
SPRY3	0.9308	1.0341	1.0347	0.9931	0.9982	0.0245
INPP1	0.8773	1.1636	0.9860	0.9659	0.9982	0.0600
C1orf27	0.9389	1.0619	1.0279	0.9641	0.9982	0.0283
LOC727788	0.9545	1.0251	1.0561	0.9571	0.9982	0.0253
FAM179B	0.9311	1.2196	1.0518	0.7904	0.9982	0.0911
AGPAT9	0.9630	1.1498	1.0332	0.8469	0.9982	0.0635
GJA5	0.9957	1.1213	0.9792	0.8967	0.9982	0.0464
TRIM67	1.0366	1.0256	1.0130	0.9177	0.9982	0.0273
RESP18	0.9694	1.0945	0.9533	0.9757	0.9982	0.0324
LOC441193	1.0601	0.9818	0.9179	1.0332	0.9982	0.0313
LOC651096	1.0547	1.0734	1.0188	0.8461	0.9982	0.0520
ATP9B	0.9180	1.1912	1.0333	0.8505	0.9982	0.0746
GSTZ1	0.8448	1.2068	1.0606	0.8808	0.9983	0.0840
LOC284749	0.9025	1.1320	1.0013	0.9571	0.9983	0.0490
LONRF2	0.9246	1.1085	0.9957	0.9642	0.9983	0.0395
NR5A2	0.9511	1.0087	1.0833	0.9498	0.9983	0.0315
LOC653217	0.9468	1.0933	1.0456	0.9073	0.9983	0.0430
PLK3	0.9480	1.1087	0.9789	0.9575	0.9983	0.0374
LOC641805	1.0003	1.0525	0.9999	0.9404	0.9983	0.0229
LOC728119	0.9224	1.2369	0.9701	0.8637	0.9983	0.0825
LOC645737	0.9862	1.0857	1.1342	0.7870	0.9983	0.0769
LOC652697	1.0326	1.0904	1.0688	0.8014	0.9983	0.0667
LOC442117	0.9271	1.1083	0.9853	0.9725	0.9983	0.0387
LOC648527	0.9914	1.0604	1.0536	0.8878	0.9983	0.0400
LOC644646	0.9869	0.9825	1.1080	0.9158	0.9983	0.0400
TNRC15	1.0279	1.2356	0.9369	0.7929	0.9983	0.0927
LOC649034	1.0659	1.1161	0.8480	0.9632	0.9983	0.0593
LOC1001323	1.0088	1.0122	1.0663	0.9059	0.9983	0.0335

ERCC-00092	0.9320	1.1483	1.0481	0.8649	0.9983	0.0627
LOC1001296	1.0025	1.1312	1.0203	0.8392	0.9983	0.0602
LOC647254	1.0267	1.1487	0.9801	0.8380	0.9983	0.0642
FXN	1.0213	1.0154	1.0948	0.8619	0.9984	0.0489
SPDEF	1.1244	1.1488	0.9342	0.7860	0.9984	0.0855
LOC645712	1.0935	1.0102	0.9665	0.9233	0.9984	0.0363
ARF6	0.8395	1.1187	1.0460	0.9893	0.9984	0.0592
LOC1001347	1.0040	1.0536	1.0306	0.9053	0.9984	0.0326
IGFL3	0.9462	1.1056	0.9273	1.0143	0.9984	0.0403
LOC648153	1.0756	1.0144	0.9477	0.9559	0.9984	0.0297
PCDHAC1	0.9349	1.0266	1.0341	0.9980	0.9984	0.0226
ANXA11	0.8478	1.2877	0.9780	0.8800	0.9984	0.1003
LOC653043	1.0632	1.1342	0.9336	0.8625	0.9984	0.0614
FGF14	0.9604	1.1002	1.0048	0.9281	0.9984	0.0374
CNNM4	0.9668	1.1852	1.0149	0.8268	0.9984	0.0739
LOC646064	0.8966	1.1297	1.0789	0.8885	0.9984	0.0620
LOC653427	1.0613	1.0730	1.0429	0.8164	0.9984	0.0610
MIR374B	1.0304	1.1044	0.9825	0.8764	0.9984	0.0478
LOC646029	0.9878	1.1350	1.0051	0.8658	0.9984	0.0551
SNORD109A	1.0180	0.9675	1.1257	0.8825	0.9984	0.0508
LOC1001290	0.9446	1.0713	1.0261	0.9519	0.9984	0.0305
PADI1	0.9588	1.1166	1.0499	0.8685	0.9984	0.0541
LOC652710	0.9583	1.0408	1.0627	0.9321	0.9985	0.0315
LOC1001295	0.8527	1.1172	1.3078	0.7162	0.9985	0.1325
LOC1001297	0.9324	1.0758	1.0457	0.9400	0.9985	0.0365
HCG27	0.9531	1.0986	1.0294	0.9128	0.9985	0.0412
AANAT	1.0567	1.0565	0.9685	0.9123	0.9985	0.0355
LRRC3B	0.9949	1.0152	1.0104	0.9734	0.9985	0.0094
CALCA	0.9126	1.1199	1.0357	0.9258	0.9985	0.0490
DEFB125	0.8925	1.2256	0.9565	0.9194	0.9985	0.0768
LOC654346	0.9663	1.1064	1.0629	0.8585	0.9985	0.0551
LOC388282	1.0353	1.1053	0.9818	0.8716	0.9985	0.0493
GLP1R	0.8995	1.0831	1.0180	0.9935	0.9985	0.0380
LOC647515	0.9594	1.1487	0.9839	0.9021	0.9985	0.0529
LOC646272	0.9331	1.0717	1.1319	0.8574	0.9985	0.0628
RNASE12	0.9826	1.0584	1.0129	0.9404	0.9985	0.0249
LOC1001336	0.9070	1.2261	1.0382	0.8229	0.9986	0.0878
SMYD1	0.9787	1.0697	1.0901	0.8558	0.9986	0.0534
BTBD15	0.9359	1.0915	0.9756	0.9913	0.9986	0.0331
LOC651361	0.8836	1.1001	1.0686	0.9420	0.9986	0.0513
LMO1	0.9090	1.1243	1.0532	0.9078	0.9986	0.0540
RNF217	0.9889	1.0154	1.0370	0.9531	0.9986	0.0181
LOC392008	1.0621	1.0617	0.9842	0.8862	0.9986	0.0417
FLJ45966	0.8711	1.1186	1.0876	0.9171	0.9986	0.0614
CAPN12	1.0033	1.0753	1.0375	0.8783	0.9986	0.0427
LOC729623	0.9486	1.1383	0.9617	0.9458	0.9986	0.0467

LOC1001319	1.0426	1.0701	1.0002	0.8815	0.9986	0.0416
CHMP4C	0.8992	1.1545	1.2331	0.7077	0.9986	0.1204
MIR302C	0.9433	1.1081	0.9789	0.9642	0.9986	0.0372
OR5L1	0.9884	1.0645	1.0002	0.9413	0.9986	0.0254
FAM90A8	0.9764	1.0887	0.9722	0.9571	0.9986	0.0303
CABYR	0.9278	1.1321	0.9991	0.9356	0.9987	0.0473
LOC391727	0.9197	1.1099	1.0832	0.8818	0.9987	0.0573
MIR1290	1.0072	1.1710	0.9025	0.9140	0.9987	0.0620
GSTTP1	1.0704	1.1102	0.9378	0.8764	0.9987	0.0549
BARX1	0.8924	1.2266	1.0586	0.8170	0.9987	0.0912
TSSK6	1.0460	1.0697	1.0466	0.8324	0.9987	0.0557
SAMD4B	0.9554	1.3312	0.9173	0.7908	0.9987	0.1163
LOC647544	0.9890	1.0190	1.0333	0.9534	0.9987	0.0177
SFTPB	0.9014	1.1367	1.0234	0.9332	0.9987	0.0528
RGS5	0.9793	1.0452	1.0483	0.9219	0.9987	0.0301
LOC388882	0.8883	1.1100	1.0456	0.9508	0.9987	0.0492
C11orf76	0.9183	1.0563	1.0643	0.9559	0.9987	0.0364
TEX14	1.0083	1.0423	0.9820	0.9622	0.9987	0.0173
SPSB4	0.9220	1.1216	1.0833	0.8680	0.9987	0.0614
IFNW1	0.9665	1.0664	1.0128	0.9491	0.9987	0.0263
ITSN1	0.9403	1.3872	0.8916	0.7757	0.9987	0.1340
LOC641767	0.9237	1.1119	1.0140	0.9452	0.9987	0.0424
KHDC1L	0.9813	1.1164	0.9357	0.9615	0.9987	0.0403
DCT	1.0136	1.0725	0.9411	0.9677	0.9987	0.0288
HOXA7	0.8657	1.0629	1.0751	0.9911	0.9987	0.0480
LOC729629	0.9379	1.1011	1.0347	0.9212	0.9987	0.0423
RSPO4	0.9602	1.0626	0.9948	0.9773	0.9987	0.0224
LOC647596	0.8776	1.2552	0.9423	0.9198	0.9987	0.0865
MIR1468	0.9801	1.0765	1.0275	0.9107	0.9987	0.0353
LOC642516	0.9701	1.1315	0.9878	0.9054	0.9987	0.0477
CNN3	0.9362	1.0487	1.0521	0.9580	0.9987	0.0302
PNMT	0.8785	1.1453	1.0462	0.9250	0.9987	0.0603
LOC441320	0.9082	1.2380	0.9738	0.8748	0.9987	0.0824
LOC158730	0.9400	1.1108	1.0744	0.8698	0.9987	0.0565
OR2A1	0.9810	1.0885	1.0818	0.8438	0.9988	0.0572
LOC644377	0.8708	1.1954	1.0222	0.9066	0.9988	0.0731
LOC284998	0.8264	1.1490	1.1757	0.8440	0.9988	0.0947
FLJ46230	0.9445	1.0148	1.0498	0.9859	0.9988	0.0223
OSTN	0.9075	1.1088	1.0335	0.9453	0.9988	0.0452
ZC3H11B	0.9981	1.2651	1.0385	0.6935	0.9988	0.1175
C1orf175	0.9103	1.0645	1.0668	0.9535	0.9988	0.0396
CTDP1	0.8678	1.2604	1.0485	0.8185	0.9988	0.1002
B4GALT6	1.0395	0.9945	1.0768	0.8844	0.9988	0.0417
ZIC4	0.9529	1.1010	0.9864	0.9549	0.9988	0.0349
AMT	1.0549	0.9798	0.9891	0.9714	0.9988	0.0190
LONRF3	1.0408	1.0151	1.0542	0.8850	0.9988	0.0388

LOC652809	0.9003	1.1810	0.9980	0.9159	0.9988	0.0644
LOC728460	0.9429	1.0824	0.9653	1.0047	0.9988	0.0307
LOC1001301	1.0108	1.0944	1.0548	0.8352	0.9988	0.0571
LOC650148	0.8871	1.0354	1.1113	0.9614	0.9988	0.0482
CDH17	0.8941	1.1108	1.0570	0.9334	0.9988	0.0510
LOC652625	0.9158	1.1120	1.0096	0.9580	0.9988	0.0423
LOC644043	1.0331	1.0746	1.0290	0.8585	0.9988	0.0479
PSD2	0.8695	1.2048	1.0597	0.8613	0.9988	0.0825
LOC1001280	0.9178	1.1067	1.0214	0.9494	0.9988	0.0420
TRPC6	0.9469	1.0263	1.1084	0.9137	0.9988	0.0435
LOC650368	0.9600	1.0725	1.0809	0.8820	0.9988	0.0477
ABCC4	0.8732	1.3198	1.0771	0.7253	0.9988	0.1290
C17orf64	0.8487	1.1255	1.0681	0.9530	0.9989	0.0616
LOC1001298	0.9331	1.0779	1.0623	0.9221	0.9989	0.0413
TPH1	0.8693	1.1350	1.1124	0.8787	0.9989	0.0723
GMFG	0.8550	1.1769	1.0996	0.8640	0.9989	0.0820
C15orf41	1.0228	1.0865	1.0237	0.8624	0.9989	0.0479
LOC196549	0.9216	1.1671	0.9640	0.9427	0.9989	0.0567
SPOCK2	0.8621	1.1829	1.0336	0.9169	0.9989	0.0710
LOC1001318	0.8667	1.1396	1.0687	0.9205	0.9989	0.0634
BMP8B	0.8951	1.1025	0.9905	1.0074	0.9989	0.0425
ANXA8	1.0334	1.0771	0.9882	0.8968	0.9989	0.0386
BTC	0.9145	1.2165	0.9787	0.8859	0.9989	0.0751
ARGFX	1.0278	1.0684	0.9751	0.9243	0.9989	0.0314
MXD1	1.0225	1.1802	0.9862	0.8067	0.9989	0.0767
LOC650009	0.9673	1.0036	1.1221	0.9026	0.9989	0.0461
LOC646676	0.9505	1.0475	1.0740	0.9237	0.9989	0.0365
SLC5A5	0.9507	1.0543	1.0030	0.9877	0.9989	0.0215
SGCZ	0.8745	1.1547	1.0236	0.9429	0.9989	0.0602
KRTAP5-11	0.9152	1.0618	1.0904	0.9283	0.9989	0.0450
MID1	0.9700	1.1713	0.9853	0.8691	0.9989	0.0630
LOC348021	0.9209	1.1007	0.9186	1.0554	0.9989	0.0466
LOC645644	1.0146	1.0458	0.9805	0.9549	0.9989	0.0198
LOC340113	0.9506	1.0623	1.0582	0.9248	0.9990	0.0358
SLC11A1	0.9350	1.0932	1.0202	0.9474	0.9990	0.0366
SLC18A2	1.0016	1.0938	0.9351	0.9653	0.9990	0.0344
LOC388248	0.9581	1.1531	1.0082	0.8765	0.9990	0.0581
LOC728095	1.0639	0.9804	1.0565	0.8951	0.9990	0.0394
LOC143941	0.9602	1.0784	1.0542	0.9032	0.9990	0.0409
OPN5	0.9827	1.1872	0.9703	0.8557	0.9990	0.0690
CAMK2N2	0.9646	1.0005	1.1115	0.9192	0.9990	0.0410
SDCBP2	0.9495	1.0523	1.0719	0.9222	0.9990	0.0371
MIR1271	0.9808	1.0913	1.0194	0.9045	0.9990	0.0389
EDF1	0.9502	1.2168	1.1066	0.7224	0.9990	0.1072
OR4D6	0.9744	1.1042	0.9959	0.9215	0.9990	0.0384
LOC1001325	0.9219	1.1428	1.0308	0.9004	0.9990	0.0558

LOC1001324	0.9472	1.0192	1.0756	0.9539	0.9990	0.0303
SVOP	1.1098	1.0032	0.9946	0.8885	0.9990	0.0452
TOP3B	0.8794	1.2162	1.0812	0.8191	0.9990	0.0916
MIR328	1.0329	1.0772	1.0344	0.8514	0.9990	0.0502
MIR578	1.0384	0.9956	1.0726	0.8893	0.9990	0.0398
C6orf142	0.9796	0.9965	1.0699	0.9501	0.9990	0.0255
RD3	0.8068	1.2700	1.0294	0.8899	0.9990	0.1013
LOC1001299	0.9813	1.0498	1.0286	0.9363	0.9990	0.0253
RNF183	0.8931	1.1426	1.0892	0.8712	0.9990	0.0685
ATP5B	0.8864	1.1757	1.0732	0.8607	0.9990	0.0756
GJA8	1.0508	0.9862	1.1022	0.8568	0.9990	0.0530
LOC1001315	0.9148	1.1982	1.0467	0.8364	0.9990	0.0793
LOC653293	1.0189	1.0702	1.0418	0.8652	0.9990	0.0458
ZNF423	0.9599	1.0981	1.0697	0.8684	0.9990	0.0527
UBB	0.8697	1.3321	1.0402	0.7542	0.9990	0.1256
CCDC147	0.9075	1.1099	1.0315	0.9472	0.9990	0.0451
LOC1001328	1.0379	1.0773	1.0161	0.8649	0.9990	0.0465
C15orf58	0.9553	1.1778	0.9566	0.9065	0.9990	0.0607
LOC1002160	0.8915	1.1025	0.9888	1.0134	0.9991	0.0434
SGSM1	0.9477	1.0925	1.0405	0.9155	0.9991	0.0409
OR52N1	0.8766	1.0600	1.1640	0.8956	0.9991	0.0687
KRT34	0.9501	1.0602	1.0844	0.9016	0.9991	0.0437
BDH2	0.9494	1.1720	0.9753	0.8997	0.9991	0.0597
GABRD	0.9142	1.1928	0.9676	0.9217	0.9991	0.0656
SLC27A6	0.8711	1.1633	1.0471	0.9149	0.9991	0.0663
SNAP47	0.8561	1.3991	0.9369	0.8042	0.9991	0.1361
LOC644672	0.9304	1.1631	1.0274	0.8755	0.9991	0.0631
OXGR1	1.0216	1.0032	1.0505	0.9211	0.9991	0.0278
FLJ39061	0.9173	1.1339	1.0199	0.9253	0.9991	0.0506
FAM53C	0.8830	1.3227	1.0477	0.7429	0.9991	0.1246
CT45A5	0.9618	1.0221	1.0993	0.9132	0.9991	0.0401
CCDC39	1.0009	1.0192	0.9606	1.0158	0.9991	0.0134
OR56A1	0.9763	0.9836	1.0832	0.9533	0.9991	0.0288
MAPT	0.9746	1.2080	0.9840	0.8299	0.9991	0.0780
ART5	0.9927	1.1014	0.9241	0.9782	0.9991	0.0372
LOC1001293	1.0334	1.0721	0.9941	0.8969	0.9991	0.0376
LOC644402	0.9109	1.1300	1.0828	0.8728	0.9991	0.0632
C16orf78	1.0106	1.1314	0.9926	0.8618	0.9991	0.0552
ACSM5	0.9493	1.0803	1.0352	0.9317	0.9991	0.0353
MIR1322	0.9941	1.0815	0.9998	0.9211	0.9991	0.0328
SH3RF1	0.8735	1.3588	1.0788	0.6854	0.9991	0.1443
LOC196120	0.9701	1.0660	1.0915	0.8689	0.9991	0.0507
OR51A4	0.9543	1.1282	0.9446	0.9694	0.9991	0.0433
OR11L1	0.8967	1.0428	1.1208	0.9363	0.9991	0.0509
T-SP1	0.9650	1.2245	0.9708	0.8363	0.9991	0.0813
LOC93556	1.0077	0.8801	1.1037	1.0051	0.9992	0.0458

FLJ35429	0.9133	1.0741	1.2139	0.7954	0.9992	0.0916
HGFAC	0.9208	1.1278	1.0202	0.9278	0.9992	0.0485
RHOH	1.0218	1.0228	1.0450	0.9071	0.9992	0.0312
KERA	0.9058	1.1329	1.0604	0.8977	0.9992	0.0582
LOC650836	0.9521	1.0966	1.0245	0.9235	0.9992	0.0388
LOC649477	1.0241	1.0507	1.0473	0.8746	0.9992	0.0420
LOC651864	0.9347	1.1051	1.0124	0.9445	0.9992	0.0393
MSMB	0.9637	1.0670	1.0239	0.9421	0.9992	0.0285
NT5C2	0.8634	1.4354	1.0529	0.6452	0.9992	0.1676
LOC441548	0.9704	0.9446	1.1408	0.9409	0.9992	0.0477
MIR133B	0.9995	0.9177	1.0491	1.0304	0.9992	0.0290
KLHL7	0.9639	1.1636	1.0791	0.7903	0.9992	0.0808
DPYSL4	0.9558	1.2600	1.0273	0.7538	0.9992	0.1045
LOC1001324	0.9166	1.0547	1.1485	0.8771	0.9992	0.0627
LOC653878	0.9238	1.0596	1.0539	0.9596	0.9992	0.0340
ZNF766	0.9686	1.3744	0.9370	0.7168	0.9992	0.1370
MBOAT1	0.8909	1.1042	1.0689	0.9329	0.9992	0.0516
SLC17A1	1.0626	0.9954	1.0633	0.8757	0.9992	0.0442
HECW1	0.9050	1.2331	0.9575	0.9013	0.9992	0.0790
LOC728804	0.9726	1.1147	0.9871	0.9225	0.9992	0.0409
MIR1302-8	1.0174	0.9999	1.0078	0.9718	0.9992	0.0098
LOC650210	0.8870	1.0849	1.1240	0.9012	0.9992	0.0613
LOC645444	1.0598	0.9713	0.9503	1.0157	0.9993	0.0244
NDFIP1	0.8070	1.3493	1.0875	0.7533	0.9993	0.1378
LOC643014	0.9066	1.1199	1.0741	0.8966	0.9993	0.0572
C7orf53	0.9862	1.1053	0.9527	0.9529	0.9993	0.0362
BZRPL1	1.0067	1.1180	0.9449	0.9275	0.9993	0.0431
ZKSCAN5	0.9498	1.1755	1.0038	0.8681	0.9993	0.0650
EHD3	0.8945	1.1929	0.9402	0.9696	0.9993	0.0664
ADCY5	1.0272	1.0245	0.9713	0.9743	0.9993	0.0153
KIRREL2	0.9592	1.1530	0.9979	0.8871	0.9993	0.0561
SPANXE	0.9404	1.0876	1.0215	0.9478	0.9993	0.0347
LMAN1L	0.9400	1.1198	1.0650	0.8724	0.9993	0.0566
RPL12	0.8564	1.2982	1.0157	0.8270	0.9993	0.1079
LOC284422	0.9932	1.1390	0.9882	0.8769	0.9993	0.0538
LOC644419	0.9350	1.1927	0.9309	0.9387	0.9993	0.0645
LOC729597	0.9585	1.1199	1.0138	0.9052	0.9993	0.0459
MIR608	0.9198	1.0306	1.1500	0.8970	0.9993	0.0581
GAS2	0.9821	1.0787	1.0241	0.9125	0.9993	0.0351
DOCK8	1.0081	1.0401	1.0263	0.9229	0.9994	0.0263
LOC644764	0.9155	1.0749	1.0765	0.9305	0.9994	0.0442
PRLHR	0.9873	0.9654	1.0397	1.0050	0.9994	0.0157
RPS4Y2	0.9244	1.2515	1.1000	0.7216	0.9994	0.1142
LOC643381	0.9736	1.0293	0.9667	1.0279	0.9994	0.0169
ZNF780A	0.8854	1.1803	1.0387	0.8931	0.9994	0.0699
TNFRSF17	0.9951	1.0030	1.0558	0.9436	0.9994	0.0230

LOC441167	1.0340	1.0701	0.9773	0.9162	0.9994	0.0337
LOC645362	1.0011	1.1664	0.9584	0.8717	0.9994	0.0618
LOC255480	0.8827	1.0074	1.1215	0.9859	0.9994	0.0490
GPR64	0.9109	1.0911	1.0395	0.9560	0.9994	0.0406
LOC1001302	0.9204	1.1154	1.0758	0.8859	0.9994	0.0566
LOC642268	1.0356	1.0837	1.0235	0.8548	0.9994	0.0499
ZNF117	0.9509	1.0593	1.0796	0.9078	0.9994	0.0416
OR5AU1	1.0181	0.9679	1.0231	0.9886	0.9994	0.0130
LOC130678	0.9210	1.1443	0.9781	0.9543	0.9994	0.0497
TAC4	1.0582	1.0724	1.0206	0.8466	0.9994	0.0521
LOC729751	0.9554	1.1669	0.9731	0.9023	0.9994	0.0578
ARHGAP27	1.0251	1.0924	0.9716	0.9087	0.9994	0.0391
LOC1001909	0.9677	1.0860	1.0041	0.9400	0.9995	0.0317
SLC7A10	0.9183	1.1919	1.0452	0.8424	0.9995	0.0766
LOC121906	0.8819	1.1906	1.0024	0.9229	0.9995	0.0685
LOC388707	0.7566	1.4387	1.0838	0.7189	0.9995	0.1678
ZNF585A	1.0110	1.2063	0.9758	0.8047	0.9995	0.0824
LOC645151	0.8209	1.1540	1.1374	0.8857	0.9995	0.0855
HTR2C	0.9834	1.0707	1.0242	0.9197	0.9995	0.0320
SEMA4B	0.9888	1.2695	0.8877	0.8519	0.9995	0.0946
RAB3GAP2	1.0937	1.3445	0.8716	0.6882	0.9995	0.1418
ARSA	1.0043	1.0740	1.0325	0.8872	0.9995	0.0401
SNORD45A	0.8795	1.1701	0.9957	0.9528	0.9995	0.0617
LOC389834	0.8950	1.1351	1.0376	0.9304	0.9995	0.0544
ZNF546	0.8506	1.0775	1.1325	0.9375	0.9995	0.0644
FLYWCH1	0.8720	1.2216	1.0268	0.8777	0.9995	0.0822
C9orf93	1.0205	1.0791	1.0004	0.8980	0.9995	0.0377
CALM1	0.8360	1.3518	1.0375	0.7728	0.9995	0.1303
LOC644704	0.9273	1.0760	1.0398	0.9550	0.9995	0.0350
LOC729933	0.8906	1.1779	0.9787	0.9510	0.9995	0.0622
LOC642113	0.9534	1.0385	1.1128	0.8935	0.9995	0.0481
LOC727895	0.8880	1.1762	1.0313	0.9026	0.9996	0.0671
NPSR1	0.9137	1.1372	0.9987	0.9486	0.9996	0.0491
LOC642420	0.9758	1.0829	0.9611	0.9783	0.9996	0.0281
LOC1001322	1.0482	1.0093	1.0433	0.8974	0.9996	0.0351
SMPX	1.0384	1.0424	1.0500	0.8675	0.9996	0.0441
C22orf15	0.9317	1.1394	1.0136	0.9136	0.9996	0.0514
ZNF214	0.9393	1.1144	1.0499	0.8948	0.9996	0.0503
CTDSPL	0.8601	1.3697	1.0437	0.7248	0.9996	0.1396
FGB	0.9859	1.1806	0.9716	0.8601	0.9996	0.0666
HEXDC	0.9039	1.2664	1.0548	0.7733	0.9996	0.1059
LOC650677	0.9671	1.0765	1.0415	0.9133	0.9996	0.0367
MGC15885	0.8983	1.0160	1.1540	0.9302	0.9996	0.0571
SPATA9	0.9299	1.1033	1.0598	0.9054	0.9996	0.0484
AKAP5	1.0583	0.9914	1.0708	0.8780	0.9996	0.0441
HMGA2	0.9730	1.1477	1.0024	0.8754	0.9996	0.0563

NPB	0.9264	1.1252	1.1166	0.8302	0.9996	0.0727
MICB	0.9901	1.1212	0.9675	0.9196	0.9996	0.0431
MIR302B	1.0060	1.0116	1.0657	0.9151	0.9996	0.0312
LOC1001312	0.8603	1.2449	0.9298	0.9635	0.9996	0.0845
LOC730105	1.0676	1.0143	0.9671	0.9495	0.9996	0.0265
MGC40574	1.0101	0.9404	1.1030	0.9450	0.9996	0.0379
STX2	0.9014	1.2945	1.0079	0.7946	0.9996	0.1075
ZC3H11A	1.0659	1.0902	0.9582	0.8842	0.9996	0.0480
GJC3	0.8903	1.1744	1.0615	0.8723	0.9996	0.0722
LOC441711	0.8851	1.1565	1.0973	0.8597	0.9996	0.0747
LOC441876	0.9687	1.0653	0.9822	0.9824	0.9996	0.0221
LOC152667	0.9403	1.0903	1.0141	0.9539	0.9997	0.0342
CST6	0.9699	1.1046	0.9573	0.9669	0.9997	0.0351
TIAM2	0.9533	1.0999	1.0480	0.8975	0.9997	0.0456
LOC152225	0.9177	0.9488	1.1008	1.0314	0.9997	0.0414
GHRLOS	0.8671	1.0549	1.0903	0.9864	0.9997	0.0492
GPR6	0.9190	1.1446	1.0321	0.9030	0.9997	0.0562
LOC651728	0.9557	1.0017	1.1411	0.9002	0.9997	0.0515
LOC1001298	0.9429	1.1221	0.9711	0.9626	0.9997	0.0412
TFF2	0.9530	1.0371	1.1215	0.8870	0.9997	0.0509
IL5	0.9782	1.1336	1.0412	0.8458	0.9997	0.0604
CHX10	1.1090	0.9866	0.9305	0.9728	0.9997	0.0383
LYPD3	0.9690	1.2298	0.9746	0.8254	0.9997	0.0841
ZBTB46	0.9961	1.2221	1.0131	0.7676	0.9997	0.0929
LOC645689	1.0233	1.0707	1.0498	0.8551	0.9997	0.0492
FAM43B	0.9252	1.0776	1.0731	0.9230	0.9997	0.0437
LOC652838	0.9608	1.0900	1.0530	0.8951	0.9997	0.0442
LOC728816	0.9242	1.1460	1.0825	0.8462	0.9997	0.0692
XYLT1	0.9764	1.0536	1.0441	0.9249	0.9997	0.0303
LOC652427	0.9771	0.9102	1.1915	0.9201	0.9997	0.0656
ZNF266	1.0036	1.2083	1.0344	0.7527	0.9997	0.0939
PTENP1	0.8576	1.2958	0.8796	0.9660	0.9998	0.1014
LOC643409	0.9779	1.0018	1.1766	0.8428	0.9998	0.0685
LOC1001283	0.9081	1.0992	0.9811	1.0106	0.9998	0.0395
LOC1001287	0.9087	1.2149	1.0553	0.8203	0.9998	0.0865
LOC392196	0.9495	1.1634	1.0228	0.8633	0.9998	0.0635
ICAM5	0.9931	1.0018	1.0664	0.9378	0.9998	0.0263
MIR185	0.9031	0.9970	1.0023	1.0967	0.9998	0.0395
LOC387939	0.8932	1.1034	0.9943	1.0082	0.9998	0.0430
OR4F5	0.9803	1.0304	1.0067	0.9817	0.9998	0.0119
C15orf59	0.9389	1.0914	1.0572	0.9117	0.9998	0.0439
FAM74A4	1.0751	1.0419	0.9789	0.9033	0.9998	0.0378
LOC645655	0.9833	1.1249	0.9580	0.9331	0.9998	0.0429
LOC729461	0.8742	1.1244	1.0905	0.9103	0.9998	0.0629
TTTY16	1.0284	0.9917	1.1029	0.8763	0.9998	0.0472
LOC1001333	0.9752	1.1253	1.0150	0.8838	0.9998	0.0500

LOC645897	0.9150	1.0812	1.0285	0.9746	0.9998	0.0357
LOC1001330	0.9968	1.0489	1.0036	0.9500	0.9998	0.0202
OR4F15	0.8996	1.0911	1.1240	0.8846	0.9998	0.0626
RNASE11	0.9514	1.0356	0.9638	1.0485	0.9998	0.0246
CPNE8	0.9680	1.1181	1.0351	0.8782	0.9999	0.0509
TECTB	1.0349	1.0448	1.0406	0.8792	0.9999	0.0403
EBF4	0.9522	1.0757	1.0652	0.9065	0.9999	0.0418
LOC653200	1.0240	1.0268	1.0833	0.8653	0.9999	0.0469
PTCHD1	0.8681	1.1026	1.1285	0.9003	0.9999	0.0673
HPS4	0.9152	1.1380	1.0538	0.8926	0.9999	0.0582
WDR47	0.9213	1.2509	0.9408	0.8867	0.9999	0.0844
PIK3C2G	0.9296	1.2406	0.9734	0.8559	0.9999	0.0838
C13orf29	1.0358	1.0347	1.0429	0.8862	0.9999	0.0379
LOC643382	0.9392	1.0348	1.1278	0.8979	0.9999	0.0514
ACER2	0.8593	1.2820	1.0418	0.8165	0.9999	0.1060
ARHGAP10	0.9697	1.3317	0.9624	0.7359	0.9999	0.1232
C1orf53	0.8210	1.4317	1.0407	0.7064	0.9999	0.1598
LOC643389	0.8881	1.0714	1.0479	0.9923	0.9999	0.0408
FMO2	0.9862	1.0127	1.0503	0.9505	0.9999	0.0211
LOC643466	0.8481	1.3004	0.9717	0.8796	0.9999	0.1035
TMEM170B	0.9695	1.1961	1.0022	0.8320	0.9999	0.0751
LOC644327	1.0752	0.9698	1.0503	0.9045	0.9999	0.0390
LOC1001294	1.1078	1.0396	0.9681	0.8842	0.9999	0.0480
AMACR	0.9060	1.4504	0.9007	0.7426	0.9999	0.1549
PCDH19	0.9241	1.1040	1.0899	0.8817	1.0000	0.0568
LOC1001322	0.9300	1.1558	0.9480	0.9661	1.0000	0.0525
LOC440550	0.9542	1.0864	1.0149	0.9444	1.0000	0.0327
CRABP2	1.0213	1.0644	1.0973	0.8170	1.0000	0.0630
SKCG-1	0.9617	1.0294	1.0775	0.9313	1.0000	0.0330
LOC1001291	0.9119	1.0811	1.1124	0.8945	1.0000	0.0563
PABPC4L	1.0057	1.0557	0.9961	0.9423	1.0000	0.0232
BPTF	1.0145	1.0712	0.9742	0.9400	1.0000	0.0282
MIR371	0.9205	1.2245	0.9374	0.9175	1.0000	0.0750
PURA	0.9914	1.1130	1.1623	0.7333	1.0000	0.0959
DHRS9	0.9615	1.0963	1.0219	0.9204	1.0000	0.0383
TCEB2	0.8941	1.3724	1.0445	0.6890	1.0000	0.1439
PSG7	0.8892	1.1039	1.0805	0.9265	1.0000	0.0540
LOC1001281	1.0401	1.0349	0.9731	0.9520	1.0000	0.0221
ITLN1	0.9339	1.0606	1.1068	0.8988	1.0000	0.0497
LOC652030	0.9488	1.0602	1.0406	0.9506	1.0000	0.0294
LOC643721	0.8069	1.1140	1.0378	1.0414	1.0000	0.0667
SIGLEC14	1.0413	1.0132	1.0251	0.9205	1.0000	0.0271
LOC645693	0.9968	1.1132	1.1521	0.7380	1.0000	0.0934
NGFR	1.0060	1.0261	1.0666	0.9015	1.0000	0.0352
MIR153-2	1.0565	1.0356	0.9800	0.9280	1.0000	0.0289
PRAMEF1	0.8864	1.1045	1.0321	0.9772	1.0000	0.0460

LOC284801	1.0456	1.0107	0.9876	0.9563	1.0000	0.0188
LOC1001310	0.9258	1.1598	0.9947	0.9199	1.0001	0.0559
LOC646568	0.9145	1.0431	1.0580	0.9846	1.0001	0.0326
C9orf70	0.9882	1.1097	1.0206	0.8817	1.0001	0.0471
CRYBA4	1.0269	1.0705	0.9834	0.9194	1.0001	0.0322
HOXA4	0.9775	1.0888	1.0150	0.9190	1.0001	0.0356
RNF213	0.9563	1.1034	0.9777	0.9629	1.0001	0.0347
NECAB2	1.0150	1.1028	1.0008	0.8817	1.0001	0.0455
ATP5J2	0.8880	1.4575	0.9832	0.6717	1.0001	0.1658
LOC1001329	0.9623	1.0575	1.0335	0.9470	1.0001	0.0269
LOC648293	0.9501	1.0710	0.9979	0.9813	1.0001	0.0256
KIAA1906	0.9666	1.1237	1.0996	0.8104	1.0001	0.0720
CXorf23	0.9205	1.0819	1.0806	0.9174	1.0001	0.0469
TBC1D25	0.9679	1.1000	1.0366	0.8958	1.0001	0.0440
SPG7	0.9805	1.2888	0.9417	0.7894	1.0001	0.1047
LOC643496	0.9228	1.1477	1.0397	0.8902	1.0001	0.0588
MIR101-2	1.0499	1.0244	1.0463	0.8799	1.0001	0.0405
PDXP	0.7851	1.3560	1.0854	0.7739	1.0001	0.1388
LOC647551	1.1167	1.0320	1.0011	0.8506	1.0001	0.0555
LOC729346	0.8867	1.1999	1.0140	0.8999	1.0001	0.0725
EP400NL	0.9721	1.0714	1.0401	0.9168	1.0001	0.0346
LOC652255	1.0470	1.1236	0.9280	0.9019	1.0001	0.0519
SNIP1	0.8975	1.2900	1.0435	0.7696	1.0001	0.1116
LOC1001293	0.9596	1.0408	1.0326	0.9676	1.0001	0.0212
LOC651777	0.8729	1.2275	0.9716	0.9285	1.0001	0.0784
LOC1001326	0.9652	1.0694	1.0874	0.8786	1.0001	0.0487
UNQ9438	0.9917	1.0290	1.0757	0.9042	1.0001	0.0363
SLC7A5P1	0.9491	1.1480	1.0096	0.8939	1.0002	0.0546
OR5H14	0.8790	1.0125	1.2006	0.9086	1.0002	0.0727
LOC1001280	0.9785	1.0683	1.0638	0.8901	1.0002	0.0421
IL23R	0.9426	1.0987	1.0357	0.9237	1.0002	0.0410
C20orf200	0.9743	1.0431	1.0669	0.9165	1.0002	0.0341
LOC643831	0.9533	1.1322	1.0636	0.8517	1.0002	0.0617
OR4K14	0.9596	1.1840	0.9938	0.8633	1.0002	0.0672
ERCC-00051	0.9296	1.1629	0.9818	0.9264	1.0002	0.0557
SYDE2	1.0742	1.1427	0.9371	0.8467	1.0002	0.0667
LOC652551	1.0296	1.0547	1.0368	0.8797	1.0002	0.0405
KIAA1045	0.8888	1.0506	1.1067	0.9547	1.0002	0.0486
MTX3	0.8410	1.3776	0.9216	0.8605	1.0002	0.1270
LOC122038	0.9052	1.1558	1.1141	0.8257	1.0002	0.0799
LILRB2	0.9666	1.0985	1.0174	0.9183	1.0002	0.0385
LOC729355	0.8696	1.3424	0.9828	0.8061	1.0002	0.1198
LOC728931	1.0031	0.9575	1.1575	0.8828	1.0002	0.0580
F2RL3	1.0255	1.0749	0.9772	0.9234	1.0002	0.0325
TEKT4	0.9700	1.0460	1.0243	0.9606	1.0002	0.0207
LOC652080	0.9431	1.0149	1.0477	0.9952	1.0002	0.0219

LOC648274	1.0023	1.0051	1.0075	0.9860	1.0002	0.0049
SPDYE1	0.9171	1.1008	1.0267	0.9564	1.0002	0.0405
SLC30A8	0.9469	0.9942	1.1555	0.9043	1.0002	0.0549
TMEM179	0.9658	1.0454	1.0405	0.9493	1.0002	0.0249
LOC727947	0.9772	1.1134	1.0806	0.8298	1.0003	0.0638
SPPL2A	0.8469	1.2515	1.1857	0.7169	1.0003	0.1295
PAR4	0.9615	1.0863	1.0061	0.9472	1.0003	0.0313
LOC649801	0.8966	1.0257	1.2325	0.8463	1.0003	0.0861
KRT1	0.9475	1.0225	1.1021	0.9291	1.0003	0.0395
RPS29	0.9401	1.2099	1.0108	0.8403	1.0003	0.0781
LOC644045	0.9555	1.1623	0.9827	0.9007	1.0003	0.0566
KRTAP5-3	0.9691	1.0750	1.0341	0.9230	1.0003	0.0338
LOC157627	1.0115	1.0997	0.9940	0.8960	1.0003	0.0417
TBC1D14	0.9366	1.3741	0.9405	0.7500	1.0003	0.1323
GLIPR1	0.9216	1.1104	1.0291	0.9402	1.0003	0.0435
ARMCX1	0.9425	1.0711	1.0451	0.9426	1.0003	0.0338
GPR103	0.9412	1.0297	1.0854	0.9450	1.0003	0.0349
LOC1001313	1.0048	1.0753	0.9957	0.9255	1.0003	0.0306
C21orf94	0.9246	1.1091	1.0462	0.9215	1.0003	0.0465
LOC651465	1.0015	1.0786	0.9650	0.9562	1.0003	0.0279
KIAA1553	0.9745	1.1075	1.0249	0.8944	1.0003	0.0447
NBN	0.9455	1.2449	1.0063	0.8046	1.0003	0.0918
HAPLN4	0.9555	1.1337	1.0378	0.8744	1.0003	0.0556
LOC646057	0.9260	1.1163	1.0801	0.8790	1.0003	0.0578
DUB4	0.9656	1.0626	1.0545	0.9187	1.0004	0.0350
F13B	0.8947	1.0726	1.0699	0.9641	1.0004	0.0433
WASPIP	0.9376	1.0840	1.0772	0.9026	1.0004	0.0469
ZFYVE28	0.9453	1.1488	0.9837	0.9237	1.0004	0.0510
LPA	0.9293	1.0730	1.0542	0.9450	1.0004	0.0369
PYCARD	0.9203	1.0870	1.0924	0.9017	1.0004	0.0517
GABRQ	0.9386	1.0890	1.1238	0.8501	1.0004	0.0642
RNU5D	0.9285	1.0500	1.0720	0.9510	1.0004	0.0356
LOC393076	0.9136	1.0454	0.9836	1.0590	1.0004	0.0332
LOC1001340	1.0585	1.0942	0.9044	0.9444	1.0004	0.0452
LOC644592	1.0471	1.0526	0.9125	0.9894	1.0004	0.0326
LOC644303	0.9555	0.9930	1.1023	0.9508	1.0004	0.0353
CDC42BPA	0.9352	1.3122	1.0049	0.7493	1.0004	0.1171
LOC729758	1.0021	1.0848	0.9260	0.9888	1.0004	0.0327
RPS5	0.9353	1.1101	1.0633	0.8929	1.0004	0.0515
NCRNA0015	0.9993	1.0578	1.0225	0.9221	1.0004	0.0287
SLC8A1	0.9100	1.1039	1.0073	0.9805	1.0004	0.0401
CST8	0.9477	1.0766	1.0578	0.9196	1.0004	0.0392
ATOH7	0.9560	1.1081	1.0173	0.9202	1.0004	0.0411
LOC441177	0.9756	0.9526	1.1944	0.8791	1.0004	0.0679
C9orf68	0.9135	1.1195	1.0580	0.9108	1.0004	0.0525
TNFRSF13C	0.9983	1.0568	0.9750	0.9716	1.0004	0.0197

LOC728690	0.9943	1.0894	0.9956	0.9225	1.0004	0.0342
LOC650621	0.8782	1.2485	0.9067	0.9683	1.0004	0.0848
LOC652526	0.8327	1.2178	1.0842	0.8670	1.0004	0.0914
PRAMEF9	0.8938	1.1606	1.1242	0.8232	1.0004	0.0835
CHST12	0.8006	1.4887	0.8993	0.8133	1.0004	0.1642
LOC400236	0.9040	1.1605	1.1633	0.7740	1.0005	0.0969
LOC1001342	1.0010	1.1096	1.0044	0.8868	1.0005	0.0455
LOC1001329	0.9744	1.1186	1.0954	0.8134	1.0005	0.0699
SNORD47	1.0008	1.1487	0.9858	0.8666	1.0005	0.0578
FLJ44082	0.9699	0.9769	1.1217	0.9333	1.0005	0.0415
LOC644157	1.0445	0.9844	0.9927	0.9803	1.0005	0.0149
SNX30	1.0169	1.2868	1.0030	0.6951	1.0005	0.1209
HIGD1A	0.8350	1.3984	1.0964	0.6721	1.0005	0.1588
GRK1	0.9032	1.1026	0.9917	1.0044	1.0005	0.0408
NFATC1	0.9900	1.0859	1.0242	0.9019	1.0005	0.0384
C10orf18	1.0012	1.1380	0.9354	0.9274	1.0005	0.0487
LOC1001339	1.0051	1.0827	1.0213	0.8929	1.0005	0.0396
LOC1001300	0.9153	1.0632	1.2087	0.8147	1.0005	0.0861
SNORA75	0.9341	1.0330	1.1552	0.8796	1.0005	0.0606
C6orf191	0.8759	1.0941	1.1079	0.9241	1.0005	0.0589
LOC642439	0.8979	1.0770	1.0643	0.9629	1.0005	0.0427
MS4A12	0.9521	1.0423	1.0220	0.9856	1.0005	0.0199
FSBP	0.9916	1.0315	1.0728	0.9061	1.0005	0.0356
TRIM6	0.9781	1.0824	0.9387	1.0028	1.0005	0.0303
TAAR8	0.8881	1.1164	1.1304	0.8671	1.0005	0.0711
KRTAP19-6	0.8758	1.1027	1.0795	0.9440	1.0005	0.0543
LOC441081	0.9811	1.0698	1.0453	0.9059	1.0005	0.0367
ZNF321	0.9472	1.0771	1.0355	0.9422	1.0005	0.0333
LOC642433	0.9950	0.9420	1.1008	0.9643	1.0005	0.0351
ABI3BP	0.9089	1.0337	1.0103	1.0491	1.0005	0.0316
LOC652629	0.9452	1.0712	1.0665	0.9192	1.0005	0.0398
SNORA16B	0.9087	1.1356	1.0142	0.9435	1.0005	0.0501
LOC1001301	0.9756	1.0762	1.0353	0.9151	1.0005	0.0352
PRED57	0.9627	1.1108	1.0035	0.9252	1.0005	0.0401
SMAD5OS	0.9238	1.2620	1.0053	0.8111	1.0005	0.0958
SPANXN1	0.9140	1.1176	1.0485	0.9221	1.0005	0.0497
TTLL13	0.9856	1.0852	1.2046	0.7269	1.0006	0.1016
KRTAP5-10	0.9821	0.9995	1.1675	0.8531	1.0006	0.0645
OR4X1	0.9266	1.1115	1.0138	0.9503	1.0006	0.0413
ARPP-21	0.9133	1.1670	0.9922	0.9298	1.0006	0.0580
LOC1001344	1.0746	1.0154	1.0176	0.8947	1.0006	0.0379
LOC440356	0.9161	1.2837	1.0103	0.7922	1.0006	0.1044
LOC440361	0.9646	1.1123	1.0374	0.8880	1.0006	0.0481
LRRC56	0.8791	1.2189	1.0173	0.8870	1.0006	0.0794
RHD	0.9638	1.0723	1.0201	0.9461	1.0006	0.0286
LOC649470	0.9636	1.1248	0.9667	0.9472	1.0006	0.0416

LOC1001320	0.9761	1.1164	0.9646	0.9452	1.0006	0.0391
SCN5A	0.9835	1.0336	1.0501	0.9352	1.0006	0.0260
TOPORS	0.8802	1.2017	1.0852	0.8354	1.0006	0.0863
HDAC5	0.8828	1.1425	1.0584	0.9187	1.0006	0.0606
ACER1	0.9324	1.0647	1.0682	0.9372	1.0006	0.0380
PTGIS	0.9199	1.0653	1.0317	0.9855	1.0006	0.0315
BOAT	0.9443	1.1657	1.0097	0.8828	1.0006	0.0608
DZIP1L	0.8644	1.1947	1.0017	0.9419	1.0006	0.0705
CALM3	0.8758	1.4908	0.9392	0.6968	1.0006	0.1713
SLC6A3	1.0327	1.0608	1.0089	0.9001	1.0006	0.0351
ZNF493	0.9261	1.1247	1.0317	0.9202	1.0007	0.0486
FAM154A	0.9941	1.0825	1.0376	0.8884	1.0007	0.0415
ABCA10	0.9225	1.1807	1.0400	0.8596	1.0007	0.0707
OR4A15	0.9463	1.0091	1.0522	0.9951	1.0007	0.0218
NKTR	0.9988	1.3584	0.8908	0.7547	1.0007	0.1293
FAM13C1	0.9768	1.0857	1.0114	0.9288	1.0007	0.0330
LOC654109	0.9412	1.1357	0.9842	0.9416	1.0007	0.0461
LOC728955	0.8906	1.1395	0.9992	0.9734	1.0007	0.0517
PHACS	0.9605	1.0466	1.0442	0.9515	1.0007	0.0259
ST8SIA1	0.9799	1.0657	1.0473	0.9100	1.0007	0.0354
LOC728440	0.9645	1.1358	0.8942	1.0083	1.0007	0.0508
FLJ10246	0.9425	1.0649	1.0958	0.8995	1.0007	0.0473
OR2L2	1.0015	1.0858	0.9700	0.9455	1.0007	0.0306
TGDS	0.7778	1.4779	0.9727	0.7744	1.0007	0.1657
ELF1	0.8473	1.5180	1.0086	0.6290	1.0007	0.1892
LOC645630	0.9281	1.1483	1.1713	0.7552	1.0007	0.0985
LOC649897	1.0012	1.0951	1.0263	0.8803	1.0007	0.0448
ADH1B	0.9621	1.0879	0.9894	0.9635	1.0007	0.0297
MIR345	0.9853	1.1926	0.9943	0.8307	1.0007	0.0742
FAM10A6	1.0229	0.9916	1.1064	0.8821	1.0007	0.0464
LOC401180	0.9444	1.1403	1.0086	0.9097	1.0007	0.0508
UNC13B	1.1691	1.2993	0.9015	0.6331	1.0007	0.1479
OR56B1	0.9514	1.1263	0.9848	0.9405	1.0008	0.0429
LOC728100	0.9655	1.1196	1.0446	0.8732	1.0008	0.0529
C20orf135	0.9692	0.9831	1.0804	0.9703	1.0008	0.0268
ACSL5	0.9932	1.0783	1.0426	0.8890	1.0008	0.0411
HTR4	1.0013	1.1206	0.9550	0.9261	1.0008	0.0428
LOC644256	0.9544	1.0785	0.9878	0.9823	1.0008	0.0269
GLI3	0.9729	1.0734	0.9835	0.9734	1.0008	0.0243
LOC650659	0.9602	1.1671	1.0930	0.7829	1.0008	0.0843
PDE3A	0.8792	1.2098	0.9635	0.9507	1.0008	0.0721
LYG2	0.9523	1.1267	1.0832	0.8410	1.0008	0.0649
MYST4	0.8666	1.2435	1.0103	0.8828	1.0008	0.0870
LOC729193	0.9676	1.0142	1.0478	0.9737	1.0008	0.0188
LOC1001284	0.8214	1.1684	1.0814	0.9321	1.0008	0.0772
DPY19L2P4	0.9324	1.1285	0.9834	0.9590	1.0008	0.0438

SCYE1	0.8305	1.1067	1.1201	0.9459	1.0008	0.0692
TNS4	1.0068	1.0036	1.0568	0.9361	1.0008	0.0248
LOC1001299	0.9684	1.1877	1.0687	0.7784	1.0008	0.0866
LOC1001291	0.9322	1.1498	0.9962	0.9252	1.0008	0.0522
LOC347549	0.9261	1.1124	1.0064	0.9585	1.0009	0.0407
LOC1001314	0.9394	1.0486	1.1076	0.9078	1.0009	0.0466
CEP170L	0.9703	1.0769	1.0420	0.9142	1.0009	0.0364
MGC3032	0.8677	1.0927	1.1747	0.8683	1.0009	0.0785
RBM44	0.8798	1.1198	1.0511	0.9527	1.0009	0.0529
TKTL2	1.0116	1.0836	1.0030	0.9053	1.0009	0.0366
LOC644464	0.9168	1.1637	1.0751	0.8479	1.0009	0.0722
OR2B11	0.9517	1.1754	1.0368	0.8395	1.0009	0.0708
SYK	0.9858	1.0880	1.0172	0.9124	1.0009	0.0364
LOC649770	0.8759	1.1440	1.0967	0.8869	1.0009	0.0697
LOC1001337	0.8286	1.1986	1.0331	0.9432	1.0009	0.0781
ZNF750	0.9122	1.0160	1.0368	1.0385	1.0009	0.0300
LOC644431	0.9226	1.0556	1.0337	0.9917	1.0009	0.0293
C14orf78	0.9201	1.1430	0.9640	0.9764	1.0009	0.0489
ABCG8	0.9914	1.1327	0.9413	0.9380	1.0009	0.0456
MIR1295	0.8051	1.1997	1.0111	0.9877	1.0009	0.0807
GOLGA8F	0.9703	1.1724	0.9680	0.8930	1.0009	0.0599
LIMS2	0.9389	1.0708	0.9567	1.0373	1.0009	0.0316
LCE6A	0.9929	1.0757	1.0312	0.9038	1.0009	0.0365
LOC730474	0.9041	1.1177	0.9659	1.0161	1.0009	0.0452
KCNH1	0.9183	1.0792	1.1160	0.8902	1.0009	0.0566
NLRP14	1.0011	1.0771	1.0617	0.8639	1.0009	0.0485
FAM133A	0.9855	1.0817	1.0093	0.9272	1.0009	0.0320
LOC1001331	0.9503	1.0646	1.0557	0.9333	1.0010	0.0344
LOC728744	0.9768	1.2092	0.9691	0.8488	1.0010	0.0754
CBX4	0.9760	1.4257	0.9454	0.6568	1.0010	0.1588
LOC146517	0.9128	1.3234	0.9778	0.7900	1.0010	0.1143
RGS16	1.0267	1.0539	0.9777	0.9456	1.0010	0.0243
PLD2	0.9097	1.1090	0.9897	0.9955	1.0010	0.0410
HCG9	1.0056	1.0074	1.0973	0.8936	1.0010	0.0417
LOC653314	0.8238	1.2384	1.1292	0.8125	1.0010	0.1079
MIR376B	0.9657	1.0727	1.0197	0.9459	1.0010	0.0285
FBXO31	0.8345	1.3399	1.0064	0.8231	1.0010	0.1205
ZNF765	0.8959	1.1942	1.0103	0.9035	1.0010	0.0695
LOC1001280	0.9254	1.0918	1.1366	0.8501	1.0010	0.0678
ALX3	0.9445	1.0991	1.0303	0.9301	1.0010	0.0395
ROR2	0.9915	1.0154	1.0950	0.9022	1.0010	0.0397
LOC643433	0.8655	1.2755	1.1511	0.7120	1.0010	0.1290
ADAMTS5	0.9443	1.0264	1.0762	0.9571	1.0010	0.0309
SRCRB4D	0.9148	1.1623	1.0936	0.8334	1.0010	0.0764
ZNF311	0.9148	1.1278	1.0058	0.9557	1.0010	0.0462
MIR720	0.9131	1.1105	1.0663	0.9142	1.0010	0.0512

C16orf3	1.0010	1.0664	0.9239	1.0128	1.0010	0.0294
NOX1	1.0225	1.0617	1.0040	0.9159	1.0010	0.0308
KIAA1641	0.9427	1.1083	1.0215	0.9317	1.0010	0.0410
LOC648905	0.9124	1.0410	1.0975	0.9533	1.0011	0.0419
SORT1	0.9692	1.3894	0.8309	0.8148	1.0011	0.1340
LOC648732	0.9686	1.0704	1.1144	0.8509	1.0011	0.0586
LOC651578	0.9449	1.1570	1.0301	0.8724	1.0011	0.0612
TSPAN19	1.0546	1.0823	1.0085	0.8591	1.0011	0.0497
LOC283547	1.0039	1.0560	1.0038	0.9408	1.0011	0.0236
LOC728128	0.8878	1.0776	1.1568	0.8822	1.0011	0.0690
LOC1001283	0.9779	1.1889	0.9841	0.8534	1.0011	0.0695
TNFAIP8L2	1.0070	1.0116	1.0100	0.9759	1.0011	0.0085
FLJ46347	1.0396	1.0095	1.0586	0.8968	1.0011	0.0362
LOC643465	0.9323	1.0297	1.0059	1.0365	1.0011	0.0239
GOLGA6	0.9028	1.1042	1.0606	0.9369	1.0011	0.0483
LOC1001321	0.8526	1.2165	1.1188	0.8167	1.0011	0.0984
CLN8	0.8967	1.2583	0.9797	0.8699	1.0011	0.0888
CCDC38	0.9525	1.0856	1.1029	0.8636	1.0011	0.0568
LOC648879	0.9430	1.1324	0.9902	0.9389	1.0011	0.0453
DEGS1	0.9040	1.2671	1.1572	0.6764	1.0012	0.1323
RPL19	0.9174	1.0834	1.1325	0.8713	1.0012	0.0632
PIK3AP1	1.0057	1.0710	1.0302	0.8977	1.0012	0.0370
LRP12	0.9416	1.1404	1.0448	0.8779	1.0012	0.0578
LOC645978	0.9569	1.1610	0.9973	0.8895	1.0012	0.0577
LOC647279	0.9289	1.0553	1.1769	0.8436	1.0012	0.0730
SCN1A	0.9298	1.1452	0.9794	0.9505	1.0012	0.0491
LOC1001345	1.0172	1.0624	0.9290	0.9961	1.0012	0.0277
LOC644044	0.9542	1.2459	0.9845	0.8202	1.0012	0.0890
CD44	0.9573	1.1660	0.9879	0.8935	1.0012	0.0584
LOC650683	0.9141	1.0921	1.1150	0.8837	1.0012	0.0596
PCDHB14	1.0221	1.1017	1.0109	0.8701	1.0012	0.0481
FKSG44	0.9285	1.0418	1.0878	0.9467	1.0012	0.0381
CSMD1	0.9095	1.3461	0.9916	0.7577	1.0012	0.1248
TTTY17C	0.9167	1.0597	1.0536	0.9748	1.0012	0.0342
OR52A4	0.9520	0.9796	1.0231	1.0502	1.0012	0.0219
GGT6	1.0265	0.9770	0.9448	1.0566	1.0012	0.0250
LOC1001340	0.8233	1.0940	1.1625	0.9251	1.0012	0.0775
FAM27L	1.1804	0.9760	0.9613	0.8873	1.0012	0.0628
LOC643665	0.9062	1.1259	0.9619	1.0110	1.0012	0.0467
C18orf62	0.9335	1.0902	1.0380	0.9432	1.0012	0.0379
FAM75A5	1.0495	1.0454	0.9763	0.9338	1.0013	0.0281
SCN3A	0.9727	1.0986	1.0561	0.8776	1.0013	0.0488
FAM12A	0.9905	1.1152	0.9460	0.9533	1.0013	0.0392
UGCG	0.7803	1.2567	1.0363	0.9317	1.0013	0.1001
LOC650040	0.8529	1.0360	1.1695	0.9466	1.0013	0.0674
LOC1001290	0.9397	1.0323	1.0229	1.0102	1.0013	0.0210

LOC652068	0.8767	1.1939	1.1468	0.7877	1.0013	0.0998
RECQL5	0.9496	1.1023	1.0082	0.9450	1.0013	0.0366
ATPBD3	0.8509	1.3662	0.9028	0.8853	1.0013	0.1221
LOC728787	0.8957	1.2013	1.0927	0.8155	1.0013	0.0885
ATOH8	0.9483	1.0124	1.0958	0.9487	1.0013	0.0349
LOC1001295	0.9126	1.0974	1.0933	0.9018	1.0013	0.0544
LOC1001330	1.0330	0.9949	1.1112	0.8661	1.0013	0.0511
LOC729587	0.8974	1.2505	1.0149	0.8425	1.0013	0.0905
C15orf29	0.9241	1.0729	1.0742	0.9340	1.0013	0.0418
LOC646677	0.8607	1.0573	1.1267	0.9604	1.0013	0.0580
LOC197135	0.9683	1.1529	1.0601	0.8240	1.0013	0.0701
LOC648553	1.0121	1.0774	0.9860	0.9298	1.0013	0.0306
LRRC18	0.9393	1.0797	1.0952	0.8911	1.0013	0.0508
TUBGCP5	1.0367	1.1764	1.0546	0.7376	1.0013	0.0932
LOC651624	1.0662	1.0380	0.9491	0.9520	1.0013	0.0299
ZNF554	0.8914	1.0462	1.1116	0.9562	1.0013	0.0486
EPHB2	1.0417	1.1615	0.9694	0.8327	1.0013	0.0688
SF3B2	0.9946	1.3441	0.8798	0.7868	1.0013	0.1219
FBXO22	0.8945	1.4499	0.8867	0.7742	1.0013	0.1520
LOC1001288	0.9001	1.2213	1.0863	0.7977	1.0013	0.0946
LOC647531	0.9525	1.0697	1.1031	0.8801	1.0013	0.0517
KLK1	0.9506	1.1431	0.9669	0.9447	1.0013	0.0475
CREB1	0.8056	1.2740	1.1197	0.8061	1.0013	0.1172
CDKN2AIPNL	0.7555	1.1533	1.3105	0.7860	1.0014	0.1371
PRSS33	0.9929	1.0623	1.0449	0.9053	1.0014	0.0353
LOC644748	0.8910	1.1307	1.0240	0.9597	1.0014	0.0510
GABARAP	0.7732	1.1620	0.9899	1.0803	1.0014	0.0838
LOC730357	0.8942	1.2251	1.0520	0.8342	1.0014	0.0876
LOC1001312	1.0145	1.0259	1.0122	0.9528	1.0014	0.0165
GBP5	0.9857	1.1007	1.0739	0.8453	1.0014	0.0575
MRGPRX1	0.9031	1.0867	1.0449	0.9708	1.0014	0.0406
PARD3B	0.9237	1.1002	0.9950	0.9867	1.0014	0.0366
LOC1001331	0.9956	1.0290	1.0628	0.9181	1.0014	0.0310
LOC1001294	1.0255	1.0610	1.0211	0.8980	1.0014	0.0356
TBC1D3B	0.9462	1.1261	1.0774	0.8559	1.0014	0.0616
C2orf54	0.9169	1.1697	0.9380	0.9810	1.0014	0.0577
SASH3	0.9629	1.0434	0.9606	1.0388	1.0014	0.0229
LOC1001292	0.9649	1.1061	1.0299	0.9047	1.0014	0.0433
LOC644366	0.9996	1.0313	1.1085	0.8662	1.0014	0.0505
BDKRB1	0.8933	1.1459	1.0163	0.9502	1.0014	0.0543
HULC	0.9304	1.0686	0.9848	1.0218	1.0014	0.0292
LOC283104	0.9693	1.0498	1.1183	0.8683	1.0014	0.0538
ATXN7L1	0.9547	1.1153	1.0036	0.9322	1.0014	0.0408
PPIAL4G	0.9384	1.1314	1.0534	0.8825	1.0014	0.0561
PLEC1	0.9752	1.1041	1.0571	0.8693	1.0014	0.0515
LOC1001348	0.8284	1.1019	1.1392	0.9363	1.0014	0.0726

LOC401101	1.0016	1.1278	0.9545	0.9219	1.0015	0.0452
LOC1001328	0.9448	1.1321	1.1623	0.7666	1.0015	0.0919
C17orf100	0.9392	1.3735	0.8918	0.8014	1.0015	0.1273
LOC93463	0.8426	1.1708	1.0637	0.9287	1.0015	0.0725
TSSK3	0.9471	1.1877	0.9541	0.9169	1.0015	0.0626
ZNF804A	0.8789	1.0935	1.1740	0.8595	1.0015	0.0782
C8orf41	0.9546	1.1256	1.0349	0.8909	1.0015	0.0508
LOC440917	0.9670	1.0890	0.9914	0.9586	1.0015	0.0300
LOC339524	0.9856	1.1546	1.0269	0.8389	1.0015	0.0651
LOC644372	0.9230	1.1321	1.0083	0.9425	1.0015	0.0472
TUSC1	1.0252	1.1061	0.9773	0.8973	1.0015	0.0437
LOC344065	1.0233	1.1676	0.9689	0.8462	1.0015	0.0666
PVRIG	1.0047	1.0726	1.0580	0.8706	1.0015	0.0460
CEP110	0.9658	1.1049	0.9538	0.9815	1.0015	0.0349
LOC1001304	0.9089	1.1999	0.9011	0.9960	1.0015	0.0695
TDGF3	0.9745	1.0763	0.9329	1.0223	1.0015	0.0309
LOC203510	1.0893	1.0741	0.9926	0.8501	1.0015	0.0547
LOC650107	0.9503	1.0524	0.9681	1.0354	1.0015	0.0250
GPR174	0.8705	1.2156	0.9781	0.9418	1.0015	0.0748
LOC644604	0.9634	1.0750	1.0581	0.9095	1.0015	0.0393
DEFB105B	1.1244	1.0388	1.0083	0.8347	1.0015	0.0608
LOC1001345	0.8538	1.1799	1.3737	0.5988	1.0015	0.1718
LOC1001286	0.9179	1.1150	1.0548	0.9184	1.0015	0.0497
LOC730272	0.9350	0.9980	1.0182	1.0550	1.0015	0.0251
LOC654335	0.9875	0.9931	1.1137	0.9119	1.0016	0.0417
LOC642571	1.0174	1.0637	1.0220	0.9031	1.0016	0.0344
AKR7A3	0.8509	1.3177	1.0936	0.7441	1.0016	0.1283
7A5	0.9768	0.9827	1.0519	0.9950	1.0016	0.0172
OR6C74	0.8949	1.1115	1.1393	0.8607	1.0016	0.0720
LOC731052	0.9080	1.0910	1.0489	0.9584	1.0016	0.0417
PCDHB8	1.0180	1.1362	0.9462	0.9059	1.0016	0.0505
LOC653480	0.9872	1.2036	1.0320	0.7836	1.0016	0.0863
LOC644845	0.8494	1.0502	1.0986	1.0082	1.0016	0.0540
PDZD7	0.9323	1.1241	1.0464	0.9035	1.0016	0.0512
FAM134B	0.9415	1.3149	1.0661	0.6839	1.0016	0.1313
HAO1	0.9850	1.1098	1.0001	0.9116	1.0016	0.0409
MIR299	0.9646	1.1413	0.9514	0.9492	1.0016	0.0467
OR8D4	0.9811	1.0452	0.9902	0.9900	1.0016	0.0147
MGC39581	1.0501	1.0984	0.9885	0.8694	1.0016	0.0495
SHROOM4	0.7584	1.3756	1.1027	0.7697	1.0016	0.1481
TUBA8	1.1582	0.9210	1.0114	0.9159	1.0016	0.0566
LOC646631	0.9754	0.9723	1.1468	0.9120	1.0016	0.0505
LOC139363	0.8570	1.1094	1.1406	0.8995	1.0016	0.0721
LOC642370	1.0133	1.0670	1.0069	0.9194	1.0016	0.0305
LOC651197	1.0672	1.1003	0.9207	0.9183	1.0016	0.0479
ANGPTL2	1.0321	1.1516	0.9199	0.9030	1.0016	0.0576

LOC1001280	0.9421	1.1250	1.0050	0.9345	1.0016	0.0440
RAB35	0.7875	1.4883	1.0185	0.7123	1.0017	0.1748
SPINK5	0.9621	1.0371	1.0458	0.9617	1.0017	0.0230
PRIMA1	1.0496	1.0962	0.8949	0.9659	1.0017	0.0446
CXCL9	0.9614	1.1098	1.0163	0.9191	1.0017	0.0412
DNAJC2	0.9431	1.1154	1.0184	0.9298	1.0017	0.0426
C10orf141	1.0181	1.0948	0.9907	0.9031	1.0017	0.0396
FLJ43806	0.9632	1.1091	0.9646	0.9699	1.0017	0.0358
LOC388152	0.8360	1.0848	1.0888	0.9972	1.0017	0.0591
BEST3	0.9784	1.0958	0.9309	1.0017	1.0017	0.0347
LOC646675	0.7674	1.4099	1.0577	0.7718	1.0017	0.1521
LOC389053	0.8850	1.1886	1.0549	0.8783	1.0017	0.0745
LOC653286	0.9450	1.0534	1.0991	0.9094	1.0017	0.0446
C1orf26	1.0416	1.0840	0.9899	0.8914	1.0017	0.0415
LOC440839	0.8606	1.0470	1.1413	0.9579	1.0017	0.0601
LOC646772	0.9626	1.0977	1.0393	0.9072	1.0017	0.0419
NOX3	1.0717	1.0815	0.9625	0.8913	1.0017	0.0456
FKBP9	0.9702	1.0859	1.0629	0.8879	1.0017	0.0454
ERCC-00025	0.9363	1.0789	1.0583	0.9334	1.0017	0.0389
CD3D	0.9491	1.0731	1.0273	0.9575	1.0017	0.0295
LOC652769	0.9382	1.0959	1.0988	0.8741	1.0017	0.0567
LOC645013	0.9155	1.0649	1.0983	0.9283	1.0018	0.0467
CPN1	0.9337	1.1588	1.0109	0.9035	1.0018	0.0570
OR6V1	0.8819	1.0769	1.0679	0.9803	1.0018	0.0455
LOC1001316	0.9344	1.1020	1.0427	0.9280	1.0018	0.0425
HOXD12	0.9712	1.0316	1.1753	0.8290	1.0018	0.0718
ITM2C	0.9291	1.3307	0.9616	0.7857	1.0018	0.1161
CDC42	0.8260	1.3385	1.0748	0.7679	1.0018	0.1305
NCRNA0020(	0.9352	1.1670	1.0164	0.8885	1.0018	0.0611
LOC402342	0.8566	1.1847	1.2052	0.7607	1.0018	0.1133
TAC3	0.9336	1.0961	1.0179	0.9595	1.0018	0.0360
SLC47A1	0.9302	1.0587	0.9824	1.0359	1.0018	0.0287
FSCN2	0.9914	1.0723	1.0345	0.9090	1.0018	0.0351
LOC653579	0.9502	1.1436	1.0022	0.9112	1.0018	0.0508
SERPINB8	0.9203	1.0479	1.1159	0.9233	1.0018	0.0483
LOC654161	0.8263	1.2191	1.1482	0.8136	1.0018	0.1060
LOC728210	0.9362	1.0806	1.0020	0.9885	1.0018	0.0299
L3MBTL	0.9534	1.0371	1.0729	0.9439	1.0018	0.0316
GAGE10	0.8788	1.1290	1.0379	0.9617	1.0018	0.0534
IFITM4P	0.9611	1.0322	1.0350	0.9791	1.0018	0.0187
ASFMR1	0.9176	1.1396	0.9805	0.9697	1.0019	0.0479
LOC1001291	0.9399	1.1767	1.1372	0.7536	1.0019	0.0976
LOC652045	1.0344	1.0643	0.9842	0.9245	1.0019	0.0306
LOC400768	0.9516	1.2144	0.9630	0.8785	1.0019	0.0733
MAML1	0.9922	1.0941	0.9880	0.9332	1.0019	0.0335
CEACAM20	0.9468	1.0302	1.0419	0.9886	1.0019	0.0216

PSORS1C3	0.8737	1.0747	1.0822	0.9770	1.0019	0.0490
LOC648568	0.9687	1.0666	1.0236	0.9488	1.0019	0.0267
OR2AG1	0.9887	1.0337	1.0860	0.8992	1.0019	0.0396
RGSL1	0.9266	1.1052	1.0632	0.9126	1.0019	0.0484
C19orf41	0.9605	1.0249	1.0976	0.9246	1.0019	0.0381
NUDT17	0.9362	1.0901	1.0326	0.9488	1.0019	0.0364
FEV	1.0445	1.0626	1.0629	0.8377	1.0019	0.0549
UCA1	1.0173	1.0704	1.0237	0.8962	1.0019	0.0372
LOC650095	0.9581	1.1955	1.0277	0.8264	1.0019	0.0768
KRTAP10-3	0.9737	0.9807	1.0540	0.9993	1.0019	0.0182
SHCBP1	0.8402	1.3376	1.0317	0.7982	1.0019	0.1229
LOC1001301	0.9753	1.1559	1.0070	0.8695	1.0019	0.0591
LOC651876	0.9296	0.9885	1.1611	0.9285	1.0019	0.0549
LOC728573	1.0165	0.9796	1.0524	0.9592	1.0019	0.0206
ZC3H12A	0.9846	1.2054	0.9659	0.8518	1.0019	0.0739
DUX5	0.9552	1.0328	1.0780	0.9418	1.0019	0.0323
LOC1001306	0.9785	1.1353	1.0097	0.8843	1.0019	0.0518
KBTBD10	0.9671	1.1210	1.0488	0.8709	1.0019	0.0538
LOC653881	0.8792	1.1701	1.1192	0.8393	1.0019	0.0834
LOC1001307	0.9450	1.1279	0.9517	0.9831	1.0020	0.0428
ECSCR	1.0962	0.8771	1.0751	0.9594	1.0020	0.0513
C3orf55	0.9684	1.0564	1.0379	0.9451	1.0020	0.0268
IGF2	0.9887	1.0768	1.0192	0.9231	1.0020	0.0320
LOC652542	0.9221	1.1228	1.0074	0.9556	1.0020	0.0439
SERPINF1	0.9694	1.0333	1.1358	0.8694	1.0020	0.0559
LOC389607	1.0089	1.0391	1.0451	0.9147	1.0020	0.0301
HTR3D	1.0090	1.0210	1.0734	0.9045	1.0020	0.0354
LOC653841	0.9312	1.0903	0.9981	0.9883	1.0020	0.0329
LOC646094	1.0485	0.9441	1.0694	0.9459	1.0020	0.0332
PKHD1L1	0.9620	1.0889	1.0517	0.9054	1.0020	0.0418
LOC649578	1.0415	0.9632	1.1013	0.9019	1.0020	0.0437
LOC652278	0.8982	1.0034	1.0468	1.0595	1.0020	0.0366
LOC728248	0.8619	1.1594	1.0727	0.9140	1.0020	0.0690
PROP1	0.8033	1.1374	1.2048	0.8626	1.0020	0.0993
PCDHA4	0.8295	1.1114	1.0675	0.9996	1.0020	0.0619
LOC647339	0.9843	1.0702	1.0208	0.9327	1.0020	0.0290
LOC1001336	0.9372	1.1615	1.0040	0.9052	1.0020	0.0570
LOC1001317	0.9311	1.1497	1.0294	0.8978	1.0020	0.0566
ARHGAP24	0.9619	1.0908	1.0178	0.9376	1.0020	0.0340
PRDM10	0.8515	1.3625	0.9286	0.8655	1.0020	0.1213
LHFPL1	0.9110	1.3061	0.7575	1.0336	1.0020	0.1160
CDH13	0.9750	1.0892	1.0681	0.8759	1.0020	0.0488
LOC645769	0.8934	1.0824	1.0689	0.9636	1.0021	0.0449
LSAMP	0.9167	1.1945	1.0415	0.8555	1.0021	0.0749
CNPY4	0.9743	1.1922	0.9670	0.8749	1.0021	0.0673
AQP12A	0.8980	1.0961	1.0868	0.9274	1.0021	0.0520

ARHGAP26	0.9257	1.1486	1.0471	0.8869	1.0021	0.0596
CAPZA3	1.0118	1.1391	0.9544	0.9031	1.0021	0.0508
OR10W1	0.9620	1.1001	1.0560	0.8903	1.0021	0.0471
NOX5	1.0428	1.0726	1.0304	0.8626	1.0021	0.0473
SEMA5B	0.9853	1.0575	1.0479	0.9178	1.0021	0.0323
OLIG1	1.0540	1.0560	0.9862	0.9122	1.0021	0.0341
FUT5	0.9490	0.9796	1.1161	0.9638	1.0021	0.0385
GTF2H4	0.9337	1.3315	0.9888	0.7544	1.0021	0.1207
RXFP4	0.9420	1.0269	1.0384	1.0012	1.0021	0.0215
TUBE1	0.8947	1.0047	1.1773	0.9318	1.0021	0.0627
LOC347281	0.9105	1.1603	1.0225	0.9152	1.0021	0.0587
VPS13D	1.0019	1.0907	0.9778	0.9380	1.0021	0.0323
RWDD1	0.7304	1.3910	1.1187	0.7684	1.0021	0.1563
CLEC14A	0.9717	1.2645	0.9696	0.8028	1.0021	0.0960
LOC646918	1.0007	1.1190	1.1098	0.7791	1.0021	0.0791
MDS1	0.9014	1.1739	1.0119	0.9213	1.0021	0.0621
LOC1001318	1.0455	1.0460	1.0605	0.8567	1.0022	0.0486
LOC652544	0.9356	1.0257	1.1279	0.9194	1.0022	0.0480
PRSS2	0.8857	1.2690	1.0684	0.7855	1.0022	0.1065
LOC492303	0.8561	1.2298	1.0204	0.9024	1.0022	0.0834
LMCD1	0.9308	1.2065	0.9586	0.9128	1.0022	0.0688
SH2D4B	0.9331	1.0281	1.1029	0.9446	1.0022	0.0397
LOC727804	0.9807	1.0842	0.9650	0.9788	1.0022	0.0276
NFE2	0.9616	1.0378	1.0711	0.9383	1.0022	0.0313
SLC12A3	0.9933	1.0621	0.9793	0.9740	1.0022	0.0204
FOXD3	0.8940	1.0120	1.0802	1.0225	1.0022	0.0391
LOC442421	0.9733	1.1407	0.9346	0.9601	1.0022	0.0469
LOC643285	0.8567	1.1299	0.9636	1.0586	1.0022	0.0593
LOC645659	0.9509	1.2027	1.0816	0.7736	1.0022	0.0919
LOC1001308	0.8820	1.1664	1.0649	0.8955	1.0022	0.0687
SGPL1	0.9549	1.2099	1.0296	0.8143	1.0022	0.0824
BEYLA	0.9664	1.0923	0.9955	0.9545	1.0022	0.0312
FBXO15	0.8377	1.3642	1.0549	0.7519	1.0022	0.1365
SNORD116-2	0.7880	1.1117	1.0692	1.0399	1.0022	0.0729
EGOT	0.8941	1.1968	0.9086	1.0093	1.0022	0.0698
LOC150786	0.9600	1.0738	1.0535	0.9216	1.0022	0.0366
LOC648569	0.9456	1.1272	1.1042	0.8318	1.0022	0.0697
LOC1001302	1.0158	0.9750	1.0261	0.9919	1.0022	0.0115
KIAA1199	0.9779	1.0198	0.9972	1.0140	1.0022	0.0094
LOC645891	0.9748	1.0312	1.1200	0.8829	1.0022	0.0497
GS85	0.9600	1.1374	0.9688	0.9427	1.0022	0.0454
FGF5	0.9970	1.0884	1.0307	0.8929	1.0022	0.0410
IRX6	1.0614	0.9950	1.0696	0.8830	1.0022	0.0431
LOC642788	0.9658	1.0496	0.9752	1.0184	1.0023	0.0195
LOC1001334	0.8507	1.0955	1.1129	0.9499	1.0023	0.0623
LOC729500	0.8074	1.3150	1.1793	0.7073	1.0023	0.1455

LOC643308	1.0285	0.9881	1.1317	0.8608	1.0023	0.0560
SERPINB2	0.9776	1.0951	1.0159	0.9205	1.0023	0.0366
LOC1001291	0.8337	1.0703	1.1687	0.9364	1.0023	0.0736
MIR505	1.0556	1.0993	1.0065	0.8477	1.0023	0.0549
LOC340900	1.0036	1.0363	1.0341	0.9351	1.0023	0.0236
LOC652227	0.9522	1.0870	1.0813	0.8886	1.0023	0.0490
C11orf2	0.9131	1.4269	1.0180	0.6513	1.0023	0.1612
LOC653543	0.9909	1.0715	1.0516	0.8951	1.0023	0.0396
LOC647361	0.9735	1.1970	1.0551	0.7835	1.0023	0.0863
DKFZp564N2	0.9580	1.0683	1.0379	0.9451	1.0023	0.0301
SPTBN2	0.9914	1.1670	1.1000	0.7510	1.0023	0.0913
ETV2	1.0455	1.2384	0.9005	0.8249	1.0023	0.0910
LOC1001307	1.0016	1.0517	1.0523	0.9037	1.0023	0.0349
TOX	1.0561	0.9985	1.0537	0.9010	1.0023	0.0363
LOC642891	1.0683	1.1062	0.9727	0.8622	1.0023	0.0545
CGB5	0.9029	1.0871	1.0454	0.9740	1.0023	0.0406
USP27X	1.2246	0.9108	0.9718	0.9022	1.0023	0.0757
GIPC2	1.0162	1.0308	1.0059	0.9566	1.0024	0.0161
C6orf1	0.9468	1.2113	1.0177	0.8336	1.0024	0.0793
NEDD4	0.9319	1.1153	1.0724	0.8898	1.0024	0.0542
ZNF527	0.8274	1.1598	1.1892	0.8330	1.0024	0.0996
GPR132	0.9400	1.0609	1.0264	0.9822	1.0024	0.0263
PTPN23	1.0386	1.1144	0.9152	0.9413	1.0024	0.0458
EPYC	0.9344	1.1222	0.9999	0.9529	1.0024	0.0422
CD8A	0.8735	1.1197	1.0395	0.9768	1.0024	0.0520
LOC1001326	0.9427	1.1172	0.9965	0.9531	1.0024	0.0400
LOC643501	0.9765	1.0835	0.9987	0.9508	1.0024	0.0287
LOC643216	0.8979	1.1778	1.0390	0.8949	1.0024	0.0674
POU2AF1	0.9015	1.1274	1.0398	0.9409	1.0024	0.0508
LOC646796	0.9902	1.0989	0.9109	1.0096	1.0024	0.0386
LOC728989	0.9351	1.1440	0.9842	0.9464	1.0024	0.0483
LOC646100	0.9500	1.2231	0.9747	0.8618	1.0024	0.0775
MIR921	0.9408	1.0315	1.1158	0.9217	1.0024	0.0447
CAT	0.7546	1.3869	1.0177	0.8506	1.0024	0.1392
LOC653501	0.8826	1.1205	1.0523	0.9544	1.0024	0.0525
LOC133609	0.9477	1.0969	1.0636	0.9016	1.0024	0.0464
PAX2	0.9324	1.0518	1.0740	0.9516	1.0024	0.0354
LOC651648	1.0253	1.1036	0.9414	0.9394	1.0024	0.0392
GABPA	0.9536	1.2389	1.0274	0.7899	1.0025	0.0931
MAFK	0.9584	1.1364	0.9457	0.9693	1.0025	0.0449
LOC1001339	0.9362	1.0046	1.0879	0.9812	1.0025	0.0318
LOC652568	0.9994	1.1067	1.0149	0.8888	1.0025	0.0447
ESRRG	0.9463	1.0722	1.0745	0.9168	1.0025	0.0414
LOC653093	1.0492	1.0880	0.9479	0.9248	1.0025	0.0393
LOC1001280	0.9521	1.1382	1.0068	0.9128	1.0025	0.0492
CUL5	0.9164	1.2132	1.1580	0.7223	1.0025	0.1135

MUC17	0.9098	1.0806	1.0358	0.9837	1.0025	0.0367
HMSD	0.9667	1.1152	1.0457	0.8825	1.0025	0.0502
CYSLTR1	1.0067	1.0970	0.9478	0.9585	1.0025	0.0340
PAM	0.9927	1.1500	1.0396	0.8278	1.0025	0.0669
LOC643968	0.9554	1.0381	1.0428	0.9737	1.0025	0.0222
LOC1001339	0.9113	1.2045	0.9994	0.8948	1.0025	0.0711
TIGIT	0.8872	1.1158	0.9884	1.0186	1.0025	0.0471
LOC1001341	0.9061	1.0814	1.0933	0.9293	1.0025	0.0493
GRK7	0.9447	1.2364	0.9620	0.8671	1.0025	0.0806
PRDM8	0.9500	1.0922	1.0798	0.8883	1.0026	0.0499
LOC649112	0.9319	1.0519	1.0350	0.9914	1.0026	0.0268
ANXA4	0.9105	1.0437	1.1188	0.9372	1.0026	0.0483
PARD6G	1.0170	1.2389	0.9434	0.8110	1.0026	0.0896
CSF1	0.9634	1.1327	1.0161	0.8981	1.0026	0.0496
TMEM61	0.9635	1.1075	1.0603	0.8790	1.0026	0.0509
LOC651580	1.0416	0.9978	1.0641	0.9069	1.0026	0.0348
LOC729538	0.9274	1.0621	1.1000	0.9209	1.0026	0.0460
PMP22	0.9596	1.0626	1.0687	0.9195	1.0026	0.0373
MIR1302-4	0.9312	0.9963	1.1180	0.9649	1.0026	0.0407
LOC441212	1.0505	1.0422	1.0601	0.8575	1.0026	0.0485
DPPA5	0.9894	1.0968	1.0117	0.9125	1.0026	0.0379
LOC91316	1.0962	1.0360	1.0782	0.8001	1.0026	0.0687
LOC1001341	0.8989	1.2177	1.0413	0.8526	1.0026	0.0822
SNORD5	0.9034	1.2529	1.0481	0.8060	1.0026	0.0971
ZP2	0.9480	1.0679	1.0552	0.9394	1.0026	0.0342
LOC646783	0.9196	1.1166	1.0069	0.9674	1.0026	0.0420
LOXL4	0.9714	1.1099	1.0625	0.8667	1.0026	0.0537
MIR19B2	0.9257	1.0964	0.9608	1.0277	1.0026	0.0377
LOC643811	0.9651	1.0880	1.1087	0.8487	1.0026	0.0603
PHF12	0.9057	1.3408	1.0051	0.7590	1.0026	0.1235
LOC653889	1.0325	1.0890	1.0830	0.8062	1.0027	0.0667
LOC650546	0.9678	1.1003	1.0853	0.8573	1.0027	0.0568
ALOX15B	0.8940	1.1319	1.1005	0.8843	1.0027	0.0659
RGPD1	0.9404	1.0521	1.1153	0.9028	1.0027	0.0491
FAM162B	0.9618	1.0328	0.9823	1.0337	1.0027	0.0182
RAF1	0.9520	1.3181	0.9656	0.7751	1.0027	0.1137
LOC642756	0.9601	1.0711	1.0303	0.9491	1.0027	0.0291
OBFC1	0.9223	1.3438	1.0329	0.7117	1.0027	0.1318
LOC441154	0.8383	1.1770	1.3174	0.6781	1.0027	0.1477
LOC1001303	0.9411	1.0220	1.0955	0.9522	1.0027	0.0357
CLVS2	1.0628	0.9799	1.0841	0.8840	1.0027	0.0455
KIAA1257	0.9222	1.1308	1.0342	0.9237	1.0027	0.0501
LOC642231	0.9964	1.1749	0.9486	0.8909	1.0027	0.0613
GKN2	0.9574	1.0713	1.0888	0.8933	1.0027	0.0467
LOC649371	1.0063	1.0659	1.0349	0.9038	1.0027	0.0352
ASMT	0.9619	1.0452	1.0721	0.9318	1.0027	0.0333

C1orf130	0.9039	0.9910	1.2231	0.8930	1.0027	0.0767
GDF9	1.1022	1.1805	0.7913	0.9370	1.0027	0.0869
LOC643083	0.9206	1.0386	1.1138	0.9380	1.0027	0.0452
ARHGAP25	0.8746	1.0575	1.1007	0.9783	1.0028	0.0497
LOC441426	0.8688	1.1683	1.0691	0.9049	1.0028	0.0703
PDZK1	0.9233	1.1164	1.0582	0.9130	1.0028	0.0503
LOC646882	0.8913	1.1488	1.0526	0.9184	1.0028	0.0601
SH3GLB2	0.9770	1.3411	0.9341	0.7589	1.0028	0.1222
ZBTB2	1.0243	1.0607	0.9733	0.9528	1.0028	0.0245
WDR42C	0.9998	1.0429	1.0501	0.9183	1.0028	0.0303
SYNGAP1	0.9683	1.0501	1.0197	0.9731	1.0028	0.0196
LBXCOR1	1.0737	1.0525	0.9670	0.9179	1.0028	0.0365
LOC442427	0.9477	1.0519	1.0960	0.9156	1.0028	0.0426
PPIAP19	0.9962	1.0587	1.0218	0.9345	1.0028	0.0261
ZBP1	0.9350	1.1647	1.0008	0.9106	1.0028	0.0572
OPTC	0.9661	1.0829	1.0055	0.9567	1.0028	0.0287
LOC652054	1.0252	1.0277	1.0582	0.9002	1.0028	0.0350
IL17REL	0.8158	1.0857	1.0605	1.0493	1.0028	0.0628
LOC647417	0.9841	1.0953	1.0491	0.8828	1.0028	0.0460
TMEM148	0.9525	1.1132	0.9888	0.9568	1.0028	0.0377
LOC644953	0.9465	1.0818	1.0558	0.9272	1.0028	0.0387
WDR86	0.9340	1.0926	1.0786	0.9062	1.0029	0.0482
FLJ38723	1.0287	1.1218	0.9198	0.9412	1.0029	0.0461
TNFSF13	0.9290	1.1505	1.0779	0.8540	1.0029	0.0677
LOC653160	1.0772	1.1010	0.9472	0.8861	1.0029	0.0516
LOC651769	0.9098	1.0968	1.0203	0.9845	1.0029	0.0389
LOC1001325	0.8956	1.2074	1.0632	0.8454	1.0029	0.0826
C12orf28	0.9803	1.0496	0.9645	1.0171	1.0029	0.0191
GTF2H2D	1.0948	1.0658	1.0069	0.8441	1.0029	0.0560
LOC91661	1.0454	1.0438	1.0980	0.8243	1.0029	0.0609
SSH2	0.8891	1.3705	0.8581	0.8939	1.0029	0.1228
LOC643604	1.0538	1.1088	0.9583	0.8907	1.0029	0.0486
LOC1002165	0.8336	1.1722	1.0045	1.0012	1.0029	0.0691
RIBC2	0.9501	1.1276	0.9882	0.9456	1.0029	0.0427
LOC727834	0.9941	1.1078	1.0065	0.9032	1.0029	0.0419
ARMC2	0.8903	1.1446	1.0546	0.9222	1.0029	0.0591
MYO16	0.9612	1.0897	1.0878	0.8729	1.0029	0.0527
LOC1001314	0.9213	1.1562	1.0772	0.8569	1.0029	0.0689
PLAC9	0.9109	1.1994	0.9709	0.9304	1.0029	0.0667
C3orf70	0.9722	1.1300	1.1031	0.8063	1.0029	0.0740
RPS25	0.9182	1.0778	1.0532	0.9625	1.0029	0.0376
LOC729041	1.0184	1.1172	0.9912	0.8848	1.0029	0.0478
MIR625	1.0056	0.9897	1.0271	0.9893	1.0029	0.0089
DKFZP434P2	0.9483	1.2608	0.9137	0.8889	1.0029	0.0868
OR9A2	0.9697	1.1085	1.0261	0.9075	1.0029	0.0427
LOC649365	0.9758	1.0576	0.9988	0.9795	1.0029	0.0189

C10orf25	0.8894	1.1088	1.0411	0.9725	1.0029	0.0470
CRISPLD2	1.0266	1.3121	0.8426	0.8304	1.0029	0.1124
LOC650439	0.9146	1.1267	1.0265	0.9439	1.0029	0.0476
WASF1	0.9366	1.0844	1.0692	0.9217	1.0029	0.0428
LOC647904	0.9561	1.1807	1.0254	0.8496	1.0029	0.0694
OVOS	0.9936	1.1311	0.9841	0.9030	1.0030	0.0473
OR4K17	0.9679	1.0420	1.0996	0.9023	1.0030	0.0430
LOC646570	0.9946	1.0063	1.0745	0.9364	1.0030	0.0283
IFT80	0.9267	1.1910	0.9942	0.8999	1.0030	0.0658
LOC1001346	0.9715	1.1385	1.0047	0.8972	1.0030	0.0505
LOC641875	0.9256	1.2346	0.9828	0.8688	1.0030	0.0806
C22orf43	0.9543	1.1508	0.9546	0.9523	1.0030	0.0493
VENTX	0.9070	1.1871	1.0198	0.8980	1.0030	0.0673
AFP	0.9114	1.0948	1.1025	0.9033	1.0030	0.0553
RNF215	0.8681	1.2938	1.0954	0.7547	1.0030	0.1201
LOC643920	0.9998	1.1632	0.9936	0.8555	1.0030	0.0629
UBAC2	0.8728	1.3542	1.0499	0.7351	1.0030	0.1336
LOC648145	0.9912	1.0877	1.0654	0.8677	1.0030	0.0496
LOC1001286	0.9816	1.0590	1.0867	0.8847	1.0030	0.0453
SRRM4	0.9192	1.0610	1.0737	0.9581	1.0030	0.0381
FLJ43950	0.9496	1.1441	0.9822	0.9362	1.0030	0.0480
ARHGEF15	0.9805	1.1298	0.9987	0.9031	1.0030	0.0471
PHF2	0.9608	1.2499	0.8886	0.9128	1.0030	0.0837
MYH11	0.9528	1.1609	1.0167	0.8818	1.0030	0.0594
LOC654258	1.0287	1.0945	0.9932	0.8957	1.0030	0.0415
ADAMDEC1	0.9402	1.1021	1.0238	0.9461	1.0031	0.0381
LOC731275	0.8816	1.1547	1.0885	0.8874	1.0031	0.0698
LOC1001302	0.9483	1.0908	0.9355	1.0376	1.0031	0.0370
RBED1	0.9624	1.1379	1.1600	0.7520	1.0031	0.0946
C18orf26	0.9338	1.0574	1.1285	0.8925	1.0031	0.0545
LOC343296	0.9453	1.0577	1.0143	0.9950	1.0031	0.0233
SNAP25	0.9640	1.1105	1.0562	0.8817	1.0031	0.0505
HCLS1	0.8926	1.0923	1.1090	0.9184	1.0031	0.0567
LOC1001296	0.9715	1.2707	0.9489	0.8212	1.0031	0.0952
FAM46B	0.9579	1.1228	1.0322	0.8994	1.0031	0.0483
GALNT8	0.9410	0.9864	1.0449	1.0401	1.0031	0.0246
LOC645098	0.8922	1.0401	1.0933	0.9868	1.0031	0.0429
AQP8	0.9659	1.1123	1.0828	0.8514	1.0031	0.0596
DPP4	0.9761	1.0912	1.0779	0.8673	1.0031	0.0521
LOC389105	0.9975	1.1051	1.0442	0.8656	1.0031	0.0509
ZNF257	1.0213	1.0961	1.0352	0.8600	1.0031	0.0504
PADI4	0.9968	1.1379	0.9119	0.9659	1.0031	0.0482
LOC1001314	0.9056	1.0810	1.0787	0.9473	1.0031	0.0451
VPREB3	0.8977	1.1875	1.0582	0.8692	1.0031	0.0742
LOC647315	0.9857	1.0307	1.1206	0.8757	1.0031	0.0509
GPR144	0.9883	1.0781	1.0247	0.9216	1.0032	0.0329

MFSD11	0.7134	1.3487	1.1317	0.8189	1.0032	0.1454
C10orf96	0.9385	1.1662	1.0163	0.8916	1.0032	0.0601
RFT1	0.8935	1.1359	0.9912	0.9920	1.0032	0.0499
UGT1A9	1.0084	1.0833	1.0172	0.9038	1.0032	0.0371
DKFZp761E1!	0.9367	0.9698	1.1625	0.9437	1.0032	0.0536
CCDC17	1.0001	1.0125	1.0394	0.9607	1.0032	0.0164
MAGEA11	0.9811	1.0919	1.0311	0.9086	1.0032	0.0388
MTMR12	1.0440	1.4437	0.8408	0.6843	1.0032	0.1643
LOC731496	0.9672	0.9951	1.0681	0.9824	1.0032	0.0224
LOC1001347	0.8839	1.1070	1.1273	0.8946	1.0032	0.0659
CHRFAM7A	0.9495	1.1509	0.9747	0.9377	1.0032	0.0498
LOC340947	0.9686	1.1397	1.0110	0.8936	1.0032	0.0516
SLC22A24	0.8756	1.1691	1.0225	0.9457	1.0032	0.0629
IL31RA	0.8436	1.1350	1.1071	0.9271	1.0032	0.0704
SLC5A9	0.9991	0.9842	1.0348	0.9948	1.0032	0.0110
ERCC-00171	0.9046	1.1028	1.0163	0.9892	1.0032	0.0408
LOC643069	0.9001	1.0232	1.1605	0.9292	1.0032	0.0586
LOC1001289	0.9667	1.1575	1.0038	0.8850	1.0032	0.0571
FHL5	0.9644	1.0356	1.1278	0.8851	1.0032	0.0517
LOC647391	0.9648	1.1837	1.0129	0.8517	1.0032	0.0690
LOC729462	0.9455	1.0580	1.0319	0.9776	1.0032	0.0255
SP6	0.8826	1.2000	1.0813	0.8491	1.0033	0.0832
DOK6	0.9616	1.2682	1.0096	0.7736	1.0033	0.1019
LOC645961	0.9947	1.0920	0.9887	0.9377	1.0033	0.0322
LOC642009	0.9206	1.1597	1.0557	0.8771	1.0033	0.0645
HIST1H3J	0.9627	1.1000	1.0368	0.9135	1.0033	0.0410
GOLGA2L1	0.9122	1.2505	0.9627	0.8877	1.0033	0.0839
LOC1001697	1.0553	1.0145	1.0542	0.8891	1.0033	0.0392
LOC441316	1.0718	0.8748	1.0520	1.0145	1.0033	0.0444
LOC648999	1.0076	1.1348	0.9745	0.8962	1.0033	0.0497
LOC1001291	0.9154	1.1235	1.0731	0.9011	1.0033	0.0559
CENPN	1.0416	1.3225	0.9253	0.7238	1.0033	0.1250
MYO1E	1.1630	1.0665	0.8996	0.8841	1.0033	0.0674
LOC729677	0.9402	1.0825	1.1561	0.8344	1.0033	0.0719
OR52J3	0.9834	1.2058	1.0069	0.8171	1.0033	0.0796
SCARNA6	1.0031	1.1008	1.0885	0.8209	1.0033	0.0646
LOC1001347	0.9493	0.9496	1.0917	1.0225	1.0033	0.0341
LOC645927	0.9598	1.1705	1.0108	0.8722	1.0033	0.0626
LOC728307	0.9501	1.1423	0.9816	0.9394	1.0033	0.0472
MIR379	0.9420	1.1321	0.9424	0.9968	1.0033	0.0448
PAPD4	0.8040	1.4321	1.0733	0.7039	1.0033	0.1628
LOC1001334	0.9116	1.1516	1.0208	0.9294	1.0033	0.0549
LOC1001318	0.9058	1.0916	1.1280	0.8880	1.0033	0.0620
LOC730658	1.0087	1.1094	0.9719	0.9234	1.0034	0.0394
OTUD3	0.8994	1.1870	1.0241	0.9031	1.0034	0.0677
LOC730226	0.9435	1.2454	0.9461	0.8785	1.0034	0.0822

TTTY4	0.9260	1.1199	1.0100	0.9577	1.0034	0.0425
LOC642650	0.9767	1.0977	1.0133	0.9258	1.0034	0.0362
LOC1001889	0.9844	1.1550	0.9568	0.9174	1.0034	0.0524
MIR208B	1.0229	1.1099	1.0353	0.8455	1.0034	0.0560
LOC1001895	0.8604	1.0657	1.1445	0.9430	1.0034	0.0632
KCNA3	0.9759	1.0617	1.0196	0.9563	1.0034	0.0235
LOC651169	0.8991	1.0965	1.1310	0.8870	1.0034	0.0642
LOC1001322	0.9541	1.1134	0.9837	0.9625	1.0034	0.0372
CDKL5	0.9319	1.1196	1.0385	0.9237	1.0034	0.0467
SLC2A4	0.8661	1.0748	1.1503	0.9225	1.0034	0.0659
ADAMTS13	0.9341	1.1278	1.0116	0.9401	1.0034	0.0451
SGCA	0.9574	1.0994	0.9510	1.0059	1.0034	0.0342
LOC1001292	1.0627	1.0481	1.0592	0.8438	1.0034	0.0533
LOC728418	0.9176	1.1740	1.0428	0.8794	1.0034	0.0667
C19orf55	1.0428	1.0715	0.9696	0.9298	1.0034	0.0326
LOC644136	0.9256	1.0196	1.0632	1.0054	1.0034	0.0287
C4orf43	0.9179	1.1055	1.0888	0.9015	1.0034	0.0543
SNORD61	0.9051	1.1876	1.0420	0.8792	1.0035	0.0710
BANF2	1.0320	0.9529	1.0840	0.9449	1.0035	0.0333
FKSG83	1.0161	1.1032	1.0485	0.8462	1.0035	0.0554
LOC389033	0.9644	1.0696	1.0254	0.9545	1.0035	0.0271
LOC641814	0.9349	1.1034	1.0661	0.9095	1.0035	0.0478
LOC643808	0.9209	1.0764	1.0564	0.9602	1.0035	0.0374
C11orf44	0.9000	1.0888	1.0676	0.9576	1.0035	0.0449
DEFB105A	0.9662	1.0232	1.0105	1.0141	1.0035	0.0127
SLC22A25	0.9970	1.0757	1.0403	0.9009	1.0035	0.0378
LOC727844	1.0530	1.1223	0.9777	0.8610	1.0035	0.0559
LOC1001305	0.9884	1.0537	1.0228	0.9492	1.0035	0.0225
UPP1	0.8907	1.1951	1.0352	0.8931	1.0035	0.0723
MIR1298	0.8950	1.0630	1.0900	0.9660	1.0035	0.0449
ADAM22	0.9365	1.0738	1.0968	0.9070	1.0035	0.0478
LOC1001308	0.9826	1.0902	1.1023	0.8391	1.0035	0.0611
TACR1	1.0091	1.0762	1.0017	0.9272	1.0035	0.0305
LOC648729	1.0103	1.1313	1.0616	0.8110	1.0035	0.0688
LOC654106	0.9643	1.0086	1.1333	0.9080	1.0036	0.0479
LOC440352	0.7547	1.2628	1.0248	0.9719	1.0036	0.1043
TMEM130	0.9514	1.1464	0.9845	0.9319	1.0036	0.0488
MPPED2	0.9380	1.0368	1.0658	0.9737	1.0036	0.0291
FPR3	0.9336	1.2520	0.8504	0.9784	1.0036	0.0870
LOC1001332	1.0109	1.1128	1.0409	0.8497	1.0036	0.0556
LOC728448	0.9568	1.0554	1.0686	0.9335	1.0036	0.0342
CNTROB	0.8965	1.2396	0.9219	0.9563	1.0036	0.0796
LOC1001337	1.0333	1.1417	0.9258	0.9136	1.0036	0.0533
CYP2A7	0.8993	1.1458	0.9379	1.0313	1.0036	0.0549
LOC643729	0.9724	1.0769	1.0138	0.9512	1.0036	0.0277
DLGAP2	1.0099	1.0439	1.0667	0.8939	1.0036	0.0384

LOC648801	1.0146	1.1648	0.9478	0.8872	1.0036	0.0597
FUCA1	0.8385	1.3795	1.0981	0.6983	1.0036	0.1502
DEFA5	0.9535	1.1239	0.9818	0.9554	1.0036	0.0406
LOC641522	0.9290	1.1151	0.9821	0.9883	1.0036	0.0395
LOC441007	0.9529	1.2200	0.9651	0.8765	1.0036	0.0748
LOC651212	1.0006	1.1388	0.9749	0.9003	1.0036	0.0498
LOC729516	1.0607	1.0552	0.9721	0.9266	1.0037	0.0327
LOC1001335	0.9864	1.0588	1.0292	0.9403	1.0037	0.0258
SOX6	0.9305	1.1529	1.0164	0.9149	1.0037	0.0545
LOC1001339	0.8697	1.2109	0.9329	1.0013	1.0037	0.0741
BMP4	0.8609	1.1389	1.1050	0.9099	1.0037	0.0694
LOC642820	0.9932	1.0432	1.1460	0.8323	1.0037	0.0654
SHH	1.0049	1.1227	1.0624	0.8248	1.0037	0.0643
C6orf176	0.8786	1.1327	1.1075	0.8960	1.0037	0.0675
LOC653643	0.9300	0.9999	1.1223	0.9625	1.0037	0.0420
DLX5	0.8643	1.1488	1.0679	0.9337	1.0037	0.0642
NFATC2	1.0058	1.0525	0.9639	0.9926	1.0037	0.0185
LOC649466	0.9824	1.1273	0.9362	0.9690	1.0037	0.0423
BACH2	0.9991	1.0573	1.0228	0.9357	1.0037	0.0256
CXCL10	0.8178	1.2202	1.1017	0.8752	1.0037	0.0947
LOC641901	0.9494	1.1233	1.0248	0.9174	1.0037	0.0458
C6orf185	0.9560	1.0225	1.0099	1.0266	1.0037	0.0163
RXRG	0.9680	1.0089	1.0794	0.9588	1.0037	0.0275
LOC728882	0.9170	1.0403	1.1060	0.9516	1.0037	0.0428
OR2A25	1.0002	1.1514	0.9959	0.8675	1.0037	0.0580
LOC641944	0.9044	1.0804	1.1557	0.8745	1.0038	0.0680
LOC647832	0.9176	0.9897	1.0821	1.0256	1.0038	0.0344
LOC644074	0.9480	1.1614	0.9546	0.9510	1.0038	0.0526
LOC649073	1.0477	1.0987	1.0303	0.8384	1.0038	0.0570
LOC654206	0.9294	1.0571	1.0693	0.9593	1.0038	0.0349
MIR1274B	0.9287	1.1199	1.0387	0.9279	1.0038	0.0466
PLCXD1	1.1006	1.2942	0.8218	0.7986	1.0038	0.1187
KILLIN	1.0281	1.0471	1.0565	0.8834	1.0038	0.0406
LOC646215	0.9675	1.0243	1.1017	0.9217	1.0038	0.0388
HOXA1	0.9896	1.0458	1.0742	0.9056	1.0038	0.0371
C20orf194	0.9717	1.0922	1.0341	0.9172	1.0038	0.0379
BCMO1	0.9070	1.1359	1.0642	0.9082	1.0038	0.0575
GLIPR1L1	0.9411	1.1187	0.9909	0.9646	1.0038	0.0396
LOC728145	0.9721	1.0971	1.0566	0.8895	1.0038	0.0462
UTS2	0.8994	1.1455	1.0440	0.9265	1.0038	0.0567
NSUN7	0.9564	1.1289	1.0287	0.9014	1.0039	0.0492
LOC646372	0.8913	1.1650	0.9968	0.9624	1.0039	0.0580
KHDC1	0.9278	1.1264	1.1047	0.8565	1.0039	0.0663
LOC400927	0.9166	1.1131	0.8925	1.0933	1.0039	0.0577
LOC729722	1.0313	1.0577	0.9777	0.9488	1.0039	0.0248
LOC440013	0.9451	1.0977	1.0212	0.9516	1.0039	0.0357

LOC1001309	0.9112	1.1795	1.0940	0.8309	1.0039	0.0804
LOC1001304	0.9379	1.1345	1.1357	0.8076	1.0039	0.0803
ZNF821	0.9592	1.3463	0.9756	0.7346	1.0039	0.1267
EEF1A2	0.9305	1.2803	0.9319	0.8731	1.0039	0.0931
ZNF564	0.9377	1.2358	1.0675	0.7746	1.0039	0.0978
LOC646548	0.9563	1.0895	1.0454	0.9247	1.0039	0.0383
CDCP2	0.9803	0.9314	1.1315	0.9725	1.0040	0.0439
ZNF639	1.0473	0.9913	1.0481	0.9292	1.0040	0.0282
TMOD2	0.8852	1.1665	1.0303	0.9339	1.0040	0.0620
LOC1001300	0.9697	1.0783	1.0824	0.8855	1.0040	0.0473
UNC5C	1.0264	1.1453	1.0242	0.8200	1.0040	0.0675
C1orf103	0.9156	1.1370	1.0801	0.8832	1.0040	0.0618
LOC440080	0.9956	1.0542	1.0407	0.9255	1.0040	0.0290
CELP	1.0455	1.0815	1.0356	0.8534	1.0040	0.0511
ITGA7	0.9479	1.0563	1.0293	0.9826	1.0040	0.0241
IFNA2	0.9036	0.9752	1.1574	0.9799	1.0040	0.0540
LOC389705	1.0034	1.1686	0.9577	0.8863	1.0040	0.0599
KCNJ15	0.9060	1.0789	1.0892	0.9419	1.0040	0.0468
BRUNOL5	0.8424	1.1145	1.1235	0.9356	1.0040	0.0691
BLOC1S3	0.9699	1.0006	1.0493	0.9962	1.0040	0.0166
LOC1001280	1.0165	1.0951	1.0240	0.8805	1.0040	0.0448
CYLN2	0.9711	1.2108	0.9857	0.8485	1.0040	0.0755
IL4I1	1.0156	1.0491	0.9546	0.9968	1.0040	0.0197
PSPN	0.8919	1.0801	0.9753	1.0688	1.0040	0.0441
BTBD11	0.9851	1.0698	1.0229	0.9384	1.0040	0.0279
LOC643302	0.9791	1.0517	1.1247	0.8606	1.0040	0.0563
LOC644056	0.9619	1.1112	0.9740	0.9691	1.0041	0.0358
GLP2R	0.9844	1.0525	0.9807	0.9986	1.0041	0.0166
EFCAB1	0.9836	1.1174	0.9953	0.9199	1.0041	0.0412
LOC1001311	0.9654	1.1934	1.0074	0.8500	1.0041	0.0714
GLRA3	0.9271	1.1234	1.0256	0.9402	1.0041	0.0454
LOC653034	0.8510	1.1287	1.0258	1.0108	1.0041	0.0574
LOC648309	0.8820	1.0398	1.0959	0.9986	1.0041	0.0453
PTF1A	0.9653	1.1459	0.9874	0.9178	1.0041	0.0495
PDE7B	1.0200	1.0324	1.0888	0.8751	1.0041	0.0455
C19orf16	1.0048	1.0500	1.0123	0.9495	1.0041	0.0207
LOC1001343	1.0367	1.0211	1.0458	0.9129	1.0041	0.0308
C8orf16	1.0028	1.0591	1.0522	0.9025	1.0041	0.0361
AREG	0.9653	1.0774	1.0679	0.9060	1.0042	0.0414
TTTY3B	0.9620	1.1251	1.0197	0.9097	1.0042	0.0462
ZNF396	0.9711	1.0814	1.0434	0.9207	1.0042	0.0360
LOC646494	0.9389	1.1526	1.0455	0.8797	1.0042	0.0602
LOC649903	0.8652	1.0721	1.1521	0.9272	1.0042	0.0656
LOC644556	0.9779	1.1601	0.9409	0.9378	1.0042	0.0528
AS3MT	0.9705	1.0679	1.0863	0.8920	1.0042	0.0452
LHFP	0.8181	1.2870	0.9596	0.9520	1.0042	0.0997

MIR449A	0.8737	1.1186	1.0785	0.9459	1.0042	0.0570
C4orf44	0.9485	1.1117	1.0612	0.8954	1.0042	0.0498
LOC651141	0.9572	1.0230	1.0195	1.0171	1.0042	0.0157
LOC1001299	1.0054	1.0510	1.0701	0.8904	1.0042	0.0403
DEFB110	0.9360	1.1182	1.0659	0.8968	1.0042	0.0524
GOLGA2LY1	0.8065	1.2346	1.0222	0.9536	1.0042	0.0890
SLC8A3	0.9770	1.0632	1.0695	0.9072	1.0042	0.0386
BLOC1S2	0.9419	1.1365	1.0402	0.8983	1.0042	0.0532
TSGA10IP	0.9929	1.0691	1.0313	0.9236	1.0042	0.0310
STK32B	0.8988	1.1572	1.0744	0.8866	1.0042	0.0666
PKD1	1.0669	1.0946	0.9667	0.8889	1.0043	0.0473
LOC1001288	0.9684	1.0383	1.1090	0.9013	1.0043	0.0447
KCNT2	0.9881	1.0531	1.0755	0.9004	1.0043	0.0393
WFDC6	1.0199	1.0515	0.9570	0.9887	1.0043	0.0203
DEFB106B	0.9377	1.1001	0.9932	0.9861	1.0043	0.0342
PNOC	0.9258	1.2240	1.0328	0.8345	1.0043	0.0837
FXYD6	0.9245	1.1543	1.0088	0.9295	1.0043	0.0536
LOC442459	0.9224	1.2878	0.8963	0.9107	1.0043	0.0947
GSN	0.9358	1.1171	1.0284	0.9358	1.0043	0.0435
TMEM90B	1.0104	1.0155	1.0395	0.9517	1.0043	0.0187
LCE5A	0.9688	1.1338	1.0370	0.8776	1.0043	0.0541
ASB4	0.9688	1.1387	1.0305	0.8791	1.0043	0.0545
TBX20	0.9525	1.0873	1.0231	0.9543	1.0043	0.0322
AFM	0.9822	0.9649	1.1238	0.9463	1.0043	0.0405
LOC1001335	0.9099	1.1464	0.9715	0.9895	1.0043	0.0503
OR8U1	0.9477	1.0564	1.0868	0.9263	1.0043	0.0396
LOC651326	0.9841	1.0890	0.9586	0.9856	1.0043	0.0289
FLJ35282	0.9570	1.0839	0.8973	1.0791	1.0043	0.0462
MBNL2	0.9890	1.0463	0.9927	0.9894	1.0043	0.0140
WDR19	1.0229	1.4633	0.8312	0.6998	1.0043	0.1668
ANKRD34C	1.0195	1.0681	1.0113	0.9184	1.0043	0.0313
LOC1001292	1.0219	1.1450	1.0446	0.8059	1.0043	0.0714
TSIX	0.9085	1.2614	1.0483	0.7992	1.0044	0.0997
HPDL	0.9869	1.0665	1.0229	0.9411	1.0044	0.0266
LOC728532	0.8004	1.3482	1.1278	0.7411	1.0044	0.1427
CYP27B1	1.0294	0.9852	1.1306	0.8723	1.0044	0.0535
COL14A1	0.9361	1.0849	1.0323	0.9643	1.0044	0.0336
HDAC7A	0.9037	1.1128	1.0842	0.9168	1.0044	0.0547
FRMD4B	0.9649	1.1032	1.0160	0.9335	1.0044	0.0371
IGFBP4	0.9305	1.0113	1.1567	0.9191	1.0044	0.0548
LOC652635	1.0976	0.9962	1.1219	0.8019	1.0044	0.0728
MYOT	0.8824	1.1465	1.0279	0.9609	1.0044	0.0559
LOC728553	0.9705	1.1093	1.0768	0.8610	1.0044	0.0563
FGF19	0.8712	1.1173	0.9303	1.0990	1.0044	0.0612
SIPA1L3	0.8706	1.2575	0.9003	0.9893	1.0044	0.0880
LOC387770	0.9346	1.1068	1.1039	0.8724	1.0044	0.0596

LAX1	0.9406	0.9076	1.1815	0.9881	1.0044	0.0613
LOC649276	0.9338	1.1038	0.9522	1.0279	1.0044	0.0389
SULT1A2	0.9805	1.2009	1.0233	0.8131	1.0045	0.0797
HAPLN1	1.0736	1.0329	0.9716	0.9398	1.0045	0.0301
LOC651668	0.9402	1.1729	0.9407	0.9641	1.0045	0.0564
TCTN1	0.9725	1.4799	0.8647	0.7008	1.0045	0.1680
PDIA3	0.9495	1.1414	1.0231	0.9039	1.0045	0.0518
KCNJ14	0.9086	1.1340	1.0626	0.9127	1.0045	0.0561
PCDHB15	0.9395	1.0719	1.1258	0.8807	1.0045	0.0569
OR5AK2	1.0781	0.9941	1.0284	0.9173	1.0045	0.0338
HYALP1	0.9005	1.1343	1.0549	0.9283	1.0045	0.0548
HOXD3	0.8371	1.2038	0.9887	0.9883	1.0045	0.0754
CXorf22	0.9636	1.0632	1.0070	0.9842	1.0045	0.0215
LOC647654	0.9428	1.0365	1.0156	1.0230	1.0045	0.0210
LOC644943	0.8441	1.1486	1.0370	0.9882	1.0045	0.0631
DEFB131	0.9689	1.0253	1.0620	0.9617	1.0045	0.0239
TNXB	0.9765	1.1481	1.0176	0.8758	1.0045	0.0564
LOC648868	1.0025	1.0502	1.0512	0.9142	1.0045	0.0322
PGBD1	1.0593	1.0312	1.0455	0.8820	1.0045	0.0412
LOC1001318	1.0021	1.1156	0.9989	0.9015	1.0045	0.0438
CEACAM21	0.9662	1.2451	0.9453	0.8616	1.0045	0.0833
MUC20	0.9816	1.1194	1.0223	0.8948	1.0045	0.0466
NLRP5	0.9672	1.0961	1.1355	0.8193	1.0045	0.0714
OR4K1	1.0160	0.9918	1.0329	0.9774	1.0045	0.0124
KCNK7	1.0310	1.0268	1.0377	0.9226	1.0045	0.0274
GJD2	1.0118	1.1752	1.0275	0.8037	1.0045	0.0764
LOC1001283	0.9405	1.1658	1.0265	0.8854	1.0046	0.0611
ZNF611	0.9741	1.0677	1.0657	0.9107	1.0046	0.0381
LOC645811	0.9900	1.0473	1.0103	0.9706	1.0046	0.0164
HSFY1	1.0170	1.0127	0.9984	0.9902	1.0046	0.0062
IFNK	0.9164	1.0003	1.1543	0.9472	1.0046	0.0528
MIR25	0.9575	1.1387	1.0273	0.8946	1.0046	0.0523
LOC651771	0.9144	1.0963	1.1042	0.9034	1.0046	0.0553
SNX14	0.9984	1.1319	1.1009	0.7870	1.0046	0.0779
LOC388524	0.9634	1.1501	1.0300	0.8747	1.0046	0.0580
LOC642566	0.9103	1.0624	1.1102	0.9354	1.0046	0.0484
LOC389396	0.9705	1.0293	1.0441	0.9744	1.0046	0.0188
FLJ45079	0.9180	1.0931	1.0654	0.9417	1.0046	0.0438
ELP4	0.8217	1.4008	1.0057	0.7901	1.0046	0.1404
LOC653545	0.9548	1.0797	1.1751	0.8088	1.0046	0.0794
DUX4C	0.9974	0.9965	1.0062	1.0182	1.0046	0.0051
ERCC-00136	0.9697	1.0402	1.0418	0.9666	1.0046	0.0210
LOC642062	1.1055	0.9472	1.0859	0.8798	1.0046	0.0545
MIR1267	1.0477	1.0697	1.0019	0.8990	1.0046	0.0379
LOC1001313	0.9206	1.1536	0.9945	0.9496	1.0046	0.0520
LOC652468	0.9111	1.0598	1.0635	0.9840	1.0046	0.0362

LOC648205	0.9644	1.1112	1.0624	0.8805	1.0046	0.0514
LOC392467	0.8586	1.1691	1.0492	0.9415	1.0046	0.0673
GALNT2	0.9217	1.1332	1.1362	0.8273	1.0046	0.0776
ITLN2	0.9490	1.0838	0.9786	1.0071	1.0046	0.0289
LOC646089	0.9279	1.1290	0.9793	0.9823	1.0046	0.0433
LOC652593	1.0685	1.0759	1.0059	0.8681	1.0046	0.0481
NDUFAF1	0.8007	1.5923	0.9349	0.6906	1.0046	0.2022
LRIG2	0.9019	1.2741	0.9904	0.8522	1.0046	0.0943
LOC728400	0.9515	1.1841	0.9805	0.9025	1.0047	0.0619
C20orf196	1.0034	1.1555	0.9995	0.8602	1.0047	0.0603
GAL3ST3	0.8792	1.1596	1.0617	0.9182	1.0047	0.0649
FLRT1	1.0358	1.1802	0.9565	0.8462	1.0047	0.0702
C11orf37	0.9416	1.0770	1.1281	0.8720	1.0047	0.0592
LOC653786	0.9620	1.2577	0.9449	0.8542	1.0047	0.0876
C1orf166	0.9602	1.3159	0.9306	0.8120	1.0047	0.1086
MIR9-1	0.9905	1.0876	1.0554	0.8852	1.0047	0.0447
SRCAP	0.9874	1.1480	1.0524	0.8310	1.0047	0.0666
PECAM1	0.9869	1.2378	0.8411	0.9530	1.0047	0.0837
CCDC158	0.9280	1.1573	0.9589	0.9745	1.0047	0.0518
LOC648809	0.9365	1.0240	1.1199	0.9384	1.0047	0.0435
LY6G6D	1.0873	1.0683	0.9614	0.9018	1.0047	0.0441
LOC643547	0.9763	1.1014	1.0634	0.8778	1.0047	0.0497
LBX1	0.9723	1.0610	1.0383	0.9472	1.0047	0.0269
LOC648438	1.0237	1.0669	0.9699	0.9584	1.0047	0.0252
FER1L3	1.0445	1.1282	0.9771	0.8691	1.0047	0.0548
BAI3	0.9147	1.1653	1.0919	0.8471	1.0047	0.0743
LOC732157	0.8792	1.0747	1.1448	0.9202	1.0047	0.0629
LOC644652	0.9395	1.0446	1.0955	0.9394	1.0047	0.0391
C9orf11	1.0186	1.0137	1.0607	0.9260	1.0047	0.0283
NME4	0.8916	1.1417	1.0226	0.9632	1.0048	0.0529
CDK6	0.8927	1.2586	1.0183	0.8494	1.0048	0.0919
OR6C1	0.9982	0.9855	1.1011	0.9343	1.0048	0.0349
LOC728032	0.9854	0.9783	1.1307	0.9248	1.0048	0.0441
LOC644824	0.9425	0.9697	1.1093	0.9977	1.0048	0.0366
SERPING1	0.9644	1.0709	1.0268	0.9570	1.0048	0.0270
DKFZp434K1!	0.9393	1.1909	0.9730	0.9160	1.0048	0.0631
CARD14	0.9602	1.1086	1.0725	0.8780	1.0048	0.0528
LOC728295	1.0408	1.0187	1.1239	0.8358	1.0048	0.0607
GOLGA6B	0.9153	1.1357	1.0043	0.9639	1.0048	0.0473
LOC283412	0.7424	1.4399	1.0563	0.7806	1.0048	0.1610
FLJ90650	0.9748	1.0042	1.0286	1.0117	1.0048	0.0112
LOC652683	0.9666	1.1664	0.9795	0.9068	1.0048	0.0561
LOC283711	0.8740	0.9514	1.0907	1.1032	1.0048	0.0556
LOC647406	1.0199	1.0275	1.0946	0.8774	1.0048	0.0457
LOC647816	0.8907	1.1086	1.0757	0.9445	1.0049	0.0520
LOC643043	0.9215	1.0613	1.0685	0.9681	1.0049	0.0360

GNG3	0.9775	1.0838	1.0172	0.9410	1.0049	0.0306
LOC651481	0.9438	1.0970	1.0726	0.9061	1.0049	0.0471
GAST	0.9823	1.0457	0.9904	1.0011	1.0049	0.0142
POU3F1	1.0071	1.0965	1.0056	0.9103	1.0049	0.0380
C6orf27	0.9998	1.0450	1.1153	0.8595	1.0049	0.0540
LOC649292	0.9556	1.1120	1.0568	0.8952	1.0049	0.0488
LILRB3	0.7523	1.5714	1.0099	0.6860	1.0049	0.2013
RNF175	0.9312	1.1744	1.0293	0.8848	1.0049	0.0640
GREM1	0.9372	1.1699	1.0188	0.8937	1.0049	0.0608
TTC34	0.9425	1.0597	1.0560	0.9615	1.0049	0.0308
LOC1001346	1.0021	1.0891	0.9886	0.9400	1.0049	0.0311
C6orf163	0.9600	1.1700	1.0845	0.8053	1.0049	0.0793
LOC651397	0.9983	1.0879	1.0351	0.8985	1.0049	0.0400
TCP10L2	0.9894	0.9854	1.0446	1.0004	1.0050	0.0136
LOC651333	1.0042	1.0553	1.0951	0.8652	1.0050	0.0502
C16orf70	0.8610	1.4426	1.0029	0.7132	1.0050	0.1574
SNORA71C	0.8756	1.1559	1.0086	0.9798	1.0050	0.0578
LOC727764	1.1164	1.0141	1.0085	0.8809	1.0050	0.0482
LOC642867	0.9470	1.0560	1.0052	1.0117	1.0050	0.0224
LOC641727	0.9610	1.1155	1.1362	0.8071	1.0050	0.0767
LOC1001288	0.9381	1.1066	1.0302	0.9450	1.0050	0.0398
ATF7IP2	0.9561	1.0928	1.0093	0.9618	1.0050	0.0316
C14orf11	0.9738	1.1095	1.0542	0.8825	1.0050	0.0494
TACR2	0.8988	1.1296	1.0488	0.9428	1.0050	0.0521
MIR101-1	0.9647	1.1515	0.9960	0.9078	1.0050	0.0521
GPR137C	0.9014	1.0822	1.1042	0.9323	1.0050	0.0515
LACTB2	0.8236	1.5531	1.0722	0.5711	1.0050	0.2094
CREB3	1.1422	1.0779	0.9675	0.8324	1.0050	0.0679
C11orf9	0.9361	1.2155	1.0575	0.8110	1.0050	0.0863
SYN1	0.9714	1.1192	1.0248	0.9047	1.0050	0.0453
CLEC12A	0.9708	1.0800	1.0662	0.9031	1.0050	0.0418
LOC282992	0.9758	1.0504	1.1331	0.8608	1.0050	0.0578
LOC728056	0.8442	1.1628	1.0531	0.9600	1.0050	0.0677
RASD2	0.9414	1.1127	0.9728	0.9932	1.0050	0.0374
DISP2	0.9617	1.1817	1.0033	0.8735	1.0050	0.0648
CHD1L	0.9995	1.2174	1.1849	0.6184	1.0050	0.1375
NKX2-3	0.9626	1.1077	1.0759	0.8739	1.0050	0.0537
LOC650580	0.9523	1.0218	1.0342	1.0120	1.0051	0.0182
TBC1D3I	0.9991	1.0195	0.9969	1.0048	1.0051	0.0051
ADAMTSL5	0.9739	1.1282	0.9748	0.9434	1.0051	0.0417
C6	0.9811	0.9907	1.0753	0.9732	1.0051	0.0237
LOC648628	0.9372	1.1825	1.0005	0.9001	1.0051	0.0627
LOC1001324	0.9929	1.1187	1.0414	0.8672	1.0051	0.0527
LOC643274	1.0508	1.0935	0.9521	0.9239	1.0051	0.0401
XPNPEP2	1.0703	1.1320	0.9522	0.8658	1.0051	0.0595
CYHR1	1.0383	1.3121	0.8771	0.7928	1.0051	0.1143

LOC647309	0.9260	1.0939	1.0735	0.9269	1.0051	0.0456
LOC613037	0.8331	1.6993	0.7652	0.7227	1.0051	0.2325
TRIM50	0.9762	1.1021	1.0738	0.8682	1.0051	0.0530
STX1B	0.9032	0.9981	1.0917	1.0274	1.0051	0.0392
DKFZP779L1(	0.9420	1.0184	1.1040	0.9562	1.0051	0.0369
LOC729724	1.0543	0.9968	0.9936	0.9757	1.0051	0.0170
LOC730401	0.9616	1.0433	1.1002	0.9154	1.0051	0.0413
CCDC153	0.9205	1.0412	1.0759	0.9829	1.0051	0.0341
LOC649238	0.8843	1.1355	1.0664	0.9343	1.0051	0.0580
LOC644946	0.9600	1.0619	0.9949	1.0037	1.0051	0.0212
LOC649546	0.9326	1.0402	1.1145	0.9332	1.0051	0.0444
LOC1001342	0.9763	1.0522	1.0483	0.9439	1.0051	0.0269
KIAA1922	0.8123	1.1523	1.0680	0.9880	1.0051	0.0725
LOC652796	0.9632	1.0713	1.0927	0.8934	1.0052	0.0468
LOC1001299	1.0070	1.0737	1.0524	0.8875	1.0052	0.0416
C19orf62	0.8165	1.4620	0.9674	0.7748	1.0052	0.1578
LOC644336	0.9006	1.0428	1.1415	0.9358	1.0052	0.0546
NPFFR2	0.9261	1.1289	1.0034	0.9623	1.0052	0.0442
CCK	1.0173	1.1443	1.0159	0.8432	1.0052	0.0618
LOC1001344	0.9625	1.1503	0.9949	0.9130	1.0052	0.0512
CA2	0.9401	1.1212	1.0084	0.9511	1.0052	0.0415
PRDM6	0.9346	0.9423	1.1794	0.9645	1.0052	0.0584
LOC1001298	1.0212	1.0913	1.0397	0.8686	1.0052	0.0479
DRD2	0.9817	1.1035	0.9693	0.9664	1.0052	0.0329
LOC1001331	0.9891	1.2326	0.9057	0.8934	1.0052	0.0787
ERCC-00142	1.0027	1.0543	1.0290	0.9349	1.0052	0.0257
TNFRSF8	0.9507	1.1290	1.0630	0.8782	1.0052	0.0561
FLJ14816	0.9983	1.0104	1.0519	0.9603	1.0052	0.0189
FAM55A	0.9372	1.0659	0.9949	1.0229	1.0052	0.0270
LOC391427	0.9827	1.0798	1.0151	0.9434	1.0052	0.0288
LOC647648	1.0178	1.0634	1.0196	0.9202	1.0052	0.0303
POU6F2	0.9289	1.0530	1.0903	0.9487	1.0052	0.0393
RPS26P11	0.8668	1.3719	1.0803	0.7020	1.0052	0.1447
C1orf220	0.9408	1.0389	1.0849	0.9565	1.0053	0.0342
DEFB103B	0.9845	1.1606	1.0508	0.8251	1.0053	0.0702
LOC647546	1.0279	0.9906	1.0603	0.9422	1.0053	0.0254
LOC642751	0.8625	1.1274	1.2445	0.7867	1.0053	0.1081
DNCL2A	0.9307	1.1676	0.9876	0.9352	1.0053	0.0556
LOC391670	0.9472	1.1168	0.9843	0.9726	1.0053	0.0380
RRH	1.0637	1.0712	0.9601	0.9261	1.0053	0.0366
CEACAM3	1.0650	1.0708	0.9485	0.9368	1.0053	0.0362
PDZRN4	0.9664	1.1045	1.0550	0.8952	1.0053	0.0465
VMAC	0.9592	1.1970	0.9471	0.9179	1.0053	0.0645
BBOX1	0.9391	1.2010	1.0214	0.8598	1.0053	0.0731
LOC439950	0.7720	1.3082	0.9967	0.9443	1.0053	0.1118
LOC646401	0.9507	1.0961	1.1014	0.8731	1.0053	0.0562

LOC647031	0.9608	1.0836	1.0775	0.8994	1.0053	0.0452
PCDH17	0.8980	1.2255	1.0632	0.8345	1.0053	0.0878
C9orf84	0.9628	1.0723	1.0526	0.9336	1.0053	0.0337
COX6BP1	0.9262	1.0842	1.1202	0.8907	1.0053	0.0569
LOC652798	1.0166	1.1624	1.0436	0.7987	1.0053	0.0758
DDX60	1.0731	1.1620	0.9599	0.8264	1.0053	0.0726
LOC652147	1.0210	1.0548	1.1503	0.7953	1.0053	0.0752
LOC1001340	0.9034	1.0786	1.0466	0.9928	1.0053	0.0383
LOC389217	1.0815	0.9753	0.9881	0.9765	1.0054	0.0255
LOC730479	0.9203	1.0664	1.1420	0.8928	1.0054	0.0594
LOC1001299	1.0234	1.0768	1.0091	0.9123	1.0054	0.0343
RAG2	0.9596	1.0098	1.0576	0.9945	1.0054	0.0203
BAZ2A	0.9047	1.2298	0.9377	0.9495	1.0054	0.0754
FCN2	1.0115	1.0226	0.9676	1.0200	1.0054	0.0128
LOC729034	1.0505	1.1218	1.0428	0.8065	1.0054	0.0686
CENPC1	0.9206	1.1135	1.0471	0.9405	1.0054	0.0455
MIR542	0.9128	1.1974	0.9800	0.9314	1.0054	0.0655
FAM83B	0.9220	1.0639	1.0990	0.9369	1.0054	0.0446
LOC646888	1.0628	1.0615	1.0198	0.8777	1.0054	0.0437
KCNS2	1.0086	1.0653	0.9507	0.9972	1.0055	0.0235
ENPP7	0.9123	1.1274	1.0034	0.9786	1.0055	0.0450
TRPV3	0.9660	1.0436	1.1043	0.9079	1.0055	0.0431
NOBOX	0.9653	1.0161	1.0471	0.9934	1.0055	0.0174
LOC646130	1.0252	1.0743	1.0224	0.9000	1.0055	0.0371
SNHG6	0.8153	1.2746	1.1299	0.8021	1.0055	0.1174
EGR3	0.9611	1.1286	1.0088	0.9235	1.0055	0.0446
DUS1L	1.0397	1.2064	0.9977	0.7782	1.0055	0.0882
PEG3AS	0.9008	0.9809	1.2798	0.8605	1.0055	0.0948
MIR611	0.8622	1.1570	0.9437	1.0590	1.0055	0.0647
PIK3R6	0.9012	1.1902	1.0042	0.9265	1.0055	0.0653
LOC646719	1.0147	1.1143	0.9744	0.9186	1.0055	0.0413
LOC648795	1.0720	1.0539	1.0263	0.8699	1.0055	0.0462
SIRPG	0.9153	1.1107	1.0638	0.9324	1.0055	0.0483
LOC645494	0.8763	1.1732	1.0445	0.9282	1.0055	0.0660
TMEM81	0.9106	1.1726	1.0299	0.9091	1.0055	0.0625
LOC283999	0.8551	1.1014	1.1443	0.9213	1.0055	0.0696
PIRT	0.9549	1.0480	1.0651	0.9542	1.0055	0.0297
CD79B	0.9544	1.1256	1.0147	0.9275	1.0056	0.0440
GPR26	0.9626	1.1031	1.0514	0.9051	1.0056	0.0443
MIR450A1	0.9795	1.2199	0.8519	0.9709	1.0056	0.0772
HORMAD2	0.8898	1.2050	1.0450	0.8826	1.0056	0.0763
TTC28	0.9547	1.0695	1.0748	0.9233	1.0056	0.0390
LOC1001303	0.9022	1.2047	1.0847	0.8307	1.0056	0.0852
RGS13	0.9119	1.1099	1.0960	0.9046	1.0056	0.0563
OR2C1	1.0459	0.9430	1.0940	0.9395	1.0056	0.0384
LOC374491	0.9079	1.1473	1.0153	0.9519	1.0056	0.0521

LOC653620	1.0841	1.0348	0.9590	0.9445	1.0056	0.0328
IFIT1L	0.9725	1.0559	1.0276	0.9665	1.0056	0.0217
HIF3A	0.9394	1.1079	1.0489	0.9262	1.0056	0.0438
VSTM1	1.0417	1.0623	0.9374	0.9809	1.0056	0.0285
PEX14	0.9794	1.2766	0.9248	0.8417	1.0056	0.0947
PKIA	0.9288	1.0653	1.0568	0.9714	1.0056	0.0332
LOC338588	0.9835	1.1182	1.1001	0.8206	1.0056	0.0685
LOC286528	0.9654	1.1701	1.0923	0.7946	1.0056	0.0820
FNDC7	0.9130	1.1737	0.9852	0.9506	1.0056	0.0579
LOC729526	0.9392	1.0512	1.0589	0.9732	1.0056	0.0294
LOC641848	0.8640	1.0985	1.3069	0.7530	1.0056	0.1236
LOC1001334	1.0879	1.1169	0.9617	0.8560	1.0056	0.0602
TMEM16D	1.0344	1.0534	1.1222	0.8126	1.0056	0.0671
LOC1001329	0.9655	1.0761	1.0704	0.9105	1.0056	0.0407
HLXB9	0.9690	1.1389	0.8961	1.0186	1.0056	0.0510
NTNG1	0.9872	1.0643	1.0480	0.9232	1.0057	0.0321
LOC644637	0.9275	1.1788	0.9033	1.0130	1.0057	0.0623
LOC642212	0.9180	1.2195	1.0024	0.8829	1.0057	0.0755
LOC652553	1.0107	0.9927	1.1078	0.9115	1.0057	0.0403
KRT5	0.9268	1.1562	1.0495	0.8903	1.0057	0.0606
LOC653867	0.9151	1.1879	0.9678	0.9520	1.0057	0.0617
FLJ35801	0.8208	1.4475	1.0111	0.7433	1.0057	0.1577
LOC440934	0.9950	1.0848	1.0493	0.8937	1.0057	0.0416
PITX3	0.8203	1.1174	1.0767	1.0084	1.0057	0.0658
NLRP10	0.9550	1.0945	1.0468	0.9265	1.0057	0.0392
LOC730236	0.9136	1.1306	1.0204	0.9582	1.0057	0.0470
VSTM2A	0.9951	1.1281	1.0857	0.8140	1.0057	0.0697
LOC1001317	0.9617	1.1051	1.0236	0.9326	1.0057	0.0382
FAM70B	0.9444	1.1370	1.0004	0.9411	1.0057	0.0458
LOC441114	0.9049	1.0519	1.1289	0.9372	1.0057	0.0518
C4B	0.9478	1.0618	1.1181	0.8952	1.0058	0.0511
PNPLA3	0.8922	1.0844	1.0923	0.9541	1.0058	0.0493
LOC641931	0.8491	1.3133	0.9230	0.9377	1.0058	0.1043
LOC339809	0.9648	1.1054	1.0123	0.9406	1.0058	0.0364
FLJ23172	0.9702	1.0826	1.0474	0.9229	1.0058	0.0362
LOC645201	0.9144	1.1710	0.9990	0.9388	1.0058	0.0579
ENOX2	1.0021	1.1166	0.9892	0.9152	1.0058	0.0416
LOC1001319	0.8730	1.0884	1.0694	0.9925	1.0058	0.0489
LOC1001328	0.9553	1.1151	1.0323	0.9205	1.0058	0.0433
KLHDC8A	0.9348	1.1795	0.9306	0.9785	1.0058	0.0589
ATP13A4	0.9903	1.1342	0.9628	0.9359	1.0058	0.0442
LOC643965	0.9473	1.0522	1.0852	0.9385	1.0058	0.0370
TCAP	0.9234	1.0628	1.0861	0.9510	1.0058	0.0403
MYH14	0.8678	1.1982	1.1103	0.8471	1.0058	0.0876
LOC441208	1.0138	1.1123	0.9662	0.9310	1.0058	0.0393
PRAMEF13	1.0057	1.1036	0.9924	0.9216	1.0058	0.0375

LOC1001328	0.8408	1.0821	1.1797	0.9206	1.0058	0.0767
LOC1001285	0.9366	1.0910	1.0724	0.9234	1.0058	0.0440
CRISP1	1.0082	1.0649	1.0081	0.9421	1.0058	0.0251
CHD6	0.8225	1.3639	1.0202	0.8167	1.0058	0.1284
LOC730313	0.7349	1.1471	1.3232	0.8181	1.0058	0.1382
IL27	0.9875	1.0424	1.0049	0.9885	1.0059	0.0128
MAP1LC3C	1.0263	1.2384	0.8873	0.8714	1.0059	0.0850
LOC649305	0.8604	1.0915	1.0206	1.0510	1.0059	0.0506
SUDS3	0.9355	1.1735	0.9950	0.9194	1.0059	0.0582
LYZL2	0.9093	1.0984	1.0653	0.9504	1.0059	0.0452
CYP4A22	1.0214	1.0280	1.0636	0.9104	1.0059	0.0331
MGC52282	1.0350	1.0657	1.0456	0.8771	1.0059	0.0434
HIPK4	0.9590	1.0732	1.0148	0.9765	1.0059	0.0253
DRP2	0.9794	0.9926	1.0917	0.9599	1.0059	0.0294
FGD2	0.9207	1.1231	1.0860	0.8938	1.0059	0.0577
PDYN	0.9417	1.2154	1.0500	0.8165	1.0059	0.0846
SNORA60	0.9423	1.0509	1.1319	0.8985	1.0059	0.0528
LOC1001303	1.0002	1.0562	1.0202	0.9471	1.0059	0.0228
SLC25A43	1.0050	1.0738	1.0257	0.9192	1.0059	0.0323
LOC255025	0.9093	1.2085	0.8919	1.0141	1.0059	0.0727
SMURF1	0.9251	1.2144	0.9784	0.9059	1.0059	0.0712
GIGYF1	1.0070	1.1440	1.0170	0.8557	1.0059	0.0590
KCNN1	0.9862	1.0611	0.9684	1.0080	1.0059	0.0201
CCDC29	1.1159	1.0837	0.9474	0.8767	1.0059	0.0565
LOC732429	0.9457	1.0842	1.0295	0.9643	1.0059	0.0317
SCUBE3	0.9596	1.1343	1.0181	0.9118	1.0059	0.0480
LOC644580	0.7922	1.0735	1.0838	1.0743	1.0060	0.0713
DEFB114	0.9670	1.0904	1.0613	0.9051	1.0060	0.0427
ATP6V1H	0.8778	1.3484	1.0597	0.7380	1.0060	0.1318
SLC6A7	0.9498	1.1224	1.0045	0.9472	1.0060	0.0410
LOC644384	0.9148	1.0641	1.0685	0.9765	1.0060	0.0370
LOC340508	0.9452	1.0877	0.9961	0.9948	1.0060	0.0297
KRT6A	0.9929	1.1033	1.0110	0.9167	1.0060	0.0383
S1PR2	0.9668	1.0332	1.0247	0.9991	1.0060	0.0149
KCNMB4	0.9711	1.1388	0.9932	0.9208	1.0060	0.0468
LOC652602	1.0966	1.0940	0.9980	0.8352	1.0060	0.0614
LOC284964	0.9629	1.1036	1.0669	0.8905	1.0060	0.0487
CCNA1	0.9274	1.2011	0.9553	0.9402	1.0060	0.0653
LOC644753	1.0565	1.0367	1.0157	0.9151	1.0060	0.0314
LOC1001315	0.9216	1.0968	1.0203	0.9853	1.0060	0.0365
YBX2	0.9679	1.0442	1.0302	0.9817	1.0060	0.0184
ITFG2	0.9036	1.3074	1.0835	0.7296	1.0060	0.1237
CHSY3	1.0099	1.1472	0.9798	0.8873	1.0060	0.0538
AMELY	0.8942	1.1570	1.0590	0.9139	1.0060	0.0623
LOC642131	1.0146	1.0213	1.0505	0.9378	1.0060	0.0240
FLJ33706	1.0714	1.0521	0.9888	0.9120	1.0060	0.0360

CLEC17A	0.8879	1.1633	1.0222	0.9508	1.0061	0.0592
LOC642701	1.0323	1.0993	0.9078	0.9848	1.0061	0.0403
LOC1001282	1.1148	1.0989	0.9839	0.8266	1.0061	0.0666
EMP2	1.0427	1.1330	0.9606	0.8879	1.0061	0.0528
C13orf36	1.0046	1.0305	1.0895	0.8996	1.0061	0.0397
LOC440092	1.0229	1.1054	1.0532	0.8428	1.0061	0.0570
LOC641365	1.0058	1.0903	1.0298	0.8984	1.0061	0.0400
IGDCC4	0.9775	1.0848	0.9910	0.9710	1.0061	0.0266
C18orf58	0.9925	1.1745	1.0578	0.7996	1.0061	0.0784
LOC642196	0.8950	1.0873	1.0947	0.9473	1.0061	0.0502
CDH3	0.9574	1.1437	0.9768	0.9465	1.0061	0.0463
LOC1001320	1.0470	1.1035	1.0560	0.8180	1.0061	0.0639
CCDC57	0.9197	1.1404	1.0713	0.8930	1.0061	0.0595
ERCC-00156	1.0244	1.0658	1.0198	0.9144	1.0061	0.0323
LOC643161	0.9303	1.0900	1.0784	0.9258	1.0061	0.0452
SNORD9	0.9746	1.2192	0.9538	0.8768	1.0061	0.0741
CARD8	1.0045	1.1641	0.9930	0.8628	1.0061	0.0617
TCEB3C	1.0715	1.0541	0.9403	0.9586	1.0061	0.0331
LOC1001287	1.0074	1.0933	1.0469	0.8769	1.0061	0.0465
FASLG	1.0283	0.9594	1.0050	1.0319	1.0061	0.0167
C22orf42	0.9957	1.0472	1.0257	0.9559	1.0061	0.0198
OR8S1	0.9832	1.0775	1.0165	0.9474	1.0061	0.0277
LOC649703	0.9546	1.0642	1.0603	0.9453	1.0061	0.0325
LOC1001279	0.8548	1.1307	1.0480	0.9910	1.0061	0.0580
LOC649945	1.0024	1.0247	1.0436	0.9539	1.0061	0.0194
PLEKHH1	0.9954	1.3484	0.8976	0.7832	1.0061	0.1221
SPINK5L2	0.8959	1.0556	1.1345	0.9387	1.0062	0.0545
CCAR1	0.9274	1.1048	1.1146	0.8780	1.0062	0.0606
LOC644072	0.9447	1.0653	1.1041	0.9105	1.0062	0.0466
NTN4	0.9794	1.0708	1.0272	0.9474	1.0062	0.0271
LOC1001322	0.9491	1.0467	1.0201	1.0089	1.0062	0.0206
LHX9	0.9787	1.0772	1.0348	0.9341	1.0062	0.0314
C1QTNF9	0.9470	1.1679	1.0149	0.8950	1.0062	0.0592
NVL	0.9245	1.5422	0.9700	0.5882	1.0062	0.1979
LOC730288	0.7954	1.2416	1.1863	0.8016	1.0062	0.1205
LOC653188	1.0460	0.9773	1.0808	0.9207	1.0062	0.0357
LOC646033	0.9762	0.9884	1.0263	1.0341	1.0062	0.0141
BMS1P5	0.6914	1.3108	1.2387	0.7840	1.0062	0.1569
FOLR3	0.9634	1.0275	0.9715	1.0625	1.0062	0.0235
LOC730286	1.0245	1.1372	0.9460	0.9172	1.0062	0.0492
LOC642976	1.0229	1.0561	1.0031	0.9429	1.0062	0.0238
DACT1	0.9630	1.1161	1.0073	0.9386	1.0062	0.0393
LOC728208	0.9132	1.1539	1.0662	0.8916	1.0062	0.0627
LOC401237	0.8028	1.1745	1.0162	1.0315	1.0062	0.0766
IBSP	0.7787	1.3052	1.0356	0.9055	1.0062	0.1126
TRIOBP	0.8905	1.2636	1.0455	0.8254	1.0062	0.0974

LOC652305	0.9981	1.1098	1.0499	0.8672	1.0062	0.0517
SLC13A3	0.9388	1.1671	1.0368	0.8823	1.0063	0.0624
RNASEL	0.9975	1.2359	1.0370	0.7546	1.0063	0.0988
LOC648329	0.8796	1.0632	1.1107	0.9716	1.0063	0.0511
LOC650804	0.9880	1.0528	1.0377	0.9466	1.0063	0.0242
TOP1P1	0.9679	1.0950	1.0492	0.9129	1.0063	0.0407
ZNF578	1.0702	1.0159	1.0318	0.9072	1.0063	0.0349
LOC613206	1.0344	1.2131	0.9095	0.8682	1.0063	0.0775
CA4	0.9218	1.1063	1.0737	0.9233	1.0063	0.0488
MCART6	0.9384	1.0793	1.1283	0.8791	1.0063	0.0585
AFAP1	0.9496	1.1481	1.0254	0.9021	1.0063	0.0537
LOC647624	0.9329	1.2026	1.0436	0.8461	1.0063	0.0769
ARMC3	0.9847	1.0110	1.0760	0.9537	1.0063	0.0260
ADCK5	0.8931	1.2346	0.9824	0.9153	1.0063	0.0784
MIR509-3	0.9477	1.1113	0.9940	0.9725	1.0063	0.0362
ZNF763	0.9626	1.2315	1.0362	0.7951	1.0064	0.0904
MAP4K3	0.7815	1.5068	0.9261	0.8111	1.0064	0.1697
LOC442245	0.9436	1.1640	0.9864	0.9314	1.0064	0.0539
MAD1L1	1.0236	1.1682	0.9331	0.9005	1.0064	0.0599
DPEP2	0.9166	1.1476	1.0018	0.9595	1.0064	0.0502
LOC647500	0.9728	1.0712	1.0147	0.9668	1.0064	0.0241
LOC401911	0.9585	1.1261	1.0228	0.9181	1.0064	0.0454
WDR93	0.9756	1.0924	1.0507	0.9068	1.0064	0.0411
LOC1001312	1.0018	1.1138	0.9558	0.9541	1.0064	0.0375
LOC1001297	0.9914	1.1433	0.9022	0.9887	1.0064	0.0501
LOC1001322	0.9275	1.1573	1.0466	0.8942	1.0064	0.0600
LOC390531	0.9620	1.0961	1.0704	0.8972	1.0064	0.0466
GPX1	0.9021	1.3715	1.0784	0.6738	1.0064	0.1472
LOC387930	0.8665	1.1630	1.1103	0.8859	1.0064	0.0761
ACCN3	0.8980	1.0651	1.0880	0.9746	1.0064	0.0437
SLC5A7	0.9719	1.1005	0.9687	0.9847	1.0064	0.0315
KIAA0232	1.0139	1.1924	0.9207	0.8987	1.0064	0.0668
LOC1001302	0.9345	1.1275	1.0968	0.8670	1.0064	0.0629
NXNL1	0.9404	1.1408	1.0293	0.9154	1.0065	0.0510
FLJ40672	0.8681	1.0711	1.1374	0.9493	1.0065	0.0604
HTRA4	0.9360	1.1411	0.9943	0.9544	1.0065	0.0465
TAS2R50	0.9727	1.0493	0.9903	1.0136	1.0065	0.0165
C1orf190	0.8896	1.0277	1.1040	1.0046	1.0065	0.0444
LOC643426	0.9966	1.0102	1.0604	0.9587	1.0065	0.0210
OR10A4	0.9679	1.0917	1.0210	0.9455	1.0065	0.0325
LOC1001311	1.0297	1.1080	1.0335	0.8548	1.0065	0.0537
CEACAM6	1.0500	0.9960	1.1240	0.8561	1.0065	0.0566
MIR1231	0.9689	1.0231	1.2015	0.8325	1.0065	0.0764
FLJ23152	0.9473	1.1346	0.9814	0.9627	1.0065	0.0433
LOC1001923	0.9506	1.1364	1.0485	0.8905	1.0065	0.0542
STX17	0.9484	1.2265	1.0562	0.7949	1.0065	0.0909

LOC1001346	0.9537	1.0398	1.0280	1.0046	1.0065	0.0191
PLCG1	1.0379	1.2632	0.9793	0.7457	1.0065	0.1063
LOC652783	1.0289	1.0145	1.1092	0.8734	1.0065	0.0490
LOC1001322	0.9160	1.1042	1.0686	0.9373	1.0065	0.0469
EBI2	0.9638	1.1572	1.0334	0.8717	1.0065	0.0602
MGC42638	0.8850	1.0718	1.0685	1.0008	1.0065	0.0437
LOC1001295	0.8938	1.2246	0.9707	0.9370	1.0065	0.0744
BSN	0.9456	1.1686	0.9766	0.9353	1.0065	0.0547
NGB	0.9685	1.0555	1.0677	0.9345	1.0065	0.0326
LOC127406	1.0077	1.1473	1.0007	0.8704	1.0065	0.0566
CMKLR1	0.9811	1.0076	1.0967	0.9408	1.0065	0.0330
ERCC-00112	1.0170	1.0849	1.0608	0.8635	1.0066	0.0497
FKRP	0.9273	1.1544	1.1011	0.8434	1.0066	0.0729
KHNYN	1.1522	1.2038	0.9154	0.7548	1.0066	0.1048
LOC643493	0.9327	1.0681	1.0901	0.9354	1.0066	0.0421
LOC642834	0.9431	1.0593	1.0823	0.9417	1.0066	0.0374
HCG26	0.8578	1.2433	0.9874	0.9378	1.0066	0.0833
KRTAP10-4	1.0304	1.0874	0.9581	0.9504	1.0066	0.0324
CGB7	0.9630	1.1224	1.1149	0.8261	1.0066	0.0705
VSIG6	0.8825	1.1801	0.9725	0.9913	1.0066	0.0625
CD160	0.9362	1.0775	1.0948	0.9180	1.0066	0.0462
LOC649264	0.9409	1.1397	1.0108	0.9350	1.0066	0.0476
LOC645850	1.0893	1.0311	0.9470	0.9590	1.0066	0.0332
C7orf34	0.9014	1.0887	1.1660	0.8703	1.0066	0.0718
PTPRT	0.9237	1.0905	1.0332	0.9791	1.0066	0.0358
ADAMTS7	1.0495	1.0980	1.0087	0.8704	1.0066	0.0489
LOC1001341	0.9169	1.0708	1.0553	0.9834	1.0066	0.0354
BCO2	0.9619	1.1474	1.0049	0.9122	1.0066	0.0506
ERCC-00043	0.9524	1.0502	1.0438	0.9801	1.0066	0.0240
SLC17A8	1.1017	1.0305	1.0144	0.8800	1.0066	0.0463
QPRT	0.9694	1.0629	0.9678	1.0264	1.0066	0.0232
MAEL	0.8877	1.1789	0.9907	0.9692	1.0066	0.0616
MMP17	1.0072	1.0604	1.0313	0.9277	1.0066	0.0285
SBNO2	0.9599	1.2977	0.9898	0.7792	1.0066	0.1076
OR1B1	0.9717	1.1125	1.0481	0.8943	1.0067	0.0472
LOC648623	0.9675	1.1537	1.0137	0.8917	1.0067	0.0551
C11orf1	0.8518	1.3464	1.0803	0.7482	1.0067	0.1328
LOC344875	0.8952	1.1672	1.0899	0.8744	1.0067	0.0722
SLC38A4	0.9241	1.2099	1.0428	0.8498	1.0067	0.0785
GALR3	0.9353	1.0807	1.0478	0.9629	1.0067	0.0344
RBP3	1.0175	1.1239	0.9781	0.9072	1.0067	0.0452
SLC22A1	0.8691	1.2210	1.0363	0.9003	1.0067	0.0801
LOC653765	0.9960	1.0528	1.0328	0.9451	1.0067	0.0237
POM121L4P	1.0516	1.1912	0.9739	0.8100	1.0067	0.0795
FAM189A1	0.8640	1.2288	0.9659	0.9682	1.0067	0.0779
BATF2	1.0240	1.0625	0.9929	0.9474	1.0067	0.0244

TRPM7	0.9166	1.1739	1.0311	0.9052	1.0067	0.0625
LOC391142	0.9746	1.0950	1.0120	0.9452	1.0067	0.0325
LOC650118	0.9759	1.0698	1.0767	0.9045	1.0067	0.0411
LOC651597	1.0022	0.9857	1.0636	0.9754	1.0067	0.0197
LOC388494	0.9799	1.0421	1.0383	0.9667	1.0067	0.0195
CBLN2	0.9826	1.4836	0.8811	0.6797	1.0067	0.1710
SLC13A2	1.1524	1.0404	0.9959	0.8383	1.0067	0.0651
MAGEA9	0.9526	1.0220	1.1125	0.9399	1.0067	0.0396
MIR148B	0.9883	1.0837	1.0478	0.9072	1.0067	0.0386
LOC647806	1.0815	1.0614	1.0489	0.8352	1.0068	0.0576
BASE	1.0565	1.0507	0.9973	0.9225	1.0068	0.0311
IVNS1ABP	1.0045	1.1392	1.0459	0.8375	1.0068	0.0631
LOC442480	0.9829	1.1154	0.9650	0.9639	1.0068	0.0365
SLC19A3	0.9487	1.1457	1.0934	0.8393	1.0068	0.0697
PTPN22	0.9512	1.0369	1.0945	0.9446	1.0068	0.0360
BAG4	0.8682	1.3011	1.0685	0.7894	1.0068	0.1144
C12orf74	0.9450	1.1371	1.0239	0.9213	1.0068	0.0487
LOC641939	0.9349	1.1532	0.9193	1.0199	1.0068	0.0536
C12orf64	0.9581	1.1611	0.9894	0.9187	1.0068	0.0534
OR8B4	0.9606	0.9988	1.1010	0.9668	1.0068	0.0325
SGK269	0.9725	1.0459	1.0806	0.9282	1.0068	0.0346
SPPL2B	0.9239	1.1579	1.0133	0.9322	1.0068	0.0542
EMID1	1.0028	1.0571	1.0322	0.9351	1.0068	0.0263
LOC647760	0.9193	1.0879	1.0868	0.9333	1.0068	0.0466
MAMDC2	0.9258	1.0954	1.0730	0.9332	1.0069	0.0449
LOC645837	0.8607	1.4045	0.8866	0.8755	1.0069	0.1327
DKFZp547K0	0.9775	1.0565	1.0678	0.9256	1.0069	0.0337
LOC652272	0.9640	1.1197	1.0276	0.9162	1.0069	0.0440
HCG4P6	0.9230	1.1057	1.0893	0.9095	1.0069	0.0525
LOC649858	0.9845	1.1070	1.0144	0.9216	1.0069	0.0386
NBEA	0.9574	1.0719	1.1224	0.8758	1.0069	0.0557
NPEPL1	0.8421	1.2564	1.1250	0.8040	1.0069	0.1097
MIR1243	0.9922	0.9845	1.0327	1.0182	1.0069	0.0112
LOC643420	0.9376	1.0495	1.0778	0.9626	1.0069	0.0337
MMRN2	1.0064	1.1289	1.0601	0.8321	1.0069	0.0634
SMAD3	1.0163	1.0925	0.9887	0.9301	1.0069	0.0337
LOC644853	0.8837	1.1526	1.0384	0.9529	1.0069	0.0580
FLJ45872	1.0102	1.1118	0.9695	0.9362	1.0069	0.0381
LOC727983	0.8973	1.1955	1.0768	0.8580	1.0069	0.0789
C11orf16	0.9646	1.0786	1.0412	0.9433	1.0069	0.0318
HEPN1	0.9774	1.0242	1.0439	0.9821	1.0069	0.0162
LOC641742	0.8872	1.1521	1.0635	0.9250	1.0069	0.0614
LOC650151	0.9504	1.1802	0.9313	0.9659	1.0069	0.0582
LOC731035	0.9836	1.0855	1.0491	0.9096	1.0070	0.0387
LOC644631	0.9727	1.0671	1.0385	0.9496	1.0070	0.0275
LOC730845	1.0727	1.1098	0.9324	0.9130	1.0070	0.0494

C1orf129	0.9366	1.0809	1.0354	0.9750	1.0070	0.0319
LOC730953	0.8862	1.0173	1.1186	1.0058	1.0070	0.0476
MIR620	0.9421	1.1673	0.9873	0.9312	1.0070	0.0548
LOC653185	0.9445	1.1337	1.0492	0.9005	1.0070	0.0525
CD86	0.9491	1.0982	1.0245	0.9562	1.0070	0.0348
LOC653962	1.0641	1.0496	0.9897	0.9245	1.0070	0.0319
LOC1001287	0.9954	1.1037	1.0115	0.9173	1.0070	0.0382
GIMAP5	0.9954	1.1232	0.9814	0.9279	1.0070	0.0414
PBXIP1	0.9781	1.1318	0.9654	0.9528	1.0070	0.0419
RANBP6	0.8996	1.2549	1.1004	0.7731	1.0070	0.1066
SP5	0.9446	1.1049	1.0351	0.9435	1.0070	0.0390
LOC651427	0.9465	1.0884	1.0288	0.9644	1.0070	0.0324
MAP2K1IP1	0.8250	1.2969	1.1557	0.7506	1.0070	0.1307
KIAA1486	0.9645	1.0358	1.0665	0.9614	1.0070	0.0262
LOC653155	1.0245	1.0491	1.0021	0.9525	1.0071	0.0206
ITGB1BP3	0.9561	1.1378	0.9889	0.9454	1.0071	0.0446
LOC1001337	1.0475	1.0611	1.0267	0.8930	1.0071	0.0387
RET	0.9814	1.1300	0.9877	0.9292	1.0071	0.0430
C7orf70	0.8213	1.3516	0.9404	0.9150	1.0071	0.1177
ZBTB7A	0.8205	1.4139	0.9728	0.8212	1.0071	0.1402
LOC441666	0.8314	1.2771	1.0216	0.8983	1.0071	0.0983
LOC1001330	0.9222	1.2214	1.0840	0.8008	1.0071	0.0920
LOC1001285	0.9327	1.1048	1.0799	0.9110	1.0071	0.0497
FAT3	0.9192	1.1136	0.9738	1.0218	1.0071	0.0412
ARHGAP12	0.8770	1.3529	0.9793	0.8193	1.0071	0.1199
LOC646345	0.9843	1.1094	0.9800	0.9548	1.0071	0.0347
GPR139	0.9141	1.0352	1.1231	0.9561	1.0071	0.0461
GPR39	0.9399	1.0502	1.1091	0.9293	1.0071	0.0436
CCDC60	0.9773	1.0826	1.0953	0.8733	1.0071	0.0519
LOC652510	0.9151	1.1067	1.0300	0.9768	1.0071	0.0407
LRP6	0.9342	1.2835	0.9749	0.8360	1.0071	0.0966
SMAGP	0.9609	1.1133	1.0788	0.8756	1.0072	0.0547
GRIN3B	0.8785	1.1868	1.0804	0.8829	1.0072	0.0762
LOC730179	1.0218	1.1010	1.0210	0.8848	1.0072	0.0449
ASAP3	0.9548	1.3343	0.9254	0.8141	1.0072	0.1132
LOC652842	0.8539	1.1117	1.0992	0.9639	1.0072	0.0611
TRY6	0.8929	1.1210	1.0708	0.9441	1.0072	0.0533
RXRA	1.0543	1.2670	0.8728	0.8347	1.0072	0.0990
IKZF1	0.9630	1.0512	1.0696	0.9451	1.0072	0.0312
LOC1001295	0.9505	1.2366	1.0278	0.8140	1.0072	0.0883
LOC652078	0.9946	1.1071	0.9756	0.9517	1.0072	0.0344
LOC650003	0.9600	1.0958	1.0753	0.8980	1.0072	0.0471
MMP16	0.9382	1.1247	1.1093	0.8568	1.0073	0.0656
HRG	0.9456	1.1267	1.0485	0.9082	1.0073	0.0496
SSR4	0.8783	1.4763	1.0459	0.6286	1.0073	0.1783
LMTK2	0.9646	1.1031	0.9647	0.9966	1.0073	0.0328

LOC440848	0.9144	1.0008	1.1300	0.9840	1.0073	0.0450
LOC729549	0.9384	1.0663	1.0604	0.9640	1.0073	0.0328
LOC643242	0.9495	1.1277	0.9434	1.0085	1.0073	0.0427
CRADD	0.9241	1.3975	0.9278	0.7798	1.0073	0.1346
RAD9B	0.8884	1.1687	1.0881	0.8839	1.0073	0.0718
PRKCABP	1.0060	1.1794	0.9792	0.8646	1.0073	0.0650
ANXA10	0.9800	1.0994	1.0961	0.8536	1.0073	0.0583
LOC646461	0.9406	1.1363	1.0206	0.9316	1.0073	0.0474
ALX4	0.8349	1.1580	1.1386	0.8978	1.0073	0.0825
ZNF204	0.9334	1.1235	1.0282	0.9442	1.0073	0.0441
LOC651561	0.9613	1.1082	1.1047	0.8551	1.0073	0.0612
LOC728310	1.0129	1.0712	1.0432	0.9021	1.0073	0.0371
SLC5A2	0.9416	1.0783	1.0664	0.9432	1.0074	0.0376
LOC646512	1.0845	1.0019	0.9808	0.9622	1.0074	0.0270
LOC647711	1.0220	1.0966	0.9481	0.9628	1.0074	0.0338
KIAA0427	0.9277	1.2228	1.1118	0.7672	1.0074	0.1006
LOC644781	0.9413	1.1468	0.9554	0.9861	1.0074	0.0474
ERCC-00113	0.9000	1.1757	1.0036	0.9503	1.0074	0.0599
AMBP	0.9958	0.9924	1.0336	1.0079	1.0074	0.0093
DUOX2	0.9623	1.0036	1.1379	0.9259	1.0074	0.0463
LOC391276	0.9697	1.0247	1.0652	0.9702	1.0074	0.0232
FAM91A2	0.9901	1.0824	1.0608	0.8964	1.0074	0.0419
LOC650346	1.0222	1.0019	1.0311	0.9745	1.0074	0.0126
LOC286456	1.0651	1.0492	1.0288	0.8867	1.0074	0.0409
MIR211	1.1330	1.0463	0.9641	0.8863	1.0074	0.0531
TMEM195	0.9691	1.0556	0.9659	1.0392	1.0074	0.0233
LOC648489	0.9203	1.0639	1.0831	0.9624	1.0074	0.0393
DAOA	1.0260	1.0272	1.0507	0.9260	1.0075	0.0278
OR2L8	0.9804	0.9976	1.0506	1.0013	1.0075	0.0151
LOC652483	0.8114	1.2392	1.1187	0.8607	1.0075	0.1025
BST2	0.8733	1.1277	0.9895	1.0395	1.0075	0.0531
SLC18A3	0.9443	1.0914	1.0524	0.9420	1.0075	0.0380
LY6K	0.9467	1.0805	1.0867	0.9162	1.0075	0.0444
LOC643799	0.8978	1.1429	1.0964	0.8930	1.0075	0.0654
PCDHGB2	0.9864	1.1197	1.0041	0.9197	1.0075	0.0416
ZNF708	1.0220	1.0440	1.0620	0.9021	1.0075	0.0361
DLD	0.8349	1.2664	1.3289	0.5999	1.0075	0.1747
LOC442512	0.9728	1.1354	1.0312	0.8907	1.0075	0.0514
LOC1001338	0.9982	1.0214	1.0975	0.9131	1.0075	0.0380
LOC652665	1.0308	1.0383	1.0965	0.8645	1.0075	0.0499
LOC644642	0.9409	1.0640	1.0518	0.9735	1.0075	0.0299
SLC2A2	0.9264	1.0907	1.0266	0.9866	1.0076	0.0345
RNF40	0.9261	1.4112	0.9635	0.7294	1.0076	0.1440
BCAS2	0.9967	1.0694	1.1534	0.8108	1.0076	0.0730
PAPSS2	1.0822	1.0033	1.0484	0.8965	1.0076	0.0404
OR9Q2	0.8952	1.0648	1.0454	1.0250	1.0076	0.0383

ANKRD26	0.9688	1.2998	1.0036	0.7582	1.0076	0.1115
LOC648154	0.9376	1.1631	1.0675	0.8623	1.0076	0.0669
LOC643153	0.9911	1.0930	1.1306	0.8158	1.0076	0.0704
CLPTM1	1.0344	1.1045	0.9716	0.9200	1.0076	0.0399
SF3A3	0.8329	1.2143	1.1126	0.8708	1.0076	0.0926
LRBA	0.8866	1.3482	1.0525	0.7433	1.0076	0.1299
FAM92A3	0.9447	1.1672	0.9777	0.9410	1.0077	0.0538
PRIM2	0.8028	1.1023	1.0667	1.0588	1.0077	0.0690
LOC1001338	1.0200	1.1404	1.0960	0.7742	1.0077	0.0817
HS3ST1	0.9828	1.1711	0.9974	0.8794	1.0077	0.0605
IL28RA	0.9245	1.0876	1.0518	0.9667	1.0077	0.0376
LOC1001329	0.9696	1.1133	0.9928	0.9550	1.0077	0.0361
CLDN11	0.9241	1.1986	1.0061	0.9019	1.0077	0.0675
SDR39U1	0.8974	1.2901	1.1394	0.7039	1.0077	0.1296
IQCA1	1.0160	1.1454	0.9887	0.8807	1.0077	0.0544
FCRLA	0.8893	1.1003	1.0704	0.9708	1.0077	0.0482
LOC1001339	0.8898	1.0481	1.1835	0.9095	1.0077	0.0684
C11orf88	0.9901	1.0462	1.0687	0.9259	1.0077	0.0319
ZNF44	0.9459	1.0975	1.0441	0.9435	1.0077	0.0380
TSPAN6	1.0000	1.1748	0.9964	0.8599	1.0077	0.0645
LOC642744	1.0973	0.9760	1.0618	0.8959	1.0078	0.0451
ELMOD3	1.0181	1.2012	1.0564	0.7552	1.0078	0.0929
ZNF83	0.8935	1.1159	1.1564	0.8652	1.0078	0.0748
KIAA1666	0.8398	1.1526	1.1321	0.9066	1.0078	0.0790
DNAI1	0.9541	1.0229	1.0468	1.0073	1.0078	0.0196
TMEFF1	0.9639	1.0949	1.0275	0.9448	1.0078	0.0340
C10orf40	0.9037	1.0077	1.0999	1.0198	1.0078	0.0403
LOC642712	0.9951	1.0688	1.0072	0.9600	1.0078	0.0227
LOC653748	0.9430	1.0919	1.0520	0.9442	1.0078	0.0379
C2orf34	0.8143	1.3741	1.0435	0.7992	1.0078	0.1343
LOC645396	0.9496	1.1068	1.0689	0.9059	1.0078	0.0477
SEL1L2	0.9154	1.0831	1.1858	0.8468	1.0078	0.0774
NCKAP5	0.9628	1.0814	1.0989	0.8881	1.0078	0.0501
LOC730796	0.9496	1.1211	1.0159	0.9448	1.0078	0.0411
ZDHHC2	1.0347	1.0657	1.0681	0.8629	1.0079	0.0489
LOC1001310	0.9034	1.1152	1.0471	0.9656	1.0079	0.0463
C12orf50	0.9401	1.1452	1.0244	0.9219	1.0079	0.0509
CYP11B1	0.9865	1.0826	0.9986	0.9639	1.0079	0.0259
MIR1237	0.9557	1.1943	0.9234	0.9582	1.0079	0.0626
LOC649563	0.9422	1.0265	1.0723	0.9906	1.0079	0.0276
LOC728729	1.0056	1.1011	1.0037	0.9212	1.0079	0.0368
LOC646484	1.0165	1.0813	1.0014	0.9324	1.0079	0.0306
LOC728012	1.0875	1.0300	0.9880	0.9260	1.0079	0.0341
SNAPC1	0.8637	1.3204	0.9983	0.8492	1.0079	0.1095
LOC642740	0.9841	1.1010	1.0773	0.8693	1.0079	0.0526
LOC729185	0.8598	1.1794	1.1047	0.8878	1.0079	0.0791

FLJ32063	1.0394	1.1068	0.9819	0.9035	1.0079	0.0431
LOC1001278	0.9712	1.0179	1.1351	0.9075	1.0079	0.0481
RDH8	0.8902	1.1048	1.1085	0.9283	1.0079	0.0575
LOC649255	0.9161	0.9930	1.1172	1.0056	1.0079	0.0414
UTY	0.9786	1.1335	1.0225	0.8972	1.0079	0.0493
ATP8A1	0.9771	1.1575	1.0293	0.8679	1.0080	0.0601
APOA5	0.9541	1.2932	0.9039	0.8807	1.0080	0.0963
LOC643755	0.9476	1.0378	0.9851	1.0615	1.0080	0.0257
LOC1001295	0.9347	1.1606	1.0622	0.8744	1.0080	0.0642
OR5A1	0.9501	1.1076	1.0288	0.9454	1.0080	0.0383
LOC653683	1.0136	1.0761	0.9885	0.9536	1.0080	0.0258
NEFM	0.9779	1.0696	0.9814	1.0030	1.0080	0.0213
LOC727984	0.7335	1.1589	1.5005	0.6390	1.0080	0.1994
LOC644761	0.8855	1.3510	1.0707	0.7247	1.0080	0.1344
LIMD1	1.0444	1.0629	1.0146	0.9101	1.0080	0.0341
CAMSAP1	0.8647	1.5137	0.9904	0.6633	1.0080	0.1815
SNORD114-3	1.0040	1.0711	1.0730	0.8839	1.0080	0.0444
SLC22A18	0.9540	1.2969	0.9618	0.8195	1.0080	0.1017
RPS19	0.8431	1.0974	1.2225	0.8692	1.0080	0.0915
LOC647295	1.0356	1.0450	0.9361	1.0154	1.0080	0.0248
RAB3A	0.9037	1.2927	0.9804	0.8553	1.0080	0.0983
CXorf12	1.0448	1.2961	0.8648	0.8266	1.0081	0.1071
ANGPT1	0.9240	1.1873	0.9867	0.9343	1.0081	0.0613
ARHGAP20	1.0640	1.1012	1.0680	0.7991	1.0081	0.0702
DLK1	0.9634	1.1552	0.9838	0.9299	1.0081	0.0503
KLK10	0.9126	1.1565	1.0774	0.8858	1.0081	0.0651
C1orf170	0.8371	1.1503	1.1046	0.9403	1.0081	0.0727
SNORA1	0.9305	1.1100	1.0155	0.9764	1.0081	0.0382
DEFB121	0.9340	1.0645	1.0652	0.9686	1.0081	0.0335
OR5M1	1.0164	1.0692	1.0292	0.9176	1.0081	0.0322
ZNF805	0.9933	1.1862	1.0810	0.7718	1.0081	0.0881
NFATC4	0.9174	1.1335	1.0478	0.9337	1.0081	0.0509
FAM22D	0.9621	1.1272	1.0549	0.8881	1.0081	0.0524
LOC90586	0.9026	1.1037	1.2302	0.7959	1.0081	0.0977
LOC644083	0.9529	1.2072	0.9822	0.8901	1.0081	0.0691
LRRIQ4	0.9437	1.1359	1.0735	0.8795	1.0081	0.0587
LOC642916	0.8361	1.2016	1.1091	0.8858	1.0081	0.0876
MIR1255B2	0.9149	1.1055	1.1093	0.9029	1.0081	0.0574
MIR1911	0.9749	1.1358	1.0236	0.8984	1.0081	0.0497
CD300LD	1.0060	1.1208	1.0253	0.8806	1.0082	0.0494
ZNF324	0.8977	1.3816	0.9705	0.7828	1.0082	0.1304
TRIM62	1.0279	1.1140	1.0654	0.8254	1.0082	0.0634
ZC3H6	0.8867	1.2034	1.0550	0.8876	1.0082	0.0762
LOC654115	1.0694	1.0140	1.0448	0.9045	1.0082	0.0364
LOC1001317	0.9586	1.0998	1.0789	0.8955	1.0082	0.0488
OR2M4	0.9214	1.1157	0.9823	1.0134	1.0082	0.0406

CD300LF	1.0101	0.9811	1.1166	0.9251	1.0082	0.0402
C10orf111	1.0283	1.1173	1.0364	0.8508	1.0082	0.0562
LOC654253	1.0638	1.2562	0.9576	0.7553	1.0082	0.1045
LOC653567	0.8609	1.2784	0.9255	0.9681	1.0082	0.0927
ZNF833	0.8729	1.1016	1.0971	0.9613	1.0082	0.0556
CHRD	1.0562	1.0790	0.8873	1.0105	1.0082	0.0428
ISY1	0.8153	1.4028	1.0686	0.7463	1.0082	0.1486
LOC730656	0.8593	1.2200	0.9441	1.0096	1.0082	0.0770
LOC1001342	1.0316	1.0927	1.0066	0.9021	1.0083	0.0397
FARP1	0.9921	1.1886	0.9709	0.8814	1.0083	0.0647
LOC1001342	1.0820	0.9801	1.0590	0.9120	1.0083	0.0388
MYOZ2	0.9928	1.0236	1.0663	0.9505	1.0083	0.0245
LOC642558	1.0141	1.0745	1.0603	0.8843	1.0083	0.0433
DLGAP3	0.8762	1.0937	1.1463	0.9171	1.0083	0.0659
MIR129-2	0.8659	1.2098	1.1970	0.7607	1.0083	0.1147
LOC643008	0.9649	1.1100	1.0553	0.9031	1.0083	0.0461
TBKBP1	0.9413	1.1068	0.9952	0.9900	1.0083	0.0350
GRIK1	0.9675	1.0979	1.0467	0.9212	1.0083	0.0395
LOC1001280	0.8974	1.1751	1.0528	0.9081	1.0084	0.0659
LOC650678	1.0274	1.1225	0.9068	0.9769	1.0084	0.0454
LOC1001339	0.9892	1.0982	1.0325	0.9136	1.0084	0.0387
EDEM3	0.9081	1.2353	1.0497	0.8403	1.0084	0.0873
LRRIQ1	0.9332	1.1415	1.0040	0.9548	1.0084	0.0468
CHIA	0.9385	1.1090	1.0142	0.9718	1.0084	0.0369
LOC728163	0.9592	1.2002	0.9578	0.9163	1.0084	0.0647
LOC441362	0.9482	1.0624	1.1209	0.9021	1.0084	0.0504
LOC644133	0.9348	1.1523	1.1097	0.8369	1.0084	0.0741
C1orf110	0.9699	1.0621	1.0430	0.9587	1.0084	0.0259
LOC399746	0.9084	1.0916	1.1185	0.9151	1.0084	0.0561
C12orf56	0.8601	1.0619	1.0782	1.0334	1.0084	0.0503
NEXN	0.9262	1.1159	1.0565	0.9350	1.0084	0.0465
LAMA4	0.9586	1.0819	1.0381	0.9552	1.0084	0.0311
CCHCR1	0.9063	1.0888	1.0581	0.9805	1.0084	0.0410
LOC646040	0.9876	0.9829	1.0796	0.9837	1.0084	0.0237
MIR1278	1.0027	1.0801	1.0675	0.8835	1.0084	0.0450
LOC374443	0.9359	1.1356	1.1336	0.8287	1.0085	0.0760
MFAP1	0.9767	1.3044	1.0315	0.7212	1.0085	0.1196
LOC646476	0.9519	1.3375	0.9012	0.8434	1.0085	0.1119
GNMT	1.0295	1.3895	0.9056	0.7094	1.0085	0.1431
FIP1L1	0.8263	1.3417	1.0324	0.8337	1.0085	0.1209
SULT6B1	0.9122	1.1034	1.0312	0.9872	1.0085	0.0400
BHLHB9	0.9601	1.2746	1.0789	0.7205	1.0085	0.1158
F2R	1.0041	1.2545	1.0046	0.7709	1.0085	0.0988
LOC728937	0.9874	1.0816	1.1261	0.8390	1.0085	0.0635
LOC646226	0.9561	1.0564	1.0962	0.9255	1.0085	0.0404
LOC440419	1.0032	1.0780	0.9606	0.9924	1.0085	0.0249

TRIM49	0.8784	1.0982	1.0560	1.0015	1.0085	0.0477
TTC24	0.9746	1.0427	1.0641	0.9527	1.0085	0.0267
LOC1001302	0.9603	1.0341	1.0906	0.9492	1.0086	0.0332
LOC642576	0.9504	0.9458	1.1230	1.0150	1.0086	0.0413
RALGAPA2	0.9328	1.0827	1.0491	0.9697	1.0086	0.0346
LOC643752	1.0228	1.0784	0.9507	0.9824	1.0086	0.0275
GSTA5	0.9203	1.1370	1.0201	0.9569	1.0086	0.0475
LOC650850	1.0651	1.0485	0.9611	0.9596	1.0086	0.0281
LOC730240	1.0773	1.1351	1.0219	0.8001	1.0086	0.0732
LOC1001298	0.9382	1.0584	1.1292	0.9086	1.0086	0.0516
LOC1001332	0.8863	1.0081	1.1537	0.9863	1.0086	0.0552
ZNF831	0.9656	1.0239	1.0748	0.9702	1.0086	0.0257
C8orf22	0.9774	1.1731	1.0439	0.8401	1.0086	0.0693
LOC642406	1.0752	1.0592	0.9475	0.9526	1.0086	0.0340
PCDHGA10	0.9503	1.1430	1.0117	0.9295	1.0086	0.0481
LOC1001310	0.9632	1.0592	1.0146	0.9975	1.0086	0.0200
KCNK15	0.8882	1.2022	1.0658	0.8783	1.0086	0.0776
FBN2	1.0364	1.1537	0.9813	0.8631	1.0086	0.0604
LOC441459	1.0384	1.1177	1.0603	0.8182	1.0086	0.0657
LOC729270	0.9325	1.1506	0.9869	0.9646	1.0086	0.0486
SLC2A10	1.0056	1.1214	1.0355	0.8721	1.0086	0.0517
LOC1001293	1.0081	1.0361	1.1152	0.8752	1.0086	0.0499
MIR891A	0.9724	1.1046	1.0038	0.9538	1.0087	0.0336
OR7E37P	1.0417	1.2121	0.9087	0.8723	1.0087	0.0770
C2orf86	0.9877	1.0605	1.1031	0.8834	1.0087	0.0481
KCNG1	0.9862	1.1704	0.9770	0.9010	1.0087	0.0572
B3GNT3	0.8993	1.1802	0.9786	0.9766	1.0087	0.0601
LOC1001281	0.9197	1.0149	1.1303	0.9700	1.0087	0.0449
CER1	1.0037	1.0523	1.0657	0.9131	1.0087	0.0345
GPC5	0.9628	1.1402	1.0402	0.8917	1.0087	0.0533
LOC648700	1.0429	1.0980	1.0082	0.8857	1.0087	0.0450
LOC644484	1.0887	1.0385	0.9302	0.9774	1.0087	0.0347
DTX3L	1.0223	1.1507	0.9276	0.9342	1.0087	0.0520
MIR375	0.9012	1.1648	1.0737	0.8952	1.0087	0.0665
OR51M1	0.8376	1.1907	1.0529	0.9537	1.0087	0.0749
TAF6	0.8897	1.2345	1.0591	0.8516	1.0087	0.0877
LOC1001314	1.0647	1.0734	1.0409	0.8559	1.0087	0.0514
LOC441253	0.8828	1.0867	1.1474	0.9180	1.0087	0.0642
THAP2	0.9973	1.0374	1.0921	0.9082	1.0087	0.0387
LOC649503	1.0333	1.0076	0.9865	1.0075	1.0087	0.0096
LOC644234	0.8747	1.2679	1.0294	0.8630	1.0087	0.0943
LOC653276	0.9443	1.1217	1.0543	0.9148	1.0088	0.0481
LOC644656	1.0726	0.9002	1.0536	1.0086	1.0088	0.0386
LOC652530	0.9414	1.0494	1.1186	0.9257	1.0088	0.0458
THY1	0.9400	1.0287	1.1641	0.9023	1.0088	0.0582
HOXA11	0.9617	1.0057	1.0861	0.9816	1.0088	0.0273

LOC728621	0.9501	1.0480	1.0886	0.9485	1.0088	0.0353
TM7SF2	1.0122	1.2319	0.9926	0.7985	1.0088	0.0886
PAEP	0.9118	1.1139	1.0405	0.9691	1.0088	0.0438
PIGZ	1.0387	1.0878	1.0032	0.9057	1.0088	0.0385
NEK5	0.9963	1.0981	0.9745	0.9665	1.0088	0.0304
LOC643797	0.9110	1.2395	0.9583	0.9266	1.0088	0.0775
LOC389300	0.8766	1.1483	1.1410	0.8695	1.0088	0.0784
LOC652100	0.9224	1.1571	0.9552	1.0007	1.0088	0.0519
LOC645335	0.9427	1.1178	1.0686	0.9062	1.0088	0.0503
MAPK11	0.9552	1.2036	1.0360	0.8407	1.0089	0.0763
XKR6	0.9309	1.1366	1.0503	0.9176	1.0089	0.0520
SFRP5	0.9403	1.1378	1.0424	0.9150	1.0089	0.0510
LOC339789	0.9821	1.0932	1.0901	0.8702	1.0089	0.0530
LOC652152	0.9775	1.0410	1.0486	0.9685	1.0089	0.0209
TNFRSF13B	0.9287	1.0316	1.0805	0.9948	1.0089	0.0320
LOC1001337	0.9822	1.1410	1.1567	0.7556	1.0089	0.0932
RARS2	0.8403	1.3455	1.0353	0.8145	1.0089	0.1225
KRT27	0.9817	1.1094	1.0433	0.9012	1.0089	0.0444
GLRX	0.8755	1.3161	1.0311	0.8129	1.0089	0.1122
LOC1001313	0.9355	1.1303	1.0899	0.8799	1.0089	0.0601
RPL6	0.9631	1.1514	1.0849	0.8363	1.0089	0.0695
LOC645279	0.9134	1.1952	1.0580	0.8690	1.0089	0.0741
YIPF3	1.0625	1.1277	1.0897	0.7559	1.0089	0.0854
TFEB	0.9289	1.1089	0.9967	1.0012	1.0089	0.0372
KCNE1L	1.0305	1.0929	1.0458	0.8666	1.0090	0.0493
OR2T4	1.0524	1.0862	0.9383	0.9590	1.0090	0.0358
TAS2R38	1.0226	1.0669	1.0591	0.8873	1.0090	0.0417
SLURP1	0.9474	1.1003	1.0955	0.8927	1.0090	0.0525
MIR571	1.0011	1.0381	1.0217	0.9749	1.0090	0.0136
LOC641846	0.8799	1.1640	1.0077	0.9843	1.0090	0.0587
SLC7A3	0.9343	1.1038	1.0286	0.9692	1.0090	0.0371
KCND3	1.0005	1.0387	1.1169	0.8798	1.0090	0.0494
TTTY6	0.9368	1.1936	0.9897	0.9160	1.0090	0.0634
ZNF788	0.9564	1.0670	1.0950	0.9176	1.0090	0.0427
GGNBP1	0.9682	1.1795	1.1243	0.7640	1.0090	0.0931
LOC646609	0.9346	1.0207	1.2001	0.8807	1.0090	0.0699
KCTD7	1.0072	1.0231	1.0873	0.9185	1.0090	0.0348
LOC643938	0.9502	1.0932	1.0514	0.9414	1.0090	0.0376
CNNM1	0.9963	1.0678	1.0179	0.9542	1.0090	0.0236
FRMPD4	1.0500	1.0960	0.9861	0.9040	1.0090	0.0416
KIAA0527	0.9462	1.1117	1.0516	0.9267	1.0091	0.0439
LOC647129	0.9812	0.9479	1.0815	1.0256	1.0091	0.0289
LOC441097	0.9141	1.0153	1.2023	0.9046	1.0091	0.0691
TXNDC11	1.1129	1.1781	0.9364	0.8089	1.0091	0.0840
LOC729427	0.9090	1.1106	1.0581	0.9586	1.0091	0.0459
GJC2	0.9460	1.2212	0.9758	0.8933	1.0091	0.0727

MED15	0.9951	1.1482	0.9293	0.9638	1.0091	0.0483
FREM3	0.9151	1.0814	0.9425	1.0973	1.0091	0.0468
P2RX4	0.9743	1.2092	1.0354	0.8175	1.0091	0.0810
PRPH2	0.8857	1.1621	0.9724	1.0161	1.0091	0.0578
LOC652614	0.9810	1.0748	1.0826	0.8980	1.0091	0.0436
CHRND	0.8756	1.0763	1.0915	0.9931	1.0091	0.0495
LOC1001304	0.9538	1.1356	1.0112	0.9358	1.0091	0.0451
LOC1001302	0.9058	1.1255	1.0489	0.9563	1.0091	0.0488
GAK	0.9168	1.4224	0.9299	0.7674	1.0091	0.1426
LOC642782	1.0231	0.9958	1.1006	0.9171	1.0091	0.0379
LOC646663	0.9141	1.0975	1.0801	0.9448	1.0091	0.0466
PRDM11	0.9641	1.1403	1.0502	0.8820	1.0091	0.0556
HBII-52-45	0.9829	1.1352	1.0174	0.9011	1.0092	0.0486
LOC392382	0.9975	1.1599	0.9843	0.8949	1.0092	0.0552
FAM83E	1.0366	1.1425	0.9767	0.8808	1.0092	0.0548
LCT	1.0305	1.0391	1.0413	0.9257	1.0092	0.0279
HRH4	0.9556	1.0818	1.0846	0.9147	1.0092	0.0435
COL9A1	1.0286	1.0804	0.9860	0.9418	1.0092	0.0296
LOC647011	1.0970	1.1711	0.8901	0.8785	1.0092	0.0737
LOC642049	0.8935	1.1189	1.0781	0.9463	1.0092	0.0533
LOC1001345	0.9672	1.1506	1.0910	0.8281	1.0092	0.0714
LOC647827	0.9634	1.0966	1.0766	0.9003	1.0092	0.0467
LOC648058	1.0202	1.0157	1.0194	0.9816	1.0092	0.0092
LOC1001343	0.9281	1.1423	1.0231	0.9434	1.0092	0.0490
TRIM78P	0.9810	1.1225	0.9197	1.0138	1.0092	0.0425
LOC728152	1.0052	1.1915	0.9182	0.9220	1.0092	0.0640
TBC1D10A	1.0058	1.2400	0.9566	0.8346	1.0093	0.0849
LOC645944	0.9525	1.1139	1.0184	0.9523	1.0093	0.0382
SIM1	1.0410	1.1180	0.9928	0.8853	1.0093	0.0487
ITGA4	0.8495	1.0377	1.0598	1.0901	1.0093	0.0543
SAMD13	0.9746	1.3606	1.0142	0.6879	1.0093	0.1378
SERHL2	0.9952	1.1153	1.0484	0.8783	1.0093	0.0501
LOC643503	0.9738	1.1482	0.9529	0.9625	1.0093	0.0465
LOC120824	0.9901	1.1295	0.9596	0.9582	1.0093	0.0407
SPRR2D	1.0186	1.1017	1.0375	0.8797	1.0094	0.0467
OR51B2	0.9014	1.1042	1.0724	0.9595	1.0094	0.0475
LDLRAD1	1.0192	1.0409	1.1406	0.8368	1.0094	0.0633
FNDC8	0.9309	1.0735	1.1031	0.9299	1.0094	0.0460
LOC1001316	0.9814	1.1398	1.0375	0.8788	1.0094	0.0545
YY2	0.9986	1.1686	1.0216	0.8487	1.0094	0.0655
MIR1256	1.0106	1.1103	0.9269	0.9897	1.0094	0.0381
LOC651476	0.9198	0.9350	1.0590	1.1237	1.0094	0.0492
HLA-DQA2	0.9339	1.0865	1.0550	0.9621	1.0094	0.0365
UQCC	0.9260	1.2759	1.0858	0.7499	1.0094	0.1122
ODF3	0.9188	1.2280	0.9336	0.9572	1.0094	0.0733
ZNF135	0.9990	1.0290	1.0580	0.9516	1.0094	0.0227

FAM75A1	1.0029	1.0941	1.0607	0.8799	1.0094	0.0471
RAB11FIP5	1.0266	1.3742	0.8421	0.7949	1.0094	0.1315
LOC653702	0.8509	0.9461	1.4704	0.7705	1.0094	0.1578
CACNB3	1.1547	1.3566	0.7526	0.7740	1.0094	0.1480
LOC728737	0.9726	1.1003	1.0586	0.9063	1.0095	0.0435
LOC440895	0.9913	1.1070	1.0433	0.8963	1.0095	0.0445
ABCA5	0.9312	1.1030	1.0775	0.9262	1.0095	0.0469
CRIM1	0.8696	1.1678	1.0669	0.9337	1.0095	0.0669
JARID1C	0.9350	1.2328	0.9664	0.9036	1.0095	0.0755
RSHL3	0.9393	1.0947	1.0795	0.9244	1.0095	0.0450
LOC1001298	0.9522	1.1459	1.0571	0.8827	1.0095	0.0579
C19orf64	0.9471	1.1805	0.9918	0.9185	1.0095	0.0590
OR2AG2	0.8988	1.2119	0.9318	0.9954	1.0095	0.0704
LOC645146	0.9790	1.1756	0.9659	0.9174	1.0095	0.0569
WNT4	0.8898	1.1876	1.0978	0.8627	1.0095	0.0793
CLSTN3	0.9854	1.1445	1.0113	0.8969	1.0095	0.0512
FOLR1	0.9541	1.1025	1.0600	0.9214	1.0095	0.0428
NRCAM	0.9476	1.1795	1.0269	0.8841	1.0095	0.0637
MIR183	1.0147	1.1184	1.0523	0.8527	1.0095	0.0565
LOC652134	0.8933	1.1126	1.0787	0.9535	1.0095	0.0517
SH2D3C	0.9606	1.1017	1.0458	0.9300	1.0095	0.0393
ADAM15	0.7910	1.5056	1.0770	0.6646	1.0095	0.1865
ACTC1	0.9482	1.0768	1.1044	0.9088	1.0095	0.0478
KIR2DL3	1.0185	1.0789	1.0280	0.9128	1.0096	0.0349
CR1L	1.0557	0.9779	1.0650	0.9397	1.0096	0.0304
LOC645553	0.9745	1.0531	1.1285	0.8821	1.0096	0.0528
LOC651062	1.0983	1.0959	1.0116	0.8325	1.0096	0.0624
LOC646812	1.0565	1.1003	0.9829	0.8987	1.0096	0.0442
XCL1	0.9252	1.1418	1.0769	0.8944	1.0096	0.0595
CTLA4	0.9289	1.1389	1.0273	0.9432	1.0096	0.0483
LOC650509	0.8781	1.0855	1.0574	1.0173	1.0096	0.0460
LOC339529	0.8867	1.0945	1.1225	0.9348	1.0096	0.0582
LPAR1	1.0121	1.0629	1.0435	0.9200	1.0096	0.0317
LOC646895	0.9605	1.1442	1.0089	0.9249	1.0096	0.0480
LOC652806	0.9038	1.0478	1.0655	1.0215	1.0096	0.0364
LOC648346	0.9604	1.1064	0.9883	0.9835	1.0097	0.0328
HTR3B	0.8814	1.0791	1.0385	1.0397	1.0097	0.0438
LOC440407	0.9253	1.1252	1.0310	0.9571	1.0097	0.0444
OR4C11	0.9900	1.0312	1.0901	0.9274	1.0097	0.0343
LOC651963	0.9500	1.0762	1.1287	0.8839	1.0097	0.0563
KIAA1602	0.9385	1.2350	1.0188	0.8464	1.0097	0.0830
LOC642769	0.9562	1.2657	0.9657	0.8511	1.0097	0.0892
RALGDS	0.8797	1.3683	1.0198	0.7710	1.0097	0.1299
TAS2R45	0.9352	1.1071	1.1114	0.8852	1.0097	0.0584
IDH3G	0.9741	1.3331	0.9442	0.7874	1.0097	0.1153
ATAD5	0.8522	1.1366	1.1716	0.8785	1.0097	0.0838

FLJ25715	1.0036	1.1511	1.0402	0.8439	1.0097	0.0636
ACCN5	0.9709	1.1150	1.0518	0.9012	1.0097	0.0467
SRL	0.9304	1.0395	1.0087	1.0604	1.0097	0.0285
LOC653557	1.0207	1.2591	0.9907	0.7685	1.0097	0.1004
LOC1001340	0.8290	1.1261	1.1428	0.9412	1.0097	0.0756
LOC728876	0.9483	1.2014	1.0119	0.8774	1.0097	0.0695
LOC729659	1.0091	1.0738	1.0437	0.9125	1.0098	0.0350
PEX10	0.9733	1.2667	1.0365	0.7625	1.0098	0.1038
C3orf56	1.0766	1.0133	1.0882	0.8610	1.0098	0.0522
PPFIA2	0.9321	1.4563	0.8803	0.7706	1.0098	0.1526
LOC644986	0.9961	1.0761	1.0431	0.9239	1.0098	0.0330
RASSF8	0.9576	1.1025	1.0672	0.9120	1.0098	0.0449
LOC642428	0.9669	0.9951	1.0820	0.9952	1.0098	0.0250
C10orf90	0.9534	1.0119	1.1293	0.9447	1.0098	0.0425
LOC652418	1.1058	1.0878	0.9548	0.8909	1.0098	0.0520
LOC651821	0.9457	1.0516	1.0627	0.9793	1.0098	0.0283
DMRTA2	1.0426	1.0510	0.9652	0.9805	1.0098	0.0216
ANKRD20A4	0.9153	1.0962	1.0197	1.0082	1.0098	0.0371
LOC652522	0.9436	1.1064	1.0764	0.9131	1.0098	0.0479
MIR605	0.9986	1.0119	1.0982	0.9306	1.0099	0.0344
IGSF3	0.9922	1.3099	0.9920	0.7454	1.0099	0.1157
APOL5	1.0005	1.0252	1.0333	0.9805	1.0099	0.0120
LOC1001347	1.0115	1.1507	0.9905	0.8868	1.0099	0.0543
RFPL3S	0.9589	1.0771	1.0238	0.9797	1.0099	0.0262
LOC1001343	0.8802	1.1430	1.0970	0.9193	1.0099	0.0647
LARP4	0.9695	1.2603	1.0872	0.7224	1.0099	0.1129
LOC643615	0.9907	1.1572	1.0149	0.8768	1.0099	0.0576
TMEM166	0.9416	1.0740	1.0767	0.9473	1.0099	0.0378
TSHZ2	0.9641	1.0146	1.0859	0.9750	1.0099	0.0276
XAGE3	0.9853	1.0316	1.0963	0.9264	1.0099	0.0360
TXK	0.9665	1.0675	1.0822	0.9234	1.0099	0.0386
TTLL3	0.9749	1.2619	0.9699	0.8330	1.0099	0.0902
IFT140	0.9433	1.1063	1.0403	0.9498	1.0099	0.0390
IL2RG	0.9393	1.0698	1.0447	0.9859	1.0099	0.0294
RIN2	1.1032	1.0152	1.0295	0.8920	1.0100	0.0438
LOC644826	1.0602	1.1218	1.0285	0.8293	1.0100	0.0633
GNASAS	0.9650	1.0188	1.1890	0.8671	1.0100	0.0674
AK7	0.9893	1.1446	1.0015	0.9045	1.0100	0.0498
INSL4	0.9193	1.1301	1.1034	0.8871	1.0100	0.0622
FOXD4L6	1.0121	1.1270	0.9924	0.9083	1.0100	0.0451
LOC1001340	0.6911	1.0687	1.1071	1.1729	1.0100	0.1084
IGSF21	0.9467	1.1221	1.0365	0.9346	1.0100	0.0438
ARHGDIB	0.8706	1.0434	1.0930	1.0329	1.0100	0.0483
LOC648668	1.0241	1.0163	0.9925	1.0070	1.0100	0.0068
LOC653618	0.9003	1.1376	1.0614	0.9407	1.0100	0.0546
NUMA1	1.0553	1.4919	0.8453	0.6475	1.0100	0.1809

LOC1001338	0.8857	1.5322	0.8902	0.7319	1.0100	0.1779
RPA4	0.9433	1.3030	0.9519	0.8418	1.0100	0.1008
ABCB1	0.9451	1.0050	1.1657	0.9242	1.0100	0.0546
LOC643468	0.9172	1.1138	0.9807	1.0283	1.0100	0.0414
LOC441958	0.9568	1.1189	0.9579	1.0064	1.0100	0.0381
CHRDL1	0.9388	1.0482	1.0902	0.9630	1.0100	0.0355
LOC1001332	0.6758	1.7458	1.0576	0.5609	1.0100	0.2673
CCL1	0.9473	1.1748	0.9875	0.9306	1.0100	0.0562
MGC21881	1.0022	1.1174	1.0970	0.8236	1.0100	0.0670
LOC645468	0.9949	1.0547	1.0071	0.9834	1.0100	0.0157
MSTN	0.9097	1.1666	0.9991	0.9647	1.0100	0.0554
LOC1001295	0.9539	1.1979	0.9937	0.8947	1.0100	0.0658
LOC729379	1.0458	1.0353	1.0948	0.8643	1.0101	0.0503
LYG1	0.9110	1.1891	1.0197	0.9203	1.0101	0.0646
MANBA	0.8164	1.4394	1.1636	0.6208	1.0101	0.1819
GSDMD	1.0276	1.0924	1.0510	0.8693	1.0101	0.0488
PRAMEF16	0.9640	1.0565	1.0264	0.9934	1.0101	0.0201
LOC644048	0.9697	1.0417	1.1002	0.9288	1.0101	0.0380
PTGER2	0.9991	1.1218	0.9929	0.9265	1.0101	0.0407
VPREB1	0.9993	1.1110	1.0390	0.8910	1.0101	0.0460
LOC650144	0.8570	1.1989	1.0016	0.9828	1.0101	0.0707
LOC646050	1.0941	1.0480	0.9249	0.9734	1.0101	0.0378
VWA5A	1.0408	1.2405	1.0079	0.7513	1.0101	0.1004
EMR3	1.0188	1.0365	1.0973	0.8878	1.0101	0.0441
FLJ43390	0.9815	1.1084	1.0322	0.9184	1.0101	0.0402
LOC1001331	0.9824	1.1456	1.0260	0.8864	1.0101	0.0538
LOC646041	0.9605	1.0889	1.0463	0.9447	1.0101	0.0345
TDRD7	1.0480	1.2944	1.0300	0.6681	1.0101	0.1290
LOC730760	0.9440	0.9974	1.1310	0.9682	1.0101	0.0417
LOC643720	0.9205	1.1587	0.9887	0.9726	1.0101	0.0516
LOC653319	0.9786	1.1290	1.0502	0.8828	1.0102	0.0524
LOC402560	0.9977	1.0800	1.1130	0.8499	1.0102	0.0587
LOC642936	0.9878	1.0712	0.9813	1.0004	1.0102	0.0207
APBA2	0.9361	1.0591	1.1511	0.8945	1.0102	0.0585
OR1S1	0.9437	1.0807	1.0643	0.9521	1.0102	0.0362
MAGI1	0.9455	1.0912	1.0594	0.9446	1.0102	0.0382
C4orf45	1.0147	1.0328	1.0158	0.9774	1.0102	0.0117
LOC650556	1.1197	1.0405	0.9788	0.9020	1.0102	0.0462
HLA-DRB3	0.9433	1.2059	1.0494	0.8423	1.0102	0.0777
LOC647193	0.9243	1.1347	1.0390	0.9428	1.0102	0.0485
TMEM170A	0.7985	1.4762	0.9964	0.7698	1.0102	0.1633
RBM12B	0.8757	1.2281	1.1761	0.7610	1.0102	0.1137
LOC642528	0.8413	1.1772	1.0202	1.0022	1.0102	0.0687
TLX1NB	0.9737	1.1542	1.0441	0.8690	1.0103	0.0600
HSPA12B	1.0071	1.1002	1.0642	0.8695	1.0103	0.0507
LOC1001286	0.9299	1.0559	1.0872	0.9680	1.0103	0.0368

LOC1001281	0.9202	1.1598	1.0293	0.9317	1.0103	0.0555
APC	1.0262	1.0592	1.0389	0.9168	1.0103	0.0319
ZNF625	0.8724	1.1261	1.1669	0.8757	1.0103	0.0791
RAB39	0.9737	1.1302	1.0376	0.8997	1.0103	0.0489
LOC643467	1.0023	1.0986	0.9649	0.9754	1.0103	0.0305
HLA-DRB4	0.9584	1.1631	1.0651	0.8546	1.0103	0.0666
ZNF107	0.9914	1.0742	1.0420	0.9337	1.0103	0.0307
LOC1001308	0.9349	1.1529	1.0795	0.8740	1.0103	0.0642
FAM166B	0.9286	1.2977	1.0255	0.7895	1.0103	0.1074
BMP15	0.9452	1.0501	1.1000	0.9460	1.0103	0.0387
LOC1001308	0.9306	1.0361	1.0601	1.0145	1.0103	0.0282
LOC649021	0.9908	1.0382	1.0608	0.9516	1.0103	0.0244
APCDD1	0.9600	1.1367	1.1040	0.8407	1.0104	0.0683
FGF22	0.9642	1.0530	1.1136	0.9106	1.0104	0.0452
CLCA4	1.0277	1.0928	1.0364	0.8845	1.0104	0.0444
ERVWE1	0.9958	1.1465	0.9722	0.9268	1.0104	0.0476
LOC729799	0.9549	1.1614	1.0825	0.8427	1.0104	0.0702
LOC648897	0.8321	1.0814	1.0922	1.0358	1.0104	0.0607
PFKFB2	0.9609	1.2846	0.9869	0.8091	1.0104	0.0995
DCLK2	0.9803	1.1225	1.0343	0.9043	1.0104	0.0459
LOC1001300	1.0565	0.9765	1.0771	0.9314	1.0104	0.0341
BLNK	1.0350	1.0617	1.0410	0.9039	1.0104	0.0360
FLJ35220	0.9769	1.1696	0.9606	0.9343	1.0104	0.0538
LOC392843	0.9329	1.1735	1.0419	0.8933	1.0104	0.0628
MIR548F2	0.9251	1.0034	1.0687	1.0444	1.0104	0.0315
LOC729694	1.0717	0.9631	1.0316	0.9751	1.0104	0.0253
ZNF385A	1.0012	1.1734	1.0468	0.8202	1.0104	0.0731
MIA2	0.9169	1.1468	0.9564	1.0216	1.0104	0.0503
ITGB1BP2	0.8954	1.1648	1.0928	0.8887	1.0104	0.0699
ODF2L	0.9598	1.0942	1.0814	0.9063	1.0104	0.0461
CMYA3	0.9347	1.1381	1.0631	0.9058	1.0104	0.0546
LOC1001289	0.8360	1.1313	1.1972	0.8772	1.0104	0.0902
GGN	0.9318	1.1458	0.9994	0.9648	1.0104	0.0472
TMEM83	0.9707	1.1160	1.0390	0.9161	1.0104	0.0432
SNORD58C	1.0460	1.0610	1.0717	0.8631	1.0105	0.0494
KIR3DX1	0.9716	1.0850	1.0377	0.9475	1.0105	0.0313
FLJ31132	0.8662	1.1172	1.0907	0.9678	1.0105	0.0581
LOC1001335	0.9463	1.1503	1.0020	0.9433	1.0105	0.0485
PSCA	1.0014	1.1065	1.0666	0.8674	1.0105	0.0524
DNAJB5	0.9133	1.2144	1.0417	0.8724	1.0105	0.0770
SMPD3	0.9945	1.0594	1.0564	0.9315	1.0105	0.0303
LOC1001335	0.9810	1.0461	1.0980	0.9168	1.0105	0.0393
RBMY2EP	0.9147	1.1243	0.9864	1.0165	1.0105	0.0435
C1orf165	1.0472	1.0945	1.0397	0.8605	1.0105	0.0514
TXNDC2	0.9399	1.0918	1.0615	0.9488	1.0105	0.0387
GPR151	1.0571	1.0997	0.9899	0.8953	1.0105	0.0445

FLJ32679	0.9479	1.0673	1.0709	0.9559	1.0105	0.0339
PGA4	0.9757	0.9807	1.0961	0.9896	1.0105	0.0287
MIR570	0.9116	1.1188	0.9293	1.0824	1.0105	0.0527
LOC731000	0.9705	1.0712	1.0738	0.9266	1.0105	0.0369
LRRC32	0.9681	1.1086	1.0358	0.9296	1.0105	0.0394
RORC	1.0776	1.1841	0.9484	0.8321	1.0105	0.0766
LPAL2	0.9749	1.0733	1.0476	0.9464	1.0105	0.0298
LOC441098	0.9227	1.1297	1.0871	0.9027	1.0105	0.0573
LOC1001278	0.9090	1.0618	1.1161	0.9553	1.0105	0.0475
DISC1	1.0203	1.0890	1.0248	0.9080	1.0105	0.0376
TRPC3	1.0201	1.0586	1.0867	0.8768	1.0105	0.0466
NTSR1	0.9149	1.1427	1.0398	0.9448	1.0105	0.0515
LOC1001285	0.9251	1.1035	1.0113	1.0023	1.0106	0.0365
FHOD3	1.0057	1.1808	1.0814	0.7744	1.0106	0.0865
LOC389043	0.9953	1.0995	0.9748	0.9726	1.0106	0.0301
MEP1B	0.9498	1.0883	1.0758	0.9284	1.0106	0.0416
RPSA	0.8545	1.2428	1.1923	0.7527	1.0106	0.1217
TSHR	0.9959	1.1210	1.0391	0.8863	1.0106	0.0489
LOC402176	0.9409	1.1031	0.9935	1.0050	1.0106	0.0338
AQP3	1.0619	1.1297	1.0089	0.8419	1.0106	0.0614
KRT74	0.8915	1.1154	1.0433	0.9924	1.0106	0.0471
GPR88	0.9999	1.0682	1.0170	0.9575	1.0106	0.0229
LOC643032	0.8986	1.2006	1.1279	0.8155	1.0106	0.0915
LOC650698	0.8697	1.0938	1.1304	0.9488	1.0107	0.0612
LOC650137	0.9659	1.1276	1.0076	0.9414	1.0107	0.0413
ZNF322B	0.9688	1.1424	0.9508	0.9807	1.0107	0.0443
LOC644612	0.9326	1.1835	1.0643	0.8624	1.0107	0.0712
LOC1001282	0.9624	1.1897	0.9671	0.9235	1.0107	0.0605
GPRC6A	0.9499	1.1333	0.9859	0.9736	1.0107	0.0415
LOC645463	0.9911	1.1533	0.9682	0.9302	1.0107	0.0492
LOC643740	0.9203	1.1188	1.1133	0.8904	1.0107	0.0611
SNORA2A	1.0180	1.1315	1.0262	0.8671	1.0107	0.0544
SLC22A10	0.9672	1.1338	1.0405	0.9014	1.0107	0.0499
OR2L3	0.9707	1.1489	1.0160	0.9073	1.0107	0.0512
LOC649151	0.9970	1.0718	1.0454	0.9286	1.0107	0.0315
LOC649947	0.9632	1.0713	1.1147	0.8937	1.0107	0.0504
LOC642204	0.9825	1.1659	1.0165	0.8780	1.0107	0.0595
ANAPC2	0.9463	1.1545	1.0450	0.8971	1.0107	0.0569
ITGA6	0.9304	1.1222	1.0332	0.9571	1.0107	0.0431
CLEC5A	0.9894	1.0706	0.9801	1.0028	1.0107	0.0205
LOC645929	0.9598	1.0988	1.0538	0.9305	1.0107	0.0394
FLT3LG	1.0015	1.0468	1.0744	0.9202	1.0107	0.0337
LOC646056	0.8550	1.2172	1.0072	0.9637	1.0108	0.0759
LOC644662	0.9495	1.1176	1.0642	0.9117	1.0108	0.0482
LOC651100	0.9953	1.0668	1.0409	0.9400	1.0108	0.0278
MIR362	1.0605	1.1199	0.9646	0.8981	1.0108	0.0493

CYP2B7P1	0.9758	1.1106	0.9987	0.9580	1.0108	0.0343
PIK3R4	0.7434	1.4656	1.0103	0.8239	1.0108	0.1616
WNT2	1.0406	1.0301	1.0660	0.9065	1.0108	0.0356
LOC1001294	1.0019	1.1692	1.0585	0.8135	1.0108	0.0744
LOC613038	0.8823	1.1321	0.9085	1.1202	1.0108	0.0669
ATXN8OS	1.0080	1.0183	1.0108	1.0061	1.0108	0.0027
SLC2A14	0.9901	1.1444	1.0461	0.8627	1.0108	0.0588
TMEM222	0.8834	1.2810	1.0717	0.8072	1.0108	0.1058
GBA3	1.0121	1.1034	1.0436	0.8843	1.0108	0.0462
LILRA1	1.0395	1.1045	0.9737	0.9257	1.0108	0.0390
LOC650342	1.1015	1.1040	0.9502	0.8876	1.0108	0.0546
LOC643958	0.9254	1.0798	1.1429	0.8952	1.0108	0.0598
ZNF18	1.0941	1.2237	0.9460	0.7796	1.0108	0.0957
LOC401507	0.9930	1.0476	1.0054	0.9974	1.0108	0.0125
PRRG3	1.0235	0.9570	1.1598	0.9031	1.0109	0.0554
LOC651921	0.8794	1.1120	1.0381	1.0140	1.0109	0.0485
KLRC1	0.9449	1.1231	1.0745	0.9010	1.0109	0.0525
HOM-TES-10	0.9880	1.0704	1.0774	0.9076	1.0109	0.0399
MYH6	0.8264	1.1874	1.0676	0.9621	1.0109	0.0768
CITED2	0.9842	1.2497	1.0062	0.8033	1.0109	0.0917
FAM22F	0.9353	1.2722	0.9791	0.8569	1.0109	0.0907
TTLL11	1.0476	1.0279	1.0519	0.9162	1.0109	0.0320
CHGA	1.0068	1.1348	1.0126	0.8894	1.0109	0.0501
MIR193A	0.9688	1.0734	1.0508	0.9506	1.0109	0.0301
DLC1	0.9250	1.0849	1.0199	1.0139	1.0109	0.0328
C6orf97	0.9810	1.1486	1.0263	0.8877	1.0109	0.0542
LOC646632	0.8899	1.2662	1.0421	0.8455	1.0109	0.0949
LOC1001339	1.0016	1.0654	0.9481	1.0286	1.0109	0.0247
CABP4	0.9435	1.0671	1.1421	0.8910	1.0109	0.0572
NACA	1.0053	1.1596	1.0877	0.7912	1.0110	0.0797
LOC650830	0.9505	1.0508	1.1559	0.8867	1.0110	0.0590
OBP2A	0.9429	1.1941	0.9984	0.9084	1.0110	0.0638
DNAH2	0.8713	1.1923	1.0221	0.9582	1.0110	0.0679
LOC653189	0.8886	1.1330	1.0756	0.9467	1.0110	0.0564
MIR626	1.0009	1.0856	1.0404	0.9170	1.0110	0.0358
ZSCAN12L1	0.9422	1.1384	1.0719	0.8914	1.0110	0.0570
LOC653652	0.9845	1.0341	1.1634	0.8619	1.0110	0.0624
LOC652763	0.8626	1.1024	1.0430	1.0360	1.0110	0.0516
S1PR5	1.0064	1.1091	1.0798	0.8487	1.0110	0.0583
KIR2DL1	1.0133	1.0081	1.0430	0.9796	1.0110	0.0130
FLJ43752	0.9840	1.0873	1.0466	0.9261	1.0110	0.0354
LOC1001297	0.9700	1.0070	1.1141	0.9529	1.0110	0.0362
LOC651695	1.0255	1.2199	0.9684	0.8302	1.0110	0.0808
CTNND2	0.9665	1.0672	1.0768	0.9335	1.0110	0.0359
PARK2	1.0032	1.1016	1.0619	0.8775	1.0110	0.0489
THSD7B	0.9801	1.1504	1.0167	0.8969	1.0110	0.0528

LOC441730	0.9849	1.0270	1.0388	0.9936	1.0110	0.0130
CX3CR1	1.0640	1.0870	0.9384	0.9547	1.0110	0.0377
MYH15	0.9428	1.1622	1.0726	0.8666	1.0110	0.0659
FAM184B	1.0249	1.2058	0.9064	0.9071	1.0111	0.0706
LOC1001323	0.9146	1.0756	1.1411	0.9129	1.0111	0.0577
FLJ40244	0.9689	1.1552	1.0581	0.8620	1.0111	0.0626
PLA2G2D	1.0099	1.0877	1.0602	0.8864	1.0111	0.0446
SMEK3P	0.8914	1.1889	1.0211	0.9428	1.0111	0.0650
MAFG	0.9293	1.1718	1.0247	0.9185	1.0111	0.0586
LOC727831	1.0133	1.0413	1.0533	0.9364	1.0111	0.0262
LOC1001299	1.0130	1.0485	1.1131	0.8697	1.0111	0.0515
LOC654192	0.9764	1.1187	1.0352	0.9140	1.0111	0.0436
ERCC-00083	1.0537	1.1229	0.9141	0.9538	1.0111	0.0474
LOC650116	0.8364	1.0826	1.1853	0.9400	1.0111	0.0769
LOC644278	0.9510	1.0807	1.0656	0.9472	1.0111	0.0360
C12orf72	0.9332	1.2451	0.9918	0.8744	1.0111	0.0816
MIR128-2	1.1538	1.0810	0.9931	0.8166	1.0111	0.0727
VAV3	0.9564	1.1584	0.9993	0.9304	1.0111	0.0511
LOC650459	0.9587	1.1551	1.0860	0.8448	1.0111	0.0688
LOC646917	1.0115	1.0182	1.1347	0.8801	1.0111	0.0520
MAFB	0.8699	1.4746	0.9314	0.7686	1.0111	0.1581
LOC652396	1.0240	1.1202	0.9133	0.9871	1.0111	0.0430
REV1	0.8481	1.3857	1.0872	0.7236	1.0111	0.1459
KLF17	1.0503	1.0889	0.9642	0.9412	1.0111	0.0350
OR4C16	0.9396	1.0888	1.1017	0.9146	1.0112	0.0489
LMOD1	0.8870	1.0955	1.0532	1.0090	1.0112	0.0450
RAX2	1.0198	1.1089	1.0081	0.9080	1.0112	0.0411
RPL41	0.8870	1.0912	1.1341	0.9325	1.0112	0.0600
GZMA	0.9445	1.0767	1.0123	1.0114	1.0112	0.0270
CDH8	1.0402	1.0808	1.0300	0.8939	1.0112	0.0406
LOC654155	0.9976	1.3782	0.8755	0.7936	1.0112	0.1293
NCALD	0.9545	1.1130	1.0673	0.9102	1.0112	0.0474
KRT12	1.0003	1.1668	0.9324	0.9454	1.0112	0.0539
ADCY10	1.0211	1.0038	1.0929	0.9271	1.0112	0.0340
INS-IGF2	0.9375	1.1367	1.0072	0.9636	1.0112	0.0442
LOC649749	0.9267	0.9498	1.1934	0.9751	1.0112	0.0615
PPP1R12A	1.0828	1.1918	1.0235	0.7470	1.0112	0.0947
VGLL2	0.9335	1.0917	1.0857	0.9342	1.0112	0.0447
LOC650188	0.9569	1.1074	1.0518	0.9289	1.0113	0.0415
CA1	0.9706	0.9613	0.9854	1.1278	1.0113	0.0392
LOC653313	1.0888	1.1226	0.9837	0.8499	1.0113	0.0614
FAM150A	1.0258	1.0652	1.0100	0.9441	1.0113	0.0252
FOXA3	0.9008	1.1433	1.0427	0.9584	1.0113	0.0528
SLC5A12	0.9408	1.0945	1.1005	0.9095	1.0113	0.0502
LOC730144	1.0119	1.0558	0.9963	0.9813	1.0113	0.0161
INPPL1	1.0595	1.4281	0.8262	0.7314	1.0113	0.1551

TMEM92	0.9393	1.1211	0.9809	1.0039	1.0113	0.0390
ERCC-00009	1.0830	1.0219	1.0314	0.9089	1.0113	0.0367
KIAA1467	0.8015	1.2919	1.1181	0.8339	1.0113	0.1175
TOP	0.9955	1.1440	1.0150	0.8908	1.0113	0.0519
LOC1001321	0.8790	1.1415	1.0878	0.9370	1.0113	0.0618
FAM179A	0.8922	1.1996	1.0632	0.8903	1.0113	0.0747
VPS26A	0.9602	1.4308	0.9417	0.7126	1.0113	0.1507
LOC388381	0.8112	1.1966	1.1988	0.8388	1.0113	0.1077
C2orf27B	0.9197	1.0754	1.0338	1.0164	1.0113	0.0329
CHST7	0.8214	1.1387	1.1667	0.9186	1.0113	0.0842
LOC643714	1.0657	1.0136	1.0025	0.9636	1.0113	0.0211
LOC1001345	0.9427	1.0626	1.0765	0.9637	1.0113	0.0340
MTERFD2	0.9482	1.1773	0.9912	0.9288	1.0114	0.0568
DUSP27	0.9424	1.2695	0.9812	0.8523	1.0114	0.0902
LOC651923	0.8974	1.2261	0.9856	0.9364	1.0114	0.0738
LOC402641	0.9444	1.1343	1.0507	0.9161	1.0114	0.0502
CCR3	0.9948	1.0714	0.9994	0.9800	1.0114	0.0204
CRYBA1	0.9462	1.0692	1.0430	0.9872	1.0114	0.0276
MCM9	0.8753	1.1631	1.0592	0.9480	1.0114	0.0631
LOC1001304	0.9699	1.0499	1.0598	0.9660	1.0114	0.0252
LOC647348	0.9783	1.1223	1.0851	0.8600	1.0114	0.0590
LOC646178	0.9698	1.2482	1.0014	0.8263	1.0114	0.0876
NPTXR	1.0863	1.0716	0.9750	0.9129	1.0114	0.0411
MMP2	1.0071	1.1443	1.0227	0.8717	1.0114	0.0558
LOC644571	0.9638	1.1142	0.9386	1.0291	1.0115	0.0392
SYNPO	0.8678	1.0944	1.1606	0.9231	1.0115	0.0693
TRHR	0.9332	1.1551	1.0613	0.8963	1.0115	0.0595
LOC652101	0.9095	1.1922	1.0895	0.8548	1.0115	0.0784
LOC650458	0.9972	1.2348	0.9126	0.9013	1.0115	0.0774
FAM158A	0.9082	1.3552	1.1158	0.6667	1.0115	0.1468
ZKSCAN4	0.9652	1.3791	1.0041	0.6975	1.0115	0.1402
DYDC1	0.9569	1.1071	1.0741	0.9079	1.0115	0.0472
SIAE	0.9645	1.0628	1.1129	0.9059	1.0115	0.0468
AKAP6	0.9137	1.1741	1.0506	0.9077	1.0115	0.0634
LOC402679	0.8846	1.1726	0.9380	1.0510	1.0115	0.0639
LOC649455	0.9008	1.2119	1.0303	0.9032	1.0115	0.0733
MCMDC1	0.9423	1.1057	1.0395	0.9587	1.0116	0.0379
LOC648907	0.9506	1.1807	0.9967	0.9183	1.0116	0.0586
LOC644243	0.9804	1.0692	1.0803	0.9163	1.0116	0.0388
LOC652367	1.0467	1.1206	0.9895	0.8895	1.0116	0.0487
LOC440105	0.8916	1.0263	1.1839	0.9446	1.0116	0.0638
RPL17	0.9707	1.1647	1.0282	0.8827	1.0116	0.0592
BTNL9	0.9273	1.0465	1.1219	0.9507	1.0116	0.0449
ART1	0.9369	1.1247	1.0842	0.9006	1.0116	0.0548
LOC649712	0.8819	1.1565	0.9907	1.0174	1.0116	0.0565
SPINK5L3	1.0096	1.1299	1.0186	0.8883	1.0116	0.0494

WFDC1	0.9609	1.0348	1.0840	0.9669	1.0116	0.0294
LOC648576	1.0594	1.1475	0.9771	0.8624	1.0116	0.0607
LOC1001283	1.0728	1.0751	1.0370	0.8616	1.0116	0.0508
SLC34A1	0.9831	1.2606	0.9383	0.8644	1.0116	0.0865
GPATCH1	0.8969	1.3178	0.9974	0.8345	1.0116	0.1074
LOC653425	1.0386	1.0923	1.0100	0.9055	1.0116	0.0393
NELL2	0.9887	1.0328	1.0323	0.9928	1.0116	0.0121
THBS1	0.9989	1.0211	1.0227	1.0038	1.0116	0.0060
C14orf184	0.8825	1.0842	1.1004	0.9794	1.0116	0.0507
LOC1001286	0.9844	1.0761	1.0562	0.9298	1.0116	0.0336
BAHCC1	0.9736	1.0731	1.0586	0.9415	1.0117	0.0321
LOC650889	1.0514	1.2126	1.0283	0.7544	1.0117	0.0950
CXCL12	1.0104	1.0784	1.0332	0.9248	1.0117	0.0322
WDFY4	0.9808	1.1717	0.9491	0.9452	1.0117	0.0539
SBDS	0.7986	1.1367	1.3054	0.8061	1.0117	0.1257
LOC1001290	0.8676	1.1319	1.0543	0.9930	1.0117	0.0558
MNX1	1.0296	1.2485	0.8951	0.8735	1.0117	0.0862
LOC648612	1.0288	1.0790	1.0738	0.8652	1.0117	0.0501
OR4Q3	0.8733	1.1434	1.1171	0.9130	1.0117	0.0691
LOC729135	1.0701	1.1952	0.9943	0.7872	1.0117	0.0855
PCTK2	0.9229	1.1432	1.1353	0.8454	1.0117	0.0753
DNAJB12	0.9173	1.3205	1.0204	0.7886	1.0117	0.1133
SECISBP2	0.9691	1.3206	0.9924	0.7649	1.0117	0.1149
CDRT15P	0.9048	1.1471	1.0758	0.9193	1.0117	0.0594
CRISPLD1	0.9374	1.0809	1.0696	0.9591	1.0117	0.0370
LOC1001304	0.9872	1.1014	1.0552	0.9032	1.0117	0.0431
KRTAP19-4	1.0454	1.0397	0.9970	0.9649	1.0118	0.0190
XAF1	0.8256	1.0980	1.2164	0.9071	1.0118	0.0889
ARPM2	0.9813	1.0785	1.0831	0.9042	1.0118	0.0429
MGC19604	0.9285	1.1131	1.0621	0.9433	1.0118	0.0451
REC8	0.9854	1.1362	1.0101	0.9154	1.0118	0.0461
LOC651987	0.9686	1.1163	0.9881	0.9742	1.0118	0.0351
ERCC-00053	0.9680	1.0541	1.0153	1.0098	1.0118	0.0176
LOC653082	1.0391	1.1277	1.0490	0.8315	1.0118	0.0633
FLJ27243	0.8728	1.1950	0.9632	1.0163	1.0118	0.0679
LOC728500	0.9875	1.1841	0.9414	0.9342	1.0118	0.0586
OR5M3	0.9301	1.1075	1.0455	0.9642	1.0118	0.0401
GIPR	0.9986	1.0163	1.0134	1.0189	1.0118	0.0046
NICN1	0.8104	1.4005	1.1182	0.7182	1.0118	0.1552
NCRNA0008	1.0831	1.0461	0.9323	0.9858	1.0118	0.0332
PLIN2	0.9249	1.1532	1.0139	0.9553	1.0118	0.0506
UGT1A5	0.9784	1.1090	1.0738	0.8862	1.0118	0.0502
ADNP2	0.8381	1.3406	1.2250	0.6438	1.0119	0.1631
STMN2	0.9014	1.1146	1.0793	0.9522	1.0119	0.0507
MAML3	0.9989	1.0564	1.0184	0.9739	1.0119	0.0174
LOC647960	1.0226	1.0472	1.0434	0.9344	1.0119	0.0264

LOC388849	0.9169	1.1710	1.0472	0.9125	1.0119	0.0616
BTAF1	0.9110	1.2831	1.0417	0.8119	1.0119	0.1019
SRGAP2L	0.9001	1.0901	1.1847	0.8728	1.0119	0.0752
FLJ36166	1.0620	1.0917	0.9972	0.8969	1.0119	0.0431
LOC647336	1.0295	0.9942	1.0707	0.9534	1.0119	0.0250
RPLP2	0.9793	1.1206	1.0700	0.8779	1.0120	0.0534
LOC1001323	1.0323	1.1363	0.9390	0.9403	1.0120	0.0468
FLJ43276	1.0085	1.1212	1.0618	0.8564	1.0120	0.0567
LOC646629	0.9899	0.9968	1.0582	1.0030	1.0120	0.0157
VN1R2	1.0064	1.0210	1.0254	0.9951	1.0120	0.0069
LOC653980	0.9449	1.1283	1.0348	0.9399	1.0120	0.0445
TBK1	0.9069	1.4535	0.8977	0.7898	1.0120	0.1496
LOC649632	0.9720	1.0879	0.9889	0.9992	1.0120	0.0259
TECTA	1.0338	1.0457	1.0249	0.9436	1.0120	0.0232
RPS14	0.9453	1.2585	1.0657	0.7785	1.0120	0.1011
MDH1	0.8436	1.5726	0.9682	0.6636	1.0120	0.1971
LOC643923	1.0036	1.0063	1.0545	0.9837	1.0120	0.0150
CPA2	0.9533	1.0980	1.1563	0.8406	1.0120	0.0713
LOC648596	0.9138	1.1065	1.1309	0.8971	1.0121	0.0618
SYT14	1.0450	1.1898	1.0256	0.7879	1.0121	0.0832
LOC1001293	0.9249	1.1123	1.0460	0.9652	1.0121	0.0418
LOC652439	0.9457	1.1146	1.0318	0.9563	1.0121	0.0392
ABR	1.0165	1.3686	0.9712	0.6921	1.0121	0.1388
LOC283922	0.9616	1.1436	1.0654	0.8779	1.0121	0.0582
MUC16	0.9598	1.1426	0.9850	0.9610	1.0121	0.0439
LOC1001317	1.0847	1.0155	1.0549	0.8934	1.0121	0.0420
OR4A16	1.0497	1.1106	1.0357	0.8525	1.0121	0.0556
MIR30D	0.9715	1.0593	1.1021	0.9156	1.0121	0.0421
LOC285908	0.9569	1.2175	0.9637	0.9104	1.0121	0.0695
SP140	1.0702	1.0775	0.9674	0.9334	1.0121	0.0363
ACSM2A	0.9411	1.1174	1.0127	0.9772	1.0121	0.0380
LOC644087	0.9741	1.1577	0.9716	0.9452	1.0121	0.0490
KRT26	0.9859	1.0513	1.0507	0.9607	1.0121	0.0230
KLB	0.9471	1.1113	1.0275	0.9627	1.0121	0.0374
NAALADL2	0.9977	1.0394	1.0562	0.9552	1.0121	0.0226
DGKB	0.9691	1.1458	1.1150	0.8186	1.0121	0.0752
C16orf89	0.9934	1.0999	1.0220	0.9334	1.0122	0.0346
TBX19	0.9290	1.1129	1.0749	0.9318	1.0122	0.0478
KRT18P34	0.9754	1.2077	0.9625	0.9031	1.0122	0.0670
SSH1	0.9408	1.1444	1.0371	0.9263	1.0122	0.0505
SOBP	0.9290	1.2360	1.0773	0.8064	1.0122	0.0929
PLCXD2	0.9601	1.1149	1.0510	0.9227	1.0122	0.0436
LCN12	0.8829	1.1190	1.0567	0.9901	1.0122	0.0505
LOC1001334	0.8340	1.3058	1.1298	0.7792	1.0122	0.1245
OR1A1	0.9813	1.0273	1.1361	0.9040	1.0122	0.0485
ADAMTS4	0.9076	1.0846	1.0652	0.9914	1.0122	0.0402

FUT6	0.9403	1.2177	1.0395	0.8513	1.0122	0.0785
SPANXN5	1.1011	1.0202	1.0195	0.9080	1.0122	0.0397
DGAT2	0.8964	1.1362	1.1402	0.8760	1.0122	0.0729
ST20	1.0024	1.0830	0.9892	0.9742	1.0122	0.0243
LOC1001286	0.9509	1.2069	0.9517	0.9393	1.0122	0.0650
SLFN14	0.9489	1.0719	1.1031	0.9249	1.0122	0.0442
LOC729208	0.9898	1.0519	1.2913	0.7158	1.0122	0.1183
SLC17A3	0.8728	1.1616	1.0588	0.9558	1.0122	0.0627
LOC649150	1.0430	1.0919	1.0323	0.8818	1.0123	0.0454
PPARD	0.9621	1.2105	0.9797	0.8968	1.0123	0.0685
CARD11	1.0073	1.1157	1.0219	0.9042	1.0123	0.0433
TIFAB	1.0425	1.0257	1.0683	0.9126	1.0123	0.0344
DMRTA1	0.9746	0.9638	1.1202	0.9906	1.0123	0.0364
MIR659	0.9868	1.0562	1.0666	0.9396	1.0123	0.0300
LOC727721	0.8286	1.2148	1.0845	0.9214	1.0123	0.0857
KRTAP13-4	1.0080	1.0927	0.9544	0.9940	1.0123	0.0291
LOC440053	0.9728	1.0153	1.0863	0.9748	1.0123	0.0265
LOC1001341	0.9342	1.1022	1.0693	0.9436	1.0123	0.0430
LOC1001314	0.8675	1.2494	0.9959	0.9365	1.0123	0.0833
LOC727775	0.9805	1.1663	1.0704	0.8321	1.0123	0.0711
OR2H2	0.9494	1.2218	1.0107	0.8674	1.0123	0.0757
C4orf17	0.9162	1.1311	1.0990	0.9030	1.0123	0.0598
OR2Z1	0.9197	1.2291	1.0496	0.8510	1.0124	0.0832
USP6	0.9397	1.1210	1.0910	0.8978	1.0124	0.0551
MYO7B	1.0331	1.0131	0.9753	1.0279	1.0124	0.0130
LOC653421	0.9497	1.3186	0.9763	0.8049	1.0124	0.1088
LOC647322	1.0632	1.3827	0.9075	0.6961	1.0124	0.1445
LOC653316	0.8827	1.0310	1.1058	1.0301	1.0124	0.0467
DAPP1	0.8673	1.3140	1.1114	0.7569	1.0124	0.1249
LOC647928	0.9830	1.1425	0.9406	0.9835	1.0124	0.0445
LOC650548	0.9313	1.0765	1.0577	0.9842	1.0124	0.0336
C5orf60	0.8590	1.0941	1.1291	0.9675	1.0124	0.0618
FGF8	1.0035	1.0447	1.0674	0.9341	1.0124	0.0293
KRTAP13-2	0.9558	1.0656	1.0819	0.9465	1.0124	0.0356
LOC731642	0.9848	1.0263	1.0256	1.0130	1.0124	0.0097
LOC644640	1.0322	1.1156	1.0116	0.8904	1.0124	0.0465
LOC1001327	1.0125	1.1765	1.0336	0.8272	1.0125	0.0717
LOC650193	0.9585	1.1759	1.0311	0.8845	1.0125	0.0621
NAT5	0.8593	1.5020	1.0036	0.6849	1.0125	0.1757
LOC390688	1.0433	1.0810	0.9961	0.9296	1.0125	0.0326
LOC651760	0.9615	1.0581	1.0521	0.9783	1.0125	0.0249
LOC652233	0.9838	1.0560	1.0750	0.9351	1.0125	0.0324
EPHA7	1.0474	0.9808	1.0162	1.0057	1.0125	0.0138
HHIPL1	0.9467	1.0702	1.0763	0.9568	1.0125	0.0352
DNAJC24	1.0954	1.1837	0.9742	0.7968	1.0125	0.0838
PTPRA	0.9938	1.2618	0.9721	0.8225	1.0125	0.0914

LOC1001325	0.7122	1.1131	1.1893	1.0356	1.0126	0.1049
OR2AT4	0.9733	1.2432	0.9687	0.8651	1.0126	0.0808
HMGCLL1	0.9749	1.1193	1.0452	0.9108	1.0126	0.0449
FLJ42220	0.8878	1.0017	1.1300	1.0308	1.0126	0.0498
LOC727884	1.0065	1.1128	0.9552	0.9758	1.0126	0.0350
FLJ35894	0.8824	1.1428	1.0516	0.9737	1.0126	0.0555
CICE	0.9319	1.2385	1.1498	0.7303	1.0126	0.1141
EYA2	0.9986	1.1210	1.0342	0.8967	1.0126	0.0464
DKFZp781N1	0.9358	1.2032	0.9778	0.9338	1.0126	0.0643
RARRES2	0.9653	1.0798	1.1551	0.8504	1.0127	0.0667
PIAS4	0.9127	1.3440	0.9246	0.8694	1.0127	0.1111
FLJ45139	0.9907	1.1334	1.0158	0.9108	1.0127	0.0460
LOC1001316	0.9251	1.0864	1.1477	0.8915	1.0127	0.0619
C1orf62	0.9053	1.1932	1.0246	0.9277	1.0127	0.0655
LOC643346	0.9098	1.1825	1.0105	0.9479	1.0127	0.0603
LOC1001325	0.8648	1.4574	0.9006	0.8280	1.0127	0.1490
LOC645273	0.9767	1.0234	1.0876	0.9633	1.0127	0.0281
TNFSF14	0.9928	1.1490	1.0183	0.8910	1.0128	0.0531
FAM26F	0.9352	1.3022	0.9046	0.9091	1.0128	0.0967
PAGE2B	0.8534	1.1461	1.1088	0.9429	1.0128	0.0691
ALPK2	1.0266	1.1004	0.9840	0.9402	1.0128	0.0341
STRA8	0.9443	1.0652	1.0920	0.9499	1.0128	0.0384
CCDC80	0.9654	1.1689	1.0253	0.8918	1.0128	0.0588
LOC387841	0.9283	1.2108	1.1436	0.7686	1.0128	0.1013
ANKRD47	1.0445	1.0902	1.0921	0.8246	1.0128	0.0637
CAPSL	0.9487	1.1449	1.0219	0.9359	1.0128	0.0479
LOC645953	0.9507	1.1338	1.0365	0.9305	1.0129	0.0464
FLJ45445	0.9596	1.0949	1.0484	0.9486	1.0129	0.0353
SLC13A5	1.0097	1.0768	1.0457	0.9194	1.0129	0.0340
LOC730861	1.0107	1.0757	0.9851	0.9801	1.0129	0.0220
LOC1001333	0.9398	1.1247	1.1081	0.8791	1.0129	0.0611
USP38	0.8315	1.2758	1.2484	0.6960	1.0129	0.1466
SLC28A2	0.9953	1.1305	1.0571	0.8687	1.0129	0.0555
KRT36	1.0144	1.1218	0.9860	0.9295	1.0129	0.0403
LOC643614	0.9491	1.0940	1.0601	0.9487	1.0129	0.0376
FABP1	0.8645	1.1287	1.0347	1.0239	1.0129	0.0548
C9orf109	0.9235	1.1352	1.0788	0.9143	1.0129	0.0555
MANSC1	0.9470	1.3147	1.0023	0.7878	1.0129	0.1104
LOC646549	0.8840	1.1957	1.0275	0.9447	1.0130	0.0676
GNB2L1	0.8864	1.1263	1.1373	0.9019	1.0130	0.0687
FLJ10324	1.0992	1.0602	0.9887	0.9037	1.0130	0.0430
NBLA00301	0.9701	1.2301	0.9918	0.8599	1.0130	0.0779
LOC646543	0.9417	1.0886	1.0935	0.9280	1.0130	0.0452
MIR1976	0.9763	1.0614	0.9972	1.0170	1.0130	0.0181
LOC642414	0.9805	1.1421	1.0200	0.9093	1.0130	0.0488
ZNF558	0.8674	1.2665	1.1000	0.8180	1.0130	0.1045

MIR1262	0.9550	1.1327	1.0768	0.8876	1.0130	0.0559
LOC728216	1.0743	1.1027	0.9946	0.8805	1.0130	0.0498
PRDM13	0.9486	0.9742	1.2093	0.9200	1.0130	0.0664
LOC653097	1.0683	1.0342	1.0627	0.8869	1.0130	0.0427
ZBTB43	0.9376	1.2807	0.9622	0.8716	1.0130	0.0912
SRPX2	0.9561	1.1532	0.9676	0.9753	1.0130	0.0469
PAGE5	0.9267	1.0756	1.0792	0.9707	1.0131	0.0382
RND3	1.0074	1.0380	1.0196	0.9872	1.0131	0.0107
LOC1001308	1.1255	1.0343	1.1185	0.7740	1.0131	0.0824
SLC2A8	0.8454	1.5693	0.8248	0.8128	1.0131	0.1855
OR51E2	0.9715	1.1739	0.9771	0.9298	1.0131	0.0546
FMO4	0.9608	1.2769	1.0191	0.7955	1.0131	0.0999
MIR886	0.9482	1.1145	1.0287	0.9609	1.0131	0.0381
LILRB1	0.8868	1.4660	0.9634	0.7361	1.0131	0.1582
ZC3H12B	1.0669	0.9656	1.0904	0.9293	1.0131	0.0389
PBOV1	0.9473	1.0508	1.0530	1.0013	1.0131	0.0250
C2orf57	1.0030	1.1153	0.9966	0.9374	1.0131	0.0371
FMO3	0.9879	1.0643	1.0591	0.9409	1.0131	0.0297
KIAA0495	0.8382	1.3758	0.9721	0.8663	1.0131	0.1243
LOC732040	1.0532	0.9654	1.0602	0.9736	1.0131	0.0253
AGC1	0.9550	1.0900	1.0912	0.9162	1.0131	0.0454
LOC643652	0.9400	1.0110	1.1010	1.0005	1.0131	0.0332
NFAM1	1.0362	1.0617	1.0299	0.9247	1.0131	0.0303
LOC1001345	0.9354	1.0819	1.1335	0.9018	1.0131	0.0560
RWDD2A	0.9591	1.3484	1.0486	0.6964	1.0131	0.1345
SLC22A18AS	1.0105	1.1132	1.0463	0.8826	1.0131	0.0484
ZNF774	0.8709	1.1283	1.0323	1.0211	1.0131	0.0532
WDR17	1.0091	1.0708	1.0378	0.9349	1.0131	0.0290
OR8B2	0.9655	1.2007	0.9887	0.8978	1.0132	0.0654
MIR9-3	0.9879	1.0936	1.0791	0.8921	1.0132	0.0466
PPP2R3A	0.9764	1.1298	1.0255	0.9210	1.0132	0.0443
BEGAIN	0.9523	1.1278	1.1271	0.8455	1.0132	0.0695
ANKRD1	1.0026	1.0976	1.0481	0.9043	1.0132	0.0412
ABCD1	0.8002	1.2801	1.1665	0.8059	1.0132	0.1235
LOC642342	1.0103	1.1408	0.9430	0.9586	1.0132	0.0449
TFCP2L1	0.9179	1.0519	1.1361	0.9468	1.0132	0.0501
MIR208A	0.9172	1.0455	1.0334	1.0567	1.0132	0.0324
LOC649948	0.9763	1.1087	1.0056	0.9621	1.0132	0.0331
LOC401242	0.8100	1.2256	1.1288	0.8883	1.0132	0.0980
LOC728800	1.0016	1.0279	1.0817	0.9417	1.0132	0.0291
CLDN1	0.8973	1.1250	1.1087	0.9219	1.0132	0.0601
OR2G6	0.9940	1.0605	1.0469	0.9513	1.0132	0.0251
RPL38	0.9290	1.0872	1.1189	0.9177	1.0132	0.0523
LOC646486	1.0267	1.1668	0.9007	0.9586	1.0132	0.0573
LCE3E	1.0173	1.1199	1.0181	0.8976	1.0132	0.0454
ANGPTL1	0.9799	1.0262	1.0855	0.9614	1.0132	0.0277

LOC1001324	1.0506	1.1647	0.9331	0.9045	1.0132	0.0596
DOCK9	0.9736	1.1711	1.0239	0.8844	1.0132	0.0600
TGFB3	0.9598	1.0671	1.0561	0.9700	1.0132	0.0281
TCF7L1	1.0466	1.0257	1.0252	0.9555	1.0132	0.0199
LRRC8A	0.8328	1.3241	1.1297	0.7665	1.0133	0.1303
FBXO38	1.0366	1.3332	0.9357	0.7475	1.0133	0.1223
OR2M3	0.9541	1.0471	1.0705	0.9815	1.0133	0.0273
PGPEP1	0.9827	1.1350	1.0318	0.9037	1.0133	0.0484
C8orf34	0.9291	1.0921	1.0555	0.9764	1.0133	0.0370
MGC27165	0.9583	1.1173	1.0150	0.9626	1.0133	0.0370
C8orf79	0.8938	1.1158	1.0570	0.9865	1.0133	0.0478
ELTD1	0.9466	1.1047	1.0161	0.9858	1.0133	0.0336
ZNF533	0.9929	1.1413	1.1185	0.8005	1.0133	0.0781
GRXCR1	0.9292	1.1524	1.0071	0.9647	1.0133	0.0490
HHLA3	0.9095	1.2134	1.0411	0.8893	1.0133	0.0747
KRTAP5-4	0.9739	1.0841	0.9370	1.0583	1.0133	0.0346
FBXW5	0.8436	1.3118	0.9686	0.9294	1.0133	0.1029
LOC730086	1.0051	0.9746	1.0739	0.9998	1.0133	0.0212
FLJ31222	0.9516	1.1730	1.0082	0.9207	1.0133	0.0562
LOC1001325	0.9012	1.3713	0.9432	0.8377	1.0134	0.1213
C11orf91	0.9914	1.1599	1.0674	0.8347	1.0134	0.0688
UBE2D1	0.9700	1.1204	0.9884	0.9746	1.0134	0.0359
PASD1	0.8996	1.1589	1.1346	0.8603	1.0134	0.0776
OR2W5	1.0169	1.1123	0.9830	0.9413	1.0134	0.0364
LOC1001338	0.9569	1.1287	1.0481	0.9197	1.0134	0.0470
RPL24	0.9489	1.1946	1.1059	0.8041	1.0134	0.0863
LOC728656	0.9741	1.1661	1.0529	0.8604	1.0134	0.0644
H19	0.9157	1.1319	0.9734	1.0325	1.0134	0.0461
LOC651830	0.9637	1.0183	1.1112	0.9603	1.0134	0.0352
CD6	0.8827	1.1312	1.1146	0.9251	1.0134	0.0639
LOC728093	1.0103	1.0711	1.0454	0.9268	1.0134	0.0314
TMEM211	1.0014	1.1165	1.1048	0.8309	1.0134	0.0661
SYP	1.0535	1.0366	0.9692	0.9942	1.0134	0.0193
C17orf59	0.9787	1.2405	1.0157	0.8188	1.0134	0.0869
CCDC19	1.0093	1.1207	1.0203	0.9034	1.0134	0.0444
HDAC4	0.9026	1.2301	0.9990	0.9220	1.0134	0.0752
LOC729446	0.8562	1.2413	1.0808	0.8754	1.0134	0.0914
LONP2	0.8691	1.4074	1.0326	0.7447	1.0134	0.1439
LOC646803	0.9619	1.1419	1.0371	0.9129	1.0134	0.0499
HYAL3	0.9738	1.3860	0.9120	0.7820	1.0134	0.1305
KRT14	0.9229	1.1290	1.0875	0.9144	1.0135	0.0554
VEZF1	0.9964	1.2976	0.9777	0.7821	1.0135	0.1064
LOC652905	1.0850	1.0857	0.9494	0.9336	1.0135	0.0416
LOC646617	0.9531	1.1355	1.1374	0.8278	1.0135	0.0755
ANKRD58	1.0597	1.0314	1.0267	0.9361	1.0135	0.0268
SUGT1L1	0.9547	1.2046	1.0206	0.8739	1.0135	0.0704

ERCC-00147	0.9752	0.9986	1.0316	1.0485	1.0135	0.0164
LOC645614	1.0366	1.1257	0.9994	0.8922	1.0135	0.0483
HBG2	0.9275	1.1636	1.0335	0.9293	1.0135	0.0558
ATOH1	0.9628	1.1559	0.9900	0.9452	1.0135	0.0484
LOC1001308	0.8539	1.2363	1.0605	0.9033	1.0135	0.0863
ERCC-00150	0.9373	1.1705	1.0156	0.9306	1.0135	0.0558
LCAP	0.9163	1.1690	0.9747	0.9941	1.0135	0.0544
OR10H4	1.0113	1.1126	1.0579	0.8724	1.0135	0.0514
NNMT	0.9716	1.1664	1.0581	0.8581	1.0136	0.0654
GCNT3	0.8749	1.0399	1.0348	1.1046	1.0136	0.0489
LOC653829	1.0266	1.1047	1.0734	0.8496	1.0136	0.0570
PHLDB3	0.9649	1.0749	1.0646	0.9499	1.0136	0.0326
LOC646309	0.9056	1.0143	1.1389	0.9957	1.0136	0.0480
LOC644976	0.9005	1.1416	1.0722	0.9401	1.0136	0.0563
LOC645682	0.9192	1.0392	1.1254	0.9707	1.0136	0.0446
LOC652805	0.9225	1.1772	1.0873	0.8675	1.0136	0.0718
LOC1001336	0.9815	1.3006	0.9049	0.8675	1.0136	0.0986
ACPT	0.9387	1.2134	1.0037	0.8988	1.0136	0.0700
C21orf41	0.9966	1.0820	1.0335	0.9425	1.0136	0.0295
LEAP-2	1.0093	1.0807	0.9989	0.9658	1.0137	0.0242
FZR1	0.9057	1.2665	1.0362	0.8464	1.0137	0.0931
SIRPD	0.8262	1.1402	1.1141	0.9742	1.0137	0.0723
LOC124685	1.0401	1.0404	1.0107	0.9635	1.0137	0.0181
LOC348174	0.9317	1.0628	1.0360	1.0243	1.0137	0.0285
CDSN	0.9874	1.1617	1.0085	0.8973	1.0137	0.0549
LOC339746	0.9873	1.1753	1.0097	0.8826	1.0137	0.0606
AMAC1L1	0.9663	1.0657	1.1245	0.8984	1.0137	0.0504
MIR1299	1.0474	1.0672	1.0072	0.9331	1.0137	0.0296
CCNI2	0.8473	1.3305	0.9043	0.9728	1.0137	0.1087
HHATL	1.0417	1.1894	0.8690	0.9548	1.0137	0.0683
OR52A1	1.0776	1.0452	0.9914	0.9408	1.0137	0.0301
LOC650406	0.9437	1.2777	0.9544	0.8791	1.0137	0.0895
SPPL3	1.1878	1.0991	0.9975	0.7706	1.0138	0.0899
LOC728678	1.0509	0.9900	0.9531	1.0611	1.0138	0.0256
PDCD1LG2	0.9340	1.1360	1.0816	0.9035	1.0138	0.0563
PTCD3	1.0063	1.1278	1.1271	0.7941	1.0138	0.0786
FAM135A	0.9286	1.1378	1.0666	0.9223	1.0138	0.0531
RNF170	0.9777	1.0543	1.0294	0.9938	1.0138	0.0173
LOC731605	0.8756	1.0841	1.1025	0.9930	1.0138	0.0519
LOC55908	0.9915	1.1362	1.1009	0.8266	1.0138	0.0696
GPR171	1.0147	1.0462	0.9891	1.0054	1.0138	0.0120
MIR429	0.9437	1.0858	1.0056	1.0202	1.0138	0.0292
LOC91948	0.9235	1.1038	1.1014	0.9266	1.0138	0.0513
CREBL2	0.9584	1.1555	1.1073	0.8341	1.0138	0.0731
DKFZP686E2	0.9666	1.2626	1.0672	0.7589	1.0138	0.1049
C4orf10	1.0280	1.0851	1.0481	0.8942	1.0139	0.0416

JOSD1	0.8904	1.4731	0.9447	0.7472	1.0139	0.1586
PLEKHM2	1.0322	1.2919	0.9013	0.8300	1.0139	0.1017
FLJ46010	0.9155	1.1252	0.9889	1.0259	1.0139	0.0436
LOC652570	1.0707	1.0979	0.9766	0.9102	1.0139	0.0432
TMEM146	0.9488	1.0513	1.1230	0.9324	1.0139	0.0449
SNORD124	0.9686	1.0548	1.0236	1.0085	1.0139	0.0179
LOC441964	0.9494	1.1475	1.0696	0.8891	1.0139	0.0582
FLJ40722	1.0116	1.1283	1.0290	0.8867	1.0139	0.0496
LOC400499	1.0137	1.1242	1.0839	0.8338	1.0139	0.0642
MEIS3	0.9709	1.0326	1.0741	0.9780	1.0139	0.0243
RPL18A	0.9872	1.1802	1.0419	0.8464	1.0139	0.0690
CABIN1	0.9760	1.2281	1.0108	0.8408	1.0139	0.0803
OR4C46	0.9112	1.1254	1.0555	0.9637	1.0139	0.0476
CD274	0.9868	1.1333	1.0227	0.9129	1.0139	0.0459
CLDN20	1.0549	1.0245	1.0551	0.9213	1.0139	0.0317
CALB2	0.9132	1.0629	1.1748	0.9049	1.0139	0.0648
LOC647102	1.0541	1.1575	1.0307	0.8136	1.0140	0.0722
LOC654172	0.9023	1.0996	1.1073	0.9467	1.0140	0.0525
LOC649144	0.9007	1.0337	1.2396	0.8819	1.0140	0.0824
SH2D6	1.0279	1.0261	1.1153	0.8866	1.0140	0.0473
LOC647888	0.9120	1.0998	1.0917	0.9525	1.0140	0.0479
ALOX5AP	0.9560	1.0398	1.1641	0.8961	1.0140	0.0581
USP19	0.9717	1.0822	1.1330	0.8690	1.0140	0.0589
CCR6	0.7862	1.4771	0.9744	0.8184	1.0140	0.1597
SSX5	1.0025	1.0533	1.0369	0.9633	1.0140	0.0199
ICAM2	0.9064	1.1558	1.0733	0.9205	1.0140	0.0605
FOXP4	1.0397	1.0648	0.9929	0.9588	1.0140	0.0237
ERCC-00044	1.0257	1.1713	0.9978	0.8613	1.0140	0.0635
RNF39	1.0333	1.0043	1.0777	0.9408	1.0140	0.0287
C21orf124	1.0134	1.0936	1.0554	0.8938	1.0140	0.0433
KCNRG	0.9940	1.1664	1.0359	0.8598	1.0140	0.0632
IRG1	1.0337	1.0967	0.9654	0.9604	1.0140	0.0322
SLC37A2	0.8482	1.0664	1.1118	1.0298	1.0141	0.0578
COL4A6	0.9940	1.0831	1.0663	0.9129	1.0141	0.0389
C9orf167	0.9091	1.1338	1.0787	0.9346	1.0141	0.0547
GABRP	1.0922	1.1027	0.9602	0.9012	1.0141	0.0496
NEU3	0.9359	1.2021	1.0246	0.8936	1.0141	0.0684
LOC1001302	1.0287	1.0532	1.0185	0.9559	1.0141	0.0207
LOC652722	0.8564	1.1614	1.0053	1.0332	1.0141	0.0626
LOC651892	0.9259	1.1990	1.1063	0.8251	1.0141	0.0847
LOC730098	0.8779	1.3499	0.9933	0.8353	1.0141	0.1168
LOC642855	0.9508	1.1422	0.9920	0.9714	1.0141	0.0435
GPR4	0.8897	1.0387	1.1164	1.0116	1.0141	0.0470
TMEM185A	0.8261	1.4366	0.9468	0.8470	1.0141	0.1433
LOC1001309	0.9290	1.0830	1.1337	0.9109	1.0141	0.0555
PDGFD	0.9533	1.1242	1.0824	0.8967	1.0141	0.0534

RNASEK	1.0483	1.2333	1.0478	0.7272	1.0141	0.1051
LOC401442	0.9786	1.1137	0.9900	0.9744	1.0142	0.0333
LOC645566	1.0553	1.0162	1.0328	0.9524	1.0142	0.0221
MIR1238	1.0093	1.0327	1.1025	0.9122	1.0142	0.0393
CRYM	0.9150	1.1853	1.0828	0.8737	1.0142	0.0728
MIR1257	0.9103	1.1590	1.0732	0.9144	1.0142	0.0614
SOCS7	0.9403	1.2166	1.0865	0.8134	1.0142	0.0875
UNC119B	0.8968	1.2761	1.0336	0.8504	1.0142	0.0956
LRFN2	1.0048	1.1700	0.9520	0.9301	1.0142	0.0542
MYH1	0.9595	1.0739	1.0881	0.9355	1.0142	0.0389
LOC400406	0.8974	1.1044	1.0624	0.9929	1.0143	0.0452
LOC728576	0.9295	1.1308	1.0428	0.9540	1.0143	0.0458
LOC728018	1.0210	1.0507	1.0589	0.9265	1.0143	0.0304
LOC651452	0.9231	1.1139	1.0825	0.9376	1.0143	0.0490
LOC648469	0.9885	1.1353	0.9887	0.9448	1.0143	0.0416
LOC285697	1.0127	1.1788	0.9872	0.8785	1.0143	0.0621
TRPS1	0.8354	1.1606	1.2189	0.8425	1.0143	0.1020
PCTK1	0.8889	1.1620	1.1949	0.8115	1.0143	0.0963
MED13	0.9993	1.1345	1.0068	0.9168	1.0144	0.0449
LOC1001304	0.9181	1.1117	1.1099	0.9177	1.0144	0.0557
DDX58	0.8341	1.2898	1.0090	0.9246	1.0144	0.0985
SAMD10	0.9092	1.2747	0.9901	0.8835	1.0144	0.0897
LAG3	0.9484	1.0852	0.9879	1.0361	1.0144	0.0296
MGC71993	0.8768	1.3720	1.0232	0.7855	1.0144	0.1289
UQCRQ	0.8416	1.2243	1.1567	0.8350	1.0144	0.1026
LOC648000	0.8506	1.3748	1.0290	0.8032	1.0144	0.1296
CMPK1	0.8239	1.2861	1.2310	0.7165	1.0144	0.1431
TCEB3B	1.0269	0.9684	1.1649	0.8974	1.0144	0.0567
LOC728006	0.7526	1.4311	1.0493	0.8246	1.0144	0.1526
FAT4	0.8980	1.0946	1.0955	0.9696	1.0144	0.0488
LOC1001345	0.9775	1.1721	1.0003	0.9077	1.0144	0.0561
ACRBP	1.0619	1.0859	1.0641	0.8457	1.0144	0.0565
NTRK1	0.9766	1.0373	1.0972	0.9466	1.0144	0.0334
LGALS12	1.0884	1.0609	0.9591	0.9493	1.0144	0.0353
WBP1	0.8817	1.3161	1.0901	0.7699	1.0144	0.1205
LOC643491	1.0148	1.0744	1.0370	0.9315	1.0144	0.0302
MAP3K7IP2	0.9974	1.1931	0.9893	0.8779	1.0144	0.0655
LOC648751	1.0339	1.0791	1.0519	0.8928	1.0144	0.0416
PON3	1.0687	1.0656	1.0114	0.9121	1.0144	0.0366
OR4C3	0.9699	1.0881	1.0529	0.9469	1.0144	0.0335
LOC730465	0.9487	1.1349	1.0165	0.9577	1.0144	0.0429
KCNK3	0.9833	1.2568	0.8772	0.9404	1.0144	0.0837
LOC642929	0.9426	1.1231	1.0640	0.9282	1.0144	0.0473
OR6K3	0.9595	1.1469	1.0620	0.8894	1.0145	0.0566
MYOM1	1.0257	1.1061	1.0163	0.9098	1.0145	0.0403
C10orf99	1.0405	1.0743	1.0332	0.9098	1.0145	0.0360

GYPE	0.9533	1.0940	1.0467	0.9639	1.0145	0.0337
BIN2	0.9783	1.0633	1.0605	0.9557	1.0145	0.0278
FAM70A	0.9740	1.0456	1.1950	0.8434	1.0145	0.0733
TMEM74	1.0091	0.9834	1.1026	0.9629	1.0145	0.0308
TOX4	0.9747	1.1421	1.0691	0.8721	1.0145	0.0585
CD2BP2	0.8319	1.4267	0.9713	0.8281	1.0145	0.1414
LOC644228	0.9429	1.0523	1.1646	0.8983	1.0145	0.0596
CXCR4	0.9598	1.1732	1.0317	0.8934	1.0145	0.0599
C15orf43	0.9142	1.2417	0.9936	0.9086	1.0145	0.0782
LOC255187	0.9510	1.0739	1.0085	1.0247	1.0145	0.0253
WSCD1	0.9323	1.1781	0.9408	1.0070	1.0146	0.0570
LOC652234	0.9174	1.0808	1.0728	0.9872	1.0146	0.0387
LOC442124	0.8521	1.1740	1.0353	0.9968	1.0146	0.0662
SNX10	1.1393	1.0489	1.1061	0.7640	1.0146	0.0856
FLJ25758	0.8895	1.1648	1.1763	0.8279	1.0146	0.0909
RAD51AP2	0.8909	1.1008	1.0958	0.9709	1.0146	0.0510
LOC284067	0.9762	1.0919	1.0787	0.9117	1.0146	0.0430
B3Gn-T6	0.9324	1.1085	1.0974	0.9201	1.0146	0.0511
LOC1001333	0.9735	1.2220	0.8921	0.9709	1.0146	0.0717
LOC1001316	0.9538	1.0156	1.0342	1.0549	1.0146	0.0218
ECEL1	0.9421	1.1951	1.0212	0.9001	1.0146	0.0652
ZNF806	0.9780	1.0802	1.0587	0.9417	1.0146	0.0328
MARK2	0.9616	1.1912	1.0095	0.8963	1.0147	0.0633
LOC1001341	1.0394	1.1058	1.0191	0.8943	1.0147	0.0442
TUBA3E	0.7673	1.5464	0.9956	0.7494	1.0147	0.1859
HIST3H2A	0.8574	1.4419	0.9452	0.8142	1.0147	0.1450
C10orf54	0.9901	1.0939	1.0257	0.9489	1.0147	0.0307
MGC50722	0.8981	1.2725	1.0294	0.8587	1.0147	0.0934
OR2T27	1.0285	0.9626	1.0620	1.0057	1.0147	0.0209
PCGEM1	0.9699	1.0431	1.0732	0.9727	1.0147	0.0258
RRBP1	0.8753	1.4905	0.9417	0.7513	1.0147	0.1634
LOC647691	0.9767	1.1381	1.0953	0.8488	1.0147	0.0650
LOC1001330	0.9344	1.0979	1.0116	1.0149	1.0147	0.0334
ANGPTL6	0.9489	1.1971	0.9966	0.9162	1.0147	0.0630
GALNT6	1.0603	1.0779	1.0056	0.9151	1.0147	0.0366
LOC654433	1.0179	1.0881	1.0163	0.9368	1.0148	0.0309
SLITRK1	0.9955	1.0470	1.0515	0.9650	1.0148	0.0209
INDO	0.9866	1.0214	1.0874	0.9637	1.0148	0.0270
LOC441204	0.9510	1.1467	1.0564	0.9049	1.0148	0.0542
METTL8	1.0665	1.0422	0.9911	0.9593	1.0148	0.0243
FZD7	0.9151	1.2671	0.9949	0.8819	1.0148	0.0874
SLC12A6	0.9609	1.1799	1.0132	0.9052	1.0148	0.0593
LOC554207	0.9709	1.0186	1.1562	0.9134	1.0148	0.0518
SNORA39	0.9877	1.1250	1.0381	0.9083	1.0148	0.0454
ALS2CR8	1.0403	1.1653	0.8824	0.9712	1.0148	0.0597
LOC642426	1.0131	1.2134	0.9338	0.8989	1.0148	0.0704

LOC649041	1.0005	1.1800	0.9830	0.8957	1.0148	0.0597
DEFB135	0.9396	1.1300	0.9836	1.0061	1.0148	0.0408
OR52B2	0.9310	1.1101	0.9656	1.0525	1.0148	0.0408
LOC645194	0.9614	1.1071	0.9391	1.0517	1.0148	0.0392
C21orf114	1.0138	0.9826	1.1215	0.9414	1.0148	0.0385
LOC647415	0.9020	1.1170	0.9975	1.0428	1.0148	0.0450
LOC649701	1.0169	1.0777	1.0263	0.9385	1.0148	0.0287
ZNF212	0.9020	1.2760	1.0484	0.8330	1.0149	0.0980
SLC7A8	0.9798	1.1518	1.0194	0.9084	1.0149	0.0511
APCS	0.9188	1.0688	1.1380	0.9339	1.0149	0.0531
LOC642851	1.0654	1.0464	1.0149	0.9328	1.0149	0.0293
LOC387867	0.8654	1.2743	1.1351	0.7847	1.0149	0.1144
LOC652435	1.0452	1.1001	1.0216	0.8926	1.0149	0.0440
SDK1	0.9085	1.1538	1.0927	0.9047	1.0149	0.0638
VDAC2	0.9151	1.0816	1.0395	1.0235	1.0149	0.0355
C5orf53	0.9724	1.2864	1.0200	0.7809	1.0149	0.1042
STATH	0.9597	1.1538	1.0330	0.9132	1.0149	0.0525
LOC653382	0.9762	1.1519	1.0472	0.8846	1.0150	0.0565
SLC32A1	1.0210	1.1059	1.0561	0.8767	1.0150	0.0493
EFHD1	0.9856	1.0630	1.0742	0.9371	1.0150	0.0326
LOC389286	0.9292	1.3324	0.9594	0.8388	1.0150	0.1089
LOC648674	0.9653	1.2423	1.0249	0.8275	1.0150	0.0863
LOC1001320	0.9706	1.1317	1.0022	0.9553	1.0150	0.0401
LOC645527	1.0050	1.0332	1.0780	0.9438	1.0150	0.0281
LOC391770	0.9830	1.1415	1.0061	0.9293	1.0150	0.0451
LOC1001283	0.9526	1.0693	1.0972	0.9409	1.0150	0.0399
C21orf71	0.9014	1.1015	1.1402	0.9169	1.0150	0.0617
SLC2A1	0.8290	1.5155	0.9499	0.7656	1.0150	0.1712
CACNA1I	0.9605	1.1076	1.0473	0.9447	1.0150	0.0382
LOC652692	0.9266	1.1371	1.0642	0.9321	1.0150	0.0516
LOC389405	0.9075	1.3184	1.1245	0.7097	1.0150	0.1319
SCARNA15	0.9613	1.0225	1.1086	0.9677	1.0150	0.0341
LOC730426	0.8735	1.1261	1.0331	1.0275	1.0150	0.0523
LOC642008	1.0107	1.0787	1.0915	0.8792	1.0150	0.0486
LOC644876	0.9488	1.1218	1.0060	0.9836	1.0150	0.0375
DIRC1	1.0317	1.0797	1.0328	0.9159	1.0150	0.0349
FLJ23865	0.9730	1.0379	1.0219	1.0275	1.0151	0.0144
LOC1001347	1.0037	1.1361	1.0296	0.8910	1.0151	0.0503
LOC441233	0.9769	1.1463	1.0448	0.8922	1.0151	0.0538
MIR548A1	0.9583	1.0701	1.0559	0.9760	1.0151	0.0280
MGC44328	0.9642	1.2031	1.0283	0.8647	1.0151	0.0711
LOC728667	0.8568	1.0792	1.1115	1.0128	1.0151	0.0566
COPA	0.8559	1.3615	1.1170	0.7258	1.0151	0.1413
LOC388397	0.9404	1.2406	1.0191	0.8604	1.0151	0.0819
PSMD4	0.9287	1.5038	0.8752	0.7529	1.0151	0.1670
LOC283157	0.9216	1.0835	1.1297	0.9258	1.0151	0.0536

LOC440434	0.9814	1.0800	1.1172	0.8819	1.0151	0.0528
LOC729684	0.9251	1.1161	1.0963	0.9231	1.0151	0.0527
CST9L	0.9793	1.0376	0.9911	1.0526	1.0152	0.0177
MIR323	0.9885	1.0800	1.0288	0.9633	1.0152	0.0255
LOC644255	1.0105	1.0877	0.9778	0.9847	1.0152	0.0252
TNS3	1.0338	1.0595	1.0224	0.9450	1.0152	0.0246
LOC652819	0.8793	1.1416	1.0963	0.9434	1.0152	0.0620
ZNF512	0.9217	1.3548	0.9823	0.8019	1.0152	0.1192
ECHDC1	0.9760	1.1328	0.9550	0.9968	1.0152	0.0401
LOC392871	0.9139	1.3554	1.0065	0.7848	1.0152	0.1222
LOC1001332	0.9034	1.1858	1.0604	0.9110	1.0152	0.0674
PGM1	0.8713	1.4744	1.0215	0.6935	1.0152	0.1671
OGT	0.8712	1.3921	1.0185	0.7789	1.0152	0.1350
SNORD101	0.9426	1.1467	1.0514	0.9200	1.0152	0.0524
CLEC4G	0.8966	1.0649	1.0676	1.0316	1.0152	0.0404
LRP1	1.0058	1.1294	1.0407	0.8849	1.0152	0.0506
LOC644719	0.9690	1.0807	1.0760	0.9351	1.0152	0.0371
INHBC	0.9002	1.1251	1.0105	1.0251	1.0152	0.0460
CLIC5	1.0056	1.0278	1.0539	0.9737	1.0152	0.0170
PNPLA5	0.8612	1.1314	0.9831	1.0852	1.0152	0.0600
LOC1001347	0.9642	1.0538	1.0766	0.9663	1.0152	0.0292
LOC647890	1.0478	1.1196	1.0674	0.8261	1.0152	0.0648
FLJ40852	1.0589	1.2650	0.8706	0.8664	1.0152	0.0946
ZDHHC22	0.9727	1.0544	1.1650	0.8689	1.0152	0.0627
SCAND3	0.9997	1.2628	0.9394	0.8590	1.0152	0.0874
ABCC1	1.0675	1.2819	0.8874	0.8242	1.0153	0.1027
ZNF574	1.0289	1.0237	1.0299	0.9785	1.0153	0.0123
LOC1001329	0.9903	1.0452	1.0680	0.9576	1.0153	0.0252
LOC729971	1.0200	1.0197	1.0345	0.9869	1.0153	0.0101
LOC647595	0.9126	1.1268	1.0593	0.9625	1.0153	0.0480
CRIP1	1.0071	1.1254	1.0338	0.8950	1.0153	0.0474
HK3	0.9761	1.0974	1.0329	0.9549	1.0153	0.0319
LOC653606	1.0256	1.0340	1.0747	0.9269	1.0153	0.0314
LOC389493	1.0072	1.1337	0.9403	0.9801	1.0153	0.0418
IER3	1.0119	0.9446	1.1575	0.9473	1.0153	0.0499
LOC646359	0.9620	1.0986	1.1105	0.8904	1.0154	0.0536
LHB	0.9966	1.1518	0.9937	0.9194	1.0154	0.0489
C9orf47	0.9733	1.0856	1.0904	0.9122	1.0154	0.0437
ASAH1	0.8145	1.3433	1.0751	0.8285	1.0154	0.1246
ANKRD30A	0.9833	1.0667	1.0298	0.9817	1.0154	0.0204
LOC647074	0.9291	1.1840	1.0849	0.8636	1.0154	0.0729
LOC1001321	0.8870	1.1809	1.0691	0.9247	1.0154	0.0677
LOC643011	0.9181	1.1206	1.1557	0.8673	1.0154	0.0720
PGM5P2	1.0752	1.0324	1.0357	0.9185	1.0154	0.0337
ERCC-00076	0.9610	1.0380	1.0276	1.0352	1.0154	0.0183
LOC647788	1.0301	1.1901	0.9511	0.8905	1.0155	0.0649

ZNF649	1.0175	1.0393	1.1178	0.8872	1.0155	0.0479
PPIL2	0.9631	1.1652	0.9784	0.9552	1.0155	0.0501
CMTM2	0.9986	1.0716	1.0695	0.9221	1.0155	0.0354
SNRP70	0.9221	1.0800	1.0385	1.0213	1.0155	0.0335
MIR1225	1.0292	1.1199	1.0178	0.8951	1.0155	0.0462
OPRK1	0.9814	1.2454	1.0945	0.7407	1.0155	0.1064
LOC1001323	0.8185	1.1291	1.1350	0.9794	1.0155	0.0749
FRAT1	0.9992	1.1686	1.0234	0.8708	1.0155	0.0610
LIN37	0.9118	1.0850	1.0680	0.9974	1.0155	0.0394
LOC645460	0.8686	1.2252	1.0514	0.9169	1.0155	0.0799
PPEF1	0.9510	1.1821	0.9337	0.9953	1.0155	0.0570
LOC644111	0.9416	1.0674	1.1126	0.9404	1.0155	0.0440
MIR21	1.0232	0.9965	1.0805	0.9620	1.0156	0.0250
LOC728320	0.9091	1.2484	1.0143	0.8904	1.0156	0.0823
C20orf191	0.9410	1.4202	0.9630	0.7379	1.0156	0.1441
C10orf110	0.9691	1.1861	1.0598	0.8473	1.0156	0.0716
GPRC5B	1.0086	1.1357	1.0633	0.8548	1.0156	0.0596
LOC643258	0.8641	1.0229	1.2274	0.9481	1.0156	0.0777
THBS3	1.0401	1.1716	0.9423	0.9084	1.0156	0.0590
LOC642144	0.9670	1.0690	0.9558	1.0706	1.0156	0.0314
LOC732134	1.0179	1.0647	1.0407	0.9392	1.0156	0.0272
C8orf31	0.9649	1.0769	1.0414	0.9793	1.0156	0.0263
MIR455	0.9803	1.0120	1.0481	1.0221	1.0156	0.0140
PPP1R9B	0.9873	1.0874	1.0611	0.9268	1.0156	0.0364
TBC1D13	0.9278	1.2760	0.9961	0.8627	1.0156	0.0909
LOC643081	1.0052	1.0273	0.9853	1.0449	1.0157	0.0130
LOC645863	0.9685	1.2201	1.0248	0.8493	1.0157	0.0773
LUZP5	0.9368	1.1300	1.0694	0.9265	1.0157	0.0501
PCLO	1.0378	1.0555	1.0689	0.9004	1.0157	0.0389
LOC1001322	0.9661	1.3337	0.9685	0.7944	1.0157	0.1136
SCAPER	1.0328	1.2996	0.9564	0.7740	1.0157	0.1091
LOC1001326	1.0220	1.1773	0.8954	0.9680	1.0157	0.0598
RNASE1	0.9914	1.0729	1.0546	0.9439	1.0157	0.0296
LOC651400	1.0541	1.0380	1.0749	0.8958	1.0157	0.0407
LOC644524	0.9676	1.1227	1.0676	0.9049	1.0157	0.0489
LOC648304	1.0110	1.0137	1.1286	0.9095	1.0157	0.0448
LOC646698	0.9627	1.1064	1.0220	0.9717	1.0157	0.0329
LOC1001295	1.0061	1.0785	1.0807	0.8975	1.0157	0.0430
LOC645848	0.9663	1.2596	0.8632	0.9738	1.0157	0.0851
LOC285442	0.9215	1.1702	1.1030	0.8682	1.0157	0.0720
LOC1001334	0.9655	1.1184	1.0257	0.9532	1.0157	0.0377
PRH1	0.9761	1.1085	1.0062	0.9721	1.0157	0.0319
LOC728747	1.0379	1.0576	1.0095	0.9579	1.0157	0.0217
OR51G2	1.0017	1.0455	1.0606	0.9551	1.0157	0.0238
CA10	0.9948	1.2029	0.9730	0.8923	1.0157	0.0662
CD276	0.9752	1.4845	0.9646	0.6386	1.0157	0.1747

LOC728832	1.0219	1.0743	1.0208	0.9460	1.0157	0.0264
LARP4B	0.9407	1.4353	0.7963	0.8907	1.0158	0.1430
SLC39A13	0.9317	1.1237	1.0458	0.9619	1.0158	0.0433
ESYT1	0.8285	1.4879	0.9965	0.7502	1.0158	0.1655
DAB2	0.9450	1.0680	1.0540	0.9963	1.0158	0.0282
LOC645648	0.9493	1.1722	1.1198	0.8219	1.0158	0.0803
HCRTR2	0.9148	1.0922	1.1201	0.9362	1.0158	0.0526
KLF5	0.9651	1.0317	1.1455	0.9209	1.0158	0.0489
DNAH5	0.9442	1.2428	0.9518	0.9246	1.0158	0.0759
MIR199B	0.8347	1.2160	1.1232	0.8895	1.0159	0.0915
MAOB	0.9221	1.1740	1.0569	0.9104	1.0159	0.0623
LOC652173	1.0571	1.0414	1.0868	0.8782	1.0159	0.0468
POLR2D	0.8370	1.5949	0.8907	0.7409	1.0159	0.1955
LOC652197	1.0244	1.0220	1.1524	0.8648	1.0159	0.0589
KIAA0082	0.9451	1.1843	1.0508	0.8834	1.0159	0.0659
LOC644763	0.9485	1.1666	1.0799	0.8685	1.0159	0.0665
MIR135A2	1.0085	1.0369	1.0628	0.9553	1.0159	0.0230
SUFU	0.9373	1.2141	1.0080	0.9044	1.0159	0.0695
LOC121952	0.9739	1.0905	1.0810	0.9184	1.0159	0.0419
FIGF	0.8753	1.2240	0.9902	0.9742	1.0159	0.0739
TMX4	0.8659	1.3267	1.1062	0.7651	1.0160	0.1259
LOC1001304	0.9012	1.3213	0.8377	1.0037	1.0160	0.1074
EEF1A1	0.9660	1.0942	1.0779	0.9258	1.0160	0.0414
LOC1001340	1.0181	1.0510	1.0435	0.9513	1.0160	0.0227
KCNC1	1.0236	1.0223	1.0758	0.9423	1.0160	0.0275
LOC399881	0.9284	1.1275	1.0261	0.9819	1.0160	0.0422
ATP6AP1L	0.9623	1.1205	1.0920	0.8892	1.0160	0.0545
MON1B	1.0109	1.2491	1.0378	0.7662	1.0160	0.0989
LOC642418	1.0627	1.0280	1.1199	0.8534	1.0160	0.0574
LOC643750	1.0492	1.0799	1.0486	0.8864	1.0160	0.0438
MPZ	0.9233	1.1758	1.0582	0.9068	1.0160	0.0631
FPGT	1.0152	1.1031	1.0900	0.8559	1.0160	0.0568
LOC653577	0.9552	0.9950	1.0208	1.0932	1.0160	0.0290
RASSF6	0.9937	1.1176	1.0503	0.9026	1.0160	0.0455
SNHG3	0.9316	1.1265	0.9828	1.0232	1.0160	0.0413
IGSF22	0.9609	1.1089	0.9877	1.0067	1.0161	0.0324
LOC1001284	0.9573	1.0818	1.0517	0.9735	1.0161	0.0301
TP53TG1	1.0961	1.0598	1.1044	0.8040	1.0161	0.0714
SNPH	0.9896	1.0540	1.0525	0.9683	1.0161	0.0219
LOC653808	0.9863	1.1398	0.9945	0.9438	1.0161	0.0427
CALCB	0.9403	1.0697	1.0220	1.0324	1.0161	0.0272
SLITRK5	0.8995	1.0927	1.0515	1.0206	1.0161	0.0416
LOC652751	0.9001	1.1794	0.9671	1.0178	1.0161	0.0595
CHRNA4	1.0290	1.0325	0.9778	1.0251	1.0161	0.0129
LOC647163	0.9352	1.0741	1.1588	0.8963	1.0161	0.0610
ZNF501	0.9166	1.0896	1.1234	0.9348	1.0161	0.0528

LOC729487	0.8767	1.0665	1.1603	0.9610	1.0161	0.0618
CD5	0.9753	1.0778	1.0383	0.9732	1.0161	0.0255
LOC440792	0.9896	1.0464	1.0779	0.9507	1.0162	0.0285
LOC401131	0.9328	1.1023	1.1251	0.9045	1.0162	0.0568
OR9G1	0.9535	1.1487	0.9913	0.9712	1.0162	0.0448
CDH11	0.9443	1.1058	1.0615	0.9532	1.0162	0.0400
FLJ16124	0.9707	1.0754	1.0359	0.9826	1.0162	0.0243
MR1	0.9136	1.2971	0.9784	0.8756	1.0162	0.0960
LOC126987	0.9787	1.0747	1.1357	0.8757	1.0162	0.0569
OR6T1	0.9490	1.0727	1.0133	1.0299	1.0162	0.0257
LOC644026	1.1322	1.0046	1.0013	0.9267	1.0162	0.0426
C2CD3	0.9172	1.2084	1.0762	0.8630	1.0162	0.0784
C17orf50	1.0267	1.0725	1.0523	0.9134	1.0162	0.0355
LOC647171	0.9260	1.1434	1.0811	0.9143	1.0162	0.0570
TLR8	1.0165	1.0313	1.0508	0.9663	1.0162	0.0181
C1orf92	1.0421	1.0352	1.0733	0.9144	1.0162	0.0349
LOC652002	0.9800	0.9649	1.1261	0.9939	1.0162	0.0371
LOC651612	1.0736	1.0114	1.0487	0.9314	1.0163	0.0310
LOC653169	0.9960	1.1782	0.9783	0.9125	1.0163	0.0569
MSH4	0.9763	1.0988	1.0199	0.9700	1.0163	0.0297
LOC732415	1.0141	1.1341	1.0075	0.9093	1.0163	0.0460
LOC643430	0.8701	1.1765	1.0876	0.9309	1.0163	0.0704
LOC1001332	1.0661	1.0439	0.9383	1.0168	1.0163	0.0279
FLJ22675	0.9984	1.1553	0.9609	0.9506	1.0163	0.0475
SNORD112	1.0083	1.1109	1.0330	0.9130	1.0163	0.0408
FLJ41941	0.7872	1.2543	1.0917	0.9322	1.0163	0.1008
SLC6A14	0.9918	1.0715	1.0359	0.9662	1.0163	0.0233
FAR2	0.9186	1.0929	1.1619	0.8919	1.0163	0.0659
LOC642733	0.9640	1.1197	1.0204	0.9613	1.0163	0.0370
LOC1001292	0.9517	1.0634	1.0873	0.9630	1.0163	0.0345
LOC132706	1.0813	1.0367	1.0546	0.8927	1.0163	0.0422
TEKT2	0.9012	1.2086	1.0613	0.8943	1.0164	0.0748
LOC730429	0.9610	1.0611	1.0729	0.9704	1.0164	0.0294
OR10G2	1.0615	1.1229	0.9834	0.8977	1.0164	0.0488
LOC653712	0.9057	1.1311	1.0821	0.9466	1.0164	0.0537
LOC1001347	0.9368	1.1499	1.0138	0.9650	1.0164	0.0473
MIR412	1.0564	1.0425	0.9978	0.9689	1.0164	0.0202
LOC645576	0.9222	1.0144	1.2301	0.8988	1.0164	0.0755
LOC645159	0.9008	1.1756	1.1109	0.8782	1.0164	0.0746
CTXN2	0.9250	1.1391	1.0632	0.9384	1.0164	0.0514
CETP	1.0797	1.0355	1.0018	0.9487	1.0164	0.0277
ARSE	1.0545	1.0820	1.0612	0.8681	1.0164	0.0498
PDHA2	0.9706	1.1031	1.0620	0.9300	1.0164	0.0399
LOC126075	0.9290	1.2345	1.0035	0.8988	1.0164	0.0759
EEFSEC	0.9638	1.1678	1.0380	0.8963	1.0164	0.0582
TEP1	0.9834	1.1749	1.0208	0.8867	1.0165	0.0599

DKK4	0.9861	1.1380	1.0235	0.9182	1.0165	0.0460
SEC14L4	0.9491	1.1133	1.0610	0.9425	1.0165	0.0422
LOC729010	1.0527	1.1675	0.9952	0.8504	1.0165	0.0659
LOC1001306	0.9342	1.2273	0.9989	0.9055	1.0165	0.0729
XIRP2	1.0538	1.1203	0.9434	0.9484	1.0165	0.0430
LOC642846	0.9458	1.0770	1.1341	0.9091	1.0165	0.0533
ZNF470	1.0629	1.0785	1.1064	0.8182	1.0165	0.0667
GPR22	0.9588	1.0437	1.0979	0.9656	1.0165	0.0333
C19orf26	0.8535	1.0962	1.1897	0.9266	1.0165	0.0769
LOC643304	1.0575	1.1449	0.9837	0.8799	1.0165	0.0562
SGCE	0.9415	1.2509	1.0419	0.8317	1.0165	0.0892
MGC4473	1.0465	1.1816	0.9709	0.8671	1.0165	0.0662
ACTN2	0.8387	1.1972	1.0735	0.9568	1.0165	0.0770
LOC1001281	1.0185	1.0020	1.0984	0.9472	1.0165	0.0313
LOC644353	0.9444	1.1813	1.0721	0.8684	1.0165	0.0691
LOC648487	0.9591	1.1381	1.0925	0.8765	1.0166	0.0602
LOC729156	1.0121	1.1781	0.9933	0.8827	1.0166	0.0609
PARD3	1.0007	1.0396	1.0716	0.9544	1.0166	0.0253
SPTLC1	0.9470	1.3054	1.0994	0.7145	1.0166	0.1246
SAP30BP	1.0469	1.1487	0.9800	0.8907	1.0166	0.0544
HIST1H4E	0.8807	1.3534	1.0255	0.8066	1.0166	0.1211
NDUFB5	0.7893	1.3472	1.1005	0.8294	1.0166	0.1301
C4orf31	0.9190	1.0685	1.0385	1.0403	1.0166	0.0332
RPS6	0.9526	1.1663	1.1811	0.7664	1.0166	0.0984
RAB39B	0.9896	1.1660	0.9779	0.9329	1.0166	0.0513
SLC14A2	0.9099	1.0383	1.1256	0.9928	1.0166	0.0450
C21orf119	1.1300	1.2959	0.8909	0.7498	1.0166	0.1217
LOC729372	0.7393	1.2339	1.1051	0.9882	1.0166	0.1052
N4BP1	1.0320	1.3011	0.8635	0.8700	1.0166	0.1025
SLAIN2	1.0474	1.1734	1.0343	0.8115	1.0166	0.0752
POL3S	0.8618	1.1543	1.0794	0.9713	1.0167	0.0638
LOC651711	1.0344	1.0912	1.0523	0.8888	1.0167	0.0443
LOC1001321	0.9088	1.0071	1.1764	0.9744	1.0167	0.0570
NPY	0.8979	1.0938	1.0934	0.9817	1.0167	0.0476
LOC652450	1.0293	1.0315	1.1064	0.8997	1.0167	0.0429
C4orf29	0.9046	1.1365	1.0825	0.9434	1.0167	0.0552
LOC727677	0.9499	1.0804	1.1607	0.8760	1.0167	0.0639
VCX3B	0.9465	1.1079	1.0299	0.9826	1.0167	0.0349
LOC388963	1.0020	1.0592	1.0547	0.9512	1.0167	0.0254
LOC645323	0.9116	1.2025	1.0324	0.9204	1.0168	0.0677
SGCG	0.9948	1.0386	1.0474	0.9863	1.0168	0.0153
PIPOX	1.1030	1.0267	0.9913	0.9460	1.0168	0.0332
PRAMEF11	1.0092	1.0548	0.9874	1.0157	1.0168	0.0141
LSG1	0.8597	1.4573	1.0577	0.6924	1.0168	0.1647
LOC646939	0.9325	1.0553	1.1171	0.9622	1.0168	0.0424
ZBTB7C	0.9154	1.0925	1.1015	0.9578	1.0168	0.0471

SAV1	0.9088	1.1914	1.1862	0.7808	1.0168	0.1027
COMT	0.8828	1.4484	1.0125	0.7235	1.0168	0.1555
IRGQ	1.0196	1.0061	1.0981	0.9434	1.0168	0.0318
PCAF	0.8979	1.1579	1.0456	0.9658	1.0168	0.0559
ADAM29	0.9420	1.1867	1.0268	0.9117	1.0168	0.0616
TTLL1	0.9614	1.3890	0.9513	0.7656	1.0168	0.1320
TRAPPC2P1	1.0964	1.1931	1.0440	0.7338	1.0168	0.0993
SNORD113-9	0.9307	1.0845	1.1025	0.9495	1.0168	0.0446
NHLRC2	0.8392	1.4931	0.9882	0.7468	1.0169	0.1664
OR2V2	0.9580	1.1841	0.9378	0.9876	1.0169	0.0567
PMP2	0.9058	1.0958	1.1664	0.8995	1.0169	0.0675
OSAP	0.9689	1.1118	1.0456	0.9412	1.0169	0.0386
LOC652682	0.9284	1.2507	1.0507	0.8376	1.0169	0.0893
LOC642615	0.8416	1.3023	1.0061	0.9176	1.0169	0.1009
APOBEC2	0.9871	1.1318	0.9735	0.9752	1.0169	0.0384
FAM109A	1.0663	1.1989	0.9827	0.8198	1.0169	0.0794
LOC641953	0.9681	1.1806	1.0158	0.9031	1.0169	0.0593
LOC645686	0.9434	1.1534	1.0293	0.9416	1.0169	0.0499
C16orf11	0.9645	1.1711	1.0242	0.9078	1.0169	0.0566
ZNF20	0.9439	1.2837	0.9678	0.8724	1.0169	0.0912
LRRC2	0.9387	1.1950	0.9910	0.9430	1.0169	0.0605
C9orf86	0.9761	1.3452	0.9269	0.8196	1.0169	0.1142
ERLIN1	0.9655	1.2074	0.9814	0.9135	1.0169	0.0651
LOC648264	0.9837	1.1046	1.0305	0.9490	1.0170	0.0337
FAM25G	0.9624	1.1151	1.0873	0.9031	1.0170	0.0504
LOC441046	0.9502	1.1836	0.9566	0.9775	1.0170	0.0558
ACTL7A	0.9774	1.1575	1.0152	0.9178	1.0170	0.0509
LOC652141	1.0343	1.0852	0.9855	0.9630	1.0170	0.0272
LOC647519	1.0487	1.0437	1.0045	0.9712	1.0170	0.0182
KRTAP10-5	1.0258	1.1477	1.0519	0.8427	1.0170	0.0637
HYMAI	0.9801	1.0815	1.0491	0.9574	1.0170	0.0290
LOC650921	0.9117	1.1374	1.0863	0.9327	1.0170	0.0559
MIR190	0.9743	1.0405	1.1229	0.9305	1.0170	0.0419
ZFAND5	0.8405	1.3502	1.0962	0.7812	1.0170	0.1304
LMO7	0.9631	1.0788	1.0921	0.9342	1.0170	0.0400
PTCH2	0.8754	1.1370	1.0410	1.0148	1.0170	0.0540
LOC646802	0.9477	1.2093	0.9895	0.9217	1.0171	0.0656
LOC1001338	1.0105	1.0553	1.0432	0.9592	1.0171	0.0215
LOC285194	0.9553	0.9955	1.0360	1.0815	1.0171	0.0271
EIF2B2	0.8015	1.4403	1.0316	0.7949	1.0171	0.1514
LOC653264	0.9790	1.1277	1.0185	0.9431	1.0171	0.0400
GBP4	1.0695	1.1110	1.0213	0.8665	1.0171	0.0534
TREML2	0.8870	1.2002	1.0256	0.9556	1.0171	0.0673
MED19	0.9141	1.4297	0.9893	0.7352	1.0171	0.1475
LOC645490	0.9864	1.0736	1.0558	0.9527	1.0171	0.0286
ARC	0.9201	1.1146	1.0866	0.9471	1.0171	0.0489

MMPL1	0.8918	1.2702	0.9736	0.9328	1.0171	0.0860
ETAA1	0.9684	1.0753	1.0787	0.9461	1.0171	0.0349
LOC731408	1.0318	1.0748	0.9986	0.9634	1.0172	0.0237
VASP	1.0909	1.0271	1.0952	0.8554	1.0172	0.0561
SIN3B	0.9566	1.2272	1.1006	0.7843	1.0172	0.0953
SLC26A11	1.0194	1.2195	0.9832	0.8466	1.0172	0.0770
BST1	0.9980	1.0477	1.0339	0.9892	1.0172	0.0140
RNF13	0.9734	1.2261	1.1388	0.7304	1.0172	0.1090
TLR5	0.9284	1.1580	1.0462	0.9363	1.0172	0.0541
TSKS	1.0542	1.0523	1.0129	0.9495	1.0172	0.0245
AYP1p1	0.6896	1.5135	1.0858	0.7800	1.0172	0.1859
HMGB4	1.0133	1.1124	1.0312	0.9120	1.0172	0.0412
EMID2	0.9562	1.1437	1.0791	0.8900	1.0172	0.0575
KRTAP19-5	1.0184	0.9297	1.1247	0.9962	1.0172	0.0405
SNORD2	1.0179	1.0532	1.0255	0.9723	1.0172	0.0168
LOC200420	0.9803	1.0963	1.1102	0.8822	1.0173	0.0536
FMO9P	1.0071	1.0244	1.1125	0.9251	1.0173	0.0384
TMEM176A	0.9799	1.1084	1.1184	0.8624	1.0173	0.0605
C14orf121	0.8655	1.1189	1.0400	1.0447	1.0173	0.0537
XG	0.9583	1.1628	1.0390	0.9091	1.0173	0.0554
OR10V1	0.9946	1.1527	1.0739	0.8481	1.0173	0.0650
RFPL3	1.0297	1.1676	0.9839	0.8881	1.0173	0.0581
FAM36A	0.9008	1.3132	1.0997	0.7555	1.0173	0.1213
LOC1001288	0.9285	1.1371	0.9998	1.0039	1.0173	0.0435
LOC1001296	0.9443	1.1698	1.0221	0.9331	1.0173	0.0545
LOC642849	0.9290	1.0445	1.1710	0.9249	1.0173	0.0582
RIN1	0.9052	1.1731	1.0516	0.9395	1.0173	0.0606
FABP5L2	0.8809	1.1580	1.1127	0.9179	1.0174	0.0692
MASP2	0.9789	1.1523	1.0445	0.8937	1.0174	0.0546
LOC1001311	0.9351	1.0779	1.1297	0.9268	1.0174	0.0511
TIGD6	1.0151	1.1354	1.0519	0.8671	1.0174	0.0560
LOC644399	0.9396	1.1811	1.0377	0.9112	1.0174	0.0609
MIR1255A	0.9862	1.1507	0.9905	0.9423	1.0174	0.0457
LOC644962	1.0329	1.0813	1.0602	0.8952	1.0174	0.0419
WDR27	1.0107	1.1160	1.0344	0.9085	1.0174	0.0427
LAMA2	0.9238	1.1571	1.0524	0.9363	1.0174	0.0548
FARP2	1.1159	1.1804	0.9613	0.8120	1.0174	0.0825
SPRR2C	0.9989	1.1014	1.0470	0.9224	1.0174	0.0380
SLC25A16	1.0239	1.2092	1.0300	0.8066	1.0174	0.0824
PRB4	0.9541	1.0291	1.1335	0.9532	1.0174	0.0426
LOC644961	0.9607	1.0707	1.0348	1.0036	1.0174	0.0234
LOC652759	0.9241	1.1040	1.0934	0.9483	1.0175	0.0472
LOC441395	0.9709	1.0849	1.1149	0.8991	1.0175	0.0502
LOC1001294	1.0025	1.0677	1.0592	0.9404	1.0175	0.0295
SAPS1	0.9702	1.2487	1.0383	0.8128	1.0175	0.0904
LOC340156	1.0891	1.0736	0.9286	0.9787	1.0175	0.0384

MGC27121	0.9332	1.1140	1.1435	0.8792	1.0175	0.0655
NOP10	0.9045	1.3687	1.0609	0.7359	1.0175	0.1346
S100A12	0.9693	1.0910	1.0687	0.9410	1.0175	0.0367
NID2	0.9620	1.2031	0.9672	0.9377	1.0175	0.0622
NKAIN1	0.9561	1.1519	1.0124	0.9497	1.0175	0.0470
MIR296	0.9872	1.1241	1.0685	0.8903	1.0175	0.0509
LOC650776	1.0369	0.9337	1.0326	1.0669	1.0175	0.0290
HSPB2	0.9415	1.1271	1.0703	0.9313	1.0175	0.0483
LOC646644	0.9746	1.1006	1.0068	0.9882	1.0175	0.0285
SPDYC	1.0116	0.9755	1.1980	0.8851	1.0176	0.0658
UNC5CL	0.9578	1.1562	1.0285	0.9278	1.0176	0.0508
LOC646909	0.9498	1.1718	1.1573	0.7913	1.0176	0.0909
MIR340	0.9631	1.1469	1.0350	0.9253	1.0176	0.0487
LOC643406	0.9938	1.0851	1.0399	0.9516	1.0176	0.0288
DSE	0.9759	1.1121	1.0972	0.8852	1.0176	0.0536
TINF2	0.9801	1.3442	0.9154	0.8308	1.0176	0.1131
OR5K1	0.8903	1.1394	0.9685	1.0723	1.0176	0.0551
GPR42	0.9356	1.0986	1.0737	0.9626	1.0176	0.0403
DYNLL2	0.8050	1.5414	1.0341	0.6901	1.0176	0.1887
LOC652794	1.0144	1.0955	1.0858	0.8749	1.0176	0.0509
SFXN5	1.0208	1.1802	0.9601	0.9095	1.0176	0.0588
WFDC2	0.9847	1.1109	1.0158	0.9593	1.0177	0.0332
LOC642527	0.9309	1.0428	1.1646	0.9323	1.0177	0.0556
C12orf39	1.0054	1.1546	1.0242	0.8865	1.0177	0.0549
LOC643037	0.9347	1.0369	1.1641	0.9350	1.0177	0.0544
RPL15	0.9523	1.3389	1.1223	0.6573	1.0177	0.1438
ANGPTL7	0.9996	1.0601	1.0157	0.9953	1.0177	0.0148
KITLG	0.9566	1.1541	1.0923	0.8678	1.0177	0.0648
LOC390364	0.8762	1.1557	1.0705	0.9684	1.0177	0.0607
LOC440313	0.9677	1.0971	1.0524	0.9538	1.0177	0.0343
ARID3B	1.0618	1.2340	0.9326	0.8425	1.0177	0.0850
IFNA7	0.9553	1.2428	0.9965	0.8763	1.0177	0.0791
LOC645789	0.8540	1.1805	1.1008	0.9355	1.0177	0.0747
LCE4A	0.9447	1.1370	1.0758	0.9134	1.0177	0.0531
PREPL	0.8992	1.3434	1.0955	0.7328	1.0177	0.1314
NLK	0.9455	1.0854	1.1208	0.9192	1.0177	0.0501
GSTTP2	0.9835	1.1153	1.0560	0.9161	1.0177	0.0433
KCNU1	0.8821	1.1096	1.1179	0.9614	1.0177	0.0578
IL17RE	0.9562	1.1399	1.0339	0.9410	1.0177	0.0455
TMX3	1.1283	1.0372	1.0698	0.8356	1.0177	0.0636
MEX3B	1.1113	1.3011	0.9438	0.7148	1.0178	0.1246
LOC149351	0.9941	1.1764	0.9789	0.9217	1.0178	0.0551
CACNA1C	0.9762	1.1209	1.0553	0.9186	1.0178	0.0443
LOC642716	0.9565	1.0581	1.1324	0.9241	1.0178	0.0477
LOC650454	0.9997	1.1015	1.0463	0.9237	1.0178	0.0376
ALS2CL	0.9841	1.0665	1.0407	0.9797	1.0178	0.0214

LRRTM3	0.9798	1.0868	0.9929	1.0117	1.0178	0.0239
LOC644001	0.9884	1.1482	1.1256	0.8090	1.0178	0.0781
MBL1P1	0.9030	1.1736	1.0416	0.9529	1.0178	0.0593
CCNJ	0.9931	1.1026	0.9648	1.0107	1.0178	0.0298
RNU5A	0.9088	1.2062	1.0494	0.9068	1.0178	0.0711
MYO18B	0.9649	1.0398	1.1287	0.9379	1.0178	0.0428
MGC87895	0.7177	1.2062	1.4088	0.7386	1.0178	0.1723
LOC647680	0.9744	1.1194	1.0266	0.9508	1.0178	0.0374
SEC31B	1.0070	1.1557	0.9728	0.9359	1.0178	0.0482
GABRG3	1.0045	1.0583	1.0574	0.9512	1.0178	0.0255
GSX2	1.0080	1.1054	0.9968	0.9613	1.0179	0.0308
LOC340204	1.1001	1.1273	0.9421	0.9020	1.0179	0.0562
LOC1001324	1.0397	1.0627	1.0073	0.9618	1.0179	0.0219
DCTN1	0.9448	1.4414	0.9397	0.7457	1.0179	0.1486
MIR553	0.9559	1.0793	1.0400	0.9963	1.0179	0.0267
LOC651997	0.8718	1.0949	1.0664	1.0384	1.0179	0.0500
MIR1226	1.0778	1.0123	0.9933	0.9881	1.0179	0.0207
SCAMP5	0.9540	1.1599	1.0245	0.9332	1.0179	0.0512
FLRT2	0.9831	1.1077	1.0216	0.9592	1.0179	0.0326
LOC642194	0.9737	1.0080	1.0572	1.0329	1.0179	0.0178
DKK2	0.9411	1.0757	1.0619	0.9931	1.0179	0.0313
ADH7	0.9788	1.0892	1.0541	0.9497	1.0179	0.0324
ANKRD56	1.0258	1.0921	1.0918	0.8620	1.0179	0.0543
ZNF442	0.9396	1.4227	0.9833	0.7261	1.0179	0.1462
ARHGAP15	1.0488	1.1210	1.0936	0.8084	1.0180	0.0714
LOC643630	0.9308	1.1801	1.1146	0.8463	1.0180	0.0778
HERV-FRD	0.9949	1.0619	1.1003	0.9148	1.0180	0.0407
LOC145783	0.9538	1.2249	0.9584	0.9348	1.0180	0.0692
OR6N1	1.0085	1.0574	1.1080	0.8981	1.0180	0.0448
VPS13C	0.9625	1.2636	1.0405	0.8054	1.0180	0.0953
HRNR	0.8878	1.0664	1.1906	0.9272	1.0180	0.0691
OR4K13	0.9786	1.1601	1.0446	0.8888	1.0180	0.0571
LOC729495	0.9098	1.3710	1.1061	0.6851	1.0180	0.1457
ST3GAL2	0.9925	1.1926	0.9798	0.9072	1.0180	0.0611
VIP	1.0014	1.0926	1.0384	0.9396	1.0180	0.0322
CRAT	0.9975	1.1883	0.9981	0.8881	1.0180	0.0624
GPR126	0.8397	1.6006	0.8374	0.7944	1.0180	0.1945
SNORD77	0.9591	1.1064	0.9709	1.0358	1.0180	0.0339
KRT81	1.0113	1.1030	1.0046	0.9535	1.0181	0.0311
CCL4L2	1.0837	1.0883	1.0147	0.8858	1.0181	0.0472
HYAL4	1.0180	1.2038	0.9839	0.8668	1.0181	0.0699
ERCC-00034	1.0430	1.0714	1.1687	0.7894	1.0181	0.0809
LOC1001290	0.9038	1.2921	1.0128	0.8639	1.0181	0.0966
LOC643329	0.9046	1.1646	1.0955	0.9079	1.0181	0.0661
NOLC1	0.7948	1.4445	1.1761	0.6571	1.0181	0.1796
LOC646443	0.9178	1.1096	1.0249	1.0203	1.0182	0.0392

LOC646521	1.1132	1.0787	1.0303	0.8504	1.0182	0.0585
FAM177B	0.9978	1.0712	1.0436	0.9602	1.0182	0.0246
MIR376A2	0.9396	1.2402	0.9598	0.9332	1.0182	0.0742
LOC441326	0.9808	1.0486	1.1242	0.9192	1.0182	0.0441
LOC1001326	0.9578	1.3036	1.0006	0.8107	1.0182	0.1035
DPYSL2	0.9394	1.4254	0.9666	0.7415	1.0182	0.1447
OR10A2	0.9517	1.0990	1.0973	0.9249	1.0182	0.0465
THBS2	0.9008	1.2284	1.0421	0.9016	1.0182	0.0775
OR4C12	1.0007	1.1562	0.9730	0.9431	1.0182	0.0475
LOC1001348	0.9151	1.1879	1.0166	0.9533	1.0182	0.0603
TTC3L	0.9499	1.2366	0.9956	0.8909	1.0183	0.0759
LOC1001345	1.0205	1.0423	0.9661	1.0442	1.0183	0.0182
NOG	0.9248	1.0369	1.1839	0.9274	1.0183	0.0611
LOC650780	0.9090	1.2504	1.0877	0.8261	1.0183	0.0947
LRRC17	0.9558	1.1395	1.0166	0.9612	1.0183	0.0427
LOC1001316	0.9589	1.1125	1.0277	0.9740	1.0183	0.0347
LOC730952	0.8996	1.2273	0.9184	1.0279	1.0183	0.0752
LOC286260	0.9351	1.1612	1.0343	0.9426	1.0183	0.0527
SCARNA9L	1.1528	1.1040	0.9235	0.8932	1.0184	0.0646
LOC727934	0.9449	1.0999	1.1371	0.8915	1.0184	0.0593
MPEG1	0.8658	1.1542	1.0668	0.9866	1.0184	0.0613
KLHL3	1.0067	1.2158	1.0011	0.8498	1.0184	0.0752
LOC642347	1.0342	1.1299	0.9622	0.9472	1.0184	0.0417
LOC1001319	0.9925	1.1804	0.9882	0.9124	1.0184	0.0570
ROCK2	0.8053	1.5464	0.9196	0.8023	1.0184	0.1781
SCARNA20	0.9652	1.0523	1.1067	0.9493	1.0184	0.0371
LOC652326	0.8564	1.3146	1.0387	0.8639	1.0184	0.1073
C1orf204	0.8397	1.3058	1.0472	0.8810	1.0184	0.1058
LUZP1	0.7374	1.5837	1.0253	0.7273	1.0184	0.2007
CLOCK	1.0108	1.2124	0.9667	0.8838	1.0184	0.0698
NEFH	1.0528	0.9548	1.1503	0.9158	1.0184	0.0526
EGR4	0.9725	1.1346	0.9785	0.9882	1.0185	0.0389
CLDN17	0.9900	1.1190	1.0644	0.9005	1.0185	0.0474
C6orf117	1.0128	1.0242	1.0016	1.0353	1.0185	0.0073
LOC643842	0.8881	1.2310	1.0662	0.8885	1.0185	0.0823
PRO1880	0.9674	1.0985	1.0096	0.9984	1.0185	0.0281
HVCN1	0.9739	1.1162	1.0621	0.9217	1.0185	0.0436
LOC650673	0.9572	1.0805	1.1043	0.9320	1.0185	0.0432
LOC728411	0.9240	1.2549	0.9753	0.9199	1.0185	0.0798
DEFB116	0.9588	1.1007	1.0564	0.9582	1.0185	0.0358
LOC1001328	0.9219	1.1526	1.0828	0.9167	1.0185	0.0590
FLJ31813	1.0013	1.0626	1.0390	0.9712	1.0185	0.0202
LOC1001325	0.9330	1.1728	1.0504	0.9178	1.0185	0.0594
LOC388621	0.8484	1.1559	1.4383	0.6316	1.0185	0.1765
APOB48R	0.9523	1.0845	1.0361	1.0013	1.0185	0.0279
LOC440014	0.8935	1.2521	0.9646	0.9639	1.0185	0.0796

LOC286297	0.9999	1.1029	1.0326	0.9387	1.0186	0.0342
CARD6	0.9841	1.2059	0.9809	0.9033	1.0186	0.0652
C5orf32	1.0231	1.3962	0.9694	0.6856	1.0186	0.1460
LOC1001313	0.9151	1.1464	1.0657	0.9472	1.0186	0.0535
PPY2	0.9305	1.1215	1.0936	0.9288	1.0186	0.0517
LOC644452	1.1690	1.0864	0.9583	0.8608	1.0186	0.0682
MGC33407	1.0051	1.0727	1.0248	0.9718	1.0186	0.0211
IRGC	1.1099	1.1421	1.0049	0.8175	1.0186	0.0732
LIPM	1.0062	1.1760	0.8901	1.0023	1.0186	0.0590
RAB6B	1.0152	1.2657	0.9777	0.8159	1.0186	0.0930
MIR144	0.9230	1.1208	0.9960	1.0348	1.0186	0.0412
LOC645225	0.9791	1.1980	1.0298	0.8678	1.0187	0.0687
MIR627	0.9699	1.2015	0.9832	0.9200	1.0187	0.0624
ZCRB1	0.9334	1.3355	1.0489	0.7568	1.0187	0.1215
DHX40P	0.8618	1.2089	1.1580	0.8460	1.0187	0.0958
CAPS	0.9752	1.1859	1.0791	0.8346	1.0187	0.0749
DLX4	0.9546	1.1002	1.0520	0.9680	1.0187	0.0347
LOC388259	1.1539	1.0331	0.9785	0.9093	1.0187	0.0517
NCRNA0008	0.9597	1.1710	1.1259	0.8183	1.0187	0.0808
LOC1001334	1.0608	1.0789	0.9843	0.9509	1.0187	0.0305
MPND	1.0099	1.3856	0.9239	0.7555	1.0187	0.1332
SIGLEC12	1.0364	1.0686	1.0501	0.9198	1.0187	0.0336
PRAP1	1.0358	1.0977	1.0169	0.9245	1.0187	0.0358
CHRNA7	0.9974	0.9980	1.0401	1.0393	1.0187	0.0121
LOC643332	0.8704	1.2356	1.0454	0.9236	1.0187	0.0810
LOC646947	0.9104	1.0656	1.1476	0.9514	1.0188	0.0541
CNOT2	0.8264	1.7038	0.8720	0.6728	1.0188	0.2323
LOC645198	1.0748	0.9716	1.0380	0.9907	1.0188	0.0233
MGC24103	1.0089	1.0399	1.0673	0.9590	1.0188	0.0232
LOC644990	0.9837	1.1604	1.1058	0.8253	1.0188	0.0743
LOC652454	0.9195	1.1307	1.0261	0.9990	1.0188	0.0436
LOC1001298	0.9057	1.2314	1.0989	0.8394	1.0188	0.0897
LOC1001337	0.9637	1.0133	1.0757	1.0226	1.0188	0.0230
LOC644429	1.0023	1.0437	1.0477	0.9818	1.0188	0.0161
LOC651586	1.0491	1.1586	1.0120	0.8558	1.0189	0.0626
BCL11B	0.9757	1.1034	1.0873	0.9091	1.0189	0.0463
VSIG2	0.9591	1.1475	1.0705	0.8985	1.0189	0.0557
PKD2L1	1.0027	1.0835	1.0595	0.9299	1.0189	0.0342
SELL	0.9356	1.1465	1.0715	0.9220	1.0189	0.0543
ZP1	1.0301	1.1619	0.9485	0.9352	1.0189	0.0521
CYLC1	0.9322	1.0946	1.0731	0.9757	1.0189	0.0388
LOC1001332	0.9767	1.0914	1.0542	0.9534	1.0189	0.0323
TTTY22	0.9882	1.1417	1.0445	0.9013	1.0189	0.0504
LOC652797	1.0577	0.9733	0.9252	1.1195	1.0189	0.0433
HNF1A	1.0909	1.1116	0.9314	0.9419	1.0189	0.0478
CCNC	0.7364	1.5072	1.0958	0.7363	1.0189	0.1835

AFF1	0.8959	1.3646	0.9869	0.8284	1.0189	0.1197
SLED1	0.8756	1.2713	1.0159	0.9130	1.0190	0.0892
NXF4	0.9974	1.1090	1.0930	0.8766	1.0190	0.0535
LOC643949	0.7636	1.3526	1.1817	0.7781	1.0190	0.1475
C17orf86	0.9420	1.1058	1.0537	0.9745	1.0190	0.0373
LOC283202	0.9254	1.1175	1.0982	0.9348	1.0190	0.0515
OAZ1	0.9813	1.2152	1.1025	0.7769	1.0190	0.0938
FAM132A	0.9310	1.0763	1.0745	0.9942	1.0190	0.0350
LOC652115	0.9294	1.1411	1.1122	0.8935	1.0190	0.0628
CTGF	0.9437	1.2004	1.1087	0.8233	1.0190	0.0841
EPC1	0.7997	1.3835	0.9912	0.9017	1.0190	0.1276
MSTO2P	1.0009	0.9218	1.1656	0.9878	1.0190	0.0518
LOC728139	0.8933	1.4840	1.0444	0.6544	1.0190	0.1745
LOC728791	0.8949	1.1752	1.2281	0.7780	1.0190	0.1086
LOC440888	0.9515	1.0317	1.0287	1.0644	1.0190	0.0239
EPB41L5	0.9729	1.2190	1.0303	0.8540	1.0191	0.0761
LOC645378	1.0194	1.1363	1.1258	0.7947	1.0191	0.0793
TMEM132E	0.9698	1.0888	1.0903	0.9273	1.0191	0.0416
LOC390683	0.9501	1.1076	1.1430	0.8756	1.0191	0.0636
GPR113	0.9006	1.1732	1.0394	0.9631	1.0191	0.0587
LOC1001335	1.0824	1.0670	0.9874	0.9395	1.0191	0.0337
LOC400968	0.9799	1.0982	1.1300	0.8682	1.0191	0.0598
LOC1001336	0.9511	0.9477	1.1795	0.9981	1.0191	0.0547
LOC1001340	0.8749	1.1394	1.0005	1.0616	1.0191	0.0558
PNMA1	0.8840	1.2346	1.1409	0.8170	1.0191	0.1002
MTA1	1.0976	1.3597	0.8997	0.7196	1.0191	0.1373
CDH12P	0.8851	1.1384	1.0809	0.9720	1.0191	0.0564
LOC642624	1.0315	1.0643	1.0056	0.9751	1.0191	0.0190
ZNF434	1.0001	1.2849	1.0135	0.7780	1.0191	0.1038
BEAN	1.0544	1.0555	1.0313	0.9352	1.0191	0.0285
LOC158572	0.8775	1.1731	1.0822	0.9436	1.0191	0.0667
PCDHGB7	0.9847	1.1005	1.0898	0.9015	1.0191	0.0471
HIST1H3C	0.9552	1.0169	1.1701	0.9343	1.0191	0.0533
LOC648855	1.1425	0.9971	1.0372	0.8998	1.0192	0.0502
LOC646429	0.9261	1.1808	1.0429	0.9269	1.0192	0.0605
KIAA0226	0.8435	1.1677	1.1598	0.9058	1.0192	0.0844
RNASE7	0.9986	1.0503	1.0112	1.0167	1.0192	0.0110
LOC440330	0.9915	0.9290	1.1365	1.0199	1.0192	0.0435
LOC1001295	0.8805	1.1204	1.0994	0.9766	1.0192	0.0561
LOC390183	0.9982	1.0814	1.1242	0.8731	1.0192	0.0553
CTCFL	0.9469	1.0604	1.0985	0.9711	1.0192	0.0360
LOC642856	0.9971	1.0505	1.1401	0.8894	1.0193	0.0524
EDNRA	0.9546	1.1406	1.0419	0.9400	1.0193	0.0463
LOC389517	0.7767	1.4232	1.0617	0.8155	1.0193	0.1487
LOC653342	0.9032	1.2037	1.0021	0.9681	1.0193	0.0648
LOC646920	0.9793	1.1519	1.0474	0.8986	1.0193	0.0537

DSP	1.0143	1.4381	0.8514	0.7733	1.0193	0.1484
KLHDC8B	0.9802	1.4113	0.9443	0.7414	1.0193	0.1408
C3orf1	0.9020	1.2965	1.0451	0.8335	1.0193	0.1024
LOC1001343	0.9672	1.1305	1.0253	0.9541	1.0193	0.0402
LOC139542	0.9366	1.0793	1.1145	0.9468	1.0193	0.0454
SLC22A13	0.9432	1.1363	1.0572	0.9405	1.0193	0.0476
C17orf77	1.1453	1.0893	0.9849	0.8578	1.0193	0.0633
CYSLTR2	1.0738	1.0672	0.9981	0.9383	1.0193	0.0320
LOC642103	0.9484	1.0830	1.1665	0.8795	1.0193	0.0647
LOC402569	1.0133	1.1032	0.9992	0.9618	1.0194	0.0300
LOC1001345	0.8630	1.1294	1.0575	1.0275	1.0194	0.0563
LOC645638	0.9633	1.1858	1.0546	0.8738	1.0194	0.0666
LOC1001311	1.0171	1.1107	1.1790	0.7706	1.0194	0.0893
LOC1001314	0.8759	1.1324	1.1092	0.9599	1.0194	0.0612
PCDHGC3	0.9699	1.1292	1.0748	0.9035	1.0194	0.0508
LOC729412	0.9783	1.0661	1.0483	0.9848	1.0194	0.0222
PTK2	0.9451	1.3173	0.9954	0.8198	1.0194	0.1059
OR2L1P	0.9475	1.0910	1.1112	0.9278	1.0194	0.0475
LOC1001298	0.9661	1.0167	1.1214	0.9734	1.0194	0.0358
CXorf1	0.9666	1.0077	1.1943	0.9089	1.0194	0.0617
POU2F3	1.0301	1.0945	1.0335	0.9195	1.0194	0.0364
FAM99A	1.0518	1.0503	1.0610	0.9145	1.0194	0.0350
MEOX2	0.9399	1.0918	1.0603	0.9856	1.0194	0.0346
FAM73B	1.0853	1.2242	1.0146	0.7536	1.0194	0.0987
MAP3K15	0.8997	1.1418	1.1154	0.9208	1.0194	0.0634
LOC728990	1.0286	1.1669	0.9048	0.9774	1.0194	0.0553
ACCSL	0.9559	1.1531	1.0597	0.9090	1.0194	0.0545
LOC643062	0.9308	1.0464	1.1640	0.9365	1.0194	0.0551
BNIPL	1.0122	1.1114	1.0105	0.9436	1.0194	0.0346
LOC644975	1.0121	1.1458	1.0745	0.8453	1.0194	0.0642
LOC642819	0.9792	1.1263	1.0469	0.9255	1.0195	0.0434
HBP1	0.9191	1.2544	1.0933	0.8110	1.0195	0.0976
TCEB3CL2	1.0468	1.1004	1.1203	0.8103	1.0195	0.0714
SNHG12	0.9397	1.1984	1.0234	0.9164	1.0195	0.0639
CTTNBP2	0.9855	1.1206	1.0404	0.9315	1.0195	0.0404
LOC1001279	0.9667	1.0163	1.1499	0.9452	1.0195	0.0459
SNORD59B	1.0136	1.1337	1.0432	0.8876	1.0195	0.0508
KSR1	1.0031	1.0289	1.1014	0.9447	1.0195	0.0325
SNORD63	1.0114	1.0260	1.0473	0.9933	1.0195	0.0114
LOC650122	0.9291	1.1181	1.1059	0.9250	1.0195	0.0535
LOC651630	0.9729	1.1490	1.0188	0.9374	1.0195	0.0462
LOC652828	0.9566	1.0412	1.1563	0.9241	1.0195	0.0519
TNFSF12-TNI	0.8803	1.1207	1.1290	0.9483	1.0196	0.0624
LOC653600	0.9270	1.0741	1.1392	0.9381	1.0196	0.0520
OR9G4	0.9897	1.1146	1.0216	0.9524	1.0196	0.0347
LOC1001289	0.9729	1.0849	1.1276	0.8929	1.0196	0.0534

TAF11	0.9295	1.0934	1.0929	0.9626	1.0196	0.0430
LOC732316	0.9667	1.2103	1.0694	0.8320	1.0196	0.0800
SARDH	0.9109	1.0518	1.1124	1.0034	1.0196	0.0425
ATP6V0E1	0.8919	1.4292	1.1014	0.6560	1.0196	0.1641
FBXO11	0.9743	1.3054	0.9976	0.8012	1.0196	0.1049
MIR30A	0.9785	1.1510	1.0055	0.9435	1.0196	0.0456
PID1	0.8670	1.1911	1.1232	0.8972	1.0196	0.0808
ADPRHL2	0.9899	1.3378	0.9314	0.8194	1.0196	0.1118
SERPINE2	0.9612	1.3719	0.9584	0.7870	1.0196	0.1243
CYP4F22	0.9278	1.1417	1.0376	0.9713	1.0196	0.0465
LOC391165	1.0700	1.1039	1.0907	0.8138	1.0196	0.0689
ERCC-00042	0.9888	1.0836	1.0802	0.9259	1.0196	0.0382
NGEF	0.8942	1.2203	1.0166	0.9475	1.0197	0.0714
LOC645993	0.9902	1.2166	0.9677	0.9042	1.0197	0.0681
LOC732447	0.9901	1.1725	1.0222	0.8939	1.0197	0.0578
FBXW11	0.9231	1.4211	1.0077	0.7268	1.0197	0.1462
WBSCR16	0.8892	1.4549	0.9069	0.8276	1.0197	0.1461
MIR219-2	0.9454	1.1395	1.0906	0.9032	1.0197	0.0566
LOC647334	0.9431	1.0811	1.0377	1.0169	1.0197	0.0288
ASB6	1.0474	1.1320	1.0115	0.8879	1.0197	0.0507
TRPM4	0.9579	1.4248	0.9111	0.7850	1.0197	0.1399
LOC648748	1.0943	1.0349	1.0146	0.9350	1.0197	0.0329
LOC653778	0.7995	1.4496	1.0595	0.7702	1.0197	0.1574
LOC440589	1.0406	1.0769	1.0403	0.9211	1.0197	0.0340
ZDHHC8P	1.0113	1.1142	1.0480	0.9055	1.0198	0.0436
LOC1001315	0.9974	1.0993	1.0314	0.9509	1.0198	0.0312
C15orf55	0.9518	1.0997	1.0780	0.9497	1.0198	0.0401
CNP	1.0334	1.0859	1.0530	0.9067	1.0198	0.0392
LOC136157	0.9898	1.1293	1.0553	0.9047	1.0198	0.0478
LCAT	0.9375	1.0721	1.0424	1.0271	1.0198	0.0290
PDE6G	0.8795	1.2312	1.0623	0.9062	1.0198	0.0812
LOC389072	1.0051	0.9784	1.1297	0.9660	1.0198	0.0375
PA2G4P4	1.0032	1.1292	1.0265	0.9203	1.0198	0.0430
LCE2B	0.9801	1.1606	0.9585	0.9800	1.0198	0.0472
MIR593	0.9364	1.1410	1.1135	0.8883	1.0198	0.0631
DMRTC2	1.1297	1.0819	0.9272	0.9405	1.0198	0.0507
GJB7	0.8803	0.9532	1.2994	0.9464	1.0198	0.0946
PTPN12	0.9488	1.3397	1.0157	0.7752	1.0198	0.1180
LOC650924	0.8446	1.1908	1.1313	0.9127	1.0199	0.0836
MIR655	0.9293	1.1524	1.0155	0.9824	1.0199	0.0476
LAPTM4A	0.9510	1.3270	1.1756	0.6258	1.0199	0.1524
LOC1001280	1.0349	1.1134	1.0500	0.8812	1.0199	0.0493
SNRPE	0.9757	1.0818	1.0725	0.9496	1.0199	0.0336
C2orf67	1.0352	1.0565	1.0215	0.9663	1.0199	0.0193
ESR1	1.0274	1.1270	1.0290	0.8963	1.0199	0.0473
OVCH1	1.0072	1.0365	1.0819	0.9542	1.0199	0.0268

NAT8	0.9509	1.0360	1.1658	0.9271	1.0199	0.0539
KRT15	0.8573	1.1997	1.0513	0.9714	1.0199	0.0719
TKTL1	0.9195	1.2626	0.9450	0.9528	1.0200	0.0812
LOC652840	0.9046	1.0558	1.0955	1.0240	1.0200	0.0411
LOC730302	0.9023	1.0222	1.1273	1.0281	1.0200	0.0460
TNP2	0.9007	1.0659	1.1676	0.9456	1.0200	0.0603
LQK1	0.9173	1.2416	1.1439	0.7772	1.0200	0.1057
LOC390617	0.9111	1.1131	1.0736	0.9821	1.0200	0.0455
LOC1001279	1.0415	1.0922	1.0607	0.8856	1.0200	0.0460
LOC1001287	0.9530	1.1142	1.1469	0.8658	1.0200	0.0666
LOC1001308	0.9728	1.1881	1.0900	0.8292	1.0200	0.0773
LOC645073	0.9615	1.1309	1.0413	0.9463	1.0200	0.0424
C9orf71	0.9552	1.0989	1.0392	0.9868	1.0200	0.0315
STAT6	0.9943	1.0646	1.0897	0.9316	1.0200	0.0357
ZNF738	0.9767	1.3881	1.0147	0.7007	1.0200	0.1412
LOC401497	0.8111	1.1329	1.1766	0.9596	1.0200	0.0840
GPR25	1.0146	1.0481	1.0266	0.9909	1.0200	0.0119
OR52N2	1.0243	1.2437	0.8216	0.9906	1.0201	0.0867
MIR381	1.0113	1.1175	1.0258	0.9256	1.0201	0.0393
MIR197	0.9502	1.0849	1.0199	1.0253	1.0201	0.0275
UBE2L6	0.9874	1.1298	1.0110	0.9521	1.0201	0.0385
LOC1001280	0.9980	1.0858	0.9941	1.0025	1.0201	0.0220
HIST4H4	0.8778	1.2413	1.0614	0.8998	1.0201	0.0843
MIR670	1.0947	1.0533	1.0193	0.9130	1.0201	0.0389
ERCC6	0.9452	1.2884	1.0312	0.8155	1.0201	0.0998
AQP9	1.0423	1.1168	0.9800	0.9413	1.0201	0.0384
LOC728132	1.0105	1.3050	0.9571	0.8078	1.0201	0.1042
ZADH2	0.9380	1.1978	0.9104	1.0342	1.0201	0.0649
LOC650235	0.9746	1.0613	1.1223	0.9223	1.0201	0.0445
RIPK1	1.0107	1.3392	0.9405	0.7901	1.0201	0.1159
TLK2	1.0137	1.2868	0.9721	0.8079	1.0201	0.0994
LOC642956	0.9208	1.0753	1.0814	1.0031	1.0201	0.0376
LOC729809	0.9962	1.1061	0.9800	0.9983	1.0202	0.0289
ACVR2A	1.0276	1.1505	1.0266	0.8759	1.0202	0.0562
LOC391126	0.8858	1.3095	1.2759	0.6095	1.0202	0.1673
LOC648167	0.8477	1.1498	1.1247	0.9584	1.0202	0.0715
rab1c	0.9639	1.0593	1.0149	1.0425	1.0202	0.0209
FAM71D	0.9667	1.0012	1.0674	1.0455	1.0202	0.0226
PRTFDC1	1.1109	1.1197	0.9697	0.8804	1.0202	0.0579
KIAA0415	0.9481	1.1763	1.0431	0.9133	1.0202	0.0588
LOC645367	0.9944	1.0655	1.0864	0.9345	1.0202	0.0347
CST7	1.0570	1.0939	0.8612	1.0688	1.0202	0.0536
C10orf116	0.9733	1.1559	1.0379	0.9138	1.0202	0.0518
LOC650781	0.9141	1.1460	1.1490	0.8720	1.0202	0.0740
LOC1001301	0.8897	1.2144	1.1127	0.8643	1.0203	0.0854
ABCG2	0.9007	1.1278	1.0642	0.9882	1.0203	0.0490

LIPT1	0.9637	1.2240	1.0860	0.8074	1.0203	0.0887
LOC441368	0.9911	1.1130	1.0196	0.9573	1.0203	0.0334
NDRG1	1.0572	1.5716	0.7292	0.7231	1.0203	0.1997
C6orf127	0.9662	1.0634	1.0724	0.9790	1.0203	0.0277
LOC649429	0.9344	1.0037	1.1495	0.9935	1.0203	0.0457
LOC732313	0.9827	1.0237	1.0768	0.9979	1.0203	0.0207
LOC284260	0.9525	1.0943	1.1713	0.8630	1.0203	0.0693
LOC653832	0.9538	1.1168	1.0804	0.9302	1.0203	0.0461
LOC644766	0.9582	1.0597	1.1269	0.9364	1.0203	0.0446
CIT	0.9767	1.1082	1.0354	0.9609	1.0203	0.0334
NUDT16	0.9313	1.3649	1.0664	0.7186	1.0203	0.1353
FBXL2	0.8960	1.2462	1.0646	0.8744	1.0203	0.0865
CD93	0.9996	1.1309	0.9953	0.9555	1.0203	0.0382
C7orf4	1.0604	1.1407	0.9908	0.8893	1.0203	0.0533
OS9	0.9389	1.2835	1.0820	0.7769	1.0203	0.1076
LOC648226	1.0504	1.1320	0.9958	0.9031	1.0203	0.0481
MIR641	0.8006	1.2138	1.1140	0.9528	1.0203	0.0909
KCNN3	0.9819	1.1082	1.0483	0.9429	1.0203	0.0365
LOC1001340	0.9595	1.2011	1.0708	0.8498	1.0203	0.0753
C14orf165	1.0064	1.1012	1.1199	0.8539	1.0203	0.0608
LOC1001296	0.9522	1.2188	1.2500	0.6604	1.0204	0.1373
LOC651787	0.9577	1.1668	1.0459	0.9111	1.0204	0.0562
LOC1001313	0.9409	1.1328	1.0461	0.9619	1.0205	0.0438
LOC1001325	0.9343	1.2367	0.9871	0.9237	1.0205	0.0734
MBD3L5	1.1234	1.1243	1.0513	0.7828	1.0205	0.0810
MIR1297	0.8520	1.1116	1.0860	1.0323	1.0205	0.0585
LOC644456	0.9092	1.1237	1.0741	0.9748	1.0205	0.0483
LOC644629	0.8717	1.2686	0.9679	0.9738	1.0205	0.0859
KIAA0776	0.9115	1.1257	1.1121	0.9327	1.0205	0.0570
DLGAP4	0.9176	1.2181	1.0807	0.8658	1.0205	0.0802
ZNF627	1.0361	1.2478	0.9974	0.8009	1.0206	0.0916
LOC644298	1.0467	1.1665	1.0406	0.8285	1.0206	0.0703
LOC652537	0.9576	1.2072	0.9457	0.9717	1.0206	0.0624
CCDC85A	0.9774	1.0801	1.0774	0.9474	1.0206	0.0341
LOC645676	0.9900	1.2332	1.0406	0.8185	1.0206	0.0853
CYP4F12	0.9986	1.1454	0.9965	0.9420	1.0206	0.0436
WNT5B	0.9600	1.1090	1.0691	0.9443	1.0206	0.0405
LOC1001318	0.9659	1.2578	1.0011	0.8577	1.0206	0.0847
LOC285176	0.9649	1.1596	1.1247	0.8333	1.0206	0.0755
FLJ46026	1.0671	1.0969	1.0233	0.8951	1.0206	0.0445
SNORA37	0.9283	1.0932	1.0647	0.9963	1.0206	0.0369
LOC643906	0.9966	1.0841	1.0910	0.9108	1.0206	0.0424
HEXIM2	0.8349	1.4154	1.0039	0.8284	1.0206	0.1377
TNRC18	0.8695	1.2592	1.0474	0.9064	1.0206	0.0883
LOC1001312	0.8919	1.2405	0.9819	0.9683	1.0206	0.0759
MIR26A1	1.0205	0.9715	1.0789	1.0116	1.0206	0.0222

C17orf102	1.0119	1.1024	1.0257	0.9425	1.0206	0.0328
SYCP2L	0.9533	1.1654	1.0134	0.9505	1.0207	0.0504
OR13J1	0.9633	1.0411	1.0880	0.9902	1.0207	0.0276
IFP38	0.8147	1.3210	1.2665	0.6805	1.0207	0.1604
DNAJC5B	0.9258	1.0554	1.1541	0.9473	1.0207	0.0527
KRT17	1.1194	1.0574	1.0085	0.8973	1.0207	0.0470
IL10RA	1.0214	1.1333	0.9420	0.9860	1.0207	0.0409
LOC648130	0.9368	1.1526	1.0390	0.9542	1.0207	0.0493
ZNF385B	0.9152	1.1555	1.2541	0.7579	1.0207	0.1129
LOC440414	0.9213	1.0427	1.1229	0.9957	1.0207	0.0423
LOC647147	0.9948	1.1836	1.1320	0.7723	1.0207	0.0919
LOC653665	1.0417	1.1274	0.9782	0.9356	1.0207	0.0417
LOC648913	1.0105	1.0580	1.1182	0.8961	1.0207	0.0470
LOC1001329	0.9203	1.1763	0.9996	0.9867	1.0207	0.0547
LOC647589	0.9281	1.1696	0.9858	0.9995	1.0207	0.0520
MIR892A	0.9848	1.0003	1.1164	0.9815	1.0208	0.0321
PHPT1	0.9936	1.2877	1.0735	0.7282	1.0208	0.1156
LAMA3	0.9064	1.2374	1.0996	0.8397	1.0208	0.0908
IFI35	1.0204	1.3800	1.0436	0.6390	1.0208	0.1515
LOC650831	0.9490	1.0845	1.0849	0.9646	1.0208	0.0371
FBXO47	1.0534	1.1143	0.9640	0.9514	1.0208	0.0386
LOC653137	0.9405	1.1102	1.1240	0.9084	1.0208	0.0561
ERCC-00160	1.0053	1.1138	0.9976	0.9665	1.0208	0.0321
LOC653154	0.9321	1.1137	1.0361	1.0012	1.0208	0.0378
MIR24-1	1.0352	1.0862	1.0359	0.9258	1.0208	0.0338
KIAA1841	1.0058	1.0534	1.0845	0.9395	1.0208	0.0316
IFI44	0.9425	1.1756	1.1878	0.7773	1.0208	0.0988
LOC652525	1.0791	1.0627	1.0264	0.9150	1.0208	0.0369
LOC644151	0.9702	1.0224	1.0015	1.0893	1.0208	0.0252
ZNRF4	1.0192	1.1198	0.9876	0.9569	1.0208	0.0354
SNORD44	0.9859	1.1445	0.9884	0.9646	1.0208	0.0416
LOC389457	0.9941	1.0731	1.1290	0.8872	1.0209	0.0524
LOC644773	0.9294	1.1168	1.0628	0.9744	1.0209	0.0423
CCDC69	1.0193	1.1333	1.0864	0.8444	1.0209	0.0633
TMEM30B	0.9093	1.0968	1.0321	1.0453	1.0209	0.0397
LOC1001319	0.6565	1.1859	1.5275	0.7137	1.0209	0.2064
LOC1001280	0.9726	1.1553	0.9965	0.9592	1.0209	0.0455
OAS2	0.9977	1.0824	1.0688	0.9347	1.0209	0.0342
LILRB5	0.9819	1.1989	0.9963	0.9066	1.0209	0.0625
YIPF2	0.8548	1.2167	1.0766	0.9355	1.0209	0.0798
LOC647645	1.0443	1.1239	0.9531	0.9624	1.0209	0.0400
SLC38A5	1.0792	1.1387	1.0057	0.8601	1.0209	0.0601
LOC649548	1.0059	1.1836	1.1861	0.7082	1.0209	0.1124
LOC645104	0.9532	1.0584	1.1023	0.9699	1.0209	0.0356
LOC644929	0.9678	1.1968	1.0555	0.8636	1.0209	0.0705
CCDC144C	1.0908	1.0427	1.0355	0.9148	1.0209	0.0374

LOC645877	0.9985	1.2468	0.9667	0.8719	1.0210	0.0799
LOC646010	1.0259	1.0464	1.1034	0.9081	1.0210	0.0410
LOC649826	0.9734	1.2581	0.9479	0.9046	1.0210	0.0803
LOC1001313	1.0184	0.9498	1.2155	0.9003	1.0210	0.0692
LOC648251	1.0518	1.1044	0.9958	0.9320	1.0210	0.0371
LOC1001346	1.0957	1.0689	1.0668	0.8526	1.0210	0.0565
LOC646127	0.9198	1.1474	1.1532	0.8636	1.0210	0.0756
LOC644371	0.9952	0.9825	1.1281	0.9782	1.0210	0.0359
TERF1	0.8772	1.1654	1.2247	0.8168	1.0210	0.1019
UBR1	0.9818	1.2038	1.0677	0.8308	1.0210	0.0782
SNORD11B	0.8552	1.2076	1.0671	0.9544	1.0210	0.0758
CSGALNACT1	1.1310	0.9834	1.0663	0.9034	1.0210	0.0495
SPTLC3	0.8882	1.1615	1.1445	0.8900	1.0211	0.0763
MYL6B	0.8195	1.3949	1.1223	0.7475	1.0211	0.1487
LOC1001316	1.1552	0.9936	1.1235	0.8120	1.0211	0.0780
LOC729439	0.8509	1.4867	0.9936	0.7531	1.0211	0.1629
OR5AS1	0.9624	1.1133	1.0471	0.9615	1.0211	0.0367
PAR1	1.1140	1.1110	0.9562	0.9032	1.0211	0.0539
GPR142	0.9784	1.1243	1.0817	0.9000	1.0211	0.0507
BLR1	0.9828	1.1411	0.9669	0.9936	1.0211	0.0404
LOC284393	1.0172	1.1865	1.0858	0.7951	1.0211	0.0830
PEG10	0.9655	1.1546	1.0724	0.8920	1.0211	0.0579
ERGIC2	0.7145	1.0668	1.5381	0.7652	1.0211	0.1890
ZNF445	0.9359	1.2108	0.9608	0.9770	1.0211	0.0638
LIMA1	1.0253	1.3297	0.9835	0.7461	1.0212	0.1198
LOC727787	0.9315	1.1629	1.0863	0.9040	1.0212	0.0620
GPR75	0.9495	1.1247	1.0089	1.0016	1.0212	0.0370
LOC645711	0.9575	1.2047	0.9606	0.9621	1.0212	0.0612
LOC1001331	0.9508	1.1280	1.0449	0.9611	1.0212	0.0414
LOC652612	0.9584	1.2125	0.9779	0.9360	1.0212	0.0643
LOC647972	0.9973	1.1013	1.0394	0.9468	1.0212	0.0327
LOC648900	0.9872	1.1168	1.0977	0.8832	1.0212	0.0542
ASB9	0.9951	1.0278	1.0312	1.0309	1.0213	0.0087
LOC441151	0.9103	1.1140	1.0106	1.0501	1.0213	0.0427
ART3	1.0134	1.0942	0.9930	0.9845	1.0213	0.0251
LOC652811	1.0228	1.1276	1.0749	0.8598	1.0213	0.0579
LOC651189	1.0604	1.0073	1.0832	0.9344	1.0213	0.0331
CIB1	0.8136	1.3390	1.1860	0.7468	1.0213	0.1433
LOC441061	0.9515	1.1533	1.0729	0.9076	1.0213	0.0562
LOC647079	0.9247	1.1422	0.9653	1.0531	1.0213	0.0484
LOC1001288	0.9146	1.2546	1.0528	0.8634	1.0214	0.0874
LOC730644	0.8889	1.1574	1.1090	0.9301	1.0214	0.0659
LOC650167	0.9451	1.0788	1.1362	0.9253	1.0214	0.0512
LOC728405	0.9611	1.1166	1.0616	0.9462	1.0214	0.0408
LOC641804	0.9631	1.1871	0.9713	0.9641	1.0214	0.0553
BAT3	1.1064	1.1896	0.9725	0.8170	1.0214	0.0815

LOC652213	0.9685	1.2052	0.9798	0.9321	1.0214	0.0621
LOC54103	1.0189	1.0529	1.0246	0.9893	1.0214	0.0130
MIR20B	1.0118	0.9954	0.9594	1.1192	1.0214	0.0344
CBLN4	1.0204	1.1072	1.0179	0.9402	1.0214	0.0341
LOC646552	0.9593	1.1076	1.0499	0.9690	1.0214	0.0352
NCRNA0016	1.0061	1.0862	1.0980	0.8955	1.0215	0.0467
LOC643972	1.0393	1.0811	1.0272	0.9382	1.0215	0.0301
PIP5K1B	1.0506	1.0799	1.0067	0.9487	1.0215	0.0285
SFRS9	0.8205	1.5720	0.9486	0.7448	1.0215	0.1883
FLJ25006	0.9611	1.0830	1.0721	0.9698	1.0215	0.0325
LOC643870	0.8916	1.1395	1.0636	0.9913	1.0215	0.0528
LOC651790	0.9047	1.1195	1.0713	0.9906	1.0215	0.0471
APM-1	1.0397	1.1640	1.0085	0.8739	1.0215	0.0596
TYR	0.8704	1.1021	1.1689	0.9447	1.0215	0.0689
KRTAP13-3	0.9817	1.1853	1.0211	0.8979	1.0215	0.0603
LOC642985	0.9500	1.1645	0.9903	0.9814	1.0215	0.0484
ERCC-00041	0.9328	1.2499	0.9809	0.9225	1.0215	0.0772
LOC1001300	0.9490	1.1921	1.0542	0.8908	1.0215	0.0661
LOC650815	0.9719	1.1911	1.0082	0.9149	1.0215	0.0597
POPDC2	0.9243	1.1462	1.1689	0.8468	1.0215	0.0802
LOC650146	1.0127	1.0586	1.0866	0.9282	1.0215	0.0347
LOC1001290	0.8731	1.2065	1.0488	0.9578	1.0215	0.0713
LOC643492	0.9084	1.3165	0.9860	0.8754	1.0216	0.1010
LOC731640	0.8208	1.2217	1.3656	0.6782	1.0216	0.1624
CSDA	0.8796	1.1893	1.2991	0.7184	1.0216	0.1345
LOC1001340	0.8820	1.1568	1.0860	0.9616	1.0216	0.0616
LOC1001332	0.8611	1.0983	1.1852	0.9418	1.0216	0.0735
FIBIN	1.0310	1.0976	1.0434	0.9144	1.0216	0.0386
RAB42	1.0046	1.1434	1.0871	0.8512	1.0216	0.0635
VSIG4	0.9698	1.1164	1.0415	0.9586	1.0216	0.0366
NUDT13	0.9235	1.2001	1.1058	0.8571	1.0216	0.0794
LOC1001347	1.1850	0.9423	0.9886	0.9706	1.0216	0.0553
ERCC-00170	0.9605	1.1457	1.0235	0.9570	1.0216	0.0441
NEUROD6	0.9421	1.1521	1.0063	0.9862	1.0217	0.0455
EIF4EBP3	0.8701	1.5148	0.9391	0.7627	1.0217	0.1683
SAPS3	1.1193	1.3309	0.8480	0.7886	1.0217	0.1257
LOC650526	0.9043	1.1281	1.0040	1.0502	1.0217	0.0468
LOC1001320	0.9954	1.0649	1.1165	0.9099	1.0217	0.0448
MGC45491	0.9033	1.1230	0.9904	1.0701	1.0217	0.0480
MIR503	0.9435	1.0634	1.1025	0.9774	1.0217	0.0369
ABCA3	0.8996	1.3242	1.1055	0.7577	1.0217	0.1235
RPLP0P2	1.0167	1.0515	1.0841	0.9347	1.0217	0.0321
CTNNA2	0.8631	1.3023	1.0313	0.8902	1.0217	0.1005
KCNJ16	0.8917	1.1970	1.0217	0.9766	1.0218	0.0643
C5orf23	0.9139	1.2119	1.0531	0.9082	1.0218	0.0717
FAHD2A	1.0227	1.0987	1.0324	0.9332	1.0218	0.0340

PYGM	0.9386	1.1005	1.0239	1.0243	1.0218	0.0331
LOC1001319	0.9358	1.2137	1.1732	0.7645	1.0218	0.1054
LOC728835	1.0102	1.1835	1.0453	0.8482	1.0218	0.0689
LOC1001279	0.9286	1.2652	1.0282	0.8654	1.0218	0.0878
ZNF818	1.0297	1.0692	1.0582	0.9302	1.0218	0.0317
C15orf53	0.9789	1.1205	0.9766	1.0113	1.0218	0.0338
LOC1001315	0.8847	1.1238	1.0774	1.0014	1.0218	0.0522
LOC729047	0.9657	1.0724	1.0168	1.0324	1.0218	0.0221
PP8961	1.0868	1.0125	1.0347	0.9533	1.0218	0.0277
LOC644284	0.9908	1.1351	1.1008	0.8608	1.0218	0.0619
LOC730259	1.0421	1.1257	0.9834	0.9364	1.0219	0.0408
C21orf86	0.9799	1.0674	1.0596	0.9806	1.0219	0.0241
DYNLL1	0.8657	1.3771	0.9798	0.8651	1.0219	0.1214
USP24	1.0326	1.3441	0.9320	0.7789	1.0219	0.1194
LOC1001289	0.9587	1.1254	1.0723	0.9313	1.0219	0.0461
LOC641852	1.0178	1.1640	1.0326	0.8731	1.0219	0.0595
LOC644099	1.0000	1.0515	1.0945	0.9416	1.0219	0.0330
RNASE2	1.0024	1.0886	1.0508	0.9458	1.0219	0.0309
LOC730862	0.9503	1.1374	1.0692	0.9308	1.0219	0.0492
PADI3	1.0603	1.1221	1.0484	0.8570	1.0219	0.0573
LOC1001293	0.9939	1.1158	1.0415	0.9366	1.0219	0.0379
RPH3A	0.9281	1.1454	1.1623	0.8520	1.0219	0.0778
SOST	1.0003	1.0996	1.0507	0.9372	1.0220	0.0348
FZD4	0.9963	1.2195	1.0610	0.8110	1.0220	0.0845
DPRXP4	0.9993	1.0828	1.0255	0.9802	1.0220	0.0223
LOC732436	0.9887	1.1646	1.0249	0.9097	1.0220	0.0533
LOC1001327	1.1187	1.1264	1.0791	0.7638	1.0220	0.0867
LOC283970	0.9328	1.2170	1.0038	0.9343	1.0220	0.0671
GPR55	0.9585	1.1242	1.0631	0.9422	1.0220	0.0433
LOC389141	0.9101	1.3492	1.0176	0.8112	1.0220	0.1169
VPS13A	0.9651	1.2843	1.0105	0.8282	1.0220	0.0956
MAX	0.9200	1.2178	1.0787	0.8716	1.0220	0.0788
LOC730007	0.9170	1.2266	1.0874	0.8570	1.0220	0.0839
LOC1001308	0.9931	1.0985	1.0184	0.9781	1.0220	0.0268
FLJ11235	0.9970	0.9940	1.0720	1.0251	1.0220	0.0181
gm127	1.1072	0.9958	1.1086	0.8766	1.0220	0.0552
C9orf98	1.0187	1.1254	1.0304	0.9138	1.0220	0.0433
NANOS2	1.0354	1.0634	1.0175	0.9719	1.0221	0.0192
LOC1001302	0.9301	1.1670	1.1779	0.8134	1.0221	0.0900
MIR1249	0.9704	1.0458	1.1678	0.9044	1.0221	0.0565
GABRA4	0.8952	1.0929	1.0851	1.0151	1.0221	0.0458
LOC728517	0.9272	1.0621	1.1992	0.8999	1.0221	0.0689
CCDC120	0.8485	1.3520	1.0222	0.8657	1.0221	0.1167
TMEM139	0.9954	1.1427	1.0892	0.8611	1.0221	0.0617
PFN3	0.9394	1.1358	1.0717	0.9415	1.0221	0.0489
WAPAL	1.0392	1.3580	0.9728	0.7185	1.0221	0.1316

RPRD2	0.9597	1.3127	0.9807	0.8355	1.0221	0.1020
LOC729669	1.0269	1.1841	1.0141	0.8636	1.0222	0.0655
POTEA	0.9771	1.1096	1.0933	0.9087	1.0222	0.0480
LOC643265	1.0052	1.1071	1.0278	0.9488	1.0222	0.0328
LOC651060	1.0008	1.1151	1.0751	0.8979	1.0222	0.0477
LOC650712	0.9436	1.1923	0.9891	0.9639	1.0222	0.0574
LOC729368	0.9758	1.1128	1.0792	0.9211	1.0222	0.0446
LOC645144	0.9450	1.1236	1.1026	0.9178	1.0222	0.0529
JAK1	1.0005	1.0372	1.0350	1.0162	1.0222	0.0086
LOC441782	0.9732	1.0989	0.9913	1.0258	1.0223	0.0278
LOC645968	0.8801	1.3256	1.1775	0.7059	1.0223	0.1404
ENTPD1	0.9830	1.2195	1.0186	0.8680	1.0223	0.0732
CYTSB	0.8819	1.2596	1.1063	0.8414	1.0223	0.0982
EHD1	0.8080	1.5125	0.9428	0.8260	1.0223	0.1661
LOC648735	0.9913	1.0169	1.1173	0.9638	1.0223	0.0335
FAM80B	1.0785	1.1252	0.9922	0.8935	1.0223	0.0510
SARM1	1.0038	1.3809	0.8423	0.8624	1.0223	0.1248
OR10G3	0.9922	1.1357	1.0353	0.9263	1.0224	0.0439
LOC647407	0.9940	1.1058	1.0481	0.9415	1.0224	0.0353
OR5H15	0.9729	1.1670	1.0027	0.9469	1.0224	0.0495
LOC649037	0.9123	1.1134	1.1140	0.9498	1.0224	0.0533
LOC642183	0.8823	1.1395	1.1834	0.8844	1.0224	0.0808
LOC729252	0.9188	1.1215	1.0577	0.9915	1.0224	0.0435
CETN3	0.8241	1.5320	1.0198	0.7135	1.0224	0.1813
ABCC12	0.9535	1.0911	1.0934	0.9516	1.0224	0.0403
LOC1001291	1.0262	1.1277	1.0355	0.9001	1.0224	0.0468
LOC729530	1.0211	1.1365	1.0554	0.8765	1.0224	0.0543
KIR2DL4	0.9477	1.1205	1.0654	0.9561	1.0224	0.0423
SLC25A6	0.8634	1.5200	0.9780	0.7284	1.0224	0.1735
PLEKHM3	1.0048	1.0989	1.0892	0.8969	1.0225	0.0469
VDR	1.0077	1.1868	1.0491	0.8462	1.0225	0.0701
LOC1001287	0.9573	1.1859	1.0452	0.9015	1.0225	0.0620
TTLL10	1.0209	1.1221	0.9731	0.9738	1.0225	0.0350
LOC728843	0.9216	1.0425	1.2284	0.8975	1.0225	0.0756
LOC649296	0.9313	1.0667	1.1612	0.9307	1.0225	0.0562
LOC642207	0.9696	1.1098	1.1217	0.8889	1.0225	0.0563
CCDC109A	0.9307	1.3850	0.9963	0.7781	1.0225	0.1292
PPP1R9A	0.9501	1.1540	1.0946	0.8915	1.0225	0.0612
PAPOLB	0.9455	1.0294	1.1506	0.9646	1.0225	0.0463
TMEM119	0.9492	1.1341	1.1016	0.9054	1.0226	0.0561
NXPH1	1.0680	1.0306	1.0560	0.9356	1.0226	0.0300
NPPA	0.9405	1.1809	1.1090	0.8599	1.0226	0.0740
APC2	1.0083	1.1101	1.0254	0.9466	1.0226	0.0337
LOC1001331	1.0146	1.1280	0.9466	1.0011	1.0226	0.0381
SDR9C7	0.9068	1.0929	1.0877	1.0029	1.0226	0.0438
KIAA1586	0.9056	1.1909	1.0881	0.9058	1.0226	0.0707

PRAMEF6	1.0176	1.1258	1.0507	0.8964	1.0226	0.0478
KRT24	0.9728	1.1565	1.0645	0.8966	1.0226	0.0563
CHRNA3	1.0011	1.1103	1.0984	0.8807	1.0226	0.0533
LOC441722	0.9497	1.0271	1.0891	1.0247	1.0226	0.0285
SUPT7L	0.9668	1.3533	1.0360	0.7345	1.0227	0.1277
FLJ45964	1.0038	1.1075	1.0198	0.9595	1.0227	0.0310
LOC1001299	1.0005	1.1326	1.0391	0.9185	1.0227	0.0445
LOC652825	1.0738	0.9746	1.1398	0.9024	1.0227	0.0525
LOC1001285	0.9493	1.0973	1.1174	0.9267	1.0227	0.0493
WWP2	0.9379	1.2039	1.0561	0.8928	1.0227	0.0695
SCRG1	0.9582	1.1047	1.0730	0.9548	1.0227	0.0388
LOC440733	0.9880	1.2162	1.0814	0.8051	1.0227	0.0863
KIR3DL1	0.9408	1.1116	1.0774	0.9611	1.0227	0.0422
LOC645000	1.0350	1.1874	1.0656	0.8029	1.0227	0.0803
OR1L6	0.8998	1.1725	1.0420	0.9767	1.0227	0.0578
LOC390332	0.9411	1.0500	1.0863	1.0138	1.0228	0.0310
TAF1	0.9959	1.1855	1.0103	0.8994	1.0228	0.0596
LOC645522	1.0298	1.1047	1.0940	0.8627	1.0228	0.0559
SFTPA2B	1.0093	1.0608	1.1543	0.8668	1.0228	0.0600
LOC1001322	1.0510	1.0818	1.1700	0.7885	1.0228	0.0821
GTPBP1	0.9541	1.1206	1.0812	0.9353	1.0228	0.0460
LOC730281	0.9593	1.1398	1.0885	0.9037	1.0228	0.0549
FAM129C	0.8982	1.1638	1.1368	0.8926	1.0228	0.0738
FLJ34047	0.9852	1.1892	1.0025	0.9144	1.0228	0.0586
LOC644379	0.9951	1.0367	1.1283	0.9312	1.0229	0.0413
LOC441554	0.9945	1.0190	1.0218	1.0562	1.0229	0.0127
CNTN6	1.0842	1.0237	1.0566	0.9270	1.0229	0.0343
RPL32P3	1.0273	1.1127	1.0214	0.9301	1.0229	0.0373
MESP2	0.8818	1.2482	1.0861	0.8755	1.0229	0.0896
LOC645581	0.9812	1.1378	1.0319	0.9408	1.0229	0.0426
OR5P3	0.9602	1.1333	1.1181	0.8800	1.0229	0.0617
MCAM	0.9476	1.1227	1.0956	0.9260	1.0230	0.0502
LOC731479	0.9961	1.1742	0.9732	0.9483	1.0230	0.0513
ANKRD13B	0.9648	1.0901	1.1136	0.9234	1.0230	0.0466
MFN1	1.0494	1.2150	0.9999	0.8276	1.0230	0.0797
LOC652377	0.8850	1.2191	1.0601	0.9277	1.0230	0.0752
PRKRA	0.9246	1.2344	1.2397	0.6933	1.0230	0.1323
C16orf7	1.0684	1.1506	1.0514	0.8216	1.0230	0.0705
NODAL	1.0519	0.9068	1.0745	1.0589	1.0230	0.0390
SNORA40	0.9691	1.1274	1.0348	0.9608	1.0230	0.0385
LOC1001309	0.9070	1.0713	1.1459	0.9679	1.0230	0.0532
C10orf122	0.9690	1.1410	1.0546	0.9274	1.0230	0.0474
IFNB1	1.0136	1.1406	1.0242	0.9138	1.0230	0.0464
LOC646786	1.0264	1.0883	1.1393	0.8382	1.0231	0.0658
LOC645228	1.0097	1.0616	1.0507	0.9702	1.0231	0.0208
LOC388079	1.0481	0.9884	1.0953	0.9606	1.0231	0.0302

NCR1	1.0796	0.9852	1.0713	0.9563	1.0231	0.0308
LOC729046	1.0431	1.0772	1.0008	0.9712	1.0231	0.0233
CD81	0.9545	1.3701	0.9283	0.8395	1.0231	0.1183
LOC651655	0.9494	1.1152	1.0022	1.0256	1.0231	0.0346
LOC1001311	1.0185	1.0531	1.0738	0.9470	1.0231	0.0278
LOC644351	1.0054	1.0251	1.1030	0.9590	1.0231	0.0300
LOC646030	1.0443	1.1015	1.0622	0.8846	1.0232	0.0477
MIR1302-1	1.0080	1.0552	1.0903	0.9391	1.0232	0.0327
LOC391347	1.0033	1.0499	1.1438	0.8957	1.0232	0.0516
MARLIN1	0.9802	1.1572	1.0371	0.9183	1.0232	0.0508
LRRC55	0.9804	1.0695	1.0240	1.0188	1.0232	0.0183
LOC389111	1.0199	1.0828	1.1223	0.8678	1.0232	0.0559
LOC730176	1.0877	1.1540	1.0407	0.8104	1.0232	0.0746
PACRG	1.0091	1.1224	1.0522	0.9090	1.0232	0.0446
LOC649711	1.0114	1.0869	1.0602	0.9344	1.0232	0.0335
LOC285768	0.9929	1.1091	1.1390	0.8519	1.0232	0.0652
MIR629	0.9676	1.1470	1.0493	0.9290	1.0232	0.0483
OR4F6	0.9825	1.1456	0.9925	0.9724	1.0232	0.0410
MIR556	0.9110	1.1479	1.1521	0.8820	1.0233	0.0734
GALIG	0.8877	1.1420	1.1419	0.9214	1.0233	0.0689
ALDH1A1	1.0047	1.0947	1.0526	0.9411	1.0233	0.0330
TPTE	1.0026	1.1814	0.9838	0.9253	1.0233	0.0552
ADRA1D	0.9916	1.1367	1.0430	0.9218	1.0233	0.0452
TTC23	0.9545	1.2630	1.0442	0.8315	1.0233	0.0910
LOC650154	0.9305	1.1561	1.1164	0.8902	1.0233	0.0662
FAU	0.9591	1.3247	1.0013	0.8082	1.0233	0.1087
DIS3	1.0050	1.0343	1.1027	0.9513	1.0233	0.0315
NSFL1C	1.0099	1.3374	1.0080	0.7381	1.0234	0.1226
SLC24A2	0.9396	1.1765	1.0360	0.9413	1.0234	0.0558
LOC644029	0.9290	1.2145	1.1006	0.8495	1.0234	0.0825
SLITRK2	1.0271	1.0486	1.1340	0.8838	1.0234	0.0519
LOC286310	0.9216	1.2311	1.0437	0.8972	1.0234	0.0763
KRT76	0.9617	1.0999	1.1107	0.9212	1.0234	0.0481
H2AFB3	0.9651	1.2062	1.0878	0.8345	1.0234	0.0799
ZDHHC15	1.0572	1.0506	1.0473	0.9385	1.0234	0.0284
MICALCL	0.9686	1.1575	0.9793	0.9883	1.0234	0.0449
OR5I1	0.9481	1.1133	1.0911	0.9413	1.0234	0.0457
NCSTN	1.0209	1.3785	0.9467	0.7478	1.0235	0.1316
LOC648705	0.8708	1.1745	1.2077	0.8409	1.0235	0.0972
OR51A7	0.9803	1.1039	1.0627	0.9469	1.0235	0.0362
C16orf35	0.8323	1.3942	1.0572	0.8102	1.0235	0.1356
LOC648976	1.0560	1.0658	1.0732	0.8990	1.0235	0.0417
RAB6A	1.0714	1.1394	1.1217	0.7615	1.0235	0.0885
VWA3A	0.9864	1.2490	0.9894	0.8692	1.0235	0.0802
LOC645284	0.9267	1.2594	1.0267	0.8811	1.0235	0.0843
ELP3	1.0295	1.2560	1.0107	0.7978	1.0235	0.0936

RASGRP2	1.0847	1.0910	1.0040	0.9145	1.0235	0.0414
LOC645167	0.9149	1.2426	0.9986	0.9381	1.0235	0.0751
LOC729581	0.8942	1.1229	1.1672	0.9099	1.0235	0.0708
PPP3R2	0.9469	1.1297	1.0890	0.9288	1.0236	0.0503
LOC645694	0.9441	1.0717	1.1105	0.9680	1.0236	0.0401
TBC1D12	1.0273	1.1291	1.0299	0.9081	1.0236	0.0452
LOC652536	1.0184	1.0930	1.0394	0.9436	1.0236	0.0310
CDK2AP2	1.0543	1.3174	1.0151	0.7077	1.0236	0.1249
KCNA10	0.9240	1.1438	1.1013	0.9253	1.0236	0.0578
SLC44A2	1.0372	1.2737	1.0363	0.7474	1.0236	0.1077
SHARPIN	0.8982	1.4713	0.9653	0.7598	1.0237	0.1552
MIR449B	0.9753	1.1317	1.0430	0.9446	1.0237	0.0415
C14orf132	1.2123	1.2413	0.8665	0.7746	1.0237	0.1189
OR8U8	1.0797	1.0326	1.1332	0.8492	1.0237	0.0617
LOC643834	0.9880	1.0997	1.0658	0.9413	1.0237	0.0361
LOC1001337	0.9834	1.0330	1.1223	0.9560	1.0237	0.0365
FRG2C	0.9222	1.2107	0.9978	0.9642	1.0237	0.0642
SLC35D1	1.0147	1.1018	1.0258	0.9526	1.0237	0.0306
C10orf12	0.9049	1.2211	1.0776	0.8914	1.0237	0.0783
INPP5K	0.8659	1.3541	1.0670	0.8080	1.0238	0.1233
LOC440082	1.0055	1.1197	1.0755	0.8944	1.0238	0.0491
LOC642140	0.9417	1.0131	1.1497	0.9906	1.0238	0.0445
LOC401399	0.8836	1.1640	1.1345	0.9132	1.0238	0.0729
LOC440005	0.9008	1.1488	1.0812	0.9645	1.0238	0.0560
MIR1296	1.0302	1.0229	1.0713	0.9709	1.0238	0.0206
LOC652077	0.9736	1.0774	1.1928	0.8515	1.0238	0.0728
LOC1001324	0.9287	1.1192	1.1288	0.9187	1.0238	0.0579
CACNA2D2	0.9851	1.1919	1.0811	0.8373	1.0238	0.0752
LOC649076	0.9053	1.1809	1.1503	0.8590	1.0239	0.0826
PPAP2B	1.0388	1.1473	1.0831	0.8264	1.0239	0.0695
LOC1001303	0.9233	1.1595	1.1233	0.8894	1.0239	0.0686
LOC1001290	1.0206	1.0080	1.1485	0.9184	1.0239	0.0474
HILS1	0.8531	1.3436	0.9992	0.8996	1.0239	0.1108
LOC645089	1.0632	1.1088	0.9969	0.9267	1.0239	0.0397
LOC651686	0.9732	1.1543	1.0193	0.9488	1.0239	0.0458
KCNJ2	0.9083	1.0602	1.2175	0.9097	1.0239	0.0737
LOC649346	0.9474	1.1574	1.0246	0.9662	1.0239	0.0474
CACHD1	0.9526	1.1032	1.1134	0.9266	1.0239	0.0490
MYBPH	0.8958	1.1189	1.1056	0.9754	1.0239	0.0536
SNX25	0.9690	1.3389	0.9844	0.8035	1.0239	0.1127
SLC38A3	0.8288	1.2695	1.0325	0.9652	1.0240	0.0922
C20orf175	1.0095	1.0657	1.1258	0.8950	1.0240	0.0491
WBP2	0.8848	1.3132	1.1170	0.7811	1.0240	0.1192
PIP	0.9611	1.1061	1.0215	1.0075	1.0240	0.0302
LOC1001305	0.9836	1.1394	1.0074	0.9657	1.0240	0.0394
RGS1	1.1161	1.0384	1.0313	0.9104	1.0240	0.0425

FLJ20444	0.9012	1.3365	1.0131	0.8454	1.0240	0.1098
SNORD76	0.9188	1.1356	1.1405	0.9014	1.0241	0.0659
SLC45A4	0.9845	1.2613	0.9220	0.9284	1.0241	0.0803
DAP3	0.8331	1.4971	1.0454	0.7207	1.0241	0.1714
NHLH1	1.0054	1.0086	1.1034	0.9789	1.0241	0.0273
LOC1001340	1.0935	1.0962	1.0319	0.8747	1.0241	0.0520
LOC644808	0.9054	1.1588	1.0255	1.0067	1.0241	0.0521
C6orf170	0.9852	1.1427	1.0970	0.8715	1.0241	0.0607
WDR69	0.8512	1.1270	1.1591	0.9590	1.0241	0.0724
DNAJB7	0.9916	1.1757	1.0363	0.8927	1.0241	0.0588
EXTL3	1.0876	1.1495	0.9654	0.8939	1.0241	0.0578
LOC642852	0.9800	1.1832	0.9511	0.9821	1.0241	0.0535
SNORD89	0.8429	1.3135	1.1594	0.7806	1.0241	0.1272
STARD6	1.0809	1.0844	1.0802	0.8513	1.0242	0.0576
ZNF271	0.8294	1.4030	0.9753	0.8892	1.0242	0.1298
C15orf56	1.0466	1.1773	0.9464	0.9265	1.0242	0.0574
LOC644137	1.0141	1.0301	1.1494	0.9032	1.0242	0.0504
LOC440737	0.7266	1.2893	1.1864	0.8946	1.0242	0.1297
LOC388938	1.1275	1.1486	0.9784	0.8425	1.0242	0.0714
MAP7D1	0.9235	1.4321	0.9946	0.7467	1.0242	0.1456
NBR2	1.0663	1.1468	1.1747	0.7091	1.0242	0.1075
MIR2117	1.0160	1.0643	1.0742	0.9425	1.0242	0.0301
C1orf104	1.0005	1.1755	1.0198	0.9013	1.0243	0.0567
AKR1C4	0.9009	1.1990	1.0632	0.9340	1.0243	0.0680
DZIP3	0.9451	1.1701	0.9951	0.9868	1.0243	0.0498
PAWR	0.8640	1.1380	1.1413	0.9538	1.0243	0.0691
DKFZP547L1	0.9912	1.0672	1.0417	0.9970	1.0243	0.0182
LOC652801	0.9389	1.1562	1.1217	0.8804	1.0243	0.0676
SLC17A9	0.8503	1.1599	1.1356	0.9514	1.0243	0.0744
LOC285299	0.9911	1.1596	1.0197	0.9268	1.0243	0.0491
LOC441056	1.0906	0.9673	1.0477	0.9917	1.0243	0.0278
IMPACT	1.0144	1.1586	1.0562	0.8681	1.0243	0.0603
LOC1001342	0.9100	1.0422	1.1603	0.9849	1.0243	0.0528
GRIN2D	1.0513	1.1305	1.0156	0.9000	1.0244	0.0479
LOC442049	0.9000	1.2215	1.0841	0.8919	1.0244	0.0793
RUNDC3A	0.9564	1.2312	1.0796	0.8304	1.0244	0.0857
SLIT2	0.9431	1.1917	1.0078	0.9550	1.0244	0.0575
LOC390738	0.8677	1.1481	1.1501	0.9317	1.0244	0.0732
CD53	0.9970	1.1431	1.0234	0.9341	1.0244	0.0438
LOC1001308	0.9193	1.0878	1.0646	1.0260	1.0244	0.0373
GABARAPL1	1.0819	1.2683	0.9503	0.7972	1.0244	0.1000
LOC732450	1.0439	1.0383	1.0727	0.9428	1.0244	0.0282
MAGEB3	1.0111	1.1603	1.0194	0.9069	1.0244	0.0520
FTSJ3	1.0115	1.4685	0.8525	0.7653	1.0244	0.1566
LOC441136	0.9773	1.1771	0.9803	0.9632	1.0245	0.0510
LEREPO4	1.0273	1.0516	1.0749	0.9441	1.0245	0.0285

PCDHGA4	0.9807	1.0533	1.0996	0.9642	1.0245	0.0317
SOX1	0.9923	1.1384	1.0478	0.9194	1.0245	0.0462
PKD1L3	0.9008	1.0910	0.9482	1.1579	1.0245	0.0601
HIVEP2	0.8022	1.3757	1.0746	0.8454	1.0245	0.1315
LOC1001909	1.0060	1.1384	1.0196	0.9340	1.0245	0.0424
RPS2	0.9668	1.3217	1.0163	0.7933	1.0245	0.1100
LOC648424	0.9107	1.1542	1.0080	1.0251	1.0245	0.0500
LOC440335	0.9699	1.0604	1.0638	1.0040	1.0245	0.0228
NFKBIA	0.8378	1.5854	1.0178	0.6571	1.0245	0.2009
SAPS2	0.8424	1.3020	1.1204	0.8333	1.0245	0.1140
LOC442406	0.9338	1.1213	1.1317	0.9115	1.0246	0.0591
LOC652128	0.8461	1.1254	1.1213	1.0054	1.0246	0.0657
GABRA6	0.9595	1.3579	0.9414	0.8395	1.0246	0.1142
C16orf55	1.0095	1.1580	1.0004	0.9304	1.0246	0.0479
LOC729383	0.8866	1.2028	1.1004	0.9085	1.0246	0.0764
MAGEL2	0.8407	1.3756	0.9834	0.8986	1.0246	0.1206
LOC653428	1.0072	1.1494	0.9813	0.9605	1.0246	0.0427
LOC644257	0.9204	1.2293	1.0109	0.9379	1.0246	0.0710
LOC401602	1.0573	1.0723	1.1014	0.8674	1.0246	0.0532
RAB5C	0.8558	1.5929	0.9680	0.6818	1.0246	0.1984
PRND	0.9751	1.0854	1.1136	0.9244	1.0247	0.0448
LOC338797	0.9898	1.1227	1.0938	0.8923	1.0247	0.0525
FLJ77644	0.9030	1.0745	1.0775	1.0436	1.0247	0.0413
RAB4B	0.8895	1.1870	1.2296	0.7925	1.0247	0.1082
UBQLN3	0.9597	1.1206	1.0343	0.9841	1.0247	0.0355
SLC5A1	0.9815	1.1303	1.0832	0.9038	1.0247	0.0509
SYT17	0.9545	1.0733	1.1434	0.9277	1.0247	0.0507
CCDC151	1.0235	1.0644	1.1164	0.8945	1.0247	0.0474
TCN1	1.0521	1.0526	1.0159	0.9783	1.0247	0.0177
LOC649993	1.0487	1.1357	0.9789	0.9356	1.0247	0.0437
LY6G6E	1.0782	1.0577	1.1021	0.8611	1.0248	0.0553
HPVC1	0.9901	1.1810	0.9838	0.9441	1.0248	0.0531
TMEM16B	0.9114	1.1809	1.0164	0.9906	1.0248	0.0566
LOC647326	0.9910	1.1842	1.0722	0.8517	1.0248	0.0700
LOC651373	0.9621	1.0822	1.0683	0.9867	1.0248	0.0297
CIB3	0.9363	1.1521	0.9995	1.0113	1.0248	0.0455
LOC1001338	0.9048	1.4107	1.0275	0.7563	1.0248	0.1401
TMEM63C	0.8315	1.2678	1.0916	0.9083	1.0248	0.0977
DDX20	0.9862	1.0423	1.1256	0.9452	1.0248	0.0390
SSX8	1.0124	1.1197	1.0437	0.9236	1.0248	0.0406
LOC197350	1.0175	1.2074	0.9321	0.9423	1.0248	0.0638
OR6C70	0.9881	1.1149	1.0848	0.9117	1.0248	0.0464
NDFIP2	0.8025	1.6158	1.0030	0.6782	1.0249	0.2080
DTX3	0.9656	1.1085	1.0604	0.9650	1.0249	0.0358
NR1D2	0.8859	1.1598	1.1430	0.9110	1.0249	0.0733
LYSMD4	0.9974	1.2809	1.0378	0.7835	1.0249	0.1020

LOC1001344	0.9682	1.1822	1.0507	0.8984	1.0249	0.0610
HELQ	1.0071	1.1926	1.0650	0.8348	1.0249	0.0743
LOC729351	1.0153	1.1051	1.0413	0.9380	1.0249	0.0346
UBE2E1	0.8566	1.3973	1.1721	0.6736	1.0249	0.1613
GRM8	1.0165	1.0976	0.9921	0.9934	1.0249	0.0249
SYT7	1.0575	1.2592	0.9402	0.8428	1.0249	0.0896
LOC1001330	1.0221	1.0438	1.0359	0.9978	1.0249	0.0101
FLJ16171	0.9842	1.1080	1.1464	0.8611	1.0249	0.0646
LOC1001319	1.0705	1.1163	1.0136	0.8994	1.0249	0.0468
IL19	0.9501	1.1295	1.0653	0.9549	1.0249	0.0439
EMX2OS	0.8029	1.3724	1.0291	0.8954	1.0249	0.1248
EDC3	1.0097	1.2752	1.0381	0.7767	1.0249	0.1019
OR1A2	0.9850	1.1603	1.0389	0.9156	1.0249	0.0517
HLA-DQB2	1.0170	1.0684	0.9512	1.0632	1.0249	0.0272
LOC285053	0.8356	1.4197	1.0766	0.7679	1.0249	0.1473
LOC729667	0.9819	1.2438	1.0256	0.8485	1.0250	0.0821
LOC643176	0.9605	1.0826	1.1093	0.9473	1.0250	0.0414
CD226	0.9512	1.1004	1.1697	0.8785	1.0250	0.0668
LOC728640	0.8304	1.4749	0.9497	0.8449	1.0250	0.1523
PLEKHA2	0.9478	1.2205	1.0454	0.8862	1.0250	0.0729
C15orf37	0.9080	1.0931	1.0617	1.0371	1.0250	0.0406
EME2	0.9987	1.0856	1.1353	0.8803	1.0250	0.0559
LOC340598	1.0327	1.2171	1.0706	0.7795	1.0250	0.0910
LOC440508	1.0239	1.1431	0.9319	1.0011	1.0250	0.0439
APOC2	0.8374	1.3219	0.9504	0.9903	1.0250	0.1041
LOC1001300	1.0165	1.0303	1.1609	0.8926	1.0251	0.0548
EPPK1	1.0945	1.0047	1.0297	0.9714	1.0251	0.0260
OR52W1	0.9528	1.1387	0.9365	1.0724	1.0251	0.0485
SNHG10	1.0333	1.0684	1.0216	0.9771	1.0251	0.0188
TLR2	0.9528	1.0141	1.1801	0.9534	1.0251	0.0536
ASL	0.9778	1.1028	1.0856	0.9343	1.0251	0.0410
C8orf80	0.8762	1.1647	1.1362	0.9235	1.0251	0.0732
OR5B3	0.9847	1.1089	1.0943	0.9126	1.0251	0.0466
LOC645915	0.9575	1.1576	1.0684	0.9171	1.0251	0.0545
LOC650651	0.9423	1.1261	1.0553	0.9769	1.0251	0.0411
LOC646863	1.0562	1.0935	1.0785	0.8724	1.0252	0.0515
SLC1A6	0.9043	1.1684	1.0292	0.9988	1.0252	0.0547
MOBKL2A	1.0419	1.1592	1.0171	0.8825	1.0252	0.0568
LOC440461	0.9410	1.0436	1.1770	0.9391	1.0252	0.0562
TRPM6	0.9787	1.1562	0.9829	0.9830	1.0252	0.0437
VNN1	1.0258	1.1048	0.9897	0.9806	1.0252	0.0283
ESPNL	0.9882	1.0583	1.0760	0.9782	1.0252	0.0246
LOC727944	0.9669	1.1939	0.9121	1.0279	1.0252	0.0610
VILL	1.0329	1.0250	1.0226	1.0205	1.0252	0.0027
LOC729392	0.9954	1.0798	1.1356	0.8902	1.0252	0.0535
LOC732445	0.9435	1.2000	1.0079	0.9496	1.0252	0.0600

EIF4G1	0.8803	1.4963	0.9127	0.8117	1.0252	0.1584
KIAA1908	0.9610	1.2269	1.0039	0.9093	1.0253	0.0699
LOC400723	0.8624	1.1986	1.0384	1.0017	1.0253	0.0691
MYSM1	1.0089	0.9721	1.2330	0.8870	1.0253	0.0738
NPHP3	0.9419	1.2002	1.0331	0.9259	1.0253	0.0629
LOC441795	0.8871	1.1067	0.9846	1.1227	1.0253	0.0554
XAGE1A	0.8776	1.5280	0.9295	0.7661	1.0253	0.1710
GORASP2	0.8632	1.4609	1.0124	0.7648	1.0253	0.1539
LOC1001334	0.9589	1.1282	1.0475	0.9667	1.0253	0.0397
LOC1001336	0.9805	1.0889	1.0776	0.9544	1.0253	0.0339
COL12A1	0.8943	1.1847	1.1352	0.8872	1.0254	0.0784
LOC642424	1.0987	1.1040	0.9878	0.9111	1.0254	0.0466
LOC613266	0.9909	1.1048	0.9669	1.0389	1.0254	0.0304
OR10S1	0.9018	1.1616	1.0040	1.0341	1.0254	0.0535
KRTAP5-9	0.9695	1.0786	1.0849	0.9685	1.0254	0.0326
HECA	0.9086	1.2343	1.0932	0.8654	1.0254	0.0854
LOC728602	0.9043	1.1872	1.1556	0.8544	1.0254	0.0852
LOC390407	1.0268	1.1027	1.1281	0.8440	1.0254	0.0642
LOC729643	0.9403	1.0589	1.1344	0.9680	1.0254	0.0443
RAI2	0.8857	1.2199	1.1213	0.8746	1.0254	0.0863
LOC645904	0.9419	1.1968	1.0273	0.9356	1.0254	0.0608
ZNF154	1.0906	1.1216	1.0822	0.8073	1.0254	0.0732
OPHN1	1.0134	1.1816	1.0755	0.8311	1.0254	0.0735
LOC650990	0.9584	1.1862	1.0690	0.8881	1.0254	0.0652
SCUBE1	1.0005	1.0537	1.1197	0.9278	1.0254	0.0406
LOC644325	0.9114	1.1103	1.0957	0.9844	1.0255	0.0473
PCDHGA12	1.0345	1.0578	1.0312	0.9783	1.0255	0.0168
TRPC7	0.9206	1.1308	1.1552	0.8953	1.0255	0.0682
SLC30A9	0.9361	1.2225	1.1243	0.8190	1.0255	0.0909
MIR1305	1.0259	1.1006	0.9481	1.0273	1.0255	0.0311
LOC650950	0.9584	1.2341	1.0614	0.8480	1.0255	0.0821
NRSN1	1.0200	1.1017	0.9948	0.9855	1.0255	0.0264
IRS1	0.9743	1.0691	1.0912	0.9675	1.0255	0.0319
LOC643612	0.9988	1.1644	1.0137	0.9251	1.0255	0.0502
LOC1001292	0.9809	1.1240	1.0423	0.9549	1.0255	0.0376
LOC653166	0.9719	1.1056	1.0624	0.9622	1.0255	0.0349
OR2F2	0.9820	1.1106	1.0485	0.9611	1.0255	0.0339
C1S	1.0432	1.0241	1.1046	0.9304	1.0256	0.0361
PDLIM5	0.7459	1.4602	1.0761	0.8201	1.0256	0.1612
LOC646249	1.0010	1.1162	1.0511	0.9341	1.0256	0.0385
PLA2G16	0.9944	1.0756	1.0855	0.9468	1.0256	0.0333
LOC653397	0.9425	1.0950	1.1281	0.9368	1.0256	0.0501
GRAMD2	0.9844	1.1596	1.0108	0.9476	1.0256	0.0465
LOC1001338	0.9284	1.2398	0.9888	0.9456	1.0256	0.0725
LOC730058	1.0175	1.1646	1.1089	0.8114	1.0256	0.0776
LOC728255	1.0336	1.0611	0.9914	1.0164	1.0256	0.0147

LAMB2L	0.9853	1.1666	1.0353	0.9154	1.0256	0.0530
LETM2	0.9937	1.0976	1.1026	0.9088	1.0257	0.0463
TRAT1	0.9126	1.1791	1.0988	0.9123	1.0257	0.0674
SERPINB10	0.8867	1.2432	1.0335	0.9393	1.0257	0.0786
DNAJC21	0.9446	1.2075	1.0426	0.9082	1.0257	0.0669
LOC1001289	0.8940	1.0698	1.0490	1.0900	1.0257	0.0447
LOC643882	0.9280	1.2677	0.9860	0.9212	1.0257	0.0820
DKFZp451A2	0.8863	1.0667	1.1217	1.0281	1.0257	0.0503
TAS2R46	1.1056	1.0469	1.0538	0.8966	1.0257	0.0450
HNF4A	0.9870	1.0845	1.0807	0.9509	1.0258	0.0336
LOC1001348	1.0577	1.1880	1.0268	0.8306	1.0258	0.0738
LOC441870	0.9767	1.2582	1.0514	0.8167	1.0258	0.0916
LOC1001304	1.0464	1.0812	1.0162	0.9592	1.0258	0.0258
LOC1001328	0.9397	1.2569	0.9732	0.9335	1.0258	0.0775
LOC646626	0.9950	1.2405	1.0038	0.8640	1.0258	0.0784
MTMR14	1.0719	1.2585	1.0111	0.7618	1.0258	0.1026
LOC1001344	0.9501	1.1286	1.1158	0.9089	1.0258	0.0563
MARCO	0.9931	1.1036	1.0177	0.9890	1.0258	0.0267
PDZK3	1.1248	1.0183	1.0758	0.8846	1.0259	0.0519
FASTKD5	0.9586	1.6650	0.7705	0.7093	1.0259	0.2196
C2orf21	0.9724	1.0871	1.1641	0.8800	1.0259	0.0626
KCNAB3	1.0174	1.0851	1.0405	0.9606	1.0259	0.0259
LOC645238	0.9824	1.0592	1.1476	0.9144	1.0259	0.0502
LOC651429	0.9858	1.0370	1.0116	1.0693	1.0259	0.0178
LOC649201	1.0236	1.0525	1.1176	0.9101	1.0259	0.0433
LOC497256	0.8994	1.1993	1.0783	0.9268	1.0259	0.0699
MRVI1	1.0021	1.1119	1.0400	0.9498	1.0259	0.0341
LOC1001284	0.9069	1.2295	1.0745	0.8929	1.0260	0.0794
KIAA0195	1.0029	1.2734	1.0129	0.8146	1.0260	0.0943
WSCD2	1.0530	1.2171	1.0192	0.8145	1.0260	0.0827
MGRN1	0.9972	1.3678	0.9853	0.7536	1.0260	0.1270
FLJ32255	0.9306	1.0798	1.1172	0.9764	1.0260	0.0436
SOX10	0.9973	1.0137	1.1383	0.9547	1.0260	0.0395
LOC728463	0.9756	1.1312	1.0052	0.9921	1.0260	0.0356
C20orf117	1.0676	1.2430	0.9428	0.8508	1.0260	0.0849
LOC653052	0.9306	1.2676	1.0102	0.8956	1.0260	0.0840
LOC642536	0.9223	1.2919	1.0096	0.8803	1.0260	0.0926
DSCR1L1	0.9396	1.1788	1.0106	0.9751	1.0260	0.0529
LOC1001297	0.8861	1.2612	1.0209	0.9359	1.0260	0.0832
CDYL2	1.0280	1.2278	0.9635	0.8850	1.0261	0.0733
LOC440350	0.8846	1.0866	1.1018	1.0314	1.0261	0.0495
RMND5B	0.9938	1.3352	1.0187	0.7567	1.0261	0.1187
MIR7-1	0.9647	1.2861	0.9749	0.8787	1.0261	0.0893
C9orf82	0.8461	1.3698	1.0531	0.8355	1.0261	0.1250
LOC1001335	0.8892	1.1848	1.0910	0.9396	1.0262	0.0681
NPPC	1.0439	1.1833	0.9753	0.9021	1.0262	0.0599

UXS1	1.0946	1.0727	0.9998	0.9377	1.0262	0.0358
ADAM20	1.0049	1.0837	1.0803	0.9360	1.0262	0.0351
CNGA1	0.9917	1.0881	0.9872	1.0381	1.0263	0.0236
LOC1001327	0.9574	1.1908	1.1536	0.8035	1.0263	0.0902
PPBP	0.8786	1.1385	1.1803	0.9079	1.0263	0.0775
MED11	0.9014	1.3748	1.0119	0.8172	1.0263	0.1228
MAPRE2	0.9176	1.3248	1.0862	0.7767	1.0263	0.1179
LOC652051	0.9493	1.1461	1.1106	0.8993	1.0263	0.0602
LOC646193	1.0156	1.0290	1.1218	0.9390	1.0263	0.0375
LOC731365	0.9964	1.2705	1.0188	0.8198	1.0263	0.0927
LOC642432	1.0043	0.9479	1.1790	0.9742	1.0264	0.0522
LOC643186	1.0326	1.0370	1.1044	0.9314	1.0264	0.0357
ITPR2	0.9902	1.3120	0.9377	0.8655	1.0264	0.0986
LOC645635	0.9194	1.1258	1.1745	0.8857	1.0264	0.0725
TFF3	1.0333	1.3352	0.9602	0.7768	1.0264	0.1162
C10orf115	1.0236	1.0970	1.0130	0.9719	1.0264	0.0260
LOC165186	0.9461	1.1487	1.1133	0.8975	1.0264	0.0616
LOC652471	0.9751	1.1627	1.0525	0.9152	1.0264	0.0534
LHX1	0.9251	1.1969	1.0243	0.9593	1.0264	0.0604
C5orf40	1.0151	1.0694	1.0941	0.9270	1.0264	0.0370
LOC1001338	0.9380	1.2113	1.0298	0.9267	1.0264	0.0658
LOC1001301	0.9933	1.1774	1.0361	0.8990	1.0265	0.0579
DAAM2	0.9770	1.1297	1.0695	0.9296	1.0265	0.0450
SERPINA9	0.8868	1.1046	1.1300	0.9844	1.0265	0.0563
PELI1	0.9936	1.3804	0.9580	0.7740	1.0265	0.1274
LOC401284	1.0137	1.0748	1.0939	0.9235	1.0265	0.0383
LOC1001308	0.9364	1.1266	1.0154	1.0275	1.0265	0.0390
PDZD8	0.8667	1.3072	1.1344	0.7977	1.0265	0.1184
CPPED1	0.9838	1.0685	1.0923	0.9615	1.0265	0.0318
VAX2	0.9275	1.0266	1.2019	0.9502	1.0265	0.0622
FOXN1	0.9502	1.0991	1.0589	0.9979	1.0265	0.0329
OR2D2	1.0094	0.9719	1.1105	1.0144	1.0266	0.0296
MIR607	1.0056	1.0568	1.0872	0.9566	1.0266	0.0287
MIR200B	1.0186	1.2740	0.9608	0.8529	1.0266	0.0893
SCGB1D4	1.0153	1.1145	1.0197	0.9570	1.0266	0.0326
LOC651513	1.0369	1.0541	1.1180	0.8974	1.0266	0.0465
LOC654201	1.0097	1.1681	1.0559	0.8727	1.0266	0.0611
C3orf50	0.9611	1.0987	1.1281	0.9185	1.0266	0.0512
LOC1001338	0.9698	1.1857	0.9662	0.9848	1.0266	0.0532
FBXO10	1.0544	1.2137	0.9924	0.8460	1.0266	0.0761
AEBP1	0.9600	1.1065	0.9648	1.0752	1.0266	0.0376
CAPG	0.9494	1.1655	0.9985	0.9931	1.0266	0.0476
LOC1001281	0.9552	1.0816	1.0654	1.0045	1.0267	0.0290
CAMKK2	0.9297	1.2172	1.1112	0.8485	1.0267	0.0840
EFCAB3	1.0728	0.9709	1.1182	0.9448	1.0267	0.0411
LOC341315	0.7552	1.0966	1.5556	0.6993	1.0267	0.1970

HIST1H2AA	0.9714	1.0643	1.1677	0.9032	1.0267	0.0574
PAF1	0.8911	1.4364	1.0254	0.7539	1.0267	0.1474
LOC642564	0.9866	1.2414	1.0169	0.8619	1.0267	0.0790
LOC1001347	0.9720	1.1040	1.0323	0.9985	1.0267	0.0286
GRIK4	0.9975	1.1417	1.0304	0.9373	1.0267	0.0429
UTP14C	0.8435	1.4741	0.9669	0.8224	1.0267	0.1525
LOC647089	0.9685	1.1328	1.0843	0.9213	1.0267	0.0492
CLUAP1	1.0549	1.1631	1.1156	0.7733	1.0267	0.0873
ASCL4	0.9858	1.1362	1.0088	0.9762	1.0268	0.0371
SULT1E1	0.9676	1.1749	1.0001	0.9644	1.0268	0.0500
LOC653305	0.9240	1.1848	1.1147	0.8835	1.0268	0.0729
TUBA3C	0.9435	1.1271	1.1705	0.8660	1.0268	0.0727
CACNA2D1	1.0482	1.1403	1.0196	0.8988	1.0268	0.0498
ZNF792	0.9123	1.2891	0.9916	0.9142	1.0268	0.0894
LOC647229	0.9112	1.1840	1.1055	0.9064	1.0268	0.0700
LOC651450	0.9517	1.1323	1.0368	0.9864	1.0268	0.0393
LOC644208	1.0002	1.1230	0.9949	0.9891	1.0268	0.0321
LOC729907	1.0074	1.0217	1.1155	0.9627	1.0268	0.0321
RBM7	0.9790	1.1744	1.1290	0.8249	1.0268	0.0792
KIAA1432	0.9099	1.1527	1.0973	0.9474	1.0268	0.0583
LOC730413	0.9217	1.2189	1.1576	0.8091	1.0268	0.0968
FOSB	0.9870	1.1756	1.0132	0.9316	1.0268	0.0524
GLO1	0.8546	1.4640	1.0338	0.7551	1.0269	0.1567
LOC728830	0.9430	1.1041	1.0653	0.9952	1.0269	0.0359
FAM102B	0.9660	1.0298	1.2208	0.8910	1.0269	0.0706
FBXW7	0.8811	1.2792	1.1096	0.8379	1.0269	0.1031
LOC1001347	0.9306	1.2820	1.0315	0.8638	1.0270	0.0917
LOC651916	0.9610	1.1403	0.8816	1.1251	1.0270	0.0632
LOC400129	0.9726	1.1870	1.0573	0.8910	1.0270	0.0632
PPP1R2P9	0.9446	1.2361	1.0225	0.9049	1.0270	0.0739
NEFL	0.9940	1.4752	0.8923	0.7467	1.0270	0.1578
OR52E5	1.0569	1.1380	1.0042	0.9090	1.0270	0.0480
MIR1234	0.9320	1.0881	1.2224	0.8657	1.0270	0.0801
NOXO1	0.9327	1.1400	1.0645	0.9711	1.0271	0.0467
LOC645836	0.9201	1.2131	1.0600	0.9151	1.0271	0.0705
LOC644923	0.9709	1.1279	1.1333	0.8762	1.0271	0.0628
LOC340268	0.8535	1.1230	1.0971	1.0348	1.0271	0.0607
OLFM4	0.9802	1.1583	1.0205	0.9494	1.0271	0.0461
LOC1001320	0.9642	1.1272	1.0924	0.9248	1.0271	0.0489
PTGER1	1.0008	1.1326	1.0266	0.9486	1.0272	0.0387
LOC642778	1.0461	1.1297	1.0328	0.8999	1.0272	0.0475
HMG20A	0.9177	1.2211	1.0786	0.8912	1.0272	0.0768
COL17A1	1.0150	1.1459	1.0473	0.9005	1.0272	0.0506
LOC646237	0.9104	1.0875	1.1015	1.0094	1.0272	0.0439
LOC1001298	0.9519	1.0930	1.0453	1.0185	1.0272	0.0295
LOC728782	0.7094	1.3839	1.1898	0.8256	1.0272	0.1569

LOC440160	0.9127	1.3925	1.0206	0.7829	1.0272	0.1311
SLC9A8	1.0020	1.1988	1.0404	0.8676	1.0272	0.0682
CYTH4	1.1296	1.0127	1.0273	0.9391	1.0272	0.0392
LOC643990	1.0627	1.1295	0.9383	0.9784	1.0272	0.0428
SEMA4D	0.8902	1.4474	0.9467	0.8246	1.0272	0.1423
RHO	1.0094	1.1108	1.0735	0.9152	1.0272	0.0428
LOC645157	0.9140	1.1086	1.4330	0.6534	1.0273	0.1643
HTR1F	1.0422	1.1568	0.9936	0.9165	1.0273	0.0503
HCRP1	1.0026	1.1895	0.9689	0.9481	1.0273	0.0552
LOC344593	0.9453	1.2112	1.0792	0.8733	1.0273	0.0747
LOC1001293	0.9249	1.0998	1.1161	0.9685	1.0273	0.0475
TTTY20	1.1174	1.1154	1.0406	0.8358	1.0273	0.0663
MUC19	1.0505	1.0460	1.0285	0.9844	1.0273	0.0151
ZHX1	1.0049	1.1910	1.0770	0.8364	1.0273	0.0743
CLEC4M	0.9848	1.1617	1.0316	0.9314	1.0273	0.0492
OR2A12	0.9927	1.0803	0.9911	1.0454	1.0274	0.0217
KLK7	0.9403	1.1314	1.0799	0.9578	1.0274	0.0466
TMEM216	1.1046	1.0892	0.9275	0.9883	1.0274	0.0421
ISL2	0.9916	1.2725	0.9116	0.9339	1.0274	0.0834
FLJ36144	0.9423	1.1034	1.1067	0.9572	1.0274	0.0449
LOC1001322	0.9919	1.1130	1.0158	0.9889	1.0274	0.0292
RPS3A	0.8906	1.2512	1.1761	0.7918	1.0274	0.1105
LOC642762	0.9290	1.0767	1.1082	0.9958	1.0274	0.0404
LOC643000	0.9591	1.1755	1.0817	0.8934	1.0274	0.0629
LOC650733	0.9502	1.1318	1.0534	0.9743	1.0274	0.0412
LOC643910	1.0783	1.0793	1.1287	0.8234	1.0274	0.0690
MIR1292	0.9422	1.1770	1.1231	0.8674	1.0275	0.0733
IL1F10	0.9531	1.1158	1.0852	0.9557	1.0275	0.0426
MGC5139	0.9647	1.4327	0.7995	0.9129	1.0275	0.1394
LOC646227	0.9447	1.0738	1.0572	1.0341	1.0275	0.0287
LOC647974	1.0346	1.0106	1.0947	0.9700	1.0275	0.0261
LOC728790	0.9278	1.0621	1.0884	1.0315	1.0275	0.0352
LOC729903	1.0359	1.2112	1.0636	0.7993	1.0275	0.0853
LOC648716	0.9593	1.1521	1.0729	0.9256	1.0275	0.0521
LOC1001288	1.0112	1.1926	1.0236	0.8825	1.0275	0.0636
ATP6V0D2	0.9861	1.1887	0.9981	0.9372	1.0275	0.0553
BCL2L2	0.8608	1.4941	1.0228	0.7326	1.0276	0.1665
LOC648085	0.9754	1.2475	1.0042	0.8832	1.0276	0.0777
TSPYL5	0.9932	1.0652	1.0513	1.0006	1.0276	0.0180
LOC401913	0.9531	1.1249	1.1235	0.9090	1.0276	0.0565
MRPL42P5	0.8702	1.2010	1.1692	0.8703	1.0277	0.0911
SLC30A10	0.9856	1.1489	1.0216	0.9545	1.0277	0.0427
ProSAPiP1	1.0226	1.1297	1.0328	0.9256	1.0277	0.0417
LOC645113	1.0495	1.2230	1.0079	0.8303	1.0277	0.0806
LOC642340	0.8395	1.1827	1.0504	1.0381	1.0277	0.0707
CYFIP2	0.9101	1.2980	1.0833	0.8194	1.0277	0.1054

TMEM111	0.8065	1.5160	1.0733	0.7151	1.0277	0.1796
FLJ44955	0.9979	1.1744	1.0353	0.9033	1.0277	0.0562
ATP6V0E2	0.7880	1.3685	1.1608	0.7936	1.0277	0.1432
KIF22	1.3430	1.1666	0.9033	0.6981	1.0277	0.1423
TTTY7B	0.9745	1.1016	1.1260	0.9089	1.0277	0.0517
RBM	0.9528	1.1253	1.0965	0.9364	1.0277	0.0485
SEZ6	1.0257	1.0742	1.1184	0.8926	1.0277	0.0489
SEC13	0.9928	1.3529	1.0091	0.7562	1.0277	0.1228
ALDH6A1	0.8369	1.5179	1.0395	0.7167	1.0278	0.1764
ANP32D	1.0716	1.0667	1.0872	0.8858	1.0278	0.0475
LOC652844	0.9912	1.2590	0.8839	0.9772	1.0278	0.0806
MGC102966	0.9151	1.2308	1.0827	0.8828	1.0278	0.0806
KANK3	0.9466	1.0544	1.0389	1.0716	1.0278	0.0279
NCRNA00111	0.9722	1.1293	1.0737	0.9361	1.0278	0.0446
QRFP	0.9257	1.0622	1.1808	0.9427	1.0279	0.0593
NUP210L	0.9967	1.1371	1.0331	0.9445	1.0279	0.0407
PPP5C	1.0434	1.1966	0.9823	0.8892	1.0279	0.0646
LARGE	1.0013	1.2366	1.0099	0.8636	1.0279	0.0772
KLRC4	0.9344	1.1226	1.0893	0.9652	1.0279	0.0460
LOC1001342	1.0077	1.3675	0.7789	0.9575	1.0279	0.1234
PCCA	0.8926	1.4735	1.0081	0.7374	1.0279	0.1586
LOC1001327	0.9263	1.1359	1.0389	1.0105	1.0279	0.0432
LOC647237	0.9554	1.0508	1.1526	0.9527	1.0279	0.0474
SLC17A7	0.9576	1.1748	1.0788	0.9004	1.0279	0.0615
LOC400987	1.0423	1.0678	1.0964	0.9054	1.0280	0.0423
CHPF	1.1014	1.2388	1.0232	0.7485	1.0280	0.1033
RNPEPL1	0.8823	1.2308	1.0729	0.9260	1.0280	0.0789
RAPGEF6	0.9250	1.6927	0.8279	0.6664	1.0280	0.2279
DRD1IP	1.0750	1.1413	0.9716	0.9242	1.0280	0.0492
LOC1001295	1.0021	1.0834	1.0655	0.9611	1.0280	0.0283
STX11	0.9729	1.0702	1.0778	0.9912	1.0280	0.0268
DEFB118	0.9931	1.1010	1.0645	0.9537	1.0281	0.0334
THBS4	1.0031	1.1112	1.0405	0.9574	1.0281	0.0325
LOC647839	0.9483	1.0810	1.0961	0.9869	1.0281	0.0359
C2orf80	0.9741	1.1743	1.0600	0.9040	1.0281	0.0582
COX15	0.9210	1.2515	1.0810	0.8589	1.0281	0.0879
RIMBP3	1.0799	1.0470	1.1247	0.8608	1.0281	0.0580
CLIP4	1.0254	1.1459	0.9602	0.9810	1.0281	0.0415
FZD1	0.9543	1.2602	1.0688	0.8292	1.0281	0.0915
LOC644935	1.1172	1.1991	1.0690	0.7272	1.0281	0.1038
GNB1	0.9914	1.3556	1.0108	0.7554	1.0283	0.1236
CRIM2	0.9215	1.1411	1.0840	0.9666	1.0283	0.0509
LOC728534	1.0553	1.1303	1.0204	0.9073	1.0283	0.0464
SPATS2	0.8903	1.4732	0.9760	0.7737	1.0283	0.1540
TMIGD1	1.0186	1.1223	1.0381	0.9343	1.0283	0.0386
LOC147975	0.9921	1.0976	1.0896	0.9340	1.0283	0.0395

CD3G	0.9698	1.0699	1.1131	0.9604	1.0283	0.0376
GABRA2	0.9128	1.1902	1.0723	0.9382	1.0284	0.0643
LOC652589	0.9749	1.2350	0.8886	1.0149	1.0284	0.0738
PARP6	1.0145	1.1834	0.9889	0.9266	1.0284	0.0549
TAF5L	1.0091	1.2541	0.9950	0.8555	1.0284	0.0828
FBXO45	0.9085	1.1954	1.1257	0.8841	1.0284	0.0778
TAF1L	0.9197	1.3675	1.0280	0.7985	1.0284	0.1224
LOC1001286	1.0309	1.1017	1.0337	0.9475	1.0285	0.0316
LOC729660	0.9311	1.2897	0.9519	0.9412	1.0285	0.0872
BAIAP2L2	0.9530	1.1822	1.0144	0.9643	1.0285	0.0530
LOC1001311	1.1055	1.0560	1.0763	0.8762	1.0285	0.0518
LOC642366	1.0411	1.0311	1.0816	0.9602	1.0285	0.0252
ASRGL1	0.9285	1.1047	1.0925	0.9883	1.0285	0.0423
RPL10	1.1083	1.0721	1.0603	0.8735	1.0285	0.0527
LOC645179	0.8985	1.0873	1.0338	1.0944	1.0285	0.0454
ERCC-00158	0.9834	1.0152	1.1447	0.9709	1.0286	0.0398
LOC731884	1.0521	1.0048	1.1392	0.9182	1.0286	0.0461
TM4SF18	0.9676	1.2228	1.0902	0.8339	1.0286	0.0832
CPNE5	1.0791	1.0087	0.9900	1.0367	1.0286	0.0194
LOC648878	0.9975	1.0848	1.0685	0.9637	1.0286	0.0288
LOC645660	0.9655	1.0191	1.1058	1.0240	1.0286	0.0290
LOC642787	1.0025	1.2386	0.9662	0.9073	1.0287	0.0727
PIB5PA	0.9925	1.2180	1.0205	0.8837	1.0287	0.0697
LOC653261	0.9742	1.1804	1.0917	0.8683	1.0287	0.0681
GLT25D2	1.0561	1.0991	1.0433	0.9162	1.0287	0.0393
MIR193B	0.9213	1.1849	1.0592	0.9493	1.0287	0.0600
GRLF1	0.9450	1.1627	1.0963	0.9107	1.0287	0.0602
RHBDL2	0.7698	1.2933	1.1335	0.9181	1.0287	0.1156
TNFSF8	1.0871	1.1680	0.9430	0.9167	1.0287	0.0597
REM1	0.9196	1.0715	1.0972	1.0266	1.0287	0.0392
LOC642720	0.9356	1.1041	1.1045	0.9706	1.0287	0.0442
RNF111	0.9482	1.2433	1.1080	0.8154	1.0287	0.0932
ZNRF1	0.8799	1.1861	1.0964	0.9526	1.0288	0.0691
SPRR3	0.9539	1.1694	1.0733	0.9185	1.0288	0.0574
TUBA3D	0.9979	1.4798	0.9375	0.6998	1.0288	0.1635
ZER1	1.2162	1.2661	0.7852	0.8476	1.0288	0.1237
ZKSCAN2	1.0723	1.2226	0.9376	0.8826	1.0288	0.0759
C1orf173	1.0428	1.0521	0.9775	1.0427	1.0288	0.0172
LOC389435	1.0246	1.1839	1.1351	0.7716	1.0288	0.0920
LOC390638	0.9687	1.1023	1.0422	1.0022	1.0288	0.0287
LOC1001303	1.0272	1.1122	0.9810	0.9950	1.0288	0.0294
LOC647335	1.0935	1.0920	0.9680	0.9618	1.0288	0.0369
TAS2R16	0.8806	1.1539	1.0852	0.9957	1.0289	0.0591
MEAF6	0.8091	1.4284	1.1156	0.7624	1.0289	0.1545
ERCC-00048	0.9550	1.0942	0.9952	1.0711	1.0289	0.0325
ZCWPW2	1.1133	1.0980	0.9771	0.9271	1.0289	0.0456

LOC390033	0.9910	1.1821	0.9977	0.9447	1.0289	0.0524
MLXIP	0.9744	1.1152	1.0489	0.9770	1.0289	0.0336
LCN9	1.0187	1.1249	1.0529	0.9192	1.0289	0.0427
LOC647344	0.9604	1.1817	0.9463	1.0273	1.0289	0.0539
LOC644537	0.9498	1.0938	1.0672	1.0048	1.0289	0.0323
LOC147646	0.9898	1.1824	1.0432	0.9002	1.0289	0.0591
BATF	1.0409	1.1321	1.0875	0.8553	1.0289	0.0608
ST6GAL2	0.9731	1.0796	1.0949	0.9681	1.0289	0.0338
SULT2A1	0.9648	1.1475	1.1178	0.8856	1.0290	0.0623
LOC731396	0.9699	1.2947	1.0284	0.8229	1.0290	0.0986
KBTBD5	1.0443	1.0298	1.0837	0.9581	1.0290	0.0262
NAP1L5	1.0235	1.1450	1.1371	0.8103	1.0290	0.0780
ZNF175	1.0436	1.2434	1.0605	0.7685	1.0290	0.0979
LOC653499	0.9747	1.0535	1.0182	1.0698	1.0290	0.0211
CCBP2	0.9972	1.0362	1.1336	0.9492	1.0290	0.0391
LOC440871	1.0289	0.9888	1.0980	1.0003	1.0290	0.0245
WDR66	1.0128	1.1793	1.0166	0.9074	1.0290	0.0561
SLC35A3	0.8803	1.3952	1.1073	0.7335	1.0291	0.1442
PRSS37	1.0704	1.1266	0.9984	0.9209	1.0291	0.0446
FAM114A2	1.0969	1.0880	1.0832	0.8483	1.0291	0.0603
RGP1	0.9991	1.1008	1.0832	0.9333	1.0291	0.0389
YIPF6	0.8164	1.4178	1.1146	0.7677	1.0291	0.1505
ATP5S	0.8211	1.4219	1.1312	0.7423	1.0291	0.1555
BTN2A2	1.0325	1.0903	1.0525	0.9414	1.0292	0.0316
SNRNP200	1.0185	1.2233	0.9925	0.8825	1.0292	0.0711
LOC728207	0.9898	1.1351	1.1215	0.8705	1.0292	0.0622
FBXL3	1.0474	1.0980	1.0920	0.8797	1.0293	0.0511
LOC1001345	1.1223	1.2270	0.8924	0.8753	1.0293	0.0867
FLJ10120	0.9560	1.1684	0.9854	1.0074	1.0293	0.0475
LOC1001289	1.0220	1.1439	1.0493	0.9020	1.0293	0.0498
LOC645724	0.9572	1.0596	1.1198	0.9806	1.0293	0.0373
CTSH	0.9350	1.4479	1.0667	0.6677	1.0293	0.1623
LOC389142	1.1126	1.1046	1.0447	0.8554	1.0293	0.0599
RPS16	1.0359	1.0220	1.1050	0.9544	1.0293	0.0309
N4BP2L2	1.0401	1.1828	1.0083	0.8860	1.0293	0.0610
TNIP3	1.0269	1.1484	1.0523	0.8897	1.0293	0.0534
LOC387826	1.1101	1.0284	1.0120	0.9668	1.0293	0.0299
OR4D5	1.0057	0.9845	1.1640	0.9630	1.0293	0.0457
ZNF230	0.9977	1.0643	1.0276	1.0276	1.0293	0.0136
TSNAXIP1	0.9199	1.2320	1.0646	0.9008	1.0293	0.0768
MATN2	0.9415	1.1910	1.0579	0.9270	1.0293	0.0613
LOC644031	0.9379	1.1658	0.9716	1.0421	1.0293	0.0504
KLHL38	0.8776	1.2560	1.0314	0.9524	1.0294	0.0818
LOC653270	1.0080	1.1952	1.0279	0.8864	1.0294	0.0635
NGFRAP1	0.9711	1.4737	0.9807	0.6921	1.0294	0.1625
LOC1001313	0.9383	1.1703	1.1278	0.8812	1.0294	0.0706

FLJ21511	0.9509	1.1460	1.0512	0.9695	1.0294	0.0446
HHAT	1.0872	1.0666	1.1020	0.8619	1.0294	0.0563
CHD3	0.9482	1.2321	1.0434	0.8938	1.0294	0.0743
C13orf39	0.9879	1.1595	1.0341	0.9361	1.0294	0.0478
LOC1001909	0.8525	1.2423	1.3355	0.6875	1.0294	0.1547
FLJ37786	1.0091	1.1320	1.0591	0.9176	1.0294	0.0450
C9orf57	0.9544	1.2338	1.0661	0.8636	1.0294	0.0797
LOC150568	1.0023	1.0486	1.0765	0.9903	1.0295	0.0201
PI3	0.9261	1.1157	1.1009	0.9753	1.0295	0.0467
LOC441440	1.0373	1.1381	1.0726	0.8700	1.0295	0.0571
IYD	0.8904	1.2804	1.1109	0.8363	1.0295	0.1026
ANP32A	0.9577	1.1070	1.0432	1.0104	1.0296	0.0313
SERPINB4	0.9773	1.0840	1.1279	0.9291	1.0296	0.0461
HSPA1L	1.0920	1.1595	0.9945	0.8724	1.0296	0.0624
TREML1	0.9393	1.0130	1.1634	1.0028	1.0296	0.0475
MTMR11	0.9712	1.1515	1.0343	0.9616	1.0296	0.0437
LOC1001287	0.9173	1.2251	1.1078	0.8684	1.0296	0.0831
RPL31P11	0.9424	1.1684	1.0851	0.9229	1.0297	0.0587
DNAJC14	1.0336	1.1679	1.0801	0.8373	1.0297	0.0699
LOC728608	1.0100	1.0614	1.0656	0.9819	1.0297	0.0203
LOC728944	0.9566	1.4433	0.9995	0.7195	1.0297	0.1510
URG4	0.9599	1.4107	1.0297	0.7187	1.0297	0.1434
PHLDA3	0.8338	1.3021	1.1863	0.7968	1.0297	0.1263
LRRN2	0.9304	1.1885	1.0509	0.9492	1.0297	0.0592
C12orf63	1.0202	1.1584	0.9767	0.9637	1.0298	0.0445
PNRC1	0.9860	1.1428	1.1548	0.8354	1.0298	0.0753
LOC1001297	1.0071	1.1238	1.0887	0.8995	1.0298	0.0498
LGALS14	1.0416	1.0804	1.0189	0.9784	1.0298	0.0213
C9orf90	0.8430	1.4351	1.1998	0.6413	1.0298	0.1777
SON	0.9022	1.4130	1.0512	0.7528	1.0298	0.1415
MIR200C	0.9620	1.0471	1.1055	1.0046	1.0298	0.0306
LOC729957	1.0389	1.1099	1.1387	0.8318	1.0298	0.0693
LOC647107	0.8986	1.0819	1.1004	1.0384	1.0298	0.0456
CLASP1	0.9373	1.3638	1.0126	0.8056	1.0298	0.1193
LOC646740	0.9878	1.1065	1.0897	0.9354	1.0298	0.0410
BEND5	1.0171	1.0869	1.0302	0.9852	1.0299	0.0212
ANKRD18B	0.9739	1.1564	1.0345	0.9546	1.0299	0.0455
LIPL2	0.9354	1.0818	1.0441	1.0582	1.0299	0.0324
LOC642343	1.0451	1.1185	1.0170	0.9388	1.0299	0.0371
LOC649909	1.0132	1.0832	1.1591	0.8640	1.0299	0.0628
DBH	1.0464	1.0287	1.0380	1.0064	1.0299	0.0086
C16orf46	0.9016	1.2578	1.1638	0.7964	1.0299	0.1083
WDR72	1.1021	1.2361	1.0456	0.7359	1.0299	0.1058
OR6C4	1.1484	1.0702	0.9737	0.9275	1.0299	0.0494
NBPF20	0.6601	1.7669	0.8972	0.7957	1.0300	0.2504
LOC642757	1.0833	1.0637	1.0279	0.9452	1.0300	0.0305

GRM6	1.0942	1.0577	0.9906	0.9775	1.0300	0.0277
FAM123C	0.9945	1.0968	1.0776	0.9512	1.0300	0.0344
LOC283314	0.9547	1.1297	1.1098	0.9258	1.0300	0.0523
LOC728323	1.0001	1.1517	1.1077	0.8606	1.0300	0.0648
RIPK3	0.9490	1.1797	0.9420	1.0495	1.0300	0.0556
FLJ30430	0.8966	1.2934	0.9673	0.9630	1.0301	0.0893
LOC389365	1.0413	1.0344	1.1051	0.9396	1.0301	0.0341
LOC1001297	0.9992	1.0710	1.0391	1.0111	1.0301	0.0160
LOC346329	0.8368	1.1124	1.1822	0.9890	1.0301	0.0758
MIR1294	1.0048	1.0285	1.0488	1.0383	1.0301	0.0094
RPLP1	0.9894	1.2003	1.0717	0.8592	1.0301	0.0716
C11orf49	1.0423	1.2846	0.9508	0.8428	1.0301	0.0941
CDK3	0.9618	1.2062	1.0377	0.9150	1.0302	0.0639
ERCC-00144	0.9683	1.2735	0.9180	0.9609	1.0302	0.0818
LOC641825	0.9412	1.1060	1.1519	0.9216	1.0302	0.0579
LDOC1	0.8606	1.3024	1.1109	0.8469	1.0302	0.1091
LOC652217	1.0314	1.1253	1.0680	0.8961	1.0302	0.0487
IGFBP5	1.0302	1.2465	1.0052	0.8389	1.0302	0.0837
OR51A2	0.9909	1.1348	1.1077	0.8875	1.0302	0.0569
PLA2G4D	0.9923	1.0668	1.0690	0.9928	1.0302	0.0218
JRK	0.9917	1.2073	0.9699	0.9521	1.0302	0.0596
LOC1001338	1.0029	1.2552	0.9772	0.8856	1.0302	0.0791
RGS17	0.8961	1.3104	1.0362	0.8782	1.0302	0.0998
LOC644787	0.9655	1.1532	1.1081	0.8941	1.0303	0.0605
FANCM	1.0055	1.0534	1.1465	0.9156	1.0303	0.0481
LOC645876	0.9716	0.9942	1.0527	1.1026	1.0303	0.0296
LOC653192	0.9322	1.1120	1.1035	0.9734	1.0303	0.0455
C7orf61	0.9335	1.1574	1.0639	0.9664	1.0303	0.0506
FBS1	1.0029	1.2070	1.0978	0.8137	1.0303	0.0834
ADAM5P	1.0296	1.0613	1.0645	0.9659	1.0304	0.0229
LOC1001335	0.9918	1.1073	1.0483	0.9741	1.0304	0.0301
LOC653692	0.8718	1.1963	1.0784	0.9750	1.0304	0.0695
RAET1L	1.0620	1.1411	1.0294	0.8889	1.0304	0.0527
SPATA20	0.8672	1.4188	1.1134	0.7222	1.0304	0.1526
LOC652552	1.0585	1.0228	1.0792	0.9611	1.0304	0.0259
LOC728509	1.0319	1.0658	1.0652	0.9586	1.0304	0.0252
ZNF329	1.0563	1.3009	0.9997	0.7648	1.0304	0.1101
LOC1001335	0.9846	1.1363	1.0586	0.9421	1.0304	0.0427
LOC497190	0.9915	1.1061	1.0599	0.9642	1.0304	0.0323
SLC2A5	1.0476	1.1377	1.0069	0.9296	1.0304	0.0433
HTR1D	0.9173	1.2546	1.0102	0.9398	1.0305	0.0773
ANKIB1	0.9319	1.4687	0.9533	0.7681	1.0305	0.1518
CD19	0.9621	1.1012	1.1458	0.9129	1.0305	0.0554
MGC45922	0.9879	1.0481	1.1331	0.9529	1.0305	0.0395
C1orf203	0.9943	1.3767	1.0698	0.6812	1.0305	0.1428
IRS4	1.0375	1.2078	0.9960	0.8809	1.0305	0.0677

UGT3A1	0.9606	1.0814	1.1005	0.9797	1.0305	0.0353
LOC652508	0.9897	1.0808	1.0872	0.9644	1.0305	0.0313
EVI2A	1.0507	1.0523	1.0937	0.9255	1.0305	0.0364
LOC648211	0.9631	1.0747	1.2115	0.8729	1.0306	0.0731
GLG1	0.9163	1.3954	1.0209	0.7896	1.0306	0.1305
LOC1001924	0.9515	1.1177	1.1056	0.9475	1.0306	0.0469
CXorf41	1.0062	1.1261	1.0632	0.9268	1.0306	0.0424
KRT40	1.0042	1.0615	1.0999	0.9569	1.0306	0.0315
CLIC3	0.9037	1.1178	1.1005	1.0005	1.0306	0.0496
LOC646743	1.0249	1.0237	0.9383	1.1356	1.0306	0.0404
CCR1	1.0284	1.1214	0.9660	1.0068	1.0306	0.0329
LOC646547	0.9871	1.2192	1.0390	0.8775	1.0307	0.0713
SNORD113-1	1.0288	1.1499	0.9839	0.9601	1.0307	0.0422
CTNNB1	0.9785	1.4249	0.9863	0.7331	1.0307	0.1439
LOC1001299	0.9053	1.1094	1.0812	1.0269	1.0307	0.0452
ZMIZ2	1.0261	1.1404	1.1453	0.8110	1.0307	0.0782
LOC1001347	0.8274	1.2521	1.1341	0.9094	1.0307	0.0982
LOC728779	1.1395	1.2450	0.8643	0.8741	1.0308	0.0957
CGNL1	1.0607	1.3705	0.9677	0.7242	1.0308	0.1336
LOC646114	0.9142	1.0779	1.1313	0.9998	1.0308	0.0473
KIF3B	1.0528	1.4311	0.9804	0.6589	1.0308	0.1585
GALNT4	1.0420	1.2343	0.9169	0.9301	1.0308	0.0734
DMP1	0.9935	1.1028	1.1551	0.8718	1.0308	0.0628
LOC646162	0.9631	1.1303	1.0931	0.9370	1.0309	0.0476
HMHB1	1.0432	1.0107	1.1225	0.9472	1.0309	0.0365
LOC643150	1.0032	1.1474	1.0331	0.9399	1.0309	0.0434
PHF20	1.0239	1.4613	0.9585	0.6799	1.0309	0.1617
LOC643791	0.8906	1.1740	1.1009	0.9582	1.0309	0.0648
LOC1001346	0.9250	1.1470	1.0928	0.9590	1.0309	0.0530
LOC644647	0.9508	1.1729	0.9725	1.0276	1.0310	0.0500
WDR26	0.9073	1.4637	1.1221	0.6308	1.0310	0.1758
DSCR10	0.9871	1.0334	1.1119	0.9916	1.0310	0.0289
LOC729459	1.0896	1.2374	1.0118	0.7852	1.0310	0.0944
FOSL1	0.9535	1.1131	1.1196	0.9378	1.0310	0.0494
LOC729789	1.0371	1.1309	1.0254	0.9310	1.0311	0.0409
RGAG4	0.9869	1.1607	1.0318	0.9449	1.0311	0.0467
KCNV2	0.9650	1.1553	1.1170	0.8871	1.0311	0.0632
LOC1001338	1.0089	1.0904	1.0283	0.9969	1.0311	0.0208
C3orf67	1.0274	1.1788	0.9448	0.9735	1.0311	0.0521
HRASLS2	1.0890	1.0143	1.0353	0.9860	1.0311	0.0218
LOC1001295	0.9227	1.2360	1.0236	0.9422	1.0311	0.0717
LOC390940	0.9724	1.3813	0.9045	0.8664	1.0311	0.1188
LOC642456	0.9521	1.1676	1.0082	0.9967	1.0311	0.0471
C9orf27	0.9602	1.2355	1.0079	0.9210	1.0312	0.0704
ASTN2	0.8724	1.2748	1.0560	0.9214	1.0312	0.0900
WNT6	0.9087	1.2610	1.0317	0.9233	1.0312	0.0814

LOC729675	1.0189	1.0976	1.0483	0.9599	1.0312	0.0288
C14orf147	0.7370	1.6069	1.0278	0.7530	1.0312	0.2032
MOBKL2C	0.9824	1.2782	1.0056	0.8585	1.0312	0.0884
LOC642018	1.0071	1.2023	1.1058	0.8095	1.0312	0.0839
IL22RA1	1.0718	1.1339	1.0684	0.8507	1.0312	0.0620
LOC727929	0.9343	1.1839	1.1128	0.8937	1.0312	0.0697
FERMT3	1.0697	1.1342	1.0778	0.8431	1.0312	0.0643
SLC27A4	0.9888	1.0420	1.0201	1.0739	1.0312	0.0179
LOC650795	0.9792	1.0835	1.0971	0.9650	1.0312	0.0343
LOC644539	1.0016	1.1086	1.1120	0.9026	1.0312	0.0499
LOC388925	0.9045	1.2818	1.1792	0.7595	1.0312	0.1206
C12orf62	0.9295	1.3457	1.1495	0.7004	1.0313	0.1393
CXCL14	1.0203	1.0189	1.0133	1.0727	1.0313	0.0139
ERCC-00017	1.0346	1.0825	1.0717	0.9365	1.0313	0.0332
STK25	1.0085	1.4225	0.9529	0.7415	1.0313	0.1425
RPL39	0.9293	1.1549	1.0916	0.9496	1.0313	0.0548
C20orf114	1.0698	1.0845	1.0664	0.9048	1.0314	0.0424
KAAG1	1.0488	1.1498	1.0249	0.9020	1.0314	0.0509
SFRP2	1.0349	1.1012	1.1471	0.8424	1.0314	0.0671
NAT15	0.9666	1.3634	0.9927	0.8029	1.0314	0.1184
CRYGN	1.0293	1.0658	0.9910	1.0394	1.0314	0.0155
LOC647509	1.0860	1.1362	1.0069	0.8966	1.0314	0.0522
LOC648080	0.9625	1.2323	1.1066	0.8245	1.0315	0.0883
LOC646774	0.9189	1.1090	1.0872	1.0109	1.0315	0.0430
CCDC90B	0.9795	1.2849	1.2119	0.6498	1.0315	0.1429
MT1M	0.9847	1.0876	1.0723	0.9814	1.0315	0.0282
LOC650455	0.9747	1.0883	1.1119	0.9512	1.0315	0.0402
PRR25	1.0311	1.1661	1.0181	0.9110	1.0316	0.0523
LOC647766	0.9338	1.1498	1.1040	0.9388	1.0316	0.0558
BCKDHB	1.0213	1.2046	1.0972	0.8033	1.0316	0.0849
TRIL	0.9813	1.0880	1.1023	0.9548	1.0316	0.0372
CSNK1A1L	1.0012	1.1146	1.1388	0.8718	1.0316	0.0611
ZMYM6	0.8739	1.4006	1.1196	0.7324	1.0316	0.1467
LOC644480	1.0194	1.0699	1.0298	1.0074	1.0316	0.0136
LOC649571	0.9685	1.1390	1.0629	0.9560	1.0316	0.0430
FAM90A7	0.9457	1.1262	1.1137	0.9408	1.0316	0.0511
MOS	1.0152	1.1813	0.9506	0.9793	1.0316	0.0516
VWC2	0.9877	1.0152	1.1257	0.9980	1.0316	0.0319
A4GALT	1.0347	1.1393	0.9889	0.9637	1.0316	0.0388
GOLGA9P	1.0205	1.1574	1.0259	0.9230	1.0317	0.0481
LOC649088	0.8920	1.1549	1.1344	0.9455	1.0317	0.0662
ENO3	0.9434	1.3443	1.0004	0.8387	1.0317	0.1094
DEFB137	0.9636	1.1172	1.0408	1.0051	1.0317	0.0326
MIR320B2	0.9572	1.0124	1.2002	0.9572	1.0318	0.0576
LOC1001336	0.8073	1.2558	1.2181	0.8459	1.0318	0.1190
ADAMTS12	1.0216	1.0519	1.0645	0.9891	1.0318	0.0168

IL28B	0.9137	1.0601	1.2240	0.9296	1.0319	0.0720
PJA1	0.9797	1.3382	0.9686	0.8410	1.0319	0.1068
MIR24-2	0.8705	1.2113	1.1242	0.9215	1.0319	0.0811
C12orf40	0.9545	1.1547	1.0564	0.9618	1.0319	0.0471
LOC653701	0.8674	1.1661	1.1936	0.9003	1.0319	0.0859
MBD3L2	1.0840	1.1482	0.9288	0.9665	1.0319	0.0509
LOC1001308	0.9202	1.1590	1.1124	0.9360	1.0319	0.0608
LGALS3	0.9392	1.1417	1.0482	0.9988	1.0320	0.0428
C5orf43	0.9029	1.2930	1.0440	0.8879	1.0320	0.0939
ZNF219	0.9971	1.2447	1.1075	0.7786	1.0320	0.0985
LOC255411	1.0997	1.0549	1.0061	0.9672	1.0320	0.0288
UCN3	0.8864	1.2027	1.0673	0.9714	1.0320	0.0679
MIB2	1.0068	1.2071	1.0852	0.8290	1.0320	0.0792
LOC1001300	1.0877	1.0985	1.0275	0.9143	1.0320	0.0422
LOC643035	1.0137	1.0704	1.0715	0.9725	1.0321	0.0240
TMPRSS9	0.9662	1.1741	1.1072	0.8808	1.0321	0.0665
BDNFOS	0.9265	1.1159	1.1043	0.9816	1.0321	0.0465
DHRS4L1	0.9082	1.1830	1.1112	0.9259	1.0321	0.0681
CLEC7A	0.9849	1.1083	1.1174	0.9178	1.0321	0.0486
RANBP2L1	1.0370	1.1842	1.0134	0.8937	1.0321	0.0596
COL18A1	1.0055	1.1987	1.0274	0.8968	1.0321	0.0624
HAPLN2	0.9144	1.2257	1.0243	0.9640	1.0321	0.0683
IRX1	1.0776	1.2736	1.0075	0.7699	1.0322	0.1040
LOC647341	0.9957	1.1338	1.0720	0.9273	1.0322	0.0449
DSCR4	1.0696	1.1527	1.0740	0.8324	1.0322	0.0693
FLJ42562	0.9147	1.1222	1.1047	0.9874	1.0322	0.0493
PTGIR	1.0473	1.1064	1.0733	0.9021	1.0323	0.0450
C15orf60	1.0371	1.1364	0.9546	1.0009	1.0323	0.0386
MIR561	0.9838	1.2914	0.9383	0.9155	1.0323	0.0875
C22orf36	1.0158	1.2200	1.0367	0.8566	1.0323	0.0744
LOC1001345	0.9720	1.0123	1.1100	1.0349	1.0323	0.0290
DSCR6	0.9247	1.2022	1.0031	0.9993	1.0323	0.0594
LOC341356	1.1431	1.1296	0.9061	0.9505	1.0323	0.0608
TMEM42	0.7692	1.5066	1.0605	0.7931	1.0323	0.1713
LOC391769	0.9274	1.2034	1.0665	0.9321	1.0323	0.0655
LOC649957	0.9094	1.1912	1.0788	0.9501	1.0324	0.0641
CES7	1.0072	1.1277	1.0585	0.9361	1.0324	0.0405
IMAA	0.9452	1.4064	0.9932	0.7847	1.0324	0.1324
TTC16	1.0229	1.2359	0.9770	0.8937	1.0324	0.0729
LOC652670	1.0317	1.1076	1.0312	0.9590	1.0324	0.0303
RASA4P	0.7931	1.6292	0.9194	0.7880	1.0324	0.2012
MLL5	0.9673	1.2353	1.0761	0.8509	1.0324	0.0818
LOC400019	1.1461	1.0498	1.0660	0.8678	1.0324	0.0588
MGC33556	0.9254	1.1243	1.1048	0.9752	1.0324	0.0487
SNORD114-2	0.9937	1.1343	1.0648	0.9369	1.0324	0.0429
DENND3	0.9729	1.1157	1.0904	0.9506	1.0324	0.0414

CHD8	0.9253	1.4396	0.8798	0.8849	1.0324	0.1361
WNT3A	0.9418	1.0813	1.0564	1.0502	1.0324	0.0309
ELF5	0.9620	1.1814	1.0836	0.9028	1.0325	0.0623
SLC9A9	1.1222	1.1261	1.0008	0.8808	1.0325	0.0583
NRBP1	0.9698	1.2541	1.0070	0.8990	1.0325	0.0772
PION	0.9733	1.1853	1.0036	0.9677	1.0325	0.0516
LOC343184	0.9929	1.2707	1.0710	0.7955	1.0325	0.0983
LOC1001313	0.8998	1.2124	1.1417	0.8762	1.0325	0.0848
LYZL4	0.9891	1.0958	1.0818	0.9633	1.0325	0.0330
LOC643953	1.0992	1.0200	1.0403	0.9706	1.0325	0.0266
LOC1001331	1.0810	1.0164	1.1118	0.9209	1.0325	0.0422
LRRC36	1.0958	1.1204	0.9949	0.9192	1.0325	0.0465
LOC727877	0.8836	1.2935	1.0304	0.9228	1.0325	0.0923
LOC728264	0.9314	1.1529	1.1040	0.9419	1.0326	0.0563
KIAA1862	0.9166	1.2283	0.9907	0.9946	1.0326	0.0677
MSR1	0.9877	1.1778	0.9892	0.9756	1.0326	0.0485
MARK4	1.0299	1.1387	0.9814	0.9803	1.0326	0.0372
LOC646992	1.0355	1.0343	1.0496	1.0110	1.0326	0.0080
VAT1	1.2124	1.3502	0.8681	0.6998	1.0326	0.1503
LOC1001316	1.1110	1.1318	1.0384	0.8493	1.0326	0.0643
LOC728914	1.0699	1.0996	1.0734	0.8875	1.0326	0.0488
TTTY3	0.9326	1.2034	1.0222	0.9723	1.0326	0.0598
HIST1H2BA	1.0954	1.0496	1.0446	0.9409	1.0326	0.0326
TMCC1	0.9786	1.1386	1.2018	0.8116	1.0326	0.0874
GATA5	0.9951	1.1950	1.0552	0.8852	1.0326	0.0646
FLG	0.9345	1.1712	1.1232	0.9016	1.0326	0.0672
RGS9	1.0412	1.1302	1.0319	0.9272	1.0326	0.0416
LOC1001318	1.1107	1.0212	1.0760	0.9227	1.0326	0.0410
IKZF4	0.9187	1.0898	1.1796	0.9424	1.0327	0.0619
SCAMP2	0.9622	1.4540	0.9103	0.8042	1.0327	0.1442
CYP4A11	0.9721	1.1576	1.0042	0.9969	1.0327	0.0422
GPR115	1.0105	1.1619	1.0635	0.8950	1.0327	0.0556
NLGN1	1.0575	1.2142	1.0128	0.8464	1.0327	0.0756
LOC144742	0.9573	1.1200	1.1633	0.8903	1.0327	0.0650
LOC391578	0.9217	1.2703	1.0970	0.8420	1.0327	0.0954
DARC	1.0805	1.0616	1.0787	0.9101	1.0327	0.0411
LOC645885	1.0683	1.1570	1.0492	0.8566	1.0328	0.0633
CAPZB	1.0248	1.3423	1.0272	0.7368	1.0328	0.1237
METTL11B	0.9786	1.2095	1.0223	0.9207	1.0328	0.0625
MIR877	1.0783	1.0311	1.0805	0.9413	1.0328	0.0326
SHB	0.9734	1.2030	0.9800	0.9748	1.0328	0.0568
SNAI2	1.0595	1.1160	1.0676	0.8882	1.0328	0.0498
LOC727969	0.9997	1.1167	1.0738	0.9410	1.0328	0.0390
LOC1001342	0.9059	1.2192	1.0280	0.9783	1.0328	0.0670
LOC1001342	0.9819	1.2197	1.2081	0.7216	1.0328	0.1173
LCN10	0.9865	1.3032	0.9494	0.8924	1.0329	0.0922

LOC647157	1.0265	1.0898	1.0696	0.9456	1.0329	0.0320
LRRC29	0.9633	1.1136	1.0844	0.9702	1.0329	0.0387
C15orf20	1.0543	1.0271	1.0788	0.9713	1.0329	0.0231
C2orf39	1.0023	1.0768	1.0745	0.9779	1.0329	0.0252
C7orf55	0.9816	1.4500	1.0270	0.6729	1.0329	0.1597
CCL16	0.9522	1.1910	1.0413	0.9471	1.0329	0.0570
UGT3A2	1.0813	1.0225	1.0189	1.0089	1.0329	0.0164
LOC653143	0.9914	1.0741	1.0014	1.0648	1.0329	0.0213
LOC647200	0.9504	1.3155	0.9460	0.9198	1.0329	0.0944
LOC1001335	0.9939	1.1697	0.9726	0.9955	1.0329	0.0459
FBXW4	0.8892	1.4297	1.0288	0.7841	1.0329	0.1414
TIGA1	0.8784	1.5018	1.0299	0.7216	1.0329	0.1685
LOC641957	0.9202	1.1165	1.0050	1.0903	1.0330	0.0445
METTL7B	1.0600	1.0347	1.0597	0.9776	1.0330	0.0194
NPM1	0.9857	1.1526	1.0573	0.9364	1.0330	0.0470
LOC727855	1.0492	1.1273	1.0205	0.9350	1.0330	0.0397
TAF7	0.9302	1.2656	1.1510	0.7853	1.0330	0.1080
LOC645839	1.0314	1.1365	1.0575	0.9068	1.0330	0.0477
LOC644444	1.0544	1.0632	1.0633	0.9512	1.0330	0.0273
OXR1	0.9885	1.3266	0.9756	0.8414	1.0330	0.1034
CGB	0.9100	1.2743	1.0096	0.9382	1.0330	0.0831
AGXT2L2	0.9850	1.2688	1.0412	0.8373	1.0331	0.0896
LOC642014	0.9119	1.0429	1.0880	1.0898	1.0331	0.0419
TYROBP	0.9790	1.1169	1.1118	0.9249	1.0332	0.0482
LOC642863	0.9737	1.0381	1.2358	0.8853	1.0332	0.0744
KIAA1543	1.1460	1.1339	1.0434	0.8094	1.0332	0.0780
USP47	0.9102	1.1834	1.1436	0.8955	1.0332	0.0757
LOC647333	1.0218	1.0715	1.1770	0.8626	1.0332	0.0655
LOC1001337	0.9326	1.2095	1.1148	0.8759	1.0332	0.0778
DLGAP1	0.9973	1.1512	1.0135	0.9709	1.0332	0.0403
LOC1001317	0.9650	1.0666	1.2450	0.8562	1.0332	0.0826
AP3B2	0.9888	1.1376	1.0530	0.9534	1.0332	0.0404
GBP7	0.9002	1.1536	1.0915	0.9876	1.0332	0.0560
LOC648846	0.9388	1.1136	1.1518	0.9287	1.0332	0.0580
RBM4	0.8720	1.4959	0.9921	0.7729	1.0333	0.1606
CRIP2	0.9854	1.3453	1.0249	0.7775	1.0333	0.1173
LOC550631	1.1067	1.0842	1.0071	0.9350	1.0333	0.0391
FUT2	0.9752	1.0562	1.0810	1.0208	1.0333	0.0230
MRP63	0.8777	1.3757	1.1766	0.7032	1.0333	0.1503
LOC1001306	0.9646	1.0705	1.1899	0.9083	1.0333	0.0621
LOC391555	1.1501	0.9540	1.0374	0.9919	1.0333	0.0425
CEACAM18	1.0289	1.1471	1.0524	0.9050	1.0333	0.0498
LOC645451	1.0429	1.1044	0.9414	1.0448	1.0334	0.0338
LOC139587	1.0643	1.0479	1.1152	0.9061	1.0334	0.0448
LOC646176	0.9922	1.0604	1.2558	0.8252	1.0334	0.0891
LRTOMT	0.9990	1.1579	1.1450	0.8317	1.0334	0.0763

CECR6	0.9169	1.1957	1.0350	0.9862	1.0335	0.0593
C17orf108	1.0896	1.1146	1.0654	0.8642	1.0335	0.0573
FA2H	0.9614	1.1401	1.0865	0.9459	1.0335	0.0475
P4HA3	1.0744	1.0065	1.0318	1.0213	1.0335	0.0146
TSPAN12	1.0118	1.1332	1.1197	0.8694	1.0335	0.0611
LOC648374	1.1084	1.0349	0.9991	0.9917	1.0335	0.0267
LOC651581	0.9309	1.0617	1.0986	1.0431	1.0336	0.0361
LOC1001339	1.0134	0.9980	1.0590	1.0639	1.0336	0.0164
KRTHB6	0.9591	1.0330	1.1258	1.0164	1.0336	0.0346
C19orf47	1.0210	1.1181	1.1034	0.8919	1.0336	0.0519
LOC643505	1.0276	1.1759	0.9833	0.9476	1.0336	0.0502
FAM109B	0.9742	1.1504	1.1035	0.9062	1.0336	0.0565
LOC644316	0.8570	1.1885	1.0991	0.9898	1.0336	0.0715
LOC554203	0.9272	1.1283	1.2323	0.8468	1.0336	0.0888
LOC1001336	1.0831	1.0425	1.0762	0.9328	1.0337	0.0348
MIR337	0.9730	1.1045	1.0757	0.9815	1.0337	0.0332
GPR183	0.9754	1.1427	1.0289	0.9876	1.0337	0.0381
ANKRD55	0.9889	1.1147	1.0906	0.9404	1.0337	0.0413
SFTA2	1.0021	1.0911	1.1298	0.9117	1.0337	0.0486
LOC643787	0.9664	1.1847	1.0335	0.9501	1.0337	0.0535
BZRAP1	0.8641	1.1878	1.0774	1.0054	1.0337	0.0678
PCGF2	1.0342	1.3985	0.9183	0.7837	1.0337	0.1319
LOC1001335	0.9170	1.1326	1.0599	1.0254	1.0337	0.0449
WDR7	0.9118	1.3800	1.0145	0.8287	1.0337	0.1215
SOX2	1.0148	1.1238	1.0796	0.9168	1.0337	0.0450
LOC653720	0.8299	1.2763	1.0531	0.9757	1.0338	0.0932
UGT1A4	0.9638	1.1729	1.0719	0.9266	1.0338	0.0557
LOC1001332	1.0008	1.1267	1.0338	0.9739	1.0338	0.0333
ZCCHC5	1.0914	1.0917	1.0043	0.9479	1.0338	0.0353
LOC646344	0.9390	1.2665	0.9889	0.9409	1.0338	0.0784
C9orf61	1.0597	1.0895	1.0804	0.9060	1.0339	0.0431
ZCCHC3	0.9903	1.1592	1.1098	0.8762	1.0339	0.0634
TMEM66	0.9429	1.4131	1.0628	0.7168	1.0339	0.1453
LOC650771	0.9315	1.1526	1.0342	1.0172	1.0339	0.0455
CHPF2	0.9706	1.2399	0.9745	0.9506	1.0339	0.0688
ARMC4	0.9850	1.0544	1.0350	1.0613	1.0339	0.0172
STAT5B	1.1506	1.1555	0.9815	0.8482	1.0339	0.0739
LOC392395	1.0023	1.1312	1.0500	0.9523	1.0339	0.0381
GOLGA2LY2	0.9300	1.1838	1.0266	0.9954	1.0340	0.0538
OSTalpha	0.9436	1.2667	1.0126	0.9130	1.0340	0.0803
LOC402571	1.1540	1.0815	0.9338	0.9666	1.0340	0.0510
LOC1001305	1.0927	1.0368	1.0587	0.9477	1.0340	0.0310
SOX3	1.0286	1.0335	1.1409	0.9330	1.0340	0.0425
LOC646134	0.9593	1.1537	1.1360	0.8871	1.0340	0.0658
ARMS2	1.0602	1.1272	0.9923	0.9564	1.0340	0.0378
LOC652265	0.9415	1.2295	1.1082	0.8569	1.0340	0.0835

ANKS1A	0.9024	1.4459	1.0086	0.7793	1.0340	0.1450
LOC653288	1.0711	1.0637	1.0735	0.9279	1.0341	0.0354
OR5H6	1.0158	1.1377	1.0438	0.9389	1.0341	0.0411
MIR610	0.9821	1.1255	1.1538	0.8750	1.0341	0.0650
LOC644518	1.0009	1.0873	1.0392	1.0091	1.0341	0.0196
LOC1001303	1.0014	1.1567	0.9572	1.0213	1.0341	0.0430
C22orf34	0.9647	1.0516	1.0932	1.0271	1.0341	0.0269
GYS2	0.9917	1.1451	1.0468	0.9530	1.0341	0.0417
FAM155B	1.0522	1.1648	1.0525	0.8671	1.0341	0.0617
RIC3	0.9954	1.0661	1.1983	0.8771	1.0342	0.0672
CELA3B	0.9826	1.0822	1.0685	1.0037	1.0342	0.0243
LOC1001304	0.8571	1.4485	1.0853	0.7460	1.0342	0.1551
KIAA1429	0.9666	1.4275	1.0076	0.7353	1.0342	0.1442
CLCNKB	1.0400	1.0472	1.1197	0.9301	1.0343	0.0391
RSPRY1	0.8505	1.3993	1.0446	0.8427	1.0343	0.1303
GZMB	1.0089	1.1798	1.0085	0.9399	1.0343	0.0511
LOC554208	0.9067	1.3474	1.0326	0.8505	1.0343	0.1111
CHRM1	1.0036	1.1244	1.0184	0.9907	1.0343	0.0306
MIRLET7F2	0.9778	1.0827	1.1760	0.9007	1.0343	0.0602
PUS10	0.8709	1.2562	1.1166	0.8936	1.0343	0.0924
MIR1826	0.9343	1.1376	1.0216	1.0438	1.0343	0.0418
LIMK1	0.9962	1.2509	0.9834	0.9068	1.0343	0.0748
CYB561D1	0.9952	1.3906	0.9811	0.7704	1.0343	0.1294
MAPKBP1	1.0482	1.1540	1.0333	0.9020	1.0344	0.0517
MIR1207	0.9628	1.1077	1.0806	0.9865	1.0344	0.0353
LOC644989	1.0573	1.1059	1.0320	0.9425	1.0344	0.0342
LOC650214	0.9654	1.1149	1.0232	1.0341	1.0344	0.0308
JAKMIP2	1.0324	1.1046	1.1060	0.8947	1.0344	0.0496
OR10H2	0.9773	1.0276	1.1082	1.0248	1.0345	0.0271
LHFPL5	1.0609	1.1065	0.9568	1.0138	1.0345	0.0321
TMEM68	0.9640	1.1554	1.1109	0.9077	1.0345	0.0588
LOC642637	1.0105	1.2046	1.0136	0.9094	1.0345	0.0616
TMEM9	0.9047	1.4500	1.0208	0.7627	1.0345	0.1482
LOC136143	0.8750	1.3529	1.1928	0.7176	1.0346	0.1450
LOC1001281	0.9849	1.1495	1.0518	0.9521	1.0346	0.0436
LOC649667	0.9745	1.0845	1.0235	1.0558	1.0346	0.0236
NKX2-8	0.9972	1.1575	0.9904	0.9934	1.0346	0.0410
KRTAP1-3	1.0104	1.1524	1.0482	0.9275	1.0346	0.0467
DOCK6	1.0340	1.2791	1.0589	0.7666	1.0346	0.1049
SCGB1C1	1.0182	1.1104	1.0093	1.0007	1.0346	0.0255
INPP5D	1.0518	1.0688	1.0727	0.9454	1.0347	0.0301
CLCA1	0.9839	1.1660	1.0041	0.9847	1.0347	0.0440
CCDC3	0.9047	1.2725	1.0471	0.9145	1.0347	0.0857
MAGED1	0.9311	1.3829	1.0528	0.7719	1.0347	0.1296
LOC646283	1.0329	1.2123	1.0388	0.8547	1.0347	0.0730
SATB1	1.0197	1.1787	0.9996	0.9408	1.0347	0.0508

MIR606	1.0144	0.9705	1.1054	1.0486	1.0347	0.0285
LOC652839	0.9820	1.2097	1.0416	0.9056	1.0347	0.0646
LOC1001307	0.9365	1.1846	1.0553	0.9625	1.0347	0.0561
LOC728126	0.7950	1.5324	1.2499	0.5617	1.0347	0.2189
KRT32	0.9033	1.0973	1.1498	0.9886	1.0348	0.0552
LOC729974	1.0541	1.0851	1.0824	0.9175	1.0348	0.0397
RPL36	0.9403	1.2314	1.1770	0.7904	1.0348	0.1031
PLXND1	0.8739	1.2025	1.0926	0.9701	1.0348	0.0716
STMN3	1.0174	1.4078	0.9982	0.7158	1.0348	0.1422
SLC1A7	1.0031	1.2440	1.0089	0.8833	1.0348	0.0755
LEPRE1	0.9986	1.3674	0.9706	0.8027	1.0348	0.1190
C21orf33	0.8935	1.4803	1.0786	0.6870	1.0348	0.1687
CYLC2	1.0052	1.0179	1.0927	1.0236	1.0349	0.0197
LOC1001330	0.9297	1.1285	1.1039	0.9774	1.0349	0.0482
TTTY4C	0.9608	1.1758	1.0496	0.9533	1.0349	0.0518
MIR135B	1.1002	1.1410	0.9892	0.9092	1.0349	0.0528
DEFB107B	0.9497	1.0586	1.1038	1.0275	1.0349	0.0324
IL10RB	0.8924	1.3324	1.1588	0.7559	1.0349	0.1297
LOC729619	0.9198	1.1302	1.1952	0.8945	1.0349	0.0751
CDC40	0.9717	1.3853	0.9433	0.8394	1.0349	0.1202
PTH2R	0.9973	1.1269	1.0390	0.9766	1.0349	0.0333
LOC154872	0.9926	1.1816	0.9813	0.9843	1.0350	0.0489
LOC1001306	1.0230	1.0730	1.1260	0.9180	1.0350	0.0443
PARP9	1.0696	1.3853	0.9026	0.7825	1.0350	0.1308
LOC1001346	0.8081	1.3140	0.9903	1.0275	1.0350	0.1046
LOC284821	0.7365	1.3590	1.1318	0.9126	1.0350	0.1349
SLC2A11	1.0537	1.1741	1.0090	0.9033	1.0350	0.0561
LOC648852	0.9978	1.0095	1.3711	0.7618	1.0350	0.1257
LOC1001306	1.0537	1.0712	1.0017	1.0135	1.0350	0.0164
LOC653348	1.0513	1.1054	1.0594	0.9240	1.0350	0.0389
C7orf10	1.0890	1.1574	1.0363	0.8574	1.0350	0.0642
CCNYL2	0.9283	1.1357	1.1257	0.9506	1.0350	0.0554
CCND2	1.0562	1.1158	0.9892	0.9790	1.0351	0.0319
LOC650843	1.1435	1.0102	1.0195	0.9670	1.0351	0.0379
YKT6	0.9745	0.9965	1.2649	0.9044	1.0351	0.0791
LOC645359	0.9897	1.1971	0.9882	0.9658	1.0352	0.0543
C1orf105	1.0219	1.2071	1.0318	0.8800	1.0352	0.0670
GPER	0.9653	1.5518	0.9141	0.7095	1.0352	0.1808
NDUFA1	1.0473	1.3520	0.9361	0.8054	1.0352	0.1166
LOC643784	1.0264	1.0520	1.1193	0.9432	1.0352	0.0364
ADK	0.8399	1.3514	1.2060	0.7437	1.0352	0.1450
RFPL2	1.0529	1.0868	1.0700	0.9314	1.0353	0.0353
FAM135B	1.0049	1.1865	1.0879	0.8618	1.0353	0.0687
RPL18	0.9873	1.1284	1.1636	0.8619	1.0353	0.0692
ZNF579	0.9398	1.2095	1.0046	0.9874	1.0353	0.0597
LOC644632	1.0201	1.1553	1.0190	0.9470	1.0353	0.0435

LOC1001286	1.1232	1.1336	1.0064	0.8782	1.0353	0.0598
SNORD90	0.9385	1.1685	1.1044	0.9301	1.0354	0.0598
ZNF260	0.8594	1.5442	0.9467	0.7912	1.0354	0.1726
MIR1252	0.9750	1.2014	0.9436	1.0216	1.0354	0.0576
ZSCAN12	0.9184	1.1991	1.2640	0.7601	1.0354	0.1185
FAM54B	0.9246	1.3045	1.1429	0.7697	1.0354	0.1179
KCTD12	1.0233	1.0988	1.1177	0.9019	1.0354	0.0489
HS3ST5	0.9411	1.0895	1.1061	1.0050	1.0354	0.0385
TFE3	0.9091	1.2852	1.1099	0.8377	1.0355	0.1012
C11orf31	0.9532	1.2363	1.1800	0.7725	1.0355	0.1069
LOC1001325	0.9140	1.1214	1.1498	0.9568	1.0355	0.0587
MIR660	0.9572	1.0995	1.0659	1.0196	1.0355	0.0308
C7orf29	1.0323	1.1952	1.0708	0.8438	1.0355	0.0728
TAT	1.0784	1.0989	1.0168	0.9481	1.0355	0.0340
GNAS	0.9738	1.2210	1.1292	0.8181	1.0355	0.0886
LOC1001300	0.9863	1.0385	1.1102	1.0071	1.0355	0.0271
ASCL3	1.0154	1.1846	1.0589	0.8834	1.0356	0.0621
NRL	1.0100	1.0865	1.0589	0.9869	1.0356	0.0227
PAQR5	1.0249	1.1289	1.0259	0.9627	1.0356	0.0344
C17orf60	0.9780	1.0068	1.3400	0.8176	1.0356	0.1097
EPN3	1.2274	1.2611	0.9698	0.6842	1.0356	0.1340
EIF1AY	0.9400	1.3165	1.1794	0.7066	1.0356	0.1345
LOC646139	1.0841	1.1469	1.0363	0.8752	1.0356	0.0581
LOC652743	0.9422	1.0360	1.2431	0.9213	1.0356	0.0735
RBMS2	0.9413	1.2475	1.1305	0.8234	1.0357	0.0948
LOC1001284	0.8843	1.2958	1.0525	0.9101	1.0357	0.0943
WIPI2	1.0289	1.3190	1.0326	0.7622	1.0357	0.1137
IGSF8	0.9798	1.1003	1.1882	0.8744	1.0357	0.0687
GTDC1	0.9500	1.1533	1.1115	0.9281	1.0357	0.0566
CENTD2	0.9309	1.2879	1.0133	0.9110	1.0358	0.0869
LOC727924	0.9701	1.2829	1.0290	0.8612	1.0358	0.0894
LOC442710	0.9058	1.1041	1.0714	1.0620	1.0358	0.0443
JMY	0.9691	1.1946	1.1304	0.8492	1.0358	0.0782
PLA2G4A	1.0638	1.1005	0.9949	0.9842	1.0358	0.0278
LOC653275	0.9173	1.1899	1.1659	0.8702	1.0358	0.0827
LOC651192	1.0323	1.1363	1.0152	0.9596	1.0358	0.0369
KCNMB1	1.0436	1.0533	1.0567	0.9900	1.0359	0.0156
LOC134121	0.9640	1.1606	1.0678	0.9514	1.0360	0.0491
ANKRD37	1.2218	1.3019	0.8905	0.7297	1.0360	0.1355
TAOK2	1.0208	1.2576	1.0001	0.8654	1.0360	0.0815
LOC650428	1.0417	1.1594	0.9610	0.9818	1.0360	0.0446
LOC400759	0.9325	1.1073	1.1191	0.9852	1.0360	0.0459
ARFGEF1	0.9989	1.4506	0.9577	0.7368	1.0360	0.1497
LOC202051	0.9787	1.1942	1.1031	0.8681	1.0360	0.0713
LOC1001279	0.9427	1.2181	1.0548	0.9286	1.0360	0.0669
CSTF2T	0.9746	1.3041	1.1826	0.6830	1.0361	0.1359

LOC392100	0.9597	1.1387	1.0732	0.9727	1.0361	0.0426
LOC341112	0.9634	1.2076	1.0554	0.9180	1.0361	0.0639
ABTB1	1.1476	1.1377	1.0095	0.8496	1.0361	0.0697
GPR153	0.9980	1.1884	0.9512	1.0069	1.0361	0.0522
LOC644338	0.9436	1.2448	1.1334	0.8227	1.0361	0.0945
ST8SIA2	1.0611	1.0732	1.0574	0.9530	1.0361	0.0279
CREM	0.9839	1.0574	1.0924	1.0109	1.0362	0.0241
LOC643700	1.0738	1.0843	1.0310	0.9556	1.0362	0.0292
LOC401358	1.0019	1.1566	1.0279	0.9582	1.0362	0.0426
C4orf6	0.9379	1.1670	1.0477	0.9921	1.0362	0.0490
PI16	1.0011	1.1149	1.0221	1.0067	1.0362	0.0266
ZNF507	0.9186	1.2025	1.1252	0.8985	1.0362	0.0755
CCDC65	1.0632	1.1557	1.0384	0.8876	1.0362	0.0556
MIR934	0.9837	1.1760	1.1843	0.8011	1.0363	0.0911
RPS15A	0.9175	1.1864	1.1889	0.8524	1.0363	0.0884
TRYX3	0.9298	1.1027	1.1091	1.0035	1.0363	0.0429
GTF2H5	1.0225	1.2091	1.0877	0.8258	1.0363	0.0801
ADRA1B	0.9696	1.0561	1.0470	1.0725	1.0363	0.0229
DYNC2H1	0.9424	1.2555	1.0485	0.8989	1.0363	0.0795
PVRL3	1.0393	1.0871	1.1005	0.9184	1.0363	0.0415
LOC390231	0.9491	1.0645	1.1247	1.0071	1.0363	0.0377
ABHD12B	0.9866	1.1360	1.0743	0.9485	1.0363	0.0424
IQCF1	0.9017	1.1593	1.0757	1.0086	1.0363	0.0544
FOXO3	0.9678	1.4937	0.9104	0.7735	1.0364	0.1578
GALNT13	0.9675	1.2067	1.0880	0.8833	1.0364	0.0706
LOC1001297	1.0053	1.1038	1.0382	0.9981	1.0364	0.0241
TLN1	1.0115	1.3144	0.9837	0.8359	1.0364	0.1004
NCRNA0002	1.1166	1.0993	1.0713	0.8583	1.0364	0.0601
LOC642155	0.9380	1.1000	1.0878	1.0198	1.0364	0.0372
CX3CL1	0.9941	1.1216	1.0947	0.9353	1.0364	0.0435
KPNA4	0.8466	1.5176	1.0439	0.7375	1.0364	0.1725
TMEM63A	1.0683	1.1375	1.0400	0.8998	1.0364	0.0499
JSRP1	0.9351	1.2141	1.0030	0.9935	1.0364	0.0611
PTPMT1	0.8753	1.3050	1.2519	0.7136	1.0364	0.1440
C8orf78	0.9717	1.0174	1.1098	1.0470	1.0365	0.0290
LOC727908	0.9826	1.1591	1.0826	0.9215	1.0365	0.0527
LOC728492	1.1283	1.2497	1.0266	0.7413	1.0365	0.1085
LHX8	0.9532	1.0765	1.0991	1.0172	1.0365	0.0327
GRB2	0.8498	1.4731	1.0613	0.7620	1.0365	0.1585
LOC731641	1.1296	1.1028	0.9734	0.9403	1.0365	0.0468
LOC646168	1.0774	1.0655	1.0344	0.9689	1.0366	0.0243
KRTAP4-8	0.9299	1.0993	1.0728	1.0442	1.0366	0.0373
UCN	1.0577	1.1993	1.0363	0.8530	1.0366	0.0711
LOC338750	0.9501	1.0591	1.0699	1.0672	1.0366	0.0289
LIF	1.0831	1.3675	0.8944	0.8014	1.0366	0.1249
ASAP1IT1	1.0136	1.0960	1.1019	0.9348	1.0366	0.0395

FLJ45832	0.9807	1.2741	0.9918	0.8999	1.0366	0.0818
GLRA1	1.0081	1.0754	1.0546	1.0083	1.0366	0.0169
PPM1H	1.0297	1.2816	1.0795	0.7556	1.0366	0.1083
LOC441126	1.0587	1.2126	1.0633	0.8118	1.0366	0.0830
GPATCH2	1.1010	1.3524	0.9370	0.7561	1.0366	0.1266
MAGEB6	0.9903	1.0589	1.0733	1.0243	1.0367	0.0186
LOC1001334	0.9798	1.1169	1.1097	0.9403	1.0367	0.0450
HINT3	1.0149	1.3623	1.0374	0.7322	1.0367	0.1288
LOC649967	0.9960	1.0932	1.0713	0.9863	1.0367	0.0268
LOC645133	1.0191	1.1821	1.0811	0.8645	1.0367	0.0665
UCP1	1.0410	1.1350	1.0650	0.9060	1.0368	0.0479
C15orf54	0.9670	1.1385	1.0805	0.9612	1.0368	0.0436
RELL2	1.0356	1.2082	0.9859	0.9175	1.0368	0.0620
CASR	0.9637	1.1528	1.1431	0.8876	1.0368	0.0660
MIR1301	0.8884	1.1535	1.1241	0.9813	1.0368	0.0621
ATP4A	1.0828	1.1561	0.9966	0.9118	1.0368	0.0529
LOC650646	0.9798	1.2045	1.2271	0.7361	1.0369	0.1147
H2AFY2	0.9840	1.3608	0.9202	0.8827	1.0369	0.1100
LOC645888	1.0566	1.2034	1.0789	0.8088	1.0369	0.0826
PPP1R7	1.0963	1.5750	0.8196	0.6570	1.0370	0.2010
CXYorf2	0.9639	1.0160	1.2193	0.9487	1.0370	0.0625
FLJ46156	0.8970	1.1891	1.0550	1.0068	1.0370	0.0605
DCAF10	1.0533	1.2277	1.0639	0.8031	1.0370	0.0876
DNAI2	0.9461	1.2011	1.0778	0.9230	1.0370	0.0645
LOC728741	0.9848	1.1755	1.0138	0.9740	1.0370	0.0469
CCDC64	1.0496	1.0155	1.1016	0.9812	1.0370	0.0257
KISS1	0.9914	1.1055	1.0857	0.9655	1.0370	0.0345
HLA-A	1.0465	1.2966	1.0624	0.7427	1.0371	0.1135
LOC1001293	1.0979	1.0568	1.0736	0.9200	1.0371	0.0399
LRRC10	0.8944	1.1839	1.1507	0.9192	1.0371	0.0757
LOC652242	1.0948	1.1299	1.0330	0.8906	1.0371	0.0528
LOC646431	0.8886	1.1001	1.3282	0.8314	1.0371	0.1129
ADRA2B	0.8895	1.2583	1.0539	0.9467	1.0371	0.0812
GLB1	0.9253	1.4037	0.9981	0.8214	1.0371	0.1275
OR4C45	0.9289	1.2243	0.9950	1.0003	1.0371	0.0645
VSIG8	0.9395	1.0745	1.0631	1.0714	1.0371	0.0326
BTBD16	0.9989	1.0499	1.1034	0.9963	1.0371	0.0253
TBX21	0.9120	1.4212	0.9605	0.8551	1.0372	0.1298
MIR10B	1.0229	0.9689	1.1782	0.9786	1.0372	0.0485
FLJ44313	1.0386	1.1699	1.0222	0.9181	1.0372	0.0517
RPS27A	0.9717	1.1960	1.1207	0.8606	1.0372	0.0751
ODZ3	1.0423	1.0361	1.0294	1.0413	1.0373	0.0030
LRRC57	1.1567	1.2026	1.0116	0.7782	1.0373	0.0955
DYNLRB1	0.8941	1.3552	1.1589	0.7409	1.0373	0.1367
OR5P2	1.0516	1.0781	1.1558	0.8636	1.0373	0.0620
FLJ46363	0.8970	1.1140	1.1516	0.9865	1.0373	0.0586

PPP1R13L	1.0570	1.2451	0.9849	0.8623	1.0373	0.0801
ABHD4	1.0395	1.2618	0.9304	0.9176	1.0373	0.0797
LOC651055	1.0150	1.1972	1.0191	0.9181	1.0373	0.0582
TMEM8B	0.9717	1.3882	1.0054	0.7840	1.0373	0.1267
CCL18	0.9929	1.1255	1.0678	0.9631	1.0373	0.0367
MIR190B	1.0645	1.0621	1.0931	0.9298	1.0374	0.0365
FLJ46284	1.0128	1.1654	0.9776	0.9939	1.0374	0.0433
LOC653125	1.0048	1.1859	0.9593	0.9995	1.0374	0.0505
LOC1001316	0.9677	1.1926	1.0130	0.9763	1.0374	0.0527
LOC647540	1.0568	1.1390	1.1387	0.8151	1.0374	0.0766
LOC644681	0.9336	1.1272	1.0694	1.0195	1.0374	0.0410
LOC646079	0.9855	1.1734	1.1056	0.8852	1.0374	0.0639
LRRC26	0.8735	1.6683	0.9092	0.6988	1.0374	0.2152
CCNL2	0.8919	1.3593	1.0172	0.8815	1.0374	0.1116
LOC651175	0.9618	1.0254	1.1919	0.9708	1.0375	0.0534
SNORD111B	0.9878	1.1192	1.1142	0.9285	1.0375	0.0473
MIR335	0.9157	1.2235	0.9903	1.0203	1.0375	0.0658
ETNK2	1.0844	1.1101	1.0110	0.9443	1.0375	0.0375
ERCC-00138	0.9942	1.0754	1.0942	0.9862	1.0375	0.0276
LOC729130	0.9957	1.1917	1.0426	0.9200	1.0375	0.0573
WDR91	0.9269	1.3480	1.0561	0.8189	1.0375	0.1143
SCT	0.9862	1.2517	1.0129	0.8992	1.0375	0.0754
SETX	0.8740	1.2230	1.0567	0.9964	1.0375	0.0726
MIR422A	0.9334	1.1504	1.0372	1.0291	1.0375	0.0444
RRP7B	0.9408	1.3532	1.0796	0.7765	1.0375	0.1221
MTCH2	0.8662	1.5642	1.0087	0.7112	1.0375	0.1858
DGKD	1.1073	1.1350	1.0282	0.8797	1.0375	0.0573
LOC729574	1.0757	1.1244	1.0366	0.9135	1.0376	0.0451
LOC645174	0.9700	1.2221	1.1511	0.8070	1.0376	0.0934
RNU4ATAC	0.5868	1.3879	1.3648	0.8108	1.0376	0.2009
SPERT	1.0213	1.1470	0.9446	1.0374	1.0376	0.0417
ZNF224	1.0846	1.1046	1.0806	0.8806	1.0376	0.0526
CLEC4E	0.9421	1.0804	1.1677	0.9602	1.0376	0.0531
GPC6	0.8081	1.3286	1.0122	1.0016	1.0376	0.1077
LOC645534	0.9939	1.0577	1.1114	0.9877	1.0377	0.0292
LOC642636	0.9599	1.1176	1.0409	1.0325	1.0377	0.0322
LOC647656	0.8761	1.2977	1.0581	0.9190	1.0377	0.0950
LOC1001309	0.9492	1.1397	1.1338	0.9285	1.0378	0.0573
LOC649942	0.9414	1.1397	1.0705	0.9998	1.0378	0.0430
OR5B2	1.0062	1.2325	0.9910	0.9217	1.0379	0.0674
WBSCR23	1.0987	1.0628	1.1258	0.8641	1.0379	0.0593
LOC643930	1.0147	1.1733	1.0662	0.8973	1.0379	0.0573
LOC158345	0.7171	1.6889	1.1025	0.6430	1.0379	0.2392
LOC729301	0.9539	1.2712	1.1510	0.7756	1.0379	0.1092
BET1L	1.1702	1.2333	0.9344	0.8140	1.0380	0.0985
METTL7A	0.8534	1.2516	1.3066	0.7402	1.0380	0.1416

MEGF9	0.9830	1.2395	1.0011	0.9283	1.0380	0.0689
CDH2	1.0323	1.0435	0.9881	1.0881	1.0380	0.0205
C1QB	1.0155	1.0754	1.0325	1.0286	1.0380	0.0130
OTUD7B	0.9993	1.1898	1.0704	0.8926	1.0380	0.0624
LOC643790	0.7264	1.5659	1.0416	0.8183	1.0381	0.1880
FAM32A	1.0702	1.5127	0.8486	0.7206	1.0381	0.1739
LOC727768	1.1433	1.1942	0.9036	0.9112	1.0381	0.0762
LOC388514	1.0314	1.1000	1.1092	0.9117	1.0381	0.0455
LOC642359	0.9515	1.0421	1.1677	0.9911	1.0381	0.0470
BCL7A	0.9889	1.1334	1.0517	0.9783	1.0381	0.0357
LOC645262	0.9637	1.1334	1.0746	0.9807	1.0381	0.0400
LOC1001326	0.9133	1.1646	1.0909	0.9837	1.0381	0.0558
LOC648796	1.1084	0.9730	1.0362	1.0349	1.0381	0.0277
MIAT	0.9234	1.2196	1.0557	0.9538	1.0381	0.0668
LOC646294	0.9858	1.2711	1.1023	0.7933	1.0381	0.1004
TTC5	0.9951	1.4490	0.9234	0.7853	1.0382	0.1437
TP53I3	0.8994	1.2289	1.1063	0.9182	1.0382	0.0789
DNAJC4	1.0282	1.5048	0.8725	0.7474	1.0382	0.1658
LOC390551	0.9153	1.1315	1.1259	0.9802	1.0382	0.0539
LOC729023	1.0636	0.9871	1.1520	0.9502	1.0382	0.0447
PCDHB10	1.0027	1.1403	1.1060	0.9041	1.0383	0.0534
FAM177A1	0.9128	1.4956	1.0481	0.6966	1.0383	0.1688
IP6K2	0.9033	1.5660	0.9449	0.7390	1.0383	0.1814
LOC147645	1.0485	1.2462	0.9102	0.9483	1.0383	0.0752
LOC1001317	0.8586	1.2448	1.0567	0.9932	1.0383	0.0803
TLL1	0.9378	1.0803	1.3447	0.7906	1.0383	0.1180
TYRO3P	1.0322	1.1240	1.0943	0.9029	1.0384	0.0490
LOC650634	0.9260	1.0954	1.0122	1.1201	1.0384	0.0440
LOC647886	0.9609	1.1557	1.1152	0.9219	1.0384	0.0572
LOC647096	0.9272	1.1470	1.2132	0.8663	1.0384	0.0838
ALAS1	0.9701	1.1617	1.0890	0.9330	1.0384	0.0529
SCGB3A2	1.0368	1.0333	1.0585	1.0252	1.0385	0.0071
TRIM2	1.0448	1.1027	1.0714	0.9351	1.0385	0.0364
LOC642149	0.9927	1.1223	1.0833	0.9557	1.0385	0.0387
PRR12	1.1102	1.0880	1.0166	0.9392	1.0385	0.0387
CCDC33	0.9250	1.2299	1.0152	0.9840	1.0385	0.0665
LOC649659	0.8878	1.3080	1.0450	0.9132	1.0385	0.0962
TFAMP1	1.0351	1.1718	1.0223	0.9249	1.0385	0.0508
KRTAP24-1	0.9249	1.2157	1.1327	0.8809	1.0385	0.0806
OR4F29	0.9697	1.2378	1.0446	0.9020	1.0385	0.0725
CLEC12B	1.0392	1.1317	0.9683	1.0151	1.0385	0.0344
RNU1G2	0.9738	1.1394	1.1665	0.8746	1.0385	0.0693
DCAF6	0.9777	1.3072	1.0658	0.8035	1.0386	0.1048
LOC1001330	0.9945	1.1498	1.0743	0.9358	1.0386	0.0467
MIR1308	1.0382	1.1726	1.0457	0.8979	1.0386	0.0561
HIST2H2AB	1.1743	1.0961	1.0972	0.7869	1.0386	0.0859

FICD	0.9913	1.3139	0.9922	0.8571	1.0386	0.0971
LOC643330	1.0127	1.1968	1.0386	0.9064	1.0386	0.0600
PDS5B	0.9315	1.4251	1.0407	0.7573	1.0386	0.1414
LOC1001312	0.9858	1.0895	1.0943	0.9850	1.0387	0.0308
SCRN2	1.0527	1.1247	1.0539	0.9233	1.0387	0.0420
FLJ32955	1.0973	1.0791	1.0238	0.9545	1.0387	0.0321
TNFAIP8	0.9786	1.3598	0.9871	0.8294	1.0387	0.1130
LOC1001301	1.1142	1.0809	1.1085	0.8512	1.0387	0.0629
LOC374395	0.9047	1.4052	1.1906	0.6543	1.0387	0.1641
PPAPDC1B	0.7475	1.4703	1.1952	0.7419	1.0387	0.1788
LOC651721	0.9248	1.1462	1.2375	0.8464	1.0387	0.0917
TTC7A	1.1585	1.1102	1.0083	0.8780	1.0387	0.0621
SCN8A	0.9805	1.2192	0.9566	0.9988	1.0388	0.0608
SNIP	0.9792	1.1552	1.0458	0.9750	1.0388	0.0421
ACOX3	0.8104	1.3923	1.0912	0.8612	1.0388	0.1327
C5orf27	0.9185	1.3026	1.0489	0.8851	1.0388	0.0948
CCL13	0.9833	1.1388	1.1403	0.8928	1.0388	0.0610
XPO5	0.8886	1.4229	1.0268	0.8169	1.0388	0.1352
LOC642337	0.9628	1.1705	1.0788	0.9430	1.0388	0.0531
FAM129B	0.9208	1.5524	0.9793	0.7026	1.0388	0.1813
MIR1289-2	0.9925	1.1241	1.0202	1.0184	1.0388	0.0291
LOC646400	0.9916	1.0816	1.1390	0.9431	1.0388	0.0440
RNU1-3	0.9101	1.2280	1.1000	0.9172	1.0388	0.0768
LOC643772	0.9772	1.1249	1.1077	0.9456	1.0389	0.0453
GSK3A	0.9304	1.2009	1.1112	0.9129	1.0389	0.0702
LOC1001306	0.9784	1.1641	1.0159	0.9971	1.0389	0.0424
LOC648057	1.0236	1.0912	1.1440	0.8966	1.0389	0.0534
LOC650468	0.9501	1.0786	1.1831	0.9437	1.0389	0.0572
FLJ43987	1.1251	1.1311	1.0217	0.8777	1.0389	0.0593
AKR1B10	1.0194	1.0384	1.1372	0.9607	1.0389	0.0367
LOC642282	1.0334	1.1148	1.0987	0.9090	1.0390	0.0468
LOC653182	1.0352	1.1809	1.0284	0.9114	1.0390	0.0552
LIPI	1.0288	1.0945	0.9945	1.0381	1.0390	0.0207
ID4	0.9348	1.1193	1.1233	0.9786	1.0390	0.0484
CYB561	0.9608	1.4742	0.9732	0.7479	1.0390	0.1540
CASK	0.8022	1.5058	0.9561	0.8919	1.0390	0.1588
LOC652753	1.0411	1.0484	1.1678	0.8987	1.0390	0.0550
LOC1001336	0.8726	1.2567	1.0749	0.9519	1.0390	0.0837
MIR33A	1.0529	1.0307	1.1115	0.9611	1.0390	0.0311
LOC643143	1.0198	1.1549	1.0170	0.9646	1.0391	0.0407
LOC645645	1.0053	1.0722	1.1345	0.9444	1.0391	0.0411
OR6C2	1.0468	1.1315	1.0830	0.8952	1.0391	0.0510
CYP8B1	1.0977	1.0807	0.9745	1.0036	1.0391	0.0297
DGAT2L3	0.9602	1.1124	1.0567	1.0273	1.0391	0.0317
ST6GALNAC3	0.9984	1.0349	1.1143	1.0091	1.0392	0.0262
EXT1	1.0647	1.2091	0.9899	0.8931	1.0392	0.0666

LOC650061	1.1036	1.0844	0.9793	0.9896	1.0392	0.0319
LOC642228	1.0381	1.1098	1.0368	0.9722	1.0392	0.0281
LOC646376	0.8863	1.0409	1.2244	1.0053	1.0392	0.0700
LYVE1	0.9789	1.1543	1.0866	0.9373	1.0392	0.0496
C15orf26	0.8915	1.1112	1.1053	1.0491	1.0392	0.0512
MIR589	0.9413	1.1278	1.0386	1.0493	1.0393	0.0382
NUP93	0.8811	1.5905	0.9989	0.6865	1.0393	0.1947
ANTXR2	0.8437	1.2760	1.1155	0.9219	1.0393	0.0974
LOC728887	1.0960	1.0014	1.0966	0.9632	1.0393	0.0338
LOC400890	0.9811	1.5282	0.9462	0.7019	1.0393	0.1744
ERCC-00117	0.9008	1.2131	1.0493	0.9941	1.0393	0.0655
SERPINA7	0.9689	1.0991	1.1329	0.9565	1.0393	0.0449
CSH2	0.9635	1.1534	1.1125	0.9280	1.0393	0.0552
HDX	0.9509	1.2643	1.0147	0.9275	1.0393	0.0772
ARHGEF17	0.9257	1.3650	0.9924	0.8744	1.0394	0.1112
C22orf28	1.0161	1.4064	1.1408	0.5941	1.0394	0.1693
LOC644625	0.8892	1.2548	1.0684	0.9455	1.0395	0.0809
ERN2	0.7784	1.2531	1.1741	0.9524	1.0395	0.1078
SGPP1	0.7726	1.5067	1.0799	0.7988	1.0395	0.1706
GCNT7	0.9430	1.1909	1.0058	1.0183	1.0395	0.0531
LOC1001317	1.0276	1.1707	1.0589	0.9008	1.0395	0.0555
SVEP1	0.9482	1.1167	1.1095	0.9836	1.0395	0.0431
NMUR2	1.0306	1.0939	1.1375	0.8962	1.0395	0.0526
PCDHGB6	1.0091	1.2211	1.0264	0.9016	1.0396	0.0665
ZFHX2	0.8700	1.1297	1.1231	1.0355	1.0396	0.0605
LOC1001315	0.9796	1.1790	1.0354	0.9645	1.0396	0.0489
MYRIP	1.0840	1.1806	1.0796	0.8143	1.0396	0.0786
LOC1001291	0.9721	1.0791	1.0976	1.0097	1.0396	0.0294
DEFA1	0.9141	1.3638	0.9924	0.8883	1.0396	0.1103
LCE2D	0.8370	1.2319	1.1194	0.9703	1.0397	0.0862
LOC1001332	1.0049	1.0959	1.0422	1.0157	1.0397	0.0203
LOC1001303	1.0840	1.1019	1.0542	0.9186	1.0397	0.0415
PLCD1	0.9197	1.3046	1.0003	0.9342	1.0397	0.0900
OR4E2	0.9615	1.1338	1.0066	1.0568	1.0397	0.0369
LOC1001328	1.0472	1.1222	1.0236	0.9658	1.0397	0.0324
ENPP3	1.0135	1.1149	1.0718	0.9586	1.0397	0.0341
SNORD48	0.7584	1.1914	1.3267	0.8824	1.0397	0.1320
WWC2	0.9428	1.1599	1.0786	0.9778	1.0398	0.0493
LOC388333	0.9080	1.0949	1.1260	1.0303	1.0398	0.0482
LOC647485	0.9253	1.1796	1.1372	0.9171	1.0398	0.0690
MIR708	1.0351	1.1431	1.1409	0.8402	1.0398	0.0712
MIP	1.0283	1.1692	1.0117	0.9501	1.0398	0.0463
LOC1001343	1.0140	1.1635	1.1681	0.8137	1.0398	0.0834
CUX2	1.0642	1.1685	1.0621	0.8648	1.0399	0.0634
P2RXL1	1.0785	1.0806	1.1034	0.8971	1.0399	0.0479
LOC728510	1.1202	1.1560	0.9933	0.8903	1.0399	0.0609

XAB2	0.9128	1.4192	1.1761	0.6516	1.0399	0.1657
KRTAP6-1	0.9581	1.1046	1.1935	0.9036	1.0399	0.0665
APBB1IP	0.9705	1.1872	1.0575	0.9446	1.0399	0.0547
LOC1001279	1.1638	1.0901	1.0299	0.8760	1.0400	0.0611
LIMK2	0.9840	1.2124	1.1109	0.8525	1.0400	0.0780
LOC1001323	0.9350	1.2030	0.9798	1.0421	1.0400	0.0586
KRTAP5-5	0.9906	1.1886	1.0227	0.9582	1.0400	0.0513
LOC1001291	0.9605	1.1491	1.1550	0.8958	1.0401	0.0660
C6orf224	1.1340	1.1217	1.0790	0.8256	1.0401	0.0725
LUZP4	1.0431	1.1201	1.0153	0.9819	1.0401	0.0295
LOC441868	0.8500	1.1650	1.1580	0.9873	1.0401	0.0755
LOC646071	1.1256	1.1131	0.9587	0.9630	1.0401	0.0458
TCEA2	0.9286	1.3762	1.0296	0.8259	1.0401	0.1195
EPHA4	1.0899	1.1197	1.0427	0.9083	1.0401	0.0467
NCKIPSD	0.9676	1.2505	1.1066	0.8359	1.0401	0.0893
LOC643771	0.9763	1.2111	1.0394	0.9338	1.0402	0.0610
MICAL2	0.9041	1.2872	1.0697	0.8998	1.0402	0.0913
LOC643805	1.0611	1.1652	1.0711	0.8635	1.0402	0.0634
LOC374920	0.9658	1.1464	1.0412	1.0076	1.0402	0.0386
THSD4	1.0313	1.1824	1.0539	0.8936	1.0403	0.0592
C3orf23	0.9207	1.4744	0.9693	0.7967	1.0403	0.1492
SCN7A	1.0091	1.1531	1.0696	0.9294	1.0403	0.0473
PLEKHO2	1.0020	1.2709	1.0305	0.8578	1.0403	0.0857
COMMD7	0.8858	1.5907	0.9439	0.7408	1.0403	0.1884
LOC440027	0.9678	1.1097	1.1047	0.9791	1.0403	0.0387
MIR519D	1.0213	1.1266	1.0278	0.9856	1.0403	0.0302
KCNE1	0.9131	1.1740	1.1468	0.9274	1.0403	0.0696
LOC254312	0.9572	1.0494	1.1912	0.9635	1.0403	0.0545
TNNC1	0.9183	1.2425	1.0607	0.9398	1.0403	0.0743
LOC642559	0.9983	1.0988	1.0528	1.0115	1.0403	0.0227
C6orf190	1.0162	1.0794	1.1032	0.9627	1.0404	0.0317
LOC1001334	0.9461	1.2741	1.0258	0.9156	1.0404	0.0813
SUCNR1	0.9122	1.0839	1.1428	1.0227	1.0404	0.0493
DGKK	1.0600	1.0149	1.0326	1.0542	1.0404	0.0104
LOC641941	1.0183	1.0930	1.1577	0.8927	1.0405	0.0569
ZDHHC18	0.8835	1.2637	1.0509	0.9639	1.0405	0.0819
BTBD12	0.9423	1.5247	0.9690	0.7260	1.0405	0.1703
C9orf7	1.0922	1.4089	0.8179	0.8430	1.0405	0.1375
DGKA	1.0326	1.1829	1.0666	0.8800	1.0405	0.0624
ZNF701	0.9672	1.2749	1.0752	0.8448	1.0405	0.0912
LOC1001298	0.9453	1.1982	1.0755	0.9433	1.0406	0.0610
LOC1001302	1.0113	1.2056	1.0142	0.9312	1.0406	0.0583
ALDH3A2	1.0962	1.4547	0.8988	0.7125	1.0406	0.1587
PEAR1	0.8828	1.1904	1.1315	0.9576	1.0406	0.0722
SCD	0.9055	1.4139	1.1211	0.7218	1.0406	0.1488
DPYD	1.0622	1.1355	1.0263	0.9383	1.0406	0.0410

DYNC1LI1	0.8992	1.1732	1.0848	1.0052	1.0406	0.0583
VTI1A	1.1235	1.0649	0.9563	1.0177	1.0406	0.0355
OR13C9	0.9627	1.1824	1.0865	0.9308	1.0406	0.0580
PRAMEF19	1.0697	1.0536	1.0721	0.9671	1.0406	0.0248
FAS	0.9986	1.2943	1.0482	0.8214	1.0406	0.0976
OLAH	0.9885	1.1386	1.0299	1.0056	1.0406	0.0337
C7orf16	0.9518	1.2433	0.9945	0.9731	1.0407	0.0681
TNK2	1.0884	1.2926	0.9649	0.8169	1.0407	0.1007
LOC650243	1.1280	1.0114	1.0248	0.9986	1.0407	0.0296
LOC648454	0.9909	1.1147	1.0528	1.0046	1.0407	0.0280
SCRT2	1.0485	1.1589	0.9926	0.9631	1.0408	0.0432
RDH12	0.9084	1.2634	1.1203	0.8710	1.0408	0.0923
ARAP1	1.0831	1.3314	0.8076	0.9409	1.0408	0.1120
LOC442096	0.9934	1.2646	0.9917	0.9134	1.0408	0.0769
U2AF1L2	0.9556	1.3194	1.1826	0.7056	1.0408	0.1346
GRB10	1.0864	1.3501	0.9216	0.8051	1.0408	0.1181
LOC648149	1.0334	1.1194	1.0299	0.9806	1.0408	0.0288
TEKT5	1.0021	1.1297	1.0150	1.0165	1.0408	0.0298
FAM134C	1.0313	1.2632	0.9966	0.8723	1.0408	0.0816
LOC647103	1.0071	1.1097	1.0631	0.9834	1.0408	0.0284
DGKE	0.9459	1.1302	1.1603	0.9270	1.0409	0.0607
SLC6A2	1.0156	1.1505	1.0394	0.9580	1.0409	0.0403
C9orf153	1.0603	1.0893	1.1074	0.9067	1.0409	0.0458
TADA3	1.0275	1.3967	0.9935	0.7461	1.0409	0.1341
LOC649360	0.9795	1.1843	1.0281	0.9718	1.0409	0.0494
FLJ45244	1.0535	1.1285	1.1616	0.8202	1.0409	0.0770
LOC1001327	0.8172	1.6907	0.9066	0.7494	1.0410	0.2189
LOC727737	1.0279	1.2370	0.9099	0.9893	1.0410	0.0698
TERC	0.9395	1.2322	1.0887	0.9038	1.0411	0.0753
C19orf33	0.9431	1.3908	1.0345	0.7959	1.0411	0.1265
LOC1001342	1.1168	1.1234	0.9829	0.9411	1.0411	0.0465
UCKL1OS	0.9544	1.1136	0.9923	1.1040	1.0411	0.0399
LOC643722	0.9943	1.2582	1.0433	0.8686	1.0411	0.0812
LOC1001290	1.0221	1.0635	1.1021	0.9769	1.0411	0.0269
ARHGAP29	0.9483	1.1342	1.1190	0.9631	1.0411	0.0495
PCGF3	1.0544	1.1226	1.1339	0.8539	1.0412	0.0649
ANKRD33	0.7167	1.4566	1.1967	0.7947	1.0412	0.1739
LOC654055	1.0974	1.1380	1.0520	0.8774	1.0412	0.0573
LOC648147	0.8990	1.0317	1.0069	1.2274	1.0412	0.0684
TMEM65	1.1001	0.9863	1.2452	0.8333	1.0412	0.0872
CNGA2	0.9563	1.2433	0.9308	1.0346	1.0412	0.0709
LOC653852	1.0015	1.1047	1.1779	0.8808	1.0412	0.0646
PLCXD3	0.9521	1.2131	1.1391	0.8608	1.0413	0.0815
LOC440487	0.9516	1.3728	1.0599	0.7808	1.0413	0.1246
LOC1001284	0.9082	1.1678	1.1339	0.9553	1.0413	0.0643
SGSM3	0.9474	1.4066	0.9498	0.8616	1.0413	0.1235

RAB17	0.9100	1.4306	1.0135	0.8112	1.0413	0.1362
SAMD7	1.0020	1.3200	0.9122	0.9312	1.0413	0.0949
AP3S2	0.9299	1.3610	1.0557	0.8187	1.0413	0.1170
RPL4	0.8979	1.2974	1.1830	0.7871	1.0413	0.1193
GYPB	0.9423	1.2693	0.9580	0.9958	1.0414	0.0768
ZBTB10	0.7765	1.3785	1.2379	0.7726	1.0414	0.1567
BLVRB	0.9062	1.3730	1.1046	0.7816	1.0414	0.1290
C19orf66	1.0134	1.1444	1.0821	0.9256	1.0414	0.0469
LOC651004	0.9566	1.1550	1.1190	0.9349	1.0414	0.0559
BRWD2	1.1342	1.2177	1.0362	0.7774	1.0414	0.0955
LOC391157	1.0744	1.1768	1.0657	0.8486	1.0414	0.0690
IL17RC	1.1117	1.1611	1.0055	0.8873	1.0414	0.0608
PI4KAP1	1.1888	1.2435	0.9364	0.7971	1.0414	0.1054
LOC648262	0.9401	1.1229	1.0854	1.0174	1.0415	0.0402
RGS7	1.0904	1.0561	1.1087	0.9107	1.0415	0.0449
LOC1001279	0.9469	1.0951	1.1792	0.9447	1.0415	0.0578
FTMT	1.2482	1.1888	0.8454	0.8836	1.0415	0.1032
SFMBT2	0.9578	1.2295	1.0716	0.9071	1.0415	0.0715
CALCR	1.0355	1.1514	1.0209	0.9584	1.0416	0.0402
TPPP	0.9988	1.0970	1.1679	0.9025	1.0416	0.0579
GADL1	0.9597	1.2134	1.0530	0.9402	1.0416	0.0623
PSMA8	0.9791	1.1759	1.0812	0.9302	1.0416	0.0547
GGT3P	0.9392	1.0526	1.1866	0.9879	1.0416	0.0536
SLFNL1	1.0376	1.1273	1.0199	0.9817	1.0416	0.0308
ITGAD	1.0118	1.0792	1.0531	1.0225	1.0417	0.0153
LOC650354	0.9953	1.1333	1.1029	0.9352	1.0417	0.0462
ARHGEF5	1.1452	1.3418	0.8841	0.7957	1.0417	0.1245
LOC643770	0.9321	1.0743	1.0329	1.1275	1.0417	0.0413
TP53TG5	1.0797	0.9939	1.0257	1.0684	1.0419	0.0198
CGRRF1	0.7690	1.4461	1.1673	0.7854	1.0419	0.1631
ZNF721	1.0394	1.3702	1.0692	0.6891	1.0420	0.1393
GABRR2	0.9979	1.0401	1.0698	1.0602	1.0420	0.0160
TLE6	1.0144	1.3304	0.9500	0.8733	1.0420	0.1004
LOC257358	0.9309	1.1966	1.1116	0.9291	1.0420	0.0670
LOC1001323	1.0343	1.0378	1.0119	1.0843	1.0421	0.0152
LOC643837	1.0431	1.1094	1.1004	0.9155	1.0421	0.0447
PLEKHM1	0.9915	1.2467	1.0220	0.9082	1.0421	0.0723
LOC1001335	1.0275	1.0397	0.9829	1.1183	1.0421	0.0282
LOC732206	0.9422	1.0844	0.9924	1.1494	1.0421	0.0463
EPX	0.8834	1.2543	1.1089	0.9219	1.0421	0.0862
SNORD43	0.8529	1.1137	1.0422	1.1597	1.0421	0.0675
LOC1001308	0.9389	1.1018	1.1829	0.9450	1.0421	0.0602
LOC728863	1.0244	1.1033	1.1140	0.9271	1.0422	0.0433
ZFP91	0.9461	1.3438	1.0749	0.8040	1.0422	0.1148
ZNF512B	1.0951	1.1700	1.0524	0.8513	1.0422	0.0681
LILRA2	0.9970	1.1214	1.0245	1.0260	1.0422	0.0272

LOC643464	1.1089	1.1253	1.0851	0.8496	1.0422	0.0647
DAPK3	0.9366	1.2404	1.0891	0.9028	1.0422	0.0775
FLJ21687	0.9397	1.1334	1.1498	0.9461	1.0422	0.0575
LOC732391	1.0168	1.1057	1.1007	0.9459	1.0423	0.0381
CHMP1B	0.9488	1.3605	0.9779	0.8819	1.0423	0.1080
ZNF440	1.0785	1.0807	1.0727	0.9374	1.0423	0.0350
FLJ23754	0.9777	1.1570	1.1610	0.8736	1.0423	0.0706
MST1	1.0792	1.3421	1.0142	0.7338	1.0423	0.1249
KCTD8	1.0402	1.3105	0.9980	0.8209	1.0424	0.1012
HIST1H3E	1.1613	1.0861	1.1051	0.8171	1.0424	0.0768
RUNDC2C	1.1074	1.1400	1.0854	0.8368	1.0424	0.0694
C21orf29	0.9668	1.1573	1.2141	0.8318	1.0425	0.0879
KRTAP10-8	0.9493	1.1442	1.0250	1.0515	1.0425	0.0402
LOC647647	0.9556	1.1179	1.1120	0.9846	1.0425	0.0423
GNG11	0.9903	1.1759	1.0340	0.9700	1.0426	0.0464
LCN1	1.0653	1.0599	1.1023	0.9428	1.0426	0.0346
OSR2	0.7738	1.3857	1.1851	0.8257	1.0426	0.1464
LOC441013	1.0690	1.1381	1.1090	0.8542	1.0426	0.0644
TMEM178	1.0446	1.1822	1.0951	0.8487	1.0426	0.0706
NEK8	0.9771	1.1601	1.1478	0.8858	1.0427	0.0669
OR10X1	0.9629	1.1813	1.0817	0.9447	1.0427	0.0553
MIR767	1.1232	1.0608	1.0270	0.9598	1.0427	0.0341
SLC19A2	0.9042	1.4315	1.0222	0.8130	1.0427	0.1365
UBC	0.9529	1.2316	1.1539	0.8324	1.0427	0.0914
ZFP2	0.9767	1.2642	1.0224	0.9077	1.0428	0.0775
LOC643338	1.0667	1.1203	1.0795	0.9045	1.0428	0.0475
ZNF84	0.9836	1.2847	1.0317	0.8712	1.0428	0.0873
FGF10	1.0106	1.0847	1.1085	0.9674	1.0428	0.0327
WDR5B	1.0808	1.1318	1.1031	0.8556	1.0428	0.0633
C10orf10	1.0591	1.2002	1.0447	0.8673	1.0428	0.0682
ZNF182	0.9411	1.2913	1.0263	0.9130	1.0429	0.0862
ABHD1	1.0598	1.2359	1.0555	0.8206	1.0429	0.0852
DDX1	0.9058	1.5558	1.0263	0.6837	1.0429	0.1851
TBC1D10B	1.0821	1.2746	1.0764	0.7387	1.0430	0.1114
MME	0.9179	1.3518	1.1356	0.7666	1.0430	0.1278
MGC34774	1.1294	1.1417	1.0426	0.8583	1.0430	0.0654
FLJ36070	1.0439	0.9773	1.1646	0.9862	1.0430	0.0431
LOC1001304	1.1153	1.1000	1.0157	0.9411	1.0430	0.0404
LOC1001297	0.8468	1.1726	1.5208	0.6319	1.0430	0.1942
SNORD74	0.8401	1.1915	1.3228	0.8179	1.0431	0.1265
CNTN3	1.0373	1.1202	1.0183	0.9966	1.0431	0.0270
FLJ41046	0.9165	1.2165	1.0706	0.9688	1.0431	0.0661
COL1A2	0.9509	1.1567	1.0794	0.9855	1.0431	0.0466
ERN1	1.1259	1.3859	0.8749	0.7859	1.0431	0.1350
ZBTB47	0.9605	1.4248	0.9007	0.8865	1.0431	0.1282
LOC1001339	0.9390	1.2916	1.0579	0.8842	1.0432	0.0904

RAB24	1.0191	1.3593	0.9330	0.8612	1.0432	0.1102
SLC6A10P	1.2069	1.3935	0.8605	0.7118	1.0432	0.1562
LOC645100	0.9521	1.3316	0.9454	0.9437	1.0432	0.0962
DIO3OS	0.9380	1.1468	1.0640	1.0241	1.0432	0.0434
GOLGA8A	0.9766	1.0666	1.1674	0.9624	1.0432	0.0474
PRR15L	0.8863	1.3814	1.1315	0.7740	1.0433	0.1352
DKFZp686O1	1.0298	1.1127	1.1072	0.9235	1.0433	0.0442
RNF11	0.9360	1.4895	1.0810	0.6668	1.0433	0.1717
LOC340511	1.0744	1.0644	1.1404	0.8941	1.0433	0.0525
GIPC1	1.0133	1.3184	1.0610	0.7807	1.0434	0.1102
BBS2	1.0432	1.4302	0.9956	0.7046	1.0434	0.1491
STX16	0.8686	1.4971	1.0149	0.7931	1.0434	0.1581
LOC729537	0.9636	1.1001	1.1221	0.9880	1.0434	0.0396
ARL5C	0.9827	1.1640	0.9539	1.0735	1.0435	0.0476
ZNF784	1.0567	1.4299	0.8545	0.8328	1.0435	0.1383
MIR1827	1.0343	1.0654	1.1272	0.9471	1.0435	0.0375
C14orf4	0.8847	1.3115	1.2410	0.7372	1.0436	0.1384
SNORD119	1.0201	1.0705	1.0977	0.9861	1.0436	0.0250
CYP27A1	0.8931	1.3174	1.1414	0.8226	1.0436	0.1140
LOC440509	0.8830	1.1498	0.9656	1.1761	1.0436	0.0711
SERGEF	0.8989	1.5044	0.9829	0.7883	1.0436	0.1587
PSMB4	0.7913	1.5375	1.1568	0.6890	1.0437	0.1928
PRR15	1.0376	1.1174	1.0511	0.9687	1.0437	0.0305
HBG1	1.0061	1.1055	1.0743	0.9890	1.0437	0.0276
LOC728607	1.0073	1.1594	1.1239	0.8844	1.0438	0.0623
LOC643284	1.0221	1.2021	1.1539	0.7971	1.0438	0.0906
AKR1B15	1.0144	1.2026	0.9643	0.9940	1.0438	0.0539
NME3	0.9188	1.4456	1.1253	0.6856	1.0438	0.1612
CDKL4	0.9654	1.2720	0.9387	0.9994	1.0439	0.0770
LOC441031	0.9998	1.1260	1.0937	0.9561	1.0439	0.0397
CHP2	1.0809	1.1537	1.0902	0.8507	1.0439	0.0664
LOC1001347	1.0607	1.1230	1.1011	0.8908	1.0439	0.0526
C14orf68	1.0393	1.0415	1.1284	0.9665	1.0439	0.0331
PLCB3	1.0161	1.1067	1.1517	0.9012	1.0439	0.0553
NMT2	0.9104	1.2318	1.2105	0.8230	1.0439	0.1039
CAPN1	1.1102	1.2562	1.1713	0.6380	1.0439	0.1386
NEURL	0.9459	1.2327	1.0608	0.9364	1.0439	0.0690
C16orf63	0.9519	1.2750	1.2289	0.7200	1.0440	0.1294
LOC641945	0.9521	1.1490	1.0500	1.0248	1.0440	0.0407
LOC642892	0.9959	1.2210	1.1211	0.8381	1.0440	0.0827
SNORD113-5	0.9673	1.1623	1.0256	1.0209	1.0440	0.0416
LENEP	1.0556	1.1107	1.0668	0.9430	1.0440	0.0357
AGAP8	1.0710	1.4314	0.8915	0.7821	1.0440	0.1422
OSBPL10	1.0479	1.1414	1.1277	0.8596	1.0441	0.0649
RASL12	1.0168	1.0644	1.1081	0.9874	1.0442	0.0265
SLC6A16	1.0712	1.1341	1.0652	0.9062	1.0442	0.0486

AFTPH	0.9675	1.3308	1.0373	0.8412	1.0442	0.1038
MIR99A	0.9916	1.0337	1.1533	0.9982	1.0442	0.0375
C10orf55	0.8829	1.2256	1.1260	0.9423	1.0442	0.0796
PROM2	1.0208	1.4650	0.9515	0.7396	1.0442	0.1525
IFNGR2	1.1062	1.3371	1.0301	0.7035	1.0442	0.1310
ABHD13	1.0372	1.1553	1.0450	0.9394	1.0442	0.0441
WIPF2	0.9932	1.2555	0.9893	0.9390	1.0443	0.0715
MS4A15	1.0210	1.0438	1.0849	1.0274	1.0443	0.0144
LOC651842	0.9724	1.0399	1.1406	1.0243	1.0443	0.0352
KIAA0922	1.1268	1.2812	0.9664	0.8030	1.0443	0.1030
C6orf70	0.9403	1.4248	0.9494	0.8628	1.0443	0.1283
MCTP1	0.9728	1.1912	1.1039	0.9094	1.0443	0.0635
LOC653492	0.9654	1.1479	1.0987	0.9654	1.0444	0.0467
CAMK2D	0.9682	1.2567	1.1091	0.8434	1.0444	0.0892
LOC644828	1.0438	1.1177	1.1167	0.8996	1.0445	0.0513
NR1H2	0.7197	1.5714	1.1172	0.7696	1.0445	0.1966
LOC729273	1.1465	1.0925	0.9943	0.9446	1.0445	0.0458
HES2	1.2750	1.1297	0.9507	0.8226	1.0445	0.0993
TSGA10	0.9953	1.2098	1.0484	0.9245	1.0445	0.0607
LOC651065	1.1082	1.0526	1.0230	0.9943	1.0445	0.0243
LOC1001317	0.9152	1.2020	1.0713	0.9897	1.0445	0.0614
C14orf82	0.8749	1.3051	1.1155	0.8827	1.0445	0.1033
DDX26B	0.9584	1.2097	1.0674	0.9428	1.0446	0.0616
NID1	0.9681	1.1618	1.1278	0.9206	1.0446	0.0591
RHOB	0.9847	1.2129	1.3475	0.6334	1.0446	0.1562
LRRC37A	1.0163	1.1246	1.2154	0.8223	1.0446	0.0846
CCL5	0.9693	1.1738	1.1875	0.8481	1.0447	0.0824
SYT11	0.8811	1.2318	1.0826	0.9832	1.0447	0.0747
RERG	1.0737	1.1475	1.0040	0.9536	1.0447	0.0422
ENTPD6	1.1645	1.3388	0.8737	0.8019	1.0447	0.1255
SIX1	0.8788	1.2228	1.1840	0.8934	1.0447	0.0920
C4orf48	0.9439	1.3798	1.0059	0.8494	1.0447	0.1162
PITPNM1	1.0421	1.3620	0.9965	0.7785	1.0448	0.1204
FLJ33651	1.0145	1.0624	1.0836	1.0186	1.0448	0.0169
GUSBL2	0.8677	1.4439	1.1177	0.7499	1.0448	0.1535
LOC730241	0.9498	1.2128	0.9882	1.0285	1.0448	0.0583
MAN1A1	0.9873	1.1408	1.2144	0.8369	1.0448	0.0839
C10orf68	0.9349	1.2046	1.1592	0.8807	1.0449	0.0804
C6orf141	1.0224	1.1016	1.0976	0.9579	1.0449	0.0342
EPN2	1.0731	1.2349	0.9836	0.8879	1.0449	0.0738
LOC642411	0.9371	1.2413	1.0396	0.9616	1.0449	0.0690
LOC1001315	1.0298	1.2314	1.0861	0.8323	1.0449	0.0826
IFNA10	1.0617	1.1251	1.0857	0.9072	1.0449	0.0477
KIAA0652	1.1315	1.2473	0.9849	0.8161	1.0449	0.0933
LOC1001320	0.9369	1.2366	1.2321	0.7742	1.0449	0.1143
MTF1	1.0049	1.2270	0.9716	0.9764	1.0450	0.0611

LOC642167	0.9289	1.2732	1.0641	0.9138	1.0450	0.0832
GMCL1L	0.9584	1.1812	1.0503	0.9902	1.0450	0.0492
ZNF589	1.1073	1.2653	0.9992	0.8085	1.0451	0.0959
FLJ12331	1.1020	1.1425	1.0385	0.8974	1.0451	0.0537
NDUFB3	0.8996	1.4693	1.0414	0.7702	1.0451	0.1518
INSL5	0.9972	1.2826	1.0921	0.8088	1.0451	0.0986
EIF2C1	0.8788	1.4619	1.0286	0.8113	1.0452	0.1462
LOC1001288	1.0890	1.1917	1.0647	0.8354	1.0452	0.0751
LOC1001346	0.9426	1.2091	1.0432	0.9861	1.0452	0.0584
FBRS	1.1133	1.3424	1.0076	0.7177	1.0453	0.1296
SPRR1A	0.8044	1.4063	1.1018	0.8684	1.0453	0.1363
OR7A17	0.8273	1.0701	1.2054	1.0784	1.0453	0.0790
LOC648517	0.9961	1.2240	1.0280	0.9332	1.0453	0.0627
LOC649700	1.0915	1.0974	1.0419	0.9504	1.0453	0.0340
LOC1001344	0.9634	1.3061	1.0067	0.9052	1.0453	0.0894
LOC646638	1.0077	1.1142	1.1114	0.9481	1.0453	0.0408
CHMP2B	0.7987	1.3172	1.3464	0.7191	1.0454	0.1663
ERCC-00081	0.9740	1.0404	1.1218	1.0453	1.0454	0.0302
OPTN	0.9549	1.3579	1.1017	0.7672	1.0454	0.1246
LOC728755	0.8425	1.5150	0.9549	0.8695	1.0454	0.1583
NKIRAS2	1.0104	1.4304	0.9996	0.7414	1.0455	0.1426
MIR1302-5	1.0226	1.2294	0.9460	0.9838	1.0455	0.0633
POLK	0.9236	1.2272	1.0699	0.9613	1.0455	0.0681
SORCS2	1.0431	1.0175	1.1361	0.9853	1.0455	0.0324
LOC643391	1.0079	1.2158	1.0496	0.9086	1.0455	0.0640
LOC649189	0.9784	1.1998	0.9377	1.0661	1.0455	0.0580
DNALI1	1.0262	1.2593	1.0941	0.8026	1.0456	0.0946
SLC1A2	1.0163	1.1333	1.0188	1.0139	1.0456	0.0293
SPAG6	0.9817	1.1404	1.1099	0.9503	1.0456	0.0468
RUNDC2A	0.9111	1.1139	1.1001	1.0575	1.0456	0.0464
LOC392563	0.9743	1.2222	1.0036	0.9824	1.0456	0.0592
COPG2	0.7817	1.5480	1.0779	0.7749	1.0456	0.1817
LPPR3	1.0327	1.1950	1.0494	0.9054	1.0456	0.0593
MIR92B	0.8785	1.2584	1.0214	1.0243	1.0457	0.0787
MIR31	0.8935	1.2702	0.9837	1.0353	1.0457	0.0804
OR8I2	0.9796	1.1970	1.0655	0.9407	1.0457	0.0568
KCNV1	0.9659	1.2026	1.0783	0.9361	1.0457	0.0606
LOC642233	0.9955	1.2641	1.0613	0.8621	1.0458	0.0838
PTPN5	0.9501	1.1731	1.0768	0.9830	1.0458	0.0502
FBXO18	0.9094	1.4267	1.0217	0.8252	1.0458	0.1332
KIAA0460	1.0705	1.2540	0.9967	0.8619	1.0458	0.0817
LOC651031	0.9133	1.1493	1.0983	1.0223	1.0458	0.0513
REXO1L1	1.0506	1.0917	1.1263	0.9148	1.0458	0.0463
LOC650114	0.9486	1.1794	1.0615	0.9940	1.0459	0.0502
LOC1001303	0.8846	1.4188	1.1317	0.7485	1.0459	0.1475
LOC653438	1.2289	1.2516	0.8969	0.8062	1.0459	0.1138

ABLIM3	1.0064	1.1366	1.0949	0.9459	1.0459	0.0430
PGLYRP1	0.9955	1.0999	1.1262	0.9621	1.0459	0.0397
H1F0	1.0416	1.2996	1.0295	0.8133	1.0460	0.0995
ZNF479	0.9865	1.0221	1.1705	1.0049	1.0460	0.0421
LOC1001317	0.9241	1.2627	1.0268	0.9704	1.0460	0.0752
TXNRD1	0.7978	1.6878	0.9640	0.7346	1.0460	0.2193
LOC284293	0.9963	1.1763	1.0479	0.9640	1.0461	0.0467
OPN1MW2	0.9001	1.1436	1.1881	0.9528	1.0462	0.0705
HERC1	0.9769	1.4396	0.9165	0.8516	1.0462	0.1336
LOC728992	0.8541	1.3772	1.1142	0.8392	1.0462	0.1271
PLUNC	0.9829	1.1526	1.0978	0.9514	1.0462	0.0474
GUCY1B3	0.8395	1.3180	1.1308	0.8965	1.0462	0.1104
LOC731477	0.9808	1.1763	1.0658	0.9620	1.0462	0.0489
GUCY2D	0.9894	1.1758	1.0603	0.9595	1.0462	0.0481
LOC730034	0.9020	1.1501	1.1324	1.0006	1.0463	0.0585
LOC390811	0.9934	1.1125	1.0963	0.9831	1.0463	0.0338
COG4	1.0221	1.4514	0.9681	0.7438	1.0463	0.1479
LOC648359	1.0716	1.1267	1.0035	0.9834	1.0463	0.0328
ACVR1C	1.0060	1.1272	1.0472	1.0052	1.0464	0.0287
LOC728125	1.0652	1.0500	1.1207	0.9497	1.0464	0.0356
LOC1001305	0.9238	1.1725	1.1176	0.9716	1.0464	0.0589
FBXO32	0.9699	1.3841	1.0106	0.8211	1.0464	0.1197
AMTN	1.0082	1.1339	1.1693	0.8746	1.0465	0.0669
LOC642101	0.9618	1.1604	1.0405	1.0234	1.0465	0.0416
EPSTI1	0.9696	1.2891	1.0179	0.9096	1.0466	0.0838
C6orf72	0.9513	1.1476	1.3200	0.7674	1.0466	0.1197
PANK3	0.9956	1.3595	1.0363	0.7950	1.0466	0.1169
ZFYVE16	0.9861	1.2332	1.0755	0.8917	1.0466	0.0726
BAGE5	0.9698	1.2272	1.0660	0.9235	1.0466	0.0671
RFXDC2	0.9948	1.2339	1.0486	0.9093	1.0466	0.0687
RAB40AL	1.0790	1.0844	1.0699	0.9534	1.0467	0.0312
HRES1	1.0401	1.2356	1.0109	0.9002	1.0467	0.0698
LOC401923	0.9324	1.0953	1.2110	0.9483	1.0467	0.0659
PCDHB12	0.9709	1.1987	1.1137	0.9038	1.0468	0.0669
HERC2	0.9415	1.4946	0.8525	0.8984	1.0468	0.1504
CADM3	0.8964	1.1388	1.1422	1.0098	1.0468	0.0588
TSPYL3	0.9269	1.1500	1.0824	1.0280	1.0468	0.0471
CAMK1	1.0799	1.3186	1.0513	0.7377	1.0469	0.1192
LOC1001341	1.0004	1.1046	1.1084	0.9741	1.0469	0.0349
LOC440978	1.0120	1.1027	1.1195	0.9533	1.0469	0.0391
LOC649181	1.0045	1.2130	1.0619	0.9083	1.0469	0.0638
VIPR1	1.0625	1.3202	1.0491	0.7560	1.0470	0.1153
ALMS1P	0.9621	1.1896	1.0382	0.9979	1.0470	0.0500
KRTAP6-2	1.0350	1.1201	1.0495	0.9832	1.0470	0.0282
SYTL3	0.9917	1.0542	1.2256	0.9164	1.0470	0.0659
P4HA2	1.0106	1.4630	0.9530	0.7615	1.0470	0.1485

UGT1A1	1.0365	1.0852	1.1196	0.9467	1.0470	0.0375
XIST	0.9091	1.3848	1.0206	0.8736	1.0470	0.1169
LOC643007	1.3661	1.0118	1.0983	0.7120	1.0470	0.1348
C18orf25	0.9811	1.2631	1.0113	0.9327	1.0470	0.0738
TULP4	0.9636	1.4591	1.0124	0.7533	1.0471	0.1484
DACT2	0.9976	1.0114	1.2370	0.9424	1.0471	0.0650
C16orf5	0.9873	1.3186	0.9678	0.9147	1.0471	0.0918
LOC1001304	1.0316	1.1880	1.0466	0.9223	1.0471	0.0545
LOC1001324	0.9699	1.2025	1.0921	0.9240	1.0471	0.0628
LOC652179	0.9775	1.3145	1.0546	0.8420	1.0471	0.0993
LOC650293	1.1006	1.0979	1.0768	0.9133	1.0471	0.0449
LOC730668	1.0061	1.1625	1.1068	0.9132	1.0472	0.0552
LOC1001340	1.0313	1.1849	1.0731	0.8995	1.0472	0.0590
LOC646003	0.9313	1.0477	1.1745	1.0356	1.0473	0.0498
LOC650757	0.9713	1.2474	1.1042	0.8664	1.0473	0.0826
GPBP1L1	1.0808	1.4461	0.9353	0.7273	1.0474	0.1514
MIR761	0.9354	1.2101	1.1257	0.9183	1.0474	0.0718
ATP6V1F	0.8579	1.4302	1.1552	0.7464	1.0474	0.1540
LOC1001301	0.9375	1.1967	1.0759	0.9796	1.0474	0.0576
AKIRIN2	0.8400	1.5520	1.1090	0.6888	1.0474	0.1893
INO80D	0.9714	1.5833	0.8446	0.7906	1.0475	0.1826
UBE2QP2	0.9956	1.1750	1.0998	0.9194	1.0475	0.0563
LOC643598	1.0122	0.9856	1.1696	1.0225	1.0475	0.0414
PART1	1.0582	1.2343	1.0200	0.8775	1.0475	0.0734
LOC650584	0.9447	1.2289	0.9761	1.0404	1.0475	0.0637
LOC652433	1.0229	1.2260	1.0067	0.9345	1.0475	0.0625
LOC650491	1.1589	1.0955	1.0479	0.8879	1.0475	0.0579
CXorf67	1.0260	1.0792	1.1009	0.9842	1.0475	0.0263
LOC653920	1.0098	1.1964	1.0015	0.9825	1.0476	0.0500
PLCH2	1.0264	1.2126	1.0532	0.8982	1.0476	0.0646
RPL13	0.7806	1.5228	1.0936	0.7934	1.0476	0.1741
LOC642097	1.0671	1.0261	1.1100	0.9873	1.0476	0.0264
LRRC4	1.0822	1.1150	1.0837	0.9097	1.0477	0.0466
GGNBP2	0.9289	1.6175	0.9786	0.6657	1.0477	0.2020
KLHL24	0.9437	1.2701	1.1832	0.7937	1.0477	0.1092
OSTM1	0.9827	1.2717	1.0783	0.8581	1.0477	0.0872
TLE1	0.8930	1.3510	1.0102	0.9367	1.0477	0.1039
SNAPC3	0.9986	1.4173	0.9877	0.7874	1.0477	0.1324
MAP3K7	1.0136	1.2658	1.0383	0.8733	1.0477	0.0813
SWAP70	0.9446	1.2009	1.2325	0.8131	1.0478	0.1014
GK5	0.9439	1.3870	1.1042	0.7560	1.0478	0.1336
LOC648794	1.0387	1.2029	0.9726	0.9769	1.0478	0.0539
TRHDE	1.0105	1.1579	1.0485	0.9744	1.0478	0.0397
CST3	0.9272	1.4156	1.0216	0.8268	1.0478	0.1289
MMP1	0.9285	1.1157	1.1547	0.9924	1.0478	0.0527
NTNG2	0.9785	1.1185	1.0970	0.9972	1.0478	0.0351

TANK	0.9786	1.3033	1.0460	0.8635	1.0478	0.0931
EFNA2	0.9814	1.0585	1.1489	1.0025	1.0479	0.0374
LOC1001290	0.8259	1.2899	1.0508	1.0249	1.0479	0.0950
FKBP3	1.0580	1.1217	1.3311	0.6809	1.0479	0.1355
SEMA3F	1.0562	1.3902	1.0846	0.6607	1.0479	0.1496
LOC648257	0.9894	1.1325	1.0490	1.0208	1.0480	0.0307
DHCR24	0.9769	1.1428	1.3205	0.7518	1.0480	0.1211
PLXNA3	1.0232	1.2954	1.0211	0.8523	1.0480	0.0917
ADRB1	1.0616	1.0945	1.1141	0.9219	1.0480	0.0434
IFIT1	0.8649	1.3019	1.2030	0.8226	1.0481	0.1200
LOC376475	1.1788	1.0900	0.9958	0.9277	1.0481	0.0548
SLC37A1	1.0050	1.3754	1.0054	0.8068	1.0481	0.1187
LOC729086	0.9842	1.1907	1.1942	0.8235	1.0482	0.0895
LOC1001345	0.6940	1.2416	1.0623	1.1948	1.0482	0.1240
SELT	0.7929	1.5648	1.0917	0.7435	1.0482	0.1886
HIST1H4B	0.9497	1.2971	1.0416	0.9045	1.0482	0.0877
CLCN4	0.9065	1.2782	1.0797	0.9286	1.0483	0.0858
LOC388255	0.9174	1.2076	1.0079	1.0605	1.0483	0.0608
C1orf168	1.1203	1.1123	1.0913	0.8695	1.0483	0.0599
PTPN20	0.8714	1.1407	1.1298	1.0516	1.0484	0.0623
LOC644992	0.9880	1.2632	0.9990	0.9433	1.0484	0.0726
MOV10L1	1.0386	1.0646	1.0498	1.0406	1.0484	0.0059
LOC644811	0.9574	1.1480	1.1355	0.9529	1.0484	0.0539
TRPC1	1.0219	1.0758	1.1426	0.9536	1.0485	0.0401
C20orf112	1.0177	1.1300	1.1757	0.8706	1.0485	0.0680
COL15A1	0.9725	1.1606	1.0585	1.0026	1.0485	0.0414
LOC649902	1.0621	1.0603	1.0575	1.0143	1.0486	0.0115
LOC650361	1.1348	1.0516	1.0632	0.9448	1.0486	0.0392
IFITM5	0.9778	1.1656	1.0730	0.9780	1.0486	0.0450
MORN1	0.9924	1.0852	1.0809	1.0361	1.0486	0.0218
C14orf28	0.9818	1.2864	1.0081	0.9182	1.0486	0.0815
GRIN2C	1.0628	1.2100	1.1620	0.7599	1.0487	0.1010
LOC650862	0.9837	1.0039	1.1570	1.0502	1.0487	0.0387
KRTHA5	1.0772	1.0625	1.0959	0.9591	1.0487	0.0306
CYP46A1	1.0902	1.1384	1.0617	0.9046	1.0487	0.0506
LOC651006	1.0311	1.1395	1.0855	0.9388	1.0487	0.0428
LOC653291	1.0200	1.2324	1.0614	0.8811	1.0487	0.0724
SYT13	1.1277	1.0740	1.0815	0.9120	1.0488	0.0471
LOC401676	0.9288	1.2813	1.2449	0.7403	1.0488	0.1298
LOC643863	1.0203	1.1282	1.1844	0.8625	1.0488	0.0708
NAPA	1.0206	1.3022	0.9981	0.8745	1.0489	0.0904
HSD17B2	1.1092	1.1219	1.0602	0.9041	1.0489	0.0500
LRRC34	1.1491	0.9584	1.1328	0.9552	1.0489	0.0533
ZNF189	1.0070	1.3130	1.0878	0.7878	1.0489	0.1085
TOMM22	0.8518	1.4422	1.1731	0.7285	1.0489	0.1611
TMEM14D	0.8639	1.3749	1.2607	0.6961	1.0489	0.1607

CLCN6	0.9634	1.3204	1.0755	0.8364	1.0489	0.1028
LOC650909	1.0314	1.2283	1.0106	0.9255	1.0489	0.0640
DSTN	0.9331	1.4071	1.2001	0.6557	1.0490	0.1631
LOC339692	1.0085	1.1469	1.0837	0.9569	1.0490	0.0417
LOC646573	0.9638	1.2322	0.9985	1.0018	1.0491	0.0616
SOD3	1.0470	1.0202	1.0728	1.0564	1.0491	0.0110
MATN1	0.9699	1.1543	1.0465	1.0257	1.0491	0.0386
CDX1	0.9695	1.1380	1.1417	0.9472	1.0491	0.0526
WNK1	0.9816	1.2551	1.1248	0.8351	1.0491	0.0906
CPVL	0.9829	1.1652	1.1400	0.9085	1.0491	0.0618
INSR	1.0145	1.1309	1.0381	1.0131	1.0491	0.0279
LOC643583	1.0076	1.0055	1.1589	1.0246	1.0491	0.0368
TMEM132B	0.8398	1.3178	1.0672	0.9719	1.0492	0.1009
COG2	0.8315	1.5428	1.0578	0.7647	1.0492	0.1761
ZNF653	0.9656	1.3169	1.0127	0.9017	1.0492	0.0921
CTAGE1	0.9831	1.1908	1.0285	0.9947	1.0493	0.0481
VN1R4	0.9102	1.1903	1.0408	1.0561	1.0494	0.0573
LOC729617	1.0307	1.1961	1.1410	0.8295	1.0494	0.0809
LRRC4C	1.0056	1.1314	1.1211	0.9393	1.0494	0.0465
C3orf34	0.6816	1.1065	1.5544	0.8550	1.0494	0.1896
LOC652735	1.0896	1.0686	1.0628	0.9768	1.0494	0.0249
DAP	0.8631	1.5142	1.0159	0.8046	1.0495	0.1612
ACAD10	1.0432	1.3403	1.0953	0.7191	1.0495	0.1278
SYNJ1	1.0041	1.1839	1.1182	0.8918	1.0495	0.0644
FOXB2	0.9961	1.1956	1.1110	0.8953	1.0495	0.0657
TTC9	1.0356	1.1763	1.0267	0.9596	1.0496	0.0455
LOC728636	1.0004	1.1505	1.0812	0.9664	1.0496	0.0414
ZNF429	0.8887	1.4668	1.0958	0.7473	1.0496	0.1564
FCGR1A	1.0057	1.1558	1.0907	0.9464	1.0497	0.0461
SVOPL	0.9934	1.1437	1.0039	1.0577	1.0497	0.0344
VCY1B	1.0538	1.0681	1.0971	0.9797	1.0497	0.0250
PTEN	0.8143	1.6327	0.9969	0.7550	1.0497	0.2010
LOC1001342	1.1110	1.1233	1.0431	0.9215	1.0497	0.0462
FRMPD1	0.9934	1.1533	1.1376	0.9146	1.0497	0.0576
UBTD1	1.1795	1.1645	1.0600	0.7950	1.0497	0.0890
C5orf4	1.0304	1.1738	1.1437	0.8510	1.0497	0.0731
LOC342979	1.0243	1.0074	1.1835	0.9839	1.0498	0.0454
LOC645296	0.9616	1.4002	1.0522	0.7852	1.0498	0.1293
MIXL1	0.9844	1.1855	1.0517	0.9776	1.0498	0.0482
LOC648987	1.0139	1.3029	1.0178	0.8648	1.0498	0.0916
OR2M5	0.9731	1.2367	0.9973	0.9922	1.0498	0.0625
HPCA	1.0252	1.0508	1.1722	0.9512	1.0498	0.0459
CYP2C9	1.1053	0.9414	1.1016	1.0513	1.0499	0.0382
OR8B8	0.9462	1.1985	1.0429	1.0119	1.0499	0.0535
MAP3K9	1.0535	1.3211	0.9480	0.8771	1.0500	0.0974
SREBF1	0.9931	1.5158	0.9740	0.7172	1.0500	0.1675

KIDINS220	1.0936	1.3797	0.9865	0.7402	1.0500	0.1325
LOC649965	1.0784	1.0902	1.1108	0.9208	1.0500	0.0436
LOC1001332	1.0289	1.1503	1.0523	0.9687	1.0500	0.0378
PLAC8L1	1.1739	1.0330	1.0434	0.9501	1.0501	0.0462
CTSO	0.9435	1.2711	1.2071	0.7789	1.0502	0.1149
LOC732215	0.6775	1.4638	1.2378	0.8217	1.0502	0.1820
IL1R1	1.0282	1.1887	1.1117	0.8726	1.0503	0.0677
VPS16	0.9364	1.4531	0.9601	0.8516	1.0503	0.1363
ISG20L1	0.7509	1.5121	1.2848	0.6535	1.0503	0.2072
LOC643350	1.0408	1.1680	1.0176	0.9751	1.0504	0.0415
MED26	1.0558	1.4113	0.9076	0.8267	1.0504	0.1293
RPS17	0.9777	1.3551	1.0773	0.7915	1.0504	0.1176
FLT1	0.9255	1.1198	1.2117	0.9446	1.0504	0.0693
ZNF563	0.9602	1.2327	1.0567	0.9523	1.0505	0.0652
LOC646823	1.0040	1.1707	1.1070	0.9204	1.0505	0.0553
LOC645643	0.9634	1.1724	1.1180	0.9483	1.0505	0.0559
ITPKB	0.9969	1.3764	0.9876	0.8412	1.0505	0.1143
REXO1L6P	0.8439	1.1912	1.1941	0.9730	1.0506	0.0862
C20orf55	0.9136	1.5660	0.9616	0.7613	1.0506	0.1770
RER1	1.1192	1.2992	1.0296	0.7547	1.0507	0.1135
SRY	0.9305	1.0552	1.1484	1.0686	1.0507	0.0450
FADD	0.9886	1.4213	1.0609	0.7320	1.0507	0.1423
TAF1C	0.9762	1.2415	1.1340	0.8511	1.0507	0.0860
MIR130B	0.9550	1.1577	1.1202	0.9701	1.0507	0.0516
SH3BP5L	0.9392	1.5737	0.8566	0.8336	1.0508	0.1758
ASH1L	1.0159	1.2201	1.0193	0.9478	1.0508	0.0588
TMEM43	0.9843	1.3430	1.0827	0.7935	1.0509	0.1144
SHISA2	1.0140	1.1126	1.1201	0.9567	1.0509	0.0396
FERD3L	0.9560	1.0952	1.1612	0.9913	1.0509	0.0471
WFS1	0.9376	1.4832	0.9461	0.8369	1.0509	0.1462
CD244	0.8932	1.2317	1.1839	0.8949	1.0510	0.0911
AKR1A1	0.9639	1.4302	1.0708	0.7390	1.0510	0.1441
CDK10	0.9915	1.4345	1.0354	0.7426	1.0510	0.1432
NEDD9	0.9965	1.2266	1.0686	0.9125	1.0510	0.0666
BACE1	0.9701	1.1472	1.1687	0.9182	1.0510	0.0628
LOC644650	0.9091	1.2372	1.1003	0.9576	1.0510	0.0741
LOC1001335	0.8608	1.3574	1.2190	0.7671	1.0511	0.1411
VEPH1	1.0099	1.1020	1.1682	0.9247	1.0512	0.0532
LOC400652	1.1567	1.0814	1.1536	0.8131	1.0512	0.0813
C2orf79	0.8264	1.4490	1.1867	0.7435	1.0514	0.1638
LOC728429	1.1104	1.0902	1.1380	0.8670	1.0514	0.0622
ALDH5A1	1.0240	1.2304	1.2331	0.7182	1.0514	0.1214
MIR2116	0.9319	1.2251	0.9579	1.0909	1.0514	0.0676
LOC1001301	1.0100	1.3756	0.9278	0.8924	1.0515	0.1108
TMEM86B	1.0588	1.1124	1.1132	0.9214	1.0515	0.0452
LOC1001292	0.9748	1.1207	1.0880	1.0224	1.0515	0.0327

KCNK9	1.0249	1.1977	1.0608	0.9225	1.0515	0.0569
KRT80	0.9306	1.2594	1.0811	0.9348	1.0515	0.0776
LOC653645	0.8720	1.1700	1.1447	1.0193	1.0515	0.0683
LOC646424	1.0084	1.2594	1.0235	0.9149	1.0516	0.0733
GPRASP2	0.9700	1.3050	1.0661	0.8652	1.0516	0.0939
UBA2	0.9686	1.2581	1.1228	0.8568	1.0516	0.0878
GSX1	0.9642	1.1395	1.0803	1.0225	1.0516	0.0377
LOC644620	0.9833	1.1120	1.1295	0.9817	1.0516	0.0401
LOC642648	1.0437	1.0074	1.2442	0.9113	1.0517	0.0700
BASP1	0.9131	1.3344	1.1949	0.7647	1.0518	0.1297
LOC644126	1.0195	1.1050	1.1106	0.9721	1.0518	0.0338
CCBE1	0.9851	1.1792	1.2132	0.8297	1.0518	0.0895
HAPLN3	1.0616	1.1279	1.0752	0.9426	1.0518	0.0391
FAM39E	0.9153	1.5074	0.9650	0.8196	1.0518	0.1548
C4orf3	0.9438	1.1432	1.1236	0.9967	1.0518	0.0485
LOC648791	0.9832	1.2051	1.0500	0.9691	1.0519	0.0540
LOC1001331	1.0097	0.9711	1.1999	1.0268	1.0519	0.0507
ERCC-00116	1.1330	1.1055	0.9805	0.9886	1.0519	0.0393
GRIN3A	1.0735	1.1278	1.1556	0.8508	1.0519	0.0692
LOC283710	0.9185	1.2114	1.0651	1.0127	1.0519	0.0612
CPAMD8	0.9926	1.0885	1.1203	1.0064	1.0520	0.0311
RSPH1	0.9858	1.2904	1.1191	0.8127	1.0520	0.1012
LOC1001312	1.0504	1.2301	1.1796	0.7481	1.0520	0.1081
SLC9A10	1.0089	1.1627	1.1383	0.8984	1.0521	0.0614
RIMS2	0.9335	1.2892	0.9011	1.0851	1.0522	0.0886
LOC1001320	0.8794	1.4320	1.1769	0.7208	1.0523	0.1580
LOC653103	0.9973	1.5291	0.8927	0.7901	1.0523	0.1645
VPS18	1.0729	1.2506	1.1056	0.7800	1.0523	0.0986
GXYLT1	0.9191	1.2780	1.1301	0.8822	1.0523	0.0930
SMPD1	1.0471	1.2946	1.0831	0.7849	1.0524	0.1046
CDNF	1.0559	1.1695	1.0323	0.9522	1.0525	0.0449
RGS19	1.0475	1.4437	0.8817	0.8370	1.0525	0.1380
LOC1001287	0.8779	1.4685	1.0707	0.7928	1.0525	0.1504
PCMT1	0.8310	1.3975	1.1489	0.8329	1.0525	0.1371
EDAR	0.9636	1.2177	1.1018	0.9273	1.0526	0.0666
LOC1001290	1.0244	1.2204	1.3204	0.6451	1.0526	0.1491
KIAA1843	0.9506	1.1264	1.0373	1.0964	1.0527	0.0387
PRSS35	0.9794	1.1554	1.0531	1.0228	1.0527	0.0375
LOC441294	0.9105	1.3393	0.9757	0.9852	1.0527	0.0970
C1orf54	0.9603	1.2605	1.0643	0.9257	1.0527	0.0753
TSPAN10	0.9934	1.3765	0.9920	0.8490	1.0527	0.1131
ZNF397OS	1.0233	1.2284	1.0491	0.9102	1.0528	0.0659
KANK4	0.9379	1.3326	1.0505	0.8901	1.0528	0.0991
TSC1	1.0259	1.3515	1.0506	0.7832	1.0528	0.1164
LOC649680	1.0147	1.0816	1.0968	1.0181	1.0528	0.0212
LOC650867	1.1442	1.1252	1.0154	0.9268	1.0529	0.0507

RDH11	0.9397	1.4133	1.0995	0.7591	1.0529	0.1388
PNLIPRP2	1.0673	1.0788	1.0751	0.9905	1.0530	0.0210
LATS2	0.9382	1.2388	1.1527	0.8822	1.0530	0.0851
ZC4H2	0.9520	1.4099	0.9731	0.8769	1.0530	0.1208
MIR153-1	0.9741	1.0853	1.1469	1.0058	1.0530	0.0391
BIRC3	0.9259	1.2842	1.1008	0.9015	1.0531	0.0889
CUEDC1	0.9683	1.4318	0.9603	0.8521	1.0531	0.1290
ARHGEF5L	0.9414	1.4607	1.0166	0.7939	1.0531	0.1435
RNF144A	1.0969	1.3713	0.9862	0.7585	1.0532	0.1273
RLBP1L1	0.9977	1.1509	1.1300	0.9343	1.0532	0.0522
LOC1001331	0.9986	1.2071	0.9842	1.0230	1.0532	0.0519
LOC651845	0.9815	1.2969	1.1488	0.7859	1.0533	0.1100
LOC1001332	1.0507	1.2704	1.0625	0.8295	1.0533	0.0900
FAM101A	0.8918	1.1012	1.2689	0.9513	1.0533	0.0843
ATP2C1	0.9871	1.3636	1.1372	0.7254	1.0533	0.1339
MKL1	0.9679	1.3725	0.9701	0.9029	1.0533	0.1075
WASH1	1.0241	1.1251	1.2766	0.7877	1.0534	0.1026
TRPM5	0.9836	1.1269	1.1345	0.9687	1.0534	0.0447
PGM3	0.9159	1.4264	1.1609	0.7109	1.0535	0.1546
TTC17	0.9326	1.5308	1.0014	0.7493	1.0535	0.1677
THYN1	0.8252	1.5164	1.1157	0.7569	1.0536	0.1728
LOC728693	1.0907	1.3757	0.9871	0.7607	1.0536	0.1276
LOC1001315	0.8386	1.2854	1.2737	0.8166	1.0536	0.1306
LOC644097	0.9877	1.2227	1.0833	0.9208	1.0536	0.0655
GABRA3	0.9506	1.2171	1.1099	0.9367	1.0536	0.0672
ACBD4	1.0117	1.2881	1.0648	0.8497	1.0536	0.0906
LOC391761	0.9536	1.1939	1.1909	0.8761	1.0536	0.0817
LOC1001301	1.0281	1.0414	1.1317	1.0135	1.0537	0.0266
BCKDHA	1.0961	1.4818	0.9372	0.6995	1.0537	0.1644
LOC399829	1.0074	1.1244	1.1135	0.9695	1.0537	0.0385
STK11	1.0251	1.2636	1.1304	0.7957	1.0537	0.0989
TAF15	0.8503	1.5944	0.9522	0.8179	1.0537	0.1825
JHDM1D	0.9956	1.2408	0.9677	1.0106	1.0537	0.0630
ARHGEF7	1.0773	1.2718	1.0116	0.8543	1.0538	0.0864
LOC644320	1.0504	1.0970	1.0634	1.0043	1.0538	0.0192
PPP1CB	0.9920	1.2989	1.1382	0.7861	1.0538	0.1090
TOMM7	1.0457	1.2525	1.1415	0.7755	1.0538	0.1019
ZCWPW1	1.0048	1.1805	1.1044	0.9254	1.0538	0.0559
ARL4C	1.0809	1.0733	1.0092	1.0523	1.0539	0.0161
PABPC5	1.0218	1.2649	1.0746	0.8546	1.0540	0.0845
RNH1	0.9172	1.4145	1.1147	0.7697	1.0540	0.1394
LOC148696	0.9073	1.3608	1.0630	0.8850	1.0540	0.1097
PRINS	1.0729	1.2468	0.9359	0.9605	1.0541	0.0708
PCSK9	0.9823	1.3344	1.0243	0.8753	1.0541	0.0986
MUL1	0.9538	1.5405	0.9613	0.7607	1.0541	0.1687
CCDC81	1.0064	1.3151	1.0404	0.8544	1.0541	0.0960

LOC440389	0.9510	1.1814	1.0864	0.9977	1.0541	0.0509
GNRH1	1.0819	1.3639	0.9965	0.7743	1.0541	0.1219
PLK2	1.1916	0.8921	1.2817	0.8511	1.0541	0.1073
PCDHA8	1.0888	1.0718	1.1483	0.9077	1.0542	0.0515
TNIP2	0.8935	1.4166	1.0554	0.8512	1.0542	0.1286
RPS6KA6	1.0400	1.3115	0.9953	0.8700	1.0542	0.0930
CYB5A	0.9398	1.3428	1.1352	0.7990	1.0542	0.1183
PCBP3	1.0138	0.9393	1.2534	1.0110	1.0544	0.0685
MIR548F4	1.0115	1.0609	1.1336	1.0117	1.0544	0.0288
CST9	1.1411	1.1724	0.9796	0.9247	1.0545	0.0605
LOC1001291	0.9966	1.1437	1.1711	0.9066	1.0545	0.0624
LOC504188	0.9466	1.1813	1.1388	0.9516	1.0546	0.0615
LOC648343	0.8844	1.2660	1.2101	0.8579	1.0546	0.1067
RPL9	1.0069	1.1714	1.1953	0.8448	1.0546	0.0815
RPS27	1.0120	1.1722	1.1227	0.9115	1.0546	0.0583
FAM117A	1.1375	1.1017	1.0454	0.9338	1.0546	0.0445
KIAA0913	0.9724	1.5222	0.9711	0.7528	1.0546	0.1642
SNAPIN	0.9083	1.6750	0.8772	0.7581	1.0546	0.2093
RGS4	1.0889	1.1575	1.0318	0.9405	1.0547	0.0459
TMEM33	1.0704	1.1936	1.2546	0.7003	1.0547	0.1242
LOC651900	1.2155	1.0095	1.0813	0.9126	1.0547	0.0638
AMAC1	1.0168	1.2790	0.9851	0.9379	1.0547	0.0765
LRRTM4	1.0408	1.1302	1.1067	0.9413	1.0547	0.0423
PPP4C	0.9035	1.4431	1.1042	0.7682	1.0548	0.1467
TSPAN13	1.0431	1.4237	0.8825	0.8699	1.0548	0.1291
AKT1	1.0697	1.5054	0.8673	0.7769	1.0548	0.1622
LOC440991	0.9668	1.3763	1.2782	0.5981	1.0549	0.1755
VMO1	1.0044	1.1110	1.1303	0.9743	1.0550	0.0386
DDB1	0.9084	1.4716	1.0715	0.7685	1.0550	0.1520
GREM2	1.0096	1.1396	1.0588	1.0122	1.0550	0.0304
PTPN13	0.9592	1.4633	1.0280	0.7696	1.0550	0.1466
GPR20	1.0337	1.2502	0.9519	0.9845	1.0551	0.0672
CEBPD	1.0407	1.3525	1.0407	0.7864	1.0551	0.1158
LOC643431	0.9929	1.1071	1.2324	0.8879	1.0551	0.0741
LOC401321	1.0465	1.1749	1.1405	0.8588	1.0552	0.0709
LOC401056	1.0887	1.0801	1.0486	1.0034	1.0552	0.0193
R3HDM2	1.0606	1.2992	1.0548	0.8064	1.0552	0.1006
CALCOCO2	0.9652	1.2528	1.0870	0.9160	1.0552	0.0750
LOC728799	1.0602	1.2335	0.9608	0.9665	1.0552	0.0636
BOLA2B	1.0377	1.2722	1.0367	0.8745	1.0553	0.0818
EID2B	0.8006	1.1470	1.5550	0.7186	1.0553	0.1907
LOC652547	0.9390	1.0550	1.1760	1.0514	1.0553	0.0484
HMGCL	1.0593	1.2845	1.1190	0.7587	1.0553	0.1098
FAM108A3	0.9395	1.4786	0.9432	0.8602	1.0554	0.1424
LOC388556	0.8479	1.4255	1.1621	0.7865	1.0555	0.1482
NPAS1	1.0803	1.1641	1.0416	0.9361	1.0555	0.0473

LOC645895	0.9080	1.3578	1.1989	0.7574	1.0555	0.1362
C16orf79	0.9887	1.2335	1.1802	0.8198	1.0556	0.0945
LOC649839	0.9919	1.1812	1.1646	0.8846	1.0556	0.0713
TRADD	1.0965	1.2734	0.9743	0.8783	1.0556	0.0852
FLJ44674	1.0746	1.2521	1.1024	0.7935	1.0557	0.0957
TBC1D5	1.0146	1.3838	0.9553	0.8690	1.0557	0.1134
CRAMP1L	1.1802	1.3910	0.8391	0.8124	1.0557	0.1397
CLUL1	1.0701	1.2440	1.0278	0.8809	1.0557	0.0747
CXorf61	0.9599	1.1828	1.1414	0.9388	1.0557	0.0621
C5orf49	0.9763	1.1555	1.1220	0.9695	1.0558	0.0484
LOC145814	0.8842	1.2037	1.1104	1.0250	1.0558	0.0679
MIR1281	0.9222	1.2015	1.1068	0.9929	1.0558	0.0616
SLC25A4	0.9514	1.4946	1.0001	0.7773	1.0559	0.1539
NAV3	1.1131	1.1524	1.0860	0.8721	1.0559	0.0628
LOC731096	1.0798	1.2909	1.0115	0.8415	1.0559	0.0930
SVIP	0.9546	1.1688	1.0484	1.0519	1.0559	0.0439
LOC648103	0.9077	1.2471	1.0372	1.0318	1.0559	0.0704
TMIGD2	0.9770	1.1660	1.0888	0.9920	1.0559	0.0443
LOC391160	1.0378	1.1559	1.0534	0.9771	1.0561	0.0371
LOC728875	1.0515	1.0868	1.0994	0.9865	1.0561	0.0253
KIF3A	0.9461	1.1864	1.1120	0.9798	1.0561	0.0563
MLYCD	0.9335	1.4845	0.9809	0.8254	1.0561	0.1465
LIPF	1.1143	1.1632	0.9925	0.9544	1.0561	0.0494
LOC642617	1.0500	1.0917	1.0940	0.9888	1.0561	0.0246
LOC642076	1.1034	1.1546	1.0384	0.9281	1.0561	0.0488
PCP2	1.0264	1.1554	1.0296	1.0133	1.0562	0.0333
PGBD3	0.9575	1.5062	1.0134	0.7477	1.0562	0.1605
SLAMF7	1.0101	1.0900	1.1127	1.0120	1.0562	0.0265
HKDC1	0.9785	1.2606	1.0337	0.9520	1.0562	0.0702
GAD1	1.0438	1.2226	1.0958	0.8630	1.0563	0.0746
SLC6A8	1.0268	1.4030	0.9621	0.8335	1.0563	0.1223
SNORD64	0.9705	1.1252	1.0982	1.0315	1.0563	0.0347
LOC643376	1.0685	1.0887	1.0357	1.0325	1.0563	0.0135
STK19	0.9854	1.4098	1.0010	0.8292	1.0563	0.1240
SNORD95	0.9561	1.2688	1.1120	0.8884	1.0563	0.0849
LOC1001343	0.9336	1.0320	1.2493	1.0107	1.0564	0.0677
GSDMC	1.0540	1.0765	1.1171	0.9780	1.0564	0.0292
LOC729900	0.9923	1.1586	1.1396	0.9352	1.0564	0.0549
RFPL1	0.9763	1.0866	1.3067	0.8564	1.0565	0.0957
LOC1001298	0.9440	1.4372	1.0591	0.7857	1.0565	0.1387
ALDH1L1	0.9218	1.1703	1.1833	0.9506	1.0565	0.0697
LIPL3	0.8957	1.2070	1.1270	0.9963	1.0565	0.0690
LOC1001329	1.0248	1.1810	1.1159	0.9044	1.0565	0.0600
ZNF605	1.0453	1.5363	0.8804	0.7643	1.0566	0.1700
LOC646098	1.0787	1.0923	1.0378	1.0176	1.0566	0.0174
KCNJ6	1.0641	1.0691	1.1684	0.9248	1.0566	0.0501

GABRB1	1.0628	1.0357	1.1390	0.9891	1.0567	0.0314
MIR194-1	1.0027	1.0512	1.1518	1.0210	1.0567	0.0333
RPL35A	1.0117	1.1832	1.1869	0.8450	1.0567	0.0815
C8orf17	1.1968	1.1264	0.9979	0.9059	1.0567	0.0650
ZNF226	1.1120	1.1229	1.1292	0.8628	1.0567	0.0647
ZNF362	1.0937	1.3540	0.9533	0.8259	1.0567	0.1132
THBD	0.9994	1.2895	0.9949	0.9431	1.0567	0.0786
HTR1E	0.8476	1.2923	1.1482	0.9388	1.0567	0.1006
SLIC1	0.9702	1.1620	1.0581	1.0368	1.0568	0.0398
UBE2E3	0.9764	1.3791	1.0779	0.7939	1.0568	0.1224
LOC643850	0.9092	1.2325	1.0979	0.9877	1.0568	0.0702
LOC1001348	1.0429	1.2362	1.0668	0.8817	1.0569	0.0725
TP53BP1	0.9868	1.3782	0.8889	0.9736	1.0569	0.1093
EEF1G	0.8602	1.4756	1.0787	0.8132	1.0569	0.1511
ZNF544	0.7778	1.5966	1.1202	0.7331	1.0569	0.1996
LOC642755	0.8583	1.5790	1.0315	0.7591	1.0570	0.1829
dJ341D10.1	1.0045	1.2624	1.1007	0.8603	1.0570	0.0844
RNF7	0.9175	1.4695	1.1377	0.7032	1.0570	0.1636
AKTIP	0.9441	1.4481	1.0268	0.8090	1.0570	0.1379
DUSP22	1.0043	1.3918	1.0420	0.7900	1.0570	0.1246
LOC729703	0.9566	1.1487	1.0740	1.0490	1.0571	0.0396
LOC650751	0.9528	1.1690	1.2009	0.9061	1.0572	0.0747
C20orf75	1.1412	1.0620	1.0777	0.9482	1.0573	0.0402
LOC729989	0.9375	1.2618	1.0467	0.9831	1.0573	0.0718
LOC643677	0.9901	1.1575	1.1396	0.9420	1.0573	0.0537
ASZ1	0.9694	1.1299	1.2255	0.9045	1.0573	0.0734
EPHA1	1.1209	1.3535	0.9197	0.8354	1.0574	0.1154
PLXDC1	0.9857	1.1592	1.0215	1.0634	1.0575	0.0374
LOC641958	0.9508	1.1898	1.1648	0.9246	1.0575	0.0696
OR13C8	1.1242	1.0130	1.0683	1.0245	1.0575	0.0252
GLOD5	1.0631	1.2319	1.0713	0.8637	1.0575	0.0754
WAS	1.0953	1.1901	1.0157	0.9289	1.0575	0.0557
DTX1	1.0033	1.1453	1.1518	0.9296	1.0575	0.0547
PKP2	1.0632	1.3246	1.0549	0.7874	1.0575	0.1097
FLJ20021	0.8771	1.4549	1.1466	0.7516	1.0575	0.1560
RBP2	1.0055	1.1718	1.0868	0.9663	1.0576	0.0456
EPB41L3	1.0618	1.1017	1.0689	0.9979	1.0576	0.0217
LOC652701	0.9840	1.1413	1.1386	0.9665	1.0576	0.0477
SMURF2	1.0387	1.1813	1.1012	0.9094	1.0577	0.0574
COX7C	1.0055	1.2587	1.0718	0.8947	1.0577	0.0763
GLTP	0.9990	1.3108	1.1854	0.7356	1.0577	0.1250
MAP3K6	0.9289	1.6095	0.9957	0.6968	1.0577	0.1948
KIAA0355	0.8673	1.6361	0.8745	0.8531	1.0577	0.1928
LOC729404	0.9392	1.1782	1.1831	0.9305	1.0577	0.0710
LOC643570	1.0557	1.2066	1.1270	0.8421	1.0578	0.0782
MAGEA10	0.9232	1.5515	1.0586	0.6982	1.0579	0.1806

TMEM59	0.8645	1.5602	1.0708	0.7364	1.0580	0.1810
TAF9B	1.0541	1.2070	1.0451	0.9257	1.0580	0.0576
LOC728591	1.0161	1.1304	1.0507	1.0347	1.0580	0.0252
HSPA1B	0.8984	1.4555	1.0833	0.7947	1.0580	0.1453
AMH	1.0330	1.2132	1.0357	0.9502	1.0581	0.0554
RSC1A1	0.8935	1.4584	1.0455	0.8350	1.0581	0.1406
C5orf13	1.0342	1.5258	0.9402	0.7323	1.0581	0.1682
LOC642993	0.9349	1.2139	1.0736	1.0101	1.0581	0.0592
ZNF841	1.2411	1.1921	1.0532	0.7462	1.0582	0.1113
PDCD7	1.0101	1.3776	1.0793	0.7660	1.0583	0.1259
LOC1001328	1.0692	1.2751	0.9838	0.9050	1.0583	0.0797
GFAP	1.1090	1.0102	1.0878	1.0261	1.0583	0.0238
DMAP1	0.8554	1.6667	0.9428	0.7684	1.0583	0.2059
LOC649616	1.0796	1.1127	1.2103	0.8310	1.0584	0.0807
KRT72	1.0025	1.1714	1.0961	0.9638	1.0585	0.0468
LOC1001923	0.9125	1.2605	1.1020	0.9589	1.0585	0.0785
CDKN2B	1.0285	1.2089	1.0574	0.9392	1.0585	0.0561
LOC402117	1.0930	1.1194	1.0804	0.9413	1.0585	0.0399
BRP44L	0.9785	1.1708	1.3545	0.7304	1.0585	0.1336
LOC285307	1.0814	1.1482	1.0792	0.9253	1.0585	0.0472
TCTE1	1.0141	1.1284	1.1398	0.9519	1.0586	0.0455
ARHGEF18	0.9746	1.4877	0.9793	0.7926	1.0586	0.1495
ZNF543	1.0467	1.2456	0.9816	0.9608	1.0587	0.0649
VIPR2	1.0806	1.1983	1.0131	0.9427	1.0587	0.0544
LOC1001336	1.1193	1.0625	1.0945	0.9587	1.0588	0.0353
LOC652202	1.0334	1.1675	1.0199	1.0144	1.0588	0.0365
EPHA3	1.0128	1.1907	1.2253	0.8066	1.0588	0.0961
UPLP	1.0057	1.1601	1.2460	0.8237	1.0589	0.0928
DHRS1	0.9315	1.4611	1.0942	0.7489	1.0589	0.1515
LOC727805	0.9366	1.2472	1.0108	1.0413	1.0590	0.0665
HIST1H3D	1.0876	1.1267	1.2251	0.7968	1.0590	0.0921
SGCB	0.9687	1.1568	1.1696	0.9411	1.0591	0.0604
MUM1L1	1.0819	1.0985	1.0968	0.9592	1.0591	0.0335
ADCY6	1.1493	1.2258	0.9912	0.8703	1.0591	0.0797
PSD3	0.9195	1.3935	1.0508	0.8727	1.0591	0.1177
PNMA2	1.1255	1.1738	0.9523	0.9851	1.0592	0.0536
IQCE	0.9922	1.2534	1.0434	0.9481	1.0593	0.0676
ZNF496	1.1728	1.0072	0.9699	1.0873	1.0593	0.0451
LOC441155	0.9581	1.3058	1.0616	0.9118	1.0594	0.0879
RNF144	1.0492	1.4050	0.9633	0.8200	1.0594	0.1245
LOC646672	1.0126	1.1440	1.3733	0.7076	1.0594	0.1389
LOC645387	0.9599	1.1891	1.1988	0.8900	1.0595	0.0790
TPT1	0.9870	1.2612	1.1561	0.8336	1.0595	0.0941
CHIC2	0.8304	1.4126	1.1215	0.8735	1.0595	0.1340
CIC	1.1918	1.2775	0.9451	0.8237	1.0595	0.1056
SNORA7A	1.0904	1.2418	1.0291	0.8769	1.0595	0.0755

LOC220433	0.9506	1.2335	1.3589	0.6956	1.0597	0.1484
LOC728857	1.1208	1.1052	1.1336	0.8791	1.0597	0.0605
BRE	0.8983	1.5467	1.0428	0.7509	1.0597	0.1729
THRSP	1.0031	1.2132	1.1521	0.8703	1.0597	0.0770
LOC1001318	0.8710	1.2148	1.0830	1.0700	1.0597	0.0709
MIR448	0.7990	1.4798	1.0516	0.9087	1.0598	0.1493
C9orf96	1.0581	1.1147	1.0648	1.0014	1.0598	0.0232
KCND2	1.1337	1.1387	1.0071	0.9597	1.0598	0.0452
CADPS2	0.9881	1.2815	1.1117	0.8578	1.0598	0.0903
TTYH3	1.1149	1.3459	1.0251	0.7534	1.0598	0.1224
HDAC6	1.2350	1.2249	0.8978	0.8819	1.0599	0.0983
LOC641751	1.1636	1.0629	1.1327	0.8806	1.0600	0.0634
LOC646942	0.9684	1.4627	1.1000	0.7089	1.0600	0.1569
ABHD10	0.9690	1.5014	1.0502	0.7197	1.0601	0.1631
C20orf185	0.9566	1.2062	1.0359	1.0420	1.0602	0.0524
XAGE1D	1.1219	1.0927	1.0687	0.9575	1.0602	0.0359
CD55	1.0249	1.1383	1.3181	0.7596	1.0602	0.1170
LOC1001297	1.1003	1.1405	1.0935	0.9066	1.0602	0.0523
FLJ25996	0.9962	1.2279	1.0991	0.9180	1.0603	0.0670
MAN2C1	0.9452	1.2753	1.1526	0.8684	1.0604	0.0935
LOC791120	0.9981	1.6494	0.8611	0.7330	1.0604	0.2037
C14orf50	0.9552	1.2188	1.0947	0.9731	1.0604	0.0612
NCRNA0021	0.9174	1.4540	1.0367	0.8338	1.0605	0.1376
CCDC93	1.0284	1.4105	1.0190	0.7842	1.0605	0.1296
SLC14A1	0.9814	1.1148	1.1871	0.9591	1.0606	0.0544
UBL5	0.9368	1.4080	1.1264	0.7715	1.0607	0.1366
ZNF428	0.9904	1.5810	0.9242	0.7472	1.0607	0.1809
FLJ41766	1.0679	1.2099	1.0328	0.9323	1.0607	0.0574
PNLIP	0.9869	1.1727	1.1035	0.9800	1.0608	0.0468
DNAJC25-GN	0.9680	1.2452	1.1714	0.8586	1.0608	0.0893
LOC729090	0.8141	1.5452	1.1669	0.7172	1.0608	0.1882
LRGUK	1.0309	1.2041	1.0315	0.9769	1.0609	0.0494
CBY3	1.0688	1.2061	1.0293	0.9395	1.0609	0.0555
FLJ32154	0.9057	1.2622	1.0713	1.0046	1.0609	0.0752
LOC1001327	0.7954	1.2330	1.1589	1.0564	1.0609	0.0956
LRRC9	1.0574	1.0928	1.1228	0.9709	1.0610	0.0329
KIAA1086	0.9740	1.1728	1.0415	1.0558	1.0610	0.0413
LOC1001308	0.7812	1.3470	1.4595	0.6566	1.0611	0.2005
LOC341604	1.0083	1.1438	1.1095	0.9828	1.0611	0.0388
SC4MOL	0.7775	1.3635	1.4758	0.6277	1.0611	0.2105
FAM38A	1.0328	1.5636	0.8879	0.7605	1.0612	0.1765
BLCAP	0.9548	1.5584	1.0226	0.7090	1.0612	0.1789
LOC652354	0.9984	1.1350	1.0996	1.0122	1.0613	0.0333
C6orf221	0.9248	1.0814	1.1678	1.0713	1.0613	0.0504
LOC440055	0.6625	1.4697	1.2343	0.8788	1.0613	0.1801
KCTD2	1.0946	1.3340	0.8973	0.9194	1.0613	0.1010

JMJD2B	1.2007	1.1578	1.0788	0.8088	1.0615	0.0880
HIST1H1D	1.0012	1.1608	1.1010	0.9831	1.0615	0.0420
LOC649952	1.1092	1.0447	1.2128	0.8797	1.0616	0.0698
MORN2	0.9783	1.5120	1.0041	0.7522	1.0617	0.1604
KCNK17	0.9074	1.2897	1.0855	0.9642	1.0617	0.0846
LOC653344	1.0130	1.6822	0.9133	0.6385	1.0617	0.2215
CHRNB4	0.7668	1.3993	1.2133	0.8677	1.0618	0.1476
TPD52	0.8800	1.4121	1.0854	0.8700	1.0619	0.1268
C20orf43	0.8965	1.4357	1.1304	0.7849	1.0619	0.1439
HKR1	0.9663	1.2620	1.1117	0.9076	1.0619	0.0793
FLJ23834	0.9103	1.1338	1.2091	0.9947	1.0620	0.0673
LOC652721	0.9925	1.2317	1.1303	0.8936	1.0620	0.0745
DGCR11	0.8402	1.6539	0.9568	0.7973	1.0621	0.2001
DEFB132	1.0707	1.1715	1.0651	0.9410	1.0621	0.0472
MAP6D1	0.9913	1.2682	1.0810	0.9078	1.0621	0.0773
LOC729967	1.0022	1.2038	1.0631	0.9792	1.0621	0.0505
LOC648289	1.1110	1.0917	1.0638	0.9820	1.0621	0.0284
BAT2	1.0175	1.4280	0.9543	0.8490	1.0622	0.1268
OR51Q1	1.1077	1.0647	1.0492	1.0274	1.0623	0.0170
CALCOCO1	1.1774	1.1443	1.0366	0.8908	1.0623	0.0646
STOML1	1.0213	1.2666	1.0690	0.8922	1.0623	0.0777
RXFP1	1.0834	1.1326	1.0637	0.9695	1.0623	0.0342
ESPN	1.3005	0.9259	1.1393	0.8836	1.0623	0.0971
LOC1001316	0.7892	1.2626	1.1338	1.0639	1.0624	0.0999
ATP1B2	0.9093	1.4051	0.9677	0.9677	1.0624	0.1151
FAM161B	1.0073	1.3348	1.0190	0.8889	1.0625	0.0954
PI4KB	1.1015	1.3690	1.0130	0.7665	1.0625	0.1243
PHLPP1	1.1072	1.3349	0.8647	0.9433	1.0625	0.1039
LOC388720	1.0959	1.1425	1.1505	0.8616	1.0626	0.0681
PI4K2A	0.9412	1.3450	1.1490	0.8155	1.0627	0.1166
FURIN	1.0295	1.2952	1.0097	0.9165	1.0627	0.0813
TDP1	0.9822	0.9888	1.4999	0.7800	1.0627	0.1536
UCHL1	0.9803	1.3121	1.0624	0.8961	1.0627	0.0898
PVRL2	1.0583	1.2651	1.0310	0.8969	1.0628	0.0761
PRKAG3	1.0997	1.0889	0.9690	1.0938	1.0628	0.0313
PSME2	0.9124	1.5939	0.9867	0.7584	1.0628	0.1833
PATE2	0.9673	1.2381	1.1475	0.8984	1.0628	0.0786
LOC642567	1.0162	1.1972	1.2041	0.8340	1.0629	0.0878
RNF103	1.0902	1.4476	1.0499	0.6643	1.0630	0.1601
SMA4	0.9497	1.5808	0.9918	0.7298	1.0630	0.1819
UGCGL2	1.0172	1.4142	0.9716	0.8494	1.0631	0.1223
LOC646037	0.9853	1.2848	1.0559	0.9265	1.0631	0.0785
CNGB3	1.0620	1.1214	1.0935	0.9756	1.0631	0.0316
HBXIP	1.0983	1.2886	1.0883	0.7774	1.0632	0.1058
EXOC7	1.0373	1.3927	1.0564	0.7662	1.0632	0.1283
RWDD2B	0.8871	1.3369	1.1873	0.8413	1.0632	0.1192

ANKRD50	1.0454	1.1663	1.1192	0.9220	1.0632	0.0532
FLII	1.0093	1.2489	1.1653	0.8295	1.0632	0.0924
ARFGAP3	1.1283	1.4043	0.9333	0.7870	1.0632	0.1335
PHKG2	0.9300	1.5538	0.9990	0.7702	1.0633	0.1704
TMEM49	1.0212	1.3637	1.1383	0.7299	1.0633	0.1319
LOC643961	1.1566	1.1680	1.1255	0.8033	1.0633	0.0872
HIST1H1C	0.9581	1.2494	1.3987	0.6472	1.0634	0.1662
ELF2	0.9034	1.4587	1.1230	0.7684	1.0634	0.1507
LOC387885	0.9749	1.0617	1.2199	0.9971	1.0634	0.0553
RNF43	1.1163	1.1699	1.1391	0.8284	1.0634	0.0791
PACSIN1	1.0922	1.3900	1.0270	0.7444	1.0634	0.1325
DKFZp434J1(	1.1669	1.1014	1.0276	0.9578	1.0634	0.0453
LOC652793	1.0216	1.0937	1.2063	0.9325	1.0635	0.0579
LOC729339	0.9932	1.1807	1.2310	0.8493	1.0635	0.0878
RABEP2	1.0568	1.1770	1.1013	0.9192	1.0636	0.0541
ABCA7	1.1284	1.1612	1.0649	0.9000	1.0636	0.0581
POU4F2	1.0195	1.1934	0.9907	1.0511	1.0637	0.0450
SNX4	0.9151	1.2958	1.3080	0.7365	1.0639	0.1422
RND1	1.1011	1.1567	1.0197	0.9781	1.0639	0.0401
LOC1001328	1.1026	1.1460	1.0693	0.9379	1.0639	0.0448
OSMR	1.0129	1.2111	1.0340	0.9978	1.0639	0.0496
KLF4	1.0238	1.1660	1.2078	0.8584	1.0640	0.0790
AGA	1.0383	1.3449	1.1423	0.7305	1.0640	0.1281
PTPRK	1.0018	1.2556	1.0258	0.9730	1.0640	0.0648
NALP5	1.0567	1.0974	1.0747	1.0275	1.0641	0.0148
CYP2A6	1.1843	1.0252	1.1298	0.9170	1.0641	0.0591
LOC727874	0.9874	1.1555	1.1070	1.0072	1.0643	0.0401
RARB	1.1060	1.2006	1.0827	0.8679	1.0643	0.0702
LOC647850	1.0051	1.1409	1.0917	1.0194	1.0643	0.0318
KAL1	1.0289	1.0237	1.1831	1.0217	1.0644	0.0396
C11orf52	1.1746	1.4250	0.8971	0.7609	1.0644	0.1478
LOC728226	1.0590	1.1464	1.1620	0.8902	1.0644	0.0623
LOC644315	1.0325	1.2916	1.1908	0.7427	1.0644	0.1198
PPM1F	1.0002	1.4715	1.0210	0.7649	1.0644	0.1476
ZNF654	0.9411	1.2741	1.1920	0.8505	1.0644	0.1005
TCP10L	0.9604	1.1801	1.2065	0.9109	1.0644	0.0753
MIR216B	0.8967	1.2061	1.1500	1.0050	1.0645	0.0702
RTN3	0.9177	1.4798	1.0379	0.8224	1.0645	0.1453
ZNF518B	0.9728	1.5301	0.9562	0.7988	1.0645	0.1601
GNPTAB	0.9160	1.3805	1.1505	0.8114	1.0646	0.1269
ORM1	1.4100	1.1646	0.9228	0.7612	1.0646	0.1419
LOC643731	0.9252	1.1429	1.1430	1.0479	1.0648	0.0516
LOC644914	1.0239	1.4135	1.0110	0.8107	1.0648	0.1261
LOC1001284	1.0158	1.1073	1.0984	1.0377	1.0648	0.0225
LOC652501	1.0117	1.1521	1.0534	1.0420	1.0648	0.0304
CD2AP	0.8708	1.3971	1.2141	0.7775	1.0649	0.1452

LOC1001329	0.8798	1.2983	1.2355	0.8460	1.0649	0.1175
GUCA2A	1.1180	1.0692	1.1208	0.9515	1.0649	0.0396
TGFA	0.9738	1.3117	1.1738	0.8002	1.0649	0.1122
LOC1001326	1.0345	1.2149	1.0801	0.9302	1.0649	0.0590
LOC149501	0.9760	1.3812	1.1238	0.7788	1.0649	0.1269
LOC728069	1.0025	1.6384	0.9997	0.6192	1.0649	0.2113
TCERG1L	1.0170	1.1634	1.0908	0.9888	1.0650	0.0392
DCDC2B	0.9844	1.0456	1.1066	1.1237	1.0651	0.0317
LRRN1	1.1507	1.1314	1.1128	0.8659	1.0652	0.0669
DPCR1	1.1374	1.1192	1.1004	0.9040	1.0652	0.0543
RAPGEF1	0.9226	1.3611	1.0865	0.8907	1.0652	0.1075
PNPLA6	0.9685	1.4847	0.9910	0.8169	1.0653	0.1451
CST2	1.0774	1.2157	0.9956	0.9724	1.0653	0.0550
LYPLAL1	0.9535	1.4593	1.2105	0.6378	1.0653	0.1760
C9orf125	0.9901	1.2505	1.1235	0.8971	1.0653	0.0773
LOC643815	0.9680	1.2728	0.8355	1.1850	1.0653	0.0999
DAGLB	0.9326	1.4296	1.1117	0.7876	1.0654	0.1383
LOC440295	1.0596	1.0922	1.1479	0.9617	1.0654	0.0391
DDX42	1.0350	1.4105	1.0493	0.7668	1.0654	0.1321
LOC284230	1.0405	1.3567	1.1032	0.7614	1.0654	0.1222
MIR639	0.8419	1.3993	0.9649	1.0557	1.0655	0.1196
MIR491	1.0290	1.2331	1.1627	0.8372	1.0655	0.0871
RAB3IP	0.9190	1.5509	1.0395	0.7528	1.0655	0.1721
CAMTA1	0.9306	1.2889	1.1756	0.8671	1.0656	0.0998
ZMYND8	0.9346	1.5385	1.0032	0.7863	1.0656	0.1640
POM121C	0.9353	1.4275	1.2019	0.6981	1.0657	0.1585
TMEM129	0.9623	1.5120	0.9332	0.8556	1.0658	0.1504
PRMT2	1.0871	1.3050	1.0764	0.7946	1.0658	0.1046
TRIM56	0.9806	1.2544	1.1535	0.8749	1.0658	0.0851
AARS2	0.8988	1.4406	1.0398	0.8841	1.0658	0.1298
SPG11	1.0379	1.4524	0.9800	0.7930	1.0658	0.1390
ADAM10	0.9093	1.5138	1.0482	0.7924	1.0659	0.1582
LOC651337	1.1969	1.0658	1.1090	0.8920	1.0659	0.0641
FRS3	0.9453	1.5999	0.8910	0.8275	1.0659	0.1796
PIN4	1.0106	1.1797	1.2550	0.8185	1.0659	0.0970
PANX2	0.9948	1.3365	1.1006	0.8318	1.0659	0.1058
GPR176	1.0015	1.3972	0.9478	0.9174	1.0660	0.1118
FXYD3	1.0178	1.2490	1.0457	0.9513	1.0660	0.0641
FAM174A	0.8898	1.4500	1.0569	0.8672	1.0660	0.1348
NMNAT2	1.0739	1.2912	1.0783	0.8206	1.0660	0.0962
LOC729616	1.0090	1.2356	0.9646	1.0551	1.0661	0.0594
LOC651959	1.0425	1.2137	1.0715	0.9369	1.0661	0.0570
LOC392425	1.0265	1.0680	1.1795	0.9908	1.0662	0.0409
C19orf56	0.8266	1.7100	0.9350	0.7931	1.0662	0.2167
GSS	1.0760	1.5029	1.0070	0.6791	1.0662	0.1693
C12orf71	1.0256	1.2802	1.1092	0.8501	1.0663	0.0894

SIGLEC9	1.0744	1.1967	0.9362	1.0579	1.0663	0.0533
LOC389101	0.9905	1.1851	1.2188	0.8708	1.0663	0.0823
AP2A1	0.8932	1.3988	1.0809	0.8925	1.0663	0.1194
EIF3L	0.9895	1.5169	1.0227	0.7365	1.0664	0.1632
ZNF346	0.9988	1.2834	0.9891	0.9942	1.0664	0.0724
PCDHB18	1.1098	1.0909	1.0980	0.9670	1.0664	0.0334
FLJ20972	1.2028	1.1647	0.9726	0.9257	1.0664	0.0688
LOC401019	1.0202	1.2344	1.1103	0.9010	1.0665	0.0705
ATP5J	1.0301	1.4161	1.0583	0.7614	1.0665	0.1344
RBM5	1.0708	1.4671	1.0350	0.6932	1.0665	0.1583
MIR1974	0.7626	1.3845	1.3254	0.7938	1.0666	0.1671
IVD	0.8157	1.5117	1.2534	0.6857	1.0666	0.1917
C6orf106	1.0489	1.3132	1.0265	0.8779	1.0666	0.0905
C14orf124	0.8557	1.7880	0.9558	0.6672	1.0667	0.2478
CLEC2L	0.7520	1.4585	1.0626	0.9942	1.0668	0.1466
LOC1001306	0.9189	1.2771	1.3145	0.7571	1.0669	0.1364
OGFOD2	1.1352	1.1151	1.1364	0.8816	1.0671	0.0620
LOC642077	0.9521	1.3535	1.0855	0.8774	1.0671	0.1047
KCNJ13	1.0166	1.1690	1.1724	0.9105	1.0671	0.0636
LOC728903	0.9084	1.1614	1.2346	0.9646	1.0672	0.0778
EIF4G3	0.9970	1.5775	1.0351	0.6595	1.0673	0.1899
PRPF19	1.0038	1.3453	1.1912	0.7289	1.0673	0.1327
LOC1001280	1.0741	1.2289	1.0057	0.9608	1.0674	0.0587
SOX9	1.0225	1.3178	0.9427	0.9867	1.0674	0.0851
LOC729466	1.0006	1.3474	1.1250	0.7969	1.0675	0.1152
LUM	0.9620	1.3341	1.1455	0.8285	1.0675	0.1101
MUCL1	1.0162	1.1141	1.1917	0.9482	1.0676	0.0536
NMNAT1	0.9405	1.3113	1.2137	0.8048	1.0676	0.1176
PLXNA1	1.0348	1.3742	0.9746	0.8871	1.0677	0.1066
LOC642153	1.0307	1.2020	1.0609	0.9773	1.0677	0.0480
LOC653284	1.0835	1.1430	1.1604	0.8844	1.0678	0.0633
CREB3L4	1.1169	1.4012	1.0068	0.7464	1.0678	0.1356
LOC1001313	0.9508	1.3104	1.3314	0.6787	1.0678	0.1564
D4S234E	1.1208	1.3029	0.9965	0.8514	1.0679	0.0957
LOC642357	0.8601	1.4233	1.0947	0.8937	1.0679	0.1293
MNDA	1.1644	1.2410	1.0818	0.7851	1.0680	0.0998
CCDC26	0.9620	1.0817	1.1185	1.1102	1.0681	0.0362
TESK2	1.1239	1.3685	0.9816	0.7983	1.0681	0.1203
NADK	1.1695	1.3183	1.0009	0.7839	1.0681	0.1148
ROMO1	1.0568	1.4436	1.0741	0.6984	1.0682	0.1522
LOC646819	0.8251	1.3252	1.4450	0.6776	1.0682	0.1870
LOC1001346	1.0938	1.2497	1.0174	0.9123	1.0683	0.0710
LOC652455	1.0119	1.3245	1.1549	0.7820	1.0683	0.1148
SNX15	0.9778	1.3457	1.0652	0.8847	1.0683	0.0995
MIR106A	0.8564	1.2406	1.2115	0.9649	1.0684	0.0939
C2orf68	1.0993	1.1703	1.0500	0.9541	1.0684	0.0454

RNF146	0.8815	1.5239	1.1082	0.7606	1.0686	0.1680
PPM1D	1.0338	1.1585	1.2440	0.8379	1.0686	0.0882
ASXL1	1.0101	1.4041	1.0534	0.8070	1.0687	0.1240
LOC389223	0.8163	1.4803	1.0727	0.9056	1.0687	0.1471
CCDC148	0.9266	1.2807	1.1241	0.9436	1.0687	0.0836
PRPF40B	1.0478	1.2078	1.1472	0.8724	1.0688	0.0733
EXOC3	0.9401	1.3653	1.0762	0.8939	1.0689	0.1061
MYBPC2	0.9433	1.2421	1.1348	0.9554	1.0689	0.0725
LOC1001342	1.0327	1.1219	1.1701	0.9512	1.0690	0.0485
AKAP13	1.0738	1.3709	0.9772	0.8540	1.0690	0.1102
SNTG1	1.0155	1.2026	1.0681	0.9897	1.0690	0.0474
FOLH1	1.1158	1.2177	1.2658	0.6769	1.0690	0.1344
NCAN	0.9387	1.4667	1.0207	0.8503	1.0691	0.1370
GCHFR	1.2067	1.4825	0.9029	0.6843	1.0691	0.1745
KLKP1	1.1324	1.2254	0.9934	0.9253	1.0691	0.0676
ETV1	0.9865	1.1647	1.1498	0.9756	1.0692	0.0510
C1orf227	0.9756	1.1639	1.1912	0.9461	1.0692	0.0631
PFAAP5	1.0624	1.2681	1.1548	0.7918	1.0693	0.1016
DCBLD2	0.9977	1.2795	1.0504	0.9500	1.0694	0.0730
PRKCH	1.1113	1.4672	0.9529	0.7464	1.0694	0.1522
SCARNA21	0.9395	1.2306	1.1570	0.9507	1.0695	0.0734
LOC127295	0.8754	1.4099	1.2120	0.7806	1.0695	0.1464
RNF145	0.9611	1.4440	1.1019	0.7714	1.0696	0.1420
RNU1A3	1.1460	1.1188	1.1906	0.8230	1.0696	0.0835
ZNF317	0.9677	1.4308	1.0947	0.7852	1.0696	0.1361
STON1-GTF2	1.1609	1.0612	1.1432	0.9133	1.0696	0.0565
PPL	1.0922	1.2106	1.0814	0.8944	1.0696	0.0653
LOC642678	0.8248	1.3395	1.1323	0.9820	1.0696	0.1097
C1orf161	1.0698	1.1971	1.1278	0.8840	1.0697	0.0671
SLC25A37	0.9191	1.5374	0.9662	0.8561	1.0697	0.1575
LOC731985	0.6851	1.5631	1.2513	0.7799	1.0698	0.2058
TSR2	0.9357	1.5593	1.0183	0.7662	1.0699	0.1714
MYO1D	1.1910	1.1973	1.0210	0.8706	1.0700	0.0780
SDSL	0.9805	1.4342	1.1913	0.6742	1.0700	0.1613
ABCC8	0.9639	1.3739	1.0640	0.8783	1.0700	0.1082
SRR	1.0398	1.3225	1.1345	0.7836	1.0701	0.1121
TMPRSS6	0.9489	1.2755	1.1717	0.8845	1.0701	0.0920
LOC728247	1.0487	1.0594	1.1129	1.0597	1.0702	0.0145
C2orf49	1.0247	1.2091	1.2241	0.8229	1.0702	0.0941
EDN1	0.9086	1.1917	1.1218	1.0588	1.0702	0.0603
NOV	1.0191	1.1390	1.2630	0.8599	1.0703	0.0860
CNR1	1.0741	1.0695	1.1894	0.9480	1.0703	0.0493
LOC729009	1.0768	1.1899	1.3964	0.6182	1.0703	0.1646
ZNF490	1.0892	1.2317	0.9990	0.9615	1.0703	0.0601
SNX16	0.9788	1.2488	1.1939	0.8599	1.0704	0.0912
LOC642989	0.7815	1.4089	1.5459	0.5450	1.0704	0.2416

PHC1	0.9457	1.2306	1.1819	0.9232	1.0704	0.0792
RDH16	0.9782	1.1742	1.1225	1.0066	1.0704	0.0466
FAM104A	0.8867	1.3838	1.2894	0.7218	1.0704	0.1585
FAM55C	0.9429	1.4436	1.0383	0.8570	1.0704	0.1298
LOC731501	0.9649	1.3648	1.1029	0.8493	1.0704	0.1110
CBX7	0.9860	1.4585	1.0106	0.8269	1.0705	0.1356
LOC644058	0.9859	1.2369	1.0507	1.0084	1.0705	0.0571
ARMCX3	0.8784	1.4899	1.0404	0.8735	1.0706	0.1451
LOC646703	0.9907	1.1330	1.1303	1.0284	1.0706	0.0361
C14orf153	0.8198	1.4278	1.2366	0.7985	1.0707	0.1560
GGT7	1.0036	1.2239	1.1159	0.9397	1.0708	0.0627
JARID1A	0.8376	1.6110	1.0731	0.7614	1.0708	0.1919
LOC645683	1.1029	1.2905	1.0297	0.8602	1.0708	0.0891
ALAD	0.9418	1.4355	1.0770	0.8290	1.0709	0.1317
GEMIN7	0.9092	1.2850	1.0514	1.0379	1.0709	0.0782
LOC646254	1.0635	1.0648	1.0783	1.0770	1.0709	0.0039
C11orf74	0.8575	1.3630	1.0810	0.9822	1.0709	0.1076
KDM3B	0.8599	1.7483	0.9274	0.7482	1.0709	0.2288
ZZZ3	1.0555	1.4536	1.0655	0.7092	1.0709	0.1521
ARHGEF3	0.9999	1.2356	1.1535	0.8949	1.0710	0.0764
LOC652458	1.1862	1.2949	0.9949	0.8081	1.0710	0.1074
SYTL2	0.9982	1.3152	1.1283	0.8431	1.0712	0.1001
GREB1	1.1101	1.1240	1.0273	1.0236	1.0712	0.0266
LOC1001295	1.0026	1.2750	1.1430	0.8644	1.0712	0.0886
LOC1001315	0.9616	1.0159	1.3386	0.9689	1.0712	0.0899
RHOBTB3	0.9318	1.3697	1.0427	0.9407	1.0713	0.1026
POLR3C	0.8088	1.6601	1.0502	0.7660	1.0713	0.2060
LRCH4	1.1509	1.2994	0.9823	0.8526	1.0713	0.0975
VLDLR	1.2173	1.2497	0.9224	0.8960	1.0714	0.0940
CA9	1.0364	1.1093	1.0778	1.0619	1.0714	0.0153
FLJ31568	1.1231	1.2971	1.0255	0.8401	1.0714	0.0954
LOC402694	0.8481	1.4725	1.2672	0.6980	1.0715	0.1799
C2orf40	0.9573	1.2048	1.2286	0.8952	1.0715	0.0849
ATP7A	0.9858	1.3723	0.9897	0.9382	1.0715	0.1009
HSD17B7	1.1017	1.5271	0.9653	0.6926	1.0717	0.1740
EPHA6	1.0796	1.2127	1.1247	0.8697	1.0717	0.0728
LCOR	1.1314	1.2973	1.1828	0.6755	1.0717	0.1365
SMG1	1.0472	1.4222	1.0631	0.7544	1.0718	0.1367
LRRC23	1.1407	1.2141	1.0343	0.8983	1.0719	0.0686
HCN4	1.0593	1.1504	1.0861	0.9916	1.0719	0.0329
LOC649431	1.1239	1.2212	0.9625	0.9802	1.0720	0.0615
SPANXN4	1.0466	1.1078	1.1169	1.0167	1.0720	0.0241
FAM82A2	1.0410	1.4732	0.9974	0.7767	1.0721	0.1457
C17orf45	0.7854	1.5806	1.2127	0.7096	1.0721	0.2025
RPS6P1	0.8219	1.2026	1.5672	0.6967	1.0721	0.1970
LOC648390	0.9023	1.4662	1.1424	0.7776	1.0721	0.1516

MTMR3	1.1435	1.2595	1.1306	0.7550	1.0721	0.1096
LOC641738	0.9056	1.2476	1.0892	1.0463	1.0722	0.0704
LMF2	1.2396	1.2565	1.0393	0.7537	1.0723	0.1171
RHPN1	1.0370	1.1907	1.1775	0.8839	1.0723	0.0718
NXPH2	1.0467	1.1593	1.1444	0.9393	1.0724	0.0509
TMEM127	0.9857	1.3459	1.0270	0.9313	1.0725	0.0932
KIAA0319L	1.0346	1.3274	1.1228	0.8054	1.0726	0.1081
DEFB124	1.0915	1.1813	1.0188	0.9989	1.0726	0.0413
CD63	0.9451	1.4329	1.1412	0.7719	1.0728	0.1418
LOC1001337	0.9445	1.2861	1.1808	0.8800	1.0729	0.0961
ZCCHC14	1.0506	1.5063	0.9417	0.7932	1.0729	0.1538
SERTAD1	0.9599	1.4440	0.9965	0.8916	1.0730	0.1256
TTTY15	1.0013	1.3944	1.0250	0.8719	1.0731	0.1122
FBN1	0.9650	1.1838	1.1650	0.9788	1.0732	0.0586
LOC648993	0.9011	1.2860	1.1719	0.9337	1.0732	0.0931
LOC387825	1.1390	1.2763	1.1380	0.7394	1.0732	0.1159
CMTM6	1.0522	1.4017	1.0975	0.7417	1.0732	0.1350
SNX29	1.1001	1.4677	0.8543	0.8711	1.0733	0.1429
FOXD4L3	1.1114	1.1762	1.0853	0.9204	1.0733	0.0544
LOC652672	1.0668	1.4246	1.0866	0.7153	1.0733	0.1448
SCAMP4	1.0988	1.2509	1.0294	0.9144	1.0734	0.0704
LOC1001347	1.0443	1.2656	1.1699	0.8146	1.0736	0.0975
C5orf35	0.8640	1.4048	1.2276	0.7982	1.0736	0.1452
ERO1L	0.9274	1.4657	1.0965	0.8050	1.0736	0.1437
TRPV6	1.0130	1.3014	1.1707	0.8096	1.0737	0.1059
SNORD114-2	0.9642	1.2003	1.1098	1.0205	1.0737	0.0517
HOTAIR	1.1169	1.0508	1.0639	1.0638	1.0738	0.0147
SHOC2	1.0448	1.4309	1.1251	0.6947	1.0739	0.1513
NFIB	1.0656	1.3680	1.0426	0.8200	1.0741	0.1126
PROS1	1.0295	1.5166	0.9528	0.7973	1.0741	0.1552
EYA3	0.9976	1.2661	1.1178	0.9150	1.0741	0.0763
CREBBP	1.0842	1.4051	1.0427	0.7645	1.0741	0.1312
PARK7	0.9167	1.6134	0.9622	0.8044	1.0742	0.1828
POU2F1	1.0819	1.3317	0.9498	0.9336	1.0743	0.0920
CLLU1	1.0504	1.1323	1.1599	0.9545	1.0743	0.0462
OR1N2	1.1083	1.1398	1.0505	0.9988	1.0743	0.0312
ATXN1L	1.1246	1.2145	1.0386	0.9196	1.0743	0.0629
ALPPL2	0.9724	1.1373	1.2686	0.9191	1.0744	0.0797
LOC1001286	0.9726	1.1561	1.1462	1.0230	1.0745	0.0455
CNTNAP3	1.0402	1.2474	1.0863	0.9241	1.0745	0.0670
HERPUD1	1.0306	1.4786	1.0134	0.7756	1.0746	0.1467
IFI6	0.8397	1.5261	1.1909	0.7418	1.0746	0.1787
OR7G3	1.1045	1.1593	1.0992	0.9357	1.0747	0.0483
CCL24	1.0206	1.2824	1.0060	0.9903	1.0748	0.0695
TBRG1	0.8994	1.4820	1.1270	0.7910	1.0749	0.1527
LOC651308	0.9805	1.2683	1.2543	0.7967	1.0749	0.1140

LOC647086	1.0331	1.2712	1.0950	0.9006	1.0750	0.0769
LOC1001306	1.0357	1.3896	1.1054	0.7693	1.0750	0.1274
FAM126B	0.8957	1.4913	1.0689	0.8442	1.0750	0.1468
PLRG1	0.8893	1.5686	1.1138	0.7287	1.0751	0.1825
COMMD1	0.8457	1.3131	1.2045	0.9374	1.0752	0.1099
LOC729236	0.8634	1.4466	1.2000	0.7907	1.0752	0.1526
DAG1	1.1081	1.4398	1.0222	0.7307	1.0752	0.1459
PIGT	0.9851	1.6248	0.9824	0.7088	1.0753	0.1943
ZNF837	1.1619	1.4211	0.8981	0.8201	1.0753	0.1365
ARD1B	1.0366	1.0531	1.0686	1.1430	1.0753	0.0235
LOC1001323	0.8423	1.1783	1.3173	0.9637	1.0754	0.1064
SERF2	1.1947	1.2382	1.1130	0.7558	1.0754	0.1096
PPP1R2	0.9986	1.5331	1.0319	0.7383	1.0755	0.1661
FAM129A	1.1318	1.3037	1.0388	0.8280	1.0756	0.0991
FLJ43663	1.0792	1.0808	1.1468	0.9961	1.0757	0.0309
RBCK1	0.9348	1.5297	1.0502	0.7884	1.0758	0.1605
ACTR1A	0.9728	1.5745	0.9940	0.7619	1.0758	0.1743
SHRM	0.8949	1.6118	1.1468	0.6499	1.0758	0.2054
NDUFA4	0.9822	1.2952	1.2537	0.7726	1.0759	0.1226
RRM2B	0.8715	1.5909	1.1043	0.7371	1.0760	0.1876
GOLGB1	1.0706	1.4356	0.9960	0.8020	1.0760	0.1325
FLJ40125	1.0469	1.1664	1.1111	0.9801	1.0761	0.0402
CRKL	1.0502	1.5979	1.0193	0.6371	1.0761	0.1977
ERCC-00162	1.0645	1.1404	1.1252	0.9747	1.0762	0.0376
GFER	1.0113	1.0229	1.3056	0.9651	1.0762	0.0775
LOC653111	1.1444	1.3581	0.9700	0.8325	1.0763	0.1136
C17orf91	1.0577	1.3336	0.9657	0.9484	1.0763	0.0890
RPS4Y1	1.0208	1.3349	1.1637	0.7861	1.0764	0.1161
CRABP1	1.0505	1.2332	1.0292	0.9929	1.0764	0.0536
KRT82	0.9614	1.1823	1.1350	1.0271	1.0765	0.0503
PHYHIP	1.0760	1.1335	1.1617	0.9346	1.0765	0.0505
LOC653623	0.9387	1.3235	1.1150	0.9287	1.0765	0.0928
HIATL2	0.7606	1.7792	0.9544	0.8122	1.0766	0.2378
TPMT	0.9774	1.3855	1.1133	0.8301	1.0766	0.1181
FLJ14712	0.9261	1.2893	1.1538	0.9373	1.0766	0.0882
LOC651713	1.0449	1.2381	1.1620	0.8619	1.0767	0.0819
EID2	0.9188	1.6582	0.9990	0.7310	1.0767	0.2018
LOC645045	0.9613	1.2754	1.2084	0.8619	1.0767	0.0984
MIR369	0.9134	1.4266	1.0153	0.9519	1.0768	0.1185
MOV10	1.3027	1.3550	0.8780	0.7716	1.0768	0.1475
GNPTG	1.0025	1.4745	1.0291	0.8012	1.0768	0.1420
NOXA1	0.9421	1.3147	1.0675	0.9831	1.0769	0.0835
FAM110A	1.0403	1.4798	1.0556	0.7318	1.0769	0.1536
LOC151760	0.9794	1.1706	1.2204	0.9374	1.0770	0.0697
CYB5RL	1.0327	1.3165	1.0410	0.9178	1.0770	0.0846
MORG1	0.8940	1.3727	1.1917	0.8504	1.0772	0.1243

MAP4K4	1.0731	1.3049	1.0696	0.8614	1.0772	0.0906
RPL5	0.7853	1.3470	1.4848	0.6922	1.0773	0.1984
RPS8	0.9958	1.3181	1.1363	0.8593	1.0774	0.0982
LOC1001330	1.0460	1.2225	1.1259	0.9151	1.0774	0.0650
TCF7L2	0.9990	1.1084	1.2490	0.9532	1.0774	0.0658
LOC1001324	1.0840	1.1806	1.0325	1.0131	1.0775	0.0375
CMTM8	1.0518	1.3826	1.1129	0.7632	1.0776	0.1271
MAP1B	0.9651	1.4196	1.0115	0.9143	1.0777	0.1157
FLOT1	1.0564	1.6347	0.9135	0.7061	1.0777	0.1991
FAM105A	1.1038	1.3676	0.9576	0.8822	1.0778	0.1070
LOC643278	0.9438	1.2669	1.1052	0.9953	1.0778	0.0715
SELP	1.0635	1.1736	1.1196	0.9548	1.0779	0.0468
KPNA6	0.9362	1.5197	0.9831	0.8728	1.0780	0.1490
CCDC130	0.9750	1.5125	0.9494	0.8750	1.0780	0.1464
MDGA1	1.1260	1.2616	1.0806	0.8438	1.0780	0.0870
SMPDL3A	0.9020	1.3936	1.1121	0.9045	1.0780	0.1161
MALT1	1.0789	1.2786	1.1467	0.8080	1.0780	0.0991
SMS	1.1960	1.5495	0.8940	0.6727	1.0781	0.1902
TMPRSS11E2	0.9468	1.1325	1.3000	0.9331	1.0781	0.0868
ZNF30	0.9690	1.3483	1.1488	0.8466	1.0782	0.1094
ST3GAL3	1.0624	1.4293	0.9626	0.8589	1.0783	0.1242
RNF130	0.9902	1.3117	1.2697	0.7418	1.0784	0.1330
TPD52L2	1.0310	1.3766	1.0603	0.8456	1.0784	0.1102
CTSD	0.9969	1.4489	1.1176	0.7503	1.0784	0.1452
A2ML1	1.0322	1.0990	1.1134	1.0691	1.0784	0.0180
UTRN	0.8819	1.2485	1.2701	0.9137	1.0785	0.1047
KYNU	0.9782	1.2072	1.2143	0.9147	1.0786	0.0774
LOC647784	1.0550	1.2968	1.1304	0.8325	1.0787	0.0964
LOC644863	0.9546	1.4837	1.1337	0.7431	1.0788	0.1568
PFKM	0.8693	1.7136	1.0425	0.6897	1.0788	0.2235
SETD3	0.9769	1.4200	1.0346	0.8836	1.0788	0.1179
LOC1001297	1.0380	1.3431	1.0994	0.8349	1.0788	0.1046
CCDC104	1.0183	1.3209	1.1969	0.7793	1.0789	0.1176
TRIM38	1.0441	1.2780	1.1188	0.8747	1.0789	0.0837
LOC285741	0.8714	1.2303	1.5064	0.7076	1.0789	0.1795
HUWE1	0.9454	1.4143	1.0208	0.9356	1.0790	0.1134
HDGF2	1.1038	1.3135	0.9887	0.9103	1.0791	0.0877
TNFSF4	1.0888	1.2878	1.0527	0.8873	1.0791	0.0822
SNORA59A	0.9867	1.3382	1.1323	0.8595	1.0792	0.1028
LOC442160	1.0215	1.1455	1.1724	0.9774	1.0792	0.0472
LOC285900	0.9502	1.3701	1.2192	0.7776	1.0793	0.1329
FTHL7	1.0296	1.3935	1.1029	0.7912	1.0793	0.1241
ZNF33A	1.0344	1.3209	1.1206	0.8413	1.0793	0.0995
LOC648392	0.9679	1.2492	1.1377	0.9626	1.0793	0.0697
OSTF1	0.7794	1.5850	1.1702	0.7828	1.0793	0.1919
ARHGDIA	0.8622	1.5495	1.1292	0.7764	1.0793	0.1738

PCDHB17	1.0142	1.3482	1.0258	0.9292	1.0794	0.0922
C20orf12	1.0880	1.1313	1.1386	0.9599	1.0794	0.0414
LOC732381	0.8467	1.1471	1.3037	1.0203	1.0795	0.0968
PTK2B	1.0586	1.4087	1.0457	0.8050	1.0795	0.1243
LOC649049	0.9306	1.4085	1.1621	0.8169	1.0795	0.1311
TRIM53	1.1554	1.1711	1.1209	0.8709	1.0796	0.0703
WDR33	0.9480	1.4771	1.1413	0.7525	1.0797	0.1544
TFDP2	0.9174	1.6246	0.9763	0.8008	1.0798	0.1852
LOC653773	1.0029	1.1287	1.4352	0.7528	1.0799	0.1419
ZNF436	1.0575	1.3561	1.0686	0.8382	1.0801	0.1062
PLA2G5	1.1196	1.1715	1.0865	0.9430	1.0802	0.0489
ZFP106	1.1153	1.5711	0.8746	0.7600	1.0803	0.1796
LOC643358	0.8495	1.5708	1.2217	0.6802	1.0806	0.1987
LOC1001295	0.9985	1.2778	1.1990	0.8470	1.0806	0.0976
SRGAP3	1.0478	1.3909	1.0593	0.8244	1.0806	0.1167
FAM166A	0.9852	1.2323	1.1843	0.9208	1.0806	0.0755
LOC651202	0.9138	1.2711	1.4547	0.6834	1.0807	0.1737
RAB32	1.1160	1.3743	1.0664	0.7666	1.0809	0.1246
FAM113A	1.0951	1.2048	1.1652	0.8584	1.0809	0.0776
LOC651123	1.0419	1.2375	1.1139	0.9306	1.0809	0.0644
LOC650812	0.9734	1.3787	1.1311	0.8405	1.0809	0.1157
ZFP36	0.8888	1.4839	1.1170	0.8340	1.0809	0.1476
LOC728453	1.0465	1.3540	1.1043	0.8191	1.0810	0.1099
ABCA2	1.0277	1.4028	1.0138	0.8796	1.0810	0.1124
RBM9	0.8713	1.5109	1.0979	0.8445	1.0812	0.1541
CBLL1	1.0212	1.4711	1.0808	0.7516	1.0812	0.1484
LOC642677	0.9591	1.3176	1.0133	1.0352	1.0813	0.0804
LOC389404	0.9040	1.2864	1.2830	0.8522	1.0814	0.1179
SOS1	1.0912	1.1021	1.1714	0.9611	1.0814	0.0439
LOC654191	1.0777	1.4007	1.0124	0.8352	1.0815	0.1181
POLR2C	0.9734	1.3509	1.2098	0.7920	1.0815	0.1240
LOC646347	1.2010	1.4673	0.8667	0.7916	1.0816	0.1563
GABARAPL2	0.9885	1.5107	1.0589	0.7685	1.0817	0.1558
ZFP28	1.0152	1.3014	1.1004	0.9099	1.0817	0.0829
LOC728671	0.9587	1.3257	1.1954	0.8477	1.0819	0.1089
ANKHD1-EIF4	1.1167	1.4195	0.9522	0.8393	1.0819	0.1261
CACNA1B	0.9983	1.1307	1.1414	1.0577	1.0820	0.0335
RPL23	0.9270	1.3294	1.4649	0.6072	1.0821	0.1952
EEF2	1.0168	1.4024	1.0858	0.8244	1.0824	0.1202
ANKRD24	1.0497	1.2530	1.0743	0.9530	1.0825	0.0626
C16orf45	0.9030	1.3685	1.2037	0.8550	1.0825	0.1226
ACRV1	0.9966	1.2736	1.1383	0.9219	1.0826	0.0779
COG1	1.0109	1.3070	1.1418	0.8710	1.0827	0.0930
LOC729859	0.9045	1.2673	1.3736	0.7854	1.0827	0.1411
[truncated: 109,200 more chars]
